# Metagenomic analysis of a throat swab sample collected in China on a patient infected with Varicella Zoster Virus

**DOI:** 10.1038/s41598-021-93230-8

**Published:** 2021-07-06

**Authors:** Hong Guo, Pierre Rivailler, Jiangxia Wang, Huanyu Wang, Wenbo Xu, Songtao Xu, Hongmei Xu, Ruiping Hu

**Affiliations:** 1 National Institute for Viral Disease Control and Prevention, Chinese Center for Disease Control and Prevention, Beijing, 102206 China; 2 College of Basic Medicine, Inner Mongolia Medical University, Hohhot, 010110 China; 3 Department of Infection Diseases Children’s Hospital of Chongqing Medical University; National Clinical Research Center for Child Health and Disorder; Ministry of Education Key Laboratory of Child Development and Disorders, Chongqing Key Laboratory of Pediatrics, Chongqing, 400014 China; 4 Center for Biosafety Mega-Science, Chinese Academy of Sciences, Wuhan, 430071 China

**Keywords:** Genome-wide association studies, High-throughput screening, Next-generation sequencing, Viral infection, Systemic lupus erythematosus

## Abstract

Varicella Zoster Virus (VZV) is endemic worldwide, causing varicella in children and zoster upon reactivation in adults. This study concerned a metagenomic analysis of a throat swab sample collected in China, on a young patient suffering from Systemic Lupus Erythematosus (SLE) and diagnosed with varicella. The complete genome sequence of a VZV strain of clade 2 has been generated. Clade 2 strains are the most prevalent in Asian countries. A comparison of 223 VZV genomes identified 77 clade specific markers, 20 of them specific to clade 2. The metagenomic analysis also identified sequences covering most of the genome of the bacteria *Schaalia odontolytica* also known as *Actinomyces odontolyticus.* VZV infection and bacterial infection in the context of SLE is further discussed. Even though the patient presented only mild symptoms, this study is a reminder that vaccination against VZV is critical to avoid severe complications like bacterial superinfection or even death in the case of immunodeficiency.

## Introduction

Varicella Zoster Virus (VZV) belongs to the alphaherpesviruses and is also known as human herpesvirus 3 (HHV3)^1^. VZV genome is a double stranded DNA of around 120 kb. It consists of two regions, large and short (_L_ and _S_), each consisting of unique and repeat sequence (U and R). Each U region is flanked by a repeat sequence, called terminal (T) and internal (I). In addition to the genome structure TR_L_U_L_IR_L_IR_S_U_S_TR_S_, the genome contains 6 repeat regions called IR1, 2, 3, 4a, 5 and 4b. IR4 is located in IR_S_ and is inversely duplicated in TRs. The genome encodes 73 open reading frames (ORFs), 3 of them being inversely duplicated in TR_S_. Sequence analysis identified 7 distinct clades (1, 2, 3, 4, 5, 6, 9). A putative clade VIII has been reported once^2^.

VZV infection usually causes varicella or chickenpox in children and zoster upon reactivation in adults. In addition to varicella and zoster, VZV infection has been associated with severe complications like bacterial superinfection, pneumonia, hepatitis, nephritis, encephalitis and even death^1^.

A live attenuated VZV vaccine has been developed based on the clade 2 Oka strain. Currently, many countries have introduced varicella vaccine in universal routine vaccination program and administered 2 doses of vaccine to control and prevent chickenpox^3^. In China, varicella vaccine became available in 1998 and is currently available in the private sector.

This study concerned a metagenomics analysis of a throat swab sample collected in China on a young patient suffering from lupus and diagnosed with varicella. In addition to generating the complete genome sequence of the VZV strain contained in the throat swab sample, the metagenomics analysis also identified bacterial sequences present in the sample. Finally, association between VZV and lupus is discussed.

## Materials and methods

### Ethical statement

The second session of the Ethics Review Committee of the National Institute for Viral Disease Control and Prevention (IVDC) at China Centers for Disease Control and Prevention (CDC) determined that the present study followed the working regulations of ethics review committee of Institute for viral disease control and prevention of Chinese Center for Disease Control and Prevention and therefore approved the study. The legal guardians of the patient involved in this study provided written informed consent to have data/samples from her medical records used in research. All methods were performed in accordance with the relevant guidelines and regulations.

### Clinical sample

A throat swab sample was collected on a 10-year-old girl diagnosed with varicella at Children's Hospital of Chongqing Medical University. The date of onset was estimated on December 21st 2018. The patient immediately found a rash all over her body after having contact with her sister with chickenpox. The patient began to exhibit macular papules that gradually turned into herpetic rash with itching, oral pain and no salivation. During the course of the disease, there was neither fever, spasm, disturbance of consciousness, headache, vomiting, unstable walking, skin/mucous membrane bleeding, abdominal pain, vomiting nor diarrhea. At the age of 9, the patient was diagnosed with Systemic Lupus Erythematosus (SLE) and Lupus Nephritis (LN) and was treated with glucocorticoids and immunosuppressants. The sample was collected in viral transport medium and stored at − 80 °C until use.

### Next generation sequencing (NGS)

Total DNA was extracted using QIAamp DNA Mini Kit (QIAGEN, Germany) then fragmented using the ultrasonicator S220 (Covaris, USA). A sequencing library was generated with the KAPA HyperPlus kit (Roche, Switzerland) and sequenced with the NovaSeq 6000 system (Illumina, USA). The paired-end reads have been deposited in the NCBI Sequencing Read Archive under the accession number PRJNA681411.

### NGS analysis pipeline

Sequence quality was assessed using FastQC (http://www.bioinformatics.babraham.ac.uk/projects/fastqc/). Sequences were cleaned up with Trimmomatic^4^ (http://www.usadellab.org/cms/index.php?page=trimmomatic). The sequences from the human genome (GRCh38) were depleted using the assembler Burrows-Wheeler Alignment tool (BWA) and Sequence Alignment/Map (SAM) tools 1.9^5,6^. The remaining sequences were processed in 2 ways: 1- assembly with SPAdes^7^ ( http://bioinf.spbau.ru/spades ); 2- mapping against the VZV reference strain Dumas (NC_001348) using BWA and SAMtools^5,6^ 1.9. The VZV-related sequences were then de novo assembled using Sequencher 5.0 (Genecodes Corp., Ann Arbor, MI, USA). The sequence strategy as well as the number of reads at each step of the analysis pipeline are shown in Fig. S1. The full-length genome sequence was annotated in Artemis^8^ 16.0.0 based on the Dumas reference genome (NC_001348) and submitted to GenBank (MW316406). The viral strain was named VZV/Chongqing.CHN/2018/V[2]. For convenience, however, the sample is referred in the manuscript as SD14.

### VZV sequence analysis

Two hundred thirty-two VZV whole genome sequences (WGS) were downloaded from GenBank. Identical or incomplete sequences were discarded. The remaining 222 genome sequences were compared with the de novo SD14 sequence (Table S1). As previously reported, the genome sequence was analyzed based on 8 regions (A to H), excluding the repeats TR_L_, IR1, IR2, IR3 and TR_S_^2,9^. Sequences were aligned using MAFFT 7.311 (http://maf.cbrc.jp/alignment/sofware/)^10^. Alignments were analyzed using BioEdit 7.0.4.1 (http://www.mbio.ncsu.edu/bioedit/bioedit.html)^11^. 7 single nucleotide polymorphisms (SNPs) found in each region were concatenated. Phylogenetic trees were generated with^12^ MEGA 6. Neighbor joining (NJ) trees were generated with the maximum composite likelihood nucleotide substitution model^12^. The phylogenetic inference was tested using the bootstrap method with 1000 replicate^13^. Bootstrap values greater than 70% were indicated. Phylogenetic trees were also generated using the maximum likelihood (ML) method in MEGA^14^. SNPs found only in SD14 genome were analyzed in Protein Variation Effect Analyzer (PROVEAN, http://provean.jcvi.org/seq_submit.php) in order to check whether these mutations had any effect on protein function^15^.

## Results

### Genome analysis of VZV strain contained in the throat swab

Close to 230 million sequencing reads were analyzed (Fig. S1). VZV-related sequences (11,275 sequences mapped on the VZV reference genome sequence strain Dumas (NC_001348)) were assembled in Sequencher. The de novo assembly generated 4 contigs. Gap sizes were estimated based on the Dumas strain, from 11 to 18 nucleotides. SD14 full genome was estimated at 125,184 nt long and 46.13% G + C content. This is in the same range as other HHV 3. For example, Dumas strain was estimated at 124,884 nt and 46.02% G + C^16^. The reiteration regions were slightly longer than what was described in Dumas (Clade 1) and pOka (clade2) (Table S2). As expected, SD14 genome encoded all known 73 ORFs, ORF62, 63 and 64 being duplicated in reverse direction in the TRs.

### Comparative genomics of 223 VZV WGS

**Figure 1:**
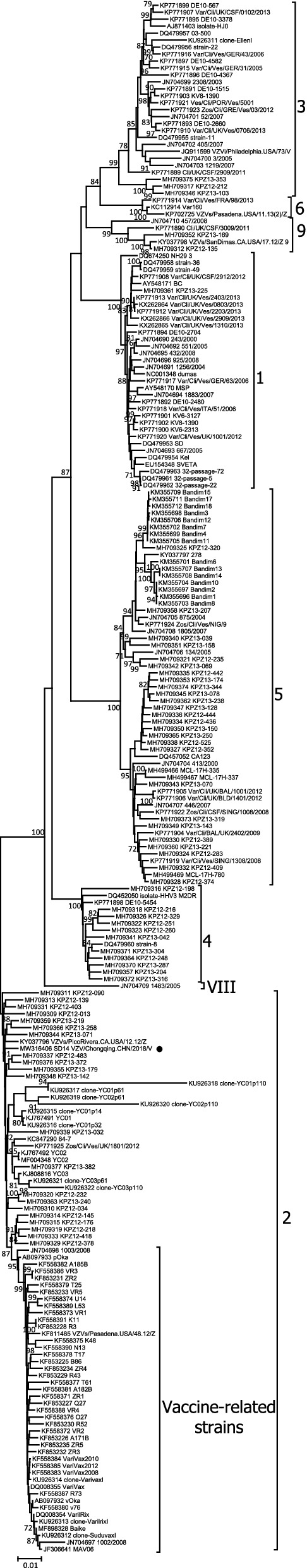
NJ phylogenetic tree of 2880 concatenated SNPs from 223 VZV genomes. Clades are indicated in brackets. Strains related to the vaccine strain Oka are also indicated in bracket. SD14 de novo sequence is indicated by a black circle. Bootstrap values greater than 70% are indicated. The corresponding ML tree is shown in Fig. S2.

The comparative genomic analysis of 223 genomes identified 2880 SNPs (Table S2). Phylogenetic trees based on the 2880 concatenated SNPs identified in the 223 genomes were generated (Fig. 1, Fig. S2). Both NJ and ML trees showed that SD14 strain belonged to clade 2 and could therefore be formerly named as VZV/Chongqing.CHN/2018/V^2^. Twelve SNPs were only found in the SD14 genomic sequence (Table 1). All 12 positions were located within ORFs but only 3 nucleotide substitutions were non-synonymous changes, L135R in ORF6, K98T in ORF37 and N47S in ORF54. None of these 3 non-synonymous substitutions were predicted to have an effect on protein function based on PROVEAN analysis. Phylogenetic trees on the 8 genomic regions were generated in order to identify any major recombination event (Fig. S3). Whereas some recombination events were identified among VZV genomes from clades 3, 6 and 9 (identified with * in Fig. S3), no evidence of major recombination event was identified for SD14 genome, as SD14 sequences were always found within the clade 2 cluster.

**Table 1:** Twelve SNPs found only in SD14 genomic sequence.

Position in dumas	Consensus	SD14	ORF	Protein function	Amino acid substitution	PROVEAN analysis
8174	a	c	6	Helicase-primase primase subunit	L135R	Neutral
22,392	t	g	15	Envelope protein UL43	No, G29	
31,976	a	g	21	Tegument protein UL37	No, V406	
35,243	a	g	22	Large tegument protein	No, A387	
40,031	t	g	22		No, V1983	
52,152	t	g	29	Single-stranded DNA-binding protein	No, V432	
66,366–7	aa	ct	37	Envelope glycoprotein H	K98T	Neutral
76,953	a	g	42	DNA packaging terminase subunit 1	No, G714	
95,845	t	c	54	Capsid portal protein	N47S	Neutral
107,382	t	c	62	Transcriptional regulator ICP4	No, V584	
108,543	t	g	62		No, P197	

### Identification of 77 clade specific markers

**Figure 2:**
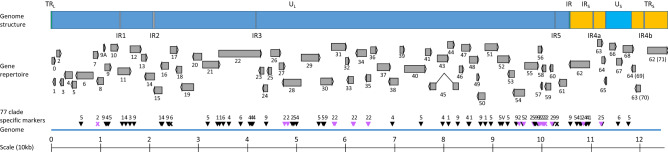
Schematic representation of VZV genome structure, gene repertoire as well as the genomic distribution of clade specific markers. Genomic regions are represented as color-coded boxes, in blue for unique regions (dark for U_L_ and light for U_S_), in green for repeat long (TR_L_ and IR_L_), in grey for internal repeats (IR1, 2, 3, 4a, 4b and 5) and in orange for repeat short (IR_S_ and TR_S_). ORFs are shown as grey arrows, rightward ORFs on the top and leftward ORFs on the bottom. Clade specific markers are shown as either triangles if they are within an ORF or an “X” if they are outside an ORF. Clades are indicated by a number (1, 2, 3, 4, 5, 6 or 9) or “V” in case of the vaccine marker. Marker specific to clade 2 are shown in purple. The genome is depicted by a thick line in the graph.

The SNPs analysis identified 77 positions that were conserved within a clade and could therefore be considered as clade specific markers (Fig. 2, Tables S3-4). The number of markers was highly variable depending on the clade, from 20 in clade 2 to only 1 in clade 3. Furthermore, the distribution of the markers throughout the genome was not random. For example, 2 markers were identified within the 70–80 kb region of the genome whereas 15 markers were identified within the 90–100 kb genomic region. Finally, 34 of the 70 ORFs featured at least one clade specific marker. If we consider the number of markers and the size of the ORF, ORF60 with 480 amino acid (AA) and 2 markers featured the most whereas ORF31 featured the less with 2 markers among 2796 AA. Regarding the 20 clade 2 specific markers (in purple in Fig. 2), 18 were located within ORFs and 6 were non-synonymous changes: C1159R in ORF28^17^, T136P in ORF31, P374F in ORF33, E128D in ORF54, H69P in ORF57 and A107T in ORF60 (Tables S2-3). The vaccine strains related to clade 2 Oka strain featured an additional marker (g911191t) (Tables S3-4). SD14 did not feature this vaccine marker and was therefore not related to vaccine strains as it was shown in the phylogenetic trees (Fig. 1, Fig. S2).

### Metagenomic analysis on the throat swab sample

Close to 230 million sequencing reads were depleted from the sequences of the human genome GRCh38 (Fig. S1). The remaining sequencing reads (~ 33 million, 14.4% of the sequencing data) were assembled using SPAdes and 159,894 contigs were generated (Table S5). The size of the contigs was highly variable, from 0.5 Mb to 78 nt, with an average of 731 nt. A blastn search among the 100 largest contigs is summarized in Table 2. The bacteria *Schaalia odontolytica* was the most prevalent hit with 15 hits and 2.2 Mb of cumulated contig size.

**Table 2:** Blastn hit on the 100 largest contigs.

blastn hit	Number of contigs	Cumulated contig size (nt)
Schaalia odontolytica	15	22,53,740
Haemophilus haemolyticus	12	9,94,917
Prevotella melaninogenica	12	6,76,310
Haemophilus parainfluenzae	10	8,57,385
Veillonella parvula	7	4,12,652
Alloprevotella	3	4,82,997
Barnesiella viscericola DSM 18,177	3	2,45,449
Porphyromonas gingivalis	3	3,32,837
Streptococcus oralis	3	1,50,553
Duncaniella dubosii	2	1,56,204
Muribaculaceae bacterium DSM 108,610	2	1,00,355
Parabacteroides distasonis	2	2,63,333
Porphyromonas asaccharolytica DSM 20,707	2	1,38,638
Porphyromonas cangingivalis	2	1,33,007
Prevotella ruminicola 23	2	1,03,666
Tannerella sp. oral taxon HOT-286	2	1,04,388
Bacteroides cellulosilyticus	1	45,367
Abiotrophia defectiva	1	44,116
Alistipes indistinctus	1	1,08,649
Bacteroides heparinolyticus	1	44,122
Bacteroides salanitronis DSM 18,170	1	49,021
Bacteroides sp. HF-5287	1	99,204
Bacteroides uniformis	1	55,360
Bacteroides xylanisolvens	1	54,303
Paraprevotella xylaniphila YIT 11,841	1	71,716
Porphyromonas crevioricanis	1	48,703
Porphyromonas pasteri	1	50,035
Prevotella copri	1	58,125
Prevotella dentalis DSM 3688	1	62,693
Prevotella fusca JCM	1	1,04,309
Prevotella intermedia	1	55,420
Uncultured bacterium	1	91,044
Uncultured prokaryote	1	1,32,948

## Discussion

This report concerned the metagenomic analysis of a throat swab sample collected in China from a young VZV patient. The phylogenetic analysis showed that this sample was of clade 2, which has been the dominant clade in Asian countries like Korea, Japan and China. In addition to clade 2, 6 other clades have been identified for VZV, one additional clade (VIII) is putative as only one strain has been reported so far (reviewed in^2^). Clades 1 and 3 are mainly observed in Europe and Americas whereas clades 4 and 5 are frequently seen in people with African origin^18,19^. Clade 6 and 9 have been reported recently and there is not enough data to conclusively assign a geographic region to these clades.

The number of complete VZV genome sequences has dramatically increased recently, 232 as of December 2020. Comparative genomic analysis showed that the VZV genome is very stable. The current analysis compared 223 genomes and identified 2880 SNPs, representing 2.3% of the genome. Several studies reported recombination events among VZV^9,20^. The present study did not identify any obvious recombination event within SD14 strain. Furthermore, the present study identified 77 nucleotide positions that are conserved within a clade. The detection of such marker is surprising giving the fact VZV genomes have been reported to easily recombine. These clade specific markers could be used as an indirect marker for lack of recombination. A genome prone to recombination would not share many positions with other related genome. Indeed, it is what has been observed. Phylogenetic analyses showed recombination for clade 3 and 6 genomes and clade 3 and 6 featured respectively only 1 and 4 clade specific markers. In contrast, clade 2 featured 20 clade markers suggesting that these genomes are not recombining frequently with genomes of other clades. A recent study identified a 58 kb region that is less recombining than the rest of the genome, from position 43,600 to 101,600^18^. The present study identified clade specific markers throughout the genome suggesting that recombination is not as frequent as previously reported and depends on the clade, clade 3 and 6 recombining more frequently than clade 2.

The present study concerned a throat swab sample. VZV samples are generally collected from rash vesicles. Among the 222 genome sequences analyzed in the present study, 137 (62%) were derived from vesicle fluid or skin lesion. Unfortunately, the sample information was not available for 64 sequences (29%). None of the analyzed sequences were from throat swab sample. To our knowledge, the present study is the first NGS analysis of a throat swab sample from a VZV patient.

Recent progress in technology allow sequencing of genetic material of a throat swab and identification of organisms present in the clinical sample. In addition to VZV-related sequences, the present metagenomic analysis identified multiple bacterial sequences. The most prevalent was from *Schaalia odontolytica*. The genome of *Schaalia odontolytica* has been estimated at 2.3 Mb (GB ID NZ_CP040006). The present study reported a cumulative contig size of 2.2 Mb suggesting that most of the bacterial genome can be detected in the sequencing data.. *Schaalia odontolytica* is also known as *Actinomyces odontolyticus*^21^. *A. odontolyticus* was first isolated in 1958 from persons with advanced dental caries^22^. In 2003, Tang et al. analyzed root canal infections from 28 Chinese patients and detected *A. odontolyticus* 16S ribosomal DNA in 30% of the cases^23^.Actinomyces are not generally detected in healthy patients^24^. Even though *A. odontolyticus* infection is relatively common in patients with dental issues, it has been linked to serious diseases, for example, neonatal sepsis^25^ or actinomycosis in a pediatric patient^26^ as well as in immunosuppressed patients^27^. VZV infection could be associated with superinfection by *Staphylococcus aureus* and *Streptococcus pyogenes*^28^. To our knowledge, any association between VZV and *A. odontolyticus* has not yet been investigated.

The patient featured in this study suffered from SLE and LN. Multiple reports of severe VZV infections in LN patients can be found in the literature. A recent report described a disseminated VZV infection likely to have caused the death of a 49-year-old LN patient^29^. A matched cohort study confirmed that adult patients with SLE presented an increased risk of disease flares if they were infected with VZV^30^. Most of the reports of an effect of VZV on SLE/LN patients concerned adult patients with herpes zoster^30,31^. To our knowledge, any effect of varicella disease on lupus remains to be reported.

In summary, this study concerned the metagenomic analysis of a throat swab sample collected in China from a young VZV patient suffering from SLE and LN. The VZV strain identified was of clade 2, clade prevalent in Asian countries. A comparison of 223 VZV genomes identified 77 clade specific markers, among them 20 were specific to clade 2. The metagenomic analysis identified sequences covering the entire genome of the bacteria *Schaalia odontolytica* also known as *A. odontolyticus* which has been linked to tooth decay as well as severe complications especially in immunocompromised patients. Even though the patient presented only mild symptoms, this study is a reminder that vaccination against VZV is critical to avoid severe complications like bacterial superinfection or even death in the case of immunodeficiency.

## Supplementary Information

Supplementary Information.

## Data Availability

The paired-end reads have been deposited in the NCBI Sequencing Read Archive under the accession number PRJNA681411. The full-length genome sequence of SD14 strain VZV/Chongqing.CHN/2018/V[2] was submitted to GenBank (MW316406).
