# Supplementary material for: Metagenomic analysis of a throat swab sample collected in China on a patient infected with Varicella Zoster Virus
Source: Sci Rep. 2021 Jul 6;11:13874. doi: 10.1038/s41598-021-93230-8 (PMC8260771; doi:10.1038/s41598-021-93230-8)
Supplement: Supplementary file 1 — Supplementary Information. [file 41598_2021_93230_MOESM1_ESM.pdf]

Metagenomic analysis of a throat swab sample collected in China on a patient infected with Varicella Zoster Virus

Hong Guo[1, 2+], Pierre Rivailler[1+], Jiangxia Wang[3], Huanyu Wang[1], Wenbo Xu[1,4], Songtao Xu[1,4*], Hongmei Xu[3*] and Ruiping Hu [2*]

1 National Institute for Viral Disease Control and Prevention, Chinese Center for Disease Control and Prevention, Beijing 102206, China.

2 College of Basic Medicine, Inner Mongolia Medical University, Hohhot 010110, China.

3 Department of Infection Diseases Children's Hospital of Chongqing Medical University; National Clinical Research Center for Child Health and Disorder; Ministry of Education Key Laboratory of Child Development and Disorders; Chongqing Key Laboratory of Pediatrics, Chongqing 400014, China.

4 Center for Biosafety Mega-Science, Chinese Academy of Sciences, Wuhan 430071, China.

*Corresponding authors: Songtao Xu, xsttz886@sina.com

Hongmei Xu, xuhongm0095@sina.com

Ruiping Hu, 783674348@qq.com

[+]These authors contributed equally to this work.

Correspondence and requests for materials should be addressed to S.X. (email: xsttz886@sina.com), H.X. (email: xuhongmwpx@yahoo.com) or R.H. (email: 783674348@qq.com)

A

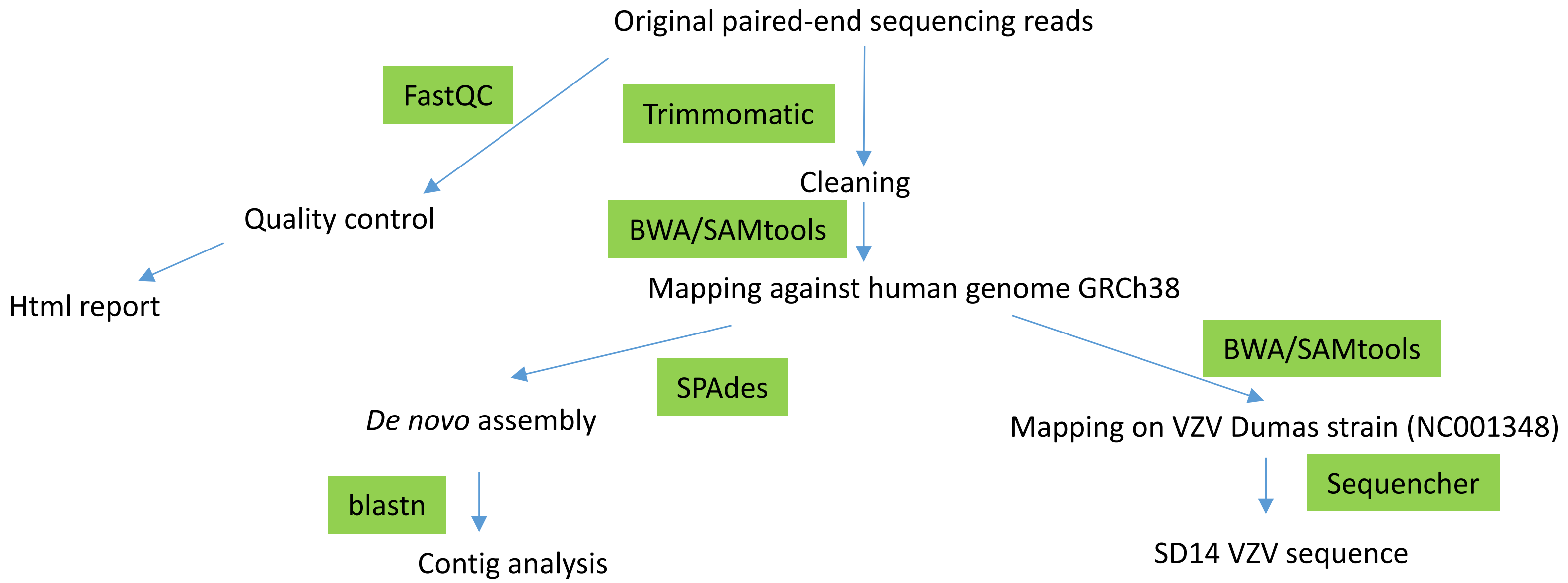

B

Number of reads			
**Total**	**After cleaning**	**Not human (% relative to total number of reads)**	**Mapped to Dumas strain NC001348 (% relative to total number of reads)**
144412431 paired-end	229,493,683	33141150 (14.4)	11275 (0.03)

Figure S1

Figure S1: Analysis pipeline and number of sequencing reads at each step of the analysis. A. Analysis pipeline. Briefly, after cleaning with trimmomatic, the sequencing reads were depleted from the human genome sequences. The remaining sequences were either assembled using SPAdes or mapped on the Dumas strain sequences. The mapped sequences were assembled with Sequencher. Analysis tools are shown in green. B. Number of sequencing reads at each step of the analysis.

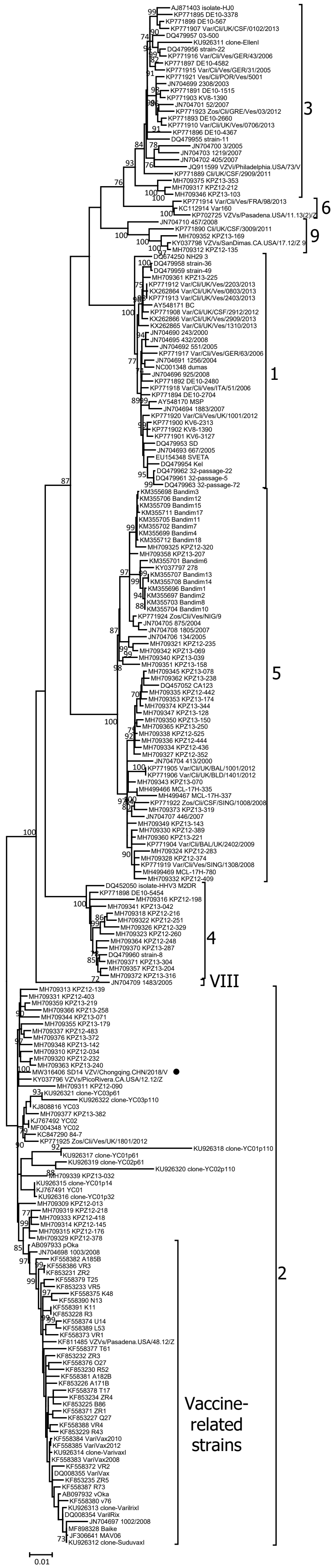
AJ871403 isolate-HJ0
KP771895 DE10-3378
KP771899 DE10-567
KP771907 Var/Cli/UK/CSF/0102/2013
DQ479957 03-500
KU926311 clone-Ellenl
DQ479956 strain-22
KP771916 Var/Cli/Ves/GER/43/2006
KP771897 DE10-4582
KP771915 Var/Cli/Ves/GER/31/2005
KP771921 Ves/Cli/POR/Ves/5001
JN704699 2308/2003
KP771891 DE10-1515
KP771903 KV8-1390
JN704701 52/2007
KP771923 Zos/Cli/GRE/Ves/03/2012
KP771893 DE10-2660
KP771910 Var/Cli/UK/Ves/0706/2013
KP771896 DE10-4367
DQ479955 strain-11
JN704700 3/2005
JN704703 1219/2007
JN704702 405/2007
JQ911599 VZVi/Philadelphia.USA/73/V
KP771889 Cli/UK/CSF/2909/2011
MH709375 KPZ13-353
MH709317 KPZ12-212
MH709346 KPZ13-103
3
KP771914 Var/Cli/Ves/FRA/98/2013
KC112914 Var160
KP702725 VZVs/Pasadena.USA/11.13(2)/Z
6
JN704710 457/2008
KP771890 Cli/UK/CSF/3009/2011
MH709352 KPZ13-169
KY037798 VZVs/SanDimas.CA.USA/17.12/Z 9
MH709312 KPZ12-135
9
DQ674250 NH29 3
DQ479958 strain-36
DQ479959 strain-49
MH709361 KPZ13-225
KP771912 Var/Cli/UK/Ves/2203/2013
KX262864 Var/Cli/UK/Ves/0803/2013
KP771913 Var/Cli/UK/Ves/2403/2013
AY548171 BC
KP771908 Var/Cli/UK/CSF/2912/2012
KX262866 Var/Cli/UK/Ves/2909/2013
KX262865 Var/Cli/UK/Ves/1310/2013
JN704690 243/2000
JN704695 432/2008
JN704692 551/2005
KP771917 Var/Cli/Ves/GER/63/2006
JN704691 1256/2004
NC001348 dumas
JN704696 925/2008
KP771892 DE10-2480
KP771918 Var/Cli/Ves/ITA/51/2006
KP771894 DE10-2704
AY548170 MSP
JN704694 1883/2007
KP771920 Var/Cli/Ves/UK/1001/2012
KP771900 KV6-2313
KP771902 KV8-1390
KP771901 KV6-3127
DQ479953 SD
JN704693 667/2005
EU154348 SVETA
DQ479954 Kel
DQ479962 32-passage-22
DQ479961 32-passage-5
DQ479963 32-passage-72
1
KM355698 Bandim3
KM355706 Bandim12
KM355709 Bandim15
KM355711 Bandim17
KM355705 Bandim11
KM355702 Bandim7
KM355699 Bandim4
KM355712 Bandim18
MH709325 KPZ12-320
MH709358 KPZ13-207
KM355701 Bandim6
KY037797 278
KM355707 Bandim13
KM355708 Bandim14
KM355696 Bandim1
KM355697 Bandim2
KM355703 Bandim8
KM355704 Bandim10
KP771924 Zos/Cli/Ves/NIG/9
JN704705 875/2004
JN704708 1805/2007
JN704706 134/2005
MH709321 KPZ12-235
MH709342 KPZ13-069
MH709340 KPZ13-039
MH709351 KPZ13-158
MH709345 KPZ13-078
MH709362 KPZ13-238
DQ457052 CA123
MH709335 KPZ12-442
MH709353 KPZ13-174
MH709374 KPZ13-344
MH709347 KPZ13-128
MH709350 KPZ13-150
MH709365 KPZ13-250
MH709338 KPZ12-525
MH709336 KPZ12-444
MH709334 KPZ12-436
MH709327 KPZ12-352
JN704704 413/2000
KP771905 Var/Cli/UK/BAL/1001/2012
KP771906 Var/Cli/UK/BLD/1401/2012
MH709343 KPZ13-070
MH499466 MCL-17H-335
MH499467 MCL-17H-337
KP771922 Zos/Cli/CSF/SING/1008/2008
MH709373 KPZ13-319
JN704707 446/2007
MH709349 KPZ13-143
MH709330 KPZ12-389
MH709360 KPZ13-221
KP771904 Var/Cli/BAL/UK/2402/2009
MH709324 KPZ12-283
MH709328 KPZ12-374
KP771919 Var/Cli/Ves/SING/1308/2008
MH499469 MCL-17H-780
MH709332 KPZ12-409
5
DQ452050 isolate-HHV3 M2DR
KP771898 DE10-5454
MH709316 KPZ12-198
MH709341 KPZ13-042
MH709318 KPZ12-216
MH709322 KPZ12-251
MH709326 KPZ12-329
MH709323 KPZ12-260
MH709364 KPZ12-248
MH709370 KPZ13-287
DQ479960 strain-8
MH709371 KPZ13-304
MH709357 KPZ13-204
MH709372 KPZ13-316
4
JN704709 1483/2005
VIII
MH709313 KPZ12-139
MH709331 KPZ12-403
MH709359 KPZ13-219
MH709366 KPZ13-258
MH709344 KPZ13-071
MH709355 KPZ13-179
MH709337 KPZ12-483
MH709376 KPZ13-372
MH709348 KPZ13-142
MH709310 KPZ12-034
MH709320 KPZ12-232
MH709363 KPZ13-240
MW316406 SD14 VZV/Chongqing.CHN/2018/V
KY037796 VZVs/PicoRivera.CA.USA/12.12/Z
MH709311 KPZ12-090
KU926321 clone-YC03p61
KU926322 clone-YC03p110
KJ808816 YC03
MH709377 KPZ13-382
KJ767492 YC02
MF004348 YC02
KC847290 84-7
KP771925 Zos/Cli/Ves/UK/1801/2012
KU926318 clone-YC01p110
KU926317 clone-YC01p61
KU926319 clone-YC02p61
KU926320 clone-YC02p110
MH709339 KPZ13-032
KU926315 clone-YC01p14
KJ767491 YC01
KU926316 clone-YC01p32
MH709309 KPZ12-013
MH709319 KPZ12-218
MH709333 KPZ12-418
MH709314 KPZ12-145
MH709315 KPZ12-176
MH709329 KPZ12-378
AB097933 pOka
JN704698 1003/2008
KF558382 A185B
KF558386 VR3
KF853231 ZR2
KF558379 T25
KF853233 VR5
KF558375 K48
KF558390 N13
KF558391 K11
KF853228 R3
KF558374 U14
KF558389 L53
KF558373 VR1
KF811485 VZVs/Pasadena.USA/48.12/Z
KF558377 T61
KF853232 ZR3
KF558376 O27
KF853230 R52
KF558381 A182B
KF853226 A171B
KF558378 T17
KF853234 ZR4
KF853225 B86
KF558371 ZR1
KF853227 Q27
KF558388 VR4
KF853229 R43
KF558384 VariVax2010
KF558385 VariVax2012
KU926314 clone-Varivaxl
KF558383 VariVax2008
KF558372 VR2
DQ008355 VariVax
KF853235 ZR5
KF558387 R73
AB097932 vOka
KF558380 v76
KU926313 clone-Varilrixl
DQ008354 VarilRix
JN704697 1002/2008
MF898328 Baike
JF306641 MAV06
KU926312 clone-Suduvaxl
2
Vaccine-related strains
0.01

Figure S2

Figure S2: ML phylogenetic tree of 2294 concatenated SNPs from 223 VZV genomes. Clades are indicated in brackets. Strains related to the vaccine strain Oka are also indicated in bracket SD14 *de novo* sequence is indicated by a black circle. Bootstrap values greater than 70% are indicated. The corresponding NJ tree is shown in Figure 1.

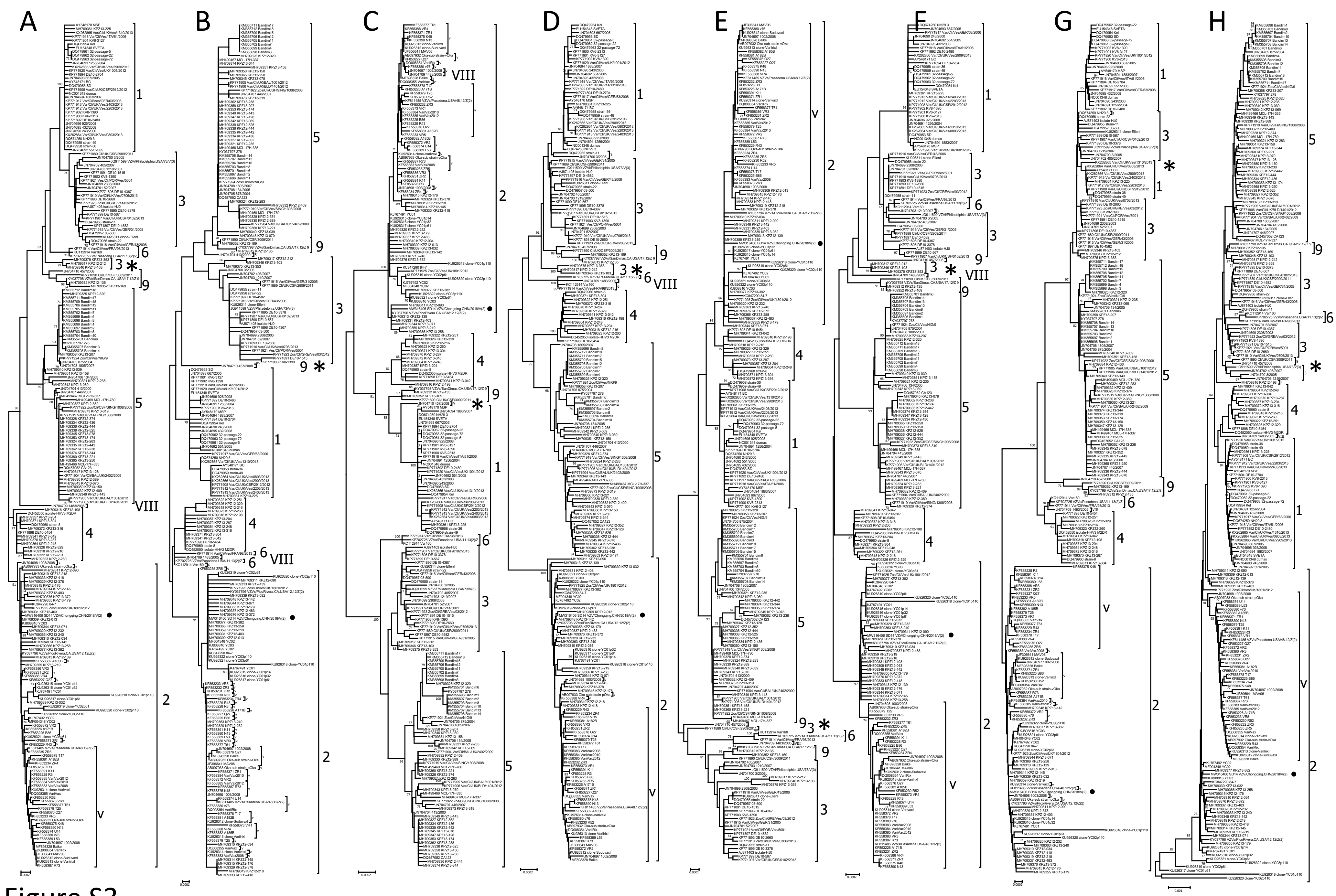

Figure S3

Figure S3: NJ phylogenetic trees on 8 genomic regions (A to H). Genomic regions were previously described (Jensen et al., 2017). Briefly, fragments A, B, C D (5') and H (3') are defined by repeat elements. Fragments D (3'), E, F, G and H (5') are defined by start/stop codon of ORFs. Sequences are identified based on the clade determined in the concatenated SNP tree in Figure 1. Putative interclade recombination events are identified with *. These concern sequences from clades 3, 6 and 9. SD14 did not appear to be involved in any interclade recombination event.

Supplementary Table 1: list of the 222 VZV genomes analyzed in this study

VZV strain	GenBank ID	Clade based on NJ tree (Figure	Length	Country	Year	Sample	Disease	Reference
Dumas	NC_001348	1	124,884	The Netherlands	Late 1970s	Unknown	Varicella	Davison, A.J., Scott, J.E., 1986. The complete DNA sequence of varicella-zoster virus. J Gen Virol. 67, 1759-1816. doi: 1710.1099/0022-1317-1767-1759-1759.
MSP	AY548170	1	124,883	Minnesota, USA	1995	Vesicle fluid	Varicella	Santos, R.A., Padilla, J.A., Hatfield, C., Grose, C., 1998. Antigenic variation of varicella zoster virus Fc receptor gE: loss of a major B cell epitope in the ectodomain. Virology. 249, 21-31. doi: 10.1006/viro.1998.9313.
BC	AY548171	1	125,459	British Columbia, Canada	1999	Vesicle fluid	Zoster	Tipples, G.A., Stephens, G.M., Sherlock, C., Bowler, M., Hoy, B., Cook, D., Grose, C., 2002. New variant of varicella-zoster virus. Emerg Infect Dis. 8, 1504-1505. doi: 1510.3201/eid0812.020118.
SD	DQ479953	1	125,087	South Dakota,USA	1980	Unknown	Unknown	Peters, G.A., Tyler, S.D., Grose, C., Severini, A., Gray, M.J., Upton, C., Tipples, G.A., 2006. A full-genome phylogenetic analysis of varicella-zoster virus reveals a novel origin of replication-based genotyping scheme and evidence of recombination between major circulating clades. J Virol. 80, 9850-9860. doi: 9810.1128/JVI.00715-00706.
Kel	DQ479954	1	125,374	Iowa,USA	2002	Unknown	Zoster	
strain 36	DQ479958	1	125,030	New Brunswick,	1998	Unknown	Varicella	
strain 49	DQ479959	1	125,041	New Brunswick,	1999	Unknown	Varicella	
32 passage 5	DQ479961	1	124,945	Texas,USA	1976	Unknown	Varicella	
32 passage 22	DQ479962	1	125,084	Texas,USA	1976	Unknown	Varicella	
32 passage 72	DQ479963	1	125,169	Texas,USA	1976	Unknown	Varicella	
NH29_3	DQ674250	1	124,811	NH, USA	2000	Unknown	Varicella	Loparev,V.N.; Unpublished; NCID, Centers for Disease Control and Prevention, 1600 Clifton Rd, Atlanta, GA 30333, USA
SVETA	EU154348	1	124,813	Russia	1999	Vesicle fluid	Varicella	Loparev, V.N., Rubtcova, E.N., Bostik, V., Tzaneva, V., Sauerbrei, A., Robo, A., Sattler-Dornbacher, E., Hanovcova, I., Stepanova, V., Splino, M., Eremin, V., Koskiniemi, M., Vankova, O.E., Schmid, D.S., 2009. Distribution of varicella-zoster virus (VZV) wild-type genotypes in northern and southern Europe: evidence for high conservation of circulating genotypes. Virology. 383, 216-225. doi: 210.1016/j.virol.2008.1010.1026. Epub 2008 Nov 1020.
243/2000	JN704690	1	124,845	Germany	2000	Unknown	Zoster	Zell, R., Taudien, S., Pfaff, F., Wutzler, P., Platzer, M., Sauerbrei, A., 2012. Sequencing of 21 varicella-zoster virus genomes reveals two novel genotypes and evidence of recombination. J Virol. 86, 1608-1622. doi: 1610.1128/JVI.06233-06211. Epub 02011 Nov 06230.
1256/2004	JN704691	1	124,757	Germany	2004	Unknown	Zoster	
551/2005	JN704692	1	124,688	Germany	2005	Unknown	Varicella	
667/2005	JN704693	1	124,884	Germany	2005	Unknown	Varicella	
1883/2007	JN704694	1	124,851	Germany	2007	Unknown	Varicella	
432/2008	JN704695	1	124,867	Germany	2008	Unknown	Zoster	
925/2008	JN704696	1	124,848	Germany	2008	Unknown	Varicella	
Var/Cli/UK/Ves/0803/2013	KX262864	1	124,852	United Kingdom	2013	Vesicle fluid	Varicella	Depledge, D.P., Brown, J., Macanovic, J., Underhill, G., Breuer, J., 2016. Viral Genome Sequencing Proves Nosocomial Transmission of Fatal Varicella. J Infect Dis. 214, 1399-1402. doi: 1310.1093/infdis/jiw1398. Epub 2016 Aug 1328.
Var/Cli/UK/Ves/1310/2013	KX262865	1	124,862	United Kingdom	2013	Vesicle fluid	Varicella	
Var/Cli/UK/Ves/2909/2013	KX262866	1	124,873	United Kingdom	2013	Vesicle fluid	Varicella	
DE10-2480	KP771892	1	124864	Sweden	2010	Vesicle fluid	Zoster	Norberg, P., Depledge, D.P., Kundu, S., Atkinson, C., Brown, J., Haque, T., Hussaini, Y., MacMahon, E., Molyneaux, P., Papaevangelou, V., Sengupta, N., Koay, E.S., Tang, J.W., Underhill, G.S., Grahn, A., Studahl, M., Breuer, J., Bergström, T., 2015. Recombination of Globally Circulating Varicella-Zoster Virus. J Virol. 89, 7133-7146. doi: 7110.1128/JVI.00437-00415.
DE10-2704	KP771894	1	124864	Sweden	2010	Vesicle fluid	Zoster	
KV6-2313	KP771901	1	124855	Sweden	2010	CSF	Encephalitis	
KV6-3127	KP771902	1	124836	Sweden	2010	CSF	Encephalitis	
KV8-1390	KP771903	1	124878	Sweden	2010	CSF	Encephalitis	
Var/Cli/UK/CSF/2912/2012	KP771908	1	124852	United Kingdom	2012	CSF	Varicella with	
Var/Cli/UK/Ves/2203/2013	KP771912	1	124818	United Kingdom	2013	Vesicle fluid	Varicella	
Var/Cli/UK/Ves/2403/2013	KP771913	1	124758	United Kingdom	2013	Vesicle fluid	Varicella	
Var/Cli/Ves/GER/63/2006	KP771917	1	124846	Germany	2006	Vesicle fluid	Varicella	
Var/Cli/Ves/ITA/51/2006	KP771918	1	124858	Italy	2006	Vesicle fluid	Varicella	
Var/Cli/Ves/UK/1001/2012	KP771920	1	124874	United Kingdom	2012	Vesicle fluid	Varicella	
KPZ13-225	MH709361	1	125,127	USA	2013	Unknown	Zoster	Jensen, N.J., Depledge, D.P., Ng, T.F.F., Leung, J., Quinlivan, M., Radford, K.W., Folster, J., Tseng, H.F., LaRussa, P., Jacobsen, S.J., Breuer, J., Schmid, D.S., 2020. Analysis of the reiteration regions (R1 to R5) of varicella-zoster virus. Virology. 546:38-50., 10.1016/j.virol.2020.1003.1008. Epub 2020 Apr 1014.
84-7	KC847290	2	125,083	China	1984	Unknown		Li Xiuling, Wang Xiaoxiao, Zhang zhongyang, Hao Chunsheng, Zhang Chen, He Weiwei.Sequencing of Whole Genome of Varicella-Zoster Virus 84-7 Strain[J].Zhongguo Sheng Wu Zhi Pin Xue Za Zhi,2010,23(01):1-8.
YC01	KJ767491	2	125,144	South Korea	2012	Unknown	Zoster	Kim,J.I., Ji,G.Y., Park,H.S. and Lee,C.H. unpublished. Microbiology, Chungbuk National University, 52 Naesudong-Ro, Cheongju, Chungbuk 361-763, Korea
YC02	KJ767492	2	125,150	South Korea	2012	Unknown	Varicella	
YC03	KJ808816	2	125,162	South Korea	2012	Unknown	Zoster	Kim, J.A., Park, S.K., Kumar, M., Lee, C.H., Shin, O.S., 2015. Insights into the role of immunosenescence during varicella zoster virus infection (shingles) in the aging cell model. Oncotarget. 6, 35324-35343. doi: 35310.18632/oncotarget.36117.
clone YC01p14	KU926315	2	125,135	South Korea	2012	Unknown	Zoster	Jeon, J.S., Won, Y.H., Kim, I.K., Ahn, J.H., Shin, O.S., Kim, J.H., Lee, C.H., 2016. Analysis of single nucleotide polymorphism among Varicella-Zoster Virus and identification of vaccine-specific sites. Virology. 496:277-286., 10.1016/j.virol.2016.1006.1017. Epub 2016 Jul 1011.
clone YC01p32	KU926316	2	125,134	South Korea	2012	Unknown	Zoster	
clone YC01p61	KU926317	2	125,138	South Korea	2012	Unknown	Zoster	
clone YC01p110	KU926318	2	125,120	South Korea	2012	Unknown	Zoster	
clone YC02p61	KU926319	2	125,121	South Korea	2012	Unknown	Varicella	
clone YC02p110	KU926320	2	125,131	South Korea	2012	Unknown	Varicella	
clone YC03p61	KU926321	2	125,150	South Korea	2012	Unknown	Varicella	
clone YC03p110	KU926322	2	125,141	South Korea	2012	Unknown	Varicella	

Supplementary Table 1: list of the 222 VZV genomes analyzed in this study

VZV strain	GenBank ID	Clade based on NJ tree (Figure	Length	Country	Year	Sample	Disease	Reference
Zos/Cli/Ves/UK/1801/2012	KP771925	2	124,882	United Kingdom	2012	Vesicle fluid	Zoster	Norberg, P., Depledge, D.P., Kundu, S., Atkinson, C., Brown, J., Haque, T., Hussaini, Y., MacMahon, E., Molyneaux, P., Papaevangelou, V., Sengupta, N., Koay, E.S., Tang, J.W., Underhill, G.S., Grahn, A., Studahl, M., Breuer, J., Bergström, T., 2015. Recombination of Globally Circulating Varicella-Zoster Virus. J Virol. 89, 7133-7146. doi: 7110.1128/JVI.00437-00415.
'092	KY037796	2	125,220	USA	2012	Skin lesion	Zoster	Jensen, N.J., Rivailler, P., Tseng, H.F., Quinlivan, M.L., Radford, K., Folster, J., Harpaz, R., LaRussa, P., Jacobsen, S., Scott Schmid, D., 2017. Revisiting the genotyping scheme for varicella-zoster viruses based on whole-genome comparisons. J Gen Virol. 98, 1434-1438. doi: 1410.1099/jgv.1430.000772. Epub 002017 Jun 000714.
YC02	MF004348	2	125,134	South Korea	2012	Unknown	Varicella	Won,Y.H., Park,J.S., Kang,J.H., Jeon,J.S., Ahn,J.H., Song,M.J., Ok Sarah,O.S. and Lee,C.H. unpublished. Microbiology, Chungbuk National University, 1, Chungdae-ro, Seowon-gu, Cheongju, Chungbuk 28644, Korea
KPZ12-013	MH709309	2	125,289	USA	2012	Skin lesion	Zoster	Jensen, N.J., Depledge, D.P., Ng, T.F.F., Leung, J., Quinlivan, M., Radford, K.W., Folster, J., Tseng, H.F., LaRussa, P., Jacobsen, S.J., Breuer, J., Schmid, D.S., 2020. Analysis of the reiteration regions (R1 to R5) of varicella-zoster virus. Virology. 546:38-50., 10.1016/j.virol.2020.1003.1008. Epub 2020 Apr 1014.
KPZ12-034	MH709310	2	125,073	USA	2012	Skin lesion	Zoster	
KPZ12-090	MH709311	2	125,196	USA	2012	Skin lesion	Zoster	
KPZ12-139	MH709313	2	124,751	USA	2012	Skin lesion	Zoster	
KPZ12-145	MH709314	2	125,187	USA	2012	Skin lesion	Zoster	
KPZ12-176	MH709315	2	125,303	USA	2012	Skin lesion	Zoster	
KPZ12-218	MH709319	2	125,082	USA	2012	Skin lesion	Zoster	
KPZ12-232	MH709320	2	124,965	USA	2012	Skin lesion	Zoster	
KPZ12-378	MH709329	2	124,922	USA	2012	Skin lesion	Zoster	
KPZ12-403	MH709331	2	125,143	USA	2012	Skin lesion	Zoster	
KPZ12-418	MH709333	2	125,030	USA	2012	Skin lesion	Zoster	
KPZ12-483	MH709337	2	125,231	USA	2012	Skin lesion	Zoster	
KPZ13-032	MH709339	2	125,032	USA	2013	Skin lesion	Zoster	
KPZ13-071	MH709344	2	125,045	USA	2013	Skin lesion	Zoster	
KPZ13-142	MH709348	2	125,220	USA	2013	Skin lesion	Zoster	
KPZ13-179	MH709355	2	125,176	USA	2013	Skin lesion	Zoster	
KPZ13-219	MH709359	2	124,940	USA	2013	Skin lesion	Zoster	
KPZ13-240	MH709363	2	124,992	USA	2019	Skin lesion	Zoster	
KPZ13-258	MH709366	2	124,928	USA	2013	Skin lesion	Zoster	
KPZ13-372	MH709376	2	124,981	USA	2013	Skin lesion	Zoster	
KPZ13-382	MH709377	2	124,999	USA	2013	Skin lesion	Zoster	
isolate HJ0	AJ871403	3	124,928	Erlangen, Germany	Unknown	Unknown	Unknown	Fickenscher,H. unpublished. Department of Virology, University of Heidelberg Medical School, Im Neuenheimer Feld 324, D-69120, GERMANY
strain 11	DQ479955	3	125,370	New Brunswick,	1996	Unknown	Zoster	Peters, G.A., Tyler, S.D., Grose, C., Severini, A., Gray, M.J., Upton, C., Tipples, G.A., 2006. A full-genome phylogenetic analysis of varicella-zoster virus reveals a novel origin of replication-based genotyping scheme and evidence of recombination between major circulating clades. J Virol. 80, 9850-9860. doi: 9810.1128/JVI.00715-00706.
strain 22	DQ479956	3	124,868	New Brunswick,	1998	Unknown	Zoster	
03-500	DQ479957	3	125,239	Alberta, Canada	2003	Unknown	Unknown	
2308/2003	JN704699	3	124,847	Germany	2003	Unknown	Varicella	Zell, R., Taudien, S., Pfaff, F., Wutzler, P., Platzer, M., Sauerbrei, A., 2012. Sequencing of 21 varicella-zoster virus genomes reveals two novel genotypes and evidence of recombination. J Virol. 86, 1608-1622. doi: 1610.1128/JVI.06233-06211. Epub 02011 Nov 06230.
3/2005	JN704700	3	124,756	Germany	2005	Unknown	Varicella	
52/2007	JN704701	3	124,816	Germany	2007	Unknown	Zoster	
405/2007	JN704702	3	124,697	Germany	2007	Unknown	Varicella	
1219/2007	JN704703	3	124,617	Germany	2007	Unknown	Zoster	
clone EllenI	KU926311	3	124,789	South Korea	2014	Unknown	Varicella	Jeon, J.S., Won, Y.H., Kim, I.K., Ahn, J.H., Shin, O.S., Kim, J.H., Lee, C.H., 2016. Analysis of single nucleotide polymorphism among Varicella-Zoster Virus and identification of vaccine-specific sites. Virology. 496:277-286., 10.1016/j.virol.2016.1006.1017. Epub 2016 Jul 1011.
Cli/UK/CSF/2909/2011	KP771889	3	124844	United Kingdom	2011	CSF	Encephalitis	Norberg, P., Depledge, D.P., Kundu, S., Atkinson, C., Brown, J., Haque, T., Hussaini, Y., MacMahon, E., Molyneaux, P., Papaevangelou, V., Sengupta, N., Koay, E.S., Tang, J.W., Underhill, G.S., Grahn, A., Studahl, M., Breuer, J., Bergström, T., 2015. Recombination of Globally Circulating Varicella-Zoster Virus. J Virol. 89, 7133-7146. doi: 7110.1128/JVI.00437-00415.
DE10-1515	KP771891	3	124766	Sweden	2010	Vesicle fluid	Zoster	
DE10-2660	KP771893	3	124859	Sweden	2010	Vesicle fluid	Zoster	
DE10-3378	KP771895	3	124875	Sweden	2010	Vesicle fluid	Zoster	
DE10-4367	KP771896	3	124864	Sweden	2010	Vesicle fluid	Zoster	
DE10-4582	KP771897	3	124866	Sweden	2010	Vesicle fluid	Zoster	
DE10-567	KP771899	3	124878	Sweden	2010	Vesicle fluid	Zoster	
KV8-1390	KP771903	3	124878	Sweden	2010	CSF	Encephalitis	
Var/Cli/UK/CSF/0102/2013	KP771907	3	124854	United Kingdom	2013	CSF	Varicella	
Var/Cli/UK/Ves/0706/2013	KP771910	3	124799	United Kingdom	2013	Vesicle fluid	Zoster with encephalitis	
Var/Cli/Ves/GER/31/2005	KP771915	3	124858	Germany	2005	Vesicle fluid	Varicella	
Var/Cli/Ves/GER/43/2006	KP771916	3	124827	Germany	2010	Vesicle fluid	Varicella	
Ves/Cli/POR/Ves/5001	KP771921	3	124792	Portugal	2010	Vesicle fluid	Varicella	
Zos/Cli/GRE/Ves/03/2012	KP771923	3	124855	Greece	2013	Vesicle fluid	Zoster	

Supplementary Table 1: list of the 222 VZV genomes analyzed in this study

VZV strain	GenBank ID	Clade based on NJ tree (Figure	Length	Country	Year	Sample	Disease	Reference
VZVi/Philadelphia.USA/73/V[3]	JQ911599	3	124,788	Philadelphia, USA	1973	Unknown	Varicella	Madupu,R., Halpin,R., Fedorova,N., Stockwell,T., Amedeo,P., Bishop,B., Edworthy,P., Gupta,N., Katzel,D., Li,K., Schobel,S., Shrivastava,S., Thovarai,V., Wang,S., Folster,J., Radford,K., Schmid,S., Wentworth,D.E. and Bellini,W. unpublished. J. Craig Venter Institute, 9704 Medical Center Drive, Rockville, MD 20850, USA
KPZ12-212	MH709317	3	125,305	USA	2012	Skin lesion	Zoster	Jensen, N.J., Depledge, D.P., Ng, T.F.F., Leung, J., Quinlivan, M., Radford, K.W., Folster, J., Tseng, H.F., LaRussa, P., Jacobsen, S.J., Breuer, J., Schmid, D.S., 2020. Analysis of the reiteration regions (R1 to R5) of varicella-zoster virus. Virology. 546:38-50., 10.1016/j.virol.2020.1003.1008. Epub 2020 Apr 1014.
KPZ13-103	MH709346	3	124,844	USA	2013	Skin lesion	Zoster	
KPZ13-353	MH709375	3	125,016	USA	2013	Skin lesion	Zoster	
isolate HHV3_M2DR	DQ452050	4	124,770	Morocco	2000	Unknown	Varicella	Norberg, P., Liljeqvist, J.A., Bergström, T., Sammons, S., Schmid, D.S., Loparev, V.N., 2006. Complete-genome phylogenetic approach to varicella-zoster virus evolution: genetic divergence and evidence for recombination. J Virol. 80, 9569-9576. doi: 9510.1128/JVI.00835-00806.
strain 8	DQ479960	4	125,451	New Brunswick, Canada	1995	Unknown	Zoster	Peters, G.A., Tyler, S.D., Grose, C., Severini, A., Gray, M.J., Upton, C., Tipples, G.A., 2006. A full-genome phylogenetic analysis of varicella-zoster virus reveals a novel origin of replication-based genotyping scheme and evidence of recombination between major circulating clades. J Virol. 80, 9850-9860. doi: 9810.1128/JVI.00715-00706.
DE10-5454	KP771898	4	124,856	Sweden	2010	Vesicle fluid	Zoster	Norberg, P., Depledge, D.P., Kundu, S., Atkinson, C., Brown, J., Haque, T., Hussaini, Y., MacMahon, E., Molyneaux, P., Papaevangelou, V., Sengupta, N., Koay, E.S., Tang, J.W., Underhill, G.S., Grahn, A., Studahl, M., Breuer, J., Bergström, T., 2015. Recombination of Globally Circulating Varicella-Zoster Virus. J Virol. 89, 7133-7146. doi: 7110.1128/JVI.00437-00415.
KPZ12-198	MH709316	4	124,776	USA	2012	Skin lesion	Zoster	Jensen, N.J., Depledge, D.P., Ng, T.F.F., Leung, J., Quinlivan, M., Radford, K.W., Folster, J., Tseng, H.F., LaRussa, P., Jacobsen, S.J., Breuer, J., Schmid, D.S., 2020. Analysis of the reiteration regions (R1 to R5) of varicella-zoster virus. Virology. 546:38-50., 10.1016/j.virol.2020.1003.1008. Epub 2020 Apr 1014.
KPZ12-216	MH709318	4	125,234	USA	2012	Skin lesion	Zoster	
KPZ12-251	MH709322	4	125,150	USA	2012	Skin lesion	Zoster	
KPZ12-260	MH709323	4	125,056	USA	2012	Skin lesion	Zoster	
KPZ12-329	MH709326	4	124,987	USA	2012	Skin lesion	Zoster	
KPZ13-042	MH709341	4	124,852	USA	2013	Skin lesion	Zoster	
KPZ13-204	MH709357	4	125,032	USA	2013	Skin lesion	Zoster	
KPZ13-248	MH709364	4	124,779	USA	2013	Skin lesion	Zoster	
KPZ13-287	MH709370	4	125,075	USA	2013	Skin lesion	Zoster	
KPZ13-304	MH709371	4	124,776	USA	2013	Skin lesion	Zoster	
KPZ13-316	MH709372	4	124,942	USA	2013	Skin lesion	Zoster	
CA123	DQ457052	5	124,771	California, USA	1990s	Vesicle fluid	Varicella	Loparev, V.N., Rubtcova, E.N., Bostik, V., Govil, D., Birch, C.J., Druce, J.D., Schmid, D.S., Croxson, M.C., 2007. Identification of five major and two minor genotypes of varicella-zoster virus strains: a practical two-amplicon approach used to genotype clinical isolates in Australia and New Zealand. J Virol. 81, 12758-12765. doi: 12710.11128/JVI.01145-12707. Epub 12007 Sep 12726.
413/2000	JN704704	5	124,838	Germany	2000	Unknown	Varicella	Zell, R., Taudien, S., Pfaff, F., Wutzler, P., Platzer, M., Sauerbrei, A., 2012. Sequencing of 21 varicella-zoster virus genomes reveals two novel genotypes and evidence of recombination. J Virol. 86, 1608-1622. doi: 1610.1128/JVI.06233-06211. Epub 02011 Nov 06230.
875/2004	JN704705	5	124,844	Germany	2004	Unknown	Varicella	
134/2005	JN704706	5	124,846	Germany	2005	Unknown	Varicella	
446/2007	JN704707	5	124,861	Germany	2007	Unknown	Varicella	
1805/2007	JN704708	5	124,848	Germany	2007	Unknown	Varicella	
Var/Cli/BAL/UK/2402/2009	KP771904	5	124,862	United Kingdom	2009	Bronchoalveolar	Varicella	Norberg, P., Depledge, D.P., Kundu, S., Atkinson, C., Brown, J., Haque, T., Hussaini, Y., MacMahon, E., Molyneaux, P., Papaevangelou, V., Sengupta, N., Koay, E.S., Tang, J.W., Underhill, G.S., Grahn, A., Studahl, M., Breuer, J., Bergström, T., 2015. Recombination of Globally Circulating Varicella-Zoster Virus. J Virol. 89, 7133-7146. doi: 7110.1128/JVI.00437-00415.
Var/Cli/UK/BAL/1001/2012	KP771905	5	124,829	United Kingdom	2012	Bronchoalveolar	Varicella	
Var/Cli/UK/BLD/1401/2012	KP771906	5	124,850	United Kingdom	2012	Blood	Varicella	
Var/Cli/Ves/SING/1308/2008	KP771919	5	124832	Singapore	2008	Vesicle fluid	Varicella	
Zos/Cli/CSF/SING/1008/2008	KP771922	5	124824	Singapore	2008	CSF	Zoster and cerebellitis	
Zos/Cli/Ves/NIG/9	KP771924	5	124867	Nigeria	2010	Vesicle fluid	Zoster	
Bandim1	KM355696	5	125,039	Guinea-Bissau	2001	Vesicle fluid	Varicella	Depledge, D.P., Gray, E.R., Kundu, S., Cooray, S., Poulsen, A., Aaby, P., Breuer, J., 2014a. Evolution of cocirculating varicella-zoster virus genotypes during a chickenpox outbreak in Guinea-Bissau. J Virol. 88, 13936-13946. doi: 13910.11128/JVI.02337-13914. Epub 12014 Oct 13931.
Bandim2	KM355697	5	124,843	Guinea-Bissau	2001	Vesicle fluid	Varicella	
Bandim3	KM355698	5	125,212	Guinea-Bissau	2001	Vesicle fluid	Varicella	
Bandim4	KM355699	5	125,212	Guinea-Bissau	2001	Vesicle fluid	Varicella	
Bandim6	KM355701	5	125,065	Guinea-Bissau	2001	Vesicle fluid	Varicella	
Bandim7	KM355702	5	124,830	Guinea-Bissau	2001	Vesicle fluid	Varicella	
Bandim8	KM355703	5	124,826	Guinea-Bissau	2001	Vesicle fluid	Varicella	
Bandim10	KM355704	5	124,783	Guinea-Bissau	2001	Vesicle fluid	Varicella	
Bandim11	KM355705	5	125,208	Guinea-Bissau	2001	Vesicle fluid	Varicella	
Bandim12	KM355706	5	125,212	Guinea-Bissau	2001	Vesicle fluid	Varicella	
Bandim13	KM355707	5	125,060	Guinea-Bissau	2001	Vesicle fluid	Varicella	
Bandim14	KM355708	5	125,065	Guinea-Bissau	2001	Vesicle fluid	Varicella	
Bandim15	KM355709	5	125,234	Guinea-Bissau	2001	Vesicle fluid	Varicella	
Bandim17	KM355711	5	125,234	Guinea-Bissau	2001	Vesicle fluid	Varicella	
Bandim18	KM355712	5	124,810	Guinea-Bissau	2001	Vesicle fluid	Varicella	

Supplementary Table 1: list of the 222 VZV genomes analyzed in this study

VZV strain	GenBank ID	Clade based on NJ tree (Figure	Length	Country	Year	Sample	Disease	Reference
278	KY037797	5	124,840	USA	2014	Skin lesion	Zoster	Jensen, N.J., Rivailler, P., Tseng, H.F., Quinlivan, M.L., Radford, K., Folster, J., Harpaz, R., LaRussa, P., Jacobsen, S., Scott Schmid, D., 2017. Revisiting the genotyping scheme for varicella-zoster viruses based on whole-genome comparisons. J Gen Virol. 98, 1434-1438. doi: 1410.1099/jgv.1430.000772. Epub 002017 Jun 000714.
MCL-17H-335	MH499466	5	124,832	Delhi, India	2017	Unknown	Unknown	Yadav,P.D. Unpublished. Maximum Containment Laboratory, National Institute of Virology, Pashan, Pune, Maharashtra 411021, India
MCL-17H-337	MH499467	5	121,562	India	2017	Unknown	Unknown	
MCL-17H-780	MH499469	5	123,769	India	2017	Unknown	Unknown	
KPZ12-235	MH709321	5	125,062	USA	2012	Skin lesion	Zoster	Jensen, N.J., Depledge, D.P., Ng, T.F.F., Leung, J., Quinlivan, M., Radford, K.W., Folster, J., Tseng, H.F., LaRussa, P., Jacobsen, S.J., Breuer, J., Schmid, D.S., 2020. Analysis of the reiteration regions (R1 to R5) of varicella-zoster virus. Virology. 546:38-50., 10.1016/j.virol.2020.1003.1008. Epub 2020 Apr 1014.
KPZ12-283	MH709324	5	125,091	USA	2012	Skin lesion	Zoster	
KPZ12-320	MH709325	5	125,072	USA	2012	Skin lesion	Zoster	
KPZ12-352	MH709327	5	124,806	USA	2012	Skin lesion	Zoster	
KPZ12-374	MH709328	5	124,859	USA	2012	Skin lesion	Zoster	
KPZ12-389	MH709330	5	124,716	USA	2012	Skin lesion	Zoster	
KPZ12-409	MH709332	5	124,984	USA	2012	Skin lesion	Zoster	
KPZ12-436	MH709334	5	124,845	USA	2012	Skin lesion	Zoster	
KPZ12-442	MH709335	5	124,911	USA	2012	Skin lesion	Zoster	
KPZ12-444	MH709336	5	124,899	USA	2012	Skin lesion	Zoster	
KPZ12-525	MH709338	5	125,065	USA	2012	Skin lesion	Zoster	
KPZ13-039	MH709340	5	125,194	USA	2013	Skin lesion	Zoster	
KPZ13-069	MH709342	5	124,915	USA	2013	Skin lesion	Zoster	
KPZ13-070	MH709343	5	124,886	USA	2013	Skin lesion	Zoster	
KPZ13-078	MH709345	5	124,808	USA	2013	Skin lesion	Zoster	
KPZ13-128	MH709347	5	125,007	USA	2013	Skin lesion	Zoster	
KPZ13-143	MH709349	5	125,103	USA	2013	Skin lesion	Zoster	
KPZ13-150	MH709350	5	125,037	USA	2013	Skin lesion	Zoster	
KPZ13-158	MH709351	5	124,613	USA	2013	Skin lesion	Zoster	
KPZ13-174	MH709353	5	124,967	USA	2013	Skin lesion	Zoster	
KPZ13-207	MH709358	5	125,123	USA	2013	Skin lesion	Zoster	
KPZ13-221	MH709360	5	124,994	USA	2013	Skin lesion	Zoster	
KPZ13-238	MH709362	5	124,996	USA	2013	Skin lesion	Zoster	
KPZ13-250	MH709365	5	124,846	USA	2013	Skin lesion	Zoster	
KPZ13-319	MH709373	5	124,951	USA	2013	Skin lesion	Zoster	
KPZ13-344	MH709374	5	125,069	USA	2013	Skin lesion	Zoster	
Var160	KC112914	6	124,884	Mexico	2007	Vesicle fluid	Varicella	Garcés-Ayala, F., Rodríguez-Castillo, A., Ortiz-Alcántara, J.M., Gonzalez-Durán, E., Segura-Candelas, J.M., Pérez-Agüeros, S.I., Escobar-Escamilla, N., Méndez-Tenorio, A., Diaz-Quiñonez, J.A., Ramirez-González, J.E., 2015. Full-Genome Sequence of a Novel Varicella-Zoster Virus Clade Isolated in Mexico. Genome Announc. 3, e00752-00715. doi: 00710.01128/genomeA.00752-00715.
Var/Cli/Ves/FRA/98/2013	KP771914	6	124822	France	2013	Vesicle fluid	Varicella	Norberg, P., Depledge, D.P., Kundu, S., Atkinson, C., Brown, J., Haque, T., Hussaini, Y., MacMahon, E., Molyneaux, P., Papaevangelou, V., Sengupta, N., Koay, E.S., Tang, J.W., Underhill, G.S., Grahn, A., Studahl, M., Breuer, J., Bergström, T., 2015. Recombination of Globally Circulating Varicella-Zoster Virus. J Virol. 89, 7133-7146. doi: 7110.1128/JVI.00437-00415.
VZVs/Pasadena.USA/11.13(2)/Z	KP702725	6	125,148	USA	2013	Skin lesion	Zoster	Jensen, N.J., Rivailler, P., Tseng, H.F., Quinlivan, M.L., Radford, K., Folster, J., Harpaz, R., LaRussa, P., Jacobsen, S., Scott Schmid, D., 2017. Revisiting the genotyping scheme for varicella-zoster viruses based on whole-genome comparisons. J Gen Virol. 98, 1434-1438. doi: 1410.1099/jgv.1430.000772. Epub 002017 Jun 000714.
457/2008	JN704710	9	124,472	Germany	2008	Unknown	Varicella	Zell, R., Taudien, S., Pfaff, F., Wutzler, P., Platzer, M., Sauerbrei, A., 2012. Sequencing of 21 varicella-zoster virus genomes reveals two novel genotypes and evidence of recombination. J Virol. 86, 1608-1622. doi: 1610.1128/JVI.06233-06211. Epub 02011 Nov 06230.
Cli/UK/CSF/3009/2011	KP771890	9		United Kingdom	2011	CSF	Encephalitis	Norberg, P., Depledge, D.P., Kundu, S., Atkinson, C., Brown, J., Haque, T., Hussaini, Y., MacMahon, E., Molyneaux, P., Papaevangelou, V., Sengupta, N., Koay, E.S., Tang, J.W., Underhill, G.S., Grahn, A., Studahl, M., Breuer, J., Bergström, T., 2015. Recombination of Globally Circulating Varicella-Zoster Virus. J Virol. 89, 7133-7146. doi: 7110.1128/JVI.00437-00415.
VZVs/SanDimas.CA.USA/17.12/Z_9	KY037798	9	125,213	USA	2012	Skin lesion	Zoster	Jensen, N.J., Rivailler, P., Tseng, H.F., Quinlivan, M.L., Radford, K., Folster, J., Harpaz, R., LaRussa, P., Jacobsen, S., Scott Schmid, D., 2017. Revisiting the genotyping scheme for varicella-zoster viruses based on whole-genome comparisons. J Gen Virol. 98, 1434-1438. doi: 1410.1099/jgv.1430.000772. Epub 002017 Jun 000714.
KPZ12-135	MH709312	9	124,930	USA	2012	Skin lesion	Zoster	Jensen, N.J., Depledge, D.P., Ng, T.F.F., Leung, J., Quinlivan, M., Radford, K.W., Folster, J., Tseng, H.F., LaRussa, P., Jacobsen, S.J., Breuer, J., Schmid, D.S., 2020. Analysis of the reiteration regions (R1 to R5) of varicella-zoster virus. Virology. 546:38-50., 10.1016/j.virol.2020.1003.1008. Epub 2020
KPZ13-169	MH709352	9	125,157	USA	2013	Skin lesion	Zoster	
1483/2005	JN704709	VIII	124,718	Germany	2005	Unknown	Varicella	Zell, R., Taudien, S., Pfaff, F., Wutzler, P., Platzer, M., Sauerbrei, A., 2012. Sequencing of 21 varicella-zoster virus genomes reveals two novel genotypes and evidence of recombination. J Virol. 86, 1608-1622. doi: 1610.1128/JVI.06233-06211. Epub 02011 Nov 06230.

Supplementary Table 1: list of the 222 VZV genomes analyzed in this study

VZV strain	GenBank ID	Clade based on NJ tree (Figure	Length	Country	Year	Sample	Disease	Reference
vOka	AB097932	vac	125,078	Japan	1976	Vaccine lot	N/A	Gomi, Y., Sunamachi, H., Mori, Y., Nagaike, K., Takahashi, M., Yamanishi, K., 2002. Comparison of the complete DNA sequences of the Oka varicella vaccine and its parental virus. J Virol. 76, 11447-11459. doi: 11410.11128/jvi.11476.11422.11447-11459.12002.
pOka	AB097933	vac	125,125	Japan	1970	Vesicle fluid	Varicella	
VarilRix	DQ008354	vac	124,821	Japan	1984	Vaccine lot	N/A	Tillieux, S.L., Halsey, W.S., Thomas, E.S., Voycik, J.J., Sathe, G.M., Vassilev, V., 2008. Complete DNA sequences of two oka strain varicella-zoster virus genomes. J Virol. 82, 11023-11044. doi: 11010.11128/JVI.00777-11008. Epub 12008 Sep 11010.
VariVax	DQ008355	vac	124,815	Japan	1995	Vaccine lot	N/A	
MAV06	JF306641	vac	124,758	South Korea	1989	Vaccine lot	N/A	Kim, J.I., Jung, G.S., Kim, Y.Y., Ji, G.Y., Kim, H.S., Wang, W.D., Park, H.S., Park, S.Y., Kim, G.H., Kwon, S.N., Lee, K.M., Ahn, J.H., Yoon, Y., Lee, C.H., 2011. Sequencing and characterization of Varicella-zoster virus vaccine strain SuduVax. Virol J. 8:547., 10.1186/1743-1422X-1188-1547.
1002/2008	JN704697	vac	124,814	Germany	2008	Vaccine lot	N/A	Zell, R., Taudien, S., Pfaff, F., Wutzler, P., Platzer, M., Sauerbrei, A., 2012. Sequencing of 21 varicella-zoster virus genomes reveals two novel genotypes and evidence of recombination. J Virol. 86, 1608-1622. doi: 1610.1128/JVI.06233-06211. Epub 02011 Nov 06230.
1003/2008	JN704698	vac	124,772	Germany	2008	Vesicle fluid	Varicella	
clone Suduvaxl	KU926312	vac	124,767	South Korea	2013	Vaccine lot	N/A	Jeon, J.S., Won, Y.H., Kim, I.K., Ahn, J.H., Shin, O.S., Kim, J.H., Lee, C.H., 2016. Analysis of single nucleotide polymorphism among Varicella-Zoster Virus and identification of vaccine-specific sites. Virology. 496:277-286., 10.1016/j.virol.2016.1006.1017. Epub 2016 Jul 1011.
clone Varilrixl	KU926313	vac	124,825	South Korea	2012	Vaccine lot	N/A	
clone Varivaxl	KU926314	vac	124,816	South Korea	2012	Vaccine lot	N/A	
ZR1	KF558371	vac	125,129	United Kingdom	2006	Skin lesion	Zoster	Depledge, D.P., Kundu, S., Jensen, N.J., Gray, E.R., Jones, M., Steinberg, S., Gershon, A., Kinchington, P.R., Schmid, D.S., Balloux, F., Nichols, R.A., Breuer, J., 2014b. Deep sequencing of viral genomes provides insight into the evolution and pathogenesis of varicella zoster virus and its vaccine in humans. Mol Biol Evol. 31, 397-409. doi: 310.1093/molbev/mst1210. Epub 2013 Oct 1025.
VR2	KF558372	vac	125,128	United Kingdom	2006	Skin lesion	Varicella	
VR1	KF558373	vac	125,125	USA	2007	Skin lesion	Varicella	
U14	KF558374	vac	125,128	USA	1997	Skin lesion	Zoster	
K48	KF558375	vac	125,125	USA	1995	Skin lesion	Zoster	
O27	KF558376	vac	125,128	USA	1999	Skin lesion	Varicella	
T61	KF558377	vac	125,125	USA	2001	Skin lesion	Zoster	
T17	KF558378	vac	125,129	USA	2000	Skin lesion	Zoster	
T25	KF558379	vac	125,125	USA	2000	Skin lesion	Zoster	
v76	KF558380	vac	125,128	USA	1988	Skin lesion	Varicella	
A182B	KF558381	vac	125,130	USA	1988	Skin lesion	Varicella	
A185B	KF558382	vac	125,130	USA	1988	Skin lesion	Varicella	
VariVax2008	KF558383	vac	125,125	USA	2008	Skin lesion	N/A	
VariVax2010	KF558384	vac	125,125	United Kingdom	2010	Skin lesion	N/A	
VariVax2012	KF558385	vac	125,125	United Kingdom	2012	Skin lesion	N/A	
VR3	KF558386	vac	125,124	United Kingdom	2006	Skin lesion	Varicella	
R73	KF558387	vac	125,124	USA	Unknown	Skin lesion	Zoster	
VR4	KF558388	vac	125,125	United Kingdom	2010	Skin lesion	Varicella	
L53	KF558389	vac	125,127	USA	1997	Skin lesion	Zoster	
N13	KF558390	vac	125,127	USA	1998	Skin lesion	Varicella	
K11	KF558391	vac	125,130	USA	1997	Skin lesion	Zoster	
VZVs/Pasadena.USA/48.12/Z[2]	KF811485	vac	125,410	USA	2012	Skin lesion	Zoster	Tseng, H.F., Schmid, D.S., Harpaz, R., LaRussa, P., Jensen, N.J., Rivailler, P., Radford, K., Folster, J., Jacobsen, S.J., 2014. Herpes zoster caused by vaccine-strain varicella zoster virus in an immunocompetent recipient of zoster vaccine. Clin Infect Dis. 58, 1125-1128. doi: 1110.1093/cid/ciu1058. Epub 2014 Jan 1126.
B86	KF853225	vac	125,128	United Kingdom	1986	Unknown	N/A	Weinert, L.A., Depledge, D.P., Kundu, S., Gershon, A.A., Nichols, R.A., Balloux, F., Welch, J.J., Breuer, J., 2015. Rates of vaccine evolution show strong effects of latency: implications for varicella zoster virus epidemiology. Mol Biol Evol. 32, 1020-1028. doi: 1010.1093/molbev/msu1406. Epub 2015 Jan 1026.
A171B	KF853226	vac	125,127	United Kingdom	1988	Unknown	Varicella	
Q27	KF853227	vac	125,071	United Kingdom	1998	Unknown	Varicella	
R3	KF853228	vac	125,112	United Kingdom	1999	Unknown	Zoster	
R43	KF853229	vac	125,081	United Kingdom	2000	Unknown	Varicella	
R52	KF853230	vac	125,130	United Kingdom	1999	Unknown	Zoster	
ZR2	KF853231	vac	125,118	United Kingdom	2006	Unknown	Zoster	
ZR3	KF853232	vac	125,072	United Kingdom	2007	Unknown	Zoster	
VR5	KF853233	vac	125,073	United Kingdom	2008	Unknown	Varicella	
ZR4	KF853234	vac	125,048	United Kingdom	2010	Unknown	Zoster	
ZR5	KF853235	vac	125,056	United Kingdom	2013	Unknown	Zoster	
Baike	MF898328	vac	125,095	China	2015	Vaccine lot	N/A	Wu, Q., Rivailler, P., Xu, S., Xu, W., 2019. Comparison of the Whole-Genome Sequence of an Oka Varicella Vaccine from China with Other Oka Vaccine Strains Reveals Sites Putatively Critical for Vaccine Efficacy. J Virol. 93, e02281-02218. doi: 02210.01128/JVI.02281-02218. Print 02019 May 02281.

Supplementary Table 2:
Size of the reiteration regions in SD14 (this study), Dumas (clade1) and pOka (clade2)

	SD14	Dumas	pOka
IR1	246	306	228
IR2	452	327	326
IR3	175	77	40
IR4	227	146	281

Supplementary Table 3: 2880 SNPs identified among 223 VZV genomes

GB-ID	Clade markers / Position in Dumas	89	90	93	94, 99, 100, 102, 106, 108, 109, 117, 118, 121, 150, 170, 178, 181, 202, 236, 240, 262, 340, 352, 371, 386, 392, 401, 424, 427, 446, 447, 460, 488, 502, 510, 520, 542, 560, 578, 607, 626, 648, 661, 685, 703, 716, 762, 763, 781, 789, 790, 791, 802, 813, 818, 831, 839, 889, 892, 922, 923, 935, 956, 967, 1000, 1028, 1054, 1066, 1095, 1184, 1208, 1252, 1310, 1340, 1343, 1402, 1455, 1498, 1565, 1568, 1600, 1602, 1631, 1694, 1748, 1758, 1775, 1838, 1840, 1844, 1845, 1870, 1881, 1900, 1953, 2043, 2048, 2137, 2151, 244
AY548170	1	t	g	a	[illegible]
AY548171	1	t	g	a	[illegible]
DQ479953	1	t	g	a	[illegible]
DQ479954	1	t	g	a	[illegible]
DQ479958	1	t	g	a	[illegible]
DQ479959	1	t	g	a	[illegible]
DQ479961	1	t	g	a	[illegible]
DQ479962	1	t	g	a	[illegible]
DQ479963	1	t	g	a	[illegible]
DQ674250	1	t	g	a	[illegible]
KX262864	1	t	g	a	[illegible]
KX262865	1	t	g	a	[illegible]
KX262866	1	t	g	a	[illegible]
EU154348	1	t	g	a	[illegible]
JN704690	1	t	g	a	[illegible]
JN704691	1	t	g	a	[illegible]
JN704692	1	t	g	a	[illegible]
JN704693	1	t	g	a	[illegible]
JN704694	1	t	g	a	[illegible]
JN704695	1	t	g	a	[illegible]
JN704696	1	t	g	a	[illegible]
NC001348	1	t	g	a	[illegible]
KP771892	1	t	g	a	[illegible]
KP771894	1	t	g	a	[illegible]
KP771900	1	t	g	a	[illegible]
KP771901	1	t	g	a	[illegible]
KP771902	1	t	g	a	[illegible]
KP771908	1	t	g	a	[illegible]
KP771912	1	t	g	a	[illegible]
KP771913	1	t	g	a	[illegible]
KP771917	1	t	g	a	[illegible]
KP771918	1	t	g	a	[illegible]
KP771920	1	t	g	a	[illegible]
MH709361	1	t	g	a	[illegible]
KU926318	2	t	g	a	[illegible]
KU926319	2	t	g	a	[illegible]
KU926320	2	t	g	a	[illegible]
KU926321	2	t	g	a	[illegible]
KU926322	2	t	g	a	[illegible]
KC847290	2	t	g	a	[illegible]
KJ767491	2	t	g	a	[illegible]
KJ767492	2	t	g	a	[illegible]
KJ808816	2	t	g	a	[illegible]
KP771925	2	t	g	a	[illegible]
KU926315	2	t	g	a	[illegible]
KU926316	2	t	g	a	[illegible]
KU926317	2	t	g	a	[illegible]
KY037796	2	t	g	a	[illegible]
MF004348	2	t	g	a	[illegible]
MH709309	2	t	g	a	[illegible]
MH709310	2	t	g	a	[illegible]
MH709311	2	t	g	a	[illegible]
MH709313	2	t	g	a	[illegible]
MH709314	2	t	g	a	[illegible]
MH709315	2	t	g	a	[illegible]
MH709319	2	t	g	a	[illegible]
MH709320	2	t	g	a	[illegible]
MH709329	2	t	g	a	[illegible]
MH709331	2	t	g	a	[illegible]
MH709333	2	t	g	a	[illegible]
MH709337	2	t	g	a	[illegible]
MH709339	2	t	g	a	[illegible]
MH709344	2	t	g	a	[illegible]
MH709348	2	t	g	a	[illegible]
MH709355	2	t	g	a	[illegible]
MH709359	2	t	g	a	[illegible]
MH709363	2	t	g	a	[illegible]
MH709366	2	t	g	a	[illegible]
MH709376	2	t	g	a	[illegible]
MH709377	2	t	g	a	[illegible]
**MW316406**	**2**	t	g	a	[illegible]
AJ871403	3	t	g	a	[illegible]
DQ479955	3	t	g	a	[illegible]
DQ479956	3	t	g	a	[illegible]
DQ479957	3	t	g	a	[illegible]
JN704699	3	t	g	a	[illegible]
JN704700	3	t	g	a	[illegible]
JN704701	3	t	g	a	[illegible]
JN704702	3	t	g	a	[illegible]
JN704703	3	t	g	a	[illegible]
KP771891	3	t	g	a	[illegible]
KP771893	3	t	g	a	[illegible]
KP771895	3	t	g	a	[illegible]
KP771896	3	t	g	a	[illegible]
KP771897	3	t	g	a	[illegible]
KP771899	3	t	g	a	[illegible]
KP771903	3	t	g	a	[illegible]
KP771907	3	t	g	a	[illegible]
KP771910	3	t	g	a	[illegible]
KP771915	3	t	g	a	[illegible]
KP771916	3	t	g	a	[illegible]
KP771921	3	t	g	a	[illegible]
KP771923	3	t	g	a	[illegible]
KP771889	3	t	g	a	[illegible]
KU926311	3	t	g	a	[illegible]
JQ911599	3	t	g	a	[illegible]
MH709317	3	t	g	a	[illegible]
MH709346	3	t	g	a	[illegible]
MH709375	3	t	g	a	[illegible]
DQ452050	4	t	g	a	[illegible]
DQ479960	4	-	t	g	[illegible]
KP771898	4	t	g	a	[illegible]
MH709316	4	t	g	a	[illegible]
MH709318	4	t	g	a	[illegible]
MH709322	4	t	g	a	[illegible]
MH709323	4	t	g	a	[illegible]
MH709326	4	t	g	a	[illegible]
MH709341	4	t	g	a	[illegible]
MH709357	4	t	g	a	[illegible]
MH709364	4	t	g	a	[illegible]
MH709370	4	t	g	a	[illegible]
MH709371	4	t	g	a	[illegible]
MH709372	4	t	g	a	[illegible]
DQ457052	5	t	g	a	[illegible]
JN704704	5	t	g	a	[illegible]
JN704705	5	t	g	a	[illegible]
JN704706	5	t	g	a	[illegible]
JN704707	5	t	g	a	[illegible]
JN704708	5	t	g	a	[illegible]
KP771904	5	t	g	a	[illegible]
KP771905	5	t	g	a	[illegible]
KP771906	5	t	g	a	[illegible]
KP771919	5	t	g	a	[illegible]
KP771922	5	t	g	a	[illegible]
KP771924	5	t	g	a	[illegible]
KM355696	5	t	g	a	[illegible]
KM355697	5	t	g	a	[illegible]
KM355698	5	t	g	a	[illegible]
KM355699	5	t	g	a	[illegible]
KM355701	5	t	g	a	[illegible]
KM355702	5	t	g	a	[illegible]
KM355703	5	t	g	a	[illegible]
KM355704	5	t	g	a	[illegible]
KM355705	5	t	g	a	[illegible]
KM355706	5	t	g	a	[illegible]
KM355707	5	t	g	a	[illegible]
KM355708	5	t	g	a	[illegible]
KM355709	5	t	g	a	[illegible]
KM355711	5	t	g	a	[illegible]
KM355712	5	t	g	a	[illegible]
[illegible]	[illegible]	[illegible]	[illegible]	[illegible]	[illegible]

Supplementary Table 3: 2880 SNPs identified among 223 VZV genomes

GB-ID	Clade markers																																																					
	Position in Dumas	89	90	93	94	99	100	102	106	108	109	117	118	121	150	170	178	181	202	236	240	262	340	352	371	386	392	401	424	427	446	447	460	488	502	510	520	542	560	578	607	626	648	661	685	703	716	762	763	781	789	790	791	802	813
MH499467	5	-	-	-	-	-	-	-	-	-	-	-	-	-	-	-	-	-	-	-	-	-	c	t	a	t	t	t	a	t	a	t	t	a	t	t	t	c	t	g	t	a	a	c	g	t	t	g	t	t	c	c	c	t	t
MH499469	5	t	g	a	t	t	g	c	a	a	c	-	c	c	g	c	g	c	t	a	t	c	c	t	a	t	t	t	a	t	a	t	t	a	t	t	t	c	t	g	t	a	a	c	g	t	t	g	t	t	c	c	c	t	t
MH709321	5	t	g	a	t	t	g	c	a	a	c	-	g	a	t	c	g	c	t	a	t	c	c	t	a	t	t	t	a	t	a	t	t	a	t	t	t	c	t	g	t	a	a	c	g	t	t	g	t	t	c	c	c	t	t
MH709324	5	t	g	a	t	t	g	c	a	a	c	-	g	a	t	c	g	c	t	a	t	c	c	t	a	t	t	t	a	t	a	t	t	a	t	t	t	c	t	g	t	a	a	c	g	t	t	g	t	t	c	c	c	t	t
MH709325	5	t	g	a	t	t	g	c	a	a	c	-	g	a	t	c	g	c	t	a	t	c	c	t	a	t	t	t	a	t	a	t	t	a	t	t	t	c	t	g	t	a	a	c	g	t	t	g	t	t	c	c	c	t	t
MH709327	5	-	-	-	-	-	-	-	-	-	-	-	g	a	t	c	g	c	t	a	t	c	c	t	a	t	t	t	a	t	a	t	t	a	t	t	t	c	t	g	t	a	a	c	g	t	t	g	t	t	c	c	c	t	t
MH709328	5	t	g	a	t	t	g	c	a	a	c	-	g	a	t	c	g	c	t	a	t	c	c	t	a	t	t	t	a	t	a	t	t	a	t	t	t	c	t	g	t	a	a	c	g	t	t	g	t	t	c	c	c	t	t
MH709330	5	t	g	a	t	t	g	c	a	a	c	-	g	a	t	c	g	c	t	a	t	c	c	t	a	t	t	t	a	t	a	t	t	a	t	t	t	c	t	g	t	a	a	c	g	t	t	g	t	t	c	c	c	t	t
MH709332	5	t	g	a	t	t	g	c	a	a	c	-	g	a	t	c	g	c	t	a	t	c	c	t	a	t	t	t	a	t	a	t	t	a	t	t	t	c	t	g	t	a	a	c	g	t	t	g	t	t	c	c	c	t	t
MH709334	5	t	g	a	t	t	g	c	a	a	c	-	g	a	t	c	g	c	t	a	t	c	c	t	a	t	t	t	a	t	a	t	t	a	t	t	t	c	t	g	t	a	a	c	g	t	t	g	t	t	c	c	c	t	t
MH709335	5	t	g	a	t	t	g	c	a	a	c	-	g	a	t	c	g	c	t	a	t	c	c	t	a	t	t	t	a	t	a	t	t	a	t	t	t	c	t	g	t	a	a	c	g	t	t	g	t	t	c	c	c	t	t
MH709336	5	t	g	a	t	t	g	c	a	a	c	-	g	a	t	c	g	c	t	a	t	c	c	t	a	t	t	t	a	t	a	t	t	a	t	t	t	c	t	g	t	a	a	c	g	t	t	g	t	t	c	c	c	t	t
MH709338	5	t	g	a	t	t	g	c	a	a	c	-	g	a	t	c	g	c	t	a	t	c	c	t	a	t	t	t	a	t	a	t	t	a	t	t	t	c	t	g	t	a	a	c	g	t	t	g	t	t	c	c	c	t	t
MH709340	5	t	g	a	t	t	g	c	a	a	c	-	g	a	t	c	g	c	t	a	t	c	c	t	a	t	t	t	a	t	a	t	t	a	t	t	t	c	t	g	t	a	a	c	g	t	t	g	t	t	c	c	c	t	t
MH709342	5	t	g	a	t	t	g	c	a	a	c	-	g	a	t	c	g	c	t	a	t	c	c	t	a	t	t	t	a	t	a	t	t	a	t	t	t	c	t	g	t	a	a	c	g	t	t	g	t	t	c	c	c	t	t
MH709343	5	t	g	a	t	t	g	c	a	a	c	-	g	a	t	c	g	c	t	a	t	c	c	t	a	t	t	t	a	t	a	t	t	a	t	t	t	c	t	g	t	a	a	c	g	t	t	g	t	t	c	c	c	t	t
MH709345	5	t	g	a	t	t	g	c	a	a	c	-	g	a	t	c	g	c	t	a	t	c	c	t	a	t	t	t	a	t	a	t	t	a	t	t	t	c	t	g	t	a	a	c	g	t	t	g	t	t	c	c	c	t	t
MH709347	5	t	g	a	t	t	g	c	a	a	c	-	g	a	t	c	g	c	t	a	t	c	c	t	a	t	t	t	a	t	a	t	t	a	t	t	t	c	t	g	t	a	a	c	g	t	t	g	t	t	c	c	c	t	t
MH709349	5	t	g	a	t	t	g	c	a	a	c	-	g	a	t	c	g	c	t	a	t	c	c	t	a	t	t	t	a	t	a	t	t	a	t	t	t	c	t	g	t	a	a	c	g	t	t	g	t	t	c	c	c	t	t
MH709350	5	t	g	a	t	t	g	c	a	a	c	-	g	a	t	c	g	c	t	a	t	c	c	t	a	t	t	t	a	t	a	t	t	a	t	t	t	c	t	g	t	a	a	c	g	t	t	g	t	t	c	c	c	t	t
MH709351	5	t	g	a	t	t	g	c	a	a	c	-	g	a	t	c	g	c	t	a	t	c	c	t	a	t	t	t	a	t	a	t	t	a	t	t	t	c	t	g	t	a	a	c	g	t	t	g	t	t	c	c	c	t	t
MH709353	5	t	g	a	t	t	g	c	a	a	c	-	g	a	t	c	g	c	t	a	t	c	c	t	a	t	t	t	a	t	a	t	t	a	t	t	t	c	t	g	t	a	a	c	g	t	t	g	t	t	c	c	c	t	t
MH709358	5	t	g	a	t	t	g	c	a	a	c	-	g	a	t	c	g	c	t	a	t	c	c	t	a	t	t	t	a	t	a	t	t	a	t	t	t	c	t	g	t	a	a	c	g	t	t	g	t	t	c	c	c	t	t
MH709360	5	t	g	a	t	t	g	c	a	a	c	-	g	a	t	c	g	c	t	a	t	c	c	t	a	t	t	t	a	t	a	t	t	a	t	t	t	c	t	g	t	a	a	c	g	t	t	g	t	t	c	c	c	t	t
MH709362	5	t	g	a	t	t	g	c	a	a	c	-	g	a	t	c	g	c	t	a	t	c	c	t	a	t	t	t	a	t	a	t	t	a	t	t	t	c	t	g	t	a	a	c	g	t	t	g	t	t	c	c	c	t	t
MH709365	5	t	g	a	t	t	g	c	a	a	c	-	g	a	t	c	g	c	t	a	t	c	c	t	a	t	t	t	a	t	a	t	t	a	t	t	t	c	t	g	t	a	a	c	g	t	t	g	t	t	c	c	c	t	t
MH709373	5	t	g	a	t	t	g	c	a	a	c	-	g	a	t	c	g	c	t	a	t	c	c	t	c	t	t	t	a	t	a	t	t	a	t	t	t	c	t	g	t	a	a	c	g	t	t	g	t	t	c	c	c	t	t
MH709374	5	t	g	a	t	t	g	c	a	a	c	-	g	a	t	c	g	c	t	a	t	c	c	t	a	t	t	t	a	t	a	t	t	a	t	t	t	c	t	g	t	a	a	c	g	t	t	g	t	t	c	c	c	t	t
KC112914	6	t	g	a	t	t	g	c	a	a	c	-	g	a	t	c	g	c	t	a	t	c	c	t	a	t	t	t	a	t	a	t	t	a	t	t	t	c	t	g	t	a	a	c	g	t	t	g	t	t	t	t	t	t	t
KP771914	6	t	g	a	t	t	g	c	a	a	c	-	g	a	t	c	g	c	t	a	t	c	c	t	a	t	t	t	a	t	a	t	t	a	t	t	t	c	t	g	t	a	a	c	g	t	t	g	t	t	t	t	t	t	t
KP702725	6	t	g	a	t	t	g	c	a	a	c	-	g	a	t	c	g	c	t	a	t	c	c	t	a	t	t	t	a	t	a	t	t	a	t	t	t	c	t	g	t	a	a	c	g	t	t	g	t	t	t	t	t	t	t
KP771890	9	t	g	a	t	t	g	c	a	a	c	-	g	a	t	c	g	c	t	a	t	c	c	t	a	t	t	t	g	t	a	t	t	a	t	t	t	c	t	g	t	a	a	c	g	t	t	g	t	t	t	t	t	t	t
JN704710	9	t	g	a	t	t	g	c	a	a	c	-	g	a	t	c	g	c	t	a	t	c	c	t	a	t	t	t	a	t	g	t	t	a	t	t	t	c	t	g	t	a	a	c	g	t	t	g	t	t	c	c	c	t	t
KY037798	9	t	g	a	t	g	-	c	a	a	c	-	g	a	t	c	g	c	t	a	t	c	c	t	a	t	t	t	a	t	g	t	t	a	t	t	t	c	t	g	t	a	a	c	g	t	t	g	t	t	c	c	c	t	t
MH709312	9	-	-	-	-	-	-	-	-	-	-	-	g	a	t	c	g	c	t	a	t	c	c	t	a	t	t	t	a	t	g	t	t	a	t	t	t	c	t	g	t	a	a	c	g	t	t	g	t	t	c	c	c	t	t
MH709352	9	t	g	a	t	g	c	c	g	a	a	-	g	a	t	t	g	t	t	a	t	c	c	t	a	t	t	t	a	t	g	t	t	a	t	t	t	c	t	g	t	a	a	c	g	t	t	g	t	t	c	c	c	t	t
AB097933	Vac	t	g	a	t	t	g	c	a	a	c	-	g	a	t	c	g	c	t	g	t	t	c	t	a	t	t	t	a	t	a	t	t	a	t	t	t	c	t	g	t	a	a	c	a	t	t	g	t	t	c	c	c	t	t
JN704698	Vac	t	g	a	t	t	g	c	a	a	c	-	g	a	t	c	c	c	t	g	t	c	c	t	a	t	t	t	a	t	a	t	t	a	t	t	t	c	t	g	t	a	a	c	a	t	t	g	t	c	c	c	c	t	t
JN704697	Vac	t	g	a	t	t	g	c	a	a	c	-	g	a	t	c	c	c	t	g	t	t	c	t	a	t	t	t	a	t	a	t	t	g	t	t	t	c	c	g	t	a	a	c	a	c	t	g	c	t	c	c	c	t	t
DQ008355	Vac	t	g	a	t	t	g	c	a	a	c	-	g	a	t	c	c	c	t	g	t	t	c	t	a	t	t	t	a	t	a	t	t	a	t	t	t	c	c	g	t	a	a	c	a	c	t	g	c	t	c	c	c	t	t
JF306641	Vac	t	g	a	t	t	g	c	a	a	c	-	g	a	t	c	c	c	t	g	t	t	c	t	a	t	t	t	a	t	a	t	t	a	t	t	t	c	c	g	t	a	a	c	a	c	t	g	c	t	c	c	c	t	t
AB097932	Vac	t	g	a	t	t	g	c	a	a	c	-	g	a	t	c	g	c	t	g	t	t	c	t	a	t	t	t	a	t	a	t	t	a	t	t	t	c	c	g	t	a	a	c	a	y	t	g	y	t	c	c	c	t	t
DQ008354	Vac	t	g	a	t	t	g	c	a	a	c	-	g	a	t	c	c	c	t	g	t	t	c	t	a	t	t	t	a	t	a	t	t	a	t	t	t	c	c	g	t	a	a	c	a	c	t	g	c	t	c	c	c	t	t
KU926312	Vac	t	g	a	t	t	g	c	a	a	c	-	g	a	t	c	c	c	t	g	t	t	c	t	a	t	t	t	a	t	a	t	t	a	t	t	t	c	c	g	t	a	a	c	a	c	t	g	c	t	c	c	c	t	t
KU926313	Vac	t	g	a	t	t	g	c	a	a	c	-	g	a	t	c	c	c	t	g	t	t	c	t	a	t	t	t	a	t	a	t	t	a	t	t	t	c	c	g	t	a	a	c	a	c	t	g	c	t	c	c	c	t	t
KU926314	Vac	t	g	a	t	t	g	c	a	a	c	-	g	a	t	c	c	c	t	g	t	t	c	t	a	t	t	t	a	t	a	t	t	a	t	t	t	c	c	g	t	a	a	c	a	c	t	g	c	t	c	c	c	t	t
KF558371	Vac	t	g	a	t	t	g	c	a	a	c	-	g	a	t	c	c	c	t	g	t	t	c	t	a	t	t	t	a	t	a	t	t	a	t	t	t	c	c	g	t	a	a	c	a	t	t	g	t	t	c	c	c	t	t
KF558372	Vac	t	g	a	t	t	g	c	a	a	c	-	g	a	t	c	c	c	t	g	t	t	c	t	a	t	t	t	a	t	a	t	t	a	t	t	t	c	t	g	t	a	a	c	a	t	t	g	c	t	c	c	c	t	t
KF558373	Vac	t	g	a	t	t	g	c	a	a	c	-	g	a	t	c	c	c	t	g	t	t	c	t	a	t	t	t	a	t	a	t	t	a	t	c	t	c	c	g	t	a	a	c	a	c	t	g	c	t	c	c	c	c	t
KF558374	Vac	t	g	a	t	t	g	c	a	a	c	-	g	a	t	c	c	c	t	g	t	t	c	t	a	t	t	t	a	t	a	t	t	a	t	t	t	c	c	g	t	a	a	c	a	c	t	g	c	t	c	c	c	t	t
KF558375	Vac	t	g	a	t	t	g	c	a	a	c	-	g	a	t	c	c	c	t	g	t	t	c	t	a	t	t	t	a	t	a	t	t	a	t	t	t	c	c	g	t	a	a	c	a	c	t	g	c	t	c	c	c	t	t
KF558376	Vac	t	g	a	t	t	g	c	a	a	c	-	g	a	t	c	c	c	t	g	t	t	c	t	a	t	t	t	a	t	a	t	t	g	t	t	t	c	c	g	t	a	a	c	a	c	t	g	t	t	c	c	c	t	t
KF558377	Vac	c	g	a	t	t	g	c	a	a	c	-	g	a	t	c	c	c	t	g	t	t	c	t	a	t	t	t	a	t	a	t	t	a	t	c	t	c	c	g	t	a	a	c	a	c	t	g	c	t	c	c	c	t	t
KF558378	Vac	t	g	a	t	t	g	c	a	a	c	-	g	a	t	c	c	c	t	g	t	t	c	t	a	t	t	t	a	t	a	t	t	a	t	t	t	c	c	g	t	a	a	c	a	c	t	g	t	t	c	c	c	t	t
KF558379	Vac	t	g	a	t	t	g	c	a	a	c	-	g	a	t	c	c	c	t	g	t	t	c	t	a	t	t	t	a	t	a	t	t	a	t	t	t	c	c	g	t	a	a	c	a	t	t	g	c	t	c	c	c	c	t
KF558380	Vac	t	g	a	t	t	g	c	a	a	c	-	g	a	t	c	c	c	t	g	t	t	c	t	a	t	t	t	a	t	a	t	t	a	t	t	t	c	c	g	t	a	a	c	a	c	t	g	c	t	c	c	c	t	t
KF558381	Vac	t	g	a	t	t	g	c	a	a	c	-	g	a	t	c	c	c	t	g	t	t	c	t	a	t	t	t	a	t	a	t	t	a	t	t	t	c	c	g	t	a	a	c	a	c	t	g	t	t	c	c	c	t	t
KF558382	Vac	t	g	a	t	t	g	c	a	a	c	-	g	a	t	c	c	c	t	g	t	t	c	t	a	t	t	t	a	t	a	t	t	g	t	t	t	c	t	g	t	a	a	c	a	t	t	g	t	t	c	c	c	t	t
KF558383	Vac	t	g	a	t	t	g	c	a	a	c	-	g	a	t	c	c	c	t	g	t	t	c	t	a	t	t	t	a	t	a	t	t	a	t	t	t	c	c	g	t	a	a	c	a	c	t	g	c	t	c	c	c	t	t
KF558384	Vac	t	g	a	t	t	g	c	a	a	c	-	g	a	t	c	c	c	t	g	t	t	c	t	a	t	t	t	a	t	a	t	t	a	t	t	t	c	c	g	t	a	a	c	a	c	t	g	c	t	c	c	c	t	t
KF558385	Vac	t	g	a	t	t	g	c	a	a	c	-	g	a	t	c	c	c	t	g	t	t	c	t	a	t	t	t	a	t	a	t	t	a	t	t	t	c	c	g	t	a	a	c	a	c	t	g	c	t	c	c	c	t	t
KF558386	Vac	t	g	a	t	t	g	c	a	a	c	-	g	a	t	c	c	c	t	g	t	t	c	t	a	t	t	t	a	t	a	t	t	a	t	t	t	c	t	g	t	a	a	c	a	t	t	g	c	t	c	c	c	t	t
KF558387	Vac	t	g	a	t	t	g	c	a	a	c	-	g	a	t	c	c	c	t	g	t	t	c	t	a	t	t	t	a	t	a	t	t	a	t	t	t	c	c	g	t	a	a	c	a	c	t	g	c	t	c	c	c	t	t
KF558388	Vac	t	g	a	t	t	g	c	a	a	c	-	g	a	t	c	c	c	t	g	t	t	c	t	a	t	t	t	a	t	a	t	t	a	t	t	t	c	t	g	t	a	a	c	a	t	t	g	m	t	c	c	c	t	t
KF558389	Vac	t	g	a	t	t	g	c	a	a	c	-	g	a	t	c	c	c	t	g	t	t	c	t	a	t	t	t	a	t	a	t	t	a	t	t	t	c	c	g	t	a	a	c	a	c	t	g	c	t	c	c	c	t	t
KF558390	Vac	t	g	a	t	t	g	c	a	a	c	-	g	a	t	c	c	c	t	g	t	t	c	t	a	t	t	t	a	t	a	t	t	g	t	t	t	c	c	g	t	a	a	c	a	c	t	g	c	t	c	c	c	t	t
KF558391	Vac	t	g	a	t	t	g	c	a	a	c	-	g	a	t	c	c	c	t	g	t	t	c	t	a	t	t	t	a	t	a	t	t	a	t	t	t	c	c	g	t	a	a	c	a	c	t	g	c	t	c	c	c	t	t
KF811485	Vac	t	g	a	t	t	g	c	a	a	c	-	g	a	t	c	c	c	t	g	t	t	c	t	a	t	t	t	a	t	a	t	t	a	t	t	t	c	c	g	t	a	a	c	a	t	t	g	c	t	c	c	c	t	t
KF853225	Vac	t	g	a	t	t	g	c	a	a	c	-	g	a	t	c	c	c	t	g	t	t	c	t	a	t	t	t	a	t	a	t	t	a	t	t	t	c	c	g	t	a	a	c	a	t	t	g	t	t	c	c	c	t	t
KF853226	Vac	t	g	a	t	t	g	c	a	a	c	-	g	a	t	c	c	c	t	g	t	t	c	t	a	t	t	t	a	t	a	t	t	a	t	t	t	c	t	g	t	a	a	c	a	t	t	g	t	t	c	c	c	t	t
KF853227	Vac	t	g	a	t	t	g	c	a	a	c	-	g	a	t	c	c	c	t	g	t	t	c	t	a	t	t	t	a	t	a	t	t	a	t	t	t	c	t	g	t	a	a	c	a	c	t	g	t	t	c	c	c	c	t
KF853228	Vac	t	g	a	t	t	g	c	a	a	c	-	g	a	t	c	c	c	t	g	t	t	c	t	a	t	t	t	a	t	a	t	t	a	t	t	t	c	c	g	t	a	a	c	a	c	t	g	c	t	c	c	c	t	t
KF853229	Vac	t	g	a	t	t	g	c	a	a	c	-	g	a	t	c	c	c	t	g	t	t	c	t	a	t	t	t	a	t	a	t	t	a	t	t	t	c	c	g	t	a	a	c	a	t	t	g	c	t	c	c	c	t	t
KF853230	Vac	t	g	a	t	t	g	c	a	a	c	-	g	a	t	c	c	c	t	g	t	t	c	t	a	t	t	t	a	t	a	t	t	a	t	c	t	c	c	g	t	a	a	c	a	c	t	g	t	t	c	c	c	t	t
KF853231	Vac	t	g	a	t	t	g	c	a	a	c	-	g	a	t	c	c	c	t	g	t	t	c	t	a	t	t	t	a	t	a	t	t	a	t	t	t	c	t	g	t	a	a	c	a	t	t	g	c	t	c	c	c	t	t
KF853232	Vac	t	g	a	t	t	g	c	a	a	c	-	g	a	t	c	c	c	t	g	t	t	c	t	a	t	t	t	a	t	a	t	t	a	t	t	t	c	c	g	t	a	a	c	a	c	t	g	t	t	c	c	c	t	t
KF853233	Vac	t	g	a	t	t	g	c	a	a	c	-	g	a	t	c	c	c	t	g	t	t	c	t	a	t	t	t	a	t	a	t	t	a	t	t	t	c	c	g	t	a	a	c	a	c	t	g	t	t	c	c	c	t	t
KF853234	Vac	t	g	a	t	t	g	c	a	a	c	-	g	a	t	c	c	c	t	g	t	t	c	t	a	t	t	t	a	t	a	t	t	a	t	t	t	c	c	g	t	a	a	c	a	c	t	g	t	t	c	c	c	t	t
KF853235	Vac	t	g	a	t	t	g	c	a	a	c	-	g	a	t	c	c	c	t	g	t	t	c	t	a	t	t	t	a	t	a	t	t	a	t	t	t	c	c	g	t	a	a	c	a	t	t	g	c	t	c	c	c	t	t
MF898328	Vac	t	g	a	t	t	g	c	a	a	c	-	g	a	t	c	c	c	t	g	t	t	c	t	a	t	t	t	a	t	a	t	t	r	t	t	t	c	c	g	t	a	a	c	a	y	t	g	c	t	c	c	c	t	t
JN704709	VIII	t	g	a	t	t	g	c	a	a	c	-	g	a	t	c	g	c	t	a	t	c	c	t	a	t	t	t	a	t	a	t	t	a	t	t	t	c	t	g	t	a	a	c	a	t	t	g	t	t	c	c	c	t	t

GB-ID	Clade markers																																														
	Position in Dumas	818	831	839	889	892	922	923	935	956	967	1000	1028	1054	1066	1095	1184	1208	1252	1310	1340	1343	1402	1455	1498	1565	1568	1600	1602	1631	1694	1748	1758	1775	1838	1840	1844	1845	1870	1881	1900	1953	2043	2048	2137	2151	244
MH499467	5	t	t	t	t	c	t	c	a	a	g	t	g	a	a	a	t	c	g	a	t	t	t	t	t	g	t	t	t	t	a	a	a	g	c	t	g	a	t	g	t	a	a	g	c	g	a
MH499469	5	t	t	t	t	c	t	c	a	a	g	t	g	a	a	a	t	c	g	a	t	t	t	t	t	g	t	t	t	t	a	a	a	g	c	t	g	a	t	g	t	a	a	g	c	g	a
MH709321	5	t	t	t	t	c	t	c	a	a	g	t	g	a	c	a	t	c	g	a	t	t	t	t	t	g	t	t	t	t	a	a	a	g	c	t	g	a	t	g	t	a	a	g	c	g	a
MH709324	5	t	t	t	t	c	t	c	a	a	g	t	g	a	a	a	t	c	g	a	t	t	t	t	t	g	t	t	t	t	a	a	a	g	c	t	g	a	t	g	t	a	a	g	c	g	a
MH709325	5	t	t	t	t	c	t	c	a	a	g	t	g	a	a	a	t	c	g	a	t	t	t	t	t	g	t	t	t	t	a	a	a	g	c	t	g	a	t	g	t	a	a	g	c	g	a
MH709327	5	t	t	t	t	c	t	c	a	a	g	t	g	a	a	a	t	c	g	a	t	t	t	t	t	g	t	t	t	t	a	a	a	g	c	t	g	a	t	g	t	a	a	g	c	g	a
MH709328	5	t	t	t	t	c	t	c	a	a	g	t	g	a	a	a	t	c	g	a	t	t	t	t	t	g	t	t	t	t	a	a	a	g	c	t	g	a	t	g	t	a	a	g	c	g	a
MH709330	5	t	t	t	t	c	t	c	a	a	g	t	g	a	a	a	t	c	g	a	t	t	t	t	t	g	t	t	t	t	a	a	a	g	c	t	g	a	t	g	t	a	a	g	c	g	a
MH709332	5	t	t	t	t	c	t	c	a	a	g	t	g	a	a	a	t	c	g	a	t	t	t	t	t	g	t	t	t	t	a	a	a	g	c	t	g	a	t	g	t	a	a	g	c	g	a
MH709334	5	t	t	t	t	c	t	c	a	a	g	t	g	a	a	a	t	c	g	a	t	t	t	t	t	g	t	t	t	t	a	a	a	g	c	t	g	a	t	g	t	a	a	g	c	g	a
MH709335	5	t	t	t	t	c	t	c	a	a	g	t	g	a	a	a	t	c	g	a	t	t	t	t	t	g	t	t	t	t	a	a	a	g	c	t	g	a	t	g	t	a	a	g	c	g	a
MH709336	5	t	t	t	t	c	t	c	a	a	g	t	g	a	a	a	t	c	g	a	t	t	t	t	t	g	t	t	t	t	a	a	a	g	c	t	g	a	t	g	t	a	a	g	c	g	a
MH709338	5	t	t	t	t	c	t	c	a	a	g	t	g	a	a	a	t	c	g	a	t	t	t	t	t	g	t	t	t	t	a	a	a	g	c	t	g	a	t	g	t	a	a	g	c	g	a
MH709340	5	t	t	t	t	c	t	c	a	a	g	t	g	a	a	a	t	c	g	a	t	t	t	t	t	g	t	t	t	t	a	a	a	g	c	t	g	a	t	g	t	a	a	g	c	g	a
MH709342	5	t	t	t	t	c	t	c	a	a	g	t	g	a	a	a	t	c	g	a	t	t	t	t	t	g	t	t	t	t	a	a	a	g	c	t	g	a	t	g	t	a	a	g	c	g	a
MH709343	5	t	t	t	t	c	t	c	a	a	g	t	g	a	a	a	t	c	g	a	t	t	t	t	t	g	t	t	t	t	a	a	a	g	c	t	g	a	t	g	t	a	a	g	c	g	a
MH709345	5	t	t	t	t	c	t	c	a	a	g	t	g	a	a	a	t	c	g	a	t	t	t	t	t	g	t	t	t	t	a	a	a	g	c	t	g	a	t	g	t	a	a	g	c	g	a
MH709347	5	t	t	t	t	c	t	c	a	a	g	t	g	a	a	a	t	c	g	a	t	t	t	t	t	g	t	t	t	t	a	a	a	g	c	t	g	a	t	g	t	a	a	g	c	g	a
MH709349	5	t	t	t	t	c	t	c	a	a	g	t	g	a	a	a	t	c	g	a	t	t	t	t	t	g	t	t	t	t	a	a	a	g	c	t	g	a	t	g	t	a	a	g	c	g	a
MH709350	5	t	t	t	t	c	t	c	t	a	g	t	g	a	a	a	t	c	g	a	t	t	t	t	t	g	t	t	t	t	a	a	a	g	c	t	g	a	t	g	t	a	a	g	c	g	a
MH709351	5	t	t	t	t	c	t	c	a	a	g	t	g	a	a	a	t	c	g	a	t	t	t	t	t	g	t	t	t	t	a	a	a	g	c	t	g	a	t	g	t	a	a	g	c	g	a
MH709353	5	t	t	t	t	c	t	c	a	a	g	t	g	a	a	a	t	c	g	a	t	t	t	t	t	g	t	t	t	t	a	a	a	g	c	t	g	a	t	g	t	a	a	g	c	g	a
MH709358	5	t	t	t	t	c	t	c	a	a	g	t	g	a	a	a	t	c	g	a	t	t	t	t	t	g	t	t	t	t	a	a	a	g	c	t	g	a	t	g	t	a	a	g	c	g	a
MH709360	5	t	t	t	t	c	t	c	a	a	g	t	g	a	a	a	t	c	g	a	t	t	t	t	t	g	t	t	t	t	a	a	a	g	c	t	g	a	t	g	t	a	a	g	c	g	a
MH709362	5	t	t	t	t	c	t	c	a	a	g	t	g	a	a	a	t	c	g	a	t	t	t	t	t	g	t	t	t	t	a	a	a	g	c	t	g	a	t	g	t	a	a	g	c	g	a
MH709365	5	t	t	t	t	c	t	c	a	a	g	t	g	a	a	a	t	c	g	a	t	t	t	t	t	g	t	t	t	t	a	a	a	g	c	t	g	a	t	g	t	a	a	g	c	g	a
MH709373	5	t	t	t	t	c	t	c	a	a	g	t	g	a	a	a	t	c	g	a	t	t	t	t	t	g	t	t	t	t	a	a	a	g	c	t	g	a	t	g	t	a	a	g	c	g	a
MH709374	5	t	t	t	t	c	t	c	a	a	g	t	g	a	a	a	t	c	g	a	t	t	t	t	t	g	t	t	t	t	a	a	a	g	c	t	g	a	t	g	t	a	a	g	c	g	a
KC112914	6	t	t	t	t	c	t	c	a	a	g	t	g	a	a	a	t	c	g	c	t	t	t	t	t	g	t	t	t	t	a	a	a	g	c	t	g	c	t	g	t	a	a	g	c	g	a
KP771914	6	t	t	t	t	c	t	c	a	a	g	t	g	a	a	a	t	c	g	a	t	t	t	t	t	g	t	t	t	t	a	a	a	g	c	t	g	a	t	g	t	a	a	g	c	g	a
KP702725	6	t	t	t	t	c	t	c	a	a	g	t	g	a	a	a	t	c	g	a	t	t	t	t	t	g	t	t	t	t	a	a	a	g	c	t	g	c	t	g	t	a	a	g	c	g	a
KP771890	9	t	t	t	t	c	t	t	a	a	g	t	g	a	a	a	t	c	g	a	t	t	t	t	t	g	t	t	t	t	a	a	a	g	c	t	g	a	t	g	t	a	a	g	c	g	a
JN704710	9	t	t	t	t	c	t	t	a	a	g	t	t	a	a	a	t	c	g	a	t	t	t	t	t	g	t	t	t	t	a	a	a	g	c	t	g	a	t	g	t	a	a	g	c	g	a
KY037798	9	t	t	t	t	c	t	t	a	a	g	t	g	a	a	a	t	c	g	a	t	t	t	t	t	g	t	t	t	t	a	a	a	g	c	t	g	a	t	g	t	a	a	g	c	g	a
MH709312	9	t	t	t	t	c	t	t	a	a	g	t	g	a	a	a	t	c	g	a	t	t	t	t	t	g	t	t	t	t	a	a	a	g	c	t	g	a	t	g	t	a	a	g	c	g	a
MH709352	9	t	t	t	t	c	t	t	a	a	g	t	g	a	a	a	t	c	g	a	t	t	t	t	t	g	t	t	t	t	a	a	a	g	c	t	g	a	t	g	t	a	a	g	c	g	a
AB097933	Vac	t	t	t	t	c	t	c	a	a	g	t	t	a	a	a	t	c	g	a	t	t	t	t	t	g	t	t	t	t	a	a	a	g	c	t	g	a	t	g	t	a	a	g	c	g	a
JN704698	Vac	t	t	t	t	c	t	c	a	a	g	t	t	a	a	a	t	c	g	a	t	t	t	t	t	g	t	t	t	t	a	a	a	g	c	t	g	a	t	g	t	a	a	g	c	g	a
JN704697	Vac	t	t	t	t	c	t	c	a	a	g	t	t	a	a	a	t	c	g	a	t	t	t	t	t	g	t	t	t	t	a	a	a	g	c	t	g	a	t	g	t	a	a	g	c	g	a
DQ008355	Vac	t	t	t	t	c	t	c	a	a	g	t	t	a	a	a	t	c	g	a	t	t	t	t	t	g	t	t	t	t	a	a	a	g	c	t	g	a	t	g	t	a	a	g	c	g	a
JF306641	Vac	t	t	t	t	c	t	c	a	a	g	t	t	a	a	a	t	c	g	a	t	t	t	t	t	g	t	t	t	t	a	a	a	g	c	t	g	a	t	g	t	a	a	g	c	g	a
AB097932	Vac	t	t	t	t	c	t	c	a	a	g	t	t	a	a	a	t	c	g	a	t	t	t	t	t	g	t	t	t	t	a	a	a	g	g	t	g	a	t	g	t	a	a	g	c	g	a
DQ008354	Vac	t	t	t	t	c	t	c	a	a	g	t	t	a	a	a	t	c	g	a	t	t	t	t	t	g	t	t	t	t	a	a	a	g	c	t	g	a	t	g	t	a	a	g	c	g	a
KU926312	Vac	t	t	t	t	c	t	c	a	a	g	t	t	a	a	a	t	c	g	a	t	t	t	t	t	g	t	t	t	t	a	a	a	g	c	t	g	a	t	g	t	a	a	g	c	g	a
KU926313	Vac	t	t	t	t	c	t	c	a	a	g	t	t	a	a	a	t	c	g	a	t	t	t	t	t	g	t	t	t	t	a	a	a	g	c	t	g	a	t	g	t	a	a	g	c	g	a
KU926314	Vac	t	t	t	t	c	t	c	a	a	g	t	t	a	a	a	t	c	g	a	t	t	t	t	t	g	t	t	t	t	a	a	a	g	c	t	g	a	t	g	t	a	a	g	c	g	a
KF558371	Vac	t	t	t	t	c	t	c	a	a	g	t	t	a	a	a	t	c	g	a	t	t	t	t	t	g	t	t	t	t	a	a	a	g	c	t	g	a	t	g	t	a	a	g	c	g	a
KF558372	Vac	t	t	t	t	c	t	c	a	a	g	t	t	g	a	a	t	c	g	a	t	t	t	t	t	g	t	t	t	t	a	a	a	g	c	t	g	a	t	g	t	a	a	g	c	g	a
KF558373	Vac	t	t	t	t	c	t	c	a	a	g	t	t	a	a	a	t	c	g	a	t	t	t	t	t	g	t	t	t	t	a	a	a	g	c	t	g	a	t	g	t	a	a	g	c	g	a
KF558374	Vac	t	t	t	t	c	t	c	a	a	g	t	t	a	a	a	t	c	g	a	t	t	t	t	t	g	t	t	t	t	a	a	a	g	c	t	g	a	t	g	t	a	a	g	c	g	a
KF558375	Vac	t	t	t	t	c	t	c	a	a	g	t	t	a	a	a	t	c	g	a	t	t	t	t	t	g	t	t	t	t	a	a	a	g	c	t	g	a	t	g	t	a	a	g	c	g	a
KF558376	Vac	t	t	t	t	c	t	c	a	a	g	t	t	a	a	a	t	c	g	a	t	t	t	t	t	g	t	t	t	t	a	a	a	g	c	t	g	a	t	g	t	a	a	g	c	g	a
KF558377	Vac	t	t	t	t	c	t	c	a	a	a	t	t	a	a	a	t	c	g	a	t	t	t	t	t	g	t	t	t	t	a	a	a	g	c	t	g	a	t	g	t	a	a	g	c	g	a
KF558378	Vac	t	t	t	t	c	t	c	a	a	g	t	t	a	a	a	t	c	g	a	t	t	t	t	t	g	t	t	t	t	g	a	a	g	c	t	g	a	t	g	t	a	a	g	c	g	a
KF558379	Vac	t	t	t	t	c	t	c	a	a	g	t	t	a	a	a	t	c	g	a	t	t	t	t	t	g	t	t	t	t	a	a	a	g	c	t	g	a	t	g	t	a	a	g	c	g	a
KF558380	Vac	t	t	t	t	c	t	c	a	a	g	t	t	a	a	a	t	c	g	a	t	t	t	t	t	g	t	t	t	t	a	a	a	g	c	t	g	a	t	g	t	a	a	g	c	g	a
KF558381	Vac	t	t	t	t	c	t	c	a	a	g	t	t	a	a	a	t	c	g	a	t	t	t	t	t	g	t	t	t	t	a	a	a	g	c	t	g	a	t	g	t	a	a	g	c	g	a
KF558382	Vac	t	t	t	t	c	t	c	a	a	g	t	t	a	a	a	t	c	g	a	t	t	t	t	t	g	t	t	t	t	a	a	a	g	c	t	g	a	t	g	t	a	a	g	c	g	a
KF558383	Vac	t	t	t	t	c	t	c	a	a	g	t	t	a	a	a	t	c	g	a	t	t	t	t	t	g	t	t	t	t	a	a	a	g	c	t	g	a	t	g	t	a	a	g	c	g	a
KF558384	Vac	t	t	t	t	c	t	c	a	a	g	t	t	a	a	a	t	c	g	a	t	t	t	t	t	g	t	t	t	t	a	a	a	g	c	t	g	a	t	g	t	a	a	g	c	g	a
KF558385	Vac	t	t	t	t	c	t	c	a	a	g	t	t	a	a	a	t	c	g	a	t	t	t	t	t	g	t	t	t	t	a	a	a	g	c	t	g	a	t	g	t	a	a	g	c	g	a
KF558386	Vac	t	t	t	t	c	t	c	a	a	g	t	t	a	a	a	t	c	g	a	t	t	t	t	t	g	t	t	t	t	a	a	a	g	c	t	g	a	t	g	t	a	a	g	c	g	a
KF558387	Vac	t	t	t	t	c	t	c	a	a	g	t	t	a	a	a	t	c	g	a	t	t	t	t	t	g	t	t	t	t	a	a	a	g	c	t	g	a	t	g	t	a	a	g	c	g	a
KF558388	Vac	t	t	t	t	c	t	c	a	a	g	t	t	a	a	a	t	c	g	a	t	t	t	t	t	g	t	t	t	t	a	a	a	g	c	t	g	a	t	g	t	a	a	g	c	g	a
KF558389	Vac	t	t	t	t	c	t	c	a	a	g	t	t	a	a	a	t	c	g	a	t	t	t	t	t	g	t	t	t	t	a	a	a	g	c	t	g	a	t	g	t	a	a	g	c	g	a
KF558390	Vac	t	t	t	t	c	t	c	a	a	g	t	t	a	a	a	t	c	g	a	t	t	t	t	t	g	t	t	t	t	a	a	a	g	c	t	g	a	t	g	t	a	a	g	c	g	a
KF558391	Vac	t	t	t	t	c	t	c	a	a	g	t	t	a	a	a	t	c	g	a	t	t	t	t	t	g	t	t	t	t	a	a	a	g	c	t	g	a	t	g	t	a	a	g	c	g	a
KF811485	Vac	t	t	t	t	c	t	c	a	a	g	t	t	a	a	a	t	c	g	a	t	t	t	t	t	g	t	t	t	t	a	a	a	g	c	t	g	a	t	g	t	a	a	g	c	g	a
KF853225	Vac	t	t	t	t	c	t	c	a	a	g	t	t	a	a	a	t	c	g	a	t	t	t	t	t	g	t	t	t	t	a	a	a	g	c	t	g	a	t	g	t	a	a	g	c	g	a
KF853226	Vac	t	t	t	t	c	t	c	a	a	g	t	t	a	a	a	t	c	g	a	t	t	t	t	t	g	t	t	t	t	a	a	a	g	c	t	g	a	t	g	t	a	a	g	c	g	a
KF853227	Vac	t	t	t	t	c	t	c	a	a	g	t	t	a	a	a	t	c	g	a	t	t	t	t	t	g	t	t	t	t	a	a	a	g	c	t	g	a	t	g	t	a	a	g	c	g	a
KF853228	Vac	t	t	t	t	c	t	c	a	a	g	t	t	a	a	a	t	c	g	a	t	t	t	t	t	g	t	t	t	t	a	a	a	g	c	t	g	a	t	g	t	a	a	g	c	g	a
KF853229	Vac	t	t	t	t	c	t	c	a	a	g	t	t	a	a	a	t	c	g	a	t	t	t	t	t	g	t	t	t	t	a	a	a	g	c	t	g	a	t	g	t	a	a	g	c	g	a
KF853230	Vac	t	t	t	t	c	t	c	a	a	g	t	t	a	a	a	t	c	g	a	t	t	t	t	t	g	t	t	t	t	a	a	a	g	c	t	g	a	t	g	t	a	a	g	c	g	a
KF853231	Vac	t	t	t	t	c	t	c	a	a	g	t	t	a	a	a	t	c	g	a	t	t	t	t	t	g	t	t	t	t	a	a	a	g	c	t	g	a	t	g	t	a	a	g	c	g	a
KF853232	Vac	t	t	t	t	c	t	c	a	a	g	t	t	a	a	a	t	c	g	a	t	t	t	t	t	g	t	t	t	t	a	a	a	g	c	t	g	a	t	g	t	a	a	g	c	g	a
KF853233	Vac	t	t	t	t	c	t	c	a	a	g	t	t	a	a	a	t	c	g	a	t	t	t	t	t	g	t	t	t	t	a	a	a	g	c	t	g	a	t	g	t	a	a	g	c	g	a
KF853234	Vac	t	t	t	t	c	t	c	a	a	g	t	t	a	a	a	t	c	g	a	t	t	t	t	t	g	t	t	t	t	a	a	a	g	c	t	g	a	t	g	t	a	a	g	c	g	a
KF853235	Vac	t	t	t	t	c	t	c	a	a	g	t	t	a	a	a	t	c	g	a	t	t	t	t	t	g	t	t	t	t	a	a	a	g	c	t	g	a	t	g	t	a	a	g	c	g	a
MF898328	Vac	t	t	t	t	c	t	c	a	a	g	t	t	a	a	a	t	c	g	a	t	t	t	t	t	g	t	t	t	t	a	a	a	g	c	t	g	a	t	g	t	a	a	g	c	g	a
JN704709	VIII	t	t	t	t	c	t	c	a	a	g	t	g	a	a	a	t	c	g	a	t	t	t	t	t	g	t	t	t	t	a	a	a	g	c	t	g	a	t	g	t	a	a	g	c	g	a

Supplementary Table 3: 2880 SN

GB-ID	Clade markers																																																																																						
	Position in Dumas	2321	2420	2423	2459	2493	2503	2504	2515	2547	2591	2596	2614	2642	2643	2676	2701	2742	2743	2778	2783	2788	2807	2942	2946	3059	3182	3188	3218	3223	3272	3317	3392	3455	3487	3505	3538	3547	3556	3569	3580	3584	3605	3619	3679	3728	3746	3760	3764	3989	4183	4222	4228	4258	4344	4402	4423	4440	4441	4446	4511	4521	4535	4601	4644	4645	4707	4735	4793	4801	4858	4890	4898	4914	4921	4993	5170	5270	5342	5421	5460	5574	5583	5596	5602	5656	5745	5767
AY548170	1	t	t	t	t	a	t	t	t	t	t	t	a	g	g	t	c	a	t	t	t	a	a	t	t	g	t	c	g	g	a	a	c	a	g	c	c	t	c	g	g	g	t	g	t	t	g	t	a	g	t	a	c	t	t	g	c	a	a	t	a	g	g	g	c	t	t	c	a	t	t	a	a	g	g	a	a	t	t	c	g	t	a	a	g	t	a	c
AY548171	1	t	t	t	t	a	t	t	t	t	t	t	a	g	g	t	c	a	t	t	t	a	a	t	t	g	t	c	g	g	a	a	c	a	g	c	c	t	c	g	g	g	t	g	t	t	g	t	a	g	t	a	c	t	t	g	c	a	a	t	a	g	g	g	c	t	g	c	a	t	t	a	a	g	g	a	a	t	t	c	g	t	a	a	g	t	a	c
DQ479953	1	t	t	t	t	a	t	t	t	t	t	t	a	g	g	t	c	a	t	t	t	a	a	t	t	a	t	c	g	g	a	a	c	a	g	c	c	t	c	g	g	g	t	g	t	t	g	t	a	g	t	a	c	t	t	g	c	a	a	t	a	g	g	g	c	t	g	c	a	t	t	a	a	g	g	a	a	t	t	c	g	t	a	a	g	t	a	c
DQ479954	1	t	t	t	t	a	t	t	t	t	t	t	a	g	g	t	c	a	t	t	t	a	a	t	t	g	t	c	g	g	a	a	c	a	g	c	c	t	c	g	g	g	t	g	t	t	g	t	a	g	t	a	c	t	t	g	c	a	a	t	a	g	g	g	c	t	g	c	a	t	t	a	a	g	g	a	a	t	g	c	g	t	a	a	g	t	a	c
DQ479958	1	t	t	t	t	a	t	t	t	t	t	t	a	g	g	t	c	a	t	t	t	a	a	t	t	g	t	c	g	g	a	a	c	a	g	c	c	t	c	g	g	g	t	g	t	t	g	t	a	g	t	a	c	t	t	g	c	a	a	t	a	g	g	g	c	t	g	c	a	t	t	a	a	g	g	a	a	t	t	c	g	t	a	a	g	t	a	c
DQ479959	1	t	t	t	t	a	t	t	t	t	t	t	a	g	g	t	c	a	t	t	t	a	a	t	t	g	t	c	g	g	a	a	c	a	g	c	c	t	c	g	g	g	t	g	t	t	g	t	a	g	t	a	c	t	t	g	c	a	a	t	a	g	g	g	c	t	g	c	a	t	t	a	a	g	g	a	a	t	t	c	g	t	a	a	g	t	a	c
DQ479961	1	t	t	t	t	a	t	t	t	t	t	t	a	g	g	t	c	a	t	t	t	a	a	t	t	g	t	c	g	g	a	a	c	a	g	c	c	t	c	g	g	g	t	g	t	t	g	t	a	g	t	a	c	t	t	g	c	a	a	t	a	g	g	g	c	t	g	c	a	t	t	a	a	g	g	a	a	t	t	c	g	t	a	a	g	t	a	c
DQ479962	1	t	t	t	t	a	t	t	t	t	t	t	a	g	g	t	c	a	t	t	t	a	a	t	t	g	t	c	g	g	a	a	c	a	g	c	c	t	c	g	g	g	t	g	t	t	g	t	a	g	t	a	c	t	t	g	c	a	a	t	a	g	g	g	c	t	g	c	a	t	t	a	a	g	g	a	a	t	t	c	g	t	a	a	g	t	a	c
DQ479963	1	t	t	t	t	a	t	t	t	t	t	t	a	g	g	t	c	a	t	t	t	a	a	t	t	g	t	c	g	g	a	a	c	a	g	c	c	t	c	g	g	g	t	g	t	t	g	t	a	g	t	a	c	t	t	g	c	a	a	t	a	g	g	g	c	t	g	c	a	t	t	a	a	g	g	a	a	t	t	c	g	t	a	a	g	t	a	c
DQ674250	1	t	t	t	t	a	t	t	t	t	t	t	a	g	g	t	c	a	t	t	t	a	a	t	t	g	t	c	g	g	a	a	c	a	g	c	c	t	c	g	g	g	t	g	t	t	g	t	a	g	t	a	c	t	t	g	c	a	a	t	a	g	g	g	c	t	g	c	a	t	t	a	a	g	g	a	a	t	t	c	g	t	a	a	g	t	a	c
KX262864	1	t	t	t	t	a	t	t	t	t	t	t	a	g	g	t	c	a	t	t	t	a	a	t	t	g	t	c	g	g	a	a	c	a	g	c	c	t	c	g	g	g	t	g	t	t	g	t	a	g	t	a	c	t	t	g	c	a	a	t	a	g	g	g	c	t	g	c	a	t	t	a	a	g	g	a	a	t	t	c	g	t	a	a	g	t	a	c
KX262865	1	t	t	t	t	a	t	t	t	t	t	t	a	g	g	t	c	a	t	t	t	a	a	t	t	g	t	c	g	g	a	a	c	a	g	c	c	t	c	g	g	g	t	g	t	t	g	t	a	g	t	a	c	t	t	g	c	a	a	t	a	g	g	g	c	t	g	c	a	t	t	a	a	g	g	a	a	t	t	c	g	t	a	a	g	t	a	c
KX262866	1	t	t	t	t	a	t	t	t	t	t	t	a	g	g	t	c	a	t	t	t	a	a	t	t	g	t	c	g	g	a	a	c	a	g	c	c	t	c	g	g	g	t	g	t	t	g	t	a	g	t	a	c	t	t	g	c	a	a	t	a	g	g	g	c	t	g	c	a	t	t	a	a	g	g	a	a	t	t	c	g	t	a	a	g	t	a	c
EU154348	1	t	t	t	t	a	t	t	t	t	t	t	a	g	g	t	c	a	t	t	t	a	a	t	t	g	t	c	g	g	a	a	c	a	g	c	c	t	c	g	g	g	t	g	t	t	g	t	a	g	t	a	c	t	t	g	c	a	a	t	a	g	g	g	c	t	g	c	a	t	t	a	a	g	g	a	a	t	t	c	g	t	a	a	g	t	a	c
JN704690	1	t	t	t	t	a	t	t	t	t	t	t	a	g	g	t	c	a	t	t	t	a	a	t	t	g	t	c	g	g	a	a	c	a	g	c	c	t	c	g	g	g	t	g	t	t	g	t	a	g	t	a	c	t	t	g	c	a	a	t	a	g	g	g	c	t	g	c	a	t	t	a	a	g	g	a	a	t	t	c	g	t	a	a	g	t	a	c
JN704691	1	t	t	t	t	a	t	t	t	t	t	t	a	g	g	t	c	a	t	t	t	a	a	t	t	g	t	c	g	g	a	a	c	a	g	c	c	t	c	g	g	g	t	g	t	t	g	t	a	g	t	a	c	t	t	g	c	a	a	t	a	g	g	g	c	t	g	c	a	t	t	c	a	g	g	a	a	t	t	c	g	t	a	a	g	t	a	c
JN704692	1	t	t	t	t	a	t	t	t	t	t	t	a	g	g	t	c	a	t	t	t	a	a	t	t	g	t	c	g	g	a	a	c	a	g	c	c	t	c	g	g	g	t	g	t	t	g	t	a	g	t	a	c	t	t	g	c	a	a	t	a	g	g	g	c	t	g	c	a	t	c	a	a	g	g	a	a	t	t	c	g	t	a	a	g	t	a	c
JN704693	1	t	t	t	t	a	t	t	t	t	t	t	a	g	g	t	c	a	t	t	t	a	a	t	t	g	t	c	g	g	a	a	c	a	g	c	c	t	c	g	g	g	t	g	t	t	g	t	a	g	t	a	c	t	t	g	c	a	a	t	a	g	g	g	c	t	g	c	a	t	t	a	a	g	g	a	a	t	t	c	g	t	a	a	g	t	a	c
JN704694	1	t	t	t	t	a	t	t	t	t	t	t	a	g	g	t	c	a	t	t	t	a	a	t	t	g	t	c	g	g	a	a	c	a	g	c	c	t	c	g	g	g	t	g	t	t	g	t	a	g	t	a	c	t	t	g	c	a	a	t	a	g	g	g	c	t	g	c	a	t	t	a	a	g	g	a	a	t	t	c	g	t	a	a	g	t	a	c
JN704695	1	t	t	t	t	a	t	t	t	t	t	t	a	g	g	t	c	a	t	t	t	a	a	t	t	g	t	c	g	g	a	a	c	a	g	c	c	t	c	g	g	g	t	g	t	t	g	t	a	g	t	a	c	t	t	g	c	a	a	t	a	g	g	g	c	t	g	c	a	t	t	a	a	g	g	a	a	t	t	c	g	t	a	a	g	t	a	c
JN704696	1	t	t	t	t	a	t	t	t	t	t	t	a	g	g	t	c	a	t	t	t	a	a	t	t	g	t	c	g	g	a	a	c	a	g	c	c	t	c	g	g	g	t	g	t	t	g	t	a	g	t	a	c	t	t	g	c	a	a	t	a	g	g	g	c	t	g	c	a	t	t	a	a	g	g	a	a	t	t	c	g	t	a	a	g	t	a	c
NC001348	1	t	t	t	t	a	t	t	t	t	t	t	a	g	g	t	c	a	t	t	t	a	a	t	t	g	t	c	g	g	a	a	c	a	g	c	c	t	c	g	g	g	t	g	t	t	g	t	a	g	t	a	c	c	t	g	c	a	a	t	a	g	g	g	c	t	g	c	a	t	t	a	a	g	g	a	a	t	t	c	g	t	a	a	g	t	a	c
KP771892	1	t	t	t	t	a	t	t	t	t	t	t	a	g	g	t	c	a	t	t	t	a	a	t	t	g	t	c	g	g	a	a	c	a	g	c	c	t	c	g	g	g	t	g	t	t	g	t	a	g	t	a	c	t	t	g	c	a	a	t	a	g	g	g	c	t	g	c	a	t	t	a	a	g	g	a	a	t	t	c	g	t	a	a	g	t	a	c
KP771894	1	t	t	t	t	a	t	t	t	t	t	t	a	g	g	t	c	a	t	t	t	a	a	t	t	g	t	c	g	g	a	a	c	a	g	c	c	t	c	g	g	g	t	g	t	t	g	t	a	g	t	a	c	t	t	g	c	a	a	t	a	g	g	g	c	t	g	c	a	t	t	a	a	g	g	a	a	t	t	c	g	t	a	a	g	t	a	c
KP771900	1	t	t	t	t	a	t	t	t	t	t	t	a	g	g	t	c	a	t	t	t	a	a	t	t	g	t	c	g	g	a	a	c	a	g	c	c	t	c	g	g	g	t	g	t	t	g	t	a	g	t	a	c	t	t	g	c	a	a	t	a	g	g	g	c	t	g	c	a	t	t	a	a	g	g	a	a	t	t	c	g	t	a	a	g	t	a	c
KP771901	1	t	t	t	t	a	t	t	t	t	t	t	a	g	g	t	c	a	t	t	t	a	a	t	t	g	t	c	g	g	a	a	c	a	g	c	c	t	c	g	g	g	t	g	t	t	g	t	a	g	t	a	c	t	t	g	c	a	a	t	a	g	g	g	c	t	g	c	a	t	t	a	a	g	g	a	a	t	t	c	g	t	a	a	g	t	a	c
KP771902	1	t	t	t	t	a	t	t	t	t	t	t	a	g	g	t	c	a	t	t	t	a	a	t	t	g	t	c	g	g	a	a	c	a	g	c	c	t	c	g	g	g	t	g	t	t	g	t	a	g	t	a	c	t	t	g	c	a	a	t	a	g	g	g	c	t	g	c	a	t	t	a	a	g	g	a	a	t	t	c	g	t	a	a	g	t	a	c
KP771908	1	t	t	t	t	a	t	t	t	t	t	t	a	g	g	t	c	a	t	t	t	a	a	t	t	g	t	c	g	g	a	a	c	a	g	c	c	t	c	g	g	g	t	g	t	t	g	t	a	g	t	a	c	t	t	g	c	a	a	t	a	g	g	g	c	t	g	c	a	t	t	a	a	g	g	a	a	t	t	c	g	t	a	a	g	t	a	c
KP771912	1	t	t	t	t	a	t	t	t	t	t	t	a	g	g	t	c	a	t	t	t	a	a	t	t	g	t	c	g	g	a	a	c	a	g	c	c	t	c	g	g	g	t	g	t	t	g	t	a	g	t	a	c	t	t	g	c	a	a	t	a	g	g	g	c	t	g	c	a	t	t	a	a	g	g	a	a	t	t	c	g	t	a	a	g	t	a	c
KP771913	1	t	t	t	t	a	t	t	t	t	t	t	a	g	g	t	c	a	t	t	t	a	a	t	t	g	t	c	g	g	a	a	c	a	g	c	c	t	c	g	g	g	t	g	t	t	g	t	a	g	t	a	c	t	t	g	c	a	a	t	a	g	g	g	c	t	g	c	a	t	t	a	a	g	g	a	a	t	t	c	g	t	a	a	g	t	a	c
KP771917	1	t	t	t	t	a	t	t	t	t	t	t	a	g	g	t	c	a	t	t	t	a	a	t	t	g	t	c	g	g	a	a	c	a	g	c	c	t	c	g	g	g	t	g	t	t	g	t	a	g	t	a	c	t	t	g	c	a	a	t	a	g	g	g	c	t	g	c	a	t	t	a	a	g	g	a	a	t	t	c	g	t	a	a	g	t	a	c
KP771918	1	t	t	t	t	a	t	t	t	t	t	t	a	g	g	t	c	a	t	t	t	a	a	t	t	g	t	c	g	g	a	a	c	a	g	c	c	t	c	g	g	g	t	t	t	t	g	t	a	g	t	a	c	t	t	g	c	a	a	t	a	g	g	g	c	t	g	c	a	t	t	a	a	g	g	a	a	t	t	c	g	t	a	a	g	t	a	c
KP771920	1	t	t	t	t	a	t	t	t	t	t	t	a	g	g	t	c	a	t	t	t	a	a	t	t	g	t	c	g	g	a	a	c	a	g	c	c	t	c	g	g	g	t	g	t	t	g	t	a	g	t	a	c	t	t	g	c	a	a	t	a	g	g	g	c	t	g	c	a	t	t	a	a	g	g	a	a	t	t	c	g	t	a	a	g	t	a	c
MH709361	1	t	t	t	t	a	t	t	t	t	t	t	a	g	g	t	c	a	t	t	t	a	a	t	t	g	t	c	g	g	a	a	c	a	g	c	c	t	c	g	g	g	t	g	t	t	g	t	a	g	t	a	c	t	t	g	c	a	a	t	a	g	g	g	c	t	g	t	a	t	t	a	a	g	g	a	a	t	t	c	g	t	a	a	g	t	a	c
KU926318	2	t	c	t	t	g	t	c	t	t	t	t	a	g	g	g	c	a	t	t	t	a	a	t	c	g	c	c	g	g	a	a	c	a	t	c	c	t	c	g	g	g	t	g	t	t	g	t	g	g	t	a	c	t	t	g	c	g	a	t	a	g	g	g	c	t	g	c	a	t	t	a	a	g	g	a	g	t	t	c	g	t	a	g	g	t	a	c
KU926319	2	c	t	t	t	a	t	t	t	t	t	t	a	g	g	t	c	a	t	t	t	a	a	t	t	g	t	c	g	g	a	a	c	a	g	c	c	t	c	g	g	g	t	g	t	t	g	t	g	g	t	a	c	t	t	g	c	a	a	t	a	g	g	g	c	t	g	c	a	t	t	a	a	g	g	a	a	t	t	c	g	t	a	g	g	t	a	c
KU926320	2	c	t	t	t	a	t	t	t	t	t	t	a	g	g	t	c	a	t	t	t	a	a	t	t	g	t	c	g	g	a	a	c	a	g	c	c	t	c	g	g	g	t	g	t	t	g	t	g	g	t	a	c	t	t	g	c	a	a	t	a	g	g	g	c	t	g	c	a	t	t	a	a	g	g	a	a	c	t	c	g	t	a	g	g	c	a	c
KU926321	2	t	t	t	t	a	t	t	t	t	t	t	a	g	g	t	c	a	t	t	t	a	a	t	t	g	t	c	g	g	a	a	c	a	g	c	c	t	c	g	g	g	t	g	t	t	g	t	g	g	t	a	c	t	t	g	c	a	a	t	a	g	g	g	c	t	g	c	a	t	t	a	a	g	g	a	a	t	t	c	g	t	a	a	g	t	a	c
KU926322	2	t	t	t	t	a	t	t	t	t	t	t	a	g	g	t	c	a	t	t	t	a	a	t	t	g	t	c	g	g	a	a	c	a	g	c	c	t	c	g	g	g	t	g	t	t	g	t	g	g	t	a	c	t	t	g	c	a	a	t	a	g	g	g	c	t	g	c	a	t	t	a	a	g	g	a	a	t	t	c	g	t	a	a	g	t	a	c
KC847290	2	t	t	t	t	a	t	t	t	t	t	t	a	g	g	t	c	a	t	t	t	a	a	t	t	g	t	c	g	g	a	a	c	a	g	c	c	t	c	g	g	g	t	g	t	t	g	t	g	g	t	a	c	t	t	g	c	a	a	t	a	g	g	g	c	t	g	c	a	t	t	a	a	g	g	a	a	t	t	c	g	t	a	a	g	t	a	c
KJ767491	2	t	t	t	t	a	t	t	t	t	t	t	a	g	g	g	c	a	t	t	t	a	a	t	t	g	t	c	g	g	a	a	c	a	t	c	c	t	c	g	g	g	t	g	t	t	g	t	g	g	t	a	c	t	t	g	c	g	a	t	a	g	g	g	c	t	g	c	a	t	t	a	a	g	g	a	a	t	t	c	g	t	a	a	g	t	a	c
KJ767492	2	t	t	t	t	a	t	t	t	t	t	t	a	g	g	t	c	a	t	t	t	a	a	t	t	g	t	c	g	g	a	a	c	a	g	c	c	t	c	g	g	g	t	g	t	t	g	t	g	g	t	a	c	t	t	g	c	a	a	t	a	g	g	g	c	t	g	c	a	t	t	a	a	g	g	a	a	t	t	c	g	t	a	a	g	t	a	c
KJ808816	2	t	t	t	t	a	t	t	t	t	t	t	a	g	g	t	c	a	t	t	t	a	a	t	t	g	t	c	g	g	a	a	c	a	g	c	c	t	c	g	g	g	t	g	t	t	g	t	g	g	t	a	c	t	t	g	c	a	a	t	a	g	g	g	c	t	g	c	a	t	t	a	a	g	g	a	a	t	t	c	g	t	a	a	g	t	a	c
KP771925	2	t	t	t	t	a	t	t	t	t	t	t	a	g	g	t	c	a	t	t	t	a	a	t	t	g	t	c	g	g	a	a	c	a	g	c	c	t	c	g	g	g	t	g	t	t	g	t	g	g	t	a	c	t	t	g	c	a	c	t	a	g	g	g	c	t	g	c	a	t	t	a	a	g	g	a	a	t	t	c	g	t	a	a	g	t	a	c
KU926315	2	t	t	t	t	a	t	t	t	t	t	t	a	g	g	g	c	a	t	t	t	a	a	t	t	g	t	c	g	g	a	a	c	a	t	c	c	t	c	g	g	g	t	g	t	t	g	t	g	g	t	a	c	t	t	g	c	g	a	t	a	g	g	g	c	t	g	c	a	t	t	a	a	g	g	a	a	t	t	c	g	t	a	a	g	t	a	c
KU926316	2	t	t	t	t	a	t	t	t	t	t	t	a	g	g	g	c	a	t	t	t	a	a	t	t	g	t	c	g	g	a	a	c	a	t	c	c	t	c	g	g	g	t	g	t	t	g	t	g	g	t	a	c	t	t	g	c	g	a	t	a	g	g	g	c	t	g	c	a	t	t	a	a	g	g	a	a	t	t	c	g	t	a	a	g	t	a	c
KU926317	2	t	t	t	t	a	t	t	t	t	t	t	a	g	g	g	c	a	t	t	t	a	a	t	t	g	t	c	g	g	a	a	c	a	t	c	c	t	c	g	g	g	t	g	t	t	g	t	g	g	t	a	c	t	t	g	c	a	a	t	a	g	g	g	c	t	g	c	a	t	t	a	a	g	g	a	a	t	t	c	g	t	a	a	g	t	a	c
KY037796	2	t	t	t	t	a	t	t	t	t	t	t	a	g	g	t	c	a	t	t	t	a	a	t	t	g	t	c	g	g	a	a	c	a	g	c	c	t	c	g	g	g	t	g	t	t	g	t	g	g	t	a	c	t	t	g	c	a	a	t	a	g	g	g	c	t	g	c	a	t	t	a	a	g	g	a	a	t	t	c	g	t	a	a	g	t	a	c
MF004348	2	t	t	t	t	a	t	t	t	t	t	t	a	g	g	t	c	a	t	t	t	a	a	t	t	g	t	c	g	g	a	a	c	a	g	c	c	t	c	g	g	g	t	g	t	t	g	t	g	g	t	a	c	t	t	g	c	a	a	t	a	g	g	g	c	t	g	c	a	t	t	a	a	g	g	a	a	t	t	c	g	t	a	a	g	t	a	c
MH709309	2	t	t	t	t	a	t	t	t	t	t	t	a	g	g	t	c	a	t	t	t	a	a	t	t	g	t	c	g	g	a	a	c	a	g	c	c	t	c	g	g	g	t	g	t	t	g	t	g	g	t	a	c	t	t	g	c	a	a	t	a	g	g	g	c	t	g	c	a	t	t	a	a	g	g	a	a	t	t	c	g	t	a	a	g	t	a	c
MH709310	2	t	t	t	t	a	t	t	t	t	t	t	a	g	g	t	c	a	t	t	t	a	a	t	t	g	t	c	g	g	a	a	c	a	g	c	c	t	c	g	g	g	t	g	t	t	g	t	g	g	t	a	c	t	t	g	c	a	a	t	a	g	g	g	c	t	g	c	a	t	t	a	a	g	g	a	a	t	t	c	g	t	a	a	g	t	a	c
MH709311	2	t	t	t	t	a	t	t	t	t	t	t	a	g	g	t	c	a	t	t	t	a	a	t	t	g	t	c	g	g	a	a	c	a	g	c	a	t	c	g	g	g	t	g	t	t	g	t	g	g	t	a	c	t	t	g	c	a	a	t	a	g	g	g	c	t	g	c	a	t	t	a	a	g	g	a	a	t	t	c	g	t	a	a	g	t	a	c
MH709313	2	t	t	t	t	a	t	t	t	t	t	t	a	g	g	t	t	a	t	t	t	a	a	t	t	g	t	c	g	g	a	a	c	a	g	c	c	t	c	g	g	g	t	g	t	t	g	t	g	g	t	a	c	t	t	g	c	a	a	t	a	g	g	g	c	t	g	c	a	t	t	a	a	g	g	a	a	t	t	c	g	t	a	a	g	t	a	c
MH709314	2	t	t	t	t	a	t	t	t	t	t	t	a	g	g	t	c	a	t	t	t	a	a	t	t	g	t	c	g	g	a	a	c	a	g	c	c	t	c	g	g	g	t	g	t	t	g	t	g	g	t	a	c	t	t	g	c	a	a	t	a	g	g	g	c	t	g	c	a	t	t	a	a	g	g	a	a	t	t	c	g	t	c	a	g	t	a	c
MH709315	2	t	t	t	t	a	t	t	t	t	t	t	a	g	g	t	c	a	t	t	t	a	a	t	t	g	t	c	g	g	a	a	c	a	g	c	c	t	c	g	g	g	t	g	t	t	g	t	g	g	t	a	c	t	t	g	c	a	a	t	a	g	g	g	c	t	g	c	a	t	t	a	a	g	g	a	a	t	t	c	g	t	a	a	g	t	a	c
MH709319	2	t	t	t	t	a	t	t	t	t	t	t	a	g	g	t	c	a	t	t	t	a	a	t	t	g	t	c	g	g	a	a	c	a	g	c	c	t	c	g	g	g	t	g	t	t	g	t	g	g	t	a	c	t	t	g	c	a	a	t	a	g	g	g	c	t	g	c	a	t	t	a	a	g	g	a	a	t	t	c	g	t	a	a	g	t	a	c
MH709320	2	t	t	t	t	a	t	t	t	t	t	t	a	g	g	t	c	a	t	t	t	a	a	t	t	g	t	c	g	g	a	a	c	a	g	c	c	t	c	g	g	g	t	g	t	t	g	t	g	g	t	a	c	t	t	g	c	a	a	t	a	g	g	g	c	t	g	c	a	t	t	a	a	g	g	a	a	t	t	c	g	t	a	a	g	t	a	c
MH709329	2	t	t	t	t	a	t	t	t	t	t	t	a	g	g	t	c	a	t	t	t	a	a	t	t	g	t	c	g	g	a	a	c	a	g	c	c	t	c	a	g	g	t	g	t	t	g	t	g	g	t	a	c	t	t	g	c	a	a	t	a	g	g	g	c	t	g	c	a	t	t	a	a	g	g	a	a	t	t	c	g	t	a	a	g	t	a	c
MH709331	2	t	t	t	t	a	t	t	t	t	t	t	a	g	g	t	c	a	t	t	t	a	a	t	t	g	t	c	g	g	a	a	c	a	g	c	c	t	c	g	g	g	t	g	t	t	g	t	g	g	t	a	c	t	t	g	c	a	a	t	a	g	g	g	c	t	g	c	a	t	t	a	a	g	g	a	a	t	t	c	g	t	a	a	g	t	a	c
MH709333	2	t	t	t	t	a	t	t	t	t	t	t	a	g	g	t	c	a	t	t	t	a	a	t	t	g	t	c	g	g	a	a	c	a	g	c	c	t	c	g	g	g	t	g	t	t	g	t	g	g	t	a	c	t	t	g	c	a	a	t	a	g	g	g	c	t	g	c	a	t	t	a	a	g	g	a	a	t	t	c	g	t	a	a	g	t	a	c
MH709337	2	t	t	t	t	a	t	t	t	t	t	t	a	a	g	t	c	a	t	t	t	a	a	t	t	g	t	c	g	g	a	a	c	a	g	c	c	t	c	g	g	g	t	g	t	t	g	t	g	g	t	a	c	t	t	g	c	a	a	t	a	g	g	g	c	t	g	c	a	t	t	a	a	g	g	a	a	t	t	c	g	t	a	a	g	t	a	c
MH709339	2	t	t	t	t	a	t	t	t	t	t	t	a	g	g	g	c	a	t	t	t	a	a	t	t	g	t	c	g	g	a	a	c	a	t	c	c	t	c	g	g	g	t	g	t	t	g	t	g	g	t	a	c	t	t	g	c	a	a	t	a	g	g	g	c	t	g	c	a	t	t	a	a	g	g	a	a	t	t	c	g	t	a	a	g	t	a	c
MH709344	2	t	t	t	t	a	t	t	t	t	t	t	a	g	g	t	c	a	t	g	t	a	a	t	t	g	t	c	g	g	a	a	c	a	g	c	c	t	c	g	g	g	t	g	t	t	g	t	g	g	t	a	c	t	t	g	c	a	a	t	a	g	g	g	c	t	g	c	a	t	t	a	a	g	g	a	a	t	t	c	g	t	a	a	g	t	a	c
MH709348	2	t	t	t	t	a	t	t	t	t	t	t	a	g	g	t	c	a	t	t	t	a	a	t	t	g	t	c	g	g	a	a	c	a	g	c	c	t	c	g	g	g	t	g	t	t	g	t	g	g	t	a	c	t	t	g	c	a	a	t	a	g	g	g	c	t	g	c	a	t	t	a	a	g	g	g	a	t	t	c	g	t	a	a	g	t	a	c
MH709355	2	t	t	t	t	a	t	t	t	t	t	t	a	g	g	t	c	a	t	t	t	a	a	t	t	g	t	c	g	g	a	a	c	a	g	c	c	t	c	g	g	g	t	g	t	t	g	t	g	g	t	a	c	t	t	g	c	a	a	t	a	g	g	g	c	t	g	c	a	t	t	a	a	g	g	a	a	t	t	c	g	t	a	a	g	t	a	c
MH709359	2	t	t	t	t	a	t	t	t	t	t	t	a	g	g	t	c	a	t	t	t	a	a	t	t	g	t	c	g	g	a	a	c	a	g	c	c	t	c	g	g	g	t	g	t	t	g	t	g	g	t	a	c	t	t	g	c	a	a	t	a	g	g	g	c	t	g	c	a	t	t	a	a	g	g	a	a	t	t	c	g	t	a	a	g	t	a	c
MH709363	2	t	t	t	t	a	t	t	t	t	t	t	a	g	g	t	c	a	t	t	t	a	a	t	t	g	t	c	g	g	a	a	c	a	g	c	c	t	c	g	g	g	t	g	t	t	g	t	g	g	t	a	c	t	t	g	c	a	a	t	a	g	g	g	c	t	g	c	a	t	t	a	a	g	g	a	a	t	t	c	g	t	a	a	g	t	a	c
MH709366	2	t	t	t	t	a	t	t	t	t	t	t	a	g	g	t	c	a	t	t	t	a	a	c	t	g	t	c	g	g	a	a	c	a	g	c	c	t	c	g	g	g	t	g	t	t	g	t	g	g	t	a	c	t	t	g	c	a	a	t	a	g	g	g	c	t	g	c	a	t	t	a	a	g	g	a	a	t	t	c	g	t	a	a	g	t	a	c
MH709376	2	t	t	t	t	a	t	t	t	t	t	t	a	a	g	t	c	a	t	t	t	a	a	t	t	g	t	c	g	g	a	a	c	a	g	c	c	t	c	g	g	g	t	g	t	t	g	t	g	g	t	a	c	t	t	g	c	a	a	t	a	g	g	g	c	t	g	c	a	t	t	a	a	g	g	a	a	t	t	c	g	t	a	a	g	t	a	c
MH709377	2	t	t	t	t	a	t	t	t	t	t	t	a	g	g	t	c	a	t	t	t	a	a	t	t	g	t	c	g	g	a	a	c	a	g	c	c	t	c	g	g	g	t	g	t	t	g	t	g	g	t	a	c	t	t	g	c	a	a	t	a	g	g	g	c	t	g	c	a	t	t	a	a	g	g	a	a	t	t	c	g	t	a	a	g	t	a	c
**MW316406**	2	t	t	t	t	a	t	t	t	t	t	t	a	g	g	t	c	a	t	t	t	a	a	t	t	g	t	c	g	g	a	a	c	a	g	c	c	t	c	g	g	g	t	g	t	t	g	t	g	g	t	a	c	t	t	g	c	a	a	t	a	g	g	g	c	t	g	c	a	t	t	a	a	g	g	a	a	t	t	c	g	t	a	a	g	t	a	c
AJ871403	3	t	t	t	t	a	t	t	t	t	t	c	a	g	a	t	c	a	t	t	t	a	a	t	t	g	t	c	g	g	a	a	c	a	g	c	c	t	c	g	g	g	t	g	t	t	g	t	a	g	t	a	c	t	t	a	c	a	a	t	a	g	g	g	c	t	g	c	a	t	t	a	a	g	g	a	a	t	t	c	g	t	a	a	g	t	a	c
DQ479955	3	t	t	t	t	a	t	t	t	t	t	c	a	g	a	t	c	a	t	t	t	a	a	t	t	g	t	c	g	g	a	a	c	a	g	c	c	t	c	g	g	g	t	g	t	t	g	t	a	g	t	a	c	t	t	a	c	a	a	t	a	g	g	g	c	t	g	c	a	t	t	a	a	g	g	a	a	t	t	c	g	t	a	a	g	t	a	c
DQ479956	3	t	t	t	t	a	t	t	t	t	t	c	a	g	a	t	c	a	t	t	t	a	a	t	t	g	t	c	g	g	a	a	c	a	g	c	c	t	c	g	g	g	t	g	t	t	g	t	a	g	t	a	c	t	t	a	c	a	a	t	a	g	g	g	c	t	g	c	a	t	t	a	a	g	g	a	a	t	t	c	g	t	a	a	g	t	a	c
DQ479957	3	t	t	t	t	a	t	t	t	t	t	c	a	g	a	t	c	a	t	t	t	a	a	t	t	g	t	c	g	g	a	a	c	a	g	c	c	t	c	g	g	g	t	g	t	t	g	t	a	g	t	a	c	t	t	a	c	a	a	t	a	g	g	g	c	t	g	c	a	t	t	a	a	g	g	a	a	t	t	c	g	t	a	a	g	t	a	c
JN704699	3	t	t	t	t	a	t	t	t	t	t	c	a	g	a	t	c	a	t	t	t	a	a	t	t	g	t	c	g	g	a	a	c	a	g	c	c	t	c	g	g	g	t	g	t	t	g	t	a	g	t	a	c	t	t	g	c	a	a	t	a	g	g	g	c	t	g	c	a	t	t	a	a	g	g	a	a	t	t	c	g	t	a	a	g	t	a	c
JN704700	3	t	t	t	t	a	t	t	t	t	t	c	a	g	a	t	c	a	t	t	t	a	a	t	t	g	t	c	g	g	a	a	c	a	a	c	c	t	c	g	g	g	g	g	t	t	g	t	a	g	t	a	c	t	t	g	c	a	a	t	a	g	g	g	t	t	g	c	a	t	t	a	a	g	g	a	a	t	t	c	g	t	a	a	g	t	a	c
JN704701	3	t	t	t	t	a	t	t	t	t	t	c	a	g	a	t	c	a	t	t	t	a	a	t	t	g	t	c	g	g	a	a	c	a	g	c	c	t	c	g	g	g	t	g	t	t	g	t	a	g	t	a	c	t	c	g	c	a	a	t	a	g	g	g	c	t	g	c	a	t	t	a	a	g	g	a	a	t	t	c	g	t	a	a	g	t	a	c
JN704702	3	t	t	t	t	a	t	t	t	t	t	c	a	g	a	t	c	a	t	t	t	a	a	t	t	g	t	c	g	g	a	a	c	a	g	c	c	t	c	g	g	g	t	g	t	c	g	t	a	g	t	a	c	t	t	g	c	a	a	t	a	g	g	g	t	t	g	c	a	t	t	a	a	g	g	a	a	t	t	c	g	t	a	a	g	t	a	c
JN704703	3	t	t	t	t	a	t	t	t	t	t	c	a	g	a	t	c	a	t	t	t	a	a	t	t	g	t	c	g	g	a	a	c	a	g	c	c	t	c	g	g	g	t	g	t	t	g	t	a	g	t	a	c	t	t	g	c	a	a	t	a	a	g	g	t	t	g	c	a	t	t	a	a	g	g	a	a	t	t	c	g	t	a	a	g	t	a	c
KP771891	3	t	t	t	t	a	t	t	t	t	t	c	a	g	a	t	c	a	t	t	t	a	a	t	t	g	t	c	g	g	a	a	c	a	g	c	c	t	c	g	g	g	t	g	t	t	g	t	a	g	t	a	c	t	t	g	c	a	a	t	a	g	g	g	c	t	g	c	a	t	t	a	a	g	g	a	a	t	t	c	g	t	a	a	g	t	a	c
KP771893	3	t	t	t	t	a	t	t	t	t	t	c	a	g	a	t	c	a	t	t	t	a	a	t	t	g	t	c	g	g	a	a	c	a	g	t	c	t	c	g	g	g	t	g	t	t	g	t	a	g	t	a	c	t	t	g	c	a	a	t	a	g	g	g	c	t	g	c	a	t	t	a	a	g	g	a	a	t	t	c	g	t	a	a	g	t	a	c
KP771895	3	t	t	t	t	a	t	t	t	t	t	c	a	g	a	t	c	a	t	t	t	a	a	t	t	g	t	c	g	g	a	a	c	a	g	c	c	t	c	g	g	g	t	g	t	t	g	t	a	g	t	a	c	t	t	a	c	a	a	t	a	g	g	g	c	t	g	c	a	t	t	a	a	g	g	a	a	t	t	c	g	t	a	a	g	t	a	c
KP771896	3	t	t	t	t	a	t	t	t	t	t	c	a	g	a	t	c	a	t	t	t	a	a	t	t	g	t	c	g	g	a	a	c	a	g	c	c	t	c	g	g	g	t	g	t	t	g	t	a	g	t	a	c	t	t	g	c	a	a	t	a	g	g	g	c	t	g	c	a	c	t	a	a	g	g	a	a	t	t	c	g	t	a	a	a	t	a	c
KP771897	3	t	t	t	t	a	t	t	t	t	t	c	a	g	a	t	c	a	t	t	t	a	a	t	t	g	t	c	g	g	a	a	t	a	g	c	c	t	c	g	g	g	t	g	t	t	g	t	a	g	t	a	c	t	t	a	c	a	a	t	a	g	g	g	c	t	g	c	a	t	t	a	a	g	g	a	a	t	t	c	g	t	a	a	g	t	a	c
KP771899	3	t	t	t	t	a	t	t	t	t	t	c	a	g	a	t	c	a	t	t	t	a	a	t	t	g	t	c	g	g	a	a	c	a	g	c	c	t	c	g	g	g	t	g	t	t	g	t	a	g	t	a	c	t	t	a	c	a	a	t	a	g	g	a	c	t	g	c	a	t	t	a	a	g	g	a	a	t	t	c	g	t	a	a	g	t	a	c
KP771903	3	t	t	t	t	a	t	t	t	t	t	c	a	g	a	t	c	a	t	t	t	a	a	t	t	g	t	c	g	g	a	a	c	a	g	c	c	t	c	g	g	g	t	g	t	t	g	t	a	t	t	a	c	t	t	g	c	a	a	t	a	g	g	g	c	t	g	c	a	t	t	a	a	g	g	a	a	t	t	c	g	t	a	a	g	t	a	c
KP771907	3	t	t	t	t	a	t	t	t	t	t	c	a	g	a	t	c	a	t	t	t	a	a	t	t	g	t	c	g	g	a	a	c	a	g	c	c	t	c	g	g	g	t	g	t	t	g	t	a	g	t	a	c	t	t	a	c	a	a	t	a	g	g	g	c	t	g	c	a	t	t	a	a	g	g	a	a	t	t	c	g	t	a	a	g	t	a	c
KP771910	3	t	t	t	t	a	t	t	t	t	t	c	a	g	a	t	c	a	t	t	t	a	a	t	t	g	t	c	g	g	a	a	c	a	g	c	c	t	c	g	g	g	t	g	t	t	g	t	a	g	t	a	c	t	t	g	c	a	a	t	a	g	g	g	c	t	g	c	a	t	t	a	a	g	g	a	a	t	t	c	g	t	a	a	g	t	a	c
KP771915	3	t	t	t	t	a	t	t	t	t	t	c	a	g	a	t	c	a	t	t	t	a	a	t	t	g	t	c	g	g	a	a	t	a	g	c	c	t	c	g	g	g	t	g	t	t	g	t	a	g	t	a	c	t	t	a	c	a	a	t	a	g	g	g	c	t	g	c	a	t	t	a	a	g	g	a	a	t	t	c	g	t	a	a	g	t	a	c
KP771916	3	t	t	t	t	a	t	t	t	t	t	c	a	g	a	t	c	a	t	t	t	a	a	t	t	g	t	c	g	g	a	a	c	a	g	c	c	t	c	g	g	g	t	g	t	t	g	t	a	g	t	a	c	t	t	a	c	a	a	t	a	g	g	g	c	t	g	c	a	t	t	a	a	g	g	a	a	t	t	c	g	t	a	a	g	t	a	c
KP771921	3	t	t	t	t	a	t	t	t	t	t	c	a	g	a	t	c	a	t	t	t	a	a	t	t	g	t	c	g	g	a	a	c	a	g	c	c	t	c	g	g	g	t	g	t	t	g	t	a	g	t	a	c	t	t	g	c	a	a	t	a	g	g	g	c	t	g	c	a	t	t	a	a	g	g	a	a	t	t	c	g	t	a	a	g	t	a	c
KP771923	3	t	t	t	t	a	t	t	t	t	t	c	a	g	a	t	c	a	t	t	t	a	a	t	t	g	t	c	g	g	a	a	c	a	g	c	c	t	c	g	g	g	t	g	t	t	g	t	a	g	t	a	c	t	t	g	c	a	a	t	a	g	g	g	c	t	g	c	a	t	t	a	a	g	g	a	a	t	t	c	g	t	a	a	g	t	a	c
KP771889	3	t	t	t	t	a	t	t	t	t	t	t	a	g	a	t	c	a	t	t	t	a	a	t	t	g	t	c	g	g	a	a	t	a	g	c	c	t	c	g	g	g	t	g	t	t	g	t	a	g	t	a	c	t	t	a	c	a	a	t	a	g	g	g	c	t	g	c	a	t	t	a	a	g	g	a	a	t	t	c	g	t	a	a	g	t	a	c
KU926311	3	t	t	t	t	a	t	t	t	t	t	c	a	g	a	t	c	a	t	t	t	a	a	t	t	g	t	c	g	g	a	a	c	a	g	c	c	t	c	g	g	g	t	g	t	t	g	t	a	g	g	a	c	t	t	a	c	a	a	t	a	g	g	g	c	t	g	c	a	t	t	a	a	g	g	a	a	t	t	t	g	t	a	a	g	t	a	c
JQ911599	3	t	t	t	t	a	t	t	t	t	t	c	g	g	a	t	c	a	t	t	t	a	g	t	t	g	t	c	g	g	a	a	c	a	a	c	c	t	c	g	g	g	t	g	t	t	g	t	a	g	t	a	c	t	t	g	c	a	a	t	a	g	g	g	t	t	g	c	a	t	t	a	a	a	g	a	a	t	t	c	g	t	a	a	g	t	a	c
MH709317	3	t	t	t	t	a	t	t	t	t	t	c	a	g	a	t	c	a	t	t	t	a	a	t	t	g	t	t	g	g	a	a	c	a	g	c	c	t	c	g	g	g	t	g	t	t	g	t	g	g	t	a	c	t	t	g	c	a	a	t	a	g	g	g	c	t	g	c	a	t	t	a	a	g	g	a	a	t	t	c	g	t	a	a	g	t	a	c
MH709346	3	t	t	t	t	a	t	t	t	t	t	c	a	g	a	t	c	a	t	t	t	a	a	t	t	g	t	t	g	g	a	a	c	a	g	c	c	t	c	g	g	g	t	g	t	t	g	t	g	g	t	a	c	t	t	g	c	a	a	t	a	g	g	g	c	t	g	c	a	t	t	a	a	g	g	a	a	t	t	c	g	t	a	a	g	t	a	c
MH709375	3	t	t	t	t	a	t	t	t	t	t	c	a	g	a	t	c	a	t	t	t	a	a	t	t	g	t	t	a	g	a	a	c	a	g	c	c	t	c	g	g	g	t	g	t	t	g	t	g	g	t	a	c	t	t	g	c	a	a	t	a	g	g	g	c	t	g	c	a	t	t	a	a	g	g	a	a	t	t	c	g	t	a	a	g	t	a	c
DQ452050	4	t	t	t	t	a	t	t	t	t	t	t	a	g	g	t	c	a	t	t	t	a	a	t	t	g	t	c	g	g	a	a	c	a	g	c	c	t	c	g	g	g	t	g	t	t	g	t	g	g	t	a	c	t	t	g	c	a	a	t	a	g	g	g	c	t	g	c	a	t	t	a	a	g	g	a	a	t	t	c	g	t	a	a	g	t	a	c
DQ479960	4	t	t	t	t	a	t	t	t	t	t	t	a	g	g	t	c	a	t	t	t	a	a	t	t	g	t	c	g	g	a	g	c	a	g	c	c	t	c	g	g	g	t	g	t	t	g	t	g	g	t	a	c	t	t	g	c	a	a	t	a	g	g	g	c	t	g	c	a	t	t	a	a	g	g	a	a	t	t	c	g	t	a	a	g	t	a	c
KP771898	4	t	t	t	t	a	t	t	t	t	t	t	a	g	g	t	c	a	t	t	t	a	a	t	t	g	t	c	g	g	a	a	c	a	g	c	c	t	c	g	g	g	t	g	t	t	g	t	g	g	t	a	c	t	t	g	c	a	a	t	a	g	g	g	c	t	g	c	a	t	t	a	a	g	g	a	a	t	t	c	g	t	a	a	g	t	a	c
MH709316	4	t	t	t	c	a	t	t	t	t	c	t	a	g	g	t	c	a	t	t	t	a	a	t	t	g	t	c	g	t	a	a	c	c	g	c	c	t	c	g	g	g	t	g	t	t	g	t	g	g	t	a	t	t	t	g	c	a	a	t	a	g	g	g	c	t	g	c	a	t	t	a	a	g	g	a	a	t	t	c	g	t	a	a	g	t	a	c
MH709318	4	t	t	t	t	a	t	t	t	t	t	t	a	g	g	t	c	a	t	t	t	a	a	t	t	g	t	c	g	g	a	a	c	a	g	c	c	t	c	g	g	g	t	g	t	t	g	t	g	g	t	a	c	t	t	g	c	a	a	t	a	g	g	g	c	t	g	c	a	t	t	a	a	g	g	a	a	t	t	c	g	t	a	a	g	t	a	c
MH709322	4	t	t	t	t	a	t	t	t	t	t	t	a	g	g	t	c	a	t	t	t	a	a	t	t	g	t	c	g	g	a	a	c	a	g	c	c	t	c	g	g	g	t	g	t	t	g	t	g	g	t	a	c	t	t	g	c	a	a	t	a	g	g	g	c	t	g	c	a	t	t	c	a	g	g	a	a	t	t	c	g	t	a	a	g	t	a	c
MH709323	4	t	t	t	t	a	t	t	t	t	t	t	a	g	g	t	c	c	t	t	t	a	a	t	t	g	t	c	g	g	a	a	c	a	g	c	c	t	c	g	g	g	t	g	t	t	g	t	g	g	t	g	c	t	t	g	c	a	a	t	a	g	g	g	c	t	g	c	a	t	t	a	a	g	g	a	a	t	t	c	g	t	a	a	g	t	a	c
MH709326	4	t	t	t	t	a	t	t	t	t	t	t	a	g	g	t	c	a	t	t	t	a	a	t	t	g	t	c	g	g	a	a	c	a	g	c	c	t	c	g	g	g	t	g	t	t	g	t	g	g	t	a	c	t	t	g	c	a	a	t	a	g	g	a	c	t	g	c	a	t	t	a	a	g	g	a	a	t	t	c	g	t	a	a	g	t	a	c
MH709341	4	t	t	t	t	a	t	t	t	t	t	t	a	g	g	t	c	a	t	t	t	a	a	t	t	g	t	c	g	g	g	a	c	a	g	c	c	t	c	g	g	g	t	g	t	t	g	t	g	g	t	a	c	t	t	g	c	a	a	t	a	g	g	g	c	t	g	c	a	t	t	a	a	g	g	a	a	t	t	c	g	t	a	a	g	t	a	c
MH709357	4	t	t	t	t	a	t	t	t	t	t	t	a	g	g	t	c	a	t	t	t	a	a	t	t	g	t	c	g	g	a	g	c	a	g	c	c	t	c	g	g	g	t	g	t	t	g	t	g	g	t	a	c	t	t	g	c	a	a	t	a	g	g	g	c	t	g	c	a	t	t	a	a	g	g	a	a	t	t	c	g	t	a	a	g	t	a	c
MH709364	4	t	t	t	t	a	t	t	t	t	t	t	a	g	g	t	c	a	t	t	t	a	a	t	t	g	t	c	g	g	a	a	c	a	g	c	c	t	c	g	g	g	t	g	t	t	g	t	g	g	t	a	c	t	t	g	c	a	a	t	a	g	g	g	c	t	g	c	a	t	t	a	a	g	g	a	a	t	t	c	g	t	a	a	g	t	a	c
MH709370	4	t	t	t	t	a	t	t	t	t	t	t	a	g	g	t	c	a	t	t	t	a	a	t	t	g	t	c	g	g	a	a	c	a	g	c	c	t	c	g	g	g	t	g	t	t	g	t	g	g	t	a	c	t	t	g	c	a	a	t	a	g	g	g	c	t	g	c	a	t	t	a	a	g	g	a	a	t	t	c	g	t	a	a	g	t	a	c
MH709371	4	t	t	t	t	a	t	t	t	t	t	t	a	g	g	t	c	a	t	t	t	a	a	t	t	g	t	c	g	g	a	g	c	a	g	c	c	t	c	g	g	g	t	g	t	t	g	t	g	g	t	a	c	t	t	g	c	a	a	t	a	g	g	g	c	t	g	c	a	t	t	a	a	g	g	a	a	t	t	c	g	t	a	a	g	t	a	c
MH709372	4	t	t	t	t	a	t	t	t	t	t	t	a	g	g	t	c	a	t	t	t	a	a	t	t	g	t	c	g	g	a	g	c	a	g	c	c	t	c	g	g	g	t	g	t	t	g	t	g	g	t	a	c	t	t	g	c	a	a	t	a	g	g	g	c	t	g	c	a	t	t	a	a	g	g	a	a	t	t	c	g	t	a	a	g	t	a	c
DQ457052	5	t	t	t	t	a	t	t	t	t	g	t	a	g	g	t	c	a	t	t	t	a	a	t	t	g	t	c	g	g	a	a	c	a	g	c	c	t	c	g	g	g	t	g	t	t	g	t	g	g	t	a	c	t	t	g	c	a	a	t	a	g	g	g	c	t	g	c	a	t	t	a	a	g	g	a	a	t	t	c	a	t	a	a	g	t	a	c
JN704704	5	t	t	t	t	a	t	t	t	t	g	t	a	g	g	t	c	a	t	t	t	a	a	t	t	g	t	c	g	g	a	a	c	a	g	c	c	t	c	g	g	g	t	g	t	t	g	t	g	g	t	a	c	t	t	g	c	a	a	t	a	g	g	g	c	t	g	c	a	t	t	a	a	g	g	a	a	t	t	c	g	t	a	a	g	t	a	c
JN704705	5	t	t	t	t	a	t	t	t	t	t	t	a	g	g	t	c	a	t	t	t	a	a	t	t	g	t	c	g	g	a	a	c	a	g	c	c	t	c	g	g	g	t	g	t	t	g	t	g	g	t	a	c	t	t	g	c	a	a	t	a	g	g	g	c	t	g	c	a	t	t	a	a	g	g	a	a	t	t	c	g	t	a	a	g	t	a	c
JN704706	5	t	t	t	t	a	t	t	t	t	t	t	a	g	g	t	c	a	t	t	t	a	a	t	t	g	t	c	g	g	a	a	c	a	g	c	c	t	c	g	t	g	t	g	g	t	g	t	g	g	t	a	c	t	t	g	c	a	a	t	a	g	g	g	c	t	g	c	a	t	t	a	a	g	g	a	a	t	t	c	g	t	a	a	g	t	a	c
JN704707	5	t	t	t	t	a	t	t	t	t	g	t	a	g	g	t	c	a	t	t	t	a	a	t	t	g	t	c	g	g	a	a	c	a	g	c	c	t	c	g	g	g	t	g	t	t	g	t	g	g	t	a	c	t	t	g	c	a	a	t	a	g	g	g	c	t	g	c	a	t	t	a	a	g	g	a	a	t	t	c	g	t	a	a	g	t	a	c
JN704708	5	t	t	t	t	a	t	t	t	t	t	t	a	g	g	t	c	a	t	t	t	a	a	t	t	g	t	c	g	g	a	a	c	a	g	c	c	t	c	g	g	g	t	g	t	t	g	t	g	g	t	a	c	t	t	g	c	a	a	t	a	g	g	g	c	t	g	c	a	t	t	a	a	g	g	a	a	t	t	c	g	t	a	a	g	t	a	c
KP771904	5	t	t	t	t	a	t	t	t	t	g	t	a	g	g	t	c	a	t	t	t	a	a	t	t	g	t	c	g	g	a	a	c	a	g	c	c	t	c	g	g	g	t	g	t	t	g	t	g	g	t	a	c	t	t	g	c	a	a	t	a	g	g	g	c	t	g	c	a	t	t	a	a	g	g	a	a	t	t	c	g	t	a	a	g	t	a	c
KP771905	5	t	t	t	t	a	t	t	t	t	g	t	a	g	g	t	c	a	t	t	t	a	a	t	t	g	t	c	g	g	a	a	c	a	g	c	c	t	c	g	g	g	t	g	t	t	g	t	g	g	t	a	c	t	t	g	c	a	a	t	a	g	g	g	c	c	g	c	a	t	t	a	a	g	g	a	a	t	t	c	g	t	a	a	g	t	a	c
KP771906	5	t	t	t	t	a	t	t	t	t	g	t	a	g	g	t	c	a	t	t	t	a	a	t	t	g	t	c	g	g	a	a	c	a	g	c	c	t	c	g	g	g	t	g	t	t	g	t	g	g	t	a	c	t	t	g	c	a	a	t	a	g	g	g	c	c	g	c	a	t	t	a	a	g	g	a	a	t	t	c	g	t	a	a	g	t	a	c
KP771919	5	t	t	t	t	a	t	t	t	t	g	t	a	g	g	t	c	a	t	t	t	a	a	t	t	g	t	c	g	g	a	a	c	a	g	c	c	t	c	g	g	g	t	g	t	t	g	t	g	g	t	a	c	t	t	g	c	a	a	t	a	g	g	g	c	t	g	c	a	t	t	a	a	g	g	a	a	t	t	c	g	t	a	a	g	t	a	c
KP771922	5	t	t	t	t	a	t	t	t	t	g	t	a	g	g	t	c	a	t	t	t	a	a	t	t	g	t	c	g	g	a	a	c	a	g	c	c	t	t	g	g	g	t	g	t	t	g	t	g	g	t	a	c	t	t	g	c	a	a	t	a	g	g	g	c	t	g	c	a	t	t	a	a	g	g	a	a	t	t	c	g	t	a	a	g	t	a	c
KP771924	5	t	t	t	t	a	t	t	t	t	t	t	a	g	g	t	c	a	t	t	t	a	a	t	t	g	t	c	g	g	a	a	c	a	g	c	c	t	c	g	g	g	t	g	t	t	g	t	g	g	t	a	c	t	t	g	c	a	a	t	a	g	g	g	c	t	g	c	a	t	t	a	a	g	g	a	a	t	t	c	g	t	a	a	g	t	a	c
KM355696	5	t	t	t	t	a	t	t	t	t	t	t	a	g	g	t	c	a	t	t	t	a	a	t	t	g	t	c	g	g	a	a	c	a	g	c	c	t	c	g	g	g	t	g	t	t	g	c	g	g	t	a	c	t	t	g	c	a	a	t	a	g	g	g	c	t	g	c	a	t	t	a	a	g	g	a	a	t	t	c	g	t	a	a	g	t	a	c
KM355697	5	t	t	t	t	a	t	t	t	t	t	t	a	g	g	t	c	a	t	t	t	a	a	t	t	g	t	c	g	g	a	a	c	a	g	c	c	t	c	g	g	g	t	g	t	t	g	c	g	g	t	a	c	t	t	g	c	a	a	t	a	g	g	g	c	t	g	c	a	t	t	a	a	g	g	a	a	t	t	c	g	t	a	a	g	t	a	c
KM355698	5	t	t	t	t	a	t	t	t	t	t	t	a	g	g	t	c	a	t	t	t	a	a	t	t	g	t	c	g	g	a	a	c	a	g	c	c	t	c	g	g	g	t	g	t	t	g	t	g	g	t	a	c	t	t	g	c	a	a	t	a	g	g	g	c	t	g	c	a	t	t	a	a	g	g	a	a	t	t	c	g	t	a	a	g	t	a	c
KM355699	5	t	t	t	t	a	t	t	t	t	t	t	a	g	g	t	c	a	t	t	t	a	a	t	t	g	t	c	g	g	a	a	c	a	g	c	c	t	c	g	g	g	t	g	t	t	g	t	g	g	t	a	c	t	t	g	c	a	a	t	a	g	g	g	c	t	g	c	a	t	t	a	a	g	g	a	a	t	t	c	g	t	a	a	g	t	a	c
KM355701	5	t	t	t	t	a	t	t	t	t	t	t	a	g	g	t	c	a	t	t	t	a	a	t	t	g	t	c	g	g	a	a	c	a	g	c	c	t	c	g	g	g	t	g	t	t	g	c	g	g	t	a	c	t	t	g	c	a	a	t	a	g	g	g	c	t	g	c	a	t	t	a	a	g	g	a	a	t	t	c	g	t	a	a	g	t	a	c
KM355702	5	t	t	t	t	a	t	t	t	t	t	t	a	g	g	t	c	a	t	t	t	a	a	t	t	g	t	c	g	g	a	a	c	a	g	c	c	t	c	g	g	g	t	g	t	t	g	t	g	g	t	a	c	t	t	g	c	a	a	t	a	g	g	g	c	t	g	c	a	t	t	a	a	g	g	a	a	t	t	c	g	t	a	a	g	t	a	c
KM355703	5	t	t	t	t	a	t	t	t	t	t	t	a	g	g	t	c	a	t	t	t	a	a	t	t	g	t	c	g	g	a	a	c	a	g	c	c	t	c	g	g	g	t	g	t	t	g	c	g	g	t	a	c	t	t	g	c	a	a	t	a	g	g	g	c	t	g	c	a	t	t	a	a	g	g	a	a	t	t	c	g	t	a	a	g	t	a	c
KM355704	5	t	t	t	t	a	t	t	t	t	t	t	a	g	g	t	c	a	t	t	t	a	a	t	t	g	t	c	g	g	a	a	c	a	g	c	c	t	c	g	g	g	t	g	t	t	g	c	g	g	t	a	c	t	t	g	c	a	a	t	a	g	g	g	c	t	g	c	a	t	t	a	a	g	g	a	a	t	t	c	g	t	a	a	g	t	a	c
KM355705	5	t	t	t	t	a	t	t	t	t	t	t	a	g	g	t	c	a	t	t	t	a	a	t	t	g	t	c	g	g	a	a	c	a	g	c	c	t	c	g	g	g	t	g	t	t	g	t	g	g	t	a	c	t	t	g	c	a	a	t	a	g	g	g	c	t	g	c	a	t	t	a	a	g	g	a	a	t	t	c	g	t	a	a	g	t	a	c
KM355706	5	t	t	t	t	a	t	t	t	t	t	t	a	g	g	t	c	a	t	t	t	a	a	t	t	g	t	c	g	g	a	a	c	a	g	c	c	t	c	g	g	g	t	g	t	t	g	t	g	g	t	a	c	t	t	g	c	a	a	t	a	g	g	g	c	t	g	c	a	t	t	a	a	g	g	a	a	t	t	c	g	t	a	a	g	t	a	c
KM355707	5	t	t	t	t	a	t	t	t	t	t	t	a	g	g	t	c	a	t	t	t	a	a	t	t	g	t	c	g	g	a	a	c	a	g	c	c	t	c	g	g	g	t	g	t	t	g	c	g	g	t	a	c	t	t	g	c	a	a	t	a	g	g	g	c	t	g	c	a	t	t	a	a	g	g	a	a	t	t	c	g	t	a	a	g	t	a	c
KM355708	5	t	t	t	t	a	t	t	t	t	t	t	a	g	g	t	c	a	t	t	t	a	a	t	t	g	t	c	g	g	a	a	c	a	g	c	c	t	c	g	g	g	t	g	t	t	g	c	g	g	t	a	c	t	t	g	c	a	a	t	a	g	g	g	c	t	g	c	a	t	t	a	a	g	g	a	a	t	t	c	g	t	a	a	g	t	a	c
KM355709	5	t	t	t	t	a	t	t	t	t	t	t	a	g	g	t	c	a	t	t	t	a	a	t	t	g	t	c	g	g	a	a	c	a	g	c	c	t	c	g	g	g	t	g	t	t	g	t	g	g	t	a	c	t	t	g	c	a	a	t	a	g	g	g	c	t	g	c	a	t	t	a	a	g	g	a	a	t	t	c	g	t	a	a	g	t	a	c
KM355711	5	t	t	t	t	a	t	t	t	t	t	t	a	g	g	t	c	a	t	t	t	a	a	t	t	g	t	c	g	g	a	a	c	a	g	c	c	t	c	g	g	g	t	g	t	t	g	t	g	g	t	a	c	t	t	g	c	a	a	t	a	g	g	g	c	t	g	c	a	t	t	a	a	g	g	a	a	t	t	c	g	t	a	a	g	t	a	c
KM355712	5	t	t	t	t	a	t	t	t	t	t	t	a	g	g	t	c	a	t	t	t	a	a	t	t	g	t	c	g	g	a	a	c	a	g	c	c	t	c	g	g	g	t	g	t	t	g	t	g	g	t	a	c	t	t	g	c	a	a	t	a	g	g	g	c	t	g	c	a	t	t	a	a	g	g	a	a	t	t	c	g	t	a	a	g	t	a	c
KY037797	5	[illegible]	[illegible]	[illegible]	[illegible]	[illegible]	[illegible]	[illegible]	[illegible]	[illegible]	[illegible]	[illegible]	[illegible]	[illegible]	[illegible]	[illegible]	[illegible]	[illegible]	[illegible]	[illegible]	[illegible]	[illegible]	[illegible]	[illegible]	[illegible]	[illegible]	[illegible]	[illegible]	[illegible]	[illegible]	[illegible]	[illegible]	[illegible]	[illegible]	[illegible]	[illegible]	[illegible]	[illegible]	[illegible]	[illegible]	[illegible]	[illegible]	[illegible]	[illegible]	[illegible]	[illegible]	[illegible]	[illegible]	[illegible]	[illegible]	[illegible]	[illegible]	[illegible]	[illegible]	[illegible]	[illegible]	[illegible]	[illegible]	[illegible]	[illegible]	[illegible]	[illegible]	[illegible]	[illegible]	[illegible]	[illegible]	[illegible]	[illegible]	[illegible]	[illegible]	[illegible]	[illegible]	[illegible]	[illegible]	[illegible]	[illegible]	[illegible]	[illegible]	[illegible]	[illegible]	[illegible]	[illegible]	[illegible]	[illegible]	[illegible]	[illegible]	[illegible]	[illegible]

Supplementary Table 3: 2880 SN

GB-ID	Clade markers																																																																																						
	Position in Dumas	2321	2420	2423	2459	2493	2503	2504	2515	2547	2591	2596	2614	2642	2643	2676	2701	2742	2743	2778	2783	2788	2807	2942	2946	3059	3182	3188	3218	3223	3272	3317	3392	3455	3487	3505	3538	3547	3556	3569	3580	3584	3605	3619	3679	3728	3746	3760	3764	3989	4183	4222	4228	4258	4344	4402	4423	4440	4441	4446	4511	4521	4535	4601	4644	4645	4707	4735	4793	4801	4858	4890	4898	4914	4921	4993	5170	5270	5342	5421	5460	5574	5583	5596	5602	5656	5745	5767
MH499467	5	t	t	t	t	a	t	t	t	t	g	t	a	g	g	t	c	a	t	t	t	a	a	t	t	g	t	c	g	g	a	a	c	a	g	c	c	t	c	g	g	g	t	g	t	t	g	t	g	g	t	a	c	t	t	g	c	a	a	t	a	g	g	g	c	t	g	c	a	t	t	a	a	g	g	a	a	t	t	c	g	t	a	a	g	t	a	c
MH499469	5	t	t	t	t	a	t	t	t	t	g	t	a	g	g	t	c	a	t	t	t	a	a	t	t	g	t	c	g	g	a	a	c	a	g	c	c	t	c	g	g	g	t	g	t	t	g	t	g	g	t	a	c	t	t	g	t	a	a	t	a	g	g	g	c	t	g	c	a	t	t	a	a	g	g	a	a	t	t	c	g	t	a	a	g	t	a	t
MH709321	5	t	t	t	t	a	t	t	t	t	t	t	a	g	g	t	c	a	t	t	t	a	a	t	t	g	t	c	g	g	a	a	c	a	g	c	c	t	c	g	g	g	t	g	t	t	g	t	g	g	t	a	c	t	t	g	c	a	a	t	a	g	g	g	c	t	g	c	c	t	t	a	a	g	g	a	a	t	t	c	g	t	a	a	g	t	a	c
MH709324	5	t	t	t	t	a	t	t	t	t	g	t	a	g	g	t	c	a	t	t	t	a	a	t	t	g	t	c	g	g	a	a	c	a	g	c	c	t	c	g	g	g	t	g	t	t	g	t	g	g	t	a	c	t	t	g	c	a	a	t	a	g	g	g	c	t	g	c	a	t	t	a	a	g	g	a	a	t	t	c	g	t	a	a	g	t	a	c
MH709325	5	t	t	t	t	a	t	t	t	t	t	t	a	g	g	t	c	a	t	t	t	a	a	t	t	g	t	c	g	g	a	a	c	a	g	c	c	t	c	g	g	a	t	g	t	t	g	t	g	g	t	a	c	t	t	g	c	a	a	t	a	g	g	g	c	t	g	c	a	t	t	a	a	g	g	a	a	t	t	c	g	t	a	a	g	t	a	c
MH709327	5	t	t	t	t	a	t	t	t	t	g	t	a	g	g	t	c	a	t	t	t	a	a	t	t	g	t	c	g	g	a	a	c	a	g	c	c	t	c	g	g	g	t	g	t	t	g	t	g	g	t	a	c	t	t	g	c	a	a	t	a	g	g	g	c	t	g	c	a	t	t	a	a	g	g	a	a	t	t	c	g	t	a	a	g	t	a	c
MH709328	5	t	t	t	t	a	t	t	t	t	g	t	a	g	g	t	c	a	t	t	t	a	a	t	t	g	t	c	g	g	a	a	c	a	g	c	c	t	c	g	g	g	t	g	t	t	g	t	g	g	t	a	c	t	t	g	c	a	a	t	a	g	g	g	c	t	g	c	a	t	t	a	a	g	g	a	a	t	t	c	g	t	a	a	g	t	a	c
MH709330	5	t	t	t	t	a	t	t	t	t	g	t	a	g	g	t	c	a	t	t	t	a	a	t	t	g	t	c	g	g	a	a	c	a	g	c	c	t	c	g	g	g	t	g	t	t	g	t	g	g	t	a	c	t	t	g	c	a	a	t	a	g	g	g	c	t	g	c	a	t	t	a	a	g	g	a	a	t	t	c	g	t	a	a	g	t	a	c
MH709332	5	t	t	t	t	a	t	t	t	t	g	t	a	g	g	t	c	a	t	t	t	a	a	t	t	g	t	c	g	g	a	a	c	a	g	c	c	t	c	g	g	g	t	g	t	t	g	t	g	g	t	a	c	t	t	g	c	a	a	t	a	g	g	g	c	t	g	c	a	t	t	a	a	g	g	a	a	t	t	c	g	t	a	a	g	t	a	c
MH709334	5	t	t	t	t	a	t	t	t	t	g	t	a	g	g	t	c	a	t	t	t	a	a	t	t	g	t	c	g	g	a	a	c	a	g	c	c	t	c	g	g	g	t	g	t	t	g	t	g	g	t	a	c	t	t	g	c	a	a	t	a	g	g	g	c	t	g	c	a	t	t	a	a	g	g	a	a	t	t	c	g	t	a	a	g	t	a	c
MH709335	5	t	t	t	t	a	t	t	t	t	g	t	a	g	g	t	c	a	t	t	t	a	a	t	t	g	t	c	g	g	a	a	c	a	g	c	c	t	c	g	g	g	t	g	t	t	g	t	g	g	t	a	c	t	t	g	c	a	a	t	a	g	g	g	c	t	g	c	a	t	t	a	a	g	g	a	a	t	t	c	g	t	a	a	g	t	a	c
MH709336	5	t	t	t	t	a	t	t	t	t	g	t	a	g	g	t	c	a	t	t	t	a	a	t	t	g	t	c	g	g	a	a	c	a	g	c	c	t	c	g	g	g	t	g	t	t	g	t	g	g	t	a	c	t	t	g	c	a	a	t	a	g	g	g	c	t	g	c	a	t	t	a	a	g	g	a	a	t	t	c	g	t	a	a	g	t	a	c
MH709338	5	t	t	t	t	a	t	t	t	t	g	t	a	g	g	t	c	a	t	t	t	a	a	t	t	g	t	c	g	g	a	a	c	a	g	c	c	t	c	g	g	g	t	g	t	t	g	t	g	g	t	a	c	t	t	g	c	a	a	t	a	g	g	g	c	t	g	c	a	t	t	a	a	g	g	a	a	t	t	c	g	t	a	a	g	t	a	c
MH709340	5	t	t	t	t	a	t	t	t	t	t	t	a	g	g	t	c	a	t	t	t	a	a	t	t	g	t	c	g	g	a	a	c	a	g	c	c	t	c	g	g	g	t	g	t	t	g	t	g	g	t	a	c	t	t	g	c	a	a	t	a	g	g	g	c	t	g	c	a	t	t	a	a	g	g	a	a	t	t	c	g	t	a	a	g	t	a	c
MH709342	5	t	t	t	t	a	t	t	t	t	t	t	a	g	g	t	c	a	t	t	t	a	a	t	t	g	t	c	g	g	a	a	c	a	g	c	c	t	c	g	g	g	t	g	t	t	g	t	g	g	t	a	c	t	t	g	c	a	a	t	a	g	g	g	c	t	g	c	a	t	t	a	a	g	g	a	a	t	t	c	g	t	a	a	g	t	a	c
MH709343	5	t	t	t	t	a	t	t	t	t	g	t	a	g	g	t	c	a	t	t	t	a	a	t	t	g	t	c	g	g	a	a	c	a	g	c	c	t	c	g	g	g	t	g	t	t	g	t	g	g	t	a	c	t	t	g	c	a	a	t	a	g	g	g	c	t	g	c	a	t	t	a	a	g	g	a	a	t	t	c	g	t	a	a	g	t	a	c
MH709345	5	t	t	t	t	a	t	t	t	t	g	t	a	g	g	t	c	a	t	t	t	a	a	t	t	g	t	c	g	g	a	a	c	a	g	c	c	t	c	g	g	g	t	g	t	t	g	t	g	g	t	a	c	t	t	g	c	a	a	t	a	g	g	g	c	t	g	c	a	t	t	a	a	g	g	a	a	t	t	c	g	t	a	a	g	t	a	c
MH709347	5	t	t	t	t	a	t	t	t	t	g	t	a	g	g	t	c	a	t	t	t	a	a	t	t	g	t	c	g	g	a	a	c	a	g	c	c	t	c	g	g	g	t	g	t	t	g	t	g	g	t	a	c	t	t	g	c	a	a	t	a	g	g	g	c	t	g	c	a	t	t	a	a	g	g	a	a	t	t	c	g	t	a	a	g	t	a	c
MH709349	5	t	t	t	t	a	t	t	t	t	g	t	a	g	g	t	c	a	t	t	t	a	a	t	t	g	t	c	g	g	a	a	c	a	g	c	c	t	c	g	g	g	t	g	t	t	g	t	g	g	t	a	c	t	t	g	c	a	a	t	a	g	g	g	c	t	g	c	a	t	t	a	a	g	g	a	a	t	t	c	g	t	a	a	g	t	a	c
MH709350	5	t	t	t	t	a	t	t	t	t	g	t	a	g	g	t	c	a	t	t	t	a	a	t	t	g	t	c	g	g	a	a	c	a	g	c	c	t	c	g	g	g	t	g	t	t	g	t	g	g	t	a	c	t	t	g	c	a	a	t	a	g	g	g	c	t	g	c	a	t	t	a	a	g	g	a	a	t	t	c	g	t	a	a	g	t	a	c
MH709351	5	t	t	t	t	a	t	t	t	t	t	t	a	g	g	t	c	a	t	t	t	a	a	t	t	g	t	c	g	g	a	a	c	a	g	c	c	t	c	g	g	g	t	g	t	t	g	t	g	g	t	a	c	t	t	g	c	a	a	c	a	g	g	g	c	t	g	c	a	t	t	a	a	g	g	a	a	t	t	c	g	t	a	a	g	t	a	c
MH709353	5	t	t	t	t	a	t	t	t	t	g	t	a	g	g	t	c	a	t	t	t	a	a	t	t	g	t	c	g	g	a	a	c	a	g	c	c	t	c	g	g	g	t	g	t	t	g	t	g	g	t	a	c	t	t	g	c	a	a	t	a	g	g	g	c	t	g	c	a	t	t	a	a	g	g	a	a	t	t	c	g	t	a	a	g	t	a	c
MH709358	5	t	t	t	t	a	t	t	t	t	t	t	a	g	g	t	c	a	t	t	t	a	a	t	t	g	t	c	g	g	a	a	c	a	g	c	c	t	c	g	g	g	t	g	t	t	g	t	g	g	t	a	c	t	t	g	c	a	a	t	a	g	g	g	c	t	g	c	a	t	t	a	a	g	g	a	a	t	t	c	g	t	a	a	g	t	a	c
MH709360	5	t	t	t	t	a	t	t	t	t	g	t	a	g	g	t	c	a	t	t	t	a	a	t	t	g	t	c	g	g	a	a	c	a	g	c	c	t	c	g	g	g	t	g	t	t	g	t	g	g	t	a	c	t	t	g	c	a	a	t	a	g	g	g	c	t	g	c	a	t	t	a	a	g	g	a	a	t	t	c	g	t	a	a	g	t	a	c
MH709362	5	t	t	t	t	a	t	t	t	t	g	t	a	g	g	t	c	a	t	t	t	a	a	t	t	g	t	c	g	g	a	a	c	a	g	c	c	t	c	g	g	g	t	g	t	t	g	t	g	g	t	a	c	t	t	g	c	a	a	t	a	g	g	g	c	t	g	c	a	t	t	a	a	g	g	a	a	t	t	c	g	t	a	a	g	t	a	c
MH709365	5	t	t	t	t	a	t	t	t	t	g	t	a	g	g	t	c	a	t	t	t	a	a	t	t	g	t	c	g	g	a	a	c	a	g	c	c	t	c	g	g	g	t	g	t	t	g	t	g	g	t	a	c	t	t	g	c	a	a	t	a	g	g	g	c	t	g	c	a	t	t	a	a	g	g	a	a	t	t	c	g	t	a	a	g	t	a	c
MH709373	5	t	t	t	t	a	t	t	t	t	g	t	a	g	g	t	c	a	t	t	t	a	a	t	t	g	t	c	g	g	a	a	c	a	g	c	c	t	t	g	g	g	t	g	t	t	g	t	g	g	t	a	c	t	t	g	c	a	a	t	a	g	g	g	c	t	g	c	a	t	t	a	a	g	g	a	a	t	t	c	g	t	a	a	g	t	a	c
MH709374	5	t	t	t	t	a	t	t	t	t	g	t	a	g	g	t	c	a	t	t	t	a	a	t	t	g	t	c	g	g	a	a	c	a	g	c	c	t	c	g	g	g	t	g	t	t	g	t	g	g	t	a	c	t	t	g	c	a	a	t	a	g	g	g	c	t	g	c	a	t	t	a	a	g	g	a	a	t	t	c	g	t	a	a	g	t	a	c
KC112914	6	t	t	t	t	a	t	t	t	t	t	c	a	g	a	t	c	a	t	t	t	a	a	t	t	g	t	c	g	g	a	a	c	a	g	c	c	t	c	g	g	g	t	g	t	t	g	t	a	g	t	a	c	t	t	g	c	a	a	t	a	g	g	g	t	t	g	c	a	t	t	a	a	g	g	a	a	t	t	c	g	t	a	a	g	t	a	c
KP771914	6	t	t	t	t	a	t	t	t	t	t	c	a	g	a	t	c	a	t	t	t	a	a	t	t	g	t	c	g	g	a	a	c	a	g	c	c	t	c	g	g	g	t	g	t	t	g	t	a	g	t	a	c	t	t	g	c	a	a	t	a	g	g	g	t	t	g	c	a	t	t	a	a	g	g	a	a	t	t	c	g	t	a	a	a	t	a	c
KP702725	6	t	t	t	t	a	t	t	t	t	t	c	a	g	a	t	c	a	t	t	t	a	a	t	t	g	t	c	g	g	a	a	c	a	g	c	c	t	c	g	g	g	t	g	t	t	g	t	a	g	t	a	c	t	t	g	c	a	a	t	a	g	g	g	t	t	g	c	a	t	t	a	a	g	g	a	a	t	t	c	g	t	a	a	g	t	a	c
KP771890	9	t	t	t	t	a	t	t	t	t	t	c	a	g	a	t	c	a	t	t	t	a	a	t	t	g	t	c	g	g	a	a	c	a	g	c	c	t	c	g	g	g	t	g	t	t	g	t	g	g	t	a	c	t	t	g	c	a	a	t	a	g	g	g	c	t	g	c	a	t	t	a	a	g	a	a	a	t	t	c	g	t	a	a	g	t	a	c
JN704710	9	t	t	t	t	a	t	t	t	t	t	c	a	g	a	t	c	a	t	t	t	a	a	t	t	g	t	c	g	g	a	a	c	a	g	c	c	t	c	g	g	g	t	g	t	t	g	t	g	g	t	a	c	t	t	g	c	a	a	t	a	g	g	g	c	t	g	c	a	t	t	a	a	g	g	a	a	t	t	c	g	t	a	a	g	t	a	c
KY037798	9	t	t	t	t	a	t	t	t	t	t	c	a	g	a	t	c	a	g	t	t	a	a	t	t	g	t	c	g	g	a	a	c	a	g	c	c	t	c	g	g	g	t	g	t	t	g	t	g	g	t	a	c	t	t	g	c	a	a	t	a	g	g	g	c	t	g	c	a	t	t	a	a	g	g	a	a	t	t	c	g	t	a	a	g	t	a	c
MH709312	9	t	t	t	t	a	t	t	t	t	t	c	a	g	a	t	c	a	g	t	t	a	a	t	t	g	t	c	g	g	a	a	c	a	g	c	c	t	c	g	g	g	t	g	t	t	g	t	g	g	t	a	c	t	t	g	c	a	a	t	a	g	g	g	c	t	g	c	a	t	t	a	a	g	g	a	a	t	t	c	g	t	a	a	g	t	a	c
MH709352	9	t	t	t	t	a	t	t	t	t	t	c	a	g	a	t	c	a	g	t	t	a	a	t	t	g	t	c	g	g	a	a	c	a	g	c	c	t	c	g	g	g	t	g	t	t	g	t	g	g	t	a	c	t	t	g	c	a	a	t	a	g	g	g	c	t	g	c	a	t	t	a	a	g	g	a	a	t	t	c	g	t	a	a	g	t	a	c
AB097933	Vac	t	t	t	t	a	t	t	t	t	t	t	a	g	g	t	c	a	t	t	t	a	a	t	t	g	t	c	g	g	a	a	c	a	g	c	c	t	c	g	g	g	t	g	t	t	g	t	g	g	t	a	c	t	t	g	c	a	a	t	a	g	g	g	c	t	g	c	a	t	t	a	a	g	g	a	a	t	t	c	g	t	a	a	g	t	a	c
JN704698	Vac	t	t	t	t	a	t	t	t	t	t	t	a	g	g	t	c	a	t	t	t	a	a	t	t	g	t	c	g	g	a	a	c	a	g	c	c	t	c	g	g	g	t	g	t	t	g	t	g	g	t	a	c	t	t	g	c	a	a	t	a	g	g	g	c	t	g	c	a	t	t	a	a	g	g	a	a	t	t	c	g	t	a	a	g	t	a	c
JN704697	Vac	t	t	t	t	a	t	t	c	t	t	t	a	g	g	t	c	a	t	t	t	a	a	t	t	g	t	c	g	g	a	a	c	a	g	c	c	t	c	g	g	g	t	g	t	t	g	t	g	g	t	a	c	t	t	g	c	a	a	t	a	g	g	g	c	t	g	c	a	t	t	a	a	g	g	a	a	t	t	c	g	t	a	a	g	t	a	c
DQ008355	Vac	t	t	t	t	a	t	t	t	t	t	t	a	g	g	t	c	a	t	t	t	a	a	t	t	g	t	c	g	g	a	a	c	a	g	c	c	t	c	g	g	g	t	g	t	t	g	t	g	g	t	a	c	t	t	g	c	a	a	t	a	g	g	g	c	t	g	c	a	t	t	a	a	g	g	a	a	t	t	c	g	t	a	a	g	t	r	c
JF306641	Vac	t	t	t	t	a	t	t	c	t	t	t	a	g	g	t	c	a	t	t	t	a	a	t	t	g	t	c	g	g	a	a	c	a	g	c	c	t	c	g	g	g	t	g	t	t	g	t	g	g	t	a	c	t	t	g	c	a	a	t	a	g	g	g	c	t	g	c	a	t	t	a	a	g	g	a	a	t	t	c	g	t	a	a	g	t	g	c
AB097932	Vac	t	t	t	t	a	t	t	y	t	t	t	a	g	g	t	c	a	t	t	t	a	a	t	t	g	t	c	g	g	a	a	c	a	g	c	c	t	c	g	g	g	t	g	t	t	g	t	g	g	t	a	c	t	t	g	c	a	a	t	a	g	g	g	c	t	g	c	a	t	t	a	a	g	g	a	a	t	t	c	g	t	a	a	g	t	g	c
DQ008354	Vac	t	t	t	t	a	t	t	c	t	t	t	a	g	g	t	c	a	t	t	t	a	a	t	t	g	t	c	g	g	a	a	c	a	g	c	c	t	c	g	g	g	t	g	t	t	g	t	g	g	t	a	c	t	t	g	c	a	a	t	a	g	g	g	c	t	g	c	a	t	t	a	a	g	g	a	a	t	t	c	g	t	a	a	g	t	g	c
KU926312	Vac	t	t	t	t	a	t	t	c	t	t	t	a	g	g	t	c	a	t	t	t	a	a	t	t	g	t	c	g	g	a	a	c	a	g	c	c	t	c	g	g	g	t	g	t	t	g	t	g	g	t	a	c	t	t	g	c	a	a	t	a	g	g	g	c	t	g	c	a	t	t	a	a	g	g	a	a	t	t	c	g	t	a	a	g	t	g	c
KU926313	Vac	t	t	t	t	a	t	t	c	t	t	t	a	g	g	t	c	a	t	t	t	a	a	t	t	g	t	c	g	g	a	a	c	a	g	c	c	t	c	g	g	g	t	g	t	t	g	t	g	g	t	a	c	t	t	g	c	a	a	t	a	g	g	g	c	t	g	c	a	t	t	a	a	g	g	a	a	t	t	c	g	t	a	a	g	t	g	c
KU926314	Vac	t	t	t	t	a	t	t	t	t	t	t	a	g	g	t	c	a	t	t	t	a	a	t	t	g	t	c	g	g	a	a	c	a	g	c	c	t	c	g	g	g	t	g	t	t	g	t	g	g	t	a	c	t	t	g	c	a	a	t	a	g	g	g	c	t	g	c	a	t	t	a	a	g	g	a	a	t	t	c	g	t	a	a	g	t	a	c
KF558371	Vac	t	t	t	t	a	t	t	t	t	t	t	a	g	g	t	c	a	t	t	t	a	a	t	t	g	t	c	g	g	a	a	c	a	g	c	c	t	c	g	g	g	t	g	t	t	g	t	g	g	t	a	c	t	t	g	c	a	a	t	a	g	g	g	c	t	g	c	a	t	t	a	a	g	g	a	a	t	t	c	g	t	a	a	g	t	a	c
KF558372	Vac	t	t	t	t	a	t	t	t	t	t	t	a	g	g	t	c	a	t	t	t	a	a	t	t	g	t	c	g	g	a	a	c	a	g	c	c	t	c	g	g	g	t	g	t	t	g	t	g	g	t	a	c	t	t	g	c	a	a	t	a	g	g	g	c	t	g	c	a	t	t	a	a	g	g	a	a	t	t	c	g	t	a	a	g	t	a	c
KF558373	Vac	t	t	t	t	a	t	t	t	t	t	t	a	g	g	t	c	a	t	t	t	a	a	t	t	g	t	c	g	g	a	a	c	a	g	c	c	t	c	g	g	g	t	g	t	t	g	t	g	g	t	a	c	t	t	g	c	a	a	t	a	g	g	g	c	t	g	c	a	t	t	a	a	g	g	a	a	t	t	c	g	t	a	a	g	t	a	c
KF558374	Vac	t	t	t	t	a	t	t	t	t	t	t	a	g	g	t	c	a	t	t	t	a	a	t	t	g	t	c	g	g	a	a	c	a	g	c	c	c	c	g	g	g	t	g	t	t	g	t	g	g	t	a	c	t	t	g	c	a	a	t	a	g	g	g	c	t	g	c	a	t	t	a	a	g	g	a	a	t	t	c	g	t	a	a	g	t	g	c
KF558375	Vac	t	t	t	t	a	c	t	t	t	t	t	a	g	g	t	c	a	t	t	t	a	a	t	t	g	t	c	g	g	a	a	c	a	g	c	c	t	c	g	g	g	t	g	t	t	g	t	g	g	t	a	c	t	t	g	c	a	a	t	a	g	g	g	c	t	g	c	a	t	t	a	a	g	g	a	a	t	t	c	g	t	a	a	g	t	g	c
KF558376	Vac	t	t	t	t	g	t	t	t	t	t	t	a	g	g	t	c	a	t	t	t	a	a	t	t	g	t	c	g	g	a	a	c	a	g	c	c	t	c	g	g	g	t	g	t	t	g	t	g	g	t	a	c	t	t	g	c	a	a	t	a	g	g	g	c	t	g	c	a	t	t	a	a	g	g	a	a	t	t	c	g	t	a	a	g	t	g	c
KF558377	Vac	t	t	t	t	a	t	t	c	t	t	t	a	g	g	t	c	a	t	t	t	a	a	t	t	g	t	c	g	g	a	a	c	a	g	c	c	t	c	g	g	g	t	g	t	t	g	t	g	g	t	a	c	t	t	g	c	a	a	t	a	g	g	g	c	t	g	c	a	t	t	a	a	g	g	a	a	t	t	c	g	t	a	a	g	t	a	c
KF558378	Vac	t	t	t	t	a	t	t	c	t	t	t	a	g	g	t	c	a	t	t	t	a	a	t	t	g	t	c	g	g	a	a	c	a	g	c	c	t	c	g	g	g	t	g	t	t	g	t	g	g	t	a	c	t	t	g	c	a	a	t	a	g	g	g	c	t	g	c	a	t	t	a	a	g	g	a	a	t	t	c	g	t	a	a	g	t	a	c
KF558379	Vac	t	t	t	t	a	t	t	t	t	t	t	a	g	g	t	c	a	t	t	t	a	a	t	t	g	t	c	g	g	a	a	c	a	g	c	c	t	c	g	g	g	t	g	t	t	g	t	g	g	t	a	c	t	t	g	c	a	a	t	a	g	g	g	c	t	g	c	a	t	t	a	a	g	g	a	a	t	t	c	g	t	a	a	g	t	g	c
KF558380	Vac	t	t	t	t	a	t	t	t	t	t	t	a	g	g	t	c	a	t	t	t	a	a	t	t	g	t	c	g	g	a	a	c	a	g	c	c	t	c	g	g	g	t	g	t	t	g	t	g	g	t	a	c	t	t	g	c	a	a	t	a	g	g	g	c	t	g	c	a	t	t	a	a	g	g	a	a	t	t	c	g	t	a	a	g	t	g	c
KF558381	Vac	t	t	t	t	a	t	t	c	t	t	t	a	g	g	t	c	a	t	t	t	a	a	t	t	g	t	c	g	g	a	a	c	a	g	c	c	t	c	g	g	g	t	g	t	t	g	t	g	g	t	a	c	t	t	g	c	a	a	t	a	g	g	g	c	t	g	c	a	t	t	a	a	g	g	a	a	t	t	c	g	t	a	a	g	t	a	c
KF558382	Vac	t	t	t	t	a	t	t	c	t	t	t	a	g	g	t	c	a	t	t	g	a	a	t	t	g	t	c	g	g	a	a	c	a	g	c	c	t	c	g	g	g	t	g	t	t	g	t	g	g	t	a	c	t	t	g	c	a	a	t	a	g	g	g	c	t	g	c	a	t	t	a	a	g	g	a	a	t	t	c	g	t	a	a	g	t	a	c
KF558383	Vac	t	t	t	t	a	t	t	t	t	t	t	a	g	g	t	c	a	t	t	t	a	a	t	t	g	t	c	g	g	a	a	c	a	g	c	c	t	c	g	g	g	t	g	t	t	g	t	g	g	t	a	c	t	t	g	c	a	a	t	a	g	g	g	c	t	g	c	a	t	t	a	a	g	g	a	a	t	t	c	g	t	a	a	g	t	a	c
KF558384	Vac	t	t	t	t	a	t	t	t	t	t	t	a	g	g	t	c	a	t	t	t	a	a	t	t	g	t	c	g	g	a	a	c	a	g	c	c	t	c	g	g	g	t	g	t	t	g	t	g	g	t	a	c	t	t	g	c	a	a	t	a	g	g	g	c	t	g	c	a	t	t	a	a	g	g	a	a	t	t	c	g	t	a	a	g	t	a	c
KF558385	Vac	t	t	t	t	a	t	t	t	t	t	t	a	g	g	t	c	a	t	t	t	a	a	t	t	g	t	c	g	g	a	a	c	a	g	c	c	t	c	g	g	g	t	g	t	t	g	t	g	g	t	a	c	t	t	g	c	a	a	t	a	g	g	g	c	t	g	c	a	t	t	a	a	g	g	a	a	t	t	c	g	t	a	a	g	t	a	c
KF558386	Vac	t	t	t	t	a	t	t	t	t	t	t	a	g	g	t	c	a	t	t	t	a	a	t	t	g	t	c	g	g	a	a	c	a	g	c	c	t	c	g	g	g	t	g	t	t	g	t	g	g	t	a	c	t	t	g	c	a	a	t	a	g	g	g	c	t	g	c	a	t	t	a	a	g	g	a	a	t	t	c	g	t	a	a	g	t	a	c
KF558387	Vac	t	t	t	t	a	t	t	c	t	t	t	a	g	g	t	c	a	t	t	t	a	a	t	t	g	t	c	g	g	a	a	c	a	g	c	c	t	c	g	g	g	t	g	t	t	g	t	g	g	t	a	c	t	t	g	c	a	a	t	a	g	g	g	c	t	g	c	a	t	t	a	a	g	g	a	a	t	t	c	g	t	a	a	g	t	g	c
KF558388	Vac	t	t	t	t	a	t	t	t	t	t	t	a	g	g	t	c	a	t	t	t	a	a	t	t	g	t	c	g	g	a	a	c	a	g	c	c	t	c	g	g	g	t	g	t	t	g	t	g	g	t	a	c	t	t	g	c	a	a	t	a	g	g	g	c	t	g	c	a	t	t	a	a	g	g	a	a	t	t	c	g	t	a	a	g	t	a	c
KF558389	Vac	t	t	t	t	a	t	t	t	t	t	t	a	g	g	t	c	a	t	t	t	a	a	t	t	g	t	c	g	g	a	a	c	a	g	c	c	t	c	g	g	g	t	g	t	t	g	t	g	g	t	a	c	t	t	g	c	a	a	t	a	g	g	g	c	t	g	c	a	t	t	a	a	g	g	a	a	t	t	c	g	t	a	a	g	t	g	c
KF558390	Vac	t	t	t	t	a	c	t	t	t	t	t	a	g	g	t	c	a	t	t	t	a	a	t	t	g	t	c	g	g	a	a	c	a	g	c	c	t	c	g	g	g	t	g	t	t	g	t	g	g	t	a	c	t	t	g	c	a	a	t	a	g	g	g	c	t	g	c	a	t	t	a	a	g	g	a	a	t	t	c	g	t	a	a	g	t	g	c
KF558391	Vac	t	t	t	t	a	t	t	t	t	t	t	a	g	g	t	c	a	t	t	t	a	a	t	t	g	t	c	g	g	a	a	c	a	g	c	c	t	c	g	g	g	t	g	t	t	g	t	g	g	t	a	c	t	t	g	c	a	a	t	a	g	g	g	c	t	g	c	a	t	t	a	a	g	g	a	a	t	t	c	g	t	a	a	g	t	a	c
KF811485	Vac	t	t	t	t	a	t	t	t	t	t	t	a	g	g	t	c	a	t	t	t	a	a	t	t	g	t	c	g	g	a	a	c	a	g	c	c	t	c	g	g	g	t	g	t	t	g	t	g	g	t	a	c	t	t	g	c	a	a	t	a	g	g	g	c	t	g	c	a	t	t	a	a	g	g	a	a	t	t	c	g	t	a	a	g	t	a	c
KF853225	Vac	t	t	t	t	a	t	t	t	t	t	t	a	g	g	t	c	a	t	t	t	a	a	t	t	g	t	c	g	g	a	a	c	a	g	c	c	t	c	g	g	g	t	g	t	t	g	t	g	g	t	a	c	t	t	g	c	a	a	t	a	g	g	g	c	t	g	c	a	t	t	a	a	g	g	a	a	t	t	c	g	t	a	a	g	t	a	c
KF853226	Vac	t	t	t	t	a	t	t	t	t	t	t	a	g	g	t	c	a	t	t	t	a	a	t	t	g	t	c	g	g	a	a	c	a	g	c	c	t	c	g	g	g	t	g	t	t	g	t	g	g	t	a	c	t	t	g	c	a	a	t	a	g	g	g	c	t	g	c	a	t	t	a	a	g	g	a	a	t	t	c	g	t	a	a	g	t	a	c
KF853227	Vac	t	t	t	t	a	t	t	t	t	t	t	a	g	g	t	c	a	t	t	t	a	a	t	t	g	t	c	g	g	a	a	c	a	g	c	c	t	c	g	g	g	t	g	t	t	g	t	g	g	t	a	c	t	t	g	c	a	a	t	a	g	g	g	c	t	g	c	a	t	t	a	a	g	g	a	a	t	t	c	g	t	a	a	g	t	a	c
KF853228	Vac	t	t	t	t	a	t	t	t	t	t	t	a	g	g	t	c	a	t	t	t	a	a	t	t	g	t	c	g	g	a	a	c	a	g	c	c	t	c	g	g	g	t	g	t	t	g	t	g	g	t	a	c	t	t	g	c	a	a	t	a	g	g	g	c	t	g	c	a	t	t	a	a	g	g	a	a	t	t	c	g	t	a	a	g	t	a	c
KF853229	Vac	t	t	t	t	a	t	t	t	t	t	t	a	g	g	t	c	a	t	t	t	a	a	t	t	g	t	c	g	g	a	a	c	a	g	c	c	t	c	g	g	g	t	g	t	t	g	t	g	g	t	a	c	t	t	g	c	a	a	t	a	g	g	g	c	t	g	c	a	t	t	a	a	g	g	a	a	t	t	c	g	t	a	a	g	t	a	c
KF853230	Vac	t	t	t	t	a	t	t	t	t	t	t	a	g	g	t	c	a	t	t	t	a	a	t	t	g	t	c	g	g	a	a	c	a	g	c	c	t	c	g	g	g	t	g	t	t	g	t	g	g	t	a	c	t	t	g	c	a	a	t	a	g	g	g	c	t	g	c	a	t	t	a	a	g	g	a	a	t	t	c	g	t	a	a	g	t	a	c
KF853231	Vac	t	t	t	t	a	t	t	t	t	t	t	a	g	g	t	c	a	t	t	t	a	a	t	t	g	t	c	g	g	a	a	c	a	g	c	c	t	c	g	g	g	t	g	t	t	g	t	g	g	t	a	c	t	t	g	c	a	a	t	a	g	g	g	c	t	g	c	a	t	t	a	a	g	g	a	a	t	t	c	g	t	a	a	g	t	a	c
KF853232	Vac	t	t	t	t	a	t	t	t	t	t	t	a	g	g	t	c	a	t	t	t	a	a	t	t	g	t	c	g	g	a	a	c	a	g	c	c	t	c	g	g	g	t	g	t	t	g	t	g	g	t	a	c	t	t	g	c	a	a	t	a	g	g	g	c	t	g	c	a	t	t	a	a	g	g	a	a	t	t	c	g	t	a	a	g	t	a	c
KF853233	Vac	t	t	t	t	a	t	t	t	t	t	t	a	g	g	t	c	a	t	t	t	a	a	t	t	g	t	c	g	g	a	a	c	a	g	c	c	t	c	g	g	g	t	g	t	t	g	t	g	g	t	a	c	t	t	g	c	a	a	t	a	g	g	g	c	t	g	c	a	t	t	a	a	g	g	a	a	t	t	c	g	t	a	a	g	t	g	c
KF853234	Vac	t	t	t	t	a	t	t	t	t	t	t	a	g	g	t	c	a	t	t	t	a	a	t	t	g	t	c	g	g	a	a	c	a	g	c	c	t	c	g	g	g	t	g	t	t	g	t	g	g	t	a	c	t	t	g	c	a	a	t	g	g	g	g	c	t	g	c	a	t	t	a	a	g	g	a	a	t	t	c	g	t	a	a	g	t	a	c
KF853235	Vac	t	t	t	t	a	t	t	t	t	t	t	a	g	g	t	c	a	t	t	t	a	a	t	t	g	t	c	g	g	a	a	c	a	g	c	c	t	c	g	g	g	t	g	t	t	g	t	g	g	t	a	c	t	t	g	c	a	a	t	a	g	g	g	c	t	g	c	a	t	t	a	a	g	g	a	a	t	t	c	g	t	a	a	g	t	a	c
MF898328	Vac	t	t	t	t	a	t	t	c	t	t	t	a	g	g	t	c	a	t	t	t	a	a	t	t	g	t	c	g	g	a	a	c	a	g	c	c	t	c	g	g	g	t	g	t	t	g	t	g	g	t	a	c	t	t	g	c	a	a	t	a	g	g	g	c	t	g	c	a	t	t	a	a	g	g	a	a	t	t	c	g	t	a	a	g	t	g	c
JN704709	VIII	t	t	c	t	a	t	t	t	t	t	t	a	g	a	t	c	a	t	t	c	a	a	t	t	g	t	c	g	g	a	a	c	a	g	c	c	t	c	g	g	g	t	g	t	t	a	t	g	g	t	a	c	t	t	g	c	a	a	t	a	g	g	g	c	t	g	c	a	t	t	a	a	g	g	a	a	t	t	c	g	g	a	a	g	t	a	c

Supplementary Table 3: 2880 SN

GB-ID	Clade markers					5																																					SD14																											2																	
	Position in Dumas	5794	5827	5849	5883	6022	6138	6250	6268	6340	6407	6416	6420	6439	6462	6551	6577	6589	6652	6669	6829	6850	6853	7042	7091	7114	7150	7198	7304	7321	7363	7445	7450	7486	7641	7740	7753	7828	8043	8068	8136	8163	8174	8293	8297	8567	8589	8621	8699	8744	8813	8837	8881	9023	9035	9089	9101	9120	9184	9196	9243	9247	9251	9288	9289	9290	9404	9455	9459	9460	9470	9479	9488	9497	9503	9512	9524	9721	9814	9936	9954	10032	10043	10045	10069	10079	10119
AY548170	1	g	c	c	g	a	c	a	c	g	g	c	c	t	c	t	t	g	t	c	a	g	g	a	c	t	g	a	t	g	g	a	a	c	g	a	c	t	t	c	c	t	a	t	t	g	g	t	a	t	c	t	a	t	c	g	g	g	a	t	g	t	g	c	c	g	a	c	c	t	a	a	g	c	c	c	a	t	c	t	c	a	g	c	g	g	g
AY548171	1	g	c	c	g	a	c	a	c	g	g	c	c	t	c	t	t	g	t	c	a	g	g	a	c	t	g	a	t	g	g	a	a	c	g	a	c	t	t	c	c	t	a	t	t	g	t	t	a	t	c	t	a	t	c	g	g	g	a	t	g	t	g	c	c	g	a	c	c	t	a	a	g	c	c	c	a	t	c	t	c	a	g	c	g	g	g
DQ479953	1	g	c	c	g	a	c	a	c	g	g	c	c	t	c	t	t	g	t	c	a	g	g	a	c	t	g	a	t	g	g	a	a	c	g	a	c	t	t	c	c	t	a	t	t	g	t	t	a	t	c	t	a	t	c	g	g	g	a	t	g	t	g	c	c	g	a	c	c	t	a	a	g	c	c	c	a	t	c	t	c	a	g	c	g	g	g
DQ479954	1	g	c	c	g	a	c	a	c	g	g	c	c	t	c	t	t	g	t	c	a	g	g	a	c	t	g	a	t	g	g	a	a	c	g	a	c	t	t	c	c	t	a	t	t	g	t	t	a	t	c	t	a	t	c	g	g	g	a	t	g	t	g	c	c	g	a	c	c	t	a	a	g	c	c	c	a	t	c	t	c	a	g	c	g	g	g
DQ479958	1	g	c	c	g	a	c	a	c	g	g	c	c	t	c	t	t	g	t	c	a	g	g	a	c	t	g	a	t	g	g	a	a	c	g	a	c	t	t	c	c	t	a	t	t	g	t	t	a	t	c	t	a	t	c	g	g	g	a	t	g	t	g	c	c	g	a	c	c	t	a	a	g	c	c	c	a	t	c	t	c	a	g	c	g	g	g
DQ479959	1	g	c	c	g	a	c	a	c	g	g	c	c	t	c	t	t	g	t	c	a	g	g	a	c	t	g	a	t	g	g	a	a	c	g	a	c	t	t	c	c	t	a	t	t	g	t	t	a	t	c	t	a	t	c	g	g	g	a	t	g	t	g	c	c	g	a	c	c	t	a	a	g	c	c	c	a	t	c	t	c	a	g	c	g	g	g
DQ479961	1	g	c	c	g	a	c	a	c	g	g	c	c	t	c	t	t	g	t	c	a	g	g	a	c	t	g	a	t	g	g	a	a	c	g	a	c	t	t	c	c	t	a	t	t	g	t	t	a	t	c	t	a	t	c	g	g	g	a	t	g	t	g	c	c	g	a	c	c	t	a	a	g	c	c	c	a	t	c	t	c	a	g	c	g	g	g
DQ479962	1	g	c	c	g	a	c	a	c	g	g	c	c	t	c	t	t	g	t	c	a	g	g	a	c	t	g	a	t	g	g	a	a	c	g	a	c	t	t	c	c	t	a	t	t	g	t	t	a	t	c	t	a	t	c	g	g	g	a	t	g	t	g	c	c	g	a	c	c	t	a	a	g	c	c	c	a	t	c	t	c	a	g	c	g	g	g
DQ479963	1	g	c	c	g	a	c	a	c	g	g	c	c	t	c	t	t	g	t	c	a	g	g	a	c	t	g	a	t	g	g	a	a	c	g	a	c	t	t	c	c	t	a	t	t	g	t	t	a	t	c	t	a	t	c	g	g	g	a	t	g	t	g	c	c	g	a	c	c	t	a	a	g	c	c	c	a	t	c	t	c	a	g	c	g	g	g
DQ674250	1	g	c	c	g	a	c	a	c	g	g	c	c	t	c	t	t	g	t	c	a	g	g	a	c	t	g	a	t	g	g	a	a	c	g	a	c	t	t	c	c	t	a	t	t	g	t	t	a	t	c	t	a	t	c	g	g	g	a	t	g	t	g	c	c	g	a	c	c	t	a	a	g	c	c	c	a	t	c	t	c	a	g	c	g	g	g
KX262864	1	g	c	c	g	a	c	a	c	g	g	c	c	t	c	t	t	g	t	c	a	g	g	a	c	t	g	a	t	g	g	a	a	c	g	a	c	t	t	c	c	t	a	t	t	g	t	t	a	t	c	t	a	t	c	g	g	g	a	t	g	t	g	c	c	g	a	c	c	t	a	a	g	c	c	c	a	t	c	t	c	a	g	c	g	g	g
KX262865	1	g	c	c	g	a	t	a	c	g	g	c	c	t	c	t	t	g	t	c	a	g	g	a	c	t	g	a	t	g	g	a	a	c	g	a	c	t	t	c	c	t	a	t	t	g	t	t	a	t	c	t	a	t	c	g	g	g	a	t	g	t	g	c	c	g	a	c	c	t	a	a	g	c	c	c	a	t	c	t	c	a	g	c	g	g	g
KX262866	1	g	c	c	g	a	c	a	c	g	g	c	c	t	c	t	t	g	t	c	a	g	g	a	c	t	g	a	t	g	g	a	a	c	g	a	c	t	t	c	c	t	a	t	t	g	t	t	a	a	c	t	a	t	c	g	g	g	a	t	g	t	g	c	c	g	a	c	c	t	a	a	g	c	c	c	a	t	c	t	c	a	g	c	g	g	g
EU154348	1	g	c	c	g	a	c	a	c	g	g	c	c	t	c	t	t	g	t	c	a	g	g	a	c	t	g	a	t	g	g	a	a	c	g	a	c	t	t	c	c	t	a	t	t	g	t	t	a	t	c	t	a	t	c	g	g	g	a	t	g	t	g	c	c	g	a	c	c	t	a	a	g	c	c	c	a	t	c	t	c	a	g	c	g	g	g
JN704690	1	g	c	c	g	a	c	a	c	g	g	c	c	t	c	t	t	g	t	c	a	g	g	a	c	t	g	a	t	g	g	a	a	c	g	a	c	t	t	c	c	t	a	t	t	g	t	t	a	t	c	t	a	t	c	g	g	g	a	t	g	t	g	c	c	g	a	c	c	t	a	a	g	c	c	c	a	t	c	t	c	a	g	c	g	g	g
JN704691	1	g	c	c	g	a	c	a	c	g	g	c	c	t	c	t	t	g	t	c	a	g	g	a	c	t	g	a	t	g	g	a	a	c	g	a	c	t	t	c	c	t	a	t	t	g	t	t	a	t	c	t	a	t	c	g	g	g	a	t	g	t	g	c	c	g	a	c	c	t	a	a	g	c	c	c	a	t	c	t	c	a	g	c	g	g	g
JN704692	1	g	c	c	g	a	c	a	c	g	g	c	c	t	c	t	t	g	t	c	a	g	g	a	c	t	g	a	t	g	g	a	a	c	g	a	c	t	t	c	c	t	a	t	t	g	t	t	a	t	c	t	a	t	c	g	g	g	a	t	g	t	g	c	c	g	a	c	c	t	a	a	g	c	c	c	a	t	c	t	c	a	g	c	g	g	g
JN704693	1	g	c	c	g	a	c	a	c	g	g	c	c	t	c	t	t	g	t	c	a	g	g	a	c	t	g	a	t	g	g	a	a	c	g	a	c	t	t	c	c	t	a	t	t	g	t	t	a	t	c	t	a	t	c	g	g	g	a	t	g	t	g	c	c	g	a	c	c	t	a	a	g	c	c	c	a	t	c	t	c	a	g	c	g	g	g
JN704694	1	g	c	c	g	a	c	a	c	g	g	c	c	t	c	t	t	g	t	c	a	g	g	a	c	t	g	a	t	g	g	a	a	c	g	a	c	t	t	c	c	t	a	t	t	g	t	t	a	t	c	t	a	t	c	g	g	g	a	t	g	t	g	c	c	g	a	c	c	t	a	a	g	c	c	c	a	t	c	t	c	a	g	c	g	g	g
JN704695	1	g	c	c	g	a	c	a	c	g	g	c	c	t	c	t	t	g	t	c	a	g	g	a	c	t	g	a	t	g	g	a	a	c	g	a	c	t	t	c	c	t	a	t	t	g	t	t	a	t	c	t	a	t	c	g	g	g	a	t	g	t	g	c	c	g	a	c	c	t	a	a	g	c	c	c	a	t	c	t	c	a	g	c	g	g	g
JN704696	1	g	c	c	g	a	c	a	c	g	g	c	c	t	c	t	t	g	t	c	a	g	g	a	c	t	g	a	t	g	g	a	a	c	g	a	c	t	t	c	c	t	a	t	t	g	t	t	a	t	c	t	a	t	c	g	g	g	a	t	g	t	g	c	c	g	a	c	c	t	a	a	g	c	c	c	a	t	c	t	c	a	g	c	g	g	g
NC001348	1	g	c	c	g	a	c	a	c	g	g	c	c	t	c	t	t	g	t	c	a	g	g	a	c	t	g	a	t	g	g	a	a	c	g	a	c	t	t	c	c	t	a	t	t	g	t	t	a	t	c	t	a	t	c	g	g	g	a	t	g	t	g	c	c	g	a	c	c	t	a	a	g	c	c	c	a	t	c	t	c	a	g	c	g	g	g
KP771892	1	g	c	c	g	a	c	a	c	g	g	c	c	t	c	t	t	g	t	c	a	g	g	a	c	t	g	a	t	g	g	a	a	c	g	a	c	t	t	c	c	t	a	t	t	g	t	t	a	t	c	t	a	t	c	g	g	g	a	t	g	t	g	c	c	g	a	c	c	t	a	a	g	c	c	c	a	t	c	t	c	a	g	c	g	g	g
KP771894	1	g	c	c	g	a	c	a	c	g	g	c	c	t	c	t	t	g	t	c	a	g	g	a	c	t	g	a	t	g	g	a	a	c	g	a	c	t	t	c	c	t	a	t	t	g	t	t	a	t	c	t	a	t	c	g	g	g	a	t	g	t	g	c	c	g	a	c	c	t	a	a	g	c	c	c	a	t	c	t	c	a	g	c	g	g	g
KP771900	1	g	c	c	g	a	c	a	c	g	g	c	c	t	c	t	t	g	t	c	a	g	g	a	c	t	g	a	t	g	g	a	a	c	g	a	c	t	t	c	c	t	a	t	t	g	t	t	a	t	c	t	a	t	c	g	g	g	a	t	g	t	g	c	c	g	a	c	c	t	a	a	g	c	c	c	a	t	c	t	c	a	g	c	g	g	g
KP771901	1	g	c	c	g	a	c	a	c	g	g	c	c	t	c	t	t	g	t	c	a	g	g	a	c	t	g	a	t	g	g	a	a	c	g	a	c	t	t	c	c	t	a	t	t	a	t	t	a	t	c	t	a	t	c	g	g	g	a	t	g	t	g	c	c	g	a	c	c	t	a	a	g	c	c	c	a	t	c	t	c	a	g	c	g	g	g
KP771902	1	g	c	c	g	a	c	a	c	g	g	c	c	t	c	t	t	g	t	c	a	g	g	a	c	t	g	a	t	g	g	a	a	c	g	a	c	t	t	c	c	t	a	t	t	g	t	t	a	t	c	t	a	t	c	g	g	g	a	t	g	t	g	c	c	g	a	c	c	t	a	a	g	c	c	c	a	t	c	t	c	a	g	c	g	g	g
KP771908	1	g	c	c	g	a	c	a	c	g	g	c	c	t	c	t	t	g	t	c	a	g	g	a	c	t	g	a	t	g	g	a	a	c	g	a	c	t	t	c	c	t	a	t	t	g	t	t	a	t	c	t	a	t	c	g	g	g	a	t	g	t	g	c	c	g	a	c	c	t	a	a	g	c	c	c	a	t	c	t	c	a	g	c	g	g	g
KP771912	1	g	c	c	g	a	c	a	c	g	g	c	c	t	c	t	t	g	t	c	a	g	g	a	c	t	g	a	t	g	g	a	a	c	g	a	c	t	t	c	c	t	a	t	t	g	t	t	a	t	c	t	a	t	c	g	g	g	a	t	g	t	g	c	c	g	a	c	c	t	a	a	g	c	c	c	a	t	c	t	c	a	g	c	g	g	g
KP771913	1	g	c	c	g	a	c	a	c	g	g	c	c	t	c	t	t	g	t	c	a	g	g	a	c	t	g	a	t	g	g	a	a	c	g	a	c	t	t	c	c	t	a	t	t	g	t	t	a	t	c	t	a	t	c	g	g	g	a	t	g	t	g	c	c	g	a	c	c	t	a	a	g	c	c	c	a	t	c	t	c	a	g	c	g	g	g
KP771917	1	g	c	c	g	a	c	a	c	g	g	c	c	t	c	t	t	g	t	c	a	g	g	a	c	t	g	a	t	g	g	a	a	c	g	a	c	t	t	c	c	t	a	t	t	g	t	t	a	t	c	t	a	t	c	g	g	g	a	t	g	t	g	c	c	g	a	c	c	t	a	a	g	c	c	c	a	t	c	t	c	a	g	c	g	g	g
KP771918	1	g	c	c	g	a	c	a	c	g	g	c	c	t	c	t	t	g	t	c	a	g	g	a	c	t	g	a	t	g	g	a	a	c	g	a	c	t	t	c	c	t	a	t	t	g	t	t	a	t	c	t	a	t	c	g	g	g	a	t	g	t	g	c	c	g	a	c	c	t	a	a	g	c	c	c	a	t	c	t	c	a	g	c	g	g	g
KP771920	1	g	c	c	g	a	c	a	c	g	g	c	c	t	c	t	t	g	t	c	a	g	g	a	c	t	g	a	t	g	g	a	a	c	g	a	c	t	t	c	c	t	a	t	t	g	t	t	a	t	c	t	a	t	c	g	g	g	a	t	g	t	t	c	c	g	a	c	c	t	a	a	g	c	c	c	a	t	c	t	c	a	g	c	g	g	g
MH709361	1	g	c	c	g	a	c	a	c	g	g	c	c	t	c	t	t	g	t	c	a	g	g	a	c	t	g	a	t	g	g	a	a	c	g	a	c	t	t	c	c	t	a	t	t	g	t	t	a	t	c	t	a	t	c	g	g	g	a	t	g	t	g	c	c	g	a	c	c	t	a	a	g	c	c	c	a	t	c	t	c	a	g	c	g	g	g
KU926318	2	g	c	c	g	a	c	a	c	g	g	c	c	c	c	t	t	g	t	c	a	g	t	a	a	t	g	a	t	g	g	a	a	c	g	a	t	t	t	c	c	t	a	t	t	g	t	t	a	t	c	t	a	t	c	g	g	g	a	t	g	t	g	c	c	g	a	c	c	c	a	a	g	c	c	c	a	t	c	t	c	a	g	c	g	a	g
KU926319	2	g	c	c	g	a	c	a	c	g	g	c	c	t	c	t	t	g	t	c	a	g	t	a	a	t	g	a	t	g	g	a	a	c	g	a	t	t	t	c	c	t	a	t	t	g	t	t	a	t	c	t	a	t	c	g	g	g	a	t	g	t	g	t	c	g	a	c	c	c	a	a	g	c	c	c	a	t	c	t	c	a	g	c	g	a	g
KU926320	2	g	c	c	g	a	c	a	c	g	g	c	c	t	c	t	c	g	t	c	a	g	t	a	a	t	g	a	t	g	g	a	a	c	g	a	t	t	t	c	c	t	a	t	t	g	t	t	a	t	c	t	a	t	c	g	g	g	a	t	g	t	g	t	c	g	a	c	c	c	a	a	g	c	c	c	a	t	c	t	c	a	g	c	g	a	g
KU926321	2	g	c	c	g	a	c	a	c	g	g	c	c	t	c	t	t	g	t	c	a	g	t	a	a	t	g	a	t	g	g	a	a	c	g	a	t	t	t	c	c	t	a	t	t	g	t	t	a	t	c	t	a	t	c	g	g	g	a	t	g	t	g	c	c	g	a	c	c	c	a	a	g	c	c	c	a	t	c	t	c	a	g	c	g	a	g
KU926322	2	g	c	c	g	a	c	a	c	g	g	c	c	t	c	t	t	g	t	c	a	g	t	a	a	t	g	a	t	g	g	a	a	c	g	a	t	t	t	c	c	t	a	t	t	g	t	t	a	t	c	t	a	t	c	g	g	g	a	t	g	t	g	c	c	g	a	c	c	c	a	a	g	c	c	c	a	t	c	t	c	a	g	c	g	a	g
KC847290	2	g	c	c	g	a	c	a	c	g	g	c	c	t	c	t	t	g	t	c	a	g	t	a	a	t	g	a	t	g	g	a	a	c	g	a	t	t	t	c	c	t	a	t	t	g	t	t	a	t	c	t	a	t	c	g	g	g	a	t	g	t	g	c	c	g	a	c	c	c	a	a	g	c	c	c	a	t	c	t	c	a	g	c	g	a	g
KJ767491	2	g	c	c	g	a	c	a	c	g	g	c	c	t	c	t	t	g	t	c	a	g	t	a	a	t	g	a	t	g	g	a	a	c	g	a	t	t	t	c	c	t	a	t	t	g	t	t	a	t	c	t	a	t	c	g	g	g	a	t	g	t	g	c	c	g	a	c	c	c	a	a	g	c	c	c	a	t	c	t	c	a	g	c	g	a	g
KJ767492	2	g	c	c	g	a	c	a	c	g	g	c	c	t	c	t	t	g	t	c	a	g	t	a	a	t	g	a	t	g	g	a	a	c	g	a	t	t	t	c	c	t	a	t	t	g	t	t	a	t	c	t	a	t	c	g	g	g	a	t	g	t	g	t	c	g	a	c	c	c	a	a	g	c	c	c	a	t	c	t	c	a	g	c	g	a	g
KJ808816	2	g	c	c	g	a	c	a	c	g	g	c	c	t	c	t	t	g	t	c	a	g	t	a	a	t	g	a	t	g	g	a	a	c	g	a	t	t	t	c	c	t	a	t	t	g	t	t	a	t	c	t	a	t	c	g	g	g	a	t	g	t	g	c	c	g	a	c	c	c	a	a	g	c	c	c	a	t	c	t	c	a	g	c	g	a	g
KP771925	2	g	c	c	g	a	c	a	c	g	g	c	c	t	c	t	t	g	t	c	a	g	t	a	a	t	g	a	t	g	g	a	a	c	g	a	t	t	t	c	c	t	a	t	t	g	t	t	a	t	c	t	a	t	c	g	g	g	a	t	g	t	g	c	c	g	a	c	c	c	a	a	g	c	c	c	a	t	c	t	c	a	g	c	g	a	g
KU926315	2	g	c	c	g	a	c	a	c	g	g	c	c	t	c	t	t	g	t	c	a	g	t	a	a	t	g	a	t	g	g	a	a	c	g	a	t	t	t	c	c	t	a	t	t	g	t	t	a	t	c	t	a	t	c	g	g	g	a	t	g	t	g	c	c	g	a	c	c	c	a	a	g	c	c	c	a	t	c	t	c	a	g	c	g	a	g
KU926316	2	g	c	c	g	a	c	a	c	g	g	c	c	t	c	t	t	g	t	c	a	g	t	a	a	t	g	a	t	g	g	a	a	c	g	a	t	t	t	c	c	t	a	t	t	g	t	t	a	t	c	t	a	t	c	g	g	g	a	t	g	t	g	c	c	g	a	c	c	c	a	a	g	c	c	c	a	t	c	t	c	a	g	c	g	a	g
KU926317	2	g	c	c	g	a	c	a	c	g	g	c	c	t	c	t	t	g	t	c	a	g	t	a	a	t	g	a	t	g	g	a	a	c	g	a	t	t	t	c	c	t	a	t	t	g	t	t	a	t	c	t	a	t	c	g	g	g	a	t	g	t	g	c	c	g	a	c	c	c	a	a	g	c	c	c	a	t	c	t	c	a	g	c	g	a	g
KY037796	2	g	c	c	g	a	c	a	c	g	g	c	c	t	c	t	t	g	t	c	a	g	t	a	a	t	g	a	t	g	g	a	a	c	g	a	t	t	t	c	c	t	a	t	t	g	t	t	a	t	c	t	a	t	c	g	g	g	a	t	g	t	g	t	c	g	a	c	c	c	a	a	g	c	c	c	a	t	c	t	c	a	g	c	g	a	g
MF004348	2	g	c	c	g	a	c	a	c	g	g	c	c	t	c	t	t	g	t	c	a	g	t	a	a	t	g	a	t	g	g	a	a	c	g	a	t	t	t	c	c	t	a	t	t	g	t	t	a	t	c	t	a	t	c	g	g	g	a	t	g	t	g	c	c	g	a	c	c	c	a	a	g	c	c	c	a	t	c	t	c	a	g	c	g	a	g
MH709309	2	g	c	c	g	a	c	a	c	g	g	c	c	t	c	t	t	g	t	c	a	g	t	a	a	t	g	a	t	g	g	a	a	c	g	a	t	t	t	c	c	t	a	t	t	g	t	t	a	t	c	t	a	t	c	g	g	g	a	t	g	t	g	c	c	g	a	c	c	c	a	a	g	c	c	c	a	t	c	t	c	a	g	c	g	a	g
MH709310	2	g	c	c	g	a	c	a	c	g	g	c	c	t	c	t	t	g	t	c	a	g	t	a	a	t	g	a	t	g	g	a	a	c	g	a	t	t	t	c	c	t	a	t	t	g	t	t	a	t	c	t	a	t	c	g	g	g	a	t	g	t	g	c	c	g	g	c	c	c	a	a	g	c	c	c	a	t	c	t	c	a	g	c	g	a	g
MH709311	2	g	c	t	g	a	c	a	c	g	g	c	c	t	c	t	t	g	t	c	a	g	t	a	a	t	g	a	t	g	g	a	a	c	g	a	t	t	t	c	c	c	a	t	t	g	t	t	a	t	c	t	a	t	c	g	g	g	a	t	g	t	g	c	c	g	a	c	c	c	a	a	g	c	c	c	t	t	c	t	c	a	g	c	g	a	g
MH709313	2	g	c	c	g	a	c	a	c	g	g	c	c	t	c	t	t	g	t	c	a	g	t	a	a	t	g	a	t	g	g	a	a	c	g	a	t	t	t	c	c	t	a	t	t	g	t	t	a	t	c	t	a	t	c	g	g	g	a	t	g	t	g	c	c	g	a	c	c	c	a	a	g	c	c	c	a	t	c	t	c	a	g	c	g	a	g
MH709314	2	g	c	c	g	a	c	a	c	g	g	c	c	t	c	t	t	g	t	c	a	g	t	a	a	t	g	a	t	g	g	a	a	c	g	a	t	t	t	c	c	t	a	t	t	g	t	t	a	t	c	t	a	t	c	g	g	g	a	t	g	t	g	c	c	t	a	c	c	c	a	a	g	c	c	c	a	t	c	t	c	a	g	c	g	a	g
MH709315	2	g	c	c	g	a	c	a	c	g	g	c	c	t	c	t	t	g	t	c	a	g	t	a	a	t	g	a	t	g	g	a	a	c	g	a	t	t	t	c	c	t	a	t	t	g	t	t	a	t	c	t	a	t	c	g	g	g	a	t	g	t	g	c	c	t	a	c	c	c	a	a	g	c	c	c	a	t	c	t	c	a	g	c	g	a	g
MH709319	2	g	c	c	g	a	c	a	c	g	g	c	c	t	a	t	t	g	t	c	a	g	t	a	a	t	g	a	t	g	g	a	a	c	g	a	t	t	t	c	c	t	a	t	t	g	t	t	a	t	c	t	a	t	c	g	g	g	a	t	g	t	g	c	c	t	a	c	c	c	a	a	g	c	c	c	a	t	c	g	c	a	g	c	g	a	g
MH709320	2	g	c	c	g	a	c	a	c	g	g	c	c	t	c	t	t	g	t	c	a	g	t	a	a	t	g	a	t	g	g	a	a	c	g	a	t	t	t	c	c	t	a	t	t	g	t	t	a	t	c	g	a	t	c	g	g	g	a	t	g	t	g	c	c	g	a	a	c	c	a	a	g	c	c	c	a	t	c	t	c	a	g	c	g	a	g
MH709329	2	g	c	c	g	a	c	a	c	g	g	c	c	t	c	t	t	g	t	c	a	g	t	a	a	t	g	a	t	g	g	a	a	c	g	a	t	t	t	c	c	t	a	t	t	g	t	t	a	t	c	t	a	t	c	g	g	g	a	t	g	t	g	c	c	t	a	c	c	c	a	a	g	c	c	c	a	t	c	t	c	a	g	c	g	a	g
MH709331	2	g	c	c	g	a	c	a	c	g	g	c	c	t	c	t	t	g	t	c	a	g	t	a	a	t	g	a	t	g	g	a	a	c	g	a	t	t	t	c	c	t	a	t	t	g	t	t	a	t	c	t	a	t	c	g	g	g	a	t	g	t	g	c	c	g	a	c	c	c	a	a	g	c	c	c	a	t	c	t	c	a	g	c	g	a	g
MH709333	2	g	c	c	a	a	c	a	c	g	g	c	c	t	c	t	t	g	t	c	a	g	t	a	a	t	g	a	t	g	g	a	a	c	g	a	t	t	t	c	c	t	a	t	t	g	t	t	a	t	c	t	a	t	c	g	g	g	a	t	g	t	g	c	c	t	a	c	c	c	a	a	g	c	c	c	a	t	c	t	c	a	g	c	g	a	g
MH709337	2	g	c	c	g	a	c	a	c	g	g	c	c	t	c	t	t	g	t	c	a	g	t	a	a	t	g	a	t	a	g	a	a	c	g	a	t	t	t	c	c	t	a	t	t	g	t	t	a	t	c	t	a	t	c	g	g	g	a	t	g	t	g	c	c	g	a	c	c	c	a	a	g	c	c	c	a	t	c	t	c	a	g	c	g	a	g
MH709339	2	g	c	c	g	a	c	a	c	g	g	c	c	t	c	t	t	g	t	c	a	g	t	a	a	t	g	a	t	g	g	a	a	c	g	a	t	t	t	c	c	t	a	t	t	g	t	t	a	t	c	t	a	t	c	g	g	g	a	t	g	t	g	c	c	g	a	c	c	c	a	a	g	c	c	c	a	t	c	t	c	a	g	c	g	a	g
MH709344	2	g	c	c	g	a	c	a	c	g	g	c	c	t	c	t	t	g	t	c	a	g	t	a	a	t	g	a	t	g	g	a	a	c	g	a	t	t	t	c	c	t	a	t	t	g	t	t	a	t	c	t	a	t	c	g	g	g	a	t	g	t	g	c	c	g	a	c	c	c	a	a	g	c	c	c	a	t	c	t	c	a	g	c	g	a	g
MH709348	2	g	c	c	g	a	c	a	c	g	g	c	c	t	c	t	t	g	t	c	a	g	t	a	a	t	g	a	t	g	g	a	a	c	g	a	t	t	t	c	c	t	a	t	t	g	t	t	a	t	c	t	a	t	c	g	g	g	a	t	g	t	g	c	c	g	a	c	c	c	a	a	g	c	c	c	a	t	c	t	c	a	g	c	g	a	g
MH709355	2	g	c	c	g	a	c	a	c	g	t	c	c	t	c	t	t	g	t	c	a	g	t	a	a	t	g	a	t	a	g	a	a	c	g	a	t	t	t	c	c	t	a	t	t	g	t	t	a	t	c	t	g	t	c	g	g	g	a	t	g	t	g	c	c	g	a	c	c	c	a	a	g	c	c	c	a	t	c	t	c	a	g	c	g	a	g
MH709359	2	g	c	c	g	a	c	a	c	g	g	c	c	t	c	t	t	g	t	c	a	g	t	a	a	t	g	a	t	g	g	a	a	c	g	a	t	t	t	c	c	t	a	t	t	g	t	t	a	t	c	t	a	t	c	g	g	g	a	t	g	t	g	c	c	g	a	c	c	c	a	a	g	c	c	c	a	t	c	t	c	a	g	c	g	a	g
MH709363	2	g	c	c	g	a	c	a	c	g	g	c	c	t	c	t	t	g	t	c	a	g	t	a	a	t	g	a	t	g	g	a	a	c	g	a	t	t	t	c	c	t	a	t	t	g	t	t	a	t	c	g	a	t	c	g	g	g	a	t	g	t	g	c	c	g	a	a	c	c	a	a	g	c	c	c	a	t	c	t	c	a	g	c	g	a	g
MH709366	2	g	c	c	g	a	c	a	c	g	g	c	c	t	c	t	t	g	t	c	a	g	t	a	a	t	g	a	t	g	g	a	a	c	g	a	t	t	t	c	c	t	a	t	t	g	t	t	a	t	c	t	a	t	c	g	g	g	a	t	g	t	g	c	t	g	a	c	c	c	a	a	g	c	c	c	a	t	c	t	c	a	g	c	g	a	g
MH709376	2	g	c	c	g	a	c	a	c	g	g	c	c	t	c	t	t	g	t	c	a	g	t	a	a	t	g	a	t	g	g	a	a	c	g	a	t	t	t	c	c	t	a	t	t	g	t	t	a	t	c	t	a	t	c	g	g	g	g	t	g	t	g	c	c	g	a	c	c	c	a	a	g	c	c	c	a	t	c	t	c	a	g	c	g	a	g
MH709377	2	g	c	c	g	a	c	a	c	g	g	c	c	t	c	t	t	g	t	c	a	g	t	a	a	t	g	a	t	g	g	a	a	c	g	a	t	t	t	c	c	t	a	t	t	g	t	t	a	t	c	t	a	t	c	g	g	g	a	t	g	t	g	c	c	g	a	c	c	c	a	a	g	c	c	c	a	t	c	t	c	a	g	c	g	a	g
**MW316406**	2	g	c	c	g	a	c	a	c	g	g	c	c	t	c	t	t	g	t	c	a	g	t	a	a	t	g	a	t	g	g	a	a	c	g	a	t	t	t	c	c	t	c	t	t	g	t	t	a	t	c	t	a	t	c	g	g	g	a	t	g	t	g	c	c	g	a	c	c	c	a	a	g	c	c	c	a	t	c	t	c	a	g	c	g	a	g
AJ871403	3	g	a	c	g	a	c	a	c	g	g	c	c	t	c	t	t	g	t	c	g	g	g	a	c	t	g	a	t	g	g	a	a	c	g	a	c	t	t	c	c	t	a	t	t	g	t	t	a	t	c	t	a	t	c	g	g	g	a	t	a	t	g	c	c	g	a	c	c	t	a	a	g	c	c	c	a	t	c	t	c	a	g	c	g	g	g
DQ479955	3	g	a	c	g	a	c	a	c	g	g	c	c	t	c	t	t	g	t	c	a	g	g	a	c	t	g	a	t	g	g	a	a	c	g	a	c	t	t	c	c	t	a	t	t	g	t	t	a	t	c	t	a	t	c	g	g	g	a	t	a	t	g	c	c	g	a	c	c	t	a	a	g	c	c	c	a	t	c	t	c	a	g	c	g	g	g
DQ479956	3	g	a	c	g	a	c	a	c	g	g	c	c	t	c	t	t	g	t	c	a	g	g	a	c	t	g	a	t	g	g	a	a	c	g	a	c	t	t	c	c	t	a	t	t	g	t	t	a	t	c	t	a	t	c	g	g	g	a	t	a	c	g	c	c	g	a	c	c	t	a	a	g	c	c	c	a	t	c	t	c	a	g	c	g	g	g
DQ479957	3	g	a	c	g	a	c	a	c	g	g	c	c	t	c	t	t	g	t	c	a	g	g	a	c	t	g	a	t	g	g	a	a	c	g	a	c	t	t	c	c	t	a	t	t	g	t	t	a	t	c	t	a	t	c	g	g	g	a	t	a	t	g	c	c	g	a	c	c	t	a	a	g	c	c	c	a	t	c	t	c	a	g	c	g	g	g
JN704699	3	g	a	c	g	a	c	a	c	g	g	t	c	t	c	t	t	g	t	c	a	g	g	a	c	t	g	a	t	g	g	a	a	c	g	a	c	t	t	c	c	t	a	t	t	g	t	t	a	t	c	t	a	t	c	g	g	g	a	t	a	t	g	c	c	g	a	c	c	t	a	a	g	c	c	c	a	t	c	t	c	a	g	c	g	g	g
JN704700	3	g	a	c	g	a	c	a	c	g	g	c	c	t	c	t	t	g	t	c	a	g	g	a	c	t	g	a	t	g	g	a	a	c	g	a	c	t	t	c	c	t	a	t	t	g	t	t	a	t	c	t	a	t	c	g	g	g	a	t	a	t	g	c	c	g	a	c	t	t	a	a	g	c	c	c	a	t	c	t	c	a	g	c	t	g	g
JN704701	3	g	a	c	g	a	c	a	c	g	g	c	c	t	c	t	t	a	t	c	a	g	g	a	c	t	g	a	t	g	g	a	a	c	g	a	c	t	t	c	c	t	a	t	t	g	t	t	a	t	c	t	a	t	c	g	g	g	a	t	a	t	g	c	c	g	a	c	c	t	a	a	g	c	c	c	a	t	c	t	c	a	g	c	g	g	g
JN704702	3	g	a	c	g	a	c	a	c	g	g	c	c	t	c	t	t	g	t	c	a	g	g	a	c	t	g	a	t	g	g	a	a	c	g	a	c	t	t	c	c	t	a	t	t	g	t	t	a	t	c	t	a	t	c	g	g	g	a	t	a	t	g	c	c	g	a	c	c	t	a	a	g	c	c	c	a	t	c	t	c	a	g	c	g	g	g
JN704703	3	g	a	c	g	a	c	a	c	g	g	c	c	t	c	t	t	g	t	c	a	g	g	a	c	t	g	a	g	g	g	a	a	c	g	a	c	t	t	c	c	t	a	t	t	g	t	t	a	t	c	t	a	t	c	g	g	g	a	t	a	t	g	c	c	g	a	c	c	t	a	a	g	c	c	c	a	t	c	t	c	a	g	c	g	g	g
KP771891	3	g	a	c	g	a	c	a	c	g	g	c	c	t	c	t	t	g	t	c	a	g	g	a	c	t	g	a	t	g	g	g	a	c	g	a	c	t	t	c	c	t	a	t	t	g	t	t	a	t	c	t	a	t	c	g	g	g	a	c	a	t	g	c	c	g	a	c	c	t	a	a	g	c	c	c	a	t	c	t	c	a	g	c	t	g	g
KP771893	3	g	a	c	g	a	c	a	c	g	g	c	c	t	c	t	t	g	t	c	a	g	g	a	c	t	g	a	t	g	g	a	a	c	g	a	c	t	t	c	c	t	a	t	t	g	t	t	a	t	c	t	a	t	c	g	g	g	a	t	a	t	g	c	c	g	a	c	c	t	a	a	g	c	c	c	a	t	c	t	c	a	g	c	g	g	g
KP771895	3	g	a	c	g	a	c	g	c	g	g	c	c	t	c	t	g	g	t	c	g	g	g	a	c	t	g	a	t	g	g	a	a	c	g	a	c	t	g	c	c	t	a	t	t	g	t	t	a	t	c	t	a	t	c	g	g	g	a	t	a	t	g	c	c	g	a	c	c	t	a	a	g	c	c	c	a	t	c	t	c	a	g	c	g	g	g
KP771896	3	g	a	c	g	a	c	a	c	g	g	c	c	t	c	t	t	g	t	c	a	g	g	a	c	t	g	a	t	g	g	a	a	c	g	a	c	t	t	c	c	t	a	t	t	g	t	t	a	t	c	t	a	t	c	g	g	g	a	t	a	t	g	c	c	g	a	c	c	t	a	a	g	c	c	c	a	t	c	t	c	a	g	c	g	g	g
KP771897	3	g	a	c	g	a	c	a	c	g	g	c	c	t	c	t	t	g	t	c	a	g	g	a	c	t	g	a	t	g	g	a	a	t	g	a	c	t	t	c	c	t	a	t	t	g	t	t	a	t	c	t	a	t	c	g	g	g	a	t	a	t	g	c	c	g	a	c	c	t	a	a	g	c	c	c	a	t	c	t	c	a	g	c	g	g	g
KP771899	3	g	a	c	g	a	c	a	c	g	g	c	c	t	c	t	t	g	t	c	g	g	g	a	c	t	g	a	t	g	g	a	a	c	g	a	c	t	t	c	c	t	a	t	t	g	t	t	a	t	c	t	a	t	c	g	g	g	a	t	a	t	g	c	c	g	a	c	c	t	a	a	g	c	c	c	a	t	c	t	c	a	g	c	g	g	g
KP771903	3	g	a	c	g	a	c	a	c	g	g	c	c	t	c	t	t	g	t	c	a	g	g	a	c	t	g	a	t	g	g	a	a	c	g	a	c	t	t	c	c	t	a	t	t	g	t	t	a	t	c	t	a	t	c	g	g	g	a	t	a	t	g	c	c	g	a	c	c	t	a	a	g	c	c	c	a	t	c	t	c	a	g	c	g	g	g
KP771907	3	g	a	c	g	a	c	a	c	g	g	c	c	t	c	t	t	g	t	c	g	g	g	a	c	t	g	a	t	g	g	a	a	c	g	a	c	t	t	c	c	t	a	t	t	g	t	t	a	t	c	t	a	t	c	g	g	g	a	t	a	t	g	c	c	g	a	c	c	t	a	a	g	c	c	c	a	t	c	t	c	a	g	c	g	g	g
KP771910	3	g	a	c	g	a	c	a	c	g	g	c	c	t	c	t	t	g	t	c	a	g	g	a	c	t	g	a	t	g	g	a	a	c	g	a	c	t	t	c	c	t	a	t	t	g	t	t	a	t	c	t	a	t	c	g	g	g	a	t	a	t	g	c	c	g	a	c	c	t	a	a	g	c	c	c	a	t	c	t	c	a	g	c	g	g	g
KP771915	3	g	a	c	g	a	c	a	c	g	g	c	c	t	c	t	t	g	t	c	a	g	g	a	c	t	g	a	t	g	g	a	a	c	g	a	c	t	t	c	c	t	a	t	t	g	t	t	a	t	c	t	a	t	c	g	a	g	a	t	a	c	g	c	c	g	a	c	c	t	a	a	g	c	c	c	a	t	c	t	c	a	g	c	g	g	g
KP771916	3	g	a	c	g	a	c	a	c	g	g	c	c	t	c	t	t	g	t	c	a	g	g	a	c	t	g	a	t	g	g	a	a	c	g	a	c	t	t	c	c	t	a	t	t	g	t	t	a	t	c	t	a	t	c	g	g	g	a	t	a	t	g	c	c	g	a	c	c	t	a	a	g	c	c	c	a	t	c	t	c	a	g	c	g	g	g
KP771921	3	g	a	c	g	a	c	a	c	g	g	c	c	t	c	t	t	g	t	c	a	g	g	a	c	t	g	a	t	g	g	a	a	c	g	a	c	t	t	c	c	t	a	t	t	g	t	t	a	t	c	t	a	t	c	g	g	g	a	t	a	t	g	c	c	g	a	c	c	t	a	a	g	c	c	c	a	t	c	t	c	a	g	c	t	g	g
KP771923	3	g	a	c	g	a	c	a	c	g	g	c	c	t	c	t	t	g	t	c	a	g	g	a	c	t	g	a	t	g	g	a	a	c	g	a	c	t	t	c	c	t	a	t	t	g	t	t	a	t	c	t	a	t	c	g	g	g	a	t	a	t	g	c	c	g	a	c	c	t	a	a	g	c	c	c	a	t	c	t	c	a	g	c	g	g	g
KP771889	3	g	a	c	g	a	c	a	c	g	g	c	c	t	c	t	t	g	t	c	a	g	g	a	c	t	g	a	t	g	g	a	a	c	g	a	c	t	t	c	c	t	a	t	t	g	t	t	a	t	c	t	a	t	c	g	g	g	a	t	a	t	g	c	c	g	a	c	c	t	a	a	g	c	c	c	a	t	c	t	c	a	g	c	g	g	g
KU926311	3	g	a	c	g	a	c	a	c	g	g	c	c	t	c	t	t	g	t	c	a	g	g	a	c	t	g	a	t	g	g	a	a	c	g	a	c	t	t	c	c	t	a	t	t	g	t	t	a	t	c	t	a	t	c	g	g	g	a	t	a	t	g	c	c	g	a	c	c	t	a	a	g	c	c	c	a	t	c	t	c	a	g	c	g	g	g
JQ911599	3	g	a	c	g	a	c	a	c	g	g	c	c	t	c	t	t	g	t	c	a	g	g	a	c	t	g	a	t	g	g	a	a	c	g	a	c	t	t	c	c	t	a	t	t	g	t	c	a	t	c	t	a	t	c	g	g	g	a	t	a	t	g	c	c	g	a	c	c	t	a	a	g	c	c	c	a	t	c	t	c	a	g	c	g	g	g
MH709317	3	g	a	c	g	a	c	a	c	g	g	c	c	t	c	t	t	g	t	c	a	g	g	g	c	t	g	a	t	g	g	a	a	c	g	a	c	t	t	c	c	t	a	t	t	g	t	t	a	t	c	t	a	t	c	g	g	g	a	t	g	t	g	c	c	g	a	c	c	t	a	a	g	c	c	c	a	t	c	t	c	a	g	c	g	g	g
MH709346	3	g	a	c	g	a	c	a	c	g	g	c	c	t	c	t	t	g	t	c	a	g	g	a	c	t	g	a	t	g	g	a	a	c	g	a	c	t	t	c	c	t	a	t	t	g	t	t	a	t	c	t	a	t	c	g	g	g	a	t	g	t	g	c	c	g	a	c	c	t	a	a	g	c	c	c	a	t	c	t	c	a	g	c	g	g	g
MH709375	3	g	a	c	g	a	c	a	c	g	g	c	c	t	c	t	t	g	t	c	a	g	g	a	c	t	g	a	t	g	g	a	a	c	g	a	c	t	t	c	c	t	a	t	t	g	t	t	a	t	c	t	a	t	c	g	g	g	a	t	g	t	g	c	c	g	a	c	c	t	a	a	g	c	c	c	a	t	c	t	c	a	g	c	g	g	g
DQ452050	4	g	c	c	g	a	c	a	c	g	g	c	c	t	c	t	t	g	a	c	a	a	t	a	a	t	g	a	t	g	g	a	a	c	g	a	t	t	t	c	c	t	a	t	t	g	t	t	a	t	c	t	a	t	c	g	g	g	a	t	g	t	g	c	c	g	a	c	c	t	g	a	g	c	c	c	a	t	c	t	c	a	g	c	g	a	g
DQ479960	4	g	c	c	g	a	c	a	c	g	g	c	c	t	c	t	t	g	a	c	a	a	t	a	a	t	g	a	t	g	g	a	a	c	g	a	t	t	t	c	c	t	a	t	t	g	t	t	a	t	c	t	a	t	c	g	g	g	a	t	g	t	g	c	c	g	a	c	c	t	a	a	g	c	c	c	a	t	c	t	c	a	g	c	g	a	g
KP771898	4	g	c	c	g	a	c	a	c	g	g	c	c	t	c	t	t	g	a	c	a	a	t	a	a	t	g	a	t	g	g	a	a	c	g	a	t	t	t	c	c	t	a	t	t	g	t	t	a	t	c	t	a	t	c	g	g	g	a	t	g	t	g	c	c	g	a	c	c	t	a	a	g	c	c	c	a	t	c	t	c	a	g	c	g	a	g
MH709316	4	g	a	c	g	a	c	a	c	g	g	c	c	t	c	t	t	g	t	c	a	a	t	a	a	t	g	a	t	g	g	a	a	c	g	a	c	t	t	c	c	t	a	t	t	g	t	t	a	t	c	t	a	t	c	g	g	g	a	t	g	t	g	c	c	g	a	c	c	t	a	a	g	c	c	c	a	t	c	t	a	a	g	c	g	g	g
MH709318	4	g	c	c	g	a	c	a	c	g	g	c	c	t	c	t	t	g	a	c	a	a	t	a	a	t	g	a	t	g	g	a	a	c	g	a	t	c	t	c	c	t	a	t	t	g	t	t	a	t	c	t	a	t	c	g	g	g	a	t	g	t	g	c	c	g	a	c	c	t	a	a	g	c	c	c	a	t	c	t	c	a	t	c	g	a	g
MH709322	4	g	c	c	g	a	c	a	c	g	g	c	c	t	c	t	t	g	a	c	a	a	t	a	a	t	g	a	t	g	g	a	a	c	g	a	t	t	t	c	c	t	a	t	t	g	t	t	a	t	c	t	a	t	c	g	g	g	a	t	g	t	g	c	c	g	a	c	c	t	a	a	g	c	c	c	a	t	c	t	c	a	g	c	g	a	g
MH709323	4	g	c	c	g	a	c	a	c	g	g	c	c	t	c	t	t	g	a	c	a	a	t	a	a	t	g	a	t	g	g	a	a	c	g	a	t	c	t	c	c	t	a	t	t	g	t	t	a	t	c	t	a	t	c	g	g	g	a	t	g	t	g	c	c	g	a	c	c	t	a	a	g	c	c	c	a	t	c	t	c	a	g	c	g	a	g
MH709326	4	g	c	c	g	a	c	a	c	g	g	c	c	t	c	t	t	g	a	c	a	a	t	a	a	t	g	a	t	g	g	a	a	c	g	a	t	c	t	c	c	t	a	t	t	g	t	t	a	t	c	t	a	t	c	g	g	g	a	t	g	t	g	c	c	g	a	c	c	t	a	a	g	c	c	c	a	t	c	t	c	a	g	c	g	a	g
MH709341	4	g	c	c	g	a	c	a	c	g	g	c	c	t	c	t	t	g	a	c	a	a	t	a	a	t	g	a	t	g	g	a	a	c	g	a	t	t	t	c	c	t	a	t	t	g	t	t	a	t	c	t	a	t	c	g	g	g	a	t	g	t	g	c	c	g	a	c	c	t	a	a	g	c	c	c	a	t	c	t	c	a	g	c	g	a	g
MH709357	4	g	c	c	g	a	c	a	c	g	g	c	c	t	c	t	t	g	a	c	a	a	t	a	a	t	g	a	t	g	g	a	a	c	g	a	t	t	t	c	c	t	a	t	t	g	t	t	a	t	c	t	a	t	c	g	g	g	a	t	g	t	g	c	c	g	a	c	c	t	a	a	g	c	c	c	a	t	c	t	c	a	g	c	g	a	g
MH709364	4	g	c	c	g	a	c	a	c	g	g	c	c	t	c	t	t	g	a	c	a	a	t	a	a	t	g	a	t	g	g	a	a	c	g	a	t	t	t	c	c	t	a	t	t	g	t	t	a	t	c	t	a	t	c	g	g	g	a	t	g	t	g	c	c	g	a	c	c	t	a	a	g	c	c	c	a	t	c	t	c	a	g	c	g	a	g
MH709370	4	g	c	c	g	a	c	a	c	a	g	c	c	t	c	t	t	g	a	c	a	a	t	a	a	t	g	a	t	g	g	a	a	c	g	a	t	t	t	c	c	t	a	t	t	g	t	t	a	t	c	t	a	t	c	g	g	g	a	t	g	t	g	c	c	g	a	c	c	t	a	a	g	c	c	c	a	t	c	t	c	a	g	c	g	a	g
MH709371	4	g	c	c	g	a	c	a	c	g	g	c	c	t	c	t	t	g	a	c	a	a	t	a	a	t	g	a	t	g	g	a	a	c	g	a	t	t	t	c	c	t	a	t	t	g	t	t	a	t	c	t	a	t	c	g	g	g	a	t	g	t	g	c	c	g	a	c	c	t	a	a	g	c	c	c	a	t	c	t	c	a	g	c	g	a	g
MH709372	4	g	c	c	g	a	c	a	c	g	g	c	c	t	c	t	t	g	a	c	a	a	t	a	a	t	g	a	t	g	g	a	a	c	g	a	t	t	t	c	c	t	a	t	t	g	t	t	a	t	c	t	a	t	c	g	g	g	a	t	g	t	g	c	c	g	a	c	c	t	a	a	g	c	c	c	a	t	c	t	c	a	g	c	g	a	g
DQ457052	5	g	c	c	g	g	c	a	c	g	g	c	c	t	c	t	t	g	a	c	a	a	t	a	a	t	g	a	t	g	g	a	a	c	g	a	c	t	t	c	c	c	a	t	t	g	t	t	a	t	c	t	a	t	c	a	g	g	a	t	g	t	g	c	c	g	a	c	c	t	a	a	g	c	c	c	a	t	c	t	c	a	g	c	g	g	g
JN704704	5	g	c	c	g	g	c	a	c	g	g	c	c	t	c	t	t	g	a	c	a	a	t	a	a	t	g	a	t	g	g	a	a	c	g	a	c	t	t	t	c	c	a	t	t	g	t	t	a	t	c	t	a	t	c	g	g	g	a	t	g	t	g	c	c	g	a	c	c	t	a	a	g	c	c	c	a	t	c	t	c	a	g	c	g	g	g
JN704705	5	g	c	c	g	g	c	a	c	g	g	c	c	t	c	t	t	g	a	c	a	a	t	a	a	t	g	a	t	g	g	a	a	c	g	a	c	t	t	c	c	t	a	t	t	g	t	t	a	t	c	t	a	t	c	g	g	g	a	t	g	t	g	c	c	g	a	c	c	t	a	a	g	c	c	c	a	t	c	t	c	a	g	c	g	g	g
JN704706	5	g	c	c	g	g	c	a	c	g	g	c	c	t	c	t	t	g	a	c	a	a	t	a	a	t	g	a	t	g	g	a	a	c	g	g	c	t	t	c	c	t	a	c	t	g	t	t	a	t	c	t	a	t	c	g	g	g	a	t	g	t	g	c	c	g	a	c	c	t	a	g	g	c	c	c	a	t	c	t	c	a	g	c	g	g	g
JN704707	5	g	c	c	g	g	c	a	c	g	g	c	c	t	c	t	t	g	a	a	a	a	t	a	a	t	g	a	t	g	g	a	a	c	g	a	c	t	t	c	c	c	a	t	t	g	t	t	a	t	c	t	a	t	c	g	g	g	a	t	g	t	g	c	c	g	a	c	c	t	a	a	g	c	c	c	a	t	c	t	c	a	g	c	g	g	g
JN704708	5	g	c	c	g	g	c	a	c	g	g	c	c	t	c	t	t	g	a	c	a	a	t	a	a	t	g	a	t	g	g	a	a	c	g	a	c	t	t	c	c	t	a	t	t	g	t	t	a	t	c	t	a	t	c	g	g	g	a	t	g	t	g	c	c	g	a	c	c	t	a	a	g	c	c	c	a	t	c	t	c	a	g	c	g	g	g
KP771904	5	g	c	c	g	g	c	a	c	g	g	c	c	t	c	t	t	g	a	c	a	a	t	a	a	t	g	a	t	g	g	a	a	c	g	a	c	t	t	c	c	c	a	t	t	g	t	t	a	t	c	t	a	t	c	a	g	g	a	t	g	t	g	c	c	g	a	c	c	t	a	a	g	c	c	c	a	t	c	t	c	a	g	c	g	g	g
KP771905	5	g	c	c	g	g	c	a	c	g	g	c	c	t	c	t	t	g	a	c	a	a	t	a	a	t	g	a	t	g	g	a	a	c	g	a	c	t	t	c	c	c	a	t	t	g	t	t	a	t	c	t	a	t	c	a	g	g	a	t	g	t	g	c	c	g	a	c	c	t	a	a	g	c	c	c	a	t	c	t	c	a	g	c	g	g	g
KP771906	5	g	c	c	g	g	c	a	c	g	g	c	c	t	c	t	t	g	a	c	a	a	t	a	a	t	g	a	t	g	g	a	a	c	g	a	c	t	t	c	c	c	a	t	t	g	t	t	a	t	c	t	a	t	c	a	g	g	a	t	g	t	g	c	c	g	a	c	c	t	a	a	g	c	c	c	a	t	c	t	c	a	g	c	g	g	g
KP771919	5	g	c	c	g	g	c	a	c	g	g	c	c	t	c	t	t	g	a	c	a	a	t	a	a	t	g	a	t	g	g	a	a	c	g	a	c	t	t	c	c	c	a	t	t	g	t	t	a	t	c	t	a	t	c	a	g	g	a	t	g	t	g	c	c	g	a	c	c	t	a	a	g	c	c	c	a	t	c	t	c	a	g	c	g	g	g
KP771922	5	g	c	c	g	g	c	a	c	g	g	c	c	t	c	t	t	g	a	c	a	a	t	a	a	t	g	a	t	g	g	a	a	c	g	a	c	t	t	c	c	c	a	t	t	g	t	t	a	t	c	t	a	t	c	a	g	t	a	t	g	t	g	c	c	g	a	c	c	t	a	a	g	c	c	c	a	t	c	t	c	a	g	c	g	g	g
KP771924	5	g	c	c	g	g	c	a	c	g	g	c	c	t	c	t	t	g	a	c	a	a	t	a	a	t	g	a	t	g	g	a	a	c	g	a	c	t	t	c	c	t	a	t	t	g	t	t	a	t	c	t	a	t	c	a	g	g	a	t	g	t	g	c	c	g	a	c	c	t	a	a	g	c	c	c	a	t	c	t	c	a	g	c	g	g	g
KM355696	5	g	c	c	g	g	c	a	c	g	g	c	c	t	c	t	t	g	a	c	a	a	t	a	a	t	g	a	t	g	g	a	a	c	g	a	c	t	t	c	c	t	a	t	t	g	t	t	a	t	c	t	a	t	c	a	g	g	a	t	g	t	g	c	c	g	a	c	c	t	a	a	g	c	c	c	a	t	c	t	c	a	g	c	g	g	g
KM355697	5	g	c	c	g	g	c	a	c	g	g	c	c	t	c	t	t	g	a	c	a	a	t	a	a	t	g	a	t	g	g	a	a	c	g	a	c	t	t	c	c	t	a	t	t	g	t	t	a	t	c	t	a	t	c	a	g	g	a	t	g	t	g	c	c	g	a	c	c	t	a	a	g	c	c	c	a	t	c	t	c	a	g	c	g	g	g
KM355698	5	g	c	c	g	g	c	a	c	g	g	c	c	t	c	t	t	g	a	c	a	a	t	a	a	t	g	a	t	g	g	a	a	c	g	a	c	t	t	c	c	t	a	t	t	g	t	t	a	t	c	t	a	t	c	a	g	g	a	t	g	t	g	c	c	g	a	c	c	t	a	a	g	c	c	c	a	t	c	t	c	a	g	c	g	g	g
KM355699	5	g	c	c	g	g	c	a	c	g	g	c	c	t	c	t	t	g	a	c	a	a	t	a	a	t	g	a	t	g	g	a	a	c	g	a	c	t	t	c	c	t	a	t	t	g	t	t	a	t	c	t	a	t	c	a	g	g	a	t	g	t	g	c	c	g	a	c	c	t	a	a	g	c	c	c	a	t	c	t	c	a	g	c	g	g	g
KM355701	5	g	c	c	g	g	c	a	c	g	g	c	c	t	c	t	t	g	a	c	a	a	t	a	a	t	g	a	t	g	g	a	a	c	g	a	c	t	t	c	c	t	a	t	t	g	t	t	a	t	c	t	a	t	c	a	g	g	a	t	g	t	g	c	c	g	a	c	c	t	a	a	g	c	c	c	a	t	c	t	c	a	g	c	g	g	g
KM355702	5	g	c	c	g	g	c	a	c	g	g	c	c	t	c	t	t	g	a	c	a	a	t	a	a	t	g	a	t	g	g	a	a	c	g	a	c	t	t	c	c	t	a	t	t	g	t	t	a	t	c	t	a	t	c	a	g	g	a	t	g	t	g	c	c	g	a	c	c	t	a	a	g	c	c	c	a	t	c	t	c	a	g	c	g	g	g
KM355703	5	g	c	c	g	g	c	a	c	g	g	c	c	t	c	t	t	g	a	c	a	a	t	a	a	t	g	a	t	g	g	a	a	c	g	a	c	t	t	c	c	t	a	t	t	g	t	t	a	t	c	t	a	t	c	a	g	g	a	t	g	t	g	c	c	g	a	c	c	t	a	a	g	c	c	c	a	t	c	t	c	a	g	c	g	g	g
KM355704	5	g	c	c	g	g	c	a	c	g	g	c	c	t	c	t	t	g	a	c	a	a	t	a	a	t	g	a	t	g	g	a	a	c	g	a	c	t	t	c	c	t	a	t	t	g	t	t	a	t	c	t	a	t	c	a	g	g	a	t	g	t	g	c	c	g	a	c	c	t	a	a	g	c	c	c	a	t	c	t	c	a	g	c	g	g	g
KM355705	5	g	c	c	g	g	c	a	c	g	g	c	c	t	c	t	t	g	a	c	a	a	t	a	a	t	g	a	t	g	g	a	a	c	g	a	c	t	t	c	c	t	a	t	t	g	t	t	a	t	c	t	a	t	c	a	g	g	a	t	g	t	g	c	c	g	a	c	c	t	a	a	g	c	c	c	a	t	c	t	c	a	g	c	g	g	g
KM355706	5	g	c	c	g	g	c	a	c	g	g	c	c	t	c	t	t	g	a	c	a	a	t	a	a	t	g	a	t	g	g	a	a	c	g	a	c	t	t	c	c	t	a	t	t	g	t	t	a	t	c	t	a	t	c	a	g	g	a	t	g	t	g	c	c	g	a	c	c	t	a	a	g	c	c	c	a	t	c	t	c	a	g	c	g	g	g
KM355707	5	g	c	c	g	g	c	a	c	g	g	c	c	t	c	t	t	g	a	c	a	a	t	a	a	t	g	a	t	g	g	a	a	c	g	a	c	t	t	c	c	t	a	t	t	g	t	t	a	t	c	t	a	t	c	a	g	g	a	t	g	t	g	c	c	g	a	c	c	t	a	a	g	c	c	c	a	t	c	t	c	a	g	c	g	g	g
KM355708	5	g	c	c	g	g	c	a	c	g	g	c	c	t	c	t	t	g	a	c	a	a	t	a	a	t	g	a	t	g	g	a	a	c	g	a	c	t	t	c	c	t	a	t	t	g	t	t	a	t	c	t	a	t	c	a	g	g	a	t	g	t	g	c	c	g	a	c	c	t	a	a	g	c	c	c	a	t	c	t	c	a	g	c	g	g	g
KM355709	5	g	c	c	g	g	c	a	c	g	g	c	c	t	c	t	t	g	a	c	a	a	t	a	a	t	g	a	t	g	g	a	a	c	g	a	c	t	t	c	c	t	a	t	t	g	t	t	a	t	c	t	a	t	c	a	g	g	a	t	g	t	g	c	c	g	a	c	c	t	a	a	g	c	c	c	a	t	c	t	c	a	g	c	g	g	g
KM355711	5	g	c	c	g	g	c	a	c	g	g	c	c	t	c	t	t	g	a	c	a	a	t	a	a	t	g	a	t	g	g	a	a	c	g	a	c	t	t	c	c	t	a	t	t	g	t	t	a	t	c	t	a	t	c	a	g	g	a	t	g	t	g	c	c	g	a	c	c	t	a	a	g	c	c	c	a	t	c	t	c	a	g	c	g	g	g
KM355712	5	g	c	c	g	g	c	a	c	g	g	c	c	t	c	t	t	g	a	c	a	a	t	a	a	t	g	a	t	g	g	a	a	c	g	a	c	t	t	c	c	t	a	t	t	g	t	t	a	t	c	t	a	t	c	a	g	g	a	t	g	t	g	c	c	g	a	c	c	t	a	a	g	c	c	c	a	t	c	t	c	a	g	c	g	g	g
KY037797	5	[illegible]	[illegible]	[illegible]	[illegible]	[illegible]	[illegible]	[illegible]	[illegible]	[illegible]	[illegible]	[illegible]	[illegible]	[illegible]	[illegible]	[illegible]	[illegible]	[illegible]	[illegible]	[illegible]	[illegible]	[illegible]	[illegible]	[illegible]	[illegible]	[illegible]	[illegible]	[illegible]	[illegible]	[illegible]	[illegible]	[illegible]	[illegible]	[illegible]	[illegible]	[illegible]	[illegible]	[illegible]	[illegible]	[illegible]	[illegible]	[illegible]	[illegible]	[illegible]	[illegible]	[illegible]	[illegible]	[illegible]	[illegible]	[illegible]	[illegible]	[illegible]	[illegible]	[illegible]	[illegible]	[illegible]	[illegible]	[illegible]	[illegible]	[illegible]	[illegible]	[illegible]	[illegible]	[illegible]	[illegible]	[illegible]	[illegible]	[illegible]	[illegible]	[illegible]	[illegible]	[illegible]	[illegible]	[illegible]	[illegible]	[illegible]	[illegible]	[illegible]	[illegible]	[illegible]	[illegible]	[illegible]	[illegible]	[illegible]	[illegible]	[illegible]	[illegible]

Supplementary Table 3: 2880 SN

GB-ID	Clade markers					5																							
	Position in Dumas	5794	5827	5849	5883	6022	6138	6250	6268	6340	6407	6416	6420	6439	6462	6551	6577	6589	6652	6669	6829	6850	6853	7042	7091	7114	7150	7198	7304
MH499467	5	g	c	c	g	g	c	a	c	g	g	c	c	t	c	t	t	g	a	c	a	a	t	a	a	t	g	a	t
MH499469	5	g	c	c	g	g	c	a	c	g	g	c	c	t	c	t	t	g	a	c	a	a	t	a	a	t	g	a	t
MH709321	5	g	c	c	g	g	c	a	c	g	g	c	c	t	c	t	t	g	a	c	a	a	t	a	a	t	a	a	t
MH709324	5	g	c	c	g	g	c	a	c	g	g	c	c	t	c	t	t	g	a	c	a	a	t	a	a	t	g	a	t
MH709325	5	g	c	c	g	g	c	a	c	g	g	c	c	t	c	t	t	g	a	c	a	a	t	a	a	t	g	a	t
MH709327	5	g	c	c	g	g	c	a	c	g	g	c	c	t	c	g	t	g	a	c	a	a	t	a	a	t	g	a	t
MH709328	5	g	c	c	g	g	c	a	c	g	g	c	c	t	c	t	t	g	a	c	a	a	t	a	a	t	g	a	t
MH709330	5	g	c	c	g	g	c	a	c	g	g	c	c	t	c	t	t	g	a	c	a	a	t	a	a	t	g	a	t
MH709332	5	g	c	c	g	g	c	a	c	g	g	c	c	t	c	t	t	g	a	c	a	a	t	a	a	t	g	a	t
MH709334	5	g	c	c	g	g	c	a	c	g	g	c	c	t	c	t	t	g	a	c	a	a	t	a	a	t	g	a	t
MH709335	5	g	c	c	g	g	c	a	c	g	g	c	c	t	c	t	t	g	a	c	a	a	t	a	a	t	g	a	t
MH709336	5	g	c	c	g	g	c	a	c	g	g	c	c	t	c	t	t	g	a	c	a	a	t	a	a	t	g	a	t
MH709338	5	g	c	c	g	g	c	a	c	g	g	c	c	t	c	t	t	g	a	c	a	a	t	a	a	t	g	a	t
MH709340	5	g	c	c	g	g	c	a	c	g	g	c	c	t	c	t	t	g	a	c	a	a	t	a	a	t	g	a	t
MH709342	5	g	c	c	g	g	c	a	c	g	g	c	c	t	c	t	t	g	a	c	a	a	t	a	a	t	g	a	t
MH709343	5	g	c	c	g	g	c	a	c	g	g	c	c	t	c	t	t	g	a	c	a	a	t	a	a	t	g	a	t
MH709345	5	g	c	c	g	g	c	a	c	g	g	c	c	t	c	t	t	g	a	c	a	a	t	a	a	t	g	a	t
MH709347	5	g	c	c	g	g	c	a	c	g	g	c	c	t	c	t	t	g	a	c	a	a	t	a	a	t	g	a	t
MH709349	5	g	c	c	g	g	c	a	c	g	g	c	c	t	c	t	t	g	a	c	a	a	t	a	a	t	g	a	t
MH709350	5	g	c	c	g	g	c	a	c	g	g	c	c	t	c	t	t	g	a	c	a	a	t	a	a	t	g	g	t
MH709351	5	g	c	c	g	g	c	a	c	g	g	c	c	t	c	t	t	g	a	c	a	a	t	a	a	c	g	a	t
MH709353	5	g	c	c	g	g	c	a	c	g	g	c	c	t	c	t	t	g	a	c	a	a	t	a	a	t	g	a	t
MH709358	5	g	c	c	g	g	c	a	c	g	g	c	c	t	c	t	t	g	a	c	a	a	t	a	a	t	g	a	t
MH709360	5	g	c	c	g	g	c	a	c	g	g	c	c	t	c	t	t	g	a	c	a	a	t	a	a	t	g	a	t
MH709362	5	g	c	c	g	g	c	a	c	g	g	c	c	t	c	t	t	g	a	c	a	a	t	a	a	t	g	a	t
MH709365	5	g	c	c	g	g	c	a	c	g	g	c	c	t	c	t	t	g	a	c	a	a	t	a	a	t	g	a	t
MH709373	5	g	c	c	g	g	c	a	c	g	g	c	c	t	c	t	t	g	a	c	a	a	t	a	a	t	g	a	t
MH709374	5	g	c	c	g	g	c	a	c	g	g	c	c	t	c	t	t	g	a	c	a	a	t	a	a	t	g	a	t
KC112914	6	g	a	c	g	a	c	a	c	g	g	c	c	t	c	t	t	g	t	c	a	g	g	a	c	t	g	a	t
KP771914	6	g	a	c	g	a	c	a	c	g	g	c	c	t	c	t	t	g	t	c	a	g	g	a	c	t	g	a	t
KP702725	6	g	a	c	g	a	c	a	c	g	g	c	c	t	c	t	t	g	t	c	a	g	g	a	c	t	g	a	t
KP771890	9	g	a	c	g	a	c	a	t	g	g	c	c	t	c	t	t	g	t	c	a	g	g	a	c	t	g	a	t
JN704710	9	a	a	c	g	a	c	a	c	g	g	c	c	t	c	t	t	g	t	c	a	g	g	a	c	t	g	a	t
KY037798	9	g	a	c	g	a	c	a	c	g	g	c	c	t	c	t	t	g	t	c	a	g	g	a	c	t	g	a	t
MH709312	9	g	a	c	g	a	c	a	c	g	g	c	c	t	c	t	t	g	t	c	a	g	g	a	c	t	g	a	t
MH709352	9	g	a	c	g	a	c	a	c	g	g	c	c	t	c	t	t	g	t	c	a	g	g	a	c	t	g	a	t
AB097933	Vac	g	c	c	g	a	c	a	c	g	g	c	c	t	c	t	t	g	t	c	a	g	t	a	a	t	g	a	t
JN704698	Vac	g	c	c	g	a	c	a	c	g	g	c	c	t	c	t	t	g	t	c	a	g	t	a	a	t	g	a	t
JN704697	Vac	g	c	c	g	a	c	a	c	g	g	c	c	t	c	t	t	g	t	c	a	g	t	a	a	t	g	a	t
DQ008355	Vac	g	c	c	g	a	c	a	c	g	g	c	c	t	c	t	t	g	t	c	a	g	t	a	a	t	g	a	t
JF306641	Vac	g	c	c	g	a	c	a	c	g	g	c	c	t	c	t	t	g	t	c	a	g	t	a	a	t	g	a	t
AB097932	Vac	g	c	c	g	a	c	a	c	g	g	c	c	t	c	t	t	g	t	c	a	g	t	a	a	t	g	a	t
DQ008354	Vac	g	c	c	g	a	c	a	c	g	g	c	c	t	c	t	t	g	t	c	a	g	t	a	a	t	g	a	t
KU926312	Vac	g	c	c	g	a	c	a	c	g	g	c	c	t	c	t	t	g	t	c	a	g	t	a	a	t	g	a	t
KU926313	Vac	g	c	c	g	a	c	a	c	g	g	c	c	t	c	t	t	g	t	c	a	g	t	a	a	t	g	a	t
KU926314	Vac	g	c	c	g	a	c	a	c	g	g	c	c	t	c	t	t	g	t	c	a	g	t	a	a	t	g	a	t
KF558371	Vac	g	c	c	g	a	c	a	c	g	g	c	c	t	c	t	t	g	t	c	a	g	t	a	a	t	g	a	t
KF558372	Vac	g	c	c	g	a	c	a	c	g	g	c	c	t	c	t	t	g	t	c	a	g	t	a	a	t	g	a	t
KF558373	Vac	g	c	c	g	a	c	a	c	g	g	c	c	t	c	t	t	g	t	c	a	g	t	a	a	t	g	a	t
KF558374	Vac	g	c	c	g	a	c	a	c	g	g	c	c	t	c	t	t	g	t	c	a	g	t	a	a	t	g	a	t
KF558375	Vac	g	c	c	g	a	c	a	c	g	g	c	c	t	c	t	t	g	t	c	a	g	t	a	a	t	g	a	t
KF558376	Vac	g	c	c	g	a	c	a	c	g	g	c	c	t	c	t	t	g	t	c	a	g	t	a	a	t	g	a	t
KF558377	Vac	g	c	c	g	a	c	a	c	g	g	c	t	t	c	t	t	g	t	c	a	g	t	a	a	t	g	a	t
KF558378	Vac	g	c	c	g	a	c	a	c	g	g	c	c	t	c	t	t	g	t	c	a	g	t	a	a	t	g	a	t
KF558379	Vac	g	c	c	g	a	c	a	c	g	g	c	c	t	c	t	t	g	t	c	a	g	t	a	a	t	g	a	t
KF558380	Vac	g	c	c	g	a	c	a	c	g	g	c	c	t	c	t	t	g	t	c	a	g	t	a	a	t	g	a	t
KF558381	Vac	g	c	c	g	a	c	a	c	g	g	c	c	t	c	t	t	g	t	c	a	g	t	a	a	t	g	a	t
KF558382	Vac	g	c	c	g	a	c	a	c	g	g	c	c	t	c	t	t	g	t	c	a	g	t	a	a	t	g	a	t
KF558383	Vac	g	c	c	g	a	c	a	c	g	g	c	c	t	c	t	t	g	t	c	a	g	t	a	a	t	g	a	t
KF558384	Vac	g	c	c	g	a	c	a	c	g	g	c	c	t	c	t	t	g	t	c	a	g	t	a	a	t	g	a	t
KF558385	Vac	g	c	c	g	a	c	a	c	g	g	c	c	t	c	t	t	g	t	c	a	g	t	a	a	t	g	a	t
KF558386	Vac	g	c	c	g	a	c	a	c	g	g	c	c	t	c	t	t	g	t	c	a	g	t	a	a	t	g	a	t
KF558387	Vac	g	c	c	g	a	c	a	c	g	g	c	c	t	c	t	t	g	t	c	a	g	t	a	a	t	g	a	t
KF558388	Vac	g	c	c	g	a	c	a	c	g	g	c	c	t	c	t	t	g	t	c	a	g	t	a	a	t	g	a	t
KF558389	Vac	g	c	c	g	a	c	a	c	g	g	c	c	t	c	t	t	g	t	c	a	g	t	a	a	t	g	a	t
KF558390	Vac	g	c	c	g	a	c	a	c	g	g	c	c	t	c	t	t	g	t	c	a	g	t	a	a	t	g	a	t
KF558391	Vac	g	c	c	g	a	c	a	c	g	g	c	c	t	c	t	t	g	t	c	a	g	t	a	a	t	g	a	t
KF811485	Vac	g	c	c	g	a	c	a	c	g	g	c	c	t	c	t	t	g	t	c	a	g	t	a	a	t	g	a	t
KF853225	Vac	g	c	c	g	a	c	a	c	g	g	c	c	t	c	t	t	g	t	c	a	g	t	a	a	t	g	a	t
KF853226	Vac	g	c	c	g	a	c	a	c	g	g	c	c	t	c	t	t	g	t	c	a	g	t	a	a	t	g	a	t
KF853227	Vac	g	c	c	g	a	c	a	c	g	g	c	c	t	c	t	t	g	t	c	a	g	t	a	a	t	g	a	t
KF853228	Vac	g	c	c	g	a	c	a	c	g	g	c	c	t	c	t	t	g	t	c	a	g	t	a	a	t	g	a	t
KF853229	Vac	g	c	c	g	a	c	a	c	g	g	c	c	t	c	t	t	g	t	c	a	g	t	a	a	t	g	a	t
KF853230	Vac	g	c	c	g	a	c	a	c	g	g	c	c	t	c	t	t	g	t	c	a	g	t	a	a	t	g	a	t
KF853231	Vac	g	c	c	g	a	c	a	c	g	g	c	c	t	c	t	t	g	t	c	a	g	t	a	a	t	g	a	t
KF853232	Vac	g	c	c	g	a	c	a	c	g	g	c	c	t	c	t	t	g	t	c	a	g	t	a	a	t	g	a	t
KF853233	Vac	g	c	c	g	a	c	a	c	g	g	c	c	t	c	t	t	g	t	c	a	g	t	a	a	t	g	a	t
KF853234	Vac	g	c	c	g	a	c	a	c	g	g	c	c	t	c	t	t	g	t	c	a	g	t	a	a	t	g	a	t
KF853235	Vac	g	c	c	g	a	c	a	c	g	g	c	c	t	c	t	t	g	t	c	a	g	t	a	a	t	g	a	t
MF898328	Vac	g	c	c	g	a	c	a	c	g	g	c	c	t	c	t	t	g	t	c	a	g	t	a	a	t	g	a	t
JN704709	VIII	g	a	c	g	a	c	a	c	g	g	c	c	t	c	t	t	g	t	c	a	g	t	a	a	t	g	a	t

GB-ID														SD14																
	7321	7363	7445	7450	7486	7641	7740	7753	7828	8043	8068	8136	8163	8174	8293	8297	8567	8589	8621	8699	8744	8813	8837	8881	9023	9035	9089	9101	9120	9184
MH499467	g	g	a	a	c	g	a	c	t	t	c	c	c	a	t	t	g	t	t	a	t	c	t	a	t	c	a	g	g	a
MH499469	g	g	a	a	c	g	a	c	t	t	c	c	c	a	t	t	g	t	t	a	t	c	t	a	t	c	a	g	g	a
MH709321	g	g	a	a	c	g	g	c	t	t	c	c	t	a	t	t	g	t	t	a	t	c	t	a	t	c	a	g	g	a
MH709324	g	g	a	a	c	g	a	c	t	t	c	c	c	a	t	t	g	t	t	a	t	c	t	a	t	c	a	g	g	a
MH709325	g	g	a	a	c	g	a	c	t	t	c	c	t	a	t	t	g	t	t	a	t	c	t	a	t	c	a	g	g	a
MH709327	g	g	a	a	c	g	a	c	t	t	c	c	c	a	t	t	g	t	t	a	t	c	t	a	t	c	a	g	g	a
MH709328	g	g	a	a	c	g	a	c	t	t	c	c	c	a	t	t	g	t	t	a	t	c	t	a	t	c	a	g	g	a
MH709330	g	g	a	a	c	g	a	c	t	t	c	c	c	a	t	t	g	t	t	a	t	c	t	a	t	c	a	g	g	a
MH709332	g	g	a	a	c	g	a	c	t	t	c	c	c	a	t	t	g	t	t	a	t	c	t	a	t	c	a	g	g	a
MH709334	g	g	a	a	c	g	a	c	t	t	c	c	c	a	t	t	g	t	t	a	t	c	t	a	t	c	a	g	g	a
MH709335	g	g	a	a	c	g	a	c	t	t	c	c	c	a	t	t	g	t	t	a	t	c	t	a	t	c	a	g	g	a
MH709336	g	g	a	a	c	g	a	c	t	t	c	c	c	a	t	t	g	t	t	a	t	c	t	a	t	c	a	g	g	a
MH709338	g	g	a	a	c	g	a	c	t	t	c	c	c	a	t	t	g	t	t	a	t	c	t	a	t	c	a	g	g	a
MH709340	g	g	a	a	c	g	a	c	t	t	c	c	t	a	t	t	g	t	t	a	t	c	t	a	t	c	a	g	g	a
MH709342	g	g	a	a	c	g	g	c	t	t	c	c	t	a	t	t	g	t	t	a	t	c	t	a	t	c	a	g	g	a
MH709343	g	g	a	c	c	g	a	c	t	t	c	c	c	a	t	t	g	t	t	a	t	c	t	a	t	c	a	g	g	a
MH709345	g	g	a	a	c	g	a	c	t	t	c	c	c	a	t	t	g	t	t	a	t	c	t	a	t	c	a	g	g	a
MH709347	g	g	a	a	c	a	a	c	t	t	c	c	c	a	t	t	g	t	t	a	t	c	t	a	t	c	a	g	g	a
MH709349	g	g	a	a	c	g	a	c	t	t	c	c	c	a	t	t	g	t	t	a	t	c	t	a	t	c	a	g	g	a
MH709350	g	g	a	a	c	g	a	c	t	t	c	c	c	a	t	t	g	t	t	a	t	c	t	a	t	c	a	g	g	a
MH709351	g	g	a	a	c	g	a	c	t	t	c	c	t	a	t	t	g	t	t	a	t	c	t	a	t	c	a	g	g	a
MH709353	g	g	a	a	c	g	a	c	t	t	c	c	c	a	t	t	g	t	t	a	t	c	t	a	t	c	a	g	g	a
MH709358	g	g	a	a	c	g	a	c	t	t	c	c	t	a	t	t	g	t	t	a	t	c	t	a	t	c	a	g	g	a
MH709360	g	g	a	a	c	g	a	c	t	t	c	c	c	a	t	t	g	t	t	a	t	c	t	a	t	c	a	g	g	a
MH709362	g	g	a	a	c	g	a	c	t	t	c	c	c	a	t	t	g	t	t	a	t	c	t	a	t	c	a	g	g	a
MH709365	g	g	a	a	c	g	a	c	t	t	c	c	c	a	t	t	g	t	t	a	t	c	t	a	t	c	a	g	g	a
MH709373	g	g	a	a	c	g	g	c	t	t	c	c	c	a	t	t	g	t	t	g	t	c	t	a	t	c	a	g	g	a
MH709374	g	g	a	a	c	g	a	c	t	t	c	c	c	a	t	t	g	t	t	a	t	c	t	a	t	c	a	g	g	a
KC112914	g	g	a	a	c	g	a	c	t	t	c	c	t	a	t	t	g	t	t	a	t	c	t	a	c	c	g	g	g	a
KP771914	g	g	a	a	c	g	a	c	t	t	c	c	t	a	t	t	g	t	t	a	t	c	t	a	c	c	g	g	g	a
KP702725	g	g	a	a	c	g	a	c	t	t	c	c	t	a	t	t	g	t	t	a	t	c	t	a	c	c	g	g	g	a
KP771890	g	g	a	a	c	g	a	c	t	t	c	c	t	a	t	t	g	t	t	a	t	c	t	a	t	a	g	g	g	a
JN704710	g	g	a	a	c	g	a	c	t	t	c	c	t	a	t	t	g	t	t	a	t	c	t	a	t	c	g	g	g	a
KY037798	g	g	a	a	c	g	a	c	t	t	c	c	t	a	t	t	g	t	t	a	t	c	t	a	t	c	g	g	g	a
MH709312	g	g	a	a	c	g	a	c	t	t	c	c	t	a	t	t	g	t	t	a	t	c	t	a	t	c	g	g	g	a
MH709352	g	g	a	a	c	g	a	c	t	t	c	c	t	a	t	t	g	t	t	a	t	c	t	a	t	c	g	g	g	a
AB097933	g	g	a	a	c	g	a	t	t	t	c	c	t	a	t	t	g	t	t	a	t	c	t	a	t	c	g	g	g	a
JN704698	g	g	a	a	c	g	a	t	t	t	c	c	t	a	t	t	g	t	t	a	t	c	t	a	t	c	g	g	g	a
JN704697	g	g	a	a	c	g	a	t	t	t	c	c	t	a	t	t	g	t	t	a	t	c	t	a	t	c	g	g	g	a
DQ008355	g	g	a	a	c	g	a	t	t	t	c	c	t	a	t	t	g	t	t	a	t	c	t	a	t	c	g	g	g	a
JF306641	g	g	a	a	c	g	a	t	t	t	c	c	t	a	t	t	g	t	t	a	t	c	t	a	t	c	g	g	g	a
AB097932	g	g	a	a	c	g	a	t	t	t	c	c	t	a	t	t	g	t	t	a	t	c	t	a	t	c	g	g	g	a
DQ008354	g	g	a	a	c	g	a	t	t	t	c	c	t	a	t	t	g	t	t	a	t	c	t	a	t	c	g	g	g	a
KU926312	g	g	a	a	c	g	a	t	t	t	c	c	t	a	t	t	g	t	t	a	t	c	t	a	t	c	g	g	g	a
KU926313	g	g	a	a	c	g	a	t	t	t	c	c	t	a	t	t	g	t	t	a	t	c	t	a	t	c	g	g	g	a
KU926314	g	g	a	a	c	g	a	t	t	t	c	c	t	a	t	t	g	t	t	a	t	c	t	a	t	c	g	g	g	a
KF558371	g	g	a	a	c	g	a	t	t	t	c	c	t	a	t	t	g	t	t	a	t	c	t	a	t	c	g	g	g	a
KF558372	g	g	a	a	c	g	a	t	t	t	c	c	t	a	t	t	g	t	t	a	c	c	t	a	t	c	g	g	g	a
KF558373	g	g	a	a	c	g	a	t	t	t	c	c	t	a	t	t	g	t	t	a	t	c	t	a	t	c	g	g	g	a
KF558374	g	g	a	a	c	g	a	t	t	t	c	c	t	a	t	t	g	t	t	a	t	c	t	a	t	c	g	g	g	a
KF558375	g	g	a	a	c	g	a	t	t	t	c	c	t	a	t	a	g	t	t	a	t	c	t	a	t	c	g	g	g	a
KF558376	g	g	a	a	c	g	a	t	t	t	c	c	t	a	t	t	g	t	t	a	t	c	t	a	t	c	g	g	g	a
KF558377	g	g	a	a	c	g	a	t	t	t	c	c	t	a	t	t	g	t	t	a	t	c	t	a	t	c	g	g	g	a
KF558378	g	g	a	a	c	g	a	t	t	t	c	c	t	a	t	t	g	t	t	a	c	c	t	a	t	c	g	g	g	a
KF558379	g	g	a	a	c	g	a	t	t	t	c	c	t	a	t	t	g	t	t	a	t	c	t	a	t	c	g	g	g	a
KF558380	g	g	a	a	c	g	a	t	t	t	c	c	t	a	t	t	g	t	t	a	t	c	t	a	t	c	g	g	g	a
KF558381	g	g	a	a	c	g	a	t	t	t	c	c	t	a	t	t	g	t	t	a	t	c	t	a	t	c	g	g	g	a
KF558382	g	g	a	a	c	g	a	t	t	t	c	c	t	a	t	t	g	t	t	a	t	c	t	a	t	c	g	g	g	a
KF558383	g	g	a	a	c	g	a	t	t	t	c	c	t	a	t	t	g	t	t	a	t	c	t	a	t	c	g	g	g	a
KF558384	g	g	a	a	c	g	a	t	t	t	c	c	t	a	t	t	g	t	t	a	t	c	t	a	t	c	g	g	g	a
KF558385	g	g	a	a	c	g	a	t	t	t	c	c	t	a	t	t	g	t	t	a	t	c	t	a	t	c	g	g	g	a
KF558386	g	g	a	a	c	g	a	t	t	t	c	c	t	a	t	t	g	t	t	a	t	c	t	a	t	c	g	g	g	a
KF558387	g	g	a	a	c	g	a	t	t	t	c	c	t	a	t	t	g	t	t	a	t	c	t	a	t	c	g	g	g	a
KF558388	g	g	a	a	c	g	a	t	t	t	c	c	t	a	t	t	g	t	t	a	t	c	t	a	t	c	g	g	g	a
KF558389	g	g	a	a	c	g	a	t	t	t	c	c	t	a	t	t	g	t	t	a	t	c	t	a	t	c	g	g	g	a
KF558390	g	g	a	a	c	g	a	t	t	t	c	c	t	a	t	t	g	t	t	a	t	c	t	a	t	c	g	g	g	a
KF558391	g	g	a	a	c	g	a	t	t	t	c	c	t	a	t	t	g	t	t	a	t	c	t	a	t	c	g	g	g	a
KF811485	g	g	a	a	c	g	a	t	t	t	c	c	t	a	t	t	g	t	t	a	t	c	t	a	t	c	g	g	g	a
KF853225	g	g	a	a	c	g	a	t	t	t	c	c	t	a	t	t	g	t	t	a	c	c	t	a	t	c	g	g	g	a
KF853226	g	g	a	a	c	g	a	t	t	t	c	c	t	a	t	t	g	t	t	a	c	c	t	a	t	c	g	g	g	a
KF853227	g	g	a	a	c	g	a	t	t	t	c	c	t	a	t	t	g	t	t	a	t	c	t	a	t	c	g	g	g	a
KF853228	g	g	a	a	c	g	a	t	t	t	c	c	t	a	t	t	g	t	t	a	t	c	t	a	t	c	g	g	g	a
KF853229	g	g	a	a	c	g	a	t	t	t	c	c	t	a	t	t	g	t	t	a	t	c	t	a	t	c	g	g	g	a
KF853230	g	g	a	a	c	g	a	t	t	t	c	c	t	a	t	t	g	t	t	a	t	c	t	a	t	c	g	g	g	a
KF853231	g	g	a	a	c	g	a	t	t	t	c	c	t	a	t	t	g	t	t	a	t	c	t	a	t	c	g	g	g	a
KF853232	g	g	a	a	c	g	a	t	t	t	c	c	t	a	t	t	g	t	t	a	t	c	t	a	t	c	g	g	g	a
KF853233	g	g	a	a	c	g	a	t	t	t	c	c	t	a	t	t	g	t	t	a	t	c	t	a	t	c	g	g	g	a
KF853234	g	g	a	a	c	g	a	t	t	t	c	c	t	a	t	t	g	t	t	a	c	c	t	a	t	c	g	g	g	a
KF853235	g	g	a	a	c	g	a	t	t	t	c	c	t	a	t	t	g	t	t	a	t	c	t	a	t	c	g	g	g	a
MF898328	g	g	a	a	c	g	a	t	t	t	c	c	t	a	t	t	g	t	t	a	t	c	t	a	t	c	g	g	g	a
JN704709	g	g	a	a	c	g	a	c	t	t	c	c	t	a	t	t	g	t	t	a	t	c	t	a	c	c	g	g	g	a

GB-ID											2																	
	9196	9243	9247	9251	9288	9289	9290	9404	9455	9459	9460	9470	9479	9488	9497	9503	9512	9524	9721	9814	9936	9954	10032	10043	10045	10069	10079	10119
MH499467	t	g	t	g	c	c	g	a	c	c	t	a	a	g	c	c	c	a	t	c	t	c	a	g	c	g	g	g
MH499469	t	g	t	g	c	c	g	a	c	c	t	a	a	g	c	c	c	a	t	c	t	c	a	g	c	g	g	g
MH709321	t	g	t	g	c	c	g	a	c	c	t	a	a	g	c	c	c	a	t	c	t	c	a	g	c	g	g	g
MH709324	t	g	t	g	c	c	g	a	c	c	t	a	a	g	c	c	c	a	t	c	t	c	a	g	c	g	g	g
MH709325	t	g	t	g	c	c	g	a	c	c	t	a	a	g	c	c	c	a	t	c	t	c	a	g	c	g	g	g
MH709327	t	g	t	g	c	c	g	a	c	c	t	a	a	g	c	c	c	a	t	c	t	c	a	g	c	g	g	g
MH709328	t	g	t	g	c	c	g	a	c	c	t	a	a	g	c	c	c	a	t	c	t	c	a	g	c	g	g	g
MH709330	t	g	t	g	c	c	g	a	c	c	t	a	a	g	c	c	c	a	t	c	t	c	a	g	c	g	g	g
MH709332	t	g	t	g	c	c	g	a	c	c	t	a	a	g	c	c	c	a	t	c	t	c	a	g	c	g	g	g
MH709334	t	g	t	g	c	c	g	a	c	c	t	a	a	g	c	c	c	a	t	c	t	c	a	g	c	g	g	g
MH709335	t	g	t	g	c	c	g	a	c	c	t	a	a	g	c	c	c	a	t	c	t	c	a	g	c	g	g	g
MH709336	t	g	t	g	c	c	g	a	c	c	t	a	a	g	c	c	c	a	t	c	t	c	a	g	c	g	g	g
MH709338	t	g	t	g	c	c	g	a	c	c	t	a	a	g	c	c	c	a	t	c	t	c	a	g	c	g	g	g
MH709340	t	g	t	g	c	c	g	a	c	c	t	a	a	g	c	c	c	a	t	c	t	c	a	g	c	g	g	g
MH709342	t	g	t	g	c	c	g	a	c	c	t	a	a	g	c	c	c	a	t	c	t	c	a	g	c	g	g	g
MH709343	t	g	t	g	c	c	g	a	c	c	t	a	a	g	c	c	c	a	t	c	t	c	a	g	c	g	g	g
MH709345	t	g	t	g	c	c	g	a	c	c	t	a	a	g	c	c	c	a	t	c	t	c	a	g	c	g	g	g
MH709347	t	g	t	g	c	c	g	a	c	c	t	a	a	g	c	c	c	a	t	c	t	c	a	g	c	g	g	g
MH709349	t	g	t	g	c	c	g	a	c	c	t	a	a	g	c	c	c	a	t	c	t	c	a	g	c	g	g	a
MH709350	t	g	t	g	c	c	g	a	c	c	t	a	a	g	c	c	c	a	t	c	t	c	a	g	c	g	g	g
MH709351	t	g	t	g	c	c	g	a	c	c	t	a	a	g	c	c	c	a	t	c	t	c	a	g	c	g	g	g
MH709353	t	g	t	g	c	c	g	a	c	c	t	a	a	g	c	c	c	a	t	c	t	c	a	g	c	g	g	g
MH709358	t	g	t	g	c	c	g	a	c	c	t	a	a	g	c	c	c	a	t	c	t	c	a	g	c	g	g	g
MH709360	t	g	t	g	c	c	g	a	c	c	t	a	a	g	c	c	c	a	t	c	t	c	a	g	c	g	g	g
MH709362	t	g	t	g	c	c	g	a	c	c	t	a	a	g	c	c	c	a	t	c	t	c	a	g	c	g	g	g
MH709365	t	g	t	g	c	c	g	a	c	c	t	a	a	g	c	c	c	a	t	c	t	c	a	g	c	g	g	g
MH709373	t	g	t	g	c	c	g	a	c	c	t	a	a	g	c	c	c	a	t	c	t	c	a	g	c	g	g	g
MH709374	t	g	t	g	c	c	g	a	c	c	t	a	a	g	c	c	c	a	t	c	t	c	a	g	c	g	g	g
KC112914	t	g	t	g	c	c	g	a	c	c	t	a	a	g	c	c	c	a	t	c	t	c	a	g	c	g	a	g
KP771914	t	g	t	g	c	c	g	a	c	c	t	a	a	g	c	c	c	a	t	c	t	c	a	g	c	g	a	g
KP702725	t	g	t	g	c	c	g	a	c	c	t	a	a	g	c	c	c	a	t	c	t	c	a	g	c	g	a	g
KP771890	t	g	t	g	c	c	g	a	c	c	t	a	a	g	c	c	c	a	c	c	t	c	a	g	c	g	g	g
JN704710	t	g	t	g	c	c	g	a	c	c	t	a	a	g	c	c	c	a	t	c	t	c	a	g	t	g	g	g
KY037798	t	g	t	g	c	c	g	a	c	c	t	a	a	g	c	c	c	a	c	c	t	c	a	g	c	g	g	g
MH709312	t	g	t	g	c	c	g	a	c	c	t	a	a	g	c	c	c	a	c	c	t	c	a	g	c	g	g	g
MH709352	t	g	t	g	c	c	g	a	c	c	t	a	a	g	c	c	c	a	c	c	t	c	a	g	c	g	g	g
AB097933	t	g	t	g	c	c	g	a	c	c	c	a	a	g	c	c	c	a	t	c	t	c	a	g	c	g	a	g
JN704698	t	g	t	g	c	c	g	a	c	c	c	a	a	g	c	c	c	a	t	c	t	c	a	g	c	g	a	g
JN704697	t	g	t	g	c	c	g	a	c	c	c	a	a	g	c	c	c	a	t	c	t	c	a	g	c	g	a	g
DQ008355	t	g	t	g	c	c	g	a	c	c	c	a	a	g	c	c	c	a	t	c	t	c	a	g	c	g	a	g
JF306641	t	g	t	g	c	c	g	a	c	c	c	a	a	g	c	c	c	a	t	c	t	c	a	g	c	g	a	g
AB097932	t	g	t	g	c	c	g	a	c	c	c	a	a	g	c	c	c	a	t	c	t	c	a	g	c	g	a	g
DQ008354	t	g	t	g	c	c	g	a	c	c	c	a	a	g	c	c	c	a	t	c	t	c	a	g	c	g	a	g
KU926312	t	g	t	g	c	c	g	a	c	c	c	a	a	g	c	c	c	a	t	c	t	c	a	g	c	g	a	g
KU926313	t	g	t	g	c	c	g	a	c	c	c	a	a	g	c	c	c	a	t	c	t	c	a	g	c	g	a	g
KU926314	t	g	t	g	c	c	g	a	c	c	c	a	a	g	c	c	c	a	t	c	t	c	a	g	c	g	a	g
KF558371	t	g	t	g	c	c	g	a	c	c	c	a	a	g	t	t	t	a	t	c	t	c	a	g	c	g	a	g
KF558372	t	g	t	g	c	c	g	a	c	c	c	a	a	g	c	c	c	a	t	c	t	c	a	g	c	g	a	g
KF558373	t	g	t	g	c	c	g	a	c	c	c	a	a	g	c	c	c	a	t	c	t	c	a	g	c	g	a	g
KF558374	t	g	t	g	c	c	g	a	c	c	c	a	a	g	c	c	c	a	t	c	t	c	a	g	c	g	a	g
KF558375	t	g	t	g	c	c	g	a	c	c	c	a	a	g	c	c	c	a	t	c	t	c	a	g	c	g	a	g
KF558376	t	g	t	g	c	c	g	a	c	c	c	a	a	g	c	c	c	a	t	c	t	c	a	g	c	g	a	g
KF558377	t	g	t	g	c	c	g	a	c	c	c	a	a	g	c	c	c	a	t	c	t	c	a	g	c	g	a	g
KF558378	t	g	t	g	c	c	g	a	c	c	c	a	a	g	c	c	c	a	t	c	t	c	a	g	c	g	a	g
KF558379	t	g	t	g	c	c	g	a	c	c	c	a	a	g	c	c	c	a	t	c	t	c	a	g	c	g	a	g
KF558380	t	g	t	g	c	c	g	a	c	c	c	a	a	g	c	c	c	a	t	c	t	c	a	g	c	g	a	g
KF558381	t	g	t	g	c	c	g	a	c	c	c	a	a	g	c	c	c	a	t	c	t	c	a	g	c	g	a	g
KF558382	t	g	t	g	c	c	g	a	c	c	c	a	a	g	c	c	c	a	t	c	t	c	a	g	c	g	a	g
KF558383	t	g	t	g	c	c	g	a	c	c	c	a	a	g	c	c	c	a	t	c	t	c	a	g	c	g	a	g
KF558384	t	g	t	g	c	c	g	a	c	c	c	a	a	g	c	c	c	a	t	c	t	c	a	g	c	g	a	g
KF558385	t	g	t	g	c	c	g	a	c	c	c	a	a	g	c	c	c	a	t	c	t	c	a	g	c	g	a	g
KF558386	t	g	t	g	c	c	g	a	c	c	c	a	a	g	c	c	c	a	t	c	t	c	a	g	c	g	a	g
KF558387	t	g	t	g	c	c	g	a	c	c	c	a	a	g	c	c	c	a	t	c	t	c	a	g	c	g	a	g
KF558388	t	g	t	g	c	c	g	a	c	c	c	a	a	g	c	c	c	a	t	c	t	c	a	g	c	g	a	g
KF558389	t	g	t	g	c	c	g	a	c	c	c	a	a	g	c	c	c	a	t	c	t	c	a	g	c	g	a	g
KF558390	t	g	t	g	c	c	g	a	c	c	c	a	a	g	c	c	c	a	t	c	t	c	a	g	c	g	a	g
KF558391	t	g	t	g	c	c	g	a	c	c	c	a	a	g	c	c	c	a	t	c	t	c	a	g	c	g	a	g
KF811485	t	g	t	g	c	c	g	a	c	c	c	a	a	g	c	c	c	a	t	c	t	c	a	g	c	g	a	g
KF853225	t	g	t	g	c	c	g	a	c	c	c	a	a	g	c	c	c	a	t	c	t	c	a	g	c	g	a	g
KF853226	t	g	t	g	c	c	g	a	c	c	c	a	a	g	c	c	c	a	t	c	t	c	a	g	c	g	a	g
KF853227	t	g	t	g	c	c	g	a	c	c	c	a	a	g	c	c	c	a	t	c	t	c	a	g	c	g	a	g
KF853228	t	g	t	g	c	c	g	a	c	c	c	a	a	g	c	c	c	a	t	c	t	c	a	g	c	g	a	g
KF853229	t	g	t	g	c	c	g	a	c	c	c	a	a	g	c	c	c	a	t	c	t	c	a	g	c	g	a	g
KF853230	t	g	t	g	c	c	g	a	c	c	c	a	a	g	c	c	c	a	t	c	t	c	a	g	c	g	a	g
KF853231	t	g	t	g	c	c	g	a	c	c	c	a	a	g	c	c	c	a	t	c	t	c	a	g	c	g	a	g
KF853232	t	g	t	g	c	c	g	a	c	c	c	a	a	g	c	c	c	a	t	c	t	c	a	g	c	g	a	g
KF853233	t	g	t	g	c	c	g	a	c	c	c	a	a	g	c	c	c	a	t	c	t	c	a	g	c	g	a	g
KF853234	t	g	t	g	c	c	g	a	c	c	c	a	a	g	c	c	c	a	t	c	t	c	a	g	c	g	a	g
KF853235	t	g	t	g	c	c	g	a	c	c	c	a	a	g	c	c	c	a	t	c	t	c	a	g	c	g	a	g
MF898328	t	g	t	g	c	c	g	a	c	c	c	a	a	g	c	c	c	a	t	c	t	c	a	g	c	g	a	g
JN704709	t	g	t	g	c	c	g	a	c	c	t	a	a	g	c	c	c	a	t	t	t	c	a	g	c	g	a	g

Supplementary Table 3: 2880 SN

GB-ID	Clade markers																		9		4			5																																																				
	Position in Dumas	10133	10165	10176	10188	10340	10344	10536	10540	10608	10654	10727	10818	10900	10946	10957	11044	11140	11149	11185	11317	11340	11361	11449	11527	11554	11725	11749	11801	11848	11861	11890	11894	11906	11934	11984	12020	12119	12140	12188	12268	12284	12285	12320	12528	12588	12612	12680	12779	12837	12847	12900	12933	12975	13173	13182	13274	13275	13298	13344	13401	13407	13458	13475	13486	13539	13585	13665	13667	13669	13739	13827	13846	13861	13870
AY548170	1	t	a	g	t	g	a	a	g	a	a	c	t	t	c	t	t	c	t	a	g	c	g	a	g	t	c	c	g	a	c	t	t	a	t	c	c	a	a	c	g	t	t	c	c	a	a	a	c	g	c	c	g	c	t	c	t	t	g	g	g	g	g	t	c	c	t	c	c	c	a	c	g	c	c
AY548171	1	t	a	g	t	g	a	a	g	a	a	c	t	t	c	t	t	c	t	a	g	c	g	a	g	t	c	c	g	a	c	t	t	a	t	c	c	a	a	c	g	t	t	c	c	a	a	a	c	g	c	c	g	t	t	c	t	t	g	g	g	g	t	t	c	c	t	c	c	c	a	c	g	c	c
DQ479953	1	t	a	g	t	g	a	a	g	a	a	c	t	t	c	t	t	c	t	a	g	c	g	a	g	t	c	c	g	a	c	t	t	a	t	c	c	a	a	c	g	t	t	c	c	a	a	a	c	g	c	c	g	t	t	c	t	t	g	g	g	g	g	t	c	c	t	c	c	c	a	c	g	c	c
DQ479954	1	t	a	g	t	g	a	a	g	a	a	c	t	t	c	t	t	c	t	a	g	c	g	a	g	t	c	c	g	a	c	t	t	a	t	c	c	a	a	c	g	t	t	c	c	a	a	a	c	g	c	c	g	t	t	c	t	t	g	g	g	g	g	t	c	c	t	c	c	c	a	c	g	c	t
DQ479958	1	t	a	g	t	g	a	a	g	a	a	c	t	t	c	t	t	c	t	a	g	c	g	a	g	t	c	c	g	a	c	t	t	a	t	c	c	a	a	c	g	t	t	c	c	a	a	a	c	g	c	c	g	t	t	c	t	t	g	g	g	g	g	t	c	c	t	c	c	c	a	c	g	c	c
DQ479959	1	t	a	g	t	g	a	a	g	a	a	c	t	t	c	t	t	c	t	a	g	c	g	a	g	t	c	c	g	a	c	t	t	a	t	c	c	a	a	c	g	t	t	c	c	a	a	a	c	g	c	c	g	t	t	c	t	t	g	g	g	g	g	t	c	c	t	c	c	c	a	c	g	c	c
DQ479961	1	t	a	g	t	g	a	a	g	a	a	c	t	t	c	t	t	c	t	a	g	c	g	a	g	t	c	c	g	a	c	t	t	a	t	c	c	a	a	c	g	t	t	c	c	a	a	a	c	g	c	c	g	t	t	c	t	t	g	g	g	g	g	t	c	c	t	c	c	c	a	c	g	c	t
DQ479962	1	t	a	g	t	g	a	a	g	a	a	c	t	t	c	t	t	c	t	a	g	c	g	a	g	t	c	c	g	a	c	t	t	a	t	c	c	a	a	c	g	t	t	c	c	a	a	a	c	g	c	c	g	t	t	c	t	t	g	g	g	g	g	t	c	c	t	c	c	c	a	c	g	c	c
DQ479963	1	t	a	g	t	g	a	a	g	a	a	c	t	t	c	t	t	c	t	a	g	c	g	a	g	t	c	c	g	a	c	t	t	a	t	c	c	a	a	c	g	t	t	c	c	a	a	a	c	g	c	c	g	t	t	c	t	t	g	g	g	g	g	t	c	c	t	c	c	c	a	c	g	c	t
DQ674250	1	t	a	g	t	g	a	a	g	a	a	c	t	t	c	t	t	c	t	a	g	c	g	a	g	t	c	c	g	a	c	t	t	a	t	c	c	a	a	c	g	t	t	c	c	a	a	a	c	g	c	c	g	t	t	c	t	t	g	g	g	g	g	t	c	c	t	c	c	c	a	c	g	c	c
KX262864	1	t	a	g	t	g	a	a	g	a	a	c	t	t	c	t	t	c	t	a	g	c	g	a	g	t	c	c	g	a	c	t	t	a	t	c	c	a	a	c	g	t	t	c	c	a	a	a	c	g	c	c	g	t	t	c	t	t	g	g	g	g	g	t	c	c	t	c	c	c	a	c	g	c	c
KX262865	1	t	a	g	t	g	a	a	g	a	a	c	t	t	c	t	t	c	t	a	g	c	g	a	g	t	c	c	g	a	c	t	t	a	t	c	c	a	a	c	g	t	t	c	c	a	a	a	c	g	c	c	g	t	t	c	t	t	g	g	g	g	g	t	c	c	t	c	c	c	a	c	g	c	c
KX262866	1	t	a	g	t	g	a	a	g	a	a	c	t	t	c	t	t	c	t	a	g	c	g	a	g	t	c	c	g	a	c	t	t	a	t	c	c	a	a	c	g	t	t	c	c	a	a	a	c	g	c	c	g	t	t	c	t	t	g	g	g	g	g	t	c	c	t	t	c	t	a	c	g	c	c
EU154348	1	t	a	g	t	g	a	a	g	a	a	c	t	t	c	t	t	c	t	a	g	c	g	a	g	t	c	c	g	a	c	t	t	a	t	c	c	a	a	c	g	t	t	c	c	a	a	a	c	g	c	c	g	t	t	c	t	t	g	g	g	g	g	t	c	c	t	c	c	c	a	c	g	c	t
JN704690	1	t	a	g	t	g	a	a	g	a	a	c	t	t	c	t	t	c	t	a	g	c	g	a	g	t	c	c	g	a	c	t	t	a	t	c	c	a	a	c	g	t	t	c	c	a	a	a	c	g	c	c	g	t	t	c	t	t	g	g	g	g	g	t	c	c	t	c	c	c	a	c	g	c	c
JN704691	1	t	a	g	t	g	a	a	g	a	a	c	t	t	c	t	t	c	t	a	g	c	g	a	g	t	c	c	g	a	c	t	t	a	t	c	c	a	a	c	g	t	t	c	c	a	a	a	c	g	c	c	g	t	t	c	t	t	g	g	g	g	g	t	c	c	t	c	c	c	a	c	g	c	c
JN704692	1	t	a	g	t	g	a	a	g	a	a	c	t	t	c	t	t	c	t	g	g	c	g	a	g	t	c	c	g	a	c	t	t	a	t	c	c	a	a	c	g	t	t	c	c	a	a	a	c	g	c	c	g	t	t	c	t	t	g	g	g	g	g	t	c	c	t	c	c	c	a	c	g	c	c
JN704693	1	t	a	g	t	g	a	a	g	a	a	c	t	t	c	t	t	c	t	a	g	c	g	a	g	t	c	c	g	a	c	t	a	a	t	c	c	a	a	c	g	t	t	c	c	a	a	a	c	g	c	c	g	t	t	c	t	t	g	g	g	g	g	t	c	c	t	c	c	c	a	c	g	c	c
JN704694	1	t	a	g	t	g	a	a	g	a	a	t	t	t	c	t	t	c	t	a	g	c	g	a	g	t	c	c	g	a	c	t	t	a	t	c	c	a	a	c	g	t	t	c	c	a	a	a	c	g	c	c	g	t	t	c	t	t	g	g	g	g	g	t	c	c	t	c	c	c	a	c	g	c	c
JN704695	1	t	a	g	t	g	a	a	g	a	a	c	t	t	c	t	t	c	t	a	g	c	g	a	g	t	c	c	g	a	c	t	t	a	t	c	c	a	a	c	g	t	t	c	c	a	a	a	c	g	c	c	g	t	t	c	t	t	g	g	g	g	g	t	c	c	t	c	c	c	a	c	g	c	c
JN704696	1	t	a	g	t	g	a	a	g	a	a	c	t	t	c	t	t	c	t	a	g	c	g	a	g	t	c	c	g	a	c	t	t	a	t	c	c	a	a	c	g	t	t	c	c	a	a	a	c	g	c	c	g	t	t	c	t	t	g	g	g	g	g	t	c	c	t	c	c	c	a	c	g	c	c
NC001348	1	t	a	g	t	g	a	a	g	a	a	c	t	t	c	t	t	c	t	a	g	c	g	a	g	t	c	c	g	a	c	t	t	a	t	c	c	a	a	c	g	t	t	c	c	a	a	a	c	g	c	c	g	t	t	c	t	t	g	g	g	g	g	t	c	c	t	c	c	c	a	c	g	c	c
KP771892	1	t	a	g	t	g	a	a	g	a	a	c	t	t	c	t	t	c	t	a	g	c	g	a	g	t	c	c	g	a	c	t	t	a	t	c	c	a	a	c	g	t	t	c	c	a	a	a	c	g	c	c	g	t	t	c	t	t	g	g	g	g	g	t	c	c	t	c	c	c	a	c	g	c	c
KP771894	1	t	a	g	t	g	a	a	g	a	a	c	t	t	c	t	t	c	t	a	g	c	g	a	g	t	c	c	g	a	c	t	t	a	t	c	c	a	a	c	g	t	t	c	c	a	a	a	c	g	c	c	g	t	t	c	t	t	g	g	g	g	g	t	c	c	t	c	c	c	a	c	g	c	c
KP771900	1	t	a	g	t	g	a	a	g	a	a	c	t	t	c	t	t	c	t	a	g	c	g	a	g	t	c	c	g	a	c	t	t	a	t	c	c	a	a	c	g	t	t	c	c	a	a	a	c	g	c	c	g	t	t	c	t	t	g	g	g	g	g	t	c	c	t	c	c	c	a	c	g	c	c
KP771901	1	t	a	g	t	g	a	a	g	a	a	c	t	t	c	t	t	c	t	a	g	c	g	a	g	t	c	c	g	a	c	t	t	a	t	c	c	a	a	c	g	t	t	c	c	a	a	a	c	g	c	c	g	t	t	c	t	t	g	g	g	g	g	t	c	c	t	c	c	c	a	a	g	c	c
KP771902	1	t	a	g	t	g	a	a	g	a	a	c	t	t	c	t	t	c	t	a	g	c	g	a	g	t	c	c	g	a	c	t	t	a	t	c	c	a	a	c	g	t	t	c	c	a	a	a	c	g	c	c	g	t	t	c	t	t	g	g	g	g	g	t	c	c	t	c	c	c	a	c	g	c	c
KP771908	1	t	a	g	t	g	a	a	g	a	a	c	t	t	c	t	t	c	t	a	g	c	g	a	g	t	c	c	g	a	c	t	t	a	t	c	c	a	a	c	g	t	t	c	c	a	a	a	c	g	c	c	g	t	t	c	t	t	g	g	g	g	g	t	c	c	t	c	c	c	a	c	t	c	c
KP771912	1	t	a	g	t	g	a	a	g	a	a	c	t	t	c	t	t	c	t	a	g	c	g	a	g	t	c	c	g	a	c	t	t	a	t	c	c	a	a	c	g	t	t	c	c	a	a	a	c	g	c	c	g	t	t	c	t	t	g	g	g	g	g	t	c	c	t	c	c	c	a	c	g	c	c
KP771913	1	t	a	g	t	g	a	a	g	a	a	c	t	t	c	t	t	c	t	a	g	c	g	a	g	t	c	c	g	a	c	t	t	a	t	c	c	a	a	c	g	t	t	c	c	a	a	a	c	g	c	c	g	t	t	c	t	t	g	g	g	g	g	t	c	c	t	c	c	c	a	c	g	c	c
KP771917	1	t	a	g	t	g	a	a	g	a	a	c	t	t	c	t	t	c	t	a	g	c	g	a	g	t	c	c	g	a	c	t	t	a	t	c	c	a	a	c	g	t	t	c	c	a	a	a	c	g	c	c	g	t	t	c	t	t	g	g	g	g	g	t	c	c	t	c	c	c	a	c	g	c	c
KP771918	1	t	a	g	t	g	a	a	g	a	a	c	t	t	c	t	t	c	t	a	g	c	g	a	g	t	c	c	g	a	c	t	t	a	t	c	c	a	a	c	g	t	t	c	c	a	a	a	c	g	c	c	g	t	t	c	t	t	a	g	g	g	g	t	c	c	t	c	c	c	a	c	g	c	c
KP771920	1	t	a	g	t	g	a	a	g	a	a	c	t	t	c	t	t	c	t	a	g	c	g	a	g	t	c	c	g	a	c	t	t	a	t	c	c	a	a	c	g	t	t	c	c	a	a	a	c	g	c	c	g	t	t	c	t	t	g	g	g	g	g	t	c	c	t	c	c	c	a	c	g	c	c
MH709361	1	t	a	g	t	g	a	a	g	a	a	c	t	t	c	t	t	c	t	a	g	c	g	a	t	t	c	c	g	a	c	t	t	a	t	c	c	a	a	c	g	t	t	c	c	a	a	a	c	g	c	c	g	t	t	c	t	t	g	g	g	g	g	t	c	t	t	c	c	c	a	c	g	c	c
KU926318	2	t	a	g	t	g	g	a	g	a	a	c	t	t	c	t	t	c	t	g	g	c	g	a	g	t	c	c	g	a	c	g	t	g	t	c	c	a	a	a	g	c	c	c	c	a	g	a	c	g	c	c	g	t	g	c	t	t	g	g	g	a	g	t	c	c	t	c	c	c	a	c	g	c	c
KU926319	2	t	a	g	t	g	a	a	g	a	a	c	t	t	c	t	t	c	t	a	g	c	g	a	g	t	c	c	g	a	c	g	t	g	t	c	c	a	a	a	g	c	c	c	c	a	a	a	c	g	c	c	g	t	g	c	t	t	g	g	g	a	g	t	c	c	t	c	t	c	a	c	g	c	c
KU926320	2	t	a	g	t	g	a	a	g	a	a	c	t	t	c	t	t	c	t	a	g	c	g	a	g	t	c	c	g	a	c	g	t	g	t	c	c	a	a	a	g	c	c	c	c	a	a	g	c	g	c	c	g	t	g	c	t	t	g	g	g	a	g	t	c	c	t	c	t	c	a	c	g	c	c
KU926321	2	t	a	g	t	g	a	a	g	a	a	c	t	t	c	t	t	c	t	a	g	c	g	a	g	t	c	c	g	a	c	g	t	g	t	c	c	a	a	a	g	c	c	c	c	a	a	a	c	g	c	c	g	t	g	c	t	t	g	g	g	a	g	t	c	c	t	c	c	c	a	c	g	c	c
KU926322	2	t	a	g	t	g	a	a	g	a	a	c	t	t	c	t	t	c	t	a	g	c	g	a	g	t	c	c	g	a	c	g	t	g	t	c	c	a	a	a	g	c	c	c	c	a	a	a	c	g	c	c	g	t	g	c	t	t	g	g	g	a	g	t	c	c	t	c	c	c	a	c	g	c	c
KC847290	2	t	a	g	t	g	a	a	g	a	a	c	t	t	c	t	t	c	t	a	g	c	g	a	g	t	c	c	g	a	c	g	t	g	t	c	c	a	a	a	g	c	c	c	c	a	a	a	c	g	c	c	g	t	g	c	t	t	g	g	g	a	g	t	c	c	a	c	c	c	a	c	g	c	c
KJ767491	2	t	a	g	t	g	a	a	g	a	a	c	t	t	c	t	t	c	t	a	g	c	g	a	g	t	c	c	g	a	c	g	t	g	t	c	c	a	a	a	g	c	c	c	c	a	a	a	c	g	c	c	g	t	g	c	t	t	g	g	g	a	g	t	c	c	t	c	c	c	a	c	g	c	c
KJ767492	2	t	a	g	t	g	a	a	g	a	a	c	t	t	c	t	t	c	t	a	g	c	g	a	g	t	c	c	g	a	c	g	t	g	t	c	c	a	a	a	g	c	c	c	c	a	a	a	c	g	c	c	g	t	g	c	t	t	g	g	g	a	g	t	c	c	t	c	t	c	a	c	g	c	c
KJ808816	2	t	a	g	t	g	a	a	g	a	a	c	t	t	c	t	t	c	t	a	g	c	g	a	g	t	c	c	g	a	c	g	t	g	t	c	c	a	a	a	g	c	c	c	c	a	a	a	c	g	c	c	g	t	g	c	t	t	g	g	g	a	g	t	c	c	t	c	c	c	a	c	g	c	c
KP771925	2	t	a	g	t	g	a	a	g	a	a	c	t	t	c	t	t	c	t	a	g	c	a	a	g	t	c	c	g	a	c	g	t	g	t	c	c	a	a	a	g	c	c	c	c	a	a	a	c	g	c	c	g	t	g	c	t	t	g	g	g	a	g	t	c	c	t	c	c	c	a	c	g	c	c
KU926315	2	t	a	g	t	g	a	a	g	a	a	c	t	t	c	t	t	c	t	a	g	c	g	a	g	t	c	c	g	a	c	g	t	g	t	c	c	a	a	a	g	c	c	c	c	a	a	a	c	g	c	c	g	t	g	c	t	t	g	g	g	a	g	t	c	c	t	c	c	c	a	c	g	c	c
KU926316	2	t	a	g	t	g	a	a	g	a	a	c	t	t	c	t	t	c	t	a	g	c	g	a	g	t	c	c	g	a	c	g	t	g	t	c	c	a	a	a	g	c	c	c	c	a	a	a	c	g	c	c	g	t	g	c	t	t	g	g	g	a	g	t	c	c	t	c	c	c	a	c	g	c	c
KU926317	2	t	a	g	t	g	a	a	g	a	a	c	t	t	c	t	t	c	t	a	g	c	g	a	g	t	c	c	g	a	c	g	t	g	t	c	c	a	a	a	g	c	c	c	c	a	a	a	c	g	c	c	g	t	g	c	t	t	g	g	g	a	g	t	c	c	t	c	c	c	a	c	g	c	c
KY037796	2	t	a	g	t	g	a	a	g	a	a	c	t	t	c	t	t	c	t	a	g	c	g	a	g	t	c	c	g	a	c	g	t	g	t	c	c	a	a	a	g	c	c	c	c	a	a	a	c	g	c	c	g	t	g	c	t	t	g	g	g	a	g	t	c	c	t	c	c	c	a	c	g	c	c
MF004348	2	t	a	g	t	g	a	a	g	a	a	c	t	t	c	t	t	c	t	a	g	c	g	a	g	t	c	c	g	a	c	g	t	g	t	c	c	a	a	a	g	c	c	c	c	a	a	a	c	g	c	c	g	t	g	c	t	t	g	g	g	a	g	t	c	c	t	c	t	c	a	c	g	c	c
MH709309	2	t	a	g	t	g	a	a	g	a	a	c	t	t	c	t	t	c	t	a	g	c	g	a	g	t	c	c	g	a	c	g	t	g	t	c	c	a	a	a	g	c	c	c	c	a	a	a	c	g	c	c	g	t	g	c	t	t	g	g	g	a	g	t	c	c	t	c	c	c	a	c	g	c	c
MH709310	2	t	a	g	t	g	a	a	g	a	a	c	t	t	c	t	t	c	t	a	g	c	g	a	g	t	c	c	g	a	c	g	t	g	t	c	c	a	a	a	g	c	c	c	c	a	a	a	c	g	c	c	g	t	g	c	t	t	g	g	g	a	g	t	c	c	t	c	c	c	c	c	g	c	c
MH709311	2	t	a	g	t	g	a	a	g	a	a	c	t	t	c	t	t	c	t	a	g	c	g	a	g	t	c	c	g	a	c	g	t	g	t	c	c	a	a	a	g	c	c	c	c	a	a	a	c	g	c	c	g	t	g	c	t	t	g	g	g	a	g	t	c	c	t	c	c	c	a	c	g	c	c
MH709313	2	t	a	g	t	g	a	a	g	a	a	c	t	t	c	t	t	c	t	a	g	t	g	a	g	t	c	c	g	a	c	g	t	g	t	c	c	a	a	a	g	c	c	c	a	a	a	a	c	g	c	c	t	t	g	c	t	t	g	g	g	a	g	t	c	c	t	c	c	c	a	c	g	c	c
MH709314	2	t	a	g	t	g	a	a	g	a	a	c	t	t	c	t	t	c	t	a	g	c	g	a	g	t	c	c	g	a	c	g	t	g	t	c	c	a	a	a	g	c	c	c	c	a	a	a	c	g	c	c	g	t	g	c	t	t	g	g	g	a	g	t	c	c	t	c	c	c	a	c	g	c	c
MH709315	2	t	a	g	t	g	a	a	g	a	a	c	t	t	c	t	t	c	t	a	g	c	g	a	g	t	c	c	g	a	c	g	t	g	t	c	c	a	a	a	g	c	c	c	c	a	a	a	c	g	c	c	g	t	g	c	t	t	g	g	g	a	g	t	c	c	t	c	c	c	a	c	g	c	c
MH709319	2	t	a	g	t	g	a	a	g	a	a	c	t	t	c	t	t	c	t	a	g	c	g	a	g	t	c	c	g	a	c	g	t	g	t	c	c	a	a	a	g	c	c	c	c	a	a	a	c	g	c	c	g	t	g	c	t	t	g	g	g	a	g	t	c	c	t	c	c	c	a	c	g	c	c
MH709320	2	t	a	g	t	g	a	a	g	a	a	c	t	t	c	t	t	c	t	a	g	c	g	a	g	t	c	c	g	a	c	g	t	g	t	c	c	a	a	a	g	c	c	c	c	a	a	a	c	g	c	c	g	t	g	c	t	t	g	g	g	a	g	t	c	c	t	c	c	c	a	c	g	c	c
MH709329	2	t	a	g	t	g	a	a	g	a	a	c	t	t	c	t	t	c	t	a	g	c	g	a	g	t	c	c	g	a	c	g	t	g	t	c	c	a	a	a	g	c	c	c	c	a	a	a	c	g	c	c	g	t	g	c	t	t	g	g	g	a	g	t	c	c	t	c	c	c	a	c	g	c	c
MH709331	2	t	a	g	t	g	a	a	g	a	a	c	t	t	c	t	t	c	t	a	g	c	g	a	g	t	c	c	g	a	c	g	t	g	t	c	c	a	a	a	g	c	c	c	c	a	a	a	c	g	c	c	g	t	g	c	t	t	g	g	g	a	g	t	c	c	t	c	c	c	a	c	g	c	c
MH709333	2	t	a	g	t	g	a	a	g	a	a	c	t	t	c	t	t	c	t	a	g	c	g	a	g	t	c	c	g	a	c	g	t	g	t	c	c	a	a	a	g	c	c	c	c	a	a	a	c	g	c	c	g	t	g	c	t	t	g	g	g	a	g	t	c	c	t	c	c	c	a	c	g	c	c
MH709337	2	t	a	g	t	g	a	a	g	a	a	c	t	t	c	t	t	c	t	a	g	c	g	a	g	t	c	c	g	a	c	g	t	g	t	c	c	a	a	a	g	c	c	c	c	a	a	a	c	g	c	c	g	t	g	c	t	t	g	g	g	a	g	t	c	c	t	c	c	c	a	c	g	c	c
MH709339	2	t	a	g	t	g	a	a	g	a	a	c	t	t	c	t	t	c	t	a	g	c	g	a	g	t	c	c	g	a	c	g	t	g	t	c	c	a	a	a	g	c	c	c	c	a	a	a	c	g	c	c	g	t	g	c	t	t	g	g	g	a	g	t	c	c	t	c	c	c	a	c	g	c	c
MH709344	2	t	a	g	t	g	a	a	g	a	a	c	t	t	c	t	t	c	t	a	g	c	g	a	g	t	c	c	g	a	c	g	t	g	g	c	c	a	a	a	g	c	c	c	c	a	a	a	c	g	c	c	g	t	g	c	t	t	g	g	g	a	g	t	c	c	t	c	c	c	a	c	g	c	c
MH709348	2	t	a	g	t	g	a	a	g	a	a	c	t	t	c	t	t	c	t	a	g	c	g	a	g	t	c	c	g	a	c	g	t	g	t	c	c	a	a	a	g	c	c	c	c	a	a	a	c	g	c	c	g	t	g	c	t	t	g	g	g	a	g	t	c	c	t	c	c	c	a	c	g	c	c
MH709355	2	t	a	g	t	g	a	a	g	a	a	c	t	t	c	t	t	c	t	a	g	c	g	a	g	t	c	c	g	a	c	g	t	g	t	c	c	a	a	a	g	c	c	c	c	a	a	a	c	g	c	c	g	t	g	c	t	t	g	g	g	a	g	t	c	c	t	c	c	c	a	c	g	c	c
MH709359	2	t	a	g	t	g	a	a	g	a	a	c	t	t	c	t	t	c	t	a	g	c	g	a	g	t	c	c	g	a	c	g	t	g	t	c	c	a	a	a	g	c	c	c	c	a	a	a	c	g	c	c	g	t	g	c	t	t	g	g	g	a	g	t	c	c	t	c	c	c	a	c	g	c	c
MH709363	2	t	a	g	t	g	a	a	g	a	a	c	t	t	c	t	t	c	t	a	g	c	g	a	g	t	c	c	g	a	c	g	t	g	t	c	c	a	a	a	g	c	c	c	c	a	a	a	c	g	c	c	g	t	g	c	t	t	g	g	g	a	g	t	c	c	t	c	c	c	a	c	g	c	c
MH709366	2	t	a	g	t	g	a	a	g	a	a	c	t	t	c	t	t	c	t	a	g	c	g	a	g	t	c	c	g	a	c	g	t	g	t	c	c	a	a	a	g	c	c	c	c	a	a	a	c	g	c	c	g	t	g	c	t	t	g	g	g	a	g	t	c	c	t	c	c	c	a	c	g	c	c
MH709376	2	t	a	g	t	g	a	a	g	a	a	c	t	t	c	t	t	c	t	a	g	c	g	a	g	t	c	c	g	a	c	g	t	g	t	c	c	a	a	a	g	c	c	c	c	a	a	a	c	g	c	c	g	t	g	c	t	t	g	g	g	a	g	t	c	c	t	c	c	c	a	c	g	c	c
MH709377	2	t	a	g	t	g	a	a	g	a	a	c	t	t	c	t	t	c	t	a	g	c	g	a	g	t	c	c	g	a	c	g	t	g	t	c	t	a	a	a	g	c	c	c	c	a	a	a	c	g	c	c	g	t	g	c	t	t	g	g	g	a	g	t	c	c	t	c	c	c	a	c	g	c	c
**MW316406**	2	t	a	g	t	g	a	a	g	a	a	c	t	t	c	t	t	c	t	a	g	c	g	a	g	t	c	c	g	a	c	g	t	g	t	c	c	a	a	a	g	c	c	c	c	a	a	a	c	g	c	c	g	t	g	c	t	t	g	g	g	a	g	t	c	c	t	c	c	c	a	c	g	c	c
AJ871403	3	t	a	g	t	g	a	a	g	a	a	c	t	t	c	t	t	c	t	a	g	c	g	a	g	t	c	c	g	a	c	t	t	a	t	c	c	a	a	c	g	c	c	c	c	a	a	a	c	g	c	c	g	t	t	c	t	t	g	g	g	g	g	t	c	c	t	c	c	c	a	c	g	c	c
DQ479955	3	t	a	g	t	g	a	a	g	a	a	c	t	t	c	t	t	c	t	a	g	c	g	a	g	t	c	c	g	a	c	t	t	a	t	c	c	a	a	c	g	t	t	c	c	a	a	a	c	g	c	c	g	t	t	c	t	t	g	g	g	g	g	t	c	c	t	c	c	c	a	c	g	c	c
DQ479956	3	t	a	g	t	g	a	a	g	a	a	c	t	t	c	t	t	c	t	a	g	c	g	a	g	t	c	c	g	a	c	t	t	a	t	c	c	g	a	c	g	c	c	c	c	a	a	a	c	g	c	c	g	t	t	c	t	t	g	g	g	g	g	t	c	c	t	c	c	c	a	c	g	c	c
DQ479957	3	t	a	g	t	g	a	a	g	a	a	c	t	t	c	t	t	c	t	a	g	c	g	a	g	t	c	t	g	a	c	t	t	a	t	c	c	g	a	c	g	c	c	c	c	a	a	a	c	g	c	c	g	t	t	c	t	t	g	g	g	g	g	t	c	c	t	c	c	c	a	c	g	c	c
JN704699	3	t	a	g	t	g	a	a	g	a	a	c	t	t	c	t	t	c	t	a	g	c	g	a	a	t	c	c	g	a	c	t	t	a	t	c	c	a	a	c	g	c	c	c	c	a	a	a	c	g	c	c	g	t	t	c	t	t	g	g	g	g	g	t	c	c	t	c	c	c	a	c	g	c	c
JN704700	3	t	a	g	t	g	a	a	g	a	a	c	t	t	c	t	t	c	t	a	g	c	g	a	g	t	c	c	g	a	c	t	t	a	t	c	t	a	a	c	g	c	c	c	c	a	a	a	c	g	c	c	g	t	t	c	t	t	g	g	g	g	g	t	c	c	t	c	c	c	a	c	g	c	c
JN704701	3	t	a	g	t	g	a	a	g	a	a	c	t	t	c	t	t	c	t	a	g	c	g	a	g	t	c	c	g	a	c	t	t	a	t	c	c	a	a	c	g	c	c	c	c	a	a	a	c	g	c	c	g	t	t	c	t	t	g	g	g	g	g	t	c	c	t	c	c	c	a	c	g	c	c
JN704702	3	t	a	g	t	g	a	a	g	a	a	c	t	t	c	t	t	c	t	a	g	c	g	a	g	t	c	c	g	a	c	t	t	a	t	c	c	a	a	c	g	c	c	c	c	a	a	a	c	g	c	c	g	t	t	c	t	t	g	g	g	g	g	t	c	c	t	c	c	c	a	c	g	c	c
JN704703	3	t	a	g	t	g	a	a	g	a	a	c	t	t	c	t	t	c	t	a	g	c	g	a	g	t	c	c	g	a	c	t	t	a	t	c	c	a	a	c	g	c	c	c	c	a	a	a	c	g	c	c	g	t	t	c	t	t	g	g	g	g	g	t	c	c	t	c	c	c	a	c	g	c	c
KP771891	3	t	a	g	t	g	a	a	g	a	a	c	t	t	c	t	t	c	t	a	g	c	g	a	g	t	c	c	g	a	c	t	t	a	t	c	c	a	a	c	g	c	c	c	c	a	a	a	c	g	c	c	g	t	t	c	t	t	g	g	g	g	g	t	c	c	t	c	c	c	a	c	g	c	c
KP771893	3	t	a	g	t	g	a	a	g	a	a	c	t	t	c	t	t	c	t	a	g	c	g	a	g	t	c	c	g	a	c	t	t	a	t	c	c	a	a	c	g	c	c	c	c	a	a	a	c	g	c	c	g	t	t	c	t	t	g	g	g	g	g	t	c	c	t	c	c	c	a	c	g	c	c
KP771895	3	t	a	a	t	g	a	a	g	a	a	c	t	t	c	t	t	c	t	a	g	c	g	a	g	t	c	c	g	a	c	t	t	a	t	c	c	a	a	c	g	c	c	c	c	a	a	a	c	g	c	c	g	t	t	c	t	t	g	g	g	g	g	t	c	c	t	c	c	c	a	c	g	c	c
KP771896	3	t	a	g	t	g	a	a	g	a	a	c	t	t	c	t	g	c	t	a	g	c	g	a	g	t	c	c	g	a	c	t	t	a	t	c	c	a	a	c	g	c	c	c	c	a	a	a	c	a	c	c	g	t	t	c	t	t	g	g	g	g	g	t	c	c	t	c	c	c	a	c	g	c	c
KP771897	3	t	a	g	t	g	a	a	g	a	a	c	t	t	c	t	t	c	t	a	g	c	g	a	g	t	c	c	g	a	c	t	t	a	t	c	c	a	a	c	g	c	c	c	c	a	a	a	c	g	c	c	g	t	t	c	t	t	g	g	g	g	g	t	c	c	t	c	c	c	a	c	g	c	c
KP771899	3	t	a	g	t	g	a	a	g	a	a	c	t	t	c	t	t	c	t	a	g	c	g	a	g	t	c	c	g	a	c	t	t	a	t	c	c	a	a	c	g	c	c	c	c	a	a	a	c	g	c	c	g	t	t	c	t	t	g	g	g	g	g	t	c	c	t	c	c	c	a	c	g	c	c
KP771903	3	t	a	g	t	g	a	a	g	a	a	c	t	t	c	t	t	c	t	a	g	c	g	a	g	t	c	c	g	a	c	t	t	a	t	c	c	a	a	c	g	c	c	c	c	a	a	a	c	g	c	c	g	t	t	c	t	t	g	g	g	g	g	t	c	c	t	c	c	c	a	c	g	c	c
KP771907	3	t	a	g	t	g	a	a	g	a	a	c	t	t	c	t	t	c	t	a	g	c	g	a	g	t	c	c	g	a	c	t	t	a	t	c	c	a	a	c	g	t	c	c	c	a	a	a	c	g	c	c	g	t	t	c	t	t	g	g	g	g	g	t	c	c	t	c	c	c	a	c	g	c	c
KP771910	3	t	a	g	t	a	a	a	g	a	a	c	t	t	c	t	t	c	t	a	g	c	g	a	g	t	c	c	g	a	c	t	t	a	t	c	c	a	a	c	g	c	c	c	c	a	a	a	c	g	c	c	g	t	t	c	t	t	g	g	g	g	g	t	c	c	t	c	c	c	a	c	g	c	c
KP771915	3	t	a	g	t	g	a	a	g	a	a	c	t	t	c	t	t	c	t	a	g	c	g	a	g	t	c	c	g	a	c	t	t	a	t	c	c	a	a	c	g	c	c	c	c	a	a	a	c	g	c	c	g	t	t	c	t	t	g	g	g	g	g	t	c	c	t	c	c	c	a	c	g	c	c
KP771916	3	t	a	g	t	g	a	a	g	a	a	c	t	t	c	t	t	c	t	a	g	c	g	a	g	t	c	c	g	a	c	t	t	a	t	c	c	g	a	c	g	c	c	c	c	a	a	a	c	g	c	c	g	t	t	c	t	t	g	g	g	g	g	t	c	c	t	c	c	c	a	c	g	c	c
KP771921	3	t	a	g	t	g	a	a	g	a	a	c	t	t	c	t	t	c	t	a	g	c	g	a	g	t	c	c	g	a	c	t	t	a	t	c	c	a	a	c	g	c	c	c	c	a	a	a	c	g	c	c	g	t	t	c	t	t	g	g	g	g	g	t	c	c	t	c	c	c	a	c	g	c	c
KP771923	3	t	a	g	t	g	a	a	g	a	a	c	t	t	c	t	t	c	t	a	g	c	g	a	g	t	c	c	g	a	c	t	t	a	t	c	c	a	a	c	g	c	c	c	c	a	a	a	c	g	c	c	g	t	t	c	t	t	g	g	g	g	g	t	c	c	t	c	c	c	a	c	g	c	c
KP771889	3	t	a	g	t	g	a	a	g	a	a	c	t	t	c	t	t	c	t	a	g	c	g	a	g	t	c	c	g	a	c	t	t	a	t	c	c	a	a	c	g	t	t	c	c	a	a	a	c	g	c	c	g	t	t	c	t	t	g	g	g	g	g	t	c	c	t	c	c	c	a	c	g	c	c
KU926311	3	t	a	g	t	g	a	g	g	a	a	c	t	t	c	t	t	c	t	a	g	c	g	a	g	t	c	c	g	a	c	t	t	a	t	c	c	g	a	c	g	c	c	c	c	a	a	a	c	g	c	c	g	t	t	c	t	t	g	g	g	g	g	t	c	c	t	c	c	c	a	c	g	c	c
JQ911599	3	t	a	g	g	g	a	a	g	a	a	c	t	t	c	t	t	c	t	a	g	c	g	a	g	t	c	c	g	a	c	t	t	a	t	c	c	a	a	c	g	c	c	c	c	a	a	a	c	g	c	t	g	t	t	c	t	t	g	g	g	g	g	t	c	c	t	c	c	c	a	c	g	c	c
MH709317	3	t	a	g	t	g	a	a	g	a	a	c	t	t	c	t	t	c	t	a	g	c	g	a	g	t	c	c	g	a	c	t	t	a	t	c	c	a	a	c	g	c	c	t	c	a	a	a	c	g	c	c	g	t	t	c	t	t	g	g	g	g	g	t	c	c	t	c	c	c	a	c	g	c	c
MH709346	3	t	a	g	t	g	a	a	g	a	a	c	t	t	c	t	t	c	t	a	g	c	g	a	g	t	c	c	g	a	c	t	t	a	t	c	c	a	a	c	g	c	c	t	c	a	a	a	c	g	c	c	g	t	t	c	t	t	g	g	g	g	g	t	c	c	t	c	c	c	a	c	g	c	c
MH709375	3	t	a	g	t	g	a	a	g	a	a	c	t	t	c	t	t	c	t	a	g	c	g	a	g	t	c	c	g	a	c	t	t	a	t	c	c	a	a	c	a	c	c	t	c	a	a	a	c	g	c	c	g	t	t	c	t	t	g	g	g	g	g	t	c	c	t	c	c	c	a	c	g	c	c
DQ452050	4	t	a	g	t	g	a	a	g	a	a	c	t	t	c	t	t	c	t	a	a	c	g	a	g	c	c	c	g	a	c	g	t	g	t	c	c	a	a	a	g	c	c	c	c	a	a	a	c	g	c	c	g	t	g	c	t	t	g	g	g	a	g	t	c	c	t	c	c	c	a	c	g	c	c
DQ479960	4	t	a	g	t	g	a	a	g	a	a	c	t	t	c	t	t	c	t	a	a	c	g	a	g	t	c	c	g	a	c	g	t	g	t	c	c	a	a	a	g	c	c	c	c	a	a	a	c	g	c	c	g	t	g	c	t	t	g	g	g	a	g	t	c	c	t	c	c	c	a	c	g	c	c
KP771898	4	t	a	g	t	g	a	a	g	a	a	c	t	t	c	t	t	c	t	a	a	c	g	a	g	t	c	c	g	a	c	g	t	g	t	c	c	a	a	a	g	c	c	c	c	a	a	a	c	g	c	c	g	t	g	c	t	t	g	g	g	a	g	t	c	c	t	c	c	c	a	c	g	c	c
MH709316	4	t	a	g	t	g	a	a	g	a	a	c	t	t	c	t	t	c	t	a	a	c	g	a	g	t	c	c	g	a	c	g	t	g	t	c	c	a	a	a	g	c	c	c	c	a	a	a	c	g	c	c	g	t	g	c	t	t	g	g	g	a	g	t	c	c	t	c	c	c	a	c	g	c	c
MH709318	4	t	a	g	t	g	a	a	g	a	a	c	t	t	c	c	t	c	t	a	a	c	g	a	g	t	c	c	g	a	c	g	t	g	t	c	c	a	a	a	g	c	c	c	c	a	a	a	c	g	c	c	g	t	g	c	t	t	g	g	g	a	g	t	c	c	t	c	c	c	a	c	g	c	c
MH709322	4	t	a	g	t	g	a	a	g	a	a	c	t	t	c	c	t	c	t	a	a	c	g	a	g	t	c	c	g	a	c	g	t	g	t	c	c	a	a	a	g	c	c	c	c	a	a	a	c	g	c	c	g	t	g	c	t	t	g	g	g	a	g	t	c	c	t	c	c	c	a	c	g	c	c
MH709323	4	t	a	g	t	g	a	a	g	a	a	c	t	t	c	c	t	c	t	a	a	c	g	a	g	t	c	c	g	a	c	g	t	g	t	c	c	a	a	a	g	c	c	c	c	a	a	a	c	g	c	c	g	t	g	c	t	t	g	g	g	a	g	t	c	c	t	c	c	c	a	c	g	c	c
MH709326	4	t	a	g	t	g	a	a	g	a	a	c	t	t	c	c	t	c	t	a	a	c	g	a	g	t	c	c	g	a	c	g	t	g	t	c	c	a	a	a	g	c	c	c	c	a	a	a	c	g	c	c	g	t	g	c	t	t	g	g	g	a	g	t	c	c	t	c	c	c	a	c	g	c	c
MH709341	4	t	a	g	t	g	a	a	g	a	a	c	t	t	c	t	t	c	t	a	a	c	g	a	g	t	c	c	g	a	c	g	t	g	t	c	c	a	a	a	g	c	c	c	c	a	a	a	c	g	c	c	g	t	g	c	t	t	g	g	g	a	g	t	c	c	t	c	c	c	a	c	g	c	c
MH709357	4	t	a	g	t	g	a	a	g	a	a	c	t	t	c	t	t	c	t	a	a	c	g	a	g	t	c	c	g	a	c	g	t	g	t	c	c	a	a	a	g	c	c	c	c	a	a	a	c	g	c	c	g	t	g	c	t	t	g	g	g	a	g	t	c	c	t	c	c	c	a	c	g	c	c
MH709364	4	t	a	g	t	g	a	a	g	a	a	c	t	t	c	t	t	c	t	a	a	c	g	a	g	t	c	c	g	a	c	g	t	g	t	c	c	a	a	a	g	c	c	c	c	a	a	a	c	g	c	c	g	t	g	c	t	t	g	g	g	a	g	t	c	c	t	c	c	c	a	c	g	c	c
MH709370	4	t	a	g	t	g	a	a	g	a	a	c	t	t	c	t	t	c	t	a	a	c	g	a	g	t	c	c	g	a	c	g	t	g	t	c	c	a	a	a	g	c	c	c	c	a	a	a	c	g	c	c	g	t	g	c	t	t	g	g	g	a	g	t	c	c	t	c	c	c	a	c	g	c	c
MH709371	4	t	a	g	t	g	a	a	g	a	a	c	t	t	c	t	t	c	t	a	a	c	g	a	g	t	c	c	g	a	c	g	t	g	t	c	c	a	a	a	g	c	c	c	c	a	a	a	c	g	c	c	g	t	g	c	t	t	g	g	g	a	g	t	c	c	t	c	c	c	a	c	g	c	c
MH709372	4	t	a	g	t	g	a	a	g	a	a	c	t	t	c	t	t	c	t	a	a	c	g	a	g	t	c	c	g	a	c	g	t	g	t	c	c	a	a	a	g	c	c	c	c	a	a	a	c	g	c	c	g	t	g	c	t	t	g	g	g	a	g	t	c	c	t	c	c	c	a	c	g	c	c
DQ457052	5	t	a	g	t	g	a	a	g	a	a	c	t	t	c	t	t	c	t	a	g	c	g	g	g	t	c	c	g	a	c	g	t	g	t	c	c	a	a	a	g	c	c	c	c	a	a	a	c	g	c	c	g	t	t	c	t	t	g	g	g	g	g	t	t	c	t	c	c	c	a	c	c	c	c
JN704704	5	t	a	g	t	g	a	a	g	a	a	c	t	t	c	t	t	c	t	a	g	c	g	g	g	t	c	c	g	a	c	g	t	g	t	c	c	a	a	a	g	c	c	c	c	a	a	a	c	g	c	c	g	t	t	c	t	t	g	g	g	g	g	t	t	c	t	c	c	c	a	c	c	c	c
JN704705	5	t	a	g	t	g	a	a	g	a	a	c	g	t	t	t	t	c	t	a	g	c	g	g	g	t	a	c	g	a	c	g	t	g	t	c	c	a	a	a	g	c	c	c	c	a	a	a	c	g	t	c	g	t	t	c	t	t	g	g	g	g	g	t	c	c	t	c	c	c	a	c	g	c	c
JN704706	5	g	a	g	t	g	a	a	g	a	a	c	t	t	c	t	t	c	t	a	g	c	g	g	g	t	c	c	g	a	c	g	t	g	t	c	c	a	a	a	g	c	c	c	c	a	a	a	c	g	c	c	g	t	t	c	t	t	g	g	g	g	g	t	c	c	t	c	c	c	a	c	g	c	c
JN704707	5	t	a	g	t	g	a	a	g	a	a	c	t	t	c	t	t	c	t	a	g	c	g	g	g	t	c	c	g	a	c	g	t	g	t	c	c	a	a	a	g	c	c	c	c	a	a	a	c	g	c	c	g	t	t	c	t	t	g	g	g	g	g	t	t	c	t	c	c	c	a	c	c	c	c
JN704708	5	t	a	g	t	g	a	a	g	a	a	c	g	t	t	t	t	c	t	a	g	c	g	g	g	t	a	c	g	a	c	g	t	g	t	c	c	a	a	a	g	c	c	c	c	a	a	a	c	g	t	c	g	t	t	c	t	t	g	g	g	g	g	t	c	c	t	c	c	c	a	c	g	c	c
KP771904	5	t	a	g	t	g	a	a	g	a	a	c	t	t	c	t	t	c	t	a	g	c	g	g	g	t	c	c	g	a	c	g	t	g	t	c	c	a	a	a	g	c	c	c	c	a	a	a	c	g	c	c	g	t	t	c	t	t	g	g	g	g	g	t	t	c	t	c	c	c	a	c	c	c	c
KP771905	5	t	a	g	t	g	a	a	g	a	a	c	t	t	c	t	t	c	t	a	g	c	g	g	g	t	c	c	g	a	c	g	t	g	t	c	c	a	a	a	g	c	c	c	c	a	a	a	c	g	c	c	g	t	t	c	t	t	g	g	g	g	g	t	t	c	t	c	c	c	a	c	c	c	c
KP771906	5	t	a	g	t	g	a	a	g	a	a	c	t	t	c	t	t	c	t	a	g	c	g	g	g	t	c	c	g	a	c	g	t	g	t	c	c	a	a	a	g	c	c	c	c	a	a	a	c	g	c	c	g	t	t	c	t	t	g	g	g	g	g	t	t	c	t	c	c	c	a	c	c	c	c
KP771919	5	t	a	g	t	g	a	a	g	a	a	c	t	t	c	t	t	c	t	a	g	c	g	g	g	t	c	c	g	a	c	g	t	g	t	c	c	a	a	a	g	c	c	c	c	a	a	a	c	g	c	c	g	t	t	c	t	t	g	g	g	g	g	t	t	c	t	c	c	c	a	c	c	c	c
KP771922	5	t	a	g	t	g	a	a	g	a	a	c	t	t	c	t	t	c	t	a	g	c	g	g	g	t	c	c	g	a	c	g	t	g	t	c	c	a	a	a	g	c	c	c	c	a	a	a	c	g	c	c	g	t	t	c	t	t	g	g	g	g	g	t	t	c	t	c	c	c	a	c	c	c	c
KP771924	5	t	a	g	t	g	a	a	g	a	a	c	g	t	t	t	t	c	t	a	g	c	g	g	g	t	a	c	g	a	c	g	t	g	t	c	c	a	a	a	g	c	c	c	c	a	a	a	c	g	t	c	g	t	t	c	t	t	g	g	g	g	g	t	c	c	t	c	c	c	a	c	g	c	c
KM355696	5	t	a	g	t	g	a	a	g	a	a	c	g	t	t	t	t	c	t	a	g	c	g	g	g	t	a	c	g	a	c	g	t	g	t	c	c	a	a	a	g	c	c	c	c	a	a	a	c	g	t	c	g	t	t	c	t	t	g	g	g	g	g	t	c	c	t	c	c	c	a	c	g	c	c
KM355697	5	t	a	g	t	g	a	a	g	a	a	c	g	t	t	t	t	c	t	a	g	c	g	g	g	t	a	c	g	a	c	g	t	g	t	c	c	a	a	a	g	c	c	c	c	a	a	a	c	g	t	c	g	t	t	c	t	t	g	g	g	g	g	t	c	c	t	c	c	c	a	c	g	c	c
KM355698	5	t	a	g	t	g	a	a	g	a	a	c	g	t	t	t	t	c	t	a	g	c	g	g	g	t	a	c	g	a	c	g	t	g	t	c	c	a	g	a	g	c	c	c	c	a	a	a	c	g	t	c	g	t	t	c	t	t	g	g	g	g	g	t	c	c	t	c	c	c	a	c	g	c	c
KM355699	5	t	a	g	t	g	a	a	g	a	a	c	g	t	t	t	t	c	t	a	g	c	g	g	g	t	a	c	g	a	c	g	t	g	t	c	c	a	g	a	g	c	c	c	c	a	a	a	c	g	t	c	g	t	t	c	t	t	g	g	g	g	g	t	c	c	t	c	c	c	a	c	g	c	c
KM355701	5	t	a	g	t	g	a	a	g	a	a	c	g	t	t	t	t	c	t	a	g	c	g	g	g	t	a	c	g	a	c	g	t	g	t	c	c	a	a	a	g	c	c	c	c	a	a	a	c	g	t	c	g	t	t	t	t	t	g	g	g	g	g	t	c	c	t	c	c	c	a	c	g	c	c
KM355702	5	t	a	g	t	g	a	a	g	a	a	c	g	t	t	t	t	c	t	a	g	c	g	g	g	t	a	c	g	a	c	g	t	g	t	c	c	a	g	a	g	c	c	c	c	a	a	a	c	g	t	c	g	t	t	c	t	t	g	g	g	g	g	t	c	c	t	c	c	c	a	c	g	c	c
KM355703	5	t	a	g	t	g	a	a	g	a	a	c	g	t	t	t	t	c	t	a	g	c	g	g	g	t	a	c	g	a	c	g	t	g	t	c	c	a	a	a	g	c	c	c	c	a	a	a	c	g	t	c	g	t	t	c	t	t	g	g	g	g	g	t	c	c	t	c	c	c	a	c	g	c	c
KM355704	5	t	a	g	t	g	a	a	g	a	a	c	g	t	t	t	t	c	t	a	g	c	g	g	g	t	a	c	g	a	c	g	t	g	t	c	c	a	a	a	g	c	c	c	c	a	a	a	c	g	t	c	g	t	t	c	t	t	g	g	g	g	g	t	c	c	t	c	c	c	a	c	g	c	c
KM355705	5	t	a	g	t	g	a	a	g	a	a	c	g	t	t	t	t	c	t	a	g	c	g	g	g	t	a	c	g	a	c	g	t	g	t	c	c	a	g	a	g	c	c	c	c	a	a	a	c	g	t	c	g	t	t	c	t	t	g	g	g	g	g	t	c	c	t	c	c	c	a	c	g	c	c
KM355706	5	t	a	g	t	g	a	a	g	a	a	c	g	t	t	t	t	c	t	a	g	c	g	g	g	t	a	c	g	a	c	g	t	g	t	c	c	a	g	a	g	c	c	c	c	a	a	a	c	g	t	c	g	t	t	c	t	t	g	g	g	g	g	t	c	c	t	c	c	c	a	c	g	c	c
KM355707	5	t	a	g	t	g	a	a	g	a	a	c	g	t	t	t	t	c	t	a	g	c	g	g	g	t	a	c	g	a	c	g	t	g	t	c	c	a	a	a	g	c	c	c	c	a	a	a	c	g	t	c	g	t	t	c	t	t	g	g	g	g	g	t	c	c	t	c	c	c	a	c	g	c	c
KM355708	5	t	a	g	t	g	a	a	g	a	a	c	g	t	t	t	t	c	t	a	g	c	g	g	g	t	a	c	g	a	c	g	t	g	t	c	c	a	a	a	g	c	c	c	c	a	a	a	c	g	t	c	g	t	t	c	t	t	g	g	g	g	g	t	c	c	t	c	c	c	a	c	g	c	c
KM355709	5	t	a	g	t	g	a	a	g	a	a	c	g	t	t	t	t	c	t	a	g	c	g	g	g	t	a	c	g	a	c	g	t	g	t	c	c	a	g	a	g	c	c	c	c	a	a	a	c	g	t	c	g	t	t	c	t	t	g	g	g	g	g	t	c	c	t	c	c	c	a	c	g	c	c
KM355711	5	t	a	g	t	g	a	a	g	a	a	c	g	t	t	t	t	c	t	a	g	c	g	g	g	t	a	c	g	a	c	g	t	g	t	c	c	a	g	a	g	c	c	c	c	a	a	a	c	g	t	c	g	t	t	c	t	t	g	g	g	g	g	t	c	c	t	c	c	c	a	c	g	c	c
KM355712	5	t	a	g	t	g	a	a	g	a	a	c	g	t	t	t	t	c	t	a	g	c	g	g	g	t	a	c	g	a	c	g	t	g	t	c	c	a	g	a	g	c	c	c	c	a	a	a	c	g	t	c	g	t	t	c	t	t	g	g	g	g	g	t	c	c	t	c	c	c	a	c	g	c	c
KY037797	5	t	a	g	t	g	a	a	g	a	a	c	g	t	t	t	t	c	t	a	g	c	g	g	g	t	a	c	g	a	c	g	t	g	t	c	c	a	a	a	g	c	c	c	c	a	a	a	c	g	t	c	g	t	t	c	t	t	g	g	g	g	g	t	c	c	t	c	c	c	a	c	g	c	c

Supplementary Table 3: 2880 SN

GB-ID	Clade markers																		9		4			5																																																				
	Position in Dumas	10133	10165	10176	10188	10340	10344	10536	10540	10608	10654	10727	10818	10900	10946	10957	11044	11140	11149	11185	11317	11340	11361	11449	11527	11554	11725	11749	11801	11848	11861	11890	11894	11906	11934	11984	12020	12119	12140	12188	12268	12284	12285	12320	12528	12588	12612	12680	12779	12837	12847	12900	12933	12975	13173	13182	13274	13275	13298	13344	13401	13407	13458	13475	13486	13539	13585	13665	13667	13669	13739	13827	13846	13861	13870
MH499467	5	t	a	g	t	g	a	a	g	a	t	c	t	t	c	t	t	c	t	a	g	c	g	g	g	t	c	c	g	a	a	g	t	g	t	c	c	a	a	a	g	c	c	c	c	a	a	a	c	g	c	c	g	t	t	c	t	t	g	g	g	g	g	t	t	c	t	c	c	c	a	c	c	c	c
MH499469	5	t	a	g	t	g	a	a	g	a	a	c	t	t	c	t	t	c	t	a	g	c	g	g	g	t	c	c	g	a	c	g	t	g	t	c	c	a	a	a	g	c	c	c	c	a	a	a	c	g	c	c	g	t	t	c	t	t	g	g	g	g	g	t	t	c	t	c	c	c	a	c	c	c	c
MH709321	5	g	a	g	t	g	a	a	g	c	a	c	t	t	c	t	t	c	t	a	g	c	g	g	g	t	c	c	a	a	c	g	t	g	t	c	c	a	a	a	g	c	c	c	c	a	a	a	c	g	c	c	g	t	t	c	t	t	g	g	g	g	g	t	c	c	t	c	c	c	a	c	g	c	c
MH709324	5	t	a	g	t	g	a	a	g	a	a	c	t	t	c	t	t	c	t	a	g	c	g	g	g	t	c	c	g	a	c	g	t	g	t	c	c	a	a	a	g	c	c	c	c	a	a	a	c	g	c	c	g	t	t	c	t	t	g	g	g	g	g	t	t	c	t	c	c	c	a	c	c	c	c
MH709325	5	t	a	g	t	g	a	a	g	a	a	c	g	t	t	t	t	c	t	a	g	c	g	g	g	t	a	c	g	a	c	g	t	g	t	c	c	a	g	a	g	c	c	c	c	a	a	a	c	g	t	c	g	t	t	c	t	t	g	t	g	g	g	t	c	c	t	c	c	c	a	c	g	c	c
MH709327	5	t	a	g	t	g	a	a	g	a	a	c	t	t	c	t	t	c	t	a	g	c	g	g	g	t	c	c	g	a	c	g	t	g	t	c	c	a	a	a	g	c	c	c	c	a	a	a	c	g	c	c	g	t	t	c	t	t	g	g	g	g	g	t	t	c	t	c	c	c	a	c	c	c	c
MH709328	5	t	a	g	t	g	a	a	g	a	a	c	t	t	c	t	t	c	t	a	g	c	g	g	g	t	c	c	g	a	c	g	t	g	t	c	c	a	a	a	g	c	c	c	c	a	a	a	c	g	c	c	g	t	t	c	t	t	g	g	g	g	g	t	t	c	t	c	c	c	a	c	c	c	c
MH709330	5	t	a	g	t	g	a	a	g	a	a	c	t	t	c	t	t	c	t	a	g	c	g	g	g	t	c	c	g	a	c	g	t	g	t	c	c	a	a	a	g	c	c	c	c	a	a	a	c	g	c	c	g	t	t	c	t	t	g	g	a	g	g	t	t	c	t	c	c	c	a	c	c	c	c
MH709332	5	t	a	g	t	g	a	a	g	a	a	c	t	t	c	t	t	c	t	a	g	c	g	g	g	t	c	c	g	a	c	g	t	g	t	c	c	a	a	a	g	c	c	c	c	a	a	a	c	g	c	c	g	t	t	c	a	a	g	g	g	g	g	t	t	c	t	c	c	c	a	c	c	c	c
MH709334	5	t	a	g	t	g	a	a	g	a	a	c	t	t	c	t	t	c	t	a	g	c	g	g	g	t	c	c	g	a	c	g	t	g	t	c	c	a	a	a	g	c	c	c	c	a	a	a	c	g	c	c	g	t	t	c	t	t	g	g	g	g	g	t	t	c	t	c	c	c	a	c	c	c	c
MH709335	5	t	a	g	t	g	a	a	g	a	a	c	t	t	c	t	t	c	t	a	g	c	g	g	g	t	c	c	g	a	c	g	t	g	t	c	c	a	a	a	g	c	c	c	c	a	a	a	c	g	c	c	g	t	t	c	t	t	g	g	g	g	g	t	t	c	t	c	c	c	a	c	c	c	c
MH709336	5	t	a	g	t	g	a	a	g	a	a	c	t	t	c	t	t	c	t	a	g	c	g	g	g	t	c	c	g	a	c	g	t	g	t	c	c	a	a	a	g	c	c	c	c	a	a	a	c	g	c	c	g	t	t	c	t	t	g	g	g	g	g	t	t	c	t	c	c	c	a	c	c	c	c
MH709338	5	t	a	g	t	g	a	a	g	a	a	c	t	t	c	t	t	c	t	a	g	c	g	g	g	t	c	c	g	a	c	g	t	g	t	c	c	a	a	a	g	c	c	c	c	a	a	a	c	g	c	c	g	t	t	c	t	t	g	g	g	g	g	t	t	c	t	c	c	c	a	c	c	c	c
MH709340	5	t	a	g	t	g	a	a	g	a	a	c	t	t	c	t	t	c	t	a	g	c	g	g	g	t	c	c	g	a	c	g	t	g	t	c	c	a	a	a	g	c	c	c	c	a	a	a	c	g	c	c	g	t	t	c	t	t	g	g	g	g	g	t	c	c	t	c	c	c	a	c	g	c	c
MH709342	5	g	a	g	t	g	a	a	g	a	a	c	t	t	c	t	t	c	t	a	g	c	g	g	g	t	c	c	g	a	c	g	t	g	t	c	c	a	a	a	g	c	c	c	c	a	a	a	c	g	c	c	g	t	t	c	t	t	g	g	g	g	g	t	c	c	t	c	c	c	a	c	g	c	c
MH709343	5	t	a	g	t	g	a	a	g	a	a	c	t	t	c	t	t	c	t	a	g	c	g	g	g	t	c	c	g	a	c	g	t	g	t	c	c	a	a	a	g	c	c	c	c	a	a	a	c	g	c	c	g	t	t	c	t	t	g	g	g	g	g	t	t	c	t	c	c	c	a	c	c	c	c
MH709345	5	t	a	g	t	g	a	a	g	a	a	c	t	t	c	t	t	c	t	a	g	c	g	g	g	t	c	c	g	a	c	g	t	g	t	c	c	a	a	a	g	c	c	c	c	a	a	a	c	g	c	c	g	t	t	c	t	t	g	g	g	g	g	t	t	c	t	c	c	c	a	c	c	c	c
MH709347	5	t	a	g	t	g	a	a	g	a	a	c	t	t	c	t	t	c	t	a	g	c	g	g	g	t	c	c	g	a	c	g	t	g	t	c	c	a	a	a	g	c	c	c	c	a	a	a	c	g	c	c	g	t	t	c	t	t	g	g	g	g	g	t	t	c	t	c	c	c	a	c	c	c	c
MH709349	5	t	a	g	t	g	a	a	g	a	a	c	t	t	c	t	t	c	t	a	g	c	g	g	g	t	c	c	g	g	c	g	t	g	t	c	c	a	a	a	g	c	c	c	c	a	a	a	c	g	c	c	g	t	t	c	t	t	g	g	g	g	g	g	t	c	t	c	c	c	a	c	c	c	c
MH709350	5	t	a	g	t	g	a	a	g	a	a	c	t	t	c	t	t	c	t	a	g	c	g	g	g	t	c	c	g	a	c	g	t	g	t	c	c	a	a	a	g	c	c	c	c	a	a	a	c	g	c	c	g	t	t	c	t	t	g	g	g	g	g	t	t	c	t	c	c	c	a	c	c	c	c
MH709351	5	t	a	g	t	g	a	a	g	a	a	c	t	t	c	t	t	c	t	a	g	c	g	g	g	t	c	c	g	a	c	g	t	g	t	c	c	a	a	a	g	c	c	c	c	a	a	a	c	g	c	c	g	t	t	c	t	t	g	g	g	g	g	t	c	c	t	c	c	c	a	c	g	c	c
MH709353	5	t	a	g	t	g	a	a	g	a	a	c	t	t	c	t	t	c	t	a	g	c	g	g	g	t	c	c	g	a	c	g	t	g	t	c	c	a	a	a	g	c	c	c	c	a	a	a	c	g	c	c	g	t	t	c	t	t	g	g	g	g	g	t	t	c	t	c	c	c	a	c	c	c	c
MH709358	5	t	a	g	t	g	a	a	g	a	a	c	g	t	t	t	t	c	t	a	g	c	g	g	g	t	a	c	g	a	c	g	t	g	t	c	c	a	a	a	g	c	c	c	c	a	a	a	c	g	t	c	g	t	t	c	t	t	g	g	g	g	g	t	c	c	t	c	c	c	a	c	g	c	c
MH709360	5	t	a	g	t	g	a	a	g	a	a	c	t	t	c	t	t	c	t	a	g	c	g	g	g	t	c	c	g	a	c	g	t	g	t	c	c	a	a	a	g	c	c	c	c	a	a	a	c	g	c	c	g	t	t	c	t	t	g	g	g	g	g	t	t	c	t	c	c	c	a	c	c	c	c
MH709362	5	t	a	g	t	g	a	a	g	a	a	c	t	t	c	t	t	c	t	a	g	c	g	g	g	t	c	c	g	a	c	g	t	g	t	c	c	a	a	a	g	c	c	c	c	a	a	a	c	g	c	c	g	t	t	c	t	t	g	g	g	g	g	t	t	c	t	c	c	c	a	c	c	c	c
MH709365	5	t	a	g	t	g	a	a	g	a	a	c	t	t	c	t	t	c	t	a	g	c	g	g	g	t	c	c	g	a	c	g	t	g	t	c	c	a	a	a	g	c	c	c	c	a	a	a	c	g	c	c	g	t	t	c	t	t	g	g	g	g	g	t	t	c	t	c	c	c	a	c	c	c	c
MH709373	5	t	a	g	t	g	a	a	g	a	a	c	t	t	c	t	t	c	t	a	g	c	g	g	g	t	c	c	g	a	c	g	t	g	t	c	c	a	a	a	g	c	c	c	c	a	a	a	c	g	c	c	g	t	t	c	t	t	g	g	g	g	g	t	t	c	t	c	c	c	a	c	c	c	c
MH709374	5	t	a	g	t	g	a	a	g	a	a	c	t	t	c	t	t	c	t	a	g	c	g	g	g	t	c	c	g	a	c	g	t	g	t	c	c	a	a	a	g	c	c	c	c	a	a	a	c	g	c	c	g	t	t	c	t	t	g	g	g	g	g	t	t	c	t	c	c	c	a	c	c	c	c
KC112914	6	t	a	g	t	g	a	a	g	a	a	c	t	t	c	t	t	c	t	a	g	c	g	a	g	t	c	c	g	a	c	g	t	g	t	c	c	a	a	c	g	c	c	c	c	a	a	a	c	g	c	c	g	t	g	c	t	t	g	g	g	a	g	t	c	c	t	c	c	c	a	c	g	c	c
KP771914	6	t	a	g	t	g	a	a	g	a	a	c	t	t	c	t	t	c	t	a	g	c	g	a	g	t	c	c	g	a	c	g	t	g	t	c	c	a	a	c	g	c	c	c	c	a	a	a	c	g	c	c	g	t	g	c	t	t	g	g	g	a	g	t	c	c	t	c	c	c	a	c	g	c	c
KP702725	6	t	a	g	t	g	a	a	g	a	a	c	t	t	c	t	t	c	t	a	g	c	g	a	g	t	c	c	g	a	c	g	t	g	t	c	c	a	a	c	g	c	c	c	c	a	a	a	c	g	c	c	g	t	g	c	t	t	g	g	g	a	g	t	c	c	t	c	c	c	a	c	g	c	c
KP771890	9	t	a	g	t	g	a	a	g	a	a	c	t	t	c	t	t	c	c	a	g	c	g	a	g	t	c	c	g	a	c	g	t	g	t	c	c	a	a	a	g	c	c	c	c	a	a	a	c	g	c	c	g	t	t	c	t	t	g	g	g	g	g	t	c	c	t	c	c	c	a	c	g	c	c
JN704710	9	t	a	g	t	g	a	a	g	a	a	c	t	t	c	t	t	t	c	a	g	c	g	a	g	t	c	c	g	a	c	g	t	g	t	c	c	a	a	a	g	c	c	c	c	a	a	a	c	g	c	c	g	t	t	c	t	t	g	g	g	g	g	t	c	c	t	c	c	c	a	c	g	c	c
KY037798	9	t	a	g	t	g	a	a	g	a	a	c	t	t	c	t	t	c	c	a	g	c	g	a	g	t	c	c	g	a	c	g	t	g	t	c	c	a	a	a	g	c	c	c	c	a	a	a	c	g	c	c	g	t	t	c	t	t	g	g	g	g	g	t	c	c	t	c	c	c	a	c	g	c	c
MH709312	9	t	a	g	t	g	a	a	g	a	a	c	t	t	c	t	t	c	c	a	g	c	g	a	g	t	c	c	g	a	c	g	t	g	t	c	c	a	a	a	g	c	c	c	c	a	a	a	c	g	c	c	g	t	t	c	t	t	g	g	g	g	g	t	c	c	t	c	c	c	a	c	g	c	c
MH709352	9	t	g	g	t	g	a	a	g	a	a	c	t	t	c	t	t	c	c	a	g	c	g	a	g	t	g	c	g	a	c	g	t	g	t	c	c	a	a	a	g	c	c	c	c	a	a	a	c	g	c	c	g	t	t	c	t	t	g	g	g	g	g	t	c	c	t	c	c	c	a	c	g	c	c
AB097933	Vac	t	a	g	t	g	a	a	g	a	a	c	t	t	c	t	t	c	t	a	g	c	g	a	g	t	c	c	g	a	c	g	t	g	t	c	c	a	a	a	g	c	c	c	c	a	a	a	c	g	c	c	g	t	g	c	t	t	g	g	g	a	g	t	c	c	t	c	c	c	a	c	g	c	c
JN704698	Vac	t	a	g	t	g	a	a	g	a	a	c	t	t	c	t	t	c	t	a	g	c	g	a	g	t	c	c	g	a	c	g	t	g	t	c	c	a	a	a	g	c	c	c	c	a	a	a	c	g	c	c	g	t	g	c	t	t	g	g	g	a	g	t	c	c	t	c	c	c	a	c	g	c	c
JN704697	Vac	t	a	g	t	g	a	a	g	a	a	c	t	c	c	t	t	c	t	a	g	c	g	a	g	t	c	c	g	a	c	g	t	g	t	c	c	a	a	a	g	c	c	c	c	a	a	a	t	g	c	c	g	t	g	c	t	t	g	g	g	g	g	t	c	c	t	c	c	c	a	c	g	c	c
DQ008355	Vac	t	a	g	t	g	a	a	g	a	a	c	t	t	c	t	t	c	t	a	g	c	g	a	g	t	c	c	g	a	c	g	t	g	t	c	c	a	a	a	g	c	c	c	c	a	a	a	c	g	c	c	g	t	g	c	t	t	g	g	g	a	g	t	c	c	t	c	c	c	a	c	g	c	c
JF306641	Vac	t	a	g	t	g	a	a	g	a	a	c	t	t	c	t	t	c	t	a	g	c	g	a	g	t	c	c	g	a	c	g	t	g	t	c	c	a	a	a	g	c	c	c	c	a	a	a	c	g	c	c	g	t	g	c	t	t	g	g	g	a	g	t	c	c	t	c	c	c	a	c	g	c	c
AB097932	Vac	t	a	g	t	g	a	a	g	a	a	c	t	y	c	t	t	c	t	a	g	c	g	a	g	t	c	c	g	a	c	g	t	g	t	c	c	a	a	a	g	c	c	c	c	a	a	a	y	g	c	c	g	t	g	c	t	t	g	g	g	a	g	t	c	c	t	c	c	c	a	c	g	c	c
DQ008354	Vac	t	a	g	t	g	a	a	g	a	a	c	t	y	c	t	t	c	t	a	g	c	g	a	g	t	c	c	g	a	c	g	t	g	t	c	c	a	a	a	g	c	c	c	c	a	a	a	y	g	c	c	g	t	g	c	t	t	g	g	g	a	g	t	c	c	t	c	c	c	a	c	g	c	c
KU926312	Vac	t	a	g	t	g	a	a	g	a	a	c	t	t	c	t	t	c	t	a	g	c	g	a	g	t	c	c	g	a	c	g	t	g	t	c	c	a	a	a	g	c	c	c	c	a	a	a	c	g	c	c	g	t	g	c	t	t	g	g	g	a	g	t	c	c	t	c	c	c	a	c	g	c	c
KU926313	Vac	t	a	g	t	g	a	a	g	a	a	c	t	t	c	t	t	c	t	a	g	c	g	a	g	t	c	c	g	a	c	g	t	g	t	c	c	a	a	a	g	c	c	c	c	a	a	a	c	g	c	c	g	t	g	c	t	t	g	g	g	a	g	t	c	c	t	c	c	c	a	c	g	c	c
KU926314	Vac	t	a	g	t	g	a	a	g	a	a	c	t	t	c	t	t	c	t	a	g	c	g	a	g	t	c	c	g	a	c	g	t	g	t	c	c	a	a	a	g	c	c	c	c	a	a	a	c	g	c	c	g	t	g	c	t	t	g	g	g	a	g	t	c	c	t	c	c	c	a	c	g	c	c
KF558371	Vac	t	a	g	t	g	a	a	g	a	a	c	t	t	c	t	t	c	t	a	g	c	g	a	g	t	c	c	g	a	c	g	t	g	t	c	c	a	a	a	g	c	c	c	c	a	a	a	c	g	c	c	g	t	g	c	t	t	g	g	g	a	g	t	c	c	t	c	c	c	a	c	g	c	c
KF558372	Vac	t	a	g	t	g	a	a	g	a	a	c	t	t	c	t	t	c	t	a	g	c	g	a	g	t	c	c	g	a	c	g	t	g	t	c	c	a	a	a	g	c	c	c	c	a	a	a	c	g	c	c	g	t	g	c	t	t	g	g	g	a	g	t	c	c	t	c	c	c	a	c	g	c	c
KF558373	Vac	t	a	g	t	g	a	a	g	a	a	c	t	c	c	t	t	c	t	a	g	c	g	a	g	t	c	c	g	a	c	g	t	g	t	c	c	a	a	a	g	c	c	c	c	a	a	a	c	g	c	c	g	t	g	c	t	t	g	g	g	a	g	t	c	c	t	c	c	c	a	c	g	c	c
KF558374	Vac	t	a	g	t	g	a	a	g	a	a	c	t	t	c	t	t	c	t	a	g	c	g	a	g	t	c	c	g	a	c	g	t	g	t	c	c	a	a	a	g	c	c	c	c	a	a	a	c	g	c	c	g	t	g	c	t	t	g	g	g	a	g	t	c	c	t	c	c	c	a	c	g	c	c
KF558375	Vac	t	a	g	t	g	a	a	g	a	a	c	t	t	c	t	t	c	t	a	g	c	g	a	g	t	c	c	g	a	c	g	t	g	t	c	c	a	a	a	g	c	c	c	c	a	a	a	c	g	c	c	g	t	g	c	t	t	g	g	g	a	g	t	c	c	t	c	c	c	a	c	g	c	c
KF558376	Vac	t	a	g	t	g	a	a	g	a	a	c	t	t	c	t	t	c	t	a	g	c	g	a	g	t	c	c	g	a	c	g	t	g	t	c	c	a	a	a	g	c	c	c	c	a	a	a	c	g	c	c	g	t	g	c	t	t	g	g	g	a	g	t	c	c	t	c	c	c	a	c	g	c	c
KF558377	Vac	t	a	g	t	g	a	a	g	a	a	c	t	t	c	t	t	c	t	a	g	c	g	a	g	t	c	c	g	a	c	g	t	g	t	c	c	a	a	a	g	c	c	c	c	a	a	a	c	g	c	c	g	t	g	c	t	t	g	g	g	a	g	t	c	c	t	c	c	c	a	c	g	c	c
KF558378	Vac	t	a	g	t	g	a	a	g	a	a	c	t	t	c	t	t	c	t	a	g	c	g	a	g	t	c	c	g	a	c	g	t	g	t	c	c	a	a	a	g	c	c	c	g	a	a	a	c	g	c	c	g	t	g	c	t	t	g	g	g	a	g	t	c	c	t	c	c	c	a	c	g	c	c
KF558379	Vac	t	a	g	t	g	a	a	a	a	a	c	t	t	c	t	t	c	t	a	g	c	g	a	g	t	c	c	g	a	c	g	t	g	t	c	c	a	a	a	g	c	c	c	c	a	a	a	c	g	c	c	g	t	g	c	t	t	g	g	g	a	g	t	c	c	t	c	c	c	a	c	g	c	c
KF558380	Vac	t	a	g	t	g	a	a	g	a	a	c	t	t	c	t	t	c	t	a	g	c	g	a	g	t	c	c	g	a	c	g	t	g	t	c	c	a	a	a	g	c	c	c	c	a	a	a	t	g	c	c	g	t	g	c	t	t	g	g	g	a	g	t	c	c	t	c	c	c	a	c	g	c	c
KF558381	Vac	t	a	g	t	g	a	a	g	a	a	c	t	t	c	t	t	c	t	a	g	c	g	a	g	t	c	c	g	a	c	g	t	g	t	c	c	a	a	a	g	c	c	c	c	a	a	a	c	g	c	c	g	t	g	c	t	t	g	g	g	a	g	t	c	c	t	c	c	c	a	c	g	c	c
KF558382	Vac	t	a	g	t	g	a	a	g	a	a	c	t	c	c	t	t	c	t	a	g	c	g	a	g	t	c	c	g	a	c	g	t	g	t	c	c	a	a	a	g	c	c	c	c	a	a	a	c	g	c	c	g	t	g	c	t	t	g	g	g	a	g	t	c	c	t	c	c	c	a	c	g	c	c
KF558383	Vac	t	a	g	t	g	a	a	g	a	a	c	t	t	c	t	t	c	t	a	g	c	g	a	g	t	c	c	g	a	c	g	t	g	t	c	c	a	a	a	g	c	c	c	c	a	a	a	c	g	c	c	g	t	g	c	t	t	g	g	g	a	g	t	c	c	t	c	c	c	a	c	g	c	c
KF558384	Vac	t	a	g	t	g	a	a	g	a	a	c	t	t	c	t	t	c	t	a	g	c	g	a	g	t	c	c	g	a	c	g	t	g	t	c	c	a	a	a	g	c	c	c	c	a	a	a	c	g	c	c	g	t	g	c	t	t	g	g	g	a	g	t	c	c	t	c	c	c	a	c	g	c	c
KF558385	Vac	t	a	g	t	g	a	a	g	a	a	c	t	t	c	t	t	c	t	a	g	c	g	a	g	t	c	c	g	a	c	g	t	g	t	c	c	a	a	a	g	c	c	c	c	a	a	a	c	g	c	c	g	t	g	c	t	t	g	g	g	a	g	t	c	c	t	c	c	c	a	c	g	c	c
KF558386	Vac	t	a	g	t	g	a	a	g	a	a	c	t	t	c	t	t	c	t	a	g	c	g	a	g	t	c	c	g	a	c	g	t	g	t	c	c	a	a	a	g	c	c	c	c	a	a	a	c	g	c	c	g	t	g	c	t	t	g	g	g	a	g	t	c	c	t	c	c	c	a	c	g	c	c
KF558387	Vac	t	a	g	t	g	a	a	g	a	a	c	t	t	c	t	t	c	t	a	g	c	g	a	g	t	c	c	g	a	c	g	t	g	t	c	c	a	a	a	g	c	c	c	c	a	a	a	c	g	c	c	g	t	g	c	t	t	g	g	g	a	g	t	c	c	t	c	c	c	a	c	g	c	c
KF558388	Vac	t	a	g	t	g	a	a	g	a	a	c	t	t	c	t	t	c	t	a	g	c	g	a	g	t	c	c	g	a	c	g	t	g	t	c	c	a	a	a	g	c	c	c	c	a	a	a	c	g	c	c	g	t	g	c	t	t	g	g	g	a	g	t	c	c	t	c	c	c	a	c	g	c	c
KF558389	Vac	t	a	g	t	g	a	a	g	a	a	c	t	c	c	t	t	c	t	a	g	c	g	a	g	t	c	c	g	a	c	g	t	g	t	c	c	a	a	a	g	c	c	c	c	a	a	a	c	g	c	c	g	t	g	c	t	t	g	g	g	a	g	t	c	c	t	c	c	c	a	c	g	c	c
KF558390	Vac	t	a	g	t	g	a	a	g	a	a	c	t	t	c	t	t	c	t	a	g	c	g	a	g	t	c	c	g	a	c	g	t	g	t	c	c	a	a	a	g	c	c	c	c	a	a	a	c	g	c	c	g	t	g	c	t	t	g	g	g	a	g	t	c	c	t	c	c	c	a	c	g	c	c
KF558391	Vac	t	a	g	t	g	a	a	g	a	a	c	t	t	c	t	t	c	t	a	g	c	g	a	g	t	c	c	g	a	c	g	t	g	t	c	c	a	a	a	g	c	c	c	c	a	a	a	c	g	c	c	g	t	g	c	t	t	g	g	g	a	g	t	c	c	t	c	c	c	a	c	g	c	c
KF811485	Vac	t	a	g	t	g	a	a	g	a	a	c	t	t	c	t	t	c	t	a	g	c	g	a	g	t	c	c	g	a	c	g	t	g	t	c	c	a	a	a	g	c	c	c	c	a	a	a	c	g	c	c	g	t	g	c	t	t	g	g	g	a	g	t	c	c	t	c	c	c	a	c	g	c	c
KF853225	Vac	t	a	g	t	g	a	a	g	a	a	c	t	t	c	t	t	c	t	a	g	c	g	a	g	t	c	c	g	a	c	g	t	g	t	c	c	a	a	a	g	c	c	c	c	a	a	a	c	g	c	c	g	t	g	c	t	t	g	g	g	a	g	t	c	c	t	c	c	c	a	c	g	c	c
KF853226	Vac	t	a	g	t	g	a	a	g	a	a	c	t	t	c	t	t	c	t	a	g	c	g	a	g	t	c	c	g	a	c	g	t	g	t	c	c	a	a	a	g	c	c	c	c	a	a	a	c	g	c	c	g	t	g	c	t	t	g	g	g	a	g	t	c	c	t	c	c	c	a	c	g	c	c
KF853227	Vac	t	a	g	t	g	a	a	g	a	a	c	t	t	c	t	t	c	t	a	g	c	g	a	g	t	c	c	g	a	c	g	t	g	t	c	c	a	a	a	g	c	c	c	c	a	a	a	c	g	c	c	g	t	g	c	t	t	g	g	g	a	g	t	c	c	t	c	c	c	a	c	g	c	c
KF853228	Vac	t	a	g	t	g	a	a	g	a	a	c	t	t	c	t	t	c	t	a	g	c	g	a	g	t	c	c	g	a	c	g	t	g	t	c	c	a	a	a	g	c	c	c	c	a	a	a	c	g	c	c	g	t	g	c	t	t	g	g	g	a	g	t	c	c	t	c	c	c	a	c	g	c	c
KF853229	Vac	t	a	g	t	g	a	a	g	a	a	c	t	t	c	t	t	c	t	a	g	c	g	a	g	t	c	c	g	a	c	g	t	g	t	c	c	a	a	a	g	c	c	c	c	a	a	a	t	g	c	c	g	t	g	c	t	t	g	g	g	a	g	t	c	c	t	c	c	c	a	c	g	c	c
KF853230	Vac	t	a	g	t	g	a	a	g	a	a	c	t	t	c	t	t	c	t	a	g	c	g	a	g	t	c	c	g	a	c	g	t	g	t	c	c	a	a	a	g	c	c	c	c	a	a	a	c	g	c	c	g	t	g	c	t	t	g	g	g	a	g	t	c	c	t	c	c	c	a	c	g	c	c
KF853231	Vac	t	a	g	t	g	a	a	g	a	a	c	t	t	c	t	t	c	t	a	g	c	g	a	g	t	c	c	g	a	c	g	t	g	t	c	c	a	a	a	g	c	c	c	c	a	a	a	c	g	c	c	g	t	g	c	t	t	g	g	g	a	g	t	c	c	t	c	c	c	a	c	g	c	c
KF853232	Vac	t	a	g	t	g	a	a	g	a	a	c	t	t	c	t	t	c	t	a	g	c	g	a	g	t	c	c	g	a	c	g	t	g	t	c	c	a	a	a	g	c	c	c	c	a	a	a	c	g	c	c	g	t	g	c	t	t	g	g	g	a	g	t	c	c	t	c	c	c	a	c	g	c	c
KF853233	Vac	t	a	g	t	g	a	a	g	a	a	c	t	t	c	t	t	c	t	a	g	c	g	a	g	t	c	c	g	a	c	g	t	g	t	c	c	a	a	a	g	c	c	c	c	a	a	a	c	g	c	c	g	t	g	c	t	t	g	g	g	a	g	t	c	c	t	c	c	c	a	c	g	c	c
KF853234	Vac	t	a	g	t	g	a	a	g	a	a	c	t	t	c	t	t	c	t	a	g	c	g	a	g	t	c	c	g	a	c	g	t	g	t	c	c	a	a	a	g	c	c	c	c	a	a	a	c	g	c	c	g	t	g	c	t	t	g	g	g	a	g	t	c	c	t	c	c	c	a	c	g	c	c
KF853235	Vac	t	a	g	t	g	a	a	g	a	a	c	t	t	c	t	t	c	t	a	g	c	g	a	g	t	c	c	g	a	c	g	t	g	t	c	c	a	a	a	g	c	c	c	c	a	a	a	c	g	c	c	g	t	g	c	t	t	g	g	g	a	g	t	c	c	t	c	c	c	a	c	g	c	c
MF898328	Vac	t	a	g	t	g	a	a	g	a	a	c	t	y	c	t	t	c	t	a	g	c	g	a	g	t	c	c	g	a	c	g	t	g	t	c	c	a	a	a	g	c	c	c	c	a	a	a	c	g	c	c	g	t	g	c	t	t	g	g	g	a	g	t	c	c	t	c	c	c	a	c	g	c	c
JN704709	VIII	t	a	g	t	g	a	g	g	a	a	c	t	t	c	t	t	c	t	a	g	c	g	a	g	t	c	c	g	a	c	g	t	g	t	c	c	a	a	c	g	c	c	c	c	a	a	a	c	g	c	c	g	t	g	c	t	t	g	g	g	a	g	t	c	c	t	c	c	c	a	c	g	c	c

Supplementary Table 3: 2880 SN

GB-ID	Clade markers					1						4												3																9																																			
	Position in Dumas	13876	13925	14345	14362	14390	14402	14432	14589	14801	15018	15182	15183	15197	15238	15299	15419	15533	15583	15608	15787	15865	15884	15896	15963	16021	16023	16047	16067	16072	16109	16145	16160	16264	16384	16418	16438	16485	16522	16705	16720	16827	16851	16903	16972	17098	17155	17163	17365	17392	17404	17534	17725	17734	17753	17770	17810	17834	17850	17973	17987	18052	18054	18082	18093	18202	18224	18240	18275	18303	18309	18323	18406	18467	18539
AY548170	1	a	c	t	c	t	t	t	a	c	c	t	c	c	a	t	t	c	g	g	c	a	a	g	c	t	g	t	a	g	g	t	t	a	t	g	g	g	a	a	a	g	g	c	a	t	t	c	t	g	t	g	a	g	g	g	g	c	c	t	t	t	t	c	t	a	g	c	t	t	c	c	g	a	t
AY548171	1	a	c	c	c	t	t	t	a	c	c	t	c	c	a	t	t	c	g	g	c	a	a	g	c	t	g	t	a	g	g	t	t	a	t	g	g	g	a	a	a	g	g	c	a	t	t	c	t	g	t	g	a	a	g	g	g	c	c	t	t	c	t	c	t	a	g	c	t	t	c	c	g	a	t
DQ479953	1	a	c	t	c	t	t	t	a	c	c	t	c	c	a	t	t	c	g	g	c	a	a	g	c	t	g	t	a	g	g	t	t	a	t	g	g	g	a	a	a	g	g	c	a	t	t	c	t	g	t	g	a	g	g	g	g	c	c	t	t	c	t	c	t	a	g	c	t	t	c	c	g	a	t
DQ479954	1	a	c	t	c	t	t	t	a	c	c	t	c	c	a	t	t	c	g	g	c	a	a	g	c	t	g	t	a	g	g	t	t	a	t	g	g	g	a	a	a	g	g	c	a	t	t	c	t	g	t	g	a	g	g	g	g	c	c	t	t	c	t	c	t	a	g	c	t	t	c	c	g	a	t
DQ479958	1	a	c	c	c	t	t	t	a	c	c	t	c	c	a	t	t	c	g	g	c	a	a	g	c	t	g	t	a	g	g	t	t	a	t	g	g	g	a	a	a	g	g	c	a	t	t	c	t	g	t	g	a	a	g	g	g	c	c	t	t	c	t	c	t	a	g	c	t	t	c	c	g	a	t
DQ479959	1	a	c	c	c	t	t	t	a	c	c	t	c	c	a	t	t	c	g	g	c	a	a	g	c	t	g	t	a	g	g	t	t	a	t	g	g	g	a	a	a	g	g	c	a	t	t	c	t	g	t	g	a	a	g	g	g	c	c	t	t	c	t	c	t	a	g	c	t	t	c	c	g	a	t
DQ479961	1	a	c	t	c	t	t	t	a	c	c	t	c	a	a	t	t	c	g	g	c	a	a	g	c	t	g	t	a	g	g	t	t	a	t	g	g	g	a	a	a	g	g	c	a	t	t	c	t	g	t	g	a	g	g	g	g	c	c	t	t	c	t	c	t	a	g	c	t	t	c	c	g	a	t
DQ479962	1	a	c	t	c	t	t	t	a	c	c	t	c	a	a	t	t	c	g	g	c	a	a	g	c	t	g	t	a	g	g	t	t	a	t	g	g	g	a	a	a	g	g	c	a	t	t	c	t	g	t	g	a	g	g	g	g	c	c	t	t	c	t	c	t	a	g	c	t	t	c	c	g	a	t
DQ479963	1	a	c	t	c	t	t	t	a	c	c	t	c	a	a	t	t	c	g	g	c	a	a	g	c	t	g	t	a	g	g	t	t	a	t	g	g	g	a	a	a	g	g	c	a	t	t	c	t	g	t	g	a	g	g	g	g	c	c	t	t	c	t	c	t	a	g	c	t	t	c	c	g	a	t
DQ674250	1	a	c	t	c	t	t	t	a	c	c	t	c	c	a	t	t	c	g	g	c	a	a	g	c	t	g	t	a	g	g	t	t	a	t	g	g	g	a	a	a	g	g	c	a	t	t	c	t	g	t	g	a	a	g	g	g	c	c	t	t	c	t	c	t	a	g	c	t	t	c	c	g	a	t
KX262864	1	a	c	c	c	t	t	t	a	c	c	t	c	c	a	t	t	c	g	g	c	a	a	g	c	t	g	t	a	g	g	t	t	a	t	g	g	g	a	a	a	g	g	c	a	t	t	c	t	g	t	g	a	a	g	g	g	c	c	t	t	c	t	c	t	a	g	c	t	t	c	c	g	a	t
KX262865	1	a	c	t	c	t	t	t	a	c	c	t	c	c	a	t	t	c	g	g	c	a	a	g	c	t	g	t	a	g	g	t	t	a	t	g	g	g	a	a	a	g	g	c	a	t	t	c	t	g	t	g	a	a	g	g	g	c	c	t	t	c	t	c	t	a	g	c	t	t	c	c	g	a	t
KX262866	1	a	c	c	c	t	t	t	a	c	c	t	c	c	a	t	t	c	g	g	c	a	a	g	c	t	g	t	a	g	g	t	t	a	t	g	g	g	a	a	a	g	g	c	a	t	t	c	t	g	t	g	a	a	g	g	g	c	c	t	t	c	t	c	t	a	g	c	t	t	c	c	g	a	t
EU154348	1	a	c	t	c	t	t	t	a	c	c	t	c	c	a	t	t	c	g	g	c	a	a	g	c	t	g	t	a	g	g	t	t	a	t	g	g	g	a	a	a	g	g	c	a	t	t	c	t	g	t	g	a	g	g	g	g	c	c	t	t	c	t	c	t	a	g	c	t	t	c	c	g	a	t
JN704690	1	a	c	t	c	t	t	t	a	c	c	t	c	c	a	t	t	c	g	g	c	a	a	g	c	t	g	t	a	g	g	g	t	a	t	g	g	g	a	a	a	g	g	c	a	t	t	c	t	g	t	g	a	g	g	g	g	c	c	t	t	c	t	c	t	a	g	c	t	t	c	c	g	a	t
JN704691	1	a	c	t	c	t	t	t	a	c	c	t	c	c	a	t	t	c	g	g	c	a	a	g	c	t	g	t	a	g	g	t	t	a	t	g	g	g	a	a	a	g	g	c	a	t	t	c	t	g	t	g	a	g	g	g	g	c	c	t	t	c	t	c	t	a	g	c	t	t	c	c	g	a	t
JN704692	1	a	c	t	c	t	t	t	a	c	c	t	c	c	a	t	t	c	g	g	c	a	a	g	c	t	g	t	a	g	g	t	t	a	t	g	g	g	a	a	a	g	g	c	a	t	t	c	t	g	t	g	a	g	g	g	g	c	c	t	t	c	t	c	t	a	g	c	t	t	c	c	g	g	t
JN704693	1	a	c	t	c	t	t	t	a	c	c	t	c	c	a	t	t	c	g	g	c	a	a	g	c	t	g	t	a	g	g	t	t	a	t	g	g	g	a	a	a	g	g	c	a	t	t	c	t	g	t	g	a	g	g	g	g	c	c	t	t	c	t	c	t	a	g	c	t	t	c	c	g	a	t
JN704694	1	a	c	t	c	t	t	t	a	c	c	t	c	c	a	t	t	c	g	g	c	a	a	g	c	t	g	t	a	g	g	t	t	a	t	g	g	g	a	a	a	g	g	c	a	t	t	c	t	g	t	g	a	g	g	g	g	c	c	t	t	t	t	c	t	a	g	c	t	t	c	c	g	a	t
JN704695	1	a	c	t	c	t	t	t	a	c	c	t	c	c	a	t	t	c	g	g	c	a	a	g	c	t	g	t	a	g	g	t	t	a	t	g	g	g	a	a	a	g	g	c	a	t	t	c	t	g	t	g	a	g	g	g	g	c	c	t	t	c	t	c	t	a	g	c	t	g	c	c	g	a	t
JN704696	1	a	c	t	c	t	t	t	a	c	c	t	c	c	a	t	t	c	g	g	c	a	a	g	c	t	g	t	a	g	g	t	t	a	t	g	g	g	a	a	a	g	g	c	a	t	t	c	t	g	t	g	a	g	g	g	g	c	c	t	t	c	t	c	t	a	g	c	t	t	c	c	g	g	t
NC001348	1	a	c	t	c	t	t	t	a	c	c	t	c	c	a	t	t	c	g	g	c	a	a	g	c	t	g	t	a	g	g	t	t	a	t	g	g	g	a	a	a	g	g	c	a	t	t	c	t	g	c	g	a	g	g	g	g	c	c	t	t	c	t	c	t	a	g	c	t	t	c	c	g	a	t
KP771892	1	a	c	t	c	t	t	t	a	c	c	t	c	c	a	t	t	c	g	g	c	a	a	g	c	t	g	t	a	g	g	t	t	a	t	g	g	g	a	a	a	g	g	c	a	t	t	c	t	g	t	g	a	g	g	g	g	c	c	t	t	c	t	c	t	a	g	c	t	t	c	c	g	a	t
KP771894	1	a	c	t	c	t	t	t	a	c	c	t	c	c	a	t	t	c	g	g	c	a	a	g	c	t	g	t	a	g	g	t	t	a	t	g	g	g	a	a	a	g	g	c	a	t	c	c	t	g	t	g	a	g	g	g	g	c	c	t	t	c	t	c	t	a	g	c	t	t	c	c	g	a	t
KP771900	1	a	c	t	c	t	t	t	a	c	c	t	c	c	a	t	t	c	g	g	c	a	a	g	c	t	g	t	a	g	g	t	t	a	t	g	g	g	a	a	a	g	g	c	a	t	t	c	t	g	t	g	a	g	g	g	g	c	c	t	t	c	t	c	t	a	g	c	t	t	c	c	g	a	t
KP771901	1	a	c	t	c	t	t	t	a	c	c	t	c	c	a	t	t	c	g	g	c	a	a	g	c	t	g	t	a	g	g	t	t	a	t	g	g	g	a	a	a	g	g	c	a	t	t	c	t	g	t	g	a	g	g	g	g	c	c	t	t	c	t	c	t	a	g	c	t	t	c	c	g	a	t
KP771902	1	a	c	t	c	t	t	t	a	c	c	t	c	c	a	t	t	c	g	g	c	a	a	g	c	t	g	t	a	g	g	t	t	a	t	g	g	g	a	a	a	g	g	c	a	t	t	c	t	g	t	g	a	g	g	g	g	c	c	t	t	c	t	c	t	a	g	c	t	t	c	c	g	a	t
KP771908	1	a	c	c	c	t	t	t	a	c	c	t	c	c	a	t	t	c	g	g	c	a	a	g	c	t	g	t	a	g	g	t	t	a	t	g	g	g	a	a	a	g	g	c	a	t	t	c	t	g	t	g	a	a	g	g	g	c	c	t	t	c	t	c	t	a	g	c	t	t	c	c	g	a	t
KP771912	1	a	c	c	c	t	t	t	a	c	c	t	c	c	a	t	t	c	g	g	c	a	a	g	c	t	g	t	a	g	g	t	t	a	t	g	g	g	a	a	a	g	g	c	a	t	t	c	t	g	t	g	a	a	g	g	g	c	c	t	t	c	t	c	t	a	g	c	t	t	c	c	g	a	t
KP771913	1	a	c	c	c	t	t	t	a	c	c	t	c	c	a	t	t	c	g	g	c	a	a	g	c	t	g	t	a	g	g	t	t	a	t	g	g	g	a	a	a	g	g	c	a	t	t	c	t	g	t	g	a	a	g	g	g	c	c	t	t	c	t	c	t	a	g	c	t	t	c	c	g	a	t
KP771917	1	a	c	t	c	t	t	t	a	c	c	t	c	c	a	t	t	c	g	g	c	a	a	g	c	t	g	t	a	g	g	t	t	a	t	g	a	g	a	a	a	g	g	c	a	t	t	c	t	g	t	g	a	g	g	g	g	c	c	t	t	c	t	c	t	a	g	c	t	t	c	c	g	a	t
KP771918	1	a	c	t	c	t	t	t	a	c	c	t	c	c	a	t	t	c	g	g	c	a	a	g	c	t	g	t	a	g	g	t	t	a	t	g	g	g	a	a	a	g	g	c	a	t	t	c	t	g	t	g	a	g	g	g	g	c	c	t	t	c	t	c	t	a	g	c	t	t	c	c	g	a	t
KP771920	1	a	c	t	c	t	t	t	a	c	c	t	c	c	a	t	t	c	g	g	c	a	a	g	c	t	g	t	a	g	g	t	t	a	t	g	g	g	a	a	a	g	g	c	a	t	t	c	t	g	t	g	a	g	g	g	g	c	c	t	t	c	t	c	t	a	g	c	t	t	c	c	g	a	t
MH709361	1	a	c	c	c	t	t	t	a	c	c	t	t	c	a	t	t	c	g	g	c	a	a	g	c	t	g	t	a	g	g	t	t	a	t	g	g	g	a	a	a	g	g	c	a	t	t	c	t	g	t	g	a	a	g	g	g	c	c	t	t	c	t	c	t	a	g	c	t	t	c	c	g	a	t
KU926318	2	g	a	t	c	c	t	t	a	a	c	t	c	c	g	t	t	c	g	g	c	a	a	g	c	t	g	c	a	g	g	t	t	a	t	g	g	g	a	a	a	g	g	c	a	t	t	c	t	g	t	g	a	g	g	g	g	t	c	t	t	c	t	c	t	a	g	c	t	t	c	c	g	a	g
KU926319	2	a	c	t	c	c	t	t	a	c	c	t	c	c	a	t	t	c	g	g	c	a	a	g	c	t	g	t	a	g	g	t	t	a	t	g	g	t	a	a	a	g	g	c	a	t	t	c	t	g	t	g	a	g	g	g	g	t	c	t	t	c	t	c	t	a	g	c	t	t	c	c	g	a	t
KU926320	2	a	c	t	c	c	t	t	a	c	c	t	c	c	a	c	t	c	g	g	c	a	a	g	c	t	g	t	a	g	g	t	t	a	t	g	g	t	a	a	a	g	g	c	a	t	t	c	t	g	t	g	a	g	g	g	g	t	c	t	t	c	t	c	t	a	g	c	c	t	c	c	g	a	t
KU926321	2	a	c	t	c	c	t	t	a	c	c	t	c	c	a	t	t	c	g	g	c	a	a	g	c	t	g	t	a	g	g	t	t	a	t	g	g	g	a	a	a	g	g	c	a	t	t	c	t	g	t	g	a	g	g	g	g	t	c	t	t	c	t	c	t	a	g	c	t	t	c	c	g	a	t
KU926322	2	a	c	t	c	c	t	t	a	c	c	t	c	c	a	t	t	c	g	g	c	a	a	g	c	t	g	t	a	g	g	t	t	a	t	g	g	g	a	a	a	g	g	c	a	t	t	c	t	g	t	g	a	g	g	g	g	t	c	t	t	c	t	c	t	a	g	c	t	t	c	c	g	a	t
KC847290	2	a	c	t	c	c	t	t	a	c	c	t	c	c	a	t	t	c	g	g	c	a	a	g	c	t	g	t	a	g	g	t	t	a	t	g	g	g	a	a	a	g	g	c	a	t	t	c	t	g	t	g	a	g	g	g	g	t	c	t	t	c	t	c	t	a	g	c	t	t	c	c	g	a	t
KJ767491	2	a	a	t	c	c	t	t	a	a	c	t	c	c	a	t	t	c	g	g	c	a	a	g	c	t	g	t	a	g	g	t	t	a	t	g	g	g	a	a	a	g	g	c	a	t	t	c	t	g	t	g	a	g	g	g	g	t	c	t	t	c	t	c	t	a	g	c	t	t	c	c	g	a	g
KJ767492	2	a	c	t	c	c	t	t	a	c	c	t	c	c	a	t	t	c	g	g	c	a	a	g	c	t	g	t	a	g	g	t	t	a	t	g	g	g	a	a	a	g	g	c	a	t	t	c	t	g	t	g	a	g	g	g	g	t	c	t	t	c	t	c	t	a	g	c	t	t	c	c	g	a	t
KJ808816	2	a	c	t	c	c	t	t	a	c	c	t	c	c	a	t	t	c	g	g	c	a	a	g	c	t	g	t	a	g	g	t	t	a	t	g	g	g	a	a	a	g	g	c	a	t	t	c	t	g	t	g	a	g	g	g	g	t	c	t	t	c	t	c	t	a	g	c	t	t	c	c	g	a	t
KP771925	2	a	c	t	c	c	t	t	a	c	c	t	c	c	a	t	t	c	g	g	c	a	a	g	c	t	g	t	a	g	g	t	t	a	t	g	g	g	a	a	a	g	g	c	a	t	t	c	t	g	t	g	a	g	g	g	g	t	c	t	t	c	t	c	t	a	g	c	t	t	t	c	g	a	t
KU926315	2	a	a	t	c	c	t	t	a	a	c	t	c	c	a	t	t	c	g	g	c	a	a	g	c	t	g	t	a	g	g	t	t	a	t	g	g	g	a	a	a	g	g	c	a	t	t	c	t	g	t	g	a	g	g	g	g	t	c	t	t	c	t	c	t	a	g	c	t	t	c	c	g	a	g
KU926316	2	a	a	t	c	c	t	t	a	a	c	t	c	c	a	t	t	c	g	g	c	a	a	g	c	t	g	t	a	g	g	t	t	a	t	g	g	g	a	a	a	g	g	c	a	t	t	c	t	g	t	g	a	g	g	g	g	t	c	t	t	c	t	c	t	a	g	c	t	t	c	c	g	a	g
KU926317	2	a	a	t	c	c	t	t	a	a	c	t	c	c	a	t	t	c	g	g	c	a	a	g	c	t	g	t	a	g	g	t	t	a	t	g	g	g	a	a	a	g	g	c	a	t	t	c	t	g	t	g	a	g	g	g	g	t	c	t	t	c	t	c	t	a	g	t	t	t	c	c	g	a	g
KY037796	2	a	c	t	c	c	t	t	a	c	a	t	c	c	a	t	t	c	g	g	c	a	a	g	c	t	g	t	a	g	g	t	t	a	t	g	g	g	a	a	a	g	g	c	a	t	t	c	t	g	t	g	a	g	g	g	g	t	c	t	t	c	t	c	t	a	g	c	t	t	c	c	g	a	t
MF004348	2	a	c	t	c	c	t	t	a	c	c	t	c	c	a	t	t	c	g	g	c	a	a	g	c	t	g	t	a	g	g	t	t	a	t	g	g	g	a	a	a	g	g	c	a	t	t	c	t	g	t	g	a	g	g	g	g	t	c	t	t	c	t	c	t	a	g	c	t	t	c	c	g	a	t
MH709309	2	a	c	t	c	c	t	t	a	c	c	t	c	c	a	t	t	c	g	g	c	a	a	g	c	t	g	t	a	g	g	t	t	a	t	g	g	g	a	a	a	g	g	c	a	t	t	c	t	g	t	g	a	g	g	g	g	t	c	t	t	c	t	c	t	a	g	c	t	t	c	c	g	a	t
MH709310	2	a	c	t	c	c	t	t	a	c	c	t	c	c	a	t	t	c	g	g	c	a	a	g	c	t	g	t	a	g	g	t	t	a	t	g	g	g	a	a	a	g	g	c	a	t	t	c	t	g	t	g	a	g	g	g	g	t	c	t	t	c	t	t	t	a	g	c	t	t	c	c	g	a	t
MH709311	2	a	c	t	c	c	t	t	a	c	c	t	c	c	a	t	t	c	g	g	c	a	a	g	c	g	g	t	a	g	g	t	t	a	t	g	g	g	a	a	a	g	t	c	a	t	t	c	t	g	t	g	a	g	g	g	g	t	c	t	t	c	t	c	t	a	g	c	t	t	c	c	g	a	t
MH709313	2	a	c	t	c	c	t	t	a	c	c	t	c	c	a	t	t	c	g	g	c	a	a	g	c	t	g	t	a	g	g	t	t	a	t	g	g	g	a	a	a	g	g	c	a	t	t	c	t	g	t	g	a	g	g	g	g	t	c	c	t	c	t	c	t	a	g	c	t	t	c	c	g	a	t
MH709314	2	a	c	t	c	c	t	t	a	c	c	t	c	c	a	t	t	c	g	g	c	a	a	g	c	t	g	t	a	g	g	t	t	g	t	g	g	g	a	a	a	g	g	c	a	t	t	c	t	g	t	g	a	g	g	g	g	t	c	t	t	c	t	t	t	a	g	c	t	t	c	c	g	a	t
MH709315	2	a	c	t	c	c	t	t	a	c	c	t	c	c	a	t	t	c	g	g	c	a	a	g	c	t	g	t	a	g	g	t	t	g	t	g	g	g	a	a	a	g	g	c	a	t	t	c	t	g	t	g	a	g	g	g	g	t	c	t	t	c	t	t	t	a	g	c	t	t	c	c	g	a	t
MH709319	2	a	c	t	c	c	t	t	a	c	c	t	c	c	a	t	c	c	g	g	c	a	a	g	c	t	g	t	a	g	g	t	t	g	t	g	g	g	a	a	a	g	g	c	a	t	t	c	t	g	t	g	a	g	g	g	g	t	c	t	t	c	t	t	t	a	g	c	t	t	c	c	g	a	t
MH709320	2	a	c	t	c	c	t	t	a	c	c	t	c	c	a	t	t	c	g	g	c	a	a	g	c	t	g	t	a	g	g	t	t	a	t	g	g	g	a	a	a	g	g	c	a	t	t	c	t	g	t	g	a	g	g	g	g	t	c	t	t	c	t	t	t	a	g	c	t	t	c	c	g	a	t
MH709329	2	a	c	t	c	c	t	t	a	c	c	t	c	c	a	t	t	c	g	g	c	a	a	g	c	t	g	t	a	g	g	t	t	g	t	g	g	g	a	a	a	g	g	c	a	t	t	c	t	g	t	g	a	g	g	g	g	t	c	t	t	c	t	t	t	a	g	c	t	t	c	c	g	a	t
MH709331	2	a	c	t	c	c	t	t	a	c	c	t	c	c	a	t	t	c	g	g	c	a	a	g	c	t	g	t	a	g	g	t	t	a	t	g	g	g	a	a	a	g	g	c	a	t	t	c	t	g	t	g	a	g	g	g	g	t	c	t	t	c	t	c	t	a	g	c	t	t	c	c	g	a	t
MH709333	2	a	c	t	c	c	t	t	a	c	c	t	c	c	a	t	t	c	g	g	c	a	a	g	c	t	g	t	a	g	g	t	t	g	t	g	g	g	a	a	a	g	g	c	a	t	t	c	t	g	t	g	a	g	g	g	g	t	c	t	t	c	t	t	t	a	g	c	t	t	c	c	g	a	t
MH709337	2	a	c	t	c	c	t	t	a	c	c	t	c	c	a	t	t	c	g	g	c	a	a	g	c	t	g	t	a	g	g	t	t	a	t	g	g	g	a	a	a	g	g	c	a	t	t	c	t	g	t	g	a	g	g	g	g	t	c	t	t	c	t	c	t	a	g	c	t	t	c	c	g	a	t
MH709339	2	a	c	t	c	c	t	t	a	c	c	t	c	c	a	t	t	c	g	g	c	a	a	g	c	t	g	t	a	g	g	t	t	a	t	g	g	g	g	a	a	g	g	c	a	t	t	c	t	g	t	g	a	g	g	g	g	t	c	t	t	c	t	c	t	a	g	c	t	t	c	c	g	a	t
MH709344	2	a	c	t	c	c	t	t	a	c	c	t	c	c	a	t	t	t	g	g	c	a	a	g	c	t	g	t	a	g	g	t	t	a	t	g	g	g	a	a	a	g	g	c	a	t	t	c	t	g	t	g	a	g	g	g	g	t	c	t	t	c	t	c	t	a	g	c	t	t	c	c	g	a	t
MH709348	2	a	c	t	c	c	t	t	a	c	c	t	c	c	a	t	t	c	g	g	c	a	a	g	c	t	g	t	a	g	g	t	t	a	t	g	g	g	a	a	a	g	g	c	a	t	t	c	t	g	t	g	a	g	g	g	g	t	c	t	t	c	t	c	t	a	g	c	t	t	c	c	g	a	t
MH709355	2	a	c	t	c	c	t	t	a	c	c	t	c	c	a	t	t	c	g	g	c	a	a	g	c	t	g	t	a	g	g	t	t	a	t	g	g	g	a	a	g	g	g	c	a	t	t	c	t	g	t	g	a	g	g	g	g	t	c	t	t	c	t	c	t	a	g	c	t	t	c	c	g	a	t
MH709359	2	a	c	t	c	c	t	t	a	c	c	t	c	c	a	t	t	c	g	g	c	a	a	g	c	t	g	t	a	g	g	t	t	a	t	g	g	g	a	a	a	g	g	c	a	t	t	c	t	g	t	g	a	g	g	g	g	t	c	t	t	c	t	c	t	a	g	c	t	t	c	c	g	a	t
MH709363	2	a	c	t	c	c	t	t	a	c	c	t	c	c	a	t	t	c	g	g	c	a	a	g	c	t	g	t	a	g	g	t	t	a	t	g	g	g	a	a	a	g	g	c	a	t	t	c	t	g	t	g	a	g	g	g	g	t	c	t	t	c	t	t	t	a	g	c	t	t	c	c	g	a	t
MH709366	2	a	c	t	c	c	t	t	a	c	c	t	c	c	a	t	t	c	g	g	c	a	a	g	c	t	g	t	a	g	g	t	t	a	t	g	g	g	a	a	a	g	g	c	a	t	t	c	t	g	t	g	a	g	g	g	g	t	c	t	t	c	t	c	t	a	g	c	t	t	c	c	g	a	t
MH709376	2	a	c	t	c	c	t	c	a	c	c	t	c	c	a	t	t	c	g	g	c	a	a	g	c	t	g	t	a	g	g	t	t	a	t	g	g	g	a	a	a	g	g	c	a	t	t	c	t	g	t	g	a	g	g	g	g	t	c	t	t	c	t	c	t	a	g	c	t	t	c	c	g	a	t
MH709377	2	a	c	t	c	c	t	t	a	c	c	t	c	c	a	t	t	c	g	g	c	a	a	g	c	t	g	t	a	g	g	t	t	a	t	g	g	g	a	a	a	g	g	c	a	t	t	c	t	g	t	g	a	g	g	g	g	t	c	t	t	c	t	c	t	a	g	c	t	t	c	c	g	a	t
**MW316406**	2	a	c	t	c	c	t	t	a	c	c	t	c	c	a	t	t	c	g	g	c	a	a	g	c	t	g	t	a	g	g	t	t	a	t	g	g	g	a	a	a	g	g	c	a	t	t	c	t	g	t	g	a	g	g	g	g	t	c	t	t	c	t	c	t	a	g	c	t	t	c	c	g	a	t
AJ871403	3	a	c	t	c	c	g	t	a	c	c	t	c	c	a	t	t	c	g	g	c	a	a	t	c	t	g	t	a	g	g	t	t	a	t	g	g	g	a	a	a	g	g	c	a	t	t	c	c	g	t	g	g	g	g	g	g	c	c	t	t	c	c	c	t	a	g	c	t	t	c	c	g	a	t
DQ479955	3	a	c	t	c	c	g	t	a	c	c	t	c	c	a	t	t	c	g	g	c	a	a	t	c	t	g	t	a	g	g	t	t	a	t	g	g	g	a	a	a	g	g	c	a	t	t	c	c	g	t	g	g	g	g	g	g	c	c	t	t	c	c	c	t	a	g	c	t	t	c	c	g	a	t
DQ479956	3	a	c	t	c	c	g	t	a	c	c	t	c	c	a	t	t	c	g	g	c	a	a	t	c	t	g	t	a	g	g	t	t	a	t	g	g	g	a	a	a	g	g	c	a	t	t	c	c	g	t	g	g	g	g	g	g	c	c	t	t	c	c	c	t	a	g	c	t	t	c	c	g	a	t
DQ479957	3	a	c	t	c	c	g	t	a	c	c	t	c	c	a	t	t	c	g	g	c	a	a	t	c	t	g	t	a	g	g	t	t	a	t	g	g	g	a	a	a	g	g	c	a	t	t	c	c	g	t	g	g	g	g	g	g	c	c	t	t	c	c	c	t	a	t	c	t	t	c	c	g	a	t
JN704699	3	a	c	t	c	c	g	t	a	c	c	t	c	c	a	t	t	c	g	g	c	a	a	t	c	t	g	t	a	g	g	t	t	a	t	g	g	g	a	a	a	g	g	c	a	t	t	c	c	g	t	g	g	g	g	g	g	c	c	t	t	c	c	c	t	a	g	c	t	t	c	c	g	a	t
JN704700	3	a	c	t	c	c	g	t	a	c	c	t	c	c	a	t	t	c	g	g	c	a	a	t	c	t	g	t	a	g	g	t	t	a	t	g	g	g	a	a	a	g	g	c	a	t	t	c	c	g	t	g	g	g	g	g	g	c	c	t	t	c	c	c	t	a	g	c	t	t	c	c	g	a	t
JN704701	3	a	c	t	c	c	g	t	a	c	c	t	c	c	a	t	t	c	g	g	c	a	a	t	c	t	g	t	a	g	g	t	t	a	t	g	g	g	a	a	a	g	g	c	a	t	t	c	c	g	t	g	g	g	g	g	g	c	c	t	t	c	c	c	t	a	g	c	t	t	c	c	g	a	t
JN704702	3	a	c	t	c	c	g	t	a	c	c	t	c	c	a	t	t	c	g	g	c	a	a	t	c	t	g	t	a	g	g	t	t	a	t	g	g	g	a	a	a	g	g	c	a	t	t	c	c	g	t	g	g	g	g	g	g	c	c	t	t	c	c	c	t	a	g	c	t	t	c	c	g	a	t
JN704703	3	a	c	t	c	c	g	t	a	c	c	t	c	c	a	t	t	c	g	g	c	a	a	t	c	t	g	t	a	g	g	t	t	a	t	g	g	g	a	a	a	g	g	c	a	t	t	c	c	g	t	g	g	g	g	g	g	c	c	t	t	c	c	c	t	a	g	c	t	t	c	c	g	a	t
KP771891	3	a	c	t	c	c	g	t	a	c	c	t	c	c	a	t	t	c	g	g	c	a	a	t	c	t	g	t	a	g	g	t	t	a	t	g	g	g	a	a	a	g	g	c	a	t	t	c	c	g	t	a	g	g	g	g	g	c	c	t	t	c	c	c	t	a	g	c	t	t	c	c	g	a	t
KP771893	3	a	c	t	c	c	g	t	a	c	c	t	c	c	a	t	t	c	g	g	c	a	a	t	c	t	g	t	a	g	g	t	t	a	t	g	g	g	a	a	a	g	g	c	g	t	t	c	c	g	t	g	g	g	g	g	g	c	c	t	t	c	c	c	t	a	g	c	t	t	c	c	g	a	t
KP771895	3	a	c	t	c	c	g	t	a	c	c	t	c	c	a	t	t	c	g	g	c	a	a	t	c	t	g	t	a	g	g	t	t	a	t	g	g	g	a	a	a	g	g	c	a	t	t	c	c	g	t	g	g	g	g	g	g	c	c	t	t	c	c	c	t	a	g	c	t	t	c	c	g	a	t
KP771896	3	a	c	t	c	c	g	t	a	c	c	t	c	c	a	t	t	c	g	g	c	a	a	t	c	t	g	t	a	g	g	t	t	a	t	g	g	g	a	a	a	g	g	c	a	t	t	c	c	g	t	g	g	g	g	g	g	c	t	t	t	c	c	c	t	a	g	c	t	t	c	c	g	a	t
KP771897	3	a	c	t	c	c	g	t	a	c	c	t	c	c	a	t	t	c	g	g	c	a	a	t	c	t	g	t	a	g	g	t	t	a	t	g	g	g	a	a	a	g	g	c	a	t	t	c	c	g	t	g	g	g	g	g	g	c	c	t	t	c	c	c	t	a	g	c	t	t	c	c	g	a	t
KP771899	3	a	c	t	c	c	g	t	a	c	c	t	c	c	a	t	t	c	g	g	c	a	a	t	c	t	g	t	a	g	g	t	t	a	t	g	g	g	a	a	a	g	g	c	a	t	t	c	c	g	t	g	g	g	g	g	g	c	c	t	t	c	c	c	t	a	g	c	t	t	c	c	g	a	t
KP771903	3	a	c	t	c	c	g	t	a	c	c	t	c	c	a	t	t	c	g	g	c	a	a	t	c	t	g	t	a	g	g	t	t	a	t	g	g	g	a	a	a	g	g	c	a	t	t	c	c	g	t	a	g	g	g	g	g	c	c	t	t	c	c	c	t	a	g	c	t	t	c	c	g	a	t
KP771907	3	a	c	t	c	c	g	t	a	c	c	t	c	c	a	t	t	c	g	g	c	a	a	t	c	t	g	t	a	g	g	t	t	a	t	g	g	g	a	a	a	g	g	c	a	t	t	c	c	g	t	g	g	g	g	g	g	c	c	t	t	c	c	c	t	a	g	c	t	t	c	c	g	a	t
KP771910	3	a	c	t	c	c	g	t	a	c	c	t	c	c	a	t	t	c	g	g	c	a	a	t	c	t	g	t	a	g	g	t	t	a	t	g	g	g	a	a	a	g	g	c	a	t	t	c	c	g	t	g	g	g	g	g	g	c	c	t	t	c	c	c	t	a	g	c	t	t	c	c	g	a	t
KP771915	3	a	c	t	c	c	g	t	a	c	c	t	c	c	a	t	t	c	g	g	c	a	a	t	c	t	g	t	a	g	g	t	t	a	g	g	g	g	a	a	a	g	g	c	a	t	t	c	c	g	t	g	g	g	g	g	g	c	c	t	t	c	c	c	t	c	g	c	t	t	c	c	g	a	t
KP771916	3	a	c	t	c	c	g	t	a	c	c	t	c	c	a	t	t	c	g	g	c	a	a	t	c	t	g	t	a	g	g	t	t	a	t	g	g	g	a	a	a	g	g	c	a	t	t	c	c	g	t	g	g	g	g	g	g	c	c	t	t	c	c	c	t	a	g	c	t	t	c	c	g	a	t
KP771921	3	a	c	t	c	c	g	t	a	c	c	t	c	c	a	t	t	c	g	g	c	a	a	t	c	t	g	t	a	g	c	t	t	a	t	g	g	g	a	a	a	g	g	c	a	t	t	c	c	g	t	g	g	g	g	g	g	c	c	t	t	c	c	c	t	a	g	c	t	t	c	c	g	a	t
KP771923	3	a	c	t	t	c	g	t	a	c	c	t	c	c	a	t	t	c	g	g	c	a	a	t	c	t	g	t	a	g	g	t	t	a	t	g	g	g	a	a	a	g	g	c	a	t	t	c	c	g	t	g	g	g	g	g	g	c	c	t	t	c	c	c	t	a	g	c	t	t	c	c	g	a	t
KP771889	3	a	c	t	c	c	g	t	a	c	c	t	c	c	a	t	t	c	g	g	c	a	a	t	c	t	g	t	a	g	g	t	t	a	g	g	g	g	a	a	a	g	g	c	a	t	t	c	c	g	t	g	g	g	g	g	g	c	c	t	t	c	c	c	t	c	g	c	t	t	c	c	g	a	t
KU926311	3	a	c	t	c	c	g	t	a	c	c	t	c	c	a	t	t	c	g	g	c	a	a	t	c	t	g	t	a	g	g	t	t	a	t	g	g	g	a	a	a	g	g	c	a	t	t	c	c	g	t	g	g	g	g	g	g	c	c	t	t	c	c	c	t	a	g	c	t	t	c	c	g	a	t
JQ911599	3	a	c	t	c	c	g	t	a	c	c	t	c	c	a	t	t	c	g	g	c	a	a	t	c	t	g	t	a	g	g	t	t	a	t	g	g	g	a	a	a	g	g	c	a	t	t	c	c	g	t	g	g	g	g	g	g	c	c	t	t	c	c	c	t	a	g	c	t	t	c	c	g	a	t
MH709317	3	a	c	t	c	c	t	t	a	c	c	t	c	c	a	t	t	c	g	t	c	a	a	t	c	t	g	t	a	g	g	t	t	a	t	g	g	g	a	a	a	g	g	a	a	t	t	c	c	g	t	g	g	g	g	g	g	c	c	t	t	c	c	c	t	a	g	c	t	t	c	c	g	a	t
MH709346	3	a	c	t	c	c	t	t	a	c	c	t	c	c	a	t	t	c	g	t	c	a	a	t	c	t	g	t	a	g	g	t	t	a	t	g	g	g	a	a	a	g	g	a	a	t	t	c	c	g	t	g	g	g	g	g	g	c	c	t	t	c	c	c	t	a	g	c	t	t	c	c	g	a	t
MH709375	3	a	c	t	c	c	t	t	a	c	c	t	c	c	a	t	t	c	g	g	c	a	a	t	c	t	g	t	a	g	g	t	t	a	t	g	g	g	a	a	a	g	g	a	a	t	t	c	c	g	t	g	g	g	g	g	g	c	c	t	t	c	c	c	t	a	g	c	t	t	c	c	g	a	t
DQ452050	4	a	c	t	c	c	t	t	a	c	c	g	c	c	a	t	t	c	g	g	c	a	a	g	c	t	g	t	a	g	g	t	t	a	t	g	g	g	a	a	a	g	g	c	a	t	t	c	t	g	t	g	a	g	g	g	g	t	c	t	t	c	t	c	t	a	g	c	t	t	c	c	g	a	t
DQ479960	4	a	c	t	c	c	t	t	a	c	c	g	c	c	a	t	t	c	g	g	c	a	a	g	c	t	g	t	a	g	g	t	t	a	t	g	g	g	a	a	a	g	g	c	a	t	t	c	t	g	t	g	a	g	g	g	g	t	c	t	t	c	t	c	t	a	g	c	t	t	c	c	g	a	t
KP771898	4	a	c	t	c	c	t	t	a	c	c	g	c	c	a	t	t	c	g	g	c	a	a	g	c	t	g	t	a	g	g	t	t	a	t	g	g	g	a	a	a	g	g	c	a	t	t	c	t	g	t	g	a	g	g	g	g	t	c	t	t	c	t	c	t	a	g	c	t	t	c	c	g	a	t
MH709316	4	a	c	t	c	c	t	t	a	c	c	g	c	c	a	t	t	c	g	g	c	a	a	g	c	t	g	t	a	g	g	t	a	a	t	g	g	g	a	a	a	g	g	c	a	t	t	c	t	g	t	g	a	g	g	g	g	t	c	t	t	c	t	c	t	a	g	c	t	t	c	c	g	a	t
MH709318	4	a	c	t	c	c	t	t	a	c	c	g	c	c	a	t	t	c	g	g	c	a	a	g	c	t	t	t	a	g	g	t	t	a	t	g	g	g	a	a	a	g	g	c	a	t	t	c	t	g	t	g	a	g	g	g	g	t	c	t	g	c	t	c	t	a	g	c	t	t	c	c	g	a	t
MH709322	4	a	c	t	c	c	t	t	a	c	c	g	c	c	a	t	t	c	g	g	c	a	a	g	c	t	t	t	a	g	g	t	t	a	t	g	g	g	a	a	a	g	g	c	a	t	t	c	t	g	t	g	a	g	g	g	g	t	c	t	g	c	t	c	t	a	g	c	t	t	c	c	g	a	t
MH709323	4	a	c	t	c	c	t	t	a	c	c	g	c	c	a	t	t	c	g	g	c	a	a	g	c	t	t	t	a	g	g	t	t	a	t	g	g	g	a	a	a	g	g	c	a	t	t	c	t	g	t	g	a	g	g	g	g	t	c	t	t	c	t	c	t	a	g	c	t	t	c	c	g	a	t
MH709326	4	a	c	t	c	c	t	t	a	c	c	g	c	c	a	t	t	c	g	g	c	a	a	g	c	t	t	t	a	g	g	t	t	a	t	g	g	g	a	a	a	g	g	c	a	t	t	c	t	g	t	g	a	g	g	g	g	t	c	t	g	c	t	c	t	a	g	c	t	t	c	c	g	a	t
MH709341	4	a	c	t	c	c	t	t	a	c	c	g	c	c	a	t	t	c	g	g	c	a	a	g	c	t	g	t	a	g	g	t	t	a	t	g	g	g	a	a	a	g	g	c	a	t	t	c	t	g	t	g	a	g	g	g	g	t	c	t	t	c	t	c	t	a	g	c	t	t	c	c	g	a	t
MH709357	4	a	c	t	c	c	t	t	a	c	c	g	c	c	a	t	t	c	g	g	c	a	a	g	c	t	g	t	a	g	g	t	t	a	t	g	g	g	a	a	a	g	g	c	a	t	t	c	t	g	t	g	a	g	g	g	g	t	c	t	t	c	t	c	t	a	g	c	t	t	c	c	g	a	t
MH709364	4	a	c	t	c	c	t	t	a	c	c	g	c	c	a	t	t	c	g	g	c	a	a	g	c	t	g	t	a	g	g	t	t	a	t	g	g	g	a	a	a	g	g	c	a	t	t	c	t	g	t	g	a	g	g	g	g	t	c	t	t	c	t	c	t	a	g	c	t	t	c	c	g	a	t
MH709370	4	a	c	t	c	c	t	t	a	c	c	g	c	c	a	t	t	c	g	g	c	a	a	g	c	t	g	t	a	g	g	t	t	a	t	g	g	g	a	a	a	g	g	c	a	t	t	c	t	g	t	g	a	g	g	g	g	t	c	t	t	c	t	c	t	a	g	c	t	t	c	c	g	a	t
MH709371	4	a	c	t	c	c	t	t	a	c	c	g	c	c	a	t	t	c	g	g	c	a	a	g	c	t	g	t	a	g	g	t	t	a	t	g	g	g	a	a	a	g	g	c	a	t	t	c	t	g	t	g	a	g	g	g	g	t	c	t	t	c	t	c	t	a	g	c	t	t	c	c	g	a	t
MH709372	4	a	c	t	c	c	t	t	a	c	c	g	c	c	a	t	t	c	g	g	c	a	a	g	c	t	g	t	a	g	g	t	t	a	t	g	g	g	a	a	a	g	g	c	a	t	t	c	t	g	t	g	a	g	g	g	g	t	c	t	t	c	t	c	t	a	g	c	t	t	c	c	g	a	t
DQ457052	5	a	c	t	c	c	t	t	a	c	c	t	c	c	a	t	t	c	g	g	c	a	a	g	c	t	g	t	a	g	g	t	t	a	t	g	g	g	a	a	a	g	g	c	a	t	t	c	c	g	t	g	g	g	g	g	g	c	c	t	t	c	c	c	t	a	g	c	t	t	c	c	g	a	t
JN704704	5	a	c	t	c	c	t	t	g	c	c	t	c	c	a	t	t	c	g	g	c	a	a	g	c	t	g	t	a	g	g	t	t	a	t	g	g	g	a	a	a	g	g	c	a	t	t	c	t	g	t	g	g	g	g	g	g	c	c	t	t	c	c	c	t	a	g	c	t	t	c	c	g	a	t
JN704705	5	a	c	t	c	c	t	t	a	c	c	t	c	c	a	t	t	c	g	g	c	a	a	g	c	t	g	t	a	g	g	t	t	a	t	g	g	g	a	a	a	g	g	c	a	t	t	c	c	g	t	g	g	g	g	g	g	c	c	t	t	c	c	c	t	a	g	c	t	t	c	c	g	a	t
JN704706	5	a	c	t	c	c	t	t	a	c	c	t	c	c	a	t	t	c	g	g	c	a	a	g	c	t	g	t	a	g	g	t	t	a	t	g	g	g	a	a	a	g	g	c	a	t	t	c	c	g	t	g	g	g	g	g	g	c	c	t	t	c	c	c	t	a	g	c	t	t	c	c	g	a	t
JN704707	5	a	c	t	c	c	t	t	a	c	c	t	c	c	a	t	t	c	g	g	c	a	a	g	c	t	g	t	a	g	g	t	t	a	t	g	g	g	a	a	a	g	g	c	a	t	t	c	c	g	t	g	g	g	g	a	g	c	c	t	t	c	c	c	t	a	g	c	t	t	c	c	g	a	t
JN704708	5	a	c	t	c	c	t	t	a	c	c	t	c	c	a	t	t	c	g	g	c	a	a	g	c	t	g	t	a	g	g	t	t	a	t	g	g	g	a	a	a	g	g	c	a	t	t	c	c	g	t	g	g	g	g	g	g	c	c	t	t	c	c	c	t	a	g	c	t	t	c	c	g	a	t
KP771904	5	a	c	t	c	c	t	t	a	c	c	t	c	c	a	t	t	c	a	g	c	a	a	g	c	t	g	t	a	g	g	t	t	a	t	g	g	g	a	a	a	g	g	c	a	t	t	c	c	g	t	g	g	g	a	g	g	c	c	t	t	c	c	c	t	a	g	c	t	t	c	t	g	a	t
KP771905	5	a	c	t	c	c	t	t	a	c	c	t	c	c	a	t	t	c	g	g	c	a	a	g	c	t	g	t	a	g	g	t	t	a	t	g	g	g	a	a	a	g	g	c	a	t	t	c	c	g	t	g	g	g	g	g	g	c	c	t	t	c	c	c	c	a	g	c	t	t	c	c	g	a	t
KP771906	5	a	c	t	c	c	t	t	a	c	c	t	c	c	a	t	t	c	g	g	c	a	a	g	c	t	g	t	a	g	g	t	t	a	t	g	g	g	a	a	a	g	g	c	a	t	t	c	c	g	t	g	g	g	g	g	g	c	c	t	t	c	c	c	c	a	g	c	t	t	c	c	g	a	t
KP771919	5	a	c	t	c	c	t	t	a	c	c	t	c	c	a	t	t	c	a	g	c	a	a	g	c	t	g	t	a	g	g	t	t	a	t	g	g	g	a	a	a	g	g	c	a	t	t	c	c	g	t	g	g	g	g	g	g	c	c	t	t	c	c	c	t	a	g	c	t	t	c	t	g	a	t
KP771922	5	a	c	t	c	c	t	t	a	c	c	t	c	c	a	t	t	c	g	g	c	a	a	g	c	t	g	t	a	g	g	t	t	a	t	g	g	g	a	a	a	g	g	c	a	t	t	c	c	g	t	g	g	g	g	g	g	c	c	t	t	c	c	c	t	a	g	c	t	t	c	c	g	a	t
KP771924	5	a	c	t	c	c	t	t	a	c	c	t	c	c	a	t	t	c	g	g	c	a	a	g	c	t	g	t	a	g	g	t	t	a	t	g	g	g	a	a	a	g	g	c	a	t	t	c	c	g	t	g	g	g	g	g	g	c	c	t	t	c	c	c	t	a	g	c	t	t	c	c	g	a	t
KM355696	5	a	c	t	c	c	t	t	a	c	c	t	c	c	a	t	t	c	g	g	c	a	a	g	c	t	g	t	a	g	g	t	t	a	t	g	g	g	a	a	a	g	g	c	a	t	t	c	c	g	t	g	g	g	g	g	g	c	c	t	t	c	c	c	t	a	g	c	t	t	c	c	g	a	t
KM355697	5	a	c	t	c	c	t	t	a	c	c	t	c	c	a	t	t	c	g	g	c	a	a	g	c	t	g	t	a	g	g	t	t	a	t	g	g	g	a	a	a	g	g	c	a	t	t	c	c	g	t	g	g	g	g	g	g	c	c	t	t	c	c	c	t	a	g	c	t	t	c	c	g	a	t
KM355698	5	a	c	t	c	c	t	t	a	c	c	t	c	c	a	t	t	c	g	g	c	a	a	g	c	t	g	t	a	g	g	t	t	a	t	g	g	g	a	a	a	g	g	c	a	t	t	c	c	g	t	g	g	g	g	g	g	c	c	t	t	c	c	c	t	a	g	c	t	t	c	c	g	a	t
KM355699	5	a	c	t	c	c	t	t	a	c	c	t	c	c	a	t	t	c	g	g	c	a	a	g	c	t	g	t	a	g	g	t	t	a	t	g	g	g	a	a	a	g	g	c	a	t	t	c	c	g	t	g	g	g	g	g	g	c	c	t	t	c	c	c	t	a	g	c	t	t	c	c	g	a	t
KM355701	5	a	c	t	c	c	t	t	a	c	c	t	c	c	a	t	t	c	g	g	c	a	a	g	c	t	g	t	a	g	g	t	t	a	t	g	g	g	a	a	a	g	g	c	a	t	t	c	c	g	t	g	g	g	g	g	g	c	c	t	t	c	c	c	t	a	g	c	t	t	c	c	g	a	t
KM355702	5	a	c	t	c	c	t	t	a	c	c	t	c	c	a	t	t	c	g	g	c	a	a	g	c	t	g	t	a	g	g	t	t	a	t	g	g	g	a	a	a	g	g	c	a	t	t	c	c	g	t	g	g	g	g	g	g	c	c	t	t	c	c	c	t	a	g	c	t	t	c	c	g	a	t
KM355703	5	a	c	t	c	c	t	t	a	c	c	t	c	c	a	t	t	c	g	g	c	a	a	g	c	t	g	t	a	g	g	t	t	a	t	g	g	g	a	a	a	g	g	c	a	t	t	c	c	g	t	g	g	g	g	g	g	c	c	t	t	c	c	c	t	a	g	c	t	t	c	c	g	a	t
KM355704	5	a	c	t	c	c	t	t	a	c	c	t	c	c	a	t	t	c	g	g	c	a	a	g	c	t	g	t	a	g	g	t	t	a	t	g	g	g	a	a	a	g	g	c	a	t	t	c	c	g	t	g	g	g	g	g	g	c	c	t	t	c	c	c	t	a	g	c	t	t	c	c	g	a	t
KM355705	5	a	c	t	c	c	t	t	a	c	c	t	c	c	a	t	t	c	g	g	c	a	a	g	c	t	g	t	a	g	g	t	t	a	t	g	g	g	a	a	a	g	g	c	a	t	t	c	c	g	t	g	g	g	g	g	g	c	c	t	t	c	c	c	t	a	g	c	t	t	c	c	g	a	t
KM355706	5	a	c	t	c	c	t	t	a	c	c	t	c	c	a	t	t	c	g	g	c	a	a	g	c	t	g	t	a	g	g	t	t	a	t	g	g	g	a	a	a	g	g	c	a	t	t	c	c	g	t	g	g	g	g	g	g	c	c	t	t	c	c	c	t	a	g	c	t	t	c	c	g	a	t
KM355707	5	a	c	t	c	c	t	t	a	c	c	t	c	c	a	t	t	c	g	g	c	a	a	g	c	t	g	t	a	g	g	t	t	a	t	g	g	g	a	a	a	g	g	c	a	t	t	c	c	g	t	g	g	g	g	g	g	c	c	t	t	c	c	c	t	a	g	c	t	t	c	c	g	a	t
KM355708	5	a	c	t	c	c	t	t	a	c	c	t	c	c	a	t	t	c	g	g	c	a	a	g	c	t	g	t	a	g	g	t	t	a	t	g	g	g	a	a	a	g	g	c	a	t	t	c	c	g	t	g	g	g	g	g	g	c	c	t	t	c	c	c	t	a	g	c	t	t	c	c	g	a	t
KM355709	5	a	c	t	c	c	t	t	a	c	c	t	c	c	a	t	t	c	g	g	c	a	a	g	c	t	g	t	a	g	g	t	t	a	t	g	g	g	a	a	a	g	g	c	a	t	t	c	c	g	t	g	g	g	g	g	g	c	c	t	t	c	c	c	t	a	g	c	t	t	c	c	g	a	t
KM355711	5	a	c	t	c	c	t	t	a	c	c	t	c	c	a	t	t	c	g	g	c	a	a	g	c	t	g	t	a	g	g	t	t	a	t	g	g	g	a	a	a	g	g	c	a	t	t	c	c	g	t	g	g	g	g	g	g	c	c	t	t	c	c	c	t	a	g	c	t	t	c	c	g	a	t
KM355712	5	a	c	t	c	c	t	t	a	c	c	t	c	c	a	t	t	c	g	g	c	a	a	g	c	t	g	t	a	g	g	t	t	a	t	g	g	g	a	a	a	g	g	c	a	t	t	c	c	g	t	g	g	g	g	g	g	c	c	t	t	c	c	c	t	a	g	c	t	t	c	c	g	a	t
KY037797	5	[illegible]	[illegible]	[illegible]	[illegible]	[illegible]	[illegible]	[illegible]	[illegible]	[illegible]	[illegible]	[illegible]	[illegible]	[illegible]	[illegible]	[illegible]	[illegible]	[illegible]	[illegible]	[illegible]	[illegible]	[illegible]	[illegible]	[illegible]	[illegible]	[illegible]	[illegible]	[illegible]	[illegible]	[illegible]	[illegible]	[illegible]	[illegible]	[illegible]	[illegible]	[illegible]	[illegible]	[illegible]	[illegible]	[illegible]	[illegible]	[illegible]	[illegible]	[illegible]	[illegible]	[illegible]	[illegible]	[illegible]	[illegible]	[illegible]	[illegible]	[illegible]	[illegible]	[illegible]	[illegible]	[illegible]	[illegible]	[illegible]	[illegible]	[illegible]	[illegible]	[illegible]	[illegible]	[illegible]	[illegible]	[illegible]	[illegible]	[illegible]	[illegible]	[illegible]	[illegible]	[illegible]	[illegible]	[illegible]	[illegible]

Supplementary Table 3: 2880 SN

GB-ID	Clade markers					1						4												3																9																																			
	Position in Dumas	13876	13925	14345	14362	14390	14402	14432	14589	14801	15018	15182	15183	15197	15238	15299	15419	15533	15583	15608	15787	15865	15884	15896	15963	16021	16023	16047	16067	16072	16109	16145	16160	16264	16384	16418	16438	16485	16522	16705	16720	16827	16851	16903	16972	17098	17155	17163	17365	17392	17404	17534	17725	17734	17753	17770	17810	17834	17850	17973	17987	18052	18054	18082	18093	18202	18224	18240	18275	18303	18309	18323	18406	18467	18539
MH499467	5	a	c	t	c	c	t	t	a	c	c	t	c	c	a	t	t	c	g	g	c	a	a	g	c	t	g	t	a	g	g	t	t	a	t	g	g	g	a	a	a	g	g	c	a	t	t	c	c	g	t	g	g	g	g	g	g	c	c	t	t	c	c	c	t	a	g	c	t	t	c	c	g	a	t
MH499469	5	a	c	t	c	c	t	t	a	c	c	t	c	c	a	t	t	c	a	g	c	a	a	g	c	t	g	t	a	g	g	t	t	a	t	g	g	g	a	a	a	g	g	c	a	t	t	c	c	g	t	g	g	g	g	g	g	c	c	t	t	c	c	c	t	a	g	c	t	t	c	t	g	a	t
MH709321	5	a	c	t	c	c	t	t	a	c	c	t	c	c	a	t	t	c	g	g	c	a	a	g	c	t	g	t	a	g	g	t	t	a	t	g	g	g	a	a	a	g	g	c	a	t	t	c	c	g	t	g	g	g	g	g	g	c	c	t	t	c	c	c	t	a	g	c	t	t	c	c	g	a	t
MH709324	5	a	c	t	c	c	t	t	a	c	c	t	c	c	a	t	t	c	a	g	c	a	a	g	c	t	g	t	a	g	g	t	t	a	t	g	g	g	a	a	a	g	g	c	a	t	t	c	c	g	t	g	g	g	g	g	g	c	c	t	t	c	c	c	t	a	g	c	t	t	c	c	g	a	t
MH709325	5	a	c	t	c	c	t	t	a	c	c	t	c	c	a	t	t	c	g	g	t	a	a	g	c	t	g	t	a	g	g	t	t	a	t	g	g	g	a	a	a	g	g	c	a	t	t	c	c	g	t	g	g	g	g	g	g	c	c	t	t	c	c	c	t	a	g	c	t	t	c	c	g	a	t
MH709327	5	a	c	t	c	c	t	t	a	c	c	t	c	c	a	t	t	c	g	g	c	a	a	g	c	t	g	t	a	g	g	t	t	a	t	g	g	g	a	a	a	g	g	c	a	t	t	c	c	g	t	g	g	g	g	g	g	c	c	t	t	c	c	c	t	a	g	c	t	t	c	c	g	a	t
MH709328	5	a	c	t	c	c	t	t	a	c	c	t	c	c	a	t	t	c	a	g	c	a	a	g	c	t	g	t	a	g	g	t	t	a	t	g	g	g	a	a	a	g	g	c	a	t	t	c	c	g	t	g	g	g	g	g	g	c	c	t	t	c	c	c	t	a	g	c	t	t	c	t	g	a	t
MH709330	5	a	c	t	c	c	t	t	a	c	c	t	c	c	a	t	t	c	a	g	c	a	a	g	c	t	g	t	a	g	g	t	t	a	t	g	g	g	a	a	a	g	g	c	a	t	t	c	c	g	t	g	g	g	g	g	g	c	c	t	t	c	c	c	t	a	g	c	t	t	c	t	g	a	t
MH709332	5	a	c	t	c	c	t	t	a	c	c	t	c	c	a	t	t	c	a	g	c	a	a	g	c	t	g	t	a	g	g	t	t	a	t	g	g	g	a	a	a	g	g	c	a	t	t	c	c	g	t	g	g	g	g	g	g	c	c	t	t	c	c	c	t	a	g	c	t	t	c	t	g	a	t
MH709334	5	a	c	t	c	c	t	t	a	c	c	t	c	c	a	t	t	c	g	g	c	a	a	g	c	t	g	t	a	g	g	t	t	a	t	g	g	g	a	a	a	g	g	c	a	t	t	c	c	g	t	g	g	g	g	g	g	c	c	t	t	c	c	c	t	a	g	c	t	t	c	c	g	a	t
MH709335	5	a	c	t	c	c	t	t	a	c	c	t	c	c	a	t	t	c	g	g	c	a	a	g	c	t	g	t	a	g	g	t	t	a	t	g	g	g	a	a	a	g	g	c	a	t	t	c	c	g	t	g	g	g	g	g	g	c	c	t	t	c	c	c	t	a	g	c	t	t	c	c	g	a	t
MH709336	5	a	c	t	c	c	t	t	a	c	c	t	c	c	a	t	t	c	g	g	c	a	a	g	c	t	g	t	a	g	g	t	t	a	t	g	g	g	a	a	a	g	g	c	a	t	t	c	c	g	t	g	g	g	g	g	g	c	c	t	t	c	c	c	t	a	g	c	t	t	c	c	g	a	t
MH709338	5	a	c	t	c	c	t	t	a	c	c	t	c	c	a	t	t	c	g	g	c	a	a	g	c	t	g	t	a	g	g	t	t	a	t	g	g	g	a	a	a	g	g	c	a	t	t	c	c	g	t	g	g	g	g	g	g	c	c	t	t	c	c	c	t	a	g	c	t	t	c	c	g	a	t
MH709340	5	a	c	t	c	c	t	t	a	c	c	t	c	c	a	t	t	c	g	g	c	a	g	g	c	t	g	t	a	g	g	t	t	a	t	g	g	g	a	a	a	g	g	c	a	t	t	c	c	g	t	g	g	g	g	g	g	c	c	t	t	c	c	c	t	a	g	c	t	t	c	c	g	a	t
MH709342	5	a	c	t	c	c	t	t	a	c	c	t	c	c	a	t	t	c	g	g	c	a	a	g	c	t	g	t	a	g	g	t	t	a	t	g	g	g	a	a	a	g	g	c	a	t	t	c	c	g	t	g	g	g	g	g	g	c	c	t	t	c	c	c	t	a	g	c	t	t	c	c	g	a	t
MH709343	5	a	c	t	c	c	t	t	a	c	c	t	c	c	a	t	t	c	g	g	c	a	a	g	c	t	g	t	a	g	g	t	t	a	t	g	g	g	a	a	a	g	g	c	a	t	t	c	c	g	t	g	g	g	g	g	g	c	c	t	t	c	c	c	t	a	g	c	t	t	c	c	g	a	t
MH709345	5	a	c	t	c	c	t	t	a	c	c	t	c	c	a	t	t	c	g	g	c	a	a	g	c	t	g	t	a	g	g	t	t	a	t	g	g	g	a	a	a	g	g	c	a	t	t	c	c	g	t	g	g	g	g	g	g	c	c	t	t	c	c	c	t	a	g	c	t	t	c	c	g	a	t
MH709347	5	a	c	t	c	c	t	t	a	c	c	t	c	c	a	t	t	c	g	g	c	a	a	g	c	t	g	t	a	g	g	t	t	a	t	g	g	g	a	a	a	g	g	c	a	t	t	c	c	g	t	g	g	g	g	g	g	c	c	t	t	c	c	c	t	a	g	c	t	t	c	c	g	a	t
MH709349	5	a	c	t	c	c	t	t	a	c	c	t	c	c	a	t	t	c	g	g	c	a	a	g	c	t	g	t	a	g	g	t	t	a	t	g	g	g	a	a	a	g	g	c	a	t	t	c	c	g	t	g	g	g	g	g	g	c	c	t	t	c	c	c	t	a	g	c	t	t	c	c	g	a	t
MH709350	5	a	c	t	c	c	t	t	a	c	c	t	c	c	a	t	t	c	g	g	c	a	a	g	c	t	g	t	a	g	g	t	t	a	t	g	g	g	a	a	a	t	g	c	a	t	t	c	c	g	t	g	g	g	g	g	g	c	c	t	t	c	c	c	t	a	g	c	t	t	c	c	g	a	t
MH709351	5	a	c	t	c	c	t	t	a	c	c	t	c	c	a	t	t	c	g	g	c	a	a	g	c	t	g	t	a	g	g	t	t	a	t	g	g	g	a	a	a	g	g	c	a	t	t	t	c	g	t	g	g	g	g	g	g	c	c	t	t	c	c	c	t	a	g	c	t	t	c	c	g	a	t
MH709353	5	a	c	t	c	c	t	t	a	c	c	t	c	c	a	t	t	c	g	g	c	a	a	g	c	t	g	t	a	g	g	t	t	a	t	g	g	g	a	a	a	g	g	c	a	t	t	c	c	g	t	g	g	g	g	g	g	c	c	t	t	c	c	c	t	a	g	c	t	t	c	c	g	a	t
MH709358	5	a	c	t	c	c	t	t	a	c	c	t	c	c	a	t	t	c	g	g	c	a	a	g	c	t	g	t	a	g	g	t	t	a	t	g	g	g	a	a	a	g	g	c	a	t	t	c	c	g	t	g	g	g	g	g	g	c	c	t	t	c	c	c	t	a	g	c	t	t	c	c	g	a	t
MH709360	5	a	c	t	c	c	t	t	a	c	c	t	c	c	a	t	t	c	a	g	c	a	a	g	c	t	g	t	a	g	g	t	t	a	t	g	g	g	a	a	a	g	g	c	a	t	t	c	c	g	t	g	g	g	g	g	g	c	c	t	t	c	c	c	t	a	g	c	t	t	c	t	g	a	t
MH709362	5	a	c	t	c	c	t	t	a	c	c	t	c	c	a	t	t	c	g	g	c	a	a	g	c	t	g	t	a	g	g	t	t	a	t	g	g	g	a	a	a	g	g	c	a	t	t	c	c	g	t	g	g	g	g	g	g	c	c	t	t	c	c	c	t	a	g	c	t	t	c	c	g	a	t
MH709365	5	a	c	t	c	c	t	t	a	c	c	t	c	c	a	t	t	c	g	g	c	a	a	g	c	t	g	t	a	g	g	t	t	a	t	g	g	g	a	a	a	t	g	c	a	t	t	c	c	g	t	g	g	g	g	g	g	c	c	t	t	c	c	c	t	a	g	c	t	t	c	c	g	a	t
MH709373	5	a	c	t	c	c	t	t	a	c	c	t	c	c	a	t	t	c	g	g	c	a	a	g	c	t	g	t	a	g	g	t	t	a	t	g	g	g	a	a	a	g	g	c	a	t	t	c	c	g	t	g	g	g	g	g	g	c	c	t	t	c	c	c	t	a	g	c	t	t	c	c	g	a	t
MH709374	5	a	c	t	c	c	t	t	a	c	c	t	c	c	a	t	t	c	g	g	c	a	a	g	c	t	g	t	a	g	g	t	t	a	t	g	g	g	a	a	a	g	g	c	a	t	t	c	c	g	t	g	g	g	g	g	g	c	c	t	t	c	c	c	t	a	g	c	t	t	c	c	g	a	t
KC112914	6	a	c	t	c	c	t	t	a	c	c	t	c	c	a	t	t	c	g	g	c	a	a	g	c	t	g	t	a	g	g	t	t	a	t	g	g	g	a	a	a	g	g	c	a	t	t	c	t	g	t	g	a	g	g	g	g	t	c	t	t	c	t	c	t	a	g	c	t	t	c	c	g	a	t
KP771914	6	a	c	t	c	c	t	t	a	c	c	t	c	c	a	t	t	c	g	g	c	a	a	g	t	t	g	t	a	g	g	t	t	a	t	g	g	g	a	a	a	g	g	c	a	c	t	c	t	g	t	g	a	g	g	g	g	t	c	t	t	c	t	c	t	a	g	c	t	t	c	c	g	a	t
KP702725	6	a	c	t	c	c	t	t	a	c	c	t	c	c	a	t	t	c	g	g	c	a	a	g	c	t	g	t	a	g	g	t	t	a	t	g	g	g	a	a	a	g	g	c	a	t	t	c	t	g	t	g	a	g	g	g	g	t	c	t	t	c	t	c	t	a	g	c	t	t	c	c	g	a	t
KP771890	9	a	c	t	c	c	t	t	a	c	c	t	c	c	a	t	t	c	g	g	c	a	a	g	c	t	g	t	a	g	g	t	t	a	t	g	g	g	a	g	a	g	g	c	a	t	t	c	c	g	t	g	g	g	g	g	g	c	c	t	t	c	c	c	t	a	g	c	t	t	c	c	g	a	t
JN704710	9	a	c	t	c	c	t	t	a	c	c	t	c	c	a	t	t	c	g	g	c	a	a	g	c	t	g	t	a	t	g	t	t	a	t	g	g	g	a	g	a	g	g	c	a	t	t	c	c	a	t	g	a	g	g	g	g	c	c	t	t	c	t	c	t	a	g	c	t	t	c	c	g	a	t
KY037798	9	a	c	t	c	c	t	t	a	c	c	t	c	c	a	t	t	c	g	g	c	a	a	g	c	t	g	t	a	g	g	t	t	a	t	g	g	g	a	g	a	g	g	c	a	t	t	c	c	g	t	g	g	g	g	g	g	c	c	t	t	c	c	c	t	a	g	c	t	t	c	c	g	a	t
MH709312	9	a	c	t	c	c	t	t	a	c	c	t	c	c	a	t	t	c	g	g	c	a	a	g	c	t	g	t	a	g	g	t	t	a	t	g	g	g	a	g	a	g	g	c	a	t	t	c	c	g	t	g	g	g	g	g	g	c	c	t	t	c	c	c	t	a	g	c	t	t	c	c	g	a	t
MH709352	9	a	c	t	c	c	t	t	a	c	c	t	c	c	a	t	t	c	g	g	c	a	a	g	c	t	g	t	a	g	g	t	t	a	t	g	g	g	a	g	a	g	g	c	a	t	t	c	c	g	t	g	g	g	g	g	g	c	c	t	t	c	c	c	t	a	g	c	t	t	c	c	g	a	t
AB097933	Vac	a	c	t	c	c	t	t	a	c	c	t	c	c	a	t	t	c	g	g	c	a	a	g	c	t	g	t	a	g	g	t	t	a	t	g	g	g	a	a	a	g	g	c	a	t	t	c	t	g	t	g	a	g	g	g	g	t	c	t	t	c	t	t	t	a	g	c	t	t	c	c	g	a	t
JN704698	Vac	a	c	t	c	c	t	t	a	c	c	t	c	c	a	t	t	c	g	g	c	a	a	g	c	t	g	t	a	g	g	t	t	a	t	g	g	g	a	a	a	g	g	c	a	t	t	c	t	g	t	g	a	g	g	g	g	t	c	t	t	c	t	t	t	a	g	c	t	t	c	c	g	a	t
JN704697	Vac	a	c	t	c	c	t	t	a	c	c	t	c	c	a	t	t	c	g	g	c	a	a	g	c	t	g	t	a	g	g	t	t	a	t	g	g	g	a	a	a	g	g	c	a	t	t	c	t	g	t	g	a	g	g	g	g	t	c	t	t	c	t	t	t	a	g	c	t	t	c	c	g	a	t
DQ008355	Vac	a	c	t	c	c	t	t	a	c	c	t	c	c	a	t	t	c	g	g	c	a	a	g	c	t	g	t	a	g	g	t	t	a	t	g	g	g	a	a	a	g	g	c	a	t	t	c	t	g	t	g	a	g	g	g	g	t	c	t	t	c	t	t	t	a	g	c	t	t	c	c	g	a	t
JF306641	Vac	a	c	t	c	c	t	t	a	c	c	t	c	c	a	t	t	c	g	g	c	a	a	g	c	t	g	t	a	g	g	t	t	a	t	g	g	g	a	a	a	g	g	c	a	t	t	c	t	g	t	g	a	g	g	g	g	t	c	t	t	c	t	t	t	a	g	c	t	t	c	c	g	a	t
AB097932	Vac	a	c	t	c	c	t	t	a	c	c	t	c	c	a	t	t	c	g	g	c	a	a	g	c	t	g	t	a	g	g	t	t	a	t	g	g	g	a	a	a	g	g	c	a	t	t	c	t	g	t	g	a	g	g	g	g	t	c	t	t	c	t	t	t	a	g	c	t	t	c	c	g	a	t
DQ008354	Vac	a	c	t	c	c	t	t	a	c	c	t	c	c	a	t	t	c	g	g	c	a	a	g	c	t	g	t	a	g	g	t	t	a	t	g	g	g	a	a	a	g	g	c	a	t	t	c	t	g	t	g	a	g	g	g	g	t	c	t	t	c	t	t	t	a	g	c	t	t	c	c	g	a	t
KU926312	Vac	a	c	t	c	c	t	t	a	c	c	t	c	c	a	t	t	c	g	g	c	a	a	g	c	t	g	t	a	g	g	t	t	a	t	g	g	g	a	a	a	g	g	c	a	t	t	c	t	g	t	g	a	g	g	g	g	t	c	t	t	c	t	t	t	a	g	c	t	t	c	c	g	a	t
KU926313	Vac	a	c	t	c	c	t	t	a	c	c	t	c	c	a	t	t	c	g	g	c	a	a	g	c	t	g	t	a	g	g	t	t	a	t	g	g	g	a	a	a	g	g	c	a	t	t	c	t	g	t	g	a	g	g	g	g	t	c	t	t	c	t	t	t	a	g	c	t	t	c	c	g	a	t
KU926314	Vac	a	c	t	c	c	t	t	a	c	c	t	c	c	a	t	t	c	g	g	c	a	a	g	c	t	g	t	a	g	g	t	t	a	t	g	g	g	a	a	a	g	g	c	a	t	t	c	t	g	t	g	a	g	g	g	g	t	c	t	t	c	t	t	t	a	g	c	t	t	c	c	g	a	t
KF558371	Vac	a	c	t	c	c	t	t	a	c	c	t	c	c	a	t	t	c	g	g	c	a	a	g	c	t	g	t	a	g	g	t	t	a	t	g	g	g	a	a	a	g	g	c	a	t	t	c	t	g	t	g	a	g	g	g	g	t	c	t	t	c	t	t	t	a	g	c	t	t	c	c	g	a	t
KF558372	Vac	a	c	t	c	c	t	t	a	c	c	t	c	c	a	t	t	c	g	g	c	a	a	g	c	t	g	t	a	g	g	t	t	a	t	g	g	g	a	a	a	g	g	c	a	t	t	c	t	g	t	g	a	g	g	g	g	t	c	t	t	c	t	t	t	a	g	c	t	t	c	c	g	a	t
KF558373	Vac	a	c	t	c	c	t	t	a	c	c	t	c	c	a	t	t	c	g	g	c	a	a	g	c	t	g	t	a	g	g	t	t	a	t	g	g	g	a	a	a	g	g	c	a	t	t	c	t	g	t	g	a	g	g	g	g	t	c	t	t	c	t	t	t	a	g	c	t	t	c	c	g	a	t
KF558374	Vac	a	c	t	c	c	t	t	a	c	c	t	c	c	a	t	t	c	g	g	c	g	a	g	c	t	g	t	a	g	g	t	t	a	t	g	g	g	a	a	a	g	g	c	a	t	t	c	t	g	t	g	a	g	g	g	g	t	c	t	t	c	t	t	t	a	g	c	t	t	c	c	g	a	t
KF558375	Vac	a	c	t	c	c	t	t	a	c	c	t	c	c	a	t	t	c	g	g	c	a	a	g	c	t	g	t	a	g	g	t	t	a	t	g	g	g	a	a	a	g	g	c	a	t	t	c	t	g	t	g	a	g	g	g	g	t	c	t	t	c	t	t	t	a	g	c	t	t	c	c	g	a	t
KF558376	Vac	a	c	t	c	c	t	t	a	c	c	t	c	c	a	t	t	c	g	g	c	a	a	g	c	t	g	t	a	g	g	t	t	a	t	g	g	g	a	a	a	g	g	c	a	t	t	c	t	g	t	g	a	g	g	g	g	t	c	t	t	c	t	t	t	a	g	c	t	t	c	c	g	a	t
KF558377	Vac	a	c	t	c	c	t	t	a	c	c	t	c	c	a	t	t	c	g	g	c	a	a	g	c	t	g	t	a	g	g	t	t	a	t	g	g	g	a	a	a	g	g	c	a	t	t	c	t	g	t	g	a	g	g	g	g	t	c	t	t	c	t	t	t	a	g	c	t	t	c	c	g	a	t
KF558378	Vac	a	c	t	c	c	t	t	a	c	c	t	c	c	a	t	t	c	g	g	c	a	a	g	c	t	g	t	a	g	g	t	t	a	t	g	g	g	a	a	a	g	g	c	a	t	t	c	t	g	t	g	a	g	g	g	g	t	c	t	t	c	t	t	t	a	g	c	t	t	c	c	a	a	t
KF558379	Vac	a	c	t	c	c	t	t	a	c	c	t	c	c	a	t	t	c	g	g	c	a	a	g	c	t	g	t	a	g	g	t	t	a	t	g	g	g	a	a	a	g	g	c	a	t	t	c	t	g	t	g	a	g	g	g	g	t	c	t	t	c	t	t	t	a	g	c	t	t	c	c	g	a	t
KF558380	Vac	a	c	t	c	c	t	t	a	c	c	t	c	c	a	t	t	c	g	g	c	a	a	g	c	t	g	t	a	g	g	t	t	a	t	g	g	g	a	a	a	g	g	c	a	t	t	c	t	g	t	g	a	g	g	g	g	t	c	t	t	c	t	t	t	a	g	c	t	t	c	c	g	a	t
KF558381	Vac	a	c	t	c	c	t	t	a	c	c	t	c	c	a	t	t	c	g	g	c	a	a	g	c	t	g	t	a	g	g	t	t	a	t	g	g	g	a	a	a	g	g	c	a	t	t	c	t	g	t	g	a	g	g	g	g	t	c	t	t	c	t	t	t	a	g	c	t	t	c	c	g	a	t
KF558382	Vac	a	c	t	c	c	t	t	a	c	c	t	c	c	a	t	t	c	g	g	c	a	a	g	c	t	g	t	a	g	g	t	t	a	t	g	g	g	a	a	a	g	g	c	a	t	t	c	t	g	t	g	a	g	g	g	g	t	c	t	t	c	t	t	t	a	g	c	t	t	c	c	g	a	t
KF558383	Vac	a	c	t	c	c	t	t	a	c	c	t	c	c	a	t	t	c	g	g	c	a	a	g	c	t	g	t	a	g	g	t	t	a	t	g	g	g	a	a	a	g	g	c	a	t	t	c	t	g	t	g	a	g	g	g	g	t	c	t	t	c	t	t	t	a	g	c	t	t	c	c	g	a	t
KF558384	Vac	a	c	t	c	c	t	t	a	c	c	t	c	c	a	t	t	c	g	g	c	a	a	g	c	t	g	t	a	g	g	t	t	a	t	g	g	g	a	a	a	g	g	c	a	t	t	c	t	g	t	g	a	g	g	g	g	t	c	t	t	c	t	t	t	a	g	c	t	t	c	c	g	a	t
KF558385	Vac	a	c	t	c	c	t	t	a	c	c	t	c	c	a	t	t	c	g	g	c	a	a	g	c	t	g	t	a	g	g	t	t	a	t	g	g	g	a	a	a	g	g	c	a	t	t	c	t	g	t	g	a	g	g	g	g	t	c	t	t	c	t	t	t	a	g	c	t	t	c	c	g	a	t
KF558386	Vac	a	c	t	c	c	t	t	a	c	c	t	c	c	a	t	t	c	g	g	c	a	a	g	c	t	g	t	a	g	g	t	t	a	t	g	g	g	a	a	a	g	g	c	a	t	t	c	t	g	t	g	a	g	g	g	g	t	c	t	t	c	t	t	t	a	g	c	t	t	c	c	g	a	t
KF558387	Vac	a	c	t	c	c	t	t	a	c	c	t	c	c	a	t	t	c	g	g	c	a	a	g	c	t	g	t	a	g	g	t	t	a	t	a	g	g	a	a	a	g	g	c	a	t	t	c	t	g	t	g	a	g	g	g	g	t	c	t	t	c	t	t	t	a	g	c	t	t	c	c	g	a	t
KF558388	Vac	a	c	t	c	c	t	t	a	c	c	t	c	c	a	t	t	c	g	g	c	a	a	g	c	t	g	t	a	g	g	t	t	a	t	g	g	g	a	a	a	g	g	c	a	t	t	c	t	g	t	g	a	g	g	g	g	t	c	t	t	c	t	t	t	a	g	c	t	t	c	c	g	a	t
KF558389	Vac	a	c	t	c	c	t	t	a	c	c	t	c	c	a	t	t	c	g	g	c	a	a	g	c	t	g	t	a	g	g	t	t	a	t	g	g	g	a	a	a	g	g	c	a	t	t	c	t	g	t	g	a	g	g	g	g	t	c	t	t	c	t	t	t	a	g	c	t	t	c	c	g	a	t
KF558390	Vac	a	c	t	c	c	t	t	a	c	c	t	c	c	a	t	t	c	g	g	c	a	a	g	c	t	g	t	a	g	g	t	t	a	t	g	g	g	a	a	a	g	g	c	a	t	t	c	t	g	t	g	a	g	g	g	g	t	c	t	t	c	t	t	t	a	g	c	t	t	c	c	g	a	t
KF558391	Vac	a	c	t	c	c	t	t	a	c	c	t	c	c	a	t	t	c	g	g	c	a	a	g	c	t	g	t	a	g	g	t	t	a	t	g	g	g	a	a	a	g	g	c	a	t	t	c	t	g	t	g	a	g	g	g	g	t	c	t	t	c	t	t	t	a	g	c	t	t	c	c	g	a	t
KF811485	Vac	a	c	t	c	c	t	t	a	c	c	t	c	c	a	t	t	c	g	g	c	g	a	g	c	t	g	t	a	g	g	t	t	a	t	g	g	g	a	a	a	g	g	c	a	t	t	c	t	g	t	g	a	g	g	g	g	t	c	t	t	c	t	t	t	a	g	c	t	t	c	c	g	a	t
KF853225	Vac	a	c	t	c	c	t	t	a	c	c	t	c	c	a	t	t	c	g	g	c	a	a	g	c	t	g	t	a	g	g	t	t	a	t	g	g	g	a	a	a	g	g	c	a	t	t	c	t	g	t	g	a	g	g	g	g	t	c	t	t	c	t	t	t	a	g	c	t	t	c	c	g	a	t
KF853226	Vac	a	c	t	c	c	t	t	a	c	c	t	c	c	a	t	t	c	g	g	c	a	a	g	c	t	g	t	a	g	g	t	t	a	t	g	g	g	a	a	a	g	g	c	a	t	t	c	t	g	t	g	a	g	g	g	a	t	c	t	t	c	t	t	t	a	g	c	t	t	c	c	g	a	t
KF853227	Vac	a	c	t	c	c	t	t	a	c	c	t	c	c	a	t	t	c	g	g	c	a	a	g	c	t	g	t	a	g	g	t	t	a	t	g	g	g	a	a	a	g	g	c	a	t	t	c	t	g	t	g	a	g	g	g	g	t	c	t	t	c	t	t	t	a	g	c	t	t	c	c	g	a	t
KF853228	Vac	a	c	t	c	c	t	t	a	c	c	t	c	c	a	t	t	c	g	g	c	a	a	g	c	t	g	t	a	g	g	t	t	a	t	g	g	g	a	a	a	g	g	c	a	t	t	c	t	g	t	g	a	g	g	g	g	t	c	t	t	c	t	t	t	a	g	c	t	t	c	c	g	a	t
KF853229	Vac	a	c	t	c	c	t	t	a	c	c	t	c	c	a	t	t	c	g	g	c	a	a	g	c	t	g	t	a	g	g	t	t	a	t	g	g	g	a	a	a	g	g	c	a	t	t	c	t	g	t	g	a	g	g	g	g	t	c	t	t	c	t	t	t	a	g	c	t	t	c	c	g	a	t
KF853230	Vac	a	c	t	c	c	t	t	a	c	c	t	c	c	a	t	t	c	g	g	c	a	a	g	c	t	g	t	a	g	g	t	t	a	t	g	g	g	a	a	a	g	g	c	a	t	t	c	t	g	t	g	a	g	g	g	g	t	c	t	t	c	t	t	t	a	g	c	t	t	c	c	g	a	t
KF853231	Vac	a	c	t	c	c	t	t	a	c	c	t	c	c	a	t	t	c	g	g	c	a	a	g	c	t	g	t	a	g	g	t	t	a	t	g	g	g	a	a	a	g	g	c	a	t	t	c	t	g	t	g	a	g	g	g	g	t	c	t	t	c	t	t	t	a	g	c	t	t	c	c	g	a	t
KF853232	Vac	a	c	t	c	c	t	t	a	c	c	t	c	c	a	t	t	c	g	g	c	a	a	g	c	t	g	t	a	g	g	t	t	a	t	g	g	g	a	a	a	g	g	c	a	t	t	c	t	g	t	g	a	g	g	g	g	t	c	t	t	c	t	t	t	a	g	c	t	t	c	c	g	a	t
KF853233	Vac	a	c	t	c	c	t	t	a	c	c	t	c	c	a	t	t	c	g	g	c	a	a	g	c	t	g	t	a	g	g	t	t	a	t	g	g	g	a	a	a	g	g	c	a	t	t	c	t	g	t	g	a	g	g	g	g	t	c	t	t	c	t	t	t	a	g	c	t	t	c	c	g	a	t
KF853234	Vac	a	c	t	c	c	t	t	a	c	c	t	c	c	a	t	t	c	g	g	c	a	a	g	c	t	g	t	g	g	g	t	t	a	t	g	g	g	a	a	a	g	g	c	a	t	t	c	t	g	t	g	a	g	g	g	g	t	c	t	t	c	t	t	t	a	g	c	t	t	c	c	g	a	t
KF853235	Vac	a	c	t	c	c	t	t	a	c	c	t	c	c	a	t	t	c	g	g	c	a	a	g	c	t	g	t	a	g	g	t	t	a	t	g	g	g	a	a	a	g	g	c	a	t	t	c	t	g	t	g	a	g	g	g	g	t	c	t	t	c	t	t	t	a	g	c	t	t	c	c	g	a	t
MF898328	Vac	a	c	t	c	c	t	t	a	c	c	t	c	c	a	t	t	c	g	g	c	a	a	g	c	t	g	t	a	g	g	t	t	a	t	g	g	g	a	a	a	g	g	c	a	t	t	c	t	g	t	g	a	g	g	g	g	t	c	t	t	c	t	t	t	a	g	c	t	t	c	c	g	a	t
JN704709	VIII	a	c	t	c	c	t	t	a	c	c	t	c	c	a	t	t	c	g	g	c	a	a	g	c	t	g	t	a	g	g	t	t	a	t	g	g	g	a	a	a	g	g	c	a	t	t	c	t	g	t	g	a	g	g	g	g	t	c	t	t	c	t	c	t	a	g	c	t	t	c	c	g	a	t

Supplementary Table 3: 2880 SN

GB-ID	Clade markers																																																																									
	Position in Dumas	18547	18603	18626	18872	18878	18905	18944	18969	19036	19064	19097	19196	19323	19373	19374	19431	19461	19476	19485	19503	19619	19667	19698	19707	19719	19746	19770	19838	19845	19924	19981	19996	20019	20076	20089	20096	20158	20164	20181	20184	20208	20388	20399	20406	20508	20532	20547	20631	20642	20656	20665	20666	20684	20687	21021	21057	21059	21127	21130	21156	21294	21297	21300	21350	21371	21509	21557	21595	21734	21777	21854	21882	21929	22005
AY548170	1	g	t	a	a	c	a	g	t	a	c	a	t	c	t	a	t	g	a	c	a	t	c	a	g	a	g	a	c	a	t	g	g	g	t	t	c	g	g	t	g	g	c	a	g	c	t	c	g	a	t	g	g	t	t	t	a	t	g	a	a	t	t	-	a	g	t	t	g	g	g	t	g	a	a
AY548171	1	g	t	a	a	c	a	g	t	a	c	a	t	c	t	a	t	t	a	c	a	t	c	a	g	a	g	a	c	a	t	g	g	g	t	t	c	g	g	t	g	g	c	a	g	c	t	c	g	a	t	g	g	t	t	t	a	t	g	a	a	t	t	-	a	g	t	t	g	g	g	t	g	a	a
DQ479953	1	g	t	a	a	c	a	g	t	a	c	a	t	c	t	a	t	g	a	c	a	t	c	a	g	a	g	a	c	a	t	g	g	g	t	t	c	g	g	t	g	g	c	a	g	c	t	c	g	a	t	g	g	t	t	t	a	t	g	a	a	t	t	-	a	g	t	c	g	g	g	t	g	a	a
DQ479954	1	g	t	a	a	c	a	g	t	a	c	a	t	c	t	a	t	g	a	c	a	t	c	a	t	a	g	a	c	a	t	g	g	g	t	t	c	g	g	t	g	g	c	a	g	c	t	c	g	a	t	g	g	t	t	t	a	t	g	a	a	t	t	-	a	g	t	t	g	g	g	t	g	a	a
DQ479958	1	g	t	a	a	c	a	g	t	a	c	a	t	c	t	a	t	g	a	c	a	t	c	a	g	a	g	a	c	a	t	g	g	g	t	t	c	g	g	t	g	g	c	a	g	c	t	c	g	a	t	g	g	t	t	t	a	t	g	a	a	t	t	-	a	g	t	t	g	g	g	t	g	a	a
DQ479959	1	g	t	a	a	c	a	g	t	a	c	a	t	c	t	a	t	g	a	c	a	t	c	a	g	a	g	a	c	a	t	g	g	g	t	t	c	g	g	t	g	g	c	a	g	c	t	c	g	a	t	g	g	t	t	t	a	t	g	a	a	t	t	-	a	g	t	t	g	g	g	t	g	a	a
DQ479961	1	g	t	a	a	c	a	g	t	a	c	a	t	c	t	a	t	g	a	c	a	t	c	a	g	a	g	a	c	a	t	g	g	g	t	t	c	g	g	t	g	g	c	a	g	c	t	c	g	a	t	g	g	t	t	t	a	t	g	a	a	t	t	-	a	g	t	t	g	g	g	t	g	a	a
DQ479962	1	g	t	a	a	c	a	g	t	a	c	a	t	c	t	a	t	g	a	c	a	t	c	a	g	a	g	a	c	a	t	g	g	g	t	t	c	g	g	t	g	g	c	a	g	c	t	c	g	a	t	g	g	t	t	t	a	t	g	a	a	t	t	-	a	g	t	t	g	g	g	t	g	a	a
DQ479963	1	g	t	a	a	c	a	g	t	a	c	a	t	c	t	a	t	g	a	c	a	t	c	a	g	a	g	a	c	a	t	g	g	g	t	t	c	g	g	t	g	g	c	a	g	c	t	c	g	a	t	g	g	t	t	t	a	t	g	a	a	t	t	-	a	g	t	t	g	g	g	t	g	a	a
DQ674250	1	g	t	a	a	c	a	g	t	a	c	a	t	c	t	a	t	g	a	c	a	t	c	a	g	a	g	a	c	a	t	g	g	g	t	t	c	g	g	t	g	g	c	a	g	c	t	c	g	a	t	g	g	t	t	t	a	t	g	a	a	t	t	-	a	g	t	t	g	g	g	t	g	a	a
KX262864	1	g	t	a	a	c	a	g	t	a	c	a	t	c	t	a	t	g	a	c	a	t	c	a	g	a	g	a	c	a	t	g	g	g	t	t	c	g	g	t	g	g	c	a	g	c	t	c	g	a	t	g	g	t	t	t	a	t	g	a	a	t	t	-	a	g	t	t	g	g	g	t	g	a	a
KX262865	1	g	t	a	a	c	a	g	t	a	c	a	t	c	t	a	t	g	a	c	a	t	c	a	g	a	g	a	c	a	t	g	g	g	t	t	c	g	g	t	g	g	c	a	g	c	t	c	g	a	t	g	g	t	t	t	a	t	g	a	a	t	t	-	a	g	t	t	g	g	g	t	g	a	a
KX262866	1	g	t	a	a	c	a	g	t	a	c	a	t	c	t	a	t	g	a	c	a	t	c	a	g	a	g	a	c	a	t	g	g	g	t	t	c	g	g	t	g	g	c	a	g	c	t	c	g	a	t	g	g	t	t	t	a	t	g	a	a	t	t	-	a	g	t	t	g	g	g	t	g	a	a
EU154348	1	g	t	a	a	c	a	g	t	a	c	a	t	c	t	a	t	g	a	c	a	t	c	a	g	a	g	a	c	a	t	g	g	g	t	t	c	g	g	t	g	g	c	a	g	c	t	c	g	a	t	g	g	t	t	t	a	t	a	a	a	t	t	-	a	g	t	t	g	g	g	t	g	a	a
JN704690	1	g	t	a	a	c	a	g	t	a	c	a	t	c	t	a	t	g	a	c	a	t	c	a	g	a	g	a	c	a	t	g	g	g	t	t	c	g	g	t	g	g	c	a	g	c	t	c	g	a	t	g	g	t	t	t	a	t	g	a	a	t	t	-	a	g	t	t	g	g	g	t	g	a	a
JN704691	1	g	t	a	a	c	a	g	t	a	c	a	t	c	t	a	t	g	a	c	a	t	c	a	g	a	a	a	c	a	t	g	g	g	t	t	c	g	g	t	g	g	c	a	g	c	t	c	g	a	t	g	g	t	t	t	a	t	g	a	a	t	t	-	a	g	t	t	g	g	g	t	g	a	a
JN704692	1	g	t	a	a	c	a	g	t	a	c	a	t	c	t	a	t	g	a	c	a	t	c	a	g	a	g	a	c	a	t	g	g	g	t	t	c	g	g	t	g	g	c	a	g	c	t	c	g	a	t	g	g	t	t	t	a	t	a	a	a	t	t	-	a	g	t	t	g	g	g	t	g	a	a
JN704693	1	g	t	a	a	c	a	g	t	a	c	a	t	c	t	a	t	g	a	c	a	t	c	a	g	a	g	a	c	a	t	g	g	g	t	t	c	g	g	t	g	g	c	a	g	c	t	c	g	a	t	g	g	t	t	t	a	t	g	a	a	t	t	-	a	g	t	t	g	g	g	t	g	a	a
JN704694	1	g	t	a	a	c	a	g	t	a	c	a	t	c	t	a	t	g	a	c	a	t	c	a	g	a	g	a	c	a	t	g	g	g	t	t	c	g	g	t	g	g	c	a	g	c	t	c	g	a	t	g	g	t	t	t	a	t	g	a	a	t	t	-	a	g	t	t	g	g	g	t	g	a	a
JN704695	1	g	t	a	a	c	a	g	t	a	c	a	t	c	t	a	t	g	a	c	a	t	c	a	g	a	g	a	c	a	t	g	g	g	t	t	c	g	g	t	g	g	c	a	g	c	t	c	g	a	t	g	g	t	t	t	a	t	a	a	a	t	t	-	a	g	t	t	g	g	g	t	g	a	a
JN704696	1	g	t	a	a	c	a	g	t	a	c	a	t	c	t	a	t	g	a	c	a	t	c	a	g	a	a	a	c	a	t	g	g	g	t	t	c	g	g	t	g	g	c	a	g	c	t	c	g	a	t	g	g	t	t	t	a	t	g	a	a	t	t	-	a	g	t	t	g	g	g	t	g	a	a
NC001348	1	g	t	a	a	c	a	g	t	a	c	a	t	c	t	a	t	g	a	c	a	t	c	a	g	a	g	a	c	a	t	g	g	g	t	t	c	g	g	t	g	g	c	a	g	c	t	c	g	a	t	g	g	t	t	t	a	t	g	a	a	t	t	-	a	g	t	t	g	g	g	t	g	a	a
KP771892	1	g	t	a	a	c	a	g	t	a	c	a	t	c	t	a	t	g	a	c	a	t	c	a	g	a	a	a	c	a	t	g	g	g	t	t	c	g	g	t	g	g	c	a	g	c	t	c	g	a	t	g	g	t	t	t	a	t	g	a	a	t	t	-	a	g	t	t	g	g	g	t	a	a	a
KP771894	1	g	t	a	a	c	a	g	t	a	c	a	t	c	t	a	t	g	a	c	a	t	c	a	g	a	g	a	c	a	t	g	g	g	t	t	c	g	g	t	g	g	c	a	g	c	t	c	g	a	t	g	g	t	t	t	a	t	g	a	a	t	t	-	a	g	t	t	g	g	g	t	g	a	a
KP771900	1	g	t	a	a	c	a	g	t	a	c	a	t	t	t	a	t	g	a	c	a	t	c	a	g	a	g	a	c	a	t	g	g	g	t	t	c	g	g	t	g	g	c	a	g	c	t	c	g	a	t	g	g	t	t	t	a	t	g	a	c	t	t	-	a	g	t	t	g	g	g	t	g	a	a
KP771901	1	g	t	a	a	c	a	g	t	a	c	a	t	c	t	a	t	g	a	c	a	t	c	a	g	a	g	a	c	a	t	g	g	g	t	t	c	g	g	t	g	g	c	a	g	c	t	c	g	a	t	g	g	t	t	t	a	t	g	a	c	t	t	-	a	g	t	t	g	g	g	t	g	a	a
KP771902	1	g	t	a	a	c	a	g	t	a	c	a	t	c	t	a	t	g	a	c	a	t	c	a	g	a	g	a	c	a	t	g	g	g	t	t	c	g	g	t	g	g	c	a	g	c	t	c	g	a	t	g	g	t	t	t	a	t	g	a	a	t	t	-	a	g	t	t	g	g	g	t	g	a	a
KP771908	1	g	t	a	a	c	a	g	t	a	c	a	t	c	t	a	t	g	a	c	a	t	c	a	g	a	g	a	c	a	t	g	g	g	t	t	c	g	g	t	g	g	c	a	g	c	t	c	g	a	t	g	g	t	t	t	a	t	g	a	a	t	t	-	a	g	g	t	g	g	g	t	g	a	a
KP771912	1	g	t	a	a	c	a	g	t	a	c	a	t	c	t	a	t	g	a	c	a	t	c	a	g	a	g	a	c	a	t	g	g	g	t	t	c	g	g	t	g	g	c	a	g	c	t	c	g	a	t	g	g	t	t	t	a	t	g	a	a	t	t	-	a	g	t	t	g	g	g	t	g	a	a
KP771913	1	g	t	a	a	c	a	g	t	a	c	a	t	c	t	a	t	g	a	c	a	t	c	a	g	a	g	a	c	a	t	g	g	g	t	t	c	g	g	t	g	g	c	a	g	c	t	c	g	a	t	g	g	t	t	t	a	t	g	a	a	t	t	-	a	g	t	t	g	g	g	t	g	a	a
KP771917	1	g	t	a	a	c	a	g	t	a	c	a	t	c	t	a	t	g	a	c	a	t	c	a	g	a	g	a	c	a	t	g	g	g	t	t	c	g	a	t	g	g	c	a	g	c	t	c	g	a	t	g	g	t	t	t	a	t	g	a	a	t	t	-	a	g	t	t	g	g	g	t	g	a	a
KP771918	1	g	t	a	a	c	a	g	t	a	c	a	t	c	t	a	t	g	a	c	a	t	c	a	g	a	g	a	c	a	t	g	g	g	t	t	c	g	g	t	g	g	c	a	g	c	t	c	g	a	t	g	g	t	t	t	a	t	g	a	a	t	t	-	a	g	t	t	g	g	g	t	g	a	a
KP771920	1	g	t	a	a	c	a	g	t	a	c	a	t	c	t	a	t	g	a	c	a	t	c	a	g	a	g	a	c	a	t	g	g	g	t	t	c	g	g	t	g	g	c	a	g	c	t	c	g	a	t	g	g	t	t	t	a	t	g	a	a	t	t	-	a	g	t	t	g	g	g	t	g	a	a
MH709361	1	g	t	a	a	c	a	g	t	a	c	a	t	c	t	a	t	g	a	c	a	t	c	a	g	a	g	a	c	a	t	g	g	g	t	t	c	g	g	t	g	g	c	a	g	c	t	c	g	a	t	g	g	t	t	t	a	t	g	a	a	t	t	-	a	g	t	t	g	g	g	t	g	a	a
KU926318	2	g	t	a	a	c	g	g	t	a	c	a	t	c	t	a	t	g	a	c	a	t	c	a	g	g	g	a	c	a	t	g	g	g	t	t	c	g	g	t	g	g	c	a	g	c	t	c	g	g	a	g	g	c	t	t	a	t	g	a	a	t	t	-	a	a	t	t	g	t	g	t	g	a	g
KU926319	2	g	t	a	a	c	a	g	t	a	c	a	t	c	t	a	t	g	a	c	a	t	c	a	g	g	g	a	c	a	t	g	g	g	t	t	c	g	g	t	g	g	c	a	g	c	t	c	g	a	a	g	g	c	t	t	a	t	g	a	a	t	t	-	a	a	t	t	g	t	g	t	g	a	a
KU926320	2	g	t	a	a	c	a	g	t	a	c	a	t	c	t	a	t	g	a	c	g	t	c	g	g	g	g	a	c	a	t	g	g	g	t	c	c	g	g	t	g	g	c	a	g	c	t	c	g	a	a	g	g	c	t	t	a	t	g	a	a	t	t	-	a	a	t	t	g	t	g	t	g	a	a
KU926321	2	g	t	a	a	c	a	g	t	a	c	a	t	c	t	a	t	g	a	c	a	t	c	a	g	g	g	a	c	a	t	g	g	g	t	t	c	g	g	t	g	g	c	a	g	c	t	c	g	a	a	g	g	c	t	t	a	t	g	a	a	t	t	-	a	a	t	t	g	t	g	t	g	a	a
KU926322	2	g	t	a	a	c	a	g	t	a	c	a	t	c	t	a	t	g	a	c	a	t	c	a	g	g	g	a	c	a	t	g	g	g	t	t	c	g	g	t	g	g	c	a	g	c	t	c	g	a	a	g	g	c	t	t	a	t	g	a	a	t	t	-	a	a	t	t	g	t	g	t	g	a	a
KC847290	2	g	t	a	a	c	a	g	t	a	c	a	t	c	t	a	t	g	a	c	a	t	c	a	g	g	g	a	c	a	t	g	g	g	t	t	c	g	g	t	g	g	c	a	g	c	t	c	g	a	a	g	g	c	t	t	a	t	g	a	a	t	t	-	a	a	t	t	g	t	g	t	g	a	a
KJ767491	2	g	t	a	a	c	a	g	t	a	c	a	t	c	t	a	t	g	a	c	a	t	c	a	g	g	g	a	c	a	t	g	g	g	t	t	c	g	g	t	g	g	c	a	g	c	t	c	g	a	a	g	g	c	t	t	a	t	g	a	a	t	t	-	a	a	t	t	g	t	g	t	g	a	a
KJ767492	2	g	t	a	a	c	a	g	t	a	c	a	t	c	t	a	t	g	a	c	a	t	c	a	g	g	g	a	c	a	t	g	g	g	t	t	c	g	g	t	g	g	c	a	g	c	t	c	g	a	a	g	g	c	t	t	a	t	g	a	a	t	t	-	a	a	t	t	g	t	g	t	g	a	a
KJ808816	2	g	t	a	a	c	a	g	t	a	c	a	t	c	t	a	t	g	a	c	a	t	c	a	g	g	g	a	c	a	t	g	g	g	t	t	c	g	g	t	g	g	c	a	g	c	t	c	g	a	a	g	g	c	t	t	a	t	g	a	a	t	t	-	a	a	t	t	g	t	g	t	g	a	a
KP771925	2	g	t	a	a	c	a	g	t	a	c	a	t	c	t	a	t	g	a	c	a	t	c	a	g	g	g	a	c	a	t	g	g	a	t	t	c	g	g	t	g	g	c	a	g	c	t	c	g	a	a	g	g	c	t	t	a	t	g	a	a	t	t	-	a	a	t	t	g	t	g	t	g	a	a
KU926315	2	g	t	a	a	c	a	g	t	a	c	a	t	c	t	a	t	g	a	c	a	t	c	a	g	g	g	a	c	a	t	g	g	g	t	t	c	g	g	t	g	g	c	a	g	c	t	c	g	a	a	g	g	c	t	t	a	t	g	a	a	t	t	-	a	a	t	t	g	t	g	t	g	a	a
KU926316	2	g	t	a	a	c	a	g	t	a	c	a	t	c	t	a	t	g	a	c	a	t	c	a	g	g	g	a	c	a	t	g	g	g	t	t	c	g	g	t	g	g	c	a	g	c	t	c	g	a	a	g	g	c	t	t	a	t	g	a	a	t	t	-	a	a	t	t	g	t	g	t	g	a	a
KU926317	2	g	t	a	a	c	a	g	t	a	c	a	t	c	t	a	t	g	a	c	a	t	c	a	g	g	g	a	c	a	t	g	g	g	t	t	c	g	g	t	g	g	c	a	g	c	t	c	g	a	a	g	g	c	t	t	a	t	g	a	a	t	t	-	a	a	t	t	g	t	g	t	g	a	a
KY037796	2	g	t	a	a	c	a	g	t	a	c	a	t	c	t	a	t	g	a	c	a	t	c	a	g	g	g	a	c	a	t	g	g	g	t	t	c	g	g	t	g	g	c	a	g	c	t	c	g	a	a	g	g	c	t	t	a	t	g	a	a	t	t	-	a	a	t	t	g	t	g	t	g	a	a
MF004348	2	g	t	a	a	c	a	g	t	a	c	a	t	c	t	a	t	g	a	c	a	t	c	a	g	g	g	a	c	a	t	g	g	g	t	t	c	g	g	t	g	g	c	a	g	c	t	c	g	a	a	g	g	c	t	t	a	t	g	a	a	t	t	-	a	a	t	t	g	t	g	t	g	a	a
MH709309	2	g	t	a	a	c	a	g	t	a	c	a	t	c	t	a	t	g	a	c	a	t	c	a	g	g	g	a	c	a	t	g	g	g	t	t	c	g	g	t	g	g	c	a	g	c	t	c	g	a	a	g	g	c	t	t	a	t	g	a	a	t	t	-	a	a	t	t	g	t	g	t	g	a	a
MH709310	2	g	t	a	a	c	a	g	c	a	c	a	t	c	t	a	t	g	a	c	a	t	c	a	g	g	g	a	c	a	t	g	g	g	t	t	c	g	g	t	g	g	c	a	g	c	t	c	g	a	a	g	g	c	t	t	a	t	g	a	a	t	t	-	a	a	t	t	g	t	g	t	g	a	a
MH709311	2	g	t	a	a	c	a	g	t	a	c	a	t	c	t	a	t	g	a	c	a	t	c	a	g	g	g	a	c	a	t	g	g	g	t	t	t	g	g	t	g	g	t	a	g	c	t	c	g	a	a	g	g	c	t	t	a	t	g	a	a	t	t	-	a	a	t	t	g	t	g	t	g	a	a
MH709313	2	g	t	a	a	c	a	g	t	a	c	a	t	c	t	a	t	g	a	c	a	t	c	a	g	g	g	a	c	a	t	g	g	g	t	t	c	g	g	t	g	g	c	a	g	c	t	c	g	a	a	g	g	c	t	t	a	t	g	a	a	t	t	-	a	a	t	t	g	t	g	t	g	a	a
MH709314	2	g	t	a	a	c	a	g	t	a	c	a	t	c	t	a	t	g	a	c	a	t	c	a	g	g	g	a	c	a	t	g	g	g	t	t	c	g	g	t	g	g	c	a	g	c	t	c	g	a	a	g	g	c	t	t	a	t	g	a	a	t	t	-	a	a	t	t	g	t	a	t	g	a	a
MH709315	2	g	t	a	a	c	a	g	t	a	c	a	t	c	t	a	t	g	a	c	a	t	c	a	g	g	g	a	c	a	t	g	g	g	t	t	c	g	g	t	g	g	c	a	g	c	t	c	g	a	a	g	g	c	t	t	a	t	g	a	a	t	t	-	a	a	t	t	g	t	a	t	g	a	a
MH709319	2	g	t	a	a	c	a	g	t	a	c	a	t	c	t	a	t	g	a	c	a	t	c	a	g	g	g	a	c	a	t	g	g	g	t	t	c	g	g	t	g	g	c	a	g	c	t	c	g	a	a	g	g	c	t	t	a	t	g	a	a	t	t	-	a	a	t	t	g	t	g	t	g	a	a
MH709320	2	g	t	a	a	c	a	g	t	a	c	a	t	c	t	a	t	g	a	c	a	t	c	a	g	g	g	a	c	a	t	g	g	g	t	t	c	g	g	t	g	g	c	a	g	c	t	c	g	a	a	g	g	c	t	t	a	t	g	a	a	t	t	-	a	a	t	t	g	t	g	t	g	a	a
MH709329	2	g	t	a	a	c	a	g	t	a	c	a	t	c	t	a	t	g	a	c	a	t	c	a	g	g	g	a	c	a	t	g	g	g	t	t	c	g	g	t	g	g	c	a	g	c	t	c	g	a	a	g	g	c	t	t	a	t	g	a	a	t	t	-	a	a	t	t	g	t	a	t	g	a	a
MH709331	2	g	t	a	a	c	a	g	t	a	c	a	t	c	t	a	t	g	a	c	a	t	c	a	g	g	g	a	c	a	t	g	g	g	t	t	c	g	g	t	g	g	c	a	g	c	t	c	g	a	a	g	g	c	t	t	a	t	g	a	a	t	t	-	a	a	t	t	g	t	g	t	g	a	a
MH709333	2	g	t	a	a	c	a	g	t	a	c	a	t	c	t	a	t	g	a	c	a	t	c	a	g	g	g	a	c	a	t	g	g	g	t	t	c	g	g	t	g	g	c	a	g	c	t	c	g	a	a	g	g	c	t	t	a	t	g	a	a	t	t	-	a	a	t	t	g	t	a	t	g	a	a
MH709337	2	g	t	a	a	c	a	g	t	a	c	a	t	c	t	a	t	g	a	c	a	t	c	a	g	g	g	a	c	a	g	g	g	g	t	t	c	g	g	t	g	g	c	a	g	c	t	c	g	a	a	g	g	c	t	g	a	t	g	a	a	t	t	-	a	a	t	t	g	t	g	t	g	a	a
MH709339	2	g	t	a	a	c	a	g	t	a	a	a	t	c	t	a	t	g	a	c	a	t	c	a	g	g	g	a	c	a	t	g	g	g	t	t	c	g	g	t	g	g	c	a	g	c	t	c	g	a	a	g	g	c	t	t	a	t	g	a	a	t	t	-	a	a	t	t	g	t	g	t	g	a	a
MH709344	2	g	t	a	a	c	a	g	t	a	c	a	t	c	t	a	t	g	a	c	a	t	c	a	g	g	g	a	c	a	t	g	g	g	t	t	c	g	g	t	g	g	c	a	g	c	t	c	g	a	a	g	g	c	t	t	a	t	g	a	a	t	t	-	a	a	t	t	g	t	g	t	g	a	a
MH709348	2	g	t	a	a	t	a	g	t	a	c	a	t	c	t	a	t	g	a	c	a	t	c	a	g	g	g	a	c	a	t	g	g	g	t	t	c	g	g	t	g	g	c	a	g	c	t	c	g	a	a	g	g	c	t	t	a	t	g	a	a	t	t	-	a	a	t	t	g	t	g	t	g	a	a
MH709355	2	g	t	a	a	c	a	g	t	a	c	a	t	c	t	a	t	g	a	c	a	t	c	a	g	g	g	a	c	a	t	g	g	g	t	t	c	g	g	t	g	g	c	a	g	c	t	c	g	a	a	g	g	c	t	t	a	t	g	a	a	t	t	-	a	a	t	t	g	t	g	t	g	a	a
MH709359	2	g	t	a	a	c	a	g	t	a	c	a	t	c	t	a	t	g	a	c	a	t	c	a	g	g	g	a	c	a	t	g	g	g	t	t	c	g	g	t	g	g	c	a	g	c	t	c	g	a	a	g	g	c	t	t	a	t	g	a	a	t	t	-	a	a	t	t	g	t	g	t	g	a	a
MH709363	2	g	t	a	a	c	a	g	t	a	c	a	t	c	t	a	t	g	a	c	a	t	c	a	g	g	g	a	c	a	t	g	g	g	t	t	c	g	g	t	g	g	c	a	g	c	t	c	g	a	a	g	g	c	t	t	a	t	g	a	a	t	t	-	a	a	t	t	g	t	g	t	g	a	a
MH709366	2	g	t	a	a	c	a	g	t	a	c	a	t	c	t	a	t	g	a	c	a	t	c	a	g	g	g	a	c	a	t	g	g	g	t	t	c	g	g	t	g	g	c	a	g	c	t	c	g	a	a	g	g	c	t	t	a	t	g	a	a	t	t	-	a	a	t	t	g	t	g	t	g	a	a
MH709376	2	g	t	a	a	c	a	g	t	a	c	a	t	c	t	a	t	g	a	c	a	t	c	a	g	g	g	a	c	a	t	g	g	g	t	t	c	g	g	t	g	g	c	a	g	c	t	c	g	a	a	g	g	c	t	t	a	t	g	a	a	t	t	-	a	a	t	t	g	t	g	t	g	a	a
MH709377	2	g	t	a	a	c	a	g	t	a	c	a	t	c	t	a	t	g	a	c	a	t	c	a	g	g	g	a	c	a	t	g	g	g	t	t	c	g	g	t	g	g	c	a	g	c	t	c	g	a	a	g	g	c	t	t	a	t	g	a	a	t	t	-	a	a	t	t	g	t	g	t	g	a	a
**MW316406**	2	g	t	a	a	c	a	g	t	a	c	a	t	c	t	a	t	g	a	c	a	t	c	a	g	g	g	a	c	a	t	g	g	g	t	t	c	g	g	t	g	g	c	a	g	c	t	c	g	a	a	g	g	c	t	t	a	t	g	a	a	t	t	-	a	a	t	t	n	t	g	t	g	a	a
AJ871403	3	g	t	a	a	c	a	g	t	a	c	a	t	c	t	a	t	g	a	c	a	t	c	a	g	a	g	a	c	a	t	t	g	g	t	t	c	g	g	t	g	g	c	a	g	c	t	c	g	a	a	g	g	c	t	t	a	t	g	c	a	t	t	-	a	g	t	t	g	t	g	t	g	a	a
DQ479955	3	g	t	a	a	c	a	g	t	a	c	a	t	c	t	a	t	g	a	c	a	t	c	a	g	a	g	a	c	a	t	g	g	g	t	t	c	g	g	t	g	g	c	a	g	c	t	c	g	a	a	g	g	c	t	t	a	t	g	c	a	t	t	-	a	g	t	t	g	t	g	t	g	a	a
DQ479956	3	g	t	a	a	c	a	g	t	a	c	a	t	c	t	a	t	g	a	c	a	t	c	a	g	a	g	a	c	a	t	g	g	g	t	t	c	g	g	t	g	g	c	a	g	c	t	c	g	a	a	g	g	c	t	t	a	t	g	c	a	t	t	-	a	g	t	t	g	t	g	t	g	a	a
DQ479957	3	g	t	a	a	c	a	g	t	a	c	a	t	c	t	a	t	g	a	c	a	t	c	a	g	a	g	a	c	a	t	g	g	g	t	t	c	g	g	t	g	g	c	a	g	c	t	c	g	a	a	g	g	c	t	t	a	t	g	c	a	t	t	-	a	g	t	t	g	t	g	t	g	a	a
JN704699	3	g	t	a	a	c	a	g	t	a	c	a	t	c	t	a	t	g	a	c	a	t	c	a	g	a	g	a	c	a	t	g	g	g	t	t	c	g	g	t	a	g	c	a	g	c	t	c	g	a	a	g	g	c	t	t	a	t	g	c	a	t	t	-	a	g	t	t	g	t	g	t	g	a	a
JN704700	3	g	t	a	a	c	a	g	t	a	c	a	t	c	t	a	t	g	a	c	a	t	c	a	g	a	g	a	c	a	t	g	g	g	t	t	c	g	g	t	g	g	c	a	g	c	t	c	g	a	t	g	g	t	t	t	a	t	g	c	a	t	t	-	a	g	t	t	g	t	g	t	g	a	a
JN704701	3	g	t	a	a	c	a	g	t	a	c	a	t	c	t	a	t	g	a	c	a	t	c	a	g	a	g	a	c	a	t	g	g	g	t	t	c	g	g	t	a	g	c	a	g	c	t	c	g	a	a	g	g	c	t	t	a	t	g	c	a	t	t	-	a	g	t	t	g	t	g	t	g	a	a
JN704702	3	g	t	a	a	c	a	g	t	a	c	a	t	c	t	a	t	g	a	t	a	t	c	a	g	a	g	a	c	a	t	g	g	g	t	t	c	g	g	t	g	g	c	a	g	c	t	c	g	a	a	g	g	t	t	t	a	t	g	c	a	t	t	-	a	g	t	t	g	t	g	t	g	a	a
JN704703	3	g	t	a	a	c	a	g	t	a	c	a	t	c	t	a	t	g	a	c	a	t	c	a	g	a	g	a	c	a	t	g	g	g	t	t	c	g	g	t	g	g	c	a	g	c	g	c	g	a	a	g	g	t	t	t	a	t	g	c	a	t	t	-	a	g	t	t	g	t	g	t	g	a	a
KP771891	3	g	t	a	a	c	a	g	t	a	c	a	t	c	t	a	t	g	a	c	a	t	c	a	g	a	g	g	c	a	t	g	g	g	t	t	c	g	g	t	a	g	c	t	g	c	t	c	g	a	a	g	g	c	t	t	a	t	g	c	a	t	t	-	a	g	t	t	g	t	g	t	g	g	a
KP771893	3	g	t	a	a	c	a	g	t	a	c	a	t	c	t	a	t	g	a	c	a	t	c	a	g	a	g	a	c	a	t	g	g	g	t	t	c	g	g	t	a	g	c	a	g	c	t	c	g	a	a	g	g	c	t	t	a	t	g	c	a	t	t	-	a	g	t	t	g	t	g	t	g	g	a
KP771895	3	g	t	a	a	c	a	g	t	a	c	a	t	c	t	a	t	g	a	c	a	t	c	a	g	a	g	a	c	a	t	t	g	g	t	t	c	g	g	t	g	g	c	a	g	c	t	c	g	a	a	g	g	c	t	t	a	t	g	c	a	t	t	-	a	g	t	t	g	t	g	t	g	a	a
KP771896	3	g	t	a	a	c	a	g	t	a	c	a	t	c	t	a	t	g	c	c	a	t	c	a	g	a	g	a	c	a	t	g	g	g	t	t	c	g	g	t	g	g	c	a	g	c	t	c	g	a	a	g	g	c	t	t	a	t	g	c	a	t	t	-	a	g	t	t	g	t	g	t	g	a	a
KP771897	3	g	t	a	a	c	a	g	t	a	c	a	t	c	t	a	t	g	a	c	a	t	c	a	g	a	g	a	c	a	t	g	g	g	t	t	c	g	g	t	g	g	c	a	g	c	t	c	g	a	a	g	g	c	t	t	a	t	g	c	a	t	t	-	a	g	t	t	g	t	g	t	g	a	a
KP771899	3	g	t	a	a	c	a	g	t	a	c	a	t	c	t	a	t	g	a	c	a	t	c	a	g	a	g	a	c	a	t	t	g	g	t	t	c	g	g	t	g	g	c	a	g	c	t	c	g	a	a	g	g	c	t	t	a	t	g	c	a	t	t	-	a	g	t	t	g	t	g	t	g	a	a
KP771903	3	g	t	a	a	c	a	g	t	a	c	a	t	c	t	a	t	g	a	c	a	t	c	a	g	a	g	a	c	a	t	g	g	g	t	t	c	g	g	t	a	g	c	t	g	c	t	c	g	a	a	g	g	c	t	t	a	t	g	c	a	t	t	-	a	g	t	t	g	t	g	t	g	a	a
KP771907	3	g	t	a	a	c	a	g	t	a	c	a	t	c	t	a	t	g	a	c	a	t	c	a	g	a	g	a	c	a	t	t	g	g	t	t	c	g	g	t	g	g	c	a	g	c	t	c	g	a	a	g	g	c	t	t	a	t	g	c	a	t	t	-	a	g	t	t	g	t	g	t	g	a	a
KP771910	3	g	t	a	a	c	a	g	t	a	c	a	t	c	t	a	t	g	a	c	a	t	c	a	g	a	g	a	c	a	t	g	g	g	t	t	c	g	g	t	a	g	c	a	g	c	t	c	g	a	a	g	g	c	t	t	a	t	g	c	a	t	t	-	a	g	t	t	g	t	g	t	g	a	a
KP771915	3	g	t	a	a	c	a	g	t	a	c	a	t	c	t	a	t	g	a	c	a	t	c	a	g	a	g	a	c	a	t	g	g	g	t	t	c	g	g	t	g	g	c	a	g	c	t	c	a	a	a	g	g	c	t	t	a	t	g	c	a	t	t	-	a	g	t	t	g	t	g	t	g	a	a
KP771916	3	g	t	a	a	c	a	g	t	a	c	a	t	c	t	a	t	g	a	c	a	t	c	a	g	a	g	a	c	a	t	g	g	g	t	t	c	g	g	t	g	g	c	a	g	c	t	c	g	a	a	g	g	c	t	t	a	t	g	c	a	t	t	-	a	g	t	t	g	t	g	t	g	a	a
KP771921	3	g	t	a	a	c	a	g	t	a	c	a	t	c	t	a	t	g	a	c	a	t	c	a	g	a	g	a	c	a	t	g	g	g	t	t	c	g	g	t	a	g	c	a	g	c	t	c	g	a	a	g	g	c	t	t	a	t	g	c	a	t	t	-	a	g	t	t	g	t	g	t	g	a	a
KP771923	3	g	t	g	g	c	a	g	t	a	c	a	t	c	t	a	t	g	a	c	a	t	c	a	g	a	g	a	c	a	t	g	g	g	t	t	c	g	g	t	a	g	c	a	g	c	t	c	g	a	a	g	g	c	t	t	a	t	g	c	a	t	t	-	a	g	t	t	g	t	g	t	g	a	a
KP771889	3	g	t	a	a	c	a	g	t	a	c	a	t	c	t	a	t	g	a	c	a	t	c	a	g	a	g	a	c	a	t	g	g	g	t	t	c	g	g	t	a	g	c	a	g	c	t	c	a	a	a	g	g	c	t	t	a	t	g	c	a	t	t	-	a	g	t	t	g	t	g	t	g	a	a
KU926311	3	g	t	a	a	c	a	g	t	a	c	a	t	c	t	a	t	g	a	c	a	t	c	a	g	a	g	a	c	a	t	g	g	g	t	t	c	g	g	t	g	g	c	a	g	c	t	c	g	a	a	g	g	c	t	t	a	t	g	c	a	t	t	-	a	g	t	t	g	t	g	t	g	a	a
JQ911599	3	g	t	a	a	c	a	g	t	a	c	a	t	c	t	a	t	g	a	c	a	t	c	a	g	a	g	a	c	a	t	g	g	g	t	t	c	g	g	t	g	g	c	a	g	c	t	c	g	a	a	g	g	c	t	t	a	t	g	c	a	t	t	-	a	g	t	t	g	t	g	t	g	a	a
MH709317	3	g	t	a	a	c	a	g	t	a	c	a	t	c	t	a	t	g	a	c	a	t	c	a	g	a	g	a	c	a	t	g	t	g	t	t	c	a	g	t	g	g	c	a	g	c	t	c	g	a	a	g	g	c	t	t	a	t	g	a	a	t	t	-	a	g	t	t	g	t	g	t	g	a	a
MH709346	3	g	t	a	a	c	a	g	t	a	c	a	t	c	t	a	t	g	a	c	a	t	c	a	g	a	g	a	c	a	t	g	t	g	t	t	c	g	g	t	g	g	c	a	g	c	t	c	g	a	a	g	g	c	t	t	a	t	g	a	a	t	t	-	a	g	t	t	g	t	g	t	g	a	a
MH709375	3	g	t	a	a	c	a	g	t	a	c	a	t	c	t	a	t	g	a	c	a	t	c	a	g	a	g	a	c	a	t	g	g	g	t	t	c	g	g	t	g	g	c	a	g	c	t	c	g	a	a	g	g	c	t	t	a	t	g	a	a	t	t	-	a	g	t	t	g	t	g	t	g	a	a
DQ452050	4	g	t	a	a	c	a	g	t	a	c	a	t	c	t	a	t	g	a	c	a	t	c	a	g	a	g	a	c	a	t	g	g	g	t	t	c	g	g	t	g	g	c	a	g	c	t	c	g	a	t	g	g	t	t	t	a	t	g	a	a	t	t	-	a	g	t	t	g	g	g	t	g	a	a
DQ479960	4	g	t	a	a	c	a	g	t	a	c	a	t	c	t	a	t	g	a	c	a	t	c	a	g	a	g	a	c	a	t	g	g	g	t	t	c	g	g	t	g	g	c	a	g	c	t	c	g	a	t	g	g	t	t	t	a	t	g	a	a	t	t	-	a	g	t	t	g	g	g	t	g	a	a
KP771898	4	g	t	a	a	c	a	g	t	a	c	a	t	c	t	a	t	g	a	c	a	t	c	a	g	a	g	a	c	a	t	g	g	g	t	t	c	g	g	t	g	g	c	a	g	c	t	c	g	a	t	g	g	t	t	t	a	t	g	a	a	g	t	-	a	g	t	t	g	g	g	t	g	a	a
MH709316	4	t	t	a	a	c	a	g	t	a	c	a	t	c	t	a	t	g	a	c	a	t	c	a	g	a	g	a	c	a	t	g	g	g	t	t	c	g	g	t	g	g	c	a	g	c	t	c	g	a	t	g	g	t	t	t	a	t	g	a	a	t	t	-	a	g	t	t	g	t	g	t	g	a	a
MH709318	4	g	t	a	a	c	a	g	t	a	c	a	t	c	t	a	t	g	a	c	a	t	c	a	g	a	g	a	c	a	t	g	g	g	t	t	c	g	g	t	g	g	c	a	g	c	t	c	g	a	t	g	g	t	t	t	a	t	g	a	a	t	t	-	a	g	t	t	g	g	g	t	g	a	a
MH709322	4	g	t	a	a	c	a	g	t	a	c	a	t	c	t	a	t	g	a	c	a	t	c	a	g	a	g	a	c	a	t	g	g	g	t	t	c	g	g	t	g	g	c	a	g	c	t	c	g	a	t	g	g	t	t	t	a	t	g	a	a	t	t	-	a	g	t	t	g	g	g	t	g	a	a
MH709323	4	g	t	a	a	c	a	g	t	a	c	a	t	c	t	a	t	g	a	c	a	t	t	a	g	a	g	a	c	a	t	g	g	g	t	t	c	g	g	t	g	g	c	a	g	c	t	c	g	a	t	g	g	t	t	t	a	t	g	a	a	t	t	-	a	g	t	t	g	g	g	t	g	a	a
MH709326	4	g	t	a	a	c	a	g	t	a	c	a	t	c	t	a	t	g	a	c	a	t	c	a	g	a	g	a	c	a	t	g	g	g	t	t	c	g	g	t	g	g	c	a	g	c	t	c	g	a	t	g	g	t	t	t	a	t	g	a	a	t	t	-	a	g	t	t	g	g	g	t	g	a	a
MH709341	4	g	t	a	a	c	a	g	t	a	c	a	t	c	t	a	t	g	a	c	a	t	c	a	g	a	g	a	c	a	t	g	g	g	t	t	c	g	g	t	g	g	c	a	g	c	t	c	g	a	t	g	g	t	t	t	a	t	g	a	a	t	t	-	a	g	t	t	g	g	g	t	g	a	a
MH709357	4	g	t	a	a	c	a	g	t	a	c	a	t	c	t	a	t	g	a	c	a	t	c	a	g	a	g	a	c	c	t	g	g	g	t	t	c	g	g	t	g	g	c	a	g	c	t	c	g	a	t	g	g	t	t	t	a	t	g	a	a	t	t	-	a	g	t	t	g	g	g	t	g	a	a
MH709364	4	g	t	a	a	c	a	g	t	a	c	a	t	c	t	a	t	g	a	c	a	t	c	a	g	a	g	a	c	a	t	g	g	g	t	t	c	g	g	t	g	a	c	a	g	c	t	c	g	a	t	g	g	t	t	t	a	t	g	a	a	t	t	-	a	g	t	t	g	g	g	t	g	a	a
MH709370	4	g	t	a	a	c	a	a	t	a	c	a	t	c	t	a	t	g	a	c	a	t	c	a	g	a	g	a	c	a	t	g	g	g	t	t	c	g	g	t	g	g	c	a	g	c	t	c	g	a	t	g	g	t	t	t	a	t	g	a	a	t	t	-	a	g	t	t	g	g	g	t	g	a	a
MH709371	4	g	t	a	a	c	a	g	t	a	c	a	t	c	t	a	t	g	a	c	a	t	c	a	g	a	g	a	c	a	t	g	g	g	t	t	c	g	g	t	g	g	c	a	g	c	t	c	g	a	t	g	g	t	t	t	a	t	g	a	a	t	t	-	a	g	t	t	g	g	g	t	g	a	a
MH709372	4	g	t	a	a	c	a	g	t	a	c	a	t	c	t	a	t	g	a	c	a	t	c	a	g	a	g	a	c	a	t	g	g	g	t	t	c	g	g	t	g	g	c	a	g	c	t	t	g	a	t	g	g	t	t	t	a	t	g	a	a	t	t	-	a	g	t	t	g	g	g	t	g	a	a
DQ457052	5	g	t	a	a	c	a	g	t	a	c	a	t	c	t	a	t	g	a	c	a	t	c	a	g	a	g	a	c	a	t	g	g	g	t	t	t	g	g	t	g	g	c	a	g	c	t	c	g	a	t	g	g	t	t	t	a	c	g	a	a	t	t	-	a	g	t	t	g	g	g	t	g	a	a
JN704704	5	g	t	a	a	c	a	g	t	a	c	a	t	c	t	a	t	g	a	c	a	t	c	a	g	a	g	a	c	a	t	g	g	g	t	t	t	g	g	t	g	g	c	a	g	c	t	c	g	a	t	g	g	t	t	t	a	c	g	a	a	t	t	-	a	g	t	t	g	g	g	t	g	a	a
JN704705	5	g	t	a	a	c	a	g	t	a	c	a	t	c	t	a	t	g	a	c	a	t	c	a	g	a	g	a	c	a	t	g	g	g	t	t	t	g	g	t	g	g	c	a	g	c	t	c	g	a	t	g	g	t	t	t	a	c	g	a	a	t	t	-	a	g	t	t	g	g	g	t	g	a	a
JN704706	5	g	t	a	a	c	a	g	t	a	c	a	t	c	t	a	t	g	a	c	a	t	c	a	g	a	g	a	c	a	t	g	g	g	t	t	t	g	g	t	g	g	c	a	g	c	t	c	g	a	t	g	g	t	t	t	a	c	g	a	a	t	t	-	c	g	t	t	g	g	g	t	g	a	a
JN704707	5	g	t	a	a	c	a	g	t	a	c	a	t	c	t	a	t	g	a	c	a	t	c	a	g	a	g	a	c	a	t	g	g	g	g	t	t	g	g	t	g	g	c	a	g	c	t	c	g	a	t	g	g	t	t	t	a	c	g	a	a	t	t	-	a	g	t	t	g	g	g	t	g	a	a
JN704708	5	g	t	a	a	c	a	g	t	a	c	a	t	c	t	a	t	g	a	c	a	t	c	a	g	a	g	a	c	a	t	g	g	g	t	t	t	g	g	t	g	g	c	a	g	c	t	c	g	a	t	g	g	t	t	t	a	c	g	a	a	t	t	-	a	g	t	t	g	g	g	t	g	a	a
KP771904	5	g	t	a	a	c	a	g	t	a	c	a	t	c	t	a	t	g	a	c	a	t	c	a	g	a	g	a	c	a	t	g	g	g	t	t	t	g	g	t	g	g	c	a	g	c	t	c	g	a	t	g	g	t	t	t	a	c	g	a	a	t	t	-	a	g	t	t	g	g	g	t	g	a	a
KP771905	5	g	t	a	a	c	a	g	t	a	c	a	t	c	t	a	t	g	a	c	a	t	c	a	g	a	g	a	c	a	t	g	g	g	t	t	t	g	g	t	g	g	c	a	g	c	t	c	g	a	t	g	g	t	t	t	a	c	g	a	a	t	t	-	a	g	t	t	g	g	g	t	g	a	a
KP771906	5	g	t	a	a	c	a	g	t	a	c	a	t	c	t	a	t	g	a	c	a	t	c	a	g	a	g	a	c	a	t	g	g	g	t	t	t	g	g	t	g	g	c	a	g	c	t	c	g	a	t	g	g	t	t	t	a	c	g	a	a	t	t	-	a	g	t	t	g	g	g	t	g	a	a
KP771919	5	g	t	a	a	c	a	g	t	a	c	a	t	c	t	a	t	g	a	c	a	t	c	a	g	a	g	a	c	a	t	g	g	g	t	t	t	g	g	t	g	g	c	a	g	c	t	c	g	a	t	g	g	t	t	t	a	c	g	a	a	t	t	-	a	g	t	t	g	g	g	t	g	a	a
KP771922	5	g	t	a	a	c	a	g	t	a	c	a	t	a	t	a	t	g	a	c	a	t	c	a	g	a	g	a	c	a	t	g	g	g	g	t	t	g	g	t	g	g	c	a	g	c	t	c	g	a	t	g	g	t	t	t	a	c	g	a	a	t	t	-	a	g	t	t	g	g	g	t	g	a	a
KP771924	5	g	t	a	a	c	a	g	t	a	c	a	t	c	t	a	t	g	a	c	a	t	c	a	g	a	g	a	c	a	t	g	g	g	g	t	t	g	g	t	g	g	c	a	g	c	t	c	g	a	t	g	g	t	t	t	a	c	g	a	a	t	t	-	a	g	t	t	g	g	g	t	g	a	a
KM355696	5	g	t	a	a	c	a	g	t	a	c	a	t	c	t	a	t	g	a	c	a	t	c	a	g	a	g	a	c	a	t	g	g	g	t	t	t	g	g	t	g	g	c	a	g	c	t	c	g	a	t	g	g	t	t	t	a	c	g	a	a	t	t	-	a	g	t	t	g	g	g	t	g	a	a
KM355697	5	g	t	a	a	c	a	g	t	a	c	a	t	c	t	a	t	g	a	c	a	t	c	a	g	a	g	a	c	a	t	g	g	g	t	t	t	g	g	t	g	g	c	a	g	c	t	c	g	a	t	g	g	t	t	t	a	c	g	a	a	t	t	-	a	g	t	t	g	g	g	t	g	a	a
KM355698	5	g	t	a	a	c	a	g	t	a	c	a	t	c	c	a	t	g	a	c	a	t	c	a	g	a	g	a	c	a	t	g	g	g	t	t	t	g	g	g	g	g	c	a	g	c	t	c	g	a	t	g	g	t	t	t	a	c	g	a	a	t	t	-	a	g	t	t	g	g	g	t	g	a	a
KM355699	5	g	t	a	a	c	a	g	t	a	c	a	t	c	c	a	t	g	a	c	a	t	c	a	g	a	g	a	c	a	t	g	g	g	t	t	t	g	g	g	g	g	c	a	g	c	t	c	g	a	t	g	g	t	t	t	a	c	g	a	a	t	t	-	a	g	t	t	g	g	g	t	g	a	a
KM355701	5	g	t	a	a	c	a	g	t	a	c	a	t	c	t	a	t	g	a	c	a	t	c	a	g	a	g	a	c	a	t	g	g	g	t	t	t	g	g	t	g	g	c	a	g	c	t	c	g	a	t	g	g	t	t	t	a	c	g	a	a	t	t	-	a	g	t	t	g	g	g	t	g	a	a
KM355702	5	g	t	a	a	c	a	g	t	a	c	a	t	c	c	a	t	g	a	c	a	t	c	a	g	a	g	a	c	a	t	g	g	g	t	t	t	g	g	g	g	g	c	a	g	c	t	c	g	a	t	g	g	t	t	t	a	c	g	a	a	t	t	-	a	g	t	t	g	g	g	t	g	a	a
KM355703	5	g	t	a	a	c	a	g	t	a	c	a	t	c	t	a	t	g	a	c	a	t	c	a	g	a	g	a	c	a	t	g	g	g	t	t	t	g	g	t	g	g	c	a	g	c	t	c	g	a	t	g	g	t	t	t	a	c	g	a	a	t	t	-	a	g	t	t	g	g	g	t	g	a	a
KM355704	5	g	t	a	a	c	a	g	t	a	c	a	t	c	t	a	t	g	a	c	a	t	c	a	g	a	g	a	c	a	t	g	g	g	t	t	t	g	g	t	g	g	c	a	g	c	t	c	g	a	t	g	g	t	t	t	a	c	g	a	a	t	t	-	a	g	t	t	g	g	g	t	g	a	a
KM355705	5	g	t	a	a	c	a	g	t	a	c	a	t	c	c	a	t	g	a	c	a	t	c	a	g	a	g	a	c	a	t	g	g	g	t	t	t	g	g	g	g	g	c	a	g	c	t	c	g	a	t	g	g	t	t	t	a	c	g	a	a	t	t	-	a	g	t	t	g	g	g	t	g	a	a
KM355706	5	g	t	a	a	c	a	g	t	a	c	a	t	c	c	a	t	g	a	c	a	t	c	a	g	a	g	a	c	a	t	g	g	g	t	t	t	g	g	g	g	g	c	a	g	c	t	c	g	a	t	g	g	t	t	t	a	c	g	a	a	t	t	-	a	g	t	t	g	g	g	t	g	a	a
KM355707	5	g	t	a	a	c	a	g	t	a	c	a	t	c	t	a	t	g	a	c	a	t	c	a	g	a	g	a	c	a	t	g	g	g	t	t	t	g	g	t	g	g	c	a	g	c	t	c	g	a	t	g	g	t	t	t	a	c	g	a	a	t	t	-	a	g	t	t	g	g	g	t	g	a	a
KM355708	5	g	t	a	a	c	a	g	t	a	c	a	t	c	t	a	t	g	a	c	a	t	c	a	g	a	g	a	c	a	t	g	g	g	t	t	t	g	g	t	g	g	c	a	g	c	t	c	g	a	t	g	g	t	t	t	a	c	g	a	a	t	t	-	a	g	t	t	g	g	g	t	g	a	a
KM355709	5	g	t	a	a	c	a	g	t	a	c	a	t	c	c	a	t	g	a	c	a	t	c	a	g	a	g	a	c	a	t	g	g	g	t	t	t	g	g	g	g	g	c	a	g	c	t	c	g	a	t	g	g	t	t	t	a	c	g	a	a	t	t	-	a	g	t	t	g	g	g	t	g	a	a
KM355711	5	g	t	a	a	c	a	g	t	a	c	a	t	c	c	a	t	g	a	c	a	t	c	a	g	a	g	a	c	a	t	g	g	g	t	t	t	g	g	g	g	g	c	a	g	c	t	c	g	a	t	g	g	t	t	t	a	c	g	a	a	t	t	-	a	g	t	t	g	g	g	t	g	a	a
KM355712	5	g	t	a	a	c	a	g	t	a	c	a	t	c	c	a	t	g	a	c	a	t	c	a	g	a	g	a	c	a	t	g	g	g	t	t	t	g	g	g	g	g	c	a	g	c	t	c	g	a	t	g	g	t	t	t	a	c	g	a	a	t	t	-	a	g	t	t	g	g	g	t	g	a	a
KY037797	5	[illegible]	[illegible]	[illegible]	[illegible]	[illegible]	[illegible]	[illegible]	[illegible]	[illegible]	[illegible]	[illegible]	[illegible]	[illegible]	[illegible]	[illegible]	[illegible]	[illegible]	[illegible]	[illegible]	[illegible]	[illegible]	[illegible]	[illegible]	[illegible]	[illegible]	[illegible]	[illegible]	[illegible]	[illegible]	[illegible]	[illegible]	[illegible]	[illegible]	[illegible]	[illegible]	[illegible]	[illegible]	[illegible]	[illegible]	[illegible]	[illegible]	[illegible]	[illegible]	[illegible]	[illegible]	[illegible]	[illegible]	[illegible]	[illegible]	[illegible]	[illegible]	[illegible]	[illegible]	[illegible]	[illegible]	[illegible]	[illegible]	[illegible]	[illegible]	[illegible]	[illegible]	[illegible]	[illegible]	[illegible]	[illegible]	[illegible]	[illegible]	[illegible]	[illegible]	[illegible]	[illegible]	[illegible]	[illegible]	[illegible]

Supplementary Table 3: 2880 SN

GB-ID	Clade markers																																																																										
	Position in Dumas	18547	18603	18626	18872	18878	18905	18944	18969	19036	19064	19097	19196	19323	19373	19374	19431	19461	19476	19485	19503	19619	19667	19698	19707	19719	19746	19770	19838	19845	19924	19981	19996	20019	20076	20089	20096	20158	20164	20181	20184	20208	20388	20399	20406	20508	20532	20547	20631	20642	20656	20665	20666	20684	20687	21021	21057	21059	21127	21130	21156	21294	21297	21300	21350	21371	21509	21557	21595	21734	21777	21854	21882	21929	22005
MH499467	5	g	t	a	a	c	a	g	t	a	c	a	t	c	t	a	t	-	a	c	a	t	c	a	g	a	g	a	c	a	t	g	g	g	t	t	t	g	g	t	g	g	c	a	g	c	t	c	g	a	t	g	g	t	t	t	-	-	g	a	a	t	c	t	a	g	t	t	g	g	g	t	g	a	a
MH499469	5	g	t	a	a	c	a	g	t	a	c	a	t	c	t	a	t	g	a	c	a	t	c	a	g	a	g	a	c	a	t	g	g	g	t	t	t	g	g	t	g	g	c	a	g	c	t	c	g	a	t	g	g	t	t	t	a	c	g	a	a	t	t	-	a	g	t	t	g	g	g	t	g	a	a
MH709321	5	g	t	a	a	c	a	g	t	a	c	a	t	c	t	a	t	g	a	c	a	t	c	a	g	a	g	a	c	a	t	g	g	g	t	t	t	g	g	t	g	g	c	a	g	c	t	c	g	a	t	g	g	t	t	t	a	c	g	a	a	t	t	-	c	g	t	t	g	g	g	t	g	a	a
MH709324	5	g	t	a	a	c	a	g	t	a	c	a	t	c	t	a	t	g	a	c	a	t	c	a	g	a	g	a	c	a	t	g	g	g	t	t	t	g	g	t	g	g	c	a	g	c	t	c	g	a	t	g	g	t	t	t	a	c	g	a	a	t	t	-	a	g	t	t	g	g	g	t	g	a	a
MH709325	5	g	t	a	a	c	a	g	t	a	c	a	t	c	t	a	t	g	a	c	a	t	c	a	g	a	g	a	c	a	t	g	g	g	t	t	t	g	g	g	g	g	c	a	g	c	t	c	g	a	t	g	g	t	t	t	c	c	g	a	a	t	t	-	a	g	t	t	g	g	g	t	g	a	a
MH709327	5	g	t	a	a	c	a	g	t	a	c	a	t	c	t	a	t	g	a	c	a	t	c	a	g	a	g	a	c	a	t	g	g	g	t	t	t	g	g	t	g	g	c	a	g	c	t	c	g	a	t	g	g	t	t	t	a	c	g	a	a	t	t	-	a	g	t	t	g	g	g	t	g	a	a
MH709328	5	g	t	a	a	c	a	g	t	a	c	a	t	c	t	a	t	g	a	c	a	t	c	a	g	a	g	a	c	a	t	g	g	g	t	t	t	g	g	t	g	g	c	a	g	c	t	c	g	a	t	g	g	t	t	t	a	c	g	a	a	t	t	-	a	g	t	t	g	g	g	t	g	a	a
MH709330	5	g	t	a	a	c	a	g	t	a	c	a	t	c	t	a	t	g	a	c	a	t	c	a	g	a	g	a	c	a	t	g	g	g	t	t	t	g	g	t	g	g	c	a	g	c	t	c	g	a	t	g	g	t	t	t	a	c	g	a	a	t	t	-	a	g	t	t	g	g	g	t	g	a	a
MH709332	5	g	t	a	a	c	a	g	t	a	c	a	t	c	t	a	t	g	a	c	a	t	c	a	g	a	g	a	c	a	t	g	g	g	t	t	t	g	g	t	g	g	c	a	a	c	t	c	g	a	t	a	a	t	t	t	a	c	g	a	a	t	t	-	a	g	t	t	g	g	g	t	g	a	a
MH709334	5	g	t	a	a	c	a	g	t	a	c	a	t	c	t	a	t	g	a	c	a	t	c	a	g	a	g	a	c	a	t	g	g	g	t	t	t	g	g	t	g	g	c	a	g	c	t	c	g	a	t	g	g	t	t	t	a	c	g	a	a	t	t	-	a	g	t	t	g	g	g	t	g	a	a
MH709335	5	g	t	a	a	c	a	g	t	a	c	a	t	c	t	a	t	g	a	c	a	t	c	a	g	a	g	a	c	a	t	g	g	g	t	t	t	g	g	t	g	g	c	a	g	c	t	c	g	a	t	g	g	t	t	t	a	c	g	a	a	t	t	-	a	g	t	t	g	g	g	t	g	a	a
MH709336	5	g	t	a	a	c	a	g	t	a	c	a	t	c	t	a	t	g	a	c	a	t	c	a	g	a	g	a	c	a	t	g	g	g	t	t	t	g	g	t	g	g	c	a	g	c	t	c	g	a	t	g	g	t	t	t	a	c	g	a	a	t	t	-	a	g	t	t	g	g	g	t	g	a	a
MH709338	5	g	t	a	a	c	a	g	t	a	c	a	t	c	t	a	t	g	a	c	a	t	c	a	g	a	g	a	c	a	t	g	g	g	t	t	t	g	g	t	g	g	c	a	g	c	t	c	g	a	t	g	g	t	t	t	a	c	g	a	a	t	t	-	a	g	t	t	g	g	g	t	g	a	a
MH709340	5	g	t	a	a	c	a	g	t	a	c	a	t	c	t	a	t	g	a	c	a	t	c	a	g	a	g	a	c	a	t	g	g	g	t	t	t	g	g	t	g	g	c	a	g	c	t	c	g	a	t	g	g	t	c	t	a	c	g	a	a	t	t	-	a	g	t	t	g	g	g	t	g	a	a
MH709342	5	g	t	a	a	c	a	g	t	a	c	a	t	c	t	a	t	g	a	c	a	t	c	a	g	a	g	a	c	a	t	g	g	g	t	t	t	g	g	t	g	g	c	a	g	c	t	c	g	a	t	g	g	t	t	t	a	c	g	a	a	t	t	-	c	g	t	t	g	g	g	t	g	a	a
MH709343	5	g	t	a	a	c	a	g	t	a	c	a	t	c	t	a	t	g	a	c	a	t	c	a	g	a	g	a	c	a	t	g	g	g	t	t	t	g	g	t	g	g	c	a	g	a	t	c	a	a	t	g	g	t	t	t	a	c	g	a	a	t	t	-	a	g	t	t	g	g	g	t	g	a	a
MH709345	5	g	t	a	a	c	a	g	t	a	c	a	g	c	t	a	t	g	a	c	a	t	c	a	g	a	g	a	c	a	t	g	g	g	t	t	t	g	g	t	g	g	c	a	g	c	t	c	g	a	t	g	g	t	t	t	a	c	g	a	a	t	t	-	a	g	t	t	g	g	g	t	g	a	a
MH709347	5	g	t	a	a	c	a	g	t	a	c	a	t	c	t	a	t	g	a	c	a	t	c	a	g	a	g	a	c	a	t	g	g	g	t	t	t	g	g	t	g	g	c	a	g	c	t	c	g	a	t	g	g	t	t	t	a	c	g	a	a	t	t	-	a	g	t	t	g	g	g	t	g	a	a
MH709349	5	g	t	a	a	c	a	g	t	a	c	a	t	c	t	a	t	g	a	c	a	t	c	a	g	a	g	a	c	a	t	g	g	g	t	t	t	g	g	t	g	g	c	a	g	c	t	c	g	a	t	g	g	t	t	t	a	c	g	a	a	t	t	-	a	g	t	t	g	g	g	t	g	a	a
MH709350	5	g	t	a	a	c	a	g	t	a	c	a	t	c	t	a	t	g	a	c	a	t	c	a	g	a	g	a	c	a	t	g	g	g	t	t	t	g	g	t	g	g	c	a	g	c	t	c	g	a	t	g	g	t	t	t	a	c	g	a	a	t	t	-	a	g	t	t	g	g	g	t	g	a	a
MH709351	5	g	c	a	a	c	a	g	t	a	c	g	t	c	t	a	t	g	a	c	a	t	c	a	g	a	g	a	c	a	t	g	g	g	t	t	t	g	g	t	g	g	c	a	g	c	t	c	g	a	t	g	g	t	t	t	a	c	g	a	a	t	t	-	a	g	t	t	g	g	g	t	g	a	a
MH709353	5	g	t	a	a	c	a	g	t	a	c	a	t	c	t	a	t	g	a	c	a	t	c	a	g	a	g	a	c	a	t	g	g	g	t	t	t	g	g	t	g	g	c	a	g	c	t	c	g	a	t	g	g	t	t	t	a	c	g	a	a	t	t	-	a	g	t	t	g	g	g	t	g	a	a
MH709358	5	g	t	a	a	c	a	g	t	a	c	a	t	c	t	a	t	g	a	c	a	t	c	a	g	a	g	a	c	a	t	g	g	g	t	t	t	g	g	t	g	g	c	a	g	c	t	c	g	a	t	g	g	t	t	t	a	c	g	a	a	t	t	-	a	g	t	t	g	g	g	t	g	a	a
MH709360	5	g	t	a	a	c	a	g	t	a	c	a	t	c	t	a	t	g	a	c	a	t	c	a	g	a	g	a	c	a	t	g	g	g	t	t	t	g	g	t	g	g	c	a	g	c	t	c	g	a	t	g	g	t	t	t	a	c	g	a	a	t	t	-	a	g	t	t	g	g	g	t	g	a	a
MH709362	5	g	t	a	a	c	a	g	t	a	c	a	t	c	t	a	t	g	a	c	a	t	c	a	g	a	g	a	c	a	t	g	g	g	t	t	t	g	g	t	g	g	c	a	g	c	t	c	g	a	t	g	g	t	t	t	a	c	g	a	a	t	t	-	a	g	t	t	g	g	g	t	g	a	a
MH709365	5	g	t	a	a	c	a	g	t	a	c	a	t	c	t	a	t	g	a	c	a	t	c	a	g	a	g	a	c	a	t	g	g	g	t	t	t	g	g	t	g	g	c	a	g	c	t	c	g	a	t	g	g	t	t	t	a	c	g	a	a	t	t	-	a	g	t	t	g	g	g	t	g	a	a
MH709373	5	g	t	a	a	c	a	g	t	a	c	a	t	c	t	a	t	g	a	c	a	t	c	a	g	a	g	a	c	a	t	g	g	g	g	t	t	g	g	t	g	g	c	a	g	c	t	c	g	a	t	g	g	t	t	t	a	c	g	a	a	t	t	-	a	g	t	t	g	g	g	t	g	a	a
MH709374	5	g	t	a	a	c	a	g	t	a	c	a	t	c	t	a	t	g	a	c	a	t	c	a	g	a	g	a	c	a	t	g	g	g	t	t	t	g	g	t	g	g	c	a	g	c	t	c	g	a	t	g	g	t	t	t	a	c	g	a	a	t	t	-	a	g	t	t	g	g	g	t	g	a	a
KC112914	6	g	t	a	a	c	a	g	t	a	c	a	t	c	t	a	t	g	a	c	a	t	c	a	g	a	g	a	c	a	t	g	g	g	t	t	c	g	g	t	g	g	c	a	g	c	t	c	g	a	t	g	g	t	t	t	a	t	g	a	a	t	t	-	a	g	t	t	g	g	g	t	g	a	a
KP771914	6	g	t	a	a	c	a	g	t	a	c	a	t	c	t	a	t	g	a	c	a	t	c	a	g	a	g	a	c	a	t	g	g	g	t	t	c	g	g	t	g	g	c	a	g	c	t	c	g	a	t	g	g	t	t	t	a	t	g	a	a	t	t	-	a	g	t	t	g	g	g	t	g	a	a
KP702725	6	g	t	a	a	c	a	g	t	a	c	a	t	c	t	a	t	g	a	c	a	t	c	a	g	a	g	a	c	a	t	g	g	g	t	t	c	g	g	t	g	g	c	a	g	c	t	c	g	a	t	g	g	t	t	t	a	t	g	a	a	t	t	-	a	g	t	t	g	g	g	t	g	a	a
KP771890	9	g	t	a	a	c	a	g	t	a	c	a	t	c	t	a	t	g	a	c	a	t	c	a	g	a	g	a	c	a	t	g	g	g	t	t	c	g	g	t	g	g	c	a	g	c	t	c	g	a	t	g	g	t	t	t	a	t	g	a	a	t	t	-	a	g	t	t	g	g	g	t	g	a	a
JN704710	9	g	t	a	a	c	a	g	t	a	c	a	t	c	t	a	t	g	a	c	a	t	c	a	g	a	g	a	c	a	t	g	g	g	t	t	c	g	g	t	g	g	c	a	g	c	t	c	g	a	t	g	g	t	t	t	a	t	g	a	a	t	t	-	a	g	t	t	g	g	g	t	g	a	a
KY037798	9	g	t	a	a	c	a	g	t	a	c	a	t	c	t	a	t	g	a	c	a	t	c	a	g	a	a	a	c	a	t	g	g	g	t	t	t	g	g	t	g	g	c	a	g	c	t	c	g	a	t	g	g	t	t	t	a	t	g	a	a	t	t	-	a	g	t	t	g	g	g	t	g	a	a
MH709312	9	g	t	a	a	c	a	g	t	a	c	a	t	c	t	a	t	g	a	c	a	t	c	a	g	a	g	a	c	a	t	g	g	g	t	t	t	g	g	t	g	g	c	a	g	c	t	c	g	a	t	g	g	t	t	t	a	t	g	a	a	t	t	-	a	g	t	t	g	g	g	t	g	a	a
MH709352	9	g	t	a	a	c	a	g	t	a	c	a	t	c	t	a	t	g	a	c	a	t	c	a	g	a	g	a	c	a	t	g	g	g	t	t	c	g	g	t	g	g	c	a	g	c	t	c	g	a	t	g	g	t	t	t	a	t	g	a	a	t	t	-	a	g	t	t	g	g	g	t	g	a	a
AB097933	Vac	g	t	a	a	c	a	g	t	a	c	a	t	c	t	a	t	g	a	c	a	t	c	a	g	g	g	a	c	a	t	g	g	g	t	t	c	g	g	t	g	g	c	a	g	c	t	c	g	a	a	g	g	c	t	t	a	t	g	a	a	t	t	-	a	a	t	t	g	t	g	t	g	a	a
JN704698	Vac	g	t	a	a	c	a	g	t	a	c	a	t	c	t	a	t	g	a	c	a	t	c	a	g	g	g	a	c	a	t	g	g	g	t	t	c	g	g	t	g	g	c	a	g	c	t	c	g	a	a	g	g	c	t	t	a	t	g	a	a	t	t	-	a	a	t	t	g	t	g	t	g	a	a
JN704697	Vac	g	t	a	a	c	a	g	t	a	c	a	t	c	t	a	c	g	a	c	a	t	c	a	g	g	g	a	c	a	t	g	g	g	t	t	c	g	g	t	g	g	c	a	g	c	t	c	g	a	a	g	g	c	t	t	a	t	g	a	a	t	t	-	a	a	t	t	g	t	g	t	g	a	a
DQ008355	Vac	g	t	a	a	c	a	g	t	a	c	a	t	c	t	a	t	g	a	c	a	t	c	a	g	g	g	a	c	a	t	g	g	g	t	t	c	g	g	t	g	g	c	a	g	c	t	c	g	a	a	g	g	c	t	t	a	t	g	a	a	t	t	-	a	a	t	t	g	t	g	t	g	a	a
JF306641	Vac	g	t	a	a	c	a	g	t	a	c	a	t	c	t	a	t	g	a	c	a	t	c	a	g	g	g	a	c	a	t	g	g	g	t	t	c	g	g	t	g	g	c	a	g	c	t	c	g	a	a	g	g	c	t	t	a	t	g	a	a	t	t	-	a	a	t	t	g	t	g	t	g	a	a
AB097932	Vac	g	t	a	a	c	a	g	t	a	c	a	t	c	t	a	y	g	a	c	a	t	c	a	g	g	g	a	c	a	t	g	g	g	t	t	c	g	g	t	g	g	c	a	g	c	t	c	g	a	a	g	g	c	t	t	a	t	g	a	a	t	t	-	a	a	t	t	g	t	g	t	g	a	a
DQ008354	Vac	g	t	a	a	c	a	g	t	a	c	a	t	c	t	a	t	g	a	c	a	t	c	a	g	g	g	a	c	a	t	g	g	g	t	t	c	g	g	t	g	g	c	a	g	c	t	c	g	a	a	g	g	c	t	t	a	t	g	a	a	t	t	-	a	a	t	t	g	t	g	t	g	a	a
KU926312	Vac	g	t	a	a	c	a	g	t	a	c	a	t	c	t	a	t	g	a	c	a	t	c	a	g	g	g	a	c	a	t	g	g	g	t	t	c	g	g	t	g	g	c	a	g	c	t	c	g	a	a	g	g	c	t	t	a	t	g	a	a	t	t	-	a	a	t	t	g	t	g	t	g	a	a
KU926313	Vac	g	t	a	a	c	a	g	t	a	c	a	t	c	t	a	t	g	a	c	a	t	c	a	g	g	g	a	c	a	t	g	g	g	t	t	c	g	g	t	g	g	c	a	g	c	t	c	g	a	a	g	g	c	t	t	a	t	g	a	a	t	t	-	a	a	t	t	g	t	g	t	g	a	a
KU926314	Vac	g	t	a	a	c	a	g	t	a	c	a	t	c	t	a	t	g	a	c	a	t	c	a	g	g	g	a	c	a	t	g	g	g	t	t	c	g	g	t	g	g	c	a	g	c	t	c	g	a	a	g	g	c	t	t	a	t	g	a	a	t	t	-	a	a	t	t	g	t	g	t	g	a	a
KF558371	Vac	g	t	a	a	c	a	g	t	a	c	a	t	c	t	a	t	g	a	c	a	c	c	a	g	g	g	a	c	a	t	g	g	g	t	t	c	g	g	t	g	g	c	a	g	c	t	c	g	a	a	g	g	c	t	t	a	t	g	a	a	t	t	-	a	a	t	t	g	t	g	t	g	a	a
KF558372	Vac	g	t	a	a	c	a	g	t	a	c	a	t	c	t	a	t	g	a	c	a	t	c	a	g	g	g	a	c	a	t	g	g	g	t	t	c	g	g	t	g	g	c	a	g	c	t	c	g	a	a	g	g	c	t	t	a	t	g	a	a	t	t	-	a	a	t	t	g	t	g	t	g	a	a
KF558373	Vac	g	t	a	a	c	a	g	t	g	c	a	t	c	t	g	t	g	a	c	a	t	c	a	g	g	g	a	c	a	t	g	g	g	t	t	c	g	g	t	g	g	c	a	g	c	t	c	g	a	a	g	g	c	t	t	a	t	g	a	a	t	t	-	a	a	t	t	g	t	g	t	g	a	a
KF558374	Vac	g	t	a	a	c	a	g	t	a	c	a	t	c	t	a	t	g	a	c	a	t	c	a	g	g	g	a	c	a	t	g	g	g	t	t	c	g	g	t	g	g	c	a	g	c	t	c	g	a	a	g	g	c	t	t	a	t	g	a	a	t	t	-	a	a	t	t	g	t	g	t	g	a	a
KF558375	Vac	g	t	a	a	c	a	g	t	a	c	a	t	c	t	a	t	g	a	c	a	t	c	a	g	g	g	a	c	a	t	g	g	g	t	t	c	g	g	t	g	g	c	a	g	c	t	c	g	a	a	g	g	c	t	t	a	t	g	a	a	t	t	-	a	a	t	t	g	t	g	t	g	a	a
KF558376	Vac	g	t	a	a	c	a	g	t	a	c	a	t	c	t	a	c	g	a	c	a	t	c	a	g	g	g	a	c	a	t	g	g	g	t	t	c	g	g	t	g	g	c	a	g	c	t	c	g	a	a	g	g	c	t	t	a	t	g	a	a	t	t	-	a	a	t	t	g	t	g	t	g	a	a
KF558377	Vac	g	t	a	a	c	a	g	t	a	c	a	t	c	t	a	t	g	a	c	a	t	c	a	g	g	g	a	c	a	t	g	g	g	t	t	c	g	g	t	g	g	c	a	g	c	t	c	g	a	a	g	g	c	t	t	a	t	g	a	a	t	t	-	a	a	t	t	g	t	g	t	g	a	a
KF558378	Vac	g	t	a	a	c	a	g	t	a	c	a	t	c	t	a	t	g	a	c	a	t	c	a	g	g	g	a	c	a	t	g	g	g	t	t	c	g	g	t	g	g	c	a	g	c	t	c	g	a	a	g	g	c	t	t	a	t	g	a	a	t	t	-	a	a	t	t	g	t	g	t	g	a	a
KF558379	Vac	g	t	a	a	c	a	g	t	a	c	a	t	c	t	a	t	g	a	c	a	t	c	a	g	g	g	a	c	a	t	g	g	g	t	t	c	g	g	t	g	g	c	a	g	c	t	c	g	a	a	g	g	c	t	t	a	t	g	a	a	t	t	-	a	a	t	t	g	t	g	t	g	a	a
KF558380	Vac	g	t	a	a	c	a	g	t	a	c	a	t	c	t	a	t	g	a	c	a	t	c	a	g	g	g	a	c	a	t	g	g	g	t	t	c	g	g	t	g	g	c	a	g	c	t	c	g	a	a	g	g	c	t	t	a	t	g	a	a	t	t	-	a	a	t	t	g	t	g	t	g	a	a
KF558381	Vac	g	t	a	a	c	a	g	t	a	c	a	t	c	t	a	t	g	a	c	a	t	c	a	g	g	g	a	c	a	t	g	g	g	t	t	c	g	g	t	g	g	c	a	g	c	t	c	g	a	a	g	g	c	t	t	a	t	g	a	a	t	t	-	a	a	t	t	t	t	g	t	g	a	a
KF558382	Vac	g	t	a	a	c	a	g	t	a	c	a	t	c	t	a	t	g	a	c	a	t	c	a	g	g	g	a	c	a	t	g	g	g	t	t	c	g	g	t	g	g	c	a	g	c	t	c	g	a	a	g	g	c	t	t	a	t	g	a	a	t	t	-	a	a	t	t	g	t	g	t	g	a	a
KF558383	Vac	g	t	a	a	c	a	g	t	a	c	a	t	c	t	a	t	g	a	c	a	t	c	a	g	g	g	a	c	a	t	g	g	g	t	t	c	g	g	t	g	g	c	a	g	c	t	c	g	a	a	g	g	c	t	t	a	t	g	a	a	t	t	-	a	a	t	t	g	t	g	t	g	a	a
KF558384	Vac	g	t	a	a	c	a	g	t	a	c	a	t	c	t	a	t	g	a	c	a	t	c	a	g	g	g	a	c	a	t	g	g	g	t	t	c	g	g	t	g	g	c	a	g	c	t	c	g	a	a	g	g	c	t	t	a	t	g	a	a	t	t	-	a	a	t	t	g	t	g	t	g	a	a
KF558385	Vac	g	t	a	a	c	a	g	t	a	c	a	t	c	t	a	t	g	a	c	a	t	c	a	g	g	g	a	c	a	t	g	g	g	t	t	c	g	g	t	g	g	c	a	g	c	t	c	g	a	a	g	g	c	t	t	a	t	g	a	a	t	t	-	a	a	t	t	g	t	g	t	g	a	a
KF558386	Vac	g	t	a	a	c	a	g	t	a	c	a	t	c	t	a	t	g	a	c	a	t	c	a	g	g	g	a	c	a	t	g	g	g	t	t	c	g	g	t	g	g	c	a	g	c	t	c	g	a	a	g	g	c	t	t	a	t	g	a	a	t	t	-	a	a	t	t	g	t	g	t	g	a	a
KF558387	Vac	g	t	a	a	c	a	g	t	a	c	a	t	c	t	a	t	g	a	c	a	t	c	a	g	g	g	a	c	a	t	g	g	g	t	t	c	g	g	t	g	g	c	a	g	c	t	c	g	a	a	g	g	c	t	t	a	t	g	a	a	t	t	-	a	a	t	t	g	t	g	t	g	a	a
KF558388	Vac	g	t	a	a	c	a	g	t	a	c	a	t	c	t	a	t	g	a	c	a	t	c	a	g	g	g	a	c	a	t	g	g	g	t	t	c	g	g	t	g	g	c	a	g	c	t	c	g	a	a	g	g	c	t	t	a	t	g	a	a	t	t	-	a	a	t	t	g	t	g	t	g	a	a
KF558389	Vac	g	t	a	a	c	a	g	t	a	c	a	t	c	t	a	t	g	a	c	a	t	c	a	g	g	g	a	c	a	t	g	g	g	t	t	c	g	g	t	g	g	c	a	g	c	t	c	g	a	a	g	g	c	t	t	a	t	g	a	a	t	t	-	a	a	t	t	g	t	g	t	g	a	a
KF558390	Vac	g	t	a	a	c	a	g	t	a	c	a	t	c	t	a	t	g	a	c	a	t	c	a	g	g	g	a	c	a	t	g	g	g	t	t	c	g	g	t	g	g	c	a	g	c	t	c	g	a	a	g	g	c	t	t	a	t	g	a	a	t	t	-	a	a	t	t	g	t	g	t	g	a	a
KF558391	Vac	g	t	a	a	c	a	g	t	a	c	a	t	c	t	a	t	g	a	c	a	t	c	a	g	g	g	a	c	a	t	g	g	g	t	t	c	g	g	t	g	g	c	a	g	c	t	c	g	a	a	g	g	c	t	t	a	t	g	a	a	t	t	-	a	a	t	t	g	t	g	t	g	a	a
KF811485	Vac	g	t	a	a	c	a	g	t	a	c	a	t	c	t	a	t	g	a	c	a	t	c	a	g	g	g	a	c	a	t	g	g	g	t	t	c	g	g	t	g	g	c	a	g	c	t	c	g	a	a	g	g	c	t	t	a	t	g	a	a	t	t	-	a	a	t	t	g	t	g	t	g	a	a
KF853225	Vac	g	t	a	a	c	a	g	t	a	c	a	t	c	t	a	t	g	a	c	a	t	c	a	g	g	g	a	c	a	t	g	g	g	t	t	c	g	g	t	g	g	c	a	g	c	t	c	-	-	-	-	-	-	-	t	a	t	g	a	a	t	t	-	a	a	t	t	g	t	g	t	g	a	a
KF853226	Vac	g	t	a	a	c	a	g	t	a	c	a	t	c	t	a	t	g	a	c	a	t	c	a	g	g	g	a	c	a	t	g	g	g	t	t	c	g	g	t	g	g	c	a	g	c	t	c	-	-	-	-	-	-	-	t	a	t	g	a	a	t	t	-	a	a	t	t	g	t	g	t	g	a	a
KF853227	Vac	g	t	a	a	c	a	g	t	a	c	a	t	c	t	a	t	g	a	c	a	t	c	a	g	g	g	a	c	a	t	g	g	g	t	t	c	g	g	t	g	g	c	a	g	c	t	c	-	-	-	-	-	-	-	t	a	t	g	a	a	t	t	-	a	a	t	t	g	t	g	t	g	a	a
KF853228	Vac	g	t	a	a	c	a	g	t	a	c	a	t	c	t	a	t	g	a	c	a	t	c	a	g	g	g	a	c	a	t	g	g	g	t	t	c	g	g	t	g	g	c	a	g	c	t	c	-	-	-	-	-	-	-	t	a	t	g	a	a	t	t	-	a	a	t	t	g	t	g	t	g	a	a
KF853229	Vac	g	t	a	a	c	a	g	t	a	c	a	t	c	t	a	t	g	a	c	a	t	c	a	g	g	g	a	c	a	t	g	g	g	t	t	c	g	g	t	g	g	c	a	g	c	t	c	-	-	-	-	-	-	-	t	a	t	g	a	a	t	t	-	a	a	t	t	g	t	g	t	g	a	a
KF853230	Vac	g	t	a	a	c	a	g	t	a	c	a	t	c	t	a	t	g	a	c	a	t	c	a	g	g	g	a	c	a	t	g	g	g	t	t	c	g	g	t	g	g	c	a	g	c	t	c	-	-	-	-	-	-	-	t	a	t	g	a	a	t	t	-	a	a	t	t	g	t	g	t	g	a	a
KF853231	Vac	g	t	a	a	c	a	g	t	a	c	a	t	c	t	a	t	g	a	c	a	t	c	a	g	g	g	a	c	a	t	g	g	g	t	t	c	g	g	t	g	g	c	a	g	c	t	c	-	-	-	-	-	-	-	t	a	t	g	a	a	t	t	-	a	a	t	t	g	t	g	t	g	a	a
KF853232	Vac	g	t	a	a	c	a	g	t	a	c	a	t	c	t	a	t	g	a	c	a	t	c	a	g	g	g	a	c	a	t	g	g	g	t	t	c	g	g	t	g	g	c	a	g	c	t	c	-	-	-	-	-	-	-	t	a	t	g	a	a	t	t	-	a	a	t	t	g	t	g	t	g	a	a
KF853233	Vac	g	t	a	a	c	a	g	t	a	c	a	t	c	t	a	t	g	a	c	a	t	c	a	g	g	g	a	c	a	t	g	g	g	t	t	c	g	g	t	g	g	c	a	g	c	t	c	-	-	-	-	-	-	-	t	a	t	g	a	a	t	t	-	a	a	t	t	g	t	g	t	g	a	a
KF853234	Vac	g	t	a	a	c	a	g	t	a	c	a	t	c	t	a	t	g	a	c	a	t	c	a	g	g	g	a	c	a	t	g	g	g	t	t	c	g	g	t	g	g	c	a	g	c	t	c	-	-	-	-	-	-	-	t	a	t	g	a	a	t	t	-	a	a	t	t	g	t	g	t	g	a	a
KF853235	Vac	g	t	a	a	c	a	g	t	a	c	a	t	c	t	a	t	g	a	c	a	t	c	a	g	g	g	a	c	a	t	g	g	g	t	t	c	g	g	t	g	g	c	a	g	c	t	c	-	-	-	-	-	-	-	t	a	t	g	a	a	t	t	-	a	a	t	t	g	t	g	t	g	a	a
MF898328	Vac	g	t	a	a	c	a	g	t	a	c	a	t	c	t	a	y	g	a	c	a	t	c	a	g	g	g	a	c	a	t	g	g	g	t	t	c	g	g	t	g	g	c	a	g	c	t	c	g	a	a	g	g	c	t	t	a	t	g	a	a	t	t	-	a	a	t	t	g	t	g	t	g	a	a
JN704709	VIII	g	t	a	a	c	a	g	t	a	c	a	t	c	t	a	t	g	a	c	a	t	c	a	g	a	g	a	t	a	t	g	g	g	t	t	c	g	g	t	g	g	c	a	g	c	t	c	g	a	t	g	g	t	t	t	a	t	g	a	a	t	t	-	a	a	t	t	g	t	g	t	g	a	a

Supplementary Table 3: 2880 SN

GB-ID	Clade markers				1			4	SD14																									9			
	Position in Dumas	22038	22152	22192	22311	22343	22350	22362	22392	22443	22460	22484	22504	22631	22670	22684	22689	22704	22714	22769	22776	22794	22876	23287	23294	23332	23337	23378	23393	23429	23665	23669	23705	23717	23722	23756	23779
AY548170	1	a	c	g	g	a	g	c	t	t	t	a	a	a	g	a	g	c	t	t	t	a	c	g	a	c	t	g	c	a	a	t	c	t	c	c	g
AY548171	1	a	c	g	g	a	g	c	t	t	t	a	a	a	g	a	g	c	t	t	t	a	c	g	a	c	t	g	c	a	g	t	c	t	c	c	g
DQ479953	1	a	c	g	g	a	g	c	t	t	t	a	a	a	g	a	g	c	t	t	t	a	c	g	a	c	t	g	c	a	g	t	c	t	c	c	g
DQ479954	1	a	c	g	g	a	g	c	t	t	t	a	a	a	g	a	g	c	t	t	t	a	c	g	a	c	t	g	t	a	g	t	c	t	c	c	g
DQ479958	1	a	c	g	g	a	g	c	t	t	t	a	a	a	g	a	g	c	t	t	t	a	c	g	a	c	t	g	c	a	g	t	c	t	c	c	g
DQ479959	1	a	c	g	g	a	g	c	t	t	t	a	a	a	g	a	g	c	t	t	t	a	c	g	a	c	t	g	c	a	g	t	c	t	c	c	g
DQ479961	1	a	c	g	g	a	g	c	t	t	t	a	a	a	g	a	g	c	t	t	t	a	c	g	a	c	t	g	c	a	g	t	c	t	c	c	g
DQ479962	1	a	c	g	g	a	g	c	t	t	t	a	a	a	g	a	g	c	t	t	t	a	c	g	a	c	t	g	c	a	g	t	c	t	c	c	g
DQ479963	1	a	c	g	g	a	g	c	t	t	t	a	a	a	g	a	g	c	t	t	t	a	c	g	a	c	t	g	c	a	g	t	c	t	c	c	g
DQ674250	1	a	c	g	g	a	g	c	t	t	t	a	a	a	g	a	g	c	t	t	t	a	c	g	a	c	t	g	c	a	g	t	c	t	c	c	g
KX262864	1	a	c	g	g	a	g	c	t	t	t	a	a	a	g	a	g	c	t	t	t	a	c	g	a	c	t	g	c	a	g	t	c	t	c	c	g
KX262865	1	a	c	g	g	a	g	c	t	t	t	a	a	a	g	a	g	c	t	t	t	a	c	g	a	c	t	g	c	a	g	t	c	t	c	c	g
KX262866	1	a	c	g	g	a	g	c	t	t	t	a	a	a	g	a	g	c	t	t	t	a	c	g	a	c	t	g	c	a	g	t	c	t	c	c	g
EU154348	1	a	c	g	g	a	g	c	t	t	t	a	a	a	g	a	g	c	t	t	t	a	c	g	a	c	t	g	c	a	g	t	c	t	c	c	g
JN704690	1	a	c	g	g	a	g	c	t	t	t	a	a	a	g	a	g	c	t	t	t	a	c	g	a	c	t	g	c	a	g	t	c	t	c	c	g
JN704691	1	a	c	g	g	a	g	c	t	t	t	a	a	a	g	a	g	c	t	t	t	a	c	g	a	c	t	g	c	a	g	t	c	t	c	c	g
JN704692	1	a	c	g	g	a	g	c	t	t	t	a	a	a	g	a	g	c	t	t	t	a	c	g	a	c	t	g	c	a	g	t	c	t	c	c	g
JN704693	1	a	c	g	g	a	g	c	t	t	t	a	a	a	g	a	g	c	t	t	t	a	c	g	a	c	t	g	c	a	g	t	c	t	c	c	g
JN704694	1	a	c	g	g	a	g	c	t	t	t	a	a	a	g	a	g	c	t	t	t	a	c	g	a	c	c	g	c	a	a	t	c	t	c	c	g
JN704695	1	a	c	g	g	a	g	c	t	t	t	a	a	a	g	a	g	c	t	t	t	a	c	g	a	c	t	g	c	a	g	t	c	t	c	c	g
JN704696	1	a	c	g	g	a	g	c	t	t	t	a	a	a	g	a	g	c	t	t	t	a	c	g	a	c	t	g	c	a	g	t	c	t	c	c	g
NC001348	1	a	c	g	g	a	g	c	t	t	t	a	a	a	g	a	g	c	t	t	t	a	c	g	a	c	t	g	c	a	g	t	c	t	c	c	g
KP771892	1	a	c	g	g	a	g	c	t	t	t	a	a	a	g	a	g	c	t	t	t	a	c	g	a	c	t	g	c	a	g	t	c	t	c	c	g
KP771894	1	a	c	g	g	a	g	c	t	t	t	a	a	a	g	a	g	c	t	t	t	a	c	g	a	c	t	g	c	a	g	t	c	t	c	c	g
KP771900	1	a	c	g	g	a	g	c	t	t	t	a	a	a	g	a	g	c	t	t	t	a	c	g	a	c	t	g	c	a	g	t	c	t	c	c	g
KP771901	1	a	c	g	g	a	g	c	t	t	t	a	a	a	g	a	g	c	t	t	t	a	c	g	a	c	t	g	c	a	g	t	c	t	c	c	g
KP771902	1	a	c	g	g	a	g	c	t	t	t	a	a	a	g	a	g	c	t	t	t	a	c	g	a	c	t	g	c	a	g	t	c	t	c	c	g
KP771908	1	a	c	g	g	a	g	c	t	t	t	a	a	a	g	a	g	c	t	t	t	a	c	g	a	c	t	g	c	a	g	t	c	t	c	c	g
KP771912	1	a	c	g	g	a	g	c	t	t	t	a	a	a	g	a	g	c	t	t	t	a	c	g	a	c	t	g	c	a	g	t	c	t	c	c	g
KP771913	1	a	c	g	g	a	g	c	t	t	t	a	a	a	g	a	g	c	t	t	t	a	c	g	a	c	t	g	c	a	g	t	c	t	c	c	g
KP771917	1	a	c	a	g	a	g	c	t	t	t	a	a	a	g	a	g	c	t	t	t	a	c	g	a	c	t	g	c	a	g	t	c	t	c	c	g
KP771918	1	a	c	g	g	a	g	c	t	t	t	a	a	a	g	a	g	c	t	t	t	a	c	g	a	c	t	g	c	a	g	t	c	t	c	c	g
KP771920	1	a	c	g	g	a	g	c	t	t	t	a	a	a	g	a	a	c	t	t	t	a	c	g	a	c	t	g	c	a	g	t	c	t	c	c	g
MH709361	1	a	c	g	g	a	g	c	t	t	t	a	a	a	g	a	g	c	t	t	t	a	c	g	a	c	t	g	c	a	g	t	c	t	c	c	g
KU926318	2	a	c	g	a	a	g	c	t	t	t	a	g	a	g	a	g	c	t	c	t	g	c	g	g	c	t	g	c	a	g	t	c	t	c	c	g
KU926319	2	a	c	g	a	a	g	c	t	t	t	a	g	a	g	a	g	c	t	t	t	g	c	g	g	c	t	g	c	a	g	t	c	t	c	c	g
KU926320	2	a	c	g	a	a	g	c	t	t	t	a	g	a	g	g	g	c	t	t	t	g	c	g	g	c	c	g	c	a	g	t	c	t	c	c	g
KU926321	2	a	c	g	a	a	g	c	t	t	t	a	g	a	g	a	g	c	t	t	t	g	c	g	g	c	t	g	c	a	g	t	c	t	c	c	g
KU926322	2	a	c	g	a	a	g	c	t	t	t	a	g	a	g	a	g	c	t	t	t	g	c	g	g	c	t	g	c	a	g	t	c	t	c	c	g
KC847290	2	a	c	g	a	a	g	c	t	t	t	a	g	a	g	a	g	c	t	t	t	g	c	g	g	c	t	g	c	a	g	t	c	t	c	c	g
KJ767491	2	a	c	g	a	a	g	c	t	t	t	a	g	a	g	a	g	c	t	t	t	g	c	g	g	c	t	g	c	a	g	t	c	t	c	c	g
KJ767492	2	a	c	g	a	a	g	c	t	t	t	a	g	a	g	a	g	c	t	t	t	g	c	g	g	c	t	g	c	a	g	t	c	t	c	c	g
KJ808816	2	a	c	g	a	a	g	c	t	t	t	a	g	a	g	a	g	c	t	t	t	g	c	g	g	c	t	g	c	a	g	t	c	t	c	c	g
KP771925	2	a	c	g	a	a	g	c	t	t	t	a	g	a	g	a	g	c	t	t	t	g	c	g	g	c	t	g	c	a	g	t	c	t	c	c	g
KU926315	2	a	c	g	a	a	g	c	t	t	t	a	g	a	g	a	g	c	t	t	t	g	c	g	g	c	t	g	c	a	g	t	c	t	c	c	g
KU926316	2	a	c	g	a	a	g	c	t	t	t	a	g	a	g	a	g	c	t	t	t	g	c	g	g	c	t	g	c	a	g	t	c	t	c	c	g
KU926317	2	a	c	g	a	a	g	c	t	t	t	a	g	a	g	a	g	c	t	t	t	g	c	g	g	c	t	g	c	a	g	t	c	t	c	c	g
KY037796	2	a	c	g	a	a	g	c	t	t	t	a	g	a	g	a	g	c	t	t	t	g	c	g	g	c	t	g	c	a	g	t	c	t	c	c	g
MF004348	2	a	c	g	a	a	g	c	t	t	t	a	g	a	g	a	g	c	t	t	t	g	c	g	g	c	t	g	c	a	g	t	c	t	c	c	g
MH709309	2	a	c	g	a	a	g	c	t	t	t	a	g	a	g	a	g	c	t	t	t	g	c	g	g	c	t	g	c	a	g	t	c	t	c	c	g
MH709310	2	a	c	g	a	a	g	c	t	t	t	a	g	a	g	a	g	c	t	t	t	g	c	g	g	c	t	g	c	a	g	t	c	t	c	c	t
MH709311	2	a	c	g	a	a	g	c	t	t	t	a	g	a	g	a	g	c	t	t	t	g	c	g	g	c	t	g	c	a	g	t	c	t	c	c	g
MH709313	2	a	c	g	a	a	g	c	t	t	t	a	g	a	g	a	g	c	t	t	t	g	c	g	g	c	t	g	c	a	g	t	c	t	c	c	g
MH709314	2	a	c	g	a	a	g	c	t	t	t	a	g	a	g	a	g	c	t	t	t	g	c	g	g	c	t	g	c	a	g	t	c	t	c	c	g
MH709315	2	a	c	g	a	a	g	c	t	t	t	a	g	a	g	a	g	c	t	t	t	g	c	g	g	c	t	g	c	a	g	t	c	t	c	c	g
MH709319	2	a	c	g	a	a	g	c	t	t	t	a	g	a	g	a	g	c	t	t	t	g	c	g	g	c	t	g	c	a	g	t	c	t	c	c	g
MH709320	2	a	c	g	a	a	g	c	t	t	t	a	g	a	g	a	g	c	t	t	t	g	c	g	g	c	t	g	c	a	g	t	c	t	c	c	g
MH709329	2	a	c	g	a	c	g	c	t	t	t	a	g	a	g	a	g	c	t	t	t	g	c	g	g	c	t	g	c	a	g	t	c	t	c	c	g
MH709331	2	a	c	g	a	a	g	c	t	t	t	a	g	a	g	a	g	c	t	t	t	g	c	g	g	c	t	g	c	a	g	t	c	t	c	c	g
MH709333	2	a	c	g	a	a	g	c	t	t	t	a	g	a	g	a	g	c	t	t	t	g	c	g	g	c	t	g	c	a	g	t	c	t	c	c	g
MH709337	2	a	c	g	a	a	g	c	t	t	t	a	g	a	g	a	g	c	t	t	t	g	c	g	g	c	t	g	c	a	g	t	c	t	c	c	g
MH709339	2	a	c	g	a	a	g	c	t	t	t	a	g	a	g	a	g	c	t	t	t	g	c	g	g	c	t	g	c	a	g	t	c	t	c	c	g
MH709344	2	a	c	g	a	a	g	c	t	t	t	a	g	a	g	a	g	c	t	t	t	g	c	g	g	c	t	g	c	a	g	t	c	t	c	c	g
MH709348	2	a	c	g	a	a	g	c	t	t	t	a	g	a	g	a	g	c	t	t	t	g	c	g	g	c	t	g	c	a	g	t	c	t	c	c	g
MH709355	2	a	t	g	a	a	g	c	t	t	t	a	g	a	g	a	g	c	t	t	t	g	c	g	g	c	t	g	c	a	g	t	c	t	c	c	g
MH709359	2	a	c	g	a	a	g	c	t	t	t	a	g	a	g	a	g	c	t	t	t	g	c	a	g	c	t	g	c	a	g	t	c	t	c	c	g
MH709363	2	a	c	g	a	a	g	c	t	t	t	a	g	a	g	a	g	c	t	t	t	g	c	g	g	c	t	g	c	a	g	t	c	t	c	c	g
MH709366	2	a	c	g	a	a	g	c	t	t	t	a	g	a	g	a	g	c	t	t	t	g	c	a	g	c	t	g	c	a	g	t	c	t	c	c	g
MH709376	2	a	c	g	a	a	g	c	t	t	t	a	g	a	g	a	g	c	t	t	t	g	c	g	g	c	t	g	c	a	g	t	c	t	c	c	g
MH709377	2	a	c	g	a	a	g	c	t	t	t	a	g	a	a	a	g	c	t	t	t	g	c	g	g	c	t	g	c	a	g	t	c	t	c	c	g
**MW316406**	2	a	c	g	a	a	g	c	g	t	t	a	g	a	g	a	g	c	t	t	t	g	c	g	g	c	t	g	c	a	g	t	c	t	c	c	g
AJ871403	3	g	c	g	a	a	g	c	t	t	t	a	a	a	g	a	g	c	t	t	t	a	c	g	g	c	t	a	c	g	g	t	c	t	c	c	g
DQ479955	3	a	c	g	a	a	g	c	t	t	t	a	a	a	g	a	g	c	t	t	t	a	c	g	g	c	t	g	c	g	g	t	c	t	c	c	g
DQ479956	3	a	c	g	a	a	g	c	t	t	t	a	a	a	g	a	g	c	t	t	t	a	a	g	g	c	t	g	c	g	g	t	c	t	c	c	g
DQ479957	3	a	c	g	a	a	g	c	t	t	t	a	a	a	g	a	g	c	t	t	t	a	c	g	g	c	t	g	c	g	g	t	c	t	c	c	g
JN704699	3	a	c	g	a	a	g	c	t	t	t	a	a	a	g	a	g	c	t	t	t	a	c	g	g	c	t	g	c	g	g	t	c	t	c	c	g
JN704700	3	a	c	g	a	a	g	c	t	t	t	a	a	a	g	a	g	c	t	t	t	a	c	g	a	c	t	g	c	g	g	t	c	t	c	c	g
JN704701	3	a	c	g	a	a	g	c	t	t	t	a	a	a	g	a	g	c	t	t	t	a	c	g	g	c	t	g	c	g	g	t	c	t	c	c	g
JN704702	3	a	c	g	a	a	g	c	t	t	t	a	a	a	g	a	g	c	t	t	t	a	c	g	g	c	t	g	c	g	g	c	c	t	c	c	g
JN704703	3	a	c	g	a	a	g	c	t	t	t	a	a	a	g	a	g	c	t	t	t	a	c	g	g	c	t	g	c	g	g	t	c	t	c	c	g
KP771891	3	a	c	g	a	a	g	c	t	t	t	a	a	a	g	a	g	c	t	t	t	a	c	g	g	c	t	g	c	g	g	t	c	t	c	c	g
KP771893	3	a	c	g	a	a	g	c	t	t	t	a	a	a	g	a	g	c	t	t	t	a	c	g	g	c	t	g	c	g	g	t	c	t	c	c	g
KP771895	3	a	c	g	a	a	g	c	t	t	t	a	a	a	g	a	g	c	t	t	t	a	c	g	g	c	t	g	c	g	g	t	c	t	c	c	g
KP771896	3	a	c	g	a	a	g	c	t	t	t	a	a	a	g	a	g	c	t	t	t	a	c	g	g	c	t	g	c	g	g	t	c	t	c	c	g
KP771897	3	a	c	g	a	a	g	c	t	t	t	a	a	a	g	a	g	c	t	t	t	a	c	g	g	c	t	g	c	g	a	t	c	t	c	c	g
KP771899	3	a	c	g	a	a	g	c	t	t	t	a	a	a	g	a	g	c	t	t	t	a	c	g	g	c	t	g	c	g	g	t	c	t	c	c	g
KP771903	3	a	c	g	a	a	g	c	t	t	t	a	a	a	g	a	g	c	t	t	t	a	c	g	g	c	t	g	c	g	g	t	c	t	c	c	g
KP771907	3	a	c	g	a	a	g	c	t	g	t	a	a	a	g	a	g	c	t	t	t	a	c	g	g	c	t	g	c	g	g	t	c	t	c	c	g
KP771910	3	a	c	g	a	a	g	c	t	t	t	a	a	a	g	a	g	c	t	t	t	a	c	g	g	c	t	g	c	g	g	t	c	t	c	c	g
KP771915	3	a	c	g	a	a	g	c	t	t	t	a	a	a	g	a	g	t	t	t	t	a	c	g	g	c	t	g	c	g	g	t	c	t	c	c	g
KP771916	3	a	c	g	a	a	g	c	t	t	t	a	a	a	g	a	g	c	t	t	t	a	a	g	g	a	t	g	c	g	g	t	c	t	c	c	g
KP771921	3	a	c	g	a	a	g	c	t	t	t	a	a	a	g	a	g	c	t	t	t	a	c	g	g	c	t	g	c	g	g	t	c	t	c	c	g
KP771923	3	a	c	g	a	a	g	c	t	t	t	a	a	a	g	a	g	c	t	t	t	a	c	g	g	c	t	g	c	g	g	t	c	t	c	c	g
KP771889	3	a	c	g	a	a	g	c	t	t	t	a	a	a	g	a	g	c	t	t	t	a	c	g	a	c	t	g	c	a	g	t	c	t	c	c	g
KU926311	3	a	c	g	a	a	g	c	t	t	t	a	a	a	g	a	g	c	t	t	t	a	a	g	g	c	t	g	c	g	g	t	c	t	c	c	g
JQ911599	3	a	c	g	a	a	g	c	t	t	t	a	a	a	g	a	g	c	t	t	t	a	c	g	g	c	t	g	c	g	g	t	c	t	c	c	g
MH709317	3	a	c	g	a	a	g	c	t	t	t	a	a	a	g	a	g	c	t	t	t	a	c	g	g	c	t	g	c	g	g	t	c	t	c	c	g
MH709346	3	a	c	g	a	a	g	c	t	t	t	a	a	a	g	a	g	c	t	t	t	a	c	g	g	c	t	g	c	g	g	t	c	t	c	c	g
MH709375	3	a	c	g	a	a	g	c	t	t	t	a	a	a	g	a	g	c	t	t	t	a	c	g	g	c	t	g	c	g	g	t	c	t	c	c	g
DQ452050	4	a	c	g	a	a	g	g	t	t	t	a	a	a	g	a	g	c	t	t	t	a	c	g	a	c	t	g	c	a	g	t	c	t	c	c	g
DQ479960	4	a	c	g	a	a	g	g	t	t	t	a	a	a	g	a	g	c	t	t	t	a	c	g	a	c	t	g	c	a	g	t	c	t	c	c	g
KP771898	4	a	c	g	a	a	g	g	t	t	t	a	a	a	g	a	g	c	t	t	t	a	c	g	a	c	t	g	c	a	g	t	c	t	c	c	g
MH709316	4	a	c	g	a	a	g	g	t	t	t	a	a	a	g	a	g	c	t	t	t	a	c	g	a	c	t	g	c	a	g	t	a	t	t	c	g
MH709318	4	a	c	g	a	a	g	g	t	t	t	a	a	a	g	a	g	c	t	t	t	a	c	g	a	c	t	g	c	a	g	t	c	t	c	c	g
MH709322	4	a	c	g	a	a	g	g	t	t	t	a	a	a	g	a	g	c	t	t	t	a	c	g	a	c	t	g	c	a	g	t	c	t	c	c	g
MH709323	4	a	c	g	a	a	g	g	t	t	t	a	a	a	g	a	g	c	t	t	t	a	c	g	a	c	t	g	c	a	g	t	c	t	c	c	g
MH709326	4	a	c	g	a	a	g	g	t	t	t	a	a	a	g	a	g	c	t	t	t	a	c	g	a	c	t	g	c	a	g	t	c	t	c	c	g
MH709341	4	a	c	g	a	a	g	g	t	t	t	a	a	a	g	a	g	c	t	t	t	a	c	g	a	c	t	g	c	a	g	t	c	t	c	c	g
MH709357	4	a	c	g	a	a	g	g	t	t	t	a	a	a	g	a	g	c	t	t	t	a	c	g	a	c	t	g	c	a	g	t	c	t	c	c	g
MH709364	4	a	c	g	a	a	g	g	t	t	t	a	a	a	g	a	g	c	t	t	t	a	c	g	a	c	t	g	c	a	g	t	c	t	c	c	g
MH709370	4	a	c	g	a	a	g	g	t	t	t	a	a	a	g	a	g	c	t	t	t	a	c	g	a	c	t	g	c	a	g	t	c	t	c	c	g
MH709371	4	a	c	g	a	a	g	g	t	t	t	a	a	a	g	a	g	c	t	t	t	a	c	g	a	c	t	g	c	a	g	t	c	t	c	c	g
MH709372	4	a	c	g	a	a	g	g	t	t	t	a	a	a	g	a	g	c	t	t	t	a	c	g	a	c	t	g	c	a	g	t	c	t	c	c	g
DQ457052	5	a	c	g	a	a	g	c	t	t	t	a	a	a	g	a	g	c	t	t	t	a	c	g	g	c	t	g	c	g	g	t	c	t	c	c	g
JN704704	5	a	c	g	a	a	g	c	t	t	t	a	a	a	g	a	g	c	t	t	t	a	c	g	g	c	t	g	c	g	g	t	c	t	c	c	g
JN704705	5	a	c	g	a	a	g	c	t	t	t	a	a	a	g	a	g	c	t	t	t	a	c	g	g	c	t	g	c	g	g	t	c	t	c	c	g
JN704706	5	a	c	g	a	a	t	c	t	t	t	a	a	a	g	a	g	c	t	t	t	a	c	g	g	c	t	g	c	g	g	t	c	t	c	c	g
JN704707	5	a	c	g	a	a	g	c	t	t	t	a	a	a	g	a	g	c	t	t	t	a	c	g	g	c	t	g	c	g	g	t	c	t	c	c	g
JN704708	5	a	c	g	a	a	g	c	t	t	t	a	a	a	g	a	g	c	t	t	t	a	c	g	g	c	t	g	c	g	g	t	c	t	c	c	g
KP771904	5	a	c	g	a	a	g	c	t	t	t	a	a	g	g	a	g	c	t	t	t	a	c	g	g	c	t	g	c	g	g	t	c	t	c	c	g
KP771905	5	a	c	g	a	a	g	c	t	t	t	a	a	a	g	a	g	c	t	t	t	a	c	g	g	c	t	g	c	g	g	t	c	t	c	c	g
KP771906	5	a	c	g	a	a	g	c	t	t	t	a	a	a	g	a	g	c	t	t	t	a	c	g	g	c	t	g	c	g	g	t	c	t	c	c	g
KP771919	5	a	c	g	a	a	g	c	t	t	t	a	a	a	g	a	g	c	t	t	t	a	c	g	g	c	t	g	c	g	g	t	c	t	c	c	g
KP771922	5	a	c	g	a	a	g	c	t	t	t	a	a	a	g	a	g	c	t	t	t	a	c	g	g	c	t	g	c	g	g	t	c	t	c	c	g
KP771924	5	a	c	g	a	a	g	c	t	t	t	a	a	a	g	a	g	c	t	t	t	a	c	g	g	c	t	g	c	g	g	t	c	t	c	c	g
KM355696	5	a	c	g	a	a	g	c	t	t	t	a	a	a	g	a	g	c	t	t	t	a	c	g	g	c	t	g	c	g	g	t	c	t	c	c	g
KM355697	5	a	c	g	a	a	g	c	t	t	t	a	a	a	g	a	g	c	t	t	t	a	c	g	g	c	t	g	c	g	g	t	c	t	c	c	g
KM355698	5	a	c	g	a	a	g	c	t	t	t	a	a	a	g	a	g	c	t	t	t	a	c	g	g	c	t	g	c	g	g	t	c	t	c	c	g
KM355699	5	a	c	g	a	a	g	c	t	t	t	a	a	a	g	a	g	c	t	t	t	a	c	g	g	c	t	g	c	g	g	t	c	t	c	c	g
KM355701	5	a	c	g	a	a	g	c	t	t	t	a	a	a	g	a	g	c	t	t	t	a	c	g	g	c	t	g	c	g	g	t	c	t	c	c	g
KM355702	5	a	c	g	a	a	g	c	t	t	t	a	a	a	g	a	g	c	t	t	t	a	c	g	g	c	t	g	c	g	g	t	c	t	c	c	g
KM355703	5	a	c	g	a	a	g	c	t	t	t	a	a	a	g	a	g	c	t	t	t	a	c	g	g	c	t	g	c	g	g	t	c	t	c	c	g
KM355704	5	a	c	g	a	a	g	c	t	t	t	a	a	a	g	a	g	c	t	t	t	a	c	g	g	c	t	g	c	g	g	t	c	t	c	c	g
KM355705	5	a	c	g	a	a	g	c	t	t	t	a	a	a	g	a	g	c	t	t	t	a	c	g	g	c	t	g	c	g	g	t	c	t	c	c	g
KM355706	5	a	c	g	a	a	g	c	t	t	t	a	a	a	g	a	g	c	t	t	t	a	c	g	g	c	t	g	c	g	g	t	c	t	c	c	g
KM355707	5	a	c	g	a	a	g	c	t	t	t	a	a	a	g	a	g	c	t	t	t	a	c	g	g	c	t	g	c	g	g	t	c	t	c	c	g
KM355708	5	a	c	g	a	a	g	c	t	t	t	a	a	a	g	a	g	c	t	t	t	a	c	g	g	c	t	g	c	g	g	t	c	t	c	c	g
KM355709	5	a	c	g	a	a	g	c	t	t	t	a	a	a	g	a	g	c	t	t	t	a	c	g	g	c	t	g	c	g	g	t	c	t	c	c	g
KM355711	5	a	c	g	a	a	g	c	t	t	t	a	a	a	g	a	g	c	t	t	t	a	c	g	g	c	t	g	c	g	g	t	c	t	c	c	g
KM355712	5	a	c	g	a	a	g	c	t	t	t	a	a	a	g	a	g	c	t	t	t	a	c	g	g	c	t	g	c	g	g	t	c	t	c	c	g
[illegible]	5	[illegible]	[illegible]	[illegible]	[illegible]	[illegible]	[illegible]	[illegible]	[illegible]	[illegible]	[illegible]	[illegible]	[illegible]	[illegible]	[illegible]	[illegible]	[illegible]	[illegible]	[illegible]	[illegible]	[illegible]	[illegible]	[illegible]	[illegible]	[illegible]	[illegible]	[illegible]	[illegible]	[illegible]	[illegible]	[illegible]	[illegible]	[illegible]	[illegible]	[illegible]	[illegible]	[illegible]

GB-ID				6																																		
	23784	23898	23904	23959	23960	24071	24085	24092	24095	24135	24328	24533	24567	24578	24628	24646	24651	24654	24656	24700	25038	25067	25089	25105	25106	25267	25288	25309	25471	25500	25558	25561	25630	25675	25783	25816	25851	25894
AY548170	c	a	c	c	a	c	g	t	t	g	a	a	g	a	c	t	a	c	a	g	a	g	c	t	t	a	a	c	t	c	g	c	t	g	g	t	t	c
AY548171	c	a	c	c	a	c	g	t	t	g	a	a	g	a	c	t	a	c	a	g	a	g	c	t	t	a	a	c	t	c	g	c	t	g	g	t	t	c
DQ479953	c	a	c	c	a	c	g	t	t	g	a	a	g	a	c	t	a	c	a	g	a	g	c	t	t	a	a	c	t	c	g	c	t	g	g	t	t	c
DQ479954	c	a	c	c	a	c	g	t	t	g	a	a	g	a	c	t	a	c	a	g	a	g	c	t	t	a	a	c	t	c	g	c	t	g	g	t	t	c
DQ479958	c	a	c	c	a	c	g	t	t	g	a	a	g	a	c	t	a	c	a	g	a	g	c	t	t	a	a	c	t	c	g	c	t	g	g	t	t	c
DQ479959	c	a	c	c	a	c	g	t	t	g	a	a	g	a	c	t	a	c	a	g	a	g	c	t	t	a	a	c	t	c	g	c	t	g	g	t	t	c
DQ479961	c	a	c	c	a	c	g	t	t	g	a	a	g	a	c	t	a	c	a	g	a	g	c	t	t	a	a	c	t	c	g	c	t	g	g	t	t	c
DQ479962	c	a	c	c	a	c	g	t	t	g	a	a	g	a	c	t	a	c	a	g	a	g	c	t	t	a	a	c	t	c	g	c	t	g	g	t	t	c
DQ479963	c	a	c	c	a	c	g	t	t	g	a	a	g	a	c	t	a	c	a	g	a	g	c	t	t	a	a	c	t	c	g	c	t	g	g	t	t	c
DQ674250	c	a	c	c	a	c	g	t	t	g	a	a	g	a	c	t	a	c	a	g	a	g	c	t	t	a	a	c	t	c	g	c	t	g	g	t	t	c
KX262864	c	a	c	c	a	c	g	t	t	g	a	a	g	a	c	t	a	c	a	g	a	g	c	t	t	a	a	c	t	c	g	c	t	g	a	t	t	c
KX262865	c	a	c	c	a	c	g	t	t	g	a	a	g	a	c	t	a	c	a	g	a	g	c	t	t	a	a	c	t	c	g	c	t	g	g	t	t	c
KX262866	c	a	c	c	a	c	g	t	t	g	a	a	g	a	c	t	a	c	a	g	a	g	c	t	t	a	a	c	t	c	g	c	t	g	g	t	t	c
EU154348	c	a	c	c	a	c	g	t	t	g	a	a	g	a	c	t	a	c	a	g	a	g	c	t	t	a	a	c	t	c	g	c	t	g	g	t	t	c
JN704690	c	a	c	c	a	c	g	t	t	g	a	a	g	a	c	t	a	c	a	g	a	g	c	t	t	a	a	c	t	c	g	c	t	g	g	t	t	c
JN704691	c	a	c	c	a	c	g	t	t	g	a	a	g	a	c	t	a	c	a	g	a	g	c	t	t	a	a	c	t	c	g	c	t	g	g	t	t	c
JN704692	c	a	c	c	a	c	g	t	t	g	a	a	g	a	c	t	a	c	a	g	a	g	c	t	t	a	a	c	t	c	g	c	t	g	g	t	t	c
JN704693	c	a	c	c	a	c	g	t	t	g	a	a	g	a	c	t	a	c	a	g	a	g	c	t	t	a	a	c	t	c	g	c	t	g	g	t	t	c
JN704694	c	a	c	c	a	c	g	t	t	g	a	a	g	a	c	t	a	c	a	g	a	g	c	t	t	a	a	c	t	c	g	c	t	g	g	t	t	c
JN704695	c	a	c	c	a	c	g	t	t	g	a	a	g	a	c	t	a	c	a	g	a	g	c	t	t	a	a	c	t	c	g	c	t	g	g	t	t	c
JN704696	c	a	c	c	a	c	g	t	t	g	a	a	g	a	c	t	a	c	a	g	a	g	c	t	t	a	a	c	t	c	g	c	t	g	g	t	t	c
NC001348	c	a	c	c	a	c	g	t	t	g	a	a	g	a	c	t	a	c	a	g	a	g	c	t	t	a	a	c	t	c	g	c	t	g	g	t	t	c
KP771892	c	a	c	c	a	c	g	t	t	g	a	a	g	a	c	t	a	c	a	g	a	g	c	t	t	a	a	c	t	c	g	c	t	g	g	t	t	c
KP771894	c	a	c	c	a	c	g	t	t	g	a	a	g	a	c	t	a	c	a	g	a	g	c	t	t	a	a	c	t	c	g	c	t	g	g	t	t	c
KP771900	c	a	c	c	a	c	g	t	t	g	a	a	g	a	c	t	a	c	a	g	a	g	c	t	t	a	a	c	t	c	g	c	t	g	g	t	t	c
KP771901	c	a	c	c	a	c	g	t	t	g	a	a	g	a	c	t	a	c	a	g	a	g	c	t	t	a	a	c	t	c	g	c	t	g	g	t	t	c
KP771902	c	a	c	c	a	c	g	t	t	g	a	a	g	a	c	t	a	c	a	g	a	g	c	t	t	a	a	c	t	c	g	c	t	g	g	t	t	c
KP771908	c	a	c	c	a	c	g	t	t	g	a	a	g	a	c	t	a	c	a	g	a	g	c	t	t	a	a	c	t	c	g	c	t	g	g	t	t	c
KP771912	c	a	c	c	a	c	g	t	t	g	a	a	g	a	c	t	a	c	a	g	a	g	c	t	t	a	a	c	t	c	g	c	t	g	a	t	t	c
KP771913	c	a	c	c	a	c	g	t	t	g	a	a	g	a	c	t	a	c	a	g	a	g	c	t	t	a	a	c	t	c	g	c	t	g	a	t	t	c
KP771917	c	a	c	c	a	c	g	t	t	g	a	a	g	a	c	t	a	c	a	g	a	g	c	t	t	a	a	c	t	c	g	c	t	g	g	t	t	c
KP771918	c	a	c	c	a	c	g	t	t	g	a	a	g	a	c	t	a	c	a	g	a	g	c	t	t	a	a	c	t	c	g	c	t	g	g	t	t	c
KP771920	c	a	c	c	a	c	g	t	t	g	a	a	g	a	c	t	a	c	a	g	a	g	c	t	t	a	a	c	t	c	g	c	t	g	g	t	t	c
MH709361	c	a	c	c	a	c	g	t	t	g	a	a	g	a	c	t	a	c	a	g	a	g	c	t	t	a	a	c	t	c	g	c	t	g	g	t	t	c
KU926318	c	a	c	c	a	c	g	t	c	g	a	a	g	g	c	c	a	t	a	g	g	a	c	t	c	g	a	c	t	c	g	c	t	g	g	t	t	c
KU926319	c	a	c	c	a	c	g	t	t	g	a	a	g	g	c	t	a	t	a	g	a	a	c	t	t	a	a	c	t	c	g	c	t	g	g	c	t	c
KU926320	c	a	c	c	a	c	g	t	t	g	a	a	g	g	c	c	a	t	a	g	a	a	c	t	t	a	g	c	t	c	g	c	t	g	g	c	t	c
KU926321	c	a	c	c	a	c	g	t	t	g	a	a	g	g	c	t	a	t	a	g	a	a	c	t	t	a	a	c	t	c	g	c	t	g	g	t	t	c
KU926322	c	a	c	c	a	c	g	t	t	g	a	a	g	g	c	t	a	t	a	g	a	a	c	t	t	a	a	c	t	c	g	c	t	g	g	t	t	c
KC847290	c	a	c	c	a	c	g	t	t	g	a	a	g	g	c	t	a	t	a	g	a	a	c	t	t	a	a	c	t	c	g	c	t	g	g	t	t	c
KJ767491	c	a	c	c	a	c	g	t	t	g	a	a	g	g	c	t	a	t	a	g	a	a	c	t	t	a	a	c	t	c	g	c	t	g	g	t	t	c
KJ767492	c	a	c	c	a	c	g	t	t	g	a	a	g	g	c	t	a	t	a	g	a	a	c	t	t	a	a	c	t	c	g	c	t	g	g	t	t	c
KJ808816	c	a	c	c	a	c	g	t	t	g	a	a	g	g	c	t	a	t	a	g	a	a	c	t	t	a	a	c	t	c	g	c	t	g	g	t	t	c
KP771925	c	a	c	c	a	c	g	t	t	g	a	a	g	g	c	t	a	t	a	g	a	a	c	t	t	a	a	c	t	c	g	a	t	g	g	t	t	c
KU926315	c	a	c	c	a	c	g	t	t	g	a	a	g	g	c	t	a	t	a	g	a	a	c	t	t	a	a	c	t	c	g	c	t	g	g	t	t	c
KU926316	c	a	c	c	a	c	g	t	t	g	a	a	g	g	c	t	a	t	a	g	a	a	c	t	t	a	a	c	t	c	g	c	t	g	g	t	t	c
KU926317	c	a	c	c	a	c	g	t	t	g	a	a	a	g	c	t	a	t	a	g	a	a	c	t	t	a	a	c	t	c	g	c	t	g	g	t	t	c
KY037796	c	a	c	c	a	c	g	t	t	g	a	a	g	g	c	t	a	t	a	g	a	a	c	t	t	a	a	c	t	c	g	c	t	g	g	t	t	c
MF004348	c	a	c	c	a	c	g	t	t	g	a	a	g	g	c	t	a	t	a	g	a	a	c	t	t	a	a	c	t	c	g	c	t	g	g	t	t	c
MH709309	c	a	c	c	a	c	a	t	t	g	a	a	g	g	c	t	a	t	a	g	a	a	c	t	t	a	a	c	t	c	g	c	t	g	g	t	t	c
MH709310	c	a	c	c	a	c	g	t	t	g	a	a	g	g	c	t	a	t	a	g	a	a	c	t	t	a	a	c	t	c	g	c	t	g	g	t	t	c
MH709311	c	a	c	c	a	c	g	t	t	g	a	a	a	g	c	t	a	t	a	g	a	a	c	t	t	a	a	c	t	c	g	c	t	g	g	t	t	c
MH709313	c	a	c	c	a	c	g	t	t	g	a	a	a	g	c	t	a	t	a	g	a	a	c	t	t	a	a	c	t	c	g	c	t	g	g	t	t	c
MH709314	c	a	c	c	a	c	g	t	t	g	a	a	g	g	c	t	a	t	a	g	a	a	c	t	t	a	a	c	c	c	g	c	t	g	g	t	t	c
MH709315	c	a	c	c	a	c	g	t	t	g	a	a	g	g	c	t	a	t	a	g	a	a	c	t	t	a	a	c	c	c	g	c	t	g	g	t	t	c
MH709319	c	a	c	c	a	c	g	t	t	g	a	a	g	g	c	t	a	t	a	g	a	a	c	t	t	a	a	c	c	c	g	c	t	g	g	t	t	c
MH709320	c	a	c	c	a	c	g	t	t	g	a	a	g	g	c	t	a	t	a	g	a	a	c	t	t	a	a	c	t	c	g	c	t	g	g	t	t	c
MH709329	c	a	c	c	a	c	g	t	t	g	a	a	g	g	c	t	a	t	a	g	a	a	c	t	t	a	a	c	c	c	g	c	t	g	g	t	t	c
MH709331	c	a	c	c	a	c	g	t	t	g	a	a	a	g	c	t	a	t	a	g	a	a	c	t	t	a	a	c	t	c	g	c	t	g	g	t	t	c
MH709333	c	a	c	c	a	c	g	t	t	g	a	a	g	g	c	t	a	t	a	g	a	a	c	t	t	a	a	c	c	c	g	c	t	g	g	t	t	c
MH709337	c	a	c	c	a	c	g	t	t	g	a	a	g	g	c	t	a	t	a	g	a	a	c	t	t	a	a	c	t	c	g	c	t	g	g	t	t	c
MH709339	c	a	c	c	a	c	g	t	t	g	a	a	g	g	c	t	a	t	a	g	a	a	c	t	t	a	a	c	t	c	g	c	t	g	g	t	t	c
MH709344	c	a	c	c	a	c	g	t	t	g	a	a	a	g	c	t	a	t	a	g	a	a	c	t	t	a	a	c	t	c	g	c	t	g	g	t	t	c
MH709348	c	a	c	c	a	c	g	t	t	g	a	a	g	g	c	t	a	t	a	g	a	a	c	t	t	a	a	c	t	c	g	c	t	g	g	t	t	c
MH709355	c	a	c	c	a	c	g	t	t	g	a	a	g	g	c	t	a	t	a	g	a	a	c	t	t	a	a	c	t	c	g	c	t	g	g	t	t	c
MH709359	c	a	c	c	a	c	g	t	t	g	a	a	a	g	c	t	a	t	a	g	a	a	c	t	t	a	a	c	t	c	g	c	t	g	g	t	t	c
MH709363	c	a	c	c	a	c	g	t	t	g	a	a	a	g	c	t	a	t	a	g	a	a	c	t	t	a	a	c	t	c	g	c	t	g	g	t	t	c
MH709366	c	a	c	c	a	c	g	t	t	g	a	a	a	g	c	t	a	t	a	g	a	a	c	t	t	a	a	c	t	c	g	c	t	g	g	t	t	c
MH709376	c	a	c	c	a	c	g	t	t	g	a	a	g	g	c	t	a	t	a	g	a	a	c	t	t	a	a	c	t	c	g	c	t	g	g	t	t	c
MH709377	c	a	c	c	a	c	g	t	t	g	a	a	g	g	c	t	a	t	a	g	a	a	c	t	t	a	a	c	t	c	g	c	t	g	g	t	t	c
**MW316406**	c	a	c	c	a	c	g	t	t	g	a	a	a	g	c	t	a	t	a	g	a	a	c	t	t	a	a	c	t	c	g	c	t	g	g	t	t	c
AJ871403	c	a	c	c	a	c	g	c	t	g	a	a	g	g	c	t	a	t	a	g	a	a	c	t	t	a	a	c	t	c	g	c	t	g	g	t	t	c
DQ479955	c	a	c	c	a	c	g	c	t	g	a	a	g	g	c	t	a	t	a	g	a	a	c	t	t	a	a	c	t	c	g	c	t	g	g	t	t	c
DQ479956	c	a	c	c	a	c	g	c	t	g	a	a	g	g	c	t	a	t	a	g	a	a	c	t	t	a	a	c	t	c	g	c	t	g	g	t	t	c
DQ479957	c	a	c	c	a	c	g	c	t	g	c	a	g	g	c	t	a	t	a	g	a	a	c	t	t	a	a	c	t	c	g	c	t	g	g	t	t	c
JN704699	c	a	c	c	a	c	g	c	t	g	a	a	g	g	c	t	a	t	a	g	a	a	c	t	t	a	a	c	t	c	g	c	t	g	g	t	t	c
JN704700	c	a	c	c	g	c	g	c	t	g	a	a	g	g	c	t	a	t	a	g	a	a	c	t	t	a	a	c	t	c	g	c	t	g	g	t	t	c
JN704701	c	a	c	c	a	c	g	c	t	g	c	a	g	g	c	t	a	t	a	g	a	a	c	t	t	a	a	c	t	c	g	c	t	g	g	t	t	c
JN704702	c	a	c	c	a	c	g	c	t	g	a	a	g	g	c	t	a	t	a	g	a	a	c	t	t	a	a	c	t	c	g	c	t	g	g	t	t	t
JN704703	c	a	c	c	a	c	g	c	t	g	a	a	g	g	c	t	a	t	a	g	a	a	c	t	t	a	a	c	t	c	g	c	t	g	g	t	t	t
KP771891	c	a	c	c	a	c	g	c	t	g	c	a	g	g	c	t	a	t	a	g	a	a	c	t	t	a	a	c	t	c	g	c	t	g	g	t	t	c
KP771893	c	a	c	c	a	c	g	c	t	g	a	a	g	g	c	t	a	t	a	g	a	a	c	t	t	a	a	c	t	c	g	c	t	g	g	t	t	c
KP771895	c	a	c	c	a	c	g	c	t	g	a	a	g	g	c	t	a	t	a	g	a	a	c	t	t	a	a	c	t	c	g	c	t	g	g	t	t	c
KP771896	c	g	c	c	a	c	g	c	t	a	a	a	g	g	c	t	a	t	a	g	a	a	c	t	t	a	a	c	t	c	g	c	t	g	g	t	t	c
KP771897	c	a	c	c	a	t	g	c	t	g	a	a	g	g	c	t	a	t	a	g	a	a	c	t	t	a	a	c	t	c	g	c	t	g	g	t	t	c
KP771899	c	a	c	c	a	c	g	c	t	g	a	a	g	g	c	t	a	t	a	g	a	a	c	t	t	a	a	c	t	c	g	c	t	g	g	t	t	c
KP771903	c	a	c	c	a	c	g	c	t	g	c	a	g	g	c	t	a	t	a	g	a	a	c	t	t	a	a	c	t	c	g	c	t	g	g	t	t	c
KP771907	c	a	c	c	a	c	g	c	t	g	a	a	g	g	c	t	a	t	a	g	a	a	c	t	t	a	a	c	t	c	g	c	t	g	g	t	t	c
KP771910	c	a	c	c	a	c	g	c	t	g	c	a	g	g	c	t	a	t	a	g	a	a	c	t	t	a	a	c	t	c	g	c	t	g	g	t	t	c
KP771915	c	a	c	c	a	t	g	c	t	g	a	a	g	g	c	t	a	t	a	t	a	a	c	t	t	a	a	c	t	c	g	c	t	g	g	t	t	c
KP771916	c	a	c	c	a	c	g	c	t	g	c	a	g	g	c	t	a	t	a	g	a	a	c	t	t	a	a	c	t	c	g	c	t	g	g	t	t	c
KP771921	c	a	c	c	a	c	g	c	t	g	c	a	g	g	c	t	a	t	a	g	a	a	c	t	t	a	a	c	t	c	g	c	t	g	g	t	t	c
KP771923	c	a	c	c	a	c	g	c	t	g	a	a	g	g	c	t	a	t	a	g	a	a	c	t	t	a	a	c	t	c	g	c	t	g	g	t	t	c
KP771889	c	a	c	c	a	t	g	c	t	g	a	a	g	g	c	t	a	t	a	t	a	a	c	t	t	a	a	c	t	c	g	c	t	g	g	t	t	c
KU926311	c	a	c	c	a	c	g	c	t	g	a	a	g	g	c	t	a	t	a	g	a	a	c	t	t	a	a	c	t	c	g	c	t	g	g	t	t	c
JQ911599	c	a	c	c	a	c	g	c	t	g	a	a	g	g	c	t	a	t	a	g	a	a	c	t	t	a	a	c	t	c	g	c	t	g	g	t	t	c
MH709317	c	a	c	c	a	c	g	c	t	g	a	a	g	g	c	t	a	t	a	g	a	a	c	t	t	a	a	c	t	c	a	c	t	g	g	t	t	c
MH709346	c	a	c	c	a	c	g	c	t	g	a	a	g	g	c	t	a	t	a	g	a	a	c	t	t	a	a	c	t	c	a	c	t	g	g	t	t	c
MH709375	c	a	c	c	a	c	g	c	t	g	a	a	g	g	c	t	a	t	a	g	a	a	c	t	t	a	a	c	t	c	a	c	t	g	g	t	t	c
DQ452050	c	a	c	c	a	c	g	t	t	g	a	a	g	a	c	t	a	c	a	g	a	g	c	t	t	a	a	c	t	c	g	c	t	g	g	t	t	c
DQ479960	c	a	c	c	a	c	g	t	t	g	a	a	g	a	c	t	a	c	a	g	a	g	c	t	t	a	a	c	t	c	g	c	t	g	g	t	t	c
KP771898	c	a	c	c	a	c	g	t	t	g	a	a	g	a	c	t	a	c	a	g	a	g	c	t	t	a	a	c	t	c	g	c	t	g	g	t	t	c
MH709316	c	a	c	c	a	c	g	t	t	g	a	a	g	a	c	t	a	c	a	g	a	g	c	t	t	a	a	c	t	c	g	c	t	g	g	t	t	c
MH709318	c	a	c	c	a	c	g	t	t	g	a	a	g	a	a	t	a	c	a	g	a	g	c	t	t	a	a	c	t	c	g	c	t	g	g	t	t	c
MH709322	t	a	c	c	a	c	g	t	t	g	a	a	g	a	a	t	a	c	a	g	a	g	c	t	t	a	a	c	t	c	g	c	t	g	g	t	t	c
MH709323	c	a	c	c	a	c	g	t	t	g	a	a	g	a	c	t	a	c	a	g	a	g	t	t	t	a	a	c	t	c	g	c	t	g	g	t	t	c
MH709326	c	a	c	c	a	c	g	t	t	g	a	a	g	a	a	t	a	c	a	g	a	g	c	t	t	a	a	c	t	c	g	c	t	g	g	t	t	c
MH709341	c	a	c	c	a	c	g	t	t	g	a	a	g	a	c	t	a	c	a	g	a	g	c	t	t	a	a	c	t	c	g	c	t	g	g	t	t	c
MH709357	c	a	c	c	a	c	g	t	t	g	a	a	g	a	c	t	a	c	a	g	a	g	c	t	t	a	a	c	t	c	g	c	t	g	g	t	t	c
MH709364	c	a	c	c	a	c	g	t	t	g	a	a	g	a	c	t	a	c	a	g	a	g	c	t	t	a	a	c	t	c	g	c	t	g	g	t	t	c
MH709370	c	a	c	c	a	c	g	t	t	g	a	a	g	a	c	t	a	c	a	g	a	g	c	t	t	a	a	c	t	c	g	c	t	g	g	t	t	c
MH709371	c	a	c	c	a	c	g	t	t	g	a	a	g	a	c	t	a	c	a	g	a	g	c	t	t	a	a	c	t	c	g	c	t	g	g	t	t	c
MH709372	c	a	c	c	a	c	g	t	t	g	a	a	g	a	c	t	a	c	a	g	a	g	c	t	t	a	a	c	t	c	g	c	t	g	g	t	t	c
DQ457052	c	a	c	c	a	c	g	c	t	g	a	c	g	g	c	t	a	t	c	g	a	a	c	t	t	a	a	c	t	c	g	c	t	g	g	t	t	c
JN704704	c	a	c	c	a	c	g	c	t	g	a	c	g	g	c	t	a	t	c	g	a	a	c	t	t	a	a	c	t	c	g	c	t	g	g	t	t	c
JN704705	c	a	c	c	a	c	g	c	t	g	a	a	g	g	c	t	a	t	a	g	a	a	c	t	t	a	a	c	t	c	g	c	t	t	g	t	t	c
JN704706	c	a	c	c	a	c	g	c	t	g	a	a	g	g	c	t	a	t	a	g	a	a	c	t	t	a	a	c	t	c	g	c	t	t	g	t	t	c
JN704707	c	a	t	c	a	c	g	c	t	g	a	c	g	g	c	t	a	t	c	g	a	a	c	t	t	a	a	c	t	c	g	c	t	g	g	t	t	c
JN704708	c	a	c	c	a	c	g	c	t	g	a	a	g	g	c	t	a	t	a	g	a	a	c	t	t	a	a	c	t	c	g	c	t	t	g	t	t	c
KP771904	c	a	c	c	a	c	g	c	t	g	a	c	g	g	c	t	a	t	c	g	a	a	c	t	t	a	a	c	t	c	g	c	t	g	g	t	t	c
KP771905	c	a	t	c	a	c	g	c	t	g	a	c	g	g	c	t	a	t	c	g	a	a	c	t	t	a	a	c	t	c	g	c	t	g	g	t	t	c
KP771906	c	a	t	c	a	c	g	c	t	g	a	c	g	g	c	t	a	t	c	g	a	a	c	t	t	a	a	c	t	c	g	c	t	g	g	t	t	c
KP771919	c	a	c	c	a	c	g	c	t	g	a	a	g	g	c	t	a	t	a	g	a	a	c	t	t	a	a	c	t	c	g	c	t	t	g	t	t	c
KP771922	c	a	t	c	a	c	g	c	t	g	a	c	g	g	c	t	a	t	c	g	a	a	c	t	t	a	a	c	t	c	g	c	t	g	g	t	t	c
KP771924	c	a	c	c	a	c	g	c	t	g	a	a	g	g	c	t	a	t	a	g	a	a	c	t	t	a	a	c	t	c	g	c	t	t	g	t	t	c
KM355696	c	a	c	c	a	c	g	c	t	g	a	a	g	g	c	t	a	t	a	g	a	a	c	t	t	a	a	c	t	c	g	c	t	t	g	t	t	c
KM355697	c	a	c	c	a	c	g	c	t	g	a	a	g	g	c	t	a	t	a	g	a	a	c	t	t	a	a	c	t	c	g	c	t	t	g	t	t	c
KM355698	c	a	c	c	a	c	g	c	t	g	a	a	g	g	c	t	a	t	a	g	a	a	c	t	t	a	a	c	t	c	g	c	t	t	g	t	t	c
KM355699	c	a	c	c	a	c	g	c	t	g	a	a	g	g	c	t	a	t	a	g	a	a	c	t	t	a	a	c	t	c	g	c	t	t	g	t	t	c
KM355701	c	a	c	c	a	c	g	c	t	g	a	a	g	g	c	t	a	t	a	g	a	a	c	t	t	a	a	c	t	c	g	c	t	t	g	t	t	c
KM355702	c	a	c	c	a	c	g	c	t	g	a	a	g	g	c	t	a	t	a	g	a	a	c	t	t	a	a	c	t	c	g	c	t	t	g	t	t	c
KM355703	c	a	c	c	a	c	g	c	t	g	a	a	g	g	c	t	a	t	a	g	a	a	c	t	t	a	a	c	t	c	g	c	t	t	g	t	t	c
KM355704	c	a	c	c	a	c	g	c	t	g	a	a	g	g	c	t	a	t	a	g	a	a	c	t	t	a	a	c	t	c	g	c	t	t	g	t	t	c
KM355705	c	a	c	c	a	c	g	c	t	g	a	a	g	g	c	t	a	t	a	g	a	a	c	t	t	a	a	c	t	c	g	c	t	t	g	t	t	c
KM355706	c	a	c	c	a	c	g	c	t	g	a	a	g	g	c	t	a	t	a	g	a	a	c	t	t	a	a	c	t	c	g	c	t	t	g	t	t	c
KM355707	c	a	c	c	a	c	g	c	t	g	a	a	g	g	c	t	a	t	a	g	a	a	c	t	t	a	a	c	t	c	g	c	t	t	g	t	t	c
KM355708	c	a	c	c	a	c	g	c	t	g	a	a	g	g	c	t	a	t	a	g	a	a	c	t	t	a	a	c	t	c	g	c	t	t	g	t	t	c
KM355709	c	a	c	c	a	c	g	c	t	g	a	a	g	g	c	t	a	t	a	g	a	a	c	t	t	a	a	c	t	c	g	c	t	t	g	t	t	c
KM355711	c	a	c	c	a	c	g	c	t	g	a	a	g	g	c	t	a	t	a	g	a	a	c	t	t	a	a	c	t	c	g	c	t	t	g	t	t	c
KM355712	c	a	c	c	a	c	g	c	t	g	a	a	g	g	c	t	a	t	a	g	a	a	c	t	t	a	a	c	t	c	g	c	t	t	g	t	t	c
[illegible]	[illegible]	[illegible]	[illegible]	[illegible]	[illegible]	[illegible]	[illegible]	[illegible]	[illegible]	[illegible]	[illegible]	[illegible]	[illegible]	[illegible]	[illegible]	[illegible]	[illegible]	[illegible]	[illegible]	[illegible]	[illegible]	[illegible]	[illegible]	[illegible]	[illegible]	[illegible]	[illegible]	[illegible]	[illegible]	[illegible]	[illegible]	[illegible]	[illegible]	[illegible]	[illegible]	[illegible]	[illegible]	[illegible]

Supplementary Table 3: 2880 SN

GB-ID	Clade markers				1			4	SD14																									9							6																																				
	Position in Dumas	22038	22152	22192	22311	22343	22350	22362	22392	22443	22460	22484	22504	22631	22670	22684	22689	22704	22714	22769	22776	22794	22876	23287	23294	23332	23337	23378	23393	23429	23665	23669	23705	23717	23722	23756	23779	23784	23898	23904	23959	23960	24071	24085	24092	24095	24135	24328	24533	24567	24578	24628	24646	24651	24654	24656	24700	25038	25067	25089	25105	25106	25267	25288	25309	25471	25500	25558	25561	25630	25675	25783	25816	25851	25894
MH499467	5	a	c	g	a	a	g	c	t	t	t	a	a	a	g	a	g	c	t	t	t	a	c	g	g	c	t	g	c	g	g	t	c	t	c	c	g	c	a	t	c	a	c	g	c	t	g	a	c	g	g	c	t	a	t	c	g	a	a	c	t	t	a	a	c	t	c	g	c	t	g	g	t	t	c
MH499469	5	a	c	g	a	a	g	c	t	t	t	a	a	a	g	a	g	c	t	t	t	a	c	g	g	c	t	g	c	g	g	t	c	t	c	c	g	c	a	c	c	a	c	g	c	t	g	a	c	g	g	c	t	a	t	c	g	a	a	c	t	t	a	a	c	t	c	g	c	t	g	g	t	t	c
MH709321	5	a	c	g	a	a	t	c	t	t	t	a	a	a	g	a	g	c	t	t	t	a	c	g	g	c	t	g	c	g	g	t	c	t	c	c	g	c	a	c	c	a	c	g	c	t	g	a	a	g	g	c	t	a	t	a	g	a	a	c	t	t	a	a	c	t	c	g	c	t	t	g	t	t	c
MH709324	5	a	c	g	a	a	g	c	t	t	t	a	a	a	g	a	g	c	t	t	t	a	c	g	g	c	t	g	c	g	g	t	c	t	c	c	g	c	a	c	c	a	c	g	c	t	g	a	c	g	g	c	t	a	t	c	g	a	a	c	t	t	a	a	c	t	c	g	c	t	g	g	t	t	c
MH709325	5	a	c	g	a	a	g	c	t	t	t	a	a	a	g	a	g	c	t	t	t	a	c	g	g	c	t	g	c	g	g	t	c	t	c	c	g	c	a	c	c	a	c	g	c	t	g	a	a	g	g	c	t	a	t	a	g	a	a	c	t	t	a	a	c	t	c	g	c	t	t	g	t	t	c
MH709327	5	a	c	g	a	a	g	c	t	t	t	a	a	a	g	a	g	c	t	t	t	a	c	g	g	c	t	g	c	g	g	t	c	t	c	c	g	c	a	c	c	a	c	g	c	t	g	a	c	g	g	c	t	a	t	c	g	a	a	c	t	t	a	a	c	t	c	g	c	t	g	g	t	t	c
MH709328	5	a	c	g	a	a	g	c	t	t	t	a	a	a	g	a	g	c	t	t	t	a	c	g	g	c	t	g	c	g	g	t	c	t	c	c	g	c	a	c	c	a	c	g	c	t	g	a	c	g	g	c	t	a	t	c	g	a	a	c	t	t	a	a	c	t	c	g	c	t	g	g	t	t	c
MH709330	5	a	c	g	a	a	g	c	t	t	t	a	a	a	g	a	g	c	t	t	t	a	c	g	g	c	t	g	c	g	g	t	c	t	c	c	g	c	a	c	c	a	c	g	c	t	g	a	c	g	g	c	t	a	t	c	g	a	a	c	t	t	a	a	c	t	c	g	c	t	g	g	t	t	c
MH709332	5	a	c	g	a	a	g	c	t	t	t	a	a	a	g	a	g	c	t	t	t	a	c	g	g	c	t	g	c	g	g	t	c	t	c	c	g	c	a	c	c	a	c	g	c	t	g	a	c	g	g	c	t	a	t	c	g	a	a	c	t	t	a	a	c	t	c	g	c	t	g	g	t	t	c
MH709334	5	a	c	g	a	a	g	c	t	t	t	a	a	a	g	a	g	c	t	t	t	a	c	g	g	c	t	g	c	g	g	t	c	t	c	c	g	c	a	c	c	a	c	g	c	t	g	a	c	g	g	c	t	a	t	c	g	a	a	c	t	t	a	a	c	t	c	g	c	t	g	g	t	t	c
MH709335	5	a	c	g	a	a	g	c	t	t	t	a	a	a	g	a	g	c	t	t	t	a	c	g	g	c	t	g	c	g	g	t	c	t	c	c	g	c	a	c	c	a	c	g	c	t	g	a	c	g	g	c	t	a	t	c	g	a	a	c	t	t	a	a	c	t	c	g	c	t	g	g	t	t	c
MH709336	5	a	c	g	a	a	g	c	t	t	t	a	a	a	g	a	g	c	t	t	t	a	c	g	g	c	t	g	c	g	g	t	c	t	c	c	g	c	a	c	c	a	c	g	c	t	g	a	c	g	g	c	t	a	t	c	g	a	a	c	t	t	a	a	c	t	c	g	c	t	g	g	t	t	c
MH709338	5	a	c	g	a	a	g	c	t	t	t	a	a	a	g	a	g	c	t	t	t	a	c	g	g	c	t	g	c	g	g	t	c	t	c	c	g	c	a	c	c	a	c	g	c	t	g	a	a	g	g	c	t	a	t	c	g	a	a	c	t	t	a	a	c	t	c	g	c	t	g	g	t	t	c
MH709340	5	a	c	g	a	a	g	c	t	t	t	a	a	a	g	a	g	c	t	t	t	a	c	g	g	c	t	g	c	g	g	t	c	t	c	c	g	c	a	c	c	a	c	g	c	t	g	a	a	g	g	c	t	a	t	a	g	a	a	c	t	t	a	a	c	t	c	g	c	t	t	g	t	c	c
MH709342	5	a	c	g	a	a	t	c	t	t	t	a	a	a	g	a	g	c	t	t	t	a	c	g	g	c	t	g	c	g	g	t	c	t	c	c	g	c	a	c	c	a	c	g	c	t	g	a	a	g	g	c	t	a	t	a	g	a	a	c	t	t	a	a	c	t	c	g	c	t	t	g	t	t	c
MH709343	5	a	c	g	a	a	g	c	t	t	t	a	a	a	g	a	g	c	t	t	c	a	c	g	g	c	t	g	c	g	g	t	c	t	c	c	g	c	a	t	c	a	c	g	c	t	g	a	c	g	g	c	t	a	t	c	g	a	a	c	t	t	a	a	c	t	c	g	c	t	g	g	t	t	c
MH709345	5	a	c	g	a	a	g	c	t	t	t	a	a	a	g	a	g	c	t	t	t	a	c	g	g	c	t	g	c	g	g	t	c	t	c	c	g	c	a	c	c	a	c	g	c	t	g	a	c	g	g	c	t	a	t	c	g	a	a	c	t	t	a	a	c	t	c	g	c	t	g	g	t	t	c
MH709347	5	a	c	g	a	a	g	c	t	t	t	a	a	a	g	a	g	c	t	t	t	a	c	g	g	c	t	g	c	g	g	t	c	t	c	c	g	c	a	c	c	a	c	g	c	t	g	a	c	g	g	c	t	a	t	c	g	a	a	c	t	t	a	a	c	t	c	g	c	t	g	g	t	t	c
MH709349	5	a	c	g	a	a	g	c	t	t	t	a	a	a	g	a	g	c	t	t	t	a	c	g	g	c	t	g	c	g	g	t	c	t	c	c	g	c	a	c	c	a	c	g	c	t	g	a	c	g	g	c	t	a	t	c	g	a	a	c	t	t	a	a	c	t	c	g	c	t	g	g	t	t	c
MH709350	5	a	c	g	a	a	g	c	t	t	t	a	a	a	g	a	g	c	t	t	t	a	c	g	g	c	t	g	c	g	g	t	c	t	c	c	g	c	a	c	c	a	c	g	c	t	g	a	c	g	g	c	t	a	t	c	g	a	a	c	c	t	a	a	c	t	c	g	c	t	g	g	t	t	c
MH709351	5	a	c	g	a	a	g	c	t	t	t	a	a	a	g	a	g	c	t	t	t	a	c	g	g	c	t	g	c	g	g	t	c	t	c	c	g	c	a	c	c	a	c	g	c	t	g	a	a	g	g	c	t	a	t	a	g	a	a	c	t	t	a	a	c	t	c	g	c	t	t	g	t	t	c
MH709353	5	a	c	g	a	a	g	c	t	t	t	a	a	a	g	a	g	c	t	t	t	a	c	g	g	c	t	g	c	g	g	t	c	t	c	c	g	c	a	c	c	a	c	g	c	t	g	a	c	g	g	c	t	a	t	c	g	a	a	c	t	t	a	a	c	t	c	g	c	t	g	g	t	t	c
MH709358	5	a	c	g	a	a	g	c	t	t	t	a	a	a	g	a	g	c	t	t	t	a	c	g	g	c	t	g	c	g	g	t	c	t	c	c	g	c	a	c	c	a	c	g	c	t	g	a	a	g	g	c	t	a	t	a	g	a	a	c	t	t	a	a	c	t	c	g	c	t	t	g	t	t	c
MH709360	5	a	c	g	a	a	g	c	t	t	t	a	a	a	g	a	g	c	t	t	t	a	c	g	g	c	t	g	c	g	g	t	c	t	c	c	g	c	a	c	c	a	c	g	c	t	g	a	c	g	g	c	t	a	t	c	g	a	a	c	t	t	a	a	c	t	c	g	c	t	g	g	t	t	c
MH709362	5	a	c	g	a	a	g	c	t	t	t	a	a	a	g	a	g	c	t	t	t	a	c	g	g	c	t	g	c	g	g	t	c	t	c	c	g	c	a	c	c	a	c	g	c	t	g	a	c	g	g	c	t	a	t	c	g	a	a	c	t	t	a	a	c	t	c	g	c	t	g	g	t	t	c
MH709365	5	a	c	g	a	a	g	c	t	t	t	a	a	a	g	a	g	c	c	t	t	a	c	g	g	c	t	g	c	g	g	t	c	t	c	c	g	c	a	c	c	a	c	g	c	t	g	a	c	g	g	c	t	a	t	c	g	a	a	c	t	t	a	a	c	t	c	g	c	t	g	g	t	t	c
MH709373	5	a	c	g	a	a	g	c	t	t	t	a	a	a	g	a	g	c	t	t	t	a	c	g	g	c	t	g	c	g	g	t	c	t	c	c	g	c	a	t	c	a	c	g	c	t	g	a	c	g	g	c	t	a	t	c	g	a	a	c	t	t	a	a	c	t	c	g	c	t	g	g	t	t	c
MH709374	5	a	c	g	a	a	g	c	t	t	t	a	a	a	g	a	g	c	t	t	t	a	c	g	g	c	t	g	c	g	g	t	c	t	c	c	g	c	a	c	c	a	c	g	c	t	g	a	c	g	g	c	t	a	t	c	g	a	a	c	t	t	a	a	c	t	c	g	c	t	g	g	t	t	c
KC112914	6	a	c	g	a	a	g	c	t	t	t	a	a	a	g	a	g	c	t	t	t	a	c	g	g	c	t	g	c	g	g	t	c	t	c	c	g	c	a	c	a	a	c	g	t	t	g	a	a	g	g	c	t	a	t	a	g	a	a	c	t	t	a	a	c	t	c	g	c	t	g	g	t	t	c
KP771914	6	a	c	g	a	a	g	c	t	t	t	a	a	a	g	a	g	c	t	t	t	a	c	g	g	c	t	g	c	g	g	t	c	t	c	c	g	c	a	c	a	a	c	g	t	t	g	a	a	g	g	c	t	a	t	a	g	a	a	c	t	t	a	a	c	t	c	g	c	t	g	g	t	t	c
KP702725	6	a	c	g	a	a	g	c	t	t	t	a	a	a	g	a	g	c	t	t	t	a	c	g	g	c	t	g	c	g	g	t	c	t	c	c	g	c	a	c	a	a	c	g	t	t	g	a	a	g	g	c	t	a	t	a	g	a	a	c	t	t	a	a	c	t	c	g	c	t	g	g	t	t	c
KP771890	9	a	c	g	a	a	g	c	t	t	t	c	a	a	g	a	g	c	t	t	t	a	c	g	a	c	t	g	c	a	g	t	c	c	c	c	g	c	a	c	c	a	c	g	t	t	g	a	a	g	a	c	t	a	c	a	g	a	g	c	t	t	a	a	c	t	c	g	c	c	g	g	t	t	c
JN704710	9	a	c	g	a	a	g	c	t	t	t	c	a	a	g	a	g	c	t	t	t	a	c	g	a	c	t	g	c	a	g	t	c	c	c	c	g	c	a	c	c	a	c	g	t	t	g	a	a	g	a	c	t	a	c	a	g	a	g	c	t	t	a	a	c	t	c	g	c	t	g	g	t	t	c
KY037798	9	a	c	g	a	a	g	c	t	t	t	c	a	a	g	a	g	c	t	t	t	a	c	g	a	c	t	g	c	a	g	t	c	c	c	c	g	c	a	c	c	a	c	g	t	t	g	a	a	g	a	c	t	a	c	a	g	a	g	c	t	t	a	a	a	t	c	g	c	t	g	g	t	t	c
MH709312	9	a	c	g	a	a	g	c	t	t	t	c	a	a	g	a	g	c	t	t	t	a	c	g	a	c	t	g	c	a	g	t	c	c	c	c	g	c	a	c	c	a	c	g	t	t	g	a	a	g	a	c	t	a	c	a	g	a	g	c	t	t	a	a	a	t	c	g	c	t	g	g	t	t	c
MH709352	9	a	c	g	a	a	g	c	t	t	t	c	a	a	g	a	g	c	t	t	t	a	c	g	a	c	t	g	c	a	g	t	c	c	c	c	g	c	a	c	c	a	c	g	t	t	g	a	a	g	a	c	t	a	c	a	g	a	g	c	t	t	a	a	c	t	c	g	c	t	g	g	t	t	c
AB097933	Vac	a	c	g	a	a	g	c	t	t	t	a	g	a	g	a	g	c	t	t	t	g	c	g	g	c	t	g	c	a	g	t	c	t	c	c	g	c	a	c	c	a	c	g	t	t	g	a	a	g	g	c	t	a	t	a	g	a	a	c	t	t	a	a	c	t	c	g	c	t	g	g	t	t	c
JN704698	Vac	a	c	g	a	a	g	c	t	t	t	a	g	a	g	a	g	c	t	t	t	g	c	g	g	c	t	g	c	a	g	t	c	t	c	c	g	c	a	c	c	a	c	g	t	t	g	a	a	g	g	c	t	a	t	a	g	a	a	c	t	t	a	a	c	t	c	g	c	t	g	g	t	t	c
JN704697	Vac	a	c	g	a	a	g	c	t	t	t	a	g	a	g	a	g	c	t	t	t	g	c	g	g	c	t	g	c	a	g	t	c	t	c	c	g	c	a	c	c	a	c	g	t	t	g	a	a	g	g	c	t	a	t	a	g	a	a	c	t	t	a	a	c	t	c	g	c	t	g	g	t	t	c
DQ008355	Vac	a	c	g	a	a	g	c	t	t	t	a	g	a	g	a	g	c	t	t	t	g	c	g	g	c	t	g	c	a	g	t	c	t	c	c	g	c	a	c	c	a	c	g	t	t	g	a	a	g	g	c	t	a	t	a	g	a	a	c	t	t	a	a	c	t	c	g	c	t	g	g	t	t	c
JF306641	Vac	a	c	g	a	a	g	c	t	t	t	a	g	a	g	a	g	c	t	t	t	g	c	g	g	c	t	g	c	a	g	t	c	t	c	c	g	c	a	c	c	a	c	g	t	t	g	a	a	g	g	c	t	a	t	a	g	a	a	c	t	t	a	a	c	t	c	g	c	t	g	g	t	t	c
AB097932	Vac	a	c	g	a	a	g	c	t	t	t	a	g	a	g	a	g	c	t	t	t	g	c	g	g	c	t	g	c	a	g	t	c	t	c	c	g	c	a	c	c	a	c	g	t	t	g	a	a	g	g	c	t	a	t	a	g	a	a	c	t	t	a	a	c	t	c	g	c	t	g	g	t	t	c
DQ008354	Vac	a	c	g	a	a	g	c	t	t	t	a	g	a	g	a	g	c	t	t	t	g	c	g	g	c	t	g	c	a	g	t	c	t	c	c	g	c	a	c	c	a	c	g	t	t	g	a	a	g	g	c	t	a	t	a	g	a	a	c	t	t	a	a	c	t	c	g	c	t	g	g	t	t	c
KU926312	Vac	a	c	g	a	a	g	c	t	t	t	a	g	a	g	a	g	c	t	t	t	g	c	g	g	c	t	g	c	a	g	t	c	t	c	c	g	c	a	c	c	a	c	g	t	t	g	a	a	g	g	c	t	a	t	a	g	a	a	c	t	t	a	a	c	t	c	g	c	t	g	g	t	t	c
KU926313	Vac	a	c	g	a	a	g	c	t	t	t	a	g	a	g	a	g	c	t	t	t	g	c	g	g	c	t	g	c	a	g	t	c	t	c	c	g	c	a	c	c	a	c	g	t	t	g	a	a	g	g	c	t	a	t	a	g	a	a	c	t	t	a	a	c	t	c	g	c	t	g	g	t	t	c
KU926314	Vac	a	c	g	a	a	g	c	t	t	t	a	g	a	g	a	g	c	t	t	t	g	c	g	g	c	t	g	c	a	g	t	c	t	c	c	g	c	a	c	c	a	c	g	t	t	g	a	a	g	g	c	t	a	t	a	g	a	a	c	t	t	a	a	c	t	c	g	c	t	g	g	t	t	c
KF558371	Vac	a	c	g	a	a	g	c	t	t	t	a	g	a	g	a	g	c	t	t	t	g	c	g	g	c	t	g	c	a	g	t	c	t	c	c	g	c	a	c	c	a	c	g	t	t	g	a	a	g	g	c	t	a	t	a	g	a	a	c	t	t	a	a	c	t	c	g	c	t	g	g	t	t	c
KF558372	Vac	a	c	g	a	a	g	c	t	t	t	a	g	a	g	a	g	c	t	t	t	g	c	g	g	c	t	g	c	a	g	t	c	t	c	c	g	c	a	c	c	a	c	g	t	t	g	a	a	g	g	c	t	a	t	a	g	a	a	c	t	t	a	a	c	t	c	g	c	t	g	g	t	t	c
KF558373	Vac	a	c	g	a	a	g	c	t	t	t	a	g	a	g	a	g	c	t	t	t	g	c	g	g	c	t	g	c	a	g	t	c	t	c	c	g	c	a	c	c	a	c	g	t	t	g	a	a	g	g	c	t	a	t	a	g	a	a	c	t	t	a	a	c	t	c	g	c	t	g	g	t	t	c
KF558374	Vac	a	c	g	a	a	g	c	t	t	t	a	g	a	g	a	g	c	t	t	t	g	c	g	g	c	t	g	c	a	g	t	c	t	c	c	g	c	a	c	c	a	c	g	t	t	g	a	a	g	g	c	t	a	t	a	g	a	a	c	t	t	a	a	c	t	c	g	c	t	g	g	t	t	c
KF558375	Vac	a	c	g	a	a	g	c	t	t	t	a	g	a	g	a	g	c	t	t	t	g	c	g	g	c	t	g	c	a	g	t	c	t	c	c	g	c	a	c	c	a	c	g	t	t	g	a	a	g	g	c	t	a	t	a	g	a	a	c	t	t	a	a	c	t	c	g	c	t	g	g	t	t	c
KF558376	Vac	a	c	g	a	a	g	c	t	t	t	a	g	a	g	a	g	c	t	t	t	g	c	g	g	c	t	g	c	a	g	t	c	t	c	c	g	c	a	c	c	a	c	g	t	t	g	a	a	g	g	c	t	a	t	a	g	a	a	c	t	t	a	a	c	t	c	g	c	t	g	g	t	t	c
KF558377	Vac	a	c	g	a	a	g	c	t	t	t	a	g	a	g	a	g	c	t	t	t	g	c	g	g	c	t	g	c	a	g	t	c	t	c	c	g	c	a	c	c	a	c	g	t	t	g	a	a	g	g	c	t	a	t	a	g	a	a	c	t	t	a	a	c	t	c	g	c	t	g	g	t	t	c
KF558378	Vac	a	c	g	a	a	g	c	t	t	t	a	g	a	g	a	g	c	t	t	t	g	c	g	g	c	t	g	c	a	g	t	c	t	c	c	g	c	a	c	c	a	c	g	t	t	g	a	a	g	g	c	t	a	t	a	g	a	a	c	t	t	a	a	c	t	c	g	c	t	g	g	t	t	c
KF558379	Vac	a	c	g	a	a	g	c	t	t	t	a	g	a	g	a	g	c	t	t	t	g	c	g	g	c	t	g	c	a	g	t	c	t	c	c	g	c	a	c	c	a	c	g	t	t	g	a	a	g	g	c	t	a	t	a	g	a	a	c	t	t	a	a	c	t	c	g	c	t	g	g	t	t	c
KF558380	Vac	a	c	g	a	a	g	c	t	t	t	a	g	a	g	a	g	c	t	t	t	g	c	g	g	c	t	g	c	a	g	t	c	t	c	c	g	c	a	c	c	a	c	g	t	t	g	a	a	g	g	c	t	a	t	a	g	a	a	c	t	t	a	a	c	t	c	g	c	t	g	g	t	t	c
KF558381	Vac	a	c	g	a	a	g	c	t	t	t	a	g	a	g	a	g	c	t	t	t	g	c	g	g	c	t	g	c	a	g	t	c	t	c	a	g	c	a	c	c	a	c	g	t	t	g	a	a	g	g	c	t	a	t	a	g	a	a	c	t	t	a	a	c	t	c	g	c	t	g	g	t	t	c
KF558382	Vac	a	c	g	a	a	g	c	t	t	t	a	g	a	g	a	g	c	t	t	t	g	c	g	g	c	t	g	c	a	g	t	c	t	c	c	g	c	a	c	c	a	c	g	t	t	g	a	a	g	g	c	t	a	t	a	g	a	a	c	t	t	a	a	c	t	c	g	c	t	g	g	t	t	c
KF558383	Vac	a	c	g	a	a	g	c	t	t	t	a	g	a	g	a	g	c	t	t	t	g	c	g	g	c	t	g	c	a	g	t	c	t	c	c	g	c	a	c	c	a	c	g	t	t	g	a	a	g	g	c	t	a	t	a	g	a	a	c	t	t	a	a	c	t	c	g	c	t	g	g	t	t	c
KF558384	Vac	a	c	g	a	a	g	c	t	t	t	a	g	a	g	a	g	c	t	t	t	g	c	g	g	c	t	g	c	a	g	t	c	t	c	c	g	c	a	c	c	a	c	g	t	t	g	a	a	g	g	c	t	a	t	a	g	a	a	c	t	t	a	a	c	t	c	g	c	t	g	g	t	t	c
KF558385	Vac	a	c	g	a	a	g	c	t	t	t	a	g	a	g	a	g	c	t	t	t	g	c	g	g	c	t	g	c	a	g	t	c	t	c	c	g	c	a	c	c	a	c	g	t	t	g	a	a	g	g	c	t	a	t	a	g	a	a	c	t	t	a	a	c	t	c	g	c	t	g	g	t	t	c
KF558386	Vac	a	c	g	a	a	g	c	t	t	t	a	g	a	g	a	g	c	t	t	t	g	c	g	g	c	t	g	c	a	g	t	c	t	c	c	g	c	a	c	c	a	c	g	t	t	g	a	a	g	g	c	t	a	t	a	g	a	a	c	t	t	a	a	c	t	c	g	c	t	g	g	t	t	c
KF558387	Vac	a	c	g	a	a	g	c	t	t	t	a	g	a	g	a	g	c	t	t	t	g	c	g	g	c	t	g	c	a	g	t	c	t	c	c	g	c	a	c	c	a	c	g	t	t	g	a	a	g	g	c	t	a	t	a	g	a	a	c	t	t	a	a	c	t	a	g	c	t	g	g	t	t	c
KF558388	Vac	a	c	g	a	a	g	c	t	t	t	a	g	a	g	a	g	c	t	t	t	g	c	g	g	c	t	g	c	a	g	t	c	t	c	c	g	c	a	c	c	a	c	g	t	t	g	a	a	g	g	c	t	a	t	a	g	a	a	c	t	t	a	a	c	t	c	g	c	t	g	g	t	t	c
KF558389	Vac	a	c	g	a	a	g	c	t	t	t	a	g	a	g	a	g	c	t	t	t	g	c	g	g	c	t	g	c	a	g	t	c	t	c	c	g	c	a	c	c	a	c	g	t	t	g	a	a	g	g	c	t	a	t	a	g	a	a	c	t	t	a	a	c	t	c	g	c	t	g	g	t	t	c
KF558390	Vac	a	c	g	a	a	g	c	t	t	t	a	g	a	g	a	g	c	t	t	t	g	c	g	g	c	t	g	c	a	g	t	c	t	c	c	g	c	a	c	c	a	c	g	t	t	g	a	a	g	g	c	t	a	t	a	g	a	a	c	t	t	a	a	c	t	c	g	c	t	g	g	t	t	c
KF558391	Vac	a	c	g	a	a	g	c	t	t	t	a	g	a	g	a	g	c	t	t	t	g	c	g	g	c	t	g	c	a	g	t	c	t	c	c	g	c	a	c	c	a	c	g	t	t	g	a	a	g	g	c	t	a	t	a	g	a	a	c	t	t	a	a	c	t	c	g	c	t	g	g	t	t	c
KF811485	Vac	a	c	g	a	a	g	c	t	t	t	a	g	a	g	a	g	c	t	t	t	g	c	g	g	c	t	g	c	a	g	t	c	t	c	c	g	c	a	c	c	a	c	g	t	t	g	a	a	g	g	c	t	g	t	a	g	a	a	c	t	t	a	a	c	t	c	g	c	t	g	g	t	t	c
KF853225	Vac	a	c	g	a	a	g	c	t	t	t	a	g	a	g	a	g	c	t	t	t	g	c	g	g	c	t	g	c	a	g	t	c	t	c	c	g	c	a	c	c	a	c	g	t	t	g	a	a	g	g	c	t	a	t	a	g	a	a	c	t	t	a	a	c	t	c	g	c	t	g	g	t	t	c
KF853226	Vac	a	c	g	a	a	g	c	t	t	t	a	g	a	g	a	g	c	t	t	t	g	c	g	g	c	t	g	c	a	g	t	c	t	c	c	g	c	a	c	c	a	c	g	t	t	g	a	a	g	g	c	t	a	t	a	g	a	a	c	t	t	a	a	c	t	c	g	c	t	g	g	t	t	c
KF853227	Vac	a	c	g	a	a	g	c	t	t	t	a	g	a	g	a	g	c	t	t	t	g	c	g	g	c	t	g	c	a	g	t	c	t	c	c	g	c	a	c	c	a	c	g	t	t	g	a	a	g	g	c	t	a	t	a	g	a	a	c	t	t	a	a	c	t	c	g	c	t	g	g	t	t	c
KF853228	Vac	a	c	g	a	a	g	c	t	t	t	a	g	a	g	a	g	c	t	t	t	g	c	g	g	c	t	g	c	a	g	t	c	t	c	c	g	c	a	c	c	a	c	g	t	t	g	a	a	g	g	c	t	a	t	a	g	a	a	c	t	t	a	a	c	t	c	g	c	t	g	g	t	t	c
KF853229	Vac	a	c	g	a	a	g	c	t	t	t	a	g	a	g	a	g	c	t	t	t	g	c	g	g	c	t	g	c	a	g	t	c	t	c	c	g	c	a	c	c	a	c	g	t	t	g	a	a	g	g	c	t	a	t	a	g	a	a	c	t	t	a	a	c	t	c	g	c	t	g	g	t	t	c
KF853230	Vac	a	c	g	a	a	g	c	t	t	t	a	g	a	g	a	g	c	t	t	t	g	c	g	g	c	t	g	c	a	g	t	c	t	c	c	g	c	a	c	c	a	c	g	t	t	g	a	a	g	g	c	t	a	t	a	g	a	a	c	t	t	a	a	c	t	c	g	c	t	g	g	t	t	c
KF853231	Vac	a	c	g	a	a	g	c	t	t	t	a	g	a	g	a	g	c	t	t	t	g	c	g	g	c	t	g	c	a	g	t	c	t	c	c	g	c	a	c	c	a	c	g	t	t	g	a	a	g	g	c	t	a	t	a	g	a	a	c	t	t	a	a	c	t	c	g	c	t	g	g	t	t	c
KF853232	Vac	a	c	g	a	a	g	c	t	t	t	a	g	a	g	a	g	c	t	t	t	g	c	g	g	c	t	g	c	a	g	t	c	t	c	c	g	c	a	c	c	a	c	g	t	t	g	a	a	g	g	c	t	g	t	a	g	a	a	c	t	t	a	a	c	t	c	g	c	t	g	g	t	t	c
KF853233	Vac	a	c	g	a	a	g	c	t	t	t	a	g	a	g	a	g	c	t	t	t	g	c	g	g	c	t	g	c	a	g	t	c	t	c	c	g	c	a	c	c	a	c	g	t	t	g	a	a	g	g	c	t	a	t	a	g	a	a	c	t	t	a	a	c	t	c	g	c	t	g	g	t	t	c
KF853234	Vac	a	c	g	a	a	g	c	t	t	t	a	g	a	g	a	g	c	t	t	t	g	c	g	g	c	t	g	c	a	g	t	c	t	c	c	g	c	a	c	c	a	c	g	t	t	g	a	a	g	g	c	t	a	t	a	g	a	a	c	t	t	a	a	c	t	c	g	c	t	g	g	t	t	c
KF853235	Vac	a	c	g	a	a	g	c	t	t	g	a	g	a	g	a	g	c	t	t	t	g	c	g	g	c	t	g	c	a	g	t	c	t	c	c	g	c	a	c	c	a	c	g	t	t	g	a	a	g	g	c	t	a	t	a	g	a	a	c	t	t	a	a	c	t	c	g	c	t	g	g	t	t	c
MF898328	Vac	a	c	g	a	a	g	c	t	t	t	a	g	a	g	a	g	c	t	t	t	g	c	g	g	c	t	g	c	a	g	t	c	t	c	c	g	c	a	c	c	a	c	g	t	t	g	a	a	g	g	c	t	a	t	a	g	a	a	c	t	t	a	a	c	t	c	g	c	t	g	g	t	t	c
JN704709	VIII	a	c	g	a	a	g	c	t	t	t	a	g	a	g	a	g	c	t	t	t	g	c	g	g	c	t	g	c	a	g	t	c	t	c	c	g	c	a	c	c	a	c	g	t	t	g	a	a	g	g	c	t	a	t	a	g	a	a	c	t	t	a	a	c	t	c	g	c	t	g	g	t	t	c

Supplementary Table 3: 2880 SN

GB-ID	Clade markers																																	
	Position in Dumas	25895	26125	26296	26572	26821	26949	27258	27259	27286	27291	27343	27394	27405	27406	27435	27469	27503	27523	27634	27797	27844	27868	27922	27942	28042	28111	28167	28168	28291	28486	28524	28602	28642
AY548170	1	g	a	a	c	c	t	c	a	t	c	t	a	c	c	c	t	c	a	t	a	t	t	a	g	t	a	c	a	c	a	a	t	g
AY548171	1	g	a	a	c	c	t	c	a	t	c	t	a	c	c	c	t	c	a	t	a	t	t	a	g	t	a	c	a	c	a	a	t	g
DQ479953	1	g	a	a	c	c	t	c	a	t	c	t	a	c	c	c	t	c	a	t	a	t	t	a	g	t	a	c	a	c	a	a	t	g
DQ479954	1	g	a	a	c	c	t	c	a	t	c	t	a	c	c	c	t	c	a	t	a	t	t	a	g	t	a	c	a	c	a	a	t	g
DQ479958	1	g	a	a	c	c	t	c	a	t	c	t	a	c	c	c	t	c	a	t	a	t	t	a	g	t	a	c	a	c	a	a	t	g
DQ479959	1	g	a	a	c	c	t	c	a	t	c	t	a	c	c	c	t	c	a	t	a	t	t	a	g	t	a	c	a	a	a	a	t	g
DQ479961	1	g	a	a	c	c	t	c	a	t	c	t	a	c	c	c	t	c	a	t	a	t	t	a	g	t	a	c	a	c	a	a	t	g
DQ479962	1	g	a	a	c	c	t	c	a	t	c	t	a	c	c	c	t	c	a	t	a	t	t	a	g	t	a	c	a	c	a	a	t	g
DQ479963	1	g	a	a	c	c	t	c	a	t	c	t	a	c	c	c	t	c	a	t	a	t	t	a	g	t	a	c	a	c	a	a	t	g
DQ674250	1	g	a	a	c	c	t	c	a	t	c	t	a	c	c	c	t	c	a	t	a	t	t	a	g	t	a	c	a	c	a	a	t	g
KX262864	1	g	a	a	c	c	t	c	a	t	c	t	a	c	c	c	t	c	a	t	a	t	t	a	g	t	a	c	a	c	a	a	t	g
KX262865	1	g	a	a	c	c	t	c	a	t	c	t	a	c	c	c	t	c	a	t	a	t	t	a	g	t	a	c	a	c	a	a	t	g
KX262866	1	g	a	a	c	c	t	c	a	t	c	t	a	c	c	c	t	c	a	t	a	t	t	a	g	t	a	c	a	c	a	a	t	g
EU154348	1	g	a	a	c	c	t	c	a	t	c	t	a	c	c	c	t	c	a	t	a	t	t	a	g	t	a	c	a	c	a	a	t	g
JN704690	1	g	a	a	c	c	t	c	a	t	c	t	a	c	c	c	t	c	a	t	a	t	t	a	g	t	a	c	a	c	a	a	t	g
JN704691	1	g	a	a	c	c	t	c	a	t	c	t	a	c	c	c	t	c	a	t	a	t	t	a	g	t	a	c	a	c	g	a	t	g
JN704692	1	g	a	a	c	c	t	c	a	t	c	t	a	c	c	c	t	c	a	t	a	t	t	a	g	t	a	c	a	c	a	a	t	g
JN704693	1	g	a	a	c	c	t	c	a	t	c	t	a	c	c	c	t	c	a	t	a	t	t	a	g	t	a	c	a	c	a	a	t	g
JN704694	1	g	a	a	c	c	t	c	a	t	c	t	a	c	c	c	t	c	a	t	a	t	t	a	g	t	a	c	a	c	a	a	t	g
JN704695	1	g	a	a	c	c	t	c	a	t	c	t	a	c	c	c	t	c	a	t	a	t	t	a	g	t	a	c	a	c	a	a	t	g
JN704696	1	g	a	a	c	c	t	c	a	t	c	t	a	c	c	c	t	c	a	t	a	t	t	a	g	t	a	c	a	c	a	a	t	g
NC001348	1	g	a	a	c	c	t	c	a	t	c	t	a	c	c	c	t	c	a	t	a	t	t	a	g	t	a	c	a	c	a	a	t	g
KP771892	1	g	a	a	c	c	t	c	a	t	c	t	a	c	c	c	t	c	a	t	a	t	t	a	g	t	a	c	a	c	a	a	t	g
KP771894	1	g	a	a	c	c	t	c	a	t	c	t	a	c	c	c	t	c	a	t	a	t	t	a	g	t	a	c	a	c	a	a	t	g
KP771900	1	g	a	a	c	c	t	c	a	t	c	t	a	c	c	c	t	c	a	t	a	t	t	a	g	t	a	c	a	c	a	a	t	g
KP771901	1	g	a	a	c	c	t	c	a	t	c	t	a	c	c	c	t	c	a	t	a	t	t	a	g	t	a	c	a	c	a	a	t	g
KP771902	1	g	a	a	c	c	t	c	a	t	c	t	a	c	c	c	t	c	a	t	a	t	t	a	g	t	a	c	a	c	a	a	t	g
KP771908	1	g	a	a	c	c	t	c	a	t	c	t	a	c	c	c	t	c	a	t	a	t	t	a	g	t	a	c	a	c	a	a	t	g
KP771912	1	g	a	a	c	c	t	c	a	t	c	t	a	c	c	c	t	c	a	t	a	t	t	a	g	t	a	c	a	c	a	a	t	g
KP771913	1	g	a	a	c	c	t	c	a	t	c	t	a	c	c	c	t	c	a	t	a	t	t	a	g	t	a	c	a	c	a	a	t	g
KP771917	1	g	a	a	c	c	t	c	a	t	c	t	a	c	c	c	t	c	a	t	a	t	t	a	g	t	a	c	a	c	a	a	t	g
KP771918	1	g	a	a	c	c	t	c	a	t	c	t	a	c	c	c	t	c	a	t	a	t	t	a	g	t	a	c	a	c	a	a	t	g
KP771920	1	g	a	a	c	c	t	c	a	t	c	t	a	c	c	c	t	c	a	t	a	t	t	a	g	t	a	c	a	c	a	a	t	g
MH709361	1	g	a	a	c	c	t	c	a	t	c	t	a	c	c	t	t	c	a	t	a	t	t	a	g	t	a	c	a	c	a	a	t	g
KU926318	2	g	a	a	c	c	c	c	a	t	c	c	a	c	c	c	t	c	a	t	a	t	t	a	g	t	a	c	a	c	a	a	t	g
KU926319	2	g	a	a	c	c	t	c	a	t	c	t	a	c	c	c	t	c	a	t	a	t	t	a	g	t	a	c	a	c	a	a	t	g
KU926320	2	g	a	a	c	c	t	c	g	t	c	t	a	c	c	c	t	c	a	t	a	t	t	a	g	c	a	c	g	c	a	a	t	g
KU926321	2	g	a	a	c	c	t	c	a	t	c	t	a	c	c	c	t	c	a	t	a	t	t	a	g	t	a	c	a	c	a	a	t	g
KU926322	2	g	a	a	c	c	t	c	a	t	c	t	a	c	c	c	t	c	a	t	a	t	t	a	g	t	a	c	a	c	a	a	t	g
KC847290	2	g	a	a	c	c	t	c	a	t	c	t	a	c	c	c	t	c	a	t	a	t	t	a	g	t	a	c	a	c	a	a	t	g
KJ767491	2	g	a	a	c	c	t	c	a	t	c	t	a	c	c	c	t	c	a	t	a	t	t	a	g	t	a	c	a	c	a	a	t	g
KJ767492	2	g	a	a	c	c	t	c	a	t	c	t	a	c	c	c	t	c	a	t	a	t	t	a	g	t	a	c	a	c	a	a	t	g
KJ808816	2	g	a	a	c	c	t	c	a	t	c	t	a	c	c	c	t	c	a	t	a	t	t	a	g	t	a	c	a	c	a	a	t	g
KP771925	2	g	a	a	c	c	t	c	a	t	c	t	a	c	c	c	t	c	a	t	a	t	t	a	g	t	a	c	a	c	a	a	t	g
KU926315	2	g	a	a	c	c	t	c	a	t	c	t	a	c	c	c	t	c	a	t	a	t	t	a	g	t	a	c	a	c	a	a	t	g
KU926316	2	g	a	a	c	c	t	c	a	t	c	t	a	c	c	c	t	c	a	t	a	t	t	a	g	t	a	c	a	c	a	a	t	g
KU926317	2	g	a	a	c	c	t	c	a	t	c	t	a	c	c	c	t	c	a	t	a	t	t	a	g	t	a	c	a	c	a	a	t	g
KY037796	2	g	a	a	c	c	t	c	a	t	c	t	a	c	c	c	t	c	a	t	a	t	t	a	g	t	a	c	a	c	a	a	t	g
MF004348	2	g	a	a	c	c	t	c	a	t	c	t	a	c	c	c	t	c	a	t	a	t	t	a	g	t	a	c	a	c	a	a	t	g
MH709309	2	g	a	a	c	c	t	c	a	t	c	t	a	c	c	c	t	c	a	t	a	t	t	a	g	t	a	c	a	c	a	a	t	g
MH709310	2	g	a	a	c	c	t	c	a	t	c	t	a	c	c	c	t	c	a	t	a	t	t	a	g	t	a	c	a	c	a	a	t	g
MH709311	2	g	a	a	c	c	t	c	a	t	c	t	a	c	c	c	t	c	a	t	a	t	t	a	g	t	a	c	a	c	a	a	t	g
MH709313	2	g	a	a	c	c	t	c	a	t	c	t	a	c	c	c	t	c	a	t	a	t	t	a	g	t	a	c	a	c	a	a	t	g
MH709314	2	g	a	a	c	c	t	c	a	c	c	t	a	c	c	c	t	c	a	t	a	t	t	a	g	t	a	c	a	c	a	a	t	g
MH709315	2	g	a	a	c	c	t	c	a	t	c	t	a	c	c	c	t	c	a	t	a	t	t	a	g	t	a	c	a	c	a	a	t	g
MH709319	2	g	a	a	c	c	t	c	a	c	c	t	a	c	c	c	t	c	a	t	a	t	t	a	g	t	a	c	a	c	a	a	t	g
MH709320	2	g	a	a	c	c	t	c	a	t	c	t	a	c	c	c	t	c	a	t	a	t	t	a	g	t	a	c	a	c	a	a	t	g
MH709329	2	g	a	a	c	c	t	c	a	t	c	t	a	c	c	c	t	c	a	t	a	t	t	a	g	t	a	c	a	c	a	a	t	a
MH709331	2	g	a	a	c	c	t	c	a	t	c	t	a	c	c	c	t	c	a	t	a	t	t	a	g	t	a	c	a	c	a	a	t	g
MH709333	2	g	a	a	c	c	t	c	a	c	c	t	a	t	t	c	t	c	a	t	a	t	t	a	g	t	a	c	a	c	a	a	t	g
MH709337	2	g	a	a	c	c	t	c	a	t	c	t	a	c	c	c	t	c	a	t	a	t	t	a	g	t	a	c	a	c	a	a	t	g
MH709339	2	g	a	a	c	c	t	c	a	t	c	t	a	c	c	c	t	c	a	t	a	t	t	g	g	t	a	c	a	c	a	a	t	g
MH709344	2	g	a	a	c	c	t	c	a	t	c	t	a	c	c	c	t	c	a	t	a	t	t	a	g	t	a	c	a	c	a	a	t	g
MH709348	2	g	a	a	c	c	t	c	a	t	c	t	a	c	c	c	t	c	a	t	a	t	t	a	g	t	a	c	a	c	a	a	t	g
MH709355	2	g	a	a	c	c	t	c	a	t	c	t	a	c	c	c	t	c	a	t	a	t	t	a	g	t	a	c	a	c	a	a	t	g
MH709359	2	g	a	a	c	c	t	c	a	t	c	t	a	c	c	c	t	c	a	t	a	t	t	a	g	t	a	c	a	c	a	a	t	g
MH709363	2	g	a	a	c	c	t	c	a	t	c	t	a	c	c	c	t	c	a	t	a	t	t	a	g	t	a	c	a	c	a	a	t	g
MH709366	2	g	a	a	c	c	t	c	a	t	c	t	a	c	c	c	t	c	a	t	a	t	t	a	g	t	a	c	a	c	a	a	t	g
MH709376	2	g	a	a	c	c	t	c	a	t	c	t	a	c	c	c	t	c	a	t	a	t	t	a	g	t	a	c	a	c	a	a	t	g
MH709377	2	g	a	a	c	c	t	c	a	t	c	t	a	c	c	c	t	c	a	t	a	t	t	a	g	t	a	c	a	c	a	a	t	g
**MW316406**	2	g	a	a	c	c	t	c	a	t	c	t	a	c	c	c	t	c	a	t	a	t	t	a	g	t	a	c	a	c	a	a	t	g
AJ871403	3	g	a	a	c	c	t	c	a	t	c	t	a	c	c	c	t	c	a	t	a	t	t	a	g	t	g	c	a	c	a	a	t	g
DQ479955	3	g	a	a	c	c	t	c	a	t	c	t	a	c	c	c	t	c	a	t	a	t	t	a	g	t	g	c	a	c	a	a	t	g
DQ479956	3	g	a	a	c	c	t	c	a	t	c	t	a	c	c	c	t	c	a	t	a	t	t	a	g	t	g	c	a	c	a	a	t	g
DQ479957	3	g	a	a	c	c	t	c	a	t	c	t	a	c	c	c	t	c	a	t	a	t	t	a	g	t	g	c	a	c	a	a	t	g
JN704699	3	g	a	a	c	c	t	c	a	t	c	t	a	c	c	c	t	c	a	t	a	t	t	a	g	t	g	c	a	c	a	a	t	g
JN704700	3	g	a	a	c	c	t	c	a	t	c	t	a	c	c	c	t	c	a	t	a	t	t	a	g	t	g	c	a	c	a	a	t	g
JN704701	3	g	a	a	c	c	t	c	a	t	c	t	a	c	c	c	t	c	a	t	a	t	t	a	g	t	g	c	a	c	a	a	t	g
JN704702	3	g	a	a	c	t	t	c	a	t	c	t	a	c	c	c	t	c	a	t	a	t	t	a	g	t	g	c	a	c	a	a	t	g
JN704703	3	g	a	a	c	c	t	c	a	t	c	t	a	c	c	c	t	c	a	t	a	t	t	a	g	t	g	c	a	c	a	a	t	g
KP771891	3	g	a	a	c	c	t	c	a	t	c	t	a	c	c	c	t	c	a	t	a	t	t	a	g	t	g	c	a	c	a	a	t	g
KP771893	3	g	a	a	c	c	t	c	a	t	c	t	a	c	c	c	t	c	a	t	a	t	t	a	g	t	g	c	a	c	a	a	t	g
KP771895	3	g	a	a	c	c	t	c	a	t	c	t	a	c	c	c	t	c	a	t	a	t	t	a	g	t	g	c	a	c	a	a	t	g
KP771896	3	g	a	a	c	c	t	c	a	t	c	t	a	c	c	c	t	c	a	t	a	t	t	a	g	t	g	c	a	c	a	a	t	g
KP771897	3	g	a	a	c	c	t	c	a	t	c	t	a	c	c	c	t	c	a	t	a	t	t	a	g	t	g	t	a	c	a	a	t	g
KP771899	3	g	a	a	c	c	t	c	a	t	c	t	a	c	c	c	t	c	a	t	a	t	t	a	g	t	g	c	a	c	a	a	t	g
KP771903	3	g	a	a	c	c	t	c	a	t	c	t	a	c	c	c	t	c	a	t	a	t	t	a	g	t	g	c	a	c	a	a	t	g
KP771907	3	g	a	a	c	c	t	c	a	t	c	t	a	c	c	c	t	c	a	t	a	t	t	a	g	t	g	c	a	c	a	a	t	g
KP771910	3	g	a	a	c	c	t	t	a	t	c	t	a	c	c	c	t	c	a	t	a	t	t	a	g	t	g	c	a	c	a	a	t	g
KP771915	3	g	a	a	c	c	t	c	a	t	c	t	a	c	c	c	t	c	a	t	a	t	t	a	g	t	g	t	a	c	a	a	t	g
KP771916	3	g	a	a	c	c	t	c	a	t	c	t	a	c	c	c	t	c	a	t	a	t	t	a	g	t	g	c	a	c	a	a	t	g
KP771921	3	g	a	a	c	c	t	c	a	t	c	t	a	c	c	c	t	c	a	t	a	t	t	a	g	t	g	c	a	c	a	a	t	g
KP771923	3	g	a	a	c	c	t	c	a	t	c	t	a	c	c	c	t	c	a	t	a	t	t	a	g	t	g	c	a	c	a	a	t	g
KP771889	3	g	a	a	c	c	t	c	a	t	c	t	a	c	c	c	t	c	a	t	a	t	t	a	g	t	g	t	a	c	a	a	t	g
KU926311	3	g	a	a	c	c	t	c	a	t	c	t	a	c	c	c	t	c	a	c	a	t	t	a	g	t	g	c	a	c	a	a	t	g
JQ911599	3	g	a	a	c	c	t	c	a	t	c	t	a	c	c	c	t	c	a	t	a	t	t	a	g	t	g	c	a	c	a	a	t	g
MH709317	3	g	a	a	c	c	t	c	a	t	c	t	a	c	c	c	t	c	a	t	a	t	t	a	g	t	g	c	a	c	a	a	t	g
MH709346	3	g	a	a	c	c	t	c	a	t	c	t	a	c	c	c	t	c	a	t	a	t	t	a	g	t	g	c	a	c	a	a	t	g
MH709375	3	g	a	a	c	c	t	c	a	t	c	t	a	c	c	c	t	c	a	t	a	t	t	a	g	t	g	c	a	c	a	a	t	g
DQ452050	4	g	a	a	c	c	t	c	a	t	c	t	a	c	c	c	t	c	a	t	a	t	t	a	g	t	a	c	a	c	a	a	t	g
DQ479960	4	g	a	a	c	c	t	c	a	t	c	t	a	c	c	c	t	c	a	t	a	t	t	a	g	t	a	c	a	c	a	a	t	g
KP771898	4	g	a	a	c	c	t	c	a	t	c	t	a	c	c	c	t	c	a	t	a	t	t	a	g	t	a	c	a	c	a	a	t	g
MH709316	4	g	a	a	t	c	t	c	a	t	c	t	a	c	c	c	t	c	a	t	a	t	t	a	g	t	a	c	a	c	a	a	t	g
MH709318	4	g	a	a	c	c	t	c	a	t	c	t	a	c	c	c	t	c	a	t	a	t	t	a	g	t	a	c	a	c	a	c	t	g
MH709322	4	g	a	a	c	c	t	c	a	t	c	t	a	c	c	c	t	c	a	t	a	t	t	a	g	t	a	c	a	c	a	c	t	g
MH709323	4	g	a	a	c	c	t	c	a	t	c	t	a	c	c	c	t	c	a	t	a	t	t	a	g	t	a	c	a	c	a	a	t	g
MH709326	4	g	a	a	c	c	t	c	a	t	c	t	a	c	c	c	t	c	a	t	a	t	t	a	g	t	a	c	a	c	a	c	t	g
MH709341	4	g	a	a	c	c	t	c	a	t	c	t	a	c	c	c	t	c	a	t	a	t	t	a	g	t	a	c	a	c	a	a	c	g
MH709357	4	g	a	a	c	c	t	c	a	t	c	t	a	c	c	c	t	c	a	t	a	t	t	a	g	t	a	c	a	c	a	a	t	g
MH709364	4	g	a	a	c	c	t	c	a	t	c	t	a	c	c	c	t	c	a	t	a	t	t	a	g	t	a	c	a	c	a	a	t	g
MH709370	4	g	a	a	c	c	t	c	a	t	c	t	a	c	c	c	t	c	a	t	a	t	t	a	g	t	a	c	a	c	a	a	t	g
MH709371	4	g	a	a	c	c	t	c	a	t	c	t	a	c	c	c	t	c	a	t	a	t	t	a	g	t	a	c	a	c	a	a	t	g
MH709372	4	g	a	a	c	c	t	c	a	t	c	t	a	c	c	c	t	c	a	t	a	t	t	a	g	t	a	c	a	c	a	a	t	g
DQ457052	5	g	a	a	t	c	t	c	a	t	c	t	a	c	c	c	t	c	a	t	a	t	t	a	g	t	g	c	a	c	a	a	t	g
JN704704	5	g	a	a	t	c	t	c	a	t	c	t	a	c	c	c	t	c	a	t	a	t	t	a	g	t	g	c	a	c	a	a	t	g
JN704705	5	g	a	g	t	c	t	c	a	t	c	t	a	c	c	c	t	c	a	t	a	t	t	a	g	t	g	c	a	c	a	a	t	g
JN704706	5	g	a	a	t	c	t	c	a	t	c	t	a	c	c	c	t	c	a	t	a	t	t	a	g	t	g	c	a	c	a	a	t	g
JN704707	5	g	a	a	t	c	t	c	a	t	c	t	a	c	c	c	t	c	a	t	a	t	t	a	g	t	g	c	a	c	a	a	t	g
JN704708	5	g	a	a	c	c	t	c	a	t	c	t	a	c	c	c	t	c	a	t	a	t	t	a	g	t	g	c	a	c	a	a	t	g
KP771904	5	a	a	a	t	c	t	c	a	t	c	t	a	c	c	c	t	c	a	t	a	t	t	a	t	t	g	c	a	c	a	a	t	g
KP771905	5	g	a	a	t	c	t	c	a	t	c	t	a	c	c	c	t	c	a	t	a	t	t	a	g	t	g	c	a	c	a	a	t	g
KP771906	5	g	a	a	t	c	t	c	a	t	c	t	a	c	c	c	t	c	a	t	a	t	t	a	g	t	g	c	a	c	a	a	t	g
KP771919	5	g	a	a	t	c	t	c	a	t	t	t	a	c	c	c	t	c	a	t	a	t	t	a	g	t	g	c	a	c	a	a	t	g
KP771922	5	g	a	a	t	c	t	c	a	t	c	t	a	c	c	c	t	c	a	t	a	t	t	a	g	t	g	c	a	c	a	a	t	g
KP771924	5	g	a	a	t	c	t	c	a	t	c	t	a	c	c	c	t	c	a	t	a	t	t	a	g	t	g	c	a	c	a	a	t	g
KM355696	5	g	a	a	t	c	t	c	a	t	c	t	g	c	c	c	t	c	a	t	a	t	t	a	g	t	g	c	a	c	a	a	t	g
KM355697	5	g	a	a	t	c	t	c	a	t	c	t	g	c	c	c	t	c	a	t	a	t	t	a	g	t	g	c	a	c	a	a	t	g
KM355698	5	g	a	a	t	c	t	c	a	t	c	t	a	c	c	c	t	c	a	t	a	t	t	a	g	t	g	c	a	c	a	a	t	g
KM355699	5	g	a	a	t	c	t	c	a	t	c	t	a	c	c	c	t	c	a	t	a	t	t	a	g	t	g	c	a	c	a	a	t	g
KM355701	5	g	a	a	t	c	t	c	a	t	c	t	a	c	c	c	t	c	a	t	a	t	t	a	g	t	g	c	a	c	a	a	t	g
KM355702	5	g	a	a	t	c	t	c	a	t	c	t	a	c	c	c	t	c	a	t	a	t	t	a	g	t	g	c	a	c	a	a	t	g
KM355703	5	g	a	a	t	c	t	c	a	t	c	t	g	c	c	c	t	c	a	t	a	t	t	a	g	t	g	c	a	c	a	a	t	g
KM355704	5	g	a	a	t	c	t	c	a	t	c	t	g	c	c	c	t	c	a	t	a	t	t	a	g	t	g	c	a	c	a	a	t	g
KM355705	5	g	a	a	t	c	t	c	a	t	c	t	a	c	c	c	t	c	a	t	a	t	t	a	g	t	g	c	a	c	a	a	t	g
KM355706	5	g	a	a	t	c	t	c	a	t	c	t	a	c	c	c	t	c	a	t	a	t	t	a	g	t	g	c	a	c	a	a	t	g
KM355707	5	g	a	a	t	c	t	c	a	t	c	t	g	c	c	c	t	c	a	t	a	t	t	a	g	t	g	c	a	c	a	a	t	g
KM355708	5	g	a	a	t	c	t	c	a	t	c	t	g	c	c	c	t	c	a	t	a	t	t	a	g	t	g	c	a	c	a	a	t	g
KM355709	5	g	a	a	t	c	t	c	a	t	c	t	a	c	c	c	t	c	a	t	a	t	t	a	g	t	g	c	a	c	a	a	t	g
KM355711	5	g	a	a	t	c	t	c	a	t	c	t	a	c	c	c	t	c	a	t	a	t	t	a	g	t	g	c	a	c	a	a	t	g
KM355712	5	g	a	a	t	c	t	c	a	t	c	t	a	c	c	c	t	c	a	t	a	t	t	a	g	t	g	c	a	c	a	a	t	g
[illegible]	[illegible]	[illegible]	[illegible]	[illegible]	[illegible]	[illegible]	[illegible]	[illegible]	[illegible]	[illegible]	[illegible]	[illegible]	[illegible]	[illegible]	[illegible]	[illegible]	[illegible]	[illegible]	[illegible]	[illegible]	[illegible]	[illegible]	[illegible]	[illegible]	[illegible]	[illegible]	[illegible]	[illegible]	[illegible]	[illegible]	[illegible]	[illegible]	[illegible]	[illegible]

GB-ID																																									
	28788	28816	28836	28886	28895	28900	29019	29020	29066	29084	29093	29188	29201	29213	29273	29300	29358	29456	29509	29546	29568	29570	29592	29600	29684	29744	29750	29831	29957	29974	30161	30206	30270	30465	30477	30486	30506	30584	30638	30675	30688
AY548170	g	a	t	c	a	t	a	t	a	a	g	a	t	g	a	a	a	a	a	a	a	g	a	a	t	c	t	c	t	g	t	a	a	t	g	t	c	a	t	g	t
AY548171	g	a	c	c	a	t	a	t	a	a	g	a	t	g	a	a	a	a	a	a	a	g	a	a	t	c	t	c	t	g	t	a	a	t	g	t	c	a	t	g	t
DQ479953	g	a	t	c	a	t	a	t	a	a	g	a	t	g	a	a	a	a	a	a	a	g	a	a	t	c	t	c	t	g	t	a	a	t	g	t	c	a	t	g	t
DQ479954	g	a	t	c	a	t	a	t	a	a	g	a	t	g	a	a	a	a	a	a	a	g	a	a	t	c	t	c	t	g	t	a	a	t	g	t	c	a	t	g	t
DQ479958	g	a	c	c	a	t	a	t	a	a	g	a	t	g	a	a	a	a	a	a	a	g	a	a	t	c	t	c	t	g	t	a	a	t	g	t	c	a	t	g	t
DQ479959	g	a	c	c	a	t	a	t	a	a	g	a	t	g	a	a	a	a	a	a	a	g	a	a	t	c	t	c	t	g	t	a	a	t	g	t	c	a	t	g	t
DQ479961	g	a	t	c	a	t	a	t	a	a	g	a	t	g	a	a	a	a	a	a	a	g	a	a	t	c	t	c	t	g	t	a	a	t	g	t	c	a	t	g	t
DQ479962	g	a	t	c	a	t	a	t	a	a	g	a	t	g	a	a	a	a	a	a	a	g	a	a	t	c	t	c	t	g	t	a	a	t	g	t	c	a	t	g	t
DQ479963	g	a	t	c	a	t	a	t	a	a	g	a	t	g	a	a	a	a	a	a	a	g	a	a	t	c	t	c	t	g	t	a	a	t	g	t	c	a	t	g	t
DQ674250	g	a	t	c	a	t	a	t	a	a	g	a	t	g	a	a	a	a	a	a	a	g	a	a	t	c	t	c	t	g	t	a	a	t	g	t	c	a	t	g	t
KX262864	g	a	c	c	a	t	a	t	a	a	a	a	t	g	a	a	a	a	a	a	a	g	a	a	t	c	t	c	t	g	t	a	a	t	g	t	c	a	t	g	t
KX262865	g	a	t	c	a	t	a	t	a	c	g	a	t	g	a	a	a	a	a	a	a	g	a	a	t	c	t	c	t	g	t	a	a	t	g	t	c	a	t	g	t
KX262866	g	a	c	c	a	t	a	t	a	a	g	a	t	g	a	a	a	a	a	a	a	g	a	a	t	c	t	c	t	g	t	a	a	t	g	t	c	a	t	g	t
EU154348	g	a	t	c	a	t	a	t	a	a	g	a	t	g	a	a	a	a	a	a	a	g	a	a	t	c	t	c	t	g	t	a	a	t	g	t	c	a	t	g	t
JN704690	g	a	t	c	a	t	a	t	a	a	g	a	t	g	a	a	a	a	a	a	a	g	a	a	t	c	t	c	t	g	t	a	a	t	g	t	c	a	t	g	t
JN704691	g	a	t	c	a	t	a	t	a	a	g	a	t	g	a	a	a	a	a	a	a	g	a	a	t	c	t	c	t	g	t	a	a	t	g	t	c	a	t	g	t
JN704692	g	a	t	c	a	t	a	t	a	a	g	a	t	g	a	a	a	a	a	a	a	g	a	a	t	c	t	c	t	g	t	a	a	t	g	t	c	a	t	g	t
JN704693	g	a	t	c	a	t	a	t	a	a	g	a	t	g	a	a	a	a	a	a	a	g	a	a	t	c	t	c	t	g	t	a	a	t	g	t	c	a	t	g	t
JN704694	g	a	t	c	a	t	a	t	a	a	g	a	t	g	a	a	a	a	a	a	a	g	a	a	t	c	t	c	t	g	t	a	a	t	g	t	c	a	t	g	t
JN704695	g	a	t	c	a	t	a	t	a	a	g	a	t	g	a	a	a	a	a	a	a	g	a	a	t	c	t	c	t	g	t	a	a	t	g	t	c	a	t	g	t
JN704696	g	a	t	c	a	t	a	t	a	a	g	a	t	g	a	a	a	a	a	a	a	g	a	a	t	c	t	c	t	g	t	a	a	t	g	t	c	a	t	g	t
NC001348	g	a	t	c	a	t	a	t	a	a	g	a	t	g	a	a	a	a	a	a	a	g	a	a	t	c	t	c	t	g	t	a	a	t	g	t	c	a	t	g	t
KP771892	g	a	t	c	a	t	a	t	a	a	g	a	t	g	a	a	a	a	a	a	a	g	a	a	t	c	t	c	t	g	t	a	a	t	g	t	c	a	t	g	t
KP771894	g	a	t	c	a	t	a	t	a	a	g	a	t	g	a	a	a	a	a	a	a	g	a	a	t	c	t	c	t	g	t	a	a	t	g	t	c	a	t	g	t
KP771900	g	a	t	c	a	t	a	t	a	a	g	a	t	g	a	a	a	a	a	a	a	g	a	a	t	c	t	c	t	g	t	a	a	t	g	t	c	a	t	g	t
KP771901	g	a	t	c	a	t	a	t	a	a	g	a	t	g	a	a	a	a	a	a	a	g	a	a	t	c	t	c	t	g	t	a	a	t	g	t	c	a	t	g	t
KP771902	g	a	t	c	a	t	a	t	a	a	g	a	t	g	a	a	a	a	a	a	a	g	a	a	t	c	t	c	t	g	t	a	a	t	g	t	c	a	t	g	t
KP771908	g	a	c	c	a	t	a	t	a	a	g	a	t	g	a	a	a	a	a	a	a	g	a	a	t	c	t	c	t	g	t	a	a	t	g	t	c	a	t	g	t
KP771912	g	a	c	c	a	t	a	t	a	a	a	a	t	g	a	a	a	a	a	a	a	g	a	a	t	c	t	c	t	g	t	a	a	t	g	t	c	a	t	g	t
KP771913	g	a	c	c	a	t	a	t	a	a	a	a	t	g	a	a	a	a	a	a	a	g	a	a	t	c	t	c	t	g	t	a	a	t	g	t	c	a	t	g	t
KP771917	g	a	t	c	a	t	a	t	a	a	g	a	t	g	a	a	a	a	a	a	a	g	a	a	t	c	t	c	t	g	t	a	a	t	g	t	c	a	t	g	t
KP771918	g	a	t	c	a	t	a	t	a	a	g	a	t	g	a	a	a	a	a	a	a	g	a	a	t	c	t	c	t	g	t	a	a	t	g	t	c	a	t	g	t
KP771920	g	a	t	c	a	t	a	t	a	a	g	a	t	g	a	a	a	a	a	a	a	g	a	a	t	c	t	c	t	g	t	a	a	t	g	t	c	a	t	g	t
MH709361	g	a	c	c	a	t	a	t	a	a	g	a	t	g	a	a	a	a	a	a	a	g	a	a	t	c	t	c	t	g	t	a	a	t	g	t	c	a	t	g	t
KU926318	g	a	t	c	a	t	a	t	a	a	g	a	t	g	g	a	a	a	a	a	a	g	a	a	t	c	t	c	t	g	t	a	a	t	g	t	c	a	t	g	t
KU926319	g	a	t	c	a	t	a	t	a	a	g	a	t	g	a	a	a	a	a	a	a	g	a	a	t	c	t	c	t	g	t	a	a	t	g	t	c	a	t	g	t
KU926320	g	a	t	c	a	t	a	t	a	a	g	a	t	g	a	a	a	a	a	a	a	g	a	a	t	c	t	c	t	g	t	a	a	t	g	t	c	a	c	g	t
KU926321	g	a	t	c	a	t	a	t	a	a	g	a	t	g	a	a	a	a	a	a	a	g	a	a	t	c	t	c	t	g	t	a	a	t	g	t	c	a	t	g	t
KU926322	g	a	t	c	a	t	a	t	a	a	g	a	t	g	a	a	a	a	a	a	a	g	a	a	t	c	t	c	t	g	t	a	a	t	g	t	c	a	t	g	t
KC847290	g	a	t	c	a	t	a	t	a	a	g	a	t	g	a	a	a	a	a	a	a	g	a	a	t	c	t	c	t	g	t	a	a	t	g	t	c	a	t	g	t
KJ767491	g	a	t	c	a	t	a	t	a	a	g	a	t	g	a	a	a	a	a	a	a	g	a	a	t	c	t	c	t	g	t	a	a	t	g	t	c	a	t	g	t
KJ767492	g	a	t	c	a	t	a	t	a	a	g	a	t	g	a	a	a	a	a	a	a	g	a	a	t	c	t	c	t	g	t	a	a	t	g	t	c	a	t	g	t
KJ808816	g	a	t	c	a	t	a	t	a	a	g	a	t	g	a	a	a	a	a	a	a	g	a	a	t	c	t	c	t	g	t	a	a	t	g	t	c	a	t	g	t
KP771925	g	a	t	c	a	t	a	t	a	a	g	a	t	g	a	a	a	a	a	a	a	g	a	a	t	c	t	c	t	g	t	a	a	t	g	t	c	a	t	g	t
KU926315	g	a	t	c	a	t	a	t	a	a	g	a	t	g	a	a	a	a	a	a	a	g	a	a	t	c	t	c	t	g	t	a	a	t	g	t	c	a	t	g	t
KU926316	g	a	t	c	a	t	a	t	a	a	g	a	t	g	a	a	a	a	a	a	a	g	a	a	t	c	t	c	t	g	t	a	a	t	g	t	c	a	t	g	t
KU926317	g	a	t	c	a	t	a	t	a	a	g	a	t	g	a	a	a	a	a	a	a	g	a	a	t	c	t	c	t	g	t	a	a	t	g	t	c	a	t	g	t
KY037796	g	a	t	c	a	t	a	t	a	a	g	a	t	g	a	a	a	a	a	a	a	g	a	a	t	c	t	c	t	g	t	a	a	t	g	t	c	a	t	g	t
MF004348	g	a	t	c	a	t	a	t	a	a	g	a	t	g	a	a	a	a	a	a	a	g	a	a	t	c	t	c	t	g	t	a	a	t	g	t	c	a	t	g	t
MH709309	g	a	t	c	a	t	a	t	a	a	g	a	t	g	a	a	a	a	a	a	a	g	a	a	t	c	t	c	t	g	t	a	a	t	g	t	c	a	t	g	t
MH709310	g	a	t	c	a	t	a	t	a	a	g	a	t	g	a	a	g	a	a	a	a	g	a	a	t	c	t	c	t	g	t	a	a	t	g	t	c	a	t	g	t
MH709311	g	a	t	c	t	t	a	t	a	a	g	a	t	g	a	a	a	a	a	a	a	g	a	a	t	c	t	c	t	g	t	a	a	t	g	t	c	a	t	g	t
MH709313	g	a	t	c	a	t	a	t	a	a	g	a	t	g	a	a	a	a	a	a	a	g	a	a	t	c	t	c	t	g	t	a	a	t	g	t	c	a	t	g	t
MH709314	g	a	t	c	a	t	a	t	a	a	g	a	g	g	a	a	a	a	a	a	a	g	a	a	t	c	t	c	t	g	t	a	a	t	g	t	c	a	t	g	t
MH709315	g	a	t	c	a	t	a	t	a	a	g	a	g	g	a	a	a	a	a	a	a	g	a	a	t	c	t	c	t	g	t	a	a	t	g	t	c	a	t	g	t
MH709319	g	a	t	c	a	t	a	t	a	a	g	a	g	g	a	a	a	a	a	a	a	g	a	a	t	c	t	c	t	g	t	a	a	t	g	t	c	a	t	g	t
MH709320	g	a	t	c	a	t	a	t	a	a	g	a	t	g	a	a	a	a	a	a	a	g	a	a	t	c	t	c	t	g	t	a	a	t	g	t	c	a	t	g	t
MH709329	g	a	t	c	a	t	a	t	a	a	g	a	g	g	a	a	a	a	a	a	a	g	a	a	t	c	t	c	t	g	t	a	a	t	g	t	c	a	t	g	t
MH709331	g	a	t	c	a	t	a	t	a	a	g	a	t	g	a	a	a	a	a	a	a	g	a	a	t	c	t	c	t	g	t	a	a	t	g	t	c	a	t	g	t
MH709333	g	a	t	c	a	t	a	t	a	a	g	a	g	g	a	a	a	a	a	a	a	g	a	a	t	c	t	c	t	g	t	a	a	t	g	t	c	a	t	g	t
MH709337	g	a	t	c	a	t	a	t	a	a	g	a	t	g	a	a	a	a	a	a	a	g	a	a	t	c	t	c	t	g	t	a	a	t	g	t	c	a	t	g	t
MH709339	g	a	t	c	a	t	a	t	a	a	g	a	t	g	a	a	a	a	a	a	a	g	a	a	t	c	t	c	t	g	t	a	a	t	g	t	c	a	t	g	t
MH709344	g	a	t	c	a	t	a	t	a	a	g	a	t	g	a	a	a	a	a	a	a	g	a	a	t	c	t	c	t	g	t	a	a	t	g	t	c	a	t	g	t
MH709348	g	a	t	c	a	t	a	t	a	a	g	a	t	g	a	a	a	a	a	a	a	g	a	a	t	c	t	c	t	g	t	a	a	t	g	t	c	a	t	g	t
MH709355	g	a	t	c	a	t	a	t	a	a	g	a	t	g	a	a	a	a	a	a	a	g	a	a	t	c	t	c	t	g	t	a	a	t	g	t	c	a	t	g	t
MH709359	g	a	t	c	a	t	a	t	a	a	g	a	t	g	a	a	a	a	a	a	a	g	a	a	t	c	t	c	t	g	t	a	a	t	g	t	c	a	t	g	t
MH709363	g	a	t	c	a	t	a	t	a	a	g	a	t	g	a	a	a	a	a	a	a	g	a	a	t	c	t	c	t	g	t	a	a	t	g	t	c	a	t	g	t
MH709366	g	a	t	c	a	t	a	t	a	a	g	a	t	g	a	a	a	a	a	a	a	g	a	a	t	c	t	c	t	g	t	a	a	t	g	t	c	a	t	g	t
MH709376	g	a	t	c	a	t	a	t	a	a	g	a	t	g	a	a	a	a	a	a	a	g	a	a	t	c	t	c	t	g	t	a	a	t	g	t	c	a	t	g	t
MH709377	g	a	t	c	a	t	a	t	a	a	g	a	t	g	a	a	a	a	a	a	a	g	a	a	t	c	t	c	t	g	t	a	a	t	g	t	c	a	t	g	t
**MW316406**	g	a	t	c	a	t	a	t	a	a	g	a	t	g	a	a	a	a	a	a	a	g	a	a	t	c	t	c	t	g	t	a	a	t	g	t	c	a	t	g	t
AJ871403	g	a	t	c	a	t	a	t	a	a	g	a	t	g	a	a	a	a	a	a	a	g	a	a	t	c	t	t	t	t	t	g	a	t	g	g	c	a	t	g	t
DQ479955	g	a	t	c	a	t	a	t	a	a	g	a	t	g	a	a	a	a	a	a	a	g	a	a	t	c	t	t	t	t	t	g	a	t	g	g	c	a	t	g	t
DQ479956	g	a	t	c	a	t	a	t	a	a	g	a	t	g	a	a	a	a	a	a	a	g	a	a	t	c	t	t	t	t	t	g	a	t	g	g	c	a	t	g	t
DQ479957	g	a	t	c	a	t	a	t	a	a	g	a	t	g	a	a	a	a	a	a	a	g	a	a	t	c	t	t	t	t	t	g	a	t	g	g	c	a	t	g	t
JN704699	g	g	t	c	a	t	a	t	a	a	g	a	t	g	a	a	a	a	a	a	a	g	a	a	t	c	t	t	t	t	t	g	a	t	g	g	c	a	t	g	t
JN704700	g	a	t	c	a	t	a	t	a	a	g	a	t	g	a	a	a	a	a	a	a	g	a	g	t	c	t	t	t	t	t	g	a	t	g	g	c	a	t	g	t
JN704701	g	a	t	c	a	t	a	t	c	a	g	a	t	g	a	a	a	a	a	a	a	g	a	a	t	c	t	t	t	t	t	g	a	t	g	g	c	a	t	g	t
JN704702	g	a	t	c	a	t	a	t	a	a	g	a	t	g	a	a	a	a	a	a	a	g	a	g	t	c	t	t	t	t	t	g	a	t	g	g	c	a	t	g	t
JN704703	g	a	t	c	a	t	a	t	a	a	g	a	t	g	a	a	a	a	a	a	a	g	a	g	t	c	t	t	t	t	t	g	a	t	g	g	c	a	t	g	t
KP771891	g	a	t	c	a	t	a	t	a	a	g	a	t	g	a	a	a	a	a	a	a	g	a	a	t	c	t	t	t	t	t	g	a	t	g	g	c	a	t	g	t
KP771893	g	a	t	c	a	t	a	t	a	a	g	a	t	g	a	a	a	a	a	a	a	g	a	a	t	c	t	t	t	t	t	g	a	t	g	g	c	a	t	g	t
KP771895	g	a	t	c	a	t	a	t	a	a	g	a	t	g	a	a	a	a	a	a	a	g	a	a	t	c	t	t	t	t	t	g	a	t	g	g	c	a	t	g	t
KP771896	g	a	t	c	a	t	a	t	a	a	g	a	t	g	a	a	a	a	a	a	a	g	a	a	t	c	t	t	t	t	t	g	a	t	g	g	c	a	t	g	t
KP771897	g	a	t	c	a	t	a	t	a	a	g	a	t	g	a	a	a	a	a	a	a	g	a	a	t	c	t	t	t	t	t	g	a	t	g	g	c	a	t	g	t
KP771899	g	a	t	c	a	t	a	t	a	a	g	a	t	g	a	a	a	a	a	a	a	g	a	a	t	c	t	t	t	t	t	g	a	t	g	g	c	a	t	g	t
KP771903	g	a	t	c	a	t	a	t	a	a	g	a	t	g	a	a	a	a	a	a	a	g	a	a	t	c	t	t	t	t	t	g	a	t	g	g	c	a	t	g	t
KP771907	g	a	t	c	a	t	a	t	a	a	g	a	t	g	a	a	a	a	a	a	a	g	a	a	t	c	t	t	t	t	t	g	a	t	g	g	c	a	t	g	t
KP771910	g	a	t	c	a	t	a	t	a	a	g	a	t	g	a	a	a	a	a	a	a	g	a	a	t	c	t	t	t	t	t	g	a	t	g	g	c	a	t	g	t
KP771915	g	a	t	c	a	t	a	t	a	a	g	a	t	g	a	a	a	a	a	a	a	g	a	a	t	c	t	t	t	t	t	g	a	t	g	g	c	a	t	g	t
KP771916	g	a	t	c	a	t	a	t	a	a	g	a	t	g	a	a	a	a	a	a	a	g	a	a	t	c	t	t	t	t	t	g	a	t	g	g	c	a	t	g	t
KP771921	g	a	t	c	a	t	a	t	a	a	g	a	t	g	a	a	a	a	a	a	a	g	a	a	t	c	t	t	t	t	t	g	a	t	g	g	c	a	t	g	t
KP771923	g	a	t	c	a	t	a	t	a	a	g	a	t	g	a	a	a	a	a	a	a	g	a	a	t	c	t	t	g	t	t	g	a	t	g	g	c	a	t	g	t
KP771889	g	a	t	c	a	t	a	t	a	a	g	a	t	g	a	a	a	a	a	a	a	g	a	a	t	c	t	t	t	t	t	g	a	t	g	t	c	a	t	g	t
KU926311	g	a	t	c	a	t	a	t	a	a	g	a	t	g	a	a	a	a	a	a	a	g	a	a	t	c	t	t	t	t	t	g	a	t	g	g	c	g	t	g	t
JQ911599	g	a	t	c	a	t	a	t	a	a	g	a	t	g	a	a	a	a	a	a	a	g	a	g	t	c	t	t	t	t	t	g	a	t	g	g	c	a	t	g	t
MH709317	g	a	t	c	a	t	a	t	a	a	g	a	t	g	a	a	a	a	a	a	a	g	a	a	t	c	t	t	t	t	t	g	a	t	g	g	c	a	t	g	t
MH709346	g	a	t	c	a	t	a	t	a	a	g	a	t	g	a	a	a	a	a	a	a	g	a	a	t	c	t	t	t	t	t	g	a	t	g	g	c	a	t	g	t
MH709375	g	a	t	c	a	t	a	t	a	a	g	a	t	g	a	a	a	a	a	a	a	g	a	a	t	c	t	t	t	t	t	g	a	t	t	g	c	a	t	g	t
DQ452050	g	a	t	c	a	t	a	t	a	a	g	g	t	g	a	a	a	a	a	a	a	g	a	a	t	c	t	c	t	g	t	a	a	t	g	t	c	a	t	g	t
DQ479960	g	a	t	c	a	t	a	t	a	a	g	a	t	g	a	a	a	a	a	a	a	g	a	a	t	c	t	c	t	g	t	a	a	t	g	t	c	a	t	g	t
KP771898	g	a	t	c	a	t	a	t	a	a	g	g	t	g	a	a	a	a	a	a	a	g	a	a	t	c	t	c	t	g	t	a	a	t	g	t	c	a	t	g	t
MH709316	g	a	t	c	a	t	a	t	a	a	g	g	t	g	a	a	a	a	a	a	a	g	a	a	t	c	t	c	t	g	t	a	a	t	g	t	c	a	t	g	t
MH709318	g	a	t	c	a	t	a	t	a	a	g	a	t	g	a	a	a	a	a	a	a	g	a	a	t	c	t	c	t	g	t	a	a	t	g	t	c	a	t	g	t
MH709322	g	a	t	c	a	t	a	t	a	a	g	a	t	g	a	a	a	a	a	a	a	g	a	a	t	c	t	c	t	g	t	a	a	t	g	t	c	a	t	g	t
MH709323	g	a	t	c	a	t	a	t	a	a	g	a	t	g	a	a	a	a	a	a	a	g	a	a	t	c	t	c	t	g	t	a	a	t	g	t	c	a	t	g	t
MH709326	g	a	t	c	a	t	a	t	a	a	g	g	t	g	a	g	a	a	a	a	a	g	a	a	t	c	t	c	t	g	t	a	a	t	g	t	c	a	t	g	t
MH709341	g	a	t	c	a	t	a	t	a	a	g	a	t	g	a	a	a	a	a	a	a	g	a	a	t	c	t	c	t	g	t	a	a	t	g	t	c	a	t	g	t
MH709357	g	a	t	c	a	t	a	t	a	a	g	a	t	g	a	a	a	a	a	a	a	g	a	a	t	c	t	c	t	g	t	a	a	t	g	t	c	a	t	g	t
MH709364	g	a	t	c	a	t	a	t	a	a	g	a	t	g	a	a	a	a	a	a	a	g	a	a	t	c	t	c	t	g	t	a	a	t	g	t	c	a	t	g	t
MH709370	g	a	t	c	a	t	a	t	a	a	g	a	t	g	a	a	a	a	a	a	a	g	a	a	t	c	t	c	t	g	t	a	a	t	g	t	c	a	t	g	t
MH709371	g	a	t	c	a	t	a	t	a	a	g	a	t	g	a	a	a	a	a	a	a	g	a	a	t	c	t	c	t	g	t	a	a	t	g	t	c	a	t	g	t
MH709372	g	a	t	c	a	t	a	t	a	a	g	a	t	g	a	a	a	a	a	a	a	g	a	a	t	c	t	c	t	g	t	a	a	t	g	t	c	a	t	g	t
DQ457052	g	a	t	c	a	t	a	t	a	a	g	a	t	g	a	a	a	a	a	a	a	g	a	a	t	c	t	t	t	t	t	g	a	t	g	g	c	a	t	g	t
JN704704	g	a	t	c	a	t	a	t	a	a	g	a	t	g	a	a	a	a	a	a	a	g	a	a	t	c	t	t	t	t	t	g	a	t	g	g	c	a	t	g	t
JN704705	g	a	t	c	a	t	a	g	a	a	g	a	t	g	a	a	a	a	a	g	a	g	a	a	t	c	t	t	t	t	t	g	c	t	g	g	c	a	t	g	t
JN704706	g	a	t	c	a	c	a	t	a	a	g	a	t	g	a	a	a	a	a	a	a	g	a	a	g	c	t	t	t	t	t	g	c	t	g	g	c	a	t	g	t
JN704707	g	a	t	c	a	t	a	t	a	a	g	a	t	g	a	a	a	a	a	a	a	g	a	a	t	c	t	t	t	t	t	g	a	t	g	g	c	a	t	g	t
JN704708	g	a	t	c	a	t	a	g	a	a	g	a	t	g	a	a	a	g	a	a	a	g	a	a	t	c	t	t	t	t	t	g	c	t	g	g	c	a	t	g	t
KP771904	g	a	t	c	a	t	a	t	a	a	g	a	t	g	a	a	a	a	a	a	a	g	a	a	t	c	t	t	t	t	t	g	a	t	g	g	c	a	t	g	t
KP771905	g	a	t	t	a	t	g	t	a	a	g	a	t	g	a	a	a	a	a	a	a	g	a	a	t	c	t	t	t	t	t	g	a	t	g	g	c	a	t	a	t
KP771906	g	a	t	t	a	t	g	t	a	a	g	a	t	g	a	a	a	a	a	a	a	g	a	a	t	c	t	t	t	t	t	g	a	t	g	g	c	a	t	a	t
KP771919	g	a	t	c	a	t	a	t	a	a	g	a	t	g	a	a	a	a	a	a	a	g	a	a	t	c	t	t	t	t	t	g	a	t	g	g	c	a	t	g	t
KP771922	g	a	t	c	a	t	a	t	a	a	g	a	t	g	a	a	a	a	a	a	a	g	a	a	t	c	t	t	t	t	t	g	a	t	g	g	c	a	t	g	t
KP771924	g	a	t	c	a	t	a	t	a	a	g	a	t	g	a	a	a	a	a	g	a	g	a	a	t	c	t	t	t	t	t	g	c	t	g	g	c	a	t	g	t
KM355696	a	a	t	c	a	t	a	t	a	a	g	a	t	g	a	a	a	a	a	g	a	a	a	a	t	t	t	t	t	t	t	g	c	t	g	g	c	a	t	g	t
KM355697	a	a	t	c	a	t	a	t	a	a	g	a	t	g	a	a	a	a	a	g	a	a	a	a	t	t	t	t	t	t	t	g	c	t	g	g	c	a	t	g	t
KM355698	g	a	t	c	a	t	a	t	a	a	g	a	t	g	a	a	a	a	a	g	a	g	a	a	t	c	t	t	t	t	t	g	c	t	g	g	c	a	t	g	t
KM355699	g	a	t	c	a	t	a	t	a	a	g	a	t	g	a	a	a	a	a	g	a	g	a	a	t	c	t	t	t	t	t	g	c	t	g	g	c	a	t	g	t
KM355701	a	a	t	c	a	t	a	t	a	a	g	a	t	g	a	a	a	a	a	g	a	a	a	a	t	t	t	t	t	t	t	g	c	g	g	g	c	a	t	g	t
KM355702	g	a	t	c	a	t	a	t	a	a	g	a	t	g	a	a	a	a	a	a	a	g	a	a	t	c	t	t	t	t	t	g	c	t	g	g	c	a	t	g	t
KM355703	a	a	t	c	a	t	a	t	a	a	g	a	t	g	a	a	a	a	a	g	a	a	a	a	t	t	t	t	t	t	t	g	c	t	g	g	c	a	t	g	t
KM355704	a	a	t	c	a	t	a	t	a	a	g	a	t	g	a	a	a	a	a	g	a	a	a	a	t	t	t	t	t	t	t	g	c	t	g	g	c	a	t	g	t
KM355705	g	a	t	c	a	t	a	t	a	a	g	a	t	g	a	a	a	a	a	a	a	g	a	a	t	c	t	t	t	t	t	g	c	t	g	g	c	a	t	g	t
KM355706	g	a	t	c	a	t	a	t	a	a	g	a	t	g	a	a	a	a	a	a	a	g	a	a	t	c	t	t	t	t	t	g	c	t	g	g	c	a	t	g	t
KM355707	a	a	t	c	a	t	a	t	a	a	g	a	t	g	a	a	a	a	a	g	a	a	a	a	t	t	t	t	t	t	t	g	c	t	g	g	c	a	t	g	t
KM355708	a	a	t	c	a	t	a	t	a	a	g	a	t	g	a	a	a	a	a	g	a	a	a	a	t	t	t	t	t	t	t	g	c	t	g	g	c	a	t	g	t
KM355709	g	a	t	c	a	t	a	t	a	a	g	a	t	g	a	a	a	a	a	g	a	g	a	a	t	c	t	t	t	t	t	g	c	t	g	g	c	a	t	g	t
KM355711	g	a	t	c	a	t	a	t	a	a	g	a	t	g	a	a	a	a	a	g	a	g	a	a	t	c	t	t	t	t	t	g	c	t	g	g	c	a	t	g	t
KM355712	g	a	t	c	a	t	a	t	a	a	g	a	t	g	a	a	a	a	a	g	a	g	a	a	t	c	t	t	t	t	t	g	c	t	g	g	c	a	t	g	t
[illegible]	[illegible]	[illegible]	[illegible]	[illegible]	[illegible]	[illegible]	[illegible]	[illegible]	[illegible]	[illegible]	[illegible]	[illegible]	[illegible]	[illegible]	[illegible]	[illegible]	[illegible]	[illegible]	[illegible]	[illegible]	[illegible]	[illegible]	[illegible]	[illegible]	[illegible]	[illegible]	[illegible]	[illegible]	[illegible]	[illegible]	[illegible]	[illegible]	[illegible]	[illegible]	[illegible]	[illegible]	[illegible]	[illegible]	[illegible]	[illegible]	[illegible]

Supplementary Table 3: 2880 SN

GB-ID	Clade markers																																				
	Position in Dumas	25895	26125	26296	26572	26821	26949	27258	27259	27286	27291	27343	27394	27405	27406	27435	27469	27503	27523	27634	27797	27844	27868	27922	27942	28042	28111	28167	28168	28291	28486	28524	28602	28642	28788	28816	28836
MH499467	5	g	a	a	t	c	t	c	a	t	c	t	a	c	c	c	t	c	a	t	t	t	t	a	g	t	g	c	a	c	a	a	t	g	g	a	t
MH499469	5	g	a	a	t	c	t	c	a	t	c	t	a	c	c	c	t	c	a	t	a	t	t	a	g	t	g	c	a	c	a	a	t	g	g	a	t
MH709321	5	g	a	a	t	c	t	c	a	t	c	t	a	c	c	c	t	c	a	t	a	c	t	a	g	t	g	c	a	c	a	a	t	g	g	a	t
MH709324	5	g	a	a	t	c	t	c	a	t	c	t	a	c	c	c	t	g	a	t	a	t	t	a	g	t	g	c	a	c	a	a	t	g	g	a	t
MH709325	5	g	a	a	t	c	t	c	a	t	c	t	a	c	c	c	t	c	a	t	a	t	t	a	g	t	g	c	a	c	a	a	t	g	g	a	t
MH709327	5	g	a	a	t	c	t	c	a	t	c	t	a	c	c	c	t	c	a	t	a	t	t	a	g	t	g	c	a	c	a	a	t	g	g	a	t
MH709328	5	g	a	a	t	c	t	c	a	t	c	t	a	c	c	c	t	c	a	t	a	t	t	a	g	t	g	c	a	c	a	a	t	g	g	a	t
MH709330	5	g	a	a	t	c	t	c	a	t	c	t	a	c	c	c	t	c	a	t	a	t	t	a	g	t	g	c	a	c	a	a	t	g	g	a	t
MH709332	5	g	a	a	t	c	t	c	a	t	c	t	a	c	c	c	c	c	a	t	a	t	t	a	g	t	g	c	a	c	a	a	t	g	g	a	t
MH709334	5	g	a	a	t	c	t	c	a	t	c	t	a	c	c	c	t	c	a	t	a	t	t	a	g	t	g	c	a	c	a	a	t	g	g	a	t
MH709335	5	g	a	a	t	c	t	c	a	t	c	t	a	c	c	c	t	c	a	t	a	t	t	a	g	t	g	c	a	c	a	a	t	g	g	a	t
MH709336	5	g	a	a	t	c	t	c	a	t	c	t	a	c	c	c	t	c	a	t	a	t	t	a	g	t	g	c	a	c	a	a	t	g	g	a	t
MH709338	5	g	a	a	t	c	t	c	a	t	c	t	a	c	c	c	t	c	a	t	a	t	t	a	g	t	g	c	a	c	a	a	t	g	g	a	t
MH709340	5	g	a	a	t	c	t	c	a	t	c	t	a	c	c	c	t	c	a	t	a	t	t	a	g	t	g	c	a	c	a	a	t	g	g	a	t
MH709342	5	g	a	a	t	c	t	c	a	t	c	t	a	c	c	c	t	c	a	t	a	c	t	a	g	t	g	c	a	c	a	a	t	g	g	a	t
MH709343	5	g	a	a	t	c	t	c	a	t	c	t	a	c	c	c	t	c	a	t	a	t	t	a	g	t	g	c	a	c	a	a	t	g	g	a	t
MH709345	5	g	a	a	t	c	t	c	a	t	c	t	a	c	c	c	t	c	a	t	a	t	t	a	g	t	g	c	a	c	a	a	t	g	g	a	t
MH709347	5	g	a	a	t	c	t	c	a	t	c	t	a	c	c	c	t	c	a	t	a	t	t	a	g	t	g	c	a	c	a	a	t	g	g	a	t
MH709349	5	g	a	a	t	c	t	c	a	t	c	t	a	c	c	c	t	c	a	t	a	t	t	a	g	t	g	c	a	c	a	a	t	g	g	a	t
MH709350	5	g	a	a	t	c	t	c	a	t	c	t	a	c	c	c	t	c	a	t	a	t	t	a	g	t	g	c	a	c	a	a	t	g	g	a	t
MH709351	5	g	a	a	t	c	t	c	a	t	c	t	a	c	c	c	t	c	a	t	a	t	t	a	g	t	g	c	a	c	a	a	t	g	g	a	t
MH709353	5	g	a	a	t	c	t	c	a	t	c	t	a	c	c	c	t	c	a	t	a	t	t	a	g	t	g	c	a	c	a	a	t	g	g	a	t
MH709358	5	g	a	a	t	c	t	c	a	t	c	t	a	c	c	c	t	c	a	t	a	t	t	a	g	t	g	c	a	c	a	a	t	g	g	a	t
MH709360	5	g	a	a	t	c	t	c	a	t	c	t	a	c	c	c	t	c	a	t	a	t	t	a	g	t	g	c	a	c	a	a	t	g	g	a	t
MH709362	5	g	a	a	t	c	t	c	a	t	c	t	a	c	c	c	t	c	a	t	a	t	t	a	g	t	g	c	a	c	a	a	t	g	g	a	t
MH709365	5	g	a	a	t	c	t	c	a	t	c	t	a	c	c	c	t	c	a	t	a	t	t	a	g	t	g	c	a	c	a	a	t	g	g	a	t
MH709373	5	g	a	a	t	c	t	c	a	t	c	t	a	c	c	c	t	c	a	t	a	t	t	a	g	t	g	c	a	c	a	a	t	g	g	a	t
MH709374	5	g	a	a	t	c	t	c	a	t	c	t	a	c	c	c	t	c	a	t	a	t	t	a	g	t	g	c	a	c	a	a	t	g	g	a	t
KC112914	6	g	a	a	c	c	t	c	a	t	c	t	a	c	c	c	t	c	a	t	a	t	t	a	g	t	g	c	a	c	a	a	t	g	g	a	t
KP771914	6	g	a	a	c	c	t	c	a	t	c	t	a	c	c	c	t	c	a	t	a	t	t	a	g	t	g	c	a	c	a	a	t	g	g	a	t
KP702725	6	g	a	a	c	c	t	c	a	t	c	t	a	c	c	c	t	c	a	t	a	t	t	a	g	t	g	c	a	c	a	a	t	g	g	a	t
KP771890	9	g	a	a	c	c	t	c	a	t	c	t	a	c	c	c	t	c	a	t	a	t	t	a	g	t	a	c	a	c	a	a	t	g	g	a	t
JN704710	9	g	a	a	c	c	t	c	a	t	c	t	a	c	c	c	t	c	a	t	a	t	t	a	g	t	a	c	a	c	a	a	t	g	g	a	t
KY037798	9	g	a	a	c	c	t	c	a	t	c	c	a	c	c	c	t	c	a	t	a	t	t	a	g	t	a	c	a	c	a	a	t	g	g	a	t
MH709312	9	g	a	a	c	c	t	c	a	t	c	c	a	c	c	c	t	c	a	t	a	t	t	a	g	t	a	c	a	c	a	a	t	g	g	a	t
MH709352	9	g	a	a	c	c	t	c	a	t	c	t	a	c	c	c	t	c	a	t	a	t	t	a	g	t	a	c	a	c	a	a	t	g	g	a	t
AB097933	Vac	g	a	a	c	c	t	c	a	t	c	t	a	c	c	c	t	c	g	t	a	t	t	a	g	t	a	c	a	c	a	a	t	g	g	a	t
JN704698	Vac	g	a	a	c	c	t	c	a	t	c	t	a	c	c	c	t	c	g	t	a	t	t	a	g	t	a	c	a	c	a	a	t	g	g	a	t
JN704697	Vac	g	g	a	c	c	t	c	a	t	c	t	a	c	c	c	t	c	g	t	a	t	t	a	g	t	a	c	a	c	a	a	t	g	g	a	t
DQ008355	Vac	g	r	a	c	c	t	c	a	t	c	t	a	c	c	c	t	c	g	t	a	t	t	a	g	t	a	c	a	c	a	a	t	g	g	a	t
JF306641	Vac	g	g	a	c	c	t	c	a	t	c	t	a	c	c	c	t	c	g	t	a	t	t	a	g	t	a	c	a	c	a	a	t	g	g	a	t
AB097932	Vac	g	g	a	c	c	t	c	a	t	c	t	a	c	c	c	t	c	g	t	a	t	t	a	g	t	a	c	a	c	a	a	t	g	g	a	t
DQ008354	Vac	g	g	a	c	c	t	c	a	t	c	t	a	c	c	c	t	c	g	t	a	t	t	a	g	t	a	c	a	c	a	a	t	g	g	a	t
KU926312	Vac	g	g	a	c	c	t	c	a	t	c	t	a	c	c	c	t	c	g	t	a	t	t	a	g	t	a	c	a	c	a	a	t	g	g	a	t
KU926313	Vac	g	g	a	c	c	t	c	a	t	c	t	a	c	c	c	t	c	g	t	a	t	t	a	g	t	a	c	a	c	a	a	t	g	g	a	t
KU926314	Vac	g	a	a	c	c	t	c	a	t	c	t	a	c	c	c	t	c	g	t	a	t	t	a	g	t	a	c	a	c	a	a	t	g	g	a	t
KF558371	Vac	g	g	a	c	c	t	c	a	t	c	t	a	c	c	c	t	c	g	t	a	t	t	a	g	t	a	c	a	c	a	a	t	g	g	a	t
KF558372	Vac	g	a	a	c	c	t	c	a	t	c	t	a	c	c	c	t	c	g	t	a	t	t	a	g	t	a	c	a	c	a	a	t	g	g	a	t
KF558373	Vac	g	a	a	c	c	t	c	a	t	c	t	a	c	c	c	t	c	g	t	a	t	t	a	g	t	a	c	a	c	a	a	t	g	g	a	t
KF558374	Vac	g	a	a	c	c	t	c	a	t	c	t	a	c	c	c	t	c	g	t	a	t	t	a	g	t	a	c	a	c	a	a	t	g	g	a	t
KF558375	Vac	g	g	a	c	c	t	c	a	t	c	t	a	c	c	c	t	c	g	t	a	t	t	a	g	t	a	c	a	c	a	a	t	g	g	a	t
KF558376	Vac	g	a	a	c	c	t	c	a	t	c	t	a	c	c	c	t	c	g	t	a	t	t	a	g	t	a	c	a	c	a	a	t	g	g	a	t
KF558377	Vac	g	g	a	c	c	t	c	a	t	c	t	a	c	c	c	t	c	g	t	a	t	t	a	g	t	a	c	a	c	a	a	t	g	g	a	t
KF558378	Vac	g	a	a	c	c	t	c	a	t	c	t	a	c	c	c	t	c	g	t	a	t	t	a	g	t	a	c	a	c	a	a	t	g	g	a	t
KF558379	Vac	g	a	a	c	c	t	c	a	t	c	t	a	c	c	c	t	c	g	t	a	t	c	a	g	t	a	c	a	c	a	a	t	g	g	a	t
KF558380	Vac	g	g	a	c	c	t	c	a	t	c	t	a	c	c	c	t	c	g	t	a	t	t	a	g	t	a	c	a	c	a	a	t	g	g	a	t
KF558381	Vac	g	a	a	c	c	t	c	a	t	c	t	a	c	c	c	t	c	g	t	a	t	t	a	g	t	a	c	a	c	a	a	t	g	g	a	t
KF558382	Vac	g	a	a	c	c	t	c	a	t	c	t	a	c	c	c	t	c	g	t	a	t	t	a	g	t	a	c	a	c	a	a	t	g	g	a	t
KF558383	Vac	g	a	a	c	c	t	c	a	t	c	t	a	c	c	c	t	c	g	t	a	t	t	a	g	t	a	c	a	c	a	a	t	g	g	a	t
KF558384	Vac	g	a	a	c	c	t	c	a	t	c	t	a	c	c	c	t	c	g	t	a	t	t	a	g	t	a	c	a	c	a	a	t	g	g	a	t
KF558385	Vac	g	a	a	c	c	t	c	a	t	c	t	a	c	c	c	t	c	g	t	a	t	t	a	g	t	a	c	a	c	a	a	t	g	g	a	t
KF558386	Vac	g	a	a	c	c	t	c	a	t	c	t	a	c	c	c	t	c	g	t	a	t	t	a	g	t	a	c	a	c	a	a	t	g	g	a	t
KF558387	Vac	g	a	a	c	c	t	c	a	t	c	t	a	c	c	c	t	c	g	t	a	t	t	a	g	t	a	c	a	c	a	a	t	g	g	a	t
KF558388	Vac	g	g	a	c	c	t	c	a	t	c	t	a	c	c	c	t	c	g	t	a	t	t	a	g	t	a	c	a	c	a	a	t	g	g	a	t
KF558389	Vac	g	a	a	c	c	t	c	a	t	c	t	a	c	c	c	t	c	g	t	a	t	t	a	g	t	a	c	a	c	a	a	t	g	g	a	t
KF558390	Vac	g	g	a	c	c	t	c	a	t	c	t	a	c	c	c	t	c	g	t	a	t	t	a	g	t	a	c	a	c	a	a	t	g	g	a	t
KF558391	Vac	g	a	a	c	c	t	c	a	t	c	t	a	c	c	c	t	c	g	t	a	t	t	a	g	t	a	c	a	c	a	a	t	g	g	a	t
KF811485	Vac	g	a	a	c	c	t	c	a	t	c	t	a	c	c	c	t	c	g	t	a	t	t	a	g	t	a	c	a	c	a	a	t	g	g	a	t
KF853225	Vac	g	a	a	c	c	t	c	a	t	c	t	a	c	c	c	t	c	g	t	a	t	t	a	g	t	a	c	a	c	a	a	t	g	g	a	t
KF853226	Vac	g	a	a	c	c	t	c	a	t	c	t	a	c	c	c	t	c	g	t	a	t	t	a	g	t	a	c	a	c	a	a	t	g	g	a	t
KF853227	Vac	g	g	a	c	c	t	c	a	t	c	t	a	c	c	c	t	c	g	t	a	t	t	a	g	t	a	c	a	c	a	a	t	g	g	a	t
KF853228	Vac	g	a	a	c	c	t	c	a	t	c	t	a	c	c	c	t	c	g	t	a	t	t	a	g	t	a	c	a	c	a	a	t	g	g	a	t
KF853229	Vac	g	a	a	c	c	t	c	a	t	c	t	a	c	c	c	t	c	g	t	a	t	t	a	g	t	a	c	a	c	a	a	t	g	g	a	t
KF853230	Vac	g	a	a	c	c	t	c	a	t	c	t	a	c	c	c	t	c	g	t	a	t	c	a	g	t	a	c	a	c	a	a	t	g	g	a	t
KF853231	Vac	g	a	a	c	c	t	c	a	t	c	t	a	c	c	c	t	c	g	t	a	t	t	a	g	t	a	c	a	c	a	a	t	g	g	a	t
KF853232	Vac	g	a	a	c	c	t	c	a	t	c	t	a	c	c	c	t	c	g	t	a	t	c	a	g	t	a	c	a	c	a	a	t	g	g	a	t
KF853233	Vac	g	a	a	c	c	t	c	a	t	c	t	a	c	c	c	t	c	g	t	a	t	t	a	g	t	a	c	a	c	a	a	t	g	g	a	t
KF853234	Vac	g	a	a	c	c	t	c	a	t	c	t	a	c	c	c	t	c	g	t	a	t	t	a	g	t	a	c	a	c	a	a	t	g	g	a	t
KF853235	Vac	g	a	a	c	c	t	c	a	t	c	t	a	c	c	c	t	c	g	t	a	t	t	a	g	t	a	c	a	c	a	a	t	g	g	a	t
MF898328	Vac	g	r	a	c	c	t	c	a	t	c	t	a	c	c	c	t	c	g	t	a	t	t	a	g	t	a	c	a	c	a	a	t	g	g	a	t
JN704709	VIII	g	g	a	c	c	t	c	a	t	c	t	a	c	c	c	t	c	g	t	a	t	t	a	g	t	a	c	a	c	a	a	t	g	g	a	t

GB-ID	28886	28895	28900	29019	29020	29066	29084	29093	29188	29201	29213	29273	29300	29358	29456	29509	29546	29568	29570	29592	29600	29684	29744	29750	29831	29957	29974	30161	30206	30270	30465	30477	30486	30506	30584	30638	30675	30688
MH499467	c	a	t	a	t	a	a	g	a	t	g	a	a	a	a	a	a	a	g	a	a	t	c	t	t	t	t	t	g	a	t	g	g	c	a	t	g	t
MH499469	c	a	t	a	t	a	a	g	a	t	g	a	a	a	a	a	a	a	g	a	a	t	c	t	t	t	t	t	g	a	t	g	g	c	a	t	g	t
MH709321	c	a	c	a	t	a	a	g	a	t	g	a	a	a	a	a	a	a	g	a	a	g	c	t	t	t	t	t	g	c	t	g	g	c	a	t	g	t
MH709324	c	a	t	a	t	a	a	g	a	t	g	a	a	a	a	a	a	a	g	a	a	t	c	t	t	t	t	t	g	a	t	g	g	c	a	t	g	t
MH709325	c	a	t	a	t	a	a	g	a	t	g	a	a	a	a	a	g	a	g	a	a	t	c	t	t	t	t	t	g	c	t	g	g	c	a	t	g	t
MH709327	c	a	t	a	t	a	a	g	a	t	g	a	a	a	a	a	a	a	g	a	a	t	c	t	t	t	t	t	g	a	t	g	g	c	a	t	g	t
MH709328	c	a	t	a	t	a	a	g	a	t	g	a	a	a	a	a	a	a	g	a	a	t	c	t	t	t	t	t	g	a	t	g	g	c	a	t	g	t
MH709330	c	a	t	a	t	a	a	g	a	t	g	a	a	a	a	a	a	a	g	a	a	t	c	t	t	t	t	t	g	a	t	g	g	c	a	t	g	t
MH709332	c	a	t	a	t	a	a	g	a	t	g	a	a	a	a	a	a	a	g	a	a	t	c	t	t	t	t	t	g	a	t	g	g	c	a	t	g	t
MH709334	c	a	t	a	t	a	a	g	a	t	g	a	a	a	a	a	a	a	g	a	a	t	c	t	t	t	t	t	g	a	t	g	g	c	a	t	g	t
MH709335	c	a	t	a	t	a	a	g	a	t	g	a	a	a	a	a	a	a	g	a	a	t	c	t	t	t	t	t	g	a	t	g	g	c	a	t	g	t
MH709336	c	a	t	a	t	a	a	g	a	t	g	a	a	a	a	a	a	a	g	a	a	t	c	t	t	t	t	t	g	a	t	g	g	c	a	t	g	t
MH709338	c	a	t	a	t	a	a	g	a	t	g	a	a	a	a	a	a	g	g	a	a	t	c	t	t	t	t	t	g	a	t	g	g	c	a	t	g	t
MH709340	c	a	t	a	t	a	a	g	a	t	g	a	a	a	a	a	a	a	g	a	a	t	c	t	t	t	t	t	g	c	t	g	g	c	a	t	g	t
MH709342	c	a	c	a	t	a	a	g	a	t	g	a	a	a	a	a	a	a	g	a	a	g	c	t	t	t	t	t	g	c	t	g	g	c	a	t	g	t
MH709343	c	a	t	a	t	a	a	g	a	t	g	a	a	a	a	a	a	a	g	a	a	t	c	t	t	t	t	t	g	a	t	g	g	c	a	t	g	t
MH709345	c	a	t	a	t	a	a	g	a	t	g	a	a	a	a	a	a	a	g	a	a	t	c	t	t	t	t	t	g	a	t	g	g	c	a	t	g	t
MH709347	c	a	t	a	t	a	a	g	a	t	g	a	a	a	a	a	a	a	g	a	a	t	c	t	t	t	t	t	g	a	t	g	g	c	a	t	g	t
MH709349	c	a	t	a	t	a	a	g	a	t	g	a	a	a	a	a	a	a	g	a	a	t	c	t	t	t	t	t	g	a	t	g	g	c	a	t	g	t
MH709350	c	a	t	a	t	a	a	g	a	t	g	a	a	a	a	a	a	a	g	a	a	t	c	t	t	t	t	t	g	a	t	g	g	c	a	t	g	t
MH709351	c	a	t	a	t	a	a	g	a	t	g	a	a	a	a	a	a	a	g	a	a	t	c	t	t	t	t	t	g	c	t	g	g	t	a	t	g	t
MH709353	c	a	t	a	t	a	a	g	a	t	g	a	a	a	a	a	a	a	g	a	a	t	c	t	t	t	t	t	g	a	t	g	g	c	a	t	g	t
MH709358	c	a	t	a	t	a	a	g	a	t	g	a	a	a	a	a	g	a	g	a	a	t	c	t	t	t	t	c	g	c	t	g	g	c	a	t	g	t
MH709360	c	a	t	a	t	a	a	g	a	t	g	a	a	a	a	a	a	a	g	a	a	t	c	t	t	t	t	t	g	a	t	g	g	c	a	t	g	t
MH709362	c	a	t	a	t	a	a	g	a	t	g	a	a	a	a	a	a	a	g	a	a	t	c	t	t	t	t	t	g	a	t	g	g	c	a	t	g	t
MH709365	c	a	t	a	t	a	a	g	a	t	g	a	a	a	a	a	a	a	g	a	a	t	c	t	t	t	t	t	g	a	t	g	g	c	a	t	g	t
MH709373	c	a	t	a	t	a	a	g	a	t	g	a	a	a	a	a	a	a	g	a	a	t	c	t	t	t	t	t	g	a	t	g	g	c	a	t	g	t
MH709374	c	a	t	a	t	a	a	g	a	t	g	a	a	a	a	a	a	a	g	a	a	t	c	t	t	t	t	t	g	a	t	g	g	c	a	t	g	t
KC112914	c	a	t	a	t	a	a	g	a	t	g	a	a	a	a	a	a	a	g	a	a	t	c	t	t	t	g	t	g	a	t	g	g	c	a	t	g	t
KP771914	c	a	t	a	t	a	a	g	a	t	g	a	a	a	a	a	a	a	g	a	a	t	c	t	t	t	g	t	g	a	t	g	g	c	a	t	g	t
KP702725	c	a	t	a	t	a	a	g	a	t	g	a	a	a	a	a	a	a	g	a	a	t	c	t	t	t	g	t	g	a	t	g	g	c	a	t	g	t
KP771890	c	a	t	a	t	a	a	g	a	t	g	a	a	a	a	g	a	a	g	a	a	t	c	t	c	t	g	t	a	a	t	g	t	c	a	t	g	t
JN704710	c	a	t	a	t	a	a	g	a	t	g	a	a	a	a	g	a	a	g	a	a	t	c	t	c	t	g	t	a	a	t	g	t	c	a	t	g	t
KY037798	c	a	t	a	t	a	a	g	a	t	g	a	a	a	a	g	a	a	g	a	a	t	c	t	c	t	g	t	a	a	t	g	t	c	a	t	g	t
MH709312	c	a	t	a	t	a	a	g	a	t	g	a	a	a	a	g	a	a	g	a	a	t	c	t	c	t	g	t	a	a	t	g	t	c	a	t	g	t
MH709352	c	a	t	a	t	a	a	g	a	t	g	a	a	a	a	g	a	a	g	a	a	t	c	t	c	t	g	t	a	a	t	g	t	c	a	t	g	t
AB097933	c	a	t	a	t	a	a	g	a	g	g	a	a	a	a	a	a	a	g	a	a	t	c	t	c	t	g	t	a	a	t	g	t	c	a	t	g	t
JN704698	c	a	t	a	t	a	a	g	a	g	g	a	a	a	a	a	a	a	g	a	a	t	c	t	c	t	g	t	a	a	t	g	t	c	a	t	g	t
JN704697	c	a	t	a	t	a	a	g	a	g	g	a	a	a	a	a	a	a	g	a	a	t	c	t	c	t	g	t	a	a	t	g	t	c	a	t	g	t
DQ008355	c	a	t	a	t	a	a	g	a	g	g	a	a	a	a	a	a	a	g	a	a	t	c	t	c	t	g	t	a	a	t	g	t	c	a	t	g	t
JF306641	c	a	t	a	t	a	a	g	a	g	g	a	a	a	a	a	a	a	g	a	a	t	c	t	c	t	g	t	a	a	t	g	t	c	a	t	g	t
AB097932	c	a	t	a	t	a	a	g	a	g	g	a	a	a	a	a	a	a	g	a	a	t	c	t	c	t	g	t	a	a	t	g	t	c	a	t	g	t
DQ008354	c	a	t	a	t	a	a	g	a	g	g	a	a	a	a	a	a	a	g	a	a	t	c	t	c	t	g	t	a	a	t	g	t	c	a	t	g	t
KU926312	c	a	t	a	t	a	a	g	a	g	g	a	a	a	a	a	a	a	g	a	a	t	c	t	c	t	g	t	a	a	t	g	t	c	a	t	g	t
KU926313	c	a	t	a	t	a	a	g	a	g	g	a	a	a	a	a	a	a	g	a	a	t	c	t	c	t	g	t	a	a	t	g	t	c	a	t	g	t
KU926314	c	a	t	a	t	a	a	g	a	g	g	a	a	a	a	a	a	a	g	a	a	t	c	t	c	t	g	t	a	a	t	g	t	c	a	t	g	t
KF558371	c	a	t	a	t	a	a	g	a	g	g	a	a	a	a	a	a	a	g	a	a	t	c	t	c	t	g	t	a	a	t	g	t	c	a	t	g	t
KF558372	c	a	t	a	t	a	a	g	a	g	g	a	a	a	a	a	a	a	g	g	a	t	c	t	c	t	g	t	a	a	t	g	t	c	a	t	g	c
KF558373	c	a	t	a	t	a	a	g	a	g	g	a	a	a	a	a	a	a	g	a	a	t	c	t	c	t	g	t	a	a	t	g	t	c	a	t	g	t
KF558374	c	a	t	a	t	a	a	g	a	g	g	a	a	a	a	a	a	a	g	g	a	t	c	t	c	t	g	t	a	a	t	g	t	c	a	t	g	c
KF558375	c	a	t	a	t	a	a	g	a	g	g	a	a	a	a	a	a	a	g	a	a	t	c	t	c	t	g	t	a	a	t	g	t	c	a	t	g	t
KF558376	c	a	t	a	t	a	a	g	a	g	g	a	a	a	a	a	a	a	g	a	a	t	c	t	c	t	g	t	a	a	t	g	t	c	a	t	g	t
KF558377	c	a	t	a	t	a	a	g	a	g	g	a	a	a	a	a	a	a	g	a	a	t	c	t	c	t	g	t	a	a	t	g	t	c	a	t	g	t
KF558378	c	a	t	a	t	a	a	g	a	g	g	a	a	a	a	a	a	a	g	a	a	t	c	t	c	t	g	t	a	a	t	g	t	c	a	t	g	t
KF558379	c	a	t	a	t	a	a	g	a	g	g	a	a	a	a	a	a	a	g	a	a	t	c	t	c	t	g	t	a	a	t	g	t	c	a	t	g	t
KF558380	c	a	t	a	t	a	a	g	a	g	g	a	a	a	a	a	a	a	g	a	a	t	c	t	c	t	g	t	a	a	t	g	t	c	a	t	g	t
KF558381	c	a	t	a	t	a	a	g	a	g	g	a	a	a	a	a	a	a	g	a	a	t	c	t	c	t	g	t	a	a	t	g	t	c	a	t	g	t
KF558382	c	a	t	a	t	a	a	g	a	g	g	a	a	a	a	a	a	a	g	g	a	t	c	t	c	t	g	t	a	a	t	g	t	c	a	t	g	t
KF558383	c	a	t	a	t	a	a	g	a	g	g	a	a	a	a	a	a	a	g	a	a	t	c	t	c	t	g	t	a	a	t	g	t	c	a	t	g	t
KF558384	c	a	t	a	t	a	a	g	a	g	g	a	a	a	a	a	a	a	g	a	a	t	c	t	c	t	g	t	a	a	t	g	t	c	a	t	g	t
KF558385	c	a	t	a	t	a	a	g	a	g	g	a	a	a	a	a	a	a	g	a	a	t	c	t	c	t	g	t	a	a	t	g	t	c	a	t	g	t
KF558386	c	a	t	a	t	a	a	g	a	g	g	a	a	a	a	a	a	a	g	a	a	t	c	t	c	t	g	t	a	a	t	g	t	c	a	t	g	t
KF558387	c	a	t	a	t	a	a	g	a	g	g	a	a	a	a	a	a	a	g	a	a	t	c	t	c	t	g	t	a	a	t	g	t	c	a	t	g	t
KF558388	c	a	t	a	t	a	a	g	a	g	g	a	a	a	a	a	a	a	g	a	a	t	c	t	c	t	g	t	a	a	t	g	t	c	a	t	g	t
KF558389	c	a	t	a	t	a	a	g	a	g	g	a	a	a	a	a	a	a	g	g	a	t	c	t	c	t	g	t	a	a	t	g	t	c	a	t	g	c
KF558390	c	a	t	a	t	a	a	g	a	g	g	a	a	a	a	a	a	a	g	a	a	t	c	t	c	t	g	t	a	a	t	g	t	c	a	t	g	t
KF558391	c	a	t	a	t	a	a	g	a	g	g	a	a	a	a	a	a	a	g	a	a	t	c	t	c	t	g	t	a	a	t	g	t	c	a	t	g	t
KF811485	c	a	t	a	t	a	a	g	a	g	g	a	a	a	a	a	a	a	g	a	a	t	c	t	c	t	g	t	a	a	t	g	t	c	a	t	g	t
KF853225	c	a	t	a	t	a	a	g	a	g	g	a	a	a	a	a	a	a	g	a	a	t	c	t	c	t	g	t	a	a	t	g	t	c	a	t	g	t
KF853226	c	a	t	a	t	a	a	g	a	g	g	a	a	a	a	a	a	a	g	a	a	t	c	t	c	t	g	t	a	a	t	g	t	c	a	t	g	t
KF853227	c	a	t	a	t	a	a	g	a	g	g	a	a	a	a	a	a	a	g	a	a	t	c	t	c	t	g	t	a	a	t	g	t	c	a	t	g	t
KF853228	c	a	t	a	t	a	a	g	a	g	g	a	a	a	a	a	a	a	g	a	a	t	c	t	c	t	g	t	a	a	t	g	t	c	a	t	g	t
KF853229	c	a	t	a	t	a	a	g	a	g	g	a	a	a	a	a	a	a	g	a	a	t	c	t	c	t	g	t	a	a	t	g	t	c	a	t	g	t
KF853230	c	a	t	a	t	a	a	g	a	g	g	a	a	a	a	a	a	a	g	g	a	t	c	t	c	t	g	t	a	a	t	g	t	c	a	t	g	t
KF853231	c	a	t	a	t	a	a	g	a	g	g	a	a	a	a	a	a	a	g	a	a	t	c	t	c	t	g	t	a	a	t	g	t	c	a	t	g	t
KF853232	c	a	t	a	t	a	a	g	a	g	g	a	a	a	a	a	a	a	g	a	a	t	c	t	c	t	g	t	a	a	t	g	t	c	a	t	g	t
KF853233	c	a	t	a	t	a	a	g	a	g	g	a	a	a	a	a	a	a	g	a	a	t	c	t	c	t	g	t	a	a	t	g	t	c	a	t	g	t
KF853234	c	a	t	a	t	a	a	g	a	g	g	a	a	a	a	a	a	a	g	a	a	t	c	t	c	t	g	t	a	a	t	g	t	c	a	t	g	t
KF853235	c	a	t	a	t	a	a	g	a	g	g	a	a	a	a	a	a	a	g	a	a	t	c	c	c	t	g	t	a	a	t	g	t	c	a	t	g	t
MF898328	c	a	t	a	t	a	a	g	a	g	g	a	a	a	a	a	a	a	g	a	a	t	c	t	c	t	g	t	a	a	t	g	t	c	a	t	g	t
JN704709	c	a	t	a	t	a	a	g	a	g	g	a	a	a	a	a	a	a	g	a	a	t	c	t	c	t	g	t	a	a	t	g	t	c	a	t	g	t

Supplementary Table 3: 2880 SN

GB-ID	Clade markers															5			SD14																												1	1															6											SD14	
	Position in Dumas	30769	30844	30859	30871	30881	30947	30948	31057	31075	31124	31289	31331	31356	31475	31619	31820	31946	31976	32021	32255	32269	32274	32513	32604	32684	32846	32879	32881	32963	32987	32990	33068	33095	33100	33173	33176	33212	33224	33287	33473	33563	33646	33654	33655	33722	33725	33728	33732	33749	33773	33832	33927	33958	34293	34466	34562	34567	34595	34613	34619	34682	34875	35007	35022	35048	35051	35088	35141	35200	35223	35225	35226	35243	35287
AY548170	1	c	c	a	c	g	c	c	c	a	g	g	c	g	g	c	c	a	a	c	a	g	a	t	t	t	g	t	t	g	c	g	t	a	a	a	c	c	c	t	g	g	g	a	t	c	t	t	a	c	g	t	t	c	c	t	g	a	c	g	a	c	t	a	t	g	t	c	c	a	c	g	g	a	t
AY548171	1	c	c	a	c	g	c	c	c	a	g	g	c	g	g	c	c	a	a	c	a	g	a	t	t	t	g	t	t	g	c	g	t	a	a	a	c	c	c	t	g	g	g	a	t	c	t	t	g	c	g	t	t	c	c	t	g	a	c	g	a	c	t	a	t	g	t	c	c	a	c	g	g	a	t
DQ479953	1	c	c	a	c	g	c	c	c	a	g	g	c	g	g	c	c	a	a	c	a	g	a	t	t	t	g	t	t	g	c	g	t	a	a	a	c	c	c	t	g	g	g	a	t	c	t	t	a	c	g	t	t	c	c	t	g	a	c	g	a	c	t	a	t	g	t	c	c	a	c	g	g	a	t
DQ479954	1	c	c	a	c	g	c	c	c	a	g	g	c	g	g	c	c	a	a	c	a	g	a	t	t	t	g	t	t	g	c	g	t	a	a	a	c	c	c	t	g	g	g	a	t	c	t	t	a	c	g	t	t	c	c	t	g	a	t	g	a	c	t	a	t	g	t	c	c	a	c	g	g	a	t
DQ479958	1	c	c	a	c	g	c	c	c	a	g	g	c	g	g	c	c	a	a	c	a	g	a	t	t	t	g	t	t	g	c	g	t	a	a	a	c	c	c	t	g	g	g	a	t	c	t	t	a	c	g	t	t	c	c	t	g	a	c	g	a	c	t	a	t	g	t	c	c	a	c	g	g	a	t
DQ479959	1	c	c	a	c	g	c	c	c	a	g	g	c	g	g	c	c	a	a	c	a	g	a	t	t	t	g	t	t	g	c	g	t	a	a	a	c	c	c	t	g	g	g	a	t	c	t	t	a	c	g	t	t	c	c	t	g	a	c	g	a	c	t	a	t	g	t	c	c	a	c	g	g	a	t
DQ479961	1	c	c	a	c	g	c	c	c	a	g	g	c	g	g	c	c	a	a	c	a	g	a	t	t	t	g	t	t	g	c	g	t	a	a	a	c	c	t	t	g	g	g	a	t	c	t	t	a	c	g	t	t	c	c	t	g	a	c	g	a	c	t	a	t	g	t	c	c	a	c	g	g	a	t
DQ479962	1	c	c	a	c	g	c	c	c	a	g	g	c	g	g	c	c	a	a	c	a	g	a	t	t	t	g	t	t	g	c	g	t	a	a	a	c	c	t	t	g	g	g	a	t	c	t	t	a	c	g	t	t	c	c	t	g	a	c	g	a	c	t	a	t	g	t	c	c	a	c	g	g	a	t
DQ479963	1	c	c	a	c	g	c	c	c	a	g	g	c	g	g	c	c	a	a	c	a	g	a	t	t	t	g	t	t	g	c	g	t	a	a	a	c	c	t	t	g	g	g	a	t	c	t	t	a	c	g	t	t	c	c	t	g	a	c	g	a	c	t	a	t	g	t	c	c	a	c	g	g	a	t
DQ674250	1	c	c	a	c	g	c	c	c	a	g	g	c	g	g	c	c	a	a	c	a	g	a	t	t	t	g	t	t	g	c	g	t	a	a	a	c	c	c	t	g	g	g	a	t	c	t	t	a	c	g	t	t	c	c	t	g	a	c	g	a	c	t	a	t	g	t	c	c	a	c	g	g	a	t
KX262864	1	c	c	a	c	g	c	c	c	a	g	g	c	g	g	c	c	a	a	c	a	g	a	t	t	t	g	t	t	g	c	g	t	a	a	a	c	c	c	t	g	g	g	a	t	c	t	t	a	c	g	t	t	c	c	t	g	a	c	g	a	c	t	a	t	g	t	c	c	a	c	g	g	a	t
KX262865	1	c	c	a	c	g	c	c	c	a	g	g	c	g	g	c	c	a	a	c	a	g	a	t	t	t	g	t	t	g	c	g	t	a	a	a	c	c	c	t	g	g	g	a	t	c	t	t	a	c	g	t	t	c	c	t	g	a	c	g	a	c	t	a	t	g	t	c	c	g	c	g	g	a	t
KX262866	1	c	c	a	c	g	c	c	c	a	g	g	c	g	g	c	c	a	a	c	a	g	a	t	t	t	g	t	t	g	c	g	t	a	a	a	c	c	c	t	g	g	g	a	t	c	t	t	a	c	g	t	t	c	c	t	g	a	c	g	a	c	t	a	t	g	t	c	c	a	c	g	g	a	t
EU154348	1	c	c	a	c	g	c	c	c	a	g	g	c	g	g	c	c	a	a	c	a	g	a	t	t	t	g	t	t	g	c	g	t	a	a	a	c	c	c	t	g	g	g	a	t	c	t	t	a	c	g	t	t	c	c	t	g	a	c	g	a	c	t	a	t	g	t	c	c	a	c	g	g	a	t
JN704690	1	c	c	a	c	g	c	c	c	a	g	g	c	g	g	c	c	a	a	c	a	g	a	t	t	t	g	t	t	g	c	g	t	a	a	a	c	c	c	t	g	g	g	a	t	c	t	t	a	c	g	t	t	c	c	t	g	a	c	g	a	c	t	a	t	g	t	c	c	a	c	g	g	a	t
JN704691	1	c	c	a	c	g	c	c	c	a	g	g	c	g	g	c	c	a	a	c	a	g	a	t	t	t	g	t	t	g	c	g	t	a	a	a	c	c	c	t	g	g	g	a	t	t	t	t	a	c	g	t	t	c	c	t	g	a	c	g	a	c	t	a	t	g	t	c	c	a	c	g	g	a	t
JN704692	1	c	c	a	c	g	c	c	c	a	g	g	c	g	g	c	c	a	a	c	a	g	a	t	t	t	g	t	t	g	c	g	t	a	a	a	c	c	c	t	g	g	g	a	t	c	t	t	a	c	g	t	t	c	c	t	g	a	c	g	a	c	t	a	t	g	t	c	c	a	c	g	g	a	t
JN704693	1	c	c	a	c	g	c	c	c	a	g	g	c	g	g	c	c	a	a	c	a	g	a	t	t	t	g	t	t	g	c	g	t	a	a	a	c	c	c	t	g	g	g	a	t	c	t	t	a	c	g	t	t	c	c	t	g	a	c	g	a	c	t	a	t	g	t	c	c	a	c	g	g	a	t
JN704694	1	c	c	a	c	g	c	c	c	a	g	g	c	g	a	c	c	a	a	c	a	g	a	t	t	t	g	t	t	g	c	g	t	a	a	a	c	c	c	t	g	g	g	a	t	c	t	t	a	c	g	t	t	t	c	t	g	a	c	g	a	c	t	a	t	g	t	c	c	a	c	g	g	a	t
JN704695	1	c	c	a	c	g	c	c	c	a	g	g	c	g	g	c	c	a	a	c	a	g	a	t	t	t	g	t	t	g	c	g	t	a	a	a	c	c	c	t	g	g	g	a	t	c	t	t	a	c	g	t	t	c	c	t	g	a	c	g	a	c	t	a	t	g	t	c	c	a	c	g	g	a	t
JN704696	1	c	c	a	c	g	c	c	c	a	g	g	c	g	g	c	c	a	a	c	a	g	a	t	t	t	g	t	t	g	c	g	t	a	a	a	c	c	c	t	g	g	g	a	t	c	t	t	a	c	g	t	c	c	c	t	g	a	c	g	a	c	t	a	t	g	t	c	c	a	c	g	g	a	t
NC001348	1	c	c	a	c	g	c	c	c	a	g	g	c	g	g	c	c	a	a	c	a	g	a	t	t	t	g	t	t	g	c	g	t	a	a	a	c	c	c	t	g	g	g	a	t	t	t	t	a	c	g	t	t	c	c	t	g	a	c	g	a	c	t	a	t	g	t	c	c	a	c	g	g	a	t
KP771892	1	c	c	a	c	g	c	c	c	a	g	g	c	g	g	c	c	a	a	c	a	g	a	t	t	t	g	t	t	g	c	g	t	a	a	a	c	c	c	t	g	g	g	a	t	c	t	t	a	c	a	t	t	c	c	t	g	a	c	g	a	c	t	a	t	a	t	c	c	a	c	g	g	a	t
KP771894	1	c	c	a	c	g	c	c	c	a	g	g	c	g	g	c	c	a	a	c	a	g	a	t	t	t	g	t	t	g	c	g	t	a	a	a	c	c	c	t	g	g	g	a	t	c	t	t	a	c	g	t	t	c	c	t	g	a	c	g	a	c	t	a	t	g	t	c	c	a	c	g	g	a	t
KP771900	1	c	c	a	c	g	c	c	c	a	g	g	c	g	g	c	c	a	a	c	a	g	a	t	t	t	g	t	t	g	c	g	t	a	a	a	c	c	c	t	g	g	g	a	t	c	t	t	a	c	g	t	t	c	c	t	g	a	c	g	a	c	t	a	t	g	t	c	c	a	c	g	g	a	t
KP771901	1	c	c	a	c	g	c	c	c	a	g	g	c	g	g	c	c	a	a	c	a	g	a	t	t	t	g	t	t	g	c	g	t	a	a	a	c	c	c	t	g	g	g	a	t	c	t	t	a	c	g	t	t	c	c	t	g	a	c	g	a	c	t	a	t	g	t	c	c	a	c	g	g	a	t
KP771902	1	c	c	a	c	g	c	c	c	a	g	g	c	g	g	c	c	a	a	c	a	g	a	t	t	t	g	t	t	g	c	g	t	a	a	a	c	c	c	t	g	g	g	a	t	c	t	t	a	c	g	t	t	c	c	t	g	a	c	g	a	c	t	a	t	g	t	c	c	a	c	g	g	a	t
KP771908	1	c	c	a	c	g	c	c	c	a	g	g	c	g	g	c	c	a	a	c	a	g	a	t	t	t	g	t	t	g	c	g	t	a	a	a	c	c	c	t	g	g	g	a	t	c	t	t	a	c	g	t	t	c	c	t	g	a	c	g	a	c	t	a	t	g	t	c	c	a	c	g	g	a	t
KP771912	1	c	c	a	c	g	c	c	c	a	g	g	c	g	g	c	c	a	a	c	a	g	a	t	t	t	g	t	t	g	c	g	t	a	a	a	c	c	c	t	g	g	g	a	t	c	t	t	a	c	g	t	t	c	c	t	g	a	c	g	a	c	t	a	t	g	t	c	c	a	c	g	g	a	t
KP771913	1	c	c	a	c	g	c	c	c	a	g	g	c	g	g	c	c	a	a	c	a	g	a	t	t	t	g	t	t	g	c	g	t	a	a	a	c	c	c	t	g	g	g	a	t	c	t	t	a	c	g	t	t	c	c	t	g	a	c	g	a	c	t	a	t	g	t	c	c	a	c	g	g	a	t
KP771917	1	c	c	a	c	g	c	c	c	a	g	g	c	g	g	c	c	a	a	c	a	g	a	t	t	t	g	t	t	g	c	g	t	a	a	a	c	c	c	t	g	g	g	a	t	c	t	t	a	c	g	t	t	c	c	t	g	a	c	g	a	c	t	a	t	g	t	c	c	a	c	g	g	a	g
KP771918	1	c	c	a	c	g	c	c	c	a	g	g	c	g	g	c	c	a	a	c	a	g	a	t	t	t	g	t	t	g	c	g	t	a	a	a	c	c	c	t	g	g	g	a	t	c	t	t	a	c	g	t	t	c	c	t	g	a	c	g	a	c	t	a	t	g	t	c	c	a	c	g	g	a	t
KP771920	1	c	c	a	c	g	c	c	c	a	g	g	c	g	g	c	c	a	a	c	a	g	a	t	t	t	g	t	t	g	c	g	t	a	a	a	c	c	c	t	g	g	g	a	t	c	t	t	a	c	g	t	t	c	c	t	g	a	c	a	a	c	t	a	t	g	t	c	c	a	c	g	g	a	t
MH709361	1	c	c	a	c	g	c	c	c	a	g	g	c	g	g	c	c	a	a	c	a	g	a	t	t	t	g	t	t	g	c	g	t	a	a	a	c	c	c	t	g	g	g	a	t	c	t	t	a	c	g	t	t	c	c	t	g	a	c	g	a	c	t	a	t	g	t	c	c	a	c	g	g	a	t
KU926318	2	c	c	a	c	g	c	c	c	a	g	g	c	g	g	c	c	a	a	c	a	g	g	t	t	t	g	t	t	g	c	g	t	a	a	a	c	c	c	t	g	g	g	a	t	c	c	c	a	c	g	t	t	c	c	t	g	a	c	g	a	c	t	g	t	g	t	c	c	a	c	g	g	a	t
KU926319	2	c	c	a	c	g	c	c	c	a	g	g	c	g	g	c	c	a	a	c	a	g	g	t	t	t	g	t	t	g	c	g	t	a	a	a	c	c	c	t	g	g	g	a	t	c	c	c	a	c	g	t	t	c	c	t	g	a	c	g	a	c	t	a	t	g	t	c	c	a	c	g	g	a	t
KU926320	2	c	c	a	c	g	c	c	c	a	g	g	c	g	g	c	c	a	a	c	a	g	g	t	t	t	g	t	t	g	c	g	t	a	a	a	c	c	c	t	g	g	g	a	t	c	c	c	a	c	g	t	t	c	c	t	g	a	c	g	a	c	t	a	c	g	t	c	c	a	c	g	g	a	t
KU926321	2	c	c	a	c	g	c	c	c	a	g	g	c	g	g	c	c	a	a	c	a	g	g	t	t	t	g	t	t	g	c	g	t	a	a	a	c	c	c	t	g	g	g	a	t	c	c	c	a	c	g	t	t	c	c	t	g	a	c	g	a	c	t	a	t	g	t	c	c	a	c	g	g	a	t
KU926322	2	c	c	a	c	g	c	c	c	a	g	g	c	g	g	c	c	a	a	c	a	g	g	t	t	t	g	t	t	g	c	g	t	a	a	a	c	c	c	t	g	g	g	a	t	c	c	c	a	c	g	t	t	c	c	t	g	a	c	g	a	c	t	a	t	g	t	c	c	a	c	g	g	a	t
KC847290	2	c	c	a	c	g	c	c	c	a	g	g	c	g	g	c	c	a	a	c	a	g	g	t	t	t	g	t	t	g	c	g	t	a	a	a	c	c	c	t	g	g	g	a	t	c	c	c	a	c	g	t	t	c	c	t	g	a	c	g	a	c	t	a	t	g	t	c	c	a	c	g	g	a	t
KJ767491	2	c	c	a	c	g	c	c	c	a	g	g	c	g	g	c	c	a	a	c	a	g	g	t	t	t	g	t	t	g	c	g	t	a	a	a	c	c	c	t	g	g	g	a	t	c	c	c	a	c	g	t	t	c	c	t	g	a	c	g	a	c	t	a	t	g	t	c	c	a	c	g	g	a	t
KJ767492	2	c	c	a	c	g	c	c	c	a	g	g	c	g	g	c	c	a	a	c	a	g	g	t	t	t	g	t	t	g	c	g	t	a	a	a	c	c	c	t	g	g	g	a	t	c	c	c	a	c	g	t	t	c	c	t	g	a	c	g	a	c	t	a	t	g	t	c	c	a	c	g	g	a	t
KJ808816	2	c	c	a	c	g	c	c	c	a	g	g	c	g	g	c	c	a	a	c	a	g	g	t	t	t	g	t	t	g	c	g	t	a	a	a	c	c	c	t	g	g	g	a	t	c	c	c	a	c	g	t	t	c	c	t	g	a	c	g	a	c	t	a	t	g	t	c	c	a	c	g	g	a	t
KP771925	2	c	c	a	c	g	c	c	c	a	g	g	c	g	g	c	c	a	a	c	a	g	g	t	t	t	g	t	t	g	c	g	t	a	a	a	c	c	c	t	g	g	g	a	t	c	c	c	a	c	g	t	t	c	c	t	g	a	c	g	a	c	t	a	t	g	t	c	c	a	c	g	g	a	t
KU926315	2	c	c	a	c	g	c	c	c	a	g	g	c	g	g	c	c	a	a	c	a	g	g	t	t	t	g	t	t	g	c	g	t	a	a	a	c	c	c	t	g	g	g	a	t	c	c	c	a	c	g	t	t	c	c	t	g	a	c	g	a	c	t	a	t	g	t	c	c	a	c	g	g	a	t
KU926316	2	c	c	a	c	g	c	c	c	a	g	g	c	g	g	c	c	a	a	c	a	g	g	t	t	t	g	t	t	g	c	g	t	a	a	a	c	c	c	t	g	g	g	a	t	c	c	c	a	c	g	t	t	c	c	t	g	a	c	g	a	c	t	a	t	g	t	c	c	a	c	g	g	a	t
KU926317	2	c	c	a	c	g	c	c	c	a	g	g	c	g	g	c	c	a	a	c	a	g	g	t	t	t	g	t	t	g	c	g	t	a	a	a	c	c	c	t	g	g	g	a	t	c	c	c	a	c	g	t	t	c	c	t	g	a	c	g	a	c	t	a	t	g	t	c	c	a	c	g	g	a	t
KY037796	2	c	c	a	c	g	c	c	c	a	g	g	c	g	g	c	c	a	a	c	a	g	g	t	t	t	g	t	t	g	c	g	t	a	a	a	c	c	c	t	g	g	g	a	t	c	c	c	a	c	g	t	t	c	c	t	g	a	c	g	a	c	t	a	t	g	t	c	c	a	c	g	g	a	t
MF004348	2	c	c	a	c	g	c	c	c	a	g	g	c	g	g	c	c	a	a	c	a	g	g	t	t	t	g	t	t	g	c	g	t	a	a	a	c	c	c	t	g	g	g	a	t	c	c	c	a	c	g	t	t	c	c	t	g	a	c	g	a	c	t	a	t	g	t	c	c	a	c	g	g	a	t
MH709309	2	c	c	a	c	g	c	c	c	a	g	g	c	g	g	c	c	a	a	c	a	g	g	t	t	t	g	t	t	g	c	g	t	a	a	a	c	c	c	t	g	g	g	a	t	c	c	c	a	c	g	t	t	c	c	t	g	a	c	g	a	c	t	a	t	g	g	c	c	a	c	a	g	a	t
MH709310	2	c	c	a	c	g	c	c	c	a	g	g	c	g	g	c	c	a	a	c	a	g	g	t	t	t	g	t	t	g	c	g	t	a	a	a	c	c	c	t	g	g	g	a	t	c	c	c	a	c	g	t	t	c	c	t	g	a	c	g	a	c	t	a	t	g	t	c	c	a	c	g	g	a	t
MH709311	2	c	c	a	c	g	c	c	c	a	g	g	c	g	g	c	c	a	a	c	a	g	g	t	t	t	g	t	t	g	c	g	t	a	a	a	c	c	c	t	g	g	g	a	t	c	c	c	a	c	g	t	t	c	c	t	g	a	c	g	a	c	t	a	t	g	t	a	c	a	c	g	a	a	t
MH709313	2	c	c	a	c	g	c	c	c	a	g	g	c	g	g	c	c	a	a	c	a	g	g	t	t	t	g	t	t	g	c	g	t	a	a	a	c	c	c	t	g	g	g	a	t	c	c	c	a	c	g	t	t	c	c	t	g	a	c	g	a	c	t	a	t	g	t	c	c	a	c	g	g	a	t
MH709314	2	c	c	a	c	g	c	c	c	a	g	g	c	g	g	c	c	a	a	c	a	g	g	t	t	t	g	t	t	g	c	g	t	a	a	a	c	c	c	t	g	g	g	a	t	c	c	c	a	c	g	t	t	c	c	t	g	a	c	g	a	c	t	a	t	g	t	c	c	a	c	g	g	a	t
MH709315	2	c	c	a	c	g	c	c	c	a	g	g	c	g	g	c	c	a	a	c	a	g	g	t	t	t	g	t	t	g	c	g	t	a	a	a	c	c	c	t	g	g	g	a	t	c	c	c	a	c	g	t	t	c	c	t	g	a	c	g	a	c	t	a	t	g	t	c	c	a	c	g	g	a	t
MH709319	2	c	c	a	c	g	c	c	c	a	g	g	c	g	g	c	c	a	a	c	a	g	g	t	t	t	g	t	t	g	c	g	t	a	a	a	c	c	c	t	g	g	g	a	t	c	c	c	a	c	g	t	t	c	c	t	g	a	c	g	a	c	t	a	t	g	t	c	c	a	c	g	g	a	t
MH709320	2	c	c	a	c	g	c	c	c	a	g	g	c	g	g	c	c	a	a	c	a	g	g	t	t	t	g	t	t	a	c	g	t	a	a	a	c	c	c	t	g	g	g	a	t	c	c	c	a	c	g	t	t	c	c	t	g	a	c	g	a	c	t	a	t	g	t	c	c	a	c	g	g	a	t
MH709329	2	c	c	a	c	g	c	c	c	a	g	g	c	g	g	c	c	a	a	c	a	g	g	t	t	t	g	t	t	g	c	g	t	a	a	a	c	c	c	t	g	g	g	a	t	c	c	c	a	c	g	t	t	c	c	t	g	a	c	g	a	c	t	a	t	g	t	c	c	a	c	g	g	a	t
MH709331	2	c	c	a	c	g	c	c	c	a	g	g	c	g	g	c	c	a	a	c	a	g	g	t	t	t	a	t	t	g	c	g	t	a	a	a	c	c	c	t	g	g	g	a	t	c	c	c	a	c	g	t	t	c	c	t	g	a	c	g	a	c	t	a	t	g	t	c	c	a	c	g	g	a	t
MH709333	2	c	c	a	c	g	c	c	c	a	g	g	c	g	g	c	c	a	a	c	a	g	g	t	t	t	g	t	t	g	c	g	t	a	a	a	c	c	c	t	g	g	g	a	t	c	c	c	a	c	g	t	t	c	c	t	g	a	c	g	a	c	t	a	t	g	t	c	c	a	c	g	g	a	t
MH709337	2	c	c	a	c	g	c	c	c	a	g	g	c	g	g	c	c	a	a	c	a	g	g	t	t	t	g	t	t	g	c	g	t	a	a	a	c	c	c	t	g	g	g	a	t	c	c	c	a	c	g	t	t	c	c	t	g	a	c	g	a	c	t	a	t	g	t	c	c	a	c	g	g	a	t
MH709339	2	c	c	a	c	g	c	c	c	a	g	g	c	g	g	c	c	a	a	c	a	g	g	t	t	t	g	t	t	g	c	g	t	a	a	a	c	c	c	t	g	g	g	a	t	c	c	c	a	c	g	t	t	c	c	t	g	a	c	g	a	c	t	a	t	g	t	c	c	a	c	g	g	a	t
MH709344	2	c	c	a	c	g	c	c	c	a	g	g	c	g	g	c	c	a	a	c	a	g	g	t	t	t	g	t	t	g	c	g	t	a	a	a	c	c	c	t	g	g	g	a	t	c	c	c	a	c	g	t	t	c	c	t	g	a	c	g	a	c	t	a	t	g	t	c	c	a	c	g	g	a	t
MH709348	2	c	c	a	c	g	t	t	c	a	g	g	c	g	g	c	c	a	a	c	a	g	g	t	t	t	g	t	t	g	c	g	t	a	a	a	c	c	c	t	g	g	g	a	t	c	c	c	a	c	g	t	t	c	c	t	g	a	c	g	a	c	t	a	t	g	t	c	c	a	c	g	g	a	t
MH709355	2	c	c	a	c	g	c	c	c	a	g	g	c	g	g	c	c	a	a	c	a	g	g	t	t	t	g	t	t	g	c	g	t	a	a	a	c	c	c	t	g	g	g	a	t	c	c	c	a	c	g	t	t	c	c	t	g	a	c	g	a	c	t	a	t	g	t	c	c	a	c	g	g	a	t
MH709359	2	c	c	a	c	g	c	c	c	a	g	g	c	g	g	c	c	a	a	c	a	g	g	t	t	t	g	t	t	g	c	g	t	a	a	a	c	c	c	t	g	g	g	a	t	c	c	c	a	c	g	t	t	c	c	t	g	a	c	g	a	c	t	a	t	g	t	c	c	a	c	g	g	a	t
MH709363	2	c	c	a	c	g	c	c	c	a	g	g	c	g	g	c	c	a	a	c	a	g	g	t	t	t	g	t	t	g	c	g	t	a	a	a	c	c	c	t	g	g	g	a	t	c	c	c	a	c	g	t	t	c	c	t	g	a	c	g	a	c	t	a	t	g	t	c	c	a	c	g	g	a	t
MH709366	2	c	c	a	c	g	c	c	c	a	g	g	c	g	g	c	c	a	a	c	a	g	g	t	t	t	g	t	t	g	c	g	t	a	a	a	c	c	c	t	g	g	g	a	t	c	c	c	a	c	g	t	t	c	c	t	g	a	c	g	a	c	t	a	t	g	t	c	c	a	c	g	g	a	t
MH709376	2	c	c	a	c	g	c	c	c	a	g	g	c	g	g	c	c	a	a	c	a	g	g	t	t	t	g	t	t	g	c	g	t	a	a	a	c	c	c	t	g	g	g	a	t	c	c	c	a	c	g	t	t	c	c	t	g	a	c	g	a	c	t	a	t	g	t	c	c	a	c	g	g	a	t
MH709377	2	c	c	a	c	g	c	c	c	a	g	g	c	g	g	c	c	a	a	c	a	g	g	t	t	t	g	t	t	g	c	g	t	a	a	a	c	c	c	t	g	g	g	a	t	c	c	c	a	c	g	t	t	c	c	t	g	a	c	g	a	c	t	a	t	g	t	c	c	a	c	g	g	a	t
**MW316406**	2	c	c	a	c	g	c	c	c	a	g	g	c	g	g	c	c	a	g	c	a	g	g	t	t	t	g	t	t	g	c	g	t	a	a	a	c	c	c	t	g	g	g	a	t	c	c	c	a	c	g	t	t	c	c	t	g	a	c	g	a	c	t	a	t	g	t	c	c	a	c	g	g	g	t
AJ871403	3	c	c	a	c	a	c	c	c	a	g	g	c	g	g	c	c	a	a	c	t	g	a	t	t	t	g	t	t	g	c	g	c	a	a	a	c	c	c	t	t	t	g	a	t	c	c	c	a	c	g	t	t	c	c	t	g	a	c	g	a	a	t	a	t	g	t	c	t	a	c	g	g	a	t
DQ479955	3	c	c	a	c	g	c	c	c	a	g	g	c	g	g	c	c	a	a	c	t	g	a	c	t	t	g	t	t	g	c	g	c	a	a	a	c	c	c	t	t	g	g	a	t	c	c	c	a	c	g	t	t	c	c	t	g	a	c	g	a	c	t	a	t	g	t	c	t	a	c	g	g	a	t
DQ479956	3	a	c	a	c	g	c	c	c	a	g	g	c	g	g	c	c	a	a	c	t	g	a	t	t	t	g	t	t	g	c	g	c	a	a	a	c	c	c	t	t	g	g	a	t	c	c	c	a	c	g	t	t	c	c	c	g	a	c	g	a	c	t	a	t	g	t	c	t	a	c	g	g	a	t
DQ479957	3	c	c	a	c	g	c	c	c	a	g	g	c	g	g	c	c	a	a	c	t	g	a	t	t	t	g	t	t	g	c	g	c	a	a	a	c	c	c	t	t	g	g	a	t	c	c	c	a	c	g	t	t	c	c	t	g	a	c	g	a	c	t	a	t	g	t	c	t	a	c	g	g	a	t
JN704699	3	c	c	a	c	g	c	c	c	a	g	g	c	g	g	c	c	a	a	c	t	g	a	t	t	t	g	t	t	g	c	g	g	a	a	a	c	c	c	t	t	g	g	c	t	c	c	c	a	c	g	t	t	c	c	t	g	a	c	g	a	c	t	a	t	g	t	c	t	a	c	g	g	a	t
JN704700	3	c	c	a	c	g	c	c	c	a	g	g	c	g	g	c	c	a	a	c	t	g	a	t	g	t	g	t	t	g	c	g	c	a	a	a	c	c	c	t	t	g	g	a	t	c	c	c	a	c	g	t	t	c	c	t	g	a	c	g	a	c	t	a	t	g	t	c	t	a	c	g	g	a	t
JN704701	3	c	c	a	c	g	c	c	c	a	g	g	c	g	g	c	c	a	a	c	t	g	a	t	t	t	g	t	t	g	c	g	c	a	a	a	c	c	c	t	t	g	g	a	t	c	c	c	a	c	g	t	t	c	c	t	g	a	c	g	a	c	t	a	t	g	t	c	t	a	c	g	g	a	t
JN704702	3	c	c	a	c	g	c	c	c	a	g	g	c	g	g	c	c	a	a	c	t	g	a	t	t	t	g	t	t	g	c	g	c	a	a	a	c	c	c	t	t	g	g	a	t	c	c	c	a	c	g	t	t	c	c	t	g	a	c	g	a	c	t	a	t	g	t	c	t	a	c	g	g	a	t
JN704703	3	c	c	a	c	g	c	c	c	a	a	g	c	g	g	c	c	a	a	c	t	g	a	t	t	t	g	t	t	g	c	g	c	a	a	a	c	c	c	t	t	g	g	a	t	c	c	c	a	c	g	t	t	c	c	t	g	a	c	g	a	c	t	a	t	g	t	c	t	a	c	g	g	a	t
KP771891	3	c	c	a	c	g	c	c	c	a	g	g	c	g	g	c	c	a	a	c	t	g	a	t	t	t	g	t	t	g	c	g	c	a	a	a	c	c	c	t	t	g	g	a	t	c	c	c	a	c	g	t	t	c	c	t	g	a	c	g	a	c	t	a	t	g	t	c	t	a	c	g	g	a	t
KP771893	3	c	c	a	c	g	c	c	c	a	g	g	c	g	g	c	c	a	a	c	t	g	a	t	t	t	g	t	t	g	c	g	c	a	a	a	c	c	c	t	t	g	g	a	t	c	c	c	a	c	g	c	t	c	c	t	g	a	c	g	a	c	t	a	t	g	t	c	t	a	c	g	g	a	t
KP771895	3	c	c	a	c	a	c	c	c	a	g	g	c	g	g	c	c	a	a	c	t	g	a	t	t	t	g	t	t	g	c	g	c	a	a	a	c	c	c	t	t	g	g	a	t	c	c	c	a	c	g	t	t	c	c	t	g	a	c	g	a	c	t	a	t	g	t	c	t	a	c	g	g	a	t
KP771896	3	c	c	a	c	g	c	c	c	a	g	g	c	g	g	c	c	a	a	c	t	g	a	t	t	t	g	t	t	g	c	g	c	a	a	a	c	c	c	t	t	g	g	a	t	c	c	c	a	c	g	t	t	c	c	t	g	a	c	g	a	c	t	a	t	g	t	c	t	a	c	g	g	a	t
KP771897	3	c	c	a	c	g	c	c	c	a	g	g	c	g	g	c	c	a	a	c	t	g	a	t	t	t	g	t	t	g	c	g	c	a	a	a	c	c	c	t	t	g	g	a	t	c	c	c	a	c	g	t	t	c	c	t	g	a	c	g	a	c	t	a	t	g	t	c	t	a	c	g	g	a	t
KP771899	3	c	c	a	c	a	c	c	c	a	g	g	c	g	g	c	c	a	a	c	t	g	a	t	t	t	g	t	t	g	c	g	c	a	a	a	c	c	c	t	t	g	g	a	t	c	c	c	a	c	g	t	t	c	c	t	g	a	c	g	a	c	t	a	t	g	t	c	t	a	c	g	g	a	t
KP771903	3	c	c	a	c	g	c	c	c	a	g	g	c	g	g	c	t	a	a	c	t	g	a	t	t	t	g	t	t	g	c	g	c	a	a	a	c	c	c	t	t	g	g	a	t	c	c	c	a	c	g	t	t	c	c	t	g	a	c	g	a	c	t	a	t	g	t	c	t	a	c	g	g	a	t
KP771907	3	c	c	a	c	a	c	c	c	a	g	g	c	g	g	c	c	a	a	c	t	g	a	t	t	t	g	t	t	g	c	g	c	a	a	a	c	c	c	t	t	g	g	a	t	c	c	c	a	c	g	t	t	c	c	t	g	a	c	g	a	c	t	a	t	g	t	c	t	a	c	g	g	a	t
KP771910	3	c	c	a	c	g	c	c	c	a	g	g	c	g	g	c	c	a	a	c	t	g	a	t	t	t	g	t	t	g	c	g	c	a	a	a	c	c	c	t	t	g	g	a	t	c	c	c	a	c	g	c	t	c	c	t	g	a	c	g	a	c	t	a	t	g	t	c	t	a	c	g	g	a	t
KP771915	3	c	c	a	c	g	c	c	c	a	g	g	c	g	g	c	c	a	a	c	t	g	a	t	t	t	g	t	t	g	c	g	c	a	a	a	c	c	c	t	t	g	g	a	t	c	c	c	a	c	g	t	t	c	c	t	g	a	c	g	a	c	t	a	t	g	t	c	t	a	c	g	g	a	t
KP771916	3	c	c	a	c	g	c	c	c	a	g	g	c	g	g	c	c	a	a	c	t	g	a	t	t	t	g	t	t	g	c	g	c	a	a	a	c	c	c	t	t	g	g	a	t	c	c	c	a	c	g	t	t	c	c	c	g	c	c	g	a	c	t	a	t	g	t	c	t	a	c	g	g	a	t
KP771921	3	c	c	a	c	g	c	c	c	a	g	g	c	g	g	c	c	a	a	c	t	g	a	t	t	t	g	t	t	g	c	g	c	a	a	a	c	c	c	t	t	g	g	a	t	c	c	c	a	c	g	t	t	c	c	t	g	a	c	g	a	c	t	a	t	g	t	c	t	a	c	g	g	a	t
KP771923	3	c	c	a	c	g	c	c	c	a	g	g	c	g	g	c	c	a	a	c	t	g	a	t	t	t	g	t	t	g	c	g	c	a	a	a	c	c	c	t	t	g	g	a	t	c	c	c	a	c	g	t	t	c	c	t	g	a	c	g	a	c	t	a	t	g	t	c	t	a	c	g	g	a	t
KP771889	3	c	c	a	c	g	c	c	c	a	g	g	c	g	g	c	c	a	a	c	t	g	a	t	t	t	g	t	t	g	c	g	c	a	a	a	c	c	c	t	t	g	g	a	t	c	c	c	a	c	g	t	t	c	c	t	g	a	c	g	a	c	t	a	t	g	t	c	t	a	c	g	g	a	t
KU926311	3	c	c	a	c	g	c	c	c	a	g	g	t	g	g	c	c	a	a	c	t	g	a	t	t	t	g	t	t	g	c	g	c	a	a	a	c	c	c	t	t	g	g	a	t	c	c	c	a	c	g	t	t	c	c	t	g	a	c	g	a	c	t	a	t	g	t	c	t	a	c	g	g	a	t
JQ911599	3	c	c	a	c	g	c	c	c	a	g	g	c	c	g	c	c	a	a	c	t	g	a	t	t	t	g	t	t	g	c	g	c	a	a	a	c	c	c	t	t	g	g	a	t	c	c	c	a	c	g	t	t	c	c	t	g	a	c	g	a	c	t	a	t	g	t	c	t	a	c	g	g	a	t
MH709317	3	c	c	a	c	g	c	c	c	a	g	g	c	g	g	c	c	a	a	c	t	g	a	t	t	t	g	t	t	g	c	g	c	a	a	a	c	c	c	t	t	g	g	a	t	c	c	c	a	c	g	t	t	c	c	t	g	a	c	g	a	c	t	a	t	g	t	c	t	a	c	g	g	a	t
MH709346	3	c	c	a	c	g	c	c	c	a	g	g	c	g	g	c	c	a	a	c	t	g	a	t	t	t	g	t	t	g	c	g	c	a	a	a	c	c	c	t	t	g	g	a	t	c	c	c	a	c	g	t	t	c	c	t	g	a	c	g	a	c	t	a	t	g	t	c	t	a	c	g	g	a	t
MH709375	3	c	c	a	c	g	c	c	c	a	g	g	c	g	g	c	c	a	a	c	t	g	a	t	t	t	g	t	t	g	c	g	c	a	a	a	c	c	c	t	t	g	g	a	t	c	c	c	a	c	g	t	t	c	c	t	g	a	c	g	a	c	t	a	t	g	t	c	t	a	c	g	g	a	t
DQ452050	4	c	c	a	t	g	c	c	c	a	g	g	c	g	g	c	c	a	a	c	a	g	g	t	t	t	g	t	t	g	c	g	t	a	a	a	c	c	c	c	g	g	g	a	t	c	c	c	a	c	g	t	t	c	c	t	g	a	c	g	a	c	t	a	t	g	t	c	c	a	c	g	g	a	t
DQ479960	4	c	c	a	c	g	c	c	c	a	g	g	c	g	g	c	c	a	a	c	a	g	g	t	t	t	g	t	t	g	c	g	t	a	a	a	c	c	c	t	g	g	g	a	t	c	c	c	a	c	g	t	t	c	c	t	g	a	c	g	a	c	t	a	t	g	t	c	c	a	c	g	g	a	t
KP771898	4	c	c	a	c	g	c	c	t	a	g	g	c	g	g	c	c	a	a	c	a	g	g	t	t	t	g	t	t	g	c	g	t	a	a	a	c	c	c	t	g	g	g	a	t	c	c	c	a	c	g	t	t	c	c	t	g	a	c	g	a	c	t	a	t	g	t	c	c	a	c	g	g	a	t
MH709316	4	c	c	a	c	g	c	c	c	a	g	g	c	g	g	c	c	a	a	c	a	g	g	t	t	t	g	t	t	g	c	g	t	a	a	a	c	c	c	t	g	g	g	a	t	c	c	c	a	c	g	t	t	c	c	t	g	a	c	g	a	c	t	a	t	g	t	c	c	a	c	g	g	a	t
MH709318	4	c	c	a	c	g	c	c	c	a	g	g	c	g	g	c	c	a	a	c	a	g	g	t	t	t	g	t	t	g	c	a	t	a	a	a	c	c	c	t	g	g	g	a	t	c	c	c	a	c	g	t	t	c	c	t	g	a	c	g	a	c	t	a	t	g	t	c	c	a	c	g	g	a	t
MH709322	4	c	c	a	c	g	c	c	c	a	g	g	c	g	g	c	c	a	a	c	a	g	g	t	t	t	g	t	t	g	c	a	t	a	a	a	c	c	c	t	g	g	g	a	t	c	c	c	a	c	g	t	t	c	c	t	g	a	c	g	a	c	t	a	t	g	t	c	c	a	c	g	g	a	t
MH709323	4	c	c	a	c	g	c	c	c	a	g	g	c	g	g	c	c	a	a	c	a	g	g	t	t	t	g	t	t	g	c	a	t	a	a	a	c	c	c	t	g	g	g	a	t	c	c	c	a	c	g	t	t	c	c	t	g	a	c	g	a	c	t	a	t	g	t	c	c	a	c	g	g	a	t
MH709326	4	c	c	a	c	g	c	c	c	a	g	g	c	g	g	c	c	a	a	c	a	g	g	t	t	t	g	t	t	g	c	a	t	a	a	a	c	c	c	t	g	g	g	a	t	c	c	c	a	c	g	t	t	c	c	t	g	a	c	g	a	c	t	a	t	g	t	c	c	a	c	g	g	a	t
MH709341	4	c	c	a	c	g	c	c	c	a	g	g	c	g	g	c	c	a	a	c	a	g	g	t	t	t	g	g	t	g	c	g	t	a	a	a	c	c	c	t	g	g	g	a	t	c	c	c	a	c	g	t	t	c	c	t	g	a	c	g	a	c	t	a	t	g	t	c	c	a	a	g	g	a	t
MH709357	4	c	c	a	c	g	c	c	c	a	g	g	c	g	g	c	c	a	a	c	a	g	g	t	t	t	g	t	t	g	c	g	t	a	a	a	c	c	c	t	g	g	g	a	t	c	c	c	a	c	g	t	t	c	c	t	g	a	c	g	a	c	t	a	t	g	t	c	c	a	c	g	g	a	t
MH709364	4	c	c	a	c	g	c	c	c	a	g	g	c	g	g	c	c	a	a	c	a	g	g	t	t	t	g	t	t	g	c	g	t	a	a	a	c	c	c	t	g	g	g	a	t	c	c	c	a	c	g	t	t	c	c	t	g	a	c	g	a	c	t	a	t	g	t	c	c	a	c	g	g	a	t
MH709370	4	c	c	a	c	g	c	c	c	a	g	g	c	g	g	c	c	a	a	c	a	g	g	t	t	t	g	t	t	g	c	g	t	a	a	a	c	c	c	t	g	g	g	a	t	c	c	c	a	c	g	t	t	c	c	t	g	a	c	g	a	c	t	a	t	g	t	c	c	a	c	g	g	a	t
MH709371	4	c	c	a	c	g	c	c	c	a	g	g	c	g	g	c	c	a	a	c	a	g	g	t	t	t	g	t	t	g	c	g	t	a	a	a	c	c	c	t	g	g	g	a	t	c	c	c	a	c	g	t	t	c	c	t	g	a	c	g	a	c	t	a	t	g	t	c	c	a	c	g	g	a	t
MH709372	4	c	c	a	c	g	c	c	c	a	g	g	c	g	g	c	c	a	a	c	a	g	g	t	t	t	g	t	t	g	c	g	t	a	a	a	c	c	c	t	g	g	g	a	t	c	c	c	a	c	g	t	t	c	c	t	g	a	c	g	a	c	t	a	t	g	t	c	c	a	c	g	g	a	t
DQ457052	5	c	c	a	c	g	c	c	c	a	g	g	c	g	g	t	c	a	a	c	t	g	a	t	t	t	g	t	t	g	c	g	c	a	a	a	c	c	c	t	t	g	g	a	t	c	c	c	a	c	g	t	t	c	c	t	g	a	c	g	a	c	t	a	t	g	t	c	c	a	c	g	g	a	t
JN704704	5	c	c	a	c	g	c	c	c	a	g	g	c	g	g	t	c	a	a	c	t	g	a	t	t	t	g	t	t	g	c	g	c	a	a	a	c	c	c	t	t	g	g	a	t	c	c	c	a	c	g	t	t	c	c	t	g	a	c	g	a	c	t	a	t	g	t	c	c	a	c	g	g	a	t
JN704705	5	c	c	a	c	g	c	c	c	a	g	g	c	g	g	t	c	a	a	c	t	g	a	t	t	t	g	t	t	g	c	g	c	a	a	a	a	c	c	t	t	g	g	a	t	c	c	c	a	c	g	t	t	c	c	t	g	a	c	g	a	c	t	a	t	g	t	c	c	a	c	g	g	a	t
JN704706	5	c	c	a	c	g	c	c	c	g	g	g	c	g	g	t	c	a	a	c	t	g	a	t	t	t	g	t	t	g	c	g	c	a	a	a	c	t	c	t	t	g	g	a	t	c	c	c	a	c	g	t	t	c	c	t	g	a	c	g	a	c	t	a	t	g	t	c	c	a	c	g	g	a	t
JN704707	5	c	c	a	c	g	c	c	c	a	g	g	c	g	g	t	c	c	a	c	t	g	a	t	t	t	g	t	t	g	c	g	c	a	a	a	c	c	c	t	t	g	a	a	t	c	c	c	a	c	g	t	t	c	c	t	g	a	c	g	a	c	t	a	t	g	t	c	c	a	c	g	g	a	t
JN704708	5	c	c	a	c	g	c	c	c	a	g	g	c	g	g	t	c	a	a	c	t	g	a	t	t	t	g	t	t	g	c	g	c	a	a	a	a	c	c	t	t	g	g	a	t	c	c	c	a	c	g	t	t	c	c	t	g	a	c	g	a	c	t	a	t	g	t	c	c	a	c	g	g	a	t
KP771904	5	c	c	a	c	g	c	c	c	a	g	g	c	g	g	t	c	a	a	c	t	g	a	t	t	t	g	t	t	g	c	g	c	a	a	c	c	c	c	t	t	g	g	a	t	c	c	c	a	c	g	t	t	c	c	t	g	a	c	g	a	c	t	a	t	g	t	c	c	a	c	g	g	a	t
KP771905	5	c	t	a	c	g	c	c	c	a	g	g	c	g	g	t	c	a	a	c	t	g	a	t	t	t	g	t	t	g	c	g	c	a	a	a	c	c	c	t	t	g	g	a	t	c	c	c	a	c	g	t	t	c	t	t	g	a	c	g	a	c	t	a	t	g	t	c	c	a	c	g	g	a	t
KP771906	5	c	t	a	c	g	c	c	c	a	g	g	c	g	g	t	c	a	a	c	t	g	a	t	t	t	g	t	t	g	c	g	c	a	a	a	c	c	c	t	t	g	g	a	t	c	c	c	a	c	g	t	t	c	t	t	g	a	c	g	a	c	t	a	t	g	t	c	c	a	c	g	g	a	t
KP771919	5	c	c	a	c	g	c	c	c	a	g	g	c	g	g	t	c	a	a	c	t	g	a	t	t	t	g	t	t	g	c	g	c	a	a	c	c	c	c	t	t	g	g	a	t	c	c	c	a	c	g	t	t	c	c	t	g	a	c	g	a	c	t	a	t	g	t	c	c	a	c	g	g	a	t
KP771922	5	c	c	a	c	g	c	c	c	a	g	g	c	g	g	t	c	c	a	c	t	g	a	t	t	t	g	t	t	g	c	g	c	a	a	a	c	c	c	t	t	g	a	a	t	c	c	c	a	c	g	t	t	c	c	t	g	a	c	g	a	c	t	a	t	g	t	c	c	a	c	g	g	a	t
KP771924	5	c	c	a	c	g	c	c	c	a	g	g	c	g	g	t	c	a	a	c	t	g	a	t	t	t	g	t	t	g	c	g	c	a	a	a	c	c	c	t	t	g	g	a	t	c	c	c	a	c	g	t	t	c	c	t	g	a	c	g	g	c	t	a	t	g	t	c	c	a	c	g	g	a	t
KM355696	5	c	c	a	c	g	c	c	c	a	g	g	c	g	g	t	c	a	a	c	t	g	a	t	t	t	g	t	t	g	c	g	c	a	a	a	c	c	c	t	t	g	g	a	t	c	c	c	a	c	g	t	t	c	c	t	g	a	c	g	g	c	t	a	t	g	t	c	c	a	c	g	g	a	t
KM355697	5	c	c	a	c	g	c	c	c	a	g	g	c	g	g	t	c	a	a	c	t	g	a	t	t	t	g	t	t	g	c	g	c	a	a	a	c	c	c	t	t	g	g	a	t	c	c	c	a	c	g	t	t	c	c	t	g	a	c	g	a	c	t	a	t	g	t	c	c	a	c	g	g	a	t
KM355698	5	c	c	a	c	g	c	c	c	a	g	g	c	g	g	t	c	a	a	c	t	g	a	t	t	t	g	t	t	g	c	g	c	a	a	a	c	c	c	t	t	g	g	a	t	c	c	c	a	c	g	t	t	c	c	t	g	a	c	g	a	c	t	a	t	g	t	c	c	a	c	g	g	a	t
KM355699	5	c	c	a	c	g	c	c	c	a	g	g	c	g	g	t	c	a	a	c	t	g	a	t	t	t	g	t	t	g	c	g	c	a	a	a	c	c	c	t	t	g	g	a	t	c	c	c	a	c	g	t	t	c	c	t	g	a	c	g	g	c	t	a	t	g	t	c	c	a	c	g	g	a	t
KM355701	5	c	c	a	c	g	c	c	c	a	g	g	c	g	g	t	c	a	a	c	t	g	a	t	t	t	g	t	t	g	c	g	c	a	a	a	c	c	c	t	t	g	g	a	t	c	c	c	a	c	g	t	t	c	c	t	g	a	c	g	a	c	t	a	t	g	t	c	c	a	c	g	g	a	t
KM355702	5	c	c	a	c	g	c	c	c	a	g	g	c	g	g	t	c	a	a	c	t	g	a	t	t	t	g	t	t	g	c	g	c	a	a	a	c	c	c	t	t	g	g	a	t	c	c	c	a	c	g	t	t	c	c	t	g	a	c	g	g	c	t	a	t	g	t	c	c	a	c	g	g	a	t
KM355703	5	c	c	a	c	g	c	c	c	a	g	g	c	g	g	t	c	a	a	c	t	g	a	t	t	t	g	t	t	g	c	g	c	a	a	a	c	c	c	t	t	g	g	a	t	c	c	c	a	c	g	t	t	c	c	t	g	a	c	g	g	c	t	a	t	g	t	c	c	a	c	g	g	a	t
KM355704	5	c	c	a	c	g	c	c	c	a	g	g	c	g	g	t	c	a	a	c	t	g	a	t	t	t	g	t	t	g	c	g	c	a	a	a	c	c	c	t	t	g	g	a	t	c	c	c	a	c	g	t	t	c	c	t	g	a	c	g	a	c	t	a	t	g	t	c	c	a	c	g	g	a	t
KM355705	5	c	c	a	c	g	c	c	c	a	g	g	c	g	g	t	c	a	a	c	t	g	a	t	t	t	g	t	t	g	c	g	c	a	a	a	c	c	c	t	t	g	g	a	t	c	c	c	a	c	g	t	t	c	c	t	g	a	c	g	a	c	t	a	t	g	t	c	c	a	c	g	g	a	t
KM355706	5	c	c	a	c	g	c	c	c	a	g	g	c	g	g	t	c	a	a	c	t	g	a	t	t	t	g	t	t	g	c	g	c	a	a	a	c	c	c	t	t	g	g	a	t	c	c	c	a	c	g	t	t	c	c	t	g	a	c	g	a	c	t	a	t	g	t	c	c	a	c	g	g	a	t
KM355707	5	c	c	a	c	g	c	c	c	a	g	g	c	g	g	t	c	a	a	c	t	g	a	t	t	t	g	t	t	g	c	g	c	a	a	a	c	c	c	t	t	g	g	a	t	c	c	c	a	c	g	t	t	c	c	t	g	a	c	g	g	c	t	a	t	g	t	c	c	a	c	g	g	a	t
KM355708	5	c	c	a	c	g	c	c	c	a	g	g	c	g	g	t	c	a	a	c	t	g	a	t	t	t	g	t	t	g	c	g	c	a	a	a	c	c	c	t	t	g	g	a	t	c	c	c	a	c	g	t	t	c	c	t	g	a	c	g	a	c	t	a	t	g	t	c	c	a	c	g	g	a	t
KM355709	5	c	c	a	c	g	c	c	c	a	g	g	c	g	g	t	c	a	a	c	t	g	a	t	t	t	g	t	t	g	c	g	c	a	a	a	c	c	c	t	t	g	g	a	t	c	c	c	a	c	g	t	t	c	c	t	g	a	c	g	a	c	t	a	t	g	t	c	c	a	c	g	g	a	t
KM355711	5	c	c	a	c	g	c	c	c	a	g	g	c	g	g	t	c	a	a	c	t	g	a	t	t	t	g	t	t	g	c	g	c	a	a	a	c	c	c	t	t	g	g	a	t	c	c	c	a	c	g	t	t	c	c	t	g	a	c	g	a	c	t	a	t	g	t	c	c	a	c	g	g	a	t
KM355712	5	c	c	a	c	g	c	c	c	a	g	g	c	g	g	t	c	a	a	c	t	g	a	t	t	t	g	t	t	g	c	g	c	a	a	a	c	c	c	t	t	g	g	a	t	c	c	c	a	c	g	t	t	c	c	t	g	a	c	g	a	c	t	a	t	g	t	c	c	a	c	g	g	a	t
[illegible]	[illegible]	[illegible]	[illegible]	[illegible]	[illegible]	[illegible]	[illegible]	[illegible]	[illegible]	[illegible]	[illegible]	[illegible]	[illegible]	[illegible]	[illegible]	[illegible]	[illegible]	[illegible]	[illegible]	[illegible]	[illegible]	[illegible]	[illegible]	[illegible]	[illegible]	[illegible]	[illegible]	[illegible]	[illegible]	[illegible]	[illegible]	[illegible]	[illegible]	[illegible]	[illegible]	[illegible]	[illegible]	[illegible]	[illegible]	[illegible]	[illegible]	[illegible]	[illegible]	[illegible]	[illegible]	[illegible]	[illegible]	[illegible]	[illegible]	[illegible]	[illegible]	[illegible]	[illegible]	[illegible]	[illegible]	[illegible]	[illegible]	[illegible]	[illegible]	[illegible]	[illegible]	[illegible]	[illegible]	[illegible]	[illegible]	[illegible]	[illegible]	[illegible]	[illegible]	[illegible]	[illegible]	[illegible]	[illegible]	[illegible]	[illegible]

Supplementary Table 3: 2880 SN

GB-ID	Clade markers															5			SD14																												1	1																6											SD14	
	Position in Dumas	30769	30844	30859	30871	30881	30947	30948	31057	31075	31124	31289	31331	31356	31475	31619	31820	31946	31976	32021	32255	32269	32274	32513	32604	32684	32846	32879	32881	32963	32987	32990	33068	33095	33100	33173	33176	33212	33224	33287	33473	33563	33646	33654	33655	33722	33725	33728	33732	33749	33773	33832	33927	33958	34293	34466	34562	34567	34595	34613	34619	34682	34875	35007	35022	35048	35051	35088	35141	35200	35223	35225	35226	35243	35287
MH499467	5	c	c	a	c	g	c	c	c	a	g	g	c	g	g	t	c	a	a	c	t	a	a	t	t	t	g	t	g	g	c	g	c	a	a	a	c	c	c	t	t	g	g	a	t	c	c	c	a	c	g	t	t	c	c	t	g	a	c	g	a	c	t	a	t	g	t	c	c	a	-	-	-	-	t
MH499469	5	c	c	a	c	g	c	c	c	a	g	a	c	g	g	t	c	a	a	c	t	g	a	t	t	t	g	t	t	g	c	g	c	a	a	c	c	c	c	t	t	g	g	a	t	c	c	c	a	c	g	t	t	c	c	t	g	a	c	g	a	c	t	a	t	g	t	c	c	a	c	g	g	a	t
MH709321	5	c	c	a	c	g	c	c	c	g	g	g	c	g	g	t	c	a	a	c	t	g	a	t	t	t	g	t	t	g	c	g	c	a	a	a	c	c	c	t	t	g	g	a	t	c	c	c	a	c	g	t	t	c	c	t	g	a	c	g	a	c	t	a	t	g	t	c	c	a	c	g	g	a	t
MH709324	5	c	c	a	c	g	c	c	c	a	g	g	c	g	g	t	c	a	a	c	t	g	a	t	t	t	g	t	t	g	c	g	c	a	a	c	c	c	c	t	t	g	g	a	t	c	c	c	a	c	g	t	t	c	c	t	g	a	c	g	a	c	t	a	t	g	t	c	c	a	c	g	g	a	t
MH709325	5	c	c	a	c	g	c	c	c	a	g	g	c	g	g	t	c	a	a	c	t	g	a	t	t	t	g	t	t	g	c	g	c	a	a	a	c	c	c	t	t	g	g	a	t	c	c	c	a	c	g	t	t	c	c	t	g	a	c	g	a	c	t	a	t	g	t	c	c	a	c	g	g	a	t
MH709327	5	c	c	a	c	g	c	c	c	a	g	g	c	g	g	t	c	a	a	c	t	g	a	t	t	t	g	t	t	g	c	g	c	a	a	a	c	c	c	t	t	g	g	a	t	c	c	c	a	c	g	t	t	c	c	t	g	a	c	g	a	c	t	a	t	g	t	c	c	a	c	g	g	a	t
MH709328	5	c	c	a	c	g	c	c	c	a	g	g	c	g	g	t	c	a	a	c	t	g	a	t	t	t	g	t	t	g	c	g	c	a	a	c	c	c	c	t	t	g	g	a	t	c	c	c	a	c	g	t	t	c	c	t	g	a	c	g	a	c	t	a	t	g	t	c	c	a	c	g	g	a	t
MH709330	5	c	c	a	c	g	c	c	c	a	g	g	c	g	g	t	c	a	a	c	t	g	a	t	t	t	g	t	t	g	c	g	c	a	a	c	c	c	c	t	t	g	g	a	t	c	c	c	a	c	g	t	t	c	c	t	g	a	c	g	a	c	t	a	t	g	t	c	c	a	c	g	g	a	t
MH709332	5	c	c	a	c	g	c	c	c	a	g	g	c	g	g	t	c	a	a	c	t	g	a	t	t	t	g	t	t	g	c	g	c	a	a	c	c	c	c	t	t	g	g	a	t	c	c	c	a	c	g	t	t	c	c	t	g	a	c	g	a	c	t	a	t	g	t	c	c	a	c	g	g	a	t
MH709334	5	c	c	a	c	g	c	c	c	a	g	g	c	g	g	t	c	a	a	c	t	g	a	t	t	t	g	t	t	g	c	g	c	a	a	a	c	c	c	t	t	g	g	a	t	c	c	c	a	c	g	t	t	c	c	t	g	a	c	g	a	c	t	a	t	g	t	c	c	a	c	g	g	a	t
MH709335	5	c	c	a	c	g	c	c	c	a	g	g	c	g	g	t	c	a	a	c	t	g	a	t	t	t	g	t	t	g	c	g	c	a	a	a	c	c	c	t	t	g	g	a	t	c	c	c	a	c	g	t	t	c	c	t	g	a	c	g	a	c	t	a	t	g	t	c	c	a	c	g	g	a	t
MH709336	5	c	c	a	c	g	c	c	c	a	g	g	c	g	g	t	c	a	a	c	t	g	a	t	t	t	g	t	t	g	c	g	c	a	a	a	c	c	c	t	t	g	g	a	t	c	c	c	a	c	g	t	t	c	c	t	g	a	c	g	a	c	t	a	t	g	t	c	c	a	c	g	g	a	t
MH709338	5	c	c	a	c	g	c	c	c	a	g	g	c	g	g	t	c	a	a	c	t	g	a	t	t	t	g	t	t	g	c	g	c	a	a	a	c	c	c	t	t	g	g	a	t	c	c	c	a	c	g	t	t	c	c	t	g	a	c	g	a	c	t	a	t	g	t	c	c	a	c	g	g	a	t
MH709340	5	c	c	a	c	g	c	c	c	a	g	g	c	g	g	t	c	a	a	c	t	g	a	t	t	t	g	t	t	g	c	g	c	a	a	a	c	c	c	t	t	g	g	a	t	c	c	c	a	c	g	t	t	c	c	t	g	a	c	g	a	c	t	a	t	g	t	c	c	a	c	g	g	a	t
MH709342	5	c	c	a	c	g	c	c	c	g	g	g	c	g	g	t	c	a	a	c	t	g	a	t	t	t	g	t	t	g	c	g	c	a	a	a	c	c	c	t	t	g	g	a	t	c	c	c	a	c	g	t	t	c	c	t	g	a	c	g	a	c	t	a	t	g	t	c	c	a	c	g	g	a	t
MH709343	5	c	c	a	c	g	c	c	c	a	g	g	c	g	g	t	c	a	a	c	t	g	a	t	t	t	g	t	t	g	c	g	c	a	a	a	c	c	c	t	t	g	g	a	t	c	c	c	a	c	g	t	t	c	c	t	g	a	c	g	a	c	t	a	t	g	t	c	c	a	c	g	g	a	t
MH709345	5	c	c	a	c	g	c	c	c	a	g	g	c	g	g	t	c	a	a	c	t	g	a	t	t	t	g	t	t	g	c	g	c	a	a	a	c	c	c	t	t	g	g	a	t	c	c	c	a	c	g	t	t	c	c	t	g	a	c	g	a	c	t	a	t	g	t	c	c	a	c	g	g	a	t
MH709347	5	c	c	a	c	g	c	c	c	a	g	g	c	g	g	t	c	a	a	c	t	g	a	t	t	t	g	t	t	g	c	g	c	a	a	a	c	c	c	t	t	g	g	a	t	c	c	c	a	c	g	t	t	c	c	t	g	a	c	g	a	c	t	a	t	g	t	c	c	a	c	g	g	a	t
MH709349	5	c	c	a	c	g	c	c	c	a	g	g	c	g	g	t	c	a	a	t	t	g	a	t	t	t	g	t	t	g	c	g	c	a	a	a	c	c	c	t	t	g	g	a	t	c	c	c	a	c	g	t	t	c	c	t	g	a	c	g	a	c	t	a	t	g	t	c	c	a	c	g	g	a	t
MH709350	5	c	c	a	c	g	c	c	c	a	g	g	c	g	g	t	c	a	a	c	t	g	a	t	t	t	g	t	t	g	c	g	c	a	a	a	c	c	c	t	t	g	g	a	t	c	c	c	a	c	g	t	t	c	c	t	g	a	c	g	a	c	t	a	t	g	t	c	c	a	c	g	g	a	t
MH709351	5	c	c	a	c	g	c	c	c	a	g	g	c	g	g	t	c	a	a	c	t	g	a	t	t	t	g	t	t	g	t	g	c	a	a	a	c	c	c	t	t	g	g	a	t	c	c	c	a	c	g	t	t	c	c	t	a	a	c	g	a	c	t	a	t	g	t	c	c	a	c	g	g	a	t
MH709353	5	c	c	a	c	g	c	c	c	a	g	g	c	g	g	t	c	a	a	c	t	g	a	t	t	t	g	t	t	g	c	g	c	a	a	a	c	c	c	t	t	g	g	a	t	c	c	c	a	c	g	t	t	c	c	t	g	a	c	g	a	c	t	a	t	g	t	c	c	a	c	g	g	a	t
MH709358	5	c	c	a	c	g	c	c	c	a	g	g	c	g	g	t	c	a	a	c	t	g	a	t	t	t	g	t	t	g	c	g	c	a	a	a	c	c	c	t	t	g	g	a	t	c	c	c	a	c	g	t	t	c	c	t	g	a	c	g	a	c	t	a	t	g	t	c	c	a	c	g	g	a	t
MH709360	5	c	c	a	c	g	c	c	c	a	g	g	c	g	g	t	c	a	a	c	t	g	a	t	t	t	g	t	t	g	c	g	c	a	a	c	c	c	c	t	t	g	g	a	t	c	c	c	a	c	g	t	t	c	c	t	g	a	c	g	a	c	t	a	t	g	t	c	c	a	c	g	g	a	t
MH709362	5	c	c	a	c	g	c	c	c	a	g	g	c	g	g	t	c	a	a	c	t	g	a	t	t	t	g	t	t	g	c	g	c	a	a	a	c	c	c	t	t	g	g	a	t	c	c	c	a	c	g	t	t	c	c	t	g	a	c	g	a	c	t	a	t	g	t	c	c	a	c	g	g	a	t
MH709365	5	c	c	a	c	g	c	c	c	a	g	g	c	g	g	t	c	a	a	c	t	g	a	t	t	t	g	t	t	g	c	g	c	a	a	a	c	c	c	t	t	g	g	a	t	c	c	c	a	c	g	t	t	c	c	t	g	a	c	g	a	c	t	a	t	g	t	c	c	a	c	g	g	a	t
MH709373	5	c	c	a	c	g	c	c	c	a	g	g	c	g	g	t	c	c	a	c	t	g	a	t	t	t	g	t	t	g	c	g	c	c	a	a	c	c	c	t	t	g	a	a	t	c	c	c	a	t	g	t	t	c	c	t	g	a	c	g	a	c	t	a	t	g	t	c	c	a	c	g	g	a	t
MH709374	5	c	c	c	c	g	c	c	c	a	g	g	c	g	g	t	c	a	a	c	t	g	a	t	t	t	g	t	t	g	c	g	c	a	a	a	c	c	c	t	t	g	g	a	t	c	c	c	a	c	g	t	t	c	c	t	g	a	c	g	a	c	t	a	t	g	t	c	c	a	c	g	g	a	t
KC112914	6	c	c	a	c	g	c	c	c	a	g	g	c	g	g	c	c	a	a	c	t	g	a	t	t	t	g	t	t	g	c	g	c	a	a	a	c	c	c	t	t	g	g	a	t	c	c	c	a	c	g	t	t	c	c	t	g	a	c	g	a	c	a	a	t	g	t	c	t	a	c	g	g	a	t
KP771914	6	c	c	a	c	g	c	c	c	a	g	g	c	g	g	c	c	a	a	c	t	g	a	t	t	t	g	t	t	g	c	g	c	a	a	a	c	c	c	t	t	g	g	a	t	c	c	c	a	c	g	t	t	c	c	t	g	a	c	g	a	c	a	a	t	g	t	c	t	a	c	g	g	a	t
KP702725	6	c	c	a	c	g	c	c	c	a	g	g	c	g	g	c	c	a	a	c	t	g	a	t	t	t	g	t	t	g	c	g	c	a	a	a	c	c	c	t	t	g	g	a	t	c	c	c	a	c	g	t	t	c	c	t	g	a	c	g	a	c	a	a	t	g	t	c	t	a	c	g	g	a	t
KP771890	9	c	c	a	c	g	c	c	c	a	g	g	c	g	g	c	c	a	a	c	a	g	a	t	t	t	g	t	t	g	c	g	t	a	a	a	c	c	c	t	g	g	g	a	t	c	c	c	a	c	g	t	t	c	c	t	g	a	c	g	a	c	t	a	t	g	t	c	c	a	c	g	g	a	t
JN704710	9	c	c	a	c	g	c	c	c	a	g	g	c	g	g	c	c	a	a	c	a	g	a	t	t	t	g	t	t	g	c	g	t	a	a	a	c	c	c	t	g	g	g	a	t	c	c	c	a	c	g	t	t	c	c	t	g	a	c	g	a	c	t	a	t	g	t	c	c	a	c	g	g	a	t
KY037798	9	c	c	a	c	g	c	c	c	a	g	g	c	g	g	c	c	a	a	c	a	g	a	t	t	t	g	t	t	g	c	g	t	a	a	a	c	c	c	t	g	g	g	a	c	c	c	c	a	c	g	t	t	c	c	t	g	a	c	g	a	c	t	a	t	g	t	c	c	a	c	g	g	a	t
MH709312	9	c	c	a	c	g	c	c	c	a	g	g	c	g	g	c	c	a	a	c	a	g	a	t	t	t	g	t	t	g	c	g	t	a	a	a	c	c	c	t	g	g	g	a	c	c	c	c	a	c	g	t	t	c	c	t	g	a	c	g	a	c	t	a	t	g	t	c	c	a	c	g	g	a	t
MH709352	9	c	c	a	c	g	c	c	c	a	g	g	c	g	g	c	c	a	a	c	a	g	a	t	t	t	g	t	t	g	c	g	t	a	a	a	c	c	c	t	g	g	g	a	t	c	c	c	a	c	g	t	t	c	c	t	g	a	c	g	a	c	t	a	t	g	t	c	c	a	c	g	g	a	t
AB097933	Vac	c	c	a	c	g	c	c	c	a	g	g	c	g	g	c	c	a	a	c	a	g	g	t	t	t	g	t	t	g	c	g	t	a	a	a	c	c	c	t	g	g	g	a	t	c	c	c	a	c	g	t	t	c	c	t	g	a	c	g	a	c	t	a	t	g	t	c	c	a	c	g	g	a	t
JN704698	Vac	c	c	a	c	g	c	c	c	a	g	g	c	g	g	c	c	a	a	c	a	g	g	t	t	t	g	t	t	g	c	g	t	a	a	a	c	c	c	t	g	g	g	a	t	c	c	c	a	c	g	t	t	c	c	t	g	a	c	g	a	c	t	a	t	g	t	c	c	a	c	g	g	a	t
JN704697	Vac	c	c	a	c	g	c	c	c	a	g	g	c	g	g	c	c	a	a	c	a	g	g	t	t	t	g	t	t	g	c	g	t	a	a	a	c	c	c	t	g	g	g	a	t	c	c	c	a	c	g	t	t	c	c	t	g	a	c	g	a	c	t	a	t	g	t	c	c	a	c	g	g	a	t
DQ008355	Vac	c	c	a	c	g	c	c	c	a	g	g	c	g	g	c	c	a	a	c	a	g	g	t	t	t	g	t	t	g	c	g	t	a	a	a	c	c	c	t	g	g	g	a	t	c	c	c	a	c	g	t	t	c	c	t	g	a	c	g	a	c	t	a	t	g	t	c	c	a	c	g	g	a	t
JF306641	Vac	c	c	a	c	g	c	c	c	a	g	g	c	g	g	c	c	a	a	c	a	g	g	t	t	t	g	t	t	g	c	g	t	a	a	a	c	c	c	t	g	g	g	a	t	c	c	c	a	c	g	t	t	c	c	t	g	a	c	g	a	c	t	a	t	g	t	c	c	a	c	g	g	a	t
AB097932	Vac	c	c	a	c	g	c	c	c	a	g	g	c	g	g	c	c	a	a	c	a	g	g	t	t	t	g	t	t	g	c	g	t	a	a	a	c	c	c	t	g	g	g	a	t	c	c	c	a	c	g	t	t	c	c	t	g	a	c	g	a	c	t	a	t	g	t	c	c	a	c	g	g	a	t
DQ008354	Vac	c	c	a	c	g	c	c	c	a	g	g	c	g	g	c	c	a	a	c	a	g	g	t	t	t	g	t	t	g	c	g	t	a	a	a	c	c	c	t	g	g	g	a	t	c	c	c	a	c	g	t	t	c	c	t	g	a	c	g	a	c	t	a	t	g	t	c	c	a	c	g	g	a	t
KU926312	Vac	c	c	a	c	g	c	c	c	a	g	g	c	g	g	c	c	a	a	c	a	g	g	t	t	t	g	t	t	g	c	g	t	a	a	a	c	c	c	t	g	g	g	a	t	c	c	c	a	c	g	t	t	c	c	t	g	a	c	g	a	c	t	a	t	g	t	c	c	a	c	g	g	a	t
KU926313	Vac	c	c	a	c	g	c	c	c	a	g	g	c	g	g	c	c	a	a	c	a	g	g	t	t	t	g	t	t	g	c	g	t	a	a	a	c	c	c	t	g	g	g	a	t	c	c	c	a	c	g	t	t	c	c	t	g	a	c	g	a	c	t	a	t	g	t	c	c	a	c	g	g	a	t
KU926314	Vac	c	c	a	c	g	c	c	c	a	g	g	c	g	g	c	c	a	a	c	a	g	g	t	t	t	g	t	t	g	c	g	t	a	a	a	c	c	c	t	g	g	g	a	t	c	c	c	a	c	g	t	t	c	c	t	g	a	c	g	a	c	t	a	t	g	t	c	c	a	c	g	g	a	t
KF558371	Vac	c	c	a	c	g	c	c	c	a	g	g	c	g	g	c	c	a	a	c	a	g	g	t	t	t	g	t	t	g	c	g	t	a	a	a	c	c	c	t	g	g	g	a	t	c	c	c	a	c	g	t	t	c	c	t	g	a	c	g	a	c	t	a	t	g	t	c	c	a	c	g	g	a	t
KF558372	Vac	c	c	a	c	g	c	c	c	a	g	g	c	g	g	c	c	a	a	c	a	g	g	t	t	t	g	t	t	g	c	g	t	a	a	a	c	c	c	t	g	g	g	a	t	c	c	c	a	c	g	t	t	c	c	t	g	a	c	g	a	c	t	a	t	g	t	c	c	a	c	g	g	a	t
KF558373	Vac	c	c	a	c	g	c	c	c	a	g	g	c	g	g	c	c	a	a	c	a	g	g	t	t	t	g	t	t	g	c	g	t	a	a	a	c	c	c	t	g	g	g	a	t	c	c	c	a	c	g	t	t	c	c	t	g	a	c	g	a	c	t	a	t	g	t	c	c	a	c	g	g	a	t
KF558374	Vac	c	c	a	c	g	c	c	c	a	g	g	c	g	g	c	c	a	a	c	a	g	g	t	t	t	g	t	t	g	c	g	t	a	a	a	c	c	c	t	g	g	g	a	t	c	c	c	a	c	g	t	t	c	c	t	g	a	c	g	a	c	t	a	t	g	t	c	c	a	c	g	g	a	t
KF558375	Vac	c	c	a	c	g	c	c	c	a	g	g	c	g	g	c	c	a	a	c	a	g	g	t	t	t	g	t	t	g	c	g	t	a	a	a	c	c	c	t	g	g	g	a	t	c	c	c	a	c	g	t	t	c	c	t	g	a	c	g	a	c	t	a	t	g	t	c	c	a	c	g	g	a	t
KF558376	Vac	c	c	a	c	g	c	c	c	a	g	g	c	g	g	c	c	a	a	c	a	g	g	t	t	t	g	t	t	g	c	g	t	a	a	a	c	c	c	t	g	g	g	a	t	c	c	c	a	c	g	t	t	c	c	t	g	a	c	g	a	c	t	a	t	g	t	c	c	a	c	g	g	a	t
KF558377	Vac	c	c	a	c	g	c	c	c	a	g	g	c	g	g	c	c	a	a	c	a	g	g	t	t	t	g	t	t	g	c	g	t	a	a	a	c	c	c	t	g	g	g	a	t	c	c	c	a	c	g	t	t	c	c	t	g	a	c	g	a	c	t	a	t	g	t	c	c	a	c	g	g	a	t
KF558378	Vac	c	c	a	c	g	c	c	c	a	g	g	c	g	g	c	c	a	a	c	a	g	g	t	t	c	g	t	t	g	c	g	t	a	a	a	c	c	c	t	g	g	g	a	t	c	c	c	a	c	g	t	t	c	c	t	g	a	c	g	a	c	t	a	t	g	t	c	c	a	c	g	g	a	t
KF558379	Vac	c	c	a	c	g	c	c	c	a	g	g	c	g	g	c	c	a	a	c	a	g	g	t	t	c	g	t	t	g	c	g	t	a	a	a	c	c	c	t	g	g	g	a	t	c	c	c	a	c	g	t	t	c	c	t	g	a	c	g	a	c	t	a	t	g	t	c	c	a	c	g	g	a	t
KF558380	Vac	c	c	a	c	g	c	c	c	a	g	g	c	g	g	c	c	a	a	c	a	g	g	t	t	t	g	t	t	g	c	g	t	a	a	a	c	c	c	t	g	g	g	a	t	c	c	c	a	c	g	t	t	c	c	t	g	a	c	g	a	c	t	a	t	g	t	c	c	a	c	g	g	a	t
KF558381	Vac	c	c	a	c	g	c	c	c	a	g	g	c	g	g	c	c	a	a	c	a	g	g	t	t	t	g	t	t	g	c	g	t	a	a	a	c	c	c	t	g	g	g	a	t	c	c	c	a	c	g	t	t	c	c	t	g	a	c	g	a	c	t	a	t	g	t	c	c	a	c	g	g	a	t
KF558382	Vac	c	c	a	c	g	c	c	c	a	g	g	c	g	g	c	c	a	a	c	a	g	g	t	t	t	g	t	t	g	c	g	t	a	a	a	c	c	c	t	g	g	g	a	t	c	c	c	a	c	g	t	t	c	c	t	g	a	c	g	a	c	t	a	t	g	t	c	c	a	c	g	g	a	t
KF558383	Vac	c	c	a	c	g	c	c	c	a	g	g	c	g	g	c	c	a	a	c	a	g	g	t	t	t	g	t	t	g	c	g	t	a	a	a	c	c	c	t	g	g	g	a	t	c	c	c	a	c	g	t	t	c	c	t	g	a	c	g	a	c	t	a	t	g	t	c	c	a	c	g	g	a	t
KF558384	Vac	c	c	a	c	g	c	c	c	a	g	g	c	g	g	c	c	a	a	c	a	g	g	t	t	t	g	t	t	g	c	g	t	a	a	a	c	c	c	t	g	g	g	a	t	c	c	c	a	c	g	t	t	c	c	t	g	a	c	g	a	c	t	a	t	g	t	c	c	a	c	g	g	a	t
KF558385	Vac	c	c	a	c	g	c	c	c	a	g	g	c	g	g	c	c	a	a	c	a	g	g	t	t	t	g	t	t	g	c	g	t	a	a	a	c	c	c	t	g	g	g	a	t	c	c	c	a	c	g	t	t	c	c	t	g	a	c	g	a	c	t	a	t	g	t	c	c	a	c	g	g	a	t
KF558386	Vac	c	c	a	c	g	c	c	c	a	g	g	c	g	g	c	c	a	a	c	a	g	g	t	t	t	g	t	t	g	c	g	t	a	g	a	c	c	c	t	g	g	g	a	t	c	c	c	a	c	g	t	t	c	c	t	g	a	c	g	a	c	t	a	t	g	t	c	c	a	c	g	g	a	t
KF558387	Vac	c	c	a	c	g	c	c	c	a	g	g	c	g	g	c	c	a	a	c	a	g	g	t	t	t	g	t	t	g	c	g	t	a	a	a	c	c	c	t	g	g	g	a	t	c	c	c	a	c	g	t	t	c	c	t	g	a	c	g	a	c	t	a	t	g	t	c	c	a	c	g	g	a	t
KF558388	Vac	c	c	a	c	g	c	c	c	a	g	g	c	g	g	c	c	a	a	c	a	g	g	t	t	t	g	t	t	g	c	g	t	a	a	a	c	c	c	t	g	g	g	a	t	c	c	c	a	c	g	t	t	c	c	t	g	a	c	g	a	c	t	a	t	g	t	c	c	a	c	g	g	a	t
KF558389	Vac	c	c	a	c	g	c	c	c	a	g	g	c	g	g	c	c	a	a	c	a	g	g	t	t	t	g	t	t	g	c	g	t	a	a	a	c	c	c	t	g	g	g	a	t	c	c	c	a	c	g	t	t	c	c	t	g	a	c	g	a	c	t	a	t	g	t	c	c	a	c	g	g	a	t
KF558390	Vac	c	c	a	c	g	c	c	c	a	g	g	c	g	g	c	c	a	a	c	a	g	g	t	t	t	g	t	t	g	c	g	t	a	a	a	c	c	c	t	g	g	g	a	t	c	c	c	a	c	g	t	t	c	c	t	g	a	c	g	a	c	t	a	t	g	t	c	c	a	c	g	g	a	t
KF558391	Vac	c	c	a	c	g	c	c	c	a	g	g	c	g	g	c	c	a	a	c	a	g	g	t	t	t	g	t	t	g	c	g	t	a	a	a	c	c	c	t	g	g	g	a	t	c	c	c	a	c	g	t	t	c	c	t	g	a	c	g	a	c	t	a	t	g	t	c	c	a	c	g	g	a	t
KF811485	Vac	c	c	a	c	g	c	c	c	a	g	g	c	g	g	c	c	a	a	c	a	g	g	t	t	t	g	t	t	g	c	g	t	a	a	a	c	c	c	t	g	g	g	a	t	c	c	c	a	c	g	t	t	c	c	t	g	a	c	g	a	c	t	a	t	g	t	c	c	a	c	g	g	a	t
KF853225	Vac	c	c	a	c	g	c	c	c	a	g	g	c	g	g	c	c	a	a	c	a	g	g	t	t	t	g	t	t	g	c	g	t	a	a	a	c	c	c	t	g	g	g	a	t	c	c	c	a	c	g	t	t	c	c	t	g	a	c	g	a	c	t	a	t	g	t	c	c	a	c	g	g	a	t
KF853226	Vac	c	c	a	c	g	c	c	c	a	g	g	c	g	g	c	c	a	a	c	a	g	g	t	t	t	g	t	t	g	c	g	t	a	a	a	c	c	c	t	g	g	g	a	t	c	c	c	a	c	g	t	t	c	c	t	g	a	c	g	a	c	t	a	t	g	t	c	c	a	c	g	g	a	t
KF853227	Vac	c	c	a	c	g	c	c	c	a	g	g	c	g	g	c	c	a	a	c	a	g	g	t	t	t	g	t	t	g	c	g	t	a	a	a	c	c	c	t	g	g	g	a	t	c	c	c	a	c	g	t	t	c	c	t	g	a	c	g	a	c	t	a	t	g	t	c	c	a	c	g	g	a	t
KF853228	Vac	c	c	a	c	g	c	c	c	a	g	g	c	g	g	c	c	a	a	c	a	g	g	t	t	t	g	t	t	g	c	g	t	a	a	a	c	c	c	t	g	g	g	a	t	c	c	c	a	c	g	t	t	c	c	t	g	a	c	g	a	c	t	a	t	g	t	c	c	a	c	g	g	a	t
KF853229	Vac	c	c	a	c	g	c	c	c	a	g	g	c	g	g	c	c	a	a	c	a	g	g	t	t	t	g	t	t	g	c	g	t	a	a	a	c	c	c	t	g	g	g	a	t	c	c	c	a	c	g	t	t	c	c	t	g	a	c	g	a	c	t	a	t	g	t	c	c	a	c	g	g	a	t
KF853230	Vac	c	c	a	c	g	c	c	c	a	g	g	c	g	g	c	c	a	a	c	a	g	g	t	t	t	g	t	t	g	c	g	t	a	a	a	c	c	c	t	g	g	g	a	t	c	c	c	a	c	g	t	t	c	c	t	g	a	c	g	a	c	t	a	t	g	t	c	c	a	c	g	g	a	t
KF853231	Vac	c	c	a	c	g	c	c	c	a	g	g	c	g	g	c	c	a	a	c	a	g	g	t	t	t	g	t	t	g	c	g	t	a	g	a	c	c	c	t	g	g	g	a	t	c	c	c	a	c	g	t	t	c	c	t	g	a	c	g	a	c	t	a	t	g	t	c	c	a	c	g	g	a	t
KF853232	Vac	c	c	a	c	g	c	c	c	a	g	g	c	g	g	c	c	a	a	c	a	g	g	t	t	t	g	t	t	g	c	g	t	a	a	a	c	c	c	t	g	g	g	a	t	c	c	c	a	c	g	t	t	c	c	t	g	a	c	g	a	c	t	a	t	g	t	c	c	a	c	g	g	a	t
KF853233	Vac	c	c	a	c	g	c	c	c	a	g	g	c	g	g	c	c	a	a	c	a	g	g	t	t	t	g	t	t	g	c	g	t	a	a	a	c	c	c	t	g	g	g	a	t	c	c	c	a	c	g	t	t	c	c	t	g	a	c	g	a	c	t	a	t	g	t	c	c	a	c	g	g	a	t
KF853234	Vac	c	c	a	c	g	c	c	c	a	g	g	c	g	g	c	c	a	a	c	a	g	g	t	t	t	g	t	t	g	c	g	t	a	a	a	c	c	c	t	g	g	g	a	t	c	c	c	a	c	g	t	t	c	c	t	g	a	c	g	a	c	t	a	t	g	t	c	c	a	c	g	g	a	t
KF853235	Vac	c	c	a	c	g	c	c	c	a	g	g	c	g	g	c	c	a	a	c	a	g	g	t	t	t	g	t	t	g	c	g	t	a	a	a	c	c	c	t	g	g	g	a	t	c	c	c	a	c	g	t	t	c	c	t	g	a	c	g	a	c	t	a	t	g	t	c	c	a	c	g	g	a	t
MF898328	Vac	c	c	a	c	g	c	c	c	a	g	g	c	g	g	c	c	a	a	c	a	g	g	t	t	t	g	t	t	g	c	g	t	a	a	a	c	c	c	t	g	g	g	a	t	c	c	c	a	c	g	t	t	c	c	t	g	a	c	g	a	c	t	a	t	g	t	c	c	a	c	g	g	a	t
JN704709	VIII	c	c	a	c	g	c	c	c	a	g	g	c	g	g	c	c	a	a	c	a	g	g	t	t	t	g	t	t	g	c	g	t	a	a	a	c	c	c	t	g	g	g	a	t	c	c	c	a	c	g	t	t	c	c	t	g	a	c	g	a	c	t	a	t	g	t	c	c	a	c	g	g	a	t

Supplementary Table 3: 2880 SN

GB-ID	Clade markers											4																												
	Position in Dumas	35291	35332	35543	35579	35589	35642	35658	35735	35819	35836	35963	35971	36005	36047	36063	36109	36131	36271	36623	36659	36801	36926	37063	37176	37211	37241	37260	37282	37285	37337	37349	37426	37447	37479	37522	37649	37775	37783	37811	37814
AY548170	1	g	c	t	t	a	t	t	g	t	c	t	t	t	t	g	g	a	c	a	a	g	a	c	c	a	t	g	g	c	c	t	t	t	c	a	a	g	a	a	t
AY548171	1	g	c	t	t	a	t	t	g	t	c	t	t	t	t	g	g	a	c	a	a	g	a	c	c	a	t	g	g	c	c	t	t	t	c	a	a	g	a	a	t
DQ479953	1	g	c	t	t	a	t	t	g	t	c	t	t	t	t	g	g	a	c	a	a	g	a	c	c	a	t	g	g	c	c	t	t	t	c	a	a	g	a	a	t
DQ479954	1	g	c	t	t	a	t	t	g	t	c	t	t	t	t	g	g	a	c	a	a	g	a	c	c	a	t	g	g	c	c	t	t	t	c	a	a	g	a	a	t
DQ479958	1	g	g	t	t	a	t	t	g	t	c	t	t	t	t	g	g	a	c	a	a	g	a	c	c	a	t	g	g	c	c	t	t	t	c	a	a	g	a	a	t
DQ479959	1	g	g	t	t	a	t	t	g	t	c	t	t	t	t	g	g	a	c	a	a	g	a	c	c	a	t	g	g	c	c	t	t	t	c	a	a	g	a	a	t
DQ479961	1	g	c	t	t	a	t	t	g	t	c	t	t	t	t	g	g	a	c	a	a	g	a	c	c	a	t	g	g	c	c	t	t	t	c	a	a	g	a	a	t
DQ479962	1	g	c	t	t	a	t	t	g	t	c	t	t	t	t	g	g	a	c	a	a	g	a	c	c	a	t	g	g	c	c	t	t	t	c	a	a	g	a	a	t
DQ479963	1	g	c	t	t	a	t	t	g	t	c	t	t	t	t	g	g	a	c	a	a	g	a	c	c	a	t	g	g	c	c	t	t	t	c	a	a	g	a	a	t
DQ674250	1	g	c	t	t	a	t	t	g	t	c	t	t	t	t	g	g	a	c	a	a	g	a	c	c	a	t	g	g	c	c	t	t	t	c	a	a	g	a	a	t
KX262864	1	g	c	t	t	a	t	t	g	t	c	t	t	t	t	g	g	a	c	a	a	g	a	c	c	a	t	g	g	c	c	t	t	t	c	a	a	g	a	a	t
KX262865	1	g	c	t	t	a	t	t	g	t	c	t	t	t	t	g	g	a	c	a	a	g	a	c	c	a	t	g	g	c	c	t	t	t	c	a	a	g	a	a	t
KX262866	1	g	c	t	t	a	t	t	g	t	c	t	t	t	t	g	g	a	c	a	a	g	a	t	c	a	t	g	g	c	c	t	t	t	c	a	a	g	a	a	t
EU154348	1	g	c	t	t	a	t	t	g	t	c	t	t	t	t	g	g	a	c	a	a	g	a	c	c	a	t	g	g	c	c	t	t	t	c	a	a	g	a	a	t
JN704690	1	g	c	t	t	a	t	t	g	t	c	t	t	t	t	g	g	a	c	a	a	g	a	c	c	a	t	g	g	c	c	t	t	t	c	a	a	g	a	a	t
JN704691	1	g	c	t	t	a	t	t	g	t	c	t	t	t	t	g	g	a	c	a	a	g	a	c	c	a	t	g	g	c	c	t	t	t	c	a	a	g	a	a	t
JN704692	1	g	c	t	t	a	t	t	g	t	c	t	t	t	t	g	g	a	c	c	a	g	a	c	c	a	t	g	g	c	c	t	t	t	c	a	a	g	a	a	t
JN704693	1	g	c	t	t	a	t	t	g	t	c	t	t	t	t	g	g	a	c	a	a	g	a	c	c	a	t	g	g	c	c	g	t	t	c	a	a	g	a	a	t
JN704694	1	g	c	t	t	a	t	t	g	t	c	t	t	t	t	g	g	a	c	a	a	g	a	c	c	a	t	g	g	c	c	t	t	t	c	a	a	g	a	a	t
JN704695	1	g	c	t	t	a	t	t	g	t	c	t	t	t	t	g	g	a	c	a	a	g	a	c	c	a	t	g	g	c	c	t	t	t	c	a	a	g	a	a	t
JN704696	1	g	c	t	t	a	t	t	g	t	c	t	t	t	t	g	g	a	c	a	a	g	a	c	c	a	t	g	g	c	c	t	t	t	c	a	a	g	a	a	t
NC001348	1	g	c	t	t	a	t	t	g	t	c	t	t	t	t	g	g	a	c	a	a	g	a	c	c	a	t	g	g	c	c	t	t	t	c	a	a	g	a	a	t
KP771892	1	g	c	t	t	a	t	t	g	t	c	t	t	t	t	g	g	a	c	a	a	g	a	c	c	a	t	g	g	c	c	t	t	t	c	a	a	g	a	a	t
KP771894	1	g	c	t	t	a	t	t	g	t	c	t	t	t	t	g	g	a	c	a	a	g	a	c	c	a	t	g	g	c	c	t	t	t	c	a	a	g	a	a	t
KP771900	1	g	c	t	t	a	t	t	g	t	c	t	t	t	t	g	g	a	c	a	a	g	a	c	c	a	t	g	g	c	c	t	t	t	c	a	a	g	a	a	t
KP771901	1	g	c	t	t	a	t	t	g	t	c	t	t	t	t	g	g	a	c	a	a	g	a	c	c	a	t	g	g	c	c	t	t	t	c	a	a	g	a	a	t
KP771902	1	g	c	t	t	a	t	t	g	t	c	t	t	t	t	g	g	a	c	a	a	g	a	c	c	a	t	g	g	c	c	t	t	t	c	a	a	g	a	a	t
KP771908	1	g	c	t	t	a	t	t	g	t	c	t	t	t	t	g	g	a	c	a	a	g	a	c	c	a	t	g	g	c	c	t	t	t	c	a	a	g	a	a	t
KP771912	1	g	c	t	t	a	t	t	g	t	c	t	t	t	t	g	g	a	c	a	a	g	a	c	c	a	t	g	g	c	c	t	t	t	c	a	a	g	a	a	t
KP771913	1	g	c	t	t	a	t	t	g	t	c	t	t	t	t	g	g	a	c	a	a	g	a	c	c	a	t	g	g	c	c	t	t	t	c	a	a	g	a	a	t
KP771917	1	g	c	t	t	a	t	t	g	t	c	t	t	t	c	g	g	a	c	a	a	g	a	c	c	a	t	g	g	c	c	t	t	t	c	a	a	g	a	a	t
KP771918	1	g	c	t	t	a	t	t	g	t	c	t	t	t	t	g	g	a	c	a	a	g	a	c	c	a	t	g	g	c	c	t	t	t	c	a	a	g	a	a	t
KP771920	1	g	c	t	t	a	t	t	g	t	c	t	t	t	t	g	g	a	c	a	a	g	a	c	c	a	t	g	g	c	c	t	t	t	c	a	a	g	a	a	t
MH709361	1	g	c	t	t	a	t	t	g	t	c	t	t	t	t	g	g	a	c	a	a	g	a	c	c	a	t	g	g	c	c	t	t	t	c	a	a	g	a	a	t
KU926318	2	g	c	c	t	a	t	t	g	t	c	t	t	t	t	g	g	a	c	a	a	g	a	c	c	g	t	g	g	c	c	t	t	c	c	a	g	g	a	a	t
KU926319	2	g	c	c	t	a	t	t	g	t	c	t	t	t	t	g	g	a	c	a	a	g	g	c	c	a	t	g	g	c	c	t	t	t	c	a	g	g	a	a	t
KU926320	2	g	c	c	t	a	t	t	g	t	c	t	t	t	t	g	g	a	c	a	a	g	g	c	c	a	t	g	g	c	c	t	t	t	c	a	g	g	a	a	t
KU926321	2	g	c	c	t	a	t	t	g	t	c	t	t	t	t	g	g	a	c	a	a	g	g	c	c	a	t	g	g	c	c	t	t	t	c	a	g	g	a	a	t
KU926322	2	g	c	c	t	g	t	t	g	t	c	t	t	t	t	g	g	a	c	a	a	g	g	c	c	a	t	g	g	c	c	t	t	t	c	a	g	g	a	a	t
KC847290	2	g	c	c	t	a	t	t	g	t	c	t	t	t	t	g	g	a	c	a	a	g	a	c	c	a	t	g	g	c	c	t	t	t	c	a	g	g	a	a	t
KJ767491	2	g	c	c	t	a	t	t	g	t	c	t	t	t	t	g	g	a	c	a	a	g	a	c	c	a	t	g	g	c	c	t	t	t	c	a	g	g	a	a	t
KJ767492	2	g	c	c	t	a	t	t	g	t	c	t	t	t	t	g	g	a	c	a	a	g	a	c	c	a	t	g	g	c	c	t	t	t	c	a	g	g	a	a	t
KJ808816	2	g	c	c	t	a	t	t	g	t	c	t	t	t	t	g	g	a	c	a	a	g	g	c	c	a	t	g	g	c	c	t	t	t	c	a	g	g	a	a	t
KP771925	2	g	c	c	t	a	t	t	g	t	c	t	t	t	t	g	g	a	c	a	a	g	a	c	c	a	t	g	g	c	c	t	t	t	c	a	g	g	a	a	t
KU926315	2	g	c	c	t	a	t	t	g	t	c	t	t	t	t	g	g	a	c	a	a	g	a	c	c	a	t	g	g	c	c	t	t	t	c	a	g	g	a	a	t
KU926316	2	g	c	c	t	a	t	t	g	t	c	t	t	t	t	g	g	a	c	a	a	g	a	c	c	a	t	g	g	c	c	t	t	t	c	a	g	g	a	a	t
KU926317	2	g	c	c	t	a	t	t	g	t	c	t	t	t	t	g	g	a	c	a	a	g	a	c	c	a	t	g	g	c	c	t	t	t	c	a	g	g	a	a	t
KY037796	2	g	c	c	t	a	t	t	g	t	c	t	t	t	t	g	g	a	c	a	a	g	a	c	c	a	t	g	g	c	c	t	t	t	c	a	g	g	a	a	t
MF004348	2	g	c	c	t	a	t	t	g	t	c	t	t	t	t	g	g	a	c	a	a	g	g	c	c	a	t	g	g	c	c	t	t	t	c	a	g	g	a	a	t
MH709309	2	g	c	c	t	a	t	t	g	t	c	t	t	t	t	g	g	a	c	a	a	g	a	c	c	a	t	g	g	c	c	t	t	t	c	a	g	g	a	a	t
MH709310	2	g	c	c	t	a	t	t	g	t	c	t	t	t	t	g	g	a	c	a	a	g	a	c	c	a	t	g	g	c	c	t	t	t	c	a	g	g	a	a	t
MH709311	2	g	c	c	t	a	t	t	g	t	c	t	t	t	t	g	g	a	c	a	a	g	a	c	c	a	t	g	g	c	c	t	t	t	c	a	a	g	a	a	t
MH709313	2	g	c	c	t	a	t	t	g	t	c	t	t	t	t	g	g	a	c	a	a	g	a	c	c	a	t	g	g	c	c	t	t	t	c	a	g	g	a	a	t
MH709314	2	g	c	c	t	a	t	t	g	t	c	t	t	t	t	g	g	a	c	a	a	g	a	c	a	a	t	g	g	c	c	t	t	t	c	a	g	g	a	a	t
MH709315	2	g	c	c	t	a	t	t	g	t	c	t	t	t	t	g	g	a	c	a	a	g	a	c	c	a	t	g	g	c	c	t	t	t	c	a	g	g	a	a	t
MH709319	2	g	c	c	t	a	t	t	g	t	c	t	t	t	t	g	g	a	c	a	a	g	a	c	c	a	t	g	g	c	c	t	t	t	c	a	g	g	a	a	t
MH709320	2	g	c	c	t	a	t	t	g	t	c	t	t	t	t	g	g	a	c	a	a	g	a	c	c	a	t	g	g	c	c	t	t	t	c	a	g	g	a	a	t
MH709329	2	g	c	c	t	a	t	t	g	t	c	t	t	t	t	g	g	a	c	a	a	g	a	c	c	a	t	g	g	a	c	t	t	t	c	a	g	g	a	a	t
MH709331	2	g	c	c	t	a	t	t	g	t	c	t	t	t	t	g	g	a	c	a	a	g	a	c	c	a	t	g	g	c	c	t	t	t	c	a	g	g	a	a	t
MH709333	2	g	c	c	t	a	t	t	g	t	c	t	t	t	t	g	g	a	c	a	a	g	a	c	c	a	t	g	g	c	c	t	t	t	c	a	g	g	a	a	t
MH709337	2	g	c	c	t	a	t	t	g	t	c	t	t	t	t	g	g	a	c	a	a	g	a	c	c	a	t	g	g	c	c	t	t	t	c	a	g	g	a	a	t
MH709339	2	g	c	c	t	a	t	t	g	t	c	t	t	t	t	g	g	a	c	a	a	g	a	c	c	a	t	g	g	c	c	t	t	t	c	a	g	g	a	a	t
MH709344	2	g	c	c	t	a	t	t	g	t	c	t	t	t	t	g	g	a	c	a	a	g	a	c	c	a	t	g	g	c	c	t	t	t	c	a	g	g	a	a	t
MH709348	2	g	c	c	t	a	t	t	g	t	c	t	t	t	t	g	g	a	c	a	a	g	a	c	c	a	t	a	g	c	c	t	t	t	c	a	g	g	a	a	t
MH709355	2	g	c	c	t	a	t	t	g	t	c	t	t	t	t	g	g	a	c	a	a	g	a	c	c	a	t	g	g	c	c	t	t	t	c	a	g	g	a	a	t
MH709359	2	g	c	c	t	a	t	t	g	t	c	t	t	t	t	g	g	a	c	a	a	g	a	c	c	a	t	g	g	c	c	t	t	t	c	a	g	g	a	a	c
MH709363	2	g	c	c	t	a	t	t	g	t	c	t	t	t	t	g	g	a	c	a	a	g	a	c	c	a	t	g	g	c	c	t	t	t	c	a	g	g	a	a	t
MH709366	2	g	c	c	t	a	t	t	g	t	c	t	t	t	t	g	g	a	c	a	a	g	a	c	c	a	t	g	g	c	c	t	c	t	c	a	g	g	a	a	c
MH709376	2	g	c	c	t	a	t	t	g	t	c	t	t	t	t	g	g	a	c	a	a	g	a	c	c	a	t	g	g	c	c	t	t	t	c	a	g	g	a	a	t
MH709377	2	g	c	c	t	a	t	t	g	t	c	t	t	t	t	g	g	a	c	a	a	g	g	c	c	a	t	g	g	c	c	t	t	t	c	a	g	g	a	a	t
**MW316406**	2	g	c	c	t	a	t	t	g	t	c	t	t	t	t	g	g	a	c	a	a	g	a	c	c	a	t	g	g	c	c	t	t	t	c	a	g	g	a	a	t
AJ871403	3	g	c	c	t	a	t	t	g	t	c	t	t	t	t	g	g	a	c	a	a	g	a	c	c	a	t	g	g	c	c	t	t	t	c	a	a	g	a	a	t
DQ479955	3	g	c	c	t	a	t	t	g	t	c	t	t	t	t	g	g	a	c	a	a	g	a	c	c	a	t	g	g	c	c	t	t	t	c	a	a	g	a	a	t
DQ479956	3	g	c	c	t	a	t	t	g	t	c	t	t	t	t	g	g	a	c	a	a	g	a	c	c	a	t	g	g	c	c	t	t	t	c	a	a	g	a	a	t
DQ479957	3	g	c	c	t	a	t	t	g	t	c	t	t	t	t	g	g	a	c	a	a	g	a	c	c	a	t	g	g	c	c	t	t	t	c	a	a	g	a	a	t
JN704699	3	g	c	c	t	a	t	t	g	t	c	t	t	t	t	g	g	a	c	a	a	g	a	c	c	a	t	g	g	c	c	t	t	t	c	a	a	g	a	a	t
JN704700	3	g	c	c	t	a	t	t	g	t	c	t	t	t	t	g	g	a	c	a	a	g	a	c	c	a	t	g	g	c	c	t	t	t	c	a	a	g	a	a	t
JN704701	3	g	c	c	t	a	c	t	g	t	c	t	t	t	t	g	g	a	c	a	a	g	a	c	c	a	t	g	g	c	c	t	t	t	c	a	a	g	a	a	t
JN704702	3	g	c	c	t	a	t	t	g	t	c	t	t	t	t	g	g	a	c	a	a	g	a	c	c	a	t	g	g	c	c	t	t	t	c	a	a	g	a	a	t
JN704703	3	g	c	c	t	a	t	t	g	t	c	t	t	t	t	g	g	a	c	a	a	g	a	c	c	a	t	g	g	c	t	t	t	t	c	a	a	g	a	a	t
KP771891	3	g	c	c	t	a	t	t	g	t	c	t	t	t	t	g	g	a	c	a	a	g	a	c	c	a	t	g	g	c	c	t	t	t	c	a	a	g	a	a	t
KP771893	3	g	c	c	t	a	t	t	g	t	c	t	t	t	t	g	g	a	c	a	g	g	a	c	c	a	t	g	g	c	c	t	t	t	c	a	a	g	a	a	t
KP771895	3	g	c	c	t	a	t	t	g	t	c	t	t	t	t	g	g	a	c	a	a	g	a	c	c	a	t	g	g	c	c	t	t	t	c	a	a	g	a	a	t
KP771896	3	g	c	c	t	a	t	t	g	t	c	t	t	t	t	g	g	a	c	a	a	g	a	c	c	a	t	g	g	c	c	t	t	t	c	a	a	g	a	a	t
KP771897	3	g	c	c	t	a	t	t	g	t	c	t	t	t	t	g	g	a	c	a	a	g	a	c	c	a	t	g	g	c	c	t	t	t	c	a	a	g	a	a	t
KP771899	3	g	c	c	t	a	t	t	g	t	c	t	t	t	t	g	g	a	c	a	a	g	a	c	c	a	t	g	g	c	c	t	t	t	c	a	a	g	a	a	t
KP771903	3	g	c	c	t	a	t	t	g	t	c	t	t	t	t	g	g	a	c	a	a	g	a	c	c	a	t	g	g	c	c	t	t	t	c	a	a	g	a	a	t
KP771907	3	g	c	c	t	a	t	t	g	t	c	t	t	t	t	g	g	a	c	a	a	g	a	c	c	a	t	g	g	c	c	t	t	t	c	a	a	g	a	a	t
KP771910	3	g	c	c	t	a	t	t	g	t	c	t	t	t	t	g	g	a	c	a	g	g	a	c	c	a	t	g	g	c	c	t	t	t	c	a	a	g	a	a	t
KP771915	3	g	c	c	t	a	t	t	g	t	c	t	t	t	t	g	g	a	c	a	a	g	a	c	c	a	t	g	g	c	c	t	t	t	c	a	a	g	a	a	t
KP771916	3	g	c	c	t	a	t	t	g	t	c	t	t	t	t	g	g	a	c	a	a	g	a	c	c	a	t	g	g	c	c	t	t	t	c	a	a	g	a	a	t
KP771921	3	g	c	c	t	a	t	t	g	t	c	t	t	t	t	g	g	a	c	a	a	g	a	c	c	a	t	g	g	c	c	t	t	t	c	a	a	g	a	a	t
KP771923	3	g	c	c	t	a	t	t	g	t	c	t	t	t	t	g	g	a	c	a	a	g	a	c	c	a	t	g	g	c	c	t	t	t	c	a	a	g	a	a	t
KP771889	3	g	c	c	t	a	t	t	g	t	c	t	t	t	t	g	g	a	c	a	a	g	a	c	c	a	t	g	g	c	c	t	t	t	c	a	a	g	a	a	t
KU926311	3	g	c	c	t	a	t	t	g	t	c	t	t	t	t	g	g	a	c	a	a	g	a	c	c	a	t	g	g	c	c	t	t	t	c	a	a	g	a	a	t
JQ911599	3	g	c	c	t	a	t	t	g	t	c	t	t	t	t	g	g	a	c	a	a	g	a	c	c	a	t	g	g	c	c	t	t	t	c	a	a	g	a	a	t
MH709317	3	g	c	c	t	a	t	t	g	t	c	t	t	t	t	g	g	a	c	a	a	g	a	c	c	a	t	g	g	c	c	t	t	t	c	a	a	g	a	a	t
MH709346	3	g	c	c	t	a	t	t	g	t	c	t	t	t	t	g	g	a	c	a	a	g	a	c	c	a	t	g	g	c	c	t	t	t	c	a	a	g	a	a	t
MH709375	3	g	c	c	t	a	t	t	g	t	c	t	t	t	t	g	g	a	c	a	a	g	a	c	c	a	t	g	g	c	c	t	t	t	c	a	a	g	a	a	t
DQ452050	4	g	c	c	t	a	t	t	g	t	c	t	g	t	t	g	g	a	c	a	a	g	a	c	c	a	t	g	g	c	c	t	t	t	c	a	g	g	a	a	t
DQ479960	4	g	c	c	t	a	t	t	g	t	c	t	g	t	t	g	g	a	c	a	a	g	a	c	c	a	t	g	g	c	c	t	t	t	c	a	g	g	a	a	t
KP771898	4	g	c	c	t	a	t	t	g	t	c	t	g	t	t	t	g	a	c	a	a	g	a	c	c	a	t	g	g	c	c	t	t	t	c	a	g	g	a	a	t
MH709316	4	g	c	c	t	a	t	t	g	t	c	t	g	t	t	g	g	a	c	a	a	g	a	c	c	a	t	g	g	c	c	t	t	t	c	a	g	g	a	a	t
MH709318	4	g	c	c	t	a	t	t	g	t	c	t	g	t	t	g	g	a	c	a	a	g	a	c	c	a	t	g	g	c	c	t	t	t	c	a	g	g	a	a	t
MH709322	4	g	c	c	t	a	t	t	g	t	c	t	g	t	t	g	g	a	c	a	a	g	a	c	c	a	t	g	g	c	c	t	t	t	c	a	g	g	a	a	t
MH709323	4	g	c	c	t	a	t	t	g	t	c	t	g	t	t	g	g	a	c	a	a	g	a	c	c	a	t	g	g	c	c	t	t	t	c	a	g	g	a	a	t
MH709326	4	g	c	c	t	a	t	t	g	t	c	t	g	t	t	g	c	a	c	a	a	g	a	c	c	a	t	g	g	c	c	t	t	t	c	a	g	g	a	a	t
MH709341	4	g	c	c	t	a	t	t	g	t	c	t	g	t	t	g	g	g	c	a	a	g	a	c	c	a	t	g	g	c	c	t	t	t	c	a	g	g	a	a	t
MH709357	4	g	c	c	t	a	t	t	g	t	c	t	g	t	t	g	g	a	c	a	a	g	a	c	c	a	t	g	g	c	c	t	t	t	c	a	g	g	a	a	t
MH709364	4	g	c	c	t	a	t	t	g	t	c	t	g	t	t	g	g	a	c	a	a	g	a	c	c	a	t	g	g	c	c	t	t	t	c	a	g	g	a	a	t
MH709370	4	g	c	c	t	a	t	t	g	t	c	t	g	t	t	g	g	a	c	a	a	g	a	c	c	a	t	g	g	c	c	t	t	t	c	a	g	g	a	a	t
MH709371	4	g	c	c	t	a	t	t	g	t	c	t	g	t	t	g	g	a	c	a	a	g	a	c	c	a	t	g	g	c	c	t	t	t	c	a	g	g	a	a	t
MH709372	4	g	c	c	c	a	t	t	g	t	c	t	g	t	t	g	g	a	c	a	a	g	a	c	c	a	t	g	g	c	c	t	t	t	c	a	g	g	a	a	t
DQ457052	5	g	c	c	t	a	t	t	g	t	c	t	t	t	t	g	g	a	c	a	a	a	a	c	c	a	t	g	g	c	c	t	t	t	c	a	a	g	a	a	t
JN704704	5	g	c	c	t	a	t	t	g	t	c	t	t	t	t	g	g	a	c	a	a	g	a	c	c	a	t	g	g	c	c	t	t	t	c	a	a	g	a	a	t
JN704705	5	g	c	c	t	a	t	t	g	t	c	t	t	t	t	g	g	a	c	a	a	g	a	c	c	a	t	g	g	c	c	t	t	t	c	g	a	g	a	a	t
JN704706	5	g	c	c	t	a	t	t	g	t	c	t	t	t	t	g	g	a	c	a	a	g	a	c	c	a	t	g	g	c	c	t	t	t	c	a	a	g	a	a	t
JN704707	5	g	c	c	t	a	t	t	g	t	c	t	t	t	t	g	g	a	c	a	a	g	a	c	c	a	t	g	g	c	c	t	t	t	c	a	a	g	a	a	t
JN704708	5	g	c	c	t	a	t	t	g	t	c	t	t	t	t	g	g	a	c	a	a	g	a	c	c	a	t	g	g	c	c	t	t	t	c	g	a	g	a	a	t
KP771904	5	g	c	c	t	a	t	t	g	t	c	t	t	t	t	g	g	a	a	a	a	g	a	c	c	a	t	g	g	c	c	t	t	t	c	a	a	g	a	a	t
KP771905	5	g	c	c	t	a	t	t	g	t	c	t	t	t	t	g	g	a	c	a	a	g	a	c	c	a	t	g	g	c	c	t	t	t	c	a	a	g	a	a	t
KP771906	5	g	c	c	t	a	t	t	g	t	c	t	t	t	t	g	g	a	c	a	a	g	a	c	c	a	t	g	g	c	c	t	t	t	c	a	a	g	a	a	t
KP771919	5	g	c	c	t	a	t	t	g	t	c	t	t	t	t	g	g	a	c	a	a	g	a	c	c	a	t	g	g	c	c	t	t	t	c	a	a	g	a	a	t
KP771922	5	g	c	c	t	a	t	t	g	t	c	t	t	c	t	g	g	a	c	a	a	g	a	c	c	a	t	g	g	c	c	t	t	t	g	a	a	g	a	a	t
KP771924	5	g	c	c	t	a	t	t	g	t	c	t	t	t	t	g	g	a	c	a	a	g	a	c	c	a	t	g	g	c	c	t	t	t	c	g	a	g	a	a	t
KM355696	5	g	c	c	t	a	t	t	g	t	c	t	t	t	t	g	g	a	c	a	a	g	a	c	c	a	t	g	g	c	c	t	t	t	c	g	a	g	a	a	t
KM355697	5	g	c	c	t	a	t	t	g	t	c	t	t	t	t	g	g	a	c	a	a	g	a	c	c	a	t	g	g	c	c	t	t	t	c	g	a	g	a	a	t
KM355698	5	g	c	c	t	a	t	g	g	t	c	t	t	t	t	g	g	a	c	a	a	g	a	c	c	a	t	g	g	c	c	t	t	t	c	g	a	g	a	a	t
KM355699	5	g	c	c	t	a	t	g	g	t	c	t	t	t	t	g	g	a	c	a	a	g	a	c	c	a	t	g	g	c	c	t	t	t	c	g	a	g	a	a	t
KM355701	5	g	c	c	t	a	t	t	g	t	c	t	t	t	t	g	g	a	c	a	a	g	a	c	c	a	t	g	g	c	c	t	t	t	c	g	a	g	a	a	t
KM355702	5	g	c	c	t	a	t	g	g	t	c	t	t	t	t	g	g	a	c	a	a	g	a	c	c	a	t	g	g	c	c	t	t	t	c	g	a	g	a	a	t
KM355703	5	g	c	c	t	a	t	t	g	t	c	t	t	t	t	g	g	a	c	a	a	g	a	c	c	a	t	g	g	c	c	t	t	t	c	g	a	g	a	a	t
KM355704	5	g	c	c	t	a	t	t	g	t	c	t	t	t	t	g	g	a	c	a	a	g	a	c	c	a	t	g	g	c	c	t	t	t	c	g	a	g	a	a	t
KM355705	5	g	c	c	t	a	t	g	g	t	c	t	t	t	t	g	g	a	c	a	a	g	a	c	c	a	t	g	g	c	c	t	t	t	c	g	a	g	a	a	t
KM355706	5	g	c	c	t	a	t	g	g	t	c	t	t	t	t	g	g	a	c	a	a	g	a	c	c	a	t	g	g	c	c	t	t	t	c	g	a	g	a	a	t
KM355707	5	g	c	c	t	a	t	t	g	t	c	t	t	t	t	g	g	a	c	a	a	g	a	c	c	a	t	g	g	c	c	t	t	t	c	g	a	g	a	a	t
KM355708	5	g	c	c	t	a	t	t	g	t	c	t	t	t	t	g	g	a	c	a	a	g	a	c	c	a	t	g	g	c	c	t	t	t	c	g	a	g	a	a	t
KM355709	5	g	c	c	t	a	t	g	g	t	c	t	t	t	t	g	g	a	c	a	a	g	a	c	c	a	t	g	g	c	c	t	t	t	c	g	a	g	a	a	t
KM355711	5	g	c	c	t	a	t	g	g	t	c	t	t	t	t	g	g	a	c	a	a	g	a	c	c	a	t	g	g	c	c	t	t	t	c	g	a	g	a	a	t
KM355712	5	g	c	c	t	a	t	g	g	t	c	t	t	t	t	g	g	a	c	a	a	g	a	c	c	a	c	g	g	c	c	t	t	t	c	g	a	g	a	a	t
KY0377[illegible]	[illegible]	[illegible]	[illegible]	[illegible]	[illegible]	[illegible]	[illegible]	[illegible]	[illegible]	[illegible]	[illegible]	[illegible]	[illegible]	[illegible]	[illegible]	[illegible]	[illegible]	[illegible]	[illegible]	[illegible]	[illegible]	[illegible]	[illegible]	[illegible]	[illegible]	[illegible]	[illegible]	[illegible]	[illegible]	[illegible]	[illegible]	[illegible]	[illegible]	[illegible]	[illegible]	[illegible]	[illegible]	[illegible]	[illegible]	[illegible]	[illegible]

GB-ID																			6															
	37902	37959	38019	38023	38036	38045	38055	38081	38091	38111	38177	38213	38223	38229	38256	38306	38309	38354	38372	38418	38600	38663	38705	38714	38717	38726	38754	38825	38868	38889	38916	39016	39023	39179
AY548170	a	g	g	c	t	c	t	a	g	a	g	g	g	a	c	g	t	c	t	g	a	t	t	g	c	t	a	c	c	t	t	c	a	g
AY548171	a	g	g	c	t	c	t	a	g	a	g	g	g	a	c	g	t	c	t	g	a	t	t	g	c	t	a	c	c	t	t	c	a	g
DQ479953	a	g	g	c	t	c	t	a	g	a	g	g	g	a	c	g	t	c	t	g	a	t	t	g	c	t	a	c	c	t	t	c	a	g
DQ479954	a	g	g	c	t	c	t	a	g	a	g	g	g	a	c	g	t	c	t	g	a	t	t	g	c	t	a	c	c	t	t	c	a	g
DQ479958	a	g	g	c	t	c	t	a	g	a	g	g	g	a	c	g	t	c	t	g	a	t	t	g	c	t	a	c	c	t	t	c	a	g
DQ479959	a	g	g	c	t	c	t	a	g	a	g	g	g	a	c	g	t	c	t	g	a	t	t	g	c	t	a	c	c	t	t	c	a	g
DQ479961	a	g	g	c	t	c	t	a	g	a	g	g	g	a	c	g	t	c	t	g	a	t	t	g	c	t	a	c	c	t	t	c	a	g
DQ479962	a	g	g	c	t	c	t	a	g	a	g	g	g	a	c	g	t	c	t	g	a	t	t	g	c	t	a	c	c	t	t	c	a	g
DQ479963	a	g	g	c	t	c	t	a	g	a	g	g	g	a	c	g	t	c	t	g	a	t	t	g	c	t	a	c	c	t	t	c	a	g
DQ674250	a	g	g	c	t	c	t	a	g	a	g	g	g	a	c	g	t	c	t	g	a	t	t	g	c	t	a	c	c	t	t	c	a	g
KX262864	a	g	g	c	t	c	t	a	g	a	g	g	g	a	c	g	t	c	t	g	a	t	t	g	c	t	a	c	c	t	t	c	a	g
KX262865	a	g	g	c	t	c	t	a	g	a	g	g	g	a	c	g	t	c	t	g	a	t	t	g	c	t	a	c	c	t	t	c	a	g
KX262866	a	g	g	c	t	c	t	a	g	a	g	g	g	a	c	g	t	c	t	g	a	t	t	g	c	t	a	c	c	t	t	c	a	g
EU154348	a	g	g	c	t	c	t	a	g	a	g	g	g	a	c	g	t	c	t	g	a	t	t	g	c	t	a	c	c	t	t	c	a	g
JN704690	a	g	g	c	t	c	t	a	g	a	g	g	g	a	c	g	t	c	t	g	a	t	t	g	c	t	a	c	c	t	t	c	a	g
JN704691	a	g	g	c	t	c	t	a	g	a	g	g	g	a	c	g	t	c	t	g	a	t	t	g	c	t	a	c	c	t	t	c	a	g
JN704692	a	g	g	c	t	c	t	a	g	a	g	g	g	a	c	g	t	c	t	g	a	t	t	g	c	t	a	c	c	t	t	c	a	g
JN704693	a	g	g	c	t	c	t	a	g	a	g	g	g	a	c	g	t	c	t	g	a	t	t	g	c	t	a	c	c	t	t	c	a	g
JN704694	a	g	g	c	t	c	t	a	g	a	g	g	g	a	c	g	t	t	t	g	a	t	t	g	c	t	a	c	c	t	t	c	a	g
JN704695	a	g	g	c	t	c	t	a	g	a	g	g	g	a	c	g	t	c	t	g	a	t	t	g	c	t	a	c	c	t	t	c	a	g
JN704696	a	g	g	c	t	c	t	a	g	a	g	g	g	a	c	g	t	c	t	g	a	t	t	g	c	t	a	c	c	t	t	c	a	g
NC001348	a	g	g	c	t	c	t	a	g	a	g	g	g	a	c	g	t	c	t	g	a	t	t	g	c	t	a	c	c	t	t	c	a	g
KP771892	a	g	g	c	t	c	t	a	g	a	g	g	g	a	c	g	t	c	t	g	a	t	t	g	c	t	a	c	c	t	t	c	a	g
KP771894	a	g	g	c	t	c	t	a	g	a	g	g	g	a	c	g	t	c	t	g	a	t	t	g	c	t	a	c	c	t	t	c	a	g
KP771900	a	g	g	c	t	c	t	a	g	a	g	g	g	a	c	g	t	c	t	g	a	t	t	g	c	t	a	c	c	t	t	c	a	g
KP771901	a	g	g	c	t	c	t	a	g	a	g	g	g	a	c	g	t	c	t	g	a	t	t	g	c	t	a	c	c	t	t	c	a	g
KP771902	a	g	g	c	t	c	t	a	g	a	g	g	g	a	c	g	t	c	t	g	a	t	t	g	c	t	a	c	c	t	t	c	a	g
KP771908	a	g	g	c	t	c	t	a	g	a	g	g	g	a	c	g	t	c	t	g	a	t	t	g	c	t	a	c	c	t	t	c	a	g
KP771912	a	g	g	c	t	c	t	a	g	a	g	g	g	a	c	g	t	c	t	g	a	t	t	g	c	t	a	c	c	t	t	c	a	g
KP771913	a	g	g	c	t	c	t	a	g	a	g	g	g	a	c	g	t	c	t	g	a	t	t	g	c	t	a	c	c	t	t	c	a	g
KP771917	a	g	g	c	t	c	t	a	g	a	g	g	g	a	c	g	t	c	t	g	a	t	t	g	c	t	a	c	c	t	t	c	a	g
KP771918	a	g	g	c	t	c	t	a	g	a	g	g	g	a	c	g	t	c	t	g	a	t	t	g	c	t	a	c	c	t	t	c	a	g
KP771920	a	g	g	c	t	c	t	a	g	a	g	g	g	a	c	g	t	c	t	g	a	t	t	g	c	t	a	c	c	t	t	c	a	g
MH709361	a	g	g	c	t	c	t	a	g	a	g	g	g	a	c	g	t	c	t	g	a	t	t	g	c	t	a	c	c	t	t	c	a	g
KU926318	g	g	g	c	t	c	c	c	g	a	a	g	g	a	c	g	t	c	t	g	g	t	t	t	t	t	a	c	c	t	t	c	g	g
KU926319	g	g	g	c	t	c	c	c	g	a	a	g	g	a	c	g	t	c	t	g	a	t	t	t	t	t	a	c	c	t	t	c	g	g
KU926320	g	g	g	c	t	c	c	c	g	a	a	g	g	a	c	g	t	c	t	g	a	t	t	t	t	c	a	c	c	t	t	c	g	g
KU926321	g	g	g	c	t	c	c	c	g	a	a	g	g	a	c	g	t	c	t	g	a	t	t	t	t	t	a	c	c	t	c	c	g	g
KU926322	g	g	g	c	t	c	c	c	g	a	a	g	g	a	c	g	t	c	t	g	a	t	t	t	t	t	a	c	c	t	c	c	g	g
KC847290	g	g	g	c	t	c	c	c	g	a	a	g	g	a	c	g	t	c	t	g	a	t	t	t	t	t	a	t	c	t	t	c	g	g
KJ767491	g	g	g	c	t	c	c	c	g	a	a	g	g	a	c	g	t	c	t	g	a	t	t	t	t	t	a	c	c	t	t	c	g	g
KJ767492	g	g	g	c	t	c	c	c	g	a	a	g	g	a	c	g	t	c	t	g	a	t	t	t	t	t	a	c	c	t	t	c	g	g
KJ808816	g	g	g	c	t	c	c	c	g	a	a	g	g	a	c	g	t	c	t	g	a	t	t	t	t	t	a	c	c	t	c	c	g	g
KP771925	g	g	g	c	t	c	c	c	g	a	a	g	g	a	c	g	t	c	t	g	a	t	t	t	t	t	a	c	c	t	t	c	g	g
KU926315	g	g	g	c	t	c	c	c	g	a	a	g	g	a	c	g	t	c	t	g	a	t	t	t	t	t	a	c	c	t	t	c	g	g
KU926316	g	g	g	c	t	c	c	c	g	a	a	g	g	a	c	g	t	c	t	g	a	t	t	t	t	t	a	c	c	t	t	c	g	g
KU926317	g	g	g	c	t	c	c	c	g	a	a	g	g	a	c	g	t	c	t	g	a	t	t	t	t	t	a	c	c	t	t	c	g	g
KY037796	g	g	g	c	t	c	c	c	g	a	a	g	g	a	c	g	t	c	t	g	a	t	t	t	t	t	a	c	c	t	t	c	g	g
MF004348	g	g	g	c	t	c	c	c	g	a	a	g	g	a	c	g	t	c	t	g	a	t	t	t	t	t	a	c	c	t	t	c	g	g
MH709309	g	g	g	c	t	c	c	c	g	a	a	g	g	a	c	g	t	c	t	g	a	t	t	t	t	t	a	c	c	t	t	c	g	g
MH709310	g	g	g	c	t	c	c	c	g	a	a	g	g	a	c	g	t	c	t	g	a	t	t	t	t	t	a	c	c	t	t	c	g	g
MH709311	g	g	g	c	t	c	c	c	g	a	a	g	g	a	c	g	t	c	t	g	a	t	t	t	t	t	a	c	c	t	t	c	g	g
MH709313	g	g	g	c	t	c	c	c	g	a	a	g	g	a	c	g	t	c	t	g	a	t	t	t	t	t	a	c	c	t	t	c	g	g
MH709314	g	g	g	c	t	c	c	c	g	a	a	g	g	a	c	g	t	c	t	g	a	t	t	t	t	t	a	c	c	t	t	c	g	g
MH709315	g	g	g	c	t	c	c	c	g	a	a	g	g	a	c	g	t	c	t	g	a	t	t	t	t	t	a	c	c	t	t	c	g	g
MH709319	g	g	g	c	t	c	c	c	g	a	a	g	g	a	c	g	t	c	t	g	a	t	t	t	t	t	a	c	c	t	t	c	g	g
MH709320	g	g	g	c	t	c	c	c	g	a	a	g	g	a	c	g	t	c	t	g	a	t	t	t	t	t	a	c	c	t	t	c	g	g
MH709329	g	g	g	c	t	c	c	c	g	a	a	g	g	a	c	g	t	c	t	g	a	t	t	t	t	t	a	c	c	t	t	c	g	g
MH709331	g	g	g	c	t	c	c	c	g	a	a	g	g	a	c	g	t	c	t	g	a	t	t	t	t	t	a	c	c	t	t	c	g	g
MH709333	g	g	g	c	t	c	c	c	g	a	a	g	g	a	c	g	t	c	t	g	a	t	t	t	t	t	a	c	c	t	t	c	g	g
MH709337	g	g	g	c	t	c	c	c	g	a	a	g	g	a	c	g	t	c	t	g	a	t	t	t	t	t	a	c	c	t	t	c	g	g
MH709339	g	g	g	c	t	c	c	c	g	a	a	g	g	a	c	g	t	c	t	g	a	t	t	t	t	t	a	c	c	t	t	c	g	a
MH709344	g	g	g	c	t	c	c	c	g	a	a	g	g	a	c	g	t	c	t	g	a	t	t	t	t	t	a	c	c	t	t	c	g	g
MH709348	g	g	g	c	t	c	c	c	g	a	a	g	g	a	c	g	t	c	t	g	a	t	t	t	t	t	a	c	c	t	t	c	g	g
MH709355	g	g	g	c	t	c	c	c	g	a	a	g	g	a	c	g	t	c	t	g	a	t	t	t	t	t	a	c	c	t	t	c	g	g
MH709359	g	g	g	c	t	c	c	c	g	a	a	g	g	a	c	g	t	c	t	g	a	t	t	t	t	t	a	c	c	t	t	c	g	g
MH709363	g	g	g	c	t	c	c	c	g	a	a	g	g	a	c	g	t	c	t	g	a	t	t	t	t	t	a	c	c	t	t	c	g	g
MH709366	g	g	g	c	t	c	c	c	g	a	a	g	g	a	c	g	t	c	t	g	a	t	t	t	t	t	a	c	c	t	t	c	g	g
MH709376	g	g	g	c	t	c	c	c	g	a	a	g	g	a	c	g	t	c	t	g	a	t	t	t	t	t	a	c	c	t	t	c	g	g
MH709377	g	g	g	c	t	c	c	c	g	a	a	g	g	a	c	g	t	c	t	g	a	t	t	t	t	t	a	c	c	t	c	c	g	g
**MW316406**	g	g	g	c	t	c	c	c	g	a	a	g	g	a	c	g	t	c	t	g	a	t	t	t	t	t	a	c	c	t	t	c	g	g
AJ871403	a	g	g	c	t	c	t	a	g	a	g	g	g	a	c	g	t	c	t	g	a	t	t	t	c	t	a	c	c	t	t	c	g	g
DQ479955	a	g	g	c	t	c	t	a	g	a	g	g	g	a	c	g	t	c	t	g	a	t	t	t	c	t	a	c	c	t	t	c	g	g
DQ479956	a	g	g	c	t	c	t	a	g	a	g	g	g	a	c	g	t	c	t	g	a	t	t	t	c	t	a	c	c	t	t	c	g	g
DQ479957	a	g	g	c	t	c	t	a	g	a	g	g	g	a	c	g	t	c	t	g	a	t	t	t	c	t	a	c	c	t	t	c	g	g
JN704699	a	g	g	c	t	c	t	a	g	a	g	g	g	a	c	g	t	c	t	g	a	t	t	t	c	t	a	c	c	t	t	c	g	g
JN704700	a	g	g	c	t	c	t	a	g	a	g	g	g	a	c	g	t	c	t	g	a	t	t	t	c	t	a	c	c	t	t	c	g	g
JN704701	a	g	g	c	t	t	t	a	g	a	g	g	g	a	c	g	t	c	t	g	a	t	t	t	c	t	a	c	c	t	t	c	g	g
JN704702	a	g	g	c	t	c	t	a	g	a	g	g	g	a	c	g	t	c	t	g	a	t	t	t	c	t	a	c	c	t	t	c	g	g
JN704703	a	g	g	c	t	c	t	a	g	a	g	g	g	a	c	g	t	c	t	g	a	t	t	g	c	t	a	c	c	t	t	c	g	g
KP771891	a	g	g	c	t	c	t	a	g	a	g	g	a	a	c	g	t	c	t	g	a	t	t	t	c	t	a	c	t	t	t	c	g	g
KP771893	a	g	g	c	t	c	t	a	g	a	g	g	g	a	c	g	t	c	t	g	a	t	t	t	c	t	a	c	c	t	t	c	g	g
KP771895	a	g	g	c	t	c	t	a	g	a	g	g	g	a	c	g	t	c	t	g	a	t	t	t	c	t	a	c	c	t	t	c	g	g
KP771896	a	g	g	c	t	c	t	a	g	a	g	g	g	a	c	g	t	c	t	g	a	t	t	t	c	t	a	c	c	t	t	c	g	g
KP771897	a	g	g	c	t	c	t	a	g	a	g	g	g	a	c	g	t	c	t	g	a	t	t	t	c	t	a	c	c	t	t	c	g	g
KP771899	a	g	g	c	t	c	t	a	g	a	g	g	g	a	c	g	t	c	t	g	a	t	t	t	c	t	a	c	t	t	t	c	g	g
KP771903	a	g	g	c	t	c	t	a	g	a	g	g	g	a	c	g	t	c	t	g	a	t	t	t	c	t	a	c	c	t	t	c	g	g
KP771907	a	g	g	c	t	c	t	a	g	a	g	g	g	a	c	g	t	c	t	g	a	t	t	t	c	t	a	c	c	t	t	c	g	g
KP771910	a	g	g	c	t	c	t	a	g	a	g	g	g	a	c	g	t	c	t	g	a	t	t	t	c	t	a	c	c	t	t	c	g	g
KP771915	a	g	g	c	t	c	t	a	g	a	g	g	g	a	c	g	t	c	t	g	a	t	t	t	c	t	a	c	c	t	t	c	g	g
KP771916	a	g	g	c	t	c	t	a	g	a	g	g	g	a	c	g	t	c	t	g	a	t	t	t	c	t	a	c	c	t	t	c	g	g
KP771921	a	g	g	c	t	c	t	a	g	a	g	g	g	a	c	g	t	c	t	g	a	t	t	t	c	t	a	c	c	t	t	c	g	g
KP771923	a	g	g	c	t	c	t	a	g	a	g	g	g	a	c	g	t	c	t	g	a	t	t	t	c	t	a	c	c	t	t	c	g	g
KP771889	a	g	g	c	t	c	t	a	g	a	g	g	g	a	c	g	t	c	t	g	a	t	t	t	c	t	a	c	c	t	t	c	a	g
KU926311	a	g	g	c	t	c	t	a	g	a	g	g	g	a	c	g	t	c	t	g	a	t	t	t	c	t	a	c	c	t	t	c	g	g
JQ911599	a	g	g	c	t	c	t	a	g	a	g	g	g	a	c	g	t	c	t	g	a	t	t	t	c	t	a	c	c	t	t	c	g	g
MH709317	a	g	g	c	t	c	t	c	g	a	g	g	g	a	c	g	t	c	t	g	a	t	t	t	c	t	a	c	c	t	t	c	g	g
MH709346	a	g	g	c	t	c	t	c	g	a	g	g	g	a	c	g	t	c	t	g	a	t	t	t	c	t	a	c	c	t	t	c	g	g
MH709375	a	g	g	c	t	c	t	c	g	a	g	g	g	a	c	g	t	c	t	g	a	t	t	t	c	t	a	c	c	t	t	c	g	g
DQ452050	a	g	g	c	t	c	c	c	g	a	a	g	g	g	c	g	t	c	t	g	a	t	t	g	c	t	a	c	c	t	t	c	g	g
DQ479960	a	g	g	c	t	c	c	c	g	a	a	g	g	a	c	g	t	c	t	g	a	t	t	g	c	t	a	c	c	t	t	c	g	g
KP771898	a	g	g	c	t	c	c	c	g	a	a	g	g	a	c	g	t	c	t	a	a	t	t	g	c	t	a	c	c	t	t	c	g	g
MH709316	a	g	g	c	t	c	c	c	g	a	a	g	g	a	c	g	t	c	t	g	a	t	t	g	c	t	a	c	c	t	t	c	g	g
MH709318	a	g	g	c	t	c	c	c	g	a	a	g	g	a	c	g	t	c	t	g	a	t	t	g	c	t	a	c	c	t	t	c	g	g
MH709322	a	g	g	c	t	c	c	c	g	a	a	g	g	a	c	a	t	c	t	g	a	t	t	g	c	t	a	c	c	t	t	c	g	g
MH709323	a	g	g	c	t	c	c	c	g	a	a	g	g	a	c	g	t	c	t	g	a	t	t	g	c	t	a	c	c	t	t	c	g	g
MH709326	a	g	g	c	t	c	c	c	g	a	a	g	g	a	c	g	t	c	t	g	a	t	t	g	c	t	a	c	c	t	t	c	g	g
MH709341	a	g	g	c	t	c	c	c	g	a	a	a	g	a	c	g	t	c	t	g	a	t	t	g	c	t	a	c	c	c	t	c	g	g
MH709357	a	g	g	c	t	c	c	c	g	a	a	g	g	a	c	g	t	c	t	g	a	t	t	g	c	t	a	c	c	t	t	c	g	g
MH709364	a	g	g	c	t	c	c	c	g	a	a	g	g	a	c	g	t	c	t	g	a	t	t	g	c	t	a	c	c	t	t	c	g	g
MH709370	a	g	g	c	t	c	c	c	t	a	a	g	g	a	c	g	t	c	t	g	a	t	t	g	c	t	a	c	c	t	t	c	g	g
MH709371	a	g	g	c	t	c	c	c	g	a	a	g	g	a	c	g	t	c	t	g	a	t	t	g	c	t	a	c	c	t	t	c	g	g
MH709372	a	g	g	c	t	c	c	c	g	a	a	g	g	a	c	g	t	c	t	g	a	t	t	g	c	t	a	c	c	t	t	c	g	g
DQ457052	a	g	g	c	t	c	t	c	g	a	g	g	g	a	c	g	t	c	t	g	a	t	t	t	c	t	a	c	c	t	t	c	g	g
JN704704	a	g	g	c	t	c	t	c	g	a	g	g	g	a	c	g	t	c	t	g	a	t	t	t	c	t	a	c	c	t	t	c	g	g
JN704705	a	g	g	c	t	c	t	c	g	a	g	g	g	a	a	g	t	c	t	g	a	t	t	t	c	t	a	c	c	t	t	c	g	g
JN704706	a	g	g	c	t	c	t	c	g	a	g	g	g	a	c	g	t	c	t	g	a	t	t	t	c	t	a	c	c	t	t	c	g	g
JN704707	a	g	g	c	t	c	t	c	g	a	g	g	g	a	a	g	t	c	t	g	a	t	t	t	c	t	a	c	c	t	t	c	g	g
JN704708	a	g	g	c	t	c	t	c	g	a	g	g	g	a	c	g	t	c	t	g	a	t	t	t	c	t	a	c	c	t	t	c	g	g
KP771904	a	g	g	c	t	c	t	c	g	a	g	g	g	a	c	g	t	c	t	g	a	t	t	t	c	t	a	c	c	t	t	c	g	g
KP771905	a	g	g	c	t	c	t	c	g	a	g	g	g	a	c	g	t	c	t	g	a	t	t	t	c	t	a	c	c	t	t	c	g	g
KP771906	a	g	g	c	t	c	t	c	g	a	g	g	g	a	c	g	t	c	t	g	a	t	t	t	c	t	a	c	c	t	t	c	g	g
KP771919	a	g	g	c	t	c	t	c	g	a	g	g	g	a	c	g	t	c	t	g	a	t	t	t	c	t	a	c	c	t	t	c	g	g
KP771922	a	g	g	c	t	c	t	c	g	a	g	g	g	a	c	g	t	c	t	g	a	t	t	t	c	t	a	c	c	t	t	c	g	g
KP771924	a	g	g	c	t	c	t	c	g	a	g	g	g	a	a	g	t	c	t	g	a	t	t	t	c	t	a	c	c	t	t	c	g	g
KM355696	a	g	g	c	t	c	t	c	g	a	g	g	g	a	c	g	t	c	t	g	a	t	t	t	c	t	a	c	t	t	t	c	g	g
KM355697	a	g	g	c	t	c	t	c	g	a	g	g	g	a	c	g	t	c	t	g	a	t	t	t	c	t	a	c	t	t	t	c	g	g
KM355698	a	g	g	c	t	c	t	c	g	a	g	g	g	a	c	g	t	c	t	g	a	t	t	t	c	t	a	c	c	t	t	c	g	g
KM355699	a	g	g	c	t	c	t	c	g	a	g	g	g	a	c	g	t	c	t	g	a	t	t	t	c	t	a	c	c	t	t	c	g	g
KM355701	a	g	g	c	t	c	t	c	g	a	g	g	g	a	c	g	t	c	t	g	a	t	t	t	c	t	a	c	t	t	t	c	g	g
KM355702	a	g	g	c	t	c	t	c	g	a	g	g	g	a	c	g	t	c	t	g	a	t	t	t	c	t	a	c	c	t	t	c	g	g
KM355703	a	g	g	c	t	c	t	c	g	a	g	g	g	a	c	g	t	c	t	g	a	t	t	t	c	t	a	c	t	t	t	c	g	g
KM355704	a	g	g	c	t	c	t	c	g	a	g	g	g	a	c	g	t	c	t	g	a	t	t	t	c	t	a	c	t	t	t	c	g	g
KM355705	a	g	g	c	t	c	t	c	g	a	g	g	g	a	c	g	t	c	t	g	a	t	t	t	c	t	a	c	c	t	t	c	g	g
KM355706	a	g	g	c	t	c	t	c	g	a	g	g	g	a	c	g	t	c	t	g	a	t	t	t	c	t	a	c	c	t	t	c	g	g
KM355707	a	g	g	c	t	c	t	c	g	a	g	g	g	a	c	g	t	c	t	g	a	t	t	t	c	t	a	c	t	t	t	c	g	g
KM355708	a	g	g	c	t	c	t	c	g	a	g	g	g	a	c	g	t	c	t	g	a	t	t	t	c	t	a	c	t	t	t	c	g	g
KM355709	a	g	g	c	t	c	t	c	g	a	g	g	g	a	c	g	t	c	t	g	a	t	t	t	c	t	a	c	c	t	t	c	g	g
KM355711	a	g	g	c	t	c	t	c	g	a	g	g	g	a	c	g	t	c	t	g	a	t	t	t	c	t	a	c	c	t	t	c	g	g
KM355712	a	g	g	c	t	c	t	c	g	a	g	g	g	a	c	g	t	c	t	g	a	t	t	t	c	t	a	c	c	t	t	c	g	g
KY0377[illegible]	[illegible]	[illegible]	[illegible]	[illegible]	[illegible]	[illegible]	[illegible]	[illegible]	[illegible]	[illegible]	[illegible]	[illegible]	[illegible]	[illegible]	[illegible]	[illegible]	[illegible]	[illegible]	[illegible]	[illegible]	[illegible]	[illegible]	[illegible]	[illegible]	[illegible]	[illegible]	[illegible]	[illegible]	[illegible]	[illegible]	[illegible]	[illegible]	[illegible]	[illegible]

Supplementary Table 3: 2880 SN

GB-ID	Clade markers											4																																																6															
	Position in Dumas	35291	35332	35543	35579	35589	35642	35658	35735	35819	35836	35963	35971	36005	36047	36063	36109	36131	36271	36623	36659	36801	36926	37063	37176	37211	37241	37260	37282	37285	37337	37349	37426	37447	37479	37522	37649	37775	37783	37811	37814	37902	37959	38019	38023	38036	38045	38055	38081	38091	38111	38177	38213	38223	38229	38256	38306	38309	38354	38372	38418	38600	38663	38705	38714	38717	38726	38754	38825	38868	38889	38916	39016	39023	39179
MH499467	5	g	c	c	t	a	t	t	g	t	c	t	t	t	t	g	g	a	c	a	a	g	a	c	c	a	t	g	g	c	c	t	t	t	c	a	a	g	a	a	t	a	g	g	c	t	c	t	c	g	a	g	g	g	a	c	g	t	c	t	g	a	t	t	t	c	t	a	c	c	t	t	c	g	g
MH499469	5	g	c	c	t	a	t	t	g	t	c	t	t	t	t	g	g	a	c	a	a	g	a	c	c	a	t	g	g	c	c	t	t	t	c	a	a	g	a	a	t	a	g	g	c	t	c	t	c	g	a	g	g	g	a	c	g	t	c	t	g	a	t	t	t	c	t	a	c	c	t	t	c	g	g
MH709321	5	g	c	c	t	a	t	t	g	t	c	t	t	t	t	g	g	a	c	a	a	g	a	c	c	a	t	g	g	c	c	t	t	t	c	a	a	g	a	a	t	a	g	g	t	t	c	t	c	g	a	g	g	g	a	c	g	t	c	t	g	a	t	t	t	c	t	a	c	c	t	t	c	g	g
MH709324	5	g	c	c	t	a	t	t	g	t	c	t	t	t	t	g	g	a	c	a	a	g	a	c	c	a	t	g	g	c	c	t	t	t	c	a	a	g	a	a	t	a	g	g	c	t	c	c	c	g	a	g	g	g	a	c	g	t	c	t	g	a	t	t	t	c	t	a	c	c	t	t	c	g	g
MH709325	5	g	c	c	t	a	t	g	g	t	c	t	t	t	t	g	g	a	c	a	a	g	a	c	c	a	t	g	a	c	c	t	t	t	c	g	a	g	a	a	t	a	g	g	c	t	c	t	c	g	a	g	g	g	a	c	g	t	c	t	g	a	t	t	t	c	t	a	c	c	t	t	c	g	g
MH709327	5	g	c	c	t	a	t	t	g	t	c	t	t	t	t	g	g	a	c	a	a	a	a	c	c	a	t	g	g	c	c	t	t	t	c	a	a	g	a	a	t	a	g	g	c	t	c	t	c	g	a	g	g	g	a	c	g	t	c	t	g	a	t	t	t	c	t	a	c	c	t	t	c	g	g
MH709328	5	g	c	c	t	a	t	t	g	t	c	t	t	t	t	g	g	a	c	a	a	g	a	c	c	a	t	g	g	c	c	t	t	t	c	a	a	g	a	a	t	a	g	g	c	t	c	t	c	g	a	g	g	g	a	c	g	t	c	t	g	a	t	t	t	c	t	a	c	c	t	t	c	g	g
MH709330	5	g	c	c	t	a	t	t	g	t	c	t	t	t	t	g	g	a	c	a	a	g	a	c	c	a	t	g	g	c	c	t	t	t	c	a	a	g	a	a	t	a	g	g	c	t	c	t	c	g	a	g	g	g	a	c	g	t	c	t	g	a	t	c	t	c	t	a	c	c	t	t	c	g	g
MH709332	5	g	c	c	t	a	t	t	g	t	t	t	t	t	t	g	g	a	c	a	a	g	a	c	c	a	t	g	g	c	c	t	t	t	c	a	a	g	a	a	t	a	g	g	c	t	c	t	c	g	a	g	g	g	a	c	g	t	c	t	g	a	t	t	t	c	t	a	c	c	t	t	c	g	g
MH709334	5	g	c	c	t	a	t	t	g	t	c	t	t	t	t	g	g	a	c	a	a	a	a	c	c	a	t	g	g	c	c	t	t	t	c	a	a	g	a	a	t	a	g	g	c	t	c	t	c	g	a	g	g	g	a	c	g	t	c	t	g	a	t	t	t	c	t	a	c	c	t	t	c	g	g
MH709335	5	g	c	c	t	a	t	t	g	t	c	t	t	t	t	g	g	a	c	a	a	a	a	c	c	a	t	g	g	c	c	t	t	t	c	a	a	g	a	a	t	a	g	g	c	t	c	t	c	g	a	g	g	g	a	c	g	t	c	t	g	a	t	t	t	c	t	a	c	c	t	t	c	g	g
MH709336	5	g	c	c	t	a	t	t	a	c	c	t	t	t	t	g	g	a	c	a	a	a	a	c	c	a	t	g	g	c	c	t	t	t	c	a	a	g	a	a	t	a	g	g	c	t	c	t	c	g	a	g	g	g	a	c	g	t	c	t	g	a	t	t	t	c	t	a	c	c	t	t	c	g	g
MH709338	5	g	c	c	t	a	t	t	g	t	c	t	t	t	t	g	g	a	c	a	a	a	a	c	c	a	t	g	g	c	c	t	t	t	c	a	a	g	a	a	t	a	g	g	c	t	c	t	c	g	a	g	g	g	a	c	g	t	c	t	g	a	t	t	t	c	t	a	c	c	t	t	c	g	g
MH709340	5	g	c	c	t	a	t	t	g	t	c	t	t	t	t	g	g	a	c	a	a	g	a	c	c	a	t	g	g	c	c	t	t	t	c	a	a	a	a	a	t	a	g	g	c	t	c	t	c	g	a	g	g	g	a	c	g	t	c	t	g	a	t	t	t	c	t	a	c	c	t	t	c	g	g
MH709342	5	g	c	c	t	a	t	t	g	t	c	t	t	t	t	g	g	a	c	a	a	g	a	c	c	a	t	g	g	c	c	t	t	t	c	a	a	g	a	a	t	a	g	g	c	t	c	t	c	g	a	g	g	g	a	c	g	t	c	t	g	a	t	t	t	c	t	a	c	c	t	t	c	g	g
MH709343	5	g	c	c	t	a	t	t	g	t	c	t	t	t	t	g	g	a	c	a	a	g	a	c	c	a	t	g	g	c	c	t	t	t	c	a	a	g	a	a	t	a	g	g	c	t	c	t	c	g	a	g	g	g	a	c	g	t	c	t	g	a	t	t	t	c	t	a	c	c	t	t	c	g	g
MH709345	5	g	c	c	t	a	t	t	g	t	c	t	t	t	t	g	g	a	c	a	a	a	a	c	c	a	t	g	g	c	c	t	t	t	c	a	a	g	a	a	t	a	g	g	c	t	c	t	c	g	a	g	g	g	a	c	g	t	c	t	g	a	t	t	t	c	t	a	c	c	t	t	c	g	g
MH709347	5	g	c	c	t	a	t	t	g	t	c	t	t	t	t	g	g	a	c	a	a	a	a	c	c	a	t	g	g	c	c	t	t	t	c	a	a	g	a	a	t	a	g	g	c	t	c	t	c	g	a	g	g	g	a	c	g	t	c	t	g	a	t	t	t	c	t	a	c	c	t	t	c	g	g
MH709349	5	g	c	c	t	a	t	t	g	t	c	t	t	t	t	g	g	a	c	a	a	g	a	c	c	a	t	g	g	c	c	t	t	t	c	a	a	g	a	a	t	a	g	g	c	t	c	t	c	g	a	g	g	g	a	c	g	c	c	t	g	a	t	t	t	c	t	a	c	c	t	t	c	g	g
MH709350	5	g	c	c	t	a	t	t	g	t	c	t	t	t	t	g	g	a	c	a	a	a	a	c	c	a	t	g	g	c	c	t	t	t	c	a	a	g	a	a	t	a	g	g	c	t	c	t	c	g	a	g	g	g	a	c	g	t	c	t	g	a	t	t	t	c	t	a	c	c	t	t	c	g	g
MH709351	5	g	c	c	t	a	t	t	g	t	c	t	t	t	t	g	g	a	c	a	a	g	a	c	c	a	t	g	g	c	c	t	t	t	c	a	a	g	a	a	t	a	g	g	c	t	c	t	c	g	a	g	g	g	a	c	g	t	c	t	g	a	t	t	t	c	t	a	c	c	t	t	c	g	g
MH709353	5	g	c	c	t	a	t	t	g	t	c	t	t	t	t	g	g	a	c	a	a	a	a	c	c	a	t	g	g	c	c	t	t	t	c	a	a	g	a	a	t	a	g	g	c	t	c	t	c	g	a	g	g	g	a	c	g	t	c	t	g	a	t	t	t	c	t	a	c	c	t	t	c	g	g
MH709358	5	g	c	c	t	a	t	t	g	t	c	t	t	t	t	g	g	a	c	a	a	g	a	c	c	a	t	g	g	c	c	t	t	t	c	a	a	g	a	a	t	a	g	g	c	t	c	t	c	g	a	g	g	g	a	c	g	t	c	t	g	a	t	t	t	c	t	a	c	c	t	t	c	g	g
MH709360	5	g	c	c	t	a	t	t	g	t	c	t	t	t	t	g	g	a	c	a	a	g	a	c	c	a	t	g	g	c	c	t	t	t	c	a	a	g	a	a	t	a	g	g	c	t	c	t	c	g	a	g	g	g	a	c	g	t	c	t	g	a	t	t	t	c	t	a	c	c	t	t	c	g	g
MH709362	5	g	c	c	t	a	t	t	g	t	c	t	t	t	t	g	g	a	c	a	a	a	a	c	c	a	t	g	g	c	c	t	t	t	c	a	a	g	a	a	t	a	g	g	c	t	c	t	c	g	a	g	g	g	a	c	g	t	c	t	g	a	t	t	t	c	t	a	c	c	t	t	c	g	g
MH709365	5	g	c	c	t	a	t	t	g	t	c	t	t	t	t	g	g	a	c	a	a	a	a	c	c	a	t	g	g	c	c	t	t	t	c	a	a	g	a	a	t	a	g	g	c	t	c	t	c	g	a	g	g	g	a	c	g	t	c	t	g	a	t	t	t	c	t	a	c	c	t	t	c	g	g
MH709373	5	g	c	c	t	a	t	t	g	t	c	t	t	t	t	g	g	a	c	a	a	g	a	c	c	a	t	g	g	c	c	t	t	t	c	a	a	g	a	a	t	a	g	g	c	t	c	t	c	g	a	g	g	g	a	c	g	t	c	t	g	a	c	t	t	c	t	a	c	c	t	t	c	g	g
MH709374	5	g	c	c	t	a	t	t	g	t	c	t	t	t	t	g	g	a	c	a	a	a	a	c	c	a	t	g	g	c	c	t	t	t	c	a	a	g	a	a	t	a	g	g	c	t	c	t	c	g	a	g	g	g	a	c	g	t	c	t	g	a	t	t	t	c	t	a	c	c	t	t	c	g	g
KC112914	6	g	c	c	t	a	t	t	g	t	c	t	t	t	t	g	g	a	c	a	a	g	a	c	c	a	t	g	g	c	c	t	t	t	c	a	a	g	a	a	t	a	g	g	c	t	c	t	c	g	a	a	g	g	a	c	g	t	c	c	g	a	t	t	t	c	t	a	c	c	t	t	c	g	g
KP771914	6	g	c	c	t	a	t	t	g	t	c	t	t	t	t	g	g	a	c	a	a	g	a	c	c	a	t	g	g	c	c	t	t	t	c	a	a	g	a	g	t	a	g	g	c	t	c	t	c	g	a	a	g	g	a	c	g	t	c	c	g	a	t	t	t	c	t	a	c	c	t	t	c	g	g
KP702725	6	g	c	c	t	a	t	t	g	t	c	t	t	t	t	g	g	a	c	a	a	g	a	c	c	a	t	g	g	c	c	t	t	t	c	a	a	g	a	a	t	a	g	g	c	t	c	t	c	g	a	a	g	g	a	c	g	t	c	c	g	a	t	t	t	c	t	a	c	c	t	t	c	g	g
KP771890	9	a	c	c	t	a	t	t	g	t	c	t	t	t	t	g	g	a	c	a	a	g	a	c	c	a	t	g	g	c	c	t	t	t	c	a	g	g	a	a	t	a	g	g	c	t	c	c	c	g	a	a	g	g	a	c	g	t	c	t	g	a	t	t	g	c	t	a	c	c	t	t	t	g	g
JN704710	9	g	c	t	t	a	t	t	g	t	c	t	t	t	t	g	g	a	c	a	a	g	a	c	c	a	t	g	g	c	c	t	t	t	c	a	g	g	a	a	t	a	g	g	c	t	c	c	a	g	a	g	g	g	a	c	g	t	c	t	g	a	t	t	g	c	t	a	c	c	t	t	c	a	g
KY037798	9	g	c	c	t	a	t	t	g	t	c	t	t	t	t	g	g	a	c	a	a	g	a	c	c	a	t	g	g	c	c	t	t	t	c	a	g	g	a	a	t	a	g	g	c	t	c	c	c	g	a	a	g	g	a	c	g	t	c	t	g	a	t	t	g	c	t	a	c	c	t	t	c	g	g
MH709312	9	g	c	c	t	a	t	t	g	t	c	t	t	t	t	g	g	a	c	a	a	g	a	c	c	a	t	g	g	c	c	t	t	t	c	a	g	g	a	a	t	a	g	g	c	t	c	c	c	g	a	a	g	g	a	c	g	t	c	t	g	a	t	t	g	c	t	a	c	c	t	t	c	g	g
MH709352	9	g	c	c	t	a	t	t	g	t	c	t	t	t	t	g	g	a	c	a	a	g	a	c	c	a	t	g	g	c	c	t	t	t	c	a	g	g	a	a	t	a	g	a	c	t	c	c	c	g	a	a	g	g	a	c	g	t	c	t	g	a	t	t	g	c	t	a	c	c	t	t	c	g	g
AB097933	Vac	g	c	c	t	a	t	t	g	t	c	t	t	t	t	g	g	a	c	a	a	g	a	c	c	a	t	g	g	c	c	t	t	t	c	a	g	g	a	a	t	g	g	g	c	t	c	c	c	g	a	a	g	g	a	c	g	t	c	t	g	a	t	t	t	t	t	a	c	c	t	t	c	g	g
JN704698	Vac	g	c	c	t	a	t	t	g	t	c	t	t	t	t	g	g	a	c	a	a	g	a	c	c	a	t	g	g	c	c	t	t	t	c	a	g	g	a	a	t	g	g	g	c	t	c	c	c	g	a	a	g	g	a	c	g	t	c	t	g	a	t	t	t	t	t	a	c	c	t	t	c	g	g
JN704697	Vac	g	c	c	t	a	t	t	g	t	c	t	t	t	t	g	g	a	c	a	a	g	a	c	c	a	t	g	g	c	c	t	t	t	c	a	g	g	a	a	t	g	g	g	c	c	c	c	c	g	a	a	g	g	a	c	g	t	c	t	g	a	t	t	t	t	t	a	c	c	t	t	c	g	g
DQ008355	Vac	g	c	c	t	a	t	t	g	t	c	t	t	t	t	g	g	a	c	a	a	g	a	c	c	a	t	g	g	c	c	t	t	t	c	a	g	g	a	a	t	g	g	g	c	y	c	c	c	g	a	a	g	g	a	c	g	t	c	t	g	a	t	t	t	t	t	a	c	c	t	t	c	g	g
JF306641	Vac	g	c	c	t	a	t	t	g	t	c	t	t	t	t	g	g	a	c	a	a	g	a	c	c	a	t	g	g	c	c	t	t	t	c	a	g	g	a	a	t	g	g	g	c	c	c	c	c	g	a	a	g	g	a	c	g	t	c	t	g	a	t	t	t	t	t	a	c	c	t	t	c	g	g
AB097932	Vac	g	c	c	t	a	t	t	g	t	c	t	t	t	t	g	g	a	c	a	a	g	a	c	c	a	t	g	g	c	c	t	t	t	c	a	g	g	a	a	t	g	g	g	c	t	c	c	c	g	a	a	g	g	a	c	g	t	c	t	g	a	t	t	t	t	t	a	c	c	t	t	c	g	g
DQ008354	Vac	g	c	c	t	a	t	t	g	t	c	t	t	t	t	g	g	a	c	a	a	g	a	c	c	a	t	g	g	c	c	t	t	t	c	a	g	g	a	a	t	g	g	g	c	y	c	c	c	g	a	a	g	g	a	c	g	t	c	t	g	a	t	t	t	t	t	a	c	c	t	t	c	g	g
KU926312	Vac	g	c	c	t	a	t	t	g	t	c	t	t	t	t	g	g	a	c	a	a	g	a	c	c	a	t	g	g	c	c	t	t	t	c	a	g	g	a	a	t	g	g	g	c	c	c	c	c	g	a	a	g	g	a	c	g	t	c	t	g	a	t	t	t	t	t	a	c	c	t	t	c	g	g
KU926313	Vac	g	c	c	t	a	t	t	g	t	c	t	t	t	t	g	g	a	c	a	a	g	a	c	c	a	t	g	g	c	c	t	t	t	c	a	g	g	a	a	t	g	g	g	c	c	c	c	c	g	a	a	g	g	a	c	g	t	c	t	g	a	t	t	t	t	t	a	c	c	t	t	c	g	g
KU926314	Vac	g	c	c	t	a	t	t	g	t	c	t	t	t	t	g	g	a	c	a	a	g	a	c	c	a	t	g	g	c	c	t	t	t	c	a	g	g	a	a	t	g	g	g	c	t	c	c	c	g	a	a	g	g	a	c	g	t	c	t	g	a	t	t	t	t	t	a	c	c	t	t	c	g	g
KF558371	Vac	g	c	c	t	a	t	t	g	t	c	t	t	t	t	g	g	a	c	a	a	g	a	c	c	a	t	g	g	c	c	t	t	t	c	a	g	g	a	a	t	g	g	g	c	c	c	c	c	g	a	a	g	g	a	c	g	t	c	t	g	a	t	t	t	t	t	a	c	c	t	t	c	g	g
KF558372	Vac	g	c	c	t	a	t	t	g	t	c	t	t	t	t	g	g	a	c	a	a	g	a	c	c	a	t	g	g	c	c	t	t	t	c	a	g	g	a	a	t	g	g	g	c	t	c	c	c	g	a	a	g	g	a	c	g	t	c	t	g	a	t	t	t	t	t	a	c	c	t	t	c	g	g
KF558373	Vac	g	c	c	t	a	t	t	g	t	c	t	t	t	t	g	g	a	c	a	a	g	a	c	c	a	t	g	g	c	c	t	t	t	c	a	g	g	a	a	t	g	g	g	c	c	c	c	c	g	a	a	g	g	a	c	g	t	c	t	g	a	t	t	t	t	t	a	c	c	t	t	c	g	g
KF558374	Vac	g	c	c	t	a	t	t	g	t	c	t	t	t	t	g	g	a	c	a	a	g	a	c	c	a	t	g	g	c	c	t	t	t	c	a	g	g	a	a	t	g	g	g	c	c	c	c	c	g	a	a	g	g	a	c	g	t	c	t	g	a	t	t	t	t	t	a	c	c	t	t	c	g	g
KF558375	Vac	g	c	c	t	a	t	t	g	t	c	t	t	t	t	g	g	a	c	a	a	g	a	c	c	a	t	g	g	c	c	t	t	t	c	a	g	g	g	a	t	g	g	g	c	c	c	c	c	g	a	a	g	g	a	c	g	t	c	t	g	a	t	t	t	t	t	a	c	c	t	t	c	g	g
KF558376	Vac	g	c	c	t	a	t	t	g	t	c	c	t	t	t	g	g	a	c	a	a	g	a	c	c	a	t	g	g	c	c	t	t	t	c	a	g	g	a	a	t	g	g	g	c	c	c	c	c	g	a	a	g	g	a	c	g	t	c	t	g	a	t	t	t	t	t	a	c	c	t	t	c	g	g
KF558377	Vac	g	c	c	t	a	t	t	g	t	c	t	t	t	t	g	g	a	c	a	a	g	a	c	c	a	t	g	g	c	c	t	t	t	c	a	g	g	a	a	t	g	a	g	c	c	c	c	c	g	c	a	g	g	a	c	g	t	c	t	g	a	t	t	t	t	t	a	c	c	t	t	c	g	g
KF558378	Vac	g	c	c	t	a	t	t	g	t	c	t	t	t	t	g	g	a	c	a	a	g	a	c	c	a	t	g	g	c	c	t	t	t	c	a	g	g	a	a	t	g	g	g	c	t	c	c	c	g	a	a	g	g	a	c	g	t	c	t	g	a	t	t	t	t	t	a	c	c	t	t	c	g	g
KF558379	Vac	g	c	c	t	a	t	t	g	t	c	t	t	t	t	g	g	a	c	a	a	g	a	c	c	a	t	g	g	c	c	t	t	t	c	a	g	g	a	a	t	g	g	g	c	c	c	c	c	g	a	a	g	g	a	c	g	t	c	t	g	a	t	t	t	t	t	a	c	c	t	t	c	g	g
KF558380	Vac	g	c	c	t	a	t	t	g	t	c	t	t	t	t	g	g	a	c	a	a	g	a	c	c	a	t	g	g	c	c	t	t	t	c	a	g	g	a	a	t	g	g	g	c	t	c	c	c	g	a	a	g	g	a	c	g	t	c	t	g	a	t	t	t	t	t	a	c	c	t	t	c	g	g
KF558381	Vac	g	c	c	t	a	t	t	g	t	c	t	t	t	t	g	g	a	c	a	a	g	a	c	c	a	t	g	g	c	c	t	t	t	c	a	g	g	a	a	t	g	g	g	c	c	c	c	c	g	a	a	g	g	a	c	g	t	c	t	g	a	t	t	t	t	t	a	c	c	t	t	c	g	g
KF558382	Vac	g	c	c	t	a	t	t	g	t	c	t	t	t	t	g	g	a	c	a	a	g	a	c	c	a	t	g	g	c	c	t	t	t	c	a	g	g	a	a	t	g	g	g	c	t	c	c	c	g	a	a	g	g	a	c	g	t	c	t	g	a	t	t	t	t	t	a	c	c	t	t	c	g	g
KF558383	Vac	g	c	c	t	a	t	t	g	t	c	t	t	t	t	g	g	a	c	a	a	g	a	c	c	a	t	g	g	c	c	t	t	t	c	a	g	g	a	a	t	g	g	g	c	t	c	c	c	g	a	a	g	g	a	c	g	t	c	t	g	a	t	t	t	t	t	a	c	c	t	t	c	g	g
KF558384	Vac	g	c	c	t	a	t	t	g	t	c	t	t	t	t	g	g	a	c	a	a	g	a	c	c	a	t	g	g	c	c	t	t	t	c	a	g	g	a	a	t	g	g	g	c	c	c	c	c	g	a	a	g	g	a	c	g	t	c	t	g	a	t	t	t	t	t	a	c	c	t	t	c	g	g
KF558385	Vac	g	c	c	t	a	t	t	g	t	c	t	t	t	t	g	g	a	c	a	a	g	a	c	c	a	t	g	g	c	c	t	t	t	c	a	g	g	a	a	t	g	g	g	c	c	c	c	c	g	a	a	g	g	a	c	g	t	c	t	g	a	t	t	t	t	t	a	c	c	t	t	c	g	g
KF558386	Vac	g	c	c	t	a	t	t	g	t	c	t	t	t	t	g	g	a	c	a	a	g	a	c	c	a	t	g	g	c	c	t	t	t	c	a	g	g	a	a	t	g	g	g	c	t	c	c	c	g	a	a	g	g	a	c	g	t	c	t	g	a	t	t	t	t	t	a	c	c	t	t	c	g	g
KF558387	Vac	g	c	c	t	a	t	t	g	t	c	t	t	t	t	g	g	a	c	a	a	g	a	c	c	a	t	g	g	c	c	t	t	t	c	a	g	g	a	a	t	g	g	g	c	t	c	c	c	g	a	a	g	g	a	c	g	t	c	t	g	a	t	t	t	t	t	a	c	c	t	t	c	g	g
KF558388	Vac	g	c	c	t	a	t	t	g	t	c	t	t	t	t	g	g	a	c	a	a	g	a	c	c	a	t	g	g	c	c	t	t	t	c	a	g	g	a	a	t	g	g	g	c	c	c	c	c	g	a	a	g	g	a	c	g	t	c	t	g	a	t	t	t	t	t	a	c	c	t	t	c	g	g
KF558389	Vac	g	c	c	t	a	t	t	g	t	c	t	t	t	t	g	g	a	c	a	a	g	a	c	c	a	t	g	g	c	c	t	t	t	c	a	g	g	a	a	t	g	g	g	c	c	c	c	c	g	a	a	g	g	a	c	g	t	c	t	g	a	t	t	t	t	t	a	c	c	t	t	c	g	g
KF558390	Vac	g	c	c	t	a	t	t	g	t	c	t	t	t	t	g	g	a	c	a	a	g	a	c	c	a	t	g	g	c	c	t	t	t	c	a	g	g	g	a	t	g	g	g	c	c	c	c	c	g	a	a	g	g	a	c	g	t	c	t	g	a	t	t	t	t	t	a	c	c	t	t	c	g	g
KF558391	Vac	g	c	c	t	a	t	t	g	t	c	t	t	t	t	g	g	a	c	a	a	g	a	c	c	a	t	g	g	c	c	t	t	t	c	a	g	g	a	a	t	g	g	g	c	t	c	c	c	g	a	a	g	g	a	c	g	t	c	t	g	a	t	t	t	t	t	g	c	c	t	t	c	g	g
KF811485	Vac	g	c	c	t	a	t	t	g	t	c	t	t	t	t	g	g	a	c	a	a	g	a	c	c	a	t	g	g	c	c	t	t	t	c	a	g	g	a	a	t	g	g	g	c	c	c	c	c	g	a	a	g	g	a	c	g	t	c	t	g	a	t	t	t	t	t	a	c	c	t	t	c	g	g
KF853225	Vac	g	c	c	t	a	t	t	g	t	c	t	t	t	t	g	g	a	c	a	a	g	a	c	c	a	t	g	g	c	c	t	t	t	c	a	g	g	a	a	t	g	g	g	c	c	c	c	c	g	a	a	g	g	a	c	g	t	c	t	g	a	t	t	t	t	t	a	c	c	t	t	c	g	g
KF853226	Vac	g	c	c	t	a	t	t	g	t	c	t	t	t	t	g	g	a	c	a	a	g	a	c	c	a	t	g	g	c	c	t	t	t	c	a	g	g	a	a	t	g	g	g	c	c	c	c	c	g	a	a	g	g	a	c	g	t	c	t	g	a	t	t	t	t	t	a	c	c	t	t	c	g	g
KF853227	Vac	g	c	c	t	a	t	t	g	t	c	t	t	t	t	g	g	a	c	a	a	g	a	c	c	a	t	g	g	c	c	t	t	t	c	a	g	g	a	a	t	g	g	g	c	t	c	c	c	g	a	a	g	g	a	c	g	t	c	t	g	a	t	t	t	t	t	a	c	c	t	t	c	g	g
KF853228	Vac	g	c	c	t	a	t	t	g	t	c	t	t	t	t	g	g	a	c	a	a	g	a	c	c	a	t	g	g	c	c	t	t	t	c	a	g	g	a	a	t	g	g	g	c	t	c	c	c	g	a	a	g	g	a	c	g	t	c	t	g	a	t	t	t	t	t	g	c	c	t	t	c	g	g
KF853229	Vac	g	c	c	t	a	t	t	g	t	c	t	t	t	t	g	g	a	c	a	a	g	a	c	c	a	t	g	g	c	c	t	t	t	c	a	g	g	a	a	t	g	g	g	c	c	c	c	c	g	a	a	g	g	a	c	g	t	c	t	g	a	t	t	t	t	t	a	c	c	t	t	c	g	g
KF853230	Vac	g	c	c	t	a	t	t	g	t	c	t	t	t	t	g	g	a	c	a	a	g	a	c	c	a	t	g	g	c	c	t	t	t	c	a	g	g	a	a	t	g	g	g	c	c	c	c	c	g	a	a	g	g	a	c	g	t	c	t	g	a	t	t	t	t	t	a	c	c	t	t	c	g	g
KF853231	Vac	g	c	c	t	a	t	t	g	t	c	t	t	t	t	g	g	a	c	a	a	g	a	c	c	a	t	g	g	c	c	t	t	t	c	a	g	g	a	a	t	g	g	g	c	t	c	c	c	g	a	a	g	g	a	c	g	t	c	t	g	a	t	t	t	t	t	a	c	c	t	t	c	g	g
KF853232	Vac	g	c	c	t	a	t	t	g	t	c	t	t	t	t	g	g	a	c	a	a	g	a	c	c	a	t	g	g	c	c	t	t	t	c	a	g	g	a	a	t	g	g	g	c	t	c	c	c	g	a	a	g	g	a	c	g	t	c	t	g	a	t	t	t	t	t	a	c	c	t	t	c	g	g
KF853233	Vac	g	c	c	t	a	t	t	g	t	c	t	t	t	t	g	g	a	c	a	a	g	a	c	c	a	t	g	g	c	c	t	t	t	c	a	g	g	a	a	t	g	g	g	c	c	c	c	c	g	a	a	g	g	a	c	g	t	c	t	g	a	t	t	t	t	t	a	c	c	t	t	c	g	g
KF853234	Vac	g	c	c	t	a	t	t	g	t	c	t	t	t	t	g	g	a	c	a	a	g	a	c	c	a	t	g	g	c	c	t	t	t	c	a	g	g	a	a	t	g	g	g	c	t	c	c	c	g	a	a	g	g	a	c	g	t	c	t	g	a	t	t	t	t	t	a	c	c	t	t	c	g	g
KF853235	Vac	g	c	c	t	a	t	t	g	t	c	t	t	t	t	g	g	a	c	a	a	g	a	c	c	a	t	g	g	c	c	t	t	t	c	a	g	g	a	a	t	g	g	g	c	t	c	c	c	g	a	a	g	g	a	c	g	t	c	t	g	a	t	t	t	t	t	a	c	c	t	t	c	g	g
MF898328	Vac	g	c	c	t	a	t	t	g	t	c	t	t	t	t	g	g	a	c	a	a	g	a	c	c	a	t	g	g	c	c	t	t	t	c	a	g	g	a	a	t	g	g	g	c	y	c	c	c	g	a	a	g	g	a	c	g	t	c	t	g	a	t	t	t	t	t	a	c	c	t	t	c	g	g
JN704709	VIII	g	c	c	t	a	t	t	g	t	c	t	t	t	t	g	g	a	c	a	a	g	a	c	c	a	t	g	g	c	c	t	t	t	c	a	g	g	a	a	t	g	g	g	c	c	c	c	c	g	a	a	g	g	a	c	g	t	c	t	g	a	t	t	t	t	t	a	c	c	t	t	c	g	g

Supplementary Table 3: 2880 SN

GB-ID	Clade markers															SD14					4						4	4													
	Position in Dumas	39227	39263	39304	39315	39346	39357	39394	39398	39516	39530	39537	39564	39866	39962	40031	40096	40130	40134	40151	40196	40370	40388	40484	40499	40534	40554	40556	40594	40619	40645	40660	40746	40752	40793	40818	40826	40982	41057	41133	41207
AY548170	1	t	g	g	g	a	a	g	t	t	a	c	c	t	c	t	t	c	t	t	c	a	a	a	c	t	g	g	t	g	g	t	t	a	g	g	a	t	t	a	g
AY548171	1	t	g	g	g	a	a	g	t	t	a	c	c	t	c	t	t	c	t	t	c	a	a	a	c	t	g	g	t	g	g	c	t	a	g	g	a	t	t	a	g
DQ479953	1	t	g	g	g	a	a	g	t	t	a	c	c	t	c	t	t	c	t	t	c	a	a	a	c	t	g	g	t	g	g	t	t	a	g	g	a	t	t	g	g
DQ479954	1	t	g	g	g	a	a	g	t	t	a	c	c	t	c	t	t	c	t	t	c	a	a	a	c	t	g	g	t	g	g	t	t	a	g	g	a	t	t	a	a
DQ479958	1	t	g	g	g	a	a	g	t	t	a	c	c	t	c	t	t	c	t	t	c	a	a	a	c	t	g	g	t	g	g	t	t	a	g	g	a	t	t	a	g
DQ479959	1	t	g	g	g	a	a	g	t	t	a	c	c	t	c	t	t	c	t	t	c	a	a	a	c	t	g	g	t	g	g	t	t	a	g	g	a	t	t	a	g
DQ479961	1	t	g	g	g	a	a	g	t	t	a	c	c	t	c	t	t	c	t	t	c	a	a	a	c	t	g	g	t	g	g	t	t	a	g	g	a	t	t	a	g
DQ479962	1	t	g	g	g	a	a	g	t	t	a	c	c	t	c	t	t	c	t	t	c	a	a	a	c	t	g	g	t	g	g	t	t	a	g	g	a	t	t	a	g
DQ479963	1	t	g	g	g	a	a	g	t	t	a	c	c	t	c	t	t	c	t	t	c	a	a	a	c	t	g	g	t	g	g	t	t	a	g	g	a	t	t	a	g
DQ674250	1	t	g	g	g	a	a	g	t	t	a	c	c	t	c	t	t	c	t	t	c	a	a	a	c	t	g	g	t	g	g	t	t	a	g	g	a	t	t	a	g
KX262864	1	t	g	g	g	a	a	g	t	t	a	c	c	t	c	t	t	c	t	t	c	a	a	a	c	t	g	g	t	g	g	t	t	a	g	g	a	t	t	a	g
KX262865	1	t	g	g	g	a	a	g	t	t	a	c	c	t	c	t	t	c	t	t	c	a	a	a	c	t	g	g	t	g	g	t	t	a	g	g	a	t	t	a	g
KX262866	1	t	g	g	g	a	a	g	t	t	a	c	c	t	c	t	t	c	t	t	c	a	a	a	c	t	g	g	t	g	g	t	t	a	g	g	a	t	t	a	g
EU154348	1	t	g	g	g	a	a	g	t	t	a	c	c	t	c	t	t	c	t	t	c	a	a	a	c	t	g	g	t	g	g	t	t	a	g	g	a	t	t	a	g
JN704690	1	t	g	g	g	a	a	g	t	t	a	c	c	t	c	t	t	c	t	t	c	a	a	a	c	t	g	g	t	g	g	t	t	a	g	g	a	t	t	a	g
JN704691	1	t	g	g	g	a	a	g	t	t	a	c	c	t	c	t	t	c	t	t	c	a	a	a	c	t	g	g	t	g	g	t	t	a	g	g	a	t	t	a	g
JN704692	1	t	g	g	g	a	a	g	t	t	a	c	t	t	c	t	t	c	t	t	c	a	a	a	c	t	g	g	t	g	g	t	t	a	g	g	a	t	t	a	g
JN704693	1	t	g	g	g	a	a	g	t	t	a	c	c	t	c	t	t	c	t	t	c	a	a	a	c	t	g	g	t	g	g	t	t	a	g	g	a	t	t	a	g
JN704694	1	t	g	g	g	a	a	g	t	t	a	c	c	t	c	t	t	c	t	t	c	a	a	a	c	t	g	g	t	g	g	t	t	a	g	g	a	t	t	a	g
JN704695	1	t	g	g	g	a	a	g	t	t	a	c	c	t	c	t	t	c	t	t	c	a	a	a	c	t	g	g	t	g	g	t	t	a	g	g	a	t	t	a	g
JN704696	1	t	g	g	g	a	a	g	t	t	a	c	c	t	c	t	t	c	t	t	c	a	a	a	c	t	g	g	t	g	g	t	t	a	g	g	a	t	t	a	g
NC001348	1	t	g	g	g	a	a	g	t	t	a	c	c	t	c	t	t	c	t	t	c	a	a	a	c	t	g	g	t	g	g	t	t	a	g	g	a	t	t	a	g
KP771892	1	t	g	g	g	a	a	g	t	t	a	c	c	t	c	t	t	c	t	t	c	a	a	a	c	t	g	g	t	g	g	t	t	a	g	g	a	t	t	a	g
KP771894	1	t	g	g	g	a	a	g	t	t	a	c	c	t	c	t	t	c	t	t	c	a	a	a	c	t	g	g	t	g	g	t	t	a	g	g	a	t	t	a	g
KP771900	1	t	g	g	g	a	a	g	t	t	a	c	c	t	c	t	t	c	t	t	c	a	a	a	c	t	g	g	t	g	g	t	t	a	g	g	a	t	t	a	g
KP771901	1	t	g	g	g	a	a	g	t	t	a	c	c	t	c	t	t	c	t	t	c	a	a	a	c	t	g	g	t	g	g	t	t	a	g	g	a	t	t	a	g
KP771902	1	t	g	g	g	a	a	g	t	t	a	c	c	t	c	t	t	c	t	t	c	a	a	a	c	t	g	g	t	g	g	t	t	a	g	g	a	t	t	a	g
KP771908	1	t	g	g	g	a	a	g	t	t	a	c	c	t	c	t	t	c	t	t	c	a	a	a	c	t	g	g	t	g	g	t	t	a	g	g	a	t	t	a	g
KP771912	1	t	g	g	g	a	a	g	t	t	a	c	c	t	c	t	t	c	t	t	c	a	a	a	c	t	g	g	t	g	g	t	t	a	g	g	a	t	t	a	g
KP771913	1	t	g	g	g	a	a	g	t	t	a	c	c	t	c	t	t	c	t	t	c	a	a	a	c	t	g	g	t	g	g	t	t	a	g	g	a	t	t	a	g
KP771917	1	t	g	g	g	a	a	g	t	t	a	c	c	t	c	t	t	c	t	t	c	a	a	a	c	t	g	g	t	g	g	t	t	a	g	g	a	t	t	a	g
KP771918	1	t	g	g	g	a	a	g	t	t	a	c	c	t	c	t	t	c	t	t	c	a	a	a	c	t	g	g	t	g	g	t	t	a	g	g	a	t	t	a	g
KP771920	1	t	g	g	g	a	a	g	t	t	a	c	c	t	c	t	t	c	t	t	c	a	a	a	c	t	g	g	t	g	g	t	t	a	g	g	a	t	t	a	g
MH709361	1	t	g	g	g	a	a	g	t	t	a	c	c	t	c	t	t	c	t	g	c	a	a	a	c	t	g	g	t	g	g	t	t	a	g	g	a	t	t	a	g
KU926318	2	t	a	g	g	a	a	a	t	t	g	c	c	t	c	t	t	c	t	t	c	a	g	a	c	t	g	g	t	g	g	t	t	a	g	g	a	t	c	a	g
KU926319	2	t	a	g	g	a	a	a	t	t	g	c	c	t	c	t	t	t	t	t	c	g	g	a	c	t	g	g	t	g	g	t	t	a	g	g	a	t	c	a	g
KU926320	2	t	a	g	g	a	a	a	t	t	g	c	c	t	c	t	t	t	t	t	c	g	g	a	c	t	g	g	t	g	g	t	t	a	g	g	a	t	c	a	g
KU926321	2	t	a	g	g	a	a	a	t	t	g	c	c	t	c	t	t	t	t	t	c	g	g	a	g	t	g	g	t	g	g	t	t	a	g	g	a	t	c	a	g
KU926322	2	t	a	g	g	a	a	a	t	t	g	c	c	t	c	t	t	t	t	t	c	g	g	a	g	t	g	g	t	g	g	t	t	a	g	g	a	t	c	a	g
KC847290	2	t	a	g	g	a	a	a	t	t	g	c	c	t	c	t	t	t	t	t	c	g	g	a	c	t	g	g	t	g	g	t	t	a	g	g	a	t	c	a	g
KJ767491	2	t	a	g	g	a	a	a	t	t	g	c	c	t	c	t	t	c	t	t	c	a	g	a	c	t	g	g	t	g	g	t	t	a	g	g	a	t	c	a	g
KJ767492	2	t	a	g	g	a	a	a	t	t	g	c	c	t	c	t	t	t	t	t	c	g	g	a	c	t	g	g	t	g	g	t	t	a	g	g	a	t	c	a	g
KJ808816	2	t	a	g	g	a	a	a	t	t	g	c	c	t	c	t	t	t	t	t	c	g	g	a	g	t	g	g	t	g	g	t	t	a	g	g	a	t	c	a	g
KP771925	2	t	a	g	a	a	g	a	t	t	g	c	c	t	c	t	t	t	t	t	c	g	g	a	c	t	g	g	t	g	g	t	t	a	g	g	a	t	c	a	g
KU926315	2	t	a	g	g	a	a	a	t	t	g	c	c	t	c	t	t	c	t	t	c	a	g	a	c	t	g	g	t	g	g	t	t	a	g	g	a	t	c	a	g
KU926316	2	t	a	g	g	a	a	a	t	t	g	c	c	t	c	t	t	c	t	t	c	a	g	a	c	t	g	g	t	g	g	t	t	a	g	g	a	t	c	a	g
KU926317	2	t	a	g	g	a	a	a	t	t	g	c	c	t	c	t	t	c	t	t	c	a	g	a	c	t	g	g	t	g	g	t	t	a	g	g	a	t	c	a	g
KY037796	2	t	a	g	g	a	a	a	t	t	g	c	c	t	c	t	t	c	t	t	c	a	g	a	c	t	g	g	t	g	g	t	t	a	g	g	a	t	c	a	g
MF004348	2	t	a	g	g	a	a	a	t	t	g	c	c	t	c	t	t	t	t	t	c	g	g	a	c	t	g	g	t	g	g	t	t	a	g	g	a	t	c	a	g
MH709309	2	t	a	g	g	a	a	a	t	t	g	c	c	t	c	t	t	c	t	t	c	a	g	a	c	t	g	g	t	g	g	t	t	a	g	g	a	t	c	a	g
MH709310	2	t	a	g	g	a	a	a	t	t	g	c	c	t	c	t	t	c	t	t	c	a	g	a	c	t	g	g	t	g	g	t	t	a	g	g	a	t	c	a	g
MH709311	2	t	a	g	g	a	a	a	t	t	g	c	c	t	c	t	t	c	t	t	c	a	g	a	c	t	g	g	t	g	g	t	t	a	g	g	a	t	c	a	g
MH709313	2	t	a	g	g	a	a	a	t	t	g	c	c	t	c	t	t	c	t	t	c	a	g	a	c	t	g	g	t	g	g	t	t	a	g	g	a	t	c	a	g
MH709314	2	t	a	g	g	a	a	a	t	t	g	c	c	t	c	t	t	c	t	t	c	a	g	a	c	t	g	g	t	g	g	t	t	a	g	g	a	t	c	a	g
MH709315	2	t	a	g	g	a	a	a	t	t	g	c	c	t	c	t	t	c	t	t	c	a	g	a	c	t	g	g	t	g	g	t	t	a	g	g	a	t	c	a	g
MH709319	2	t	a	g	g	a	a	a	t	t	g	c	c	t	c	t	t	c	t	t	c	a	g	a	c	t	g	g	t	g	g	t	t	a	g	g	a	t	c	a	g
MH709320	2	t	a	g	g	a	a	a	t	t	g	c	c	t	c	t	t	c	t	t	c	a	g	a	c	t	g	g	t	g	g	t	t	a	g	g	a	t	c	a	g
MH709329	2	t	a	g	g	a	a	a	t	t	g	c	c	t	c	t	t	c	t	t	c	a	g	a	c	t	g	g	t	g	g	t	t	a	g	g	a	t	c	a	g
MH709331	2	t	a	g	g	a	a	a	t	t	g	c	c	t	c	t	t	c	t	t	c	a	g	a	c	t	g	g	t	g	g	t	t	a	g	g	a	t	c	a	g
MH709333	2	t	a	g	g	a	a	a	t	t	g	c	c	t	c	t	t	c	t	t	c	a	g	a	c	t	g	g	t	g	g	t	t	a	g	g	a	t	c	a	g
MH709337	2	t	a	g	g	a	a	a	t	t	g	c	c	t	c	t	t	c	t	t	c	a	g	a	c	t	g	g	t	g	g	t	t	a	g	g	a	t	c	a	g
MH709339	2	t	a	g	g	a	a	a	t	t	g	c	c	t	c	t	t	c	t	t	c	a	g	a	c	t	g	g	t	g	g	t	t	a	g	g	c	t	c	a	g
MH709344	2	t	a	a	g	c	a	a	t	t	g	c	c	t	c	t	t	c	t	t	c	a	g	a	c	t	g	g	t	g	g	t	t	a	g	g	a	t	c	a	g
MH709348	2	t	a	g	g	a	a	a	t	t	g	c	c	t	c	t	t	c	t	t	c	a	g	a	c	t	g	g	t	g	g	t	t	a	g	g	a	t	c	a	g
MH709355	2	t	a	g	g	a	a	a	t	t	g	c	c	t	c	t	t	c	t	t	c	a	g	a	c	t	g	g	t	g	g	t	t	a	g	g	a	t	c	a	g
MH709359	2	t	a	g	g	a	a	a	t	t	g	c	c	t	c	t	t	c	t	t	c	a	g	a	c	t	g	g	t	g	g	t	t	a	g	g	a	t	c	a	g
MH709363	2	t	a	g	g	a	a	a	t	t	g	c	c	t	c	t	t	c	t	t	c	a	g	a	c	t	g	g	t	g	g	t	t	a	g	g	a	t	c	a	g
MH709366	2	t	a	g	g	a	a	a	g	t	g	c	c	t	c	t	t	c	t	t	c	a	g	a	c	t	g	g	t	g	g	t	t	a	g	g	a	t	c	a	g
MH709376	2	t	a	g	g	a	a	a	t	t	g	c	c	t	c	t	t	c	t	t	c	a	g	a	c	t	g	g	t	g	g	t	t	a	g	g	a	t	c	a	g
MH709377	2	t	a	g	g	a	a	a	t	t	g	c	c	t	c	t	t	t	t	t	c	g	g	a	c	t	g	g	t	g	g	t	t	a	g	g	a	t	c	a	g
**MW316406**	2	t	a	g	g	a	a	a	t	t	g	c	c	t	c	g	t	c	t	t	c	a	g	a	c	t	g	g	t	g	g	t	t	a	g	g	a	t	c	a	g
AJ871403	3	t	g	g	g	a	a	a	t	t	g	c	c	t	c	t	t	c	t	t	c	a	g	a	c	t	g	g	t	g	g	t	t	a	g	g	a	t	t	a	g
DQ479955	3	t	g	g	g	a	a	a	t	t	g	c	c	t	c	t	t	c	t	t	c	a	g	a	t	t	g	g	t	g	g	t	t	a	g	g	a	t	t	a	g
DQ479956	3	t	g	g	g	a	a	a	t	t	g	c	c	t	c	t	t	c	t	t	c	a	g	a	c	t	g	g	t	g	g	t	t	a	g	g	a	t	t	a	g
DQ479957	3	t	g	g	g	a	a	a	t	t	g	c	c	t	c	t	t	c	t	t	c	a	g	a	c	t	g	g	t	g	g	t	t	a	g	g	a	t	t	a	g
JN704699	3	t	g	g	g	a	a	a	t	t	g	c	c	t	c	t	t	c	t	t	c	a	g	a	c	t	g	g	t	g	g	t	t	a	g	g	a	t	t	a	g
JN704700	3	t	g	g	g	a	a	a	t	t	g	c	c	t	c	t	t	c	t	t	c	a	a	a	c	t	g	g	t	g	g	t	t	a	g	g	a	t	t	a	g
JN704701	3	t	g	g	g	a	a	a	t	t	g	c	c	t	c	t	t	c	t	t	c	a	g	a	g	t	g	g	t	g	g	t	t	a	g	g	a	t	t	a	g
JN704702	3	t	g	g	g	a	a	a	t	t	a	c	c	t	c	t	t	c	t	t	c	a	g	g	c	t	g	g	t	g	g	t	t	a	g	g	a	t	t	a	g
JN704703	3	t	g	g	g	a	a	a	t	t	g	c	c	t	c	t	t	c	t	t	c	a	g	a	c	t	g	g	t	g	g	t	t	a	g	g	a	t	t	a	g
KP771891	3	t	g	g	g	a	a	a	t	t	g	c	c	t	c	t	t	c	t	t	c	a	g	a	c	t	g	g	t	g	g	t	t	a	g	g	a	t	t	a	g
KP771893	3	t	g	g	g	a	a	a	t	t	g	c	c	t	c	t	t	c	t	t	c	a	g	a	c	t	g	g	t	g	g	t	t	a	g	g	a	t	t	a	g
KP771895	3	t	g	g	g	a	a	a	t	t	g	c	c	t	c	t	t	c	t	t	c	a	g	a	c	t	g	g	t	g	g	t	t	a	g	g	a	t	t	a	g
KP771896	3	t	g	g	g	a	a	a	t	t	g	c	c	t	c	t	t	c	c	t	c	a	g	a	c	t	g	g	t	g	g	t	t	a	g	g	a	t	t	a	g
KP771897	3	t	g	g	g	a	a	a	t	t	g	c	c	t	c	t	t	c	t	t	c	a	g	a	c	t	g	g	t	g	g	t	t	a	g	g	a	t	t	a	g
KP771899	3	t	g	g	g	a	a	a	t	t	g	c	c	t	c	t	t	c	t	t	c	a	g	a	c	t	g	g	t	g	g	t	t	a	g	g	a	t	t	a	g
KP771903	3	t	g	g	g	a	a	a	t	t	g	c	c	t	c	t	t	c	t	t	c	a	g	a	c	t	g	g	t	g	g	t	t	a	g	g	a	t	t	a	g
KP771907	3	t	g	g	g	a	a	a	t	t	g	c	c	t	c	t	t	c	t	t	c	a	g	a	c	t	g	g	t	g	g	t	t	a	g	g	a	t	t	a	g
KP771910	3	t	g	g	g	a	a	a	t	t	g	c	c	t	c	t	t	c	t	t	c	a	g	a	c	t	g	g	t	g	g	t	t	a	g	g	a	t	t	a	g
KP771915	3	t	g	g	g	a	a	a	t	t	g	c	c	t	c	t	t	c	t	t	c	a	g	a	c	t	g	g	t	g	g	t	t	a	g	g	a	t	t	a	g
KP771916	3	t	g	g	g	a	a	a	t	t	g	c	c	t	c	t	t	c	t	t	c	a	g	a	c	t	g	g	t	g	g	t	t	a	g	g	a	t	t	a	g
KP771921	3	t	g	g	g	a	a	a	t	t	g	c	c	t	c	t	t	c	t	t	c	a	g	a	c	t	g	g	t	g	g	t	t	a	g	g	a	t	t	a	g
KP771923	3	t	g	g	g	a	a	a	t	t	g	c	c	t	c	t	t	c	t	t	c	a	g	a	c	t	g	g	t	g	g	t	t	a	g	g	a	t	t	a	g
KP771889	3	t	g	g	g	a	a	a	t	t	g	c	c	t	c	t	t	c	t	t	c	a	g	a	c	t	g	g	t	g	g	t	t	a	g	g	a	t	t	a	g
KU926311	3	t	g	g	g	a	a	a	t	t	g	c	c	t	c	t	t	c	t	t	c	a	g	a	c	t	g	g	t	g	g	t	t	a	g	g	a	t	t	a	g
JQ911599	3	t	g	g	g	a	a	a	t	t	g	c	c	t	c	t	t	c	t	t	c	a	g	a	c	t	g	g	t	g	g	t	t	a	g	g	a	t	t	a	g
MH709317	3	t	g	g	g	a	a	a	t	t	g	c	c	t	c	t	t	c	t	t	c	a	g	a	c	t	g	g	t	g	g	t	t	a	g	g	a	t	t	a	g
MH709346	3	t	g	g	g	a	a	a	t	t	g	c	c	t	c	t	t	c	t	t	c	a	g	a	c	t	g	g	t	g	g	t	t	a	g	g	a	t	t	a	g
MH709375	3	t	g	g	g	a	a	a	t	t	g	c	c	t	c	t	t	c	t	t	c	a	g	a	c	t	g	g	t	g	g	t	t	a	g	g	a	t	t	a	g
DQ452050	4	t	g	g	g	a	a	g	t	t	a	c	c	t	c	t	t	c	t	t	t	a	g	a	c	t	a	a	t	g	g	t	t	a	g	g	a	t	t	a	g
DQ479960	4	t	g	g	g	a	a	g	t	t	a	c	c	t	c	t	t	c	t	t	t	a	g	a	c	t	a	a	t	g	g	t	t	a	g	g	a	t	t	a	g
KP771898	4	t	g	g	g	a	a	g	t	t	a	c	c	t	c	t	t	c	t	t	t	a	g	a	c	t	a	a	t	g	g	t	t	a	g	g	a	t	t	a	g
MH709316	4	t	a	g	g	a	a	a	t	t	a	c	c	t	c	t	t	c	t	t	t	a	g	a	c	t	a	a	t	g	g	t	t	a	g	g	a	t	t	a	g
MH709318	4	t	g	g	g	a	a	g	t	t	a	c	c	t	c	t	t	c	t	t	t	a	g	a	c	t	a	a	t	g	g	t	t	a	g	g	a	t	t	a	g
MH709322	4	t	g	g	g	a	a	g	t	t	a	c	c	t	c	t	t	c	t	t	t	a	g	a	c	t	a	a	t	g	g	t	t	a	g	g	a	t	t	a	g
MH709323	4	t	g	g	g	a	a	g	t	t	a	c	c	t	c	t	t	c	t	t	t	a	g	a	c	t	a	a	t	g	g	t	t	a	g	g	a	t	t	a	g
MH709326	4	t	g	g	g	a	a	g	t	t	a	c	c	t	c	t	t	c	t	t	t	a	g	a	c	t	a	a	t	g	g	t	t	a	g	g	a	t	t	a	g
MH709341	4	t	g	g	g	a	a	g	t	t	a	c	c	t	c	t	t	c	t	t	t	a	g	a	c	t	a	a	t	g	g	t	t	a	g	g	a	t	t	a	g
MH709357	4	t	g	g	g	a	a	g	t	t	a	c	c	t	c	t	t	c	t	t	t	a	g	a	c	t	a	a	t	g	g	t	t	a	g	g	a	t	t	a	g
MH709364	4	t	g	g	g	a	a	g	t	t	a	c	c	c	c	t	t	c	t	t	t	a	g	a	c	t	a	a	t	g	g	t	t	a	g	g	a	t	t	a	g
MH709370	4	t	g	g	g	a	a	g	t	t	a	c	c	t	c	t	t	c	t	t	t	a	g	a	c	t	a	a	t	g	g	t	t	a	g	g	a	t	t	a	g
MH709371	4	t	g	g	g	a	a	g	t	t	a	c	c	t	c	t	t	c	t	t	t	a	g	a	c	t	a	a	t	g	a	t	t	a	g	g	a	g	t	a	g
MH709372	4	t	g	g	g	a	a	g	t	t	a	c	c	t	c	t	t	c	t	t	t	a	g	a	c	t	a	a	t	g	g	t	t	a	g	g	a	t	t	a	g
DQ457052	5	t	g	g	g	a	a	a	t	t	g	c	c	t	c	t	t	c	t	t	c	a	g	a	c	t	g	g	t	g	g	t	g	a	g	g	a	t	t	a	g
JN704704	5	t	g	g	g	a	a	a	t	t	g	c	c	t	c	t	t	c	t	t	c	a	g	a	c	t	g	g	t	g	g	t	t	a	g	g	a	t	t	a	g
JN704705	5	t	g	g	g	a	a	a	t	t	g	c	c	t	t	t	t	c	t	t	c	a	g	a	c	t	g	g	t	g	g	t	t	a	g	g	a	t	t	a	g
JN704706	5	t	g	g	g	a	a	a	t	t	g	c	c	t	c	t	t	c	t	t	c	a	g	a	c	t	g	g	t	g	g	t	t	a	g	g	a	t	t	a	g
JN704707	5	t	g	g	g	a	a	a	t	t	a	c	c	t	c	t	t	c	t	t	c	a	g	a	c	t	g	g	t	g	g	t	t	a	a	g	a	t	t	a	g
JN704708	5	t	g	g	g	a	a	a	t	t	g	c	c	t	t	t	t	c	t	t	c	a	g	a	c	t	g	g	t	g	g	t	t	a	g	g	a	t	t	a	g
KP771904	5	t	g	g	g	a	a	a	t	t	g	c	c	t	c	t	t	c	t	t	c	a	g	a	c	t	g	g	t	g	g	t	t	a	g	g	a	t	t	a	g
KP771905	5	t	g	g	g	a	a	a	t	t	g	c	c	t	c	t	t	c	t	t	c	a	g	a	c	t	g	g	t	g	g	t	t	a	g	g	a	t	t	a	g
KP771906	5	t	g	g	g	a	a	a	t	t	g	c	c	t	c	t	t	c	t	t	c	a	g	a	c	t	g	g	t	g	g	t	t	a	g	a	a	t	t	a	g
KP771919	5	t	g	g	g	a	a	a	t	t	g	c	c	t	c	t	t	c	t	t	c	a	g	a	c	t	g	g	t	g	g	t	t	a	g	g	a	t	t	a	g
KP771922	5	t	g	g	g	a	a	a	t	t	g	c	c	t	c	t	t	c	t	t	c	a	g	a	c	t	g	g	t	g	g	t	t	a	g	g	a	t	t	a	g
KP771924	5	t	g	g	g	a	a	a	t	t	g	c	c	t	c	t	t	c	t	t	c	a	g	a	c	t	g	g	t	g	g	t	t	a	g	g	a	t	t	a	g
KM355696	5	t	g	g	g	a	a	a	t	t	g	c	c	t	c	t	t	c	t	t	c	a	g	a	c	t	g	g	t	g	g	t	t	a	g	g	a	t	t	a	g
KM355697	5	t	g	g	g	a	a	a	t	t	g	c	c	t	c	t	t	c	t	t	c	a	g	a	c	t	g	g	t	g	g	t	t	a	g	g	a	t	t	a	g
KM355698	5	t	g	g	g	a	a	a	t	t	g	c	c	t	c	t	t	c	t	t	c	a	g	a	c	t	g	g	t	g	g	t	t	a	g	g	a	t	t	a	g
KM355699	5	t	g	g	g	a	a	a	t	t	g	c	c	t	c	t	t	c	t	t	c	a	g	a	c	t	g	g	t	g	g	t	t	a	g	g	a	t	t	a	g
KM355701	5	t	g	g	g	a	a	a	t	t	g	c	c	t	c	t	t	c	t	t	c	a	g	a	c	t	g	g	t	g	g	t	t	a	g	g	a	t	t	a	g
KM355702	5	t	g	g	g	a	a	a	t	t	g	c	c	t	c	t	t	c	t	t	c	a	g	a	c	t	g	g	t	g	g	t	t	a	g	g	a	t	t	a	g
KM355703	5	t	g	g	g	a	a	a	t	t	g	c	c	t	c	t	t	c	t	t	c	a	g	a	c	t	g	g	t	g	g	t	t	a	g	g	a	t	t	a	g
KM355704	5	t	g	g	g	a	a	a	t	t	g	c	c	t	c	t	t	c	t	t	c	a	g	a	c	t	g	g	t	g	g	t	t	a	g	g	a	t	t	a	g
KM355705	5	t	g	g	g	a	a	a	t	t	g	c	c	t	c	t	t	c	t	t	c	a	g	a	c	t	g	g	t	g	g	t	t	a	g	g	a	t	t	a	g
KM355706	5	t	g	g	g	a	a	a	t	t	g	c	c	t	c	t	t	c	t	t	c	a	g	a	c	t	g	g	t	g	g	t	t	a	g	g	a	t	t	a	g
KM355707	5	t	g	g	g	a	a	a	t	t	g	c	c	t	c	t	t	c	t	t	c	a	g	a	c	t	g	g	t	g	g	t	t	a	g	g	a	t	t	a	g
KM355708	5	t	g	g	g	a	a	a	t	t	g	c	c	t	c	t	t	c	t	t	c	a	g	a	c	t	g	g	t	g	g	t	t	a	g	g	a	t	t	a	g
KM355709	5	t	g	g	g	a	a	a	t	t	g	c	c	t	c	t	t	c	t	t	c	a	g	a	c	t	g	g	t	g	g	t	t	a	g	g	a	t	t	a	g
KM355711	5	t	g	g	g	a	a	a	t	t	g	c	c	t	c	t	t	c	t	t	c	a	g	a	c	t	g	g	t	g	g	t	t	a	g	g	a	t	t	a	g
KM355712	5	t	g	g	g	a	a	a	t	t	g	c	c	t	c	t	t	c	t	t	c	a	g	a	c	t	g	g	t	g	g	t	t	a	g	g	a	t	t	a	g
KY037797	[illegible]	[illegible]	[illegible]	[illegible]	[illegible]	[illegible]	[illegible]	[illegible]	[illegible]	[illegible]	[illegible]	[illegible]	[illegible]	[illegible]	[illegible]	[illegible]	[illegible]	[illegible]	[illegible]	[illegible]	[illegible]	[illegible]	[illegible]	[illegible]	[illegible]	[illegible]	[illegible]	[illegible]	[illegible]	[illegible]	[illegible]	[illegible]	[illegible]	[illegible]	[illegible]	[illegible]	[illegible]	[illegible]	[illegible]	[illegible]	[illegible]

GB-ID																																	
	41212	41213	41333	41372	41443	41544	41558	41588	41618	41715	41734	41737	41762	41764	41777	41799	41873	41883	41885	41966	41969	41993	42033	42036	42053	42069	42110	42118	42176	42242	42372_1	42372_2	42380
AY548170	c	a	c	t	t	g	g	c	c	g	a	a	c	g	t	g	a	t	c	g	a	g	t	a	g	c	g	a	c	c	-	-	c
AY548171	c	a	c	t	t	g	g	c	c	g	a	a	c	g	t	g	a	t	c	g	a	g	t	a	g	c	g	a	c	c	-	-	c
DQ479953	c	a	c	t	t	g	g	c	c	g	a	a	c	g	t	g	a	t	c	g	a	g	t	a	g	c	g	a	c	c	-	-	c
DQ479954	c	a	c	t	t	g	g	c	c	g	a	a	c	g	t	g	a	t	c	g	a	g	t	a	g	c	g	a	c	c	-	-	c
DQ479958	c	a	c	t	t	g	g	c	c	g	a	a	c	g	t	g	a	t	c	g	a	g	t	a	g	c	g	a	c	c	-	-	c
DQ479959	c	a	c	t	t	g	g	c	c	g	a	a	c	g	t	g	a	t	c	g	a	g	t	a	g	c	g	a	c	c	-	-	c
DQ479961	c	a	c	t	t	g	g	c	c	g	a	a	c	g	t	g	a	t	c	g	a	g	t	a	g	c	g	a	c	c	-	-	c
DQ479962	c	a	c	t	t	g	g	c	c	g	a	a	c	g	t	g	a	t	c	g	a	g	t	a	g	c	g	a	c	c	-	-	c
DQ479963	c	a	c	t	t	g	g	c	c	g	a	a	c	g	t	g	a	t	c	g	a	g	t	a	g	c	g	a	c	c	-	-	c
DQ674250	c	a	c	t	t	g	g	c	c	g	a	a	c	g	t	g	a	t	c	g	a	g	t	a	g	c	g	a	c	c	-	-	c
KX262864	c	a	c	t	t	g	g	c	c	g	a	a	c	g	t	g	a	t	c	g	a	g	t	a	g	c	g	a	c	c	-	-	c
KX262865	c	a	c	t	t	g	g	c	c	g	a	a	c	g	t	g	a	t	c	g	a	g	t	a	g	c	g	a	c	c	-	-	c
KX262866	c	a	c	t	t	g	g	c	c	g	a	a	c	g	t	g	a	t	c	g	a	g	t	a	g	c	g	a	c	c	-	-	c
EU154348	c	a	c	t	t	g	g	c	c	g	a	a	c	g	t	g	a	t	c	g	a	g	t	a	g	c	g	a	c	c	-	-	c
JN704690	c	a	c	t	t	g	g	c	c	g	a	a	c	g	t	g	a	t	c	g	a	g	t	a	g	c	g	a	c	c	-	-	c
JN704691	c	a	c	t	t	g	g	t	c	g	a	a	c	g	t	g	a	t	c	g	a	g	t	a	g	c	g	a	c	a	-	-	c
JN704692	c	a	c	t	t	g	g	c	c	g	a	a	c	g	t	g	a	t	c	a	a	g	t	a	g	c	g	a	c	c	-	-	c
JN704693	c	a	c	t	t	g	g	c	c	g	a	a	c	g	t	g	a	t	c	g	a	g	t	a	g	c	g	a	c	c	-	-	c
JN704694	c	a	c	t	t	g	g	c	c	g	a	a	c	g	t	g	a	t	c	g	a	g	t	a	g	c	g	a	c	c	-	-	c
JN704695	c	a	c	t	t	g	g	c	c	g	a	a	c	g	t	g	a	t	c	g	a	g	t	a	g	c	g	a	c	c	-	-	c
JN704696	c	a	c	t	t	g	g	c	c	g	a	a	c	g	t	g	a	t	c	g	a	g	t	a	g	c	g	a	c	a	-	-	c
NC001348	c	a	c	t	t	g	g	c	c	g	a	a	c	g	t	g	a	t	c	g	a	g	t	a	g	c	g	a	c	c	-	-	c
KP771892	c	a	c	t	t	g	g	c	c	g	a	a	c	g	t	g	a	t	c	g	a	g	t	a	g	c	g	a	c	c	-	-	c
KP771894	c	a	c	t	t	g	g	c	c	g	a	a	c	g	t	g	a	t	c	g	a	g	t	a	g	c	g	a	c	c	-	-	c
KP771900	c	a	c	t	t	g	g	c	c	g	a	a	c	g	t	g	a	t	c	g	a	g	t	a	g	c	g	a	c	c	-	-	c
KP771901	c	a	c	t	t	g	g	c	c	g	a	a	c	g	t	g	a	t	c	g	a	g	t	a	g	c	g	a	c	c	-	-	c
KP771902	c	a	c	t	t	g	g	c	c	g	a	a	c	g	t	g	a	t	c	g	a	g	t	a	g	c	g	a	c	c	-	-	c
KP771908	c	a	c	t	t	g	g	c	c	g	a	a	c	g	t	g	a	t	c	g	a	g	t	a	g	c	g	a	c	c	-	-	c
KP771912	c	a	c	t	t	g	g	c	c	g	a	a	c	g	t	g	a	t	c	g	a	g	t	a	g	c	g	a	c	c	-	-	c
KP771913	c	a	c	t	t	g	g	c	c	g	a	a	c	g	t	g	a	t	c	g	a	g	t	a	g	c	g	a	c	c	-	-	c
KP771917	c	a	c	t	t	g	g	c	c	g	a	a	c	g	t	g	a	t	c	g	a	g	t	a	g	c	a	a	c	c	-	-	c
KP771918	c	a	a	t	t	g	g	c	c	g	a	a	c	g	t	g	a	t	c	g	a	g	t	a	g	c	g	a	c	c	-	-	c
KP771920	c	a	c	t	t	g	g	c	c	g	a	a	c	g	t	g	a	t	c	g	a	g	t	a	g	c	g	a	c	c	-	-	c
MH709361	c	a	c	t	t	g	g	c	c	g	a	a	c	g	t	g	a	t	c	g	a	g	t	a	g	c	g	a	c	c	-	-	c
KU926318	c	a	c	t	t	g	g	c	t	g	a	a	c	a	t	g	a	t	c	g	a	g	t	a	g	g	g	a	t	c	-	-	c
KU926319	c	a	c	t	t	g	g	c	t	g	a	a	c	a	t	g	a	t	c	g	a	g	t	a	g	g	g	a	t	c	-	-	c
KU926320	c	a	c	t	t	g	g	c	t	g	a	a	c	a	t	g	a	t	c	g	a	g	t	a	g	g	g	a	t	c	-	-	c
KU926321	c	a	c	t	t	g	g	c	t	g	a	a	c	a	t	g	a	t	c	g	a	g	t	a	g	g	g	a	t	c	-	-	c
KU926322	c	a	c	t	c	g	g	c	t	g	a	a	c	a	t	g	a	t	c	g	a	g	t	a	g	g	g	a	t	c	-	-	c
KC847290	c	a	c	t	t	g	g	c	t	g	a	a	c	a	t	g	a	t	c	g	a	g	t	a	g	g	g	a	t	c	-	-	c
KJ767491	c	a	c	t	t	g	g	c	t	g	a	a	c	a	t	g	a	t	c	g	a	g	t	a	g	g	g	a	t	c	-	-	c
KJ767492	c	a	c	t	t	g	g	c	t	g	a	a	c	a	t	g	a	t	c	g	a	g	t	a	g	g	g	a	t	c	-	-	c
KJ808816	c	a	c	t	t	g	g	c	t	g	a	a	c	a	t	g	a	t	c	g	a	g	t	a	g	g	g	a	t	c	-	-	c
KP771925	c	a	c	t	t	g	g	c	t	g	a	a	c	a	t	g	a	t	c	g	a	g	t	a	g	g	g	a	t	c	-	-	c
KU926315	c	a	c	t	t	g	g	c	t	g	a	a	c	a	t	g	a	t	c	g	a	g	t	a	g	g	g	a	t	c	-	-	c
KU926316	c	a	c	t	t	g	g	c	t	g	a	a	c	a	t	g	a	t	c	g	a	g	t	a	g	g	g	a	t	c	-	-	c
KU926317	c	a	c	t	t	g	g	c	t	g	a	a	c	a	t	g	a	t	c	g	a	g	t	a	g	g	g	a	t	c	-	-	c
KY037796	c	a	c	t	t	g	g	c	t	g	a	a	c	a	t	g	a	t	c	g	a	g	t	a	g	g	g	a	t	c	-	-	c
MF004348	c	a	c	t	t	g	g	c	t	g	a	a	c	a	t	g	a	t	c	g	a	g	t	a	g	g	g	a	t	c	-	-	c
MH709309	c	a	c	t	t	g	g	c	t	g	a	a	c	a	t	g	a	t	c	g	a	g	t	a	g	g	g	a	t	c	-	-	c
MH709310	c	a	c	t	t	g	g	c	t	g	a	a	c	a	t	g	a	t	c	g	a	g	t	a	g	g	g	a	t	c	-	-	c
MH709311	c	a	c	t	t	g	g	c	t	g	a	a	c	a	t	g	a	t	c	g	a	t	t	a	g	g	g	a	t	c	-	-	c
MH709313	c	a	c	t	t	g	g	c	t	g	a	a	c	a	t	g	a	t	c	g	a	g	t	a	g	g	g	a	t	c	-	-	c
MH709314	c	a	c	t	t	g	g	c	t	g	a	a	c	a	t	g	a	t	c	g	a	g	t	a	g	g	g	a	t	c	-	-	c
MH709315	c	a	c	t	t	g	g	c	t	g	a	a	c	a	t	g	a	t	c	g	a	g	t	a	g	g	g	a	t	c	-	-	c
MH709319	c	a	c	t	t	g	g	c	t	g	a	a	c	a	t	g	a	t	c	g	a	g	t	a	g	g	g	a	t	c	-	-	c
MH709320	c	a	c	t	t	g	g	c	t	g	a	a	c	a	t	g	a	t	c	g	a	g	t	a	g	g	g	a	t	c	-	-	c
MH709329	c	a	c	t	t	g	g	c	t	g	a	a	c	a	t	g	a	t	c	g	a	g	t	a	g	g	g	a	t	c	-	-	c
MH709331	c	a	c	t	t	g	g	c	t	g	a	a	c	a	t	g	a	t	c	g	a	g	t	a	g	g	g	a	t	c	-	-	c
MH709333	c	a	c	t	t	g	g	c	t	g	a	a	c	a	t	g	a	t	c	g	a	g	t	a	g	g	g	a	t	c	-	-	c
MH709337	c	a	c	t	t	g	g	c	t	g	a	a	c	a	t	g	a	t	c	g	a	g	t	a	g	g	g	a	t	c	-	-	c
MH709339	c	a	c	t	t	g	g	c	t	g	a	a	c	a	t	g	a	t	c	g	a	g	g	a	g	g	g	a	t	c	-	-	c
MH709344	c	a	c	t	t	g	g	c	t	g	a	a	c	a	t	g	a	t	c	g	a	g	t	a	g	g	g	a	t	c	-	-	c
MH709348	a	a	c	t	t	g	g	c	t	g	a	a	c	a	t	g	a	t	c	g	a	g	t	a	g	g	g	a	t	c	-	-	c
MH709355	c	a	c	t	t	g	g	c	t	g	a	a	c	a	t	g	a	t	c	g	a	g	t	a	g	g	g	a	t	c	a	a	c
MH709359	c	a	c	t	t	g	g	c	t	g	a	a	c	a	t	g	a	t	c	g	a	g	t	a	g	g	g	a	t	c	-	-	c
MH709363	c	a	c	t	t	g	g	c	t	g	a	a	c	a	t	g	a	t	c	g	a	g	t	a	g	g	g	a	t	c	-	-	c
MH709366	c	a	c	t	t	g	g	c	t	g	a	a	c	a	t	g	a	t	c	g	a	g	t	a	g	g	g	a	t	c	-	-	c
MH709376	c	a	c	t	t	g	g	c	t	g	a	a	c	a	t	g	a	t	c	g	a	g	t	a	g	g	g	a	t	c	-	-	c
MH709377	c	a	c	t	t	g	g	c	t	g	a	a	c	a	t	g	a	t	c	g	a	g	t	a	g	g	g	a	t	c	-	-	c
**MW316406**	c	a	c	t	t	g	g	c	t	g	a	a	c	a	t	g	a	t	c	g	a	g	t	a	g	g	g	a	t	c	-	-	c
AJ871403	c	a	c	t	t	g	g	c	t	g	a	a	c	g	t	g	a	t	c	g	a	g	t	a	g	g	g	a	c	c	-	-	c
DQ479955	c	a	c	t	t	g	g	c	c	g	a	a	c	g	t	g	a	t	c	g	a	g	t	a	g	g	g	a	c	c	-	-	c
DQ479956	c	a	c	t	t	g	g	c	t	g	a	a	c	g	t	g	a	t	c	g	a	g	t	a	g	g	g	a	c	c	-	-	c
DQ479957	c	a	c	t	t	g	g	c	t	g	a	a	c	g	t	g	a	t	c	g	a	g	t	a	g	g	g	a	c	c	-	-	c
JN704699	c	a	c	t	t	g	g	c	t	g	a	a	c	g	t	g	a	t	t	g	a	g	t	a	g	g	g	a	c	c	-	-	c
JN704700	c	a	c	t	t	g	g	c	c	g	a	a	c	g	t	g	a	t	c	g	a	g	t	a	g	g	g	a	c	c	-	-	c
JN704701	c	a	c	t	t	g	g	c	t	g	a	a	c	g	t	g	a	t	t	g	a	g	t	a	a	g	g	a	c	c	-	-	c
JN704702	c	a	c	t	t	g	g	c	t	g	a	a	c	g	t	g	a	t	c	g	a	g	t	a	g	g	g	a	c	c	-	-	c
JN704703	c	a	c	t	t	g	g	c	t	g	a	a	c	g	t	g	a	t	c	g	a	g	t	a	g	g	g	a	c	c	-	-	c
KP771891	c	a	c	t	t	g	g	c	t	g	a	a	c	g	t	g	a	t	c	g	a	g	t	a	g	g	g	a	c	c	-	-	c
KP771893	c	a	c	t	t	g	g	c	t	g	a	a	c	g	t	g	a	t	c	g	a	g	t	a	g	g	g	a	c	c	-	-	c
KP771895	c	a	c	c	t	g	g	c	t	g	g	a	c	g	t	g	a	t	c	g	a	g	t	a	g	g	g	a	c	c	-	-	c
KP771896	c	a	c	t	t	g	g	c	t	g	a	a	c	g	t	g	a	t	c	g	a	g	t	a	g	g	g	a	c	c	-	-	c
KP771897	c	a	c	t	t	g	g	c	t	g	a	a	c	g	t	g	a	t	c	g	a	g	t	a	g	g	g	a	c	c	-	-	c
KP771899	c	a	c	t	t	g	g	c	t	g	a	a	c	g	t	g	a	t	c	g	a	g	t	a	g	g	g	a	c	c	-	-	c
KP771903	c	a	c	t	t	g	g	c	t	g	a	a	c	g	t	g	a	t	t	g	a	g	t	a	g	g	g	a	c	c	-	-	c
KP771907	c	a	c	t	t	g	g	c	t	g	a	a	c	g	t	g	a	t	c	g	a	g	t	a	g	g	g	a	c	c	-	-	c
KP771910	c	a	c	t	t	g	g	c	t	g	a	a	c	g	t	g	a	t	t	g	a	g	t	a	a	g	g	a	c	c	-	-	c
KP771915	c	a	c	t	t	g	g	c	t	g	a	a	c	g	t	g	a	t	c	g	a	g	t	a	g	g	g	a	c	c	-	-	c
KP771916	c	a	c	t	t	g	g	c	t	g	a	a	c	g	t	g	a	t	c	g	a	g	t	a	g	g	g	a	c	c	-	-	c
KP771921	c	a	c	t	t	g	g	c	t	g	a	a	c	g	t	g	a	t	t	g	a	g	t	a	g	g	g	a	c	c	-	-	c
KP771923	c	a	c	t	t	g	g	c	t	g	a	a	c	g	t	g	a	t	t	g	a	g	t	a	a	g	g	a	c	c	-	-	c
KP771889	c	a	c	t	t	g	g	c	t	g	a	a	c	g	t	g	a	t	c	g	a	g	t	a	g	g	g	a	c	c	-	-	c
KU926311	c	a	c	t	t	g	g	c	t	g	a	a	c	g	t	g	a	t	c	g	a	g	t	a	g	g	g	a	c	c	-	-	c
JQ911599	c	a	c	t	t	g	g	c	t	g	a	a	c	g	t	g	a	t	c	g	c	g	t	a	g	g	g	a	c	c	-	-	c
MH709317	c	a	c	t	t	g	g	c	t	g	a	a	c	g	t	g	a	t	c	g	a	g	t	a	g	g	g	a	c	c	-	-	c
MH709346	c	a	c	t	t	g	g	c	t	g	a	a	c	g	t	g	a	t	c	g	a	g	t	a	g	g	g	a	c	c	-	-	c
MH709375	c	a	c	t	t	g	g	c	t	g	a	a	c	g	t	g	a	t	c	g	a	g	t	a	g	g	g	a	c	c	-	-	c
DQ452050	c	a	c	t	t	g	g	c	t	g	a	a	c	g	t	g	a	t	c	g	a	g	t	a	g	c	g	a	c	c	-	-	c
DQ479960	c	a	c	t	t	g	g	c	c	g	a	a	c	g	c	g	a	c	c	g	a	g	t	a	g	c	g	a	c	c	-	-	c
KP771898	c	a	c	t	t	g	g	c	c	g	a	a	c	g	t	g	a	t	c	g	a	g	t	a	g	c	g	a	c	c	-	-	c
MH709316	c	a	c	t	t	g	g	c	c	g	a	a	c	g	t	g	a	t	c	g	a	g	t	a	g	c	g	a	c	c	-	-	c
MH709318	c	a	c	t	t	g	g	c	c	g	a	a	c	g	t	g	a	t	c	g	a	g	t	a	g	c	g	a	c	c	-	-	c
MH709322	c	a	c	t	t	g	g	c	c	g	a	a	c	g	t	g	a	t	c	g	a	g	t	a	g	c	g	a	c	c	-	-	c
MH709323	c	a	c	t	t	g	g	c	c	g	a	a	c	g	t	g	a	t	c	g	a	g	t	a	g	c	g	a	c	c	-	-	c
MH709326	c	a	c	t	t	a	g	c	c	g	a	a	c	g	t	g	a	t	c	g	a	g	t	a	g	c	g	a	c	c	-	-	c
MH709341	c	a	c	t	t	g	g	c	c	g	a	a	c	g	t	g	a	t	c	g	a	g	t	a	g	c	g	a	c	c	-	-	c
MH709357	c	a	c	t	t	g	g	c	c	g	a	a	c	g	t	g	a	t	c	g	a	g	t	a	g	c	g	a	c	c	-	-	c
MH709364	c	a	c	t	t	g	g	c	c	g	a	a	c	g	t	g	a	t	c	g	a	g	t	a	g	c	g	a	c	c	-	-	c
MH709370	c	a	c	t	t	g	g	c	c	g	a	a	c	g	t	g	a	t	c	g	a	g	t	a	g	c	g	a	c	c	-	-	c
MH709371	c	a	c	t	t	g	g	c	c	g	a	a	c	g	t	g	a	t	c	g	a	g	t	a	g	c	g	a	c	c	-	-	c
MH709372	c	a	c	t	t	g	g	c	c	a	a	a	c	g	t	g	a	t	c	g	a	g	t	a	g	c	g	a	c	c	-	-	c
DQ457052	c	a	c	t	t	g	a	c	t	g	a	a	c	g	t	a	a	t	c	g	a	g	t	a	g	g	g	a	c	c	-	-	c
JN704704	c	a	c	t	t	g	g	c	c	g	a	a	c	g	t	g	c	t	c	g	a	g	t	a	g	g	g	a	c	c	-	-	c
JN704705	c	a	c	t	t	g	g	c	t	g	a	a	c	g	t	g	a	t	c	g	a	g	t	a	g	g	g	a	c	c	-	-	c
JN704706	c	a	c	t	t	g	g	c	c	g	a	a	c	g	t	g	a	t	c	g	a	g	t	a	g	g	g	a	c	c	-	-	c
JN704707	c	a	c	t	t	g	g	c	t	g	a	a	c	g	t	g	a	t	c	g	a	g	t	a	g	g	g	a	c	c	-	-	c
JN704708	c	a	c	t	t	g	a	c	t	g	a	a	c	g	t	g	a	t	c	g	a	g	t	a	g	g	g	a	c	c	-	-	c
KP771904	c	a	c	t	t	g	a	c	t	g	a	a	c	g	t	g	a	t	c	g	a	g	t	a	g	g	g	a	c	c	-	-	c
KP771905	c	a	c	t	t	g	a	c	t	g	a	a	c	g	t	g	a	t	c	g	a	g	t	a	g	g	g	a	c	c	-	-	c
KP771906	c	g	c	t	t	g	a	c	t	g	a	a	c	g	t	g	a	t	c	g	a	g	t	a	g	g	g	a	c	c	-	-	c
KP771919	c	a	c	t	t	g	a	c	t	g	a	a	c	g	t	g	a	t	c	g	a	g	t	a	g	g	g	a	c	c	-	-	c
KP771922	c	a	c	t	t	g	a	c	t	g	a	a	c	g	t	g	a	t	c	g	a	g	t	a	g	g	g	a	c	c	-	-	c
KP771924	c	a	c	t	t	g	a	c	t	g	a	a	c	g	t	g	a	t	c	g	a	g	t	a	g	g	g	a	c	c	-	-	c
KM355696	c	a	c	t	t	g	a	c	t	g	a	a	c	g	t	g	a	t	c	g	a	g	t	a	g	g	g	a	c	c	-	-	c
KM355697	c	a	c	t	t	g	a	c	t	g	a	a	c	g	t	g	a	t	c	g	a	g	t	a	g	g	g	a	c	c	-	-	c
KM355698	c	a	c	t	t	g	a	c	t	g	a	a	c	g	t	g	a	t	c	g	a	g	t	a	g	g	g	a	c	c	-	-	c
KM355699	c	a	c	t	t	g	a	c	t	g	a	a	c	g	t	g	a	t	c	g	a	g	t	a	g	g	g	a	c	c	-	-	c
KM355701	c	a	c	t	t	g	a	c	t	g	a	a	c	g	t	g	a	t	c	g	a	g	t	a	g	g	g	a	c	c	-	-	c
KM355702	c	a	c	t	t	g	a	c	t	g	a	a	c	g	t	g	a	t	c	g	a	g	t	a	g	g	g	a	c	c	-	-	c
KM355703	c	a	c	t	t	g	a	c	t	g	a	a	c	g	t	g	a	t	c	g	a	g	t	a	g	g	g	a	c	c	-	-	c
KM355704	c	a	c	t	t	g	a	c	t	g	a	a	c	g	t	g	a	t	c	g	a	g	t	a	g	g	g	a	c	c	-	-	c
KM355705	c	a	c	t	t	g	a	c	t	g	a	a	c	g	t	g	a	t	c	g	a	g	t	a	g	g	g	a	c	c	-	-	c
KM355706	c	a	c	t	t	g	a	c	t	g	a	a	c	g	t	g	a	t	c	g	a	g	t	a	g	g	g	a	c	c	-	-	c
KM355707	c	a	c	t	t	g	a	c	t	g	a	a	c	g	t	g	a	t	c	g	a	g	t	a	g	g	g	a	c	c	-	-	c
KM355708	c	a	c	t	t	g	a	c	t	g	a	a	c	g	t	g	a	t	c	g	a	g	t	a	g	g	g	a	c	c	-	-	c
KM355709	c	a	c	t	t	g	a	c	t	g	a	a	c	g	t	g	a	t	c	g	a	g	t	a	g	g	g	a	c	c	-	-	c
KM355711	c	a	c	t	t	g	a	c	t	g	a	a	c	g	t	g	a	t	c	g	a	g	t	a	g	g	g	a	c	c	-	-	c
KM355712	c	a	c	t	t	g	a	c	t	g	a	a	c	g	t	g	a	t	c	g	a	g	t	a	g	g	g	a	c	c	-	-	c
KY037797	[illegible]	[illegible]	[illegible]	[illegible]	[illegible]	[illegible]	[illegible]	[illegible]	[illegible]	[illegible]	[illegible]	[illegible]	[illegible]	[illegible]	[illegible]	[illegible]	[illegible]	[illegible]	[illegible]	[illegible]	[illegible]	[illegible]	[illegible]	[illegible]	[illegible]	[illegible]	[illegible]	[illegible]	[illegible]	[illegible]	[illegible]	[illegible]	[illegible]

Supplementary Table 3: 2880 SN

GB-ID	Clade markers															SD14					4						4	4																																															
	Position in Dumas	39227	39263	39304	39315	39346	39357	39394	39398	39516	39530	39537	39564	39866	39962	40031	40096	40130	40134	40151	40196	40370	40388	40484	40499	40534	40554	40556	40594	40619	40645	40660	40746	40752	40793	40818	40826	40982	41057	41133	41207	41212	41213	41333	41372	41443	41544	41558	41588	41618	41715	41734	41737	41762	41764	41777	41799	41873	41883	41885	41966	41969	41993	42033	42036	42053	42069	42110	42118	42176	42242	42372_1	42372_2	42380
MH499467	5	t	g	g	g	a	a	a	t	g	g	a	c	t	c	t	t	c	t	t	c	a	g	a	c	t	g	g	t	g	g	t	t	a	g	g	a	t	t	a	g	c	a	c	t	t	g	a	c	t	-	a	a	c	g	t	g	a	t	c	g	a	g	t	a	g	g	g	a	c	c	-	-	a
MH499469	5	t	g	g	g	a	a	a	t	t	g	c	c	t	c	t	t	c	t	t	c	a	g	a	c	t	g	g	t	g	g	t	t	a	g	g	a	t	t	a	g	c	a	c	t	t	g	a	c	t	g	a	a	c	g	t	g	a	t	c	g	a	g	t	a	g	g	g	a	c	c	-	-	c
MH709321	5	t	g	g	g	a	a	a	t	t	g	c	c	t	c	t	t	c	t	t	c	a	g	a	c	t	g	g	t	g	g	t	t	a	g	g	a	t	t	a	g	c	a	c	t	t	g	a	c	t	g	a	a	c	g	t	g	a	t	c	g	a	g	t	a	g	g	g	a	c	c	-	-	c
MH709324	5	t	g	g	g	a	a	a	t	t	g	c	c	t	c	t	t	c	t	t	c	a	g	a	c	t	g	g	t	g	g	t	t	a	g	g	a	t	t	a	g	c	a	c	t	t	g	g	c	t	g	a	a	c	g	t	g	a	t	c	g	a	g	t	a	g	g	g	a	c	c	-	-	c
MH709325	5	t	g	g	g	a	a	a	t	t	g	c	c	t	c	t	t	c	t	t	c	a	g	a	c	t	g	g	t	g	g	t	t	a	g	g	a	t	t	a	g	c	a	c	t	t	g	a	c	t	g	a	a	c	g	t	g	a	t	c	g	a	g	t	a	g	g	g	a	c	c	-	-	c
MH709327	5	t	g	g	g	a	a	a	t	t	g	c	c	t	c	t	t	c	t	t	c	a	g	a	c	t	g	g	t	g	g	t	t	a	g	g	a	t	t	a	g	c	a	c	t	t	g	a	c	t	g	a	a	c	g	t	g	a	t	c	g	a	g	t	a	g	g	g	a	c	c	-	-	c
MH709328	5	t	g	g	g	a	a	a	t	t	g	c	c	t	c	t	t	c	t	t	c	a	g	a	c	t	g	g	t	g	g	t	t	a	g	g	a	t	t	a	g	c	a	c	t	t	g	a	c	t	g	a	a	c	g	t	g	a	t	c	g	a	g	t	a	g	g	g	a	c	c	-	-	c
MH709330	5	t	g	g	g	a	a	a	t	t	g	c	c	t	c	t	t	c	t	t	c	a	g	a	c	t	g	g	t	g	g	t	t	a	g	g	a	t	t	a	g	c	a	c	t	t	g	a	c	t	g	a	a	c	g	t	g	a	t	c	g	a	g	t	a	g	g	g	a	c	c	-	-	c
MH709332	5	t	g	g	g	a	a	a	t	t	g	c	c	t	c	t	t	c	t	t	c	a	g	a	c	t	g	g	t	g	g	t	t	a	g	g	a	t	t	a	g	c	a	c	t	t	g	a	c	t	g	a	a	c	g	t	g	a	t	c	g	a	g	t	a	g	g	g	a	c	c	-	-	c
MH709334	5	t	g	g	g	a	a	a	t	t	g	c	c	t	c	t	t	c	t	t	c	a	g	a	c	t	g	g	t	g	g	t	t	a	g	g	a	t	t	a	g	c	a	c	t	t	g	a	c	t	g	a	a	c	g	t	g	a	t	c	g	a	g	t	a	g	g	g	a	c	c	-	-	c
MH709335	5	t	g	g	g	a	a	a	t	t	g	c	c	t	c	t	t	c	t	t	c	a	g	a	c	t	g	g	t	g	g	t	t	a	g	g	a	t	t	a	g	c	a	c	t	t	g	a	c	t	g	a	a	c	g	t	g	a	t	c	g	a	g	t	a	g	g	g	a	c	c	-	-	c
MH709336	5	t	g	g	g	a	a	a	t	t	g	c	c	t	c	t	t	c	t	t	c	a	g	a	c	t	g	g	t	g	g	t	t	a	g	g	a	t	t	a	g	c	a	c	t	t	g	a	c	t	g	a	a	c	g	t	g	a	t	c	g	a	g	t	a	g	g	g	a	c	c	-	-	c
MH709338	5	t	g	g	g	a	a	a	t	t	g	c	c	t	c	t	t	c	t	t	c	a	g	a	c	t	g	g	t	g	g	t	t	a	g	g	a	t	t	a	g	c	a	c	t	t	g	a	c	t	g	a	a	c	g	t	g	a	t	c	g	a	g	t	a	g	g	g	a	c	c	-	-	c
MH709340	5	t	g	g	g	a	a	a	t	t	g	c	c	t	c	t	t	c	t	t	c	a	g	a	c	t	g	g	t	g	g	t	t	a	g	g	a	t	t	a	g	c	a	c	t	t	g	a	c	t	g	a	a	c	g	t	g	a	t	c	g	a	g	t	a	g	g	g	a	c	c	-	-	c
MH709342	5	t	g	g	g	a	a	a	t	t	g	c	c	t	c	t	t	c	t	t	c	a	g	a	c	t	g	g	t	g	g	t	t	a	g	g	a	t	t	a	g	c	a	c	t	t	g	a	c	t	g	a	a	c	g	t	g	a	t	c	g	a	g	t	a	g	g	g	a	c	c	-	-	c
MH709343	5	t	g	g	g	a	a	a	t	t	g	c	c	t	c	t	t	c	t	t	c	a	g	a	c	t	g	g	t	g	g	t	t	a	g	g	a	t	t	a	g	c	a	c	t	t	g	a	c	t	g	a	a	c	g	t	g	a	t	c	g	a	g	t	a	g	g	g	a	c	c	-	-	c
MH709345	5	t	g	g	g	a	a	a	t	t	g	c	c	t	c	t	t	c	t	t	c	a	g	a	c	t	g	g	t	g	g	t	t	a	g	g	a	t	t	a	g	c	a	c	t	t	g	a	c	t	g	a	a	c	g	t	g	a	t	c	g	a	g	t	a	g	g	g	a	c	c	-	-	c
MH709347	5	t	g	g	g	a	a	a	t	t	g	c	c	t	c	t	t	c	t	t	c	a	g	a	c	t	g	g	t	g	g	t	t	a	g	g	a	t	t	a	g	c	a	c	t	t	g	a	c	t	g	a	a	c	g	t	g	a	t	c	g	a	g	t	a	g	g	g	a	c	c	-	-	c
MH709349	5	t	g	g	g	a	a	a	t	t	g	c	c	t	c	t	t	c	t	t	c	a	g	a	c	t	g	g	t	g	g	t	t	a	g	g	a	t	t	a	g	c	a	c	t	t	g	a	c	t	g	a	a	c	g	t	g	a	t	c	g	a	g	t	a	g	g	g	a	c	c	-	-	c
MH709350	5	t	g	g	g	a	a	a	t	t	g	c	c	t	c	t	t	c	t	t	c	a	g	a	c	t	g	g	t	g	g	t	t	a	g	g	a	t	t	a	g	c	a	c	t	t	g	a	c	t	g	a	a	c	g	t	g	a	t	c	g	a	g	t	a	g	g	g	a	c	c	-	-	c
MH709351	5	t	g	g	g	a	a	a	t	t	g	c	c	t	c	t	t	c	t	t	c	a	g	a	c	t	g	g	t	g	g	t	t	a	g	g	a	t	t	a	g	c	a	c	t	t	g	a	c	t	g	a	a	t	g	t	g	a	t	c	g	a	g	t	a	g	g	g	a	c	c	-	-	c
MH709353	5	t	g	g	g	a	a	a	t	t	g	c	c	t	c	t	t	c	t	t	c	a	g	a	c	t	g	g	t	g	g	t	t	a	g	g	a	t	t	a	g	c	a	c	t	t	g	a	c	t	g	a	a	c	g	t	g	a	t	c	g	a	g	t	a	g	g	g	a	c	c	-	-	c
MH709358	5	t	g	g	g	a	a	a	t	t	g	c	c	t	c	t	t	c	t	t	c	a	g	a	c	t	g	g	t	g	g	t	t	a	g	g	a	t	t	a	g	c	a	c	t	t	g	a	c	t	g	a	a	c	g	t	g	a	t	c	g	a	g	t	a	g	g	g	a	c	c	-	-	c
MH709360	5	t	g	g	g	a	a	a	t	t	g	c	c	t	c	t	t	c	t	t	c	a	g	a	c	t	g	g	t	g	g	t	t	a	g	g	a	t	t	a	g	c	a	c	t	t	g	a	c	t	g	a	a	c	g	t	g	a	t	c	g	a	g	t	a	g	g	g	a	c	c	-	-	c
MH709362	5	t	g	g	g	a	a	a	t	t	g	c	c	t	c	t	t	c	t	t	c	a	g	a	c	t	g	g	t	g	g	t	t	a	g	g	a	t	t	a	g	c	a	c	t	t	g	a	c	t	g	a	a	c	g	t	g	a	t	c	g	a	g	t	a	g	g	g	a	c	c	-	-	c
MH709365	5	t	g	g	g	a	a	a	t	t	g	c	c	t	c	t	t	c	t	t	c	a	g	a	c	t	g	g	t	g	g	t	t	a	g	g	a	t	t	a	g	c	a	c	t	t	g	a	c	t	g	a	a	c	g	t	g	a	t	c	g	a	g	t	a	g	g	g	a	c	c	-	-	c
MH709373	5	t	g	g	g	a	a	a	t	t	g	c	c	t	c	t	t	c	t	t	c	a	g	a	c	t	g	g	t	g	g	t	t	a	g	g	a	t	t	a	g	c	a	c	t	t	g	a	c	t	g	a	a	c	g	t	g	a	t	c	g	a	g	t	a	g	g	g	a	c	c	-	-	c
MH709374	5	t	g	g	g	a	a	a	t	t	g	c	c	t	c	t	t	c	t	t	c	a	g	a	c	t	g	g	t	g	g	t	t	a	g	g	a	t	t	a	g	c	a	c	t	t	g	a	c	t	g	a	a	c	g	t	g	a	t	c	g	a	g	t	a	g	g	g	a	c	c	-	-	c
KC112914	6	t	g	g	g	a	a	a	t	t	g	c	c	t	c	t	t	c	t	t	c	a	g	a	c	t	g	g	t	a	g	t	t	a	g	g	a	t	t	a	g	c	a	c	t	t	g	g	c	t	g	a	a	c	g	t	g	a	t	c	g	a	g	t	c	g	g	g	a	c	c	-	-	c
KP771914	6	t	g	g	g	a	a	a	t	t	g	c	c	t	c	t	t	c	t	t	c	a	g	a	c	t	g	g	t	a	g	t	t	a	g	g	a	t	t	a	g	c	a	c	t	t	g	g	c	t	g	a	a	c	g	t	g	a	t	c	g	a	g	t	c	g	g	g	a	c	c	-	-	c
KP702725	6	t	g	g	g	a	a	a	t	t	g	c	c	t	c	t	t	c	t	t	c	a	g	a	c	a	g	g	t	a	g	t	t	c	g	g	a	t	t	a	g	c	a	c	t	t	g	g	c	t	g	a	a	c	g	t	g	a	t	c	g	a	g	t	c	g	g	g	a	c	c	-	-	c
KP771890	9	t	g	g	g	a	a	g	t	t	a	c	c	t	c	t	t	c	t	t	c	a	g	a	c	t	g	g	t	a	g	t	t	a	g	g	a	t	t	a	g	c	a	c	t	t	g	g	c	c	g	a	a	c	g	t	g	a	t	c	g	a	g	t	a	g	g	g	a	c	c	-	-	c
JN704710	9	t	g	g	g	a	a	g	t	t	a	c	c	t	c	t	t	c	t	t	c	a	a	a	c	t	g	g	t	g	g	t	t	a	g	g	a	t	t	a	g	c	a	c	t	t	g	g	c	c	g	a	a	c	g	t	g	a	t	c	g	a	g	t	a	g	g	g	a	c	c	-	-	c
KY037798	9	t	g	g	g	a	a	g	t	t	a	c	c	t	c	t	t	c	t	t	c	a	g	a	c	t	g	g	t	g	g	t	t	a	g	g	a	t	t	a	g	c	a	c	t	t	g	g	c	c	g	a	g	c	g	t	g	a	t	c	g	a	g	t	a	g	g	g	a	c	c	-	-	c
MH709312	9	t	g	g	g	a	a	g	t	t	a	c	c	t	c	t	t	c	t	t	c	a	g	a	c	t	g	g	t	g	g	t	t	a	g	g	a	t	t	a	g	c	a	c	t	t	g	g	c	c	g	a	g	c	g	t	g	a	t	c	g	a	g	t	a	g	g	g	a	c	c	-	-	c
MH709352	9	t	g	g	g	a	a	g	t	t	a	c	c	t	c	t	t	c	t	t	c	a	g	a	c	t	g	g	t	g	g	t	t	a	g	g	a	t	t	a	g	c	a	c	t	t	g	g	c	c	g	a	g	c	g	t	g	a	t	c	g	a	g	t	a	g	g	g	a	c	c	-	-	c
AB097933	Vac	t	a	g	g	a	a	a	t	t	g	c	c	t	c	t	t	c	t	t	c	a	g	a	c	t	g	g	t	g	g	t	t	a	g	g	a	t	c	a	g	c	a	c	t	t	g	g	c	t	g	a	a	c	a	t	g	a	t	c	g	a	g	t	a	g	g	g	a	t	c	-	-	c
JN704698	Vac	t	a	g	g	a	a	a	t	t	g	c	c	t	c	t	t	c	t	t	c	a	g	a	c	t	g	g	t	g	g	t	t	a	g	g	a	t	c	a	g	c	a	c	t	t	g	g	c	t	g	a	a	c	a	t	g	a	t	c	g	a	g	t	a	g	g	g	a	t	c	-	-	c
JN704697	Vac	g	a	g	g	a	a	a	t	t	g	c	c	t	c	t	t	c	t	t	c	a	g	a	c	t	g	g	t	g	g	t	t	a	g	g	a	t	c	a	g	c	a	c	t	t	g	g	c	c	g	a	a	c	a	t	g	a	t	c	g	a	g	t	a	g	g	g	a	t	c	-	-	c
DQ008355	Vac	t	a	g	g	a	a	a	t	t	g	c	c	t	c	t	t	c	t	t	c	a	g	a	c	t	g	g	t	g	g	t	t	a	g	g	a	t	c	a	g	c	a	c	t	t	g	g	c	t	g	a	a	c	a	t	g	a	t	c	g	a	g	t	a	g	g	g	a	t	c	-	-	c
JF306641	Vac	t	a	g	g	a	a	a	t	t	g	c	c	t	c	t	t	c	t	t	c	a	g	a	c	t	g	g	t	g	g	t	t	a	g	g	a	t	c	a	g	c	a	c	t	t	g	g	c	t	g	a	a	c	a	t	g	a	t	c	g	a	g	t	a	g	g	g	a	t	c	-	-	c
AB097932	Vac	k	a	g	g	a	a	a	t	t	g	c	c	t	c	t	t	c	t	t	c	a	g	a	c	t	g	g	t	g	g	t	t	a	g	g	a	t	c	a	g	c	a	c	t	t	g	g	c	t	g	a	a	c	a	t	g	a	t	c	g	a	g	t	a	g	g	g	a	t	c	-	-	c
DQ008354	Vac	k	a	g	g	a	a	a	t	t	g	c	c	t	c	t	t	c	t	t	c	a	g	a	c	t	g	g	t	g	g	t	t	a	g	g	a	t	c	a	g	c	a	c	t	t	g	g	c	t	g	a	a	c	a	t	g	a	t	c	g	a	g	t	a	g	g	g	a	t	c	-	-	c
KU926312	Vac	t	a	g	g	a	a	a	t	t	g	c	c	t	c	t	t	c	t	t	c	a	g	a	c	t	g	g	t	g	g	t	t	a	g	g	a	t	c	a	g	c	a	c	t	t	g	g	c	t	g	a	a	c	a	t	g	a	t	c	g	a	g	t	a	g	g	g	a	t	c	-	-	c
KU926313	Vac	t	a	g	g	a	a	a	t	t	g	c	c	t	c	t	t	c	t	t	c	a	g	a	c	t	g	g	t	g	g	t	t	a	g	g	a	t	c	a	g	c	a	c	t	t	g	g	c	t	g	a	a	c	a	t	g	a	t	c	g	a	g	t	a	g	g	g	a	t	c	-	-	c
KU926314	Vac	t	a	g	g	a	a	a	t	t	g	c	c	t	c	t	t	c	t	t	c	a	g	a	c	t	g	g	t	g	g	t	t	a	g	g	a	t	c	a	g	c	a	c	t	t	g	g	c	t	g	a	a	c	a	t	g	a	t	c	g	a	g	t	a	g	g	g	a	t	c	-	-	c
KF558371	Vac	t	a	g	g	a	a	a	t	t	g	c	c	t	c	t	t	c	t	t	c	a	g	a	c	t	g	g	g	g	g	t	t	a	g	g	a	t	c	a	g	c	a	c	t	-	g	g	c	t	g	a	a	c	a	t	g	a	t	c	g	a	g	t	a	g	g	g	a	t	c	-	-	c
KF558372	Vac	t	a	g	g	a	a	a	t	t	g	c	c	t	c	t	t	c	t	t	c	a	g	a	c	t	g	g	t	g	g	t	t	a	g	g	a	t	c	a	g	c	a	c	t	t	g	g	c	t	g	a	a	c	a	t	g	a	t	c	g	a	g	t	a	g	g	g	a	t	c	-	-	c
KF558373	Vac	t	a	g	g	a	a	a	t	t	g	c	c	t	c	t	t	c	t	t	c	a	g	a	c	t	g	g	t	g	g	t	t	a	g	g	a	t	c	a	g	c	a	c	t	t	g	g	c	t	g	a	a	c	a	t	g	a	t	c	g	a	g	t	a	g	g	g	a	t	c	-	-	c
KF558374	Vac	t	a	g	g	a	a	a	t	t	g	c	c	t	c	t	t	c	t	t	c	a	g	a	c	t	g	g	t	g	g	t	t	a	g	g	a	t	c	a	g	c	a	c	t	t	g	g	c	t	g	a	a	c	a	t	g	a	t	c	g	a	g	t	a	g	g	g	a	t	c	-	-	c
KF558375	Vac	t	a	g	g	a	a	a	t	t	g	c	c	t	c	t	t	c	t	t	c	a	g	a	c	t	g	g	t	g	g	t	t	a	g	g	a	t	c	a	g	c	a	c	t	t	g	g	c	t	g	a	a	c	a	t	g	a	t	c	g	a	g	t	a	g	g	g	a	t	c	-	-	c
KF558376	Vac	t	a	g	g	a	a	a	t	t	g	c	c	t	c	t	t	c	t	t	c	a	g	a	c	t	g	g	t	g	g	t	t	a	g	g	a	t	c	a	g	c	a	c	t	t	g	g	c	t	g	a	a	c	a	t	g	a	t	c	g	a	g	t	a	g	g	g	a	t	c	-	-	c
KF558377	Vac	t	a	g	g	a	a	a	t	t	g	c	c	t	c	t	t	c	t	t	c	a	g	a	c	t	g	g	t	g	g	t	t	a	g	g	a	t	c	a	g	c	a	c	t	t	g	g	c	t	g	a	a	c	a	t	g	a	t	c	g	a	g	t	a	g	g	g	a	t	c	-	-	c
KF558378	Vac	t	a	g	g	a	a	a	t	t	g	c	c	t	c	t	c	c	t	t	c	a	g	a	c	t	g	g	t	g	g	t	t	a	g	g	a	t	c	a	g	c	a	c	t	t	g	g	c	t	g	a	a	c	a	t	g	a	t	c	g	a	g	t	a	g	g	g	a	t	c	-	-	c
KF558379	Vac	t	a	g	g	a	a	a	t	t	g	c	c	t	c	t	t	c	t	t	c	a	g	a	c	t	g	g	t	g	g	t	t	a	g	g	a	t	c	a	g	c	a	c	t	t	g	g	c	t	g	a	a	c	a	t	g	a	t	c	g	a	g	t	a	g	g	g	a	t	c	-	-	c
KF558380	Vac	g	a	g	g	a	a	a	t	t	g	c	c	t	c	t	t	c	t	t	c	a	g	a	c	t	g	g	t	g	g	t	t	a	g	g	a	t	c	a	g	c	a	c	t	t	g	g	c	t	g	a	a	c	a	t	g	a	t	c	g	a	g	t	a	g	g	g	a	t	c	-	-	c
KF558381	Vac	t	a	g	g	a	a	a	t	t	g	c	c	t	c	t	t	c	t	t	c	a	g	a	c	t	g	g	t	g	g	t	t	a	g	g	a	t	c	a	g	c	a	c	t	t	g	g	c	t	g	a	a	c	a	t	g	a	t	c	g	a	g	t	a	g	g	g	a	t	c	-	-	c
KF558382	Vac	t	a	g	g	a	a	a	t	t	g	c	c	t	c	t	t	c	t	t	c	a	g	a	c	t	g	g	t	g	g	t	t	a	g	g	a	t	c	a	g	c	a	c	t	t	g	g	c	t	g	a	a	c	a	t	g	a	t	c	g	a	g	t	a	g	g	g	a	t	c	-	-	c
KF558383	Vac	t	a	g	g	a	a	a	t	t	g	c	c	t	c	t	t	c	t	t	c	a	g	a	c	t	g	g	t	g	g	t	t	a	g	g	a	t	c	a	g	c	a	c	t	t	g	g	c	t	g	a	a	c	a	t	g	a	t	c	g	a	g	t	a	g	g	g	a	t	c	-	-	c
KF558384	Vac	t	a	g	g	a	a	a	t	t	g	c	c	t	c	t	t	c	t	t	c	a	g	a	c	t	g	g	t	g	g	t	t	a	g	g	a	t	c	a	g	c	a	c	t	t	g	g	c	t	g	a	a	c	a	t	g	a	t	c	g	a	g	t	a	g	g	g	a	t	c	-	-	c
KF558385	Vac	t	a	g	g	a	a	a	t	t	g	c	c	t	c	t	t	c	t	t	c	a	g	a	c	t	g	g	t	g	g	t	t	a	g	g	a	t	c	a	g	c	a	c	t	t	g	g	c	t	g	a	a	c	a	t	g	a	t	c	g	a	g	t	a	g	g	g	a	t	c	-	-	c
KF558386	Vac	t	a	g	g	a	a	a	t	t	g	c	c	t	c	t	t	c	t	t	c	a	g	a	c	t	g	g	t	g	g	t	t	a	g	g	a	t	c	a	g	c	a	c	t	t	g	g	c	t	g	a	a	c	a	t	g	a	t	c	g	a	g	t	a	g	g	g	a	t	c	-	-	c
KF558387	Vac	t	a	g	g	a	a	a	t	t	g	c	c	t	c	t	t	c	t	t	c	a	g	a	c	t	g	g	t	g	g	t	t	a	g	g	a	t	c	a	g	c	a	c	t	t	g	g	c	t	g	a	a	c	a	t	g	a	t	c	g	a	g	t	a	g	g	g	a	t	c	-	-	c
KF558388	Vac	t	a	g	g	a	a	a	t	t	g	c	c	t	c	t	t	c	t	t	c	a	g	a	c	t	g	g	t	g	g	t	t	a	g	g	a	t	c	a	g	c	a	c	t	t	g	g	c	t	g	a	a	c	a	t	g	a	t	c	g	a	g	t	a	g	g	g	a	t	c	-	-	c
KF558389	Vac	t	a	g	g	a	a	a	t	t	g	c	c	t	c	t	t	c	t	t	c	a	g	a	c	t	g	g	t	g	g	t	t	a	g	g	a	t	c	a	g	c	a	c	t	t	g	g	c	t	g	a	a	c	a	t	g	a	t	c	g	a	g	t	a	g	g	g	a	t	c	-	-	c
KF558390	Vac	t	a	g	g	a	a	a	t	t	g	c	c	t	c	t	t	c	t	t	c	a	g	a	c	t	g	g	t	g	g	t	t	a	g	g	a	t	c	a	g	c	a	c	t	t	g	g	c	t	g	a	a	c	a	t	g	a	t	c	g	a	g	t	a	g	g	g	a	t	c	-	-	c
KF558391	Vac	t	a	g	g	a	a	a	t	t	g	c	c	t	c	t	t	c	t	t	c	a	g	a	c	t	g	g	t	g	g	t	t	a	g	g	a	t	c	a	g	c	a	c	t	t	g	g	c	t	g	a	a	c	a	t	g	a	t	c	g	a	g	t	a	g	g	g	a	t	c	-	-	c
KF811485	Vac	t	a	g	g	a	a	a	t	t	g	c	c	t	c	t	t	c	t	t	c	a	g	a	c	t	g	g	t	g	g	t	t	a	g	g	a	t	c	a	g	c	a	c	t	t	g	g	c	t	g	a	a	c	a	t	g	a	t	c	g	a	g	t	a	g	g	g	a	t	c	-	-	c
KF853225	Vac	t	a	g	g	a	a	a	t	t	g	c	c	t	c	t	t	c	t	t	c	a	g	a	c	t	g	g	t	g	g	t	t	a	g	g	a	t	c	a	g	c	a	c	t	t	g	g	c	t	g	a	a	c	a	t	g	a	t	c	g	a	g	t	a	g	g	g	a	t	c	-	-	c
KF853226	Vac	t	a	g	g	a	a	a	t	t	g	c	c	t	c	t	c	c	t	t	c	a	g	a	c	t	g	g	t	g	g	t	t	a	g	g	a	t	c	a	g	c	a	c	t	t	g	g	c	t	g	a	a	c	a	t	g	a	t	c	g	a	g	t	a	g	g	g	a	t	c	-	-	c
KF853227	Vac	t	a	g	g	a	a	a	t	t	g	c	c	t	c	t	t	c	t	t	c	a	g	a	c	t	g	g	t	g	g	t	t	a	g	g	a	t	c	a	g	c	a	c	t	t	g	g	c	t	g	a	a	c	a	t	g	a	t	c	g	a	g	t	a	g	g	g	a	t	c	-	-	c
KF853228	Vac	t	a	g	g	a	a	a	t	t	g	c	c	t	c	t	t	c	t	t	c	a	g	a	c	t	g	g	t	g	g	t	t	a	g	g	a	t	c	a	g	c	a	c	t	t	g	g	c	t	g	a	a	c	a	t	g	a	t	c	g	a	g	t	a	g	g	g	a	t	c	-	-	c
KF853229	Vac	t	a	g	g	a	a	a	t	t	g	c	c	t	c	t	t	c	t	t	c	a	g	a	c	t	g	g	t	g	g	t	t	a	g	g	a	t	c	a	g	c	a	c	t	t	g	g	c	t	g	a	a	c	a	t	g	a	t	c	g	a	g	t	a	g	g	g	a	t	c	-	-	c
KF853230	Vac	t	a	g	g	a	a	a	t	t	g	c	c	t	c	t	t	c	t	t	c	a	g	a	c	t	g	g	t	g	g	t	t	a	g	g	a	t	c	a	g	c	a	c	t	t	g	g	c	t	g	a	a	c	a	t	g	a	t	c	g	a	g	t	a	g	g	g	a	t	c	-	-	c
KF853231	Vac	t	a	g	g	a	a	a	t	t	g	c	c	t	c	t	t	c	t	t	c	a	g	a	c	t	g	g	t	g	g	t	t	a	g	g	a	t	c	a	g	c	a	c	t	t	g	g	c	t	g	a	a	c	a	t	g	a	t	c	g	a	g	t	a	g	g	g	a	t	c	-	-	c
KF853232	Vac	t	a	g	g	a	a	a	t	t	g	c	c	t	c	t	t	c	t	t	c	a	g	a	c	t	g	g	t	g	g	t	t	a	g	g	a	t	c	a	g	c	a	c	t	t	g	g	c	t	g	a	a	c	a	t	g	a	t	c	g	a	g	t	a	g	g	g	a	t	c	-	-	c
KF853233	Vac	t	a	g	g	a	a	a	t	t	g	c	c	t	c	t	t	c	t	t	c	a	g	a	c	t	g	g	t	g	g	t	t	a	g	g	a	t	c	a	g	c	a	c	t	t	g	g	c	t	g	a	a	c	a	t	g	a	t	c	g	a	g	t	a	g	g	g	a	t	c	-	-	c
KF853234	Vac	t	a	g	g	a	a	a	t	t	g	c	c	t	c	t	t	c	t	t	c	a	g	a	c	t	g	g	t	g	g	t	t	a	g	g	a	t	c	a	g	c	a	c	t	t	g	g	c	t	g	a	a	c	a	t	g	a	t	c	g	a	g	t	a	g	g	g	a	t	c	-	-	c
KF853235	Vac	t	a	g	g	a	a	a	t	t	g	c	c	t	c	t	t	c	t	t	c	a	g	a	c	t	g	g	t	g	g	t	t	a	g	g	a	t	c	a	g	c	a	c	t	t	g	g	c	t	g	a	a	c	a	t	g	a	t	c	g	a	g	t	a	g	g	g	a	t	c	-	-	c
MF898328	Vac	k	a	g	g	a	a	a	t	t	g	c	c	t	c	t	t	c	t	t	c	a	g	a	c	t	g	g	t	g	g	t	t	a	g	g	a	t	c	a	g	c	a	c	t	t	g	g	c	t	g	a	a	c	a	t	g	a	t	c	g	a	g	t	a	g	g	g	a	t	c	-	-	c
JN704709	VIII	g	a	g	g	a	a	a	t	t	g	c	c	t	c	t	t	c	t	t	c	a	g	a	c	t	g	g	t	g	g	t	t	a	g	g	a	t	c	a	g	c	a	c	t	t	g	g	c	t	g	a	a	c	g	t	g	a	t	c	g	a	g	t	c	g	g	g	a	c	a	-	-	c

Supplementary Table 3: 2880 SN

GB-ID	Clade markers																																								9																															
	Position in Dumas	42386	42391	42391_1	42392	42393	42394	42401	42405	42407	42410	42417	42418	42419	42421	42434	42436	42476	42544	42565	42587	42659	42735	42743	42798	42799	42812	42899	42941	42950	42974	43091	43109	43162	43195	43238	43253	43262	43316	43345	43401	43503	43563	43629	43695	43707	43878	43977	44001	44037	44111	44119	44154	44208	44232	44259	44266	44462	44507	44577	44721	44835	44892	44943	44994	44995	45246	45261	45267	45275	45358	45426	45445	45452
AY548170	1	t	g	-	-	a	a	a	a	c	t	c	t	g	g	c	c	t	c	c	t	c	t	g	g	g	t	c	c	g	g	g	g	c	a	a	c	t	t	a	g	a	c	a	a	g	g	g	t	c	c	c	a	t	a	a	t	t	t	t	c	c	c	c	g	t	g	t	c	c	c	a	t	a
AY548171	1	t	g	-	-	a	a	a	a	c	t	c	t	g	g	c	c	t	c	c	t	c	t	g	g	g	t	c	c	g	g	g	g	c	a	a	c	t	t	a	g	a	c	a	a	g	g	g	t	c	c	c	a	t	a	a	t	t	t	t	t	c	c	c	g	t	g	t	c	c	c	a	t	a
DQ479953	1	t	g	-	-	-	a	a	a	c	t	c	t	g	g	c	c	t	c	c	t	c	t	g	g	g	t	c	c	g	g	g	g	c	a	a	c	t	t	a	g	a	c	a	a	g	g	g	g	c	c	c	a	t	a	a	t	t	t	t	c	c	c	c	g	t	g	t	c	c	c	a	t	a
DQ479954	1	t	g	-	a	a	a	a	a	c	t	c	t	g	g	c	c	t	c	c	t	c	t	g	g	g	t	c	c	g	g	g	g	c	a	a	c	t	t	a	g	a	c	a	a	g	g	g	t	c	c	c	a	t	a	a	t	t	t	t	c	c	c	c	g	t	g	t	c	c	c	a	t	a
DQ479958	1	t	g	-	a	a	a	a	a	c	t	c	t	g	g	c	c	t	c	c	t	c	t	g	g	g	t	c	c	g	g	g	g	c	a	a	c	t	t	a	g	a	c	a	a	g	g	g	t	c	c	c	a	t	a	a	t	t	t	t	c	c	c	c	g	t	g	t	c	c	c	a	t	a
DQ479959	1	t	g	-	a	a	a	a	a	c	t	c	t	g	g	c	c	t	c	c	t	c	t	g	g	g	t	c	c	g	g	g	g	c	a	a	c	t	t	a	g	a	c	a	a	g	g	g	t	c	c	c	a	t	a	a	t	t	t	t	c	c	c	c	g	t	g	t	c	c	c	a	t	a
DQ479961	1	t	g	-	-	-	a	a	a	c	t	c	t	g	g	c	c	t	c	c	t	c	t	g	g	g	t	c	c	g	g	g	g	c	a	a	c	t	t	a	g	a	c	a	a	g	g	g	t	c	c	c	a	t	a	a	t	t	t	t	c	c	c	c	g	t	g	t	c	c	t	a	t	a
DQ479962	1	t	g	-	-	-	a	a	a	c	t	c	t	g	g	c	c	t	c	c	t	c	t	g	g	g	t	c	c	g	g	g	g	c	a	a	c	t	t	a	g	a	c	a	a	g	g	g	t	c	c	c	a	t	a	a	t	t	t	t	c	c	c	c	g	t	g	t	c	c	t	a	t	a
DQ479963	1	t	g	-	-	-	a	a	a	c	t	c	t	g	g	c	c	t	c	c	t	c	t	g	g	g	t	c	c	g	g	g	g	c	a	a	c	t	t	a	g	a	c	a	a	g	g	g	t	c	c	c	a	t	a	a	t	t	t	t	c	c	c	c	g	t	g	t	c	c	c	a	t	a
DQ674250	1	t	g	-	-	-	a	a	a	c	t	c	t	g	g	c	c	t	t	c	t	c	t	g	g	g	t	c	c	g	g	g	g	c	a	a	c	t	t	a	g	a	c	a	a	g	g	g	t	c	c	c	a	t	a	a	t	t	t	t	c	c	c	c	g	t	g	t	c	c	c	a	t	a
KX262864	1	t	g	-	a	a	a	a	a	c	t	c	t	g	g	c	c	t	c	c	t	c	t	g	g	g	t	c	c	g	g	g	g	c	a	a	c	t	t	a	g	a	c	a	a	g	g	g	t	c	c	c	a	t	a	a	t	t	t	t	c	c	c	c	g	t	g	t	c	c	c	a	t	a
KX262865	1	t	g	-	a	a	a	a	a	c	t	c	t	g	g	c	c	t	c	c	t	c	t	g	g	g	t	c	c	g	g	g	g	c	a	a	c	t	t	a	g	a	c	a	a	g	g	g	t	c	c	c	a	t	a	a	t	t	t	t	c	c	c	c	g	t	g	t	c	c	c	a	t	a
KX262866	1	t	g	-	a	a	a	a	a	c	t	c	t	g	g	c	c	t	c	c	t	c	t	g	g	g	t	c	c	g	g	g	g	c	a	a	c	t	t	a	g	a	c	a	a	g	g	g	t	c	c	c	a	t	a	a	t	t	t	t	c	c	c	c	g	t	g	t	c	c	c	a	t	a
EU154348	1	t	g	-	-	a	a	a	a	c	t	c	t	g	g	c	c	t	c	c	t	c	t	g	g	g	t	c	c	g	g	g	g	c	a	a	c	t	t	a	g	a	c	a	a	g	g	g	t	c	c	c	a	t	a	a	t	t	t	t	c	c	c	c	g	t	g	t	c	c	c	a	t	a
JN704690	1	t	g	-	a	a	a	a	a	c	t	c	t	g	g	c	c	t	c	c	t	c	t	g	g	g	g	c	c	g	g	g	g	c	a	a	c	t	t	a	g	a	c	a	a	g	g	g	t	c	c	c	a	t	a	a	t	t	t	t	c	c	c	c	g	t	g	t	c	c	c	a	t	a
JN704691	1	t	g	-	a	a	a	a	a	c	t	c	t	g	g	c	c	t	c	c	t	c	t	g	g	g	t	c	c	g	g	g	g	c	a	a	c	t	t	a	g	a	c	a	a	g	g	g	t	c	c	c	a	t	a	a	t	t	t	t	c	c	c	c	g	t	g	t	c	c	c	a	t	a
JN704692	1	t	g	-	a	a	a	a	a	c	t	c	t	g	g	c	c	t	c	c	t	c	t	g	g	g	t	c	c	g	g	g	g	c	a	a	c	t	t	a	g	a	c	a	a	g	g	g	t	c	c	c	a	t	a	a	t	t	t	t	c	c	c	c	g	t	g	t	c	c	c	a	t	a
JN704693	1	t	g	-	a	a	a	a	a	c	t	c	t	g	g	c	c	t	c	c	t	c	t	g	g	g	t	a	c	g	g	g	g	c	a	a	c	t	t	a	g	a	c	a	a	g	g	g	t	c	c	c	a	t	a	a	t	t	t	t	c	c	c	c	g	t	g	t	c	c	c	a	t	a
JN704694	1	t	g	-	a	a	a	a	a	c	t	c	t	g	g	c	c	t	c	c	t	c	t	g	g	g	t	c	c	g	g	g	g	c	a	a	c	t	t	a	g	a	c	a	a	g	g	g	t	c	c	c	a	t	a	a	t	t	t	t	c	c	c	c	g	t	g	t	c	c	c	a	t	a
JN704695	1	t	g	-	a	a	a	a	a	c	t	c	t	g	g	c	c	t	c	c	t	c	t	g	g	g	t	c	c	g	g	g	g	c	a	a	c	t	t	a	g	a	c	a	a	g	g	g	t	c	c	c	a	t	a	a	t	t	t	t	c	c	c	c	g	t	g	t	c	c	c	a	t	a
JN704696	1	t	g	-	a	a	a	a	a	c	t	c	t	g	g	c	c	t	c	c	t	c	t	g	g	g	t	c	c	g	g	g	g	c	a	a	c	t	t	a	g	a	c	a	a	g	g	g	t	c	c	c	a	t	a	a	t	t	t	t	c	c	c	c	g	t	g	t	c	c	c	a	t	a
NC001348	1	t	g	-	a	a	a	a	a	c	t	c	t	g	g	c	c	t	c	c	t	c	t	g	g	g	t	c	c	g	g	g	g	c	a	a	c	t	t	a	g	a	c	a	a	g	g	g	t	c	c	c	a	t	a	a	t	t	t	t	c	c	c	c	g	t	g	t	c	c	c	a	t	a
KP771892	1	t	g	-	a	a	a	a	a	c	t	c	t	g	g	c	c	t	c	c	t	t	t	g	g	g	t	c	c	g	g	g	g	c	a	a	c	t	t	a	g	a	c	a	a	g	g	g	t	c	c	c	a	t	a	a	t	t	t	t	c	c	c	c	g	t	g	t	c	c	c	a	t	a
KP771894	1	t	g	-	a	a	a	a	a	c	t	c	t	g	g	c	c	t	c	c	t	c	t	g	g	g	t	c	c	g	g	g	g	c	a	a	c	t	t	a	g	a	c	a	a	g	g	g	t	c	c	c	a	t	a	a	t	t	t	t	c	c	c	c	g	t	g	t	c	c	c	a	t	a
KP771900	1	t	g	-	a	a	a	a	a	c	t	c	t	g	g	c	c	t	c	c	t	c	t	g	g	g	t	c	c	g	g	g	g	c	a	a	c	t	t	a	g	a	c	a	a	g	g	g	t	c	c	c	a	t	a	a	t	t	t	t	c	c	c	c	g	t	g	t	c	c	c	a	t	a
KP771901	1	t	g	-	a	a	a	a	a	c	t	c	t	g	g	c	c	t	c	c	t	c	t	g	g	g	t	c	c	g	g	g	g	c	a	a	c	t	t	a	g	a	c	a	a	g	g	g	t	c	c	c	a	t	a	a	t	t	t	t	c	c	c	c	g	t	g	t	c	c	c	a	t	a
KP771902	1	t	g	-	a	a	a	a	a	c	t	c	t	g	g	c	c	t	c	c	t	c	t	g	g	g	t	c	c	g	g	g	g	c	a	a	c	t	t	a	g	a	c	a	a	g	g	g	t	c	c	c	a	t	a	a	t	t	t	t	c	c	c	c	g	t	g	t	c	c	c	a	t	a
KP771908	1	t	g	-	a	a	a	a	a	c	t	c	t	g	g	c	c	t	c	c	t	c	t	g	g	g	t	c	c	g	g	g	g	c	a	a	c	t	t	a	g	a	c	a	a	g	g	g	t	c	c	c	a	t	a	a	t	t	t	t	c	c	c	c	g	t	g	t	c	c	c	a	t	a
KP771912	1	t	g	-	a	a	a	a	a	c	t	c	t	g	g	c	c	t	c	c	t	c	t	g	g	g	t	c	c	g	g	g	g	c	a	a	c	t	t	a	g	a	c	a	a	g	g	g	t	c	c	c	a	t	a	a	t	t	t	t	c	c	c	c	g	t	g	t	c	c	c	a	t	a
KP771913	1	t	g	-	a	a	a	a	a	c	t	c	t	g	g	c	c	t	c	c	t	c	t	g	g	g	t	c	c	g	g	g	g	c	a	a	c	t	t	a	g	a	c	a	a	g	g	g	t	c	c	c	a	t	a	a	t	t	t	t	c	c	c	c	g	t	g	t	c	c	c	a	t	a
KP771917	1	t	g	-	a	a	a	a	a	c	t	c	t	g	g	c	c	t	c	c	t	c	t	g	g	g	t	c	c	g	g	g	g	c	a	a	c	t	t	a	g	a	c	a	a	g	g	g	t	c	c	c	a	t	a	a	t	t	t	t	c	c	c	c	g	t	g	t	c	c	c	a	t	a
KP771918	1	t	g	-	a	a	a	a	a	c	t	c	t	g	g	c	c	t	c	c	t	c	t	g	g	g	t	c	c	g	g	g	g	c	a	a	c	t	c	a	g	a	c	a	a	g	g	g	t	c	c	c	a	t	a	a	t	t	t	t	c	c	c	c	g	t	g	t	c	c	c	a	t	a
KP771920	1	t	g	-	a	a	a	a	a	c	t	c	t	g	g	c	c	t	c	c	t	c	t	g	g	g	t	c	c	g	g	g	g	c	a	a	c	t	t	a	g	a	c	a	a	g	g	g	t	c	c	c	a	t	a	a	t	t	t	t	c	c	c	c	g	t	g	t	c	c	c	a	t	a
MH709361	1	t	g	-	a	a	g	a	a	c	t	c	t	g	g	c	c	t	c	c	t	c	t	g	g	g	t	c	c	g	g	g	g	c	a	a	c	t	t	a	g	a	c	a	a	g	g	g	t	c	c	c	a	t	a	a	t	t	t	t	c	c	c	c	g	t	g	t	c	c	c	a	t	a
KU926318	2	t	g	-	-	-	a	a	a	c	t	c	t	g	g	c	c	g	c	c	t	c	t	g	g	g	t	c	c	g	g	g	g	c	a	a	c	c	t	a	g	a	c	a	g	g	g	g	t	c	c	c	a	t	a	a	t	t	t	t	c	t	c	c	g	t	g	t	c	c	c	a	t	a
KU926319	2	t	g	-	-	a	a	a	a	c	t	c	t	g	g	c	c	g	c	c	t	c	t	g	g	g	t	c	c	g	g	g	g	c	a	a	c	c	t	a	g	a	c	a	a	g	g	g	t	c	c	c	a	t	a	a	t	t	t	t	c	t	c	c	g	c	g	t	c	c	c	a	t	a
KU926320	2	t	g	-	-	a	a	a	a	c	t	c	t	g	g	c	c	g	c	c	t	c	t	g	g	g	t	c	c	g	g	g	g	c	a	a	c	c	t	a	g	a	c	a	a	g	g	g	t	c	c	c	a	t	a	a	t	t	t	t	c	t	c	c	g	c	g	t	c	c	c	a	t	a
KU926321	2	t	g	-	a	a	a	a	a	c	t	c	t	g	g	c	c	g	c	c	t	c	t	g	g	g	t	c	c	g	g	g	g	c	a	a	c	c	t	a	g	a	c	a	a	g	g	g	t	c	c	c	a	t	a	a	t	t	t	t	c	t	c	c	g	c	g	t	c	c	c	a	t	a
KU926322	2	t	g	-	-	a	a	a	a	c	t	c	t	g	g	c	c	g	c	c	t	c	t	g	g	g	t	c	c	g	g	g	g	c	a	a	c	c	t	a	g	a	c	a	a	g	g	g	t	c	c	c	a	t	a	a	t	t	t	t	c	t	c	c	g	c	g	t	c	c	c	a	t	a
KC847290	2	t	g	-	-	a	a	a	a	c	t	c	t	g	g	c	c	g	c	c	t	c	t	g	g	g	t	c	c	g	g	g	g	c	a	a	c	c	t	a	g	a	c	a	a	g	g	g	t	c	c	c	a	t	a	a	t	t	t	t	c	t	c	c	g	c	g	t	c	c	c	a	t	a
KJ767491	2	t	g	-	-	-	a	a	a	c	t	c	t	g	g	c	c	g	c	c	t	c	t	g	g	g	t	c	c	g	g	g	g	c	a	a	c	c	t	a	g	a	c	a	a	g	g	g	t	c	c	c	a	t	a	a	t	t	t	t	c	t	c	c	g	t	g	t	c	c	c	a	t	a
KJ767492	2	t	g	-	-	a	a	a	a	c	t	c	t	g	g	c	c	g	c	c	t	c	t	g	g	g	t	c	c	g	g	g	g	c	a	a	c	c	t	a	g	a	c	a	a	g	g	g	t	c	c	c	a	t	a	a	t	t	t	t	c	t	c	c	g	c	g	t	c	c	c	a	t	a
KJ808816	2	t	g	-	a	a	a	a	a	c	t	c	t	g	g	c	c	g	c	c	t	c	t	g	g	g	t	c	c	g	g	g	g	c	a	a	c	c	t	a	g	a	c	a	a	g	g	g	t	c	c	c	a	t	a	a	t	t	t	t	c	t	c	c	g	c	g	t	c	c	c	a	t	a
KP771925	2	t	g	-	a	a	a	a	a	c	t	c	t	g	g	c	c	g	c	c	t	c	t	g	g	g	t	c	c	g	g	g	g	c	a	a	c	c	t	a	g	a	c	a	a	g	g	g	t	c	c	c	a	t	a	a	t	t	t	t	c	t	c	c	g	c	g	t	c	c	c	a	t	a
KU926315	2	t	g	-	-	-	a	a	a	c	t	c	t	g	g	c	c	g	c	c	t	c	t	g	g	g	t	c	c	g	g	g	g	c	a	a	c	c	t	a	g	a	c	a	a	g	g	g	t	c	c	c	a	t	a	a	t	t	t	t	c	t	c	c	g	t	g	t	c	c	c	a	t	a
KU926316	2	t	g	-	-	-	a	a	a	c	t	c	t	g	g	c	c	g	c	c	t	c	t	g	g	g	t	c	c	g	g	g	g	c	a	a	c	c	t	a	g	a	c	a	a	g	g	g	t	c	c	c	a	t	a	a	t	t	t	t	c	t	c	c	g	t	g	t	c	c	c	a	t	a
KU926317	2	t	g	-	-	-	a	a	a	c	t	c	t	g	g	c	c	g	c	c	t	c	t	g	g	g	t	c	c	g	g	g	g	c	a	a	c	c	t	a	g	a	c	a	a	g	g	g	t	c	c	c	a	t	a	a	t	t	t	t	c	t	c	c	g	t	g	t	c	c	c	a	t	a
KY037796	2	t	g	-	-	-	a	a	a	c	t	c	t	g	g	c	c	g	c	c	t	c	t	g	g	g	t	c	c	g	g	g	g	c	a	a	c	c	t	a	g	a	c	a	a	g	g	g	t	c	c	c	a	t	a	a	t	t	t	t	c	t	c	c	g	t	g	t	c	c	c	a	t	a
MF004348	2	t	g	-	-	a	a	a	a	c	t	c	t	g	g	c	c	g	c	c	t	c	t	g	g	g	t	c	c	g	g	g	g	c	a	a	c	c	t	a	g	a	c	a	a	g	g	g	t	c	c	c	a	t	a	a	t	t	t	t	c	t	c	c	g	c	g	t	c	c	c	a	t	a
MH709309	2	t	g	-	a	g	a	a	c	c	t	c	t	g	g	c	c	g	c	c	t	c	t	g	g	g	t	c	c	g	g	g	g	c	a	a	c	c	t	a	g	a	c	a	a	g	g	g	t	c	c	c	a	t	c	a	t	t	t	t	c	t	c	c	g	t	g	t	c	c	c	a	t	a
MH709310	2	t	g	-	g	a	a	a	a	c	t	c	t	g	g	c	c	g	c	c	t	c	t	g	g	g	t	c	c	g	g	g	g	c	a	a	c	c	t	a	g	a	c	a	a	g	g	g	t	c	c	c	a	t	a	a	t	t	t	t	c	t	c	c	g	t	g	t	c	c	c	a	t	a
MH709311	2	t	g	-	-	-	-	a	a	c	t	c	t	g	g	c	c	g	c	c	t	c	t	g	g	g	t	c	c	g	g	g	g	c	a	a	c	c	t	a	g	a	c	a	a	g	g	g	t	c	c	c	a	t	a	a	t	t	t	t	c	t	c	c	g	t	g	t	c	c	c	a	t	a
MH709313	2	t	g	-	a	g	a	a	a	c	t	c	t	g	g	c	c	g	c	c	t	c	t	g	g	g	t	c	c	g	g	g	g	c	a	a	c	c	t	a	g	a	c	a	a	g	g	g	t	c	c	c	a	t	a	a	t	t	t	t	c	t	c	c	g	t	g	t	c	c	c	a	t	a
MH709314	2	t	g	-	a	a	a	a	a	c	t	c	t	g	g	c	c	g	c	c	t	c	t	g	g	g	t	c	c	g	g	g	g	c	a	a	c	c	t	a	g	a	c	a	a	g	g	g	t	c	c	c	a	t	a	a	t	t	t	t	c	t	c	c	g	t	g	t	c	c	c	a	t	a
MH709315	2	t	g	-	a	g	a	a	a	c	t	c	t	g	g	c	c	g	c	c	t	c	t	g	g	g	t	c	c	g	g	g	g	c	a	a	c	c	t	a	g	a	c	a	a	g	g	g	t	c	c	c	a	t	a	a	t	t	t	t	c	t	c	c	g	t	g	t	c	c	c	a	t	a
MH709319	2	t	g	-	-	a	a	a	a	c	t	c	t	g	g	c	c	g	c	c	t	c	t	g	g	g	t	c	c	g	g	g	g	c	a	a	c	c	t	a	g	a	c	a	a	g	g	g	t	c	c	c	a	t	a	a	t	t	t	t	c	t	c	c	g	t	g	t	c	c	c	a	t	a
MH709320	2	t	g	-	a	a	a	a	a	c	t	c	t	g	g	c	c	g	c	c	t	c	t	g	g	g	t	c	c	g	g	g	g	c	a	a	c	c	t	a	g	a	a	a	a	g	g	g	t	c	c	c	a	t	a	a	t	t	t	t	c	t	c	c	g	t	g	t	c	c	c	a	t	a
MH709329	2	t	g	-	-	a	a	a	a	c	t	c	t	g	g	c	c	g	c	c	t	c	t	g	g	g	t	c	c	g	g	g	g	c	a	a	c	c	t	a	g	a	c	a	a	g	g	g	t	c	c	c	a	t	a	a	t	t	t	t	c	t	c	c	g	t	g	t	c	c	c	a	t	a
MH709331	2	t	g	-	a	a	a	a	a	c	t	c	t	g	g	c	c	g	c	c	t	c	t	g	g	g	t	c	c	g	g	g	g	c	a	a	c	c	t	a	g	a	c	a	a	g	g	g	t	c	c	c	a	t	a	a	t	t	t	t	c	t	c	c	g	t	g	t	c	c	c	a	t	a
MH709333	2	t	g	-	-	a	a	a	a	c	t	c	t	g	g	c	c	g	c	c	t	c	t	g	g	g	t	c	c	g	g	g	g	c	a	a	c	c	t	a	g	a	c	a	a	g	g	g	t	c	c	c	a	t	a	a	t	t	t	t	c	t	c	c	g	t	g	t	c	c	c	a	t	a
MH709337	2	t	g	-	a	a	a	a	a	c	t	c	t	g	g	c	c	g	c	c	t	c	t	g	g	g	t	c	c	g	g	g	g	c	a	a	c	c	t	a	g	a	c	a	a	g	g	g	t	c	c	c	a	t	a	a	t	t	t	t	c	t	c	c	g	t	g	t	c	c	c	a	t	a
MH709339	2	t	a	-	-	-	-	a	a	c	t	c	t	g	g	c	c	g	c	c	g	c	t	g	g	g	t	c	c	g	g	g	g	c	a	a	c	c	t	a	g	a	c	a	a	g	g	g	t	c	c	c	a	t	a	a	t	t	t	t	c	t	c	c	a	t	g	t	c	c	c	a	t	a
MH709344	2	t	g	-	a	a	a	a	a	c	t	c	t	g	g	c	c	g	c	c	t	c	t	g	g	g	t	c	c	g	g	g	g	c	a	a	c	c	t	a	g	a	c	a	a	g	g	g	t	c	c	c	a	t	a	a	t	t	t	t	c	t	c	c	g	t	g	t	c	c	c	a	t	a
MH709348	2	t	g	-	a	a	a	a	a	c	t	c	t	g	g	c	c	g	c	c	t	c	t	g	g	g	t	c	c	g	g	g	g	c	a	a	c	c	t	a	g	a	c	a	a	g	g	g	t	c	c	c	a	t	a	a	t	t	t	t	c	t	c	c	g	t	g	t	c	c	c	a	t	a
MH709355	2	t	g	-	a	a	a	a	a	c	t	c	t	g	g	c	c	g	c	c	c	c	t	g	g	g	t	c	c	g	g	g	g	c	a	a	c	c	t	a	a	a	c	a	a	g	g	g	t	c	c	c	a	t	a	a	t	t	t	t	c	t	c	c	g	t	g	t	c	c	c	a	t	a
MH709359	2	t	g	-	a	g	a	a	a	c	t	c	t	g	g	c	c	g	c	c	t	c	t	g	g	g	t	c	c	g	g	g	g	c	a	a	c	c	t	a	g	a	c	a	a	g	g	g	t	c	c	c	a	t	a	a	t	t	t	t	c	t	c	c	g	t	g	t	c	c	c	a	t	a
MH709363	2	t	g	-	a	a	a	a	a	c	t	c	t	g	g	c	c	g	c	c	t	c	t	g	g	g	t	c	c	g	g	g	g	c	a	a	c	c	t	a	g	a	c	a	a	g	g	g	t	c	c	c	a	t	a	a	t	t	t	t	c	t	c	c	g	t	g	t	c	c	c	a	t	a
MH709366	2	t	g	-	g	a	a	a	a	c	t	c	t	g	g	c	c	g	c	c	t	c	t	g	g	g	t	c	c	g	g	g	g	c	a	a	c	c	t	a	g	a	c	a	a	g	g	g	t	c	c	c	a	t	a	a	t	t	t	t	c	t	c	t	g	t	g	t	c	c	c	a	t	a
MH709376	2	t	g	-	a	g	a	a	a	c	t	c	t	g	g	c	c	g	c	c	t	c	t	g	g	g	t	c	c	g	g	g	g	c	a	a	c	c	t	a	g	a	c	a	a	g	g	g	t	c	c	c	a	t	a	a	t	t	t	t	c	t	c	c	g	t	g	t	c	c	c	a	t	a
MH709377	2	t	g	-	a	a	a	a	a	c	t	c	t	g	g	c	c	g	c	c	t	c	t	a	g	g	t	c	c	g	g	g	g	c	a	a	c	c	t	a	g	a	c	a	a	g	g	g	t	c	c	c	a	t	a	a	t	t	t	t	c	t	c	c	g	c	g	t	c	c	c	a	t	a
**MW316406**	2	t	g	-	-	-	a	a	a	c	t	c	t	g	g	c	c	g	c	c	t	c	t	g	g	g	t	c	c	g	g	g	g	c	a	a	c	c	t	a	g	a	c	a	a	g	g	g	t	c	c	c	a	t	a	a	t	t	t	t	c	t	c	c	g	t	g	t	c	c	c	a	t	a
AJ871403	3	t	g	-	-	-	-	a	a	c	t	c	t	g	g	c	c	g	c	c	t	c	t	g	g	g	t	c	c	g	g	g	g	c	a	a	c	c	t	a	g	a	c	a	a	g	g	g	t	c	c	c	a	t	a	a	t	t	t	t	c	t	c	c	g	t	g	t	c	c	c	a	t	a
DQ479955	3	t	g	-	-	-	-	a	a	c	t	c	t	g	g	c	c	g	c	c	t	c	t	g	g	g	t	c	c	g	g	g	g	c	a	a	c	c	t	a	g	a	c	a	a	g	g	g	t	c	c	c	a	t	a	a	t	t	t	t	c	t	c	c	g	t	g	t	c	c	c	a	t	a
DQ479956	3	t	g	-	-	-	a	a	a	c	t	c	t	g	g	c	c	g	c	c	t	c	t	g	g	g	t	c	c	g	g	g	g	c	a	a	c	c	t	a	g	a	c	a	a	g	g	g	t	c	c	c	a	t	a	a	t	t	t	t	c	t	c	c	g	t	g	t	c	c	c	a	t	a
DQ479957	3	t	g	-	-	a	a	a	a	c	t	c	t	g	g	c	c	g	c	c	t	c	t	g	g	g	t	c	c	g	g	g	g	c	a	a	c	c	t	a	g	a	c	a	a	g	g	g	t	c	c	c	a	t	a	a	t	t	t	t	c	t	c	c	g	t	g	t	c	c	c	a	t	a
JN704699	3	t	g	-	a	a	a	a	a	c	t	c	t	g	g	c	c	g	c	c	t	c	t	g	g	g	t	c	c	g	g	g	g	c	c	a	c	c	t	a	g	a	c	a	a	g	g	g	t	c	c	c	a	t	a	a	t	t	t	t	c	t	c	c	g	t	g	t	c	c	c	a	t	a
JN704700	3	t	g	-	a	a	a	a	a	c	t	c	t	g	g	c	c	g	c	c	t	c	t	g	g	g	t	c	c	g	g	g	g	c	a	a	c	t	t	a	g	a	c	a	a	g	g	g	t	c	c	c	a	t	a	a	t	t	t	t	c	t	c	c	g	t	g	t	c	c	c	a	t	a
JN704701	3	t	g	-	-	a	a	a	a	c	t	c	t	g	g	c	c	g	c	c	t	c	t	g	g	g	t	c	c	g	g	g	g	c	a	a	c	c	t	a	g	a	c	a	a	g	g	g	t	c	c	c	a	t	a	a	t	t	t	t	c	t	c	c	g	t	g	t	c	c	c	a	t	a
JN704702	3	t	g	-	a	a	a	a	a	c	t	c	t	g	g	c	c	g	c	c	t	c	t	g	g	g	t	c	c	g	g	g	g	c	a	a	c	c	t	a	g	a	c	a	a	g	g	g	t	c	c	c	a	t	a	a	t	t	t	t	c	t	c	c	g	t	g	t	c	c	c	a	t	a
JN704703	3	t	g	-	a	a	a	a	a	c	t	c	t	g	g	c	c	g	c	c	t	c	t	g	g	g	t	c	c	g	g	g	g	c	a	a	c	c	t	a	g	a	c	a	a	g	g	g	t	c	c	c	a	t	a	a	t	t	t	t	c	t	c	c	g	t	g	t	c	c	c	a	t	a
KP771891	3	t	g	-	a	a	a	a	a	c	t	c	t	g	g	c	c	g	c	c	t	c	t	g	g	g	t	c	c	g	g	g	g	c	a	a	c	c	t	a	g	a	c	a	a	g	g	g	t	c	c	c	a	t	a	a	t	t	t	t	c	t	c	c	g	t	g	t	c	c	c	a	t	a
KP771893	3	t	g	-	a	a	a	a	a	c	t	c	t	g	g	c	c	g	c	c	t	c	t	g	g	g	t	c	c	g	g	g	g	c	a	a	c	c	t	a	g	a	c	a	a	g	t	g	t	c	c	c	a	t	a	a	t	t	t	t	c	t	c	c	g	t	g	t	c	c	c	a	t	a
KP771895	3	t	g	-	a	a	a	a	a	c	t	c	t	g	g	c	c	g	c	c	t	c	t	g	g	g	t	c	c	g	g	g	g	c	a	a	c	c	t	a	g	a	c	a	a	g	g	g	t	c	c	c	a	t	a	a	t	t	t	t	c	t	c	c	g	t	g	t	c	c	c	a	t	a
KP771896	3	t	g	-	a	a	a	a	a	c	t	c	t	g	g	c	c	g	c	c	t	c	t	g	g	g	t	c	c	g	g	g	g	c	a	a	c	c	t	a	g	a	c	a	a	g	g	g	t	c	c	c	a	t	a	a	t	t	t	t	t	t	c	c	g	t	g	t	c	c	c	a	t	a
KP771897	3	t	g	-	a	a	a	a	a	c	t	c	t	g	g	c	c	g	c	c	t	c	t	g	g	g	t	c	c	g	g	g	g	c	a	a	c	c	t	a	g	a	c	a	a	g	g	g	t	c	c	c	a	t	a	a	t	t	t	t	c	t	c	c	g	t	g	t	c	c	c	a	t	a
KP771899	3	t	g	-	a	a	a	a	a	c	t	c	t	g	g	c	c	g	c	c	t	c	t	g	g	g	t	c	c	g	g	g	g	c	a	a	c	c	t	a	g	a	c	a	a	g	g	g	t	c	c	c	a	t	a	a	t	t	t	t	c	t	c	c	g	t	g	t	c	c	c	a	t	a
KP771903	3	t	g	-	a	a	a	a	a	c	t	c	t	g	g	c	c	g	c	c	t	c	t	g	g	g	t	c	c	g	g	g	g	c	a	a	c	c	t	a	g	a	c	a	a	g	g	g	t	c	c	c	a	t	a	a	t	t	t	t	c	t	c	c	g	t	g	t	c	c	c	a	t	a
KP771907	3	t	g	-	a	a	a	a	a	c	t	c	t	g	g	c	c	g	c	c	t	c	t	g	g	g	t	c	c	g	g	g	g	c	a	a	c	c	t	a	g	a	c	a	a	g	g	g	t	c	c	c	a	t	a	a	t	t	t	t	c	t	c	c	g	t	g	t	c	c	c	a	t	a
KP771910	3	t	g	a	a	a	a	a	a	c	t	c	t	g	g	c	c	g	c	c	t	c	t	g	g	g	t	c	c	g	g	g	g	c	a	a	c	c	t	a	g	a	c	a	a	g	g	g	t	c	c	c	a	t	a	a	t	t	t	t	c	t	c	c	g	t	g	t	c	c	c	a	t	a
KP771915	3	t	g	-	a	a	a	a	a	c	t	c	t	g	g	c	c	g	c	c	t	c	t	g	g	g	t	c	c	g	g	g	g	c	a	a	c	c	t	a	g	a	c	a	a	g	g	g	t	c	c	c	a	t	a	a	c	t	t	t	c	t	c	c	g	t	g	t	c	c	c	a	t	a
KP771916	3	t	g	-	a	a	a	a	a	c	t	c	t	g	g	c	c	g	c	c	t	c	t	g	g	g	t	c	c	g	g	g	g	c	a	a	c	c	t	a	g	a	c	a	a	g	g	g	t	c	c	c	a	t	a	a	t	t	t	t	c	t	c	c	g	t	g	t	c	c	c	a	t	a
KP771921	3	t	g	-	a	a	a	a	a	c	t	c	t	g	g	c	c	g	c	c	t	c	t	g	g	g	t	c	c	g	g	g	g	c	a	a	c	c	t	a	g	a	c	a	a	g	g	g	t	c	c	c	a	t	a	a	t	t	t	t	c	t	c	c	g	t	g	t	c	c	c	a	t	a
KP771923	3	t	g	-	a	a	a	a	a	c	t	c	t	g	g	c	c	g	c	c	t	c	t	g	g	g	t	c	c	g	g	g	g	c	a	a	c	c	t	a	g	a	c	g	a	g	g	g	t	c	c	c	a	t	a	a	t	t	t	t	c	t	c	c	g	t	g	t	c	c	c	a	t	a
KP771889	3	t	g	-	a	a	a	a	a	c	t	c	t	g	g	c	c	g	c	c	t	c	t	g	g	g	t	c	c	g	g	g	g	c	a	a	c	c	t	a	g	a	c	a	a	g	g	g	t	c	c	c	a	t	a	a	t	t	t	t	c	t	c	c	g	t	g	t	c	c	c	a	t	a
KU926311	3	t	g	-	-	a	a	a	a	c	t	c	t	g	g	c	c	g	c	c	t	c	t	g	g	g	t	c	c	g	g	g	g	c	a	a	c	c	t	a	g	a	c	a	a	g	g	g	t	c	c	c	a	t	a	a	t	t	t	t	c	t	c	c	g	t	g	t	c	c	c	a	t	a
JQ911599	3	t	g	-	-	-	-	a	a	c	t	c	t	g	g	c	c	g	c	c	t	c	t	g	g	g	t	c	c	g	g	g	g	c	a	a	c	c	t	a	g	a	c	a	a	g	g	g	t	t	c	c	a	t	a	a	t	t	t	t	c	t	c	c	g	t	g	t	c	c	c	a	t	a
MH709317	3	t	g	-	a	a	a	a	a	c	t	c	t	g	g	c	c	g	c	c	t	c	t	g	g	g	t	c	c	g	g	g	g	c	a	a	c	c	t	a	g	a	c	a	a	g	g	g	t	c	c	c	c	t	a	a	t	t	t	t	c	t	c	c	g	t	g	t	c	c	c	a	t	a
MH709346	3	t	g	-	a	a	g	a	a	c	t	c	t	g	g	c	c	g	c	c	t	c	t	g	a	a	t	c	c	g	g	g	g	c	a	a	c	c	t	a	g	a	c	a	a	g	g	g	t	c	c	c	c	t	a	a	t	t	t	t	c	t	c	c	g	t	g	t	c	c	c	a	t	a
MH709375	3	t	g	-	a	a	a	a	a	c	t	c	t	g	g	c	c	g	c	c	t	c	t	g	g	g	t	c	c	g	g	g	g	c	a	a	c	c	t	a	g	a	c	a	a	g	g	g	t	c	c	c	a	t	a	a	t	t	t	t	c	t	c	c	g	t	g	t	c	c	c	a	t	a
DQ452050	4	t	g	-	-	-	-	a	a	c	t	c	t	g	g	c	c	t	c	c	t	c	t	g	g	g	t	c	c	g	g	g	g	t	a	a	c	t	t	a	g	a	c	a	a	g	g	g	t	c	c	c	a	t	a	a	t	t	t	t	c	c	c	c	g	t	g	t	c	c	c	a	t	a
DQ479960	4	t	g	-	-	-	-	a	a	c	t	c	t	g	g	c	c	t	c	c	t	c	t	g	g	g	t	c	c	g	g	g	g	c	a	a	c	t	t	a	g	a	c	a	a	g	g	g	t	c	c	c	a	t	a	a	t	t	t	t	c	c	c	c	g	t	g	t	c	c	c	a	t	a
KP771898	4	t	g	-	a	a	a	a	a	c	t	c	t	g	g	c	c	t	c	c	t	c	t	g	g	g	t	c	c	g	g	g	g	c	a	a	c	t	t	a	g	a	c	a	a	g	g	g	t	c	c	c	a	t	a	a	t	t	t	t	c	c	c	c	g	t	g	t	c	c	c	a	t	a
MH709316	4	t	-	-	-	-	-	a	a	c	t	c	t	g	g	c	c	t	c	c	t	c	t	g	g	g	t	c	c	g	g	g	g	c	a	a	c	t	t	a	g	a	c	a	a	g	g	g	t	c	c	c	a	t	a	a	t	t	t	t	c	c	c	c	g	t	g	c	c	a	c	a	t	a
MH709318	4	t	g	-	a	a	g	a	a	c	t	c	t	g	g	c	c	t	c	c	t	c	t	g	g	g	t	c	c	g	g	g	g	c	a	a	c	t	t	a	g	a	c	a	a	g	g	g	t	c	c	c	a	t	a	a	t	t	t	t	c	c	c	c	g	t	g	t	c	c	c	g	t	a
MH709322	4	t	g	-	a	a	a	a	a	c	t	c	t	g	g	c	c	t	c	c	t	c	t	g	g	g	t	c	c	g	g	g	g	c	a	a	c	t	t	a	g	a	c	a	a	g	g	g	t	c	c	c	a	t	a	a	t	t	t	t	c	c	c	c	g	t	g	t	c	c	c	g	t	a
MH709323	4	t	g	-	a	a	g	a	a	c	t	c	t	g	g	c	c	t	c	c	t	c	t	g	g	g	t	c	c	g	g	g	g	c	a	a	c	t	t	a	g	a	c	a	a	g	g	g	t	c	c	c	a	t	a	a	t	t	t	t	c	c	c	c	g	t	g	t	c	c	c	a	t	a
MH709326	4	t	g	-	a	a	a	a	a	c	t	c	t	g	g	c	c	t	c	c	t	c	t	g	g	g	t	c	c	g	g	g	g	c	a	a	c	t	t	a	g	a	c	a	a	g	g	g	t	c	c	c	a	t	a	a	t	t	t	t	c	c	c	c	g	t	g	t	c	c	c	a	t	a
MH709341	4	t	g	-	a	a	a	c	a	c	t	c	t	g	g	c	c	t	c	c	t	c	t	g	g	g	t	c	c	g	g	g	g	c	a	a	c	t	t	a	g	a	c	a	a	g	g	g	t	c	c	c	a	t	a	a	t	t	t	t	c	c	c	c	g	t	g	t	c	c	c	a	t	a
MH709357	4	t	g	-	a	a	a	a	a	c	t	c	t	g	g	c	c	t	c	c	t	c	t	g	g	g	t	c	c	g	g	g	g	c	a	a	c	t	t	a	g	a	c	a	a	g	g	g	t	c	c	c	a	t	a	a	t	t	t	t	c	c	c	c	g	t	g	t	c	c	c	a	g	a
MH709364	4	t	-	-	-	-	-	a	a	c	t	c	t	g	g	c	c	t	c	c	t	c	t	g	g	g	t	c	c	g	g	g	g	c	a	a	c	t	t	a	g	a	c	a	a	g	g	g	t	c	c	c	a	t	a	a	t	t	t	t	c	c	c	c	g	t	g	t	c	c	c	a	t	a
MH709370	4	t	-	-	-	-	-	a	a	c	t	c	t	g	g	c	c	t	c	c	t	c	t	g	g	g	t	c	c	g	g	g	g	c	a	a	c	t	t	a	g	a	c	a	a	g	g	g	t	c	c	c	a	t	a	a	t	t	t	t	c	c	c	c	g	t	g	t	c	c	c	a	t	a
MH709371	4	t	-	-	-	-	-	a	a	c	t	c	t	g	g	c	c	t	c	c	t	c	t	g	g	g	t	c	c	g	g	g	g	c	a	a	c	t	t	a	g	a	c	a	a	g	g	g	t	c	c	c	a	t	a	a	t	t	t	t	c	c	c	c	g	t	g	t	c	c	c	a	t	a
MH709372	4	t	g	-	a	a	g	a	a	c	t	c	t	g	g	c	c	t	c	c	t	c	t	g	g	g	t	c	c	g	g	g	g	c	a	a	c	t	t	a	g	a	c	a	a	g	g	g	t	c	c	c	a	t	a	a	t	t	t	t	c	c	c	c	g	t	a	t	c	c	c	a	t	a
DQ457052	5	t	g	-	-	-	-	a	a	c	t	c	t	g	g	c	c	g	c	c	t	c	t	g	g	g	t	c	c	t	g	g	g	c	a	a	c	c	t	a	g	a	c	a	a	g	g	g	t	c	c	c	a	t	a	a	t	g	t	t	c	t	c	c	g	t	g	t	c	c	c	a	t	a
JN704704	5	t	g	-	a	a	a	a	a	c	t	c	t	g	g	c	c	g	c	c	t	c	t	g	g	g	t	c	c	g	g	a	g	c	a	a	c	c	t	a	g	a	c	a	a	g	g	g	t	c	c	c	a	t	a	a	t	t	t	t	c	t	c	c	g	t	g	t	c	c	c	a	t	a
JN704705	5	t	g	-	a	a	a	a	a	c	t	c	t	g	g	c	c	g	c	c	t	c	t	g	g	g	t	c	c	g	g	g	g	c	a	a	c	c	t	a	g	a	c	a	a	g	g	g	t	c	c	c	a	t	a	a	t	t	t	t	c	t	c	c	g	t	g	t	c	c	c	a	t	a
JN704706	5	t	g	-	a	a	a	a	a	c	t	c	t	g	g	c	c	g	c	c	t	c	t	g	g	g	t	c	c	g	g	g	g	c	a	a	c	c	t	a	g	a	c	a	a	g	g	g	t	c	c	c	a	t	a	a	t	t	t	t	c	t	c	c	g	t	g	t	c	c	c	a	t	a
JN704707	5	t	g	-	a	a	a	a	a	c	t	c	t	g	g	c	c	g	c	c	t	c	t	g	g	g	t	c	c	g	g	g	g	c	a	a	c	c	t	a	g	a	c	a	a	g	g	g	t	c	c	c	a	t	a	a	t	t	t	t	c	t	c	c	g	t	g	t	c	c	c	a	t	a
JN704708	5	t	g	-	a	a	a	a	a	c	t	c	t	g	g	c	c	g	c	c	t	c	t	g	g	g	t	c	c	g	g	g	g	c	a	a	c	c	t	a	g	a	c	a	a	g	g	g	t	c	c	c	a	t	a	a	t	t	t	t	c	c	c	c	g	t	g	t	c	c	c	a	t	a
KP771904	5	t	g	-	a	a	a	a	a	c	t	c	t	g	g	c	c	g	c	c	t	c	t	g	g	g	t	c	c	g	g	g	g	c	a	a	c	c	t	a	g	a	c	a	a	g	g	g	t	c	c	c	a	t	a	a	t	t	t	t	c	t	c	c	g	t	g	t	c	c	c	a	t	a
KP771905	5	t	g	-	a	a	a	a	a	c	t	c	t	g	g	c	c	g	c	c	t	c	t	g	g	g	t	c	c	g	g	g	g	c	a	a	c	c	t	a	g	a	c	a	a	g	g	g	t	c	a	c	a	t	a	a	t	t	t	t	c	t	c	c	g	t	g	t	c	c	c	a	t	a
KP771906	5	t	g	-	a	a	a	a	a	c	t	c	t	g	g	c	c	g	c	c	t	c	t	g	g	g	t	c	c	g	g	g	g	c	a	a	c	c	t	a	g	a	c	a	a	g	g	g	t	c	a	c	a	t	a	a	t	t	t	t	c	t	c	c	g	t	g	t	c	c	c	a	t	a
KP771919	5	t	g	-	a	a	a	a	a	c	t	c	t	g	g	c	c	g	c	c	t	c	t	g	g	g	t	c	c	g	g	g	g	c	a	a	c	c	t	a	g	a	c	a	a	g	g	g	t	c	c	c	a	t	a	a	t	t	t	t	c	t	c	c	g	t	g	t	c	c	c	a	t	a
KP771922	5	t	g	-	a	a	a	a	a	c	t	c	t	g	g	c	c	g	c	c	t	c	t	g	g	g	t	c	c	g	g	g	g	c	a	a	c	c	t	a	g	a	c	a	a	g	g	g	t	c	c	c	a	t	a	a	t	t	t	t	c	t	c	c	g	t	g	t	c	c	c	a	t	a
KP771924	5	t	g	-	a	a	a	a	a	c	t	c	t	g	g	c	c	g	c	c	t	c	t	g	g	g	t	c	c	g	g	g	g	c	a	a	c	c	t	a	g	a	c	a	a	g	g	g	t	c	c	c	a	t	a	a	t	t	t	t	c	t	c	c	g	t	g	t	c	c	c	a	t	a
KM355696	5	t	g	-	a	a	a	a	a	c	t	c	t	g	g	c	c	g	c	c	t	c	t	g	g	g	t	c	c	g	g	g	g	c	a	a	c	c	t	a	g	a	c	a	a	g	g	g	t	c	c	c	a	t	a	g	t	t	t	t	c	t	c	c	g	t	g	t	c	c	c	a	t	a
KM355697	5	t	g	-	a	a	a	a	a	c	t	c	t	g	g	c	c	g	c	c	t	c	t	g	g	g	t	c	c	g	g	g	g	c	a	a	c	c	t	a	g	a	c	a	a	g	g	g	t	c	c	c	a	t	a	g	t	t	t	t	c	t	c	c	g	t	g	t	c	c	c	a	t	a
KM355698	5	t	g	-	a	a	a	a	a	c	t	c	t	g	g	c	c	g	c	c	t	c	t	g	g	g	t	c	c	g	g	g	g	c	a	a	c	c	t	a	g	a	c	a	a	g	g	g	t	c	c	c	a	t	a	a	t	t	t	t	c	t	c	c	g	t	g	t	c	c	c	a	t	a
KM355699	5	t	g	-	a	a	a	a	a	c	t	c	t	g	g	c	c	g	c	c	t	c	t	g	g	g	t	c	c	g	g	g	g	c	a	a	c	c	t	a	g	a	c	a	a	g	g	g	t	c	c	c	a	t	a	a	t	t	t	t	c	t	c	c	g	t	g	t	c	c	c	a	t	a
KM355701	5	t	g	-	a	a	a	a	a	c	t	c	t	g	g	c	c	g	c	c	t	c	t	g	g	g	t	c	c	g	g	g	g	c	a	a	c	c	t	a	g	a	c	a	a	g	g	g	t	c	c	c	a	t	a	g	t	t	t	t	c	t	c	c	g	t	g	t	c	c	c	a	t	a
KM355702	5	t	g	-	a	a	a	a	a	c	t	c	t	g	g	c	c	g	c	c	t	c	t	g	g	g	t	c	c	g	g	g	g	c	a	a	c	c	t	a	g	a	c	a	a	g	g	g	t	c	c	c	a	t	a	a	t	t	t	t	c	t	c	c	g	t	g	t	c	c	c	a	t	a
KM355703	5	t	g	-	a	a	a	a	a	c	t	c	t	g	g	c	c	g	c	c	t	c	t	g	g	g	t	c	c	g	g	g	g	c	a	a	c	c	t	a	g	a	c	a	a	g	g	g	t	c	c	c	a	t	a	g	t	t	t	t	c	t	c	c	g	t	g	t	c	c	c	a	t	a
KM355704	5	t	g	-	a	a	a	a	a	c	t	c	t	g	g	c	c	g	c	c	t	c	t	g	g	g	t	c	c	g	g	g	g	c	a	a	c	c	t	a	g	a	c	a	a	g	g	g	t	c	c	c	a	t	a	g	t	t	t	t	c	t	c	c	g	t	g	t	c	c	c	a	t	a
KM355705	5	t	g	-	a	a	a	a	a	c	t	c	t	g	g	c	c	g	c	c	t	c	t	g	g	g	t	c	c	g	g	g	g	c	a	a	c	c	t	a	g	a	c	a	a	g	g	g	t	c	c	c	a	t	a	a	t	t	t	t	c	t	c	c	g	t	g	t	c	c	c	a	t	a
KM355706	5	t	g	-	a	a	a	a	a	c	t	c	t	g	g	c	c	g	c	c	t	c	t	g	g	g	t	c	c	g	g	g	g	c	a	a	c	c	t	a	g	a	c	a	a	g	g	g	t	c	c	c	a	t	a	a	t	t	t	t	c	t	c	c	g	t	g	t	c	c	c	a	t	a
KM355707	5	t	g	-	a	a	a	a	a	c	t	c	t	g	g	c	c	g	c	c	t	c	t	g	g	g	t	c	c	g	g	g	g	c	a	a	c	c	t	a	g	a	c	a	a	g	g	g	t	c	c	c	a	g	a	g	t	t	t	t	c	t	c	c	g	t	g	t	c	c	c	a	t	a
KM355708	5	t	g	-	a	a	a	a	a	c	t	c	t	g	g	c	c	g	c	c	t	c	t	g	g	g	t	c	c	g	g	g	g	c	a	a	c	c	t	a	g	a	c	a	a	g	g	g	t	c	c	c	a	g	a	g	t	t	t	t	c	t	c	c	g	t	g	t	c	c	c	a	t	a
KM355709	5	t	g	-	a	a	a	a	a	c	t	c	t	g	g	c	c	g	c	c	t	c	t	g	g	g	t	c	c	g	g	g	g	c	a	a	c	c	t	a	g	a	c	a	a	g	g	g	t	c	c	c	a	t	a	a	t	t	t	t	c	t	c	c	g	t	g	t	c	c	c	a	t	a
KM355711	5	t	g	-	a	a	a	a	a	c	t	c	t	g	g	c	c	g	c	c	t	c	t	g	g	g	t	c	c	g	g	g	g	c	a	a	c	c	t	a	g	a	c	a	a	g	g	g	t	c	c	c	a	t	a	a	t	t	t	t	c	t	c	c	g	t	g	t	c	c	c	a	t	a
KM355712	5	t	g	-	a	a	a	a	a	c	t	c	t	g	g	c	c	g	c	c	t	c	t	g	g	g	t	c	c	g	g	g	g	c	a	a	c	c	t	a	g	a	c	a	a	g	g	g	t	c	c	c	a	t	a	a	t	t	t	t	c	t	c	c	g	t	g	t	c	c	c	a	t	a

Supplementary Table 3: 2880 SN

GB-ID	Clade markers																																								9																																	
	Position in Dumas	42386	42391	42391_1	42392	42393	42394	42401	42405	42407	42410	42417	42418	42419	42421	42434	42436	42476	42544	42565	42587	42659	42735	42743	42798	42799	42812	42899	42941	42950	42974	43091	43109	43162	43195	43238	43253	43262	43316	43345	43401	43503	43563	43629	43695	43707	43878	43977	44001	44037	44111	44119	44154	44208	44232	44259	44266	44462	44507	44577	44721	44835	44892	44943	44994	44995	45246	45261	45267	45275	45358	45426	45445	45452
MH499467	5	t	g	-	-	a	a	a	a	a	a	a	a	t	t	a	a	-	c	a	t	c	t	g	g	g	t	c	c	g	g	g	g	c	a	a	c	c	t	a	g	a	c	a	a	g	g	g	t	c	c	c	a	t	a	a	t	t	t	t	c	t	c	c	g	t	g	t	c	c	c	a	t	a
MH499469	5	t	g	-	-	-	-	a	a	c	t	c	t	g	g	c	c	g	c	c	t	c	t	g	g	g	t	c	c	g	g	g	g	c	a	a	c	c	t	a	g	a	c	a	a	g	g	g	t	c	c	c	a	t	a	a	t	t	t	t	c	t	c	c	g	t	g	t	c	c	c	a	t	a
MH709321	5	t	-	-	-	a	g	a	a	c	t	c	t	g	g	c	c	g	c	c	t	c	t	g	g	g	t	c	c	g	g	g	g	c	a	a	c	c	t	a	g	a	c	a	a	g	g	g	t	c	c	c	a	t	a	a	t	t	t	t	c	t	c	c	g	t	g	t	c	c	c	a	t	a
MH709324	5	t	g	-	a	a	a	a	a	c	t	c	t	g	g	c	c	g	c	c	t	c	t	g	g	g	t	c	c	g	g	g	g	c	a	a	c	c	t	a	g	a	c	a	a	g	g	a	t	c	c	c	a	t	a	a	t	t	t	t	c	t	c	c	g	t	g	t	c	c	c	a	t	a
MH709325	5	t	g	-	a	a	a	a	a	c	t	c	t	g	g	c	c	g	c	c	t	c	t	g	g	g	t	c	c	g	g	g	g	c	a	a	c	c	t	a	g	a	c	a	a	g	g	g	t	c	c	c	a	t	a	a	t	t	t	t	c	t	c	c	g	t	g	t	c	c	c	a	t	a
MH709327	5	t	g	-	a	a	a	a	a	c	t	c	t	g	g	c	c	g	c	c	t	c	t	g	g	g	t	c	c	g	g	g	g	c	a	a	c	c	t	a	g	a	c	a	a	g	g	g	t	c	c	c	a	t	a	a	t	t	t	t	c	t	c	c	g	t	g	t	c	c	c	a	t	a
MH709328	5	t	g	-	a	a	g	a	a	c	t	c	t	g	g	c	c	g	c	c	t	c	t	g	g	g	t	c	c	g	g	g	g	c	a	a	c	c	t	a	g	a	c	a	a	g	g	g	t	c	c	c	a	t	a	a	t	t	t	t	c	t	c	c	g	t	g	t	c	c	c	a	t	a
MH709330	5	t	g	-	a	a	g	a	a	c	t	c	t	g	g	c	c	g	c	c	t	c	t	g	g	g	t	c	c	g	g	g	g	c	a	a	c	c	t	a	g	a	c	a	a	g	g	g	t	c	c	c	a	t	a	a	t	t	t	t	c	t	c	c	g	t	g	t	c	c	c	a	t	a
MH709332	5	t	g	-	a	a	g	a	a	c	t	c	t	g	g	c	c	g	c	c	t	c	t	g	g	g	t	c	c	g	g	g	g	c	a	a	c	c	t	a	g	a	c	a	a	g	g	g	t	c	c	c	a	t	a	a	t	t	t	t	c	t	c	c	g	t	g	t	c	c	c	a	t	a
MH709334	5	t	g	-	a	a	g	a	a	c	t	c	t	g	g	c	c	g	c	c	t	c	t	g	g	g	t	c	c	g	g	g	g	c	a	a	t	c	t	a	g	a	c	a	a	g	g	g	t	c	c	c	a	t	a	a	t	t	t	t	c	t	c	c	g	t	g	t	c	c	c	a	t	a
MH709335	5	t	g	-	a	a	g	a	a	c	t	c	t	g	g	c	c	g	c	c	t	c	t	g	g	g	t	c	c	g	g	g	g	c	a	a	c	c	t	a	g	a	c	a	a	g	g	g	t	c	c	c	a	t	a	a	t	t	t	t	c	t	c	c	g	t	g	t	c	c	c	a	t	a
MH709336	5	t	g	-	a	a	g	a	a	c	t	c	t	g	g	c	c	g	c	c	t	c	t	g	g	g	t	c	c	g	g	g	g	c	a	t	c	c	t	a	g	a	c	a	a	g	g	g	t	c	c	c	a	t	a	a	t	t	t	t	c	t	c	c	g	t	g	t	c	c	c	a	t	a
MH709338	5	t	g	-	a	a	a	a	a	c	t	c	t	g	g	c	c	g	c	c	t	c	t	g	g	g	t	c	c	g	g	g	g	c	a	a	c	c	t	a	g	a	c	a	a	g	g	g	t	c	c	c	a	t	a	a	t	t	t	t	c	t	c	c	g	t	g	t	c	c	c	a	t	a
MH709340	5	t	g	-	a	a	g	a	a	c	t	c	t	g	g	c	c	g	c	c	t	c	t	g	g	g	t	c	t	g	g	g	g	c	a	a	c	c	t	a	g	a	c	a	a	g	g	g	t	c	c	c	a	t	a	a	t	t	t	t	c	t	c	c	g	t	g	t	c	c	c	a	t	g
MH709342	5	t	g	-	a	a	g	a	a	c	t	c	t	g	g	c	c	g	c	c	t	c	t	g	g	g	t	c	c	g	g	g	g	c	a	a	c	c	t	a	g	a	c	a	a	g	g	g	t	c	c	c	a	t	a	a	t	t	t	t	c	t	c	c	g	t	g	t	c	c	c	a	t	a
MH709343	5	t	g	-	a	a	g	a	a	c	t	c	t	g	g	c	c	g	c	c	t	c	t	g	g	g	t	c	c	g	g	g	g	c	a	a	c	c	t	a	g	a	c	a	a	g	g	g	t	c	c	c	a	t	a	a	t	t	t	t	c	t	c	c	g	t	g	t	c	c	c	a	t	a
MH709345	5	t	g	-	a	a	g	a	a	c	t	c	t	g	g	c	c	g	c	c	t	c	t	g	g	g	t	c	c	g	g	g	g	c	a	a	c	c	t	a	g	a	c	a	a	g	g	g	t	c	c	c	a	t	a	a	t	t	t	t	c	t	c	c	g	t	g	t	c	c	c	a	t	a
MH709347	5	t	g	-	a	a	a	a	a	c	t	c	t	g	g	c	c	g	c	c	t	c	t	g	g	g	t	c	c	g	g	g	g	c	a	a	c	c	t	a	g	a	c	a	a	g	g	g	t	c	c	c	a	t	a	a	t	t	t	t	c	t	c	c	g	t	g	t	c	c	c	a	t	a
MH709349	5	t	g	-	a	g	a	a	a	c	t	c	t	g	g	c	c	g	c	c	t	c	t	g	g	g	t	c	c	g	g	g	g	c	a	a	c	c	t	a	g	a	c	a	a	g	g	g	t	c	c	c	a	t	a	a	t	t	t	t	c	t	c	c	g	t	g	t	c	c	c	a	t	a
MH709350	5	t	g	-	a	a	a	a	a	c	t	c	t	g	g	c	c	g	c	c	t	c	t	g	g	g	t	c	c	g	g	g	g	c	a	a	c	c	t	a	g	a	c	a	a	g	g	g	t	c	c	c	a	t	a	a	t	t	t	t	c	t	c	c	g	t	g	t	c	c	c	a	t	a
MH709351	5	t	g	-	a	a	a	a	a	c	t	c	t	g	g	c	c	g	c	c	t	c	t	g	g	g	t	c	c	g	g	g	g	c	a	a	c	c	t	a	g	a	c	a	a	g	g	g	t	c	c	c	a	t	a	a	t	t	t	t	c	t	c	c	g	t	g	t	c	c	c	a	t	g
MH709353	5	t	g	-	a	a	g	a	a	c	t	c	t	g	g	c	c	g	c	c	t	c	t	g	g	g	t	c	c	g	g	g	g	c	a	a	c	c	t	a	g	a	c	a	a	g	g	g	t	c	c	c	a	t	a	a	t	t	t	t	c	t	c	c	g	t	g	t	c	c	c	a	t	a
MH709358	5	t	g	-	a	a	a	a	a	c	t	c	t	g	g	c	c	g	c	c	t	c	t	g	g	g	t	c	c	g	g	g	g	c	a	a	c	c	t	a	g	a	c	a	a	g	g	g	t	c	c	c	a	t	a	a	t	t	t	t	c	t	c	c	g	t	g	t	c	c	c	a	t	a
MH709360	5	t	g	-	a	a	g	a	a	c	t	c	t	g	g	c	c	g	c	c	t	c	t	g	g	g	t	c	c	g	g	g	g	c	a	a	c	c	t	a	g	a	c	a	a	g	g	g	t	c	c	c	a	t	a	a	t	t	t	t	c	t	c	c	g	t	g	t	c	c	c	a	t	a
MH709362	5	t	g	-	a	a	g	a	a	c	t	c	t	g	g	c	c	g	c	c	t	c	t	g	g	g	t	c	c	g	g	g	g	c	a	a	c	c	t	a	g	a	c	a	a	g	g	g	t	c	c	c	a	t	a	a	t	t	t	t	c	t	c	c	g	t	g	t	c	c	c	a	t	a
MH709365	5	t	g	-	-	-	-	a	a	c	t	c	t	g	g	c	c	g	c	c	t	c	t	g	g	g	t	c	c	g	g	g	g	c	a	a	c	c	t	a	g	a	c	a	a	g	g	g	t	c	c	c	a	t	a	a	t	t	t	t	c	t	c	c	g	t	g	t	c	c	c	a	t	a
MH709373	5	-	-	-	-	-	g	a	a	c	t	c	t	g	g	c	c	g	c	c	t	c	t	g	g	g	t	c	c	g	g	g	g	c	a	a	c	c	t	a	g	a	c	a	a	a	g	g	t	c	c	c	a	t	a	a	t	t	t	t	c	t	c	c	g	t	g	t	c	c	c	a	t	a
MH709374	5	t	g	-	a	a	a	a	a	c	t	c	t	g	g	c	c	g	c	c	t	c	t	g	g	g	t	c	c	g	g	g	g	c	a	a	c	c	t	a	g	a	c	a	a	g	g	g	t	c	c	c	a	t	a	a	t	t	t	t	c	t	c	c	g	t	g	t	c	c	c	a	t	a
KC112914	6	t	g	-	a	a	a	a	a	c	t	c	t	g	g	c	c	g	c	c	t	c	t	g	g	g	t	c	c	g	a	g	g	c	a	a	c	c	t	a	g	a	c	a	a	g	g	g	t	c	c	c	a	t	a	a	t	t	t	t	c	t	c	c	g	t	g	t	c	c	c	a	t	a
KP771914	6	t	g	-	a	a	a	a	a	c	t	c	t	g	g	c	c	g	c	c	t	c	t	g	g	g	t	c	c	g	a	g	g	c	a	a	c	c	t	a	g	a	c	a	a	g	g	g	t	c	c	c	a	t	a	a	t	t	t	t	c	t	c	c	g	t	g	t	c	c	c	a	t	a
KP702725	6	t	g	-	-	-	-	a	a	c	t	c	t	g	g	c	c	g	c	c	t	c	t	g	g	g	t	c	c	g	a	g	g	c	a	a	c	c	t	a	g	a	c	a	a	g	g	g	t	c	c	c	a	t	a	a	t	t	t	t	c	t	c	c	g	t	g	t	c	c	c	a	t	a
KP771890	9	t	g	-	a	a	a	a	a	c	t	c	t	g	g	c	c	g	c	c	t	c	t	g	g	g	t	c	c	g	g	g	g	c	a	a	c	c	t	a	g	g	c	a	a	g	g	g	t	c	c	c	a	t	a	a	t	t	t	c	c	c	c	c	g	t	g	t	c	c	c	a	t	a
JN704710	9	t	g	-	a	a	a	a	a	c	t	c	t	g	g	c	c	t	c	c	t	c	t	g	g	g	t	c	c	g	g	g	g	c	a	a	c	c	t	a	g	g	c	a	a	g	g	g	t	c	c	c	a	t	a	a	t	t	t	t	c	c	c	c	g	t	g	t	c	c	c	a	t	a
KY037798	9	t	g	-	-	a	a	a	a	c	t	c	t	g	g	c	c	g	c	c	t	c	t	g	g	g	t	c	c	g	g	g	g	c	a	a	c	c	t	a	g	g	c	a	a	g	g	g	t	c	c	c	a	t	a	a	t	t	t	c	c	c	c	c	g	t	g	t	t	c	c	a	t	a
MH709312	9	t	g	-	a	a	a	a	a	c	t	c	t	g	g	c	c	g	c	c	t	c	t	g	g	g	t	c	c	g	g	g	g	c	a	a	c	c	t	a	g	g	c	a	a	g	g	g	t	c	c	c	a	t	a	a	t	t	t	c	c	c	c	c	g	t	g	t	t	c	c	a	t	a
MH709352	9	t	g	-	a	a	a	a	a	c	t	c	t	g	g	c	c	g	c	c	t	c	t	g	g	g	t	c	c	g	g	g	g	c	a	a	c	c	t	a	g	g	c	a	a	g	g	g	t	c	c	c	a	t	a	a	t	t	t	c	c	c	c	c	g	t	g	t	t	c	c	a	t	a
AB097933	Vac	t	g	-	-	-	-	a	a	c	t	c	t	g	g	c	c	g	c	c	t	c	t	g	g	g	t	c	c	g	g	g	g	c	a	a	c	c	t	a	g	a	c	a	a	g	g	g	t	c	c	c	a	t	a	a	t	t	t	t	c	t	c	c	g	t	g	t	c	c	c	a	t	a
JN704698	Vac	t	g	-	a	a	a	a	a	c	t	c	t	g	g	c	c	g	c	c	t	c	t	g	g	g	t	c	c	g	g	g	g	c	a	a	c	c	t	a	g	a	c	a	a	g	g	g	t	c	c	c	a	t	a	a	t	t	t	t	c	t	c	c	g	t	g	t	c	c	c	a	t	a
JN704697	Vac	t	g	-	a	a	a	a	a	c	t	c	t	g	g	c	c	g	c	c	t	c	t	g	g	g	t	c	c	g	g	g	g	c	a	a	c	c	t	a	g	a	c	a	a	g	g	g	t	c	c	c	a	t	a	a	t	t	t	t	c	t	c	c	g	t	g	t	c	c	c	a	t	a
DQ008355	Vac	t	g	-	-	a	a	a	a	c	t	c	t	g	g	c	c	g	c	c	t	c	t	g	g	g	t	c	c	g	g	g	g	c	a	a	c	c	t	a	g	a	c	a	a	g	g	g	t	c	c	c	a	t	a	a	t	t	t	t	c	t	c	c	g	t	g	t	c	c	c	a	t	a
JF306641	Vac	t	g	-	-	-	a	a	a	c	t	c	t	g	g	c	c	g	c	c	t	c	t	g	g	g	t	c	c	g	g	g	g	c	a	a	c	c	t	a	g	a	c	a	a	g	g	g	t	c	c	c	a	t	a	a	t	t	t	t	c	t	c	c	g	t	g	t	c	c	c	a	t	a
AB097932	Vac	t	g	-	-	-	-	a	a	c	t	c	t	g	g	c	c	g	c	c	t	c	t	g	g	g	t	c	c	g	g	g	g	c	a	a	c	c	t	a	g	a	c	a	a	g	g	g	t	c	c	c	a	t	a	a	t	t	t	t	c	t	c	c	g	t	g	t	c	c	c	a	t	a
DQ008354	Vac	t	g	-	a	a	a	a	a	c	t	c	t	g	g	c	c	g	c	c	t	c	t	g	g	g	t	c	c	g	g	g	g	c	a	a	c	c	t	a	g	a	c	a	a	g	g	g	t	c	c	c	a	t	a	a	t	t	t	t	c	t	c	c	g	t	g	t	c	c	c	a	t	a
KU926312	Vac	t	g	-	a	a	a	a	a	c	t	c	t	g	g	c	c	g	c	c	t	c	t	g	g	g	t	c	c	g	g	g	g	c	a	a	c	c	t	a	g	a	c	a	a	g	g	g	t	c	c	c	a	t	a	a	t	t	t	t	c	t	c	c	g	t	g	t	c	c	c	a	t	a
KU926313	Vac	t	g	-	a	a	a	a	a	c	t	c	t	g	g	c	c	g	c	c	t	c	t	g	g	g	t	c	c	g	g	g	g	c	a	a	c	c	t	a	g	a	c	a	a	g	g	g	t	c	c	c	a	t	a	a	t	t	t	t	c	t	c	c	g	t	g	t	c	c	c	a	t	a
KU926314	Vac	t	g	-	-	a	a	a	a	c	t	c	t	g	g	c	c	g	c	c	t	c	t	g	g	g	t	c	c	g	g	g	g	c	a	a	c	c	t	a	g	a	c	a	a	g	g	g	t	c	c	c	a	t	a	a	t	t	t	t	c	t	c	c	g	t	g	t	c	c	c	a	t	a
KF558371	Vac	t	g	-	-	a	a	a	a	c	t	c	t	g	g	c	c	g	c	c	t	c	t	g	g	g	t	c	c	g	g	g	g	c	a	a	c	c	t	a	g	a	c	a	a	g	g	g	t	c	c	c	a	t	a	a	t	t	t	t	c	t	c	c	g	t	g	t	c	c	c	a	t	a
KF558372	Vac	t	g	-	-	a	a	a	a	c	t	c	t	g	g	c	c	g	c	c	t	c	t	g	g	g	t	c	c	g	g	g	g	c	a	a	c	c	t	a	g	a	c	a	a	g	g	g	t	c	c	c	a	t	a	a	t	t	t	t	c	t	c	c	g	t	g	t	c	c	c	a	t	a
KF558373	Vac	t	g	-	-	a	a	a	a	c	t	c	t	g	g	c	c	g	c	c	t	c	t	g	g	g	t	c	c	g	g	g	g	c	a	a	c	c	t	a	g	a	c	a	a	g	g	g	t	c	c	c	a	t	a	a	t	t	c	t	c	t	c	c	g	t	g	t	c	c	c	a	t	a
KF558374	Vac	t	g	-	-	a	a	a	a	c	t	c	t	g	g	c	c	g	c	c	t	c	t	g	g	g	t	c	c	g	g	g	g	c	a	a	c	c	t	a	g	a	c	a	a	g	g	g	t	c	c	c	a	t	a	a	t	t	t	t	c	t	c	c	g	t	g	t	c	c	c	a	t	a
KF558375	Vac	t	g	-	-	-	-	a	a	c	t	c	t	g	g	c	c	g	c	c	t	c	t	g	g	g	t	c	c	g	g	g	g	c	a	a	c	c	t	c	g	a	c	a	a	g	g	g	t	c	c	c	a	t	a	a	t	t	t	t	c	t	c	c	g	t	g	t	c	c	c	a	t	a
KF558376	Vac	t	g	-	-	a	a	a	a	c	t	c	t	g	g	c	c	g	c	c	t	c	t	g	g	g	t	c	c	g	g	g	g	c	a	a	c	c	t	a	g	a	c	a	a	g	g	g	t	c	c	c	a	t	a	a	t	t	t	t	c	t	c	c	g	t	g	t	c	c	c	a	t	a
KF558377	Vac	t	g	-	-	-	-	a	a	c	t	c	t	g	g	c	c	g	c	c	t	c	c	g	g	g	t	c	c	g	g	g	g	c	a	a	c	c	t	a	g	a	c	a	a	g	g	g	t	c	c	c	a	t	a	a	t	t	t	t	c	t	c	c	g	t	g	t	c	c	c	a	t	a
KF558378	Vac	t	g	-	-	-	a	a	a	c	t	c	t	g	g	c	c	g	c	c	t	c	t	g	g	g	t	c	c	g	g	g	g	c	a	a	c	c	t	a	g	a	c	a	a	g	g	g	t	c	c	c	a	t	a	a	t	t	t	t	c	t	c	c	g	t	g	t	c	c	c	a	t	a
KF558379	Vac	t	g	-	-	-	-	a	a	c	t	c	t	g	g	c	c	g	c	c	t	c	t	g	g	g	t	c	c	g	g	g	g	c	a	a	c	c	t	a	g	a	c	a	a	g	g	g	t	c	c	c	a	t	a	a	t	t	t	t	c	t	c	c	g	t	g	t	c	c	c	a	t	a
KF558380	Vac	t	g	-	a	a	a	a	a	c	t	c	t	g	g	c	c	g	c	c	t	c	t	g	g	g	t	c	c	g	g	g	g	c	a	a	c	c	t	a	g	a	c	a	a	g	g	g	t	c	c	c	a	t	a	a	t	t	t	t	c	t	c	c	g	t	g	t	c	c	c	a	t	a
KF558381	Vac	t	g	-	-	a	a	a	a	c	t	c	t	g	g	c	c	g	c	c	t	c	t	g	g	g	t	c	c	g	g	g	g	c	a	a	c	c	t	a	g	a	c	a	a	g	g	g	t	c	c	c	a	t	a	a	t	t	t	t	c	t	c	c	g	t	g	t	c	c	c	a	t	a
KF558382	Vac	t	g	-	-	a	a	a	a	c	t	c	t	g	g	c	c	g	c	c	t	c	t	g	g	g	t	c	c	g	g	g	g	c	a	a	c	c	t	a	g	a	c	a	a	g	g	g	t	c	c	c	a	t	a	a	t	t	t	t	c	t	c	c	g	t	g	t	c	c	c	a	t	a
KF558383	Vac	t	g	-	-	-	-	a	a	c	t	c	t	g	g	c	c	g	c	c	t	c	t	g	g	g	t	c	c	g	g	g	g	c	a	a	c	c	t	a	g	a	c	a	a	g	g	g	t	c	c	c	a	t	a	a	t	t	t	t	c	t	c	c	g	t	g	t	c	c	c	a	t	a
KF558384	Vac	t	g	-	-	-	-	a	a	c	t	c	t	g	g	c	c	g	c	c	t	c	t	g	g	g	t	c	c	g	g	g	g	c	a	a	c	c	t	a	g	a	c	a	a	g	g	g	t	c	c	c	a	t	a	a	t	t	t	t	c	t	c	c	g	t	g	t	c	c	c	a	t	a
KF558385	Vac	t	g	-	-	-	-	a	a	c	t	c	t	g	g	c	c	g	c	c	t	c	t	g	g	g	t	c	c	g	g	g	g	c	a	a	c	c	t	a	g	a	c	a	a	g	g	g	t	c	c	c	a	t	a	a	t	t	t	t	c	t	c	c	g	t	g	t	c	c	c	a	t	a
KF558386	Vac	t	g	-	-	-	a	a	a	c	t	c	t	g	g	c	c	g	c	c	t	c	t	g	g	g	t	c	c	g	g	g	g	c	a	a	c	c	t	a	g	a	c	a	a	g	g	g	t	c	c	c	a	t	a	a	t	t	t	t	c	t	c	c	g	t	g	t	c	c	c	a	t	a
KF558387	Vac	t	g	-	-	-	-	a	a	c	t	c	t	g	g	c	c	g	c	c	t	c	t	g	g	g	t	c	c	g	g	g	g	c	a	a	c	c	t	a	g	a	c	a	a	g	g	g	t	c	c	c	a	t	a	a	t	t	t	t	c	t	c	c	g	t	g	t	c	c	c	a	t	a
KF558388	Vac	t	g	-	-	-	-	a	a	c	t	c	t	g	g	c	c	g	c	c	t	c	t	g	g	g	t	c	c	g	g	g	g	c	a	a	c	c	t	a	g	a	c	a	a	g	g	g	t	c	c	c	a	t	a	a	t	t	t	t	c	t	c	c	g	t	g	t	c	c	c	a	t	a
KF558389	Vac	t	g	-	-	a	a	a	a	c	t	c	t	g	g	c	c	g	c	c	t	c	t	g	g	g	t	c	c	g	g	g	g	c	a	a	c	c	t	a	g	a	c	a	a	g	g	g	t	c	c	c	a	t	a	a	t	t	t	t	c	t	c	c	g	t	g	t	c	c	c	a	t	a
KF558390	Vac	t	g	-	-	-	a	a	a	c	t	c	t	g	g	c	c	g	c	c	t	c	t	g	g	g	t	c	c	g	g	g	g	c	a	a	c	c	t	c	g	a	c	a	a	g	g	g	t	c	c	c	a	t	a	a	t	t	t	t	c	t	c	c	g	t	g	t	c	c	c	a	t	a
KF558391	Vac	t	g	-	-	a	a	a	a	c	t	c	t	g	g	c	c	g	c	c	t	c	t	g	g	g	t	c	c	g	g	g	g	c	a	a	c	c	t	a	g	a	c	a	a	g	g	g	t	c	c	c	a	t	a	a	t	t	t	t	c	t	c	c	g	t	g	t	c	c	c	a	t	a
KF811485	Vac	t	g	-	-	a	a	a	a	c	t	c	t	g	g	c	c	g	c	c	t	c	t	g	g	g	t	c	c	g	g	g	g	c	a	a	c	c	t	c	g	a	c	a	a	g	g	g	t	c	c	c	a	t	a	a	t	t	t	t	c	t	c	c	g	t	g	t	c	c	c	a	t	a
KF853225	Vac	t	g	-	-	-	-	a	a	c	t	c	t	g	g	c	c	g	c	c	t	c	t	g	g	g	t	c	c	g	g	g	g	c	a	a	c	c	t	a	g	a	c	a	a	g	g	g	t	c	c	c	a	t	a	a	t	t	t	t	c	t	c	c	g	t	g	t	c	c	c	a	t	a
KF853226	Vac	t	g	-	-	-	-	a	a	c	t	c	t	g	g	c	c	g	c	c	t	c	t	g	g	g	t	c	c	g	g	g	g	c	a	a	c	c	t	a	g	a	c	a	a	g	g	g	t	c	c	c	a	t	a	a	t	t	t	t	c	t	c	c	g	t	g	t	c	c	c	a	t	a
KF853227	Vac	t	g	-	a	a	a	a	a	c	t	c	t	g	g	c	c	g	c	c	t	c	t	g	g	g	t	c	c	g	g	g	g	c	a	a	c	c	t	a	g	a	c	a	a	g	g	g	t	c	c	c	a	t	a	a	t	t	t	t	c	t	c	c	g	t	g	t	c	c	c	a	t	a
KF853228	Vac	t	g	-	-	-	-	a	a	c	t	c	t	g	g	c	c	g	c	c	t	c	t	g	g	g	t	c	c	g	g	g	g	c	a	a	c	c	t	a	g	a	c	a	a	g	g	g	t	c	c	c	a	t	a	a	t	t	t	t	c	t	c	c	g	t	g	t	c	c	c	a	t	a
KF853229	Vac	t	g	-	a	a	a	a	a	c	t	c	t	g	g	c	c	g	c	c	t	c	t	g	g	g	t	c	c	g	g	g	g	c	a	a	c	c	t	a	g	a	c	a	a	g	g	g	t	c	c	c	a	t	a	a	t	t	t	t	c	t	c	c	g	t	g	t	c	c	c	a	t	a
KF853230	Vac	t	g	-	-	a	a	a	a	c	t	c	t	g	g	c	c	g	c	c	t	c	t	g	g	g	t	c	c	g	g	g	g	c	a	a	c	c	t	a	g	a	c	a	a	g	g	g	t	c	c	c	a	t	a	a	t	t	t	t	c	t	c	c	g	t	g	t	c	c	c	a	t	a
KF853231	Vac	t	g	-	-	-	a	a	a	c	t	c	t	g	g	c	c	g	c	c	t	c	t	g	g	g	t	c	c	g	g	g	g	c	a	a	c	c	t	a	g	a	c	a	a	g	g	g	t	c	c	c	a	t	a	a	t	t	t	t	c	t	c	c	g	t	g	t	c	c	c	a	t	a
KF853232	Vac	t	g	-	-	-	a	a	a	c	t	c	t	g	g	c	c	g	c	c	t	c	t	g	g	g	t	c	c	g	g	g	g	c	a	a	c	c	t	a	g	a	c	a	a	g	g	g	t	c	c	c	a	t	a	a	t	t	t	t	c	t	c	c	g	t	g	t	c	c	c	a	t	a
KF853233	Vac	t	g	-	-	-	-	a	a	c	t	c	t	g	g	c	c	g	c	c	t	c	t	g	g	g	t	c	c	g	g	g	g	c	a	a	c	c	t	a	g	a	c	a	a	g	g	g	t	c	c	c	a	t	a	a	t	t	t	t	c	t	c	c	g	t	g	t	c	c	c	a	t	a
KF853234	Vac	t	g	-	-	-	a	a	a	c	t	c	t	g	g	c	c	g	c	c	t	c	t	g	g	g	t	c	c	g	g	g	g	c	a	a	c	c	t	a	g	a	c	a	a	g	g	g	t	c	c	t	a	t	a	a	t	t	t	t	c	t	c	c	g	t	g	t	c	c	c	a	t	a
KF853235	Vac	t	g	-	-	a	a	a	a	c	t	c	t	g	g	c	c	g	c	c	t	c	t	g	g	g	t	c	c	g	g	g	g	c	a	a	c	c	t	a	g	a	c	a	a	g	g	g	t	c	c	c	a	t	a	a	t	t	t	t	c	t	c	c	g	t	g	t	c	c	c	a	t	a
MF898328	Vac	t	g	-	-	-	a	a	a	c	t	c	t	g	g	c	c	g	c	c	t	c	t	g	g	g	t	c	c	g	g	g	g	c	a	a	c	c	t	a	g	a	c	a	a	g	g	g	t	c	c	c	a	t	a	a	t	t	t	t	c	t	c	c	g	t	g	t	c	c	c	a	t	a
JN704709	VIII	t	g	-	a	a	a	a	a	c	t	c	t	g	g	c	c	g	c	c	t	c	t	g	g	g	t	c	c	g	a	g	t	c	a	a	c	c	t	a	g	a	c	a	a	g	g	g	t	c	c	c	a	t	a	a	t	t	t	t	c	t	c	c	g	t	g	t	c	c	c	a	t	a

Supplementary Table 3: 2880 SN

GB-ID	Clade markers																											2								2										2		5												4													
	Position in Dumas	45459	45505	45531	45640	45642	45717	46008	46264	46305	46315	46356	46363	46398	46477	46609	46672	46687	46703	46735	46822	46909	46978	47002	47026	47050	47074	47149	47162	47354	47511	47542	47622	47686	47697	47754	47940	47981	47996	48011	48050	48276	48635	48675	48724	48754	48825	48990	49005	49011	49034	49036	49038	49128	49134	49209	49269	49386	49389	49442	49535	49602	49742	49776	49908	49987	50081	50123	50157	50349	50382	50519	50555	50559	50593
AY548170	1	a	g	t	g	g	g	a	g	t	a	c	g	c	g	g	a	a	a	g	t	g	c	t	g	c	t	t	a	g	c	t	t	c	t	a	c	g	c	g	t	a	c	t	a	t	g	c	a	c	c	c	c	a	g	a	a	a	c	c	g	c	g	a	a	t	c	t	t	c	a	t	t	g	g
AY548171	1	a	g	t	g	g	g	a	g	t	a	c	g	c	g	g	a	a	a	g	t	g	c	t	g	c	t	t	a	g	c	t	t	c	t	a	c	g	c	g	t	a	c	t	a	t	g	c	a	c	c	c	c	a	g	a	a	a	c	c	g	c	g	a	a	t	c	t	t	c	a	t	t	g	g
DQ479953	1	a	g	t	g	g	g	a	g	t	a	c	g	c	g	g	a	a	a	g	t	g	c	t	g	c	t	t	a	g	c	t	t	c	t	a	c	g	c	g	t	a	c	t	a	t	g	c	a	c	c	c	c	a	g	a	a	a	c	c	g	c	g	a	a	t	c	t	t	c	a	t	t	g	g
DQ479954	1	a	g	t	g	g	g	a	g	t	a	c	g	c	g	g	a	a	a	g	t	g	c	t	g	c	t	t	a	g	c	t	t	c	t	a	c	g	c	g	t	a	c	t	a	t	g	c	a	c	c	c	c	a	g	a	a	a	c	c	g	c	g	a	a	t	c	t	t	c	a	t	t	g	g
DQ479958	1	a	g	t	g	g	g	a	g	t	a	c	g	c	g	g	a	a	a	g	t	g	c	g	g	c	t	t	a	g	c	t	t	c	t	a	c	g	c	g	t	a	c	t	a	t	g	c	a	c	c	c	c	a	g	g	a	a	c	c	g	c	g	a	a	t	c	t	t	c	a	t	c	g	g
DQ479959	1	a	g	t	g	g	g	a	g	t	a	c	g	c	g	g	a	a	a	g	t	g	c	g	g	c	t	t	a	g	c	t	t	c	t	a	c	g	t	g	t	a	c	t	a	t	g	c	a	c	c	c	c	a	g	g	a	a	c	c	g	c	g	a	a	t	c	t	t	c	a	t	c	g	g
DQ479961	1	a	g	t	g	g	g	a	g	t	a	c	g	c	g	g	a	a	a	g	t	g	c	t	g	c	t	t	a	g	c	t	t	c	t	a	c	g	c	g	t	a	c	t	a	t	g	c	a	c	c	c	c	a	g	a	a	a	c	c	g	c	g	a	a	t	c	t	t	c	a	t	t	g	g
DQ479962	1	a	g	t	g	g	g	a	g	t	a	c	g	c	g	g	a	a	a	g	t	g	c	t	g	c	t	t	a	g	c	t	t	c	t	a	c	g	c	g	t	a	c	t	a	t	g	c	a	c	c	c	c	a	g	a	a	a	c	c	g	c	g	a	a	t	c	t	t	c	a	t	t	g	g
DQ479963	1	a	g	t	g	g	g	a	g	t	a	c	g	c	g	g	a	a	a	g	t	g	c	t	g	c	t	t	a	g	c	t	t	c	t	a	c	g	c	g	t	a	c	t	a	t	g	c	a	c	c	c	c	a	g	a	a	a	c	c	g	c	g	a	a	t	c	t	t	c	a	t	t	g	g
DQ674250	1	a	g	t	g	g	g	a	g	t	a	c	g	c	g	g	a	a	a	g	t	g	c	t	g	c	t	t	a	g	c	t	t	c	t	a	c	g	c	g	t	a	c	t	a	t	g	c	a	c	c	c	c	a	g	a	a	a	c	c	g	c	g	a	a	t	c	t	t	c	a	t	t	g	g
KX262864	1	a	g	t	g	g	g	a	g	t	a	c	g	c	g	g	a	a	a	g	t	g	c	t	g	c	t	t	a	g	c	t	t	c	c	a	c	g	c	g	t	a	c	t	a	t	g	c	a	c	c	c	c	a	g	a	a	a	c	c	g	c	g	a	a	t	c	t	t	c	a	t	t	g	g
KX262865	1	a	g	t	g	g	g	a	g	t	a	c	g	c	g	g	a	a	a	g	t	g	c	t	g	c	t	t	a	g	c	t	t	c	t	a	c	g	c	g	t	a	c	t	a	t	g	c	a	c	c	c	c	a	g	a	a	a	c	c	g	c	g	a	a	t	c	t	t	c	a	t	t	g	g
KX262866	1	a	g	t	g	g	g	a	g	t	a	c	g	c	g	g	a	a	a	g	t	g	c	t	g	c	t	t	a	g	c	t	t	c	t	a	c	g	c	g	t	a	c	t	a	t	g	c	a	c	c	c	c	a	g	a	a	a	c	c	g	c	g	a	a	t	c	t	t	c	a	t	t	g	g
EU154348	1	a	g	t	g	g	g	a	g	t	a	c	g	c	g	g	a	a	a	g	t	g	c	t	g	c	t	t	a	g	c	t	t	c	t	a	c	g	c	g	t	a	c	t	a	t	g	c	a	c	c	c	c	a	g	a	a	a	c	c	g	c	g	a	a	t	c	t	t	c	a	t	t	g	g
JN704690	1	a	g	t	g	g	g	a	g	t	a	c	g	c	g	g	a	a	a	g	t	g	c	t	g	c	t	t	a	g	c	t	t	c	t	a	c	g	c	g	t	a	c	t	a	t	g	c	a	c	c	c	c	a	g	a	a	a	c	c	g	c	g	a	a	t	c	t	t	c	a	t	t	g	g
JN704691	1	a	g	t	g	g	g	a	g	t	a	c	g	c	g	g	a	a	a	g	t	g	c	t	g	c	t	t	a	g	c	t	t	c	t	a	c	g	c	g	t	a	c	t	a	t	g	c	a	c	c	c	c	a	g	a	a	a	c	c	g	c	g	a	a	t	c	t	t	c	a	t	t	g	g
JN704692	1	a	g	t	g	g	g	a	g	t	a	c	g	c	g	g	a	a	a	g	t	g	c	t	g	c	t	t	a	g	c	t	t	c	t	a	c	g	c	g	t	a	c	t	a	t	g	c	a	c	c	c	c	a	g	a	a	a	c	c	g	c	g	a	a	t	c	t	t	c	a	t	t	g	g
JN704693	1	a	g	t	g	g	g	a	g	t	a	c	g	c	g	g	a	a	a	g	t	g	c	t	g	c	t	t	a	g	c	t	t	c	t	a	c	g	c	g	t	a	c	t	a	t	g	c	a	c	c	c	c	a	g	a	a	a	c	c	g	c	g	a	a	t	c	t	t	c	a	t	t	g	g
JN704694	1	a	g	t	a	g	g	a	g	t	a	c	g	c	g	g	a	a	a	g	t	g	c	t	g	c	t	t	a	g	c	t	t	c	t	a	c	g	c	g	t	a	c	t	a	t	g	c	a	c	c	c	c	a	g	a	a	a	c	c	g	c	g	a	a	t	c	t	t	c	a	t	t	g	g
JN704695	1	a	g	t	g	g	g	a	g	t	a	c	g	c	g	g	a	a	a	g	t	g	c	t	g	c	t	t	a	g	c	t	t	c	t	a	c	g	c	g	t	a	c	t	a	t	g	c	a	c	c	c	c	a	g	a	a	a	c	c	g	c	g	a	a	t	c	t	t	c	a	t	t	g	g
JN704696	1	a	g	t	g	g	g	a	g	t	a	c	g	c	g	g	a	a	a	g	t	g	c	t	g	c	t	t	a	g	t	t	t	c	t	a	c	g	c	g	t	a	c	t	a	t	g	c	a	c	c	c	c	a	g	a	a	a	c	c	g	c	g	a	a	t	c	t	t	c	a	t	t	g	g
NC001348	1	a	g	t	g	g	g	a	g	t	a	c	g	c	g	g	a	a	a	g	t	g	c	t	g	c	t	t	a	g	c	t	t	c	t	a	c	g	c	g	t	a	c	t	a	t	g	c	a	c	c	c	c	a	g	a	a	a	c	c	g	c	g	a	a	t	c	t	t	c	a	t	t	g	g
KP771892	1	a	g	t	g	g	g	a	g	t	a	c	g	c	g	g	a	a	a	g	t	g	c	t	g	c	t	t	a	g	c	t	t	c	t	a	c	g	c	g	t	a	c	t	a	t	g	c	a	c	c	c	c	a	g	a	a	a	c	c	g	c	g	a	a	t	c	t	t	c	a	t	t	g	g
KP771894	1	a	g	t	g	g	g	a	g	t	a	c	g	c	g	g	a	a	a	g	t	g	c	t	g	c	t	t	a	g	c	t	t	c	t	a	c	g	c	g	t	a	c	t	a	t	g	c	a	c	c	c	c	a	g	a	a	a	c	c	g	c	g	a	a	t	c	t	t	c	a	t	t	g	g
KP771900	1	a	g	t	g	g	g	a	g	t	a	c	g	c	g	g	a	a	a	g	t	g	c	t	g	c	t	t	a	g	c	t	t	c	t	a	c	g	c	g	t	a	c	t	a	t	g	c	a	c	c	c	c	a	g	a	a	a	c	c	g	c	g	a	a	t	c	t	t	c	a	t	t	g	g
KP771901	1	a	g	t	g	g	g	a	g	t	a	c	g	c	g	g	a	a	a	g	t	g	c	t	g	c	t	t	a	g	c	t	t	c	t	a	c	g	c	g	t	a	c	t	a	t	g	c	a	c	c	c	c	a	g	a	a	a	c	c	g	c	g	a	a	t	c	t	t	c	a	t	t	g	g
KP771902	1	a	g	t	g	g	g	a	g	t	a	c	g	c	g	g	a	a	a	g	t	g	c	t	g	c	t	t	a	g	c	t	t	c	t	a	c	g	c	g	t	a	c	t	a	t	g	c	a	c	c	c	c	a	g	a	a	a	c	c	g	c	g	a	a	t	c	t	t	c	a	t	t	g	g
KP771908	1	a	g	t	g	g	g	a	g	t	a	c	g	c	g	g	a	a	a	g	t	g	c	t	g	c	t	t	a	g	c	t	t	c	t	a	c	g	c	g	t	a	c	t	a	t	g	c	a	c	c	c	c	a	g	a	a	a	c	c	g	c	g	a	a	t	c	t	t	c	a	t	t	g	g
KP771912	1	a	g	t	g	g	g	a	g	t	a	c	g	c	g	g	a	a	a	g	t	g	c	t	g	c	t	t	a	g	c	t	t	c	c	a	c	g	c	g	t	a	c	t	a	t	g	c	a	c	c	c	c	a	g	a	a	a	c	c	g	c	g	a	a	t	c	t	t	c	a	t	t	g	g
KP771913	1	a	g	t	g	g	g	a	g	t	a	c	g	c	g	g	a	a	a	g	t	g	c	t	g	c	t	t	a	g	c	t	t	c	c	a	c	g	c	g	t	a	c	t	a	t	g	c	a	c	c	c	c	a	g	a	a	a	c	c	g	c	g	a	a	t	c	t	t	c	a	t	t	g	g
KP771917	1	a	g	t	g	g	g	a	g	t	a	c	g	c	g	g	a	a	a	g	t	g	c	t	g	c	t	t	a	g	c	t	t	c	t	a	c	g	c	g	t	a	c	t	a	g	g	c	a	c	c	c	c	a	g	a	a	a	c	c	g	c	g	a	a	t	c	t	t	c	a	t	t	g	g
KP771918	1	a	g	t	g	g	g	a	g	t	a	c	g	c	g	g	a	a	a	g	t	g	c	t	g	c	t	t	a	g	c	t	t	c	t	a	c	g	c	g	t	a	c	t	a	t	g	c	a	c	c	c	c	a	g	a	a	a	c	c	g	c	g	a	a	t	c	t	t	c	a	t	t	g	g
KP771920	1	a	g	t	g	g	g	a	g	t	a	c	g	c	g	g	a	a	a	g	t	g	c	t	g	c	t	t	a	g	c	t	t	c	t	a	c	g	c	g	t	a	c	t	a	t	g	c	a	c	c	c	c	a	g	a	a	a	c	c	g	c	g	a	a	t	c	t	t	c	a	t	t	g	g
MH709361	1	a	g	t	g	g	g	a	g	t	a	c	g	c	g	g	a	a	a	g	t	g	c	t	g	c	t	t	a	g	c	t	t	c	t	a	c	g	c	g	t	a	c	t	a	t	g	c	a	c	c	c	c	a	g	a	a	a	c	c	g	c	g	a	a	t	c	t	t	c	a	t	t	g	g
KU926318	2	a	a	t	g	g	g	a	g	t	a	c	g	c	g	g	a	a	g	g	t	g	c	t	g	c	t	t	g	g	c	t	t	c	t	a	t	g	c	g	c	a	c	t	a	t	a	c	a	c	c	c	c	a	g	a	a	a	c	c	a	c	g	a	a	t	a	t	t	c	a	t	t	g	g
KU926319	2	a	g	t	g	g	g	a	g	t	a	c	g	c	g	g	a	a	a	g	t	g	c	t	g	c	t	t	g	g	c	t	t	c	t	a	t	g	c	g	c	a	c	t	a	t	a	c	a	c	c	c	c	a	g	a	a	a	c	c	a	c	g	a	a	t	a	t	t	c	a	t	t	g	g
KU926320	2	a	g	t	g	g	g	a	g	t	a	c	g	c	g	g	g	a	a	g	t	g	c	t	g	c	t	t	g	g	c	t	t	c	t	g	t	g	c	g	c	a	c	t	a	t	a	c	a	c	c	c	c	a	g	a	a	a	c	c	a	c	g	a	c	t	a	t	t	c	a	t	t	g	g
KU926321	2	a	g	t	g	g	g	a	g	t	a	c	g	c	g	g	a	a	a	g	t	g	c	t	g	c	t	t	g	g	c	t	t	c	t	a	t	g	c	g	c	a	c	t	a	t	a	c	a	c	c	c	c	a	g	a	a	a	c	c	a	c	g	a	a	t	a	t	t	c	a	t	t	g	a
KU926322	2	a	g	t	g	g	g	a	g	t	a	c	g	c	g	g	a	a	a	g	t	g	c	t	g	c	t	t	g	g	c	t	t	c	t	a	t	g	c	g	c	a	c	t	a	t	a	c	a	c	c	c	c	a	g	a	a	a	c	c	a	c	g	a	a	t	a	t	t	c	a	t	t	g	a
KC847290	2	a	g	t	g	g	g	a	g	t	a	c	g	c	g	g	a	a	a	g	t	g	c	t	g	c	t	t	g	g	c	g	t	c	t	a	t	g	c	g	c	a	c	t	a	t	a	c	a	c	c	c	c	a	g	a	a	a	c	c	a	c	g	a	a	t	a	t	t	c	a	t	t	g	g
KJ767491	2	a	a	t	g	g	g	a	g	t	a	c	g	c	g	g	a	a	a	g	t	g	c	t	g	c	t	t	g	g	c	t	t	c	t	a	t	g	c	g	c	a	c	t	a	t	a	c	a	c	c	c	c	a	g	a	a	a	c	c	a	c	g	a	a	t	a	t	t	c	a	t	t	g	g
KJ767492	2	a	g	t	g	g	g	a	g	t	a	c	g	c	g	g	a	a	a	g	t	g	c	t	g	c	t	t	g	g	c	t	t	c	t	a	t	g	c	g	c	a	c	t	a	t	a	c	a	c	c	c	c	a	g	a	a	a	c	c	a	c	g	a	a	t	a	t	t	c	a	t	t	g	g
KJ808816	2	a	g	t	g	g	g	a	g	t	a	c	g	c	g	g	a	a	a	g	t	g	c	t	g	c	t	t	g	g	c	t	t	c	t	a	t	g	c	g	c	a	c	t	a	t	a	c	a	c	c	c	c	a	g	a	a	a	c	c	a	c	g	a	a	t	a	t	t	c	a	t	t	g	a
KP771925	2	a	g	t	g	g	g	a	g	t	a	c	g	c	g	g	a	a	a	g	t	g	c	t	g	c	t	t	g	g	c	t	t	c	t	a	t	g	c	g	c	a	c	t	a	t	a	t	a	c	c	c	c	a	g	a	a	a	c	c	a	c	g	a	a	t	a	t	t	c	a	t	t	g	g
KU926315	2	a	a	t	g	g	g	a	g	t	a	c	g	c	g	g	a	a	a	g	t	g	c	t	g	c	t	t	g	g	c	t	t	c	t	a	t	g	c	g	c	a	c	t	a	t	a	c	a	c	c	c	c	a	g	a	a	a	c	c	a	c	g	a	a	t	a	t	t	c	a	t	t	g	g
KU926316	2	a	a	t	g	g	g	a	g	t	a	c	g	c	g	g	a	a	a	g	t	g	c	t	g	c	t	t	g	g	c	t	t	c	t	a	t	g	c	g	c	a	c	t	a	t	a	c	a	c	c	c	c	a	g	a	a	a	c	c	a	c	g	a	a	t	a	t	t	c	a	t	t	g	g
KU926317	2	a	a	t	g	g	g	a	g	t	a	c	g	c	g	g	a	a	a	g	t	g	c	t	g	c	t	t	g	g	c	t	t	c	t	a	t	g	c	g	c	a	c	t	a	t	a	c	a	c	c	c	c	a	g	a	a	a	c	c	a	c	g	a	a	t	a	t	t	c	a	t	t	g	g
KY037796	2	a	g	t	g	g	g	a	g	t	a	c	g	c	g	g	a	a	a	g	t	g	c	t	g	c	t	t	g	g	c	t	t	c	t	a	t	g	c	g	c	a	c	t	a	t	a	c	a	c	c	c	c	a	g	a	a	a	c	c	a	c	g	a	a	t	a	t	t	c	a	t	t	g	g
MF004348	2	a	g	t	g	g	g	a	g	t	a	c	g	c	g	g	a	a	a	g	t	g	c	t	g	c	t	t	g	g	c	t	t	c	t	a	t	g	c	g	c	a	c	t	a	t	a	c	a	c	c	c	c	a	g	a	a	a	c	c	a	c	g	a	a	t	a	t	t	c	a	t	t	g	g
MH709309	2	a	g	t	g	g	g	a	g	t	a	c	g	c	g	g	a	a	a	g	t	g	c	t	g	c	g	t	g	g	c	t	t	c	t	a	t	g	c	g	c	a	c	t	a	t	a	c	a	c	c	c	c	a	g	a	a	a	c	c	a	c	g	a	a	t	a	t	t	c	a	t	t	g	g
MH709310	2	a	g	t	g	g	g	a	g	t	a	c	g	c	g	g	a	a	a	g	t	g	c	t	g	c	t	t	g	g	c	t	t	c	t	a	t	g	c	g	c	a	c	t	a	t	a	c	a	c	c	c	c	a	g	a	a	a	c	c	a	c	g	a	a	t	a	t	t	c	a	t	t	g	g
MH709311	2	a	g	t	g	g	g	a	g	t	a	c	g	c	g	g	a	a	a	g	t	g	c	t	g	c	t	t	g	g	c	t	t	c	t	a	t	g	c	t	c	a	c	t	a	t	a	c	a	c	c	c	c	a	g	a	a	a	c	a	g	c	g	a	a	t	a	t	t	c	a	t	t	g	g
MH709313	2	a	g	t	g	g	g	a	g	t	a	c	g	c	g	g	a	a	a	g	t	g	c	t	g	c	t	t	g	g	c	t	t	c	t	a	t	g	c	g	c	a	c	t	a	t	a	c	a	c	c	c	c	a	g	a	a	a	c	c	a	c	g	a	a	t	a	t	t	c	a	t	t	g	g
MH709314	2	a	g	t	g	g	g	a	g	t	a	c	g	c	g	g	a	a	a	g	t	g	c	t	g	c	t	t	g	g	c	t	t	c	t	a	t	g	c	g	c	a	c	t	a	t	a	c	a	c	c	c	c	a	g	a	a	a	c	c	a	c	g	a	a	t	a	t	t	c	a	t	t	g	g
MH709315	2	a	g	t	g	g	g	a	g	t	a	c	g	c	g	g	a	a	a	g	t	g	c	t	g	c	t	t	g	g	c	t	t	c	t	a	t	g	c	g	c	a	c	t	a	t	a	c	a	c	c	c	c	a	g	a	a	a	c	c	a	c	g	a	a	t	a	t	t	c	a	t	t	g	g
MH709319	2	a	g	t	g	g	g	a	g	t	a	c	g	c	g	g	a	a	a	g	t	g	c	t	g	c	t	t	g	g	c	t	t	c	t	a	t	g	c	g	c	a	c	t	a	t	a	c	a	c	c	c	c	a	g	a	a	a	c	c	a	c	g	a	a	t	a	t	t	c	a	t	t	g	g
MH709320	2	a	g	t	g	g	g	a	g	t	a	c	g	c	g	g	a	a	a	g	t	g	c	t	g	c	t	t	g	g	c	t	t	c	t	a	t	g	c	g	c	a	c	t	a	t	a	c	a	c	c	c	c	a	g	a	a	a	c	c	a	c	g	a	a	t	a	t	t	c	a	t	t	g	g
MH709329	2	a	g	t	g	g	g	a	g	t	a	c	g	c	g	g	a	a	a	g	t	g	c	t	g	c	t	t	g	g	c	t	t	c	t	a	t	g	c	g	c	a	c	t	a	t	a	c	a	c	c	c	c	a	g	a	a	a	c	c	a	c	g	a	a	t	a	t	t	c	a	t	t	g	g
MH709331	2	a	g	t	g	g	g	a	g	t	a	c	g	c	g	g	a	a	a	g	t	g	c	t	g	c	t	t	g	g	c	t	t	c	t	a	t	g	c	g	c	a	c	t	a	t	a	c	a	c	c	c	c	a	g	a	a	a	c	c	a	c	g	a	a	t	a	t	t	c	a	t	t	g	g
MH709333	2	a	g	t	g	g	g	a	g	t	a	c	g	c	g	g	a	a	a	g	t	g	c	t	g	c	t	t	g	g	c	t	t	c	t	a	t	g	c	g	c	a	c	t	a	t	a	c	a	c	c	c	c	a	g	a	a	a	c	c	a	c	g	a	a	t	a	t	t	c	a	t	t	g	g
MH709337	2	a	g	t	g	g	g	a	g	t	a	c	g	c	g	g	a	a	a	g	t	g	c	t	g	c	t	t	g	g	c	t	t	c	t	a	t	g	c	g	c	a	c	t	a	t	a	c	a	c	c	c	c	a	g	a	a	a	c	c	a	c	g	a	a	t	a	t	t	c	a	t	t	g	g
MH709339	2	a	a	t	g	g	g	a	g	t	a	c	g	c	g	g	a	a	a	g	t	g	c	t	g	c	t	t	g	g	c	t	t	c	t	a	t	g	c	g	c	a	c	t	a	t	a	c	a	c	t	t	t	a	g	a	a	a	c	c	a	c	g	a	a	t	a	t	t	c	a	t	t	g	g
MH709344	2	a	g	t	g	g	g	a	g	t	a	c	g	c	g	g	a	a	a	g	t	g	c	t	g	c	t	t	g	g	c	t	t	c	t	a	t	g	c	g	c	a	c	t	a	t	a	c	a	c	c	c	c	a	g	a	g	a	c	c	a	c	g	a	a	t	a	t	t	c	a	t	t	g	g
MH709348	2	a	g	t	g	g	g	a	g	t	a	c	g	c	g	g	a	a	a	g	t	g	c	t	g	c	t	t	g	g	c	t	t	c	t	a	t	g	c	g	c	a	c	t	a	t	a	c	a	c	c	c	c	a	g	a	a	a	c	c	a	c	g	a	a	t	a	t	t	c	a	t	t	g	g
MH709355	2	a	g	t	g	g	g	a	g	t	a	c	g	c	g	g	a	a	a	g	t	g	c	t	g	c	t	t	g	g	c	t	t	c	t	a	t	g	c	g	c	a	c	t	a	t	a	c	a	c	c	c	c	a	g	a	a	a	c	c	a	c	g	a	a	t	a	t	t	c	a	t	t	g	g
MH709359	2	a	g	c	g	g	g	a	g	t	a	c	g	c	g	g	a	a	a	g	t	g	c	t	g	c	t	t	g	g	c	t	t	c	t	a	t	g	c	g	c	a	c	t	a	t	a	c	a	c	c	c	c	a	g	a	a	a	c	c	a	c	g	a	a	t	a	t	t	c	a	t	t	g	g
MH709363	2	a	g	t	g	g	g	a	g	t	a	c	g	c	g	g	a	a	a	g	t	g	c	t	g	c	t	t	g	g	c	t	t	c	t	a	t	g	c	g	c	a	c	t	a	t	a	c	a	c	c	c	c	a	g	a	a	a	c	c	a	c	g	a	a	t	a	t	t	c	a	t	t	g	g
MH709366	2	a	g	c	g	g	g	a	g	t	a	c	g	c	g	g	a	a	a	g	t	g	c	t	g	c	t	t	g	g	c	t	t	c	t	a	t	g	c	g	c	a	c	t	a	t	a	c	a	c	c	c	c	a	g	a	a	a	c	c	a	c	g	a	a	t	a	t	t	c	a	t	t	g	g
MH709376	2	a	g	t	g	g	g	a	a	t	a	c	g	c	g	g	a	a	a	g	t	g	c	t	g	c	t	t	g	g	c	t	t	c	t	a	t	g	c	g	c	a	c	t	a	t	a	c	a	c	c	c	c	a	g	a	a	a	c	c	a	c	g	a	a	t	a	t	t	c	a	t	t	g	g
MH709377	2	a	g	t	g	g	g	a	g	t	a	c	g	c	g	g	a	a	a	g	t	g	c	t	g	c	t	t	g	g	c	t	t	c	t	a	t	g	c	g	c	a	c	t	a	t	a	c	a	c	c	c	c	a	g	a	a	a	c	c	a	c	g	a	a	t	a	t	t	c	a	t	t	g	g
**MW316406**	2	a	g	t	g	g	g	a	g	t	a	c	g	c	g	g	a	a	a	g	t	g	c	t	g	c	t	t	g	g	c	t	t	c	t	a	t	g	c	g	c	a	c	t	a	t	a	c	a	c	c	c	c	a	g	a	a	a	c	c	a	c	g	a	a	t	a	t	t	c	a	t	t	g	g
AJ871403	3	a	g	t	g	g	g	a	g	t	a	c	g	c	g	g	a	a	a	g	t	g	c	t	g	c	t	t	a	g	c	t	t	c	t	a	c	g	c	g	t	a	c	t	a	t	g	c	a	c	c	c	c	a	g	a	a	a	c	c	g	c	g	a	a	t	c	t	t	c	a	t	t	g	g
DQ479955	3	a	g	t	g	g	g	a	g	t	a	c	g	c	g	g	a	a	a	g	t	g	c	t	g	c	t	t	a	g	c	t	t	c	t	a	c	g	c	g	t	a	c	t	a	t	g	c	a	c	c	c	c	a	g	a	a	a	c	c	g	c	g	a	a	t	c	t	t	c	a	t	t	g	g
DQ479956	3	a	g	t	g	g	g	a	g	t	a	c	g	c	g	g	a	a	a	g	t	g	c	t	g	c	t	t	a	g	c	t	t	c	t	a	c	g	c	g	t	a	c	t	a	t	g	c	a	c	c	c	c	a	g	a	a	a	t	c	g	c	g	a	a	t	c	t	t	c	a	t	t	g	g
DQ479957	3	a	g	t	g	g	g	a	g	t	a	c	g	c	g	g	a	a	a	g	t	g	c	t	g	c	t	g	a	g	c	t	t	c	t	a	c	g	c	g	t	a	c	t	a	t	g	c	a	c	c	c	c	a	g	a	a	a	c	c	g	c	g	a	a	t	c	t	t	c	a	t	t	g	g
JN704699	3	a	g	t	g	g	g	a	g	t	a	c	g	c	g	g	a	a	a	g	t	g	c	t	g	c	t	t	a	g	c	t	t	c	t	a	c	g	c	g	t	a	c	c	a	t	g	c	a	c	c	c	c	a	g	a	a	a	c	c	g	c	g	a	a	t	c	t	t	c	a	t	t	g	g
JN704700	3	a	g	t	g	g	g	a	g	t	a	c	g	c	g	g	a	a	a	g	c	g	c	t	g	c	t	t	a	g	c	t	t	t	t	a	c	g	c	g	t	a	c	t	a	t	g	c	a	c	c	c	c	a	g	a	a	a	c	c	g	c	g	a	a	t	c	t	t	c	a	t	t	g	g
JN704701	3	a	g	t	g	g	g	a	g	t	a	c	g	c	g	g	a	a	a	g	t	g	c	t	g	c	t	t	a	g	c	t	t	c	t	a	c	g	c	g	t	a	c	c	a	t	g	c	a	c	c	c	c	a	g	a	a	a	c	c	g	c	g	a	a	t	c	t	t	c	a	t	t	g	g
JN704702	3	a	g	t	g	g	g	a	g	t	a	c	g	c	g	g	a	a	a	g	t	g	c	t	g	c	t	t	a	g	c	t	t	c	t	a	c	g	c	g	t	a	c	t	a	t	g	c	a	c	c	c	c	a	g	a	a	a	c	c	g	c	g	a	g	t	c	t	t	c	a	t	t	g	g
JN704703	3	a	g	t	g	g	g	a	g	t	a	c	g	c	g	g	a	a	a	g	t	g	c	t	g	c	t	t	a	g	c	t	t	c	t	a	c	a	c	g	t	a	c	t	a	t	g	c	a	c	c	c	c	a	g	a	a	a	c	c	g	c	g	a	a	t	c	t	t	c	a	t	t	g	g
KP771891	3	a	g	t	g	g	g	a	g	t	a	c	g	c	g	g	a	a	a	g	t	g	c	t	g	c	t	t	a	g	c	t	t	c	t	a	c	g	c	g	t	a	c	c	a	t	g	c	a	c	c	c	c	a	g	a	a	a	c	c	g	c	g	a	a	t	c	t	t	c	a	t	t	g	g
KP771893	3	a	g	t	g	g	g	a	g	t	a	c	g	c	g	g	a	a	a	g	t	g	c	t	g	c	t	t	a	g	c	t	t	c	t	a	c	g	c	g	t	a	c	c	a	t	g	c	a	c	c	c	c	a	g	a	a	a	c	c	g	c	g	a	a	t	c	t	t	c	a	t	t	g	g
KP771895	3	a	g	t	g	g	g	a	g	t	a	c	g	c	g	g	a	a	a	g	t	g	c	t	g	c	t	t	a	g	c	t	t	c	t	a	c	g	c	g	t	a	c	t	a	t	g	c	a	c	c	c	c	a	g	a	a	a	c	c	g	c	g	a	a	t	c	t	t	c	a	t	t	g	g
KP771896	3	a	g	t	g	g	g	a	g	t	a	c	g	c	g	g	a	a	a	g	t	g	c	t	g	c	t	t	a	g	c	t	t	c	t	a	c	g	c	g	t	a	c	t	a	t	g	c	a	c	c	c	c	a	g	a	a	a	c	c	g	c	g	a	a	t	c	t	t	c	a	t	t	g	g
KP771897	3	a	g	t	g	g	g	a	g	c	a	c	g	c	g	g	a	a	a	g	t	g	c	t	g	c	t	t	a	g	c	t	t	c	t	a	c	g	c	g	t	a	c	t	a	t	g	c	a	t	c	c	c	a	g	a	a	a	c	c	g	c	g	a	a	t	c	t	t	c	a	t	t	g	g
KP771899	3	a	g	t	g	g	g	a	g	t	a	c	g	c	g	g	a	a	a	g	t	g	c	t	g	c	t	t	a	g	c	t	t	c	t	a	c	g	c	g	t	a	c	t	a	t	g	c	a	c	c	c	c	a	g	a	a	a	c	c	g	c	g	a	a	t	c	t	t	c	a	t	t	g	g
KP771903	3	a	g	t	g	g	g	a	g	t	a	c	g	c	g	g	a	a	a	g	t	g	c	t	g	c	t	t	a	g	c	t	t	c	t	a	c	g	c	g	t	a	c	c	a	t	g	c	a	c	c	c	c	a	g	a	a	a	c	c	g	c	g	a	a	t	c	t	t	c	a	t	t	g	g
KP771907	3	a	g	t	g	g	g	a	g	t	a	c	g	c	g	g	a	a	a	g	t	g	c	t	g	c	t	t	a	g	c	t	t	c	t	a	c	g	c	g	t	a	c	t	a	t	g	c	a	c	c	c	c	a	g	a	a	a	c	c	g	c	g	a	a	t	c	t	t	c	a	t	t	g	g
KP771910	3	a	g	t	g	g	g	a	g	t	a	c	g	c	g	g	a	a	a	g	t	g	c	t	g	c	t	t	a	g	c	t	t	c	t	a	c	g	c	g	t	a	c	c	a	t	g	c	a	c	c	c	c	a	g	a	a	a	c	c	g	c	g	a	a	t	c	t	t	c	a	t	t	g	g
KP771915	3	a	g	t	g	g	g	a	g	t	a	c	g	c	a	g	a	a	a	g	t	g	c	t	g	c	t	t	a	g	c	t	t	c	t	a	c	g	c	g	t	a	c	t	a	t	g	c	a	c	c	c	c	a	g	a	a	a	c	c	g	c	g	a	a	t	c	t	t	c	a	t	t	g	g
KP771916	3	a	g	t	g	g	g	a	g	t	a	c	g	c	g	g	a	a	a	g	t	g	c	t	g	c	t	t	a	g	c	t	t	c	t	a	c	g	c	g	t	a	c	t	a	t	g	c	a	c	c	c	c	a	g	a	a	a	t	c	g	c	g	a	a	t	c	t	t	c	a	t	t	g	g
KP771921	3	a	g	t	g	g	g	a	g	t	a	c	g	c	g	g	a	a	a	g	t	g	c	t	g	c	t	t	a	g	c	t	t	c	t	a	c	g	c	g	t	a	c	c	a	t	g	c	a	c	c	c	c	a	g	a	a	a	c	c	g	c	g	a	a	t	c	t	t	c	a	t	t	g	g
KP771923	3	a	g	t	g	g	g	a	g	t	a	c	g	c	g	g	a	g	a	g	t	g	c	t	g	c	t	t	a	g	c	t	t	c	t	a	c	g	c	g	t	a	c	c	a	t	g	c	a	c	c	c	c	a	g	a	a	a	c	c	g	c	g	a	a	t	c	t	t	c	a	t	t	g	g
KP771889	3	a	g	t	g	g	g	a	g	t	a	c	g	c	a	g	a	a	a	g	t	g	c	t	g	c	t	t	a	g	c	t	t	c	t	a	c	g	c	g	t	a	c	t	a	t	g	c	a	c	c	c	c	a	g	a	a	a	c	c	g	c	g	a	a	t	c	t	t	c	a	t	t	g	g
KU926311	3	a	g	t	g	g	g	a	g	t	a	c	g	c	g	g	a	a	a	g	t	a	c	t	g	c	t	t	a	g	c	t	t	c	t	a	c	g	c	g	t	a	c	t	a	t	g	c	a	c	c	c	c	a	g	a	a	a	t	c	g	c	g	a	a	t	c	t	t	c	a	t	t	g	g
JQ911599	3	a	g	t	g	g	g	a	g	t	a	c	g	c	g	g	a	a	a	g	t	g	c	t	g	c	t	t	a	g	c	t	t	c	t	a	c	g	c	g	t	a	c	t	a	t	g	c	a	c	c	c	c	a	g	a	a	a	c	c	g	c	g	a	a	t	c	t	t	c	a	t	t	g	g
MH709317	3	a	g	t	g	g	g	a	g	t	a	c	g	c	g	g	a	a	a	g	t	g	c	t	g	c	t	t	a	g	c	t	t	c	t	a	c	g	c	g	c	g	c	t	a	t	g	c	a	c	c	c	c	a	g	a	a	a	c	c	g	c	g	a	a	t	c	t	t	c	a	t	t	g	g
MH709346	3	a	g	t	g	g	g	a	g	t	a	c	g	c	g	g	a	a	a	g	t	g	c	t	g	c	t	t	a	g	c	t	t	c	t	a	c	g	c	g	c	g	c	t	a	t	g	c	a	c	c	c	c	a	g	a	a	a	c	c	g	c	g	a	a	t	c	t	t	c	a	t	t	g	g
MH709375	3	a	g	t	g	g	g	a	g	t	a	c	a	c	g	g	a	a	a	g	t	g	c	t	g	c	t	t	a	g	c	t	t	c	t	a	c	g	c	g	c	g	c	t	a	t	g	c	a	c	c	c	c	a	g	a	a	a	c	c	g	c	g	a	a	t	c	t	t	c	a	t	t	g	g
DQ452050	4	a	g	t	g	g	g	a	g	t	a	c	g	c	g	g	a	a	a	g	t	g	c	t	g	c	t	t	a	g	c	t	t	c	t	a	c	g	c	g	c	a	c	t	a	t	g	c	a	c	c	c	c	a	g	a	a	a	c	c	a	t	g	a	a	t	c	t	t	c	a	t	t	g	g
DQ479960	4	a	g	t	g	g	g	a	g	t	a	c	g	c	g	g	a	a	a	g	t	g	c	t	g	c	t	t	a	g	c	t	t	c	t	a	c	g	c	g	c	a	c	t	a	t	g	c	a	c	c	c	c	a	g	a	a	a	c	c	a	t	g	a	a	t	c	t	t	c	a	t	t	g	g
KP771898	4	a	g	t	g	g	g	a	g	t	a	c	g	c	g	g	a	a	a	g	t	g	c	t	g	c	t	t	a	g	c	t	t	c	t	a	c	g	c	g	c	a	c	t	a	t	g	c	a	c	c	c	c	a	g	a	a	a	c	c	a	t	g	a	a	t	c	t	t	c	a	t	t	g	g
MH709316	4	a	g	t	g	g	g	a	g	t	a	c	g	c	g	g	a	a	a	g	t	g	c	t	g	c	t	t	a	g	c	t	t	c	t	a	c	g	c	g	c	a	c	t	a	t	g	c	a	c	c	c	c	a	g	a	a	a	c	c	a	t	g	a	a	t	c	t	t	c	a	t	t	g	g
MH709318	4	a	g	t	g	g	g	a	g	t	a	c	g	c	g	g	a	a	a	g	t	g	c	t	g	c	t	t	a	g	c	t	t	c	t	a	c	g	c	g	c	a	c	t	g	t	g	c	a	c	c	c	c	a	g	a	a	a	c	c	a	t	g	a	a	t	c	t	t	c	a	t	t	g	g
MH709322	4	a	g	t	g	g	g	a	g	t	a	c	g	c	g	g	a	a	a	g	t	g	c	t	g	c	t	t	a	g	c	t	t	c	t	a	c	g	c	g	c	a	c	t	g	t	g	c	a	c	c	c	c	a	g	a	a	a	c	c	a	t	g	a	a	t	c	t	t	c	a	t	t	g	g
MH709323	4	a	g	t	g	g	g	a	g	t	a	c	g	c	g	g	a	a	a	g	t	g	t	t	g	c	t	t	a	g	c	t	t	c	t	a	c	g	c	g	c	a	c	t	a	t	g	c	a	c	c	c	c	a	g	a	a	a	c	c	a	t	g	a	a	t	c	t	t	c	a	t	t	g	g
MH709326	4	a	g	t	g	g	g	g	g	t	a	c	g	c	g	g	a	a	a	g	t	g	c	t	g	c	t	t	a	g	c	t	t	c	t	a	c	g	c	g	c	a	c	t	a	t	g	c	a	c	c	c	c	a	g	a	a	a	c	c	a	t	g	a	a	t	c	t	t	c	a	t	t	g	g
MH709341	4	a	g	t	g	g	g	a	g	t	a	c	g	a	g	g	a	a	a	g	t	g	c	t	g	c	t	t	a	g	c	t	t	c	t	a	c	g	c	g	c	a	c	t	a	t	g	c	a	c	c	c	c	a	g	a	a	a	c	c	a	t	g	a	a	t	c	t	t	c	a	t	t	g	g
MH709357	4	c	g	t	g	g	g	a	g	t	a	c	g	c	g	g	a	a	a	g	t	g	c	t	g	c	t	t	a	g	c	t	t	c	t	a	c	g	c	g	c	a	c	t	a	t	g	c	a	c	c	c	c	a	g	a	a	a	c	c	a	t	g	a	a	t	c	t	t	c	a	t	t	g	g
MH709364	4	a	g	t	g	g	g	a	g	t	a	c	g	c	g	g	a	a	a	g	t	g	c	t	g	c	t	t	a	g	c	t	t	c	t	a	c	g	c	g	c	a	c	t	a	t	g	c	a	c	c	c	c	a	g	a	a	a	c	c	a	t	g	a	a	t	c	t	t	c	a	t	t	g	g
MH709370	4	a	g	t	g	g	g	a	g	t	a	c	g	c	g	g	a	a	a	g	t	g	c	t	g	c	t	t	a	g	c	t	t	c	t	a	c	g	c	g	c	a	c	t	a	t	g	c	a	c	c	c	c	a	g	a	a	a	c	c	a	t	g	a	a	t	c	t	t	c	a	t	t	g	g
MH709371	4	a	g	t	g	g	g	a	g	t	a	c	g	c	g	g	a	a	a	g	t	g	c	t	g	c	t	t	a	g	c	t	t	c	t	a	c	g	c	g	c	a	c	t	a	t	g	c	a	c	c	c	c	a	g	a	a	a	c	c	a	t	g	a	a	t	c	t	t	c	a	t	t	g	g
MH709372	4	a	g	t	g	g	g	a	g	t	a	c	g	c	g	g	a	a	a	g	t	g	c	t	g	c	t	t	a	g	c	t	t	c	t	a	c	g	c	g	c	a	c	t	a	t	g	c	a	c	c	c	c	a	g	a	a	a	c	c	a	t	g	a	a	t	c	t	t	c	a	t	t	g	g
DQ457052	5	a	g	t	g	g	g	a	g	t	a	c	g	c	g	g	a	a	a	g	t	g	c	t	g	t	t	t	a	g	c	t	t	c	t	a	c	g	c	g	c	a	c	t	a	t	g	c	g	c	c	c	c	a	g	a	a	a	c	c	g	c	g	a	a	t	c	t	t	c	a	t	t	a	g
JN704704	5	a	g	t	g	g	g	a	g	t	a	c	g	c	g	g	a	a	a	g	t	g	c	t	a	c	t	t	a	g	c	t	t	c	t	a	c	g	c	g	t	a	c	t	a	t	g	c	g	c	c	c	c	a	g	a	a	a	c	c	g	c	g	a	a	t	c	t	t	c	a	t	t	a	g
JN704705	5	a	g	t	g	g	g	a	g	t	a	c	g	c	g	g	a	a	a	g	t	g	c	t	g	c	t	t	a	g	c	t	t	c	t	a	c	g	c	g	c	a	c	t	a	t	g	c	g	c	c	c	c	a	g	a	a	a	c	c	g	c	g	a	a	t	c	t	t	c	a	t	t	g	g
JN704706	5	a	g	t	g	g	g	a	g	t	g	c	g	c	g	g	a	a	a	g	t	g	c	t	g	c	t	t	a	g	c	t	t	c	t	a	c	g	c	g	c	a	c	t	a	t	g	c	g	c	c	c	c	a	g	a	a	a	c	c	g	c	g	a	a	t	c	t	t	c	a	t	t	g	g
JN704707	5	a	g	t	g	g	g	a	g	t	a	c	g	c	g	g	a	a	a	g	t	g	c	t	g	c	t	t	a	g	c	t	t	c	t	a	c	g	c	g	c	a	c	t	a	t	g	c	g	c	c	c	c	a	g	a	a	a	c	c	g	c	g	a	a	t	c	t	t	c	a	t	t	a	g
JN704708	5	a	g	t	g	g	g	a	g	t	a	c	g	c	g	g	a	a	a	g	t	g	c	t	g	c	t	t	a	g	c	t	t	c	t	a	c	g	c	g	c	a	c	t	a	t	g	c	g	c	c	c	c	a	g	a	a	a	c	c	g	c	g	a	a	t	c	t	t	c	a	t	t	g	g
KP771904	5	a	g	t	g	g	g	a	g	t	a	c	g	c	g	g	a	a	a	a	t	g	c	t	g	c	t	t	a	g	c	t	t	c	t	a	c	g	c	g	c	a	c	t	a	t	g	c	g	c	c	c	c	a	g	a	a	a	c	c	g	c	g	a	a	t	c	t	t	c	a	t	t	a	g
KP771905	5	a	g	t	g	g	g	a	g	t	a	c	g	c	g	g	a	a	a	g	t	g	c	t	g	c	t	t	a	g	c	t	t	c	t	a	c	g	c	g	c	a	c	t	a	t	g	c	g	c	c	c	c	a	g	a	a	a	c	c	g	c	g	a	a	t	c	t	t	c	a	t	t	a	g
KP771906	5	a	g	t	g	g	g	a	g	t	a	c	g	c	g	g	a	a	a	g	t	g	c	t	g	c	t	t	a	g	c	t	t	c	t	a	c	g	c	g	c	a	c	t	a	t	g	c	g	c	c	c	c	a	g	a	a	a	c	c	g	c	g	a	a	t	c	t	t	c	a	t	t	a	g
KP771919	5	a	g	t	g	g	g	a	g	t	a	c	g	c	g	g	a	a	a	g	t	g	c	t	g	c	t	t	a	g	c	t	t	c	t	a	c	g	c	g	c	a	c	t	a	t	g	c	g	c	c	c	c	a	g	a	a	a	c	c	g	c	g	a	a	t	c	t	t	c	a	t	t	a	g
KP771922	5	a	g	t	g	g	g	a	g	t	a	c	g	c	g	g	a	a	a	g	t	g	c	t	g	c	t	t	a	g	c	t	t	c	t	a	c	g	c	g	c	a	c	t	a	t	g	c	g	c	c	c	c	a	g	a	a	a	c	c	g	c	g	a	a	t	c	t	c	c	a	t	t	a	g
KP771924	5	a	g	t	g	g	g	a	g	t	a	c	g	c	g	g	a	a	a	g	t	g	c	t	g	c	t	t	a	g	c	t	t	c	t	a	c	g	c	g	c	a	c	t	a	t	g	c	g	c	c	c	c	a	g	a	a	a	c	c	g	c	g	a	a	t	c	t	t	c	a	t	t	a	g
KM355696	5	a	g	t	g	g	g	a	g	t	a	c	g	c	g	g	a	a	a	g	t	g	c	t	g	c	t	t	a	t	c	t	t	c	t	a	c	g	c	g	c	a	c	t	a	t	g	c	g	c	c	c	c	a	g	a	a	a	c	c	g	c	g	a	a	t	c	t	t	c	a	t	t	g	g
KM355697	5	a	g	t	g	g	g	a	g	t	a	c	g	c	g	g	a	a	a	g	t	g	c	t	g	c	t	t	a	t	c	t	t	c	t	a	c	g	c	g	c	a	c	t	a	t	g	c	g	c	c	c	c	a	g	a	a	a	c	c	g	c	g	a	a	t	c	t	t	c	a	t	t	g	g
KM355698	5	a	g	t	g	g	g	a	g	t	a	c	g	c	g	g	a	a	a	g	t	g	c	t	g	c	t	t	a	g	c	t	t	c	t	a	c	g	c	g	c	a	c	t	a	t	g	c	g	c	c	c	c	a	g	a	a	a	c	c	g	c	g	a	a	t	c	t	t	c	a	t	t	g	g
KM355699	5	a	g	t	g	g	g	a	g	t	a	c	g	c	g	g	a	a	a	g	t	g	c	t	g	c	t	t	a	g	c	t	t	c	t	a	c	g	c	g	c	a	c	t	a	t	g	c	g	c	c	c	c	a	g	a	a	a	c	c	g	c	g	a	a	t	c	t	t	c	a	t	t	g	g
KM355701	5	a	g	t	g	g	g	a	g	t	a	c	g	c	g	g	a	a	a	g	t	g	c	t	g	c	t	t	a	g	c	t	t	c	t	a	c	g	c	g	c	a	c	t	a	t	g	c	g	c	c	c	c	a	g	a	a	a	c	c	g	c	g	a	a	t	c	t	t	c	a	t	t	g	g
KM355702	5	a	g	t	g	g	g	a	g	t	a	c	g	c	g	g	a	a	a	g	t	g	c	t	g	c	t	t	a	g	c	t	t	c	t	a	c	g	c	g	c	a	c	t	a	t	g	c	g	c	c	c	c	a	g	a	a	a	c	c	g	c	g	a	a	t	c	t	t	c	a	t	t	g	g
KM355703	5	a	g	t	g	g	g	a	g	t	a	c	g	c	g	g	a	a	a	g	t	g	c	t	g	c	t	t	a	t	c	t	t	c	t	a	c	g	c	g	c	a	c	t	a	t	g	c	g	c	c	c	c	a	g	a	a	a	c	c	g	c	g	a	a	t	c	t	t	c	a	t	t	g	g
KM355704	5	a	g	t	g	g	g	a	g	t	a	c	g	c	g	g	a	a	a	g	t	g	c	t	g	c	t	t	a	t	c	t	t	c	t	a	c	g	c	g	c	a	c	t	a	t	g	c	g	c	c	c	c	a	g	a	a	a	c	c	g	c	g	a	a	t	c	t	t	c	a	t	t	g	g
KM355705	5	a	g	t	g	g	g	a	g	t	a	c	g	c	g	g	a	a	a	g	t	g	c	t	g	c	t	t	a	g	c	t	t	c	t	a	c	g	c	g	c	a	c	t	a	t	g	c	g	c	c	c	c	a	g	a	a	a	c	c	g	c	g	a	a	t	c	t	t	c	a	t	t	g	g
KM355706	5	a	g	t	g	g	g	a	g	t	a	c	g	c	g	g	a	a	a	g	t	g	c	t	g	c	t	t	a	g	c	t	t	c	t	a	c	g	c	g	c	a	c	t	a	t	g	c	g	c	c	c	c	a	g	a	a	a	c	c	g	c	g	a	a	t	c	t	t	c	a	t	t	g	g
KM355707	5	a	g	t	g	g	g	a	g	t	a	c	g	c	g	g	a	a	a	g	t	g	c	t	g	c	t	t	a	g	c	t	t	c	t	a	c	g	c	g	c	a	c	t	a	t	g	c	g	c	c	c	c	a	g	a	a	a	c	c	g	c	g	a	a	t	c	t	t	c	a	t	t	g	g
KM355708	5	a	g	t	g	g	g	a	g	t	a	c	g	c	g	g	a	a	a	g	t	g	c	t	g	c	t	t	a	g	c	t	t	c	t	a	c	g	c	g	c	a	c	t	a	t	g	c	g	c	c	c	c	a	g	a	a	a	c	c	g	c	g	a	a	t	c	t	t	c	a	t	t	g	g
KM355709	5	a	g	t	g	g	g	a	g	t	a	c	g	c	g	g	a	a	a	g	t	g	c	t	g	c	t	t	a	g	c	t	t	c	t	a	c	g	c	g	c	a	c	t	a	t	g	c	g	c	c	c	c	a	g	a	a	a	c	c	g	c	g	a	a	t	c	t	t	c	a	t	t	g	g
KM355711	5	a	g	t	g	g	g	a	g	t	a	c	g	c	g	g	a	a	a	g	t	g	c	t	g	c	t	t	a	g	c	t	t	c	t	a	c	g	c	g	c	a	c	t	a	t	g	c	g	c	c	c	c	a	g	a	a	a	c	c	g	c	g	a	a	t	c	t	t	c	a	t	t	g	g
KM355712	5	a	g	t	g	g	g	a	g	t	a	c	g	c	g	g	a	a	a	g	t	g	c	t	g	c	t	t	a	g	c	t	t	c	t	a	c	g	c	g	c	a	c	t	a	t	g	c	g	c	c	c	c	a	g	a	a	a	c	c	g	c	g	a	a	t	c	t	t	c	a	t	t	g	g
KY037797	5	[illegible]	[illegible]	[illegible]	[illegible]	[illegible]	[illegible]	[illegible]	[illegible]	[illegible]	[illegible]	[illegible]	[illegible]	[illegible]	[illegible]	[illegible]	[illegible]	[illegible]	[illegible]	[illegible]	[illegible]	[illegible]	[illegible]	[illegible]	[illegible]	[illegible]	[illegible]	[illegible]	[illegible]	[illegible]	[illegible]	[illegible]	[illegible]	[illegible]	[illegible]	[illegible]	[illegible]	[illegible]	[illegible]	[illegible]	[illegible]	[illegible]	[illegible]	[illegible]	[illegible]	[illegible]	[illegible]	[illegible]	[illegible]	[illegible]	[illegible]	[illegible]	[illegible]	[illegible]	[illegible]	[illegible]	[illegible]	[illegible]	[illegible]	[illegible]	[illegible]	[illegible]	[illegible]	[illegible]	[illegible]	[illegible]	[illegible]	[illegible]	[illegible]	[illegible]	[illegible]	[illegible]	[illegible]	[illegible]	[illegible]

Supplementary Table 3: 2880 SN

GB-ID	Clade markers																											2								2										2		5													4													
	Position in Dumas	45459	45505	45531	45640	45642	45717	46008	46264	46305	46315	46356	46363	46398	46477	46609	46672	46687	46703	46735	46822	46909	46978	47002	47026	47050	47074	47149	47162	47354	47511	47542	47622	47686	47697	47754	47940	47981	47996	48011	48050	48276	48635	48675	48724	48754	48825	48990	49005	49011	49034	49036	49038	49128	49134	49209	49269	49386	49389	49442	49535	49602	49742	49776	49908	49987	50081	50123	50157	50349	50382	50519	50555	50559	50593
MH499467	5	-	g	t	g	g	g	a	g	t	a	c	g	c	g	g	a	a	a	g	t	g	c	t	g	c	t	t	a	g	c	t	t	c	t	a	c	g	c	g	c	a	c	t	a	t	g	c	g	c	c	c	c	a	g	a	a	a	c	c	g	c	g	a	a	t	c	t	t	c	a	t	t	a	g
MH499469	5	a	g	t	g	g	g	a	g	t	a	c	g	c	g	g	a	a	a	g	t	g	c	t	g	c	t	t	a	g	c	t	t	c	t	a	c	g	c	g	c	a	c	t	a	t	g	c	g	c	c	c	c	a	g	a	a	a	c	c	g	c	g	a	a	t	c	t	t	c	a	t	t	a	g
MH709321	5	a	g	t	g	g	g	a	g	t	g	c	g	c	g	g	a	a	a	g	t	g	c	t	g	c	t	t	a	g	c	t	t	c	t	a	c	g	c	g	c	a	c	t	a	t	g	c	g	c	c	c	c	a	g	a	a	a	c	c	g	c	g	a	a	t	c	t	t	c	a	t	t	g	g
MH709324	5	a	g	t	g	g	g	a	g	t	a	c	g	c	g	g	a	a	a	g	t	g	c	t	g	c	t	t	a	g	c	t	t	c	t	a	c	g	c	g	c	a	c	t	a	t	g	c	g	c	c	c	c	a	g	a	a	g	c	c	g	c	g	a	a	t	c	t	t	c	a	t	t	a	g
MH709325	5	a	g	t	g	g	g	a	g	t	a	c	g	c	g	g	a	a	a	g	t	g	c	t	g	c	t	t	a	g	c	t	t	c	t	a	c	g	c	g	c	a	c	t	a	t	g	c	g	c	c	c	c	a	a	a	a	a	c	c	g	c	g	a	a	t	c	t	t	c	a	t	t	g	g
MH709327	5	a	g	t	g	g	g	a	g	t	a	c	g	c	g	g	a	a	a	g	t	g	c	t	g	t	t	t	a	g	c	t	t	c	t	a	c	g	c	g	c	a	c	t	a	t	g	c	g	c	c	c	c	g	g	a	a	a	c	c	g	c	g	a	a	t	c	t	t	c	a	t	t	a	g
MH709328	5	a	g	t	g	g	g	a	g	t	a	c	g	c	g	g	a	a	a	g	t	g	c	t	g	c	t	t	a	g	c	t	t	c	t	a	c	g	c	g	c	a	c	t	a	t	g	c	g	c	c	c	c	a	g	a	a	a	c	c	g	c	g	a	a	t	c	t	t	c	a	t	t	a	g
MH709330	5	a	g	t	g	g	g	a	g	t	a	c	g	c	g	g	a	a	a	g	t	g	c	t	g	c	t	t	a	g	c	t	t	c	t	a	c	g	c	g	c	a	c	t	a	t	g	c	g	c	c	c	c	a	g	a	a	a	c	c	g	c	g	a	a	t	c	t	t	c	a	t	t	a	g
MH709332	5	a	g	t	g	g	g	a	g	t	a	c	g	c	g	g	a	a	a	g	t	g	c	t	g	c	t	t	a	g	c	t	g	c	t	a	c	g	c	g	c	a	c	t	a	t	g	c	g	c	c	c	c	a	g	a	a	a	c	c	g	c	g	a	a	t	c	t	t	c	a	c	t	a	g
MH709334	5	a	g	t	g	g	g	a	g	t	a	c	g	c	g	g	a	a	a	g	t	g	c	t	g	t	t	t	a	g	c	t	t	c	t	a	c	g	c	g	c	a	c	t	a	t	g	c	g	c	c	c	c	a	g	a	a	a	c	c	g	c	g	a	a	t	c	t	t	c	a	t	t	a	g
MH709335	5	a	g	t	g	g	g	a	g	t	a	t	g	c	g	g	a	a	a	g	t	g	c	t	g	t	t	t	a	g	c	t	t	c	t	a	c	g	c	g	c	a	c	t	a	t	g	c	g	c	c	c	c	a	g	a	a	a	c	c	g	c	g	a	a	t	c	t	t	c	a	t	t	a	g
MH709336	5	a	g	t	g	g	g	a	g	t	a	c	g	c	g	g	a	a	a	g	t	g	c	t	g	t	t	t	a	g	c	t	t	c	t	a	c	g	c	g	c	a	c	t	a	t	g	c	g	c	c	c	c	a	g	a	a	a	c	c	g	c	g	a	a	t	c	t	t	c	a	t	t	a	g
MH709338	5	a	g	t	g	g	g	a	g	t	a	c	g	c	g	g	a	a	a	g	t	g	c	t	g	t	t	t	a	g	c	t	t	c	t	a	c	g	c	g	c	a	c	t	a	t	g	c	g	c	c	c	c	a	g	a	a	a	c	c	g	c	g	a	a	t	c	t	t	c	a	t	t	a	g
MH709340	5	a	g	t	g	g	g	a	g	t	a	c	g	c	g	g	a	a	a	g	t	g	c	t	g	c	t	t	a	g	c	t	t	c	t	a	c	g	c	g	c	a	c	t	a	t	g	c	g	c	c	c	c	a	g	a	a	a	c	c	g	c	g	a	a	t	c	t	t	t	a	t	t	g	g
MH709342	5	a	g	t	g	g	g	a	g	t	g	c	g	c	g	g	a	a	a	g	t	g	c	t	g	c	t	t	a	g	c	t	t	c	t	a	c	g	c	g	c	a	c	t	a	t	g	c	g	c	c	c	c	a	g	a	a	a	c	c	g	c	g	a	a	t	c	t	t	c	a	t	t	g	g
MH709343	5	a	g	t	g	g	g	a	g	t	a	c	g	c	g	g	a	a	a	g	t	g	c	t	g	c	t	t	a	g	c	t	t	c	t	a	c	g	c	g	c	a	c	t	a	t	g	c	g	c	c	c	c	a	g	a	a	a	c	c	g	c	g	a	a	t	c	t	t	c	a	t	t	a	g
MH709345	5	a	g	t	g	g	g	a	g	t	a	c	g	c	g	g	a	a	a	g	t	g	c	t	g	t	t	t	a	g	c	t	t	c	t	a	c	g	c	g	c	a	c	t	a	t	g	c	g	c	c	c	c	a	g	a	a	a	c	c	g	c	g	a	a	t	c	t	t	c	a	t	t	a	g
MH709347	5	a	g	t	g	g	g	a	g	t	a	c	g	c	g	g	a	a	a	g	t	g	c	t	g	t	t	t	a	g	c	t	t	c	t	a	c	g	c	g	c	a	c	t	a	t	g	c	g	c	c	c	c	a	g	a	a	a	c	c	g	c	g	a	a	t	c	t	t	c	a	t	t	a	g
MH709349	5	a	g	t	g	g	g	a	g	t	a	c	g	c	g	g	a	a	a	g	t	g	c	t	g	c	t	t	a	g	c	t	t	c	t	a	c	g	c	g	c	a	c	t	a	t	g	c	g	c	c	c	c	a	g	a	a	a	c	c	g	c	g	a	a	t	c	t	t	c	a	t	t	a	g
MH709350	5	a	g	t	g	g	g	a	g	t	a	c	g	c	g	g	a	a	a	g	t	g	c	t	g	t	t	t	a	g	c	t	t	c	t	a	c	g	c	g	c	a	c	t	a	t	g	c	g	c	c	c	c	a	g	a	a	a	c	c	g	c	g	a	a	t	c	t	t	c	a	t	t	a	g
MH709351	5	a	g	t	g	g	g	a	g	t	a	c	g	c	g	g	a	a	a	g	t	g	c	t	g	c	t	t	a	g	c	t	t	c	t	a	c	g	c	g	c	a	c	t	a	t	g	c	g	c	c	c	c	a	g	a	a	a	c	c	g	c	g	a	a	t	c	t	t	c	a	t	t	g	g
MH709353	5	a	g	t	g	g	g	a	g	t	a	c	g	c	g	g	a	a	a	g	t	g	c	t	g	t	t	t	a	g	c	t	t	c	t	a	c	g	c	g	c	a	c	t	a	t	g	c	g	c	c	c	c	a	g	a	a	a	c	c	g	c	g	a	a	t	c	t	t	c	a	t	t	a	g
MH709358	5	a	g	t	g	g	g	a	g	t	a	c	g	c	g	g	a	a	a	g	t	g	c	t	g	c	t	t	a	g	c	t	t	c	t	a	c	g	c	g	c	a	c	t	a	t	g	c	g	c	c	c	c	a	g	a	a	a	c	c	g	c	g	a	a	t	c	t	t	c	a	t	t	a	g
MH709360	5	a	g	t	g	g	g	a	g	t	a	c	g	c	g	g	a	a	a	g	t	g	c	t	g	c	t	t	a	g	c	t	t	c	t	a	c	g	c	g	c	a	c	t	a	t	g	c	g	c	c	c	c	a	g	a	a	a	c	c	g	c	g	a	a	t	c	t	t	c	a	t	t	g	g
MH709362	5	a	g	t	g	g	g	a	g	t	a	c	g	c	g	g	a	a	a	g	t	g	c	t	g	t	t	t	a	g	c	t	t	c	t	a	c	g	c	g	c	a	c	t	a	t	g	c	g	c	c	c	c	a	g	a	a	a	c	c	g	c	g	a	a	t	c	t	t	c	a	t	t	a	g
MH709365	5	a	g	t	g	g	g	a	g	t	a	c	g	c	g	g	a	a	a	g	t	g	c	t	g	t	t	t	a	g	c	t	t	c	t	a	c	g	c	g	c	a	c	t	a	t	g	c	g	c	c	c	c	a	g	a	a	a	c	c	g	c	g	a	a	t	c	t	t	c	a	t	t	a	g
MH709373	5	a	g	t	g	g	g	a	g	t	a	c	g	c	g	g	a	a	a	g	t	g	c	t	g	c	t	t	a	g	c	t	t	c	t	a	c	g	c	g	c	a	c	t	a	t	g	c	g	c	c	c	c	a	g	a	a	a	c	c	g	c	g	a	a	t	c	t	t	c	a	t	t	a	g
MH709374	5	a	g	t	g	g	g	a	g	t	a	c	g	c	g	g	a	a	a	g	t	g	c	t	g	t	t	t	a	g	c	t	t	c	t	a	c	g	c	g	c	a	c	t	a	t	g	c	g	c	c	c	c	a	g	a	a	a	c	c	g	c	g	a	a	t	c	t	t	c	a	t	t	a	g
KC112914	6	a	g	t	g	g	g	a	g	t	a	c	g	c	g	a	a	a	a	g	t	g	c	t	g	c	t	t	a	g	c	t	t	c	t	a	c	g	c	g	c	a	c	t	a	t	g	c	a	c	c	c	c	a	g	a	a	a	c	c	g	c	g	a	a	t	c	t	t	c	a	t	t	g	g
KP771914	6	a	g	t	g	g	g	a	g	t	a	c	g	c	g	a	a	a	a	g	t	g	c	t	g	c	t	t	a	g	c	t	t	c	t	a	c	g	c	g	c	a	c	t	a	t	g	c	a	c	c	c	c	a	g	a	a	a	c	c	g	c	g	a	a	t	c	t	t	c	a	t	t	g	g
KP702725	6	a	g	t	g	g	g	a	g	t	a	c	g	c	g	a	a	a	a	g	t	g	c	t	g	c	t	t	a	g	c	t	t	c	t	a	c	g	c	g	c	a	c	t	a	t	g	c	a	c	c	c	c	a	g	a	a	a	c	c	g	c	g	a	a	t	c	t	t	c	a	t	t	g	g
KP771890	9	a	g	t	g	g	g	a	g	t	a	c	g	c	g	g	a	a	a	g	t	g	c	t	g	c	t	t	a	g	c	t	t	c	t	a	c	g	c	g	c	a	c	t	a	t	g	c	a	c	c	c	c	a	g	g	a	a	c	c	g	c	g	a	a	t	c	a	t	c	a	t	t	g	g
JN704710	9	a	g	t	g	g	g	a	g	t	a	c	g	c	g	g	a	a	a	g	t	g	c	t	g	c	t	t	a	g	c	t	t	c	t	a	c	g	c	g	c	a	c	t	a	t	g	c	a	c	c	c	c	a	g	a	a	a	c	c	g	c	g	a	a	t	c	t	t	c	a	t	t	g	g
KY037798	9	a	g	t	g	c	g	a	g	t	a	c	g	c	g	g	a	a	a	g	t	g	c	t	g	c	t	t	a	g	c	t	t	c	t	a	c	g	c	g	c	a	c	t	a	t	g	c	a	c	c	c	c	a	g	g	a	a	c	c	g	c	g	a	a	t	c	t	t	c	a	t	t	g	g
MH709312	9	a	g	t	g	c	g	a	g	t	a	c	g	c	g	g	a	a	a	g	t	g	c	t	g	c	t	t	a	g	c	t	t	c	t	a	c	g	c	g	c	a	c	t	a	t	g	c	a	c	c	c	c	a	g	g	a	a	c	c	g	c	g	a	a	t	c	t	t	c	a	t	t	g	g
MH709352	9	a	g	t	g	g	g	a	g	t	a	c	g	c	g	g	a	a	a	g	t	g	c	t	g	c	t	t	a	g	c	t	t	c	t	a	c	g	c	g	c	a	c	t	a	t	g	c	a	c	c	c	c	a	g	g	a	a	c	c	g	c	g	a	a	t	c	t	t	c	a	t	t	g	g
AB097933	Vac	a	g	t	g	g	g	a	g	t	a	c	g	c	g	g	a	a	a	g	t	g	c	t	g	c	t	t	g	g	c	t	t	c	t	a	t	g	c	g	c	a	c	t	a	t	a	c	a	c	c	c	c	a	g	a	a	a	c	c	a	c	g	a	a	t	a	t	t	c	a	t	t	g	g
JN704698	Vac	a	g	t	g	g	g	a	g	t	a	c	g	c	g	g	a	a	a	g	t	g	c	t	g	c	t	t	g	g	c	t	t	c	t	a	t	g	c	g	c	a	c	t	a	t	a	c	a	c	c	c	c	a	g	a	a	a	c	c	a	c	g	a	a	t	a	t	t	c	a	t	t	g	g
JN704697	Vac	a	g	t	g	g	g	a	g	t	a	c	g	c	g	g	a	a	a	g	t	g	c	t	g	c	t	t	g	g	c	t	t	c	t	a	t	g	c	g	c	a	c	t	a	t	a	c	a	c	c	c	c	a	g	a	a	a	c	c	a	c	g	a	a	t	a	t	t	c	a	t	t	g	g
DQ008355	Vac	a	g	t	g	g	g	a	g	t	a	c	g	c	g	g	a	a	a	g	t	g	c	t	g	c	t	t	g	g	c	t	t	c	t	a	t	g	c	g	c	a	c	t	a	t	a	c	a	c	c	c	c	a	g	a	a	a	c	c	a	c	g	a	a	t	a	t	t	c	a	t	t	g	g
JF306641	Vac	a	g	t	g	g	g	a	g	t	a	c	g	c	g	g	a	a	a	g	t	g	c	t	g	c	t	t	g	g	c	t	t	c	t	a	t	g	c	g	c	a	c	t	a	t	a	c	a	c	c	c	c	a	g	a	a	a	c	c	a	c	g	a	a	t	a	t	t	c	a	t	t	g	g
AB097932	Vac	a	g	t	g	g	g	a	g	t	a	c	g	c	g	g	a	a	a	g	t	g	c	t	g	c	t	t	g	g	c	t	t	c	t	a	t	g	c	g	c	a	c	t	a	t	a	c	a	c	c	c	c	a	g	a	a	a	c	c	a	c	g	a	a	t	a	t	t	c	a	t	t	g	g
DQ008354	Vac	a	g	t	g	g	g	a	g	t	a	c	g	c	g	g	a	a	a	g	t	g	c	t	g	c	t	t	g	g	c	t	t	c	t	a	t	g	c	g	c	a	c	t	a	t	a	c	a	c	c	c	c	a	g	a	a	a	c	c	a	c	g	a	a	t	a	t	t	c	a	t	t	g	g
KU926312	Vac	a	g	t	g	g	g	a	g	t	a	c	g	c	g	g	a	a	a	g	t	g	c	t	g	c	t	t	g	g	c	t	t	c	t	a	t	g	c	g	c	a	c	t	a	t	a	c	a	c	c	c	c	a	g	a	a	a	c	c	a	c	g	a	a	t	a	t	t	c	a	t	t	g	g
KU926313	Vac	a	g	t	g	g	g	a	g	t	a	c	g	c	g	g	a	a	a	g	t	g	c	t	g	c	t	t	g	g	c	t	t	c	t	a	t	g	c	g	c	a	c	t	a	t	a	c	a	c	c	c	c	a	g	a	a	a	c	c	a	c	g	a	a	t	a	t	t	c	a	t	t	g	g
KU926314	Vac	a	g	t	g	g	g	a	g	t	a	c	g	c	g	g	a	a	a	g	t	g	c	t	g	c	t	t	g	g	c	t	t	c	t	a	t	g	c	g	c	a	c	t	a	t	a	c	a	c	c	c	c	a	g	a	a	a	c	c	a	c	g	a	a	t	a	t	t	c	a	t	t	g	g
KF558371	Vac	a	g	t	g	g	g	a	g	t	a	c	g	c	g	g	a	a	a	g	t	g	c	t	g	c	t	t	g	g	c	t	t	c	t	a	t	g	c	g	c	a	c	t	a	t	a	c	a	c	c	c	c	a	g	a	a	a	c	c	a	c	g	a	a	t	a	t	t	c	a	t	t	g	g
KF558372	Vac	a	g	t	g	g	g	a	g	t	a	c	g	c	g	g	a	a	a	g	t	g	c	t	g	c	t	t	g	g	c	t	t	c	t	a	t	g	c	g	c	a	c	t	a	t	a	c	a	c	c	c	c	a	g	a	a	a	c	c	a	c	g	a	a	t	a	t	t	c	a	t	t	g	g
KF558373	Vac	a	g	t	g	g	g	a	g	t	a	c	g	c	g	g	a	a	a	g	t	g	c	t	g	c	t	t	g	g	c	t	t	c	t	a	t	g	c	g	c	a	c	t	a	t	a	c	a	c	c	c	c	a	g	a	a	a	c	c	a	c	g	a	a	t	a	t	t	c	a	t	t	g	g
KF558374	Vac	a	g	t	g	g	g	a	g	t	a	c	g	c	g	g	a	a	a	g	t	g	c	t	g	c	t	t	g	g	c	t	t	c	t	a	t	g	c	g	c	g	a	t	a	t	a	c	a	c	c	c	c	a	g	a	a	a	c	c	a	c	g	a	a	t	a	t	t	c	a	t	t	g	g
KF558375	Vac	a	g	t	g	g	g	a	g	t	a	c	g	c	g	g	a	a	a	g	t	g	c	t	g	c	t	t	g	g	c	t	t	c	t	a	t	g	c	g	c	a	c	t	a	t	a	c	a	c	c	c	c	a	g	a	a	a	c	c	a	c	g	a	a	t	a	t	t	c	a	t	t	g	g
KF558376	Vac	a	g	t	g	g	g	a	g	t	a	c	g	c	g	g	a	a	a	g	t	g	c	t	g	c	t	t	g	g	c	t	t	c	t	a	t	g	c	g	c	a	c	t	a	t	a	c	a	c	c	c	c	a	g	a	a	a	c	c	a	c	g	a	a	t	a	t	t	c	a	t	t	g	g
KF558377	Vac	a	g	t	g	g	g	a	g	t	a	c	g	c	g	g	a	a	a	g	t	g	c	t	g	c	t	t	g	g	c	t	t	c	t	a	t	g	c	g	c	a	c	t	a	t	a	c	a	c	c	c	c	a	g	a	a	a	c	c	a	c	g	a	a	t	a	t	t	c	a	t	t	g	g
KF558378	Vac	a	g	t	g	g	g	a	g	t	a	c	g	c	g	g	a	a	a	g	t	g	c	t	g	c	t	t	g	g	c	t	t	c	t	a	t	g	c	g	c	a	c	t	a	t	a	c	a	c	c	c	c	a	g	a	a	a	c	c	a	c	g	a	a	t	a	t	t	c	a	t	t	g	g
KF558379	Vac	a	g	t	g	g	g	a	g	t	a	c	g	c	g	g	a	a	a	g	t	g	c	t	g	c	t	t	g	g	c	t	t	c	t	a	t	g	c	g	c	a	c	t	a	t	a	c	a	c	c	c	c	a	g	a	a	a	c	c	a	c	g	a	a	t	a	t	t	c	a	t	t	g	g
KF558380	Vac	a	g	t	g	g	g	a	g	t	a	c	g	c	g	g	a	a	a	g	t	g	c	t	g	c	t	t	g	g	c	t	t	c	t	a	t	g	c	g	c	a	c	t	a	t	a	c	a	c	c	c	c	a	g	a	a	a	c	c	a	c	g	a	a	t	a	t	t	c	a	t	t	g	g
KF558381	Vac	a	g	t	g	g	g	a	g	t	a	c	g	c	g	g	a	a	a	g	t	g	c	t	g	c	t	t	g	g	c	t	t	c	t	a	t	g	c	g	c	a	c	t	a	t	a	c	a	c	c	c	c	a	g	a	a	a	c	c	a	c	g	a	a	t	a	t	t	c	g	t	t	g	g
KF558382	Vac	a	g	t	g	g	g	a	g	t	a	c	g	c	g	g	a	a	a	g	t	g	c	t	g	c	t	t	g	g	c	t	t	c	t	a	t	g	c	g	c	a	c	t	a	t	a	c	a	c	c	c	c	a	g	a	a	a	c	c	a	c	g	a	a	t	a	t	t	c	a	t	t	g	g
KF558383	Vac	a	g	t	g	g	g	a	g	t	a	c	g	c	g	g	a	a	a	g	t	g	c	t	g	c	t	t	g	g	c	t	t	c	t	a	t	g	c	g	c	a	c	t	a	t	a	c	a	c	c	c	c	a	g	a	a	a	c	c	a	c	g	a	a	t	a	t	t	c	a	t	t	g	g
KF558384	Vac	a	g	t	g	g	g	a	g	t	a	c	g	c	g	g	a	a	a	g	t	g	c	t	g	c	t	t	g	g	c	t	t	c	t	a	t	g	c	g	c	a	c	t	a	t	a	c	a	c	c	c	c	a	g	a	a	a	c	c	a	c	g	a	a	t	a	t	t	c	a	t	t	g	g
KF558385	Vac	a	g	t	g	g	g	a	g	t	a	c	g	c	g	g	a	a	a	g	t	g	c	t	g	c	t	t	g	g	c	t	t	c	t	a	t	g	c	g	c	a	c	t	a	t	a	c	a	c	c	c	c	a	g	a	a	a	c	c	a	c	g	a	a	t	a	t	t	c	a	t	t	g	g
KF558386	Vac	a	g	t	g	g	g	a	g	t	a	c	g	c	g	g	a	a	a	g	t	g	c	t	g	c	t	t	g	g	c	t	t	c	t	a	t	g	c	g	c	a	c	t	a	t	a	c	a	c	c	c	c	a	g	a	a	a	c	c	a	c	g	a	a	t	a	t	t	c	a	t	t	g	g
KF558387	Vac	a	g	t	g	g	g	a	g	t	a	c	g	c	g	g	a	a	a	g	t	g	c	t	g	c	t	t	g	g	c	t	t	c	t	a	t	g	c	g	c	a	c	t	a	t	a	c	a	c	c	c	c	a	g	a	a	a	c	c	a	c	g	a	a	t	a	t	t	c	a	t	t	g	g
KF558388	Vac	a	g	t	g	g	g	a	g	t	a	c	g	c	g	g	a	a	a	g	t	g	c	t	g	c	t	t	g	g	c	t	t	c	t	a	t	g	c	g	c	a	c	t	a	t	a	c	a	c	c	c	c	a	g	a	a	a	c	c	a	c	g	a	a	t	a	t	t	c	a	t	t	g	g
KF558389	Vac	a	g	t	g	g	g	a	g	t	a	c	g	c	g	g	a	a	a	g	t	g	c	t	g	c	t	t	g	g	c	t	t	c	t	a	t	g	c	g	c	g	c	t	a	t	a	c	a	c	c	c	c	a	g	a	a	a	c	c	a	c	g	g	a	t	a	t	t	c	a	t	t	g	g
KF558390	Vac	a	g	t	g	g	g	a	g	t	a	c	g	c	g	g	a	a	a	g	t	g	c	t	g	c	t	t	g	g	c	t	t	c	t	a	t	g	c	g	c	a	c	t	a	t	a	c	a	c	c	c	c	a	g	a	a	a	c	c	a	c	g	a	a	t	a	t	t	c	a	t	t	g	g
KF558391	Vac	a	g	t	g	g	g	a	g	t	a	c	g	c	g	g	a	a	a	g	t	g	c	t	g	c	t	t	g	g	c	t	t	c	t	a	t	g	c	g	c	a	c	t	a	t	a	c	a	c	c	c	c	a	g	a	a	a	c	c	a	c	g	a	a	t	a	t	t	c	a	t	t	g	g
KF811485	Vac	a	g	t	g	g	g	a	g	t	a	c	g	c	g	g	a	a	a	g	t	g	c	t	g	c	t	t	g	g	c	t	t	c	t	a	t	g	c	g	c	a	c	t	a	t	a	c	a	c	c	c	c	a	g	a	a	a	c	c	a	c	g	a	a	t	a	t	t	c	g	t	t	g	g
KF853225	Vac	a	g	t	g	g	g	a	g	t	a	c	g	c	g	g	a	a	a	g	t	g	c	t	g	c	t	t	g	g	c	t	t	c	t	a	t	g	c	g	c	a	c	t	a	t	a	c	a	c	c	c	c	a	g	a	a	a	c	c	a	c	g	a	a	t	a	t	t	c	a	t	t	g	g
KF853226	Vac	a	g	t	g	g	g	a	g	t	a	c	g	c	g	g	a	a	a	g	t	g	c	t	g	c	t	t	g	g	c	t	t	c	t	a	t	g	c	g	c	a	c	t	a	t	a	c	a	c	c	c	c	a	g	a	a	a	c	c	a	c	g	a	a	t	a	t	t	c	a	t	t	g	g
KF853227	Vac	a	g	t	g	g	g	a	g	t	a	c	g	c	g	g	a	a	a	g	t	g	c	t	g	c	t	t	g	g	c	t	t	c	t	a	t	g	c	g	c	a	c	t	a	t	a	c	a	c	c	c	c	a	g	a	a	a	c	c	a	c	g	a	a	t	a	t	t	c	a	t	t	g	g
KF853228	Vac	a	g	t	g	g	g	a	g	t	a	c	g	c	g	g	a	a	a	g	t	g	c	t	g	c	t	t	g	g	c	t	t	c	t	a	t	g	c	g	c	a	c	t	a	t	a	c	a	c	c	c	c	a	g	a	a	a	c	c	a	c	g	a	a	t	a	t	t	c	a	t	t	g	g
KF853229	Vac	a	g	t	g	g	g	a	g	t	a	c	g	c	g	g	a	a	a	g	t	g	c	t	g	c	t	t	g	g	c	t	t	c	t	a	t	g	c	g	c	a	c	t	a	t	a	c	a	c	c	c	c	a	g	a	a	a	c	c	a	c	g	a	a	t	a	t	t	c	a	t	t	g	g
KF853230	Vac	a	g	t	g	g	g	a	g	t	a	c	g	c	g	g	a	a	a	g	t	g	c	t	g	c	t	t	g	g	c	t	t	c	t	a	t	g	c	g	c	a	c	t	a	t	a	c	a	c	c	c	c	a	g	a	a	a	c	c	a	c	g	a	a	t	a	t	t	c	a	t	t	g	g
KF853231	Vac	a	g	t	g	g	g	a	g	t	a	c	g	c	g	g	a	a	a	g	t	g	c	t	g	c	t	t	g	g	c	t	t	c	t	a	t	g	c	g	c	a	c	t	a	t	a	c	a	c	c	c	c	a	g	a	a	a	c	c	a	c	g	a	a	t	a	t	t	c	a	t	t	g	g
KF853232	Vac	a	g	t	g	g	g	a	g	t	a	c	g	c	g	g	a	a	a	g	t	g	c	t	g	c	t	t	g	g	c	t	t	c	t	a	t	g	c	g	c	a	c	t	a	t	a	c	a	c	c	c	c	a	g	a	a	a	c	c	a	c	g	a	a	t	a	t	t	c	a	t	t	g	g
KF853233	Vac	a	g	t	g	g	g	a	g	t	a	c	g	c	g	g	a	a	a	g	t	g	c	t	g	c	t	t	g	g	c	t	t	c	t	a	t	g	c	g	c	a	c	t	a	t	a	c	a	c	c	c	c	a	g	a	a	a	c	c	a	c	g	a	a	t	a	t	t	c	a	t	t	g	g
KF853234	Vac	a	g	t	g	g	g	a	g	t	a	c	g	c	g	g	a	a	a	g	t	g	c	t	g	c	t	t	g	g	c	t	t	c	t	a	t	g	c	g	c	a	c	t	a	t	a	c	a	c	c	c	c	a	g	a	a	a	c	c	a	c	g	a	a	t	a	t	t	c	a	t	t	g	g
KF853235	Vac	a	g	t	g	g	g	a	g	t	a	c	g	c	g	g	a	a	a	g	t	g	c	t	g	c	t	t	g	g	c	t	t	c	t	a	t	g	c	g	c	a	c	t	a	t	a	c	a	c	c	c	c	a	g	a	a	a	c	c	a	c	g	a	a	t	a	t	t	c	a	t	t	g	g
MF898328	Vac	a	g	t	g	g	g	a	g	t	a	c	g	c	g	g	a	a	a	g	t	g	c	t	g	c	t	t	g	g	c	t	t	c	t	a	t	g	c	g	c	a	c	t	a	t	a	c	a	c	c	c	c	a	g	a	a	a	c	c	a	c	g	a	a	t	a	t	t	c	a	t	t	g	g
JN704709	VIII	a	g	t	g	g	a	a	g	t	a	c	g	c	g	a	a	a	a	g	t	g	c	t	g	c	t	t	a	g	c	t	t	c	t	a	c	g	c	g	c	a	c	t	a	t	g	c	a	c	c	c	c	a	g	a	a	a	c	c	g	c	g	a	a	t	c	t	t	c	a	t	t	g	g

Supplementary Table 3: 2880 SN

GB-ID	Clade markers																											SD14																														5																
	Position in Dumas	50671	50673	50710	50801	50823	50920	51036	51168	51183	51190	51200	51206	51334	51510	51606	51609	51681	51687	51696	51714	51720	51762	51840	51920	51929	51940	52114	52152	52162	52192	52209	52237	52362	52365	52497	52588	52644	52761	52796	52833	52917	52977	53068	53082	53139	53304	53436	53482	53523	53595	53938	53967	53993	54071	54096	54132	54139	54231	54264	54351	54359	54360	54362	54371	54393	54435	54474	54485	54529	54550	54555	54556	54562	54564
AY548170	1	a	t	a	t	a	c	c	c	c	g	c	g	g	c	c	c	c	t	g	c	a	c	g	t	c	c	t	t	c	a	a	t	a	c	a	c	a	t	c	a	a	a	t	g	a	g	t	a	a	t	g	c	g	c	a	t	g	a	a	a	a	c	t	c	a	c	t	c	c	g	c	t	t	c
AY548171	1	a	t	a	t	a	c	c	c	c	g	c	g	g	c	c	c	c	t	g	c	a	c	g	t	c	c	t	t	c	a	a	t	a	c	a	c	a	t	c	a	a	a	t	g	a	g	t	a	a	t	g	c	g	c	a	t	g	a	a	a	a	c	t	c	a	c	t	c	c	g	c	t	t	c
DQ479953	1	a	t	a	t	a	c	c	c	c	g	c	g	g	c	c	c	c	t	g	c	a	c	g	c	c	c	t	t	c	a	a	t	a	c	a	c	a	t	c	a	a	a	t	g	a	g	t	a	g	t	g	c	g	c	a	t	g	a	a	a	a	c	t	c	a	c	t	c	c	g	c	t	t	c
DQ479954	1	a	t	a	t	a	c	c	c	c	g	c	g	g	c	c	c	c	t	g	c	a	c	g	c	c	c	c	t	c	a	a	c	a	c	a	c	a	t	c	a	a	a	t	g	a	g	t	a	g	t	g	c	g	c	a	t	g	a	a	a	a	c	t	c	a	c	t	c	c	g	c	t	t	c
DQ479958	1	a	t	a	t	a	c	c	c	c	g	c	g	g	c	c	c	c	t	g	c	a	c	g	t	c	c	t	t	c	a	a	t	a	c	a	c	a	t	c	a	a	a	t	g	a	g	t	a	a	t	g	c	g	c	a	t	g	a	a	a	a	c	t	c	a	c	t	c	c	g	c	t	t	c
DQ479959	1	a	t	a	t	a	c	c	c	c	g	c	g	g	c	c	c	c	t	g	c	a	c	g	t	c	c	t	t	c	a	a	t	a	c	a	c	a	t	c	a	a	a	t	g	a	g	t	a	a	t	g	c	g	c	a	t	g	a	a	a	a	c	t	c	a	c	t	c	c	g	c	t	t	c
DQ479961	1	a	t	a	t	a	c	c	c	c	g	c	g	g	c	c	c	c	t	g	c	a	c	g	c	c	c	t	t	c	a	a	t	a	c	a	c	a	t	c	a	a	a	t	g	a	g	t	a	g	t	g	c	g	c	a	t	g	a	a	a	a	c	t	c	a	c	t	c	c	g	c	t	t	c
DQ479962	1	a	t	a	t	a	c	c	c	c	g	c	g	g	c	c	c	c	t	g	c	a	c	g	c	c	c	t	t	c	a	a	t	a	c	a	c	a	t	c	a	a	a	t	g	a	g	t	a	g	t	g	c	g	c	a	t	g	a	a	a	a	c	t	c	a	c	t	c	c	g	c	t	t	c
DQ479963	1	a	t	a	t	a	c	c	c	c	g	c	g	g	c	c	c	c	t	g	c	a	c	g	c	c	c	t	t	c	a	a	t	a	c	a	c	a	t	c	a	a	a	t	g	a	g	t	a	g	t	g	c	g	c	a	t	g	a	a	a	a	c	t	c	a	c	t	c	c	g	c	t	t	c
DQ674250	1	a	t	a	t	a	c	c	c	c	g	c	g	g	c	c	c	c	t	g	c	a	c	g	t	c	c	t	t	c	a	a	t	a	c	a	c	a	t	c	a	a	a	t	g	a	g	t	a	a	t	g	c	g	c	a	t	g	a	a	a	a	c	t	c	a	c	t	c	c	g	c	-	t	c
KX262864	1	a	t	a	t	a	c	c	c	c	g	c	g	g	c	c	c	c	t	g	c	a	c	g	t	c	c	t	t	c	a	a	t	a	c	a	c	a	t	c	a	a	a	t	g	a	g	t	a	a	t	g	c	g	c	a	t	g	a	a	a	a	c	t	c	a	c	t	c	c	g	c	t	t	c
KX262865	1	a	t	a	t	a	c	c	c	c	g	c	g	g	c	c	c	c	t	g	c	a	c	g	t	c	c	t	t	c	a	a	t	a	c	a	c	a	t	c	a	a	a	t	g	a	g	t	a	a	t	g	c	g	c	a	t	g	a	a	a	a	c	t	c	a	c	t	c	c	g	c	t	t	c
KX262866	1	a	t	a	t	a	c	c	c	c	g	c	g	g	c	c	c	c	t	g	c	a	c	g	t	c	c	t	t	c	a	a	t	a	c	a	c	a	t	c	a	a	a	t	g	a	g	t	a	a	t	g	c	g	c	a	t	g	a	a	a	a	c	t	c	a	c	t	c	c	g	c	t	t	c
EU154348	1	a	t	a	t	a	c	c	c	c	g	c	g	g	c	c	c	c	t	g	c	a	c	g	c	c	c	t	t	c	a	a	t	a	c	a	c	a	t	c	a	a	a	t	g	a	g	t	a	g	t	g	c	g	c	a	t	g	a	a	a	a	c	t	c	a	c	t	c	c	g	c	t	t	c
JN704690	1	a	t	a	t	a	c	c	c	c	g	c	g	g	c	c	c	c	t	g	c	a	c	g	t	c	c	t	t	c	a	a	t	a	c	a	c	a	t	c	a	a	a	t	g	a	g	t	a	a	t	g	c	g	c	a	t	g	a	a	a	a	c	t	c	a	c	t	c	c	g	c	t	t	c
JN704691	1	a	t	a	t	a	c	c	c	c	g	c	g	g	c	c	c	c	t	g	c	a	c	g	t	c	c	t	t	c	a	a	t	a	c	a	c	a	t	c	a	a	a	t	g	a	g	t	a	a	t	g	c	g	c	a	t	g	a	a	c	a	c	t	c	a	c	t	c	c	g	c	t	t	c
JN704692	1	a	t	a	t	a	c	c	c	c	g	c	g	g	c	c	c	c	t	g	c	a	c	g	t	c	c	t	t	c	a	a	t	a	c	a	c	a	t	c	a	a	a	t	g	a	g	t	a	a	g	g	c	g	c	a	t	g	a	a	a	a	c	t	c	a	c	t	c	c	g	c	t	t	c
JN704693	1	a	t	a	t	a	c	c	c	c	g	c	g	g	c	c	c	c	t	g	c	a	c	g	c	c	c	t	t	c	a	a	t	a	c	a	c	a	t	c	a	a	a	t	g	a	g	t	a	g	t	g	c	g	c	a	t	g	a	a	a	a	c	t	c	a	c	t	c	c	g	c	t	t	t
JN704694	1	a	t	a	t	a	c	c	c	c	g	c	g	g	c	c	c	c	t	g	c	a	c	g	t	c	c	t	t	c	a	a	t	a	c	a	c	a	t	c	a	a	a	t	g	a	g	t	a	a	t	g	c	g	c	a	t	g	a	a	a	a	c	t	c	a	c	t	c	c	g	c	t	t	c
JN704695	1	a	t	a	t	a	c	c	c	c	g	c	g	g	c	c	c	c	t	g	c	a	c	g	t	c	c	t	t	c	a	a	t	a	c	a	c	a	t	c	a	a	a	t	g	a	g	t	a	a	t	g	c	g	c	a	t	g	a	a	a	a	c	t	c	a	c	t	c	c	g	c	t	t	c
JN704696	1	a	t	a	t	a	c	c	c	c	g	c	g	g	c	c	c	c	t	g	c	a	c	g	t	c	c	t	t	c	a	a	t	a	c	a	c	a	t	c	a	a	a	t	g	a	g	t	a	a	t	g	c	g	c	a	t	g	a	a	c	a	c	t	c	a	c	t	c	c	g	c	t	t	c
NC001348	1	a	t	a	t	a	c	c	c	c	g	c	g	g	c	c	c	c	t	g	c	a	c	g	t	c	c	t	t	c	a	a	t	a	c	a	c	a	t	c	a	a	a	t	g	a	g	t	a	a	t	g	c	g	c	a	t	g	a	a	a	a	c	t	c	a	c	t	c	c	g	c	t	t	t
KP771892	1	a	t	a	t	a	c	c	c	c	g	c	g	g	c	c	c	c	t	g	c	a	c	g	t	c	c	t	t	c	a	a	t	a	c	a	c	a	t	c	a	a	a	t	g	a	g	t	a	a	t	g	c	g	c	a	t	g	a	a	a	a	c	t	c	a	c	t	c	c	g	c	t	t	c
KP771894	1	a	t	a	t	a	c	c	c	c	g	c	g	g	c	t	c	c	t	g	c	a	c	g	t	c	c	t	t	c	a	a	t	g	c	a	c	a	t	c	a	a	a	t	g	a	g	t	a	a	t	g	c	g	c	a	t	g	a	a	a	a	c	t	c	a	c	t	c	c	g	c	t	t	c
KP771900	1	a	t	a	t	a	c	c	c	c	g	c	g	g	c	c	c	c	t	g	c	a	c	g	t	c	c	t	t	c	a	a	t	a	c	a	c	a	t	c	a	a	a	t	g	a	g	t	a	g	t	g	c	g	c	a	t	g	a	a	a	a	c	t	c	a	t	t	c	c	g	c	t	t	c
KP771901	1	a	t	a	t	a	c	c	c	c	g	c	g	g	c	c	c	c	t	g	c	a	c	g	t	c	c	t	t	c	a	a	t	a	c	a	c	a	t	c	a	a	a	t	g	a	g	t	a	g	t	g	c	g	c	a	t	a	a	a	a	a	c	t	c	a	c	t	c	c	g	c	t	t	c
KP771902	1	a	t	a	t	a	c	c	c	c	g	c	g	g	c	c	c	c	t	g	c	a	c	g	t	c	c	t	t	c	a	a	t	a	c	a	c	a	t	c	a	a	a	t	g	a	g	t	a	g	t	g	c	g	c	a	t	g	a	a	a	a	c	t	c	a	c	t	c	c	g	c	t	t	c
KP771908	1	a	t	a	t	a	c	c	c	c	g	c	g	g	c	c	c	c	t	g	c	a	c	g	t	c	c	t	t	c	a	a	t	a	c	a	c	a	t	c	a	a	a	t	g	a	g	t	a	a	t	g	c	g	c	a	t	g	a	a	a	a	c	t	c	a	c	t	c	c	g	c	t	t	c
KP771912	1	a	t	a	t	a	c	c	c	c	g	c	g	g	c	c	c	c	t	g	c	a	c	g	t	c	c	t	t	c	a	a	t	a	c	a	c	a	t	c	a	a	a	t	g	a	g	t	a	a	t	g	c	g	c	a	t	g	a	a	a	a	c	t	c	a	c	t	c	c	g	c	t	t	c
KP771913	1	a	t	a	t	a	c	c	c	c	g	c	g	g	c	c	c	c	t	g	c	a	c	g	t	c	c	t	t	c	a	a	t	a	c	a	c	a	t	c	a	a	a	t	g	a	g	t	a	a	t	g	c	g	c	a	t	g	a	a	a	a	c	t	c	a	c	t	c	c	g	c	t	t	c
KP771917	1	a	t	a	t	a	c	c	c	c	g	c	g	g	c	c	c	c	t	g	c	a	c	g	t	c	c	t	t	c	a	a	t	a	c	a	c	a	t	c	a	a	a	t	g	a	g	t	a	a	t	g	c	g	c	a	t	g	a	a	a	a	c	t	c	a	c	t	c	c	g	c	t	t	c
KP771918	1	a	t	a	t	a	c	c	c	c	g	c	g	g	c	c	c	c	t	g	c	a	c	g	t	c	c	t	t	c	a	a	t	a	c	a	c	a	t	c	a	a	a	t	g	a	g	t	a	a	t	g	c	g	c	a	t	g	a	a	a	a	c	t	c	a	c	t	c	c	g	c	t	t	c
KP771920	1	a	t	a	t	a	c	c	c	c	g	c	g	g	c	c	c	c	t	g	c	a	c	g	t	c	c	t	t	c	a	a	t	a	c	a	c	a	t	c	a	a	a	t	g	a	g	t	a	g	t	g	c	g	c	a	t	g	a	a	a	a	c	t	c	a	c	t	c	c	g	c	t	t	c
MH709361	1	a	t	a	t	a	c	c	c	c	g	c	g	g	c	c	c	c	t	g	c	a	c	g	t	c	c	t	t	c	a	a	t	a	c	a	c	a	t	c	a	a	a	t	g	a	g	t	a	a	t	g	c	g	c	a	t	g	a	a	a	a	c	t	c	a	c	t	c	c	g	c	t	t	c
KU926318	2	a	t	a	t	a	c	t	t	c	g	c	g	g	c	c	c	c	t	g	c	a	c	g	t	c	c	t	t	c	a	a	t	a	c	a	c	a	t	c	a	g	a	t	g	a	g	c	c	a	t	g	c	g	c	a	t	g	a	a	-	-	c	t	c	a	c	t	c	c	g	-	c	t	c
KU926319	2	a	t	a	t	a	c	c	t	c	g	c	g	g	c	c	c	c	t	g	c	a	c	g	t	c	c	t	t	c	a	a	t	a	c	a	c	a	t	c	a	g	a	t	g	a	g	t	c	a	t	g	c	g	c	a	t	g	a	a	-	-	-	t	c	a	c	t	c	c	g	c	-	t	c
KU926320	2	a	c	a	t	a	c	c	t	c	g	c	g	g	c	c	c	c	t	g	c	a	c	g	t	c	c	t	t	c	a	a	t	a	c	a	c	a	t	c	a	g	a	t	g	a	g	t	c	a	t	g	c	g	c	a	t	g	a	a	-	-	c	t	c	a	c	t	c	c	g	-	c	t	c
KU926321	2	a	t	a	t	a	c	c	t	c	g	c	g	g	c	c	c	c	t	g	c	a	c	g	t	c	c	t	t	c	a	a	t	a	c	a	c	a	t	c	a	g	a	t	g	a	g	t	c	a	t	g	c	g	c	a	t	g	a	a	-	-	-	t	c	a	c	t	c	c	g	c	-	t	c
KU926322	2	a	t	a	t	a	c	c	t	c	g	c	g	g	c	c	c	c	t	g	c	a	c	g	t	c	c	t	t	c	a	a	t	a	c	a	c	a	t	c	a	g	a	t	g	a	g	t	c	a	t	g	c	g	c	a	t	g	a	a	-	-	-	t	c	a	c	t	c	c	g	c	-	t	c
KC847290	2	a	t	a	t	a	c	c	t	c	g	c	g	g	c	c	c	c	t	g	c	a	c	g	t	c	c	t	t	c	a	a	t	a	c	a	c	a	t	c	a	g	a	t	g	g	g	t	c	a	t	g	c	g	c	a	t	g	a	a	-	-	-	t	c	a	c	t	c	c	g	c	-	t	c
KJ767491	2	a	t	a	t	a	c	t	t	c	g	c	g	g	c	c	c	c	t	g	c	a	c	g	t	c	c	t	t	c	a	a	t	a	c	a	c	a	t	c	a	g	a	t	g	a	g	t	c	a	t	g	c	g	c	a	t	g	a	a	-	-	c	t	c	a	c	t	c	c	g	-	c	t	c
KJ767492	2	a	t	a	t	a	c	c	t	c	g	c	g	g	c	c	c	c	t	g	c	a	c	g	t	c	c	t	t	c	a	a	t	a	c	a	c	a	t	c	a	g	a	t	g	a	g	t	c	a	t	g	c	g	c	a	t	g	a	a	-	-	-	t	c	a	c	t	c	c	g	c	-	t	c
KJ808816	2	a	t	a	t	a	c	c	t	c	g	c	g	g	c	c	c	c	t	g	c	a	c	g	t	c	c	t	t	c	a	a	t	a	c	a	c	a	t	c	a	g	a	t	g	a	g	t	c	a	t	g	c	g	c	a	t	g	a	a	-	-	-	t	c	a	c	t	c	c	g	c	-	t	c
KP771925	2	a	t	a	t	a	c	c	t	c	g	c	g	g	c	c	c	c	t	g	c	a	c	g	t	c	c	t	t	c	a	a	t	a	c	a	c	a	t	c	a	g	a	t	g	a	g	t	c	a	t	g	c	g	c	a	t	g	a	a	a	a	c	t	c	a	c	t	c	c	g	c	t	t	c
KU926315	2	a	t	a	t	a	c	t	t	c	g	c	g	g	c	c	c	c	t	g	c	a	c	g	t	c	c	t	t	c	a	a	t	a	c	a	c	a	t	c	a	g	a	t	g	a	g	t	c	a	t	g	c	g	c	a	t	g	a	a	-	-	c	t	c	a	c	t	c	c	g	-	c	t	c
KU926316	2	a	t	a	t	a	c	t	t	c	g	c	g	g	c	c	c	c	t	g	c	a	c	g	t	c	c	t	t	c	a	a	t	a	c	a	c	a	t	c	a	g	a	t	g	a	g	t	c	a	t	g	c	g	c	a	t	g	a	a	-	-	c	t	c	a	c	t	c	c	g	-	c	t	c
KU926317	2	a	t	a	t	a	c	t	t	c	g	c	g	g	c	c	c	c	t	g	c	a	c	g	t	c	c	t	t	c	a	a	t	a	c	a	c	a	t	c	a	g	a	t	g	a	g	t	c	a	t	g	c	g	c	a	t	g	a	a	-	-	c	t	c	a	c	t	c	c	g	-	c	t	c
KY037796	2	a	t	a	t	a	c	c	t	c	g	c	g	g	c	c	c	c	t	g	c	a	c	g	t	c	c	t	t	c	a	a	t	a	c	a	c	a	t	c	a	g	a	t	g	a	g	t	c	a	t	g	c	g	c	a	t	g	a	a	-	-	-	t	c	a	c	t	c	c	g	c	-	t	c
MF004348	2	a	t	a	t	a	c	c	t	c	g	c	g	g	c	c	c	c	t	g	c	a	c	g	t	c	c	t	t	c	a	a	t	a	c	a	c	a	t	c	a	g	a	t	g	a	g	t	c	a	t	g	c	g	c	a	t	g	a	a	-	-	-	t	c	a	c	t	c	c	g	c	-	t	c
MH709309	2	a	t	a	t	a	c	c	t	c	g	c	g	g	c	c	c	c	t	g	c	a	c	g	t	c	c	t	t	c	a	a	t	a	c	a	c	a	t	c	a	g	a	t	g	a	g	t	c	a	t	g	c	g	c	a	t	g	a	a	a	c	t	t	c	a	c	t	c	c	g	c	t	t	c
MH709310	2	a	t	a	t	a	c	c	t	c	g	c	g	g	c	c	c	c	t	g	c	a	c	g	t	c	c	t	t	c	a	a	t	a	c	a	c	a	t	c	a	g	a	t	g	a	g	t	c	a	t	g	c	g	c	a	t	g	a	a	a	a	c	t	c	a	c	t	c	c	g	c	c	t	c
MH709311	2	a	t	a	t	a	c	c	t	c	g	c	g	g	c	c	c	c	g	g	c	a	c	g	t	c	c	t	t	c	a	a	t	a	c	a	c	a	t	c	a	g	a	t	g	a	g	t	c	a	t	g	c	g	c	a	t	g	a	a	a	a	c	t	c	a	c	t	c	c	g	c	t	t	c
MH709313	2	a	t	a	t	a	c	c	t	c	g	c	g	g	c	c	c	c	t	g	c	a	c	g	t	c	c	t	t	c	a	c	t	a	c	a	c	a	t	c	a	g	a	t	g	a	g	t	c	a	t	g	c	g	c	a	t	g	a	a	a	a	c	t	c	a	c	t	c	c	g	c	t	g	c
MH709314	2	a	t	a	t	a	c	c	t	c	g	c	g	g	c	c	c	c	t	g	c	a	c	g	t	c	c	t	t	c	a	a	t	a	c	a	c	a	t	c	a	g	a	t	g	a	g	t	c	a	t	a	c	g	c	a	t	g	a	a	a	c	t	t	c	a	c	t	c	c	g	c	t	t	c
MH709315	2	a	t	a	t	a	c	c	t	c	g	c	g	g	c	c	c	c	t	g	c	a	c	g	t	c	c	t	t	c	a	a	t	a	c	a	c	a	t	c	a	g	a	t	g	a	g	t	c	a	t	a	c	g	c	a	t	g	a	a	a	a	c	t	c	a	c	t	c	c	g	c	c	t	c
MH709319	2	a	t	a	t	a	c	c	t	c	g	c	g	g	c	c	c	c	t	g	c	a	c	g	t	c	c	t	t	c	a	a	t	a	c	a	c	a	t	c	a	g	a	t	g	a	g	t	c	a	t	g	c	g	c	a	t	g	a	a	a	a	c	t	c	a	c	t	c	c	g	t	c	t	c
MH709320	2	a	t	a	t	a	c	c	t	c	g	c	g	g	c	c	c	c	t	g	c	a	c	g	t	c	c	t	t	c	a	a	t	a	c	a	c	a	t	c	a	g	a	t	g	a	g	t	c	a	t	g	c	g	c	a	t	g	a	a	a	a	c	t	c	a	c	t	c	c	g	c	c	t	c
MH709329	2	a	t	a	t	a	c	c	t	c	g	c	g	g	c	c	t	c	t	g	c	a	c	g	t	c	c	t	t	c	a	a	t	a	c	a	c	a	t	c	a	g	a	t	g	a	g	t	c	a	t	a	c	g	c	a	t	g	a	a	a	c	c	t	c	a	c	t	c	c	g	c	t	t	c
MH709331	2	a	t	a	t	a	c	c	t	c	g	c	g	g	c	c	c	c	t	g	c	a	c	g	t	c	c	t	t	c	a	a	t	a	c	a	c	a	t	c	a	g	a	t	g	a	g	t	c	a	t	g	c	g	c	a	t	g	a	a	a	a	c	t	c	a	c	t	c	c	g	c	t	t	c
MH709333	2	a	t	a	t	a	c	c	t	c	g	c	g	g	c	c	c	c	t	g	c	a	c	g	t	c	c	t	t	c	a	a	t	a	c	a	c	a	t	c	a	g	a	t	g	a	g	t	c	a	t	a	c	g	c	a	t	g	a	a	a	a	c	t	c	a	c	t	c	c	g	t	c	t	c
MH709337	2	a	t	a	t	a	c	c	t	c	g	c	g	g	c	c	c	c	t	g	c	a	c	g	t	c	c	t	t	c	a	a	t	a	c	a	c	a	t	c	a	g	a	t	g	a	g	t	c	a	t	g	c	g	c	a	t	g	a	a	a	c	t	t	c	a	c	t	c	c	g	t	c	t	c
MH709339	2	a	t	a	t	a	c	c	t	c	g	c	g	g	c	c	c	c	t	g	c	a	c	g	t	c	c	t	t	c	a	a	t	a	c	a	c	a	t	c	a	g	a	t	g	a	g	t	c	a	t	g	c	g	c	a	t	g	a	a	a	a	c	t	c	a	c	t	c	t	g	c	t	t	c
MH709344	2	a	t	a	t	a	c	c	t	c	g	c	g	g	c	c	c	c	t	g	c	a	c	g	t	c	c	t	t	c	a	a	t	a	c	a	c	a	t	c	g	g	a	t	g	a	g	t	c	a	t	g	c	g	c	a	t	g	a	a	a	a	c	c	c	a	c	t	c	c	g	t	c	t	c
MH709348	2	a	t	a	t	a	c	c	t	c	g	c	g	g	c	c	c	c	t	g	c	a	c	g	t	c	c	t	t	c	a	a	t	a	c	a	c	a	t	c	a	g	a	t	g	a	g	t	c	a	t	g	c	g	c	a	t	g	a	a	a	c	t	t	c	a	c	t	c	c	g	c	c	t	c
MH709355	2	a	t	a	t	a	c	c	t	c	g	c	g	g	c	c	c	c	t	g	c	a	c	g	t	c	c	t	t	c	a	a	t	a	c	a	c	a	t	c	a	g	a	t	g	a	g	t	c	a	t	g	c	g	c	a	t	g	a	a	a	c	t	t	c	a	c	t	c	c	g	t	c	t	c
MH709359	2	a	t	a	t	a	c	c	t	c	g	c	g	g	c	c	c	c	t	g	c	a	c	g	t	c	c	t	t	c	a	a	t	a	c	a	c	a	t	c	a	g	a	t	g	a	a	t	c	a	t	g	c	g	c	a	t	g	a	a	a	a	c	t	c	a	c	t	c	c	g	t	c	t	c
MH709363	2	a	t	a	t	a	c	c	t	c	g	c	g	g	c	c	c	c	t	g	c	a	c	g	t	c	c	t	t	c	a	a	t	a	c	a	c	a	t	c	a	g	a	t	g	a	g	t	c	a	t	g	c	g	c	a	t	g	a	a	a	a	c	t	c	a	c	t	c	c	g	c	c	t	c
MH709366	2	a	t	a	t	a	c	c	t	c	g	c	g	g	c	c	c	c	t	g	c	a	c	g	t	c	c	t	t	c	a	a	t	a	c	a	c	a	t	c	a	g	a	t	g	a	g	t	c	a	t	g	c	g	c	a	t	g	a	a	a	a	c	t	c	a	c	t	c	c	g	t	c	t	c
MH709376	2	a	t	a	t	a	c	c	t	c	g	c	g	g	c	c	c	c	t	g	c	a	c	g	t	c	c	t	t	c	a	a	t	a	c	a	c	a	t	c	a	g	a	t	g	a	g	t	c	a	t	g	c	g	c	a	t	g	a	a	a	c	t	t	c	a	c	t	c	c	g	t	c	t	c
MH709377	2	a	t	a	t	a	c	c	t	c	g	c	g	g	c	c	c	c	t	g	c	a	c	g	t	c	c	t	t	c	a	a	t	a	c	a	c	a	t	c	a	g	a	t	g	a	g	t	c	a	t	g	c	g	c	a	t	g	a	a	a	a	c	t	c	a	c	t	c	c	g	c	t	t	c
**MW316406**	2	a	t	a	t	a	c	c	t	c	g	c	g	g	c	c	c	c	t	g	c	a	c	g	t	c	c	t	g	c	a	a	t	a	c	a	c	a	t	c	a	g	a	t	g	a	g	t	c	a	t	g	c	g	c	a	t	g	a	a	-	-	-	t	c	a	c	t	c	c	g	c	-	t	c
AJ871403	3	a	t	a	t	a	c	c	c	c	g	c	g	g	c	c	c	c	t	g	c	a	c	g	t	a	c	t	t	c	a	a	t	a	c	a	c	a	t	c	a	a	a	t	g	a	g	t	a	a	t	g	c	g	c	a	t	g	a	a	a	a	c	t	c	a	c	t	c	c	g	c	t	t	c
DQ479955	3	a	t	a	t	a	c	c	c	c	g	c	g	g	c	c	c	c	t	g	c	a	c	g	t	c	c	t	t	c	a	a	t	a	c	a	c	a	t	c	a	a	a	t	g	a	g	t	a	a	t	g	c	g	c	a	t	g	a	a	a	a	c	t	c	a	c	t	c	c	g	c	t	t	c
DQ479956	3	a	t	a	t	a	c	c	c	c	g	c	g	g	c	c	c	c	t	g	c	a	a	g	t	c	c	t	t	c	a	a	t	a	c	a	c	a	t	c	a	a	a	t	g	a	g	t	a	a	t	g	c	g	c	a	t	g	a	a	a	a	c	t	c	a	c	t	c	c	a	c	t	t	c
DQ479957	3	a	t	a	t	a	c	c	c	c	g	c	g	g	c	c	c	c	t	g	c	a	a	g	t	c	c	t	t	c	a	a	t	a	c	a	c	a	t	c	a	a	a	t	g	a	g	t	a	a	t	g	c	g	c	a	t	g	a	a	a	a	c	t	c	a	c	t	c	c	g	c	t	t	c
JN704699	3	a	t	a	t	a	c	c	c	c	g	c	g	g	c	c	c	c	t	g	c	a	c	g	t	c	c	t	t	c	a	a	t	a	c	a	c	a	t	c	a	a	a	t	g	a	g	t	a	a	t	g	c	g	c	a	t	g	a	a	a	a	c	t	c	a	c	t	c	c	g	c	t	t	c
JN704700	3	a	t	a	t	a	c	c	c	c	g	c	g	g	c	c	c	c	t	g	c	a	c	g	t	c	c	t	t	c	a	a	t	a	c	a	c	a	t	c	a	a	a	t	g	a	g	t	a	a	t	g	c	g	c	a	t	g	a	a	a	a	c	t	c	a	c	t	c	c	g	c	t	t	t
JN704701	3	a	t	a	t	a	c	c	c	c	g	c	g	g	c	c	c	c	t	g	c	a	c	g	t	c	c	t	t	c	a	a	t	a	c	a	c	a	t	c	a	a	a	t	g	a	g	t	a	a	t	g	c	g	c	a	t	g	a	a	a	a	c	t	c	a	c	t	c	c	g	c	t	t	c
JN704702	3	a	t	a	t	a	c	c	c	c	g	c	g	g	c	c	c	c	t	g	c	a	c	g	t	c	c	t	t	c	a	a	t	a	c	a	c	a	t	c	a	a	a	t	g	a	g	t	a	a	t	g	c	g	c	a	t	g	a	a	a	a	c	t	c	a	c	t	c	c	g	c	t	t	c
JN704703	3	a	t	a	t	a	c	c	c	c	g	c	g	g	c	c	c	c	t	g	c	a	c	g	t	c	c	t	t	c	a	a	t	a	c	a	c	a	t	c	a	a	a	t	g	a	g	t	a	a	t	g	c	g	c	a	t	g	a	a	a	a	c	t	c	a	c	t	c	c	g	c	t	t	c
KP771891	3	a	t	a	t	a	c	c	c	c	g	c	g	g	c	c	c	c	t	g	c	a	c	g	t	c	t	t	t	c	a	a	t	a	c	a	c	a	t	c	a	a	a	t	g	a	g	t	a	a	t	g	c	g	c	a	t	g	a	a	a	a	c	t	c	a	c	t	c	c	g	c	t	t	c
KP771893	3	a	t	a	t	a	c	c	c	c	g	c	g	g	c	c	c	c	t	g	c	a	c	g	t	c	c	t	t	c	a	a	t	a	c	a	c	a	t	c	a	a	a	t	g	a	g	t	a	a	t	g	c	g	c	a	t	g	a	a	a	a	c	t	c	a	c	t	c	c	g	c	t	t	c
KP771895	3	a	t	a	t	a	c	c	c	t	g	c	g	g	c	c	c	c	t	g	a	a	c	g	t	c	c	t	t	c	a	a	t	a	c	a	c	a	t	c	a	a	a	t	g	a	g	t	a	a	t	g	c	g	c	a	t	g	a	a	a	a	c	t	c	a	c	t	c	c	g	c	t	t	c
KP771896	3	a	t	a	t	a	c	c	c	c	g	c	g	g	c	c	c	c	t	g	c	a	c	g	t	c	c	t	t	c	a	a	t	a	c	a	c	a	t	c	a	a	a	t	g	a	g	t	a	a	t	g	c	g	c	a	t	g	a	a	a	a	c	t	c	a	c	t	c	c	g	c	t	t	c
KP771897	3	a	t	a	t	a	c	c	c	c	g	c	g	g	c	c	c	c	t	g	c	a	c	g	t	c	c	t	t	c	a	a	t	a	c	a	c	a	t	c	a	a	a	t	g	a	g	t	a	a	t	g	c	g	c	a	t	g	a	a	a	a	c	t	c	a	c	t	c	c	g	c	t	t	c
KP771899	3	a	t	a	t	a	c	c	c	c	g	c	g	g	c	c	c	c	t	g	c	a	c	g	t	c	c	t	t	c	a	a	t	a	c	a	c	a	t	c	a	a	a	t	g	a	g	t	a	a	t	g	c	g	c	a	t	g	a	a	a	a	c	t	c	a	c	t	c	c	g	c	t	t	c
KP771903	3	a	t	a	t	a	c	c	c	c	g	c	g	g	c	c	c	c	t	g	c	a	c	g	t	c	t	t	t	c	a	a	t	a	c	a	c	a	t	c	a	a	a	t	g	a	g	t	a	a	t	g	c	g	c	a	t	g	a	a	a	a	c	t	c	a	c	t	c	c	g	c	t	t	c
KP771907	3	a	t	a	t	a	c	c	c	c	g	c	g	g	c	c	c	c	t	g	c	a	c	g	t	c	c	t	t	c	a	a	t	a	c	a	c	a	t	c	a	a	a	t	g	a	g	t	a	a	t	g	c	g	c	a	t	g	a	a	a	a	c	t	c	a	c	t	c	c	g	c	t	t	c
KP771910	3	a	t	a	t	a	c	c	c	c	g	c	g	g	c	c	c	c	t	g	c	a	c	g	t	c	c	t	t	c	a	a	t	a	c	a	c	a	t	c	a	a	a	t	g	a	g	t	a	a	t	g	c	g	c	a	t	g	a	g	a	a	c	t	c	a	c	t	c	c	g	c	t	t	c
KP771915	3	a	t	a	t	a	c	c	c	c	g	c	g	g	c	c	c	c	t	g	c	a	c	g	t	c	c	t	t	c	a	a	t	a	c	a	c	a	t	c	a	a	a	t	g	a	g	t	a	a	t	g	c	g	c	a	t	g	a	a	a	a	c	t	c	a	c	t	c	c	g	c	t	t	c
KP771916	3	a	t	a	t	a	c	c	c	c	g	c	g	g	c	c	c	t	t	g	c	a	a	g	t	c	c	t	t	c	a	a	t	a	c	a	c	a	t	c	a	a	a	t	g	a	g	t	a	a	t	g	c	g	c	a	t	g	a	a	a	a	c	t	c	a	c	t	c	c	g	c	t	t	c
KP771921	3	a	t	a	t	a	c	c	c	c	g	c	g	g	c	c	c	c	t	g	c	a	c	g	t	c	c	t	t	c	a	a	t	a	c	a	c	a	t	c	a	a	a	t	g	a	g	t	a	a	t	g	c	g	c	a	t	g	a	a	a	a	c	t	c	a	c	t	c	c	g	c	t	t	c
KP771923	3	a	t	a	t	a	c	c	c	c	g	c	g	g	c	c	c	c	t	g	c	a	c	g	t	c	c	t	t	c	a	a	t	a	c	a	c	a	t	c	a	a	a	t	g	a	g	t	a	a	t	g	c	g	c	a	t	g	a	a	a	a	c	t	c	a	c	t	c	c	g	c	t	t	c
KP771889	3	a	t	a	t	a	c	c	c	c	g	c	g	g	c	c	c	c	t	g	c	a	c	g	t	c	c	t	t	c	a	a	t	a	c	a	c	a	t	c	a	a	a	t	g	a	g	t	a	a	t	g	c	g	c	a	t	g	a	a	a	a	c	t	c	a	c	t	c	c	g	c	t	t	c
KU926311	3	a	t	a	t	g	c	c	c	c	g	c	g	g	c	c	c	c	t	g	c	a	a	g	t	c	c	t	t	c	a	a	t	a	c	a	c	a	t	c	a	a	a	t	g	a	g	t	a	a	t	g	c	g	c	a	t	g	a	a	a	a	c	t	c	a	c	t	c	c	g	c	t	t	c
JQ911599	3	a	t	a	t	a	c	c	c	c	g	c	g	g	c	c	c	c	t	g	c	a	c	g	t	c	c	t	t	c	a	a	t	a	c	a	c	a	t	c	a	a	a	t	g	a	g	t	a	a	t	g	c	g	c	a	t	g	a	a	a	a	c	t	c	a	c	t	c	c	g	c	t	t	c
MH709317	3	a	t	a	t	a	c	c	c	c	g	c	g	g	c	c	c	c	t	g	c	a	c	g	t	c	c	t	t	c	a	a	t	a	c	a	c	a	t	c	a	a	a	t	g	a	g	t	a	a	t	g	c	g	c	a	t	g	a	a	a	a	c	t	c	a	c	t	c	c	g	c	t	t	c
MH709346	3	a	t	a	t	a	c	c	c	c	g	c	g	t	c	c	c	c	t	g	c	a	c	g	t	c	c	t	t	c	a	a	t	a	c	a	c	a	t	c	a	a	a	t	g	a	g	t	a	a	t	g	c	g	c	a	t	g	a	a	a	a	c	t	c	a	c	t	c	c	g	c	t	t	c
MH709375	3	a	t	a	t	a	c	c	c	c	g	c	g	g	c	c	c	c	t	g	c	a	c	g	t	c	c	t	t	c	a	a	t	a	c	a	c	a	t	c	a	a	a	t	g	a	g	t	a	a	t	g	c	g	c	a	t	g	a	a	a	a	c	t	c	a	c	t	c	c	g	c	t	t	c
DQ452050	4	a	t	a	t	a	c	c	c	c	g	t	g	g	c	c	c	c	t	g	c	a	c	a	t	c	c	t	t	c	a	a	t	a	t	a	c	a	t	c	a	g	g	g	g	a	g	t	a	a	t	g	c	g	c	a	t	g	a	a	-	-	-	t	c	a	c	t	c	c	g	c	-	t	c
DQ479960	4	a	t	a	t	a	c	c	c	c	g	t	g	g	c	c	c	c	t	g	c	a	c	a	t	c	c	t	t	c	a	a	t	a	t	a	c	a	t	c	a	g	g	t	g	a	g	t	a	a	t	g	c	g	c	a	t	g	a	a	-	-	-	t	c	c	c	t	c	c	g	c	-	t	c
KP771898	4	a	t	a	t	a	c	c	c	c	g	t	g	g	c	c	c	c	t	g	c	a	c	a	t	c	c	t	t	c	a	a	t	a	t	a	c	a	t	c	a	g	g	t	g	a	g	t	a	a	t	g	c	g	c	a	t	g	a	a	a	a	c	t	c	a	c	t	c	c	g	c	t	t	c
MH709316	4	a	t	a	t	a	a	c	t	c	g	t	g	g	c	c	c	c	t	g	c	a	c	a	t	c	c	t	t	c	a	a	t	a	t	a	c	a	t	c	a	g	g	t	g	a	g	t	a	a	t	g	c	g	c	a	t	g	a	a	a	a	c	t	c	a	c	t	c	c	g	c	t	t	c
MH709318	4	a	t	a	t	a	c	c	c	c	g	t	g	g	c	c	c	c	t	g	c	a	c	a	t	c	c	t	t	c	a	a	t	a	t	a	c	a	t	c	a	g	g	t	g	a	g	t	a	a	t	g	c	g	c	a	t	g	a	a	a	a	c	t	c	a	c	t	c	c	g	t	c	t	c
MH709322	4	a	t	a	t	a	c	c	c	c	g	t	g	g	c	c	c	c	t	g	c	a	c	a	t	c	c	t	t	c	a	a	t	a	t	a	c	a	t	c	a	g	g	t	g	a	g	t	a	a	t	g	c	g	c	a	t	g	a	a	a	c	c	t	c	a	c	t	c	c	g	c	t	t	c
MH709323	4	a	t	a	t	a	c	c	c	c	g	t	g	g	c	c	c	c	t	g	c	a	c	a	t	c	c	t	t	c	a	a	t	a	t	a	c	a	t	c	a	g	g	t	g	a	g	t	a	a	t	g	c	g	c	a	t	g	a	a	a	c	c	t	c	a	c	t	c	c	g	t	c	t	c
MH709326	4	a	t	a	t	a	c	c	c	c	g	t	g	g	c	c	c	c	t	g	c	a	c	a	t	c	c	t	t	c	a	a	t	a	t	a	c	a	t	c	a	g	g	t	g	a	g	t	a	a	t	g	c	g	c	a	t	g	a	a	a	a	c	t	c	a	c	t	c	c	g	c	t	t	c
MH709341	4	a	t	a	t	a	c	c	c	c	g	t	g	g	c	c	c	c	t	g	c	a	c	a	t	c	c	t	t	c	a	a	t	a	t	a	c	a	t	c	a	g	g	t	g	a	g	t	a	a	t	g	c	g	c	a	t	g	a	a	a	a	c	t	c	a	c	t	c	c	g	c	t	t	c
MH709357	4	a	t	a	t	a	c	c	c	c	g	t	g	g	c	c	c	c	t	g	c	a	c	a	t	c	c	t	t	c	a	a	t	a	t	a	c	a	t	c	a	g	g	t	g	a	g	t	a	a	t	g	c	g	c	a	t	g	a	a	a	a	t	t	c	a	c	t	c	c	g	t	c	t	c
MH709364	4	a	t	a	t	a	c	c	c	c	g	t	g	g	c	c	c	c	t	g	c	a	c	a	t	c	c	t	t	c	a	a	t	a	t	a	c	a	t	c	a	g	g	t	g	a	g	t	a	a	t	g	c	g	c	a	t	g	a	a	a	a	c	t	c	a	c	t	c	c	g	c	t	t	c
MH709370	4	a	t	a	t	a	c	c	c	c	g	t	g	g	c	c	c	c	t	g	c	g	c	a	t	c	c	t	t	c	a	a	t	a	t	a	c	a	t	c	a	g	g	t	g	a	g	t	a	a	t	g	c	g	c	a	t	g	a	a	a	a	c	t	c	a	c	t	c	c	g	c	t	t	c
MH709371	4	a	t	a	t	a	c	c	c	c	g	t	g	g	c	c	c	c	t	g	c	a	c	a	t	c	c	t	t	c	a	a	t	a	t	a	c	a	t	c	a	g	g	t	g	a	g	t	a	a	t	g	c	g	c	a	t	g	a	a	a	a	c	t	c	c	c	t	c	t	g	c	t	t	c
MH709372	4	a	t	a	t	a	c	c	c	c	g	t	g	g	c	c	c	c	t	g	c	a	c	a	t	c	c	t	t	c	a	a	t	a	t	a	c	a	t	c	a	g	g	t	g	a	g	t	a	a	t	g	c	g	c	a	t	g	a	a	a	c	c	t	c	a	c	t	c	c	g	c	t	t	c
DQ457052	5	a	t	a	t	a	c	c	c	c	g	c	g	g	c	c	c	c	t	g	c	a	c	g	t	c	c	t	t	c	a	a	t	a	t	a	c	a	t	c	a	g	g	t	g	a	g	t	a	a	t	g	c	g	c	a	t	g	c	a	-	a	c	t	c	a	c	t	c	c	g	c	-	t	c
JN704704	5	a	t	a	t	a	c	c	c	c	g	c	g	g	c	c	c	c	t	g	c	a	c	g	t	c	c	t	t	c	a	a	t	a	t	a	c	a	c	c	a	g	g	t	g	a	g	t	a	a	t	g	c	g	c	a	c	g	c	a	-	-	c	t	c	a	c	t	c	c	g	c	t	t	c
JN704705	5	a	t	a	t	a	c	c	c	c	g	c	g	g	c	c	c	c	t	g	c	a	c	g	t	c	c	t	t	c	a	a	t	a	t	a	c	a	t	c	a	g	g	t	g	a	g	t	a	a	t	g	c	g	c	a	t	g	c	a	-	-	c	t	c	a	c	t	c	c	g	c	t	t	c
JN704706	5	a	t	a	t	a	c	c	c	c	g	c	g	g	c	c	c	c	t	g	c	a	c	g	t	c	c	t	t	c	a	a	t	a	t	a	c	a	t	c	a	g	g	t	g	a	g	t	a	a	t	g	c	g	c	a	t	g	c	a	-	-	c	t	c	a	c	t	c	c	g	c	t	t	c
JN704707	5	a	t	a	t	a	c	c	c	c	g	c	g	g	c	c	c	c	t	g	c	a	c	g	t	c	c	t	t	c	a	a	t	a	t	a	c	a	t	c	a	g	g	t	g	a	g	t	a	a	t	g	c	g	c	a	t	g	c	a	-	-	c	t	c	a	c	t	c	c	g	c	t	t	c
JN704708	5	a	t	a	t	a	c	c	c	c	g	c	g	g	c	c	c	c	t	g	c	a	c	g	t	c	c	t	t	c	a	a	t	a	t	a	c	a	t	c	a	g	g	t	g	a	g	t	a	a	t	g	c	g	c	a	t	g	c	a	-	-	c	t	c	a	c	t	c	c	g	c	t	t	c
KP771904	5	a	t	a	t	a	c	c	c	c	g	c	g	g	c	c	c	c	t	g	c	a	c	g	t	c	c	t	t	c	a	a	t	a	t	a	c	a	t	c	a	g	g	t	g	a	g	t	a	a	t	g	a	g	c	a	t	g	c	a	a	a	c	t	c	a	c	t	c	c	g	c	t	t	c
KP771905	5	a	t	a	t	a	c	c	c	c	g	c	g	g	c	c	c	c	t	g	c	a	c	g	t	c	c	t	t	c	a	a	t	a	t	a	c	a	t	c	a	g	g	t	g	a	g	t	a	a	t	g	a	g	c	a	t	g	c	a	a	a	c	t	c	a	c	t	c	c	g	c	t	t	c
KP771906	5	a	t	a	t	a	c	c	c	c	g	c	g	g	c	c	c	c	t	g	c	a	c	g	t	c	c	t	t	c	a	a	t	a	t	a	c	a	t	c	a	g	g	t	g	a	g	t	a	a	t	g	c	g	c	a	t	g	c	a	a	a	c	t	c	a	c	t	c	c	g	c	t	t	c
KP771919	5	a	t	a	t	a	c	c	c	c	g	c	g	g	c	c	c	c	t	g	c	a	c	g	t	c	c	t	t	c	a	a	t	a	t	a	c	a	t	c	a	g	g	t	g	a	g	t	a	a	t	g	c	g	c	a	t	g	c	a	a	a	c	t	c	a	c	t	c	c	g	c	t	t	c
KP771922	5	g	t	a	t	a	c	c	c	c	g	c	g	g	t	c	c	c	t	g	c	a	c	g	t	c	c	t	t	c	a	a	t	a	t	a	c	a	t	c	a	g	g	t	g	a	g	t	a	a	t	g	c	g	c	a	t	g	c	a	a	a	c	t	c	a	c	t	c	c	g	c	t	t	c
KP771924	5	a	t	a	t	a	c	c	c	c	g	c	g	g	c	c	c	c	t	g	c	a	c	g	t	c	c	t	t	c	a	a	t	a	t	a	c	a	t	c	a	g	g	t	g	a	g	t	a	a	t	g	c	g	c	a	t	g	c	a	a	a	c	t	c	a	c	t	c	c	g	c	t	t	c
KM355696	5	a	t	a	t	a	c	c	c	c	g	c	g	g	c	c	c	c	t	g	c	a	c	g	t	c	c	t	t	c	a	a	t	a	t	a	c	a	t	c	a	g	g	t	g	a	g	t	a	a	t	g	c	g	c	g	t	g	c	a	a	a	c	t	c	a	c	t	c	c	g	c	t	t	c
KM355697	5	a	t	a	t	a	c	c	c	c	g	c	g	g	c	c	c	c	t	g	c	a	c	g	t	c	c	t	t	c	a	a	t	a	t	a	c	a	t	c	a	g	g	t	g	a	g	t	a	a	t	g	c	g	c	g	t	g	c	a	a	a	c	t	c	a	c	t	c	c	g	c	t	t	c
KM355698	5	a	t	a	t	a	c	c	c	c	g	c	g	g	c	c	c	c	t	g	c	a	c	g	t	c	c	t	t	c	a	a	t	a	t	a	c	a	t	c	a	g	g	t	g	a	g	t	a	a	t	g	c	g	c	a	t	g	c	a	a	a	c	t	c	a	c	t	c	c	g	c	t	t	c
KM355699	5	a	t	a	t	a	c	c	c	c	g	c	g	g	c	c	c	c	t	g	c	a	c	g	t	c	c	t	t	c	a	a	t	a	t	a	c	a	t	c	a	g	g	t	g	a	g	t	a	a	t	g	c	g	c	a	t	g	c	a	a	a	c	t	c	a	c	t	c	c	g	c	t	t	c
KM355701	5	a	t	a	t	a	c	c	c	c	g	c	g	g	c	c	c	c	t	g	c	a	c	g	t	c	c	t	t	c	a	a	t	a	t	a	c	a	t	c	a	g	g	t	g	a	g	t	a	a	t	g	c	g	c	g	t	g	c	a	a	a	c	t	c	a	c	t	c	c	g	c	t	t	c
KM355702	5	a	t	a	t	a	c	c	c	c	g	c	g	g	c	c	c	c	t	g	c	a	c	g	t	c	c	t	t	c	a	a	t	a	t	a	c	a	t	c	a	g	g	t	g	a	g	t	a	a	t	g	c	g	c	a	t	g	c	a	a	a	c	t	c	a	c	t	c	c	g	c	t	t	c
KM355703	5	a	t	a	t	a	c	c	c	c	g	c	g	g	c	c	c	c	t	g	c	a	c	g	t	c	c	t	t	c	a	a	t	a	t	a	c	a	t	c	a	g	g	t	g	a	g	t	a	a	t	g	c	g	c	g	t	g	c	a	a	a	c	t	c	a	c	t	c	c	g	c	t	t	c
KM355704	5	a	t	a	t	a	c	c	c	c	g	c	g	g	c	c	c	c	t	g	c	a	c	g	t	c	c	t	t	c	a	a	t	a	t	a	c	a	t	c	a	g	g	t	g	a	g	t	a	a	t	g	c	g	c	g	t	g	c	a	a	a	c	t	c	a	c	t	c	c	g	c	t	t	c
KM355705	5	a	t	a	t	a	c	c	c	c	g	c	g	g	c	c	c	c	t	g	c	a	c	g	t	c	c	t	t	c	a	a	t	a	t	a	c	a	t	c	a	g	g	t	g	a	g	t	a	a	t	g	c	g	c	a	t	g	c	a	a	a	c	t	c	a	c	t	c	c	g	c	t	t	c
KM355706	5	a	t	a	t	a	c	c	c	c	g	c	g	g	c	c	c	c	t	g	c	a	c	g	t	c	c	t	t	c	a	a	t	a	t	a	c	a	t	c	a	g	g	t	g	a	g	t	a	a	t	g	c	g	c	a	t	g	c	a	a	a	c	t	c	a	c	t	c	c	g	c	t	t	c
KM355707	5	a	t	a	t	a	c	c	c	c	g	c	g	g	c	c	c	c	t	g	c	a	c	g	t	c	c	t	t	c	a	a	t	a	t	a	c	a	t	c	a	g	g	t	g	a	g	t	a	a	t	g	c	g	c	g	t	g	c	a	a	a	c	t	c	a	c	t	c	c	g	c	t	t	c
KM355708	5	a	t	a	t	a	c	c	c	c	g	c	g	g	c	c	c	c	t	g	c	a	c	g	t	c	c	t	t	c	a	a	t	a	t	a	c	a	t	c	a	g	g	t	g	a	g	t	a	a	t	g	c	g	c	g	t	g	c	a	a	a	c	t	c	a	c	t	c	c	g	c	t	t	c
KM355709	5	a	t	a	t	a	c	c	c	c	g	c	g	g	c	c	c	c	t	g	c	a	c	g	t	c	c	t	t	c	a	a	t	a	t	a	c	a	t	c	a	g	g	t	g	a	g	t	a	a	t	g	c	g	c	a	t	g	c	a	a	a	c	t	c	a	c	t	c	c	g	c	t	t	c
KM355711	5	a	t	a	t	a	c	c	c	c	g	c	g	g	c	c	c	c	t	g	c	a	c	g	t	c	c	t	t	c	a	a	t	a	t	a	c	a	t	c	a	g	g	t	g	a	g	t	a	a	t	g	c	g	c	a	t	g	c	a	a	a	c	t	c	a	c	t	c	c	g	c	t	t	c
KM355712	5	a	t	a	t	a	c	c	c	c	g	c	g	g	c	c	c	c	t	g	c	a	c	g	t	c	c	t	t	c	a	a	t	a	t	a	c	a	t	c	a	g	g	t	g	a	g	t	a	a	t	g	c	g	c	a	t	g	c	a	a	a	c	t	c	a	c	t	c	c	g	c	t	t	c
[illegible]	5	a	t	a	t	a	c	c	c	c	g	c	g	g	c	c	c	c	t	g	c	a	c	g	t	c	c	t	t	c	a	a	t	a	t	a	c	a	t	c	a	g	g	t	g	a	g	t	a	a	t	g	c	g	c	a	t	g	c	a	[illegible]	[illegible]	[illegible]	[illegible]	[illegible]	[illegible]	[illegible]	[illegible]	[illegible]	[illegible]	[illegible]	[illegible]	[illegible]	[illegible]	[illegible]

Supplementary Table 3: 2880 SN

GB-ID	Clade markers																												SD14								
	Position in Dumas	50671	50673	50710	50801	50823	50920	51036	51168	51183	51190	51200	51206	51334	51510	51606	51609	51681	51687	51696	51714	51720	51762	51840	51920	51929	51940	52114	52152	52162	52192	52209	52237	52362	52365	52497	52588
MH499467	5	a	t	a	t	a	c	c	c	c	g	c	g	g	c	c	c	c	t	g	c	a	c	g	t	c	c	t	t	c	a	a	t	a	t	a	c
MH499469	5	a	t	a	t	a	c	c	c	c	g	c	g	g	c	c	c	c	t	g	c	a	c	g	t	c	c	t	t	c	a	a	t	a	t	a	c
MH709321	5	a	t	a	t	a	c	c	c	c	g	c	g	g	c	c	c	c	t	g	c	a	c	g	t	c	c	t	t	c	a	a	t	a	t	a	c
MH709324	5	a	t	a	t	a	c	c	c	c	g	c	g	g	c	c	c	c	t	g	c	a	c	g	t	c	c	t	t	c	a	a	t	a	t	a	c
MH709325	5	a	t	a	t	a	c	c	c	c	g	c	g	g	c	c	c	c	t	g	c	a	c	g	t	c	c	t	t	c	a	a	t	a	t	a	c
MH709327	5	a	t	a	t	a	c	c	c	c	g	c	g	g	c	c	c	c	t	g	c	a	c	g	t	c	c	t	t	c	a	a	t	a	t	a	c
MH709328	5	a	t	a	t	a	c	c	c	c	g	c	g	g	c	c	c	c	t	g	c	a	c	g	t	c	c	t	t	c	a	a	t	a	t	a	c
MH709330	5	a	t	a	t	a	c	c	c	c	g	c	g	g	c	c	c	c	t	g	c	a	c	g	t	c	c	t	t	c	a	a	t	a	t	a	c
MH709332	5	a	t	a	t	a	c	c	c	c	g	c	g	g	c	c	c	c	t	g	c	a	c	g	t	c	c	t	t	c	a	a	t	a	t	a	c
MH709334	5	a	t	a	t	a	c	c	c	c	g	c	g	g	c	c	c	c	t	g	c	a	c	g	t	c	c	t	t	c	a	a	t	a	t	a	c
MH709335	5	a	t	a	t	a	c	c	c	c	g	c	g	g	c	c	c	c	t	g	c	a	c	g	t	c	c	t	t	c	a	a	t	a	t	a	c
MH709336	5	a	t	a	t	a	c	c	c	c	g	c	g	g	c	c	c	c	t	g	c	a	c	g	t	c	c	t	t	c	a	a	t	a	t	a	c
MH709338	5	a	t	a	t	a	c	c	c	c	g	c	g	g	c	c	c	c	t	g	c	a	c	g	t	c	c	t	t	c	a	a	t	a	t	a	c
MH709340	5	a	t	a	t	a	c	c	c	c	g	c	g	g	c	c	c	c	t	a	c	a	c	g	t	c	c	t	t	c	a	a	t	a	t	a	c
MH709342	5	a	t	a	t	a	c	c	c	c	g	c	g	g	c	c	c	c	t	g	c	a	c	g	t	c	c	t	t	c	a	a	t	a	t	a	c
MH709343	5	a	t	a	t	a	c	c	c	c	g	c	g	g	c	c	c	c	t	g	c	a	c	g	t	c	c	t	t	c	a	a	t	a	t	a	c
MH709345	5	a	t	a	t	a	c	c	c	c	g	c	g	g	c	c	c	c	t	g	c	a	c	g	t	c	c	t	t	c	a	a	t	a	t	a	c
MH709347	5	a	t	a	t	a	c	c	c	c	g	c	g	g	c	c	c	c	t	g	c	a	c	g	t	c	c	t	t	a	a	a	t	a	t	a	c
MH709349	5	a	t	a	t	a	c	c	c	c	g	c	g	g	c	c	c	c	t	g	c	a	c	g	t	c	c	t	t	c	a	a	t	a	t	a	c
MH709350	5	a	t	a	g	a	c	c	c	c	g	c	g	g	c	c	c	c	t	g	c	a	c	g	t	c	c	t	t	c	a	a	t	a	t	a	c
MH709351	5	a	t	a	t	a	c	c	c	c	g	c	g	g	c	c	c	c	t	g	c	a	c	g	t	c	c	t	t	c	a	a	t	a	t	a	c
MH709353	5	a	t	c	t	a	c	c	c	c	g	c	g	g	c	c	c	c	t	g	c	a	c	g	t	c	c	t	t	c	a	a	t	a	t	a	c
MH709358	5	a	t	a	t	a	c	c	c	c	g	c	g	g	c	c	c	c	t	g	c	a	c	g	t	c	c	t	t	c	a	a	t	a	t	a	c
MH709360	5	a	t	a	t	a	c	c	c	c	g	c	g	g	c	c	c	c	t	g	c	a	c	g	t	c	c	t	t	c	a	a	t	a	t	a	c
MH709362	5	a	t	a	t	a	c	c	c	c	g	c	g	g	c	c	c	c	t	g	c	a	c	g	t	c	c	t	t	c	a	a	t	a	t	a	c
MH709365	5	a	t	a	t	a	c	c	c	c	g	c	g	g	c	c	c	c	t	g	c	a	c	g	t	c	c	t	t	c	a	a	t	a	t	a	c
MH709373	5	a	t	a	t	a	c	c	c	c	g	c	g	g	c	c	c	c	t	g	c	a	c	g	t	c	c	t	t	c	a	a	t	a	t	a	c
MH709374	5	a	t	a	t	a	c	c	c	c	g	c	g	g	c	c	c	c	t	g	c	a	c	g	t	c	c	t	t	c	a	a	t	a	t	a	c
KC112914	6	a	t	a	t	a	c	c	c	c	g	c	g	g	c	c	c	c	t	g	c	a	c	g	t	c	c	t	t	c	a	a	t	a	c	g	c
KP771914	6	a	t	a	t	a	c	c	c	c	g	c	g	g	c	c	c	c	t	g	c	a	c	g	t	c	c	t	t	c	a	a	t	a	c	g	c
KP702725	6	a	t	a	t	a	c	c	c	c	g	c	g	g	c	c	c	c	t	g	c	a	c	g	t	c	c	t	t	c	a	a	t	a	c	g	c
KP771890	9	a	t	a	t	a	c	c	c	c	g	c	g	g	c	c	c	c	t	g	c	a	c	g	t	c	c	t	t	c	a	a	t	a	c	a	c
JN704710	9	a	t	a	t	a	c	c	c	c	g	c	g	g	c	c	c	c	t	g	c	a	c	g	t	c	c	t	t	c	a	a	t	a	c	a	c
KY037798	9	a	t	a	t	a	c	c	c	t	g	c	g	g	c	c	c	c	t	g	c	a	c	g	t	c	c	t	t	c	a	a	t	a	c	a	c
MH709312	9	a	t	a	t	a	c	c	c	t	g	c	g	g	c	c	c	c	t	g	c	a	c	g	t	c	c	t	t	c	a	a	t	a	c	a	c
MH709352	9	a	t	a	t	a	c	c	c	t	g	c	g	g	c	c	c	c	t	g	c	a	c	g	t	c	c	t	t	c	a	a	t	a	c	a	c
AB097933	Vac	a	t	a	t	a	c	c	t	c	g	c	g	g	c	c	c	c	t	g	c	a	c	g	t	c	c	t	t	c	a	a	t	a	c	a	c
JN704698	Vac	a	t	a	t	a	c	c	t	c	g	c	g	g	c	c	c	c	t	g	c	a	c	g	t	c	c	t	t	c	a	a	t	a	c	a	c
JN704697	Vac	a	t	a	t	a	c	c	t	c	g	c	g	g	c	c	c	c	t	g	c	a	c	g	t	c	c	t	t	c	a	a	t	a	c	a	c
DQ008355	Vac	a	t	a	t	a	c	c	t	c	g	c	g	g	c	c	c	c	t	g	c	a	c	g	t	c	c	t	t	c	a	a	t	a	c	a	c
JF306641	Vac	a	t	a	t	a	c	c	t	c	g	c	g	g	c	c	c	c	t	g	c	a	c	g	t	c	c	t	t	c	a	a	t	a	c	a	c
AB097932	Vac	a	t	a	t	a	c	c	t	c	g	c	g	g	c	c	c	c	t	g	c	a	c	g	t	c	c	t	t	c	a	a	t	a	c	a	c
DQ008354	Vac	a	t	a	t	a	c	c	t	c	g	c	g	g	c	c	c	c	t	g	c	a	c	g	t	c	c	t	t	c	a	a	t	a	c	a	c
KU926312	Vac	a	t	a	t	a	c	c	t	c	g	c	g	g	c	c	c	c	t	g	c	a	c	g	t	c	c	t	t	c	a	a	t	a	c	a	c
KU926313	Vac	a	t	a	t	a	c	c	t	c	g	c	g	g	c	c	c	c	t	g	c	a	c	g	t	c	c	t	t	c	a	a	t	a	c	a	c
KU926314	Vac	a	t	a	t	a	c	c	t	c	g	c	g	g	c	c	c	c	t	g	c	a	c	g	t	c	c	t	t	c	a	a	t	a	c	a	c
KF558371	Vac	a	t	a	t	a	c	c	t	c	g	c	g	g	c	c	c	c	t	g	c	a	c	g	t	c	c	t	t	c	a	a	t	a	c	a	c
KF558372	Vac	a	t	a	t	a	c	c	t	c	g	c	g	g	c	c	c	c	t	g	c	a	c	g	t	c	c	t	t	c	a	a	t	a	c	a	c
KF558373	Vac	a	t	a	t	a	c	c	t	c	g	c	g	g	c	c	c	c	t	g	c	a	c	g	t	c	c	t	t	c	a	a	t	a	c	a	c
KF558374	Vac	a	t	a	t	a	c	c	t	c	g	c	g	g	c	c	c	c	t	g	c	a	c	g	t	c	c	t	t	c	g	a	t	a	c	a	c
KF558375	Vac	a	t	a	t	a	c	c	t	c	g	c	g	g	c	c	c	c	t	g	c	a	c	g	t	c	c	t	t	c	a	a	t	a	c	a	c
KF558376	Vac	a	t	a	t	a	c	c	t	c	a	c	g	g	c	c	c	c	t	g	c	a	c	g	t	c	c	t	t	c	g	a	t	a	c	a	c
KF558377	Vac	a	t	a	t	a	c	c	t	c	g	c	a	g	c	c	c	c	t	g	c	a	c	g	t	c	c	t	t	c	a	a	t	a	c	a	c
KF558378	Vac	a	t	a	t	a	c	c	t	c	g	c	g	g	c	c	c	c	t	g	c	a	c	g	t	c	c	t	t	c	a	a	t	a	c	a	c
KF558379	Vac	a	t	a	t	a	c	c	t	c	g	c	g	g	c	c	c	c	t	g	c	a	c	g	t	c	c	t	t	c	g	a	t	a	c	a	c
KF558380	Vac	a	t	a	t	a	c	c	t	c	g	c	g	g	c	c	c	c	t	g	c	a	c	g	t	c	c	t	t	c	a	a	t	a	c	a	c
KF558381	Vac	a	t	a	t	a	c	c	t	c	g	c	g	g	c	c	c	c	t	g	c	a	c	g	t	c	c	t	t	c	g	a	t	a	c	a	c
KF558382	Vac	a	t	a	t	a	c	c	t	c	g	c	g	g	c	c	c	c	t	g	c	a	c	g	t	c	c	t	t	c	a	a	t	a	c	a	c
KF558383	Vac	a	t	a	t	a	c	c	t	c	g	c	g	g	c	c	c	c	t	g	c	a	c	g	t	c	c	t	t	c	a	a	t	a	c	a	c
KF558384	Vac	a	t	a	t	a	c	c	t	c	g	c	g	g	c	c	c	c	t	g	c	a	c	g	t	c	c	t	t	c	a	a	t	a	c	a	c
KF558385	Vac	a	t	a	t	a	c	c	t	c	g	c	g	g	c	c	c	c	t	g	c	a	c	g	t	c	c	t	t	c	a	a	t	a	c	a	c
KF558386	Vac	a	t	a	t	a	c	c	t	c	g	c	g	g	c	c	c	c	t	g	c	a	c	g	t	c	c	t	t	c	g	a	t	a	c	a	c
KF558387	Vac	a	t	a	t	a	c	c	t	c	g	c	g	g	c	c	c	c	t	g	c	a	c	g	t	c	c	t	t	c	a	a	t	a	c	a	c
KF558388	Vac	a	t	a	t	a	c	c	t	c	g	c	g	g	c	c	c	c	t	g	c	a	c	g	t	c	c	t	t	c	a	a	t	a	c	a	c
KF558389	Vac	a	t	a	t	a	c	c	t	c	g	c	g	g	c	c	c	c	t	g	c	a	c	g	t	c	c	t	t	c	a	a	t	a	c	a	c
KF558390	Vac	a	t	a	t	a	c	c	t	c	g	c	g	g	c	c	c	c	t	g	c	a	c	g	t	c	c	t	t	c	a	a	t	a	c	a	c
KF558391	Vac	a	t	a	t	a	c	c	t	c	g	c	g	g	c	c	c	c	t	g	c	a	c	g	t	c	c	t	t	c	a	a	t	a	c	a	c
KF811485	Vac	a	t	a	t	a	c	c	t	c	g	c	g	g	c	c	c	c	t	g	c	a	c	g	t	c	c	t	t	c	a	a	t	a	c	a	c
KF853225	Vac	a	t	a	t	a	c	c	t	c	g	c	g	g	c	c	c	c	t	g	c	a	c	g	t	c	c	t	t	c	a	a	t	a	c	a	c
KF853226	Vac	a	t	a	t	a	c	c	t	c	g	c	g	g	c	c	c	c	t	g	c	a	c	g	t	c	c	t	t	c	a	a	t	a	c	a	c
KF853227	Vac	a	t	a	t	a	c	c	t	c	g	c	g	g	c	c	c	c	t	g	c	a	c	g	t	c	c	t	t	c	a	a	t	a	c	a	c
KF853228	Vac	a	t	a	t	a	c	c	t	c	g	c	g	g	c	c	c	c	t	g	c	a	c	g	t	c	c	t	t	c	a	a	t	a	c	a	c
KF853229	Vac	a	t	a	t	a	c	c	t	c	g	c	g	g	c	c	c	c	t	g	c	a	c	g	t	c	c	t	t	c	a	a	t	a	c	a	c
KF853230	Vac	a	t	a	t	a	c	c	t	c	g	c	g	g	c	c	c	c	t	g	c	a	c	g	t	c	c	t	t	c	a	a	t	a	c	a	c
KF853231	Vac	a	t	a	t	a	c	c	t	c	g	c	g	g	c	c	c	c	t	g	c	a	c	g	t	c	c	t	t	c	g	a	t	a	c	a	c
KF853232	Vac	a	t	a	t	a	c	c	t	c	g	c	g	g	c	c	c	c	t	g	c	a	c	g	t	c	c	t	t	c	a	a	t	a	c	a	c
KF853233	Vac	a	t	a	t	a	c	c	t	c	g	c	g	g	c	c	c	c	t	g	c	a	c	g	t	c	c	t	t	c	a	a	t	a	c	a	c
KF853234	Vac	a	t	a	t	a	c	c	t	c	g	c	g	g	c	c	c	c	t	g	c	a	c	g	t	c	c	t	t	c	a	a	t	a	c	a	c
KF853235	Vac	a	t	a	t	a	c	c	t	c	g	c	g	g	c	c	c	c	t	g	c	a	c	g	t	c	c	t	t	c	a	a	t	a	c	a	c
MF898328	Vac	a	t	a	t	a	c	c	t	c	g	c	g	g	c	c	c	c	t	g	c	a	c	g	t	c	c	t	t	c	a	a	t	a	c	a	c
JN704709	VIII	a	t	a	t	a	c	c	c	c	g	t	g	g	c	c	c	c	t	g	c	a	c	a	t	c	c	t	t	c	a	a	t	a	c	g	t

GB-ID																						5																
	52644	52761	52796	52833	52917	52977	53068	53082	53139	53304	53436	53482	53523	53595	53938	53967	53993	54071	54096	54132	54139	54231	54264	54351	54359	54360	54362	54371	54393	54435	54474	54485	54529	54550	54555	54556	54562	54564
MH499467	a	t	c	a	g	g	t	a	a	g	t	a	a	t	g	c	g	c	a	t	g	c	a	a	a	c	t	c	a	c	t	c	c	g	c	-	t	c
MH499469	a	t	c	a	g	g	t	g	a	g	t	a	a	t	g	c	g	c	a	t	g	c	a	a	a	c	t	c	a	c	t	c	c	g	c	-	t	c
MH709321	a	t	c	a	g	g	t	g	a	g	t	a	a	t	g	c	g	c	a	t	g	c	a	a	a	c	t	c	a	c	t	c	c	g	c	t	t	c
MH709324	a	t	c	a	g	g	t	g	a	g	t	a	a	t	g	c	g	c	a	t	g	c	a	a	a	c	t	c	a	c	c	c	c	g	c	c	t	c
MH709325	a	t	c	a	g	g	t	g	a	g	t	a	a	t	g	c	g	c	a	t	g	c	a	a	a	c	t	c	a	c	t	c	c	g	c	c	t	c
MH709327	c	t	c	a	g	g	t	g	a	g	t	a	a	t	g	c	g	t	a	t	g	c	a	a	c	c	t	c	a	c	t	c	c	g	t	c	t	c
MH709328	a	t	c	a	g	g	t	g	a	g	t	a	a	t	g	c	g	c	a	t	g	c	a	a	a	c	t	c	a	c	t	c	c	g	c	-	t	c
MH709330	a	t	c	a	g	g	t	g	a	g	t	a	a	t	g	c	g	c	a	t	g	c	a	a	a	c	t	c	a	c	t	c	c	g	t	c	t	c
MH709332	a	t	c	a	g	g	t	g	a	g	t	a	a	t	g	c	g	c	a	t	g	c	a	a	a	c	t	c	a	c	t	c	c	g	t	c	t	c
MH709334	a	t	c	a	g	g	t	g	a	g	t	a	a	t	g	c	g	c	a	t	g	c	a	a	a	c	t	c	a	c	t	c	c	g	t	c	t	c
MH709335	a	t	c	a	g	g	t	g	a	g	t	a	a	t	g	c	g	c	a	t	g	c	a	a	a	c	t	c	a	c	t	c	c	g	c	c	t	c
MH709336	a	t	c	a	g	g	t	g	a	g	t	a	a	t	g	c	g	c	a	t	g	c	a	a	c	t	t	c	a	c	t	c	c	g	-	-	t	c
MH709338	a	t	c	a	g	g	t	g	a	g	t	a	a	t	g	c	g	c	a	t	g	c	a	a	c	c	t	c	a	c	t	c	c	g	c	c	t	c
MH709340	a	t	c	a	g	g	t	g	a	g	t	a	a	t	g	c	g	c	a	t	g	c	a	a	a	c	t	c	a	c	t	c	c	g	c	t	t	c
MH709342	a	t	c	a	g	g	t	g	a	g	t	a	a	t	g	c	g	c	a	t	g	c	a	a	c	c	t	c	a	c	t	c	c	g	t	c	t	c
MH709343	a	t	c	a	g	g	t	g	a	g	t	a	a	t	g	c	g	c	a	t	g	c	a	a	a	c	t	c	a	c	t	c	c	g	t	c	t	c
MH709345	a	t	c	a	g	g	t	g	a	g	t	a	a	t	g	c	g	c	a	t	g	c	a	a	c	c	t	c	a	c	t	c	c	g	c	t	t	c
MH709347	a	t	c	a	g	g	t	g	a	g	t	a	a	t	g	c	g	c	a	t	g	c	a	a	c	c	t	c	a	c	t	t	c	g	t	c	t	c
MH709349	a	t	c	a	g	g	t	g	a	g	t	a	a	t	g	c	g	c	a	t	g	c	a	a	a	c	t	c	a	c	t	c	c	g	c	t	t	c
MH709350	a	t	c	a	g	g	t	g	a	g	t	a	a	t	g	c	g	c	a	t	g	c	a	a	a	c	t	c	a	c	t	c	c	g	c	-	t	c
MH709351	a	t	c	a	g	g	t	g	a	g	t	a	a	t	g	c	g	c	a	t	g	c	a	a	a	c	t	c	a	c	t	c	c	g	c	t	t	c
MH709353	a	t	c	a	g	g	t	g	a	g	t	a	a	t	g	c	g	c	a	t	g	c	a	a	a	c	t	c	a	c	t	c	c	g	c	c	t	c
MH709358	a	t	c	a	g	g	t	g	a	g	t	a	a	t	g	c	g	c	a	t	g	c	a	a	a	c	t	c	a	c	t	c	c	g	c	t	t	c
MH709360	a	t	c	a	g	g	t	g	a	g	t	a	a	t	g	c	g	c	a	t	g	c	a	a	a	c	t	c	a	c	t	c	c	g	c	t	t	c
MH709362	a	t	c	a	g	g	t	g	a	g	t	a	a	t	g	c	g	c	a	t	g	c	a	a	a	c	t	c	a	c	t	c	c	g	c	c	t	c
MH709365	a	t	c	a	g	g	t	g	a	g	t	a	a	t	g	c	g	c	a	t	g	c	a	a	a	c	t	c	a	c	t	c	c	g	-	-	t	c
MH709373	a	t	c	a	g	g	t	g	a	g	t	a	a	t	g	c	g	c	a	t	g	c	a	a	a	c	t	c	a	c	t	c	c	g	c	t	t	c
MH709374	a	t	c	a	g	g	t	g	a	g	t	a	a	t	g	c	g	c	a	t	g	c	a	a	a	c	t	c	a	c	t	c	c	g	c	t	t	c
KC112914	a	t	c	a	g	g	t	g	a	g	t	a	a	t	g	c	g	c	a	t	g	a	a	-	a	c	t	c	a	c	c	c	c	g	c	t	t	c
KP771914	a	t	c	a	g	g	t	g	a	g	t	a	a	t	g	c	g	c	a	t	g	a	a	a	a	c	t	c	a	c	t	c	c	g	c	t	t	c
KP702725	a	t	c	a	g	g	t	g	a	g	t	a	a	t	g	c	g	c	a	t	g	a	a	-	a	c	t	c	a	c	t	c	c	g	c	t	t	c
KP771890	a	t	t	a	a	a	t	g	a	g	t	a	a	t	g	c	g	c	a	t	g	a	a	a	a	c	t	c	a	c	t	c	c	g	c	t	t	c
JN704710	a	t	c	a	a	a	t	g	a	g	t	a	a	t	g	c	g	c	a	t	g	a	a	a	a	c	t	c	a	c	t	c	c	g	c	t	t	t
KY037798	a	t	c	a	a	a	t	g	a	g	t	a	a	t	g	c	g	c	a	t	g	a	a	a	a	c	t	c	a	c	t	c	c	g	c	t	t	c
MH709312	a	t	c	a	a	a	t	g	a	g	t	a	a	t	g	c	g	c	a	t	g	a	a	a	a	c	t	c	a	c	t	c	c	g	c	t	t	c
MH709352	a	t	c	a	a	a	t	g	a	g	t	a	a	t	g	c	g	c	a	t	g	a	a	a	a	c	t	t	a	c	t	c	c	g	c	t	t	c
AB097933	a	t	c	a	g	a	t	g	a	g	t	c	a	t	a	c	g	c	a	t	g	a	a	-	-	c	t	c	a	c	t	c	c	g	-	c	t	c
JN704698	a	t	c	a	g	a	t	g	a	g	t	c	a	t	a	c	g	c	a	t	g	a	a	-	-	-	t	c	a	c	t	c	c	g	c	t	t	c
JN704697	a	t	c	a	g	a	t	g	a	g	t	c	a	t	a	c	g	c	a	t	g	a	a	a	a	c	t	c	a	c	t	c	c	g	c	t	t	c
DQ008355	a	t	c	a	g	a	t	g	a	g	t	c	a	t	a	c	g	c	a	t	g	a	a	-	-	c	t	c	a	c	t	c	c	g	-	c	t	c
JF306641	a	t	c	a	g	a	t	g	a	g	t	c	a	t	a	c	g	c	a	t	g	a	a	-	-	c	t	c	a	c	t	c	c	g	-	c	t	c
AB097932	a	t	c	a	g	a	t	g	a	g	t	c	a	t	a	c	g	c	a	t	g	a	a	-	-	c	t	c	a	c	t	c	c	g	-	c	t	c
DQ008354	a	t	c	a	g	a	t	g	a	g	t	c	a	t	a	c	g	c	a	t	g	a	a	-	-	c	t	c	a	c	t	c	c	g	-	c	t	c
KU926312	a	t	c	a	g	a	t	g	a	g	t	c	a	t	a	c	g	c	a	t	g	a	a	-	-	c	t	c	a	c	t	c	c	g	-	c	t	c
KU926313	a	t	c	a	g	a	t	g	a	g	t	c	a	t	a	c	g	c	a	t	g	a	a	-	-	c	t	c	a	c	t	c	c	g	-	c	t	c
KU926314	a	t	c	a	g	a	t	g	a	g	t	c	a	t	a	c	g	c	a	t	g	a	a	-	-	c	t	c	a	c	t	c	c	g	-	c	t	c
KF558371	a	t	c	a	g	a	t	g	a	g	t	c	a	t	a	c	g	c	a	t	g	a	a	-	-	c	t	c	a	c	t	c	c	g	-	c	t	c
KF558372	a	t	c	a	g	a	t	g	a	g	t	c	a	t	a	c	g	c	a	t	g	a	a	-	-	c	t	c	a	c	t	c	c	g	-	c	t	c
KF558373	a	t	c	a	g	a	t	g	a	g	t	c	a	t	a	c	g	c	a	t	g	a	a	-	-	c	t	c	a	c	t	c	c	g	-	c	t	c
KF558374	a	t	c	a	g	a	t	g	a	g	t	c	a	t	a	c	g	c	a	t	g	a	a	-	-	c	t	c	a	c	t	c	c	g	-	c	t	c
KF558375	a	t	c	a	g	a	t	g	a	g	t	c	a	t	a	c	g	c	a	t	g	a	a	-	-	c	t	c	a	c	t	c	c	g	-	c	t	c
KF558376	a	t	c	a	g	a	t	g	a	g	t	c	a	t	a	c	g	c	a	t	g	a	a	-	-	c	t	c	a	c	t	c	c	g	-	c	t	c
KF558377	a	t	c	a	g	a	t	g	a	g	t	c	a	t	a	c	a	c	a	t	g	a	a	-	-	c	t	c	a	c	t	c	c	g	-	c	t	c
KF558378	a	t	c	a	g	a	t	g	a	g	t	c	a	t	a	c	g	c	a	t	g	a	a	-	-	c	t	c	a	c	t	c	c	g	-	c	t	c
KF558379	a	t	c	a	g	a	t	g	a	g	t	c	a	t	a	c	g	c	a	t	g	a	a	-	-	c	t	c	a	c	t	c	c	g	-	c	t	c
KF558380	a	t	c	a	g	a	t	g	a	g	t	c	a	t	a	c	g	c	a	t	g	a	a	-	-	c	t	c	a	c	t	c	c	g	-	c	t	c
KF558381	a	t	c	a	g	a	t	g	a	g	t	c	a	t	a	c	g	c	a	t	g	a	a	-	-	c	t	c	a	c	t	c	c	g	-	c	t	c
KF558382	a	t	c	a	g	a	t	g	a	g	t	c	a	t	a	c	g	c	a	t	g	a	a	-	-	c	t	c	a	c	t	c	c	g	-	c	t	c
KF558383	a	t	c	a	g	a	t	g	a	g	t	c	a	t	a	c	g	c	a	t	g	a	a	-	-	c	t	c	a	c	t	c	c	g	-	c	t	c
KF558384	a	t	c	a	g	a	t	g	a	g	t	c	a	t	a	c	g	c	a	t	g	a	a	-	-	c	t	c	a	c	t	c	c	g	-	c	t	c
KF558385	a	t	c	a	g	a	t	g	a	g	t	c	a	t	a	c	g	c	a	t	g	a	a	-	-	c	t	c	a	c	t	c	c	g	-	c	t	c
KF558386	a	t	c	a	g	a	t	g	a	g	t	c	a	t	a	c	g	c	a	t	g	a	a	-	-	c	t	c	a	c	t	c	c	g	-	c	t	c
KF558387	a	t	c	a	g	a	t	g	a	g	t	c	a	t	a	c	g	c	a	t	g	a	a	-	-	c	t	c	a	c	t	c	c	g	-	c	t	c
KF558388	a	t	c	a	g	a	t	g	a	g	t	c	a	t	a	c	g	c	a	t	g	a	a	-	-	c	t	c	a	c	t	c	c	g	-	c	t	c
KF558389	a	t	c	a	g	a	t	g	a	g	t	c	a	t	a	c	g	c	a	t	g	a	a	-	-	c	t	c	a	c	t	c	c	g	-	c	t	c
KF558390	a	t	c	a	g	a	t	g	a	g	t	c	a	t	a	c	g	c	a	t	g	a	a	-	-	c	t	c	a	c	t	c	c	g	-	c	t	c
KF558391	a	t	c	a	g	a	t	g	a	g	t	c	a	t	a	c	g	c	a	t	g	a	a	-	-	c	t	c	a	c	t	c	c	g	-	c	t	c
KF811485	a	t	c	a	g	a	t	g	a	g	t	c	a	t	a	c	g	c	a	t	g	a	a	-	-	c	t	c	a	c	t	c	c	g	-	c	t	c
KF853225	a	t	c	a	g	a	t	g	a	g	t	c	a	t	a	c	g	c	a	t	g	a	a	-	-	c	t	c	a	c	t	c	c	g	-	c	t	c
KF853226	a	t	c	a	g	a	t	g	a	g	t	c	a	t	a	c	g	c	a	t	g	a	a	-	-	c	t	c	a	c	t	c	c	g	-	c	t	c
KF853227	a	t	c	a	g	a	t	g	a	g	t	c	a	t	a	c	g	c	a	t	g	a	a	-	-	c	t	c	a	c	t	c	c	g	-	c	t	c
KF853228	a	t	c	a	g	a	t	g	a	g	t	c	a	t	a	c	g	c	a	t	g	a	a	-	-	c	t	c	a	c	t	c	c	g	-	c	t	c
KF853229	a	t	c	a	g	a	t	g	a	g	t	c	a	t	a	c	g	c	a	t	g	a	a	-	-	c	t	c	a	c	t	c	c	g	-	c	t	c
KF853230	a	t	c	a	g	a	t	g	a	g	t	c	a	t	a	c	g	c	a	t	g	a	a	-	-	c	t	c	a	c	t	c	c	g	-	c	t	c
KF853231	a	t	c	a	g	a	t	g	a	g	t	c	a	t	a	c	g	c	a	t	g	a	a	-	-	c	t	c	a	c	t	c	c	g	-	c	t	c
KF853232	a	t	c	a	g	a	t	g	a	g	t	c	a	t	a	c	g	c	a	t	g	a	a	-	-	c	t	c	a	c	t	c	c	g	-	c	t	c
KF853233	a	t	c	a	g	a	t	g	a	g	t	c	a	t	a	c	g	c	a	t	g	a	a	-	-	c	t	c	a	c	t	c	c	g	-	c	t	c
KF853234	a	t	c	a	g	a	t	g	a	g	t	c	a	t	a	c	g	c	a	t	g	a	a	-	-	c	t	c	a	c	t	c	c	g	-	c	t	c
KF853235	a	t	c	a	g	a	t	g	a	g	t	c	a	t	a	c	g	c	a	t	g	a	a	-	-	c	t	c	a	c	t	c	c	g	-	c	t	c
MF898328	a	t	c	a	g	a	t	g	a	g	t	c	a	t	a	c	g	c	a	t	g	a	a	-	-	c	t	c	a	c	t	c	c	g	-	c	t	c
JN704709	a	t	c	a	g	g	t	g	a	g	t	a	a	t	g	c	g	c	a	t	g	a	a	-	a	c	t	c	a	c	t	c	c	g	c	t	t	c

Supplementary Table 3: 2880 SN

GB-ID	Clade markers									5			9																																		2			2																									
	Position in Dumas	54598	54602	54631	54734	54782	54814	54926	54949	54962	55004	55059	55148	55243	55322	55325	55463	55479	55700	55724	55730	55817	55820	55832	55968	55974	55978	55986	56009	56021	56099	56151	56171	56189	56198	56278	56525	56648	56807	56942	56965	57006	57025	57047	57080	57206	57224	57271	57301	57397	57444	57508	57524	57643	57706	57886	57887	57916	57955	58054	58072	58117	58156	58162	58183	58325	58364	58441	58459	58495	58510	58549	58595	58742	58771
AY548170	1	t	t	g	a	c	t	a	t	t	t	t	g	c	c	c	a	g	a	a	t	a	a	a	a	g	g	g	g	t	a	c	a	a	t	a	g	g	g	a	c	c	t	t	t	a	a	t	a	g	a	t	c	g	c	g	g	g	c	c	a	a	c	t	g	a	a	g	c	t	c	c	a	t	a
AY548171	1	t	t	g	a	c	t	a	t	t	t	t	g	c	c	c	a	g	a	a	t	a	a	a	a	g	g	g	g	t	a	c	a	a	t	a	g	g	g	a	c	c	t	t	t	a	a	t	a	g	a	t	c	g	c	g	g	g	c	c	a	a	c	t	g	a	a	g	c	t	c	c	a	t	a
DQ479953	1	t	t	g	a	c	t	a	t	t	t	t	g	c	c	c	a	g	a	a	t	a	a	a	a	g	g	g	g	t	a	c	a	a	t	a	g	g	g	a	c	c	t	t	t	a	a	t	a	g	a	t	c	g	c	g	g	g	c	c	a	a	c	t	g	a	a	g	c	t	c	c	a	t	a
DQ479954	1	t	t	g	a	c	t	a	t	t	t	t	g	c	c	c	a	g	a	a	t	a	a	a	a	g	g	g	g	t	a	c	a	a	t	a	g	g	g	a	c	c	t	t	t	a	a	t	a	g	a	t	c	g	c	g	g	g	c	c	a	a	c	t	g	a	a	g	c	t	c	c	a	t	a
DQ479958	1	t	t	g	a	c	t	a	t	t	t	t	g	c	c	c	a	g	a	a	t	a	a	a	a	g	g	g	g	t	a	c	a	a	t	a	g	g	g	a	c	c	t	t	t	a	a	t	a	g	a	t	c	g	c	g	g	g	c	c	a	a	c	t	g	a	a	g	c	t	c	c	a	t	a
DQ479959	1	t	t	g	a	c	t	a	t	t	t	t	g	c	c	c	a	g	a	a	t	a	a	a	a	g	g	g	g	t	a	c	a	a	t	a	g	g	g	a	c	c	t	t	t	a	a	t	a	g	a	t	c	g	c	g	g	g	c	c	a	a	c	t	g	a	a	g	c	t	c	c	a	t	a
DQ479961	1	t	t	g	a	c	t	a	t	t	t	t	g	c	c	c	a	g	a	a	t	a	a	a	a	g	g	g	g	t	a	c	a	a	t	a	g	g	g	a	c	c	t	t	t	a	a	t	a	g	a	t	c	g	c	g	g	g	c	c	a	a	c	t	g	a	a	g	c	t	c	c	a	t	a
DQ479962	1	t	t	g	a	c	t	a	t	t	t	t	g	c	c	c	a	g	a	a	t	a	a	a	a	g	g	g	g	t	a	c	a	a	t	a	g	g	g	a	c	c	t	t	t	a	a	t	a	g	a	t	c	g	c	g	g	g	c	c	a	a	c	t	g	a	a	g	c	t	c	c	a	t	a
DQ479963	1	t	t	g	a	c	t	a	t	t	t	t	g	c	c	c	a	g	a	a	t	a	a	a	a	g	g	g	g	t	a	c	a	a	t	a	g	g	g	a	c	c	t	t	t	a	a	t	a	g	a	t	c	g	c	g	g	g	c	c	a	a	c	t	g	a	a	g	c	t	c	c	a	t	a
DQ674250	1	t	t	g	a	c	t	a	t	t	t	t	g	c	c	c	a	g	a	a	t	a	a	a	a	g	g	g	g	t	a	c	a	a	t	a	g	g	g	a	c	c	t	t	t	a	a	t	a	g	a	t	c	g	c	g	g	g	c	c	a	a	c	t	g	a	a	g	c	t	c	c	a	t	a
KX262864	1	t	t	g	a	c	t	a	t	t	t	t	g	c	c	c	a	g	a	a	t	a	a	a	a	g	g	g	g	t	a	c	a	a	t	a	g	g	g	a	c	c	t	t	t	a	a	t	a	g	a	t	c	g	c	g	g	g	c	c	a	a	c	t	g	a	a	g	c	t	c	c	a	t	a
KX262865	1	t	t	g	a	c	t	a	t	t	t	t	g	c	c	c	a	g	a	a	t	a	a	a	a	g	g	g	g	t	a	c	a	a	t	a	g	g	g	a	c	c	t	t	t	a	a	t	a	g	a	t	c	g	c	g	g	g	c	c	a	a	c	t	g	a	a	g	c	t	c	c	a	t	a
KX262866	1	t	t	g	a	c	t	a	t	t	t	t	g	c	c	c	a	g	a	a	t	a	a	a	a	g	g	g	g	t	a	c	a	a	t	a	g	g	g	a	c	c	t	t	t	a	a	t	a	g	a	t	c	g	c	g	g	g	c	c	a	a	c	t	g	a	a	g	c	t	c	c	a	t	a
EU154348	1	t	t	g	a	c	t	a	t	t	t	t	g	c	c	c	a	g	a	a	t	a	a	a	a	g	g	g	g	t	a	c	a	a	t	a	g	g	g	a	c	c	t	t	t	a	a	t	a	g	a	t	c	g	c	g	g	g	c	c	a	a	c	t	g	a	a	g	c	t	c	c	a	t	a
JN704690	1	t	t	g	a	c	t	a	t	t	t	t	g	c	c	c	a	g	a	a	t	a	a	a	a	g	g	g	g	t	a	c	a	a	t	a	g	g	g	a	c	c	t	t	t	a	a	t	a	g	a	t	c	g	c	g	g	g	c	c	a	a	c	t	g	a	a	g	c	t	c	c	a	t	a
JN704691	1	t	t	g	a	c	t	a	t	t	t	t	g	c	c	c	a	g	a	a	t	a	a	a	a	g	g	g	g	c	a	c	a	a	t	a	g	g	g	a	c	c	t	t	t	a	a	t	a	g	a	t	c	g	c	g	g	g	c	c	a	a	c	t	g	a	a	g	c	t	c	c	a	t	a
JN704692	1	t	t	g	a	c	t	a	t	t	t	t	g	c	c	c	a	g	a	a	t	a	a	a	a	g	g	g	g	t	a	c	a	a	t	a	g	g	g	a	c	c	t	t	t	a	a	t	a	g	a	t	c	g	c	g	g	g	c	c	a	a	c	t	g	a	a	g	c	t	c	c	a	t	a
JN704693	1	t	t	g	a	c	t	a	t	t	t	t	g	c	c	c	a	g	a	a	t	a	a	a	a	g	g	g	g	t	a	c	a	a	t	a	g	g	g	a	c	c	t	t	t	a	a	t	a	g	a	t	c	g	c	g	g	g	c	c	a	a	c	t	g	a	a	g	c	t	c	c	a	t	a
JN704694	1	t	t	g	a	c	t	a	t	t	t	t	g	c	c	c	a	g	a	a	t	a	a	a	a	g	g	g	g	t	a	c	a	a	t	a	g	g	g	a	c	c	t	t	t	a	a	t	a	g	a	t	c	g	c	g	g	g	c	c	a	a	c	t	g	a	a	g	c	t	c	c	a	t	a
JN704695	1	t	t	g	a	c	t	a	t	t	t	t	g	c	c	c	a	g	a	a	t	a	a	a	a	g	g	g	g	t	a	c	a	a	t	a	g	g	g	a	c	c	t	t	t	a	a	t	a	g	a	t	c	g	c	g	g	g	c	c	a	a	c	t	g	a	a	g	c	t	c	c	a	t	a
JN704696	1	t	t	g	a	c	t	a	t	t	t	t	g	c	c	c	a	g	a	a	t	a	a	a	a	g	g	g	g	t	a	c	a	a	t	a	g	g	g	a	c	c	t	t	t	a	a	t	a	g	a	t	c	g	c	g	g	g	c	c	a	a	c	t	g	a	a	g	c	t	c	c	a	t	a
NC001348	1	t	t	g	a	c	t	a	t	t	t	t	g	c	c	c	a	g	a	a	t	a	a	a	a	g	g	g	g	t	a	c	a	a	t	a	g	g	g	a	c	c	t	t	t	a	a	t	a	g	a	t	c	g	c	g	g	g	c	c	a	a	c	t	g	a	a	g	c	t	c	c	a	t	a
KP771892	1	t	t	g	a	c	t	a	t	t	t	t	g	c	c	c	a	g	a	a	t	a	a	a	a	g	g	g	g	t	a	c	a	a	t	a	g	g	g	a	c	c	t	t	t	a	a	t	a	g	a	t	c	g	c	g	g	g	c	c	a	a	c	t	g	a	a	g	c	t	c	c	a	t	a
KP771894	1	t	t	g	a	c	t	a	t	t	t	t	g	c	c	c	a	g	a	a	t	a	a	a	a	g	g	g	g	t	a	c	c	a	t	a	g	g	g	a	c	c	t	t	t	a	a	t	a	g	a	t	c	g	c	g	g	g	c	c	a	a	c	t	g	a	a	g	c	t	c	c	a	t	a
KP771900	1	t	t	g	a	c	t	a	t	t	t	t	g	c	c	c	a	g	a	a	t	a	a	a	a	g	g	g	g	t	a	c	a	a	t	a	g	g	g	a	c	c	t	t	t	a	a	t	a	g	a	t	c	g	c	g	g	g	c	c	a	a	c	t	g	a	a	g	c	t	c	c	a	t	a
KP771901	1	t	t	g	a	c	t	a	t	t	t	t	g	c	c	c	a	g	a	a	t	a	a	a	a	g	g	g	g	t	a	c	a	a	t	a	g	g	g	a	c	c	t	t	t	a	a	t	a	g	a	t	c	g	c	g	g	g	c	c	a	a	c	t	g	a	a	g	c	t	c	c	a	t	a
KP771902	1	t	t	g	a	c	t	a	t	t	t	t	g	c	c	c	a	g	a	a	t	a	a	a	a	g	g	g	g	t	a	c	a	a	t	a	g	g	g	a	c	c	t	t	t	a	a	t	a	g	a	t	c	g	c	a	g	a	c	c	a	a	c	t	g	a	a	g	c	t	c	c	a	t	a
KP771908	1	t	t	g	a	c	t	a	t	t	t	t	g	c	c	c	a	g	a	a	t	a	a	a	a	g	g	g	g	t	a	c	a	a	t	a	g	g	g	a	c	c	t	t	t	a	a	t	a	g	a	t	c	g	c	g	g	g	c	c	a	a	c	t	g	a	a	g	c	t	c	c	a	t	a
KP771912	1	t	t	g	a	c	t	a	t	t	t	t	g	c	c	c	a	g	a	a	t	a	a	a	a	g	g	g	g	t	a	c	a	a	t	a	g	g	g	a	c	c	t	t	t	a	a	t	a	g	a	t	c	g	c	g	g	g	c	c	a	a	c	t	g	a	a	g	c	t	c	c	a	t	a
KP771913	1	t	t	g	a	c	t	a	t	t	t	t	g	c	c	c	a	g	a	a	t	a	a	a	a	g	g	g	g	t	a	c	a	a	t	a	g	g	g	a	c	c	t	t	t	a	a	t	a	g	a	t	c	g	c	g	g	g	c	c	a	a	c	t	g	a	a	g	c	t	c	c	a	t	a
KP771917	1	t	t	g	a	c	t	a	t	t	t	t	g	c	c	c	a	g	a	a	t	a	a	a	a	g	g	g	g	t	a	c	a	a	t	a	g	g	g	a	c	c	t	t	t	a	a	t	a	g	a	t	c	g	c	g	g	g	c	c	a	a	c	t	g	a	a	g	c	t	c	c	a	t	a
KP771918	1	t	t	g	a	c	t	a	t	t	t	t	g	c	c	c	a	g	a	a	t	a	a	a	a	g	g	g	g	t	a	c	a	a	t	a	g	g	g	a	c	c	t	t	t	a	a	t	a	g	a	t	c	g	c	g	g	g	c	c	a	a	c	t	g	a	a	g	c	t	c	c	a	t	a
KP771920	1	t	t	g	a	c	t	a	t	t	t	t	g	c	c	c	a	g	a	a	t	a	a	a	a	g	g	g	g	t	a	c	a	a	t	a	g	g	g	a	c	c	t	t	t	a	a	t	a	g	a	t	c	g	c	g	g	g	c	c	a	a	c	t	g	a	a	g	c	t	c	c	a	t	a
MH709361	1	t	t	g	a	c	t	a	t	t	t	t	g	c	c	c	a	g	a	a	t	a	a	a	a	g	g	g	g	t	a	c	a	a	t	a	g	g	g	a	c	c	t	t	t	a	a	t	a	g	a	t	c	g	c	g	g	g	c	c	a	a	c	t	g	a	a	g	c	t	c	c	a	t	a
KU926318	2	c	t	g	a	c	t	a	t	t	t	t	g	c	t	t	a	g	a	a	t	a	a	a	a	g	g	g	g	t	a	c	a	a	t	a	g	g	g	a	c	c	c	t	t	a	c	t	c	t	a	c	c	g	c	g	g	g	c	c	a	a	c	t	g	a	a	g	c	t	c	t	a	t	a
KU926319	2	t	t	g	a	c	t	a	t	t	t	t	g	c	c	c	a	g	a	a	t	a	a	a	a	g	g	g	g	t	a	c	a	a	t	a	g	g	g	a	c	c	t	t	t	a	c	t	c	t	a	t	c	g	c	g	g	g	c	c	a	a	c	t	g	a	a	g	c	t	c	c	a	t	a
KU926320	2	t	t	g	a	c	t	a	t	t	t	t	g	c	c	c	a	g	a	a	t	a	a	a	a	g	g	g	g	t	a	c	a	g	t	a	g	g	g	a	c	c	t	t	t	a	c	t	c	t	a	t	c	g	c	g	g	g	c	c	a	a	c	t	g	g	c	g	c	t	c	c	a	t	a
KU926321	2	t	t	g	a	c	t	a	t	t	t	t	g	c	c	c	a	g	a	a	t	a	a	a	a	g	g	g	g	t	a	c	a	a	t	a	g	g	g	a	c	c	t	t	t	a	c	t	c	t	a	t	c	g	c	g	g	g	c	c	a	a	c	t	g	a	a	g	c	t	c	c	a	t	a
KU926322	2	t	t	g	a	c	t	a	t	t	t	t	g	c	c	c	a	g	a	a	t	a	a	a	a	g	g	g	g	t	a	c	a	a	t	a	g	g	g	g	c	c	t	t	t	a	c	t	c	t	a	t	c	g	c	g	g	g	c	c	a	a	c	t	g	a	a	g	c	t	c	c	a	t	a
KC847290	2	t	t	g	a	c	t	a	t	t	t	t	g	c	c	c	a	g	a	a	t	a	a	a	a	g	g	g	g	t	a	c	a	a	t	a	g	g	g	a	c	c	t	t	t	a	c	t	c	t	a	t	c	g	c	g	g	g	c	c	a	a	c	t	g	a	a	g	c	t	c	c	a	t	a
KJ767491	2	t	t	g	a	c	t	a	t	t	t	t	g	c	t	t	a	g	a	a	t	a	a	a	a	g	g	g	g	t	a	c	a	a	t	a	g	g	g	a	c	c	t	t	t	a	c	t	c	t	a	t	c	g	c	g	g	g	c	c	a	a	c	t	g	a	a	g	c	t	c	t	a	t	a
KJ767492	2	t	t	g	a	c	t	a	t	t	t	t	g	c	c	c	a	g	a	a	t	a	a	a	a	g	g	g	g	t	a	c	a	a	t	a	g	g	g	a	c	c	t	t	t	a	c	t	c	t	a	t	c	g	c	g	g	g	c	c	a	a	c	t	g	a	a	g	c	t	c	c	a	t	a
KJ808816	2	t	t	g	a	c	t	a	t	t	t	t	g	c	c	c	a	g	a	a	t	a	a	a	a	g	g	g	g	t	a	c	a	a	t	a	g	g	g	a	c	c	t	t	t	a	c	t	c	t	a	t	c	g	c	g	g	g	c	c	a	a	c	t	g	a	a	g	c	t	c	c	a	t	a
KP771925	2	t	t	g	a	c	t	a	t	t	t	t	g	c	c	c	a	g	a	a	t	a	a	a	a	g	g	g	g	t	a	c	a	a	t	a	g	g	g	a	c	c	t	t	t	a	c	t	c	t	a	t	c	g	c	g	g	g	c	c	a	a	c	t	g	a	a	g	c	t	c	c	a	t	a
KU926315	2	t	t	g	a	c	t	a	t	t	t	t	g	c	t	t	a	g	a	a	t	a	a	a	a	g	g	g	g	t	a	c	a	a	t	a	g	g	g	a	c	c	t	t	t	a	c	t	c	t	a	t	c	g	c	g	g	g	c	c	a	a	c	t	g	a	a	g	c	t	c	t	a	t	a
KU926316	2	t	t	g	a	c	t	a	t	t	t	t	g	c	t	c	a	g	a	a	t	a	a	a	a	g	g	g	g	t	a	c	a	a	t	a	g	g	g	a	c	c	t	t	t	a	c	t	c	t	a	t	c	g	c	g	g	g	c	c	a	a	c	t	g	a	a	g	c	t	c	t	a	t	a
KU926317	2	t	t	g	a	c	t	a	t	t	t	t	g	c	t	t	a	g	a	a	t	a	a	a	a	g	g	g	g	t	a	c	a	a	t	a	g	g	g	a	c	c	t	t	t	a	c	t	c	t	a	t	c	g	c	g	g	g	c	c	a	a	c	t	g	a	a	g	c	t	c	t	a	t	a
KY037796	2	t	t	g	a	c	t	a	t	t	t	t	g	c	c	c	a	g	a	a	t	a	a	a	a	g	g	g	g	t	a	c	a	a	t	a	g	g	g	a	c	c	t	t	t	a	c	t	c	t	a	t	c	g	c	g	g	g	c	c	a	a	c	t	g	a	a	g	c	t	c	c	a	t	a
MF004348	2	t	t	g	a	c	t	a	t	t	t	t	g	c	c	c	a	g	a	a	t	a	a	a	a	g	g	g	g	t	a	c	a	a	t	a	g	g	g	a	c	c	t	t	t	a	c	t	c	t	a	t	c	g	c	g	g	g	c	c	a	a	c	t	g	a	a	g	c	t	c	c	a	t	a
MH709309	2	t	t	g	a	c	t	a	t	t	t	t	g	c	c	c	a	g	a	a	t	a	a	a	a	g	g	g	g	t	a	c	a	a	t	a	g	g	g	a	c	c	t	t	t	a	c	t	c	t	a	t	c	g	c	g	g	g	c	c	a	a	c	t	g	a	a	g	c	t	c	c	a	t	a
MH709310	2	t	t	g	a	c	t	a	t	t	t	t	g	c	c	c	a	g	a	a	t	a	a	a	a	g	g	g	g	t	a	c	a	a	t	a	g	g	g	a	c	c	t	t	t	a	c	t	c	t	a	t	c	g	c	g	g	g	c	c	a	a	c	t	g	a	a	g	c	t	c	c	a	t	a
MH709311	2	t	t	g	a	c	t	a	t	t	t	t	g	t	c	c	a	g	a	a	t	a	a	a	a	g	g	g	g	t	a	c	a	a	t	a	g	g	g	a	c	c	t	t	t	a	c	t	c	t	a	t	c	g	c	g	g	g	c	c	a	a	c	t	g	a	a	g	c	t	c	c	a	t	a
MH709313	2	t	t	g	a	c	t	a	t	t	t	t	g	c	c	c	a	g	a	a	t	a	a	a	a	g	g	g	g	t	a	c	a	a	t	a	g	g	g	a	c	c	t	t	t	a	c	t	c	t	a	t	c	g	c	g	g	g	c	c	a	a	c	t	g	a	a	g	c	t	c	c	a	t	a
MH709314	2	t	t	g	a	c	t	a	t	t	t	t	g	c	c	c	a	g	a	a	t	a	g	a	a	g	g	g	g	t	a	c	a	a	t	a	g	g	g	a	c	c	t	t	t	a	c	t	c	t	a	t	c	g	c	g	g	g	c	c	a	a	c	t	g	a	a	g	c	t	c	c	a	t	a
MH709315	2	t	t	g	a	c	t	a	t	t	t	t	g	c	c	c	a	g	a	a	t	a	a	a	a	g	g	g	g	t	a	c	a	a	t	a	g	g	g	a	c	c	t	t	t	a	c	t	c	t	a	t	c	g	c	g	g	g	c	c	a	a	c	t	g	a	a	g	c	t	c	c	a	t	a
MH709319	2	t	t	g	a	c	t	a	t	t	t	t	g	c	c	c	a	g	a	a	t	a	g	a	a	g	g	g	g	t	a	c	a	a	t	g	g	g	g	a	c	c	t	t	t	a	c	t	c	t	a	t	c	g	c	g	g	g	c	c	a	a	c	t	a	a	a	g	c	t	c	c	a	t	a
MH709320	2	t	t	g	a	c	t	a	t	t	t	t	g	c	c	c	a	g	a	a	t	a	a	a	a	g	g	g	g	t	a	c	a	a	t	a	g	g	g	a	c	c	t	t	t	a	c	t	c	t	a	t	c	g	c	g	g	g	c	c	a	a	c	t	g	a	a	g	c	t	c	c	a	t	a
MH709329	2	t	t	g	a	c	t	a	t	t	t	t	g	c	c	c	a	g	a	a	t	a	g	a	a	g	g	g	g	t	a	c	a	a	t	a	g	g	g	a	c	c	t	t	t	a	c	t	c	t	a	t	c	g	c	g	g	g	c	c	a	a	c	t	g	a	a	g	c	t	c	c	a	t	a
MH709331	2	t	t	g	a	c	t	a	t	t	t	t	g	c	c	c	a	g	a	a	t	c	a	a	a	g	g	g	g	t	a	c	a	a	t	a	g	g	g	a	c	c	t	t	t	a	c	t	c	t	a	t	c	g	c	g	g	g	c	c	a	a	c	t	g	a	a	g	c	t	c	c	a	t	a
MH709333	2	t	t	g	a	c	t	a	t	t	t	t	g	c	c	c	a	g	a	a	t	a	g	a	a	g	g	g	g	t	a	c	a	a	t	a	g	g	g	a	c	c	t	t	t	a	c	t	c	t	a	t	c	g	c	g	g	g	c	c	a	a	c	t	g	a	a	g	c	t	c	c	a	t	a
MH709337	2	t	t	g	a	c	t	a	t	t	t	t	g	c	c	c	a	g	a	a	t	a	a	a	a	g	g	g	g	t	a	c	a	a	t	a	g	g	g	a	c	c	t	t	t	a	c	t	c	t	a	t	c	g	c	g	g	g	c	c	a	a	c	t	g	a	a	g	c	t	c	c	a	t	a
MH709339	2	t	t	a	a	c	t	a	t	t	t	t	g	c	c	c	a	g	a	a	t	a	a	a	a	g	g	g	g	t	g	c	a	a	t	a	g	g	g	a	c	c	t	t	t	a	c	t	c	t	a	t	c	g	c	g	g	g	c	t	a	a	c	t	g	a	a	g	c	t	c	c	a	t	a
MH709344	2	t	t	g	a	c	t	a	t	t	t	t	g	c	c	c	a	t	a	a	t	a	a	a	a	g	g	g	g	t	a	c	a	a	t	a	g	g	g	a	c	c	t	t	t	a	c	t	c	t	a	t	c	g	c	g	g	g	c	c	a	a	c	t	g	a	a	g	c	t	c	c	a	t	a
MH709348	2	t	t	g	a	c	t	a	t	t	t	t	g	c	c	c	a	g	a	a	t	a	a	a	a	g	g	g	g	t	a	c	a	a	t	a	g	g	g	a	c	c	t	t	t	a	c	t	c	t	a	t	c	g	c	g	g	g	c	c	a	a	c	t	g	a	a	g	c	t	c	c	a	t	a
MH709355	2	t	t	g	a	c	t	a	t	t	t	t	g	c	c	c	a	g	a	a	t	a	a	a	a	g	g	g	g	t	a	c	a	a	t	a	g	g	g	a	c	c	t	t	t	a	c	t	c	t	a	t	c	g	c	g	g	g	c	c	a	a	c	t	g	a	a	g	c	t	c	c	a	t	a
MH709359	2	t	t	g	a	c	t	a	t	t	t	t	g	c	c	c	a	g	a	a	t	a	a	c	a	g	g	g	g	t	a	c	a	a	t	a	g	g	g	a	c	c	t	t	t	a	c	t	c	t	a	t	c	g	c	g	g	g	c	c	a	a	c	t	g	a	a	g	c	t	c	c	a	t	a
MH709363	2	t	c	g	a	c	t	a	t	t	t	t	g	c	c	c	a	g	a	a	t	a	a	a	a	g	g	g	g	t	a	c	a	a	t	a	g	g	g	a	c	c	t	t	t	a	c	t	c	t	a	t	c	g	c	g	g	g	c	c	a	a	c	t	g	a	a	g	c	t	c	c	a	t	a
MH709366	2	t	t	g	a	c	t	a	t	t	t	t	g	c	c	c	a	g	a	a	t	a	a	c	a	g	g	g	g	t	a	c	a	a	t	a	g	g	g	a	c	c	t	t	t	a	c	t	c	t	a	t	c	g	c	g	g	g	c	c	a	a	c	t	g	a	a	a	c	t	c	c	a	t	a
MH709376	2	t	t	g	a	c	t	a	t	t	t	t	g	c	c	c	a	g	a	a	t	a	a	a	a	g	g	g	g	t	a	c	a	a	t	a	g	g	g	a	c	c	t	t	t	a	c	t	c	t	a	t	c	g	c	g	g	g	c	c	a	a	c	t	g	a	a	g	c	t	c	c	a	t	a
MH709377	2	t	t	g	a	c	t	a	t	t	t	t	g	c	c	c	a	g	a	a	t	a	a	a	a	g	g	g	g	t	a	c	a	a	t	a	g	g	g	a	c	c	t	t	t	a	c	t	c	t	a	t	c	g	c	g	g	g	c	c	a	a	c	t	g	a	a	g	c	t	c	c	a	t	a
**MW316406**	2	t	t	g	a	c	t	a	t	t	t	t	g	c	c	c	a	g	a	a	t	a	a	a	a	g	g	g	g	t	a	c	a	a	t	a	g	g	g	a	c	c	t	t	t	a	c	t	c	t	a	t	c	g	c	g	g	g	c	c	a	a	c	t	g	a	a	g	c	t	c	c	a	t	a
AJ871403	3	t	t	g	a	c	t	a	t	t	t	t	g	c	c	c	a	g	a	a	t	a	a	a	a	g	g	g	g	t	a	c	a	a	t	a	g	g	g	a	c	c	t	t	t	a	a	t	a	g	a	t	c	g	c	g	g	g	c	c	a	a	c	t	g	a	a	g	c	t	c	c	a	t	a
DQ479955	3	t	t	g	a	c	t	a	t	t	t	t	g	c	c	c	a	g	a	a	t	a	a	a	a	g	g	g	g	t	a	c	a	a	t	a	g	g	g	a	c	c	t	t	t	a	a	t	a	g	a	t	c	g	c	g	g	g	c	c	a	a	c	t	g	a	a	g	c	t	a	c	a	t	a
DQ479956	3	t	t	g	a	c	t	a	t	t	t	t	g	c	c	c	t	g	a	a	t	a	a	a	a	g	g	g	g	t	a	c	a	a	t	a	g	g	g	a	c	c	t	t	t	a	a	t	a	g	a	t	c	g	c	g	g	g	c	c	a	a	c	t	g	a	a	g	c	t	c	c	a	t	a
DQ479957	3	t	t	g	a	c	t	a	t	t	t	t	g	c	c	c	a	g	a	a	t	a	a	a	a	g	g	g	g	t	a	c	a	a	t	a	g	g	g	a	c	c	t	t	t	a	a	t	a	g	a	t	c	g	c	g	g	g	c	c	a	a	c	t	g	a	a	g	c	t	c	c	a	t	a
JN704699	3	t	t	g	a	c	t	g	t	t	t	t	g	c	c	c	a	g	a	a	t	a	a	a	a	g	g	g	g	t	a	c	a	a	t	a	g	g	g	a	c	c	t	t	t	a	a	t	a	g	a	t	c	g	c	g	g	g	c	c	a	a	c	t	g	a	a	g	c	t	c	c	a	t	a
JN704700	3	t	t	g	a	c	t	a	t	t	t	t	g	c	c	c	a	g	a	a	t	a	a	a	a	g	g	g	g	t	a	c	a	a	t	a	g	g	g	a	c	c	t	t	t	a	a	t	a	g	a	t	c	g	c	g	g	g	c	c	a	c	c	t	g	a	a	g	c	t	c	c	a	t	a
JN704701	3	t	t	g	a	c	t	g	t	t	t	t	g	c	c	c	a	g	a	a	t	a	a	a	a	g	g	g	g	t	a	c	a	a	t	a	g	g	g	a	c	c	t	t	t	a	a	t	a	g	a	t	c	g	c	g	g	g	c	c	a	a	c	t	g	a	a	g	c	t	c	c	a	t	a
JN704702	3	t	t	g	a	c	t	a	t	t	t	t	g	c	c	c	a	g	a	a	t	a	a	a	a	g	g	g	g	t	a	c	a	a	t	a	g	g	g	a	c	c	t	t	t	a	a	t	a	g	a	t	c	g	c	g	g	g	c	c	a	a	c	t	g	a	a	g	c	t	c	c	a	t	a
JN704703	3	t	t	g	a	c	t	a	t	t	t	t	g	c	c	c	a	g	a	a	t	a	a	a	a	g	g	g	g	t	a	c	a	a	t	a	g	g	g	a	a	c	t	t	t	a	a	t	a	g	a	t	c	g	c	g	g	g	c	c	a	a	c	t	g	a	a	g	c	t	c	c	a	t	a
KP771891	3	t	t	g	a	c	t	g	t	t	t	t	g	c	c	c	a	g	a	a	t	a	a	a	a	g	g	g	g	t	a	c	a	a	t	a	g	g	t	a	c	c	t	t	t	a	a	t	a	g	a	t	c	g	c	g	g	g	c	c	a	a	c	t	g	a	a	g	c	t	c	c	a	t	a
KP771893	3	t	t	g	a	c	t	g	t	t	t	t	g	c	c	c	a	g	a	a	t	a	a	a	a	g	g	g	g	t	a	c	a	a	t	a	g	g	g	a	c	c	t	t	t	a	a	t	a	g	a	t	c	g	c	g	g	g	c	c	a	a	c	t	g	a	a	g	c	t	c	c	a	t	a
KP771895	3	t	t	g	a	c	t	a	t	t	t	t	g	c	c	c	a	g	a	a	t	a	a	a	a	g	g	g	g	t	a	c	a	a	t	a	g	g	g	a	c	c	t	t	t	a	a	t	a	g	a	t	c	g	c	g	g	g	c	c	a	a	c	t	g	a	a	g	c	t	c	c	a	t	a
KP771896	3	t	t	g	a	c	t	a	t	t	t	t	g	c	c	c	a	g	a	a	t	a	a	a	a	g	g	g	g	t	a	c	a	a	t	a	g	g	g	a	c	c	t	t	t	a	a	t	a	g	a	t	c	g	c	g	g	g	c	c	a	a	c	t	g	a	a	g	c	t	c	c	a	t	a
KP771897	3	t	t	g	a	c	t	a	t	t	t	t	g	c	c	c	a	g	a	a	t	a	a	a	a	g	g	g	g	t	a	c	a	a	t	a	g	g	g	a	c	c	t	t	t	a	a	g	a	g	a	t	c	g	c	g	g	g	c	c	a	a	c	t	g	a	a	g	c	t	c	c	a	t	a
KP771899	3	t	t	g	a	c	t	a	t	t	t	t	g	c	c	c	a	g	a	a	t	a	a	a	a	g	g	g	g	t	a	c	a	a	t	a	g	g	g	a	c	c	t	t	t	a	a	t	a	g	a	t	c	g	c	g	g	g	c	c	a	a	c	t	g	a	a	g	c	t	c	c	a	t	a
KP771903	3	t	t	g	a	c	t	g	t	t	t	t	g	c	c	c	a	g	a	a	t	a	a	a	a	g	g	g	g	t	a	c	a	a	t	a	a	g	t	a	c	c	t	t	t	a	a	t	a	g	a	t	c	g	c	g	g	g	c	c	a	a	c	t	g	a	a	g	c	t	c	c	a	t	a
KP771907	3	t	t	g	a	c	t	a	t	t	t	t	g	c	c	c	a	g	a	a	t	a	a	a	a	g	g	g	g	t	a	c	a	a	t	a	g	g	g	a	c	t	t	t	t	a	a	t	a	g	a	t	c	g	c	g	g	g	c	c	a	a	c	t	g	a	a	g	c	t	c	c	a	t	a
KP771910	3	t	t	g	a	c	t	g	t	t	t	t	g	c	c	c	a	g	a	a	t	a	a	a	a	g	g	g	g	t	a	c	a	a	t	a	g	g	g	a	c	c	t	t	t	a	a	t	a	g	a	t	c	g	c	g	g	g	c	c	a	a	c	t	g	a	a	g	c	t	c	c	a	t	a
KP771915	3	t	t	g	a	c	t	a	t	t	t	t	g	c	c	c	a	g	a	a	t	a	a	a	a	g	g	g	g	t	a	c	a	a	t	a	g	g	g	a	c	c	t	t	t	a	a	t	a	g	a	t	c	g	c	g	g	g	c	c	a	a	c	t	g	a	a	g	c	t	c	c	a	t	a
KP771916	3	t	t	g	a	c	t	a	t	t	t	t	g	c	c	c	t	g	a	a	t	a	a	a	a	g	g	g	g	t	a	c	a	a	t	a	g	g	g	a	c	c	t	t	t	a	a	t	a	g	a	t	c	g	c	g	g	g	c	c	a	a	c	t	g	a	a	g	c	t	c	c	a	t	a
KP771921	3	t	t	g	a	c	t	g	t	t	t	t	g	c	c	c	a	g	a	a	t	a	a	a	a	g	g	g	g	t	a	c	a	a	t	a	g	g	g	a	c	c	t	t	t	a	a	t	a	g	a	t	c	g	c	g	g	g	c	c	a	a	c	t	g	a	a	g	c	t	c	c	a	t	a
KP771923	3	t	t	g	a	c	t	g	t	t	t	t	g	c	c	c	a	g	a	a	t	a	a	a	a	g	g	g	g	t	a	c	a	a	t	a	g	g	g	a	c	c	t	t	t	a	a	t	a	g	a	t	c	g	c	g	g	g	c	c	a	a	c	t	g	a	a	g	c	t	c	c	a	t	a
KP771889	3	t	t	g	a	c	t	a	t	t	t	t	g	c	c	c	a	g	a	a	t	a	a	a	a	g	g	g	g	t	a	c	a	a	t	a	g	g	g	a	c	c	t	t	t	a	a	t	a	g	a	t	c	g	c	g	g	g	c	c	a	a	c	t	g	a	a	g	c	t	c	c	a	t	a
KU926311	3	t	t	g	a	c	t	a	t	t	t	t	g	c	c	c	t	g	a	a	t	a	a	a	a	g	g	g	g	t	a	c	a	a	t	a	g	g	g	a	c	c	t	t	t	a	a	t	a	g	a	t	c	g	c	g	g	g	c	c	a	a	c	t	g	a	a	g	c	t	c	c	a	t	a
JQ911599	3	t	t	g	a	c	t	a	t	t	t	t	g	c	c	c	a	g	a	a	t	a	a	a	a	g	g	g	g	t	a	c	a	a	t	a	g	g	g	a	c	c	t	t	t	a	a	t	a	g	a	t	c	g	c	g	g	g	c	c	a	a	c	t	g	a	a	g	c	t	c	c	a	t	a
MH709317	3	t	t	g	a	c	t	a	t	t	t	t	g	c	c	c	a	g	a	a	t	a	a	a	a	g	g	g	g	t	a	c	a	a	t	a	g	g	g	a	c	c	t	t	t	a	a	t	a	g	a	t	c	g	c	g	g	g	c	c	a	a	c	t	g	a	a	g	c	t	c	c	a	t	a
MH709346	3	t	t	g	a	c	t	a	t	t	t	t	g	c	c	c	a	g	a	a	t	a	a	a	a	g	g	g	g	t	a	c	a	a	t	a	g	g	g	a	c	c	t	t	t	a	a	t	a	g	a	t	c	g	c	g	g	g	c	c	a	a	c	t	g	a	a	g	c	t	c	c	a	t	a
MH709375	3	t	t	g	a	c	t	a	t	t	t	t	g	c	c	c	a	g	a	a	t	a	a	a	a	g	g	g	g	t	a	c	a	a	t	a	g	g	g	a	c	c	t	t	t	a	a	t	a	g	a	t	c	g	c	g	g	g	c	c	a	a	c	t	g	a	a	g	c	t	c	c	a	t	a
DQ452050	4	t	t	g	a	c	t	a	t	t	t	t	g	c	c	c	a	g	a	a	t	a	a	a	a	g	g	g	g	t	a	c	a	a	t	a	g	g	g	a	c	c	t	t	t	a	a	t	c	g	a	t	c	g	c	g	g	g	t	c	a	a	c	t	g	a	a	g	c	t	c	c	a	t	a
DQ479960	4	t	t	g	a	c	t	a	t	t	t	t	g	c	c	c	a	g	a	a	t	a	a	a	a	g	g	g	g	t	a	c	a	a	t	a	g	g	g	a	c	c	t	t	t	a	a	t	c	g	a	t	c	g	c	g	g	g	t	c	a	a	a	t	g	a	a	g	c	t	c	c	a	t	a
KP771898	4	c	t	g	a	c	t	a	t	t	t	t	g	c	c	c	a	g	a	a	t	a	a	a	a	g	g	g	g	t	a	c	a	a	t	a	g	g	g	a	c	c	t	t	t	a	a	t	c	g	a	t	c	g	c	g	g	g	t	c	a	a	c	t	g	a	a	g	c	t	c	c	a	t	a
MH709316	4	t	t	g	a	c	c	a	t	t	t	t	g	c	c	c	a	g	a	a	t	a	a	a	a	g	g	g	g	t	a	c	a	a	t	a	g	g	g	a	c	c	t	t	t	a	a	t	c	g	a	t	c	g	c	g	g	g	t	c	a	a	c	t	g	a	a	g	c	t	c	c	a	t	a
MH709318	4	t	t	g	a	c	t	a	t	t	t	t	g	c	c	c	a	g	a	a	t	a	a	a	a	g	g	g	g	t	a	c	a	a	t	a	g	g	g	a	c	c	t	t	t	a	a	t	c	g	a	t	c	g	c	g	g	g	t	c	a	a	c	t	g	a	a	g	c	t	c	c	a	t	a
MH709322	4	t	t	g	a	c	t	a	t	t	t	t	g	c	c	c	a	g	a	a	t	a	a	a	a	g	g	g	g	t	a	c	a	a	t	a	g	g	g	a	c	c	t	t	t	a	a	t	c	g	a	t	c	g	c	g	g	g	t	c	a	a	c	t	g	a	a	g	c	t	c	c	a	t	a
MH709323	4	t	t	g	a	c	t	a	t	t	t	t	g	c	c	c	a	g	a	a	t	a	a	a	a	g	g	g	g	t	a	c	a	a	t	a	g	a	g	a	c	c	t	t	t	a	a	t	c	g	a	t	c	g	c	g	g	g	t	c	a	a	c	t	g	a	a	g	c	t	c	c	a	t	a
MH709326	4	t	t	g	a	c	t	a	t	t	t	t	g	c	c	c	a	g	a	a	t	a	a	a	a	g	g	g	g	t	a	c	a	a	t	a	g	g	g	a	c	c	t	t	t	a	a	t	c	g	a	t	c	g	c	g	g	g	t	c	a	a	c	t	g	a	a	g	c	t	c	c	a	t	a
MH709341	4	t	t	g	a	c	t	a	t	t	t	t	g	c	c	c	a	g	a	a	t	a	a	a	a	g	g	g	g	t	a	c	a	a	t	a	g	g	g	a	c	c	t	t	t	a	a	t	c	g	a	t	c	g	c	g	g	g	t	c	a	a	c	t	g	a	a	g	c	t	c	c	a	t	a
MH709357	4	t	t	g	a	c	t	a	t	t	t	t	g	c	c	c	a	g	a	a	t	a	a	a	a	g	g	g	g	t	a	c	a	a	t	a	g	g	g	a	c	c	t	t	t	a	a	t	c	g	a	t	c	g	c	g	g	g	t	c	a	a	c	t	g	a	a	g	c	t	c	c	a	t	a
MH709364	4	t	t	g	a	c	t	a	t	t	t	t	g	c	c	c	a	g	a	a	t	a	a	a	a	g	g	g	g	t	a	c	a	a	t	a	g	g	g	a	c	c	t	t	t	a	a	t	c	g	a	t	c	g	c	g	g	g	t	c	a	a	c	t	g	a	a	g	c	t	c	c	a	t	a
MH709370	4	t	t	g	a	c	t	a	t	t	t	t	g	c	c	c	a	g	a	a	t	a	a	a	a	g	g	g	g	t	a	c	a	a	t	a	g	g	g	a	c	c	t	t	t	a	a	t	c	g	a	t	c	g	c	g	g	g	t	c	a	a	c	t	g	a	a	g	c	t	c	c	a	t	a
MH709371	4	t	t	g	a	c	t	a	t	t	t	t	g	c	c	c	a	g	a	a	t	a	a	a	a	g	g	g	g	t	a	c	a	a	t	a	g	g	g	a	c	c	t	t	t	a	a	t	c	g	a	t	c	g	c	g	g	g	t	c	a	a	c	t	g	a	a	g	c	t	c	c	a	t	a
MH709372	4	t	t	g	a	c	t	a	t	t	t	t	g	c	c	c	a	g	a	a	t	a	a	a	a	g	g	g	g	t	a	c	a	a	t	a	g	g	g	a	c	c	t	t	t	a	a	t	c	g	a	t	c	g	c	g	g	g	t	c	a	a	c	t	g	a	a	g	c	t	c	c	a	t	a
DQ457052	5	t	t	g	a	c	t	a	t	c	t	g	g	c	c	c	a	g	a	a	t	a	g	a	a	g	g	g	g	t	a	c	a	a	t	a	g	g	g	a	c	c	t	t	t	a	a	t	c	g	a	t	c	g	c	g	g	g	t	c	a	a	c	t	g	a	a	g	c	t	c	c	a	t	a
JN704704	5	t	t	g	a	c	t	a	t	c	t	g	g	c	c	c	a	g	a	a	t	a	a	a	a	g	g	g	g	t	a	c	a	a	t	a	g	g	g	a	c	c	t	t	t	a	a	t	c	g	a	t	c	g	c	g	g	g	t	c	a	a	c	t	g	a	a	g	c	t	c	c	a	t	a
JN704705	5	t	t	g	c	c	t	a	t	c	t	t	g	c	c	c	a	g	a	a	t	a	a	a	a	g	g	g	g	t	a	c	a	a	t	a	g	g	g	a	c	c	t	t	t	a	a	t	c	g	a	t	c	g	c	g	g	g	t	c	a	a	c	t	g	a	a	g	c	t	c	c	a	t	a
JN704706	5	t	t	g	a	c	t	a	g	c	t	t	g	c	c	c	a	g	a	a	t	a	a	a	a	g	g	g	g	t	a	c	a	a	t	a	g	g	g	a	c	c	t	t	t	a	a	t	c	g	a	t	c	g	c	g	g	g	t	c	a	a	c	t	g	a	a	g	c	t	c	c	a	t	a
JN704707	5	t	t	g	a	c	t	a	t	c	t	g	g	c	c	c	a	g	a	a	t	a	a	a	a	g	g	g	g	t	a	c	a	a	t	a	g	g	g	a	c	c	t	c	t	a	a	t	c	g	a	t	c	g	c	g	g	g	t	c	a	a	c	t	g	a	a	g	c	t	c	c	a	t	a
JN704708	5	t	t	g	c	c	t	a	t	c	t	t	g	c	c	c	a	g	a	a	t	a	a	a	a	g	g	g	g	t	a	c	a	a	t	a	g	g	g	a	c	c	t	t	t	a	a	t	c	g	a	t	c	g	c	g	g	g	t	c	a	a	c	t	g	a	a	g	c	t	c	c	a	t	a
KP771904	5	t	t	g	a	c	t	a	t	c	t	g	g	c	c	c	a	g	a	a	t	a	a	a	a	g	g	g	g	t	a	c	a	a	t	a	g	g	g	a	c	c	t	t	t	a	a	t	c	g	a	t	c	g	c	g	g	g	t	c	a	a	c	t	g	a	a	g	c	t	c	c	a	t	a
KP771905	5	t	t	g	a	c	t	a	t	c	t	g	g	c	c	c	a	g	a	a	t	a	a	a	a	g	g	g	g	t	a	c	a	a	t	a	g	g	g	a	c	c	t	t	t	a	a	t	c	g	a	t	c	g	c	g	g	g	t	c	a	a	c	t	g	a	a	g	c	t	c	c	a	t	a
KP771906	5	t	t	g	a	c	t	a	t	c	t	g	g	c	c	c	a	g	a	a	t	a	a	a	a	g	g	g	g	t	a	c	a	a	t	a	g	g	g	a	c	c	t	t	t	a	a	t	c	g	a	t	c	g	c	g	g	g	t	c	a	a	c	t	g	a	a	g	c	t	c	c	a	t	a
KP771919	5	t	t	g	a	c	t	a	t	c	t	g	g	c	c	c	a	g	a	a	t	a	a	a	a	g	g	g	g	t	a	c	a	a	t	a	g	g	g	a	c	c	t	t	t	a	a	t	c	g	a	t	c	g	t	g	g	g	t	c	a	a	c	t	g	a	a	g	t	t	c	c	a	t	a
KP771922	5	t	t	g	a	c	t	a	t	c	t	g	g	c	c	c	a	g	a	a	t	a	a	a	a	g	g	g	g	t	a	c	a	a	t	a	g	g	g	a	c	c	t	c	t	a	a	t	c	g	a	t	c	g	c	g	g	g	t	c	a	a	c	t	g	a	a	g	c	t	c	c	a	g	a
KP771924	5	t	t	g	c	c	t	a	t	c	t	t	g	c	c	c	a	g	a	a	t	a	a	a	a	g	g	g	g	t	a	c	a	a	t	a	g	g	g	a	c	c	t	t	t	a	a	t	c	g	a	t	c	g	c	g	g	g	t	c	a	a	c	c	g	a	a	g	c	t	c	c	a	t	a
KM355696	5	t	t	g	c	c	t	a	t	c	t	t	g	c	c	c	a	g	a	a	t	a	a	a	a	g	g	g	g	t	a	c	a	a	t	a	g	g	g	a	c	c	t	t	t	a	a	t	c	g	a	t	c	g	c	g	g	g	t	c	a	a	c	t	g	a	a	g	c	t	c	c	a	t	a
KM355697	5	t	t	g	c	c	t	a	t	c	t	t	g	c	c	c	a	g	a	a	t	a	a	a	a	g	g	g	g	t	a	c	a	a	t	a	g	g	g	a	c	c	t	t	t	a	a	t	c	g	a	t	c	g	c	g	g	g	t	c	a	a	c	t	g	a	a	g	c	t	c	c	a	t	a
KM355698	5	t	t	g	c	c	t	a	t	c	t	t	g	c	c	c	a	g	a	a	t	a	a	a	a	g	g	g	g	t	a	t	a	a	t	a	g	g	g	a	c	c	t	t	t	a	a	t	c	g	a	t	c	g	c	g	g	g	t	c	a	a	c	t	g	a	a	g	c	t	c	c	a	t	a
KM355699	5	t	t	g	c	c	t	a	t	c	t	t	g	c	c	c	a	g	a	a	t	a	a	a	a	g	g	g	g	t	a	c	a	a	t	a	g	g	g	a	c	c	t	t	t	a	a	t	c	g	a	t	c	g	c	g	g	g	t	c	a	a	c	t	g	a	a	g	c	t	c	c	a	t	a
KM355701	5	t	t	g	c	c	t	a	t	c	t	t	g	c	c	c	a	g	a	a	t	a	a	a	a	g	g	g	g	t	a	c	a	a	t	a	g	g	g	a	c	c	t	t	t	a	a	t	c	g	a	t	c	g	c	g	g	g	t	c	a	a	c	t	g	a	a	g	c	t	c	c	a	t	a
KM355702	5	t	t	g	c	c	t	a	t	c	t	t	g	c	c	c	a	g	a	a	t	a	a	a	a	g	g	g	g	t	a	c	a	a	t	a	g	g	g	a	c	c	t	t	t	a	a	t	c	g	a	t	c	g	c	g	g	g	t	c	a	a	c	t	g	a	a	g	c	t	c	c	a	t	a
KM355703	5	t	t	g	c	c	t	a	t	c	t	t	g	c	c	c	a	g	a	a	t	a	a	a	a	g	g	g	g	t	a	c	a	a	t	a	g	g	g	a	c	c	t	t	t	a	a	t	c	g	a	t	c	g	c	g	g	g	t	c	a	a	c	t	g	a	a	g	c	t	c	c	a	t	a
KM355704	5	t	t	g	c	c	t	a	t	c	t	t	g	c	c	c	a	g	a	a	t	a	a	a	a	g	g	g	g	t	a	c	a	a	t	a	g	g	g	a	c	c	t	t	t	a	a	t	c	g	a	t	c	g	c	g	g	g	t	c	a	a	c	t	g	a	a	g	c	t	c	c	a	t	a
KM355705	5	t	t	g	c	c	t	a	t	c	t	t	g	c	c	c	a	g	a	a	t	a	a	a	a	g	g	g	g	t	a	c	a	a	t	a	g	g	g	a	c	c	t	t	t	a	a	t	c	g	a	t	c	g	c	g	g	g	t	c	a	a	c	t	g	a	a	g	c	t	c	c	a	t	a
KM355706	5	t	t	g	c	c	t	a	t	c	t	t	g	c	c	c	a	g	a	a	t	a	a	a	a	g	g	g	g	t	a	c	a	a	t	a	g	g	g	a	c	c	t	t	t	a	a	t	c	g	a	t	c	g	c	g	g	g	t	c	a	a	c	t	g	a	a	g	c	t	c	c	a	t	a
KM355707	5	t	t	g	c	c	t	a	t	c	t	t	g	c	c	c	a	g	a	a	t	a	a	a	a	g	g	g	g	t	a	c	a	a	t	a	g	g	g	a	c	c	t	t	t	a	a	t	c	g	a	t	c	g	c	g	g	g	t	c	a	a	c	t	g	a	a	g	c	t	c	c	a	t	a
KM355708	5	t	t	g	c	c	t	a	t	c	t	t	g	c	c	c	a	g	a	a	t	a	a	a	a	g	g	g	g	t	a	c	a	a	t	a	g	g	g	a	c	c	t	t	t	a	a	t	c	g	a	t	c	g	c	g	g	g	t	c	a	a	c	t	g	a	a	g	c	t	c	c	a	t	a
KM355709	5	t	t	g	c	c	t	a	t	c	t	t	g	c	c	c	a	g	a	a	t	a	a	a	a	g	g	g	g	t	a	c	a	a	t	a	g	g	g	a	c	c	t	t	t	a	a	t	c	g	a	t	c	g	c	g	g	g	t	c	a	a	c	t	g	a	a	g	c	t	c	c	a	t	a
KM355711	5	t	t	g	c	c	t	a	t	c	t	t	g	c	c	c	a	g	a	a	t	a	a	a	a	g	g	g	g	t	a	c	a	a	t	a	g	g	g	a	c	c	t	t	t	a	a	t	c	g	a	t	c	g	c	g	g	g	t	c	a	a	c	t	g	a	a	g	c	t	c	c	a	t	a
KM355712	5	t	t	g	c	c	t	a	t	c	t	t	g	c	c	c	a	g	a	a	t	a	a	a	a	g	g	g	g	t	a	c	a	a	t	a	g	g	g	a	c	c	t	t	t	a	a	t	c	g	a	t	c	g	c	g	g	g	t	c	a	a	c	t	g	a	a	g	c	t	c	c	a	t	a
KY037797	5	[illegible]	[illegible]	[illegible]	[illegible]	[illegible]	[illegible]	[illegible]	[illegible]	[illegible]	[illegible]	[illegible]	[illegible]	[illegible]	[illegible]	[illegible]	[illegible]	[illegible]	[illegible]	[illegible]	[illegible]	[illegible]	[illegible]	[illegible]	[illegible]	[illegible]	[illegible]	[illegible]	[illegible]	[illegible]	[illegible]	[illegible]	[illegible]	[illegible]	[illegible]	[illegible]	[illegible]	[illegible]	[illegible]	[illegible]	[illegible]	[illegible]	[illegible]	[illegible]	[illegible]	[illegible]	[illegible]	[illegible]	[illegible]	[illegible]	[illegible]	[illegible]	[illegible]	[illegible]	[illegible]	[illegible]	[illegible]	[illegible]	[illegible]	[illegible]	[illegible]	[illegible]	[illegible]	[illegible]	[illegible]	[illegible]	[illegible]	[illegible]	[illegible]	[illegible]	[illegible]	[illegible]	[illegible]	[illegible]	[illegible]

Supplementary Table 3: 2880 SN

GB-ID	Clade markers									5			9																								
	Position in Dumas	54598	54602	54631	54734	54782	54814	54926	54949	54962	55004	55059	55148	55243	55322	55325	55463	55479	55700	55724	55730	55817	55820	55832	55968	55974	55978	55986	56009	56021	56099	56151	56171	56189	56198	56278	56525
MH499467	5	t	t	g	a	c	t	a	t	c	t	g	g	c	c	c	a	g	a	a	t	a	a	a	a	g	g	g	g	t	a	c	a	a	t	a	g
MH499469	5	t	t	g	a	c	t	a	t	c	t	g	g	c	c	c	a	g	a	a	t	a	a	a	a	g	g	g	g	t	a	c	a	a	t	a	g
MH709321	5	t	t	g	a	c	t	a	t	c	t	t	g	c	c	c	a	g	a	a	t	a	a	a	a	g	g	g	g	t	a	c	a	a	t	a	g
MH709324	5	t	t	g	a	c	t	a	t	c	t	g	g	c	c	c	a	g	a	a	t	a	a	a	a	g	g	g	g	t	a	c	a	a	t	a	g
MH709325	5	t	t	g	c	c	t	a	t	c	t	t	g	c	c	c	a	g	a	a	t	a	a	a	a	g	g	g	g	t	a	c	a	a	t	a	g
MH709327	5	t	t	g	a	c	t	a	t	c	t	g	g	c	c	c	a	g	a	a	t	a	g	a	a	g	g	g	g	t	a	c	a	a	t	a	g
MH709328	5	t	t	g	a	c	t	a	t	c	t	g	g	c	c	c	a	g	a	a	t	a	a	a	a	g	g	g	g	t	a	c	a	a	t	a	g
MH709330	5	t	t	g	a	c	t	a	t	c	t	g	g	c	c	c	a	g	a	a	c	a	a	a	a	g	g	g	g	t	a	c	a	a	t	a	g
MH709332	5	t	t	g	a	c	t	a	t	c	t	g	g	c	c	c	a	g	a	a	t	a	a	a	a	g	g	g	a	t	a	c	a	a	t	a	g
MH709334	5	t	t	g	a	c	t	a	t	c	t	g	g	c	c	c	a	g	a	a	t	a	g	a	a	g	g	g	g	t	a	c	a	a	t	a	g
MH709335	5	t	t	g	a	c	t	a	t	c	t	g	g	c	c	c	a	g	a	a	t	a	g	a	a	g	g	g	g	t	a	c	a	a	t	a	g
MH709336	5	t	t	g	a	c	t	a	t	c	t	g	g	c	c	c	a	g	a	a	t	a	g	a	a	g	g	g	g	t	a	c	a	a	t	a	g
MH709338	5	t	t	g	a	c	t	a	t	c	t	g	g	c	c	c	a	g	a	a	t	a	g	a	a	g	g	g	g	t	a	c	a	a	t	a	g
MH709340	5	t	t	g	a	c	t	a	t	c	t	t	g	c	c	c	a	g	a	a	t	a	a	a	a	g	g	g	g	t	a	c	a	a	t	a	g
MH709342	5	t	t	g	a	c	t	a	t	c	t	t	g	c	c	c	a	g	a	a	t	a	a	a	a	g	g	g	g	t	a	c	a	a	t	a	g
MH709343	5	t	t	g	a	c	t	a	t	c	t	g	g	c	c	c	a	g	a	a	t	a	a	a	a	g	g	g	g	t	a	c	a	a	t	a	g
MH709345	5	t	t	g	a	c	t	a	t	c	t	g	g	c	c	c	a	g	a	a	t	a	g	a	a	g	g	g	g	t	a	c	a	a	t	a	g
MH709347	5	t	t	g	a	c	t	a	t	c	t	g	g	c	c	c	a	g	a	a	t	a	g	a	a	g	g	g	g	t	a	c	a	a	t	a	g
MH709349	5	t	t	g	a	c	t	a	t	c	t	g	g	c	c	c	a	g	a	a	t	a	a	a	a	g	g	g	g	t	a	c	a	a	t	a	g
MH709350	5	t	t	g	a	c	t	a	t	c	t	g	g	c	c	c	a	g	a	a	t	a	g	a	g	g	g	g	g	t	a	c	a	a	c	a	g
MH709351	5	t	t	g	a	c	t	a	t	c	t	t	g	c	c	c	a	g	a	a	t	a	a	a	a	g	g	g	g	t	a	c	a	a	t	a	g
MH709353	5	t	t	g	a	c	t	a	t	c	t	g	g	c	c	c	a	g	a	a	t	a	g	a	a	g	g	g	g	t	a	c	a	a	t	a	g
MH709358	5	t	t	g	c	c	t	a	t	c	t	t	g	c	c	c	a	g	a	a	t	a	a	a	a	g	g	g	g	t	a	c	a	a	t	a	g
MH709360	5	t	t	g	a	c	t	a	t	c	t	g	g	c	c	c	a	g	a	a	t	a	a	a	a	g	g	a	g	t	a	c	a	a	t	a	g
MH709362	5	t	t	g	a	c	t	a	t	c	t	g	g	c	c	c	a	g	a	a	t	a	g	a	a	g	g	g	g	t	a	c	a	a	t	a	g
MH709365	5	t	t	g	a	c	t	a	t	c	t	g	g	c	c	c	a	g	a	a	t	a	g	a	g	g	g	g	g	t	a	c	a	a	t	a	g
MH709373	5	t	t	g	a	c	t	a	t	c	t	g	g	c	c	c	a	g	a	a	t	a	a	a	a	g	g	g	g	t	a	c	a	a	t	a	g
MH709374	5	t	t	g	a	c	t	a	t	c	t	g	g	c	c	c	a	g	a	a	t	a	g	a	a	g	g	g	g	t	a	c	a	a	t	a	g
KC112914	6	t	t	g	a	c	t	a	t	t	c	t	g	c	c	c	a	g	a	a	t	a	a	a	a	g	g	g	g	t	a	c	a	a	t	a	g
KP771914	6	t	t	g	a	c	t	a	t	t	c	t	g	c	c	c	a	g	a	a	t	a	a	a	a	g	g	g	g	t	a	c	a	a	t	a	g
KP702725	6	t	t	g	a	c	t	a	t	t	c	t	g	c	c	c	a	g	a	g	t	a	a	a	a	g	g	g	g	t	a	c	a	a	t	a	g
KP771890	9	t	t	g	a	c	t	a	t	t	t	t	a	c	c	c	a	g	a	a	t	a	a	a	a	g	g	g	g	t	a	c	a	a	t	a	g
JN704710	9	t	t	g	a	c	t	a	t	t	t	t	a	c	c	c	a	g	a	a	t	a	a	a	a	g	g	g	g	t	a	c	a	a	t	a	g
KY037798	9	t	t	g	a	c	t	a	t	t	t	t	a	c	c	c	a	g	a	a	t	a	a	a	a	g	g	g	g	t	a	c	a	a	t	a	g
MH709312	9	t	t	g	a	c	t	a	t	t	t	t	a	c	c	c	a	g	a	a	t	a	a	a	a	g	g	g	g	t	a	c	a	a	t	a	g
MH709352	9	t	t	g	a	t	t	a	t	t	t	t	a	c	c	c	a	g	a	a	t	a	a	a	a	g	g	g	g	t	a	c	a	a	t	a	g
AB097933	Vac	t	t	g	a	c	t	a	t	t	t	t	g	c	c	c	a	g	a	a	t	a	g	a	a	g	g	g	g	t	a	c	a	a	t	a	g
JN704698	Vac	t	t	g	a	c	t	a	t	t	t	t	g	c	c	c	a	g	a	a	t	a	g	a	a	g	g	g	g	t	a	c	a	a	t	a	g
JN704697	Vac	t	t	g	a	c	t	a	t	t	t	t	g	c	c	c	a	g	a	a	t	a	g	a	a	g	g	g	g	t	a	c	a	a	t	a	g
DQ008355	Vac	t	t	g	a	c	t	a	t	t	t	t	g	c	c	c	a	g	a	a	t	a	g	a	a	g	g	g	g	t	a	c	a	a	t	a	g
JF306641	Vac	t	t	g	a	c	t	a	t	t	t	t	g	c	c	c	a	g	a	a	t	a	g	a	a	g	g	g	g	t	a	c	a	a	t	a	g
AB097932	Vac	t	t	g	a	c	t	a	t	t	t	t	g	c	c	c	a	g	a	a	t	a	g	a	a	g	g	g	g	t	a	c	a	a	t	a	g
DQ008354	Vac	t	t	g	a	c	t	a	t	t	t	t	g	c	c	c	a	g	a	a	t	a	g	a	a	g	g	g	g	t	a	c	a	a	t	a	g
KU926312	Vac	t	t	g	a	c	t	a	t	t	t	t	g	c	c	c	a	g	a	a	t	a	g	a	a	g	g	g	g	t	a	c	a	a	t	a	g
KU926313	Vac	t	t	g	a	c	t	a	t	t	t	t	g	c	c	c	a	g	a	a	t	a	g	a	a	g	g	g	g	t	a	c	a	a	t	a	g
KU926314	Vac	t	t	g	a	c	t	a	t	t	t	t	g	c	c	c	a	g	a	a	t	a	g	a	a	g	g	g	g	t	a	c	a	a	t	a	g
KF558371	Vac	t	t	g	a	c	t	a	t	t	t	t	g	c	c	c	a	g	a	a	t	a	g	a	a	g	g	g	g	t	a	c	a	a	t	a	g
KF558372	Vac	t	t	g	a	c	t	a	t	t	t	t	g	c	c	c	a	g	a	a	t	a	g	a	a	g	g	g	g	t	a	c	a	a	t	a	g
KF558373	Vac	t	t	g	a	c	t	a	t	t	t	t	g	c	c	c	a	g	a	a	t	a	g	a	a	g	g	g	g	t	a	c	a	a	t	a	g
KF558374	Vac	t	t	g	a	c	t	a	t	t	t	t	g	c	c	c	a	g	a	a	t	a	g	a	a	g	g	g	g	t	a	c	a	a	t	a	g
KF558375	Vac	t	t	g	a	c	t	a	t	t	t	t	g	c	c	c	a	g	a	a	t	a	g	a	a	g	g	g	g	t	a	c	a	a	t	a	g
KF558376	Vac	t	t	g	a	c	t	a	t	t	t	t	g	c	c	c	a	g	a	a	t	a	g	a	a	g	g	g	g	t	a	c	a	a	t	a	g
KF558377	Vac	t	t	g	a	c	t	a	t	t	t	t	g	c	c	c	a	g	a	a	t	a	g	a	a	g	g	g	g	t	a	c	a	a	t	a	g
KF558378	Vac	t	t	g	a	c	t	a	t	t	t	t	g	c	c	c	a	g	a	a	t	a	g	a	a	g	g	g	g	t	a	c	a	a	t	a	g
KF558379	Vac	t	t	g	a	c	t	a	t	t	t	t	g	c	c	c	a	g	a	a	t	a	g	a	a	g	g	g	g	t	a	c	a	a	t	a	g
KF558380	Vac	t	t	g	a	c	t	a	t	t	t	t	g	c	c	c	a	g	a	a	t	a	g	a	a	g	g	g	g	t	a	c	a	a	t	a	g
KF558381	Vac	t	t	g	a	c	t	a	t	t	t	t	g	c	c	c	a	g	a	a	t	a	g	a	a	g	g	g	g	t	a	c	a	a	t	a	g
KF558382	Vac	t	t	g	a	c	t	a	t	t	t	t	g	c	c	c	a	g	a	a	t	a	g	a	a	g	g	g	g	t	a	c	a	a	t	a	g
KF558383	Vac	t	t	g	a	c	t	a	t	t	t	t	g	c	c	c	a	g	a	a	t	a	g	a	a	g	g	g	g	t	a	c	a	a	t	a	g
KF558384	Vac	t	t	g	a	c	t	a	t	t	t	t	g	c	c	c	a	g	a	a	t	a	g	a	a	g	g	g	g	t	a	c	a	a	t	a	g
KF558385	Vac	t	t	g	a	c	t	a	t	t	t	t	g	c	c	c	a	g	a	a	t	a	g	a	a	g	g	g	g	t	a	c	a	a	t	a	g
KF558386	Vac	t	t	g	a	c	t	a	t	t	t	t	g	c	c	c	a	g	a	a	t	a	g	a	a	g	g	g	g	t	a	c	a	a	t	a	g
KF558387	Vac	t	t	g	a	c	t	a	t	t	t	t	g	c	c	c	a	g	a	a	t	a	g	a	a	g	g	g	g	t	a	c	a	a	t	a	g
KF558388	Vac	t	t	g	a	c	t	a	t	t	t	t	g	c	c	c	a	g	a	a	t	a	g	a	a	g	g	g	g	t	a	c	a	a	t	a	g
KF558389	Vac	t	t	g	a	c	t	a	t	t	t	t	g	c	c	c	a	g	a	a	t	a	g	a	a	g	g	g	g	t	a	c	a	a	t	a	g
KF558390	Vac	t	t	g	a	c	t	a	t	t	t	t	g	c	c	c	a	g	a	a	t	a	g	a	a	g	g	g	g	t	a	c	a	a	t	a	g
KF558391	Vac	t	t	g	a	c	t	a	t	t	t	t	g	c	c	c	a	g	a	a	t	a	g	a	a	g	g	g	g	t	a	c	a	a	t	a	g
KF811485	Vac	t	t	g	a	c	t	a	t	t	t	t	g	c	c	c	a	g	a	a	t	a	g	a	a	g	g	g	g	t	a	c	a	a	t	a	g
KF853225	Vac	t	t	g	a	c	t	a	t	t	t	t	g	c	c	c	a	g	a	a	t	a	g	a	a	g	g	g	g	t	a	c	a	a	t	a	g
KF853226	Vac	t	t	g	a	c	t	a	t	t	t	t	g	c	c	c	a	g	a	a	t	a	g	a	a	g	g	g	g	t	a	c	a	a	t	a	g
KF853227	Vac	t	t	g	a	c	t	a	t	t	t	t	g	c	c	c	a	g	a	a	t	a	g	a	a	g	g	g	g	t	a	c	a	a	t	a	g
KF853228	Vac	t	t	g	a	c	t	a	t	t	t	t	g	c	c	c	a	g	a	a	t	a	g	a	a	g	g	g	g	t	a	c	a	a	t	a	g
KF853229	Vac	t	t	g	a	c	t	a	t	t	t	t	g	c	c	c	a	g	a	a	t	a	g	a	a	g	g	g	g	t	a	c	a	a	t	a	g
KF853230	Vac	t	t	g	a	c	t	a	t	t	t	t	g	c	c	c	a	g	a	a	t	a	g	a	a	g	g	g	g	t	a	c	a	a	t	a	g
KF853231	Vac	t	t	g	a	c	t	a	t	t	t	t	g	c	c	c	a	g	a	a	t	a	g	a	a	g	g	g	g	t	a	c	a	a	t	a	g
KF853232	Vac	t	t	g	a	c	t	a	t	t	t	t	g	c	c	c	a	g	a	a	t	a	g	a	a	g	g	g	g	t	a	c	a	a	t	a	g
KF853233	Vac	t	t	g	a	c	t	a	t	t	t	t	g	c	c	c	a	g	a	a	t	a	g	a	a	g	g	g	g	t	a	c	a	a	t	a	g
KF853234	Vac	t	t	g	a	c	t	a	t	t	t	t	g	c	c	c	a	g	a	a	t	a	g	a	a	g	g	g	g	t	a	c	a	a	t	a	g
KF853235	Vac	t	t	g	a	c	t	a	t	t	t	t	g	c	c	c	a	g	a	a	t	a	g	a	a	g	g	g	g	t	a	c	a	a	t	a	g
MF898328	Vac	t	t	g	a	c	t	a	t	t	t	t	g	c	c	c	a	g	a	a	t	a	g	a	a	g	g	g	g	t	a	c	a	a	t	a	g
JN704709	VIII	t	t	g	a	c	t	a	t	t	c	t	g	c	c	c	a	g	a	a	t	a	a	a	a	g	g	g	g	t	a	c	a	a	t	a	g

GB-ID										2			2																									
	56648	56807	56942	56965	57006	57025	57047	57080	57206	57224	57271	57301	57397	57444	57508	57524	57643	57706	57886	57887	57916	57955	58054	58072	58117	58156	58162	58183	58325	58364	58441	58459	58495	58510	58549	58595	58742	58771
MH499467	g	g	a	c	c	t	t	t	a	a	t	c	g	a	t	c	g	c	g	g	g	t	c	a	a	c	t	g	a	a	g	c	t	c	c	a	t	a
MH499469	g	g	a	c	c	t	t	t	a	a	t	c	g	a	t	c	g	c	g	g	g	t	c	a	a	c	t	g	a	a	g	t	t	c	c	a	t	a
MH709321	g	g	a	c	c	t	t	t	a	a	t	c	g	a	t	c	g	c	g	g	g	t	c	a	a	c	t	g	a	a	g	c	t	c	c	a	t	a
MH709324	g	g	a	c	c	t	t	t	a	a	t	c	g	a	t	c	g	c	g	g	g	t	c	a	a	c	t	g	a	a	g	t	t	c	c	a	t	a
MH709325	g	g	a	c	c	t	t	t	a	a	t	c	g	a	t	c	g	c	g	g	g	t	c	a	a	c	t	g	a	a	g	c	t	c	c	a	t	a
MH709327	g	g	a	c	c	t	t	t	a	a	t	c	g	a	t	c	g	c	g	g	g	t	c	a	a	c	t	g	a	a	g	c	t	c	c	a	t	a
MH709328	g	g	a	c	c	t	t	t	a	a	t	c	g	a	t	c	g	c	g	g	g	t	c	a	a	c	t	g	a	a	g	t	t	c	c	a	t	a
MH709330	g	g	a	c	c	t	t	t	a	a	t	c	g	a	t	c	g	c	g	g	g	t	c	a	a	c	t	g	a	a	g	c	t	c	c	a	t	a
MH709332	g	g	a	c	c	t	t	t	a	a	t	c	g	a	t	c	g	c	g	g	g	t	c	c	a	c	t	g	a	a	g	t	t	c	c	a	t	a
MH709334	g	g	a	c	c	t	t	t	a	a	t	c	g	a	t	c	g	c	g	g	g	t	c	a	a	c	t	g	a	a	g	c	t	c	c	a	t	a
MH709335	g	g	a	c	c	t	t	t	a	a	t	c	g	a	t	c	g	c	g	g	g	t	c	a	a	c	t	g	a	a	g	c	t	c	c	a	t	a
MH709336	g	g	a	c	c	t	t	t	a	a	t	c	g	a	t	c	g	c	g	g	g	t	c	a	a	c	t	g	a	a	g	c	t	c	c	a	t	a
MH709338	g	g	a	c	c	t	t	t	a	a	t	c	g	a	t	c	g	c	g	g	g	t	c	a	a	c	t	g	a	a	g	c	t	c	c	a	t	a
MH709340	g	g	a	c	c	t	t	t	a	a	t	c	g	a	t	c	g	c	g	g	g	t	c	a	a	c	t	g	a	a	g	c	t	c	c	a	t	a
MH709342	g	g	a	c	c	t	t	t	a	a	t	c	g	a	t	c	g	c	g	g	g	t	c	a	a	c	t	g	a	a	g	c	t	c	c	a	t	a
MH709343	g	g	a	c	c	t	t	t	a	a	t	c	g	a	t	g	g	c	g	g	g	t	c	a	a	c	t	g	a	a	g	c	t	c	c	a	t	a
MH709345	g	g	a	c	c	t	t	t	a	a	t	c	g	a	t	c	g	c	g	g	g	t	c	a	a	c	t	g	a	a	g	c	t	c	c	a	t	a
MH709347	g	g	a	c	c	t	t	t	a	a	t	c	g	a	t	c	g	c	g	g	g	t	c	a	a	c	t	g	a	a	g	c	t	c	c	a	t	a
MH709349	g	g	a	c	c	t	t	t	a	a	t	c	g	a	t	c	g	c	g	g	g	t	c	a	a	c	t	g	a	a	g	c	t	c	c	a	t	a
MH709350	g	g	a	c	c	t	t	t	a	a	t	c	g	a	t	c	g	c	g	g	g	t	c	a	a	c	t	g	a	a	g	c	t	c	c	a	t	a
MH709351	g	g	a	c	c	t	t	t	a	a	t	c	g	a	t	c	g	c	g	g	g	t	c	a	a	c	t	g	a	a	g	c	t	c	c	a	t	a
MH709353	g	g	a	c	c	t	t	t	a	a	t	c	g	a	t	c	g	c	g	g	g	t	c	a	a	c	t	g	a	a	g	c	t	c	c	a	t	a
MH709358	g	g	a	c	c	t	t	t	a	a	t	c	g	a	t	c	g	c	g	g	g	t	c	a	a	c	t	g	a	a	g	c	t	c	c	a	t	a
MH709360	g	g	a	c	c	t	t	t	a	a	t	c	g	a	t	c	g	c	g	g	g	t	c	a	a	c	t	g	a	a	g	c	t	c	c	a	t	a
MH709362	g	g	a	c	c	t	t	t	a	a	t	c	g	a	t	c	a	c	g	g	g	t	c	a	a	c	t	g	a	a	g	c	t	c	c	a	t	a
MH709365	g	g	a	c	c	t	t	t	a	a	t	c	g	a	t	c	g	c	g	g	g	t	c	a	a	c	t	g	a	a	g	c	t	c	c	a	t	a
MH709373	g	g	a	c	c	t	c	t	a	a	t	c	g	a	t	c	g	c	g	g	g	t	c	a	a	c	t	g	a	a	g	c	t	c	c	a	t	a
MH709374	g	g	a	c	c	t	t	t	a	a	t	c	g	a	t	c	g	c	g	g	g	t	c	a	a	c	t	g	a	a	g	c	t	c	c	a	t	a
KC112914	g	g	a	c	c	t	t	t	a	a	t	c	g	a	t	c	g	c	g	g	g	t	c	a	a	c	t	g	a	a	g	c	t	c	c	a	t	a
KP771914	g	g	a	c	c	t	t	t	a	a	t	c	g	a	t	c	g	c	g	g	g	t	c	a	a	c	t	g	a	a	g	c	t	c	c	a	t	a
KP702725	g	g	a	c	c	t	t	t	a	a	t	c	g	g	t	c	g	c	g	g	g	t	c	a	a	c	t	g	a	a	g	c	t	c	c	a	t	a
KP771890	g	g	a	c	c	t	t	t	a	a	t	a	g	a	t	c	g	c	g	g	g	c	c	a	a	c	t	g	a	a	g	c	t	c	c	a	t	a
JN704710	g	g	a	c	c	t	t	t	a	a	t	a	g	a	t	c	g	c	g	g	g	c	c	a	a	c	t	g	a	a	g	c	t	c	c	a	t	a
KY037798	g	g	a	c	c	t	t	t	a	a	t	a	g	a	t	c	g	c	g	g	g	c	c	a	a	c	t	g	a	a	g	c	t	c	c	a	t	a
MH709312	g	g	a	c	c	t	t	t	a	a	t	a	g	a	t	c	g	c	g	g	g	c	c	a	a	c	t	g	a	a	g	c	t	c	c	a	t	a
MH709352	g	g	a	c	c	t	t	t	a	a	t	a	g	a	t	c	g	c	g	g	g	c	c	a	a	c	t	g	a	a	g	c	t	c	c	a	t	a
AB097933	g	g	a	c	c	t	t	t	a	c	t	c	t	a	t	c	g	c	g	g	g	c	c	a	a	c	t	g	a	a	g	c	t	c	c	a	t	a
JN704698	g	g	a	c	c	t	t	t	a	c	t	c	t	a	t	c	g	c	g	g	g	c	c	a	a	c	t	g	a	a	g	c	t	c	c	a	t	a
JN704697	g	g	a	c	c	t	t	t	a	c	t	c	t	a	t	c	g	c	g	g	g	c	c	a	a	c	t	g	a	a	g	c	t	c	c	a	t	a
DQ008355	g	g	a	c	c	t	t	t	a	c	t	c	t	a	t	c	g	c	g	g	g	c	c	a	a	c	t	g	a	a	g	c	t	c	c	g	t	a
JF306641	g	g	a	c	c	t	t	t	a	c	t	c	t	a	t	c	g	c	g	g	g	c	c	a	a	c	t	g	a	a	g	c	t	c	c	g	t	a
AB097932	g	g	a	c	c	t	t	t	a	c	t	c	t	a	t	c	g	c	g	g	g	c	c	a	a	c	t	g	a	a	g	c	t	c	c	r	t	a
DQ008354	g	g	a	c	c	t	t	t	a	c	t	c	t	a	t	c	g	c	g	g	g	c	c	a	a	c	t	g	a	a	g	c	t	c	c	r	t	a
KU926312	g	g	a	c	c	t	t	t	a	c	t	c	t	a	t	c	g	c	g	g	g	c	c	a	a	c	t	g	a	a	g	c	t	c	c	g	t	a
KU926313	g	g	a	c	c	t	t	t	a	c	t	c	t	a	t	c	g	c	g	g	g	c	c	a	a	c	t	g	a	a	g	c	t	c	c	a	t	a
KU926314	g	g	a	c	c	t	t	t	a	c	t	c	t	a	t	c	g	c	g	g	g	c	c	a	a	c	t	g	a	a	g	c	t	c	c	a	t	a
KF558371	g	g	a	c	c	t	t	t	a	c	t	c	t	a	t	c	g	c	g	g	g	c	c	a	a	c	t	g	a	a	g	c	t	c	c	g	t	g
KF558372	g	g	a	c	c	t	t	t	a	c	t	c	t	a	t	c	g	c	g	g	g	c	c	a	a	c	t	g	a	a	g	c	t	c	c	g	t	a
KF558373	g	g	a	c	c	t	t	c	a	c	t	c	t	a	t	c	g	c	g	g	g	c	c	a	a	c	t	g	a	a	g	c	t	c	c	g	t	a
KF558374	g	g	a	c	c	t	t	t	a	c	t	c	t	a	t	c	g	c	g	g	g	c	c	a	a	c	t	g	a	a	g	c	t	c	c	a	t	a
KF558375	g	g	a	c	c	t	t	t	a	c	t	c	t	a	t	c	g	c	g	g	g	c	c	a	a	c	t	g	a	a	g	c	t	c	c	g	t	a
KF558376	g	g	a	c	c	t	t	t	a	c	t	c	t	a	t	c	g	c	g	g	g	c	c	a	a	c	t	g	a	a	g	c	t	c	c	g	t	a
KF558377	g	g	a	c	c	t	t	t	a	c	t	c	t	a	t	c	g	c	g	g	g	c	c	a	a	c	t	g	a	a	g	c	t	c	c	g	t	a
KF558378	g	g	a	c	c	t	t	t	a	c	t	c	t	a	t	c	g	c	g	g	g	c	c	a	a	c	t	g	a	a	g	c	t	c	c	g	t	a
KF558379	g	g	a	c	c	t	t	t	a	c	t	c	t	a	t	c	g	c	g	g	g	c	c	a	a	c	t	g	a	a	g	c	t	c	c	a	t	a
KF558380	g	g	a	c	c	t	t	t	a	c	t	c	t	a	t	c	g	c	g	g	g	c	c	a	a	c	t	g	a	a	g	c	t	c	c	a	t	a
KF558381	g	g	a	c	c	t	t	t	a	c	t	c	t	a	t	c	g	c	g	g	g	c	c	a	a	c	t	g	a	a	g	c	t	c	c	g	t	a
KF558382	g	g	a	c	c	t	t	t	g	c	t	c	t	a	t	c	g	c	g	g	g	c	c	a	a	c	t	g	a	a	g	c	t	c	c	a	t	a
KF558383	g	g	a	c	c	t	t	t	a	c	t	c	t	a	t	c	g	c	g	g	g	c	c	a	a	c	t	g	a	a	g	c	t	c	c	g	t	a
KF558384	g	g	a	c	c	t	t	t	a	c	t	c	t	a	t	c	g	c	g	g	g	c	c	a	a	c	t	g	a	a	g	c	t	c	c	g	t	a
KF558385	g	g	a	c	c	t	t	t	a	c	t	c	t	a	t	c	g	c	g	g	g	c	c	a	a	c	t	g	a	a	g	c	t	c	c	g	t	a
KF558386	g	g	a	c	c	t	t	t	a	c	t	c	t	a	t	c	g	c	g	g	g	c	c	a	a	c	t	g	a	a	g	c	t	c	c	g	t	a
KF558387	g	g	a	c	c	t	t	t	a	c	t	c	t	a	t	c	g	c	g	g	g	c	c	a	a	c	t	g	a	a	g	c	t	c	c	g	t	a
KF558388	g	g	a	c	c	t	t	t	a	c	t	c	t	a	t	c	g	c	g	g	g	c	c	a	a	c	t	g	a	a	g	c	t	c	c	a	t	a
KF558389	g	g	a	c	c	t	t	t	a	c	t	c	t	a	t	c	g	c	g	g	g	c	c	a	a	c	t	g	a	a	g	c	t	c	c	g	t	a
KF558390	g	g	a	c	c	t	t	t	a	c	t	c	t	a	t	c	g	c	g	g	g	c	c	a	a	c	t	g	a	a	g	c	t	c	c	g	t	a
KF558391	g	g	a	c	c	t	t	t	a	c	t	c	t	a	t	c	g	c	g	g	g	c	c	a	a	c	t	g	a	a	g	c	t	c	c	g	t	a
KF811485	g	g	a	c	c	t	t	t	a	c	t	c	t	a	t	c	g	c	g	g	g	c	c	a	a	c	t	g	a	a	g	c	t	c	c	g	t	a
KF853225	g	g	a	c	c	t	t	t	a	c	t	c	t	a	t	c	g	c	g	a	g	c	c	a	a	c	t	g	a	a	g	c	t	c	c	g	t	a
KF853226	g	g	a	c	c	t	t	t	a	c	t	c	t	a	t	c	g	c	g	g	g	c	c	a	a	c	t	g	a	a	g	c	t	c	c	g	t	a
KF853227	g	g	a	c	c	t	t	t	a	c	t	c	t	a	t	c	g	c	g	g	g	c	c	a	a	c	t	g	a	a	g	c	t	c	c	a	t	a
KF853228	g	g	a	c	c	t	t	t	a	c	t	c	t	a	t	c	g	c	g	g	g	c	c	a	a	c	t	g	a	a	g	c	t	c	c	g	t	a
KF853229	g	g	a	c	c	t	t	t	a	c	t	c	t	a	t	c	g	c	g	g	g	c	c	a	a	c	t	g	a	a	g	c	t	c	c	g	t	a
KF853230	g	g	a	c	c	t	t	c	a	c	t	c	t	a	t	c	g	c	g	g	g	c	c	a	a	c	t	g	a	a	g	c	t	c	c	a	t	a
KF853231	g	g	a	c	c	t	t	t	a	c	t	c	t	a	t	c	g	c	g	g	g	c	c	a	a	c	t	g	a	a	g	c	t	c	c	g	t	a
KF853232	g	g	a	c	c	t	t	t	a	c	t	c	t	a	t	c	g	c	g	g	g	c	c	a	a	c	t	g	a	a	g	c	t	c	c	g	t	a
KF853233	g	g	a	c	c	t	t	t	a	c	t	c	t	a	t	c	g	c	g	g	g	c	c	a	a	c	t	g	a	a	g	c	c	c	c	a	t	a
KF853234	g	g	a	c	c	t	t	t	a	c	t	c	t	a	t	c	g	c	g	g	g	c	c	a	a	c	t	g	a	a	g	c	t	c	c	g	t	a
KF853235	g	g	a	c	c	t	t	t	a	c	t	c	t	a	t	c	g	c	g	g	g	c	c	a	a	c	t	g	a	a	g	c	t	c	c	g	t	a
MF898328	g	g	a	c	c	t	t	t	a	c	t	c	t	a	t	c	g	c	g	g	g	c	c	a	a	c	t	g	a	a	g	c	t	c	c	g	t	a
JN704709	g	g	a	c	c	t	t	t	a	a	t	c	g	a	t	c	g	c	g	g	g	t	c	a	a	c	t	g	a	a	g	c	t	c	c	a	t	a

Supplementary Table 3: 2880 SN

GB-ID	Clade markers																																																										2	2														
	Position in Dumas	58791	58831	58914	58950	59094	59199	59287	59293	59377	59386	59395	59591	59713	59752	59781	59830	59954	59981	59987	60003	60098	60158	60224	60266	60268	60269	60270	60277	60279_1	60279_2	60280	60292	60374	60405	60447	60458	60469	60479	60537	60552	60556	60558	60561	60599	60614	60630	60659	60681	60701	60777	60781	60821	60839	60840	60845	60990	61008	61018	61019	61029	61052	61180	61201	61202	61239	61267	61281	61338	61365	61629	61644	61656	61667
AY548170	1	a	c	a	t	a	t	a	a	t	a	t	a	a	t	a	a	a	g	t	g	t	a	c	c	-	-	a	a	-	-	c	t	t	c	t	t	g	g	c	a	t	c	c	t	g	a	c	t	t	a	t	c	g	g	c	c	t	g	g	a	g	g	t	t	g	t	t	g	c	c	t	a	t
AY548171	1	a	c	a	t	a	t	a	a	t	a	t	a	a	t	a	a	a	g	t	g	t	a	c	c	-	-	a	a	-	-	c	t	t	c	t	t	g	g	c	a	t	c	c	t	g	a	c	t	t	a	t	c	g	g	c	c	c	g	g	a	g	g	t	t	g	t	t	g	c	c	t	a	t
DQ479953	1	a	c	a	t	a	t	a	a	t	a	t	a	a	t	a	a	a	g	t	g	t	a	c	c	-	a	a	a	-	-	c	t	t	c	t	t	g	g	c	a	t	c	c	t	g	a	c	t	t	a	t	c	g	g	c	c	c	g	g	a	g	g	t	t	g	t	t	g	c	c	t	a	t
DQ479954	1	a	c	a	t	a	t	a	a	t	a	t	a	a	t	a	a	a	g	t	g	t	a	c	c	a	a	a	a	-	-	c	t	t	c	t	t	g	g	c	a	t	c	c	t	g	a	c	t	t	a	t	c	g	g	c	c	c	g	g	a	g	g	t	t	g	t	t	g	c	c	t	a	t
DQ479958	1	a	c	a	t	a	t	a	a	t	a	t	a	a	t	a	a	a	g	t	g	t	a	c	c	-	-	a	a	-	-	c	t	t	c	t	t	g	g	c	a	t	c	c	t	g	a	c	t	t	a	t	c	g	g	c	c	c	g	g	a	g	g	t	t	g	t	t	g	c	c	t	a	t
DQ479959	1	a	c	a	t	a	t	a	a	t	a	t	a	a	t	a	a	a	g	t	g	t	a	c	c	-	-	a	a	-	-	c	t	t	c	t	t	g	g	c	a	t	c	c	t	g	a	c	t	t	a	t	c	g	g	c	c	c	g	g	a	g	g	t	t	g	t	t	g	c	c	t	a	t
DQ479961	1	a	c	a	t	a	t	a	a	t	a	t	a	a	t	a	a	a	g	t	g	t	a	c	c	a	a	a	a	-	-	c	t	t	c	t	t	g	g	c	a	t	c	c	t	g	a	c	t	t	a	t	c	g	g	c	c	c	g	g	a	g	g	t	t	g	t	t	g	c	c	t	a	t
DQ479962	1	a	c	a	t	a	t	a	a	t	a	t	a	a	t	a	a	a	g	t	g	t	a	c	c	a	a	a	a	-	-	c	t	t	c	t	t	g	g	c	a	t	c	c	t	g	a	c	t	t	a	t	c	g	g	c	c	c	g	g	a	g	g	t	t	g	t	t	g	c	c	t	a	t
DQ479963	1	a	c	a	t	a	t	a	a	t	a	t	a	a	t	a	a	a	g	t	g	t	a	c	c	a	a	a	a	-	-	c	t	t	c	t	t	g	g	c	a	t	c	c	t	g	a	c	t	t	a	t	c	g	g	c	c	c	g	g	a	g	g	t	t	g	c	t	g	c	c	t	a	t
DQ674250	1	a	c	a	t	a	t	a	a	t	a	t	a	a	t	a	a	a	g	t	g	t	a	c	c	-	-	a	a	-	-	c	t	t	c	t	t	g	g	c	a	t	c	c	t	g	a	c	t	t	a	t	c	g	g	c	c	c	g	g	a	g	g	t	t	g	t	t	g	c	c	t	a	t
KX262864	1	a	c	a	t	a	t	a	a	t	a	t	a	a	t	a	a	a	g	t	g	t	a	c	c	a	a	a	a	-	-	c	t	t	c	t	t	g	g	c	a	t	c	c	t	g	a	c	t	t	a	t	c	g	g	c	c	c	g	g	a	g	g	t	t	g	t	t	g	c	c	t	a	t
KX262865	1	a	c	a	t	a	t	a	a	t	a	t	a	a	t	a	a	a	g	t	g	t	a	c	c	a	a	a	a	-	-	c	t	t	c	t	t	g	g	c	a	t	c	c	t	g	a	c	t	t	a	t	c	g	g	c	c	c	g	g	a	g	g	t	t	g	t	t	g	c	c	t	a	t
KX262866	1	a	c	a	t	a	t	a	a	t	a	t	a	a	t	a	a	a	g	t	g	t	a	c	c	a	a	a	a	-	-	c	t	t	c	t	t	g	g	c	a	t	c	c	t	g	a	c	t	t	a	t	c	g	g	c	c	c	g	g	a	g	g	t	t	g	t	t	g	c	c	t	a	t
EU154348	1	a	c	a	t	a	t	a	a	t	a	t	a	a	t	a	a	a	a	t	g	t	a	c	c	-	a	a	a	-	-	c	t	t	c	t	t	g	g	c	a	t	c	c	t	g	a	c	t	t	a	t	c	g	g	c	c	c	g	g	a	g	g	t	t	g	t	t	g	c	c	t	a	t
JN704690	1	a	c	a	t	a	t	a	a	t	a	t	a	a	t	a	a	a	g	t	g	t	a	c	c	a	a	a	a	-	-	c	t	t	c	t	t	g	g	c	a	t	c	c	t	g	a	c	t	t	a	t	c	g	g	c	c	c	g	g	a	g	g	t	t	g	t	t	g	c	c	t	a	t
JN704691	1	a	c	a	t	a	t	a	a	t	a	t	a	a	t	a	a	a	g	t	g	t	a	c	c	a	a	a	a	-	-	c	t	t	c	t	t	g	g	c	a	t	c	c	t	g	a	c	t	t	a	t	c	g	g	c	c	c	g	g	a	g	g	t	t	g	t	t	g	c	c	t	a	t
JN704692	1	a	c	a	t	a	t	a	a	t	a	t	a	a	t	a	a	a	g	t	g	t	a	c	c	a	a	a	a	-	-	c	t	t	c	t	t	g	g	c	a	t	c	c	t	g	a	c	t	t	a	t	c	g	g	c	c	c	g	g	a	g	g	t	t	g	t	t	g	c	c	t	a	t
JN704693	1	a	c	a	t	a	t	a	a	t	a	t	a	a	t	a	a	a	g	t	g	t	a	c	c	a	a	a	a	-	-	c	t	t	c	t	t	g	g	c	a	t	c	c	t	g	a	c	t	t	a	t	c	g	g	c	c	c	g	g	a	g	g	t	t	g	t	t	g	c	c	t	a	t
JN704694	1	a	c	a	t	a	t	a	a	t	a	t	a	a	t	a	a	a	g	t	g	t	a	c	c	a	a	a	a	-	-	c	t	t	c	t	t	g	g	c	a	t	c	c	t	g	a	c	t	t	a	t	c	g	g	c	c	c	g	g	a	g	g	t	t	g	t	t	g	c	c	t	a	t
JN704695	1	a	c	a	t	a	t	a	a	t	a	t	a	a	t	a	a	a	g	t	g	t	a	c	c	a	a	a	a	-	-	c	t	t	c	t	t	g	g	c	a	t	c	c	t	g	a	c	t	t	a	t	c	g	g	c	c	c	g	g	a	g	g	t	t	g	t	t	g	c	c	t	a	t
JN704696	1	a	c	a	t	a	t	a	a	t	a	t	a	a	t	a	a	a	g	t	g	t	a	c	c	a	a	a	a	-	-	c	t	t	c	t	t	g	g	c	a	t	c	c	t	g	a	c	t	t	a	t	c	a	a	c	c	c	g	g	a	g	g	t	t	g	t	t	g	c	c	t	a	t
NC001348	1	a	c	a	t	a	t	a	a	t	a	t	a	a	t	a	a	a	g	t	g	t	a	c	c	a	a	a	a	-	-	c	t	t	c	t	t	g	g	c	a	t	c	c	t	g	a	c	t	t	a	t	c	g	g	c	c	c	g	g	a	g	g	t	t	g	t	t	g	c	c	t	a	t
KP771892	1	a	c	a	t	a	t	a	a	t	a	t	a	a	t	a	a	a	g	t	g	t	a	c	c	a	a	a	a	-	-	c	t	t	c	t	t	g	g	c	a	t	c	c	t	g	a	c	t	t	a	t	c	g	g	c	c	c	g	g	a	g	g	t	t	g	t	t	g	c	c	t	a	t
KP771894	1	a	c	a	t	a	t	a	a	t	a	t	a	a	t	a	a	a	g	t	g	t	a	c	c	-	-	-	a	-	-	c	t	t	c	t	t	g	g	c	a	t	c	c	t	g	a	c	t	t	a	t	c	g	g	c	c	c	g	g	a	g	g	t	t	g	t	t	g	c	c	t	a	t
KP771900	1	a	c	a	t	a	t	a	a	t	a	t	a	a	t	a	a	a	g	t	g	t	a	c	c	a	a	a	a	-	-	c	t	t	c	t	t	g	g	c	a	t	c	c	t	g	a	c	t	t	a	t	c	g	g	c	c	c	g	g	a	g	g	t	t	g	t	t	g	c	c	t	a	t
KP771901	1	a	c	a	t	a	t	a	a	t	a	t	a	a	t	a	a	a	g	t	g	t	a	c	c	a	a	a	a	-	-	c	t	t	c	t	t	g	g	c	a	t	c	c	t	g	a	c	t	t	a	t	c	g	g	c	c	c	g	g	a	g	g	t	t	g	t	t	g	c	c	t	a	t
KP771902	1	a	c	a	t	a	t	a	a	t	a	t	a	a	t	a	a	a	g	t	g	t	a	c	c	a	a	a	a	-	-	c	t	t	c	t	t	g	g	c	a	t	c	c	t	g	a	c	t	t	a	t	c	g	g	c	c	c	g	g	a	g	g	t	t	g	t	t	g	c	c	t	a	t
KP771908	1	a	c	a	t	a	t	a	a	t	a	t	a	a	t	a	a	a	g	t	g	t	a	c	c	a	a	a	a	-	-	c	t	t	c	t	t	g	g	c	a	t	c	c	t	g	a	c	t	t	a	t	c	g	g	c	c	c	g	g	a	g	g	t	t	g	t	t	g	c	c	t	a	t
KP771912	1	a	c	a	t	a	t	a	a	t	a	t	a	a	t	a	a	a	g	t	g	t	a	c	c	a	a	a	a	-	-	c	t	t	c	t	t	g	g	c	a	t	c	c	t	g	a	c	t	t	a	t	c	g	g	c	c	c	g	g	a	g	g	t	t	g	t	t	g	c	c	t	a	t
KP771913	1	a	c	a	t	a	t	a	a	t	a	t	a	a	t	a	a	a	g	t	g	t	a	c	c	a	a	a	a	-	-	c	t	t	c	t	t	g	g	c	a	t	c	c	t	g	a	c	t	t	a	t	c	g	g	c	c	c	g	g	a	g	g	t	t	g	t	t	g	c	c	t	a	t
KP771917	1	a	c	a	t	a	t	a	a	t	a	t	a	a	t	a	a	a	g	t	g	t	a	c	c	a	a	a	a	-	-	c	t	t	c	t	t	g	g	c	a	t	c	c	t	g	a	c	t	t	a	t	c	g	g	c	c	c	g	g	a	g	g	t	t	g	t	t	g	c	c	t	a	t
KP771918	1	a	c	a	t	a	t	a	a	t	a	t	a	a	t	a	a	a	g	t	g	t	a	c	c	a	a	a	a	-	-	c	t	t	c	t	t	g	g	c	a	t	c	c	t	g	a	c	t	t	a	t	c	g	g	c	c	c	g	g	a	g	g	t	t	g	t	t	g	c	c	t	a	t
KP771920	1	a	c	a	t	a	t	a	a	t	a	t	a	a	t	a	a	a	g	t	g	t	a	c	c	a	a	a	a	-	-	c	t	t	c	t	t	g	g	c	a	t	c	c	t	g	a	c	g	t	a	t	c	g	g	c	c	c	g	g	a	g	g	t	t	g	t	t	g	c	c	t	a	t
MH709361	1	a	c	a	t	a	t	a	a	t	a	t	a	a	t	a	a	a	g	t	g	t	a	c	c	a	a	a	a	-	-	c	t	t	c	t	t	g	g	c	a	t	c	c	t	g	a	c	t	t	a	t	c	g	g	c	c	c	g	g	a	g	g	t	t	g	t	t	g	c	c	t	a	t
KU926318	2	g	c	g	t	a	t	a	a	t	a	t	a	a	t	a	a	a	g	t	g	t	a	c	c	a	a	a	a	-	c	c	t	c	a	t	t	g	g	c	a	t	c	c	t	g	a	c	t	t	a	g	c	g	g	c	c	c	a	a	a	g	g	c	c	g	t	t	g	c	c	t	a	t
KU926319	2	a	c	a	t	a	t	a	a	t	a	t	a	a	t	a	a	a	g	t	g	t	a	c	c	-	a	a	a	-	c	c	t	t	a	t	t	g	g	c	a	t	c	c	t	g	a	c	t	t	a	g	c	g	g	c	c	c	a	a	a	g	g	c	c	g	t	t	g	c	c	t	a	t
KU926320	2	a	c	a	t	a	t	a	a	t	a	t	a	a	t	a	g	a	g	t	g	t	a	c	c	-	a	a	a	-	c	c	t	t	a	t	t	g	g	c	a	t	c	c	t	g	g	c	t	t	a	g	c	g	g	c	c	c	a	a	a	g	g	c	c	g	t	t	g	c	c	t	a	t
KU926321	2	a	c	a	t	a	t	a	a	t	a	t	a	a	t	a	a	a	g	t	g	t	a	c	c	a	a	a	a	-	c	c	t	t	a	t	t	g	g	c	a	t	c	c	t	g	a	c	t	t	a	g	c	g	g	c	c	c	a	a	a	g	g	c	c	g	t	t	g	c	c	t	a	t
KU926322	2	a	c	a	t	a	t	a	a	t	a	t	a	a	t	a	a	a	g	t	g	t	a	c	c	a	a	a	a	-	c	c	t	t	a	t	t	g	g	c	a	t	c	c	t	g	a	c	t	t	a	g	c	g	g	c	c	c	a	a	a	g	g	c	c	g	t	t	g	c	c	t	a	t
KC847290	2	a	c	a	t	a	t	a	a	t	a	t	a	a	t	a	a	a	g	t	g	t	a	c	c	a	a	a	a	-	c	c	t	t	a	t	t	g	g	c	a	t	c	c	t	g	a	c	t	t	a	g	c	g	g	c	c	c	a	a	a	g	g	c	c	g	t	t	g	c	c	t	a	t
KJ767491	2	a	c	a	t	a	t	a	a	t	a	t	a	a	t	a	a	a	g	t	g	t	a	c	c	a	a	a	a	-	c	c	t	t	a	t	t	g	g	c	a	t	c	c	t	g	a	c	t	t	a	g	c	g	g	c	c	c	a	a	a	g	g	c	c	g	t	t	g	c	c	t	a	t
KJ767492	2	a	c	a	t	a	t	a	a	t	a	t	a	a	t	a	a	a	g	t	g	t	a	c	c	-	a	a	a	-	c	c	t	t	a	t	t	g	g	c	a	t	c	c	t	g	a	c	t	t	a	g	c	g	g	c	c	c	a	a	a	g	g	c	c	g	t	t	g	c	c	t	a	t
KJ808816	2	a	c	a	t	a	t	a	a	t	a	t	a	a	t	a	a	a	g	t	g	t	a	c	c	a	a	a	a	-	c	c	t	t	a	t	t	g	g	c	a	t	c	c	t	g	a	c	t	t	a	g	c	g	g	c	c	c	a	a	a	g	g	c	c	g	t	t	g	c	c	t	a	t
KP771925	2	a	c	a	t	a	t	a	a	t	a	t	a	a	t	a	a	a	g	t	g	t	a	c	c	a	a	a	-	-	a	c	t	t	a	t	t	a	g	c	a	t	c	c	t	g	a	c	t	t	a	g	c	g	g	c	c	c	a	a	a	g	g	c	c	g	t	t	g	c	c	t	a	t
KU926315	2	a	c	a	t	a	t	a	a	t	a	t	a	a	t	a	a	a	g	t	g	t	a	c	c	a	a	a	a	-	c	c	t	t	a	t	t	g	g	c	a	t	c	c	t	g	a	c	t	t	a	g	c	g	g	c	c	c	a	a	a	g	g	c	c	g	t	t	g	c	c	t	a	t
KU926316	2	a	c	a	t	a	t	a	a	t	a	t	a	a	t	a	a	a	g	t	g	t	a	c	c	a	a	a	a	-	c	c	t	t	a	t	t	g	g	c	a	t	c	c	t	g	a	c	t	t	a	g	c	g	g	c	c	c	a	a	a	g	g	c	c	g	t	t	g	c	c	t	a	t
KU926317	2	a	c	a	t	a	t	a	a	t	a	t	a	a	t	a	a	a	g	t	g	t	a	c	c	a	a	a	a	-	c	c	t	t	a	t	t	g	g	c	a	t	c	c	t	g	a	c	t	t	a	g	c	g	g	c	c	c	a	a	a	g	g	c	c	g	t	t	g	c	c	t	a	t
KY037796	2	a	c	a	t	a	t	a	a	t	a	t	a	a	t	a	a	a	g	t	g	t	a	c	c	-	-	a	a	-	c	c	t	t	a	t	t	g	g	c	a	t	c	c	t	g	a	c	t	t	a	g	c	g	g	c	c	c	a	a	a	g	g	c	c	g	t	t	g	c	c	t	a	t
MF004348	2	a	c	a	t	a	t	a	a	t	a	t	a	a	t	a	a	a	g	t	g	t	a	c	c	-	a	a	a	-	c	c	t	t	a	t	t	g	g	c	a	t	c	c	t	g	a	c	t	t	a	g	c	g	g	c	c	c	a	a	a	g	g	c	c	g	t	t	g	c	c	t	a	t
MH709309	2	a	c	a	t	a	t	a	a	t	a	t	a	a	t	a	a	a	g	t	g	t	a	c	c	a	a	a	a	-	c	c	t	t	a	t	t	g	g	c	a	t	c	c	t	g	a	c	t	t	a	g	c	g	g	c	c	c	a	a	a	g	g	c	c	g	t	t	g	c	c	t	a	t
MH709310	2	a	c	a	t	a	t	a	a	t	a	t	a	a	t	a	a	a	g	t	g	t	a	c	c	a	a	a	a	-	c	c	t	t	a	t	t	g	g	c	a	t	c	c	t	g	a	c	t	t	a	g	c	g	g	c	c	c	a	a	a	g	g	c	c	g	t	t	g	c	c	t	a	t
MH709311	2	a	c	a	t	a	t	a	a	t	a	t	a	a	t	a	a	a	g	t	g	t	a	c	c	-	-	a	a	-	c	c	t	t	a	t	t	g	g	c	a	t	c	c	t	g	a	c	t	t	a	g	c	g	g	c	c	c	a	a	a	g	g	c	c	g	t	t	a	c	c	t	a	t
MH709313	2	a	c	a	t	a	t	a	a	t	a	t	a	a	t	a	a	a	g	t	g	t	a	c	c	a	a	a	a	-	c	c	t	t	a	t	t	g	g	c	a	t	c	c	t	g	a	c	t	t	a	g	c	g	g	c	c	c	a	a	a	g	g	c	c	g	t	t	g	c	c	t	a	t
MH709314	2	a	c	a	t	a	t	a	a	t	a	t	a	a	t	a	a	a	g	t	g	t	a	c	c	a	a	a	a	-	c	c	t	t	a	t	t	g	g	c	a	t	c	t	t	g	a	c	t	t	a	g	c	g	g	c	c	c	a	a	a	g	g	c	c	g	t	t	g	c	c	t	a	t
MH709315	2	a	c	a	t	a	t	a	a	t	a	t	a	a	t	a	a	a	g	t	g	t	a	c	c	a	a	a	g	-	c	c	t	t	a	t	t	g	g	c	a	t	c	c	t	g	a	c	t	t	a	g	c	g	g	c	c	c	a	a	a	g	g	c	c	g	t	t	g	c	c	t	a	t
MH709319	2	a	c	a	t	a	t	a	a	t	a	t	a	a	t	a	a	a	g	t	g	t	a	c	a	a	a	a	a	-	c	c	t	t	a	t	t	g	g	c	a	t	c	c	t	g	a	c	t	t	a	g	c	g	g	c	c	c	a	a	a	g	g	c	c	g	t	t	g	c	c	t	a	t
MH709320	2	a	c	a	t	a	t	a	a	t	a	t	a	a	t	a	a	a	g	t	g	t	a	c	c	a	a	a	a	-	c	c	t	t	a	t	t	g	g	c	a	t	c	c	t	g	a	c	t	t	a	g	c	g	g	c	c	c	a	a	a	g	g	c	c	g	t	t	g	c	c	t	a	t
MH709329	2	a	c	a	t	a	t	a	a	t	a	t	a	a	t	a	a	a	g	t	g	t	a	c	c	a	a	a	a	-	c	c	t	t	a	t	t	g	g	c	a	t	c	c	t	g	a	c	t	t	a	g	c	g	g	c	c	c	a	a	a	g	g	c	c	g	t	t	g	c	c	t	a	t
MH709331	2	a	c	a	t	a	t	a	a	t	a	t	a	a	t	a	a	a	g	t	g	t	a	c	c	a	a	a	a	c	c	c	t	t	a	t	t	g	g	c	a	t	c	c	t	g	a	c	t	t	a	g	c	g	g	c	c	c	a	a	a	g	g	c	c	g	t	t	g	c	c	t	a	t
MH709333	2	a	c	a	t	a	t	a	a	t	a	t	a	a	t	a	a	a	g	t	g	t	a	c	c	a	a	a	a	-	c	c	t	t	a	t	t	g	g	c	a	t	c	c	t	g	a	c	t	t	a	g	c	g	g	c	c	c	a	a	a	g	g	c	c	g	t	t	g	c	c	t	a	t
MH709337	2	a	c	a	t	a	t	a	a	t	a	t	a	a	t	a	a	a	g	t	g	t	a	c	c	a	a	a	a	-	c	c	t	t	a	t	t	g	g	c	a	t	c	c	t	g	a	c	t	t	a	g	c	g	g	c	c	c	a	a	a	g	g	c	c	g	t	t	g	c	c	t	a	t
MH709339	2	a	c	a	t	a	t	a	a	t	a	t	a	g	t	a	a	a	g	t	g	t	a	c	c	a	a	a	-	-	-	c	t	t	a	t	t	g	g	c	a	t	c	c	t	g	g	c	t	t	a	g	c	g	g	c	c	c	a	a	a	g	g	c	c	g	t	t	g	c	c	t	a	t
MH709344	2	a	c	a	t	a	t	a	a	t	a	t	a	a	t	a	a	a	g	t	g	t	a	c	-	-	c	a	a	-	c	c	t	t	a	t	t	g	g	c	a	t	c	c	t	g	a	c	t	t	a	g	c	g	g	c	c	c	a	a	a	g	g	c	c	g	t	t	g	c	c	t	a	t
MH709348	2	a	c	a	t	a	t	a	a	t	a	t	a	a	t	a	a	a	g	t	g	t	a	c	c	a	a	a	a	-	c	c	t	t	a	t	t	g	g	c	a	t	c	c	t	g	a	c	t	t	a	g	c	g	g	c	c	c	a	a	a	g	g	c	c	g	t	t	g	c	c	t	a	t
MH709355	2	a	c	a	t	a	t	a	a	t	a	t	a	a	t	a	a	a	g	t	g	t	a	c	c	a	a	a	a	-	c	c	t	t	a	t	t	g	g	c	a	t	c	c	t	g	a	c	t	t	a	g	c	g	g	c	c	c	a	a	a	g	g	c	c	g	t	t	g	c	c	t	a	t
MH709359	2	a	c	a	t	a	t	a	a	t	a	t	a	a	t	a	a	a	g	t	g	t	a	c	c	a	a	a	a	-	c	c	t	t	a	t	g	g	g	c	a	t	c	c	t	g	a	c	t	t	a	g	c	g	g	c	c	c	a	a	a	g	g	c	c	g	t	t	g	c	c	t	a	t
MH709363	2	a	c	a	t	a	t	a	a	t	a	t	a	a	t	a	a	a	g	t	g	t	a	c	c	-	a	a	a	-	c	c	t	t	a	t	t	g	g	c	a	t	c	c	t	g	a	c	t	t	a	g	c	g	g	c	c	c	a	a	a	g	g	c	c	g	t	t	g	c	c	t	a	t
MH709366	2	a	c	a	t	a	t	a	a	t	a	t	a	a	t	a	a	a	g	t	g	t	a	c	c	-	a	a	a	-	c	c	t	t	a	t	t	g	g	c	a	t	c	c	t	g	a	c	t	t	a	g	c	g	g	c	c	c	a	a	a	g	g	c	c	g	t	t	g	c	c	t	a	t
MH709376	2	a	c	a	t	a	t	a	a	t	a	t	a	a	t	a	a	a	g	t	g	t	a	c	c	a	a	a	a	-	c	c	t	t	a	t	t	g	g	c	a	t	c	c	t	g	a	c	t	t	a	g	c	g	g	c	c	c	a	a	a	g	g	c	c	g	t	t	g	c	c	t	a	t
MH709377	2	a	c	a	t	a	t	a	a	t	a	t	a	a	t	a	a	a	g	t	g	t	a	c	c	a	a	a	a	-	c	c	t	t	a	t	t	g	g	c	a	t	c	c	t	g	a	c	t	t	a	g	c	g	g	c	c	c	a	a	a	g	g	c	c	g	t	t	g	c	c	t	a	t
**MW316406**	2	a	c	a	t	a	t	a	a	t	a	t	a	a	t	a	a	a	g	t	g	t	a	c	c	-	a	a	a	-	c	c	t	t	a	t	t	g	g	c	a	t	c	c	t	g	a	c	t	t	a	g	c	g	g	c	c	c	a	a	a	g	g	c	c	g	t	t	g	c	c	t	a	t
AJ871403	3	a	c	a	t	a	t	a	a	t	a	t	a	a	t	a	a	a	g	t	g	t	a	c	c	-	-	a	a	-	-	-	t	t	c	t	t	g	g	c	a	t	c	c	t	a	a	c	t	t	a	t	c	g	g	c	c	c	g	g	a	g	g	t	t	g	t	t	g	c	c	t	a	t
DQ479955	3	a	c	a	t	a	t	a	a	t	a	t	a	a	t	c	a	a	g	t	a	t	a	c	c	-	-	a	a	-	-	c	t	t	c	t	t	g	g	c	a	t	c	c	t	g	a	c	t	t	a	t	c	g	g	c	c	c	g	g	a	g	g	t	t	g	t	t	g	c	c	t	a	t
DQ479956	3	a	c	a	t	a	t	a	a	t	a	t	a	a	t	a	a	a	g	t	g	t	a	c	c	a	a	a	a	-	-	c	t	t	c	t	t	g	g	c	a	t	c	c	t	g	a	c	t	t	a	t	c	g	g	c	c	c	g	g	a	g	g	t	t	g	t	t	g	c	c	t	a	t
DQ479957	3	a	c	a	t	a	t	a	a	t	a	t	a	a	t	a	a	a	g	t	g	t	a	c	c	a	a	a	a	-	-	c	t	t	c	t	t	g	g	a	a	t	c	c	t	g	a	c	t	t	a	t	c	g	g	c	c	c	g	g	a	g	g	t	t	g	t	t	g	c	c	t	a	t
JN704699	3	a	c	a	t	a	t	a	a	t	c	t	a	a	t	a	a	a	g	t	g	t	a	c	c	a	a	a	a	-	-	c	t	t	c	t	t	g	g	c	a	t	c	c	t	g	a	c	t	t	a	t	c	g	g	c	c	c	g	g	a	g	g	t	t	g	t	t	g	c	c	t	a	t
JN704700	3	a	c	a	t	a	t	a	a	t	a	c	a	a	t	a	a	a	g	t	g	t	a	c	c	a	a	a	a	-	-	c	t	t	c	t	t	g	g	c	a	t	c	c	t	g	a	c	t	t	a	t	c	g	g	c	c	c	g	g	a	g	g	t	t	g	t	t	g	c	c	t	a	t
JN704701	3	a	c	a	t	a	t	a	a	t	c	t	a	a	t	a	a	a	g	t	g	t	a	c	c	-	-	-	a	-	-	c	t	t	c	t	t	g	g	c	a	t	c	c	t	g	a	c	t	t	a	t	c	g	g	c	c	c	g	g	a	g	g	t	t	g	t	t	g	c	c	t	a	t
JN704702	3	a	c	a	t	a	t	a	a	t	a	t	a	a	t	a	a	a	g	t	g	t	a	c	c	a	a	a	a	-	-	c	t	t	c	t	t	g	g	c	a	t	c	c	t	g	a	c	t	t	a	t	c	g	g	c	c	c	g	g	a	g	g	t	t	g	t	t	g	c	c	t	a	t
JN704703	3	a	c	a	t	a	t	a	a	t	a	t	a	a	t	a	a	a	g	t	g	t	a	c	c	a	a	a	a	-	-	c	t	t	c	t	t	g	g	c	a	t	c	c	t	g	a	c	t	t	a	t	c	g	g	c	c	c	g	g	a	g	g	t	t	g	t	t	g	c	c	t	a	t
KP771891	3	a	c	a	t	a	t	a	a	t	c	t	a	a	t	a	a	a	g	t	g	t	a	c	c	a	a	a	a	-	-	c	t	t	c	t	t	g	g	c	a	t	c	c	t	g	a	c	t	t	a	t	c	g	g	c	c	c	g	g	a	g	g	t	t	g	t	t	g	c	c	t	a	t
KP771893	3	a	c	a	t	a	t	a	a	t	c	t	a	a	t	a	a	a	g	t	g	t	a	c	c	a	a	a	a	-	-	c	t	t	c	t	t	g	g	c	a	t	c	c	t	g	a	c	t	t	a	t	c	g	g	c	c	c	g	g	a	g	g	t	t	g	t	t	g	c	c	t	a	t
KP771895	3	a	c	a	t	a	t	a	a	t	a	t	a	a	t	a	a	a	g	t	g	t	a	c	c	a	a	a	a	-	-	c	t	t	c	t	t	g	g	c	a	t	c	c	t	g	a	c	t	t	a	t	c	g	g	c	c	c	g	g	a	g	g	t	t	g	t	t	g	c	c	t	a	t
KP771896	3	a	c	a	t	a	t	a	a	t	a	t	a	a	t	a	a	a	g	t	g	t	a	c	c	a	a	a	a	-	-	c	t	t	c	t	t	g	g	c	a	t	c	c	t	g	a	c	t	t	a	t	c	g	g	c	c	c	g	g	a	g	g	t	t	g	t	t	g	t	c	t	a	t
KP771897	3	a	c	a	t	a	t	a	a	t	a	t	a	a	t	a	a	a	g	t	g	t	a	c	c	a	a	a	a	-	-	a	t	t	c	t	t	g	g	c	a	t	c	c	t	g	a	c	t	t	a	t	c	g	g	c	c	c	g	g	a	g	g	t	t	g	t	t	g	c	c	t	a	t
KP771899	3	a	c	a	t	a	t	a	a	t	a	t	a	a	t	a	a	a	g	t	g	t	a	c	c	a	a	a	a	-	-	c	t	t	c	t	t	g	g	c	a	t	c	c	t	g	a	c	t	t	a	t	c	g	g	c	c	c	g	g	a	g	g	t	t	g	t	t	g	c	c	t	a	t
KP771903	3	a	c	a	t	a	t	a	a	t	c	t	a	a	t	a	a	a	g	t	g	t	a	c	c	a	a	a	a	-	-	c	t	t	c	t	t	g	g	c	a	t	c	c	t	g	a	c	t	t	a	t	c	g	g	c	c	c	g	g	a	g	g	t	t	g	t	t	g	c	c	t	a	t
KP771907	3	a	c	a	t	a	t	a	a	t	a	t	a	a	t	a	a	a	g	t	g	t	a	t	c	a	a	a	a	-	-	c	t	t	c	t	t	g	g	c	a	t	c	c	t	g	a	c	t	t	a	t	c	g	g	c	c	c	g	g	a	g	g	t	t	g	t	t	g	c	c	t	a	t
KP771910	3	a	c	a	t	a	t	a	a	t	c	t	a	a	t	a	a	a	g	t	g	t	a	c	c	a	a	a	a	-	-	c	t	t	c	t	t	g	g	c	a	t	c	c	t	g	a	c	t	t	a	t	c	g	g	c	c	c	g	g	a	g	g	t	t	g	t	t	g	c	c	t	a	t
KP771915	3	a	c	a	t	a	t	a	a	t	a	t	a	a	t	a	a	a	g	t	g	t	a	c	c	a	a	a	a	-	-	c	t	t	c	t	t	g	g	c	a	t	c	c	t	g	a	c	t	t	a	t	c	g	g	c	c	c	g	g	a	g	g	t	t	g	t	t	g	c	c	t	a	t
KP771916	3	a	c	a	t	a	t	a	a	t	a	t	a	a	t	a	a	a	g	t	g	t	a	c	c	a	a	a	a	-	-	c	t	t	c	t	t	g	g	c	a	t	c	c	t	g	a	c	t	t	a	t	c	g	g	c	c	c	g	g	a	g	g	t	t	g	t	t	g	c	c	t	a	t
KP771921	3	a	c	a	t	a	t	a	a	t	c	t	a	a	t	a	a	a	g	t	g	t	a	c	c	a	a	a	a	-	-	c	t	t	c	t	t	g	g	c	a	t	c	c	t	g	a	c	t	t	a	t	c	g	g	c	c	c	g	g	a	a	g	t	t	g	t	t	g	c	c	t	a	t
KP771923	3	a	c	a	t	a	t	a	a	t	c	t	a	a	t	a	a	a	g	t	g	t	a	c	c	a	a	a	a	-	-	c	t	t	c	t	t	g	g	c	a	t	c	c	t	g	a	c	t	t	a	t	c	g	g	c	c	c	g	g	a	g	g	t	t	g	t	t	g	c	c	t	a	t
KP771889	3	a	c	a	t	a	t	a	a	t	a	t	a	a	t	a	a	a	g	t	g	t	a	c	c	a	a	a	a	-	-	c	t	t	c	t	t	g	g	c	a	t	c	c	t	g	a	c	t	t	a	t	c	g	g	c	c	c	g	g	a	g	g	t	t	g	t	t	g	c	c	t	a	t
KU926311	3	a	c	a	t	a	t	a	a	t	a	t	a	a	t	a	a	a	g	t	g	t	a	c	c	a	a	a	a	-	-	c	t	t	c	t	t	g	g	c	a	t	c	c	t	g	a	c	t	t	a	t	c	g	g	c	a	c	g	g	a	g	g	t	t	g	t	t	g	c	c	t	a	t
JQ911599	3	a	c	a	c	a	t	a	a	t	a	t	a	a	t	a	a	a	g	t	g	t	a	c	c	-	-	a	a	-	-	c	t	t	c	t	t	g	g	c	a	t	c	c	t	g	a	g	t	t	a	t	c	g	g	c	c	c	g	g	a	g	g	t	t	g	t	t	g	c	c	t	a	t
MH709317	3	a	c	a	t	a	t	a	a	t	a	t	a	a	t	a	a	a	g	c	g	t	a	c	c	a	a	a	a	-	-	c	t	t	c	t	t	g	g	c	a	t	c	c	t	g	a	c	t	t	a	t	c	g	g	c	c	c	g	g	a	g	g	t	t	g	t	t	g	c	c	t	a	t
MH709346	3	a	c	a	t	a	t	a	a	t	a	t	a	a	t	a	a	a	g	c	g	t	a	c	c	a	a	a	a	-	-	c	t	t	c	t	t	g	g	c	a	t	c	c	t	g	a	c	t	t	a	t	c	g	g	c	c	c	g	g	a	g	g	t	t	g	t	t	g	c	c	t	a	t
MH709375	3	a	c	a	t	a	t	a	a	t	a	t	a	a	t	a	a	a	g	c	g	t	a	c	c	a	a	a	a	-	-	c	t	t	c	t	t	g	g	c	a	t	c	c	t	g	a	c	t	t	a	t	c	g	g	c	c	c	g	g	a	g	g	t	t	g	t	g	g	c	c	t	a	t
DQ452050	4	a	c	a	t	a	t	a	a	t	a	t	a	a	t	a	a	a	g	t	g	c	a	c	c	-	-	-	a	-	-	a	t	t	a	t	t	g	g	c	a	t	c	c	t	g	a	c	t	t	a	g	c	g	g	c	c	c	g	g	a	g	g	c	c	g	t	t	g	c	c	t	a	t
DQ479960	4	a	c	a	t	a	t	a	a	t	a	t	a	a	t	a	a	a	g	t	g	t	a	c	c	-	-	-	a	-	-	a	t	t	a	t	t	g	g	c	a	t	c	c	t	g	a	c	t	t	a	g	c	g	g	c	c	c	g	g	a	g	g	c	c	g	t	t	g	c	c	t	a	t
KP771898	4	a	c	a	t	a	t	a	a	t	a	t	a	a	t	a	a	a	g	t	g	t	a	c	c	-	-	a	a	-	-	c	t	t	a	t	t	g	g	c	a	t	c	c	t	g	a	c	t	t	a	g	c	g	g	c	c	c	g	g	a	g	g	c	c	g	t	t	g	c	c	t	a	t
MH709316	4	a	c	a	t	a	t	a	a	t	a	t	a	a	t	a	a	a	g	t	g	t	a	c	c	-	-	c	a	-	-	a	t	t	a	t	t	g	g	c	a	t	c	c	t	g	a	c	t	t	a	g	c	g	g	c	c	c	g	g	a	g	g	c	c	g	t	t	g	c	c	t	a	t
MH709318	4	a	c	a	t	a	t	a	a	t	a	t	a	a	t	a	a	a	g	t	g	t	a	c	c	a	c	a	a	-	-	a	t	t	a	t	t	g	g	c	a	t	c	c	t	g	a	c	t	t	a	g	c	g	g	c	c	c	g	g	a	g	g	c	c	g	t	t	g	c	c	t	a	t
MH709322	4	a	c	a	t	a	t	a	a	t	a	t	a	a	t	a	a	a	g	t	g	t	a	c	c	a	a	a	a	-	-	a	t	t	a	t	t	g	g	c	a	t	c	c	t	g	a	c	t	t	a	g	c	g	g	c	c	c	g	g	a	g	g	c	c	g	t	t	g	c	c	t	a	t
MH709323	4	a	c	a	t	a	t	a	a	t	a	t	a	a	t	a	a	c	g	t	g	t	a	c	c	a	a	a	a	-	-	a	t	t	a	t	t	g	g	c	a	t	c	c	t	g	a	c	t	t	a	g	c	g	g	c	c	c	g	g	a	g	g	c	c	g	t	t	g	c	c	t	a	t
MH709326	4	a	c	a	t	a	t	a	a	t	a	t	a	a	t	a	a	a	g	t	g	t	a	c	c	a	a	a	a	-	-	a	t	t	a	t	t	g	g	c	a	t	c	c	t	g	a	c	t	t	a	g	c	g	g	c	c	c	g	g	a	g	g	c	c	g	t	t	g	c	t	t	a	t
MH709341	4	a	c	a	t	a	t	a	a	t	a	t	a	a	t	a	a	a	g	t	g	t	a	c	c	a	a	a	a	-	-	a	t	t	a	t	t	g	g	c	a	t	c	c	t	g	a	c	t	t	a	g	c	g	g	c	c	c	g	g	a	g	g	c	c	g	t	t	g	c	c	t	a	t
MH709357	4	a	c	a	t	a	t	a	a	t	a	t	a	a	t	a	a	a	g	t	g	t	a	c	c	a	a	a	a	-	-	a	t	t	a	t	t	g	g	c	a	t	c	c	t	g	a	c	t	t	a	g	c	g	g	c	c	c	g	g	a	g	g	c	c	g	t	t	g	c	c	t	a	t
MH709364	4	a	c	a	t	a	t	a	a	t	a	t	a	a	t	a	a	a	g	t	g	t	a	c	c	-	-	-	a	-	-	a	t	t	a	t	t	g	g	c	a	t	c	c	t	g	a	c	t	t	a	g	c	g	g	c	c	c	g	g	a	g	g	c	c	g	t	t	g	c	c	t	a	t
MH709370	4	a	c	a	t	a	t	a	a	t	a	t	a	a	t	a	a	a	g	t	g	t	a	c	c	a	a	a	a	-	-	a	t	t	a	t	t	g	g	c	a	t	c	c	t	g	a	c	t	t	a	g	c	g	g	c	c	c	g	g	a	g	g	c	c	g	t	t	g	c	c	t	a	t
MH709371	4	a	c	a	t	a	t	a	a	t	a	t	a	a	t	a	a	a	g	t	g	t	a	c	c	-	-	-	a	-	-	a	t	t	a	t	t	g	g	c	a	t	c	c	t	g	a	c	t	t	a	g	c	g	g	c	c	c	g	g	a	g	g	c	c	g	t	t	g	c	c	t	a	t
MH709372	4	a	c	a	t	a	t	a	a	t	a	t	a	a	t	a	a	a	g	t	g	t	a	c	c	a	a	a	a	-	-	a	t	t	a	t	t	g	g	c	a	t	c	c	t	g	a	c	t	t	a	g	c	g	g	c	c	c	g	g	a	g	g	c	c	g	t	t	g	c	c	t	a	t
DQ457052	5	a	c	a	t	a	t	a	a	t	a	t	a	a	t	a	a	a	g	t	g	t	a	c	c	a	a	a	-	-	-	c	t	t	a	t	t	g	g	c	a	t	c	c	t	g	a	c	t	t	a	g	c	g	g	c	c	c	g	g	a	g	g	c	c	g	t	t	g	c	c	t	a	t
JN704704	5	a	c	a	t	a	t	a	a	t	a	t	a	a	t	a	a	a	g	t	g	t	a	c	c	a	a	a	a	-	-	c	t	t	a	t	t	g	g	c	a	t	c	c	t	g	a	c	t	t	a	g	c	g	g	c	c	c	g	g	a	g	g	t	t	g	t	t	g	c	c	t	a	t
JN704705	5	a	c	a	t	a	t	a	a	t	a	t	a	a	t	a	a	a	g	t	g	t	a	c	c	a	a	a	a	-	-	c	t	t	a	t	t	g	g	c	a	t	c	c	t	g	a	c	t	t	a	g	c	g	g	c	c	c	g	g	a	g	g	c	c	g	t	t	g	c	c	t	a	t
JN704706	5	a	c	a	t	a	t	a	a	t	a	t	a	a	t	a	a	a	g	t	g	t	a	c	c	a	a	a	a	-	-	c	t	t	a	t	t	g	g	c	a	t	c	c	t	g	a	c	t	t	a	g	c	g	g	t	c	c	g	g	a	g	g	c	c	g	t	t	g	c	c	t	a	t
JN704707	5	a	c	a	t	a	t	a	a	t	a	t	a	a	t	a	a	a	g	t	g	t	a	c	c	a	a	a	a	-	-	c	t	t	a	t	t	g	g	c	a	t	c	c	t	g	a	c	t	g	a	g	c	g	g	c	c	c	g	g	a	g	g	c	c	g	t	t	g	c	c	t	a	t
JN704708	5	a	c	a	t	a	t	a	a	t	a	t	a	a	t	a	a	a	g	t	g	t	a	c	c	a	a	a	a	-	-	c	t	t	a	t	t	g	g	c	a	t	c	c	t	g	a	c	t	t	a	g	c	g	g	c	c	c	g	g	a	g	g	c	c	g	t	t	g	c	c	t	a	t
KP771904	5	a	c	a	t	a	t	a	a	t	a	t	a	a	t	a	a	a	g	t	g	t	a	c	c	a	a	a	a	-	-	c	t	t	a	t	t	g	g	c	a	t	c	c	t	g	a	c	t	t	a	g	c	g	g	c	c	c	g	g	a	g	g	c	c	g	t	t	g	c	c	t	a	t
KP771905	5	a	c	a	t	a	t	a	a	c	a	t	a	a	t	a	a	a	g	t	g	t	a	c	c	a	a	a	a	-	-	-	t	t	a	t	t	g	g	c	a	t	c	c	t	g	a	c	t	t	a	g	c	g	g	c	c	c	g	g	a	g	g	c	c	g	t	t	g	c	c	t	a	t
KP771906	5	a	c	a	t	a	t	a	a	c	a	t	a	a	t	a	a	a	g	t	g	t	a	c	c	a	a	a	a	-	-	c	t	t	a	t	t	g	g	c	a	t	c	c	t	g	a	c	t	t	a	g	c	g	g	c	c	c	g	g	a	g	g	c	c	g	t	t	g	c	c	t	a	t
KP771919	5	a	c	a	t	a	t	a	a	t	a	t	a	a	t	a	a	a	g	t	g	t	a	c	c	a	a	a	a	-	-	c	t	t	a	t	t	g	g	c	a	t	c	c	t	g	a	c	t	t	a	g	c	g	g	c	c	c	g	g	a	g	g	c	c	g	t	t	g	c	c	t	a	t
KP771922	5	a	c	a	t	a	t	a	a	t	a	t	a	a	g	a	a	a	g	t	g	t	a	c	c	a	a	a	a	-	-	c	t	t	a	t	t	g	g	c	a	t	c	c	t	g	a	c	t	t	a	g	c	g	g	c	c	c	g	g	a	g	g	c	c	g	t	t	g	c	c	t	a	t
KP771924	5	a	c	a	t	a	t	a	a	t	a	t	a	a	t	a	a	a	g	t	g	t	a	c	c	a	a	a	a	-	-	c	t	t	a	t	t	g	g	c	a	t	c	c	t	g	a	c	t	t	a	g	c	g	g	c	c	c	g	g	a	g	g	c	c	g	t	t	g	c	c	t	a	t
KM355696	5	a	c	a	t	a	c	a	a	t	a	t	a	a	t	a	a	a	g	t	g	t	a	c	c	a	a	a	a	-	-	c	a	t	a	t	t	g	g	c	a	t	c	c	t	g	a	c	t	t	a	g	c	g	g	c	c	c	g	g	a	g	g	c	c	g	t	t	g	c	c	t	a	t
KM355697	5	a	c	a	t	a	t	a	a	t	a	t	a	a	t	a	a	a	g	t	g	t	a	c	c	a	a	a	a	-	-	c	a	t	a	t	t	g	g	c	a	t	c	c	t	g	a	c	t	t	a	g	c	g	g	c	c	c	g	g	a	g	g	c	c	g	t	t	g	c	c	t	a	t
KM355698	5	a	c	a	t	a	t	a	a	t	a	t	a	a	t	a	a	a	g	t	g	t	a	c	c	a	a	a	a	-	-	c	t	t	a	t	t	g	g	c	a	t	c	c	t	g	a	c	t	t	a	g	c	g	g	c	c	c	g	g	a	g	g	c	c	g	t	t	g	c	c	t	a	t
KM355699	5	a	c	a	t	a	t	a	a	t	a	t	a	a	t	a	a	a	g	t	g	t	a	c	c	a	a	a	a	-	-	c	t	t	a	t	t	g	g	c	a	t	c	c	t	g	a	c	t	t	a	g	c	g	g	c	c	c	g	g	a	g	g	c	c	g	t	t	g	c	c	t	a	t
KM355701	5	a	c	a	t	a	t	a	a	t	a	t	a	a	t	a	a	a	g	t	g	t	a	c	c	a	a	a	a	-	-	c	a	t	a	t	t	g	g	c	a	t	c	c	t	g	a	c	t	t	a	g	c	g	g	c	c	c	g	g	a	g	g	c	c	g	t	t	g	c	c	t	a	t
KM355702	5	a	c	a	t	a	t	a	a	t	a	t	a	a	t	a	a	a	g	t	g	t	a	c	c	a	a	a	a	-	-	c	t	t	a	t	t	g	g	c	a	t	c	c	t	g	a	c	t	t	a	g	c	g	g	c	c	c	g	g	a	g	g	c	c	g	t	t	g	c	c	t	a	t
KM355703	5	a	c	a	t	a	t	a	a	t	a	t	a	a	t	a	a	a	g	t	g	t	a	c	c	a	a	a	a	-	-	c	a	t	a	t	t	g	g	c	a	t	c	c	t	g	a	c	t	t	a	g	c	g	g	c	c	c	g	g	a	g	g	c	c	g	t	t	g	c	c	t	a	t
KM355704	5	a	c	a	t	a	t	a	a	t	a	t	a	a	t	a	a	a	g	t	g	t	a	c	c	a	a	a	a	-	-	c	a	t	a	t	t	g	g	c	a	t	c	c	t	g	a	c	t	t	a	g	c	g	g	c	c	c	g	g	a	g	g	c	c	g	t	t	g	c	c	t	a	t
KM355705	5	a	c	a	t	a	t	a	a	t	a	t	a	a	t	a	a	a	g	t	g	t	a	c	c	a	a	a	a	-	-	c	t	t	a	t	t	g	g	c	a	t	c	c	t	g	a	c	t	t	a	g	c	g	g	c	c	c	g	g	a	g	g	c	c	g	t	t	g	c	c	t	a	t
KM355706	5	a	c	a	t	a	t	a	a	t	a	t	a	a	t	a	a	a	g	t	g	t	a	c	c	a	a	a	a	-	-	c	t	t	a	t	t	g	g	c	a	t	c	c	t	g	a	c	t	t	a	g	c	g	g	c	c	c	g	g	a	g	g	c	c	g	t	t	g	c	c	t	a	t
KM355707	5	a	c	a	t	a	t	a	a	t	a	t	a	a	t	a	a	a	g	t	g	t	a	c	c	a	a	a	a	-	-	c	a	t	a	t	t	g	g	c	a	t	c	c	t	g	a	c	t	t	a	g	a	g	g	c	c	c	g	g	a	g	g	c	c	g	t	t	g	c	c	t	a	t
KM355708	5	a	c	a	t	a	t	a	a	t	a	t	a	a	t	a	a	a	g	t	g	t	a	c	c	a	a	a	a	-	-	c	a	t	a	t	t	g	g	c	a	t	c	c	t	g	a	c	t	t	a	g	a	g	g	c	c	c	g	g	a	g	g	c	c	g	t	t	g	c	c	t	a	t
KM355709	5	a	c	a	t	a	t	a	a	t	a	t	a	a	t	a	a	a	g	t	g	t	a	c	c	a	a	a	a	-	-	c	t	t	a	t	t	g	g	c	a	t	c	c	t	g	a	c	t	t	a	g	c	g	g	c	c	c	g	g	a	g	g	c	c	g	t	t	g	c	c	t	a	t
KM355711	5	a	c	a	t	a	t	a	a	t	a	t	a	a	t	a	a	a	g	t	g	t	a	c	c	a	a	a	a	-	-	c	t	t	a	t	t	g	g	c	a	t	c	c	t	g	a	c	t	t	a	g	c	g	g	c	c	c	g	g	a	g	g	c	c	g	t	t	g	c	c	t	a	t
KM355712	5	a	c	a	t	a	t	a	a	t	a	t	a	a	t	a	a	a	g	t	g	t	a	c	c	a	a	a	a	-	-	c	t	t	a	t	t	g	g	c	a	t	c	c	t	g	a	c	t	t	a	g	c	g	g	c	c	c	g	g	a	g	g	c	c	g	t	t	g	c	c	t	a	t
KY037797	5	[illegible]	[illegible]	[illegible]	[illegible]	[illegible]	[illegible]	[illegible]	[illegible]	[illegible]	[illegible]	[illegible]	[illegible]	[illegible]	[illegible]	[illegible]	[illegible]	[illegible]	[illegible]	[illegible]	[illegible]	[illegible]	[illegible]	[illegible]	[illegible]	[illegible]	[illegible]	[illegible]	[illegible]	[illegible]	[illegible]	[illegible]	[illegible]	[illegible]	[illegible]	[illegible]	[illegible]	[illegible]	[illegible]	[illegible]	[illegible]	[illegible]	[illegible]	[illegible]	[illegible]	[illegible]	[illegible]	[illegible]	[illegible]	[illegible]	[illegible]	[illegible]	[illegible]	[illegible]	[illegible]	[illegible]	[illegible]	[illegible]	[illegible]	[illegible]	[illegible]	[illegible]	[illegible]	[illegible]	[illegible]	[illegible]	[illegible]	[illegible]	[illegible]	[illegible]	[illegible]	[illegible]	[illegible]	[illegible]

Supplementary Table 3: 2880 SN

GB-ID	Clade markers																																																									2	2														
	Position in Dumas	58791	58831	58914	58950	59094	59199	59287	59293	59377	59386	59395	59591	59713	59752	59781	59830	59954	59981	59987	60003	60098	60158	60224	60266	60268	60269	60270	60277	60279_1	60279_2	60280	60292	60374	60405	60447	60458	60469	60479	60537	60552	60556	60558	60561	60599	60614	60630	60659	60681	60701	60777	60781	60821	60839	60840	60845	60990	61008	61018	61019	61029	61052	61180	61201	61202	61239	61267	61281	61338	61365	61629	61644	61656	61667
MH499467	5	a	c	a	t	a	t	a	a	t	a	t	a	a	-	a	a	a	g	t	g	t	a	c	c	a	a	a	-	-	-	c	t	t	a	t	t	g	g	c	a	c	c	c	t	g	a	c	t	t	a	g	c	g	g	c	c	c	g	g	a	g	g	c	c	g	t	t	g	c	c	t	a	t
MH499469	5	a	c	a	t	a	t	a	a	t	a	t	a	a	t	a	a	a	g	t	g	t	a	c	c	a	a	a	-	-	-	c	t	t	a	t	t	g	g	c	a	t	c	c	t	g	a	c	t	t	a	g	c	g	g	c	c	c	g	g	a	g	g	c	c	g	t	t	g	c	c	t	a	t
MH709321	5	a	t	a	t	a	t	a	a	t	a	t	a	a	t	a	a	a	g	t	g	t	a	c	c	a	a	a	a	-	-	c	t	t	a	t	t	g	g	c	a	t	c	c	t	g	a	c	t	t	a	g	c	g	g	c	c	c	g	g	a	g	g	c	c	g	t	t	g	c	c	t	a	t
MH709324	5	a	c	a	t	a	t	a	a	t	a	t	a	a	t	a	a	a	g	t	g	t	a	c	c	a	a	a	a	-	a	c	t	t	a	c	t	g	g	c	a	t	c	c	t	g	a	c	t	t	a	g	c	g	g	c	c	c	g	g	a	g	g	c	c	g	t	t	g	c	c	t	a	t
MH709325	5	a	c	a	t	a	t	a	a	t	a	t	a	a	t	a	a	a	g	t	g	t	a	c	c	a	a	a	a	-	-	c	t	t	a	t	t	g	g	c	a	t	c	c	t	g	a	c	t	t	a	g	c	g	g	c	c	c	g	g	a	g	g	c	c	g	t	t	g	c	c	t	a	t
MH709327	5	a	c	a	t	a	t	a	a	t	a	t	a	a	t	a	a	a	g	t	g	t	a	c	c	a	a	a	a	-	-	c	t	t	a	t	t	g	g	c	a	t	c	c	t	g	a	c	t	t	a	g	c	g	g	c	c	c	g	g	a	g	g	c	c	g	t	t	g	c	c	t	a	t
MH709328	5	a	c	a	t	a	t	a	a	t	a	t	a	a	t	a	a	a	g	t	g	t	a	c	c	a	a	a	a	-	-	c	t	t	a	t	t	g	g	c	a	t	c	c	t	g	a	c	t	t	a	g	c	g	g	c	c	c	g	g	a	g	g	c	c	g	t	t	g	c	c	t	a	t
MH709330	5	a	c	a	t	a	t	a	a	t	a	t	a	a	t	a	a	a	g	t	g	t	a	c	c	c	a	a	a	-	-	c	t	t	a	t	t	g	g	c	a	t	c	c	t	g	a	c	t	t	a	g	c	g	g	c	c	c	g	g	a	g	g	c	c	g	t	t	g	c	c	t	a	t
MH709332	5	a	c	a	t	a	t	a	a	t	a	t	a	a	t	a	a	a	g	t	g	t	a	c	c	a	a	a	a	-	-	c	t	t	a	t	t	g	g	c	a	t	c	c	t	g	a	c	t	t	a	g	c	g	g	c	c	c	g	g	a	g	g	c	c	g	t	t	g	c	c	t	a	t
MH709334	5	a	c	a	t	a	t	a	a	t	a	t	a	a	t	a	a	a	g	t	g	t	a	c	a	a	a	a	a	-	-	c	t	t	a	t	t	g	g	c	a	t	c	c	t	g	a	c	t	t	a	g	c	g	g	c	c	c	g	g	a	g	g	c	c	g	t	t	g	c	c	t	a	t
MH709335	5	a	c	a	t	a	t	a	a	t	a	t	a	a	t	a	a	a	g	t	g	t	a	c	c	a	a	a	a	-	-	c	t	t	a	t	t	g	g	c	a	t	c	c	t	g	a	c	t	t	a	g	c	g	g	c	c	c	g	g	a	g	g	c	c	g	t	t	g	c	c	t	a	t
MH709336	5	a	c	a	t	a	t	a	a	t	a	t	a	a	t	a	a	a	g	t	g	t	a	c	c	a	a	a	a	-	-	c	t	t	a	t	t	g	g	c	a	t	c	c	t	g	a	c	t	t	a	g	c	g	g	c	c	c	g	g	a	g	g	c	c	g	t	t	g	c	c	t	a	t
MH709338	5	a	c	a	t	a	t	a	a	t	a	t	a	a	t	a	a	a	g	t	g	t	a	c	c	a	a	a	a	-	-	c	t	t	a	t	t	g	g	c	a	t	c	c	t	g	a	c	t	t	a	g	c	g	g	c	c	c	g	g	a	g	g	c	c	g	t	t	g	c	c	t	a	t
MH709340	5	a	c	a	t	a	t	a	a	t	a	t	a	a	t	a	a	a	g	t	g	t	a	c	c	a	a	a	a	-	-	c	t	t	a	t	t	g	g	c	a	t	c	c	t	g	a	c	t	t	a	g	c	g	g	c	c	c	g	g	a	g	g	c	c	g	t	t	g	c	c	t	c	t
MH709342	5	a	c	a	t	a	t	a	a	t	a	t	a	a	t	a	a	a	g	t	g	t	a	c	c	a	a	a	a	-	-	c	t	t	a	t	t	g	g	c	a	t	c	c	t	g	a	c	t	t	a	g	c	g	g	c	c	c	g	g	a	g	g	c	c	g	t	t	g	c	c	t	a	t
MH709343	5	a	c	a	t	a	t	a	a	t	a	t	a	a	t	a	a	a	g	t	g	t	a	c	c	a	a	a	a	-	-	c	t	t	a	t	t	g	g	c	a	t	c	c	t	g	a	c	t	t	a	g	c	g	g	c	c	c	g	g	a	g	g	c	c	g	t	t	g	c	c	t	a	t
MH709345	5	a	c	a	t	a	t	a	a	t	a	t	a	a	t	a	a	a	g	t	g	t	a	c	c	c	a	a	a	-	-	c	t	t	a	t	t	g	g	c	a	t	c	c	t	g	a	c	t	t	a	g	c	g	g	c	c	c	g	g	a	g	g	c	c	g	t	t	g	c	c	t	a	t
MH709347	5	a	c	a	t	a	t	a	a	t	a	t	a	a	t	a	a	a	g	t	g	t	a	c	c	a	a	a	a	-	-	c	t	t	a	t	t	g	g	c	a	t	c	c	t	g	a	c	t	t	a	g	c	g	g	c	c	c	g	g	a	g	g	c	c	g	t	t	g	c	c	t	a	t
MH709349	5	a	c	a	t	a	t	a	a	t	a	t	a	a	t	a	a	a	g	t	g	t	a	c	c	a	a	a	a	-	-	c	t	t	a	t	t	g	g	c	a	t	c	c	t	g	a	c	t	t	a	g	c	g	g	c	c	c	g	g	a	g	g	c	c	g	t	t	g	c	c	t	a	t
MH709350	5	a	c	a	t	a	t	a	a	t	a	t	a	a	t	a	a	a	g	t	g	t	a	c	c	n	n	n	-	-	-	c	t	t	a	t	t	g	g	c	a	t	c	c	t	g	a	c	t	t	a	g	c	g	g	c	c	c	g	g	a	g	g	c	c	g	t	t	g	c	c	t	a	t
MH709351	5	a	c	a	t	a	t	a	a	t	a	t	a	a	t	a	a	a	g	t	g	t	a	c	c	a	a	a	a	-	-	c	t	t	a	t	t	g	g	c	a	t	a	c	t	g	a	c	t	t	a	g	c	g	g	c	c	c	g	g	a	g	g	c	c	g	t	t	g	c	c	t	a	t
MH709353	5	a	c	a	t	a	t	a	a	t	a	t	a	a	t	a	a	a	g	t	g	t	a	c	c	a	a	a	a	-	-	c	t	t	a	t	t	g	g	c	a	t	c	c	t	g	a	c	t	t	a	g	c	g	g	c	c	c	g	g	a	g	g	c	c	g	t	t	g	c	c	t	a	t
MH709358	5	a	c	a	t	a	t	a	a	t	a	t	a	a	t	a	a	a	g	t	g	t	a	c	c	a	a	a	a	-	-	c	t	t	a	t	t	g	g	c	a	t	c	c	a	g	a	c	t	t	a	g	c	g	g	c	c	c	g	g	a	g	g	c	c	g	t	t	g	c	c	t	a	t
MH709360	5	a	c	a	t	a	t	a	a	t	a	t	a	a	t	a	a	a	g	t	g	t	a	c	c	a	a	a	a	-	-	c	t	t	a	t	t	g	g	c	a	t	c	c	t	g	a	c	t	t	a	g	c	g	g	c	c	c	g	g	a	g	g	c	c	g	t	t	g	c	c	t	a	t
MH709362	5	a	c	a	t	a	t	a	a	t	a	t	a	a	t	a	a	a	g	t	g	t	a	c	c	a	a	a	a	-	-	c	t	t	a	t	t	g	g	c	a	t	c	c	t	g	a	c	t	t	a	g	c	g	g	c	c	c	g	g	a	g	g	c	c	g	t	t	g	c	c	t	a	t
MH709365	5	a	c	a	t	a	t	a	a	t	a	t	a	a	t	a	a	a	g	t	g	t	a	c	c	a	a	a	-	-	-	c	t	t	a	t	t	g	g	c	a	t	c	c	t	g	a	c	t	t	a	g	c	g	g	c	c	c	g	g	a	g	g	c	c	g	t	t	g	c	c	t	a	t
MH709373	5	a	c	a	t	a	t	a	a	t	a	t	a	a	t	a	a	a	g	t	g	t	a	c	c	c	a	a	a	-	-	c	t	t	a	t	t	g	g	c	a	t	c	c	t	g	a	c	t	t	a	g	c	g	g	c	c	c	g	g	a	g	g	c	c	g	t	t	g	c	c	t	a	t
MH709374	5	a	c	a	t	a	t	a	a	t	a	t	a	a	t	a	a	a	g	t	g	t	a	c	c	a	a	a	a	-	-	c	t	t	a	t	t	g	g	c	a	t	c	c	t	g	a	c	t	t	a	g	c	g	g	c	c	c	g	g	a	g	g	c	c	g	t	t	g	c	c	t	a	t
KC112914	6	a	c	a	t	a	t	a	a	t	a	t	a	a	t	a	a	a	g	t	g	t	g	c	c	a	a	a	a	-	-	c	t	t	a	t	t	g	g	c	a	t	c	c	t	g	a	c	t	t	a	g	c	g	g	c	c	c	g	g	a	g	a	t	t	g	t	t	g	c	c	t	a	t
KP771914	6	a	c	a	t	a	t	a	a	t	a	t	a	a	t	a	a	a	g	t	g	t	g	c	c	a	a	a	a	-	-	c	t	t	a	t	t	g	g	c	a	t	c	c	t	g	a	c	t	t	a	g	c	g	g	c	c	c	g	g	a	g	g	t	t	g	t	t	g	c	c	t	a	t
KP702725	6	a	c	a	t	a	t	a	a	t	a	t	a	a	t	a	a	a	g	t	g	t	g	c	c	-	-	a	a	-	-	c	t	t	a	t	t	g	g	c	a	t	c	c	t	g	a	c	t	t	a	g	c	g	g	c	c	c	g	g	a	g	g	t	t	g	t	t	g	c	c	t	a	t
KP771890	9	a	c	a	t	a	t	a	a	t	a	t	a	a	t	a	a	a	g	t	g	t	a	c	c	a	a	a	a	-	-	c	t	t	c	t	t	g	g	c	a	t	c	c	t	g	a	c	t	t	a	t	c	g	g	c	c	c	g	g	a	g	g	t	t	t	t	t	g	c	c	t	a	t
JN704710	9	a	c	a	t	a	t	a	a	t	a	t	a	a	t	a	a	a	g	t	g	t	a	c	c	a	a	a	a	-	-	c	t	t	c	t	t	g	g	c	a	t	c	c	t	g	a	c	t	t	g	t	c	g	g	c	c	c	g	g	a	g	g	t	t	g	t	t	g	c	c	t	a	t
KY037798	9	a	c	a	t	a	t	a	a	t	a	t	a	a	t	a	a	a	g	t	g	t	a	c	c	-	-	a	a	-	-	c	t	t	c	t	t	g	a	c	a	t	c	c	t	g	a	c	t	t	g	t	c	g	g	c	c	c	g	g	a	g	g	t	t	g	t	t	g	c	c	t	a	t
MH709312	9	a	c	a	t	a	t	a	a	t	a	t	a	a	t	a	a	a	g	t	g	t	a	c	c	-	-	a	a	-	-	c	t	t	c	t	t	g	a	c	a	t	c	c	t	g	a	c	t	t	g	t	c	g	g	c	c	c	g	g	a	g	g	t	t	g	t	t	g	c	c	t	a	t
MH709352	9	a	c	a	t	a	t	a	a	t	a	t	a	a	t	a	a	a	g	t	g	t	a	c	c	a	a	a	a	-	-	c	t	t	c	t	t	g	a	c	a	t	c	c	t	g	a	c	t	t	g	t	c	g	g	c	c	c	g	g	a	g	g	t	t	g	t	t	g	c	c	t	a	c
AB097933	Vac	a	c	a	t	a	t	a	a	t	a	t	a	a	t	a	a	a	g	t	g	t	a	c	c	-	-	a	a	-	c	c	t	t	a	t	t	g	g	c	a	t	c	c	t	g	a	c	t	t	a	g	c	g	g	c	c	c	a	a	a	g	g	c	c	g	t	t	g	c	c	t	a	t
JN704698	Vac	a	c	g	t	a	t	a	a	t	a	t	a	a	t	a	a	a	g	t	g	t	a	c	c	a	a	a	-	-	c	c	t	t	a	t	t	g	g	c	a	t	c	c	t	g	a	c	t	t	a	g	c	g	g	c	c	c	a	a	a	g	g	c	c	g	t	t	g	c	c	t	a	t
JN704697	Vac	a	c	a	t	a	t	g	a	t	a	t	a	a	t	a	a	a	g	t	g	t	a	c	c	a	a	a	a	-	a	c	t	t	a	t	t	g	g	c	a	t	c	c	t	g	a	c	t	t	a	g	c	g	g	c	c	c	a	a	a	g	g	c	c	g	t	t	g	c	c	t	a	t
DQ008355	Vac	a	c	a	t	a	t	r	a	t	a	t	a	a	t	a	a	a	g	t	g	t	a	c	c	-	a	a	a	-	c	c	t	t	a	t	t	g	g	c	a	t	c	c	t	g	a	c	t	t	a	g	c	g	g	c	c	c	a	a	a	g	g	c	c	g	t	t	g	c	c	t	a	t
JF306641	Vac	a	c	a	t	a	t	g	a	t	a	t	a	a	t	a	a	a	g	t	g	t	a	c	c	-	a	a	a	-	c	c	t	t	a	t	t	g	g	c	a	t	c	c	t	g	a	c	t	t	a	g	c	g	g	c	c	c	a	a	a	g	g	c	c	g	t	t	g	c	c	t	a	t
AB097932	Vac	a	c	a	t	a	t	r	a	t	a	t	a	a	t	a	a	a	g	t	g	t	a	c	c	-	-	a	a	-	c	c	t	t	a	t	t	g	g	c	a	t	c	c	t	g	a	c	t	t	a	g	c	g	g	c	c	c	a	a	a	g	g	c	c	g	t	t	g	c	c	t	a	t
DQ008354	Vac	a	c	a	t	a	t	r	a	t	a	t	a	a	t	a	a	a	g	t	g	t	a	c	c	a	a	a	a	-	c	c	t	t	a	t	t	g	g	c	a	t	c	c	t	g	a	c	t	t	a	g	c	g	g	c	c	c	a	a	a	g	g	c	c	g	t	t	g	c	c	t	a	t
KU926312	Vac	a	c	a	t	a	t	g	a	t	a	t	a	a	t	a	a	a	g	t	g	t	a	c	c	a	a	a	a	-	c	c	t	t	a	t	t	g	g	c	a	t	c	c	t	g	a	c	t	t	a	g	c	g	g	c	c	c	a	a	a	g	g	c	c	g	t	t	g	c	c	t	a	t
KU926313	Vac	a	c	a	t	a	t	g	a	t	a	t	a	a	t	a	a	a	g	t	g	t	a	c	c	a	a	a	a	-	c	c	t	t	a	t	t	g	g	c	a	t	c	c	t	g	a	c	t	t	a	g	c	g	g	c	c	c	a	a	a	g	g	c	c	g	t	t	g	c	c	t	a	t
KU926314	Vac	a	c	a	t	a	t	a	a	t	a	t	a	a	t	a	a	a	g	t	g	t	a	c	c	a	a	a	a	-	c	c	t	t	a	t	t	g	g	c	a	t	c	c	t	g	a	c	t	t	a	g	c	g	g	c	c	c	a	a	a	g	g	c	c	g	t	t	g	c	c	t	a	t
KF558371	Vac	a	c	a	t	a	t	g	a	t	a	t	a	a	t	a	a	a	g	t	g	t	a	c	c	a	a	a	a	-	c	c	t	t	a	t	t	g	g	c	a	t	c	c	t	g	a	c	t	t	a	g	c	g	g	c	c	c	a	a	a	g	g	c	c	g	t	t	g	c	c	t	a	t
KF558372	Vac	a	c	a	t	a	t	g	a	t	a	t	a	a	t	a	a	a	g	t	g	t	a	c	c	-	a	a	a	-	c	c	t	t	a	t	t	g	g	c	a	t	c	c	t	g	a	c	t	t	a	g	c	g	g	c	c	c	a	a	a	g	g	c	c	g	t	t	g	c	c	t	a	t
KF558373	Vac	a	c	a	t	a	t	a	a	t	a	t	a	a	t	a	a	a	g	t	g	t	a	c	c	-	-	a	a	-	c	c	t	t	a	t	t	g	g	c	a	t	c	c	t	g	a	c	t	t	a	g	c	g	g	c	c	c	a	a	a	g	g	c	c	g	t	t	g	c	c	t	a	t
KF558374	Vac	a	c	a	t	a	t	a	a	t	a	t	a	a	t	a	a	a	g	t	g	t	a	c	c	-	a	a	a	-	c	c	t	t	a	t	t	g	g	c	a	t	c	c	t	g	a	c	t	t	a	g	c	g	g	c	c	c	a	a	a	g	g	c	c	g	t	t	g	c	c	t	a	t
KF558375	Vac	a	c	a	t	a	t	a	a	t	a	t	a	a	t	a	a	a	g	t	g	t	a	c	c	-	-	a	a	-	c	c	t	t	a	t	t	g	g	c	c	t	c	c	t	g	a	c	t	t	a	g	c	g	g	c	c	c	a	a	g	g	g	c	c	g	t	t	g	c	c	t	a	t
KF558376	Vac	a	c	a	t	a	t	a	a	t	a	t	a	a	t	a	a	a	g	t	g	t	a	c	c	-	-	a	a	-	c	c	t	t	a	t	t	g	g	c	a	t	c	c	t	g	a	c	t	t	a	g	c	g	g	c	c	c	a	a	a	g	g	c	c	g	t	t	g	c	c	t	a	t
KF558377	Vac	a	c	a	t	a	t	a	a	t	a	t	a	a	t	a	a	a	g	t	g	t	a	c	c	-	-	a	a	-	c	c	t	t	a	t	t	g	g	c	a	t	c	c	t	g	a	c	t	t	a	g	c	g	g	c	c	c	a	a	a	g	g	c	c	g	t	t	g	c	t	t	a	t
KF558378	Vac	a	c	a	t	a	t	a	a	t	a	t	a	a	t	a	a	a	g	t	g	t	a	c	c	-	a	a	a	-	c	c	t	t	a	t	t	g	g	c	a	t	c	c	t	g	a	c	t	t	a	g	c	g	g	c	c	c	a	a	a	g	g	c	c	g	t	t	g	c	c	t	a	t
KF558379	Vac	a	c	a	t	a	t	a	a	t	a	t	a	a	t	a	a	a	g	t	g	t	a	c	c	-	-	a	a	-	c	c	t	t	a	t	t	g	g	c	a	t	c	c	t	g	a	c	t	t	a	g	c	g	g	c	c	c	a	a	a	g	g	c	c	g	t	t	g	c	c	c	a	t
KF558380	Vac	a	c	a	t	a	t	a	a	t	a	t	a	a	t	a	a	a	g	t	g	t	a	c	c	-	-	a	a	-	c	c	t	t	a	t	t	g	g	c	a	t	c	c	t	g	a	c	t	t	a	g	c	g	g	c	c	c	a	a	a	g	g	c	c	g	t	t	g	c	c	t	a	t
KF558381	Vac	a	c	a	t	a	t	a	a	t	a	t	a	a	t	a	a	a	g	t	g	t	a	c	c	-	a	a	a	-	c	c	t	t	a	t	t	g	g	c	a	t	c	c	t	g	a	c	t	t	a	g	c	g	g	c	c	c	a	a	a	g	g	c	c	g	t	t	g	c	c	t	a	t
KF558382	Vac	a	c	a	t	a	t	a	a	t	a	t	a	a	t	a	a	a	g	t	g	t	a	c	c	-	a	a	a	-	c	c	t	t	a	t	t	g	g	c	a	t	c	c	t	g	a	c	t	t	a	g	c	g	g	c	c	c	a	a	g	g	g	c	c	g	t	t	g	c	c	t	a	t
KF558383	Vac	a	c	a	t	a	t	a	a	t	a	t	a	a	t	a	a	a	g	t	g	t	a	c	c	-	-	a	a	-	c	c	t	t	a	t	t	g	g	c	a	t	c	c	t	g	a	c	t	t	a	g	c	g	g	c	c	c	a	a	a	g	g	c	c	g	t	t	g	c	c	t	a	t
KF558384	Vac	a	c	a	t	a	t	a	a	t	a	t	a	a	t	a	a	a	g	t	g	t	a	c	c	-	-	a	a	-	c	c	t	t	a	t	t	g	g	c	a	t	c	c	t	g	a	c	t	t	a	g	c	g	g	c	c	c	a	a	a	g	g	c	c	g	t	t	g	c	c	t	a	t
KF558385	Vac	a	c	a	t	a	t	a	a	t	a	t	a	a	t	a	a	a	g	t	g	t	a	c	c	-	-	a	a	-	c	c	t	t	a	t	t	g	g	c	a	t	c	c	t	g	a	c	t	t	a	g	c	g	g	c	c	c	a	a	a	g	g	c	c	g	t	t	g	c	c	t	a	t
KF558386	Vac	a	c	a	t	a	t	a	a	t	a	t	a	a	t	a	a	a	g	t	g	t	a	c	c	-	a	a	a	-	c	c	t	t	a	t	t	g	g	c	a	t	c	c	t	g	a	c	t	t	a	g	c	g	g	c	c	c	a	a	a	g	g	c	c	g	t	t	g	c	c	t	a	t
KF558387	Vac	a	c	a	t	a	t	g	a	t	a	t	a	a	t	a	a	a	g	t	g	t	a	c	c	-	-	a	a	-	c	c	t	t	a	t	t	g	g	c	a	t	c	c	t	g	a	c	t	t	a	g	c	g	g	c	c	c	a	a	a	g	g	c	c	g	t	t	g	c	c	t	a	t
KF558388	Vac	a	c	a	t	a	t	a	a	t	a	t	a	a	t	a	a	a	g	t	g	t	a	c	c	-	-	a	a	-	c	c	t	t	a	t	t	g	g	c	a	t	c	c	t	g	a	c	t	t	a	g	c	g	g	c	c	c	a	a	a	g	g	c	c	g	t	t	g	c	c	t	a	t
KF558389	Vac	a	c	a	t	a	t	g	a	t	a	t	a	a	t	a	a	a	g	t	g	t	a	c	c	-	-	a	a	-	c	c	t	t	a	t	t	g	g	c	a	t	c	c	t	g	a	c	t	t	a	g	c	g	g	c	c	c	a	a	g	g	g	c	c	g	t	t	g	c	c	t	a	t
KF558390	Vac	a	c	a	t	a	t	a	a	t	a	t	a	a	t	a	a	a	g	t	g	t	a	c	c	-	a	a	a	-	c	c	t	t	a	t	t	g	g	c	c	t	c	c	t	g	a	c	t	t	a	g	c	g	g	c	c	c	a	a	g	g	g	c	c	g	t	t	g	c	c	t	a	t
KF558391	Vac	a	c	a	t	a	t	a	a	t	a	t	a	a	t	a	a	a	g	t	g	t	a	c	c	a	a	a	a	-	c	c	t	t	a	t	t	g	g	c	a	t	c	c	t	g	a	c	t	t	a	g	c	g	g	c	c	c	a	a	a	g	g	c	c	g	t	t	g	c	c	c	a	t
KF811485	Vac	a	c	a	t	a	t	a	a	t	a	t	a	a	t	a	a	a	g	t	g	t	a	c	c	a	a	a	a	-	c	c	t	t	a	t	t	g	g	c	a	t	c	c	t	g	a	c	t	t	a	g	c	g	g	c	c	c	a	a	g	g	g	c	c	g	t	t	g	c	c	t	a	t
KF853225	Vac	a	c	a	t	a	t	g	a	t	a	t	a	a	t	a	a	a	g	t	g	t	a	c	c	-	a	a	a	-	c	c	t	t	a	t	t	g	g	c	a	t	c	c	t	g	a	c	t	t	a	g	c	g	g	c	c	c	a	a	a	g	g	c	c	g	t	t	g	c	c	t	a	t
KF853226	Vac	a	c	a	t	a	t	g	a	t	a	t	a	a	t	a	a	a	g	t	g	t	a	c	c	-	-	a	a	-	c	c	t	t	a	t	t	g	g	c	a	t	c	c	t	g	a	c	t	t	a	g	c	g	g	c	c	c	a	a	a	g	g	c	c	g	t	t	g	c	c	t	a	t
KF853227	Vac	a	c	a	t	a	t	g	a	t	a	t	a	a	t	a	a	a	g	t	g	t	a	c	c	-	a	a	a	-	c	c	t	t	a	t	t	g	g	c	a	t	c	c	t	g	a	c	t	t	a	g	c	g	g	c	c	c	a	a	a	g	g	c	c	g	t	t	g	c	c	t	a	t
KF853228	Vac	a	c	a	t	a	t	a	a	t	a	t	a	a	t	a	a	a	g	t	g	t	a	c	c	-	-	a	a	-	c	c	t	t	a	t	t	g	g	c	a	t	c	c	t	g	a	c	t	t	a	g	c	g	g	c	c	c	a	a	a	g	g	c	c	g	t	t	g	c	c	c	a	t
KF853229	Vac	a	c	a	t	a	t	a	a	t	a	t	g	a	t	a	a	a	g	t	g	t	a	c	c	-	a	a	a	-	c	c	t	t	a	t	t	g	g	c	a	t	c	c	t	g	a	c	t	t	a	g	c	g	g	c	c	c	a	a	a	g	g	c	c	g	t	t	g	c	c	t	a	t
KF853230	Vac	a	c	a	t	a	t	a	a	t	a	t	a	a	t	a	a	a	g	t	g	t	a	c	c	-	a	a	a	-	c	c	t	t	a	t	t	g	g	c	a	t	c	c	t	g	a	c	t	t	a	g	c	g	g	c	c	c	a	a	a	g	g	c	c	g	t	t	g	c	c	t	a	t
KF853231	Vac	a	c	a	t	a	t	a	a	t	a	t	a	a	t	a	a	a	g	t	g	t	a	c	c	-	a	a	a	-	c	c	t	t	a	t	t	g	g	c	a	t	c	c	t	g	a	c	t	t	a	g	c	g	g	c	c	c	a	a	a	g	g	c	c	g	t	t	g	c	c	t	a	t
KF853232	Vac	a	c	a	t	a	t	a	a	t	a	t	a	a	t	a	a	a	g	t	g	t	a	c	c	-	-	a	a	-	c	c	t	t	a	t	t	g	g	c	a	t	c	c	t	g	a	c	t	t	a	g	c	g	g	c	c	c	a	a	a	g	g	c	c	g	t	t	g	c	c	t	a	t
KF853233	Vac	a	c	a	t	a	t	a	g	t	a	t	g	a	t	a	a	a	g	t	g	t	a	c	c	a	a	a	a	-	c	c	t	t	a	t	t	g	g	c	a	t	c	c	t	g	a	c	t	t	a	g	c	g	g	c	c	c	a	a	a	g	g	c	c	g	t	t	g	c	c	t	a	t
KF853234	Vac	a	c	a	t	g	t	a	a	t	a	t	g	a	t	a	a	a	g	t	g	t	a	c	c	-	a	a	a	-	c	c	t	t	a	t	t	g	g	c	a	t	c	c	t	g	a	c	t	t	a	g	c	g	g	c	c	c	a	a	a	g	g	c	c	g	t	t	g	c	c	t	a	t
KF853235	Vac	a	c	a	t	a	t	g	a	t	a	t	a	a	t	a	a	a	g	t	g	t	a	c	c	-	a	a	a	-	c	c	t	t	a	t	t	g	g	c	a	t	c	c	t	g	a	c	t	t	a	g	c	g	g	c	c	c	a	a	a	g	g	c	c	g	t	t	g	c	c	t	a	t
MF898328	Vac	a	c	a	t	a	t	g	a	t	a	t	a	a	t	a	a	a	g	t	g	t	a	c	c	a	a	a	a	-	c	c	t	t	a	t	t	g	g	c	a	t	c	c	t	g	a	c	t	t	a	g	c	g	g	c	c	c	a	a	a	g	g	c	c	g	t	t	g	c	c	t	a	t
JN704709	VIII	a	c	a	t	a	t	a	a	t	a	t	a	a	t	a	a	a	g	t	g	t	g	c	c	a	a	a	a	-	-	c	t	t	a	t	t	g	g	c	a	t	c	c	t	g	a	c	t	t	a	g	c	g	g	c	c	c	g	g	a	g	g	t	t	g	t	t	g	c	c	t	a	t

Supplementary Table 3: 2880 SN

GB-ID	Clade markers																																				2					2																																	
	Position in Dumas	61710	61755	61938	61941	62001	62123	62153	62307	62378	62381	62414	62513	62792	62816	62902	62998	63071	63089	63389	63443	63448	63452	63488	63500	63620	63641	63723	63728	63837	63942	64021	64067	64087	64090	64102	64136	64162	64191	64232	64257	64259	64264	64313	64375	64396	64423	64460	64482	64483	64492	64493	64513	64522	64529	64569	64580	64703	64735	64740	64884	64910	64989	65074	65100	65124	65144	65157	65205	65292	65340	65358	65446	65448	65598
AY548170	1	a	c	g	c	t	c	g	a	t	a	a	a	a	g	g	a	a	t	t	c	t	a	t	g	c	t	g	c	t	g	c	a	t	t	g	a	g	g	c	t	t	g	a	t	t	t	t	g	t	c	a	t	c	g	a	c	g	g	c	a	a	c	c	a	a	c	a	c	c	c	a	a	a	g
AY548171	1	a	c	g	c	t	c	g	a	t	a	a	a	a	g	g	a	a	t	t	c	c	a	t	g	c	t	g	c	t	g	c	a	t	t	g	a	g	g	c	t	t	g	a	t	t	t	t	g	t	c	a	t	c	g	a	c	g	g	c	a	a	c	c	a	a	c	a	c	c	c	a	a	a	g
DQ479953	1	a	c	g	c	t	c	g	a	t	a	a	a	a	g	g	a	a	t	t	c	t	a	t	g	c	t	g	c	t	g	c	a	t	t	g	a	g	g	c	t	t	g	a	t	t	t	t	g	t	c	a	t	c	g	a	c	g	g	c	a	a	c	c	a	a	c	a	c	c	c	a	a	a	g
DQ479954	1	a	c	g	c	t	c	g	a	t	a	a	a	a	g	g	a	a	t	t	c	t	a	t	g	c	t	g	c	t	g	c	a	t	t	g	a	g	g	c	t	t	g	a	t	t	t	t	g	t	c	a	t	c	g	a	c	g	g	c	a	a	c	c	a	a	c	a	c	c	c	a	a	a	g
DQ479958	1	a	c	g	c	t	c	g	a	t	a	a	a	a	g	g	a	a	t	t	c	c	a	t	g	c	t	g	c	t	g	c	a	t	t	g	a	g	g	c	t	t	g	a	t	t	t	t	g	t	c	a	t	c	g	a	c	g	g	c	a	a	c	c	a	a	c	a	c	c	c	c	a	a	g
DQ479959	1	a	c	g	c	t	c	g	a	t	a	a	a	a	g	g	a	a	t	t	c	c	a	t	g	c	t	g	c	t	g	c	a	t	t	g	a	g	g	c	t	t	g	a	t	t	t	t	g	t	c	a	t	c	g	a	c	g	g	c	a	a	c	c	a	a	c	a	c	c	c	c	a	a	g
DQ479961	1	a	c	g	c	t	c	g	a	t	a	a	a	a	g	g	a	a	t	t	c	t	a	t	g	c	t	g	c	t	g	c	a	t	t	g	a	g	g	c	t	t	g	a	t	t	t	t	g	t	c	a	t	c	g	a	c	g	g	c	a	a	c	c	a	a	c	a	c	c	c	a	a	a	g
DQ479962	1	a	c	g	c	t	c	g	a	t	a	a	a	a	g	g	a	a	t	t	c	t	a	t	g	c	t	g	c	t	g	c	a	t	t	g	a	g	g	c	t	t	g	a	t	t	t	t	g	t	c	a	t	c	g	a	c	g	g	c	a	a	c	c	a	a	c	a	c	c	c	a	a	a	g
DQ479963	1	a	c	g	c	t	c	g	a	t	a	a	a	a	g	g	a	a	t	t	c	t	a	t	g	c	t	g	c	t	g	c	a	t	t	g	a	g	g	c	t	t	g	a	t	t	t	t	g	t	c	a	t	c	g	a	c	g	g	c	a	a	c	c	a	a	c	a	c	c	c	a	a	a	g
DQ674250	1	a	c	g	c	t	c	g	a	t	a	a	a	a	g	g	a	a	t	t	c	t	a	t	g	c	t	g	c	t	g	c	a	t	t	g	a	g	g	c	t	t	g	a	t	t	t	t	g	t	c	a	t	c	g	a	c	g	g	c	a	a	t	c	a	g	c	a	c	c	c	a	g	g	g
KX262864	1	a	c	g	c	t	c	g	a	t	a	a	a	a	g	a	a	a	t	t	c	c	a	t	g	c	t	g	c	t	g	c	a	t	t	g	a	g	g	c	t	t	g	a	t	t	t	t	g	t	c	a	t	c	g	a	c	g	g	c	a	a	c	c	a	a	c	a	c	c	c	a	a	a	g
KX262865	1	a	c	g	c	t	c	g	a	t	a	a	a	a	g	g	a	a	t	t	c	t	a	t	g	c	t	g	c	t	g	c	a	t	t	g	a	a	g	c	t	t	g	a	t	t	t	t	g	t	c	a	t	c	g	a	c	g	g	c	a	a	c	c	a	a	c	a	c	c	c	a	a	a	g
KX262866	1	a	c	g	c	t	c	g	a	t	a	a	a	a	g	g	a	a	t	t	c	c	a	t	g	c	t	g	c	t	g	c	a	t	t	g	a	g	g	c	t	t	g	a	t	t	t	t	g	t	c	a	t	c	g	a	c	g	g	c	a	a	c	c	a	a	c	a	c	c	c	a	a	a	g
EU154348	1	a	c	g	c	t	c	g	a	t	a	a	a	a	g	g	a	a	t	t	c	t	a	t	g	c	t	g	c	t	g	c	a	t	t	g	a	g	g	c	t	t	g	a	t	t	t	t	g	t	c	a	t	c	g	a	c	g	g	c	a	a	c	c	a	a	c	a	c	c	c	a	a	a	g
JN704690	1	a	c	g	c	t	c	g	a	t	c	a	a	a	g	g	a	a	t	t	c	t	a	t	g	c	t	g	c	t	g	c	a	t	t	g	a	g	g	c	t	t	g	a	t	t	t	t	g	t	c	a	t	c	g	a	c	g	g	c	a	a	c	c	a	a	c	a	c	c	c	a	a	a	g
JN704691	1	a	c	g	c	t	c	g	a	t	a	a	a	a	g	g	a	a	t	t	c	t	a	t	g	c	t	g	c	t	g	c	a	t	t	g	a	g	g	c	t	t	g	a	t	t	t	t	g	t	c	a	t	c	g	a	c	g	g	c	a	a	c	c	a	a	c	a	c	c	c	a	a	a	g
JN704692	1	a	c	g	c	t	c	g	a	t	c	a	a	a	g	g	a	a	t	t	c	t	a	t	g	c	t	g	c	t	g	c	a	t	t	g	a	g	g	c	t	t	g	a	t	t	t	t	g	t	c	a	t	c	g	a	c	g	g	c	a	a	c	c	a	a	c	a	c	c	c	a	a	a	g
JN704693	1	a	c	g	c	t	c	g	a	t	a	a	a	a	g	g	a	a	t	t	c	t	a	t	g	c	t	g	c	t	g	c	a	t	t	g	a	g	g	c	t	t	g	a	t	t	t	t	g	t	c	a	t	c	g	a	c	g	g	c	a	a	c	c	a	a	c	a	c	c	c	a	a	a	g
JN704694	1	a	c	g	c	t	c	g	a	t	a	a	a	a	g	g	a	a	t	t	c	t	a	t	g	c	t	g	c	t	g	c	a	t	t	g	a	g	g	c	t	t	g	a	t	t	t	t	g	t	c	a	t	c	g	a	c	g	g	c	a	a	c	c	a	a	c	a	c	c	c	a	a	a	g
JN704695	1	a	c	g	c	t	c	g	a	t	c	a	a	a	g	g	a	a	t	t	c	t	a	t	g	c	t	g	c	t	g	c	a	t	t	g	a	g	g	c	t	t	g	a	t	c	t	t	g	t	c	a	t	c	g	a	c	g	g	c	a	a	c	c	a	a	c	a	c	c	c	a	a	a	g
JN704696	1	a	c	g	c	t	c	g	a	t	a	a	a	a	g	g	a	a	t	t	c	t	a	t	g	c	t	g	c	t	g	c	a	t	t	g	a	g	g	c	t	t	g	a	t	t	t	t	g	t	c	a	t	c	g	a	c	g	g	c	a	a	c	c	a	a	c	a	c	c	c	a	a	a	g
NC001348	1	a	c	g	c	t	c	g	a	t	a	a	a	a	g	g	a	a	t	t	c	t	a	t	g	c	t	g	c	t	g	c	a	t	t	g	a	g	g	c	t	t	g	a	t	t	t	t	g	t	c	a	t	c	g	a	c	g	g	c	a	a	c	c	a	a	c	a	c	c	c	a	a	a	g
KP771892	1	a	c	g	c	t	c	g	a	t	a	a	a	a	g	g	a	a	t	t	c	t	a	t	g	c	t	g	c	t	g	c	a	t	t	g	a	g	g	c	t	t	g	a	t	t	t	t	g	t	c	a	t	c	g	a	c	g	g	c	a	a	c	c	a	a	c	a	c	c	c	a	a	a	g
KP771894	1	a	c	g	c	t	c	g	a	t	a	a	a	a	g	g	a	a	t	t	c	t	a	t	g	c	t	g	c	t	g	c	a	t	t	g	a	g	g	c	t	t	g	a	t	t	t	t	g	t	c	a	t	c	g	a	c	g	g	c	a	a	c	c	a	a	c	a	c	c	c	a	a	a	g
KP771900	1	a	c	g	c	t	c	g	a	t	a	a	a	a	g	g	a	a	t	t	c	t	a	t	g	c	t	g	c	t	g	c	a	t	t	g	a	g	g	c	t	t	g	a	t	t	t	t	g	t	c	a	t	c	g	a	c	g	g	c	a	a	c	c	a	a	c	a	c	c	c	a	a	a	g
KP771901	1	a	c	g	c	t	c	g	a	t	a	a	a	a	g	g	a	a	t	t	c	t	a	t	g	c	t	g	c	t	g	c	a	t	t	g	a	g	g	c	t	t	g	a	t	t	t	t	g	t	c	a	t	c	g	a	c	g	g	c	a	a	c	c	a	a	c	a	c	c	c	a	a	a	g
KP771902	1	a	c	g	c	t	c	g	a	t	a	a	a	a	g	g	a	a	t	t	c	t	a	t	g	c	t	g	c	t	g	c	a	t	t	g	a	g	g	c	t	t	g	a	t	t	t	t	g	t	c	a	t	c	g	a	c	g	g	c	a	a	c	c	a	a	c	a	c	c	c	a	a	a	g
KP771908	1	a	c	g	c	t	c	g	a	t	a	a	a	a	g	g	a	a	t	t	c	c	a	t	g	c	t	g	c	t	g	c	a	t	t	g	a	g	g	c	t	t	g	a	t	t	t	t	g	t	c	a	t	c	g	a	c	g	g	c	a	a	c	c	a	a	c	a	c	c	c	a	a	a	g
KP771912	1	a	c	g	c	t	c	g	a	t	a	a	a	a	g	a	a	a	t	t	c	c	a	t	g	c	t	g	c	t	g	c	a	t	t	g	a	g	g	c	t	t	g	a	t	t	t	t	g	t	c	a	t	c	g	a	c	g	g	c	a	a	c	c	a	a	c	a	c	c	c	a	a	a	g
KP771913	1	a	c	g	c	t	c	g	a	t	a	a	a	a	g	a	a	a	t	t	c	c	a	t	g	c	t	g	c	t	g	c	a	t	t	g	a	g	g	c	t	t	g	a	t	t	t	t	g	t	c	a	t	c	g	a	c	g	g	c	a	a	c	c	a	a	c	a	c	c	c	a	a	a	g
KP771917	1	a	c	g	c	t	c	g	a	t	a	a	a	a	g	g	a	a	t	t	c	t	a	t	g	c	t	g	c	t	g	c	a	t	t	g	a	g	g	c	t	t	g	a	t	t	t	t	g	t	c	a	t	c	g	a	c	g	g	c	a	a	c	c	a	a	c	a	c	c	c	a	a	a	g
KP771918	1	a	c	g	c	t	c	g	a	t	a	a	a	a	g	g	a	a	t	t	c	t	a	t	g	c	t	g	c	t	g	c	a	t	t	g	a	g	g	c	t	t	g	a	t	t	t	t	g	t	c	a	t	c	g	a	c	g	g	c	a	a	c	c	a	a	c	a	c	c	c	a	a	a	g
KP771920	1	a	c	g	c	t	c	g	a	t	a	a	a	a	g	g	a	a	t	t	c	t	a	t	g	c	t	g	c	t	g	c	a	t	t	g	a	g	g	c	t	t	g	a	t	t	t	t	g	t	c	a	t	c	g	a	c	g	g	c	a	a	c	c	a	a	c	a	c	c	c	a	a	a	g
MH709361	1	a	c	g	c	t	c	g	a	t	a	a	a	a	g	g	a	a	t	t	c	c	a	t	g	c	t	g	c	t	g	c	a	t	t	g	a	g	g	c	t	t	g	a	t	t	t	t	g	t	c	c	t	c	g	a	c	g	g	c	a	a	c	c	a	a	c	a	c	c	c	a	a	a	g
KU926318	2	a	c	g	c	t	c	g	a	t	a	a	a	a	g	g	a	a	t	t	c	t	a	t	g	c	t	g	c	t	g	c	a	c	t	g	g	g	g	c	t	c	g	a	c	t	t	t	g	t	c	a	t	c	g	a	c	g	g	c	a	a	t	c	a	a	c	a	c	c	c	a	a	a	g
KU926319	2	a	c	g	c	t	c	g	a	t	a	a	a	a	g	g	a	a	t	t	c	t	a	t	g	c	t	g	c	t	g	c	a	t	t	g	g	g	g	c	t	c	g	a	c	t	t	t	g	t	c	a	t	c	g	a	c	g	g	c	a	g	t	c	a	a	c	a	c	c	c	a	a	a	g
KU926320	2	a	c	g	c	t	c	g	g	t	a	a	a	a	g	g	a	a	t	t	c	t	a	t	g	c	t	g	c	t	g	c	a	t	t	g	g	g	g	c	t	c	g	a	c	t	t	t	g	t	c	a	t	c	g	a	c	g	g	c	a	a	t	c	a	a	c	a	c	c	c	a	a	a	g
KU926321	2	a	c	g	c	t	c	g	a	t	a	a	a	a	g	g	a	a	t	t	c	t	a	t	g	c	t	g	c	t	g	c	a	t	t	g	g	g	g	c	t	c	g	a	c	t	t	t	g	t	c	a	t	c	g	a	c	g	g	c	a	a	t	c	a	a	c	a	c	c	c	a	a	a	g
KU926322	2	a	c	g	c	t	c	g	a	t	a	a	a	a	g	g	a	a	t	t	c	t	a	t	g	c	t	g	c	t	g	c	a	t	t	g	g	g	g	c	t	c	g	a	c	t	t	t	g	t	c	a	t	c	g	a	c	g	g	c	a	a	c	c	a	a	c	a	c	c	c	a	a	a	g
KC847290	2	a	c	g	c	t	c	g	a	t	a	a	a	a	g	g	a	a	t	t	c	t	a	t	g	c	t	g	c	t	g	c	a	t	t	g	g	g	g	c	t	c	g	a	c	t	t	t	g	t	c	a	t	c	g	a	c	g	g	c	a	a	t	c	a	a	c	a	c	c	c	a	a	a	g
KJ767491	2	a	c	g	c	t	c	g	a	t	a	a	a	a	g	g	a	a	t	t	c	t	a	t	g	c	t	g	c	t	g	c	a	t	t	g	g	g	g	c	t	c	g	a	c	t	t	t	g	t	c	a	t	c	g	a	c	g	g	c	a	a	t	c	a	a	c	a	c	c	c	a	g	g	g
KJ767492	2	a	c	g	c	t	c	g	a	t	a	a	a	a	g	g	a	a	t	t	c	t	a	t	g	c	t	g	c	t	g	c	a	t	t	g	g	g	g	c	t	c	g	a	c	t	t	t	g	t	c	a	t	c	g	a	c	g	g	c	a	a	t	c	a	a	c	a	c	c	c	a	a	a	g
KJ808816	2	a	c	g	c	t	c	g	a	t	a	a	a	a	g	g	a	a	t	t	c	t	a	t	g	c	t	g	c	t	g	c	a	t	t	g	g	g	g	c	t	c	g	a	c	t	t	t	g	t	c	a	t	c	g	a	c	g	g	c	a	a	t	c	a	a	c	a	c	c	c	a	a	a	g
KP771925	2	a	c	g	c	t	c	g	a	t	a	a	a	a	g	g	a	a	t	t	c	t	a	t	g	c	t	g	c	t	g	c	a	t	t	g	g	g	g	c	t	c	g	a	c	t	t	t	g	t	c	a	t	c	g	a	c	g	g	c	a	a	t	c	a	a	c	a	c	c	c	a	g	g	g
KU926315	2	a	c	g	c	t	c	g	a	t	a	a	a	a	g	g	a	a	t	t	c	t	a	t	g	c	t	g	c	t	g	c	a	t	t	g	g	g	g	c	t	c	g	a	c	t	t	t	g	t	c	a	t	c	g	a	c	g	g	c	a	a	t	c	a	a	c	a	c	c	c	a	a	a	g
KU926316	2	a	c	g	c	t	c	g	a	t	a	a	a	a	g	g	a	a	t	t	c	t	a	t	g	c	t	g	c	t	g	c	a	t	t	g	g	g	g	c	t	c	g	a	c	t	t	t	g	t	c	a	t	c	g	a	c	g	g	c	a	a	t	c	a	a	c	a	c	c	c	a	g	g	g
KU926317	2	a	c	g	c	t	c	g	a	t	a	a	a	a	g	g	a	a	t	t	c	t	a	t	g	c	t	g	c	t	g	c	a	t	t	g	g	g	g	c	t	c	g	a	c	t	t	t	g	t	c	a	t	c	g	a	c	g	g	c	a	a	t	c	a	a	c	a	c	c	c	a	g	g	g
KY037796	2	a	c	g	c	t	c	g	a	t	a	a	a	a	g	g	a	a	t	t	c	t	a	t	g	c	t	g	c	t	g	c	a	t	t	g	g	g	g	c	t	c	g	a	c	t	t	t	g	t	c	a	t	c	g	a	c	g	g	c	a	a	t	c	a	a	c	a	c	c	c	a	a	a	g
MF004348	2	a	c	g	c	t	c	g	a	t	a	a	a	a	g	g	a	a	t	t	c	t	a	t	g	c	t	g	c	t	g	c	a	t	t	g	g	g	g	c	t	c	g	a	c	t	t	t	g	t	c	a	t	c	g	a	c	g	g	c	a	a	t	c	a	a	c	a	c	c	c	a	a	a	g
MH709309	2	a	c	g	c	t	c	g	a	t	a	a	a	a	g	g	a	a	t	t	c	t	a	t	g	c	t	g	c	t	g	c	a	t	t	g	g	g	g	c	t	c	g	a	c	t	t	c	g	t	c	a	t	c	g	a	c	g	g	c	a	a	c	c	a	a	c	a	c	c	c	a	a	a	g
MH709310	2	a	c	g	c	t	c	g	a	t	a	a	a	a	g	g	a	a	t	t	c	t	a	t	g	c	t	g	c	t	g	c	a	t	t	g	g	g	g	c	t	c	g	a	c	t	t	t	g	t	c	a	t	c	g	a	c	g	g	c	a	a	t	c	a	a	c	a	c	c	c	a	a	a	g
MH709311	2	a	c	g	c	t	c	g	a	t	a	a	a	a	g	g	a	a	t	t	c	t	a	t	g	c	t	g	c	t	g	c	a	t	t	g	g	g	g	c	t	c	g	a	c	t	t	t	g	t	c	a	t	c	g	a	c	g	g	c	a	a	t	c	a	a	c	a	c	c	c	a	a	a	g
MH709313	2	a	c	g	c	t	c	g	a	t	a	a	a	a	g	g	a	g	t	t	c	t	a	t	g	c	t	g	c	t	g	c	a	t	t	g	g	g	g	c	t	c	g	a	c	t	t	t	g	t	c	a	t	c	g	a	c	g	g	c	a	a	t	c	g	a	c	a	c	c	c	a	a	a	g
MH709314	2	a	c	g	c	t	c	g	a	t	a	a	a	a	g	g	a	a	t	t	c	t	a	t	g	c	t	g	c	t	g	c	a	t	t	g	g	g	g	c	t	c	g	a	c	t	t	t	g	t	c	a	t	c	g	a	c	g	g	c	a	a	t	c	a	a	c	a	c	c	c	a	a	a	g
MH709315	2	a	c	g	c	t	c	g	a	t	a	a	a	a	g	g	a	a	t	t	c	t	a	t	g	c	t	g	c	t	g	c	a	t	t	g	g	g	g	c	t	c	g	a	c	t	t	t	g	t	c	a	t	c	g	a	c	g	g	c	a	a	t	c	a	a	c	a	c	c	c	a	a	a	g
MH709319	2	a	c	g	c	t	c	g	a	t	a	a	a	a	g	g	a	a	t	t	c	t	a	t	g	c	t	g	c	t	g	c	a	t	t	g	g	g	g	c	t	c	g	a	c	t	t	t	g	t	c	a	t	c	g	a	c	g	g	c	a	a	t	c	a	a	c	a	c	c	c	a	a	a	g
MH709320	2	a	c	g	c	t	c	g	a	t	a	a	a	a	g	g	a	a	t	t	c	t	a	t	g	c	t	g	c	t	g	c	a	t	t	g	g	g	g	c	t	c	g	a	c	t	t	t	g	t	c	a	t	c	g	a	c	g	g	c	a	a	t	c	a	a	c	a	c	c	c	a	a	a	g
MH709329	2	a	c	g	c	t	c	g	a	t	a	a	a	a	g	g	a	a	t	t	c	t	a	t	g	c	t	g	c	t	g	c	a	t	t	g	g	g	g	c	t	c	g	a	c	t	t	t	g	t	c	a	t	c	g	a	c	g	g	c	a	a	t	c	a	a	c	a	c	c	c	a	a	a	g
MH709331	2	a	c	g	c	t	c	g	a	t	a	a	a	a	g	g	a	a	t	t	c	t	a	t	g	c	t	g	c	t	g	c	a	t	t	g	g	g	g	c	t	c	g	a	c	t	t	t	g	t	c	a	t	c	g	a	c	g	g	c	a	a	t	c	a	a	c	a	c	a	c	a	a	a	g
MH709333	2	a	c	g	c	t	c	g	a	t	a	a	a	a	g	g	a	a	t	t	c	t	a	t	g	c	t	g	c	t	g	c	a	t	t	g	g	g	g	c	t	c	g	a	c	t	t	t	g	t	c	a	t	c	g	a	c	g	g	c	a	a	t	c	a	a	c	a	c	c	c	a	a	a	g
MH709337	2	a	c	g	c	t	c	g	a	t	a	a	a	a	g	g	a	a	t	t	c	t	a	t	g	c	t	g	c	t	g	c	a	t	g	g	g	g	g	c	t	c	g	a	c	t	t	t	g	t	c	a	t	c	g	a	c	g	g	c	a	a	t	c	a	a	c	a	c	c	c	a	a	a	g
MH709339	2	a	c	g	c	t	c	g	a	t	a	a	a	a	g	g	a	a	t	t	c	t	a	t	g	c	t	g	c	t	a	c	a	t	t	g	g	g	g	c	t	c	g	a	c	t	t	t	g	t	c	a	t	c	g	a	c	g	g	c	a	a	t	c	a	a	c	a	c	c	c	a	a	a	g
MH709344	2	a	c	g	c	t	c	g	a	t	a	a	a	a	g	g	a	a	t	t	c	t	a	t	g	c	t	g	c	t	g	c	a	t	t	g	g	g	g	c	t	c	g	a	c	t	t	t	g	t	c	a	t	c	g	a	c	g	g	c	a	a	t	c	a	a	c	a	c	c	c	a	a	a	g
MH709348	2	a	c	g	c	t	c	g	a	t	a	a	a	a	g	g	a	a	t	t	c	t	a	t	g	c	t	g	c	t	g	c	a	t	t	g	g	g	g	c	t	c	g	a	c	t	t	t	g	t	c	a	t	c	g	a	c	g	g	c	a	a	t	c	a	a	c	a	c	c	c	a	a	a	g
MH709355	2	a	c	g	c	t	c	g	a	t	a	a	a	a	g	g	a	a	t	t	c	t	a	t	g	c	t	g	c	t	g	c	a	t	t	g	g	g	g	c	t	c	g	a	c	t	t	t	g	t	c	a	t	c	g	a	c	g	g	c	a	a	t	c	a	a	c	a	c	c	c	a	a	a	g
MH709359	2	a	c	g	c	t	c	g	a	t	a	a	a	a	g	g	a	a	t	t	c	t	a	t	g	c	t	g	c	t	g	c	a	t	t	g	g	g	g	c	t	c	g	a	c	t	t	t	g	t	c	a	t	c	g	a	c	g	g	c	a	a	t	c	a	a	c	a	c	c	c	a	a	a	g
MH709363	2	a	c	g	c	t	c	g	a	t	a	a	a	a	g	g	a	a	t	t	c	t	a	t	g	c	t	g	c	t	g	c	a	t	t	g	g	g	g	c	t	c	g	a	c	t	t	t	g	t	c	a	t	c	g	a	c	g	g	c	a	a	t	c	a	a	c	a	c	c	c	a	a	a	g
MH709366	2	a	c	g	c	t	c	g	a	t	a	a	a	a	g	g	a	a	t	t	c	t	a	t	g	c	t	g	c	t	g	c	a	t	t	g	g	g	g	c	t	c	g	a	c	t	t	t	g	t	c	a	t	c	g	a	c	g	g	c	a	a	t	c	a	a	c	a	c	c	c	a	a	a	g
MH709376	2	a	c	g	c	t	c	g	a	t	a	a	a	a	g	g	a	a	t	t	c	t	a	t	g	c	t	g	c	t	g	c	a	t	g	g	g	g	g	c	t	c	g	a	c	t	t	t	g	t	c	a	t	c	g	a	c	g	g	c	a	a	t	c	a	a	c	a	c	c	c	a	a	a	g
MH709377	2	a	c	g	c	t	c	g	a	t	a	a	a	a	g	g	a	a	t	t	t	t	a	t	g	c	t	g	c	t	g	c	a	t	t	g	g	g	g	c	t	c	g	a	c	t	t	t	g	t	c	a	t	c	g	a	c	g	g	c	a	a	t	c	a	a	c	a	c	c	c	a	a	a	g
**MW316406**	2	a	c	g	c	t	c	g	a	t	a	a	a	a	g	g	a	a	t	t	c	t	a	t	g	c	t	g	c	t	g	c	a	t	t	g	g	g	g	c	t	c	g	a	c	t	t	t	g	t	c	a	t	c	g	a	c	g	g	c	a	a	t	c	a	a	c	a	c	c	c	a	a	a	g
AJ871403	3	a	c	g	c	t	c	g	a	t	a	a	a	a	g	g	a	a	t	t	c	t	a	t	g	c	t	g	c	t	g	c	a	t	t	g	a	g	a	c	t	t	g	a	t	t	t	t	g	t	c	a	t	c	g	a	c	g	g	c	a	a	c	c	a	a	c	a	c	c	c	a	a	a	g
DQ479955	3	a	c	g	c	t	c	g	a	c	a	a	a	a	g	g	a	a	t	t	c	t	a	t	g	c	t	g	c	t	g	c	a	t	t	g	a	g	a	c	t	t	g	a	t	t	t	t	g	t	c	a	t	c	g	a	c	g	g	c	a	a	c	c	a	a	c	a	c	c	c	a	a	a	a
DQ479956	3	a	c	g	c	t	c	g	a	t	a	a	a	a	g	g	a	a	t	t	c	t	a	t	g	c	t	g	c	t	g	c	a	t	t	g	a	g	a	c	t	t	g	a	t	t	t	t	g	t	c	a	t	c	g	a	c	g	g	c	a	a	c	c	a	a	c	a	c	c	c	a	a	a	g
DQ479957	3	a	c	g	c	t	c	g	a	t	a	a	a	a	g	g	a	a	t	t	c	t	a	t	g	c	t	g	c	t	g	c	a	t	t	g	a	g	a	c	t	t	g	a	t	t	t	t	g	t	c	a	t	c	a	a	c	g	g	c	a	a	c	c	a	a	c	a	c	c	t	a	a	a	g
JN704699	3	a	c	g	c	t	c	g	a	t	a	a	a	a	g	g	a	a	t	t	c	t	a	t	g	c	t	g	c	t	g	c	a	t	t	g	a	g	a	c	t	t	g	a	t	t	t	t	g	t	c	a	t	c	g	a	c	g	g	c	a	a	c	c	a	a	c	a	c	c	c	a	a	a	g
JN704700	3	a	c	g	c	t	c	g	a	t	a	a	a	a	g	g	a	a	t	t	c	t	a	t	g	c	t	g	c	t	g	c	a	t	t	g	a	g	a	c	t	t	g	a	t	t	t	t	g	t	c	a	t	c	g	a	c	g	t	c	a	a	c	c	a	a	c	a	c	c	c	a	a	a	g
JN704701	3	a	c	g	c	t	c	g	a	t	a	a	a	a	g	g	a	a	t	t	c	t	a	t	g	c	t	g	c	t	g	c	a	t	t	g	a	g	a	c	t	t	g	a	t	t	t	t	g	t	c	a	t	c	g	a	c	g	g	c	a	a	c	c	a	a	c	a	c	c	c	a	a	a	g
JN704702	3	a	c	g	c	t	c	g	a	t	a	a	a	a	g	g	a	a	t	t	c	t	a	t	g	c	t	g	c	t	g	c	a	t	t	g	a	g	a	c	t	t	g	a	t	t	t	t	g	t	c	a	t	c	g	a	c	g	g	c	a	a	c	c	a	a	c	a	c	c	c	a	a	a	g
JN704703	3	a	c	g	c	t	c	g	a	t	a	a	a	a	g	g	a	a	t	t	c	t	a	t	g	c	t	g	c	t	g	c	a	t	t	g	a	g	a	c	t	t	g	a	t	t	t	t	g	t	c	a	t	c	g	a	c	g	g	c	a	a	c	c	a	a	c	a	c	c	c	a	a	a	g
KP771891	3	a	c	g	c	t	c	g	a	t	a	a	a	a	g	g	a	a	t	t	c	t	a	t	g	c	t	g	c	t	g	c	a	t	t	g	a	g	a	c	t	t	g	a	t	t	t	t	g	t	t	a	t	c	g	a	c	g	g	c	a	a	c	c	a	a	c	a	c	c	c	a	a	a	g
KP771893	3	a	c	g	c	t	c	g	a	t	a	a	a	a	g	g	a	a	t	t	c	t	a	t	g	c	t	g	c	t	g	c	a	t	t	g	a	g	a	c	t	t	g	a	t	t	t	t	g	t	c	a	t	c	g	a	t	g	g	c	a	a	c	c	a	a	c	a	c	c	c	a	a	a	g
KP771895	3	a	c	g	c	t	c	g	a	t	a	a	a	a	g	g	a	a	t	t	c	t	a	t	g	c	t	g	c	t	g	c	a	t	t	g	a	g	a	c	t	t	g	a	t	t	t	t	g	t	c	a	t	c	g	a	c	g	g	c	a	a	c	c	a	a	c	a	c	c	c	a	a	a	g
KP771896	3	a	c	g	c	t	c	g	a	t	a	a	a	a	g	g	a	a	t	t	c	t	a	t	g	c	t	g	c	t	g	c	a	t	t	g	a	g	a	c	g	t	g	a	t	t	t	t	g	t	c	a	t	c	g	a	c	g	g	c	a	a	c	c	a	a	c	a	c	c	c	a	a	a	g
KP771897	3	a	c	g	c	t	c	g	a	t	a	a	a	a	g	g	a	a	t	t	c	t	a	t	g	c	t	g	c	t	g	c	a	t	t	g	a	g	a	c	t	t	g	a	t	t	t	t	g	t	c	a	t	c	g	a	c	g	g	c	a	a	c	c	a	a	c	a	c	c	c	a	a	a	g
KP771899	3	a	c	g	c	t	c	g	a	t	a	a	a	a	g	g	a	a	t	t	c	t	a	t	g	c	t	g	c	t	g	c	a	t	t	g	a	g	a	c	t	t	g	a	t	t	t	t	g	t	c	a	t	c	g	a	c	g	g	c	a	a	c	c	a	a	c	a	c	c	c	a	a	a	g
KP771903	3	a	c	g	c	t	c	g	a	t	a	a	a	a	g	g	a	a	t	t	c	t	a	t	g	c	t	g	c	t	g	c	a	t	t	g	a	g	a	c	t	t	g	a	t	t	t	t	g	t	c	a	t	c	g	a	c	g	g	c	a	a	c	c	a	a	c	a	c	c	c	a	a	a	g
KP771907	3	a	c	g	c	t	c	g	a	t	a	a	a	a	g	g	a	a	t	t	c	t	a	t	g	c	t	g	c	t	g	c	a	t	t	g	a	g	a	c	t	t	g	a	t	t	t	t	g	t	c	a	t	c	g	a	c	g	g	c	a	a	c	c	a	a	c	a	c	c	c	a	a	a	g
KP771910	3	a	c	g	c	t	c	g	a	t	a	a	a	a	g	g	a	a	t	t	c	t	a	t	g	c	t	g	c	t	g	c	a	t	t	g	a	g	a	c	t	t	g	a	t	t	t	t	g	t	c	a	t	c	g	a	c	g	g	c	a	a	c	c	a	a	c	a	c	c	c	a	a	a	g
KP771915	3	a	c	g	c	t	c	g	a	t	a	a	a	a	g	g	a	a	t	t	c	t	a	t	g	c	t	g	c	t	g	c	a	t	t	g	a	g	a	c	t	t	g	a	t	t	t	t	g	t	c	a	t	c	g	a	c	g	g	c	a	a	c	c	a	a	c	a	c	c	c	a	a	a	g
KP771916	3	a	c	g	c	t	c	g	a	t	a	a	a	a	g	g	a	a	t	t	c	t	a	t	g	c	t	g	c	t	g	c	a	t	t	g	a	g	a	c	t	t	g	a	t	t	t	t	g	t	c	a	t	c	g	a	c	g	g	c	a	a	c	c	a	a	c	a	c	c	c	a	a	a	g
KP771921	3	a	c	g	c	t	c	g	a	t	a	a	a	a	g	g	a	a	t	t	c	t	a	t	g	c	t	g	c	t	g	c	a	t	t	g	a	g	a	c	t	t	g	a	t	t	t	t	g	t	c	a	t	c	g	a	c	g	g	c	a	a	c	c	a	a	c	a	c	c	c	a	a	a	g
KP771923	3	a	c	g	c	t	c	g	a	t	a	a	a	a	g	g	a	a	t	t	c	t	a	t	g	c	t	g	c	t	g	c	a	t	t	g	a	g	a	c	t	t	g	a	t	t	t	t	g	t	c	a	t	c	g	a	c	g	g	c	a	a	c	c	a	a	c	a	c	c	c	a	a	a	g
KP771889	3	a	c	g	c	t	c	g	a	t	a	a	a	a	g	g	a	a	t	t	c	t	a	t	g	c	t	g	c	t	g	c	a	t	t	g	a	g	g	c	t	t	g	a	t	t	t	t	g	t	c	a	t	c	g	a	c	g	g	c	a	a	c	c	a	a	c	a	c	c	c	a	a	a	g
KU926311	3	a	c	g	c	t	c	g	a	t	a	a	a	a	g	g	a	a	t	t	c	t	a	t	g	c	t	g	c	t	g	c	a	t	t	g	a	g	a	c	t	t	g	a	t	t	t	t	g	t	c	a	t	c	g	a	c	g	g	c	g	a	c	c	a	a	c	a	c	c	c	a	a	a	g
JQ911599	3	a	c	g	c	t	c	g	a	t	a	a	a	a	g	g	a	a	t	t	c	t	a	t	g	c	t	g	c	t	g	c	a	t	t	g	a	g	a	c	t	t	g	a	t	t	t	t	g	t	c	a	t	c	g	a	c	g	g	c	a	a	c	c	a	a	c	a	c	c	c	a	a	a	g
MH709317	3	a	c	g	c	t	c	g	a	t	a	a	a	a	g	g	a	a	t	t	c	t	a	t	g	c	g	g	t	t	g	c	a	t	t	g	a	g	a	c	t	t	g	a	c	t	t	t	g	t	c	a	t	c	g	a	c	g	g	c	a	a	c	c	a	a	c	a	c	c	c	a	a	a	g
MH709346	3	a	c	g	c	t	c	g	a	t	a	a	a	a	g	g	a	a	t	t	c	t	a	t	g	c	g	g	t	t	g	c	a	t	t	g	a	g	a	c	t	t	g	a	c	t	t	t	g	t	c	a	t	c	g	a	c	g	g	c	a	a	c	c	a	a	c	a	c	c	c	a	a	a	g
MH709375	3	a	c	g	c	t	c	g	a	t	a	a	a	a	g	g	a	a	t	t	c	t	g	t	g	c	g	g	t	t	g	c	a	t	t	g	a	g	a	c	t	t	g	a	c	t	t	t	g	t	c	a	t	c	g	a	c	g	g	c	a	a	c	c	a	a	c	a	c	c	c	a	a	a	g
DQ452050	4	a	c	g	c	t	c	g	a	t	a	a	a	a	g	g	a	a	t	t	c	t	a	t	g	c	t	g	c	t	g	c	a	t	t	g	a	g	g	c	t	t	g	a	c	t	t	t	g	t	c	a	t	c	g	a	c	a	g	t	a	a	c	c	a	a	c	a	c	c	c	a	a	a	g
DQ479960	4	a	c	g	c	t	c	g	a	t	a	a	a	a	g	g	a	a	t	t	c	t	a	t	g	c	t	g	c	t	g	c	a	t	t	g	a	g	g	c	t	t	g	a	c	t	t	t	g	t	c	a	t	c	g	a	c	a	g	t	a	a	c	c	a	a	c	a	t	c	c	a	a	a	g
KP771898	4	a	c	g	c	t	c	g	a	t	a	a	a	a	g	g	a	a	t	t	c	t	a	t	g	c	t	g	c	t	g	c	a	t	t	g	a	g	g	c	t	t	g	a	c	t	t	t	g	t	c	a	t	c	g	a	c	a	g	t	a	a	c	c	a	a	c	a	c	c	c	a	a	a	g
MH709316	4	a	c	g	c	t	t	g	a	t	a	a	a	a	g	g	a	a	t	t	c	t	a	t	g	c	t	g	c	t	g	c	a	t	t	g	a	g	g	a	t	t	g	a	c	t	t	t	g	t	c	a	t	c	g	a	c	a	g	t	a	a	c	c	a	a	c	a	c	c	c	a	a	a	g
MH709318	4	a	c	g	c	t	c	g	a	t	a	a	a	a	g	g	a	a	t	t	c	t	a	t	g	c	t	g	c	t	g	c	a	t	t	g	a	g	g	c	t	t	g	a	c	t	t	t	g	g	c	a	t	c	g	a	c	a	g	t	a	a	c	c	a	a	c	a	c	c	c	a	a	a	g
MH709322	4	a	c	g	c	t	c	g	a	t	a	a	a	a	g	g	a	a	t	t	c	t	a	t	g	c	t	g	c	t	g	c	a	t	t	g	a	g	g	c	t	t	g	a	c	t	t	t	g	t	c	a	t	c	g	a	c	a	g	t	a	a	c	c	a	a	c	a	c	c	c	a	a	a	g
MH709323	4	a	c	g	c	t	c	g	a	t	a	a	a	a	g	g	a	a	t	t	c	t	a	t	g	c	t	g	c	t	g	c	a	t	t	g	a	g	g	c	t	t	g	a	c	t	t	t	g	t	c	a	t	c	g	g	c	a	g	t	a	a	c	c	a	a	c	a	c	c	c	a	a	a	g
MH709326	4	a	c	g	c	t	c	g	a	t	a	a	a	a	g	g	a	a	t	t	c	t	a	t	g	c	t	g	c	t	g	c	a	t	t	g	a	g	g	c	t	t	g	a	c	t	t	t	g	t	c	a	t	c	g	a	c	a	g	t	a	a	c	c	a	a	c	a	c	c	c	a	a	a	g
MH709341	4	a	c	g	c	t	c	g	a	t	a	a	a	a	g	g	a	a	t	t	c	t	a	g	g	c	t	g	c	t	g	c	a	t	t	g	a	g	g	c	t	t	g	a	c	t	t	t	g	t	c	a	t	c	g	a	c	a	g	t	a	a	c	c	a	a	t	a	c	c	c	a	a	a	g
MH709357	4	a	c	g	c	t	c	g	a	t	a	a	a	a	g	g	a	a	t	t	c	t	a	t	g	c	t	g	c	t	g	c	a	t	t	g	a	g	g	c	t	t	g	a	c	t	t	t	g	t	c	a	t	c	g	a	c	a	g	t	a	a	c	c	a	a	c	a	c	c	c	a	a	a	g
MH709364	4	a	c	g	c	t	c	t	a	t	a	a	a	a	g	g	a	a	t	t	c	t	a	t	g	c	t	g	c	t	g	c	a	t	t	g	a	g	g	c	t	t	g	a	c	t	t	t	g	t	c	a	t	c	g	a	c	a	g	t	a	a	c	c	a	a	c	a	c	c	c	a	a	a	g
MH709370	4	a	c	g	c	t	c	g	a	t	a	a	a	a	g	g	a	a	t	t	c	t	a	t	g	c	t	g	c	t	g	c	a	t	t	g	a	g	g	c	t	t	g	a	c	t	t	t	g	t	c	a	t	c	g	a	c	a	g	t	a	a	c	c	a	a	c	a	c	c	c	a	a	a	g
MH709371	4	a	c	g	c	t	c	g	a	t	a	a	a	a	g	g	a	a	t	t	c	t	a	t	g	c	t	g	c	t	g	c	a	t	t	g	a	g	g	c	t	t	g	a	c	t	t	t	g	t	c	a	t	c	g	a	c	a	g	t	a	a	c	c	a	a	c	a	t	c	c	a	a	a	g
MH709372	4	a	c	g	c	t	c	g	a	t	a	a	a	a	g	g	a	a	t	t	c	t	a	t	g	c	t	g	c	t	g	c	a	t	t	g	a	g	g	c	t	t	g	a	c	t	t	t	g	t	c	a	t	c	g	a	c	a	g	t	a	a	c	c	a	a	c	a	t	c	c	a	a	a	g
DQ457052	5	a	c	g	c	t	c	g	a	t	a	a	a	a	g	g	a	a	t	t	c	t	a	t	g	c	g	g	c	t	g	c	a	t	t	g	a	g	g	c	t	t	g	a	c	t	t	t	g	t	c	a	t	t	g	a	c	a	g	t	a	a	c	c	a	a	c	a	c	c	c	a	a	a	g
JN704704	5	a	c	g	c	t	c	g	a	t	a	a	a	a	g	g	a	a	t	t	c	t	a	t	g	c	g	g	c	t	g	c	a	t	t	g	a	g	g	c	t	t	a	a	c	t	t	t	t	t	c	a	t	t	g	a	c	a	g	t	a	a	c	c	a	a	c	a	c	c	c	a	a	a	g
JN704705	5	a	c	g	c	t	c	g	a	t	a	a	a	a	g	g	a	a	t	t	c	t	a	t	g	c	g	g	c	t	g	c	a	t	t	g	a	g	g	c	t	t	g	a	c	t	t	t	g	t	c	a	t	t	g	a	c	a	g	t	a	a	c	c	a	a	c	a	c	c	c	a	a	a	g
JN704706	5	g	c	g	c	t	c	g	a	t	a	a	a	a	g	g	a	a	t	t	c	t	a	t	g	c	g	g	c	t	g	c	a	t	t	g	a	g	g	c	t	t	g	a	c	t	t	t	g	t	c	a	t	t	g	a	c	a	g	t	a	a	c	c	a	a	c	a	c	c	c	a	a	a	g
JN704707	5	a	c	g	c	t	c	g	a	t	a	a	a	a	g	g	a	a	t	t	c	t	a	t	t	c	g	g	c	t	g	c	a	t	t	g	a	g	g	c	t	t	g	a	c	t	t	t	g	t	c	a	t	t	g	a	c	a	g	t	a	a	c	c	a	a	c	a	c	c	c	a	a	a	g
JN704708	5	a	c	g	c	t	c	g	a	t	a	a	a	a	g	g	a	a	t	t	c	t	a	t	g	c	g	g	c	t	g	c	a	t	t	g	a	g	g	c	t	t	g	a	t	t	t	t	g	t	c	a	t	c	g	a	c	a	g	t	a	a	c	c	a	a	c	a	c	c	c	a	a	a	g
KP771904	5	a	c	g	c	t	c	g	a	t	a	a	a	a	g	g	a	a	t	t	c	t	a	t	g	c	g	g	c	t	g	c	a	t	t	g	a	g	g	c	t	t	g	a	c	t	t	t	g	t	c	a	t	t	g	a	c	a	g	t	a	a	c	c	a	a	c	a	c	c	c	a	a	a	g
KP771905	5	a	c	g	c	t	c	g	a	t	a	a	a	a	g	g	a	a	t	t	c	t	a	t	g	c	g	g	c	t	g	c	a	t	t	g	a	g	g	c	t	t	g	a	c	t	t	t	g	t	c	a	t	t	g	a	c	a	g	t	a	a	c	c	a	a	c	a	c	c	c	a	a	a	g
KP771906	5	a	c	g	c	t	c	g	a	t	a	a	a	a	g	g	a	a	t	t	c	t	a	t	g	c	g	g	c	t	g	c	a	t	t	g	a	g	g	c	t	t	g	a	c	t	t	t	g	t	c	a	t	t	g	a	c	a	g	t	a	a	c	c	a	a	c	a	c	c	c	a	a	a	g
KP771919	5	a	c	g	c	t	c	g	a	t	a	a	a	a	g	g	a	a	t	t	c	t	a	t	g	c	g	g	c	t	g	c	a	t	t	g	a	g	g	c	t	t	g	a	c	t	t	t	g	t	c	a	t	t	g	a	c	a	g	t	a	a	c	c	a	a	c	a	c	c	c	a	a	a	g
KP771922	5	a	c	g	c	t	c	g	a	t	a	a	a	a	g	g	a	a	t	t	c	t	a	t	g	c	g	g	c	t	g	c	a	t	t	g	a	g	g	c	t	t	g	a	c	t	t	t	g	t	c	a	t	t	g	a	c	a	g	t	a	a	c	c	a	a	c	a	c	c	c	a	a	a	g
KP771924	5	a	c	g	c	t	c	g	a	t	a	a	a	a	g	g	a	a	t	t	c	t	a	t	g	c	g	g	c	t	g	c	a	t	t	g	a	g	g	c	t	t	g	a	c	t	t	t	g	t	c	a	t	t	g	a	c	a	g	t	a	a	c	c	a	a	c	a	c	c	c	a	a	a	g
KM355696	5	a	c	g	c	t	c	g	a	t	a	a	a	a	g	g	a	a	t	t	c	t	a	t	g	c	g	g	c	t	g	c	a	t	t	g	a	g	g	c	t	t	g	a	c	t	t	t	g	t	c	a	t	t	g	a	c	a	g	t	a	a	c	c	a	a	c	a	c	c	c	a	a	a	g
KM355697	5	a	c	g	c	t	c	g	a	t	a	a	a	a	g	g	a	a	t	t	c	t	a	t	g	c	g	g	c	t	g	c	a	t	t	g	a	g	g	c	t	t	g	a	c	t	t	t	g	t	c	a	t	t	g	a	c	a	g	t	a	a	c	c	a	a	c	a	c	c	c	a	a	a	g
KM355698	5	a	c	g	c	t	c	g	a	t	a	a	a	a	a	g	a	a	t	t	c	t	a	t	g	c	g	g	c	t	g	c	a	t	t	g	a	g	g	c	t	t	g	a	c	t	t	t	g	t	c	a	t	t	g	a	c	a	g	t	a	a	c	c	a	a	c	a	c	c	c	a	a	a	g
KM355699	5	a	c	g	c	t	c	g	a	t	a	a	a	a	a	g	a	a	t	t	c	t	a	t	g	c	g	g	c	t	g	c	a	t	t	g	a	g	g	c	t	t	g	a	c	t	t	t	g	t	c	a	t	t	g	a	c	a	g	t	a	a	c	c	a	a	c	a	c	c	c	a	a	a	g
KM355701	5	a	c	g	c	t	c	g	a	t	a	a	a	a	g	g	a	a	t	t	c	t	a	t	g	c	g	g	c	t	g	c	a	t	t	g	a	g	g	c	t	t	g	a	c	t	t	t	g	t	c	a	t	t	g	a	c	a	g	t	a	a	c	c	a	a	c	a	c	c	c	a	a	a	g
KM355702	5	a	c	g	c	t	c	g	a	t	a	a	a	a	a	g	a	a	t	t	c	t	a	t	g	c	g	g	c	t	g	c	a	t	t	g	a	g	g	c	t	t	g	a	c	t	t	t	g	t	c	a	t	t	g	a	c	a	g	t	a	a	c	c	a	a	c	a	c	c	c	a	a	a	g
KM355703	5	a	c	g	c	t	c	g	a	t	a	a	a	a	g	g	a	a	t	t	c	t	a	t	g	c	g	g	c	t	g	c	a	t	t	g	a	g	g	c	t	t	g	a	c	t	t	t	g	t	c	a	t	t	g	a	c	a	g	t	a	a	c	c	a	a	c	a	c	c	c	a	a	a	g
KM355704	5	a	c	g	c	t	c	g	a	t	a	a	a	a	g	g	a	a	t	t	c	t	a	t	g	c	g	g	c	t	g	c	a	t	t	g	a	g	g	c	t	t	g	a	c	t	t	t	g	t	c	a	t	t	g	a	c	a	g	t	a	a	c	c	a	a	c	a	c	c	c	a	a	a	g
KM355705	5	a	c	g	c	t	c	g	a	t	a	a	a	a	a	g	a	a	t	t	c	t	a	t	g	c	g	g	c	t	g	c	a	t	t	g	a	g	g	c	t	t	g	a	c	t	t	t	g	t	c	a	t	t	g	a	c	a	g	t	a	a	c	c	a	a	c	a	c	c	c	a	a	a	g
KM355706	5	a	c	g	c	t	c	g	a	t	a	a	a	a	a	g	a	a	t	t	c	t	a	t	g	c	g	g	c	t	g	c	a	t	t	g	a	g	g	c	t	t	g	a	c	t	t	t	g	t	c	a	t	t	g	a	c	a	g	t	a	a	c	c	a	a	c	a	c	c	c	a	a	a	g
KM355707	5	a	c	g	c	t	c	g	a	t	a	a	a	a	g	g	a	a	t	t	c	t	a	t	g	c	g	g	c	t	g	c	a	t	t	g	a	g	g	c	t	t	g	a	c	t	t	t	g	t	c	a	c	t	g	a	c	a	g	t	a	a	c	c	a	a	c	a	c	c	c	a	a	a	g
KM355708	5	a	c	g	c	t	c	g	a	t	a	a	a	a	g	g	a	a	t	t	c	t	a	t	g	c	g	g	c	t	g	c	a	t	t	g	a	g	g	c	t	t	g	a	c	t	t	t	g	t	c	a	c	t	g	a	c	a	g	t	a	a	c	c	a	a	c	a	c	c	c	a	a	a	g
KM355709	5	a	c	g	c	t	c	g	a	t	a	a	a	a	a	g	a	a	t	t	c	t	a	t	g	c	g	g	c	t	g	c	a	t	t	g	a	g	g	c	t	t	g	a	c	t	t	t	g	t	c	a	t	t	g	a	c	a	g	t	a	a	c	c	a	a	c	a	c	c	c	a	a	a	g
KM355711	5	a	c	g	c	t	c	g	a	t	a	a	a	a	a	g	a	a	t	t	c	t	a	t	g	c	g	g	c	t	g	c	a	t	t	g	a	g	g	c	t	t	g	a	c	t	t	t	g	t	c	a	t	t	g	a	c	a	g	t	a	a	c	c	a	a	c	a	c	c	c	a	a	a	g
KM355712	5	a	c	g	c	t	c	g	a	t	a	a	a	a	a	g	a	a	t	t	c	t	a	t	g	c	g	g	c	t	g	c	a	t	t	g	a	g	g	c	t	t	g	a	c	t	t	t	g	t	c	a	t	t	g	a	c	a	g	t	a	a	c	c	a	a	c	a	c	c	c	a	a	a	g
KY037797	5	a	c	g	t	t	c	g	a	t	a	a	a	a	g	g	a	a	t	t	c	t	a	t	g	c	g	g	c	t	g	c	a	t	t	g	a	g	g	c	t	t	g	a	c	t	t	t	g	t	c	a	t	t	g	a	c	a	g	t	a	a	c	c	a	a	c	a	c	c	c	a	a	a	g

Supplementary Table 3: 2880 SN

GB-ID	Clade markers																																			2					2																																	
	Position in Dumas	61710	61755	61938	61941	62001	62123	62153	62307	62378	62381	62414	62513	62792	62816	62902	62998	63071	63089	63389	63443	63448	63452	63488	63500	63620	63641	63723	63728	63837	63942	64021	64067	64087	64090	64102	64136	64162	64191	64232	64257	64259	64264	64313	64375	64396	64423	64460	64482	64483	64492	64493	64513	64522	64529	64569	64580	64703	64735	64740	64884	64910	64989	65074	65100	65124	65144	65157	65205	65292	65340	65358	65446	65448	65598
MH499467	5	a	c	g	c	t	c	g	a	t	a	a	a	a	g	g	a	a	t	t	c	t	a	t	g	c	g	g	c	t	g	c	a	t	t	g	a	g	g	c	t	t	g	a	c	t	t	t	g	t	c	a	t	t	g	a	c	a	g	t	a	a	c	-	-	-	-	-	c	c	c	a	a	a	g
MH499469	5	a	c	g	c	t	c	g	a	t	a	a	a	a	g	g	a	a	t	t	c	t	a	t	g	c	g	g	c	t	g	c	a	t	t	g	a	g	g	c	t	t	g	a	c	t	t	t	g	t	c	a	t	t	g	a	c	a	g	t	a	a	c	c	a	a	c	a	c	c	c	a	a	a	g
MH709321	5	a	c	a	c	t	c	g	a	t	a	a	a	a	g	g	a	a	t	t	c	t	a	t	g	c	g	g	c	t	g	c	a	t	t	g	a	g	g	c	t	t	g	a	c	t	t	t	g	t	c	a	t	t	g	a	c	a	g	t	a	a	c	c	a	a	c	a	c	c	c	a	a	a	g
MH709324	5	a	c	g	c	t	c	g	a	t	a	a	a	a	g	g	a	a	t	t	c	t	a	t	g	c	g	g	c	t	g	c	a	t	t	g	a	g	g	c	t	t	g	a	c	t	t	t	g	t	c	a	t	t	g	a	c	a	g	t	a	a	c	c	a	a	c	a	c	c	c	a	a	a	g
MH709325	5	a	c	g	c	t	c	g	a	t	a	a	a	a	a	g	a	a	t	t	c	t	a	t	g	c	g	g	c	t	g	c	a	t	t	g	a	g	g	c	t	t	g	a	c	t	t	t	g	t	c	a	t	t	g	a	c	a	g	t	a	a	c	c	a	a	c	a	c	c	c	a	a	a	g
MH709327	5	a	c	g	c	t	c	g	a	t	a	a	a	a	g	g	a	a	t	t	c	t	a	t	g	c	g	g	c	t	g	c	a	t	t	g	a	g	g	c	t	t	g	a	c	t	t	t	g	t	c	a	t	t	g	a	c	a	g	t	a	a	c	c	a	a	c	a	c	c	c	a	a	a	g
MH709328	5	a	c	g	c	t	c	g	a	t	a	a	a	a	g	g	a	a	t	t	c	t	a	t	g	c	g	g	c	t	g	c	a	t	t	g	a	g	g	c	t	t	g	a	c	t	t	t	g	t	c	a	t	t	g	a	c	a	g	t	a	a	c	c	a	a	c	a	c	c	c	a	a	a	g
MH709330	5	a	c	g	c	g	c	g	a	t	a	a	a	a	g	g	a	a	t	t	c	t	a	t	g	c	g	g	c	t	g	c	a	t	t	g	a	g	g	c	t	t	g	a	c	t	t	t	g	t	c	a	t	t	g	a	c	a	g	t	a	a	c	c	a	a	c	a	c	c	c	a	a	a	g
MH709332	5	a	c	g	c	t	c	g	a	t	a	a	a	a	g	g	a	a	t	t	c	t	a	t	g	c	g	g	c	t	g	c	a	t	t	g	a	g	g	c	t	t	g	a	c	t	t	t	g	t	c	a	t	t	g	a	c	a	g	t	a	a	c	c	a	a	c	a	c	c	c	a	a	a	g
MH709334	5	a	c	g	c	t	c	g	a	t	a	a	a	a	g	g	a	a	t	t	c	t	a	t	g	c	g	g	c	t	g	c	a	t	t	g	a	g	g	c	t	t	g	a	c	t	t	t	g	t	c	a	t	t	g	a	c	a	g	t	a	a	c	c	a	a	c	a	c	c	c	a	a	a	g
MH709335	5	a	c	g	c	t	c	g	a	t	a	a	a	a	g	g	a	a	t	t	c	t	a	t	g	c	g	g	c	t	g	c	a	t	t	g	a	g	g	c	t	t	g	a	c	t	t	t	g	t	c	a	t	t	g	a	c	a	g	t	a	a	c	c	a	a	c	a	c	c	c	a	a	a	g
MH709336	5	a	c	g	c	t	c	g	a	t	a	a	a	a	g	g	a	a	t	t	c	t	a	t	g	c	g	g	c	t	g	c	a	t	t	g	a	g	g	c	t	t	g	a	c	t	t	t	g	t	c	a	t	t	g	a	c	a	g	t	a	a	c	c	a	a	c	a	c	c	c	a	a	a	g
MH709338	5	a	c	g	c	t	c	g	a	t	a	a	a	a	g	g	a	a	t	t	c	t	a	t	g	c	g	g	c	t	g	c	a	t	t	g	a	g	g	c	t	t	g	a	c	t	t	t	g	t	c	a	t	t	g	a	c	a	g	t	a	a	c	c	a	a	c	a	c	c	c	a	a	a	g
MH709340	5	a	c	g	c	t	c	g	a	t	a	a	a	a	g	g	a	a	t	t	c	t	a	t	g	c	g	g	c	t	g	c	a	t	t	g	a	g	g	c	t	t	g	a	c	t	t	t	g	t	c	a	t	t	g	a	c	a	g	t	a	a	c	c	a	a	c	a	c	c	c	a	a	a	g
MH709342	5	a	c	g	c	t	c	g	a	t	a	a	a	a	g	g	a	a	t	t	c	t	a	t	g	c	g	g	c	t	g	c	a	t	t	g	a	g	g	c	t	t	g	a	c	t	t	t	g	t	c	a	t	t	g	a	c	a	g	t	a	a	c	c	a	a	c	a	c	c	c	a	a	a	g
MH709343	5	a	c	g	c	t	c	g	a	t	a	a	a	a	g	g	a	a	t	t	c	t	a	t	g	c	g	g	c	t	g	c	a	t	t	g	a	g	g	c	t	t	g	a	c	t	t	t	g	t	c	a	t	t	g	a	c	a	g	t	a	a	c	c	a	a	c	a	c	c	c	a	a	a	g
MH709345	5	a	c	g	c	t	c	g	a	t	a	a	a	a	g	g	a	a	t	t	c	t	a	t	g	c	g	g	c	t	g	c	a	t	t	g	a	g	g	c	t	t	g	a	c	t	t	t	g	t	c	a	t	t	g	a	c	a	g	t	a	a	c	c	a	a	c	a	c	c	c	a	a	a	g
MH709347	5	a	c	g	c	t	c	g	a	t	a	a	a	a	g	g	a	a	t	t	c	t	a	t	g	c	g	g	c	t	g	t	a	t	t	g	a	g	g	c	t	t	g	a	c	t	t	t	g	t	c	a	t	t	g	a	c	a	g	t	a	a	c	c	a	a	c	a	c	c	c	a	a	a	g
MH709349	5	a	c	g	c	t	c	g	a	t	a	a	a	a	g	g	a	a	t	t	c	t	a	t	g	c	g	g	c	t	g	c	a	t	t	g	a	g	g	c	t	t	g	a	c	t	t	t	g	t	c	a	t	t	g	a	c	a	g	t	a	a	c	c	a	a	c	a	c	c	c	a	a	a	g
MH709350	5	a	c	g	c	t	c	g	a	t	a	a	a	a	g	g	a	a	t	t	c	t	a	t	g	c	g	g	c	t	g	c	a	t	t	g	a	g	g	c	t	t	g	a	c	t	t	t	g	t	c	a	t	t	g	a	c	a	g	t	a	a	c	c	a	a	c	a	c	c	c	a	a	a	g
MH709351	5	a	c	g	c	t	c	g	a	t	a	a	a	a	g	g	a	a	t	t	c	t	a	t	g	c	g	g	c	t	g	c	a	t	t	t	a	g	g	c	t	t	g	a	c	t	t	t	g	t	c	a	t	t	g	a	c	a	g	t	a	a	c	c	a	a	c	a	c	c	c	a	a	a	g
MH709353	5	a	t	g	c	t	c	g	a	t	a	a	a	a	g	g	a	a	t	t	c	t	a	t	g	c	g	g	c	t	g	c	a	t	t	g	a	g	g	c	t	t	g	a	c	t	t	t	g	t	c	a	t	t	g	a	c	a	g	t	a	a	c	c	a	a	c	a	c	c	c	a	a	a	g
MH709358	5	a	c	g	c	t	c	g	a	t	a	a	a	a	g	g	a	a	t	t	c	t	a	t	g	c	g	g	c	t	g	c	a	t	t	g	a	g	g	c	t	t	g	a	c	t	t	t	g	t	c	a	t	t	g	a	c	a	g	t	a	a	c	c	a	a	c	a	c	c	c	a	a	a	g
MH709360	5	a	c	g	c	t	c	g	a	t	a	a	a	a	g	g	a	a	t	t	c	t	a	t	g	c	g	g	c	t	g	c	a	t	t	g	a	g	g	c	t	t	g	a	c	t	t	t	g	t	c	a	t	t	g	a	c	a	g	t	a	a	c	c	a	a	c	a	c	c	c	a	a	a	g
MH709362	5	a	c	g	c	t	c	g	a	t	a	a	a	a	g	g	a	a	t	t	c	t	a	t	g	c	g	g	c	t	g	c	a	t	t	g	a	g	g	c	t	t	g	a	c	t	t	t	g	t	c	a	t	t	g	a	c	a	g	t	a	a	c	c	a	a	c	a	c	c	c	a	a	a	g
MH709365	5	a	c	g	c	t	c	g	a	t	a	a	a	a	g	g	a	a	t	t	c	t	a	t	g	c	g	g	c	t	g	c	a	t	t	g	a	g	g	c	t	t	g	a	c	t	t	t	g	t	c	a	t	t	g	a	c	a	g	t	a	a	c	c	a	a	c	a	c	c	c	a	a	a	g
MH709373	5	a	c	g	c	t	c	g	a	t	a	a	a	a	g	g	a	a	t	t	c	t	a	t	g	c	g	g	c	t	g	c	a	t	t	g	a	g	g	c	t	t	g	a	c	t	t	t	g	t	c	a	t	t	g	a	c	a	g	t	a	a	c	c	a	a	c	a	c	c	c	a	a	a	g
MH709374	5	a	c	g	c	t	c	g	a	t	a	a	a	a	g	g	a	a	t	t	c	t	a	t	g	c	g	g	c	t	g	c	a	t	t	g	a	g	g	c	t	t	g	a	c	t	t	t	g	t	c	a	t	t	g	a	c	a	g	t	a	a	c	c	a	a	c	a	c	c	c	a	a	a	g
KC112914	6	a	c	g	c	t	c	g	a	t	a	a	a	a	g	g	a	a	t	t	c	t	a	t	g	c	t	g	c	t	g	c	a	t	t	g	a	g	a	c	t	t	g	a	c	t	t	t	g	t	c	a	t	c	g	a	c	g	g	c	a	a	c	c	a	a	c	a	c	c	c	a	a	a	g
KP771914	6	a	c	g	c	t	c	g	a	t	a	a	a	a	g	g	a	a	t	t	c	t	a	t	g	c	t	g	c	t	g	c	a	t	t	g	a	g	a	c	t	t	g	a	c	t	t	t	g	t	c	a	t	c	g	a	c	g	g	c	a	a	c	c	a	a	c	a	c	c	c	a	a	a	g
KP702725	6	a	c	g	c	t	c	g	a	t	a	a	a	a	g	g	g	a	t	t	c	t	a	t	g	c	t	g	c	g	g	c	a	t	t	g	a	g	a	c	t	t	g	a	c	t	t	t	g	t	c	a	t	c	g	a	c	g	g	c	a	a	c	c	a	a	c	a	c	c	c	a	a	a	g
KP771890	9	a	c	g	c	t	c	g	a	t	a	a	a	a	g	g	a	a	t	t	c	t	a	t	g	c	t	g	c	t	g	c	a	t	t	g	a	g	g	c	t	t	g	a	c	t	t	t	g	t	c	a	t	c	g	a	c	g	g	c	a	a	c	c	a	a	c	a	c	c	c	a	a	a	g
JN704710	9	a	c	g	c	t	c	g	a	t	a	a	a	a	g	g	a	a	t	t	c	t	a	t	g	c	t	g	c	t	g	c	a	t	t	g	a	g	g	c	t	t	g	a	c	t	t	t	g	t	c	a	t	c	g	a	c	g	g	c	a	a	c	c	a	a	c	a	c	c	c	a	a	a	g
KY037798	9	a	c	g	c	t	c	g	a	t	a	a	a	a	g	g	a	a	t	t	c	t	a	t	g	c	t	g	c	t	g	c	a	t	t	g	a	g	g	c	t	t	g	a	c	t	t	t	g	t	c	a	t	c	g	a	c	g	g	c	a	a	c	c	a	a	c	a	c	c	c	a	a	a	g
MH709312	9	a	c	g	c	t	c	g	a	t	a	a	a	a	g	g	a	a	t	t	c	t	a	t	g	c	t	g	c	t	g	c	a	t	t	g	a	g	g	c	t	t	g	a	c	t	t	t	g	t	c	a	t	c	g	a	c	g	g	c	a	a	c	c	a	a	c	a	c	c	c	a	a	a	g
MH709352	9	a	c	g	c	t	c	g	a	t	a	a	a	a	g	g	a	a	t	t	c	t	a	t	g	c	t	g	c	t	g	c	a	t	t	g	a	g	g	c	t	t	g	a	c	t	t	t	g	t	c	a	t	c	g	a	c	g	g	c	a	a	c	c	a	a	c	a	c	c	c	a	a	a	g
AB097933	Vac	a	c	g	c	t	c	g	a	t	a	a	a	a	g	g	a	a	t	t	c	t	a	t	g	c	t	g	c	t	g	c	a	t	t	g	g	g	g	c	t	c	g	a	c	t	t	t	g	t	c	a	t	c	g	a	c	g	g	c	a	a	t	c	a	a	c	a	c	c	c	a	a	a	g
JN704698	Vac	a	c	g	c	t	c	g	a	t	a	a	a	a	g	g	a	a	t	t	c	t	a	t	g	c	t	g	c	t	g	c	a	t	t	g	g	g	g	c	t	c	g	a	c	t	t	t	g	t	c	a	t	c	g	a	c	g	g	c	a	a	t	c	a	a	c	a	c	c	c	a	a	a	g
JN704697	Vac	a	c	g	c	t	c	g	a	t	a	a	a	a	g	g	a	a	t	t	c	t	a	t	g	c	t	g	c	t	g	c	g	t	t	g	g	g	g	c	t	c	g	g	c	t	t	t	g	t	c	a	t	c	g	a	c	g	g	c	a	a	t	c	a	a	c	a	c	c	c	a	a	a	g
DQ008355	Vac	a	c	g	c	t	c	g	a	t	a	a	a	a	g	g	a	a	t	t	c	t	a	t	g	c	t	g	c	t	g	c	r	t	t	g	g	g	g	c	t	c	g	a	c	t	t	t	g	t	c	a	t	c	g	a	c	g	g	c	a	a	t	c	a	a	c	a	c	c	c	a	a	a	g
JF306641	Vac	a	c	g	c	t	c	g	a	t	a	a	a	a	g	g	a	a	t	t	c	t	a	t	g	c	t	g	c	t	g	c	g	t	t	g	g	g	g	c	t	c	g	a	c	t	t	t	g	t	c	a	t	c	g	a	c	g	g	c	a	a	t	c	a	a	c	a	c	c	c	a	a	a	g
AB097932	Vac	a	c	g	c	t	c	g	a	t	a	a	a	a	g	g	a	a	t	t	c	t	a	t	g	c	t	g	c	t	g	c	g	t	t	g	g	g	g	c	t	c	g	a	c	t	t	t	g	t	c	a	t	c	g	a	c	g	g	c	a	a	t	c	a	a	c	a	c	c	c	a	a	a	g
DQ008354	Vac	a	c	g	c	t	c	g	a	t	a	a	a	a	g	g	a	a	t	t	c	t	a	t	g	c	t	g	c	t	g	c	g	t	t	g	g	g	g	c	t	c	g	a	c	t	t	t	g	t	c	a	t	c	g	a	c	g	g	c	a	a	t	c	a	a	c	a	c	c	c	a	a	a	g
KU926312	Vac	a	c	g	c	t	c	g	a	t	a	a	a	a	g	g	a	a	t	t	c	t	a	t	g	c	t	g	c	t	g	c	g	t	t	g	g	g	g	c	t	c	g	a	c	t	t	t	g	t	c	a	t	c	g	a	c	g	g	c	a	a	t	c	a	a	c	a	c	c	c	a	a	a	g
KU926313	Vac	a	c	g	c	t	c	g	a	t	a	a	a	a	g	g	a	a	t	t	c	t	a	t	g	c	t	g	c	t	g	c	g	t	t	g	g	g	g	c	t	c	g	a	c	t	t	t	g	t	c	a	t	c	g	a	c	g	g	c	a	a	t	c	a	a	c	a	c	c	c	a	a	a	g
KU926314	Vac	a	c	g	c	t	c	g	a	t	a	a	a	a	g	g	a	a	t	t	c	t	a	t	g	c	t	g	c	t	g	c	g	t	t	g	g	g	g	c	t	c	g	a	c	t	t	t	g	t	c	a	t	c	g	a	c	g	g	c	a	a	t	c	a	a	c	a	c	c	c	a	a	a	g
KF558371	Vac	a	c	g	c	t	c	g	a	t	a	a	a	a	g	g	a	a	t	g	c	t	a	t	g	c	t	g	c	t	g	c	a	t	t	g	g	g	g	c	t	c	g	a	c	t	t	t	g	t	c	a	t	c	g	a	c	g	g	c	a	a	t	c	a	a	c	a	c	c	c	a	a	a	g
KF558372	Vac	a	c	g	c	t	c	g	a	t	a	a	a	a	g	g	a	a	t	t	c	t	a	t	g	c	t	g	c	t	g	c	g	t	t	g	g	g	g	c	t	c	g	a	c	t	t	t	g	t	c	a	t	c	g	a	c	g	g	c	a	a	t	c	a	a	c	a	c	c	c	a	a	a	g
KF558373	Vac	a	c	g	c	t	c	g	a	t	a	a	a	a	g	g	a	a	t	t	c	t	a	t	g	c	t	g	c	t	g	c	a	t	t	g	g	g	g	c	t	c	g	a	c	t	t	t	g	t	c	a	t	c	g	a	c	g	g	c	a	a	t	c	a	a	c	a	c	c	c	a	a	a	g
KF558374	Vac	a	c	g	c	t	c	g	a	t	a	a	a	a	g	g	a	a	t	t	c	t	a	t	g	c	t	g	c	t	g	c	a	t	t	g	g	g	g	c	t	c	g	a	c	t	t	t	g	t	c	a	t	c	g	a	c	g	g	c	a	a	t	c	a	a	c	a	c	c	c	a	a	a	g
KF558375	Vac	a	c	g	c	t	c	g	a	t	a	a	a	a	g	g	a	a	t	t	c	t	a	t	g	c	t	g	c	t	g	c	a	t	t	g	g	g	g	c	t	c	g	a	c	t	t	t	g	t	c	a	t	c	g	a	c	g	g	c	a	a	t	c	a	a	c	a	c	c	c	a	a	a	g
KF558376	Vac	a	c	g	c	t	c	g	a	t	a	a	a	g	g	g	a	a	t	t	c	t	a	t	g	c	t	g	c	t	g	c	a	t	t	g	g	g	g	c	t	c	g	a	c	t	t	t	g	t	c	a	t	c	g	a	c	g	g	c	a	a	t	c	a	a	c	a	c	c	c	a	a	a	g
KF558377	Vac	a	c	g	c	t	c	g	a	t	a	a	a	a	g	g	a	a	t	t	c	t	a	t	g	c	t	g	c	t	g	c	a	t	t	g	g	g	g	c	t	c	g	a	c	t	t	t	g	t	c	a	t	c	g	a	c	g	g	c	a	a	t	t	a	a	c	a	c	c	c	a	a	a	g
KF558378	Vac	a	c	g	c	t	c	g	a	t	a	a	a	a	g	g	a	a	t	t	c	t	a	t	g	c	t	g	c	t	g	c	a	t	t	g	g	g	g	c	t	c	g	a	c	t	t	t	g	t	c	a	t	c	g	a	c	g	g	c	a	a	t	c	a	a	c	a	c	c	c	a	a	a	g
KF558379	Vac	a	c	g	c	t	c	g	a	t	a	t	a	a	g	g	a	a	t	t	c	t	a	t	g	c	t	g	c	t	g	c	a	t	t	g	g	g	g	c	t	c	g	a	c	t	t	t	g	t	c	a	t	c	g	a	c	g	g	c	a	a	t	c	a	a	c	a	c	c	c	a	a	a	g
KF558380	Vac	a	c	g	c	t	c	g	a	t	a	a	a	a	g	g	a	a	t	t	c	t	a	t	g	c	t	g	c	t	g	c	g	t	t	g	g	g	g	c	t	c	g	a	c	t	t	t	g	t	c	a	t	c	g	a	c	g	g	c	a	a	t	c	a	a	c	a	c	c	c	a	a	a	g
KF558381	Vac	a	c	g	c	t	c	g	a	t	a	a	a	a	g	g	a	a	t	t	c	t	a	t	g	c	t	g	c	t	g	c	a	t	t	g	g	g	g	c	t	c	g	a	c	t	t	t	g	t	c	a	t	c	g	a	c	g	g	c	a	a	t	c	a	a	c	a	c	c	c	a	a	a	g
KF558382	Vac	a	c	g	c	t	c	g	a	t	a	a	g	a	g	g	a	a	t	t	c	t	a	t	g	c	t	g	c	t	g	c	a	t	t	g	g	g	g	c	t	c	g	a	c	t	t	t	g	t	c	a	t	c	g	a	c	g	g	c	a	a	t	c	a	a	c	a	c	c	c	a	a	a	g
KF558383	Vac	a	c	g	c	t	c	g	a	t	a	a	a	a	g	g	a	a	t	t	c	t	a	t	g	c	t	g	c	t	g	c	a	t	t	g	g	g	g	c	t	c	g	a	c	t	t	t	g	t	c	a	t	c	g	a	c	g	g	c	a	a	t	c	a	a	c	a	c	c	c	a	a	a	g
KF558384	Vac	a	c	g	c	t	c	g	a	t	a	a	a	a	g	g	a	a	t	t	c	t	a	t	g	c	t	g	c	t	g	c	a	t	t	g	g	g	g	c	t	c	g	a	c	t	t	t	g	t	c	a	t	c	g	a	c	g	g	c	a	a	t	c	a	a	c	a	c	c	c	a	a	a	g
KF558385	Vac	a	c	g	c	t	c	g	a	t	a	a	a	a	g	g	a	a	t	t	c	t	a	t	g	c	t	g	c	t	g	c	a	t	t	g	g	g	g	c	t	c	g	a	c	t	t	t	g	t	c	a	t	c	g	a	c	g	g	c	a	a	t	c	a	a	c	a	c	c	c	a	a	a	g
KF558386	Vac	a	c	g	c	t	c	g	a	t	a	a	a	a	g	g	a	a	t	t	c	t	a	t	g	c	t	g	c	t	g	c	a	t	t	g	g	g	g	c	t	c	g	a	c	t	t	t	g	t	c	a	t	c	g	a	c	g	g	c	a	a	t	c	a	a	c	a	c	c	c	a	a	a	g
KF558387	Vac	a	c	g	c	t	c	g	a	t	a	a	a	a	g	g	a	a	t	t	c	t	a	t	g	c	t	g	c	t	g	c	g	t	t	g	g	g	g	c	t	c	g	a	c	t	t	t	g	t	c	a	t	c	g	a	c	g	g	c	a	a	t	c	a	a	c	a	c	c	c	a	a	a	g
KF558388	Vac	a	c	g	c	t	c	g	a	t	a	a	a	a	g	g	a	a	t	t	c	t	a	t	g	c	t	g	c	t	g	c	a	t	t	g	g	g	g	c	t	c	g	a	c	t	t	t	g	t	c	a	t	c	g	a	c	g	g	c	a	a	t	c	a	a	c	a	c	c	c	a	a	a	g
KF558389	Vac	a	c	g	c	t	c	g	a	t	a	a	a	a	g	g	a	a	t	t	c	t	a	t	g	c	t	g	c	t	g	c	g	t	t	g	g	g	g	c	t	c	g	a	c	t	t	t	g	t	c	a	t	c	g	a	c	g	g	c	a	a	t	c	a	a	c	a	c	c	c	a	a	a	g
KF558390	Vac	a	c	g	c	t	c	g	a	t	a	a	a	a	g	g	a	a	t	t	c	t	a	t	g	c	t	g	c	t	g	c	a	t	t	g	g	g	g	c	t	c	g	a	c	t	t	t	g	t	c	a	t	c	g	a	c	g	g	c	a	a	t	c	a	a	c	a	c	c	c	a	a	a	g
KF558391	Vac	a	c	g	c	t	c	g	a	t	a	a	a	a	g	g	a	a	c	t	c	t	a	t	g	c	t	g	c	t	g	c	a	t	t	g	g	g	g	c	t	c	g	a	c	t	t	t	g	t	c	a	t	c	g	a	c	g	g	c	a	a	t	c	a	a	c	a	c	c	c	a	a	a	g
KF811485	Vac	a	c	g	c	t	c	g	a	t	a	a	a	a	g	g	a	a	t	t	c	t	a	t	g	c	t	a	c	t	g	c	g	t	t	g	g	g	g	c	t	c	g	a	c	t	t	t	g	t	c	a	t	c	g	a	c	g	g	c	a	a	t	c	a	a	c	a	c	c	c	a	a	a	g
KF853225	Vac	a	c	g	c	t	c	g	a	t	a	a	a	a	g	g	a	a	t	t	c	t	a	t	g	c	t	g	c	t	g	c	a	t	t	g	g	g	g	c	t	c	g	a	c	t	t	t	g	t	c	a	t	c	g	a	c	g	g	c	a	a	t	c	a	a	c	a	c	c	c	a	a	a	g
KF853226	Vac	a	c	g	c	t	c	g	a	t	a	a	a	a	g	g	a	a	t	t	c	t	a	t	g	c	t	g	c	t	g	c	a	t	t	g	g	g	g	c	t	c	g	a	c	t	t	t	g	t	c	a	t	c	g	a	c	g	g	c	a	a	t	c	a	a	c	a	c	c	c	a	a	a	g
KF853227	Vac	a	c	g	c	t	c	g	a	t	a	a	a	a	g	g	a	a	t	g	c	t	a	t	g	c	t	g	c	t	g	c	a	t	t	g	g	g	g	c	t	c	g	a	c	t	c	t	g	t	c	a	t	c	g	a	c	g	g	c	a	a	t	c	a	a	c	t	c	c	c	a	a	a	g
KF853228	Vac	a	c	g	c	t	c	g	a	t	a	a	a	a	g	g	a	a	c	t	c	t	a	t	g	c	t	g	c	t	g	c	a	t	t	g	g	g	g	c	t	c	g	a	c	t	t	t	g	t	c	a	t	c	g	a	c	g	g	c	a	a	t	c	a	a	c	a	c	c	c	a	a	a	g
KF853229	Vac	a	c	g	c	t	c	g	a	t	a	a	a	a	g	g	a	a	t	t	c	t	a	t	g	c	t	g	c	t	g	c	a	t	t	g	g	g	g	c	t	c	g	a	c	t	t	t	g	t	c	a	t	c	g	a	c	g	g	c	a	a	t	c	a	a	c	a	c	c	c	a	a	a	g
KF853230	Vac	a	c	g	c	t	c	g	a	t	a	a	a	a	g	g	a	a	t	t	c	t	a	t	g	c	t	g	c	t	g	c	g	t	t	g	g	g	g	c	t	c	g	a	c	t	t	t	g	t	c	a	t	c	g	a	c	g	g	c	a	a	t	c	a	a	c	a	c	c	c	a	a	a	g
KF853231	Vac	a	c	g	c	t	c	g	a	t	a	a	a	a	g	g	a	a	t	t	c	t	a	t	g	c	t	g	c	t	g	c	a	t	t	g	g	g	g	c	t	c	g	a	c	t	t	t	g	t	c	a	t	c	g	a	c	g	g	c	a	a	t	c	a	a	c	a	c	c	c	a	a	a	g
KF853232	Vac	a	c	g	c	t	c	g	a	t	a	a	a	a	g	g	a	a	t	t	c	t	a	t	g	c	t	g	c	t	g	c	a	t	t	g	g	g	g	c	t	c	g	a	c	t	t	t	g	t	c	a	t	c	g	a	c	g	g	c	a	a	t	c	a	a	c	a	c	c	c	a	a	a	g
KF853233	Vac	a	c	g	c	t	c	g	a	t	a	a	a	a	g	g	a	a	t	t	c	t	a	t	g	c	t	g	c	t	g	c	a	t	t	g	g	g	g	c	t	c	g	a	c	t	t	t	g	t	c	a	t	c	g	a	c	g	g	c	a	a	t	c	a	a	c	a	c	c	c	a	a	a	g
KF853234	Vac	a	c	g	c	t	c	g	a	t	a	a	a	a	g	g	a	a	t	g	c	t	a	t	g	c	t	g	c	t	g	c	a	t	t	g	g	g	g	c	t	c	g	a	c	t	t	t	g	t	c	a	t	c	g	a	c	g	g	c	a	a	t	c	a	a	c	a	c	c	c	a	a	a	g
KF853235	Vac	a	c	g	c	t	c	g	a	t	a	a	a	g	g	g	a	a	t	t	c	t	a	t	g	c	t	g	c	t	g	c	a	t	t	g	g	g	g	c	t	c	g	a	c	t	t	t	g	t	c	a	t	c	g	a	c	g	g	c	a	a	t	c	a	a	c	a	c	c	c	a	a	a	g
MF898328	Vac	a	c	g	c	t	c	g	a	t	a	a	a	a	g	g	a	a	t	t	c	t	a	t	g	c	t	g	c	t	g	c	g	t	t	g	g	g	g	c	t	c	g	r	c	t	t	t	g	t	c	a	t	c	g	a	c	g	g	c	a	a	t	c	a	a	c	a	c	c	c	a	a	a	g
JN704709	VIII	a	c	g	c	t	c	g	a	t	a	a	a	a	g	g	a	a	t	t	c	t	a	t	g	t	t	g	c	t	g	c	a	t	t	g	a	g	a	c	t	t	g	a	c	t	t	t	g	t	c	a	t	c	g	a	c	g	g	c	a	a	c	c	a	a	c	a	c	c	c	a	a	a	g

Supplementary Table 3: 2880 SN

GB-ID	Clade markers																					SD14	SD14																																																			4					
	Position in Dumas	65610	65669	65683	65751	65759	65762	65768	65858	65878	66003	66063	66073	66085	66106	66112	66247	66288	66304	66311	66350	66366	66367	66385	66450	66499	66568	66623	66646	66849	66879	66880	66947	66951	67025	67028	67114	67146	67244	67327	67331	67341	67348	67429	67477	67573	67603	67622	67724	67847	67883	67963	67999	68050	68101	68140	68150	68172	68212	68254	68331	68344	68371	68518	68662	68693	68818	69034	69043	69067	69072	69089	69124	69148	69211
AY548170	1	a	t	c	g	c	c	t	c	t	g	t	t	a	t	t	c	g	g	g	c	a	a	c	t	t	g	a	g	t	t	a	t	a	t	t	t	a	g	t	t	c	g	t	t	t	a	c	a	c	g	t	a	c	t	a	a	g	c	c	g	t	c	g	t	c	t	a	g	a	a	a	g	t	g
AY548171	1	a	t	c	g	c	c	t	c	t	g	t	t	a	t	t	c	g	g	g	c	a	a	c	t	t	g	a	g	t	t	a	t	a	t	t	t	a	g	t	t	c	g	t	t	t	a	c	a	c	g	t	a	c	t	a	a	g	c	c	g	t	c	g	t	c	t	a	g	a	a	a	g	t	g
DQ479953	1	a	t	c	g	c	c	t	c	t	g	t	t	a	t	t	c	g	g	g	c	a	a	c	t	t	g	a	g	t	t	a	t	a	t	t	t	a	g	t	t	c	g	t	t	t	a	c	a	c	g	t	a	c	t	a	a	g	c	c	g	t	c	g	t	c	t	a	g	a	a	a	g	t	g
DQ479954	1	a	t	c	g	c	c	t	c	t	g	t	t	a	t	t	c	g	g	g	c	a	a	c	t	t	g	a	g	t	t	a	t	a	t	t	t	a	g	t	t	c	g	t	t	t	a	c	a	c	g	t	a	c	t	a	a	g	c	c	g	t	c	g	t	c	t	a	g	a	a	a	g	t	g
DQ479958	1	a	t	c	g	c	c	t	c	t	g	t	t	a	t	t	c	g	g	g	c	a	a	c	t	t	g	a	g	t	t	a	t	a	t	t	t	a	g	t	t	c	g	t	t	t	a	c	a	c	g	t	a	c	t	a	a	g	c	c	g	t	c	g	t	c	t	a	g	a	a	a	g	t	g
DQ479959	1	a	t	c	g	c	c	t	c	t	g	t	t	a	t	t	c	g	g	g	c	a	a	c	t	t	g	a	g	t	t	a	t	a	t	t	t	a	g	t	t	c	g	t	t	t	a	c	a	c	g	t	a	c	t	a	a	g	c	c	g	t	c	g	t	c	t	a	g	a	a	a	g	t	g
DQ479961	1	a	t	c	g	c	c	t	c	t	g	t	t	a	t	t	c	g	g	g	c	a	a	c	t	t	g	a	g	t	t	a	t	a	t	t	t	a	g	t	t	c	g	t	t	t	a	c	a	c	g	t	a	c	t	a	a	g	c	c	g	t	c	a	t	c	t	a	g	a	a	a	g	t	g
DQ479962	1	a	t	c	g	c	c	t	c	t	g	t	t	a	t	t	c	g	g	g	c	a	a	c	t	t	g	a	g	t	t	a	t	a	t	t	t	a	g	t	t	c	g	t	t	t	a	c	a	c	g	t	a	c	t	a	a	g	c	c	g	t	c	a	t	c	t	a	g	a	a	a	g	t	g
DQ479963	1	a	t	c	g	c	c	t	c	t	g	t	t	a	t	t	c	g	g	g	c	a	a	c	t	t	g	a	g	t	t	a	t	a	t	t	t	a	g	t	t	c	g	t	t	t	a	c	a	c	g	t	a	c	t	a	a	g	c	c	g	t	c	a	t	c	t	a	g	a	a	a	g	t	g
DQ674250	1	a	t	c	g	c	c	t	c	t	g	t	t	a	t	t	c	g	g	g	c	a	a	c	t	t	g	a	t	t	t	a	t	g	t	t	t	a	g	t	t	c	g	t	t	t	a	c	a	c	g	t	a	c	t	a	a	a	c	c	g	t	c	g	t	c	c	a	g	a	a	a	g	t	g
KX262864	1	a	t	c	g	c	c	t	c	t	g	t	t	a	t	t	c	g	g	g	c	a	a	c	t	t	g	a	g	t	t	a	t	a	t	t	t	a	g	t	t	c	g	t	t	t	a	c	a	c	g	t	a	c	t	a	a	g	c	c	g	t	c	g	t	c	t	a	g	a	a	a	g	t	g
KX262865	1	a	t	c	g	c	c	t	c	t	g	t	t	a	t	t	c	g	g	g	c	a	a	c	t	t	g	a	g	t	t	a	t	a	t	t	t	a	g	t	t	c	g	t	t	t	a	c	a	c	g	t	a	c	t	a	a	g	c	c	g	t	c	g	t	c	t	a	g	a	a	a	g	t	g
KX262866	1	a	t	c	g	c	c	t	c	t	g	t	t	a	t	t	c	g	g	g	c	a	a	c	t	t	g	a	g	t	t	a	t	a	t	t	t	a	g	t	t	c	g	t	t	t	a	c	a	c	g	t	a	c	t	a	a	g	c	c	g	t	c	g	t	c	t	a	g	a	a	a	g	t	g
EU154348	1	a	t	c	g	c	c	t	c	t	g	t	t	a	t	t	c	g	g	g	c	a	a	c	t	t	g	a	g	t	t	a	t	a	t	t	t	a	g	t	t	c	g	t	t	t	a	c	a	c	g	t	a	c	t	a	a	g	c	c	g	t	c	g	t	c	t	a	g	a	a	a	g	t	g
JN704690	1	a	t	c	g	c	c	t	c	t	g	t	t	a	t	t	c	g	g	g	c	a	a	c	t	t	g	a	g	t	t	a	t	a	t	t	t	a	g	t	t	c	g	t	t	t	a	c	a	c	g	t	a	c	t	a	a	g	c	c	g	t	c	g	t	c	t	a	g	a	a	a	g	t	g
JN704691	1	a	t	c	g	c	c	t	c	t	g	t	t	a	t	t	c	g	g	g	c	a	a	c	t	t	g	a	g	t	t	a	t	a	t	t	t	a	g	t	t	c	g	t	t	t	a	c	a	c	g	t	a	c	t	a	a	g	c	c	g	t	c	g	t	c	t	a	g	a	a	a	g	t	g
JN704692	1	a	t	c	g	c	c	t	c	t	g	t	t	a	t	t	c	g	g	g	c	a	a	c	t	t	g	a	g	t	t	a	t	a	t	t	t	a	g	t	t	c	g	t	t	t	a	c	a	c	g	t	a	c	t	a	a	g	c	c	g	t	c	g	t	c	t	a	g	a	a	a	g	t	g
JN704693	1	a	t	c	g	c	c	t	c	t	g	t	t	a	t	t	c	g	g	g	c	a	a	c	t	t	g	a	g	t	t	a	t	a	t	t	t	a	g	t	t	c	g	t	t	t	a	c	a	c	g	t	g	c	t	a	a	g	c	c	g	t	c	g	t	c	t	a	g	a	a	a	g	t	g
JN704694	1	a	t	c	g	c	c	t	c	t	g	t	t	a	t	t	c	g	g	g	c	a	a	c	t	t	g	a	g	t	t	a	t	a	t	t	t	a	g	t	t	c	g	t	t	t	a	c	a	c	g	t	a	t	t	a	a	g	c	c	g	t	c	g	t	c	t	a	g	a	a	a	g	t	g
JN704695	1	a	t	c	g	c	c	t	c	t	g	t	t	a	t	t	c	g	g	g	c	a	a	c	t	t	g	a	g	t	t	a	t	a	t	t	t	a	g	t	t	c	g	t	t	t	a	c	a	c	g	t	a	c	t	a	a	g	c	c	g	t	c	g	t	c	t	a	g	a	a	a	g	t	g
JN704696	1	a	c	c	g	c	c	t	c	t	g	t	t	a	t	t	c	g	g	g	t	a	a	c	t	t	g	a	g	t	t	a	t	a	t	t	t	a	g	t	t	c	g	t	t	t	a	c	a	c	g	t	a	c	t	a	a	g	c	c	g	t	c	g	t	c	t	a	g	a	a	a	g	t	g
NC001348	1	a	c	c	g	c	c	t	c	t	g	t	t	a	t	t	c	g	g	g	c	a	a	c	t	t	g	a	g	t	c	a	t	a	t	t	t	a	g	t	t	c	g	t	t	t	a	c	a	c	g	t	a	c	t	a	a	g	c	c	g	t	c	g	t	c	t	a	g	a	a	a	g	t	g
KP771892	1	a	t	c	g	c	c	t	c	t	g	t	t	a	t	t	c	g	g	g	c	a	a	c	t	t	g	a	g	t	t	a	t	a	t	t	t	a	g	t	t	c	g	t	t	t	a	c	a	c	g	t	a	c	t	a	a	g	c	c	g	t	c	g	t	c	t	a	g	a	a	a	g	t	g
KP771894	1	a	t	c	g	c	c	t	c	t	g	t	t	a	t	t	c	g	g	g	c	a	a	c	t	t	g	a	g	t	t	a	t	a	t	t	t	a	g	t	t	c	g	t	t	t	a	c	a	c	g	t	a	c	t	a	a	g	c	c	g	t	c	g	t	c	t	a	g	a	a	a	g	t	g
KP771900	1	a	t	c	g	c	c	t	c	t	g	t	t	a	t	t	c	g	g	g	c	a	a	c	t	t	g	a	g	t	t	a	t	a	t	t	t	a	g	t	t	c	g	t	t	t	a	c	a	c	g	t	a	c	t	a	a	g	c	c	g	t	c	g	t	c	t	a	g	a	a	a	g	t	g
KP771901	1	a	t	c	g	c	c	t	c	t	g	t	t	a	t	t	c	g	g	g	c	a	a	c	t	t	g	a	g	t	t	a	t	a	t	t	t	a	g	t	t	c	g	t	t	t	a	c	a	c	g	t	a	c	t	a	a	g	c	c	g	t	c	g	t	c	t	a	g	a	a	a	g	t	g
KP771902	1	a	t	c	g	c	c	t	c	t	g	t	t	a	t	t	c	g	g	g	c	a	a	c	t	t	g	a	g	t	t	a	t	a	t	t	t	a	g	t	t	c	g	t	t	t	a	c	a	c	g	t	a	c	t	a	a	g	c	c	g	t	c	g	t	c	t	a	g	a	a	a	g	t	g
KP771908	1	a	t	c	g	c	c	t	c	t	g	t	t	a	t	t	c	g	g	g	c	a	a	c	t	t	g	a	g	t	t	a	t	a	t	t	t	a	g	t	t	c	g	t	t	t	a	c	a	c	g	t	a	c	t	a	a	g	c	c	g	t	c	g	t	c	t	a	g	a	a	a	g	t	g
KP771912	1	a	t	c	g	c	c	t	c	t	g	t	t	a	t	t	c	g	g	g	c	a	a	c	t	t	g	a	g	t	t	a	t	a	t	t	t	a	g	t	t	c	g	t	t	t	a	c	a	c	g	t	a	c	t	a	a	g	c	c	g	t	c	g	t	c	t	a	g	a	a	a	g	t	g
KP771913	1	a	t	c	g	c	c	t	c	t	g	t	t	a	t	t	c	g	g	g	c	a	a	c	t	t	g	a	g	t	t	a	t	a	t	t	t	a	g	t	t	c	g	t	t	t	a	c	a	c	g	t	a	c	t	a	a	g	c	c	g	t	c	g	t	c	t	a	g	a	a	a	g	t	g
KP771917	1	a	t	c	g	c	c	t	c	t	a	t	t	a	t	t	c	g	g	g	c	a	a	c	t	t	g	a	g	t	t	a	t	a	t	t	t	a	g	t	t	c	g	t	t	t	a	c	a	c	g	t	a	c	t	a	a	g	c	c	g	t	c	g	t	c	t	a	g	a	a	a	g	t	g
KP771918	1	a	t	c	g	c	c	t	c	t	g	t	t	a	t	t	c	g	g	g	c	a	a	c	t	t	g	a	g	t	t	a	t	a	t	t	t	a	g	t	t	c	g	t	t	t	a	c	a	c	g	t	a	c	t	a	a	g	c	c	g	t	c	g	t	c	t	a	g	a	a	a	g	t	g
KP771920	1	a	t	c	g	c	c	t	c	t	g	t	t	a	t	t	c	g	g	g	c	a	a	c	t	t	g	a	g	t	t	a	t	a	t	t	t	a	g	t	t	c	g	t	t	t	a	c	a	c	g	t	a	c	t	a	a	g	c	c	g	t	c	g	t	c	t	a	g	a	a	a	g	t	g
MH709361	1	a	t	c	g	c	c	t	c	t	g	t	t	a	t	t	c	g	g	g	c	a	a	c	t	t	g	a	g	t	t	a	t	a	t	t	t	a	g	t	t	c	g	t	t	t	a	c	a	c	g	t	a	c	t	a	a	g	c	c	g	t	c	g	t	c	t	a	g	a	a	a	g	t	g
KU926318	2	a	t	c	g	c	c	t	c	t	g	t	t	a	t	t	t	g	g	g	c	a	a	c	t	t	g	a	t	t	t	a	t	a	t	t	c	a	g	t	t	c	g	t	t	t	a	c	a	c	g	t	a	c	t	a	a	a	c	c	g	t	c	g	t	c	t	a	g	a	a	a	g	t	g
KU926319	2	a	t	c	g	c	c	c	c	t	g	t	t	a	t	t	t	g	g	g	c	a	a	c	t	t	g	a	t	t	t	a	t	g	t	t	c	a	g	t	t	c	g	t	t	t	a	c	a	c	g	t	a	c	t	a	a	a	c	c	g	t	c	g	t	c	t	a	g	a	a	a	g	t	g
KU926320	2	a	t	c	g	c	c	t	c	t	g	t	t	a	t	t	c	g	g	g	c	a	a	c	t	t	g	a	t	t	t	a	t	a	t	t	t	a	g	t	t	c	g	t	t	t	a	c	a	c	g	t	a	c	t	a	a	a	c	c	g	t	c	g	t	c	t	a	g	a	a	a	g	t	g
KU926321	2	a	t	c	g	c	c	t	c	t	g	t	t	a	t	t	c	g	g	g	c	a	a	c	t	t	g	a	t	t	t	a	t	a	t	t	t	a	g	t	t	c	g	t	t	t	a	c	a	c	g	t	a	c	t	a	a	a	c	c	g	t	c	g	t	c	t	a	g	a	a	a	g	t	g
KU926322	2	a	t	c	g	c	c	t	c	t	g	t	t	a	t	t	c	g	g	g	c	a	a	c	t	t	g	a	g	t	t	a	t	a	t	t	t	a	g	t	t	c	g	t	t	t	a	c	a	c	g	t	a	c	t	a	a	g	c	c	g	t	c	g	t	c	t	a	g	a	a	a	g	t	g
KC847290	2	a	t	c	g	c	c	t	c	t	g	t	t	a	t	t	c	g	g	g	c	a	a	c	t	t	g	a	t	t	t	a	t	a	t	t	t	a	g	t	t	c	g	t	t	t	a	c	a	c	g	t	a	c	t	a	a	a	c	c	g	t	c	g	t	c	t	a	g	a	a	a	g	t	g
KJ767491	2	a	t	c	g	c	c	t	c	t	g	t	t	a	t	t	c	g	g	g	c	a	a	c	t	t	g	a	t	t	t	a	t	a	t	t	t	a	g	t	t	c	g	t	t	t	a	c	a	c	g	t	a	c	t	a	a	a	c	c	g	t	c	g	t	c	t	a	g	a	a	a	g	t	g
KJ767492	2	a	t	c	g	c	c	t	c	t	g	t	t	a	t	t	t	g	g	g	c	a	a	c	t	t	g	a	t	t	t	a	t	a	t	t	c	a	g	t	t	c	g	t	t	t	a	c	a	c	g	t	a	c	t	a	a	a	c	c	g	t	c	g	t	c	t	a	g	a	a	a	g	t	g
KJ808816	2	a	t	c	g	c	c	t	c	t	g	t	t	a	t	t	c	g	g	g	c	a	a	c	t	t	g	a	t	t	t	a	t	a	t	t	t	a	g	t	t	c	g	t	t	t	a	c	a	c	g	t	a	c	t	a	a	a	c	c	g	t	c	g	t	c	t	a	g	a	a	a	g	t	g
KP771925	2	a	t	c	g	c	c	t	c	t	g	t	t	a	t	t	c	g	g	g	c	a	a	c	t	t	g	a	t	t	t	a	t	a	t	t	t	a	g	t	t	c	g	t	t	t	a	c	a	c	g	t	a	c	t	a	a	a	c	c	g	t	c	g	t	c	t	a	g	a	a	a	g	t	g
KU926315	2	a	t	c	g	c	c	t	c	t	g	t	t	a	t	t	c	g	g	g	c	a	a	c	t	t	g	a	t	t	t	a	t	a	t	t	t	a	g	t	t	c	g	t	t	t	a	c	a	c	g	t	a	c	t	a	a	a	c	c	g	t	c	g	t	c	t	a	g	a	a	a	g	t	g
KU926316	2	a	t	c	g	c	c	t	c	t	g	t	t	a	t	t	c	g	g	g	c	a	a	c	t	t	g	a	t	t	t	a	t	a	t	t	t	a	g	t	t	c	g	t	t	t	a	c	a	c	g	t	a	c	t	a	a	a	c	c	g	t	c	g	t	c	t	a	g	a	a	a	g	t	g
KU926317	2	a	t	c	g	c	c	t	c	t	g	t	t	a	t	t	c	g	g	g	c	a	a	c	t	t	g	a	t	t	t	a	t	a	t	t	t	a	g	t	t	c	g	t	t	t	a	c	a	c	g	t	a	c	t	a	a	a	c	c	g	t	c	g	t	c	t	a	g	a	a	a	g	t	g
KY037796	2	a	t	c	g	c	c	t	c	t	g	t	t	a	t	t	c	g	g	g	c	a	a	c	t	t	g	a	t	t	t	c	t	a	t	t	t	a	g	t	t	c	g	t	t	t	a	c	a	c	g	t	a	c	t	a	a	a	c	c	g	t	c	g	t	c	t	a	g	a	a	a	g	t	g
MF004348	2	a	t	c	g	c	c	t	c	t	g	t	t	a	t	t	t	g	g	g	c	a	a	c	t	t	g	a	t	t	t	a	t	a	t	t	c	a	g	t	t	c	g	t	t	t	a	c	a	c	g	t	a	c	t	a	a	a	c	c	g	t	c	g	t	c	t	a	g	a	a	a	g	t	g
MH709309	2	a	t	c	g	c	c	t	c	t	g	t	t	a	t	t	c	g	g	g	c	a	a	c	t	t	g	a	t	t	t	a	t	a	t	t	t	a	g	t	t	c	g	t	g	t	a	c	a	c	g	t	a	c	t	a	a	a	c	c	g	t	c	g	t	c	t	a	g	a	a	a	g	t	g
MH709310	2	a	t	c	g	c	c	t	c	t	g	t	t	a	t	t	c	g	g	g	c	a	a	c	t	t	g	a	t	t	t	a	t	a	t	t	t	a	g	t	t	c	g	t	t	t	a	c	a	c	g	t	a	c	t	a	a	a	c	c	g	t	c	g	t	c	t	a	g	a	a	a	g	t	g
MH709311	2	a	t	c	g	c	c	t	c	t	g	t	t	a	t	t	c	g	g	g	c	a	a	c	t	t	g	a	t	c	t	a	t	a	t	t	t	a	g	t	t	c	g	t	t	t	a	c	a	c	g	t	a	c	t	a	a	a	c	c	g	t	c	g	t	c	t	a	g	a	a	a	g	t	g
MH709313	2	a	t	c	g	c	c	t	c	t	g	t	t	a	t	t	c	g	g	g	c	a	a	t	t	t	g	a	t	t	t	a	t	a	t	t	t	a	g	t	t	c	g	t	t	t	a	c	a	c	g	t	a	c	t	a	a	a	c	c	g	t	c	g	c	c	t	a	g	a	a	a	g	t	g
MH709314	2	a	t	c	g	c	c	t	c	t	g	t	t	a	t	t	c	g	g	g	c	a	a	c	t	t	g	a	t	t	t	a	t	a	t	t	t	a	g	t	t	c	g	t	t	t	a	c	a	c	g	t	a	c	t	a	a	a	c	c	g	t	c	g	t	c	t	a	g	a	a	a	g	t	g
MH709315	2	a	t	c	g	c	c	t	c	t	g	t	t	a	t	t	c	g	a	g	c	a	a	c	t	t	g	a	t	t	t	a	t	a	t	t	t	a	g	t	t	c	g	t	t	t	a	c	a	c	g	t	a	c	t	a	a	a	c	c	g	t	c	g	t	c	t	a	g	a	a	a	g	t	g
MH709319	2	a	t	c	g	c	c	t	c	t	g	t	t	a	t	t	c	g	g	g	c	a	a	c	t	t	g	a	t	t	t	a	t	a	t	t	t	a	a	t	t	c	g	t	t	t	a	c	a	c	g	t	a	c	t	a	a	a	c	c	g	t	c	g	t	c	t	a	g	a	a	a	g	t	g
MH709320	2	a	t	c	g	c	c	t	c	t	g	t	t	a	t	t	c	g	g	g	c	a	a	c	t	t	g	a	t	t	t	a	t	a	t	t	t	a	g	t	t	c	g	t	t	t	a	c	a	c	g	t	a	c	t	a	a	a	c	c	g	t	c	g	t	c	t	a	g	a	a	a	g	t	g
MH709329	2	a	t	c	g	c	c	t	c	t	g	t	t	a	t	t	c	g	g	g	c	a	a	c	t	t	g	a	t	t	t	a	t	a	t	t	t	a	g	t	t	c	g	t	t	t	a	c	a	c	g	t	a	c	t	a	a	a	c	c	g	t	c	g	t	c	t	a	g	a	a	a	g	t	g
MH709331	2	a	t	c	g	c	c	t	c	t	g	t	t	a	t	t	c	g	g	g	c	a	a	c	t	t	g	a	t	t	t	a	t	a	t	t	t	a	g	t	t	c	g	t	t	t	a	c	a	c	g	t	a	c	t	a	a	a	c	c	a	t	c	g	t	c	t	a	g	a	a	a	g	t	g
MH709333	2	a	t	c	g	c	c	t	c	t	g	t	t	a	t	t	c	g	g	g	c	a	a	c	t	t	g	a	t	t	t	a	t	a	t	t	t	a	g	t	t	c	g	t	t	t	a	c	a	c	g	t	a	c	t	a	a	a	c	c	g	t	c	g	t	c	t	a	g	a	a	a	g	t	g
MH709337	2	a	t	c	g	c	c	t	c	t	g	t	t	a	t	t	c	g	g	g	c	a	a	c	t	t	g	a	t	t	t	a	t	a	t	t	t	a	g	t	t	c	g	t	t	t	a	c	a	c	g	t	a	c	t	a	a	a	c	c	g	t	c	g	t	c	t	a	g	a	a	a	g	t	g
MH709339	2	a	t	c	g	c	c	t	c	t	g	t	t	a	t	t	c	g	g	g	c	a	a	c	t	t	g	a	t	t	t	a	t	a	t	t	t	a	g	t	t	c	g	t	t	t	a	c	a	c	g	t	a	c	t	a	a	a	c	c	g	t	c	g	t	c	t	a	g	a	a	a	a	t	g
MH709344	2	a	t	c	g	c	c	t	c	t	g	t	t	a	t	t	c	g	g	g	c	a	a	c	t	t	g	a	t	t	t	a	t	a	t	t	t	a	g	t	t	c	g	t	t	t	a	c	a	c	g	t	a	c	t	a	a	a	c	c	g	t	c	g	t	c	t	a	g	a	a	a	g	t	g
MH709348	2	a	t	c	g	c	c	t	c	t	g	t	t	a	t	t	c	g	g	g	c	a	a	c	t	t	g	a	t	t	t	a	t	a	t	t	t	a	g	t	t	c	g	t	t	t	a	c	a	c	g	t	a	c	t	a	a	a	c	c	g	t	c	g	t	c	t	a	g	a	a	a	g	t	g
MH709355	2	a	t	c	g	c	c	t	c	t	g	t	t	a	t	t	c	g	g	g	c	a	a	c	t	t	g	a	t	t	t	a	t	a	t	t	t	a	g	t	t	c	g	t	t	t	a	c	a	c	g	t	a	c	t	a	a	a	c	c	g	t	c	g	t	c	t	a	g	a	a	a	g	t	g
MH709359	2	a	t	c	g	c	c	t	c	t	g	t	t	a	t	t	c	g	g	g	c	a	a	c	t	t	g	a	t	t	t	a	t	a	t	t	t	a	g	t	t	c	g	t	t	t	a	c	a	c	g	t	a	c	t	a	a	a	c	c	g	t	c	g	t	c	t	a	g	a	a	a	g	t	g
MH709363	2	a	t	c	g	c	c	t	c	t	g	t	t	a	t	t	c	g	g	g	c	a	a	c	t	t	g	a	t	t	t	a	t	a	t	t	t	a	g	t	t	c	g	t	t	t	a	c	a	c	g	t	a	c	t	a	a	a	c	c	g	t	c	g	t	c	t	a	g	a	a	a	g	t	g
MH709366	2	a	t	c	g	c	c	t	c	t	g	t	t	a	t	t	c	g	g	g	c	a	a	c	t	t	g	a	t	t	t	a	t	a	t	t	t	a	g	t	t	c	g	t	t	t	a	c	a	c	g	t	a	c	t	a	a	a	c	c	g	t	c	g	t	c	t	a	g	a	a	a	g	t	g
MH709376	2	a	t	c	g	c	c	t	c	t	g	t	t	a	t	t	c	g	g	g	c	a	a	c	t	t	g	a	t	t	t	a	t	a	t	t	t	a	g	t	t	c	g	t	t	t	a	c	a	c	g	t	a	c	t	a	a	a	c	c	g	t	c	g	t	c	t	a	g	a	a	a	g	t	g
MH709377	2	a	t	c	g	c	c	t	c	t	g	t	t	a	t	t	c	g	g	g	c	a	a	c	t	t	g	a	t	t	t	a	t	a	t	t	t	a	g	t	t	c	g	t	t	t	a	c	a	c	g	t	a	c	t	a	a	a	c	c	g	t	c	g	t	c	t	a	g	a	a	a	g	t	g
**MW316406**	2	a	t	c	g	c	c	t	c	t	g	t	t	a	t	t	c	g	g	g	c	c	t	c	t	t	g	a	t	t	t	a	t	a	t	t	t	a	g	t	t	c	g	t	t	t	a	c	a	c	g	t	a	c	t	a	a	a	c	c	g	t	c	g	t	c	t	a	g	a	a	a	g	t	g
AJ871403	3	a	t	c	g	c	c	t	c	t	g	t	t	a	t	g	c	a	g	g	c	a	a	c	t	t	g	a	g	t	t	a	t	a	t	t	t	a	g	t	t	c	g	t	t	t	a	c	a	c	g	t	a	c	c	a	a	a	c	t	g	t	c	g	t	c	t	a	g	a	a	a	g	t	g
DQ479955	3	a	t	c	g	c	c	t	c	t	g	t	t	a	t	g	c	a	g	g	c	a	a	c	t	t	g	a	g	t	t	a	t	a	t	t	t	a	g	t	t	c	g	t	t	t	a	c	a	c	g	t	a	c	c	a	a	a	c	t	g	t	c	g	t	c	t	a	g	a	a	a	g	t	g
DQ479956	3	a	t	c	g	c	c	t	c	t	g	t	t	a	t	g	c	a	g	g	c	a	a	c	t	t	g	a	g	t	t	a	t	a	t	t	t	a	g	g	t	c	g	t	t	t	a	c	a	c	g	t	a	c	c	a	a	a	c	t	g	t	c	g	t	c	t	a	g	a	a	a	g	t	g
DQ479957	3	a	t	c	g	c	c	t	c	t	g	t	t	a	t	g	c	a	g	g	c	a	a	c	t	t	g	a	g	t	t	a	t	a	t	t	t	a	g	t	t	c	g	t	t	t	a	c	a	c	g	t	a	c	c	a	a	a	c	t	g	t	c	g	t	c	t	a	g	a	a	a	g	t	g
JN704699	3	a	t	c	g	c	c	t	c	t	g	t	t	a	t	g	c	g	g	g	c	a	a	c	t	t	g	a	g	t	t	a	t	a	t	t	t	a	g	t	t	c	g	t	t	t	a	c	a	c	g	t	a	c	c	a	a	a	c	t	g	t	c	g	t	c	t	a	g	a	a	a	g	t	g
JN704700	3	a	c	c	g	c	c	t	c	t	g	t	t	a	t	g	c	g	g	g	c	a	a	c	t	t	g	a	g	t	t	g	t	a	t	t	t	a	g	t	t	c	g	t	t	t	a	c	a	c	g	t	a	c	c	a	a	a	c	t	g	t	c	g	t	c	t	a	g	a	a	a	g	t	g
JN704701	3	a	t	c	g	c	c	t	c	t	g	t	t	a	t	g	c	a	g	g	c	a	a	c	t	t	g	a	g	t	t	a	t	a	t	t	t	a	g	t	t	c	g	t	t	t	a	c	a	c	g	t	a	c	c	a	a	a	c	t	g	t	c	g	t	c	t	a	g	a	a	a	g	t	g
JN704702	3	a	t	c	g	c	c	t	c	t	g	t	t	a	t	g	c	g	g	g	c	a	a	c	t	t	g	a	g	t	t	g	t	a	t	t	t	a	g	t	t	c	g	t	t	t	a	c	a	c	g	t	a	c	c	a	a	a	c	t	g	t	c	g	t	c	t	a	g	a	a	a	g	t	g
JN704703	3	a	t	c	g	c	c	t	c	t	g	t	t	a	t	g	c	a	g	g	c	a	a	c	t	t	g	a	g	t	t	g	t	a	t	t	t	a	g	t	t	c	a	t	t	t	a	c	a	c	g	t	a	c	c	a	a	a	c	t	g	t	c	g	t	c	t	a	g	a	a	a	g	t	g
KP771891	3	a	t	c	g	c	c	t	c	t	g	t	t	a	t	g	c	a	g	g	c	a	a	c	t	t	g	a	g	t	t	a	t	a	t	t	t	a	g	t	t	c	g	t	t	t	a	c	a	c	g	t	a	c	c	a	a	a	c	t	g	t	c	g	t	c	t	a	g	a	a	a	g	t	g
KP771893	3	a	t	c	g	c	c	t	c	t	g	t	t	a	t	g	c	a	g	g	c	a	a	c	t	t	g	a	g	t	t	a	t	a	t	t	t	a	g	t	t	c	g	t	t	t	a	c	a	c	g	t	a	c	c	a	a	a	c	t	g	t	c	g	t	c	t	a	g	a	a	a	g	t	g
KP771895	3	a	t	c	g	c	c	t	c	t	g	t	t	a	t	g	c	a	g	a	c	a	a	c	t	t	g	a	g	t	t	a	t	a	c	t	t	a	g	t	t	c	g	t	t	t	a	c	a	c	g	t	a	c	c	a	a	a	c	t	g	t	c	g	t	c	t	a	g	a	a	a	g	t	g
KP771896	3	a	t	c	g	c	c	t	c	t	g	t	t	a	t	g	c	a	g	g	c	a	a	c	t	t	g	a	g	t	t	a	t	a	t	t	t	a	g	t	t	t	g	t	t	t	a	c	a	c	g	t	a	c	c	a	a	a	c	t	g	t	c	g	t	c	t	a	g	a	a	a	g	t	g
KP771897	3	a	t	c	g	c	c	t	c	t	g	t	t	a	t	g	c	a	g	g	c	a	a	c	t	t	g	a	g	t	t	a	t	a	t	t	t	a	g	t	t	c	g	t	t	t	a	c	a	c	g	t	a	c	c	a	a	a	c	t	g	t	c	g	t	c	t	a	g	a	a	a	g	t	g
KP771899	3	a	t	c	g	c	c	t	c	t	g	t	t	a	t	g	c	a	g	g	c	a	a	c	t	t	g	a	g	t	t	a	t	a	t	t	t	a	g	t	t	c	g	t	t	t	a	c	a	c	g	t	a	c	c	a	a	a	c	t	g	t	c	g	t	c	t	a	g	a	a	a	g	t	g
KP771903	3	a	t	c	g	c	c	t	c	t	g	t	t	a	t	g	c	a	g	g	c	a	a	c	t	t	g	a	g	t	t	a	t	a	t	t	t	a	g	t	t	c	g	t	t	t	a	c	a	c	g	t	a	c	c	a	a	a	c	t	g	t	c	g	t	c	t	a	g	a	a	a	g	t	g
KP771907	3	a	t	c	g	c	c	t	c	t	g	t	t	a	t	g	c	a	g	g	c	a	a	c	t	t	g	a	g	t	t	a	t	a	t	t	t	a	g	t	t	c	g	t	t	t	a	c	a	c	a	t	a	c	c	a	a	a	c	t	g	t	c	g	t	c	t	a	g	a	a	a	g	t	g
KP771910	3	a	t	c	g	c	c	t	c	t	g	t	t	a	t	g	c	a	g	g	c	a	a	c	t	t	g	a	g	t	t	a	t	a	t	t	t	a	g	t	t	c	g	t	t	t	a	c	a	c	g	t	a	c	c	a	a	a	c	t	g	t	c	g	t	c	t	a	g	a	a	a	g	t	g
KP771915	3	a	t	c	g	c	c	t	c	t	g	t	t	a	t	g	c	a	g	g	c	a	a	c	t	t	g	a	g	t	t	a	t	a	t	t	t	a	g	t	t	c	g	t	t	t	a	c	a	c	g	t	a	c	c	a	a	a	c	t	g	t	c	g	t	c	t	a	g	a	a	a	g	t	g
KP771916	3	a	t	c	g	c	c	t	c	t	g	t	t	a	t	g	c	a	g	g	c	a	a	c	t	t	g	a	g	t	t	a	t	a	t	t	t	a	g	g	t	c	g	t	t	t	a	c	a	c	g	t	a	c	c	a	a	a	c	t	g	t	c	g	t	c	t	a	g	a	a	a	g	t	g
KP771921	3	a	t	c	g	c	c	t	c	t	g	t	t	a	t	g	c	a	g	g	c	a	a	c	t	t	g	a	g	t	t	a	t	a	t	t	t	a	g	t	g	c	g	t	t	t	a	c	a	c	g	t	a	c	c	a	a	a	c	t	g	t	c	g	t	c	t	a	g	a	a	a	g	t	g
KP771923	3	a	t	c	g	c	c	t	c	t	g	t	t	a	t	g	c	a	g	g	c	a	a	c	t	t	g	a	g	t	t	a	t	a	t	t	t	a	g	t	t	c	g	t	t	t	a	c	a	c	g	t	a	c	c	a	a	a	c	t	g	t	c	g	t	c	t	a	g	a	a	a	g	t	g
KP771889	3	a	t	c	g	c	c	t	c	t	g	t	t	a	t	g	c	g	g	g	c	a	a	c	t	t	g	a	g	t	t	a	t	a	t	t	t	a	g	t	t	c	g	t	t	t	a	c	a	c	g	t	a	c	t	a	a	g	c	t	g	t	c	g	t	c	t	a	g	a	a	a	g	t	g
KU926311	3	a	t	c	g	c	c	t	c	t	g	t	t	a	t	g	c	a	g	g	c	a	a	c	t	t	g	a	g	t	t	a	t	a	t	t	t	a	g	g	t	c	g	t	t	t	a	c	a	c	g	t	a	c	c	a	a	a	c	t	g	t	c	g	t	c	t	a	g	a	a	a	g	t	g
JQ911599	3	a	t	c	g	c	c	t	c	c	g	t	t	a	t	g	c	a	g	g	c	a	a	c	t	t	g	a	g	t	t	g	t	a	t	t	t	a	g	t	t	c	g	t	t	t	a	c	a	c	g	t	a	c	c	a	a	a	c	t	g	t	c	g	t	c	t	a	g	a	a	a	g	t	g
MH709317	3	a	t	c	g	c	c	t	c	t	g	t	t	a	t	g	c	a	g	g	c	a	a	c	t	t	g	a	g	t	t	a	t	a	t	t	t	a	g	t	t	c	g	t	t	t	a	c	a	c	g	t	a	c	t	a	a	a	c	t	g	t	c	g	t	c	t	a	g	a	a	a	g	t	g
MH709346	3	a	t	c	g	c	c	t	c	t	g	t	c	a	t	g	c	a	g	g	c	a	a	c	t	t	g	a	g	t	t	a	t	a	t	t	t	a	g	t	t	c	g	t	t	t	a	c	a	c	g	t	a	c	t	a	a	a	c	t	g	t	c	g	t	c	t	a	g	a	a	a	g	t	g
MH709375	3	a	t	c	g	c	c	t	c	t	g	t	t	a	t	g	c	a	g	g	c	a	a	c	t	t	g	a	g	t	t	a	t	a	t	t	t	a	g	t	t	c	g	t	t	t	a	c	a	c	g	t	a	c	t	a	a	a	c	t	g	t	c	g	t	c	t	a	g	a	a	a	g	t	g
DQ452050	4	a	t	c	g	c	c	t	c	t	g	t	t	a	t	t	c	g	g	g	c	a	a	c	t	t	g	a	g	t	t	a	t	a	t	t	t	a	g	t	t	c	g	t	t	t	a	c	c	c	g	t	a	c	t	a	a	a	c	c	g	t	c	g	t	c	t	a	g	c	a	a	g	t	g
DQ479960	4	a	t	c	g	c	t	t	c	t	g	t	t	a	t	t	c	g	g	g	c	a	a	c	t	t	g	a	g	t	t	a	t	a	t	t	t	a	g	t	t	c	g	t	t	t	a	c	a	c	g	t	a	c	t	a	a	a	c	c	g	t	c	g	t	c	t	a	g	c	a	a	g	t	g
KP771898	4	a	t	c	g	c	c	t	c	t	g	t	t	a	t	t	c	g	g	g	c	a	a	c	t	t	g	a	g	t	t	a	t	a	t	t	t	a	g	t	t	c	g	t	t	t	a	c	a	c	g	t	a	c	t	a	a	a	c	c	g	t	c	g	t	c	t	a	g	c	a	a	g	t	g
MH709316	4	a	t	c	g	c	c	t	a	t	g	t	t	a	a	t	c	g	g	g	c	a	a	c	t	t	g	a	g	t	t	a	t	a	t	t	t	a	g	t	t	c	g	t	t	t	a	c	a	c	g	t	a	c	t	a	a	a	c	c	g	t	c	g	t	c	t	a	g	c	a	a	g	t	g
MH709318	4	a	t	c	g	c	c	t	c	t	g	t	t	a	t	t	c	g	g	g	c	a	a	c	t	t	g	a	g	t	t	a	t	a	t	t	t	a	g	t	t	c	g	t	t	t	a	c	a	c	g	t	a	c	t	a	a	a	c	c	g	t	c	g	t	c	t	a	g	c	a	a	g	t	g
MH709322	4	a	t	a	g	c	c	t	c	t	g	t	t	a	t	t	c	g	g	g	c	a	a	c	t	t	g	a	g	t	t	a	t	a	t	t	t	a	g	t	t	c	g	t	t	t	a	c	a	c	g	t	a	c	t	a	a	a	c	c	g	t	c	g	t	c	t	a	g	c	a	a	g	t	g
MH709323	4	a	t	c	g	c	c	t	c	t	g	t	t	a	t	t	c	g	g	g	c	a	a	c	t	t	g	a	g	t	t	a	t	a	t	t	t	a	g	t	t	c	g	t	t	t	a	c	a	c	g	t	a	c	t	a	a	a	c	c	g	t	c	g	t	c	t	a	g	c	a	a	g	t	g
MH709326	4	a	t	c	g	c	c	t	c	t	g	t	t	a	t	t	c	g	g	g	c	a	a	c	t	t	g	a	g	t	t	a	t	a	t	t	t	a	g	t	t	c	g	t	t	t	a	c	a	c	g	t	a	c	t	a	a	a	c	c	g	t	c	g	t	c	t	a	g	c	a	a	g	t	g
MH709341	4	a	t	c	g	c	c	t	c	t	g	t	t	a	t	t	c	g	g	g	c	a	a	c	t	t	g	a	g	t	t	a	t	a	t	t	t	a	g	t	t	c	g	t	t	t	a	c	a	c	g	t	a	c	t	a	a	a	c	c	g	t	c	g	t	c	t	a	g	c	a	a	g	t	g
MH709357	4	a	t	c	g	c	t	t	c	t	g	t	t	a	t	t	c	g	g	g	c	a	a	c	t	t	a	a	g	t	t	a	t	a	t	t	t	a	g	t	t	c	g	t	t	t	a	c	a	c	g	t	a	c	t	a	a	a	c	c	g	t	c	g	t	a	t	a	g	c	a	a	g	t	g
MH709364	4	a	t	c	g	c	t	t	c	t	g	t	t	a	t	t	c	g	g	g	c	a	a	c	t	t	g	c	g	t	t	a	t	a	t	t	t	a	g	t	t	c	g	t	t	t	a	c	a	c	g	t	a	c	t	a	a	a	c	c	g	t	c	g	t	c	t	a	a	c	a	a	g	t	g
MH709370	4	a	t	c	g	c	t	t	c	t	g	t	t	a	t	t	c	g	g	g	c	a	a	c	t	t	g	a	g	t	t	a	t	a	t	t	t	a	g	t	t	c	g	t	t	t	a	c	a	c	g	t	a	c	t	a	a	a	c	c	g	t	c	g	t	c	t	a	g	c	a	a	g	t	g
MH709371	4	a	t	c	g	c	t	t	c	t	g	t	t	a	t	t	c	g	g	g	c	a	a	c	t	t	g	a	g	t	t	a	t	a	t	t	t	a	g	t	t	c	g	t	t	t	a	c	a	c	g	t	a	c	t	a	a	a	c	c	g	t	c	g	t	c	t	a	g	c	a	a	g	t	g
MH709372	4	a	t	c	g	c	t	t	c	t	g	t	t	a	t	t	c	g	g	g	c	a	a	c	t	t	g	a	g	t	t	a	t	a	t	t	t	a	g	t	t	c	g	t	t	t	a	c	a	c	g	t	a	c	t	a	a	a	c	c	g	t	c	g	t	c	t	a	g	c	a	a	g	t	g
DQ457052	5	a	t	c	g	c	c	t	c	t	g	t	t	a	t	t	c	g	g	g	c	a	a	c	t	t	g	a	g	t	t	a	t	a	t	t	t	a	g	t	t	c	g	t	t	t	a	c	a	c	g	t	a	c	t	a	a	a	c	c	g	t	c	g	t	c	t	a	g	a	a	a	g	t	g
JN704704	5	a	t	c	g	c	c	t	c	t	g	t	t	a	t	t	c	g	g	g	c	a	a	c	t	t	g	a	g	t	t	a	t	a	t	t	t	a	g	t	t	c	g	t	t	t	a	c	a	c	g	t	a	c	t	a	a	a	c	c	g	t	t	g	t	c	t	a	g	a	a	a	g	t	g
JN704705	5	a	t	c	g	c	c	t	c	t	g	t	t	a	t	t	c	g	g	g	c	a	a	c	t	t	g	a	g	t	t	a	t	a	t	t	t	a	g	t	t	c	g	t	t	t	a	c	a	c	g	t	a	c	t	a	a	a	c	c	g	t	t	g	t	c	t	a	g	a	a	a	g	t	g
JN704706	5	a	t	c	g	c	c	t	c	t	g	t	t	a	t	t	c	g	g	g	c	a	a	c	t	t	g	a	g	t	t	a	t	a	t	t	t	a	g	t	t	c	g	t	t	t	a	c	a	c	g	t	a	c	t	a	a	a	c	c	g	t	t	g	t	c	t	a	g	a	a	a	g	t	g
JN704707	5	a	t	c	g	c	c	t	c	t	g	t	t	a	t	t	c	g	g	g	c	a	a	c	t	t	g	a	g	t	t	a	t	a	t	t	t	a	g	t	t	c	g	t	t	t	a	c	a	c	g	t	a	c	t	a	a	a	c	c	g	t	t	g	t	c	t	a	g	a	a	a	g	t	g
JN704708	5	a	t	c	g	c	c	t	c	t	g	t	t	a	t	t	c	g	g	g	c	a	a	c	t	t	g	a	g	t	t	a	t	a	t	t	t	a	g	t	t	c	g	t	t	t	a	c	a	c	g	t	a	c	t	a	a	a	c	c	g	t	t	g	t	c	t	g	g	a	a	a	g	t	g
KP771904	5	a	t	c	a	c	c	t	c	t	g	t	t	a	t	t	c	g	g	g	c	a	a	c	t	t	g	a	g	t	t	a	t	a	t	t	t	a	g	t	t	c	g	t	t	t	a	c	a	c	g	t	a	c	t	a	a	a	c	c	g	t	t	g	t	c	t	a	g	a	a	a	g	t	g
KP771905	5	a	t	c	g	c	c	t	c	t	g	t	t	a	t	t	c	g	g	g	c	a	a	c	t	t	g	a	g	t	t	a	t	a	t	t	t	a	g	t	t	c	g	t	t	t	a	c	a	c	g	t	a	c	t	a	a	a	c	c	g	t	t	g	t	c	t	a	g	a	a	a	g	t	g
KP771906	5	a	t	c	g	c	c	t	c	t	g	t	t	a	t	t	c	g	g	g	c	a	a	c	t	t	g	a	g	t	t	a	t	a	t	t	t	a	g	t	t	c	g	t	t	t	a	c	a	c	g	t	a	c	t	a	a	a	c	c	g	t	t	g	t	c	t	a	g	a	a	a	g	t	g
KP771919	5	a	t	c	g	c	c	t	c	t	g	t	t	a	t	t	c	g	g	g	c	a	a	c	t	t	g	a	g	t	t	a	t	a	t	t	t	a	g	t	t	c	g	t	t	t	a	c	a	c	g	t	a	c	t	a	a	a	c	c	g	t	t	g	t	c	t	a	g	a	a	a	g	t	g
KP771922	5	a	t	c	g	c	c	t	c	t	g	t	t	a	t	t	c	g	g	g	c	a	a	c	t	t	g	a	g	t	t	a	t	a	t	t	t	a	g	t	t	c	g	t	t	t	a	c	a	c	g	t	a	c	t	a	a	a	c	c	g	t	t	g	t	c	t	a	g	a	a	a	g	t	g
KP771924	5	a	t	c	g	c	c	t	c	t	g	t	t	a	t	t	c	g	g	g	c	a	a	c	t	t	g	a	g	t	t	a	t	a	t	t	t	a	g	t	t	c	g	t	t	t	a	c	a	c	g	t	a	c	t	a	a	a	c	c	g	t	t	g	t	c	t	a	g	a	a	a	g	t	g
KM355696	5	a	t	c	g	c	c	t	c	t	g	t	t	a	t	t	c	g	g	g	c	a	a	c	t	t	g	a	g	t	t	a	t	a	t	t	t	a	g	t	t	c	g	t	t	t	a	c	a	c	g	t	a	c	t	a	a	a	c	c	g	t	t	g	t	c	t	a	g	a	a	a	g	t	g
KM355697	5	a	t	c	g	c	c	t	c	t	g	t	t	a	t	t	c	g	g	g	c	a	a	c	t	t	g	a	g	t	t	a	t	a	t	t	t	a	g	t	t	c	g	t	t	t	a	c	a	c	g	t	a	c	t	a	a	a	c	c	g	t	t	g	t	c	t	a	g	a	a	a	g	t	g
KM355698	5	a	t	c	g	c	c	t	c	t	g	t	t	a	t	t	c	g	g	g	c	a	a	c	t	t	g	a	g	t	t	a	t	a	t	t	t	a	g	t	t	c	g	t	t	t	a	c	a	c	g	t	a	c	t	a	a	a	c	c	g	t	t	g	t	c	t	a	g	a	a	a	g	t	g
KM355699	5	a	t	c	g	c	c	t	c	t	g	t	t	a	t	t	c	g	g	g	c	a	a	c	t	t	g	a	g	t	t	a	t	a	t	t	t	a	g	t	t	c	g	t	t	t	a	c	a	c	g	t	a	c	t	a	a	a	c	c	g	t	t	g	t	c	t	a	g	a	a	a	g	t	g
KM355701	5	a	t	c	g	c	c	t	c	t	g	t	t	a	t	t	c	g	g	g	c	a	a	c	t	t	g	a	g	t	t	a	t	a	t	t	t	a	g	t	t	c	g	t	t	t	a	c	a	c	g	t	a	c	t	a	a	a	c	c	g	t	t	g	t	c	t	a	g	a	a	a	g	t	g
KM355702	5	a	t	c	g	c	c	t	c	t	g	t	t	a	t	t	c	g	g	g	c	a	a	c	t	t	g	a	g	t	t	a	t	a	t	t	t	a	g	t	t	c	g	t	t	t	a	c	a	c	g	t	a	c	t	a	a	a	c	c	g	t	t	g	t	c	t	a	g	a	a	a	g	t	g
KM355703	5	a	t	c	g	c	c	t	c	t	g	t	t	a	t	t	c	g	g	g	c	a	a	c	t	t	g	a	g	t	t	a	t	a	t	t	t	a	g	t	t	c	g	t	t	t	a	c	a	c	g	t	a	c	t	a	a	a	c	c	g	t	t	g	t	c	t	a	g	a	a	a	g	t	g
KM355704	5	a	t	c	g	c	c	t	c	t	g	t	t	a	t	t	c	g	g	g	c	a	a	c	t	t	g	a	g	t	t	a	t	a	t	t	t	a	g	t	t	c	g	t	t	t	a	c	a	c	g	t	a	c	t	a	a	a	c	c	g	t	t	g	t	c	t	a	g	a	a	a	g	t	g
KM355705	5	a	t	c	g	c	c	t	c	t	g	t	t	a	t	t	c	g	g	g	c	a	a	c	t	t	g	a	g	t	t	a	t	a	t	t	t	a	g	t	t	c	g	t	t	t	a	c	a	c	g	t	a	c	t	a	a	a	c	c	g	t	t	g	t	c	t	a	g	a	a	a	g	t	g
KM355706	5	a	t	c	g	c	c	t	c	t	g	t	t	a	t	t	c	g	g	g	c	a	a	c	t	t	g	a	g	t	t	a	t	a	t	t	t	a	g	t	t	c	g	t	t	t	a	c	a	c	g	t	a	c	t	a	a	a	c	c	g	t	t	g	t	c	t	a	g	a	a	a	g	t	g
KM355707	5	a	t	c	g	c	c	t	c	t	g	t	t	a	t	t	c	g	g	g	c	a	a	c	t	t	g	a	g	t	t	a	t	a	t	t	t	a	g	t	t	c	g	t	t	t	a	c	a	c	g	t	a	c	t	a	a	a	c	c	g	t	t	g	t	c	t	a	g	a	a	a	g	t	g
KM355708	5	a	t	c	g	c	c	t	c	t	g	t	t	a	t	t	c	g	g	g	c	a	a	c	t	t	g	a	g	t	t	a	t	a	t	t	t	a	g	t	t	c	g	t	t	t	a	c	a	c	g	t	a	c	t	a	a	a	c	c	g	t	t	g	t	c	t	a	g	a	a	a	g	t	g
KM355709	5	a	t	c	g	c	c	t	c	t	g	t	t	a	t	t	c	g	g	g	c	a	a	c	t	t	g	a	g	t	t	a	t	a	t	t	t	a	g	t	t	c	g	t	t	t	a	c	a	c	g	t	a	c	t	a	a	a	c	c	g	t	t	g	t	c	t	a	g	a	a	a	g	t	g
KM355711	5	a	t	c	g	c	c	t	c	t	g	t	t	a	t	t	c	g	g	g	c	a	a	c	t	t	g	a	g	t	t	a	t	a	t	t	t	a	g	t	t	c	g	t	t	t	a	c	a	c	g	t	a	c	t	a	a	a	c	c	g	t	t	g	t	c	t	a	g	a	a	a	g	t	g
KM355712	5	a	t	c	g	c	c	t	c	t	g	t	t	a	t	t	c	g	g	g	c	a	a	c	t	t	g	a	g	t	t	a	t	a	t	t	t	a	g	t	t	c	g	t	t	t	a	c	a	c	g	t	a	c	t	a	a	a	c	c	g	t	t	g	t	c	t	a	g	a	a	a	g	t	g
[illegible]	[illegible]	[illegible]	[illegible]	[illegible]	[illegible]	[illegible]	[illegible]	[illegible]	[illegible]	[illegible]	[illegible]	[illegible]	[illegible]	[illegible]	[illegible]	[illegible]	[illegible]	[illegible]	[illegible]	[illegible]	[illegible]	[illegible]	[illegible]	[illegible]	[illegible]	[illegible]	[illegible]	[illegible]	[illegible]	[illegible]	[illegible]	[illegible]	[illegible]	[illegible]	[illegible]	[illegible]	[illegible]	[illegible]	[illegible]	[illegible]	[illegible]	[illegible]	[illegible]	[illegible]	[illegible]	[illegible]	[illegible]	[illegible]	[illegible]	[illegible]	[illegible]	[illegible]	[illegible]	[illegible]	[illegible]	[illegible]	[illegible]	[illegible]	[illegible]	[illegible]	[illegible]	[illegible]	[illegible]	[illegible]	[illegible]	[illegible]	[illegible]	[illegible]	[illegible]	[illegible]	[illegible]	[illegible]	[illegible]	[illegible]	[illegible]

Supplementary Table 3: 2880 SN

GB-ID	Clade markers																					SD14	SD14																																																	4					
	Position in Dumas	65610	65669	65683	65751	65759	65762	65768	65858	65878	66003	66063	66073	66085	66106	66112	66247	66288	66304	66311	66350	66366	66367	66385	66450	66499	66568	66623	66646	66849	66879	66880	66947	66951	67025	67028	67114	67146	67244	67327	67331	67341	67348	67429	67477	67573	67603	67622	67724	67847	67883	67963	67999	68050	68101	68140	68150	68172	68212	68254	68331	68344	68371	68518	68662	68693	68818	69034	69043	69067	69072	69089	69124	69148	69211
MH499467	5	a	t	c	g	c	c	t	c	t	g	t	t	a	t	t	c	g	g	g	c	a	a	c	t	t	g	a	g	t	t	a	g	a	t	t	t	a	g	-	-	-	-	t	t	t	a	c	a	c	g	t	a	c	t	a	a	a	c	c	g	t	t	g	t	c	t	a	g	a	a	a	g	c	g
MH499469	5	a	t	c	g	c	c	t	c	t	g	t	t	a	t	t	c	g	g	g	c	a	a	c	t	t	g	a	g	t	t	a	t	a	t	t	t	a	g	t	t	c	g	t	t	t	a	c	a	c	g	t	a	c	t	a	a	a	c	c	g	t	t	g	t	c	t	a	g	a	a	a	g	t	g
MH709321	5	a	t	c	g	c	c	t	c	t	g	t	t	a	t	t	c	g	g	g	c	a	a	c	t	t	g	a	g	t	t	a	t	a	t	t	t	a	g	t	t	c	g	t	t	t	a	c	a	t	g	t	a	c	t	a	a	a	c	c	g	t	t	g	t	c	t	a	g	a	a	a	g	t	g
MH709324	5	a	t	c	g	c	c	t	c	t	g	t	t	a	t	t	c	g	g	g	c	a	a	c	t	t	g	a	g	t	t	a	t	a	t	t	t	a	g	t	t	c	g	t	t	t	a	c	a	c	g	t	a	c	t	a	a	a	c	c	g	t	t	g	t	c	t	a	g	a	a	a	g	t	g
MH709325	5	a	t	c	g	c	c	t	c	t	g	t	t	a	t	t	c	g	g	g	c	a	a	c	t	t	g	a	g	t	t	a	t	a	t	t	t	a	g	t	t	c	g	t	t	t	a	c	a	c	g	t	a	c	t	a	a	a	c	c	g	t	t	g	t	c	t	a	g	a	a	a	g	t	g
MH709327	5	a	t	c	g	c	c	t	c	t	g	t	t	a	t	t	c	g	g	g	c	a	a	c	t	t	g	a	g	t	t	a	t	a	t	t	t	a	g	t	t	c	g	t	t	t	a	c	a	c	g	t	a	c	t	a	a	a	c	c	g	t	t	g	t	c	t	a	g	a	a	a	g	t	g
MH709328	5	a	t	c	g	c	c	t	c	t	g	t	t	a	t	t	c	g	g	g	c	a	a	c	t	t	g	a	g	t	t	a	t	a	t	t	t	a	g	t	t	c	g	t	t	t	a	c	a	c	g	t	a	c	t	a	a	a	c	c	g	t	t	g	t	c	t	a	g	a	a	a	g	t	g
MH709330	5	a	t	c	g	c	c	t	c	t	g	t	t	a	t	t	c	g	g	g	c	a	a	c	t	t	g	a	g	t	t	a	t	a	t	t	t	a	g	t	t	c	g	t	t	t	a	c	a	c	g	t	a	c	t	a	a	a	c	c	g	t	t	g	t	c	t	a	g	a	a	a	g	t	g
MH709332	5	c	t	c	g	c	c	t	c	t	g	t	t	a	t	t	c	g	g	g	c	a	a	c	t	t	g	a	g	t	t	a	t	a	t	t	t	a	g	t	t	c	g	t	t	t	a	c	a	c	g	t	a	c	t	a	a	a	c	c	g	t	t	g	t	c	t	a	g	a	a	a	g	t	g
MH709334	5	a	t	c	g	c	c	t	c	t	g	t	t	a	t	t	c	g	g	g	c	a	a	c	t	t	g	a	g	t	t	a	t	a	t	t	t	a	g	t	t	c	g	t	t	t	a	c	a	c	g	t	a	c	t	a	a	a	c	c	g	t	t	g	t	c	t	a	g	a	a	a	g	t	a
MH709335	5	a	t	c	g	c	c	t	c	t	g	t	t	a	t	t	c	g	g	g	c	a	a	c	t	t	g	a	g	t	t	a	t	a	t	t	t	a	g	t	t	c	g	t	t	t	a	c	a	c	g	t	a	c	t	a	a	a	c	c	g	t	t	g	t	c	t	a	g	a	a	a	g	t	g
MH709336	5	a	t	c	g	c	c	t	c	t	g	t	t	a	t	t	c	g	g	g	c	a	a	c	t	t	g	a	g	t	t	a	t	a	t	t	t	a	g	t	t	c	g	t	t	t	a	c	a	c	g	t	a	c	t	a	a	a	c	c	g	t	t	g	t	c	t	a	g	a	a	a	g	t	g
MH709338	5	a	t	c	g	c	c	t	c	t	g	t	t	a	t	t	c	g	g	g	c	a	a	c	t	t	g	a	g	t	t	a	t	a	t	t	t	a	g	t	t	c	g	t	t	t	a	c	a	c	g	t	a	c	t	a	a	a	c	c	g	t	t	g	t	c	t	a	g	a	a	a	g	t	g
MH709340	5	a	t	c	g	c	c	t	c	t	g	t	t	a	t	t	c	g	g	g	c	a	a	c	t	t	g	a	g	t	t	a	t	a	t	t	t	a	g	t	t	c	g	t	t	t	a	c	a	c	g	t	a	c	t	a	a	a	c	c	g	t	t	g	t	c	t	a	g	a	a	a	g	t	g
MH709342	5	a	t	c	g	c	c	t	c	t	g	t	t	a	t	t	c	g	g	g	c	a	a	c	t	t	g	a	g	t	t	a	t	a	t	t	t	a	g	t	t	c	g	t	t	t	a	c	a	t	g	t	a	c	t	a	a	a	c	c	g	t	t	g	t	c	t	a	g	a	a	a	g	t	g
MH709343	5	a	t	c	g	c	c	t	c	t	g	t	t	a	t	t	c	g	g	g	c	a	a	c	t	t	g	a	g	t	t	a	t	a	t	t	t	a	g	t	t	c	g	t	t	t	a	c	a	c	g	t	a	c	t	a	a	a	c	c	g	t	t	g	t	c	t	a	g	a	a	a	g	t	g
MH709345	5	a	t	c	g	c	c	t	c	t	g	t	t	a	t	t	c	g	g	g	c	a	a	c	t	t	g	a	g	t	t	a	t	a	t	t	t	a	g	t	t	c	g	t	t	t	a	c	a	c	g	t	a	c	t	a	a	a	c	c	g	t	t	g	t	c	t	a	g	a	a	a	g	t	g
MH709347	5	a	t	c	g	c	c	t	c	t	g	t	t	a	t	t	c	g	g	g	c	a	a	c	t	t	g	a	g	t	t	a	t	a	t	t	t	a	g	t	t	c	g	t	t	t	a	c	a	c	g	t	a	c	t	a	a	a	c	c	g	t	t	g	t	c	t	a	g	a	a	a	g	t	g
MH709349	5	a	t	c	g	c	c	t	c	t	g	t	t	a	t	t	c	g	g	g	c	a	a	c	t	t	g	a	g	t	t	a	t	a	t	t	t	a	g	t	t	c	g	t	t	t	a	c	a	c	g	t	a	c	t	a	a	a	c	c	g	t	t	g	t	c	t	a	g	a	a	a	g	t	g
MH709350	5	a	t	c	g	c	c	t	c	t	g	t	t	a	t	t	c	g	g	g	c	a	a	c	t	t	g	a	g	t	t	a	t	a	t	t	t	a	g	t	t	c	g	t	t	t	a	c	a	c	g	t	a	c	t	a	a	a	c	c	g	t	t	g	t	c	t	a	g	a	a	a	g	t	g
MH709351	5	a	t	c	g	c	c	t	c	t	g	t	t	a	t	t	c	g	g	g	c	a	a	c	t	t	g	a	g	t	t	a	t	a	t	t	t	a	g	t	t	c	g	t	t	t	a	c	a	c	g	t	a	c	t	a	a	a	c	c	g	t	t	g	t	c	t	a	g	a	a	a	g	t	g
MH709353	5	a	t	c	g	c	c	t	c	t	g	t	t	a	t	t	c	g	g	g	c	a	a	c	t	t	g	a	g	t	t	a	t	a	t	t	t	a	g	t	t	c	g	t	t	t	a	c	a	c	g	t	a	c	t	a	a	a	c	c	g	t	t	g	t	c	t	a	g	a	a	g	g	t	g
MH709358	5	a	t	c	g	c	c	t	c	t	g	t	t	a	t	t	c	g	g	g	c	a	a	c	t	t	g	a	g	t	t	a	t	a	t	t	t	a	g	t	t	c	g	t	t	t	a	c	a	c	g	t	a	c	t	a	a	a	c	c	g	t	t	g	t	c	t	a	g	a	a	a	g	t	g
MH709360	5	a	t	c	g	c	c	t	c	t	g	t	t	a	t	t	c	g	g	g	c	a	a	c	t	t	g	a	g	t	t	a	t	a	t	t	t	a	g	t	t	c	g	t	t	t	a	c	a	c	g	t	a	c	t	a	a	a	c	c	g	t	t	g	t	c	t	a	g	a	a	a	g	t	g
MH709362	5	a	t	c	g	c	c	t	c	t	g	t	t	a	t	t	c	g	g	g	c	a	a	c	t	t	g	a	g	t	t	a	t	a	t	t	t	a	g	t	t	c	g	t	t	c	a	c	a	c	g	t	a	c	t	a	a	a	c	c	g	t	t	g	t	c	t	a	g	a	a	a	g	t	g
MH709365	5	a	t	c	g	c	c	t	c	t	g	t	t	a	t	t	c	g	g	g	c	a	a	c	t	t	g	a	g	t	t	a	t	a	t	t	t	a	g	t	t	c	g	t	t	t	a	c	a	c	g	t	a	c	t	a	a	a	c	c	g	t	t	g	t	c	t	a	g	a	a	a	g	t	g
MH709373	5	a	t	c	g	c	c	t	c	t	g	t	t	a	t	t	c	g	g	g	c	a	a	c	t	t	g	a	g	t	t	a	t	a	t	t	t	a	g	t	t	c	g	t	t	t	a	c	a	c	g	t	a	c	t	a	a	a	c	c	g	t	t	g	t	c	t	a	g	a	a	a	g	t	g
MH709374	5	a	t	c	g	c	c	t	c	t	g	t	t	a	t	t	c	g	g	g	c	a	a	c	t	t	g	a	g	t	t	a	t	a	t	t	t	a	g	t	t	c	g	t	t	t	a	c	a	c	g	t	a	c	t	a	a	a	c	c	g	t	t	g	t	c	t	a	g	a	a	a	g	t	g
KC112914	6	a	t	c	g	c	c	t	c	t	g	t	t	a	t	g	c	a	g	g	c	a	a	c	t	t	g	a	g	t	t	a	t	a	t	t	t	a	g	t	t	c	g	g	t	t	a	a	a	c	g	t	a	c	t	a	c	a	c	t	g	t	c	g	t	c	t	a	g	a	a	a	g	t	g
KP771914	6	a	t	c	g	c	c	t	c	t	g	t	t	a	t	g	c	a	g	g	c	a	a	c	t	t	g	a	g	t	t	a	t	a	t	t	t	a	g	t	t	c	g	g	t	t	a	a	a	c	g	t	a	c	t	a	c	a	c	t	g	t	c	g	t	c	t	a	g	a	a	a	g	t	g
KP702725	6	a	t	c	g	c	c	t	c	t	g	t	t	a	t	g	c	a	g	g	c	a	a	c	t	t	g	a	g	t	t	a	t	a	t	t	t	a	g	t	t	c	g	g	t	t	a	a	a	c	g	t	a	c	t	a	c	a	c	t	g	t	c	g	t	c	t	a	g	a	a	a	g	t	g
KP771890	9	a	t	c	g	c	c	t	c	t	g	t	t	a	t	g	c	a	g	g	c	a	a	c	t	t	g	a	g	t	t	a	t	a	t	g	t	a	g	t	t	c	g	t	t	t	a	c	a	c	g	t	a	c	t	a	a	a	c	t	g	c	c	g	t	c	t	a	g	a	a	a	g	t	g
JN704710	9	a	t	c	g	c	c	t	c	t	g	t	t	g	t	g	c	g	g	g	c	a	a	c	t	t	g	a	g	t	c	a	t	a	t	t	t	a	g	t	t	c	g	t	t	t	a	c	a	c	g	t	a	c	t	a	a	a	c	c	g	t	c	g	t	c	t	a	g	a	a	a	g	t	g
KY037798	9	a	t	c	g	c	c	t	c	t	g	t	t	a	t	g	c	a	g	g	c	a	a	c	t	t	g	a	g	t	t	a	t	a	t	g	t	a	g	t	t	c	g	t	t	t	a	c	a	c	g	t	a	c	t	a	a	a	t	t	g	t	c	g	t	c	t	a	g	a	a	a	g	t	g
MH709312	9	a	t	c	g	c	c	t	c	t	g	t	t	a	t	g	c	a	g	g	c	a	a	c	t	t	g	a	g	t	t	a	t	a	t	g	t	a	g	t	t	c	g	t	t	t	a	c	a	c	g	t	a	c	t	a	a	a	t	t	g	t	c	g	t	c	t	a	g	a	a	a	g	t	g
MH709352	9	a	t	c	g	t	c	t	c	t	g	t	t	a	t	g	c	a	g	g	c	a	a	c	t	t	g	a	g	t	t	a	t	a	t	g	t	a	g	t	t	c	g	t	t	t	a	c	a	c	g	t	a	c	t	a	a	a	t	t	g	t	c	g	t	c	t	a	g	a	a	a	g	t	g
AB097933	Vac	a	t	c	g	c	c	t	c	t	g	t	t	a	t	t	c	g	g	g	c	a	a	c	t	t	g	a	t	t	t	a	t	a	t	t	t	a	g	t	t	c	g	t	t	t	a	c	a	c	g	t	a	c	t	a	a	a	c	c	g	t	c	g	t	c	t	a	g	a	a	a	g	t	g
JN704698	Vac	a	t	c	g	c	c	t	c	t	g	t	t	a	t	t	c	g	g	g	c	a	a	c	t	t	g	a	t	t	t	a	t	a	t	t	t	a	g	t	t	c	g	t	t	t	a	c	a	c	g	t	a	c	t	a	a	a	c	c	g	t	c	g	t	c	t	a	g	a	a	a	g	t	g
JN704697	Vac	a	t	c	g	c	c	t	c	t	g	t	t	a	t	t	c	g	g	g	c	a	a	c	t	t	g	a	t	t	t	a	t	a	t	t	t	a	g	t	t	c	g	t	t	t	a	c	a	c	g	t	a	c	t	a	a	a	c	c	g	t	c	g	t	c	t	a	g	a	a	a	g	t	g
DQ008355	Vac	a	t	c	g	c	c	t	c	t	g	t	t	a	t	t	c	g	g	g	c	a	a	c	t	t	g	a	t	t	t	a	t	a	t	t	t	a	g	t	t	c	g	t	t	t	a	c	a	c	g	t	a	c	t	a	a	a	c	c	g	t	c	g	t	c	t	a	g	a	a	a	g	t	g
JF306641	Vac	a	t	c	g	c	c	t	c	t	g	t	t	a	t	t	c	g	g	g	c	a	a	c	t	t	g	a	t	t	t	a	t	a	t	t	t	a	g	t	t	c	g	t	t	t	a	c	a	c	g	t	a	c	t	a	a	a	c	c	g	t	c	g	t	c	t	a	g	a	a	a	g	t	g
AB097932	Vac	a	t	c	g	c	c	t	c	t	g	t	t	a	t	t	c	g	g	g	c	a	a	c	t	t	g	a	t	t	t	a	t	a	t	t	t	a	g	t	t	c	g	t	t	t	a	c	a	c	g	t	a	c	t	a	a	a	c	c	g	t	c	g	t	c	t	a	g	a	a	a	g	t	g
DQ008354	Vac	a	t	c	g	c	c	t	c	t	g	t	t	a	t	t	c	g	g	g	c	a	a	c	t	t	g	a	t	t	t	a	t	a	t	t	t	a	g	t	t	c	g	t	t	t	a	c	a	c	g	t	a	c	t	a	a	a	c	c	g	t	c	g	t	c	t	a	g	a	a	a	g	t	g
KU926312	Vac	a	t	c	g	c	c	t	c	t	g	t	t	a	t	t	c	g	g	g	c	a	a	c	t	t	g	a	t	t	t	a	t	a	t	t	t	a	g	t	t	c	g	t	t	t	a	c	a	c	g	t	a	c	t	a	a	a	c	c	g	t	c	g	t	c	t	a	g	a	a	a	g	t	g
KU926313	Vac	a	t	c	g	c	c	t	c	t	g	t	t	a	t	t	c	g	g	g	c	a	a	c	t	t	g	a	t	t	t	a	t	a	t	t	t	a	g	t	t	c	g	t	t	t	a	c	a	c	g	t	a	c	t	a	a	a	c	c	g	t	c	g	t	c	t	a	g	a	a	a	g	t	g
KU926314	Vac	a	t	c	g	c	c	t	c	t	g	t	t	a	t	t	c	g	g	g	c	a	a	c	t	t	g	a	t	t	t	a	t	a	t	t	t	a	g	t	t	c	g	t	t	t	a	c	a	c	g	t	a	c	t	a	a	a	c	c	g	t	c	g	t	c	t	a	g	a	a	a	g	t	g
KF558371	Vac	a	t	c	g	c	c	t	c	t	g	t	t	a	t	t	c	g	g	g	c	a	a	c	t	t	g	a	t	t	t	a	t	a	t	t	t	a	g	t	t	c	g	t	t	t	a	c	a	c	g	t	a	c	t	a	a	a	c	c	g	t	c	g	t	c	t	a	g	a	a	a	g	t	g
KF558372	Vac	a	t	c	g	c	c	t	c	t	g	t	t	a	t	t	c	g	g	g	c	a	a	c	t	t	g	a	t	t	t	a	t	a	t	t	t	a	g	t	t	c	g	t	t	t	a	c	a	c	g	t	a	c	t	a	a	a	c	c	g	t	c	g	t	c	t	a	g	a	a	a	g	t	g
KF558373	Vac	a	t	c	g	c	c	t	c	t	g	t	t	a	t	t	c	g	g	g	c	a	a	c	t	t	g	a	t	t	t	a	t	a	t	t	t	a	g	t	t	c	g	t	t	t	a	c	a	c	g	t	a	c	t	a	a	a	c	c	g	t	c	g	t	c	t	a	g	a	a	a	g	t	g
KF558374	Vac	a	t	c	g	c	c	t	c	t	g	t	t	a	t	t	c	g	g	g	c	a	a	c	t	t	g	a	t	t	t	a	t	a	t	t	t	a	g	t	t	c	g	t	t	t	a	c	a	c	g	t	a	c	t	a	a	a	c	c	g	t	c	g	t	c	t	a	g	a	a	a	g	t	g
KF558375	Vac	a	t	c	g	c	c	t	c	t	g	t	t	a	t	t	c	g	g	g	c	a	a	c	c	t	g	a	t	t	t	a	t	a	t	t	t	a	g	t	t	c	g	t	t	t	a	c	a	c	g	t	a	c	t	a	a	a	c	c	g	t	c	g	t	c	t	a	g	a	a	a	g	t	g
KF558376	Vac	a	t	c	g	c	c	t	c	t	g	t	t	a	t	t	c	g	g	g	c	a	a	c	t	t	g	a	t	t	t	a	t	a	t	t	t	a	g	t	t	c	g	t	t	t	a	c	a	c	g	t	a	c	t	a	a	a	c	c	g	t	c	g	t	c	t	a	g	a	a	a	g	t	g
KF558377	Vac	a	t	c	g	c	c	t	c	t	g	t	t	a	t	t	c	g	g	g	c	a	a	c	t	t	g	a	t	t	t	a	t	a	t	t	t	a	g	t	t	c	g	t	t	t	a	c	a	c	g	t	a	c	t	a	a	a	c	c	g	t	c	g	t	c	t	a	g	a	a	a	g	t	g
KF558378	Vac	a	t	c	g	c	c	t	c	t	g	t	t	a	t	t	c	g	g	g	c	a	a	c	t	t	g	a	t	t	t	a	t	a	t	t	t	a	g	t	t	c	g	t	t	t	a	c	a	c	g	t	a	c	t	a	a	a	c	c	g	t	c	g	t	c	t	a	g	a	a	a	g	t	g
KF558379	Vac	a	t	c	g	c	c	t	c	t	g	t	t	a	t	t	c	g	g	g	c	a	a	c	t	t	g	a	t	t	t	a	t	a	t	t	t	a	g	t	t	c	g	t	t	t	a	c	a	c	g	t	a	c	t	a	a	a	c	c	g	t	c	g	t	c	t	a	g	a	a	a	g	t	g
KF558380	Vac	a	t	c	g	c	c	t	c	t	g	t	t	a	t	t	c	g	g	g	c	a	a	c	t	t	g	a	t	t	t	a	t	a	t	t	t	a	g	t	t	c	g	t	t	t	a	c	a	c	g	t	a	c	t	a	a	a	c	c	g	t	c	g	t	c	t	a	g	a	a	a	g	t	g
KF558381	Vac	a	t	c	g	c	c	t	c	t	g	t	t	a	t	t	c	g	g	g	c	a	a	c	t	t	g	a	t	t	t	a	t	a	t	t	t	a	g	t	t	c	g	t	t	t	a	c	a	c	g	t	a	c	t	a	a	a	c	c	g	t	c	g	t	c	t	a	g	a	a	a	g	t	g
KF558382	Vac	a	t	c	g	c	c	t	c	t	g	t	t	a	t	t	c	g	g	g	c	a	a	c	t	t	g	a	t	t	t	a	t	a	t	t	t	a	g	t	t	c	g	t	t	t	g	c	a	c	g	c	a	c	t	a	a	a	c	c	g	t	c	g	t	c	t	a	g	a	a	a	g	t	g
KF558383	Vac	a	t	c	g	c	c	t	c	t	g	t	t	a	t	t	c	g	g	g	c	a	a	c	t	t	g	a	t	t	t	a	t	a	t	t	t	a	g	t	t	c	g	t	t	t	a	c	a	c	g	t	a	c	t	a	a	a	c	c	g	t	c	g	t	c	t	a	g	a	a	a	g	t	g
KF558384	Vac	a	t	c	g	c	c	t	c	t	g	t	t	a	t	t	c	g	g	g	c	a	a	c	t	t	g	a	t	t	t	a	t	a	t	t	t	a	g	t	t	c	g	t	t	t	a	c	a	c	g	t	a	c	t	a	a	a	c	c	g	t	c	g	t	c	t	a	g	a	a	a	g	t	g
KF558385	Vac	a	t	c	g	c	c	t	c	t	g	t	t	a	t	t	c	g	g	g	c	a	a	c	t	t	g	a	t	t	t	a	t	a	t	t	t	a	g	t	t	c	g	t	t	t	a	c	a	c	g	t	a	c	t	a	a	a	c	c	g	t	c	g	t	c	t	a	g	a	a	a	g	t	g
KF558386	Vac	a	t	c	g	c	c	t	c	t	g	t	t	a	t	t	c	g	g	g	c	a	a	c	t	t	g	a	t	t	t	a	t	a	t	t	t	a	g	t	t	c	g	t	t	t	a	c	a	c	g	t	a	c	t	a	a	a	c	c	g	t	c	g	t	c	t	a	g	a	a	a	g	t	g
KF558387	Vac	a	t	c	g	c	c	t	c	t	g	t	t	a	t	t	c	g	g	g	c	a	a	c	t	t	g	a	t	t	t	a	t	a	t	t	t	a	g	t	t	c	g	t	t	t	a	c	a	c	g	t	a	c	t	a	a	a	c	c	g	t	c	g	t	c	t	a	g	a	a	a	g	t	g
KF558388	Vac	a	t	c	g	c	c	t	c	t	g	t	t	a	t	t	c	g	g	g	c	a	a	c	t	c	g	a	t	t	t	a	t	a	t	t	t	a	g	t	t	c	g	t	t	t	a	c	a	c	g	t	a	c	t	a	a	a	c	c	g	t	c	g	t	c	t	a	g	a	a	a	g	t	g
KF558389	Vac	a	t	c	g	c	c	t	c	t	g	t	t	a	t	t	c	g	g	g	c	a	a	c	t	t	g	a	t	t	t	a	t	a	t	t	t	a	g	t	t	c	g	t	t	t	a	c	a	c	g	t	a	c	t	a	a	a	c	c	g	t	c	g	t	c	t	a	g	a	a	a	g	t	g
KF558390	Vac	a	t	c	g	c	c	t	c	t	g	t	t	a	t	t	c	g	g	g	c	a	a	c	c	t	g	a	t	t	t	a	t	a	t	t	t	a	g	t	t	c	g	t	t	t	a	c	a	c	g	t	a	c	t	a	a	a	c	c	g	t	c	g	t	c	t	a	g	a	a	a	g	t	g
KF558391	Vac	a	t	c	g	c	c	t	c	t	g	t	t	a	t	t	c	g	g	g	c	a	a	c	t	t	g	a	t	t	t	a	t	a	t	t	t	a	g	t	t	c	g	t	t	t	a	c	a	c	g	t	a	c	t	a	a	a	c	c	g	t	c	g	t	c	t	a	g	a	a	a	g	t	g
KF811485	Vac	a	t	c	g	c	c	t	c	t	g	t	t	a	t	t	c	g	g	g	c	a	a	c	t	t	g	a	t	t	t	a	t	a	t	t	t	a	g	t	t	c	g	t	t	t	a	c	a	c	g	t	a	c	t	a	a	a	c	c	g	t	c	g	t	c	t	a	g	a	a	a	g	t	g
KF853225	Vac	a	t	c	g	c	c	t	c	t	g	t	t	a	t	t	c	g	g	g	c	a	a	c	t	t	g	a	t	t	t	a	t	a	t	t	t	a	g	t	t	c	g	t	t	t	a	c	a	c	g	t	a	c	t	a	a	a	c	c	g	t	c	g	t	c	t	a	g	a	a	a	g	t	g
KF853226	Vac	a	t	c	g	c	c	t	c	t	g	t	t	a	t	t	c	g	g	g	c	a	a	c	t	t	g	a	t	t	t	a	t	a	t	t	t	a	g	t	t	c	g	t	t	t	a	c	a	c	g	t	a	c	t	a	a	a	c	c	g	t	c	g	t	c	t	a	g	a	a	a	g	t	g
KF853227	Vac	a	t	c	g	c	c	t	c	t	g	t	t	a	t	t	c	g	g	g	c	a	a	c	t	t	g	a	t	t	t	a	t	a	t	t	t	a	g	t	t	c	g	t	t	t	a	c	a	c	g	t	a	c	t	a	a	a	c	c	g	t	c	g	t	c	t	a	g	a	a	a	g	t	g
KF853228	Vac	a	t	c	g	c	c	t	c	t	g	t	t	a	t	t	c	g	g	g	c	a	a	c	t	t	g	a	t	t	t	a	t	a	t	t	t	a	g	t	t	c	g	t	t	t	a	c	a	c	g	t	a	c	t	a	a	a	c	c	g	t	c	g	t	c	t	a	g	a	a	a	g	t	g
KF853229	Vac	a	t	c	g	c	c	t	c	t	g	t	t	a	t	t	c	g	g	g	c	a	a	c	t	t	g	a	t	t	t	a	t	a	t	t	t	a	g	t	t	c	g	t	t	t	a	c	a	c	g	t	a	c	t	a	a	a	c	c	g	t	c	g	t	c	t	a	g	a	a	a	g	t	g
KF853230	Vac	a	t	c	g	c	c	t	c	t	g	c	t	a	t	t	c	g	g	g	c	a	a	c	t	t	g	a	t	t	t	a	t	a	t	t	t	a	g	t	t	c	g	t	t	t	a	c	a	c	g	t	a	c	t	a	a	a	c	c	g	t	c	g	t	c	t	a	g	a	a	a	g	t	g
KF853231	Vac	a	t	c	g	c	c	t	c	t	g	t	t	a	t	t	c	g	g	g	c	a	a	c	t	t	g	a	t	t	t	a	t	a	t	t	t	a	g	t	t	c	g	t	t	t	a	c	a	c	g	t	a	c	t	a	a	a	c	c	g	t	c	g	t	c	t	a	g	a	a	a	g	t	g
KF853232	Vac	a	t	c	g	c	c	t	c	t	g	t	t	a	t	t	c	g	g	g	c	a	a	c	t	t	g	a	t	t	t	a	t	a	t	t	t	a	g	t	t	c	g	t	t	t	a	c	a	c	g	t	a	c	t	a	a	a	c	c	g	t	c	g	t	c	t	a	g	a	a	a	g	t	g
KF853233	Vac	a	t	c	g	c	c	t	c	t	g	t	t	a	t	t	c	g	g	g	c	a	a	c	t	t	g	a	t	t	t	a	t	a	t	t	t	g	g	t	t	c	g	t	t	t	a	c	a	c	g	t	a	c	t	c	a	a	c	c	g	t	c	g	t	c	t	a	g	a	a	a	g	t	g
KF853234	Vac	a	t	c	g	c	c	t	c	t	g	t	t	a	t	t	c	g	g	g	c	a	a	c	t	t	g	a	t	t	t	a	t	a	t	t	t	a	g	t	t	c	g	t	t	t	a	c	a	c	g	t	a	c	t	a	a	a	c	c	g	t	c	g	t	c	t	a	g	a	a	a	g	t	g
KF853235	Vac	a	t	c	g	c	c	t	c	t	g	t	t	a	t	t	c	g	g	g	c	a	a	c	t	t	g	a	t	t	t	a	t	a	t	t	t	a	g	t	t	c	g	t	t	t	a	c	a	c	g	t	a	c	t	a	a	a	c	c	g	t	c	g	t	c	t	a	g	a	g	a	g	t	g
MF898328	Vac	a	t	c	g	c	c	t	c	t	g	t	t	a	t	t	c	g	g	g	c	a	a	c	t	t	g	a	t	t	t	a	t	a	t	t	t	a	g	t	t	c	g	t	t	t	a	c	a	c	g	t	a	c	t	a	a	a	c	c	g	t	c	g	t	c	t	a	g	a	a	a	g	t	g
JN704709	VIII	a	t	c	g	c	c	t	c	t	g	t	t	a	t	g	c	g	g	g	c	a	a	c	t	t	g	a	g	t	t	a	t	a	t	t	t	a	g	t	t	c	g	g	t	t	a	a	a	c	g	t	a	c	t	a	c	a	c	t	g	t	c	g	t	c	t	a	g	a	a	a	g	t	g

Supplementary Table 3: 2880 SN

GB-ID	Clade markers																																																																											
	Position in Dumas	69275	69277	69286	69328	69349	69405	69424	69457	69619	69623	69751	69756	70014	70057	70198	70344	70350	70479	70586	70651	70664	70694	70794	70817	70848	71167	71196	71252	71262	71304	71324	71575	71618	71626	71818	71981	72092	72127	72219	72277	72283	72288	72349	72395	72403	72418	72429	72469	72526	72605	72613	72970	72997	73060	73145	73170	73181	73225	73417	73432	73433	73533	73561	73645	73669	73846	73993	74042	74071	74123	74233	74251	74293	74360
AY548170	1	g	c	a	g	a	t	g	g	t	a	c	t	c	t	g	t	c	g	a	c	a	a	a	g	g	t	c	t	t	t	g	t	g	c	t	g	t	t	g	c	c	g	g	g	g	a	g	c	c	t	a	c	c	c	c	t	a	c	c	g	g	c	a	c	a	a	c	a	c	g	c	c	a	a
AY548171	1	g	c	a	g	a	t	g	g	t	a	c	c	c	t	g	t	c	g	a	c	a	a	a	g	g	t	c	t	t	t	g	t	g	c	t	g	t	t	g	c	c	g	g	g	g	a	g	c	c	t	a	c	c	c	c	t	a	c	c	g	g	c	a	c	a	a	c	a	c	g	c	c	a	a
DQ479953	1	g	c	a	g	a	t	g	g	t	a	c	t	c	t	g	t	t	g	a	c	a	a	a	g	g	t	c	t	t	t	g	t	g	c	t	g	t	t	g	c	c	g	g	g	g	a	g	c	c	t	a	c	c	c	c	t	a	c	c	g	g	c	a	c	a	a	c	a	c	g	c	c	a	a
DQ479954	1	g	c	a	g	a	t	g	g	t	a	c	t	c	t	g	t	c	g	a	c	a	a	a	g	g	t	c	t	t	t	g	t	g	c	t	g	t	t	g	c	c	g	g	g	g	a	g	c	c	t	a	c	c	c	c	t	a	c	c	g	g	c	a	c	a	a	c	a	a	g	c	c	a	a
DQ479958	1	g	c	a	g	a	t	g	g	t	a	c	c	c	t	g	t	c	g	a	c	a	a	a	g	g	t	c	t	t	t	g	t	g	c	t	g	t	t	g	c	c	g	g	g	g	a	g	c	c	t	a	c	c	c	c	t	a	c	c	g	g	c	a	c	a	a	c	a	c	g	c	c	a	a
DQ479959	1	g	c	a	g	a	t	g	g	t	a	c	c	c	t	g	t	c	g	a	c	a	a	a	g	g	t	c	t	t	t	g	t	g	c	t	g	t	t	g	c	c	g	g	g	g	a	g	c	c	t	a	c	c	c	c	t	a	c	c	g	g	c	a	c	a	a	c	a	c	g	c	c	a	a
DQ479961	1	g	c	a	g	a	t	g	g	t	a	c	t	c	t	g	t	c	g	a	c	a	a	a	g	g	t	c	t	t	t	g	t	g	c	t	g	t	t	g	c	c	g	g	g	g	a	g	c	c	t	a	c	c	c	c	t	a	c	c	g	g	c	a	c	a	a	c	a	a	g	c	c	a	a
DQ479962	1	g	c	a	g	a	t	g	g	t	a	c	t	c	t	g	t	c	g	a	c	a	a	a	g	g	t	c	t	t	t	g	t	g	c	t	g	t	t	g	c	c	g	g	g	g	a	g	c	c	t	a	c	c	c	c	t	a	c	c	g	g	c	a	c	a	a	c	a	a	g	c	c	a	a
DQ479963	1	g	c	a	g	a	t	g	g	t	a	c	t	c	t	g	t	c	g	a	c	a	a	a	g	g	t	c	t	t	t	g	t	g	c	t	g	t	t	g	c	c	g	g	g	g	a	g	c	c	t	a	c	c	c	c	t	a	c	c	g	g	c	a	c	a	a	c	a	a	g	c	c	a	a
DQ674250	1	g	c	a	g	a	t	g	g	t	a	c	c	c	t	g	t	c	g	a	c	a	a	a	g	g	t	c	t	t	t	g	t	g	c	t	g	t	t	g	c	c	g	g	g	g	a	g	c	c	t	a	c	t	c	c	t	a	c	c	g	g	c	a	c	a	a	c	a	c	g	c	c	a	a
KX262864	1	g	c	a	g	a	t	g	g	t	a	c	c	c	t	g	t	c	g	a	c	c	a	a	g	g	t	c	t	t	t	g	t	g	c	t	g	t	t	g	c	c	g	g	g	g	a	g	c	c	t	a	c	c	c	c	t	a	c	c	g	g	c	a	c	a	a	c	a	c	g	c	c	a	a
KX262865	1	g	c	a	g	a	t	g	g	t	a	c	c	c	t	g	t	c	g	a	c	a	a	a	g	g	t	c	t	t	t	g	t	g	c	t	g	t	t	g	c	c	g	g	g	g	a	g	c	c	t	a	c	c	c	c	t	a	c	c	g	g	c	a	c	a	a	c	a	c	g	c	c	a	a
KX262866	1	g	c	a	g	a	t	g	g	t	a	c	t	c	t	g	t	c	g	a	c	a	a	a	g	g	t	c	t	t	t	g	t	g	c	t	g	t	t	g	c	c	g	g	g	g	a	g	c	c	t	a	c	c	c	c	t	a	c	c	g	g	c	a	c	a	a	c	a	c	g	c	c	a	a
EU154348	1	g	c	a	g	a	t	g	g	t	a	c	t	c	t	g	t	c	g	a	c	a	a	a	g	g	t	c	t	t	t	g	t	g	c	t	g	t	t	g	c	c	g	g	g	g	a	g	c	c	t	a	c	c	c	c	t	a	c	c	g	g	c	a	c	a	a	c	a	a	g	c	c	a	a
JN704690	1	g	c	a	g	a	t	g	g	t	a	c	t	c	t	g	t	c	g	a	c	a	a	a	g	g	t	c	t	t	t	g	t	g	c	t	g	t	t	g	c	c	g	g	g	g	a	g	c	c	t	a	c	c	c	c	t	a	c	c	g	g	c	a	c	a	a	c	a	c	g	c	c	a	a
JN704691	1	g	c	a	g	a	t	g	g	t	a	c	t	c	t	g	t	c	g	a	c	a	a	a	g	g	t	c	t	c	t	g	t	g	c	t	g	t	t	g	c	c	g	g	g	g	c	g	c	c	t	a	c	c	c	c	t	a	c	c	g	g	c	a	c	a	a	t	a	c	g	c	c	a	a
JN704692	1	g	c	a	g	a	t	g	g	t	a	c	t	c	t	g	t	c	g	a	c	a	a	a	g	g	t	c	t	t	t	g	t	g	c	t	g	t	t	g	c	c	g	g	g	g	a	g	c	c	t	a	c	c	c	c	t	a	c	c	g	g	c	a	c	a	a	c	a	c	g	c	c	a	a
JN704693	1	g	c	a	g	a	t	g	g	t	a	c	t	c	t	g	t	c	g	a	c	a	a	a	g	g	t	c	t	t	t	g	t	g	c	t	g	t	t	g	c	c	g	g	g	g	a	g	c	c	t	a	c	c	c	c	t	a	c	c	g	g	c	a	c	a	a	c	a	c	g	c	c	a	a
JN704694	1	g	c	a	g	a	t	g	g	t	a	c	t	c	t	g	t	c	g	a	c	a	a	a	g	g	t	c	t	t	t	g	t	g	c	t	g	t	t	g	c	c	g	g	g	g	a	g	c	c	t	a	c	c	c	c	t	a	c	c	g	g	c	a	c	a	a	c	a	c	g	c	c	a	a
JN704695	1	g	c	a	g	a	t	g	g	t	a	c	t	c	t	g	t	c	g	a	c	a	a	a	g	g	t	c	t	t	t	g	t	g	c	t	g	t	t	g	c	c	g	g	g	g	a	g	c	c	t	a	c	c	c	c	t	a	c	c	g	g	c	a	c	a	a	c	a	c	g	c	c	a	a
JN704696	1	g	c	a	g	a	t	g	g	t	a	c	t	c	t	g	t	c	g	a	c	a	a	a	g	g	t	c	t	t	t	g	t	g	c	t	g	t	t	g	c	c	g	g	g	g	a	g	c	c	t	a	c	c	c	c	t	a	c	c	g	g	c	a	c	a	a	c	a	c	g	c	c	a	a
NC001348	1	g	c	a	g	a	t	g	g	t	a	c	t	c	t	g	t	c	g	a	c	a	a	a	g	g	t	c	t	t	t	g	t	g	c	t	g	t	t	g	c	c	g	g	g	g	a	g	c	c	t	a	c	c	c	c	t	a	c	c	g	g	c	a	c	a	a	t	a	c	g	c	c	a	a
KP771892	1	g	c	a	g	a	t	g	g	t	a	c	t	c	t	g	t	c	g	a	c	a	a	a	g	g	t	c	t	t	t	g	t	g	c	t	g	t	t	g	c	c	g	g	g	g	a	g	c	c	t	a	c	c	c	c	t	a	c	c	g	g	c	a	c	a	a	c	a	c	g	c	c	a	a
KP771894	1	g	c	a	g	a	t	g	g	t	a	c	t	c	t	g	t	c	g	a	c	a	a	a	g	g	t	c	t	t	t	g	t	g	c	t	g	t	t	g	c	c	g	g	g	g	a	g	c	c	t	a	c	c	c	c	t	a	c	c	g	g	c	a	c	a	a	c	a	c	g	c	c	a	a
KP771900	1	g	c	a	g	a	t	g	g	t	a	c	t	c	t	g	t	c	g	a	c	a	a	a	g	g	t	c	t	t	t	g	t	g	c	t	g	t	t	g	c	c	g	g	g	g	a	g	c	c	t	a	c	c	c	c	t	a	c	c	g	g	c	a	c	a	a	c	a	c	g	c	c	a	a
KP771901	1	g	c	a	g	a	t	g	g	t	a	c	t	c	t	g	t	c	g	a	c	a	a	a	g	g	t	c	t	t	t	g	t	g	t	t	g	t	t	g	c	c	g	g	g	g	a	g	c	c	t	a	c	c	c	c	t	a	c	c	g	g	c	a	c	a	a	c	a	c	g	c	c	a	a
KP771902	1	g	c	a	g	a	t	g	g	t	a	c	t	c	t	g	t	c	g	a	c	a	a	a	g	g	t	c	t	t	t	g	t	g	c	t	g	t	t	g	c	c	g	g	g	g	a	g	c	c	t	a	c	c	c	c	t	a	c	c	g	g	c	a	c	a	a	c	a	c	g	c	c	a	a
KP771908	1	g	c	a	g	a	t	g	g	t	a	c	c	c	t	g	t	c	g	a	c	a	c	a	g	g	t	c	t	t	t	g	t	g	c	t	g	t	t	g	c	c	g	g	g	g	a	g	c	c	t	a	c	c	c	c	t	a	c	c	g	g	c	a	c	a	a	c	a	c	g	c	c	a	a
KP771912	1	g	c	a	g	a	t	g	g	t	a	c	c	c	t	g	t	c	g	a	c	a	a	a	g	g	t	c	t	t	t	g	t	g	c	t	g	t	t	g	c	c	g	g	g	g	a	g	c	c	t	a	c	c	c	c	t	a	c	c	g	g	c	a	c	a	a	c	a	c	g	c	c	a	a
KP771913	1	g	c	a	g	a	t	g	g	t	a	c	c	c	t	g	t	c	g	a	c	a	a	a	g	g	t	c	t	t	t	g	t	g	c	t	g	t	t	g	c	c	g	g	g	g	a	g	c	c	t	a	c	c	c	c	t	a	c	c	g	g	c	a	c	a	a	c	a	c	g	c	c	a	a
KP771917	1	g	c	a	g	a	t	g	g	t	a	c	t	c	t	g	t	c	g	a	c	a	a	a	g	g	t	c	t	t	t	g	t	g	c	t	g	t	t	g	c	c	g	g	g	g	a	g	c	c	t	a	c	c	c	c	t	a	c	c	g	g	c	a	c	a	a	c	a	c	g	c	c	a	a
KP771918	1	g	c	a	g	a	t	g	g	t	a	c	t	c	t	g	t	c	g	a	c	a	a	a	g	g	t	c	t	t	t	g	t	g	c	t	g	t	t	g	c	c	g	g	g	g	a	g	c	c	t	a	c	c	c	c	t	a	c	c	g	g	c	a	c	a	a	c	a	c	g	c	c	a	a
KP771920	1	g	c	a	g	a	t	g	g	t	a	c	t	c	t	g	t	c	g	a	c	a	a	a	g	g	t	c	t	t	t	g	t	g	c	t	g	t	t	g	c	c	g	g	g	g	a	g	c	c	t	a	c	c	c	c	t	a	c	c	g	g	c	a	c	a	a	c	a	c	g	c	c	a	a
MH709361	1	g	c	a	g	a	t	g	g	t	a	c	c	c	t	g	t	c	g	a	c	a	a	a	g	g	t	c	t	t	t	g	t	g	c	t	g	t	t	g	c	c	g	g	g	g	a	g	c	c	t	a	c	c	c	c	t	a	c	c	g	g	c	a	c	a	a	c	a	c	g	c	c	a	a
KU926318	2	g	c	g	g	a	t	g	g	t	a	c	c	c	t	g	t	c	g	a	c	a	a	a	g	g	t	c	t	t	t	g	t	g	c	t	g	t	t	g	c	c	g	g	g	g	a	g	c	c	t	a	c	t	c	c	t	a	c	c	g	g	c	a	c	a	a	c	a	c	g	c	c	a	a
KU926319	2	g	c	g	g	a	t	g	g	t	a	c	c	c	t	g	t	c	g	a	c	a	a	a	g	g	t	c	t	t	c	g	t	g	c	t	g	t	t	g	c	c	g	g	g	g	a	g	c	c	t	a	c	t	c	c	t	a	c	c	g	g	c	a	c	a	a	c	a	c	g	c	c	a	a
KU926320	2	g	c	a	g	a	t	g	g	t	a	c	c	c	t	g	t	c	g	a	c	a	a	a	g	g	t	c	t	t	t	g	t	g	c	t	g	t	t	g	c	c	g	g	g	g	a	g	c	c	t	a	c	t	c	c	t	a	c	c	g	g	c	a	c	a	a	c	a	c	g	c	c	a	a
KU926321	2	g	c	a	g	a	t	g	g	t	a	c	c	c	t	g	t	c	g	a	c	a	a	a	g	g	t	c	t	t	t	g	t	g	c	t	g	t	t	g	c	c	g	g	g	g	a	g	c	c	t	a	c	t	c	c	t	a	c	c	g	g	c	a	c	a	a	c	a	c	g	c	c	a	a
KU926322	2	g	c	a	g	a	t	g	g	t	a	c	c	c	t	g	t	c	g	a	c	a	a	a	g	g	t	c	t	t	t	g	t	g	c	t	g	t	t	g	c	c	g	g	g	g	a	g	c	c	t	a	c	c	c	c	t	a	c	c	g	g	c	a	c	a	a	c	a	c	g	c	c	a	a
KC847290	2	g	c	a	g	a	t	g	g	t	a	c	c	c	t	g	c	c	g	a	c	a	a	a	g	g	t	c	t	t	t	g	t	g	c	t	g	t	t	g	c	c	g	g	g	g	a	g	c	c	t	a	c	t	c	t	t	a	c	c	g	g	c	a	c	a	a	c	a	c	g	c	c	a	a
KJ767491	2	g	c	a	g	a	t	g	g	t	a	c	c	c	t	g	t	c	g	a	c	a	a	a	g	g	t	c	t	t	t	g	t	g	c	t	g	t	t	g	c	c	g	g	g	g	a	g	c	c	t	a	c	t	c	c	t	a	c	c	g	g	c	a	c	a	a	c	a	c	g	c	c	a	a
KJ767492	2	g	c	a	g	a	t	g	g	t	a	c	c	c	t	g	t	c	g	a	c	a	a	a	g	g	t	c	t	t	t	g	t	g	c	t	g	t	t	g	c	c	g	g	g	g	a	g	c	c	t	a	c	t	c	c	t	a	c	c	g	g	c	a	c	a	a	c	a	c	g	c	c	a	a
KJ808816	2	g	c	a	g	a	t	g	g	t	a	c	c	c	t	g	t	c	g	a	c	a	a	a	g	g	t	c	t	t	t	g	t	g	c	t	g	t	t	g	c	c	g	g	g	g	a	g	c	c	t	a	c	t	c	c	t	a	c	c	g	g	c	a	c	a	a	c	a	c	g	c	c	a	a
KP771925	2	g	c	a	g	a	t	g	g	t	a	c	c	c	t	g	t	c	g	a	c	a	a	a	g	g	t	c	t	t	t	g	t	g	c	t	g	t	t	g	c	c	g	g	g	g	a	g	c	c	t	a	c	t	c	c	t	a	c	c	g	g	c	a	c	a	a	c	a	c	g	c	c	a	a
KU926315	2	g	c	a	g	a	t	g	g	t	a	c	c	c	t	g	t	c	g	a	c	a	a	a	g	g	t	c	t	t	t	g	t	g	c	t	g	t	t	g	c	c	g	g	g	g	a	g	c	c	t	a	c	t	c	c	t	a	c	c	g	g	c	a	c	a	a	c	a	c	g	c	c	a	a
KU926316	2	g	c	a	g	a	t	g	g	t	a	c	c	c	t	g	t	c	g	a	c	a	a	a	g	g	t	c	t	t	t	g	t	g	c	t	g	t	t	g	c	c	g	g	g	g	a	g	c	c	t	a	c	t	c	c	t	a	c	c	g	g	c	a	c	a	a	c	a	c	g	c	c	a	a
KU926317	2	g	c	a	g	a	t	g	g	t	a	c	c	c	t	g	t	c	g	a	c	a	a	a	g	g	t	c	t	t	t	g	t	g	c	t	g	t	t	g	c	c	g	g	g	g	a	g	c	c	t	a	c	t	c	c	t	a	c	c	g	g	c	a	c	a	a	c	a	c	g	c	c	a	a
KY037796	2	c	c	a	g	a	t	g	g	t	a	c	c	c	t	g	t	c	g	a	c	a	a	a	g	g	t	c	t	t	t	g	t	g	c	t	g	t	t	g	c	c	g	g	g	g	a	g	c	c	t	a	c	t	c	c	t	a	c	c	g	g	c	a	c	a	a	c	a	c	g	c	c	a	a
MF004348	2	g	c	a	g	a	t	g	g	t	a	c	c	c	t	g	t	c	g	a	c	a	a	a	g	g	t	c	t	t	t	g	t	g	c	t	g	t	t	g	c	c	g	g	g	g	a	g	c	c	t	a	c	t	c	c	t	a	c	c	g	g	c	a	c	a	a	c	a	c	g	c	c	a	a
MH709309	2	g	c	a	g	a	t	g	g	t	a	c	c	c	t	g	t	c	g	a	c	a	a	a	g	g	t	c	t	t	t	g	t	g	c	t	g	t	t	a	c	c	g	g	g	g	a	g	c	c	t	a	c	t	c	c	t	a	c	c	g	g	c	g	c	a	a	c	a	c	g	c	c	a	a
MH709310	2	g	c	a	g	a	t	g	g	t	a	c	c	c	t	g	t	c	g	a	c	a	a	a	g	g	t	c	t	t	t	g	t	g	c	t	g	c	t	g	c	c	g	g	g	g	a	g	c	c	t	a	c	t	c	c	t	a	c	c	g	g	c	a	c	a	a	c	a	c	g	c	c	a	a
MH709311	2	g	c	a	g	a	t	g	g	t	a	c	c	c	t	g	t	c	g	a	c	a	a	a	g	a	t	c	t	t	t	g	t	g	c	t	g	t	t	g	c	c	g	g	g	g	a	g	c	c	t	a	c	t	c	c	t	a	c	c	g	g	c	a	c	a	a	c	a	c	g	c	c	a	a
MH709313	2	g	c	a	g	a	t	g	g	t	a	c	c	c	t	g	t	c	g	a	c	a	a	a	g	g	t	c	t	t	t	g	t	g	c	t	g	t	t	g	c	c	g	g	g	g	a	g	c	c	t	a	c	t	c	c	t	a	c	c	g	g	c	a	c	a	a	c	a	c	g	c	c	a	a
MH709314	2	g	c	a	g	g	t	g	g	t	a	c	c	c	t	g	t	c	g	a	c	a	a	a	g	g	t	c	t	t	t	g	t	g	c	t	g	t	t	g	c	c	g	g	g	g	a	g	c	c	t	a	c	t	c	c	t	a	c	c	g	g	c	g	c	a	a	c	a	c	g	c	c	a	a
MH709315	2	g	c	a	g	g	t	g	g	t	a	c	c	c	t	g	t	c	g	a	c	a	a	a	g	g	t	c	t	t	t	g	t	g	c	t	g	t	t	a	c	c	g	g	g	g	a	g	c	c	t	a	c	t	c	c	t	a	c	c	g	g	c	g	c	a	a	c	a	c	g	c	c	a	a
MH709319	2	g	c	a	g	g	t	g	g	t	a	c	c	c	t	g	t	c	g	a	c	a	a	a	g	g	t	c	t	t	t	g	t	g	c	t	g	t	t	g	c	c	g	g	g	g	a	g	c	c	t	a	c	t	c	c	t	a	c	c	g	g	c	g	c	a	a	c	a	c	g	c	c	a	a
MH709320	2	g	c	a	g	a	t	g	g	t	a	c	c	c	t	g	t	c	g	a	c	a	a	a	g	g	t	c	t	t	t	g	t	g	c	t	g	t	t	g	c	c	g	g	g	g	a	g	c	c	t	a	c	t	c	c	t	a	c	c	g	g	c	a	c	a	a	c	a	c	g	c	c	a	a
MH709329	2	g	c	a	g	g	t	g	g	t	a	c	c	c	t	g	t	c	g	a	c	a	a	a	g	g	t	c	t	t	t	g	t	g	c	t	g	t	t	g	c	c	g	g	g	g	a	g	c	c	t	a	c	t	c	c	t	a	c	c	g	g	c	g	c	a	a	c	a	c	g	c	c	a	a
MH709331	2	g	c	a	g	a	t	g	g	t	a	c	c	c	t	g	t	c	g	a	c	a	a	a	g	g	t	c	t	t	t	g	t	g	c	t	g	t	t	g	c	c	g	g	g	g	a	g	c	c	t	a	c	t	c	c	t	a	c	c	g	g	c	a	c	a	a	c	a	c	g	c	c	a	a
MH709333	2	g	c	a	g	g	t	g	g	t	a	c	c	c	t	g	t	c	g	a	c	a	a	a	g	g	t	c	t	t	t	g	t	g	c	t	g	t	t	g	c	c	g	g	g	g	a	g	c	c	t	a	c	t	c	c	t	a	c	c	g	g	c	g	c	a	a	c	a	c	g	c	c	a	a
MH709337	2	g	c	a	g	a	t	g	g	t	a	c	c	c	t	g	t	c	g	a	c	a	a	a	g	g	t	c	t	t	t	g	t	g	c	t	g	t	t	g	c	c	g	a	g	g	a	g	c	c	t	a	c	t	c	c	t	a	c	c	g	g	c	a	c	a	a	c	a	c	g	c	c	a	a
MH709339	2	g	c	a	g	a	t	g	g	t	a	c	c	c	t	g	t	c	g	a	c	a	a	a	g	g	t	c	t	t	t	g	t	g	c	t	g	t	t	g	c	c	g	g	g	g	a	g	c	c	t	a	c	t	c	c	g	a	c	c	g	g	c	a	c	a	a	c	a	c	g	c	c	a	a
MH709344	2	g	c	a	g	a	t	g	g	t	a	c	c	c	t	g	t	c	g	a	c	a	a	a	g	g	t	c	t	t	t	g	t	g	c	t	g	t	t	g	c	c	g	g	g	g	a	g	c	c	t	a	c	t	c	c	t	a	c	c	g	g	c	a	c	a	a	c	g	c	g	c	c	a	a
MH709348	2	g	c	a	g	a	t	g	g	t	a	c	c	c	t	g	t	c	g	a	c	a	a	a	g	g	t	c	t	t	t	g	t	g	c	t	g	t	t	g	a	c	a	g	g	g	a	g	c	c	t	a	c	t	c	c	t	a	c	c	g	g	c	a	c	a	a	c	a	c	g	c	c	a	a
MH709355	2	g	c	a	g	a	t	g	g	t	a	c	c	c	t	g	t	c	g	a	c	a	a	a	g	g	t	c	t	t	t	t	t	g	c	t	g	t	t	g	c	c	g	a	g	g	a	g	c	c	t	a	c	t	c	c	t	a	c	c	g	g	c	a	c	a	a	c	a	c	g	c	c	a	a
MH709359	2	g	c	a	g	a	t	g	g	t	a	c	c	c	t	g	t	c	g	a	c	a	a	a	g	g	t	c	t	t	t	g	t	g	c	t	g	t	t	g	c	c	g	g	g	g	a	g	c	c	t	a	c	t	c	c	t	a	c	c	g	g	c	a	c	a	a	c	a	c	g	c	c	a	a
MH709363	2	g	c	a	g	a	t	g	g	t	a	c	c	c	t	g	t	c	g	a	c	a	a	a	g	g	t	c	t	t	t	g	t	g	c	t	g	t	t	g	c	c	g	g	g	g	a	g	c	c	t	a	c	t	c	c	t	a	c	c	g	g	c	a	c	a	a	c	a	c	g	c	c	a	a
MH709366	2	g	c	a	g	a	t	g	g	t	a	c	c	c	t	g	t	c	g	a	c	a	a	a	g	g	c	c	t	t	t	g	t	g	c	t	g	t	t	g	c	c	g	a	g	g	a	g	c	c	t	a	c	t	c	c	t	a	c	c	g	g	c	a	c	a	a	c	a	c	g	c	c	a	a
MH709376	2	g	c	a	g	a	t	g	g	t	a	c	c	c	t	g	t	c	g	a	c	a	a	a	g	g	t	c	t	t	t	g	t	g	c	t	g	t	t	g	c	c	g	a	g	g	a	g	c	c	t	a	c	t	c	c	t	a	c	c	g	g	c	a	c	a	a	c	a	c	g	c	c	a	a
MH709377	2	g	c	a	g	a	t	g	g	t	a	c	c	c	t	g	t	c	g	a	c	a	a	a	g	g	t	c	t	t	t	g	t	g	c	t	g	t	t	g	c	c	g	g	g	g	a	g	c	c	t	a	c	t	c	c	t	a	c	c	g	g	c	a	c	a	a	c	a	c	g	c	c	a	a
**MW316406**	2	g	c	a	g	a	t	g	g	t	a	c	c	c	t	g	t	c	g	a	c	a	a	a	g	g	t	c	t	t	t	g	t	g	c	t	g	t	t	g	c	c	g	g	g	g	a	g	c	c	t	a	c	t	c	c	t	a	c	c	g	g	c	a	c	a	a	c	a	c	g	c	c	a	a
AJ871403	3	g	c	a	g	a	t	a	g	t	a	c	c	a	t	g	t	c	g	a	c	a	a	a	g	g	t	t	t	t	t	g	t	g	c	t	g	t	t	g	c	c	g	g	g	g	a	g	c	c	t	a	c	c	c	c	t	a	c	c	g	g	c	a	c	a	a	c	a	c	g	c	t	g	a
DQ479955	3	g	c	a	g	a	t	a	g	t	a	c	c	a	t	g	t	c	g	a	g	a	a	a	g	g	t	t	t	t	t	g	t	g	c	t	g	t	t	g	c	c	g	g	g	g	a	g	c	c	t	a	c	c	c	c	t	a	c	c	g	g	c	a	c	a	a	c	a	c	g	c	c	g	a
DQ479956	3	g	c	a	g	a	t	a	g	t	a	c	c	a	t	g	t	c	g	a	c	a	a	a	g	g	t	t	t	t	t	g	t	g	c	t	g	t	t	g	c	c	g	g	g	g	a	g	c	c	t	a	c	c	c	c	t	a	c	c	g	g	c	a	c	a	a	c	a	c	g	c	c	g	a
DQ479957	3	g	c	a	g	a	t	a	g	t	a	c	c	a	t	g	t	c	g	a	c	a	a	a	g	g	t	t	t	t	t	g	t	g	c	t	g	t	t	g	c	c	g	g	g	g	a	g	c	c	t	a	c	c	c	c	t	a	c	c	g	g	c	a	c	a	a	c	a	c	g	c	c	g	a
JN704699	3	g	c	a	g	a	t	a	g	t	a	c	c	a	t	g	t	c	g	a	c	a	a	a	g	g	t	t	t	t	t	g	t	g	c	t	g	t	t	g	c	c	g	g	g	g	a	g	c	c	t	a	c	c	c	c	t	a	c	c	g	g	c	a	c	a	a	c	a	c	g	c	c	g	a
JN704700	3	g	c	a	g	a	t	a	g	t	a	c	c	a	t	g	t	c	g	a	c	a	a	a	g	g	t	t	t	t	t	g	t	g	c	t	g	t	t	g	c	c	g	g	g	g	a	g	c	c	t	a	c	c	t	c	t	a	c	c	g	g	c	a	c	a	a	c	a	c	g	c	c	a	a
JN704701	3	g	c	a	g	a	t	a	g	t	a	c	c	a	t	g	t	c	g	a	c	a	a	a	g	g	t	t	t	t	t	g	t	g	c	t	g	t	t	g	c	c	g	g	g	g	a	a	c	c	t	a	c	c	c	c	t	a	c	c	g	g	c	a	c	a	a	c	a	c	g	c	c	g	a
JN704702	3	g	c	a	g	a	t	a	g	t	a	c	c	a	t	g	t	c	g	a	c	a	a	a	g	g	t	t	t	t	t	g	t	g	c	t	g	t	t	g	c	c	g	g	g	g	a	g	c	c	t	a	c	c	c	c	t	a	c	c	g	g	c	a	c	a	a	c	a	c	g	c	c	a	a
JN704703	3	g	c	a	g	a	t	a	g	t	a	c	c	a	t	g	t	c	g	a	c	a	a	a	g	g	t	t	t	t	t	g	t	g	c	t	g	t	t	g	c	c	g	g	g	g	a	g	c	c	t	a	c	c	c	c	t	a	c	c	g	g	c	a	c	a	a	c	a	c	g	c	c	a	a
KP771891	3	g	c	a	g	a	t	a	g	t	a	c	c	a	t	g	t	c	g	a	c	a	a	a	g	g	t	t	t	t	t	g	t	g	c	t	g	t	t	g	c	c	g	g	g	g	a	g	c	c	t	a	c	c	c	c	t	a	c	c	g	g	c	a	c	a	a	c	a	c	g	c	c	g	a
KP771893	3	g	c	a	g	a	t	a	g	t	a	c	c	a	t	g	t	c	g	a	c	a	a	a	g	g	t	t	t	t	t	g	t	g	c	t	g	t	t	g	c	c	g	g	g	g	a	g	c	c	t	a	c	c	c	c	t	a	c	c	g	g	c	a	c	a	a	c	a	c	g	c	c	g	a
KP771895	3	g	c	a	g	a	t	a	g	t	a	c	c	a	t	g	t	c	g	a	c	a	a	a	g	g	t	t	t	t	t	g	t	g	c	t	g	t	t	g	c	c	g	g	g	g	a	g	c	c	t	a	c	c	c	c	t	a	c	c	g	g	c	a	c	a	a	c	a	c	g	c	t	g	a
KP771896	3	g	c	a	g	a	t	a	g	t	a	c	c	a	t	g	t	c	g	a	c	a	a	a	g	g	t	t	t	t	t	g	t	g	c	t	g	t	t	g	c	c	g	g	g	g	a	g	c	c	t	a	c	c	c	c	t	a	c	c	g	g	c	a	c	a	a	c	a	c	g	c	c	g	a
KP771897	3	g	c	a	g	a	t	a	t	t	a	c	c	a	t	g	t	c	g	a	c	a	a	a	g	g	t	t	t	t	t	g	t	g	c	t	g	t	t	g	c	c	g	g	g	a	a	g	c	c	t	a	c	c	c	c	t	a	c	c	g	g	c	a	c	a	a	c	a	c	g	c	c	g	a
KP771899	3	g	c	a	g	a	t	a	g	t	a	c	c	a	t	g	t	c	g	a	c	a	a	a	g	g	t	t	t	t	t	g	t	g	c	t	g	t	t	g	c	c	g	g	g	g	a	g	c	c	t	a	c	c	c	c	t	a	c	c	g	g	c	a	c	a	a	c	a	c	g	c	t	g	a
KP771903	3	g	c	a	g	a	t	a	g	t	a	c	c	a	t	g	t	c	g	a	c	a	a	a	g	g	t	t	t	t	t	g	t	g	c	t	g	t	t	g	c	c	g	g	g	a	a	g	c	c	t	a	c	c	c	c	t	a	c	c	g	g	c	a	c	a	a	c	a	c	g	c	c	g	a
KP771907	3	g	c	a	g	a	t	a	g	t	a	c	c	a	t	g	t	c	g	a	c	a	a	a	g	g	t	t	t	t	t	g	t	g	c	t	g	t	t	g	c	c	g	g	g	g	a	g	c	c	t	a	c	c	c	c	t	a	c	c	g	g	c	a	c	a	a	c	a	c	g	c	t	g	a
KP771910	3	g	c	a	g	a	t	a	g	t	a	c	c	a	t	g	t	c	g	a	c	a	a	a	g	g	t	t	t	t	t	g	t	g	c	t	g	t	t	g	c	c	g	g	g	g	a	g	c	c	t	a	c	c	c	c	t	a	c	c	g	g	c	a	c	a	a	c	a	c	g	c	c	g	a
KP771915	3	g	c	a	g	a	t	a	g	t	a	c	c	a	t	g	t	c	g	a	c	a	a	a	g	g	t	t	t	t	t	g	t	g	c	t	g	t	t	g	c	c	g	g	g	g	a	g	c	c	t	a	c	c	c	c	t	a	c	c	g	g	c	a	c	a	a	c	a	c	g	c	c	g	a
KP771916	3	g	c	a	g	a	t	a	g	t	a	c	c	a	t	g	t	c	g	a	c	a	a	a	g	g	t	t	t	t	t	g	t	g	c	t	g	t	t	g	c	c	g	g	g	g	a	g	c	c	t	a	c	c	c	c	t	a	c	c	g	g	c	a	c	a	a	c	a	c	g	c	c	g	a
KP771921	3	g	c	a	g	a	t	a	g	t	a	c	c	a	t	g	t	c	g	a	c	a	a	a	g	g	t	t	t	t	t	g	t	g	c	t	g	t	t	g	c	c	g	g	g	g	a	g	c	c	t	a	c	c	c	c	t	a	c	c	g	g	c	a	c	a	a	c	a	c	g	c	c	g	a
KP771923	3	g	c	a	g	a	t	a	g	t	a	c	c	a	t	a	t	c	g	a	c	a	a	a	g	g	t	t	t	t	t	g	t	g	c	t	g	t	t	g	c	c	g	g	g	g	a	g	c	c	t	a	c	c	c	c	t	a	c	c	g	g	c	a	c	a	a	c	a	c	g	c	c	a	a
KP771889	3	g	c	a	g	a	t	a	g	t	a	c	t	a	t	g	t	c	g	a	c	a	a	a	g	g	t	t	t	t	t	g	t	g	c	t	g	t	t	g	c	c	g	g	g	g	a	g	c	c	t	a	c	c	c	c	t	a	c	c	g	g	c	a	c	a	a	c	a	c	g	c	c	g	a
KU926311	3	g	c	a	g	a	t	a	g	t	a	c	c	a	t	g	t	c	g	a	c	a	a	a	g	g	t	t	t	t	t	g	t	g	c	t	g	t	t	g	c	c	g	g	g	g	a	g	c	c	t	a	c	c	c	c	t	a	c	c	g	g	c	a	c	a	a	c	a	c	g	c	c	g	a
JQ911599	3	g	c	a	g	a	t	a	g	t	a	c	c	a	t	g	t	c	g	a	c	a	a	a	g	g	t	t	t	t	t	g	t	g	c	t	t	t	t	g	c	c	g	g	g	g	a	g	c	c	t	a	c	c	c	c	t	a	c	c	g	g	c	a	c	a	a	c	a	c	g	c	c	g	a
MH709317	3	g	c	a	g	a	t	a	g	t	a	c	c	a	t	g	t	c	g	a	c	a	a	a	g	g	t	t	t	t	t	g	t	g	c	t	g	t	t	g	c	c	g	g	g	g	a	g	c	c	t	a	c	c	c	c	t	a	c	c	g	g	c	a	c	g	c	c	a	c	g	c	c	g	a
MH709346	3	g	c	a	g	a	t	a	g	t	a	c	c	a	t	g	t	c	g	a	c	a	a	a	g	g	t	t	t	t	t	g	t	g	c	t	g	t	t	g	c	t	g	g	g	g	a	g	c	c	t	a	c	c	c	c	t	a	c	c	g	g	c	a	c	g	c	c	a	c	g	c	c	g	a
MH709375	3	g	c	a	a	a	t	a	g	t	a	c	c	a	t	g	t	c	g	a	c	a	a	a	g	g	t	t	t	t	t	g	t	g	c	t	g	t	t	g	c	c	g	g	g	g	a	g	c	c	t	a	c	c	c	c	t	a	c	c	g	g	c	a	c	g	c	c	a	c	g	c	c	g	a
DQ452050	4	g	c	a	g	a	t	g	g	t	a	c	c	c	t	g	t	c	g	a	c	a	a	a	g	g	t	c	t	t	t	g	t	g	c	t	g	t	t	g	c	c	g	g	g	g	a	g	c	c	t	a	c	t	c	c	t	a	c	c	g	g	c	a	c	a	a	c	a	c	g	c	c	a	a
DQ479960	4	g	c	a	g	a	t	g	g	t	a	c	c	c	t	g	t	c	g	a	c	a	a	a	g	g	t	c	t	t	t	g	t	g	c	t	g	t	t	g	c	c	g	g	g	g	a	g	c	c	t	a	c	t	c	c	t	a	c	c	g	g	c	a	c	a	a	c	a	c	g	c	c	a	a
KP771898	4	g	c	a	g	a	t	g	g	t	a	c	c	c	t	g	t	c	g	a	c	a	a	a	g	g	t	c	t	t	t	g	t	g	c	t	g	t	t	g	c	c	g	g	g	g	a	g	c	c	t	a	c	t	c	c	t	a	c	c	g	g	c	a	c	a	a	c	a	c	g	c	c	a	a
MH709316	4	g	c	a	g	a	t	g	g	t	a	c	c	c	t	g	t	c	g	a	c	a	a	a	a	g	t	c	t	t	t	g	t	g	c	t	g	t	t	g	c	c	g	g	g	g	a	g	c	c	t	a	t	t	c	c	t	a	c	c	g	g	c	a	c	a	a	c	a	c	g	c	c	a	a
MH709318	4	g	c	a	g	a	t	g	g	t	a	c	c	c	t	g	t	c	g	a	c	a	a	a	g	g	t	c	t	t	t	g	t	g	c	t	g	t	t	g	c	c	g	g	g	g	a	g	c	c	t	a	c	t	c	c	t	a	c	c	g	g	c	a	c	a	a	c	a	c	g	c	c	a	a
MH709322	4	g	c	a	g	a	t	g	g	t	a	c	c	c	t	g	t	c	t	a	c	a	a	a	g	g	t	c	t	t	t	g	t	g	c	t	g	t	t	g	c	c	g	g	g	g	a	g	c	c	t	a	c	t	c	c	t	a	c	c	g	g	c	a	c	a	a	c	a	c	g	c	c	a	a
MH709323	4	g	t	a	g	a	t	g	g	t	a	c	c	c	t	g	t	c	g	a	c	a	a	a	g	g	t	c	t	t	t	g	t	g	c	t	g	t	t	g	c	c	g	g	g	g	a	g	c	c	t	a	c	t	c	c	t	a	c	c	g	g	c	a	c	a	a	c	a	c	g	t	c	a	a
MH709326	4	g	c	a	g	a	t	g	g	t	a	c	c	c	t	g	t	c	g	a	c	a	a	a	g	g	t	c	t	t	t	g	t	g	c	t	g	t	t	g	c	c	g	g	g	g	a	g	c	c	t	a	c	t	c	c	t	a	c	c	g	g	c	a	c	a	a	c	a	c	g	c	c	a	a
MH709341	4	g	c	a	g	a	t	g	g	t	a	c	c	c	t	g	t	c	g	a	c	a	a	a	g	g	t	c	t	t	t	g	t	g	c	t	g	t	t	g	c	c	g	g	g	g	a	g	c	c	t	a	c	t	c	c	t	a	c	c	g	g	c	a	c	a	a	c	a	c	g	c	c	a	a
MH709357	4	g	c	a	g	a	t	g	g	t	a	c	c	c	t	g	t	c	g	a	c	a	a	a	g	g	t	c	t	t	t	g	t	g	c	t	g	t	t	g	c	c	g	g	g	g	a	g	c	c	t	a	c	t	c	c	t	a	c	c	g	g	c	a	c	a	a	c	a	c	g	c	c	a	a
MH709364	4	g	c	a	g	a	t	g	g	t	a	c	c	c	t	g	t	c	g	a	c	a	a	a	g	g	t	c	t	t	t	g	t	g	c	t	g	t	t	g	c	c	g	g	g	g	a	g	c	c	t	a	c	t	c	c	t	a	c	c	g	g	c	a	c	a	a	c	a	c	g	c	c	a	t
MH709370	4	g	c	a	g	a	t	g	g	t	a	c	c	c	t	g	t	c	g	a	c	a	a	a	g	g	t	c	t	t	t	g	t	g	c	t	g	t	t	g	c	c	g	g	g	g	a	g	c	c	t	a	c	t	c	c	t	a	c	c	g	g	c	a	c	a	a	c	a	c	g	c	c	a	a
MH709371	4	g	c	a	g	a	t	g	g	t	a	c	c	c	t	g	t	c	g	a	c	a	a	a	g	g	t	c	t	t	t	g	t	g	c	t	g	t	t	g	c	c	g	g	g	g	a	g	c	c	t	a	c	t	c	c	t	a	c	c	g	g	c	a	c	a	a	c	a	c	g	c	c	a	a
MH709372	4	g	c	a	g	a	t	g	g	t	a	c	c	c	t	g	t	c	g	a	c	a	a	a	g	g	t	c	t	t	t	g	t	g	c	t	g	t	t	g	c	c	g	g	g	g	a	g	c	c	t	a	c	t	c	c	t	a	c	c	g	g	c	a	c	a	a	c	a	c	g	c	c	a	a
DQ457052	5	g	c	a	g	a	t	g	g	t	a	c	c	c	t	g	t	c	g	a	c	a	a	a	g	g	t	c	t	t	t	g	t	g	c	t	g	t	t	g	c	c	g	g	g	g	a	g	c	c	t	c	c	t	c	c	t	a	c	c	g	g	c	a	c	a	a	c	a	c	g	c	c	a	a
JN704704	5	g	c	a	g	a	t	g	g	t	a	c	c	c	t	g	t	c	g	a	c	a	a	a	g	g	t	c	t	t	t	g	t	g	c	t	g	t	t	g	c	c	g	g	g	g	a	g	c	c	t	a	c	t	c	c	t	a	c	c	g	g	c	a	c	a	a	c	a	c	g	c	c	a	a
JN704705	5	g	c	a	g	a	t	g	g	t	a	c	c	c	t	g	t	c	g	a	c	a	a	a	g	g	t	c	t	t	t	g	t	g	c	t	g	t	t	g	c	c	g	g	g	g	a	g	c	c	t	a	c	t	c	c	t	a	c	c	g	g	c	a	c	a	a	c	a	c	g	c	c	a	a
JN704706	5	g	c	a	g	a	t	g	g	t	a	c	c	c	t	g	t	c	g	a	c	a	a	a	g	g	t	c	t	t	t	g	t	g	c	t	g	t	t	g	c	c	g	g	g	g	a	g	c	c	t	a	c	t	c	c	t	a	c	c	g	g	c	a	c	a	a	c	a	c	g	c	c	a	a
JN704707	5	g	c	a	g	a	t	g	g	t	a	c	c	c	t	g	t	c	g	a	c	a	a	a	g	g	t	c	t	t	t	g	t	g	c	t	g	t	t	g	c	c	g	g	g	g	a	g	c	c	t	a	c	t	c	c	t	a	c	c	g	g	c	a	t	a	a	c	a	c	g	c	c	a	a
JN704708	5	g	c	a	g	a	t	g	g	t	a	c	c	c	t	g	t	c	g	a	c	a	a	a	g	g	t	c	t	t	t	g	t	g	c	t	g	t	t	g	c	c	g	g	g	g	a	g	c	c	t	a	c	t	c	c	t	a	c	c	g	g	c	a	c	a	a	c	a	c	g	c	c	a	a
KP771904	5	g	c	a	g	a	t	g	g	t	g	c	c	c	t	g	t	c	g	a	c	a	a	a	g	g	t	c	t	t	t	g	t	g	c	t	g	t	t	g	c	c	g	g	g	g	a	g	c	c	t	a	c	t	c	c	t	c	c	c	g	g	c	a	c	a	a	c	a	c	g	c	c	a	a
KP771905	5	g	c	a	g	a	t	g	g	t	a	c	c	c	t	g	t	c	g	a	c	a	a	a	g	g	t	c	t	t	t	g	t	g	c	t	g	t	t	g	c	c	g	g	g	g	a	g	c	c	t	a	c	t	c	c	t	a	a	c	g	g	c	a	c	a	a	c	a	c	g	c	c	a	a
KP771906	5	g	c	a	g	a	t	g	g	t	a	c	c	c	t	g	t	c	g	a	c	a	a	a	g	g	t	c	t	t	t	g	t	g	c	t	g	t	t	g	c	c	g	g	g	g	a	g	c	c	t	a	c	t	c	c	t	a	a	c	g	g	c	a	c	a	a	c	a	c	g	c	c	a	a
KP771919	5	g	c	a	g	a	t	g	g	t	a	c	c	c	t	g	t	c	g	a	c	a	a	a	g	g	t	c	t	t	t	g	t	g	c	t	g	t	t	g	c	c	g	g	g	g	a	g	c	c	t	a	c	t	c	c	t	a	c	c	g	g	c	a	c	a	a	c	a	c	g	c	c	a	a
KP771922	5	g	c	a	g	a	t	g	g	t	a	c	c	c	t	g	t	c	g	a	c	a	a	a	g	g	t	c	t	t	t	g	t	g	c	t	g	t	t	g	c	c	g	g	g	g	a	g	c	c	t	a	c	t	c	c	t	a	c	c	g	g	c	a	c	a	a	c	a	c	g	c	c	a	a
KP771924	5	g	c	a	g	a	t	g	g	t	a	c	c	c	t	g	t	c	g	a	c	a	a	a	g	g	t	c	t	t	t	g	t	g	c	t	g	t	t	g	c	c	g	g	g	g	a	g	c	c	t	a	c	t	c	c	t	a	c	c	g	g	c	a	c	a	a	c	a	c	g	c	c	a	a
KM355696	5	g	c	a	g	a	t	g	g	t	a	c	c	c	t	g	t	c	g	a	c	a	a	a	g	g	t	c	t	t	t	g	t	g	c	t	g	t	t	g	c	c	g	g	g	g	a	g	c	c	c	a	c	t	c	c	t	a	c	c	g	g	c	a	c	a	a	c	a	c	g	c	c	a	a
KM355697	5	g	c	a	g	a	t	g	g	t	a	c	c	c	t	g	t	c	g	a	c	a	a	a	g	g	t	c	t	t	t	g	t	g	c	t	g	t	t	g	c	c	g	g	g	g	a	g	c	c	c	a	c	t	c	c	t	a	c	c	g	g	c	a	c	a	a	c	a	c	g	c	c	a	a
KM355698	5	g	c	a	g	a	t	g	g	t	a	c	c	c	t	g	t	c	g	a	c	a	a	a	g	g	t	c	t	t	t	g	t	g	c	t	g	t	t	g	c	c	g	g	g	g	a	g	c	c	t	a	c	t	c	c	t	a	c	c	g	g	c	a	c	a	a	c	a	c	g	c	c	a	a
KM355699	5	g	c	a	g	a	t	g	g	t	a	c	c	c	t	g	t	c	g	a	c	a	a	a	g	g	t	c	t	t	t	g	t	g	c	t	g	t	t	g	c	c	g	g	g	g	a	g	c	c	t	a	c	t	c	c	t	a	c	c	g	g	c	a	c	a	a	c	a	c	g	c	c	a	a
KM355701	5	g	c	a	g	a	t	g	g	t	a	c	c	c	t	g	t	c	g	a	c	a	a	a	g	g	t	c	t	t	t	g	t	g	c	t	g	t	t	g	c	c	g	g	g	g	a	g	c	c	c	a	c	t	c	c	t	a	c	c	g	g	c	a	c	a	a	c	a	c	g	c	c	a	a
KM355702	5	g	c	a	g	a	t	g	g	t	a	c	c	c	t	g	t	c	g	a	c	a	a	a	g	g	t	c	t	t	t	g	t	g	c	t	g	t	t	g	c	c	g	g	g	g	a	g	c	c	t	a	c	t	c	c	t	a	c	c	g	g	c	a	c	a	a	c	a	c	g	c	c	a	a
KM355703	5	g	c	a	g	a	t	g	g	t	a	c	c	c	t	g	t	c	g	a	c	a	a	a	g	g	t	c	t	t	t	g	t	g	c	t	g	t	t	g	c	c	g	g	g	g	a	g	c	c	c	a	c	t	c	c	t	a	c	c	g	g	c	a	c	a	a	c	a	c	g	c	c	a	a
KM355704	5	g	c	a	g	a	t	g	g	t	a	c	c	c	t	g	t	c	g	a	c	a	a	a	g	g	t	c	t	t	t	g	t	g	c	t	g	t	t	g	c	c	g	g	g	g	a	g	c	c	c	a	c	t	c	c	t	a	c	c	g	g	c	a	c	a	a	c	a	c	g	c	c	a	a
KM355705	5	g	c	a	g	a	t	g	g	t	a	c	c	c	t	g	t	c	g	a	c	a	a	a	g	g	t	c	t	t	t	g	t	g	c	t	g	t	t	g	c	c	g	g	g	g	a	g	c	c	t	a	c	t	c	c	t	a	c	c	g	g	c	a	c	a	a	c	a	c	g	c	c	a	a
KM355706	5	g	c	a	g	a	t	g	g	t	a	c	c	c	t	g	t	c	g	a	c	a	a	a	g	g	t	c	t	t	t	g	t	g	c	t	g	t	t	g	c	c	g	g	g	g	a	g	c	c	t	a	c	t	c	c	t	a	c	c	g	g	c	a	c	a	a	c	a	c	g	c	c	a	a
KM355707	5	g	c	a	g	a	t	g	g	t	a	c	c	c	t	g	t	c	g	a	c	a	a	a	g	g	t	c	t	t	t	g	t	g	c	t	g	t	t	g	c	c	g	g	g	g	a	g	c	c	c	a	c	t	c	c	t	a	c	c	g	g	c	a	c	a	a	c	a	c	g	c	c	a	a
KM355708	5	g	c	a	g	a	t	g	g	t	a	c	c	c	t	g	t	c	g	a	c	a	a	a	g	g	t	c	t	t	t	g	t	g	c	t	g	t	t	g	c	c	g	g	g	g	a	g	c	c	c	a	c	t	c	c	t	a	c	c	g	g	c	a	c	a	a	c	a	c	g	c	c	a	a
KM355709	5	g	c	a	g	a	t	g	g	t	a	c	c	c	t	g	t	c	g	a	c	a	a	a	g	g	t	c	t	t	t	g	t	g	c	t	g	t	t	g	c	c	g	g	g	g	a	g	c	c	t	a	c	t	c	c	t	a	c	c	g	g	c	a	c	a	a	c	a	c	g	c	c	a	a
KM355711	5	g	c	a	g	a	t	g	g	t	a	c	c	c	t	g	t	c	g	a	c	a	a	a	g	g	t	c	t	t	t	g	t	g	c	t	g	t	t	g	c	c	g	g	g	g	a	g	c	c	t	a	c	t	c	c	t	a	c	c	g	g	c	a	c	a	a	c	a	c	g	c	c	a	a
KM355712	5	g	c	a	g	a	t	g	g	t	a	c	c	c	t	g	t	c	g	a	c	a	a	a	g	g	t	c	t	t	t	g	t	g	c	t	g	t	t	g	c	c	g	g	g	g	a	g	c	c	t	a	c	t	c	c	t	a	c	c	g	g	c	a	c	a	a	c	a	c	g	c	c	a	a
KY037797	[illegible]	[illegible]	[illegible]	[illegible]	[illegible]	[illegible]	[illegible]	[illegible]	[illegible]	[illegible]	[illegible]	[illegible]	[illegible]	[illegible]	[illegible]	[illegible]	[illegible]	[illegible]	[illegible]	[illegible]	[illegible]	[illegible]	[illegible]	[illegible]	[illegible]	[illegible]	[illegible]	[illegible]	[illegible]	[illegible]	[illegible]	[illegible]	[illegible]	[illegible]	[illegible]	[illegible]	[illegible]	[illegible]	[illegible]	[illegible]	[illegible]	[illegible]	[illegible]	[illegible]	[illegible]	[illegible]	[illegible]	[illegible]	[illegible]	[illegible]	[illegible]	[illegible]	[illegible]	[illegible]	[illegible]	[illegible]	[illegible]	[illegible]	[illegible]	[illegible]	[illegible]	[illegible]	[illegible]	[illegible]	[illegible]	[illegible]	[illegible]	[illegible]	[illegible]	[illegible]	[illegible]	[illegible]	[illegible]	[illegible]	[illegible]	[illegible]

Supplementary Table 3: 2880 SN

GB-ID	Clade markers																																																																										
	Position in Dumas	69275	69277	69286	69328	69349	69405	69424	69457	69619	69623	69751	69756	70014	70057	70198	70344	70350	70479	70586	70651	70664	70694	70794	70817	70848	71167	71196	71252	71262	71304	71324	71575	71618	71626	71818	71981	72092	72127	72219	72277	72283	72288	72349	72395	72403	72418	72429	72469	72526	72605	72613	72970	72997	73060	73145	73170	73181	73225	73417	73432	73433	73533	73561	73645	73669	73846	73993	74042	74071	74123	74233	74251	74293	74360
MH499467	5	g	c	a	g	a	t	g	g	t	a	c	c	c	t	g	t	c	g	a	c	a	a	a	g	g	t	c	t	t	t	g	g	g	c	t	g	t	t	g	c	c	g	g	g	g	a	g	c	c	t	a	c	t	c	c	t	a	c	c	g	g	c	a	c	a	a	c	a	c	g	c	c	a	a
MH499469	5	g	c	a	g	a	t	g	g	t	a	c	c	c	t	g	t	c	g	a	c	a	a	a	g	g	t	c	t	t	t	g	t	g	c	t	g	t	t	g	c	c	g	g	g	g	a	g	c	c	t	a	c	t	c	c	t	a	c	c	g	g	c	a	c	a	a	c	a	c	g	c	c	a	a
MH709321	5	g	c	a	g	a	t	g	g	t	a	c	c	c	t	g	t	c	g	a	c	a	a	a	g	g	t	c	t	t	t	g	t	g	c	t	g	t	t	g	c	c	g	g	g	g	a	g	c	c	t	a	c	t	c	c	t	a	c	c	a	a	c	a	c	a	a	c	a	c	g	c	c	a	a
MH709324	5	g	c	a	g	a	t	g	g	t	a	c	c	c	t	g	t	c	g	a	c	a	a	a	g	g	t	c	t	t	t	g	t	t	c	c	g	t	t	g	c	c	g	g	g	g	a	g	c	c	t	a	c	t	c	c	t	a	c	c	g	g	c	a	c	a	a	c	a	c	g	c	c	a	a
MH709325	5	g	c	a	g	a	t	g	g	t	a	c	c	c	t	g	t	c	g	a	c	a	a	a	g	g	t	c	t	t	t	g	t	g	c	t	g	t	t	g	c	c	g	g	g	g	a	g	c	c	t	a	c	t	c	c	t	a	c	c	g	g	c	a	c	a	a	c	a	c	g	c	c	a	a
MH709327	5	g	c	a	g	a	t	g	g	t	a	c	c	c	t	g	t	c	g	a	c	a	a	a	g	g	t	c	t	t	t	g	t	g	c	t	g	t	t	g	c	c	g	g	g	g	a	g	c	c	t	c	c	t	c	c	t	a	c	c	g	g	c	a	c	a	a	c	a	c	g	c	c	a	a
MH709328	5	g	c	a	g	a	t	g	g	t	a	c	c	c	t	g	t	c	g	a	c	a	a	a	g	g	t	c	t	t	t	g	t	g	c	t	g	t	t	g	c	c	g	g	g	g	a	g	c	c	t	a	c	t	c	c	t	a	c	c	g	g	c	a	c	a	a	c	a	c	g	c	c	a	a
MH709330	5	g	c	a	g	a	t	g	g	t	a	c	c	c	t	g	t	c	g	a	c	a	a	a	g	g	t	c	t	t	t	g	t	g	c	t	g	t	t	g	c	c	g	g	g	g	a	g	c	c	t	a	c	t	c	c	t	a	c	c	g	g	c	a	c	a	a	c	a	c	g	c	c	a	a
MH709332	5	g	c	a	g	a	t	g	g	t	a	c	c	c	t	g	t	c	g	a	c	a	a	a	g	g	t	c	t	t	t	g	t	g	c	t	g	t	t	g	c	c	g	g	g	g	a	g	c	c	t	a	c	t	c	c	t	a	c	c	g	g	c	a	c	a	a	c	a	c	g	c	c	a	a
MH709334	5	g	c	a	g	a	t	g	g	t	a	c	c	c	t	g	t	c	g	a	c	a	a	a	g	g	t	c	t	t	t	g	t	g	c	t	g	t	t	g	c	c	g	g	g	g	a	g	c	c	t	c	c	t	c	c	t	a	c	c	g	g	c	a	c	a	a	c	a	c	g	c	c	a	a
MH709335	5	g	c	a	g	a	t	g	g	t	a	c	c	c	t	g	t	c	g	a	c	a	a	a	g	g	t	c	t	t	t	g	t	g	c	t	g	t	t	g	c	c	g	g	g	g	a	g	c	c	t	c	c	t	c	c	t	a	c	c	g	g	c	a	c	a	a	c	a	c	g	c	c	a	a
MH709336	5	g	c	a	g	a	t	g	g	t	a	c	c	c	t	g	t	c	g	a	c	a	a	a	g	g	t	c	t	t	t	g	t	g	c	t	g	t	t	g	c	c	g	g	g	g	a	g	c	c	t	c	c	t	c	c	t	a	c	c	g	g	c	a	c	a	a	c	a	c	g	c	c	a	a
MH709338	5	g	c	a	g	a	t	g	g	t	a	c	c	c	t	g	t	c	g	a	c	a	a	a	g	g	t	c	t	t	t	g	t	g	c	t	g	t	t	g	c	c	g	g	g	g	a	g	c	c	t	c	c	t	c	c	t	a	c	c	g	g	c	a	c	a	a	c	a	c	g	c	c	a	a
MH709340	5	g	c	a	g	a	c	g	g	t	a	c	c	c	g	g	t	c	g	a	c	a	a	a	g	g	t	c	t	t	t	g	t	g	c	t	g	t	t	g	c	c	g	g	g	g	a	g	c	c	t	a	c	t	c	c	t	a	c	c	g	g	c	a	c	a	a	c	a	c	g	c	c	a	a
MH709342	5	g	c	a	g	a	t	g	g	t	a	c	c	c	t	g	t	c	g	a	c	a	a	a	g	g	t	c	t	t	t	g	t	g	c	t	g	t	t	g	c	c	g	g	g	g	a	g	c	c	t	a	c	t	c	c	t	a	c	c	g	g	c	a	c	a	a	c	a	c	g	c	c	a	a
MH709343	5	g	c	a	g	a	t	g	g	t	a	c	c	c	t	g	t	c	g	a	c	a	a	a	g	g	t	c	t	t	t	g	t	g	c	t	g	t	t	g	c	c	g	g	g	g	a	g	c	c	t	a	c	t	c	c	t	a	c	c	g	g	c	a	c	a	a	c	a	c	g	c	c	a	a
MH709345	5	g	c	a	g	a	t	g	g	t	a	c	c	c	t	g	t	c	g	a	c	a	a	a	g	g	t	c	t	t	t	g	t	g	c	t	g	t	t	g	c	c	g	g	g	g	a	g	c	c	t	c	c	t	c	c	t	a	c	c	g	g	c	a	c	a	a	c	a	c	g	c	c	a	a
MH709347	5	g	c	a	g	a	t	g	g	t	a	c	c	c	t	g	t	c	g	a	c	a	a	a	g	g	t	c	t	t	t	g	t	g	c	t	g	t	t	g	c	c	g	g	g	g	a	g	c	c	t	c	c	t	c	c	t	a	c	c	g	g	c	a	c	a	a	c	a	c	g	c	c	a	a
MH709349	5	g	c	a	g	a	t	g	g	t	a	c	c	c	t	g	t	c	g	a	c	a	a	a	g	g	t	c	t	t	t	g	t	g	c	t	g	t	t	g	c	c	g	g	g	g	a	g	c	c	t	a	c	t	c	c	t	a	c	c	g	g	c	a	c	a	a	c	a	c	g	c	c	a	a
MH709350	5	g	c	a	g	a	t	g	g	t	a	c	c	c	t	g	t	c	g	a	c	a	a	a	g	g	t	c	t	t	t	g	t	g	c	t	g	t	t	g	c	c	g	g	g	g	a	g	c	c	t	c	c	t	c	c	t	a	c	c	g	g	c	a	c	a	a	c	a	c	g	c	c	a	a
MH709351	5	g	c	a	g	a	t	g	g	t	a	c	c	c	t	g	t	c	g	a	c	a	a	a	g	g	t	c	t	t	t	g	t	g	c	t	g	t	t	g	c	c	g	g	g	g	a	g	c	c	t	a	c	t	c	c	t	a	c	c	g	g	c	a	c	a	a	c	a	c	g	c	c	a	a
MH709353	5	g	c	a	g	a	t	g	g	t	a	c	c	c	t	g	t	c	g	a	c	a	a	a	g	g	t	c	t	t	t	g	t	g	c	t	g	t	t	g	c	c	g	g	g	g	a	g	c	c	t	c	c	t	c	c	t	a	c	c	g	g	c	a	c	a	a	c	a	c	g	c	c	a	a
MH709358	5	g	c	a	g	a	t	g	g	t	a	c	c	c	t	g	t	c	g	a	c	a	a	a	g	g	t	c	t	t	t	g	t	g	c	t	g	t	t	g	c	c	g	g	g	g	a	g	c	c	t	a	c	t	c	c	t	a	c	c	g	g	c	a	c	a	a	c	a	c	a	c	c	a	a
MH709360	5	g	c	a	g	a	t	g	g	t	a	c	c	c	t	g	t	c	g	a	c	a	a	a	g	g	t	c	t	t	t	g	t	g	c	t	g	t	t	g	c	c	g	g	g	g	a	g	c	c	t	a	c	t	c	c	t	a	c	c	g	g	c	a	c	a	a	c	a	c	g	c	c	a	a
MH709362	5	g	c	a	g	a	t	g	g	t	a	c	c	c	t	g	t	c	g	a	c	a	a	a	g	g	t	c	t	t	t	g	t	g	c	t	g	t	t	g	c	c	g	g	g	g	a	g	c	c	t	c	c	t	c	c	t	a	c	c	g	g	c	a	c	a	a	c	a	c	g	c	c	a	a
MH709365	5	g	c	a	g	a	t	g	g	t	a	c	c	c	t	g	t	c	g	a	c	a	a	a	g	g	t	c	t	t	t	g	t	g	c	t	g	t	t	g	c	c	g	g	g	g	a	g	c	c	t	c	c	t	c	c	t	a	c	c	g	g	c	a	c	a	a	c	a	c	g	c	c	a	a
MH709373	5	g	c	a	g	a	t	g	g	t	a	c	c	c	t	g	t	c	g	a	c	a	a	a	g	g	t	c	t	t	t	g	t	g	c	t	g	t	t	g	c	c	g	g	g	g	a	g	c	c	t	a	c	t	c	c	t	a	c	c	g	g	c	a	c	a	a	c	a	c	g	c	c	a	a
MH709374	5	g	c	a	g	a	t	g	g	t	a	c	c	c	t	g	t	c	g	a	c	a	a	c	g	g	t	c	t	t	t	g	t	g	c	t	g	t	t	g	c	c	g	g	g	g	a	g	c	c	t	c	c	t	c	c	t	a	c	c	g	g	c	a	c	a	a	c	a	c	g	c	c	a	a
KC112914	6	g	c	a	g	a	c	a	g	t	a	c	c	a	t	g	t	c	g	a	c	a	a	a	g	g	t	t	t	t	t	g	t	t	c	t	g	t	t	g	c	c	g	g	g	g	a	g	c	a	t	a	c	c	c	c	t	a	c	c	g	g	c	a	c	a	a	c	a	c	g	c	c	a	a
KP771914	6	g	c	a	g	a	c	a	g	t	a	c	c	a	t	g	t	c	g	c	c	a	a	a	g	g	t	t	t	t	t	g	t	t	c	t	g	t	t	g	c	c	g	g	g	g	a	g	c	a	t	a	c	c	c	c	t	a	c	c	g	g	c	a	c	a	a	c	a	c	g	c	c	a	a
KP702725	6	g	c	a	g	a	c	a	g	t	a	t	c	a	t	g	t	c	g	a	c	a	a	a	g	g	t	t	t	t	t	g	t	t	c	t	g	t	t	g	c	c	g	g	g	g	a	g	c	a	t	a	c	c	c	c	t	a	c	a	g	g	c	a	c	a	a	c	a	c	g	c	c	a	a
KP771890	9	g	c	a	g	a	t	a	g	t	a	c	c	a	t	g	t	c	g	a	c	a	a	a	g	g	t	c	t	t	t	g	t	g	c	t	g	t	t	g	c	c	g	g	g	g	a	g	c	c	t	a	c	c	c	c	t	a	c	c	g	g	c	a	c	a	a	c	a	c	g	c	c	a	a
JN704710	9	g	c	a	g	a	t	a	g	t	a	c	t	a	t	g	t	c	g	a	c	a	a	a	g	g	t	c	t	t	t	g	t	g	c	t	g	t	t	g	c	c	g	g	g	g	a	g	c	c	t	a	c	c	c	c	t	a	c	c	g	g	c	a	c	a	a	t	a	c	g	c	c	a	a
KY037798	9	g	c	a	g	a	t	a	g	t	a	c	c	a	t	g	t	c	g	a	c	a	a	a	g	g	t	c	t	t	t	g	t	g	c	t	g	t	t	g	c	c	g	g	g	g	a	g	c	c	t	a	c	c	c	c	t	a	c	c	g	g	c	a	c	a	a	c	a	c	g	c	c	a	a
MH709312	9	g	c	a	g	a	t	a	g	t	a	c	c	a	t	g	t	c	g	a	c	a	a	a	g	g	t	c	t	t	t	g	t	g	c	t	g	t	t	g	c	c	g	g	g	g	a	g	c	c	t	a	c	c	c	c	t	a	c	c	g	g	c	a	c	a	a	c	a	c	g	c	c	a	a
MH709352	9	g	c	a	g	a	t	a	g	t	a	c	c	a	t	g	t	c	g	a	c	a	a	a	g	g	t	c	t	t	t	g	t	g	c	t	g	t	t	g	c	c	g	g	g	g	a	g	c	c	t	a	c	c	c	c	t	a	c	c	g	g	t	a	c	a	a	c	a	c	g	c	c	a	a
AB097933	Vac	g	c	a	g	g	t	g	g	t	a	c	c	c	t	g	t	c	g	a	c	a	a	a	g	g	t	c	t	t	t	g	t	g	c	t	g	t	t	g	c	c	g	g	g	g	a	g	c	c	t	a	c	t	c	c	t	a	c	c	g	g	c	a	c	a	a	c	a	c	g	c	c	a	a
JN704698	Vac	g	c	a	g	g	t	g	g	t	a	c	c	c	t	g	t	c	g	a	c	a	a	a	g	g	t	c	t	t	t	g	t	g	c	t	g	t	t	g	c	c	g	g	g	g	a	g	c	c	t	a	c	t	c	c	t	a	c	c	g	g	c	a	c	a	a	c	a	c	g	c	c	a	a
JN704697	Vac	g	c	a	g	g	t	g	g	t	a	c	c	c	t	g	t	c	g	a	c	a	a	a	g	g	t	c	c	t	t	g	t	g	c	t	g	t	t	g	c	c	g	g	g	g	a	g	c	c	t	a	c	t	c	c	t	a	c	c	g	g	c	a	c	a	a	c	a	c	g	c	c	a	a
DQ008355	Vac	g	c	a	g	g	t	g	g	t	a	c	c	c	t	g	t	c	g	a	c	a	a	a	g	g	t	c	y	t	t	g	t	g	c	t	g	t	t	g	c	c	g	g	g	g	a	g	c	c	t	a	c	t	c	c	t	a	c	c	g	g	c	a	c	a	a	c	a	c	g	c	c	a	a
JF306641	Vac	g	c	a	g	g	t	g	g	t	a	c	c	c	t	g	t	c	g	a	c	a	a	a	g	g	t	c	c	t	t	g	t	g	c	t	g	t	t	g	c	c	g	g	g	g	a	g	c	c	t	a	c	t	c	c	t	a	c	c	g	g	c	a	c	a	a	c	a	c	g	c	c	a	a
AB097932	Vac	g	c	a	g	g	t	g	g	t	a	c	c	c	t	g	t	c	g	a	c	a	a	a	g	g	t	c	c	t	t	g	t	g	c	t	g	t	t	g	c	c	g	g	g	g	a	g	c	c	t	a	c	t	c	c	t	a	c	c	g	g	c	a	c	a	a	c	a	c	g	c	c	a	a
DQ008354	Vac	g	c	a	g	g	t	g	g	t	a	c	c	c	t	g	t	c	g	a	c	a	a	a	g	g	t	c	c	t	t	g	t	g	c	t	g	t	t	g	c	c	g	g	g	g	a	g	c	c	t	a	c	t	c	c	t	a	c	c	g	g	c	a	c	a	a	c	a	c	g	c	c	a	a
KU926312	Vac	g	c	a	g	g	t	g	g	t	a	c	c	c	t	g	t	c	g	a	c	a	a	a	g	g	t	c	c	t	t	g	t	g	c	t	g	t	t	g	c	c	g	g	g	g	a	g	c	c	t	a	c	t	c	c	t	a	c	c	g	g	c	a	c	a	a	c	a	c	g	c	c	a	a
KU926313	Vac	g	c	a	g	g	t	g	g	t	a	c	c	c	t	g	t	c	g	a	c	a	a	a	g	g	t	c	c	t	t	g	t	g	c	t	g	t	t	g	c	c	g	g	g	g	a	g	c	c	t	a	c	t	c	c	t	a	c	c	g	g	c	a	c	a	a	c	a	c	g	c	c	a	a
KU926314	Vac	g	c	a	g	g	t	g	g	t	a	c	c	c	t	g	t	c	g	a	c	a	a	a	g	g	t	c	c	t	t	g	t	g	c	t	g	t	t	g	c	c	g	g	g	g	a	g	c	c	t	a	c	t	c	c	t	a	c	c	g	g	c	a	c	a	a	c	a	c	g	c	c	a	a
KF558371	Vac	g	c	a	g	g	t	g	g	t	a	c	c	c	t	g	t	c	g	a	c	a	a	a	g	g	t	c	c	t	t	g	t	g	c	t	g	t	t	g	c	c	g	g	g	g	a	g	c	c	t	a	c	t	c	c	t	a	c	c	g	g	c	a	c	a	a	c	a	c	g	c	c	a	a
KF558372	Vac	g	c	a	g	g	t	g	g	t	a	c	c	c	t	g	t	c	g	a	c	a	a	a	g	g	t	c	t	t	t	g	t	g	c	t	g	t	t	g	c	c	g	g	g	g	a	g	c	c	t	a	c	t	c	c	t	a	c	c	g	g	c	a	c	a	a	c	a	c	g	c	c	a	a
KF558373	Vac	g	c	a	g	g	t	g	g	t	a	c	c	c	t	g	t	c	g	a	c	a	a	a	g	g	t	c	t	t	t	g	t	g	c	t	g	t	t	g	c	c	g	g	g	g	a	g	c	c	t	a	c	t	c	c	t	a	c	c	g	g	c	a	c	a	a	c	a	c	g	c	c	a	a
KF558374	Vac	g	c	a	g	g	t	g	g	t	a	c	c	c	t	g	t	c	g	a	c	a	a	a	g	g	t	c	t	t	t	g	t	g	c	t	g	t	t	g	c	c	g	g	g	g	a	g	c	c	t	a	c	t	c	c	t	a	c	c	g	g	c	a	c	a	a	c	a	c	g	c	c	a	a
KF558375	Vac	g	c	a	g	g	t	g	g	t	a	c	c	c	t	g	t	c	g	a	c	a	a	a	g	g	t	c	c	t	t	g	t	g	c	t	g	t	t	g	c	c	g	g	g	g	a	g	c	c	t	a	c	t	c	c	t	a	c	c	g	g	c	a	c	a	a	c	a	c	g	c	c	a	a
KF558376	Vac	g	c	a	g	g	t	g	g	t	a	c	c	c	t	g	t	c	g	a	c	a	a	a	g	g	t	c	c	t	t	g	t	g	c	t	g	t	t	g	c	c	g	g	g	g	a	g	c	c	t	a	c	t	c	c	t	a	c	c	g	g	c	a	c	a	a	c	a	c	g	c	c	a	a
KF558377	Vac	g	c	a	g	g	t	g	g	t	a	c	c	c	t	g	t	c	g	a	c	a	a	a	g	g	t	c	t	t	t	g	t	g	c	t	g	t	t	g	c	c	g	g	a	g	a	g	c	c	t	a	c	t	c	c	t	a	c	c	g	g	c	a	c	a	a	c	a	c	g	c	c	a	a
KF558378	Vac	g	c	a	g	g	t	g	g	t	a	c	c	c	t	g	t	c	g	a	c	a	a	a	g	g	t	c	t	t	t	g	t	g	c	t	g	t	t	g	c	c	g	g	g	g	a	g	c	c	t	a	c	t	c	c	t	a	c	c	g	g	c	a	c	a	a	c	a	c	g	c	c	a	a
KF558379	Vac	g	c	a	g	g	t	g	g	t	a	c	c	c	t	g	t	c	g	a	c	a	a	a	g	g	t	c	t	t	t	g	t	g	c	t	g	t	t	g	c	c	g	g	g	g	a	g	c	c	t	a	c	t	c	c	t	a	c	c	g	g	c	a	c	a	a	c	a	c	g	c	c	a	a
KF558380	Vac	g	c	a	g	g	t	g	g	t	a	c	c	c	t	g	t	c	g	a	c	a	a	a	g	g	t	c	c	t	t	g	t	g	c	t	g	t	t	g	c	c	g	g	g	g	a	g	c	c	t	a	c	t	c	c	t	a	c	c	g	g	c	a	c	a	a	c	a	c	g	c	c	a	a
KF558381	Vac	g	c	a	g	g	t	g	g	t	a	c	c	c	t	g	t	c	g	a	c	a	a	a	g	g	t	c	c	t	t	g	t	g	c	t	g	t	t	g	c	c	g	g	g	g	a	g	c	c	t	a	c	t	c	c	t	a	c	c	g	g	c	a	c	a	a	c	a	c	g	c	c	a	a
KF558382	Vac	g	c	a	g	g	t	g	g	t	a	c	c	c	t	g	t	c	g	a	c	a	a	a	g	g	t	c	c	t	t	g	t	g	c	t	g	t	t	g	c	c	g	g	g	g	a	g	c	c	t	a	c	t	c	c	t	a	c	c	g	g	c	a	c	a	a	c	a	c	g	c	c	a	a
KF558383	Vac	g	c	a	g	g	t	g	g	t	a	c	c	c	t	g	t	c	g	a	c	a	a	a	g	g	t	c	t	t	t	g	t	g	c	t	g	t	t	g	c	c	g	g	g	g	a	g	c	c	t	a	c	t	c	c	t	a	c	c	g	g	c	a	c	a	a	c	a	c	g	c	c	a	a
KF558384	Vac	g	c	a	g	g	t	g	g	t	a	c	c	c	t	g	t	c	g	a	c	a	a	a	g	g	t	c	t	t	t	g	t	g	c	t	g	t	t	g	c	c	g	g	g	g	a	g	c	c	t	a	c	t	c	c	t	a	c	c	g	g	c	a	c	a	a	c	a	c	g	c	c	a	a
KF558385	Vac	g	c	a	g	g	t	g	g	t	a	c	c	c	t	g	t	c	g	a	c	a	a	a	g	g	t	c	t	t	t	g	t	g	c	t	g	t	t	g	c	c	g	g	g	g	a	g	c	c	t	a	c	t	c	c	t	a	c	c	g	g	c	a	c	a	a	c	a	c	g	c	c	a	a
KF558386	Vac	g	c	a	g	g	t	g	g	t	a	c	c	c	t	g	t	c	g	a	c	a	a	a	g	g	t	c	c	t	t	g	t	g	c	t	g	t	t	g	c	c	g	g	g	g	a	g	c	c	t	a	c	t	c	c	t	a	c	c	g	g	c	a	c	a	a	c	a	c	g	c	c	a	a
KF558387	Vac	g	c	a	g	g	t	g	g	t	a	c	c	c	t	g	t	c	g	a	c	a	a	a	g	g	t	c	t	t	t	g	t	g	c	t	g	t	c	g	c	c	g	g	g	g	a	g	c	c	t	a	c	t	c	c	t	a	c	c	g	g	c	a	c	a	a	c	a	c	g	c	c	a	a
KF558388	Vac	g	c	a	g	g	t	g	g	t	a	c	c	c	t	g	t	c	g	a	c	a	a	a	g	g	t	c	c	t	t	g	t	g	c	t	g	t	t	g	c	c	g	g	g	g	a	g	c	c	t	a	c	t	c	c	t	a	c	c	g	g	c	a	c	a	a	c	a	c	g	c	c	a	a
KF558389	Vac	g	c	a	g	g	t	g	g	t	a	c	c	c	t	g	t	c	g	a	c	a	a	a	g	g	t	c	t	t	t	g	t	g	c	t	g	t	t	g	c	c	g	g	g	g	a	g	c	c	t	a	c	t	c	c	t	a	c	c	g	g	c	a	c	a	a	c	a	c	g	c	c	a	a
KF558390	Vac	g	c	a	g	g	t	g	g	t	a	c	c	c	t	g	t	c	g	a	c	a	a	a	g	g	t	c	c	t	t	g	t	g	c	t	g	t	t	g	c	c	g	g	g	g	a	g	c	c	t	a	c	t	c	c	t	a	c	c	g	g	c	a	c	a	a	c	a	c	g	c	c	a	a
KF558391	Vac	g	c	a	g	g	t	g	g	t	a	c	c	c	t	g	t	c	g	a	c	a	a	a	g	g	t	c	c	t	t	g	t	g	c	t	g	t	t	g	c	c	g	g	g	g	a	g	c	c	t	a	c	t	c	c	t	a	c	c	g	g	c	a	c	a	a	c	a	c	g	c	c	a	a
KF811485	Vac	g	c	a	g	g	t	g	g	t	a	c	c	c	t	g	t	c	g	a	c	a	a	a	g	g	t	c	c	t	t	g	t	g	c	t	g	t	t	g	c	c	g	g	g	g	a	g	c	c	t	a	c	t	c	c	t	a	c	c	g	g	c	a	c	a	a	c	a	c	g	c	c	a	a
KF853225	Vac	g	c	a	g	g	t	g	g	t	a	c	c	c	t	g	t	c	g	a	c	a	a	a	g	g	t	c	t	t	t	g	t	g	c	t	g	t	t	g	c	c	g	g	g	g	a	g	c	c	t	a	c	t	c	c	t	a	c	c	g	g	c	a	c	a	a	c	a	c	g	c	c	a	a
KF853226	Vac	g	c	a	g	g	t	g	g	t	a	c	c	c	t	g	t	c	g	a	c	a	a	a	g	g	t	c	c	t	t	g	t	g	c	t	g	t	t	g	c	c	g	g	g	g	a	g	c	c	t	a	c	t	c	c	t	a	c	c	g	g	c	a	c	a	a	c	a	c	g	c	c	a	a
KF853227	Vac	g	c	a	g	g	t	g	g	t	a	c	c	c	t	g	t	c	g	a	c	a	a	a	g	g	t	c	c	t	t	g	t	g	c	t	g	t	t	g	c	c	g	g	g	g	a	g	c	c	t	a	c	t	c	c	t	a	c	c	g	g	c	a	c	a	a	c	a	c	g	c	c	a	a
KF853228	Vac	g	c	a	g	g	t	g	g	t	a	c	c	c	t	g	t	c	g	a	c	a	a	a	g	g	t	c	c	t	t	g	t	g	c	t	g	t	t	g	c	c	g	g	g	g	a	g	c	c	t	a	c	t	c	c	t	a	c	c	g	g	c	a	c	a	a	c	a	c	g	c	c	a	a
KF853229	Vac	g	c	a	g	g	t	g	g	t	a	c	c	c	t	g	t	c	g	a	c	a	a	a	g	g	t	c	t	t	t	g	t	g	c	t	g	t	t	g	c	c	g	g	g	g	a	g	c	c	t	a	c	t	c	c	t	a	c	c	g	g	c	a	c	a	a	c	a	c	g	c	c	a	a
KF853230	Vac	g	c	a	g	g	t	g	g	t	a	c	c	c	t	g	t	c	g	a	c	a	a	a	g	g	t	c	t	t	t	g	t	g	c	t	g	t	t	g	c	c	g	g	g	g	a	g	c	c	t	a	c	t	c	c	t	a	c	c	g	g	c	a	c	a	a	c	a	c	g	c	c	a	a
KF853231	Vac	g	c	a	g	g	t	g	g	t	a	c	c	c	t	g	t	c	g	a	c	a	a	a	g	g	t	c	c	t	t	g	t	g	c	t	g	t	t	g	c	c	g	g	g	g	a	g	c	c	t	a	c	t	c	c	t	a	c	c	g	g	c	a	c	a	a	c	a	c	g	c	c	a	a
KF853232	Vac	g	c	a	g	g	t	g	g	t	a	c	c	c	t	g	t	c	g	a	c	a	a	a	g	g	t	c	c	t	t	g	t	g	c	t	g	t	t	g	c	c	g	g	g	g	a	g	c	c	t	a	c	t	c	c	t	a	c	c	g	g	c	a	c	a	a	c	a	c	g	c	c	a	a
KF853233	Vac	g	c	a	g	g	t	g	g	t	a	c	c	c	t	g	t	c	g	a	c	a	a	a	g	g	t	c	t	t	t	g	t	g	c	t	g	t	t	g	c	c	g	g	g	g	a	g	c	c	t	a	c	t	c	c	t	a	c	c	g	g	c	a	c	a	a	c	a	c	g	c	c	a	a
KF853234	Vac	g	c	a	g	g	t	g	g	t	a	c	c	c	t	g	t	c	g	a	c	a	a	a	g	g	t	c	t	t	t	g	t	g	c	t	g	t	t	g	c	c	g	g	g	g	a	g	c	c	t	a	c	t	c	c	t	a	c	c	g	g	c	a	c	a	a	c	a	c	g	c	c	a	a
KF853235	Vac	g	c	a	g	g	t	g	g	t	a	c	c	c	t	g	t	c	g	a	c	a	a	a	g	g	t	c	t	t	t	g	t	g	c	t	g	t	t	g	c	c	g	g	g	g	a	g	c	c	t	a	c	t	c	c	t	a	c	c	g	g	c	a	c	a	a	c	a	c	g	c	c	a	a
MF898328	Vac	g	c	a	g	g	t	g	g	t	a	c	c	c	t	g	t	c	g	a	c	a	a	a	g	g	t	c	c	t	t	g	t	g	c	t	g	t	t	g	c	c	g	g	g	g	a	g	c	c	t	a	c	t	c	c	t	a	c	c	g	g	c	a	c	a	a	c	a	c	g	c	c	a	a
JN704709	VIII	g	c	a	g	a	c	g	g	c	a	c	c	a	t	g	t	c	g	a	c	a	a	a	g	g	t	t	t	t	t	g	t	t	c	t	g	t	t	g	c	c	g	g	g	g	a	g	a	a	t	a	c	c	c	c	t	a	c	c	g	g	c	a	c	a	a	c	a	c	g	c	c	a	a

Supplementary Table 3: 2880 SN

GB-ID	Clade markers						5																																														SD14																							
	Position in Dumas	74452	74560	74563	74620	74638	74699	74743	74782	74861	74932	74941	74943	74972	75042	75054	75073	75103	75116	75173	75177	75251	75255	75256	75259	75568	75637	75746	75751	75755	75836	75888	75897	76131	76212	76218	76236	76290	76293	76329	76377	76389	76509	76510	76522	76530	76575	76779	76846	76907	76939	76953	76985	76992	77102	77154	77169	77220	77286	77393	77424	77580	77592	77616	77619	77691	77729	77817	77898	77913	77951	77979	77985	78090	78105
AY548170	1	t	a	a	c	g	t	c	a	g	a	g	g	g	g	c	c	a	t	t	c	g	c	c	c	g	c	a	c	c	c	a	g	g	a	a	a	c	c	c	c	t	g	t	t	c	c	g	t	a	a	a	t	a	t	t	a	t	g	g	a	g	a	a	t	g	t	g	c	t	a	a	a	t	g
AY548171	1	t	a	a	c	g	t	c	a	g	a	g	g	g	g	c	c	a	t	t	c	g	c	c	c	g	c	a	c	c	c	a	g	g	a	a	a	c	c	c	c	t	g	c	t	c	c	g	t	a	a	a	t	a	t	t	a	t	g	g	a	g	a	a	t	g	t	g	t	t	a	a	a	t	g
DQ479953	1	t	a	a	c	g	t	c	a	g	a	g	g	g	g	c	c	a	t	t	c	g	c	c	c	g	c	a	c	c	c	a	g	g	a	a	a	c	c	c	c	t	g	c	t	c	c	g	t	a	a	a	t	a	t	t	a	t	g	g	a	g	a	a	t	g	t	g	t	t	a	a	a	t	g
DQ479954	1	t	a	a	c	g	t	c	a	g	a	g	g	g	g	c	c	a	t	t	c	g	c	c	c	g	c	a	c	c	c	a	g	g	a	a	a	c	c	c	t	t	g	c	t	c	c	g	t	a	a	a	t	a	t	t	a	t	g	g	a	g	a	a	c	g	t	g	t	t	a	a	a	t	g
DQ479958	1	t	a	a	c	g	t	c	a	g	a	g	g	g	g	c	c	a	t	t	c	g	c	c	c	g	c	a	c	c	c	a	g	g	a	a	a	c	c	c	c	t	g	c	t	c	c	g	t	a	a	a	t	a	t	t	a	t	g	g	a	g	a	a	t	g	t	g	t	t	a	a	a	t	g
DQ479959	1	t	a	a	c	g	t	c	a	g	a	g	g	g	g	c	c	a	t	t	c	g	c	c	c	g	c	a	c	c	c	a	g	g	a	a	a	c	c	c	c	t	g	c	t	c	c	g	t	a	a	a	t	a	t	t	a	t	g	g	a	g	a	a	t	g	t	g	t	t	a	a	a	t	g
DQ479961	1	t	a	a	c	g	t	c	a	g	a	g	g	g	g	c	c	a	t	t	c	g	c	c	c	g	c	a	c	c	c	a	g	g	a	a	a	c	c	c	t	t	g	c	t	c	c	g	t	a	a	a	t	a	t	t	a	t	g	g	a	g	a	a	t	g	t	g	t	t	a	a	a	t	g
DQ479962	1	t	a	a	c	g	t	c	a	g	a	g	g	g	g	c	c	a	t	t	c	g	c	c	c	g	c	a	c	c	c	a	g	g	a	a	a	c	c	c	t	t	g	c	t	c	c	g	t	a	a	a	t	a	t	t	a	t	g	g	a	g	a	a	t	g	t	g	t	t	a	a	a	t	g
DQ479963	1	t	a	a	c	g	t	c	a	g	a	g	g	g	g	c	c	a	t	t	c	g	c	c	c	g	c	a	c	c	c	a	g	g	a	a	a	c	c	c	t	t	g	c	t	c	c	g	t	a	a	a	t	a	t	t	a	t	g	g	a	g	a	a	t	g	t	g	t	t	a	a	a	t	g
DQ674250	1	t	a	a	c	g	t	c	g	g	a	g	g	g	g	c	c	a	t	t	c	g	c	c	c	g	c	a	c	c	c	a	g	g	a	a	a	c	c	c	c	t	g	c	c	c	c	g	t	a	a	a	t	a	c	t	a	t	g	g	a	g	a	a	t	g	c	g	t	t	a	a	a	t	g
KX262864	1	t	a	a	c	g	t	c	a	g	a	g	g	g	g	c	c	a	t	t	c	g	c	c	c	g	c	a	c	c	c	a	g	g	a	a	a	c	c	c	c	t	g	c	t	c	c	g	t	a	a	a	t	a	t	t	a	t	g	g	a	g	a	a	t	g	t	g	t	t	a	a	a	t	g
KX262865	1	t	a	a	c	g	t	c	a	g	a	g	g	g	g	c	c	a	t	t	c	g	c	c	c	g	c	a	c	c	c	a	g	g	a	a	a	c	c	c	c	t	g	c	t	c	c	g	t	a	a	a	t	a	t	t	a	t	g	g	a	g	a	a	t	g	t	g	t	t	a	a	a	t	g
KX262866	1	t	a	a	c	g	t	c	a	g	a	g	g	g	g	c	c	a	t	t	c	g	c	c	c	g	c	a	c	c	c	a	g	g	a	a	a	c	c	c	c	t	g	c	t	c	c	g	t	a	a	a	t	a	t	t	a	t	g	g	a	g	a	a	t	g	t	g	t	t	a	a	a	t	g
EU154348	1	t	a	a	c	g	t	c	a	g	a	g	g	g	g	c	c	a	t	t	c	g	c	c	c	g	c	a	c	c	c	a	g	g	a	a	a	c	c	c	t	t	g	c	t	c	c	g	t	a	a	a	t	a	t	t	a	t	g	g	a	g	a	a	t	g	t	g	t	t	a	a	a	t	g
JN704690	1	t	a	a	c	g	t	c	a	g	a	g	g	g	g	c	c	a	t	t	c	g	c	c	c	g	c	a	c	c	c	a	g	g	a	a	a	c	c	c	c	t	g	c	t	c	c	g	t	a	a	a	t	a	t	t	a	t	g	g	a	g	a	a	t	g	t	g	t	t	a	a	a	t	g
JN704691	1	t	a	a	c	g	t	c	a	g	a	g	g	g	g	c	c	a	t	t	c	g	c	c	c	g	c	a	c	c	c	a	g	g	a	a	a	c	c	c	c	t	g	c	t	c	c	g	t	a	a	a	t	a	t	t	a	t	g	g	a	g	a	a	t	g	t	g	t	t	a	a	a	t	g
JN704692	1	t	a	a	c	g	t	c	a	g	a	g	g	g	g	c	c	a	t	t	c	g	c	c	c	g	c	a	c	c	c	a	g	g	a	a	a	c	c	c	c	t	g	c	t	c	c	g	t	a	a	a	t	a	t	t	a	t	g	g	a	g	a	a	t	g	t	g	t	t	a	a	a	t	g
JN704693	1	t	a	a	c	g	t	c	a	g	a	g	g	g	g	c	c	a	t	t	c	g	c	c	c	g	c	a	c	c	c	a	g	g	a	a	a	c	c	c	c	t	g	c	t	c	c	g	t	a	a	a	t	a	t	t	a	t	g	g	a	g	a	a	t	g	t	g	t	t	a	a	g	t	g
JN704694	1	t	a	a	c	g	t	c	a	g	a	g	g	g	g	c	c	a	t	t	c	g	c	c	c	g	c	a	c	c	c	a	g	g	a	a	a	c	c	c	c	t	g	t	t	c	c	g	t	a	a	a	t	a	t	t	a	t	g	g	a	g	a	a	t	g	t	g	t	t	g	a	a	t	g
JN704695	1	t	a	a	c	g	t	c	a	g	a	g	g	g	g	c	c	a	t	t	c	g	c	c	c	g	c	a	c	c	c	a	g	g	a	a	a	c	c	c	c	t	g	c	t	c	c	g	t	a	a	a	t	a	t	t	a	t	g	g	a	g	a	a	t	g	t	g	t	t	a	a	a	t	g
JN704696	1	t	a	a	c	g	t	c	a	g	a	g	g	g	g	c	c	a	t	t	c	g	c	c	c	g	c	a	c	c	c	a	g	g	a	a	a	c	c	c	c	t	g	c	t	c	c	g	t	a	a	a	t	a	t	t	a	t	g	g	a	g	a	a	t	g	t	g	t	t	a	a	a	t	g
NC001348	1	t	a	a	c	g	t	c	a	g	a	g	g	g	g	c	c	a	t	t	c	g	c	c	c	g	c	a	c	c	c	a	g	g	a	a	a	c	c	c	c	t	g	c	t	c	c	g	t	a	a	a	t	a	t	t	a	t	g	g	a	g	a	a	t	g	t	g	t	t	a	a	a	t	g
KP771892	1	t	a	a	c	g	t	c	a	g	a	g	g	g	g	c	c	a	t	t	c	g	c	c	c	g	c	a	c	c	c	a	g	g	a	a	a	c	c	c	c	t	g	c	t	c	c	g	t	a	a	a	t	a	t	t	a	t	g	g	a	g	a	a	t	g	t	g	t	t	a	a	a	t	g
KP771894	1	t	a	a	c	g	t	c	a	g	a	g	g	g	g	c	c	a	t	t	c	g	c	c	c	g	c	a	c	c	c	a	g	g	a	a	a	c	c	c	c	t	g	c	t	c	c	g	t	a	a	a	t	a	t	t	a	t	g	g	a	g	a	a	t	g	t	g	t	t	a	a	a	t	g
KP771900	1	t	a	a	c	g	t	c	a	g	a	g	g	g	g	c	c	a	t	t	c	g	c	c	c	g	c	a	c	c	c	a	g	g	a	a	a	c	c	c	c	t	g	c	t	c	c	g	t	a	a	a	t	a	t	t	a	t	g	g	a	g	a	a	t	g	t	g	t	t	a	a	a	t	g
KP771901	1	t	a	a	c	g	t	c	a	g	a	g	g	g	g	c	c	a	t	t	c	g	c	c	c	g	c	a	c	c	c	a	g	g	a	a	a	c	c	c	c	t	g	c	t	c	c	g	t	a	a	a	t	a	t	t	a	t	g	g	a	g	a	a	t	g	t	g	t	t	a	a	a	t	g
KP771902	1	t	a	a	c	g	t	c	a	g	a	g	g	g	g	c	c	a	t	t	c	g	c	c	c	g	c	a	c	c	c	a	g	g	a	a	a	c	c	c	c	t	g	c	t	c	c	g	t	a	a	a	t	a	t	t	a	t	g	g	a	g	a	a	t	g	t	g	t	t	a	a	a	t	g
KP771908	1	t	a	a	c	g	t	c	a	g	a	g	g	g	g	c	c	a	t	t	c	g	c	c	c	g	c	a	c	c	c	a	g	g	a	a	a	c	c	c	c	t	g	c	t	c	c	g	t	a	a	a	t	a	t	t	a	t	g	g	a	g	a	a	t	g	t	g	t	t	a	a	a	t	g
KP771912	1	t	a	a	c	g	t	c	a	g	a	g	g	g	g	c	c	a	t	t	c	g	c	c	c	g	c	a	c	c	c	a	g	g	a	a	a	c	c	c	c	t	g	c	t	c	c	g	t	a	a	a	t	a	t	t	a	t	g	g	a	g	a	a	t	g	t	g	t	t	a	a	a	t	g
KP771913	1	t	a	a	c	g	t	c	a	g	a	g	g	g	g	c	c	a	t	t	c	g	c	c	c	g	c	a	c	c	c	a	g	g	a	a	a	c	c	c	c	t	g	c	t	c	c	g	t	a	a	a	t	a	t	t	a	t	g	g	a	g	a	a	t	g	t	g	t	t	a	a	a	t	g
KP771917	1	t	a	a	c	g	t	c	a	g	a	g	g	g	g	c	c	a	t	t	c	g	c	c	c	g	c	a	c	c	c	a	g	g	a	a	a	c	c	c	c	t	g	c	t	c	c	g	t	a	a	a	t	a	t	t	a	t	g	g	a	g	a	a	t	g	t	g	t	t	a	a	a	t	g
KP771918	1	t	a	a	c	g	t	c	a	g	a	g	g	g	g	c	c	a	t	t	c	g	c	c	c	g	c	a	c	c	c	a	g	g	a	a	a	c	c	c	c	t	g	c	t	c	c	g	t	a	a	a	t	a	t	t	a	t	g	g	a	t	a	a	t	g	t	g	t	t	a	a	a	t	g
KP771920	1	t	a	a	c	g	t	c	a	g	a	g	g	g	g	c	c	a	t	t	c	g	c	c	c	g	c	a	c	c	c	a	g	g	a	a	a	c	c	c	c	t	g	c	t	c	c	g	t	a	a	a	t	a	t	t	a	t	g	g	a	g	a	a	t	g	t	g	t	t	a	a	a	t	g
MH709361	1	t	a	a	c	g	t	c	a	g	a	g	g	g	g	c	c	a	t	t	c	g	c	c	c	g	c	a	c	c	c	a	g	t	a	a	a	c	c	c	c	t	g	c	t	c	c	g	t	a	a	a	t	a	t	t	a	t	g	g	a	g	a	a	t	g	t	g	t	t	a	a	a	t	g
KU926318	2	t	a	a	c	g	t	c	a	g	a	g	g	g	g	c	c	a	t	t	c	g	c	c	c	g	c	a	c	c	c	a	g	t	a	a	a	c	c	c	c	t	g	c	t	c	c	g	t	a	a	a	t	a	t	t	a	t	g	g	a	g	a	a	t	g	t	g	t	t	a	a	a	t	g
KU926319	2	t	a	a	c	g	t	c	a	g	a	g	g	g	g	c	c	a	t	t	c	g	c	c	c	g	c	a	c	c	c	a	g	t	a	a	a	c	c	c	c	t	g	c	t	c	c	g	t	a	a	a	t	a	t	t	a	c	g	g	a	g	a	a	t	g	t	g	t	t	a	a	a	t	g
KU926320	2	t	a	a	c	g	t	c	a	g	a	g	g	g	g	c	c	a	t	t	c	g	c	c	c	g	c	a	c	c	c	a	g	t	a	a	a	c	c	c	c	t	g	c	t	c	c	g	t	a	a	a	t	a	t	t	a	t	g	g	a	g	a	a	t	g	t	g	t	t	a	a	a	t	g
KU926321	2	t	a	a	c	g	t	c	a	g	a	g	g	g	g	c	c	a	t	t	c	g	c	c	c	g	c	a	c	c	c	a	g	t	a	a	a	c	c	c	c	t	g	c	t	c	c	g	t	a	a	a	t	a	t	t	a	t	g	g	a	g	a	a	t	g	t	g	t	t	a	a	a	t	g
KU926322	2	t	a	a	c	g	t	c	a	g	a	g	g	g	g	c	c	a	t	t	c	g	c	c	c	g	c	a	c	c	c	a	g	g	a	a	a	c	c	c	c	t	g	c	t	c	c	g	t	a	a	a	t	a	t	t	a	t	g	g	a	g	a	a	t	g	t	g	t	t	a	a	a	t	g
KC847290	2	t	a	a	c	g	t	c	a	g	a	g	g	g	g	c	c	a	t	t	c	g	c	c	c	g	c	a	c	c	c	a	g	t	a	a	a	c	c	c	c	t	g	c	t	c	c	g	g	a	a	a	t	a	t	t	a	t	g	g	a	g	a	a	t	g	t	g	t	t	a	a	a	t	g
KJ767491	2	t	a	a	c	g	t	c	a	g	a	g	g	g	g	c	c	a	t	t	c	g	c	c	c	g	c	a	c	c	c	a	g	g	a	a	a	c	c	c	c	t	g	c	t	c	c	g	t	a	a	a	t	a	t	t	a	t	g	g	a	g	a	a	t	g	t	g	t	t	a	a	a	t	g
KJ767492	2	t	a	a	c	g	t	c	a	g	a	g	g	g	g	c	c	a	t	t	c	g	c	c	c	g	c	a	c	c	c	a	g	t	a	a	a	c	c	c	c	t	g	c	t	c	c	g	t	a	a	a	t	a	t	t	a	t	g	g	a	g	a	a	t	g	t	g	t	t	a	a	a	t	g
KJ808816	2	t	a	a	c	g	t	c	a	g	a	g	g	g	g	c	c	a	t	t	c	g	c	c	c	g	c	a	c	c	c	a	g	t	a	a	a	c	c	c	c	t	g	c	t	c	c	g	t	a	a	a	t	a	t	t	a	t	g	g	a	g	a	a	t	g	t	g	t	t	a	a	a	t	g
KP771925	2	t	a	a	c	g	t	c	a	g	a	g	g	g	g	c	c	a	t	t	c	g	c	c	c	g	c	a	c	c	c	a	g	t	a	a	a	c	c	c	c	t	g	c	t	c	c	g	t	a	c	a	t	a	t	t	a	t	g	g	a	g	a	a	t	g	t	g	t	t	a	a	a	t	g
KU926315	2	t	a	a	c	g	t	c	a	g	a	g	g	g	g	c	c	a	t	t	c	g	c	c	c	g	c	a	c	c	c	a	g	g	a	a	a	c	c	c	c	t	g	c	t	c	c	g	t	a	a	a	t	a	t	t	a	t	g	g	a	g	a	a	t	g	t	g	t	t	a	a	a	t	g
KU926316	2	t	a	a	c	g	t	c	a	g	a	g	g	g	g	c	c	a	t	t	c	g	c	c	c	g	c	a	c	c	c	a	g	g	a	a	a	c	c	c	c	t	g	c	t	c	c	g	t	a	a	a	t	a	t	t	a	t	g	g	a	g	a	a	t	g	t	g	t	t	a	a	a	t	g
KU926317	2	t	a	a	c	g	t	c	a	g	a	g	g	g	g	c	c	a	t	t	c	g	c	c	c	g	c	a	c	c	c	a	g	g	a	a	a	c	c	c	c	t	g	c	t	c	c	g	t	a	a	a	t	a	t	t	a	t	g	g	a	g	a	a	t	g	t	g	t	t	a	a	a	t	g
KY037796	2	t	a	a	c	g	t	c	a	g	a	g	g	g	g	c	c	a	t	t	c	g	c	c	c	g	c	a	c	c	c	a	g	g	a	a	a	c	c	c	c	t	g	c	t	c	c	g	t	a	a	a	t	a	t	t	a	t	g	g	a	g	a	a	t	g	t	g	t	t	a	a	a	g	g
MF004348	2	t	a	a	c	g	t	c	a	g	a	g	g	g	g	c	c	a	t	t	c	g	c	c	c	g	c	a	c	c	c	a	g	t	a	a	a	c	c	c	c	t	g	c	t	c	c	g	t	a	a	a	t	a	t	t	a	t	g	g	a	g	a	a	t	g	t	g	t	t	a	a	a	t	g
MH709309	2	t	a	a	c	g	t	c	a	g	a	g	g	g	g	c	c	a	t	t	c	g	c	c	c	g	c	a	c	c	c	a	g	g	a	a	a	c	c	c	c	t	g	c	t	t	c	g	t	a	a	a	t	a	t	t	a	t	g	g	a	g	a	a	t	g	t	g	t	t	a	a	a	t	g
MH709310	2	t	a	a	c	g	t	c	a	g	a	g	g	g	g	c	c	a	t	t	c	g	c	c	c	g	c	a	c	c	c	a	g	g	a	a	a	c	c	c	c	t	g	c	t	c	c	g	t	a	a	a	t	a	t	t	a	t	g	g	a	g	a	a	t	g	t	g	t	t	a	a	a	t	g
MH709311	2	t	a	a	c	g	t	c	a	g	a	g	g	g	g	c	c	a	t	t	c	g	c	c	c	g	c	a	c	c	c	a	g	g	a	a	a	c	c	c	c	t	g	c	t	c	c	g	t	a	a	a	t	a	t	t	a	t	g	g	a	g	a	a	t	g	t	g	t	t	a	a	a	t	g
MH709313	2	t	a	a	c	g	t	c	a	g	a	g	g	g	g	c	c	a	g	t	c	g	c	c	c	g	c	a	c	c	c	a	g	g	a	a	a	c	c	c	c	t	g	c	t	c	c	g	t	a	a	a	t	a	t	t	a	t	g	g	c	g	a	a	t	g	t	g	t	t	a	a	a	t	g
MH709314	2	t	a	a	c	g	t	c	a	g	a	g	g	g	g	c	c	a	t	t	c	g	c	c	c	g	c	a	c	c	c	a	g	g	a	a	a	c	c	c	c	t	g	c	t	t	c	g	t	a	a	a	t	a	t	t	a	t	g	g	a	g	a	a	t	g	t	g	t	t	a	a	a	t	g
MH709315	2	t	a	a	c	g	t	c	a	g	a	g	g	g	g	c	c	a	t	t	c	g	c	c	c	g	c	a	c	c	c	a	g	g	a	a	a	c	c	c	c	t	g	c	t	t	c	g	t	a	a	a	t	a	t	t	a	t	g	g	a	g	a	a	t	g	t	g	t	t	a	a	a	t	g
MH709319	2	c	a	a	c	g	t	c	a	g	a	g	g	g	g	c	c	a	t	t	c	g	c	c	c	g	c	a	c	c	c	a	g	g	a	a	a	c	c	c	c	t	g	c	t	t	c	g	t	a	a	a	t	a	t	t	a	t	g	g	a	g	a	a	t	g	t	g	t	t	a	a	a	t	g
MH709320	2	t	a	a	c	g	t	c	a	g	a	g	g	g	g	c	c	a	t	t	c	g	c	c	c	g	c	a	c	c	c	a	g	g	a	a	c	c	c	c	c	t	g	c	t	c	c	g	t	a	a	a	t	a	t	t	a	t	g	g	a	g	a	a	t	g	t	g	t	t	a	a	a	t	g
MH709329	2	t	a	a	c	g	t	c	a	g	a	g	g	g	g	c	c	a	t	t	c	g	c	c	c	g	c	a	c	c	c	a	g	g	a	a	a	c	c	c	c	t	g	c	t	t	c	g	t	a	a	a	t	a	t	t	a	t	g	g	a	g	a	a	t	g	t	a	t	t	a	a	a	t	g
MH709331	2	t	a	a	c	g	t	c	a	g	a	g	g	g	g	c	c	a	t	t	c	g	c	c	c	g	c	a	c	c	c	a	g	g	a	a	a	c	c	c	c	t	g	c	t	c	c	g	t	a	a	a	t	a	t	t	a	t	g	g	a	g	a	a	t	g	t	g	t	t	a	a	a	t	g
MH709333	2	t	a	a	c	g	t	c	a	g	a	g	g	g	g	c	c	a	t	t	c	g	c	c	c	g	c	a	c	c	c	a	g	g	a	a	a	c	c	c	c	t	g	c	t	t	c	g	t	a	a	a	t	a	t	t	a	t	g	g	a	g	a	a	t	g	t	g	t	t	a	a	a	t	g
MH709337	2	t	a	a	c	g	t	c	a	g	a	g	g	g	g	c	c	a	t	t	c	g	c	c	c	g	c	a	c	c	c	a	g	g	a	a	a	c	c	c	c	t	g	c	t	c	c	g	t	a	a	a	t	a	t	t	a	t	g	g	a	g	a	a	t	g	t	g	t	t	a	a	a	t	g
MH709339	2	t	a	a	c	g	t	c	a	g	a	g	g	g	g	c	c	a	t	t	t	g	c	c	c	g	c	a	c	c	c	a	g	g	a	a	a	c	c	c	c	t	g	c	t	c	c	g	t	a	a	a	t	a	t	t	a	t	g	g	a	g	a	a	t	g	t	g	t	t	a	a	a	t	g
MH709344	2	t	a	a	c	g	t	c	a	g	a	g	g	g	g	c	c	a	t	t	c	g	c	c	c	g	c	a	c	c	c	a	g	g	a	a	a	c	c	c	c	t	g	c	t	c	c	g	t	a	a	a	t	a	t	t	a	t	g	g	a	g	a	a	t	g	t	g	t	t	a	a	a	t	g
MH709348	2	t	a	a	c	g	t	c	a	g	a	g	g	g	g	c	c	a	t	t	c	g	c	c	c	g	c	a	c	c	c	a	g	g	a	a	a	c	c	c	c	t	g	c	t	c	c	g	t	a	a	a	t	a	t	t	a	t	g	g	a	g	a	a	t	g	t	g	t	t	a	a	a	t	g
MH709355	2	t	a	a	c	g	t	c	a	g	a	g	g	g	g	c	c	a	t	t	c	g	c	c	c	g	c	a	c	c	c	a	g	g	a	a	a	c	c	c	c	t	g	c	t	c	c	g	t	a	a	a	t	a	t	t	a	t	g	g	a	g	a	a	t	g	t	g	t	t	a	a	a	t	g
MH709359	2	t	a	a	c	g	t	c	a	g	a	g	g	g	g	c	c	a	t	t	c	g	c	c	c	g	c	a	c	c	c	a	g	g	a	a	a	c	c	c	c	t	g	c	t	c	c	g	t	a	a	a	t	a	t	t	a	t	g	g	a	g	a	a	t	g	t	g	t	t	a	a	a	t	g
MH709363	2	t	a	a	c	g	t	c	a	g	a	g	g	g	g	c	c	a	t	t	c	g	c	c	c	g	c	a	c	c	c	a	g	g	a	a	c	c	c	c	c	t	g	c	t	c	c	g	t	a	a	a	t	a	t	t	a	t	g	g	a	g	a	a	t	g	t	g	t	t	a	a	a	t	g
MH709366	2	t	a	a	c	g	t	c	a	g	a	g	g	g	g	c	c	a	t	t	c	g	c	c	c	g	c	a	c	c	c	a	g	g	a	a	a	c	c	c	c	t	g	c	t	c	c	g	t	a	a	a	t	a	t	t	a	t	g	g	a	g	a	a	t	g	t	g	t	t	a	a	a	t	g
MH709376	2	t	a	a	c	g	t	c	a	g	a	g	g	g	g	c	c	a	t	t	c	g	c	c	c	g	c	a	c	c	c	a	g	g	a	a	a	c	c	c	t	t	g	c	t	c	c	g	t	a	a	a	t	a	t	t	a	t	g	g	a	g	a	a	t	g	t	g	t	t	a	a	a	t	g
MH709377	2	t	a	a	c	g	t	c	a	g	a	g	g	g	g	c	c	a	t	t	c	g	c	c	c	g	c	a	c	c	c	a	g	t	a	a	a	c	c	c	c	t	g	c	t	c	c	g	t	a	a	a	t	a	t	t	a	t	g	g	a	g	a	a	t	g	t	g	t	t	a	a	a	t	g
**MW316406**	2	t	a	a	c	g	t	c	a	g	a	g	g	g	g	c	c	a	t	t	c	g	c	c	c	g	c	a	c	c	c	a	g	g	a	a	a	c	c	c	c	t	g	c	t	c	c	g	t	a	a	g	t	a	t	t	a	t	g	g	a	g	a	a	t	g	t	g	t	t	a	a	a	t	g
AJ871403	3	t	a	c	c	g	t	c	a	g	a	g	g	g	g	c	c	g	t	t	c	g	c	c	c	g	c	a	c	c	c	a	g	g	a	a	a	c	c	c	c	t	g	c	t	c	c	a	t	a	a	a	t	a	t	c	a	t	g	g	a	g	a	a	t	g	t	g	t	t	a	a	a	t	g
DQ479955	3	t	a	a	c	g	t	a	a	g	a	g	g	g	g	c	c	g	t	t	c	g	c	c	c	g	c	a	c	c	c	a	g	g	a	a	a	c	c	c	c	t	g	c	t	c	c	a	t	a	a	a	t	a	t	c	a	t	g	g	a	g	a	a	t	g	t	g	t	t	a	a	a	t	g
DQ479956	3	t	a	a	c	g	t	c	a	g	a	g	g	g	g	c	c	g	t	t	c	g	c	c	c	g	c	a	c	c	c	a	g	g	a	a	a	c	c	c	c	t	g	c	t	c	c	a	t	a	a	a	t	a	t	c	a	t	g	g	a	g	a	a	t	g	t	g	t	t	a	a	a	t	g
DQ479957	3	t	a	a	c	g	t	c	a	g	a	g	g	g	g	c	c	g	t	t	c	g	c	c	c	g	c	a	c	c	c	a	g	g	a	a	a	c	c	c	c	t	g	c	t	c	c	a	t	a	a	a	t	a	t	c	a	t	g	g	a	g	a	a	t	g	t	g	t	t	a	a	a	t	g
JN704699	3	t	a	a	c	g	t	c	a	g	a	g	g	a	g	c	c	g	t	t	c	g	c	c	c	g	c	a	c	c	c	a	g	g	a	a	a	c	c	c	c	t	g	c	t	c	c	a	t	a	a	a	t	a	t	c	a	t	g	g	a	g	a	a	t	g	t	g	t	t	a	a	a	t	g
JN704700	3	t	a	a	c	g	t	c	a	g	a	g	g	g	g	c	c	g	t	t	c	g	c	c	c	g	c	a	c	c	c	a	g	g	a	a	a	c	c	c	c	t	g	c	t	c	c	a	t	a	a	a	t	a	t	c	a	t	g	g	a	g	a	a	t	g	t	g	t	t	a	a	a	t	g
JN704701	3	t	a	a	c	g	t	c	a	g	a	g	g	g	g	c	c	g	t	t	c	g	c	c	c	g	c	a	c	t	c	a	g	g	a	a	a	c	c	c	c	t	g	c	t	c	c	a	t	a	a	a	t	a	t	c	a	t	g	g	a	g	a	a	t	g	t	g	t	t	a	a	a	t	g
JN704702	3	t	a	a	c	g	t	c	a	g	a	g	g	g	g	c	c	g	t	t	c	g	c	c	c	g	c	a	c	c	c	a	g	g	a	a	a	c	c	c	c	t	g	c	t	c	c	a	t	a	a	a	t	a	t	c	a	t	g	g	a	g	a	a	t	g	t	g	t	t	a	a	a	t	g
JN704703	3	t	a	a	c	g	t	c	a	g	a	g	g	g	g	c	c	g	t	t	c	g	c	c	c	g	c	a	c	c	c	a	g	g	a	a	a	c	c	c	c	t	g	c	t	c	c	a	t	a	a	a	t	a	t	c	a	t	g	g	a	g	a	a	t	g	t	g	t	t	a	a	a	t	g
KP771891	3	t	a	a	c	g	t	c	a	g	a	g	g	g	g	c	c	g	t	t	c	g	c	c	c	g	c	a	c	c	c	a	g	g	a	a	a	c	c	c	c	t	g	c	t	c	c	a	t	a	a	a	t	a	t	c	a	t	g	g	a	g	a	a	t	g	t	g	t	t	a	a	a	t	g
KP771893	3	t	a	a	c	g	t	c	a	g	g	g	g	g	g	c	c	g	t	t	c	g	c	c	c	g	t	a	c	c	c	a	g	g	a	a	a	c	c	c	c	t	g	c	t	c	c	a	t	a	a	a	t	a	t	c	a	t	g	g	a	g	a	a	t	a	t	g	t	t	a	a	a	t	g
KP771895	3	t	a	a	c	g	t	c	a	g	a	g	g	g	g	c	c	g	t	t	c	g	c	c	c	g	c	a	c	c	c	a	g	g	a	a	a	c	c	c	c	t	g	c	t	c	c	a	t	a	a	a	t	a	t	c	a	t	g	g	a	g	a	a	t	g	t	g	t	t	a	a	a	t	g
KP771896	3	t	a	a	c	g	t	c	a	g	a	g	g	g	g	c	c	g	t	t	c	g	c	c	c	g	c	a	c	c	c	a	g	g	a	a	a	c	c	c	c	t	g	c	t	c	c	a	t	a	a	a	t	a	t	c	a	t	g	g	a	g	a	a	t	g	t	g	t	t	a	a	a	t	g
KP771897	3	t	a	a	c	g	t	c	a	g	a	g	g	g	g	c	c	g	t	t	c	a	c	c	c	g	c	a	c	c	c	a	g	g	a	a	a	c	c	c	c	t	g	c	t	c	c	a	t	a	a	a	t	a	t	c	a	t	g	g	a	g	a	a	t	g	t	g	t	t	a	a	a	t	g
KP771899	3	t	a	a	c	g	t	c	a	g	a	g	g	g	g	c	c	g	t	t	c	g	c	c	c	g	c	a	c	c	c	a	g	g	a	a	a	c	c	c	c	t	g	c	t	c	c	a	t	a	a	a	t	a	t	c	a	t	g	g	a	g	a	a	t	g	t	g	t	t	a	a	a	t	g
KP771903	3	t	a	a	c	g	t	c	a	g	a	g	g	g	g	c	c	g	t	t	c	g	c	c	c	g	c	a	c	c	c	a	g	g	a	a	a	c	c	c	c	t	g	c	t	c	c	a	t	a	a	a	t	a	t	c	a	t	g	g	a	g	a	a	t	g	t	g	t	t	a	a	a	t	g
KP771907	3	t	a	a	c	g	t	c	a	g	a	g	g	g	g	c	c	g	t	t	c	g	c	c	c	g	c	a	c	c	c	a	g	g	a	a	a	c	c	c	c	t	g	c	t	c	c	a	t	a	a	a	t	a	t	c	a	t	g	g	a	g	a	a	t	g	t	g	t	t	a	a	a	t	g
KP771910	3	t	a	a	c	g	t	c	a	g	g	g	g	g	g	c	c	g	t	t	c	g	c	c	c	g	c	a	c	c	c	a	t	g	a	a	a	c	c	c	c	t	g	c	t	c	c	a	t	a	a	a	t	a	t	c	a	t	g	g	a	g	a	a	t	a	t	g	t	t	a	a	a	t	g
KP771915	3	t	a	a	c	g	t	c	a	g	a	g	g	g	g	c	c	g	t	t	c	g	c	c	c	g	c	a	c	c	c	a	g	g	a	a	a	c	c	c	c	t	g	c	t	c	c	a	t	a	a	a	t	a	t	c	a	t	g	g	a	g	a	a	t	g	t	g	t	t	a	a	a	t	g
KP771916	3	t	a	a	c	g	t	c	a	g	a	g	g	g	g	c	c	g	t	t	c	g	c	c	c	g	c	a	c	c	c	a	g	g	a	a	a	c	c	c	c	t	g	c	t	c	c	a	t	a	a	a	t	a	t	c	a	t	g	g	a	g	a	a	t	g	t	g	t	t	a	a	a	t	g
KP771921	3	t	a	a	c	g	t	c	a	g	a	g	g	g	g	c	c	g	t	t	c	g	c	c	c	g	c	a	c	c	c	a	g	g	a	a	a	c	c	c	c	t	g	c	t	c	c	a	t	a	a	a	t	a	t	c	a	t	a	g	a	g	a	a	t	g	t	g	t	t	a	a	a	t	g
KP771923	3	t	a	a	c	g	t	c	a	g	a	g	g	g	g	c	c	g	t	t	c	g	c	c	c	g	c	g	c	c	c	a	g	g	a	a	a	c	c	c	c	t	g	c	t	c	c	a	t	a	a	a	t	a	t	c	a	t	g	g	a	g	a	a	t	g	t	g	t	t	a	a	a	t	g
KP771889	3	t	a	a	c	g	t	c	a	g	a	g	g	g	g	c	c	g	t	t	c	g	c	c	c	g	c	a	c	c	c	a	g	g	a	a	a	c	c	c	c	t	g	c	t	c	c	a	t	a	a	a	t	a	t	t	a	t	g	g	a	g	a	a	t	g	t	g	t	t	a	a	a	t	g
KU926311	3	t	a	a	c	g	t	c	a	g	a	g	g	g	g	c	c	g	t	t	c	g	c	c	c	g	c	a	c	c	c	a	g	g	a	a	a	c	c	c	c	t	g	c	t	c	c	a	t	a	a	a	t	a	t	c	a	t	g	g	a	g	a	a	t	g	t	g	t	t	a	a	a	t	g
JQ911599	3	t	a	a	c	g	t	c	a	g	a	g	g	g	g	c	c	g	t	t	c	g	c	c	c	a	c	a	c	c	c	a	g	g	a	a	a	c	c	c	c	t	g	c	t	c	c	a	t	a	a	a	t	a	t	c	a	t	g	g	a	g	a	c	t	g	t	g	t	t	a	a	a	t	g
MH709317	3	t	a	a	c	g	t	c	a	g	a	a	g	g	g	c	c	g	t	t	c	g	c	c	c	g	c	a	a	c	c	a	g	g	a	a	a	c	c	c	c	c	a	c	t	c	c	a	t	c	a	a	t	a	t	c	a	t	g	g	a	g	a	a	t	g	t	g	t	t	a	a	a	t	g
MH709346	3	t	a	a	c	g	t	c	a	g	a	a	g	g	g	c	c	g	t	t	c	g	c	c	c	g	c	a	a	c	c	a	g	g	a	a	a	c	c	c	c	c	a	c	t	c	c	a	t	c	a	a	t	a	t	c	a	t	g	g	a	g	a	a	t	g	t	g	t	t	a	a	a	t	g
MH709375	3	t	a	a	c	g	t	c	a	g	a	a	g	g	g	c	c	g	t	t	c	g	c	c	c	g	c	a	c	c	c	a	g	g	a	a	a	c	c	c	c	c	g	c	t	c	c	a	t	c	a	a	t	a	t	t	a	t	g	g	a	g	a	a	t	g	t	g	t	t	a	a	a	t	g
DQ452050	4	t	a	a	c	g	t	c	a	g	a	g	a	g	g	c	c	a	t	t	c	g	c	c	c	g	c	a	c	c	c	a	g	g	a	a	a	c	c	c	c	t	g	c	t	c	c	g	t	a	a	a	t	a	t	t	a	t	g	g	a	g	a	a	t	g	t	g	t	t	a	a	a	t	g
DQ479960	4	t	a	a	c	g	t	c	a	g	a	g	g	g	g	c	c	a	t	t	c	g	c	c	c	g	c	a	c	c	c	a	g	g	a	a	a	c	c	c	c	t	g	c	t	c	c	g	t	a	a	a	t	a	t	t	a	t	g	g	a	g	a	a	t	g	t	g	t	t	a	a	a	t	g
KP771898	4	t	a	a	c	g	t	c	a	g	a	g	g	g	g	c	c	a	t	t	c	g	c	c	c	g	c	a	c	c	c	a	g	g	a	a	a	c	c	c	c	t	g	c	t	c	c	g	t	a	a	a	t	a	t	t	a	t	g	g	a	g	a	a	t	g	t	g	t	t	a	a	a	t	g
MH709316	4	t	a	a	c	g	t	c	a	g	a	g	g	g	g	c	c	a	t	t	c	g	c	c	c	g	c	a	c	c	c	a	g	g	a	a	a	c	c	c	c	t	g	c	t	c	c	g	t	a	a	a	t	a	t	t	a	t	g	g	a	g	a	a	t	g	t	g	t	t	a	a	a	t	g
MH709318	4	t	a	a	c	g	t	c	a	g	a	g	g	g	g	c	c	a	t	t	c	g	c	c	c	g	c	a	c	c	c	a	g	g	a	a	a	c	c	c	c	t	g	c	t	c	c	g	t	a	a	a	t	a	t	t	a	t	g	g	a	g	a	a	t	g	t	g	t	t	a	a	a	t	g
MH709322	4	t	a	a	c	g	t	c	a	g	a	g	g	g	g	c	c	a	t	t	c	g	c	c	c	g	c	a	c	c	c	a	g	g	a	a	a	c	c	c	c	t	g	c	t	c	c	g	t	a	a	a	t	a	t	t	a	t	g	g	a	g	a	a	t	g	t	g	t	t	a	a	a	t	g
MH709323	4	t	a	a	c	g	t	c	a	g	a	g	g	g	g	c	c	a	t	t	c	g	c	c	c	g	c	a	c	c	c	a	g	g	g	a	a	c	c	c	c	t	g	c	t	c	c	g	t	a	a	a	t	a	t	t	a	t	g	g	a	g	a	a	t	g	t	g	t	t	a	a	a	t	g
MH709326	4	t	a	a	t	g	t	c	a	g	a	g	g	g	g	c	c	a	t	t	c	g	c	c	c	g	c	a	c	c	c	a	g	g	a	a	a	c	c	c	c	t	g	c	t	c	c	g	t	a	a	a	t	a	t	t	a	t	g	g	a	g	a	a	t	g	t	g	t	t	a	a	a	t	g
MH709341	4	t	a	a	c	g	t	c	a	g	a	g	g	g	g	c	c	a	t	t	c	g	c	c	c	g	c	a	c	c	c	a	g	g	a	a	a	c	c	c	c	t	g	c	t	c	c	g	t	a	a	a	t	a	t	t	a	t	g	g	a	g	a	a	t	g	t	g	t	t	a	a	a	t	g
MH709357	4	t	a	a	c	g	t	c	a	g	a	g	g	g	g	c	c	a	t	t	c	g	c	c	c	g	c	a	c	c	c	a	g	g	a	a	a	c	c	c	c	t	g	c	t	c	c	g	t	a	a	a	t	a	t	t	a	t	g	g	a	g	a	a	t	g	t	g	t	t	a	a	a	t	g
MH709364	4	t	a	a	c	g	t	c	a	g	a	a	g	g	g	c	c	a	t	t	c	g	c	c	c	g	c	a	c	c	c	a	g	g	a	a	a	c	c	c	c	t	g	c	t	c	c	g	t	a	a	a	t	a	t	t	a	t	g	g	a	g	a	a	t	g	t	g	t	c	a	a	a	t	g
MH709370	4	t	a	a	c	g	t	c	a	g	a	g	g	g	g	c	c	a	t	t	c	g	c	c	c	g	c	a	c	c	c	a	g	g	a	a	a	c	c	c	c	t	g	c	t	c	c	g	t	a	a	a	t	a	t	t	a	t	g	g	a	g	a	a	t	g	t	g	t	t	a	a	a	t	g
MH709371	4	t	a	a	c	g	t	c	a	g	a	g	g	g	g	c	c	a	t	t	c	g	c	c	c	g	c	a	c	c	c	a	g	g	a	a	a	c	c	c	c	t	g	c	t	c	c	g	t	a	a	a	t	a	t	t	a	t	g	g	a	g	a	a	t	g	t	g	t	t	a	a	a	t	g
MH709372	4	t	a	a	c	g	t	c	a	g	a	g	g	g	g	c	c	a	t	t	c	g	c	c	c	g	c	a	c	c	c	a	g	g	a	a	a	c	c	c	c	t	g	c	t	c	c	g	t	a	a	a	t	a	t	t	a	t	g	g	a	g	a	a	t	g	t	g	t	t	a	a	a	t	g
DQ457052	5	t	a	a	c	g	g	c	a	g	a	g	g	g	g	c	c	a	t	t	c	g	t	t	t	g	c	a	c	c	c	a	g	g	a	a	a	a	c	c	c	t	g	c	t	c	c	a	t	a	a	a	t	a	t	t	a	t	g	g	a	g	g	a	t	g	t	g	t	t	a	a	a	t	g
JN704704	5	t	a	a	c	g	g	c	a	g	a	g	g	g	g	c	c	g	t	t	c	g	c	c	c	g	c	a	c	c	c	a	g	g	a	a	a	c	c	c	c	t	g	c	t	c	c	a	t	a	a	a	t	a	t	t	a	t	g	g	a	g	a	a	t	g	t	g	t	t	a	a	a	t	g
JN704705	5	t	a	a	c	g	g	c	a	g	a	g	g	g	g	c	c	g	t	t	c	g	c	c	c	g	c	a	c	c	c	c	g	g	a	a	a	c	c	c	c	t	g	c	t	c	c	a	t	a	a	a	g	a	t	t	a	t	g	g	a	g	a	a	t	g	t	g	t	t	a	a	a	t	g
JN704706	5	t	a	a	c	g	g	c	a	g	a	g	g	g	g	c	c	g	t	t	c	g	c	c	c	g	c	a	c	c	c	a	g	g	a	a	a	c	c	c	c	t	g	c	t	c	c	a	t	a	a	a	g	a	t	t	a	t	g	g	a	g	a	a	t	g	t	g	t	t	a	a	a	t	g
JN704707	5	t	a	a	c	g	g	c	a	c	a	g	g	g	g	c	c	g	t	t	c	g	c	c	c	g	c	a	c	c	c	a	g	g	a	a	a	c	c	c	c	t	g	c	t	c	c	a	t	a	a	a	t	a	t	t	a	t	g	g	a	g	a	a	t	g	t	g	t	t	a	a	a	t	a
JN704708	5	t	a	a	c	g	g	c	a	g	a	g	g	g	g	c	c	g	t	t	c	g	c	c	c	g	c	a	c	c	c	c	g	g	a	a	a	c	c	c	c	t	g	c	t	c	c	a	t	a	a	a	g	a	t	t	a	t	g	g	a	g	a	a	t	g	t	g	t	t	a	a	a	t	g
KP771904	5	t	a	a	c	g	g	c	a	g	a	g	g	g	g	c	c	g	t	t	c	g	c	c	c	g	c	a	c	c	c	a	g	g	a	a	a	c	c	c	c	t	g	c	t	c	c	a	t	a	a	a	t	a	t	t	a	t	g	g	a	g	a	a	t	g	t	g	t	t	a	a	a	t	g
KP771905	5	t	a	a	c	g	g	c	a	g	a	g	g	g	g	c	c	g	t	t	c	g	c	c	c	g	c	a	c	c	c	a	g	g	a	a	a	c	c	c	c	t	g	c	t	c	c	a	t	a	a	a	t	a	t	t	a	t	g	g	a	g	a	a	t	g	t	g	t	t	a	a	a	t	g
KP771906	5	t	a	a	c	g	g	c	a	g	a	g	g	g	g	c	c	g	t	t	c	g	c	c	c	g	c	a	c	c	c	a	g	g	a	a	a	c	c	c	c	t	g	c	t	c	c	a	t	a	a	a	t	a	t	t	a	t	g	g	a	g	a	a	t	g	t	g	t	t	a	a	a	t	g
KP771919	5	t	a	a	c	g	g	c	a	g	a	g	g	g	g	c	c	g	t	t	c	g	c	c	c	g	c	a	c	c	c	a	g	g	a	a	a	c	c	c	c	t	g	c	t	c	c	a	t	a	a	a	t	a	t	t	a	t	g	g	a	g	a	a	t	g	t	g	t	t	a	a	a	t	g
KP771922	5	t	a	a	c	g	g	c	a	g	a	g	g	g	g	c	c	g	t	t	c	g	c	c	c	g	c	a	c	c	c	c	g	g	a	a	a	c	c	c	c	t	g	c	t	c	c	a	t	a	a	a	g	a	t	t	a	t	g	g	a	g	a	a	t	g	t	g	t	t	a	a	a	t	g
KP771924	5	t	a	a	c	g	g	c	a	g	a	g	g	g	g	c	c	g	t	t	c	g	c	c	c	g	c	a	c	c	c	c	g	g	a	a	a	c	c	c	c	t	g	c	t	c	c	a	t	a	a	a	g	a	t	t	a	t	g	g	a	g	a	a	t	g	t	g	t	t	a	a	a	t	g
KM355696	5	t	a	a	c	g	g	c	a	g	a	g	g	g	g	c	c	g	t	t	c	g	c	c	c	g	c	a	c	c	c	c	g	g	a	a	a	c	c	c	c	t	g	c	t	c	c	a	t	a	a	a	g	a	t	t	a	t	g	g	a	g	a	a	t	g	t	g	t	t	a	a	a	t	g
KM355697	5	t	a	a	c	g	g	c	a	g	a	g	g	g	g	c	c	g	t	t	c	g	c	c	c	g	c	a	c	c	c	c	g	g	a	a	a	c	c	c	c	t	g	c	t	c	c	a	t	a	a	a	g	a	t	t	a	t	g	g	a	g	a	a	t	g	t	g	t	t	a	a	a	t	g
KM355698	5	t	a	a	c	g	g	c	a	g	a	g	g	g	g	c	c	g	t	t	c	g	c	c	c	g	c	a	c	c	c	c	g	g	a	a	a	c	c	c	c	t	g	c	t	c	c	a	t	a	a	a	g	a	t	t	a	t	g	g	a	g	a	a	t	g	t	g	t	t	a	a	a	t	g
KM355699	5	t	a	a	c	g	g	c	a	g	a	g	g	g	g	c	c	g	t	t	c	g	c	c	c	g	c	a	c	c	c	c	g	g	a	a	a	c	c	c	c	t	g	c	t	c	c	a	t	a	a	a	g	a	t	t	a	t	g	g	a	g	a	a	t	g	t	g	t	t	a	a	a	t	g
KM355701	5	t	a	a	c	g	g	c	a	g	a	g	g	g	g	c	c	g	t	t	c	g	c	c	c	g	c	a	c	c	c	c	g	g	a	a	a	c	c	c	c	t	g	c	t	c	c	a	t	a	a	a	g	a	t	t	a	t	g	g	a	g	a	a	t	g	t	g	t	t	a	a	a	t	g
KM355702	5	t	a	a	c	g	g	c	a	g	a	g	g	g	g	c	c	g	t	t	c	g	c	c	c	g	c	a	c	c	c	c	g	g	a	a	a	c	c	c	c	t	g	c	t	c	c	a	t	a	a	a	g	a	t	t	a	t	g	g	a	g	a	a	t	g	t	g	t	t	a	a	a	t	g
KM355703	5	t	a	a	c	g	g	c	a	g	a	g	g	g	g	c	c	g	t	t	c	g	c	c	c	g	c	a	c	c	c	c	g	g	a	a	a	c	c	c	c	t	g	c	t	c	c	a	t	a	a	a	g	a	t	t	a	t	g	g	a	g	a	a	t	g	t	g	t	t	a	a	a	t	g
KM355704	5	t	a	a	c	g	g	c	a	g	a	g	g	g	g	c	c	g	t	t	c	g	c	c	c	g	c	a	c	c	c	c	g	g	a	a	a	c	c	c	c	t	g	c	t	c	c	a	t	a	a	a	g	a	t	t	a	t	g	g	a	g	a	a	t	g	t	g	t	t	a	a	a	t	g
KM355705	5	t	a	a	c	g	g	c	a	g	a	g	g	g	g	c	c	g	t	t	c	g	c	c	c	g	c	a	c	c	c	c	g	g	a	a	a	c	c	c	c	t	g	c	t	c	c	a	t	a	a	a	g	a	t	t	a	t	g	g	a	g	a	a	t	g	t	g	t	t	a	a	a	t	g
KM355706	5	t	a	a	c	g	g	c	a	g	a	g	g	g	g	c	c	g	t	t	c	g	c	c	c	g	c	a	c	c	c	c	g	g	a	a	a	c	c	c	c	t	g	c	t	c	c	a	t	a	a	a	g	a	t	t	a	t	g	g	a	g	a	a	t	g	t	g	t	t	a	a	a	t	g
KM355707	5	t	a	a	c	g	g	c	a	g	a	g	g	g	a	c	c	g	t	t	c	g	c	c	c	g	c	a	c	c	c	c	g	g	a	a	a	c	c	c	c	t	g	c	t	c	c	a	t	a	a	a	g	a	t	t	a	t	g	g	a	g	a	a	t	g	t	g	t	t	a	a	a	t	g
KM355708	5	t	a	a	c	g	g	c	a	g	a	g	g	g	a	c	c	g	t	t	c	g	c	c	c	g	c	a	c	c	c	c	g	g	a	a	a	c	c	c	c	t	g	c	t	c	c	a	t	a	a	a	g	a	t	t	a	t	g	g	a	g	a	a	t	g	t	g	t	t	a	a	a	t	g
KM355709	5	t	a	a	c	g	g	c	a	g	a	g	g	g	g	c	c	g	t	t	c	g	c	c	c	g	c	a	c	c	c	c	g	g	a	a	a	c	c	c	c	t	g	c	t	c	c	a	t	a	a	a	g	a	t	t	a	t	g	g	a	g	a	a	t	g	t	g	t	t	a	a	a	t	g
KM355711	5	t	a	a	c	g	g	c	a	g	a	g	g	g	g	c	c	g	t	t	c	g	c	c	c	g	c	a	c	c	c	c	g	g	a	a	a	c	c	c	c	t	g	c	t	c	c	a	t	a	a	a	g	a	t	t	a	t	g	g	a	g	a	a	t	g	t	g	t	t	a	a	a	t	g
KM355712	5	t	a	a	c	g	g	c	a	g	a	g	g	g	g	c	c	g	t	t	c	g	c	c	c	g	c	a	c	c	c	c	g	g	a	a	a	c	c	c	c	t	g	c	t	c	c	a	t	a	a	a	g	a	t	t	a	t	g	g	a	g	a	a	t	g	t	g	t	t	a	a	a	t	g
KY037797	5	[illegible]	[illegible]	[illegible]	[illegible]	[illegible]	[illegible]	[illegible]	[illegible]	[illegible]	[illegible]	[illegible]	[illegible]	[illegible]	[illegible]	[illegible]	[illegible]	[illegible]	[illegible]	[illegible]	[illegible]	[illegible]	[illegible]	[illegible]	[illegible]	[illegible]	[illegible]	[illegible]	[illegible]	[illegible]	[illegible]	[illegible]	[illegible]	[illegible]	[illegible]	[illegible]	[illegible]	[illegible]	[illegible]	[illegible]	[illegible]	[illegible]	[illegible]	[illegible]	[illegible]	[illegible]	[illegible]	[illegible]	[illegible]	[illegible]	[illegible]	[illegible]	[illegible]	[illegible]	[illegible]	[illegible]	[illegible]	[illegible]	[illegible]	[illegible]	[illegible]	[illegible]	[illegible]	[illegible]	[illegible]	[illegible]	[illegible]	[illegible]	[illegible]	[illegible]	[illegible]	[illegible]	[illegible]	[illegible]	[illegible]

Supplementary Table 3: 2880 SN

GB-ID	Clade markers						5																																														SD14																							
	Position in Dumas	74452	74560	74563	74620	74638	74699	74743	74782	74861	74932	74941	74943	74972	75042	75054	75073	75103	75116	75173	75177	75251	75255	75256	75259	75568	75637	75746	75751	75755	75836	75888	75897	76131	76212	76218	76236	76290	76293	76329	76377	76389	76509	76510	76522	76530	76575	76779	76846	76907	76939	76953	76985	76992	77102	77154	77169	77220	77286	77393	77424	77580	77592	77616	77619	77691	77729	77817	77898	77913	77951	77979	77985	78090	78105
MH499467	5	t	a	a	c	g	g	c	a	g	a	g	g	g	g	c	c	g	t	t	c	g	c	c	c	g	c	a	c	c	c	a	g	g	a	a	a	c	t	c	c	t	g	c	t	c	c	a	t	a	a	a	t	a	t	t	a	t	g	g	a	g	a	a	t	g	t	g	t	t	a	a	a	t	g
MH499469	5	t	a	a	c	g	g	c	a	g	a	g	g	g	g	c	c	g	t	t	c	g	c	c	c	g	c	a	c	c	c	a	g	g	a	a	a	c	c	c	c	t	g	c	t	c	c	a	t	a	a	a	t	a	t	t	a	t	g	g	a	g	a	a	t	g	t	g	t	t	a	a	a	t	g
MH709321	5	t	a	a	c	g	g	c	a	g	a	g	g	g	g	c	c	g	t	t	c	g	c	c	c	g	c	a	c	c	c	a	g	g	a	a	a	c	c	c	c	t	g	c	t	c	c	a	t	a	a	a	g	a	t	t	a	t	g	g	a	g	a	a	t	g	t	g	t	t	a	a	a	t	g
MH709324	5	t	a	a	c	g	g	c	a	g	a	g	g	g	g	c	c	g	t	t	c	g	c	c	c	g	c	a	c	c	c	a	g	g	a	a	a	c	c	c	c	t	g	c	t	c	c	a	t	a	a	a	t	a	t	t	a	t	g	g	a	g	a	a	t	g	t	g	t	t	a	a	a	t	g
MH709325	5	t	a	a	c	g	g	c	a	g	a	g	g	g	g	c	c	g	t	t	c	g	c	c	c	g	c	a	c	c	c	c	g	g	a	a	a	c	c	c	c	t	g	c	t	c	c	a	t	a	a	a	g	a	t	t	a	t	g	g	a	g	a	a	t	g	t	g	t	t	a	a	a	t	g
MH709327	5	t	a	a	c	g	g	c	a	g	a	g	g	g	g	c	c	g	t	t	c	g	c	c	c	g	c	a	c	c	c	a	g	g	a	a	a	c	c	c	c	t	g	c	t	c	c	a	t	a	a	a	t	a	t	t	a	t	g	g	a	g	g	a	t	g	t	g	t	t	a	a	a	t	g
MH709328	5	t	a	a	c	g	g	c	a	g	a	g	g	g	g	c	c	g	t	t	c	g	c	c	c	g	c	a	c	c	c	a	g	g	a	a	a	c	c	c	c	t	g	c	t	c	c	a	t	a	a	a	t	a	t	t	a	t	g	g	a	g	a	a	t	g	t	g	t	t	a	a	a	t	g
MH709330	5	t	a	a	c	g	g	c	a	g	a	g	g	g	g	c	c	g	t	t	c	g	c	c	c	g	c	a	c	c	c	a	g	g	a	a	a	c	c	c	c	t	g	c	t	c	c	a	t	a	a	a	t	a	t	t	a	t	g	g	a	g	a	a	t	g	t	g	t	t	a	a	a	t	g
MH709332	5	t	a	a	c	g	g	c	a	g	a	g	g	g	g	c	c	g	t	t	c	g	c	c	c	g	c	a	c	c	c	a	g	g	a	a	a	c	c	c	c	t	g	c	t	c	c	a	t	a	a	a	t	a	t	t	a	t	g	g	a	g	a	a	t	g	t	g	t	t	a	a	a	t	g
MH709334	5	t	a	a	c	g	g	c	a	g	a	g	g	g	g	c	c	g	t	t	c	g	c	c	c	g	c	a	c	c	c	a	g	g	a	a	a	c	c	c	c	t	g	c	t	c	c	a	t	a	a	a	t	a	t	t	a	t	g	g	a	g	g	a	t	g	t	g	t	t	a	a	a	t	g
MH709335	5	t	a	a	c	g	g	c	a	g	a	g	g	g	g	c	c	g	t	g	c	g	t	t	t	g	c	a	c	c	c	a	g	g	a	a	a	c	c	c	c	t	g	c	t	c	c	a	t	a	a	a	t	a	t	t	a	t	g	g	a	g	g	a	t	g	t	g	t	t	a	a	a	t	g
MH709336	5	t	a	a	c	g	g	c	a	g	a	g	g	g	g	c	c	g	t	t	c	g	c	c	c	g	c	a	c	c	c	a	g	g	a	a	a	c	c	c	c	t	g	c	t	c	c	a	t	a	a	a	t	a	t	t	a	t	g	g	a	g	g	a	t	g	t	g	t	t	a	a	a	t	g
MH709338	5	t	a	a	c	g	g	c	a	g	a	g	g	g	g	c	c	g	t	t	c	g	c	c	c	g	c	a	c	c	c	a	g	g	a	a	a	c	c	c	c	t	g	c	t	c	c	a	t	a	a	a	t	a	t	t	a	t	g	g	a	g	g	a	t	g	t	g	t	t	a	a	a	t	g
MH709340	5	t	a	a	c	g	g	c	a	g	a	g	g	g	g	c	c	g	t	t	c	g	c	c	c	g	c	a	c	c	c	a	g	g	a	a	a	c	c	c	c	t	g	c	t	c	c	a	t	a	a	a	t	a	t	t	a	t	g	g	a	g	a	a	t	g	t	g	t	t	a	a	a	t	g
MH709342	5	t	a	a	c	g	g	c	a	g	a	g	g	g	g	c	c	g	t	t	c	g	c	c	c	g	c	a	c	c	c	a	g	g	a	a	a	c	c	c	c	t	g	c	t	c	c	a	t	a	a	a	g	a	t	t	a	t	g	g	a	g	a	a	t	g	t	g	t	t	a	a	a	t	g
MH709343	5	t	a	a	c	g	g	c	a	g	a	g	g	g	g	c	c	g	t	t	c	g	c	c	c	g	c	a	c	c	c	a	g	g	a	a	a	c	c	c	c	t	g	c	t	c	c	a	t	a	a	a	t	a	t	t	a	t	g	g	a	g	a	a	t	g	t	g	t	t	a	a	a	t	g
MH709345	5	t	a	a	c	g	g	c	a	g	a	g	g	g	g	c	c	g	t	t	c	g	t	t	t	g	c	a	c	c	c	a	g	g	a	a	a	a	c	c	c	t	g	c	t	c	c	a	t	a	a	a	t	a	t	t	a	t	g	g	a	g	g	a	t	g	t	g	t	t	a	a	a	t	g
MH709347	5	t	a	a	c	g	g	c	a	g	a	g	g	g	g	c	c	g	t	t	c	g	c	c	c	g	c	a	c	c	c	a	g	g	a	a	a	c	c	c	c	t	g	c	t	c	c	a	t	a	a	a	t	a	t	t	a	t	g	g	a	g	g	a	t	g	t	g	t	t	a	a	a	t	g
MH709349	5	t	a	a	c	g	g	c	a	g	a	g	g	g	g	c	c	g	t	t	c	g	c	c	c	g	c	a	c	c	c	a	g	g	a	a	a	c	c	c	c	t	g	c	t	c	c	a	t	a	a	a	t	a	t	t	a	t	g	g	a	g	a	a	t	g	t	g	t	t	a	a	a	t	g
MH709350	5	t	a	a	c	g	g	c	a	g	a	g	g	g	g	c	c	g	t	t	c	g	c	c	c	g	c	a	c	c	c	a	g	g	a	a	a	c	c	c	c	t	g	c	t	c	c	a	t	a	a	a	t	a	t	t	a	t	g	g	a	g	g	a	t	g	t	g	t	t	a	a	a	t	g
MH709351	5	t	a	a	c	g	g	c	a	g	a	g	g	g	g	c	c	g	t	t	c	g	c	c	c	g	c	a	c	c	c	a	g	g	a	a	a	c	c	c	c	t	g	c	t	c	c	a	t	a	a	a	t	a	t	t	a	t	g	g	a	g	a	a	t	g	t	g	t	t	a	a	a	t	g
MH709353	5	t	a	a	c	g	g	c	a	g	a	g	g	g	g	c	c	g	t	t	c	g	t	t	t	g	c	a	c	c	c	a	g	g	a	a	a	c	c	c	c	t	g	c	t	c	c	a	t	a	a	a	t	a	t	t	a	t	g	g	a	g	g	a	t	g	t	g	t	t	a	a	a	t	g
MH709358	5	t	a	a	c	g	g	c	a	g	a	g	g	g	g	c	c	g	t	t	c	g	c	c	c	g	c	a	c	c	c	c	g	g	a	a	a	c	c	c	c	t	g	c	t	c	c	a	t	a	a	a	g	a	t	t	a	t	g	g	a	g	a	a	t	g	t	g	t	t	a	a	a	t	g
MH709360	5	t	a	a	c	g	g	c	a	g	a	g	g	g	g	c	c	g	t	t	c	g	c	c	c	g	c	a	c	c	c	a	g	g	a	a	a	c	c	c	c	t	g	c	t	c	c	a	t	a	a	a	t	a	t	t	a	t	g	g	a	g	a	a	t	g	t	g	t	t	a	a	a	t	g
MH709362	5	t	a	a	c	g	g	c	a	g	a	g	g	g	g	c	c	g	t	t	c	g	t	t	t	g	c	a	c	c	c	a	g	g	a	a	a	a	c	c	c	t	g	c	t	c	c	a	t	a	a	a	t	a	t	t	a	t	g	g	a	g	g	a	t	g	t	g	t	t	a	c	a	t	g
MH709365	5	t	a	a	c	g	g	c	a	g	a	g	g	g	g	c	c	g	t	t	c	g	c	c	c	g	c	a	c	c	c	a	g	g	a	a	a	c	c	c	c	t	g	c	t	c	c	a	t	a	a	a	t	a	t	t	a	t	g	g	a	g	g	a	t	g	t	g	t	t	a	a	a	t	g
MH709373	5	t	a	a	c	g	g	c	a	g	a	g	g	g	g	t	c	g	t	t	c	g	c	c	c	g	c	a	c	c	c	a	g	g	a	a	a	c	c	c	c	t	g	c	t	c	c	a	t	a	a	a	t	a	t	t	a	t	g	g	a	g	a	a	t	g	t	g	t	t	a	a	a	t	g
MH709374	5	t	a	a	c	g	g	c	a	g	a	g	g	g	g	c	c	g	t	t	c	g	t	t	t	g	c	a	c	c	c	a	g	g	a	a	a	c	c	c	c	t	g	c	t	c	c	a	t	a	a	a	t	a	t	t	a	t	g	g	a	g	g	a	t	g	t	g	t	t	a	a	a	t	g
KC112914	6	t	a	a	c	g	t	c	a	g	a	g	g	g	g	c	a	g	t	t	c	g	c	c	c	g	c	a	c	c	c	a	g	g	a	a	a	c	c	c	c	t	g	c	t	c	c	a	t	a	a	a	t	a	t	c	g	t	g	g	a	g	a	a	t	g	t	g	t	t	a	a	a	t	g
KP771914	6	t	a	a	c	g	t	c	a	g	a	g	g	g	g	c	a	g	t	t	c	g	c	c	c	g	c	a	c	c	c	a	g	g	a	a	a	c	c	c	c	t	g	c	t	c	c	a	t	a	a	a	t	a	t	c	a	t	g	g	a	g	a	a	t	g	t	g	t	t	a	a	a	t	g
KP702725	6	t	g	a	c	g	t	c	a	g	a	g	g	g	g	c	a	g	t	t	c	g	c	c	c	g	c	a	c	c	c	a	g	g	a	a	a	c	c	c	c	t	g	c	t	c	c	a	t	a	a	a	t	a	t	c	g	t	g	g	a	g	a	a	t	g	t	g	t	t	a	a	a	t	g
KP771890	9	t	a	a	c	g	t	c	a	g	a	g	g	g	g	c	c	g	t	t	c	g	c	c	c	g	c	a	c	c	c	a	g	g	a	a	a	c	c	c	c	t	g	c	t	c	c	a	t	a	a	a	t	a	t	c	a	t	g	g	a	g	a	a	t	g	t	g	t	t	a	a	a	t	g
JN704710	9	t	a	a	c	g	t	c	a	g	a	g	g	g	g	c	c	g	t	t	c	g	c	c	c	g	c	a	c	c	c	a	g	g	a	a	a	c	c	c	c	t	g	c	t	c	c	a	t	a	a	a	t	a	t	t	a	t	g	g	a	g	a	a	t	g	t	g	t	t	a	a	a	t	g
KY037798	9	t	a	a	c	g	t	c	a	g	a	g	g	g	g	c	c	g	t	t	c	g	c	c	c	g	c	a	c	c	c	a	g	g	a	a	a	c	c	c	c	t	g	c	t	c	a	a	t	a	a	a	t	c	t	c	a	t	g	g	a	g	a	a	t	g	t	g	t	t	a	a	a	t	g
MH709312	9	t	a	a	c	g	t	c	a	g	a	g	g	g	g	c	c	g	t	t	c	g	c	c	c	g	c	a	c	c	c	a	g	g	a	a	a	c	c	c	c	t	g	c	t	c	c	a	t	a	a	a	t	c	t	c	a	t	g	g	a	g	a	a	t	g	t	g	t	t	a	a	a	t	g
MH709352	9	t	a	a	c	g	t	c	a	g	a	g	g	g	g	c	c	g	t	t	c	g	c	c	c	g	c	a	c	c	t	a	g	g	a	a	a	c	c	c	c	t	g	c	t	c	c	a	t	a	a	a	t	a	t	c	a	t	g	g	a	g	a	a	t	g	t	g	t	t	a	a	a	t	g
AB097933	Vac	t	a	a	c	g	t	c	a	g	a	g	g	g	g	c	c	a	t	t	c	g	c	c	c	g	c	a	c	c	c	a	g	g	a	a	a	c	c	c	c	t	g	c	t	t	c	g	t	a	a	a	t	a	t	t	a	t	g	g	a	g	a	a	t	g	t	g	t	t	a	a	a	t	g
JN704698	Vac	t	a	a	c	g	t	c	a	g	a	g	g	g	g	c	c	a	t	t	c	g	c	c	c	g	c	a	c	c	c	a	g	g	a	a	a	c	c	c	c	t	g	c	t	t	c	g	t	a	a	a	t	a	t	t	a	t	g	g	a	g	a	a	t	g	t	g	t	t	a	a	a	t	g
JN704697	Vac	t	a	a	c	g	t	c	a	g	a	g	g	g	g	c	c	a	t	t	c	g	c	c	c	g	c	a	c	c	c	a	g	g	a	a	a	c	c	c	c	t	g	c	t	t	c	g	t	a	a	a	t	a	t	t	a	t	g	g	a	g	a	a	t	g	t	g	t	t	a	a	a	t	g
DQ008355	Vac	t	a	a	c	g	t	c	a	g	a	g	g	g	g	c	c	a	t	t	c	g	c	c	c	g	c	a	c	c	c	a	g	g	a	a	a	c	c	c	c	t	g	c	t	t	c	g	t	a	a	a	t	a	t	t	a	t	g	g	a	g	a	a	t	g	t	g	t	t	a	a	a	t	g
JF306641	Vac	t	a	a	c	g	t	c	a	g	a	g	g	g	g	c	c	a	t	t	c	g	c	c	c	g	c	a	c	c	c	a	g	g	a	a	a	c	c	c	c	t	g	c	t	t	c	g	t	a	a	a	t	a	t	t	a	t	g	g	a	g	a	a	t	g	t	g	t	t	a	a	a	t	g
AB097932	Vac	t	a	a	c	g	t	c	a	g	a	g	g	g	g	c	c	a	t	t	c	g	c	c	c	g	c	a	c	c	c	a	g	g	a	a	a	c	c	c	c	t	g	c	t	t	c	g	t	a	a	a	t	a	t	t	a	t	g	g	a	g	a	a	t	g	t	g	t	t	a	a	a	t	g
DQ008354	Vac	t	a	a	c	g	t	c	a	g	a	g	g	g	g	c	c	a	t	t	c	g	c	c	c	g	c	a	c	c	c	a	g	g	a	a	a	c	c	c	c	t	g	c	t	t	c	g	t	a	a	a	t	a	t	t	a	t	g	g	a	g	a	a	t	g	t	g	t	t	a	a	a	t	g
KU926312	Vac	t	a	a	c	g	t	c	a	g	a	g	g	g	g	c	c	a	t	t	c	g	c	c	c	g	c	a	c	c	c	a	g	g	a	a	a	c	c	c	c	t	g	c	t	t	c	g	t	a	a	a	t	a	t	t	a	t	g	g	a	g	a	a	t	g	t	g	t	t	a	a	a	t	g
KU926313	Vac	t	a	a	c	g	t	c	a	g	a	g	g	g	g	c	c	a	t	t	c	g	c	c	c	g	c	a	c	c	c	a	g	g	a	a	a	c	c	c	c	t	g	c	t	t	c	g	t	a	a	a	t	a	t	t	a	t	g	g	a	g	a	a	t	g	t	g	t	t	a	a	a	t	g
KU926314	Vac	t	a	a	c	g	t	c	a	g	a	g	g	g	g	c	c	a	t	t	c	g	c	c	c	g	c	a	c	c	c	a	g	g	a	a	a	c	c	c	c	t	g	c	t	t	c	g	t	a	a	a	t	a	t	t	a	t	g	g	a	g	a	a	t	g	t	g	t	t	a	a	a	t	g
KF558371	Vac	t	a	a	c	g	t	c	a	g	a	g	g	g	g	c	c	a	t	t	c	g	c	c	c	g	c	a	c	c	c	a	g	g	a	a	a	c	c	c	c	t	g	c	t	t	c	g	t	a	a	a	t	a	t	t	a	t	g	g	a	g	a	a	t	g	t	g	t	t	a	a	a	t	g
KF558372	Vac	t	a	a	c	g	t	c	a	g	a	g	g	g	g	c	c	a	t	t	c	g	c	c	c	g	c	a	c	c	c	a	g	g	a	a	a	c	c	c	c	t	g	c	t	t	c	g	t	a	a	a	t	a	t	t	a	t	g	g	a	g	a	a	t	g	t	g	t	t	a	a	a	t	g
KF558373	Vac	t	a	a	c	g	t	c	a	g	a	g	g	g	g	c	c	a	t	t	c	g	c	c	c	g	c	a	c	c	c	a	g	g	a	a	a	c	c	c	c	t	g	c	t	t	c	g	t	a	a	a	t	a	t	t	a	t	g	g	a	g	a	a	t	g	t	g	t	t	a	a	a	t	g
KF558374	Vac	t	a	a	c	g	t	c	a	g	a	g	g	g	g	c	c	a	t	t	c	g	c	c	c	g	c	a	c	c	c	a	g	g	a	a	a	c	c	c	c	t	g	c	t	t	c	g	t	a	a	a	t	a	t	t	a	t	g	g	a	g	a	a	t	g	t	g	t	t	a	a	a	t	g
KF558375	Vac	t	a	a	c	g	t	c	a	g	a	g	g	g	g	c	c	a	t	t	c	g	c	c	c	g	c	a	c	c	c	a	g	g	a	a	a	c	c	c	c	t	g	c	t	t	c	g	t	a	a	a	t	a	t	t	a	t	g	g	a	g	a	a	t	g	t	g	t	t	a	a	a	t	g
KF558376	Vac	t	a	a	c	g	t	c	a	g	a	g	g	g	g	c	c	a	t	t	c	g	c	c	c	g	c	a	c	c	c	a	g	g	a	a	a	c	c	c	c	t	g	c	t	t	c	g	t	a	a	a	t	a	t	t	a	t	g	g	a	g	a	a	t	g	t	g	t	t	a	a	a	t	g
KF558377	Vac	t	a	a	c	g	t	c	a	g	a	g	g	g	g	c	c	a	t	t	c	g	c	c	c	g	c	a	c	c	c	a	g	g	a	a	a	c	c	c	c	t	g	c	t	t	c	g	t	a	a	a	t	a	t	t	a	t	g	g	a	g	a	a	t	g	t	g	t	t	a	a	a	t	g
KF558378	Vac	t	a	a	c	g	t	c	a	g	a	g	g	g	g	c	c	a	t	t	c	g	c	c	c	g	c	a	c	c	c	a	g	g	a	a	a	c	c	c	c	t	g	c	t	t	c	g	t	a	a	a	t	a	t	t	a	t	g	g	a	g	a	a	t	g	t	g	t	t	a	a	a	t	g
KF558379	Vac	t	a	a	c	g	t	c	a	g	a	g	g	g	g	c	c	a	t	t	c	g	c	c	c	g	c	a	c	c	c	a	g	g	a	a	a	c	c	c	c	t	g	c	t	t	c	g	t	a	a	a	t	a	t	t	a	t	g	g	a	g	a	a	t	g	t	g	t	t	a	a	a	t	g
KF558380	Vac	t	a	a	c	g	t	c	a	g	a	g	g	g	g	c	c	a	t	t	c	g	c	c	c	g	c	a	c	c	c	a	g	g	a	a	a	c	c	c	c	t	g	c	t	t	c	g	t	a	a	a	t	a	t	t	a	t	g	g	a	g	a	a	t	g	t	g	t	t	a	a	a	t	g
KF558381	Vac	t	a	a	c	g	t	c	a	g	a	g	g	g	g	c	c	a	t	t	c	g	c	c	c	g	c	a	c	c	c	a	g	g	a	a	a	c	c	c	c	t	g	c	t	t	c	g	t	a	a	a	t	a	t	t	a	t	g	g	a	g	a	a	t	g	t	g	t	t	a	a	a	t	g
KF558382	Vac	t	a	a	c	g	t	c	a	g	a	g	g	g	g	c	c	a	t	t	c	g	c	c	c	g	c	a	c	c	c	a	g	g	a	a	a	c	c	c	c	t	g	c	t	t	c	g	t	a	a	a	t	a	t	t	a	t	g	g	a	g	a	a	t	g	t	g	t	t	a	a	a	t	g
KF558383	Vac	t	a	a	c	g	t	c	a	g	a	g	g	g	g	c	c	a	t	t	c	g	c	c	c	g	c	a	c	c	c	a	g	g	a	a	a	c	c	c	c	t	g	c	t	t	c	g	t	a	a	a	t	a	t	t	a	t	g	g	a	g	a	a	t	g	t	g	t	t	a	a	a	t	g
KF558384	Vac	t	a	a	c	g	t	c	a	g	a	g	g	g	g	c	c	a	t	t	c	g	c	c	c	g	c	a	c	c	c	a	g	g	a	a	a	c	c	c	c	t	g	c	t	t	c	g	t	a	a	a	t	a	t	t	a	t	g	g	a	g	a	a	t	g	t	g	t	t	a	a	a	t	g
KF558385	Vac	t	a	a	c	g	t	c	a	g	a	g	g	g	g	c	c	a	t	t	c	g	c	c	c	g	c	a	c	c	c	a	g	g	a	a	a	c	c	c	c	t	g	c	t	t	c	g	t	a	a	a	t	a	t	t	a	t	g	g	a	g	a	a	t	g	t	g	t	t	a	a	a	t	g
KF558386	Vac	t	a	a	c	g	t	c	a	g	a	g	g	g	g	c	c	a	t	t	c	g	c	c	c	g	c	a	c	c	c	a	g	g	a	a	a	c	c	c	c	t	g	c	t	t	c	g	t	a	a	a	t	a	t	t	a	t	g	g	a	g	a	a	t	g	t	g	t	t	a	a	a	t	g
KF558387	Vac	t	a	a	c	a	t	c	a	g	a	g	g	g	g	c	c	a	t	t	c	g	c	c	c	g	c	a	c	c	c	a	g	g	a	a	a	c	c	c	c	t	g	c	t	t	c	g	t	a	a	a	t	a	t	t	a	t	g	g	a	g	a	a	t	g	t	g	t	t	a	a	a	t	g
KF558388	Vac	t	a	a	c	g	t	c	a	g	a	g	g	g	g	c	c	a	t	t	c	g	c	c	c	g	c	a	c	c	c	a	g	g	a	a	a	c	c	c	c	t	g	c	t	t	c	g	t	a	a	a	t	a	t	t	a	t	g	g	a	g	a	a	t	g	t	g	t	t	a	a	a	t	g
KF558389	Vac	t	a	a	c	g	t	c	a	g	a	g	g	g	g	c	c	a	t	t	c	g	c	c	c	g	c	a	c	c	c	a	g	g	a	a	a	c	c	c	c	t	g	c	t	t	c	g	t	a	a	a	t	a	t	t	a	t	g	g	a	g	a	a	t	g	t	g	t	t	a	a	a	t	g
KF558390	Vac	t	a	a	c	g	t	c	a	g	a	g	g	g	g	c	c	a	t	t	c	g	c	c	c	g	c	a	c	c	c	a	g	g	a	a	a	c	c	c	c	t	g	c	t	t	c	g	t	a	a	a	t	a	t	t	a	t	g	g	a	g	a	a	t	g	t	g	t	t	a	a	a	t	g
KF558391	Vac	t	a	a	c	g	t	c	a	g	a	g	g	g	g	c	c	a	t	t	c	g	c	c	c	g	c	a	c	c	c	a	g	g	a	a	a	c	c	c	c	t	g	c	t	t	c	g	t	a	a	a	t	a	t	t	a	t	g	g	a	g	a	a	t	g	t	g	t	t	a	a	a	t	g
KF811485	Vac	t	a	a	c	g	t	c	a	g	a	g	g	g	g	c	c	a	t	t	c	g	c	c	c	g	c	a	c	c	c	a	g	g	a	a	a	c	c	c	c	t	g	c	t	t	c	g	t	a	a	a	t	a	t	t	a	t	g	g	a	g	a	a	t	g	t	g	t	t	a	a	a	t	g
KF853225	Vac	t	a	a	c	g	t	c	a	g	a	g	g	g	g	c	c	a	t	t	c	g	c	c	c	g	c	a	c	c	c	a	g	g	a	a	a	c	c	c	c	t	g	c	t	t	c	g	t	a	a	a	t	a	t	t	a	t	g	g	a	g	a	a	t	g	t	g	t	t	a	a	a	t	g
KF853226	Vac	t	a	a	c	g	t	c	a	g	a	g	g	g	g	c	c	a	t	t	c	g	c	c	c	g	c	a	c	c	c	a	g	g	a	a	a	c	c	c	c	t	g	c	t	t	c	g	t	a	a	a	t	a	t	t	a	t	g	g	a	g	a	a	t	g	t	g	t	t	a	a	a	t	g
KF853227	Vac	t	a	a	c	g	t	c	a	g	a	g	g	g	g	c	c	a	t	t	c	g	c	c	c	g	c	a	c	c	c	a	g	g	a	a	a	c	c	c	c	t	g	c	t	t	c	g	t	a	a	a	t	a	t	t	a	t	g	g	a	g	a	a	t	g	t	g	t	t	a	a	a	t	g
KF853228	Vac	t	a	a	c	g	t	c	a	g	a	g	g	g	g	c	c	a	t	t	c	g	c	c	c	g	c	a	c	c	c	a	g	g	a	a	a	c	c	c	c	t	g	c	t	t	c	g	t	a	a	a	t	a	t	t	a	t	g	g	a	g	a	a	t	g	t	g	t	t	a	a	a	t	g
KF853229	Vac	t	a	a	c	g	t	c	a	g	a	g	g	g	g	c	c	a	t	t	c	g	c	c	c	g	c	a	c	c	c	a	g	g	a	a	a	c	c	c	c	t	g	c	t	t	c	g	t	a	a	a	t	a	t	t	a	t	g	a	a	g	a	a	t	g	t	g	t	t	a	a	a	t	g
KF853230	Vac	t	a	a	c	g	t	c	a	g	a	g	g	g	g	c	c	a	t	t	c	g	c	c	c	g	c	a	c	c	c	a	g	g	a	a	a	c	c	c	c	t	g	c	t	t	c	g	t	a	a	a	t	a	t	t	a	t	g	g	a	g	a	a	t	g	t	g	t	t	a	a	a	t	g
KF853231	Vac	t	a	a	c	g	t	c	a	g	a	g	g	g	g	c	c	a	t	t	c	g	c	c	c	g	c	a	c	c	c	a	g	g	a	a	a	c	c	c	c	t	g	c	t	t	c	g	t	a	a	a	t	a	t	t	a	t	g	g	a	g	a	a	t	g	t	g	t	t	a	a	a	t	g
KF853232	Vac	t	a	a	c	g	t	c	a	g	a	g	g	g	g	c	c	a	t	t	c	g	c	c	c	g	c	a	c	c	c	a	g	g	a	a	a	c	c	c	c	t	g	c	t	t	c	g	t	a	a	a	t	a	t	t	a	t	g	g	a	g	a	a	t	g	t	g	t	t	a	a	a	t	g
KF853233	Vac	t	a	a	c	g	t	c	a	g	a	g	g	g	g	c	c	a	t	t	c	g	c	c	c	g	c	a	c	c	c	a	g	g	a	g	a	c	c	c	c	t	g	c	t	t	c	g	t	a	a	a	t	a	t	t	a	t	g	g	a	g	a	a	t	g	t	g	t	t	a	a	a	t	g
KF853234	Vac	t	a	a	c	g	t	c	a	g	a	g	g	g	g	c	c	a	t	t	c	g	c	c	c	g	c	a	c	c	c	a	g	g	a	a	a	c	c	c	c	t	g	c	t	t	c	g	t	a	a	a	t	a	t	t	a	t	g	g	a	g	a	a	t	g	t	g	t	t	a	a	a	t	g
KF853235	Vac	t	a	a	c	g	t	c	a	g	a	g	g	g	g	c	c	a	t	t	c	g	c	c	c	g	c	a	c	c	c	a	g	g	a	g	a	c	c	c	c	t	g	c	t	t	c	g	t	a	a	a	t	a	t	t	a	t	g	g	a	g	a	a	t	g	t	g	t	t	a	a	a	t	g
MF898328	Vac	t	a	a	c	g	t	c	a	g	a	g	g	g	g	c	c	a	t	t	c	g	c	c	c	g	c	a	c	c	c	a	g	g	a	a	a	c	c	c	c	t	g	c	t	t	c	g	t	a	a	a	t	a	t	t	a	t	g	g	a	g	a	a	t	g	t	g	t	t	a	a	a	t	g
JN704709	VIII	t	a	a	c	g	t	c	a	g	a	g	g	g	g	c	a	g	t	t	c	g	c	c	c	g	c	a	c	c	c	a	g	g	a	a	a	c	c	c	c	t	g	c	t	c	c	a	t	a	a	a	t	a	t	c	a	t	g	g	a	g	a	a	t	g	t	g	t	t	a	a	a	t	g

Supplementary Table 3: 2880 SN

GB-ID	Clade markers																																																																									
																						4																										1																											
	Position in Dumas	78107	78144	78145	78220	78385	78526	78545	78663	78666	78698	78699	78705	78730	78756	78813	78834	78850	79009	79094	79137	79190	79210	79263	79300	79363	79393	79463	79471	79555	79609	79658	79770	79973	80080	80095	80244	80298	80306	80435	80436	80521	80578	80625	80692	80737	80832	80840	80899	80959	80966	80986	80998	81090	81095	81187	81268	81289	81322	81353	81419	81454	81464	81466	81472	81478	81712	81738	81762	81854	81886	81895	81940	81967	81973
AY548170	1	a	t	c	t	c	c	g	g	a	t	g	c	g	c	g	t	t	a	g	c	t	a	a	a	t	t	g	t	a	g	g	t	a	a	g	g	g	g	a	g	t	t	a	g	a	a	a	g	a	c	t	t	a	a	c	c	c	t	t	t	t	c	a	g	g	a	c	c	g	t	a	g	c	c
AY548171	1	a	t	c	t	c	c	g	g	a	c	g	c	g	c	g	t	t	a	g	c	t	a	a	a	t	t	g	t	a	g	g	t	a	a	g	g	g	g	a	g	t	t	a	g	a	a	a	g	a	c	t	t	a	a	c	c	c	t	t	t	t	c	a	g	g	a	c	c	g	t	a	g	c	c
DQ479953	1	a	t	c	t	c	c	g	g	a	c	g	c	g	c	g	t	t	a	g	c	t	a	a	a	t	t	g	t	a	g	g	t	a	a	g	g	g	g	a	g	t	t	a	g	a	a	a	g	a	c	t	t	a	a	c	c	c	t	t	t	t	c	a	g	g	a	c	c	g	t	a	g	c	c
DQ479954	1	a	t	c	t	c	c	g	g	a	c	g	c	g	c	g	t	t	a	g	c	t	a	a	a	t	t	g	t	a	g	g	t	a	a	g	g	g	g	a	g	t	t	a	g	a	a	a	g	a	c	t	t	a	a	c	c	c	t	t	t	t	c	a	g	g	a	c	c	g	t	a	g	c	c
DQ479958	1	a	t	c	t	c	c	g	g	a	c	g	c	g	c	g	t	t	a	g	g	t	a	a	a	t	t	g	t	a	g	g	t	a	a	g	g	g	g	a	g	t	t	a	g	a	a	a	g	a	c	t	t	a	a	c	c	c	t	t	t	t	c	a	g	g	a	c	c	g	t	a	g	c	c
DQ479959	1	a	t	c	t	c	c	g	g	a	c	g	c	g	c	g	t	t	a	g	g	t	a	a	a	t	t	g	t	a	g	g	t	a	a	g	g	g	g	a	g	t	t	a	g	a	a	a	g	a	c	t	t	a	a	c	c	c	t	t	t	t	c	a	g	g	a	c	c	g	t	a	g	c	c
DQ479961	1	a	t	c	t	c	c	g	g	a	c	g	c	g	c	g	t	t	a	g	c	t	a	a	a	t	t	g	t	a	g	g	t	a	a	g	g	g	g	a	g	t	t	a	g	a	a	a	g	a	c	t	t	a	a	c	c	c	t	t	t	t	c	a	g	g	a	c	c	g	t	a	g	c	c
DQ479962	1	a	t	c	t	c	c	g	g	a	c	g	c	g	c	g	t	t	a	g	c	t	a	a	a	t	t	g	t	a	g	g	t	a	a	g	g	g	g	a	g	t	t	a	g	a	a	a	g	a	c	t	t	a	a	c	c	c	t	t	t	t	c	a	g	g	a	c	c	g	t	a	g	c	c
DQ479963	1	a	t	c	t	c	c	g	g	a	c	g	c	g	c	g	t	t	a	g	c	t	a	a	a	t	t	g	t	a	g	g	t	a	a	g	g	g	g	a	g	t	t	a	g	a	a	a	g	a	c	c	t	a	a	c	c	c	t	t	t	t	c	a	g	g	a	c	c	g	t	a	g	c	c
DQ674250	1	a	t	c	t	c	c	g	g	a	c	g	c	g	c	g	t	t	a	g	c	t	a	a	a	t	t	g	t	a	g	g	t	a	a	g	t	g	g	a	g	t	t	a	g	a	a	g	g	a	c	t	t	g	a	t	c	c	t	t	t	t	c	a	g	g	a	c	c	g	t	a	g	c	c
KX262864	1	a	t	c	t	c	c	g	g	a	c	g	c	g	c	g	t	t	a	g	c	t	a	a	a	t	t	g	t	a	g	g	t	a	a	g	g	g	g	a	g	t	t	a	g	a	a	a	g	a	c	t	t	a	a	c	c	c	t	t	t	t	c	a	g	g	a	c	c	g	t	a	g	c	c
KX262865	1	a	t	c	t	c	c	g	g	a	c	g	c	g	c	g	t	t	a	g	c	t	a	a	a	t	t	g	t	a	g	g	t	a	a	g	g	g	g	a	g	t	t	a	g	g	a	a	g	a	c	t	t	a	a	c	c	c	t	t	t	t	c	a	g	g	a	c	c	g	t	a	g	c	c
KX262866	1	a	t	c	t	c	c	g	g	a	c	g	c	g	c	g	t	t	a	g	c	t	a	a	a	t	t	g	t	a	g	g	t	a	a	g	g	g	g	a	g	t	t	a	g	a	a	a	g	a	c	t	t	a	a	c	c	c	t	t	t	t	c	a	g	g	a	c	c	g	t	a	g	c	c
EU154348	1	a	c	c	t	c	c	g	g	a	c	g	c	g	c	g	t	t	a	g	c	t	a	a	a	t	t	g	t	a	g	g	t	a	a	g	g	g	g	a	g	t	t	a	g	a	a	a	g	a	c	t	t	a	a	c	c	c	t	t	t	t	c	a	g	g	a	c	c	g	t	a	g	c	c
JN704690	1	a	t	c	t	c	c	g	g	a	c	g	c	g	c	g	t	t	a	g	c	t	a	a	a	t	t	g	t	a	g	g	t	a	a	g	g	g	g	a	g	t	t	a	g	a	a	a	g	a	c	t	t	a	a	c	c	c	t	t	t	t	c	a	g	g	a	c	c	g	t	a	g	c	c
JN704691	1	a	t	c	t	c	c	g	g	a	c	g	c	g	c	g	t	t	a	g	c	t	a	a	a	t	t	g	t	a	g	g	t	a	a	g	g	g	g	a	g	t	t	a	g	a	a	a	g	a	c	t	t	a	a	c	c	c	t	t	t	t	c	a	g	g	a	c	c	g	t	a	g	c	c
JN704692	1	a	t	c	t	c	c	g	g	a	c	g	c	g	c	g	t	t	a	g	c	t	a	a	a	t	t	g	t	a	g	g	t	a	a	g	g	g	g	a	g	t	t	a	g	a	a	a	g	a	c	t	t	a	a	c	c	c	t	t	t	t	c	a	g	g	a	c	c	g	t	a	g	c	c
JN704693	1	a	t	c	t	c	c	g	g	a	c	g	c	g	c	g	t	t	a	g	c	t	a	a	a	t	t	g	t	a	g	g	t	a	a	g	g	g	g	a	g	t	t	a	g	a	a	a	g	a	c	t	t	a	a	c	c	c	t	t	t	t	c	a	g	g	a	c	c	g	t	a	g	c	c
JN704694	1	a	t	c	t	c	c	g	g	a	c	g	c	g	c	g	t	t	a	g	c	t	a	a	a	t	t	g	t	a	g	g	t	a	a	g	g	g	g	a	g	t	t	a	a	a	a	a	g	a	c	t	t	a	a	c	c	c	t	t	t	t	c	a	g	g	a	c	c	g	t	a	g	c	c
JN704695	1	a	t	c	t	c	c	g	g	a	c	g	c	g	c	g	t	t	a	g	c	t	a	a	a	t	t	g	t	a	g	g	t	a	a	g	g	g	g	a	g	t	t	a	g	a	a	a	g	a	c	t	t	a	a	c	c	c	t	t	t	t	c	a	g	g	a	c	c	g	t	a	g	c	c
JN704696	1	a	t	c	t	c	c	g	g	a	c	g	c	g	c	g	t	t	a	g	c	t	a	a	a	t	t	g	t	a	g	g	t	a	a	g	g	g	g	a	g	t	t	a	g	a	a	a	g	a	c	t	t	a	a	c	c	c	t	t	t	t	c	a	g	g	a	c	c	g	t	a	g	c	c
NC001348	1	a	t	c	t	c	c	g	g	a	c	g	c	g	c	g	t	t	a	g	c	t	a	a	a	t	t	g	t	a	g	g	t	a	a	g	g	g	g	a	g	t	t	a	g	a	a	a	g	a	c	t	t	a	a	c	c	c	t	t	t	t	c	a	g	g	a	c	c	g	t	a	g	c	c
KP771892	1	a	t	c	t	c	c	g	g	a	c	g	c	g	c	g	t	t	a	g	c	t	a	a	a	t	t	g	t	a	g	g	t	a	a	g	g	g	g	a	g	t	t	a	g	a	g	a	g	a	c	t	t	a	a	c	c	c	t	t	t	t	c	a	g	g	a	c	c	g	t	a	g	c	c
KP771894	1	a	t	c	t	c	c	g	g	a	c	g	c	g	c	g	t	t	a	g	c	t	a	a	a	t	t	g	t	a	g	g	t	a	a	g	g	g	g	a	g	t	t	a	g	a	a	a	g	a	c	t	t	a	a	c	c	c	t	t	t	t	c	a	g	g	a	c	c	g	t	a	g	c	c
KP771900	1	a	t	c	t	c	c	g	g	a	c	g	c	g	c	g	t	t	a	g	c	t	a	a	a	t	t	g	t	a	g	g	t	a	a	g	g	g	g	a	g	t	t	a	g	a	a	a	g	a	c	t	t	a	a	c	c	c	t	t	t	t	c	a	g	g	a	c	c	g	t	a	g	c	c
KP771901	1	a	t	c	t	c	c	g	g	a	c	g	c	g	c	g	t	t	a	g	c	t	a	a	a	t	t	g	t	a	g	g	t	a	a	g	g	g	g	a	g	t	t	a	g	a	a	a	g	a	c	t	t	a	a	c	c	c	t	t	t	t	c	a	g	g	a	c	c	g	t	a	g	c	c
KP771902	1	a	t	c	t	c	c	g	g	a	c	g	c	g	c	g	t	t	a	g	c	t	a	a	a	t	t	g	t	a	g	g	t	a	a	g	g	g	g	a	g	t	t	a	g	a	a	a	g	a	c	t	t	a	a	c	c	c	t	t	t	t	c	a	g	g	a	c	c	g	t	a	g	c	c
KP771908	1	a	t	c	t	c	c	g	g	a	c	g	c	g	c	g	t	t	a	g	c	t	a	a	a	t	t	g	t	a	g	g	t	a	a	g	g	g	g	a	g	t	t	a	g	a	a	a	g	a	c	t	t	a	a	c	c	c	t	t	t	t	t	a	g	g	a	c	c	g	t	a	g	c	c
KP771912	1	a	t	c	t	c	c	g	g	a	c	g	c	g	c	g	t	t	a	g	c	t	a	a	a	t	t	g	t	a	g	g	t	a	a	g	g	g	g	a	g	t	t	a	g	a	a	a	g	a	c	t	t	a	a	c	c	c	t	t	t	t	c	a	g	g	a	c	c	g	t	a	g	c	c
KP771913	1	a	t	c	t	c	c	g	g	a	c	g	c	g	c	g	t	t	a	g	c	t	a	a	a	t	t	g	t	a	g	g	t	a	a	g	g	g	g	a	g	t	t	a	g	a	a	a	g	a	c	t	t	a	a	c	c	c	t	t	t	t	c	a	g	g	a	c	c	g	t	a	g	c	c
KP771917	1	a	t	c	t	c	c	g	g	a	c	g	c	g	c	g	t	t	a	g	c	t	a	a	a	t	t	g	t	a	g	g	t	a	a	g	g	g	g	a	g	t	t	a	g	a	a	a	g	a	c	t	t	a	a	c	c	c	t	t	t	t	c	a	g	g	a	c	c	g	t	a	g	c	c
KP771918	1	a	t	c	t	c	c	g	g	a	c	g	c	g	c	g	t	t	a	g	c	t	a	a	a	t	t	g	t	a	g	g	t	a	a	g	g	g	g	a	g	t	t	a	g	a	a	a	g	a	c	t	t	a	a	c	c	c	t	t	t	t	c	a	g	g	a	c	c	g	t	a	g	c	c
KP771920	1	a	t	c	c	c	c	g	g	a	c	g	c	g	c	g	t	t	a	g	c	t	a	a	a	t	t	g	t	a	g	g	t	a	a	g	g	g	g	a	g	t	t	a	g	a	a	a	g	a	c	t	t	a	a	c	c	c	t	t	t	t	c	a	g	g	a	c	c	g	t	a	g	c	c
MH709361	1	a	t	c	t	c	c	g	g	a	c	g	c	g	c	g	t	t	a	g	c	t	a	a	a	t	t	g	t	a	g	g	t	a	a	g	g	g	g	a	g	t	t	a	g	a	a	a	g	a	c	t	t	a	a	c	c	c	t	t	t	t	c	a	g	g	a	c	c	g	t	a	g	c	c
KU926318	2	a	t	c	t	c	c	g	g	a	c	g	c	g	c	g	t	t	a	g	c	t	a	a	a	t	t	g	t	a	g	g	t	a	a	g	t	g	g	a	a	t	t	a	g	a	a	g	g	a	c	t	t	a	a	t	c	c	t	t	t	t	c	a	g	g	a	c	c	g	t	a	g	c	c
KU926319	2	a	t	c	t	c	c	g	g	a	c	g	c	g	c	g	t	t	a	g	c	t	a	a	a	t	t	g	t	a	g	g	t	a	a	g	t	g	g	a	a	t	t	a	g	a	a	g	g	a	c	t	c	a	a	t	c	c	t	t	t	t	c	a	g	g	a	c	c	g	t	a	g	c	c
KU926320	2	a	t	c	t	c	c	g	g	a	c	g	c	g	c	g	t	t	a	g	c	t	a	a	a	t	t	g	t	a	g	g	t	a	a	g	t	g	g	a	g	t	t	a	g	a	a	g	g	a	c	t	t	a	a	t	c	c	t	t	t	t	c	a	g	g	a	c	c	g	t	a	g	c	c
KU926321	2	a	t	c	t	c	c	g	g	a	c	g	c	g	c	g	t	t	a	g	c	t	a	a	a	t	t	g	t	a	g	g	t	a	a	g	t	g	g	a	g	t	t	a	g	a	a	g	g	a	c	t	t	a	a	t	c	c	t	t	t	t	c	a	g	g	a	c	c	g	t	a	g	c	c
KU926322	2	a	t	c	t	c	c	g	g	a	c	g	c	g	c	g	t	t	a	g	c	t	a	a	a	t	t	g	t	a	g	g	t	a	a	g	g	g	g	a	g	t	t	a	g	a	a	a	g	a	c	t	t	a	a	c	c	c	t	t	t	t	c	a	g	g	a	c	c	g	t	a	g	c	c
KC847290	2	a	t	c	t	c	c	g	g	a	c	g	c	g	c	g	t	t	a	g	c	t	a	a	a	t	t	g	t	a	g	g	t	a	a	g	t	g	g	a	g	t	t	a	g	a	a	g	g	a	c	t	t	a	a	t	c	c	t	t	t	t	c	a	g	g	a	c	c	g	t	a	g	c	c
KJ767491	2	a	t	c	t	c	c	g	g	a	c	g	c	g	c	g	t	t	a	g	c	t	a	a	a	t	t	g	t	a	g	g	t	a	a	g	t	g	g	a	g	t	t	a	g	a	a	g	g	a	c	t	t	a	a	t	c	c	t	t	t	t	c	a	g	g	a	c	c	g	t	a	g	c	c
KJ767492	2	a	t	c	t	c	c	g	g	a	c	g	c	g	c	g	t	t	a	g	c	t	a	a	a	t	t	g	t	a	g	g	t	a	a	g	t	g	g	a	a	t	t	a	g	a	a	g	g	a	c	t	t	a	a	t	c	c	t	t	t	t	c	a	g	g	a	c	c	g	t	a	g	c	c
KJ808816	2	a	t	c	t	c	c	g	g	a	c	g	c	g	c	g	t	t	a	g	c	t	a	a	a	t	t	g	t	a	g	g	t	a	a	g	t	g	g	a	g	t	t	a	g	a	a	g	g	a	c	t	t	a	a	t	c	c	t	t	t	t	c	a	g	g	a	c	c	g	t	a	g	c	c
KP771925	2	a	t	c	t	c	c	g	g	a	c	g	c	g	c	g	t	t	a	g	c	t	a	a	a	t	t	g	t	a	g	g	t	a	a	g	t	g	g	a	g	t	t	a	g	a	a	g	g	a	c	t	t	a	a	t	c	c	t	t	t	t	c	a	g	g	a	c	c	g	t	a	g	c	c
KU926315	2	a	t	c	t	c	c	g	g	a	c	g	c	g	c	g	t	t	a	g	c	t	a	a	a	t	t	g	t	a	g	g	t	a	a	g	t	g	g	a	g	t	t	a	g	a	a	g	g	a	c	t	t	a	a	t	c	c	t	t	t	t	c	a	g	g	a	c	c	g	t	a	g	c	c
KU926316	2	a	t	c	t	c	c	g	g	a	c	g	c	g	c	g	t	t	a	g	c	t	a	a	a	t	t	g	t	a	g	g	t	a	a	g	t	g	g	a	g	t	t	a	g	a	a	g	g	a	c	t	t	a	a	t	c	c	t	t	t	t	c	a	g	g	a	c	c	g	t	a	g	c	c
KU926317	2	a	t	c	t	c	c	g	g	a	c	g	c	g	c	g	t	t	a	g	c	t	a	a	a	t	t	g	t	a	g	g	t	a	a	g	t	g	g	a	g	t	t	a	g	a	a	g	g	a	c	t	t	a	a	t	c	c	t	t	t	t	c	a	g	g	a	c	c	g	t	a	g	c	c
KY037796	2	a	t	c	t	c	c	g	g	a	c	g	c	g	c	g	t	t	a	g	c	t	a	a	a	t	t	g	t	a	g	g	a	a	a	g	t	g	g	a	g	t	t	a	g	a	a	g	g	a	c	t	t	a	a	t	c	t	t	t	t	t	c	a	g	g	a	c	c	g	t	a	g	c	c
MF004348	2	a	t	c	t	c	c	g	g	a	c	g	c	g	c	g	t	t	a	g	c	t	a	a	a	t	t	g	t	a	g	g	t	a	a	g	t	g	g	a	a	t	t	a	g	a	a	g	g	a	c	t	t	a	a	t	c	c	t	t	t	t	c	a	g	g	a	c	c	g	t	a	g	c	c
MH709309	2	a	t	c	t	c	c	g	g	a	c	g	c	g	c	g	t	t	a	g	c	t	a	a	a	t	t	g	t	a	g	g	t	a	a	g	t	g	g	a	g	t	t	a	g	a	a	g	g	a	c	t	t	a	a	t	c	c	t	t	t	t	c	a	g	g	a	c	c	g	t	a	g	c	c
MH709310	2	a	t	c	t	c	c	g	g	a	c	g	c	g	c	g	t	t	a	g	c	t	a	a	a	t	t	g	t	a	g	g	t	a	a	g	t	g	g	a	g	t	t	a	g	a	a	g	g	a	c	t	t	a	a	t	c	c	t	t	t	t	c	a	g	g	a	c	c	g	t	a	g	c	c
MH709311	2	a	t	c	t	c	c	g	g	a	c	g	c	g	c	g	t	t	a	g	c	t	c	a	a	t	t	g	t	a	g	g	t	a	a	g	t	g	g	a	g	t	t	a	g	a	a	g	g	a	c	t	t	a	a	t	c	c	t	t	t	t	c	g	g	g	a	c	c	g	t	a	g	c	c
MH709313	2	a	t	c	t	c	c	g	g	a	c	g	c	g	c	g	t	t	a	g	c	t	a	a	a	t	t	g	t	a	g	g	t	a	a	g	t	g	g	a	g	t	t	a	g	a	a	g	g	a	c	t	t	a	a	t	c	c	t	t	t	t	c	a	g	g	a	c	c	g	t	a	g	c	c
MH709314	2	a	t	c	t	c	c	g	g	a	c	g	c	g	c	g	t	t	a	g	c	t	a	a	a	t	t	g	t	a	g	g	t	a	a	g	t	g	g	a	g	t	t	a	g	a	a	g	g	a	c	t	t	a	a	t	c	c	t	t	t	t	c	a	g	g	a	c	c	g	t	a	g	c	c
MH709315	2	a	t	c	t	c	c	g	g	a	c	g	c	g	c	g	t	t	a	g	c	t	a	a	a	t	t	g	t	a	g	g	t	a	a	g	t	g	g	a	g	t	t	a	g	a	a	g	g	a	c	t	t	a	a	t	c	c	t	t	t	t	c	a	g	g	a	c	c	g	t	a	g	c	c
MH709319	2	a	t	c	t	c	c	g	g	a	c	g	c	g	c	g	t	t	a	g	c	t	a	a	a	t	t	g	t	a	g	g	t	a	a	g	t	g	g	a	g	t	t	a	g	a	a	g	a	a	c	t	t	a	a	t	c	c	t	t	t	t	c	a	g	g	a	c	c	g	t	a	g	c	c
MH709320	2	a	t	c	t	c	c	g	g	a	c	g	c	g	c	g	t	t	a	g	c	t	a	a	a	t	t	g	t	a	g	g	t	a	a	g	t	g	g	a	g	t	t	a	g	a	a	g	g	a	c	t	t	a	a	t	c	c	t	t	t	t	c	a	g	g	a	c	c	g	t	a	g	c	c
MH709329	2	a	t	c	t	c	c	g	g	a	c	g	c	g	c	g	t	t	a	g	c	t	a	a	a	t	t	g	t	a	g	g	t	a	a	g	t	g	g	a	g	t	t	a	g	a	a	g	g	a	c	t	t	a	a	t	c	c	t	t	t	t	c	a	g	g	a	c	c	g	t	a	g	c	c
MH709331	2	a	t	c	t	c	c	g	g	a	c	g	c	g	c	g	t	t	a	g	c	t	a	a	a	t	t	g	t	a	g	g	t	a	a	g	t	g	g	a	g	t	t	a	g	a	a	g	g	a	c	t	t	a	a	t	c	c	t	t	t	t	c	a	g	g	a	c	c	g	t	a	g	c	c
MH709333	2	a	t	c	t	c	c	g	g	a	c	g	c	g	c	g	t	t	a	g	c	t	a	a	a	t	t	g	t	a	g	g	t	a	a	g	t	g	g	a	g	t	t	a	g	a	a	g	g	a	c	t	t	a	a	t	c	c	t	t	t	t	c	a	g	g	a	c	c	g	t	a	g	c	c
MH709337	2	a	t	c	t	c	c	g	g	a	c	g	c	g	c	g	t	t	a	g	c	t	a	a	a	t	t	g	t	a	g	g	t	a	a	g	t	g	g	a	g	t	t	a	g	a	a	g	g	g	c	t	t	a	a	t	c	c	t	t	t	t	c	a	g	g	a	c	t	g	t	a	g	c	c
MH709339	2	a	t	c	t	c	c	g	g	a	c	g	c	g	c	g	t	t	a	g	c	t	a	a	a	t	t	g	t	a	g	g	t	a	a	g	t	g	g	a	g	t	t	a	g	a	a	g	g	a	c	t	t	a	c	t	c	c	t	t	t	t	c	a	g	g	a	c	c	g	t	a	g	c	c
MH709344	2	a	t	c	t	c	c	g	g	a	c	g	c	g	c	g	t	t	a	g	c	t	a	a	a	t	t	g	t	a	g	g	t	a	a	g	t	g	g	a	g	t	g	a	g	a	a	g	g	a	c	t	t	a	a	t	c	c	t	t	t	t	c	a	g	g	a	c	c	g	t	a	g	c	c
MH709348	2	a	t	c	t	c	c	g	g	a	c	g	c	g	c	g	t	t	a	g	c	t	a	a	a	t	t	g	t	a	g	g	t	a	a	g	t	g	g	a	g	t	t	a	g	a	a	g	g	a	c	t	t	a	a	t	c	c	t	t	t	t	c	a	g	g	a	c	c	g	t	a	g	c	c
MH709355	2	a	t	c	t	c	c	g	g	a	c	g	c	g	c	g	t	t	a	g	c	t	a	a	g	t	t	g	t	a	g	g	t	a	a	g	t	g	g	c	g	t	t	a	g	a	a	g	g	a	c	t	t	a	a	t	c	c	t	t	t	t	c	a	g	g	a	c	c	g	t	a	g	c	c
MH709359	2	a	t	c	t	c	c	g	g	a	c	g	c	g	c	g	t	t	a	g	c	t	a	a	a	t	t	g	t	a	g	g	t	a	a	g	t	g	g	a	g	t	t	a	g	a	a	g	g	a	c	t	t	a	a	t	c	c	t	t	t	t	c	a	g	g	a	c	c	g	t	a	g	c	c
MH709363	2	a	c	c	t	c	c	g	g	a	c	g	c	g	c	g	t	t	a	g	c	t	a	a	a	t	t	g	t	a	g	g	t	a	a	g	t	g	g	a	g	t	t	a	g	a	a	g	g	a	c	t	t	a	a	t	c	c	t	t	t	t	c	a	g	g	a	c	c	g	t	a	g	c	c
MH709366	2	a	t	c	t	c	c	g	g	a	c	g	c	g	c	g	t	t	a	g	c	t	a	a	a	t	t	g	t	a	g	g	t	a	a	g	t	g	g	a	g	t	t	a	g	a	a	g	g	a	c	t	t	a	a	t	c	c	t	t	t	t	c	a	g	g	a	c	c	g	t	a	g	c	c
MH709376	2	a	t	c	t	c	c	g	g	a	c	g	c	g	c	g	t	t	a	g	c	t	a	a	a	t	t	g	t	a	g	g	t	a	a	g	t	g	g	a	g	t	t	a	g	a	a	g	g	a	c	t	t	a	a	t	c	c	t	t	t	t	c	a	g	g	a	c	c	g	t	a	g	c	c
MH709377	2	a	t	c	t	c	c	g	g	a	c	g	c	g	c	g	t	t	a	g	c	t	a	a	a	t	t	g	t	a	g	g	t	a	a	g	t	g	g	a	g	t	t	a	g	a	a	g	g	a	c	t	t	a	a	t	c	c	t	t	t	t	c	a	g	g	a	c	c	g	t	a	g	c	c
**MW316406**	2	a	t	c	t	c	c	g	g	a	c	g	c	g	c	g	t	t	a	g	c	t	a	a	a	t	t	g	t	a	g	g	t	a	a	g	t	g	g	a	g	t	t	a	g	a	a	g	g	a	c	t	t	a	a	t	c	c	t	t	t	t	c	a	g	g	a	c	c	g	t	a	g	c	c
AJ871403	3	a	t	c	t	t	c	a	g	a	c	g	c	g	c	g	t	t	a	g	c	t	a	a	a	t	c	g	t	a	g	g	t	a	a	g	t	g	t	a	g	t	t	a	g	a	a	g	g	a	c	t	t	a	a	c	c	c	t	t	t	t	c	a	g	g	a	c	c	a	t	a	g	c	c
DQ479955	3	a	t	c	t	t	c	a	g	a	c	g	c	g	c	g	t	t	a	g	c	t	a	a	a	t	t	g	t	a	g	g	t	a	a	g	t	g	t	a	g	t	t	a	g	a	a	g	g	a	c	t	t	a	a	c	c	c	t	t	t	t	c	a	g	g	a	c	c	a	t	a	g	c	c
DQ479956	3	a	t	c	t	t	c	a	g	a	c	g	c	g	c	g	t	t	a	g	c	t	c	a	a	t	t	g	t	a	g	g	t	a	a	g	t	g	t	a	g	t	t	a	g	a	a	g	g	a	c	t	t	a	a	c	c	c	t	t	t	t	c	a	g	g	a	c	c	a	t	a	g	c	c
DQ479957	3	a	t	c	t	t	c	a	g	a	c	g	c	g	c	g	t	t	a	g	c	t	c	a	a	t	t	g	t	a	g	g	t	a	a	g	t	g	t	a	g	t	t	a	g	a	a	g	g	a	c	t	t	a	a	c	c	c	t	t	t	t	c	a	g	g	a	c	c	a	t	a	g	c	c
JN704699	3	a	t	c	t	t	c	a	g	a	c	g	c	g	c	g	t	t	a	g	c	t	a	a	a	t	t	g	t	a	g	g	t	a	a	g	t	g	t	a	g	t	t	a	g	a	a	g	g	a	c	t	t	a	a	c	c	c	t	t	t	t	c	a	g	g	a	c	c	a	t	a	g	c	c
JN704700	3	a	t	c	t	t	c	a	g	a	c	g	c	a	c	g	t	t	a	g	c	t	a	a	a	t	t	g	t	a	g	g	t	a	a	g	t	g	t	a	g	t	t	a	g	a	a	g	g	a	c	t	t	a	a	c	c	c	t	t	t	t	c	a	g	g	a	c	c	a	t	a	g	c	c
JN704701	3	a	t	c	t	t	c	a	g	a	c	g	c	g	c	g	t	t	a	g	c	t	a	a	a	t	t	g	t	a	g	g	t	a	a	g	t	g	t	a	g	t	t	a	g	a	a	g	g	a	c	t	t	a	a	c	c	c	t	t	t	t	c	a	g	g	a	c	c	a	t	a	g	c	c
JN704702	3	a	t	c	t	t	c	a	g	a	c	g	c	g	c	g	t	t	a	t	c	t	a	a	a	t	t	g	t	a	g	g	t	a	a	g	t	g	t	a	g	t	t	a	g	a	a	g	g	a	c	t	t	a	a	c	c	c	t	t	t	t	c	a	g	g	a	c	c	a	t	a	g	c	c
JN704703	3	a	t	c	t	t	c	a	g	a	c	g	c	g	c	g	t	t	a	t	c	t	a	a	a	t	t	g	t	a	g	g	t	a	a	g	t	g	t	a	g	t	t	a	g	a	a	g	g	a	c	t	t	a	a	c	c	c	t	t	g	t	c	a	g	g	a	c	c	a	t	a	g	c	c
KP771891	3	a	t	c	t	t	c	a	g	a	c	g	c	g	c	g	t	t	a	g	c	t	a	a	a	t	t	g	t	a	g	g	t	a	a	g	t	g	t	a	g	t	t	a	g	a	a	g	g	a	c	t	t	a	a	c	c	c	t	t	t	t	c	a	g	g	a	c	c	a	t	a	g	c	c
KP771893	3	a	t	c	t	t	c	a	g	a	c	g	c	g	c	g	t	t	a	g	c	t	a	a	a	t	t	g	t	a	g	g	t	a	a	g	t	g	t	a	g	t	t	a	g	a	a	g	g	a	c	t	t	a	a	c	c	c	t	t	t	t	c	a	g	g	a	c	c	a	t	a	g	c	c
KP771895	3	a	t	c	t	t	c	a	g	a	c	g	c	g	c	g	t	t	a	g	c	t	a	a	a	t	t	g	t	a	g	g	t	a	a	g	t	g	t	a	g	t	t	a	g	a	a	g	g	a	c	t	t	a	a	c	c	c	t	t	t	t	c	a	g	g	a	c	c	a	t	a	g	c	c
KP771896	3	a	t	c	t	t	c	a	g	a	c	g	c	g	c	g	t	t	a	g	c	t	a	a	a	t	t	g	t	a	g	g	t	a	a	g	t	g	t	a	g	t	t	a	g	a	a	g	g	a	c	t	t	a	a	c	c	c	t	t	t	t	c	a	g	g	a	a	c	a	t	a	g	c	c
KP771897	3	a	t	c	t	t	c	a	g	a	c	g	c	g	c	g	t	t	a	g	c	t	a	a	a	t	t	g	t	a	g	g	t	a	a	g	t	g	t	a	g	t	t	a	g	a	a	g	g	a	c	t	t	a	a	c	c	c	t	t	t	t	c	a	g	g	a	c	c	a	t	a	g	c	c
KP771899	3	a	t	c	t	t	c	a	g	a	c	g	c	g	c	g	t	t	a	g	c	t	a	a	a	t	t	g	t	g	g	g	t	a	a	g	t	g	t	a	g	t	t	a	g	a	a	g	g	a	c	t	t	a	a	c	c	c	t	t	t	t	c	a	g	g	a	c	c	a	t	a	g	c	c
KP771903	3	a	t	c	t	t	c	a	g	a	c	g	c	g	c	g	t	t	a	g	c	t	a	a	a	t	t	g	t	a	g	g	t	a	a	g	t	g	t	a	g	t	t	a	g	a	a	g	g	a	c	t	t	a	a	c	c	c	t	t	t	t	c	a	g	g	a	c	c	a	t	a	g	c	c
KP771907	3	a	t	c	t	t	c	a	g	a	c	g	c	g	c	g	t	t	a	g	c	t	a	a	a	t	t	g	t	a	g	g	t	a	a	g	t	g	t	a	g	t	t	a	g	a	a	g	g	a	c	t	t	a	a	c	c	c	t	t	t	t	c	a	g	g	a	c	c	a	t	a	g	c	c
KP771910	3	a	t	c	t	t	c	a	g	a	c	g	c	g	c	g	t	t	a	g	c	t	a	a	a	t	t	g	t	a	g	g	t	a	a	g	t	g	t	a	g	t	t	a	g	a	a	g	g	a	c	t	t	a	a	c	c	c	t	t	t	t	c	a	g	g	a	c	c	a	t	a	g	c	c
KP771915	3	a	t	c	t	t	c	a	g	a	c	g	c	g	c	g	t	t	a	g	c	t	a	a	a	t	t	g	t	a	g	g	t	a	a	g	t	g	t	a	g	t	t	a	g	a	a	g	g	a	c	t	t	a	a	c	c	c	t	t	t	t	c	a	g	g	a	c	c	a	t	a	g	c	c
KP771916	3	a	t	c	t	t	c	a	g	a	c	g	c	g	c	g	t	t	a	g	c	t	c	a	a	t	t	g	t	a	g	g	t	a	a	g	t	g	t	a	g	t	t	a	g	a	a	g	g	a	c	t	t	a	a	c	c	c	t	t	t	t	c	a	g	g	a	c	c	a	t	a	g	c	c
KP771921	3	a	t	c	t	t	c	a	g	a	c	g	c	g	t	g	t	t	a	g	c	t	a	a	a	t	t	g	t	a	g	g	t	a	a	g	t	g	t	a	g	t	t	a	g	a	a	g	g	a	c	t	t	a	a	c	c	c	t	t	t	t	c	a	g	g	a	c	c	a	t	a	g	c	c
KP771923	3	a	t	c	t	t	c	a	g	a	c	g	c	g	c	g	t	t	a	g	c	t	a	a	a	t	t	g	t	a	g	g	t	a	a	g	t	g	t	a	g	t	t	a	g	a	a	g	g	a	c	t	t	a	a	c	c	c	t	t	t	t	c	a	g	g	a	c	c	a	t	a	g	c	c
KP771889	3	a	t	c	t	c	c	a	g	a	c	g	c	g	c	g	t	t	a	g	c	t	a	a	a	t	t	g	t	a	g	g	t	a	a	g	g	g	g	a	g	t	t	a	g	a	a	g	g	a	c	t	t	a	a	c	c	c	t	t	t	t	c	a	g	g	a	c	c	a	t	a	g	c	c
KU926311	3	a	t	c	t	t	c	a	g	a	c	g	c	g	c	g	t	t	a	g	c	t	c	a	a	t	t	g	t	a	g	g	t	a	a	g	t	g	t	a	g	t	t	a	g	a	a	g	g	a	c	t	t	a	a	c	c	c	t	t	t	t	c	a	g	g	a	c	c	a	t	a	g	c	c
JQ911599	3	a	t	c	t	t	c	a	g	a	c	g	c	g	c	g	t	t	a	t	c	t	a	a	a	t	t	g	t	a	g	g	t	a	a	g	t	g	t	a	g	t	t	a	g	a	a	g	g	a	c	t	t	a	a	c	c	c	t	t	t	t	c	a	g	g	a	c	c	a	t	a	g	c	c
MH709317	3	a	t	c	t	t	c	a	g	a	c	a	c	g	c	g	t	t	a	g	c	t	a	a	a	t	t	g	t	a	g	g	t	a	a	g	t	g	t	a	g	t	t	a	g	a	a	g	g	a	c	t	t	a	a	c	c	c	t	t	t	c	c	a	g	g	a	c	c	a	t	a	g	c	c
MH709346	3	a	t	c	t	t	c	a	g	a	c	g	c	g	c	g	t	t	a	g	c	t	a	a	a	t	t	g	t	a	g	g	t	a	g	g	t	g	t	a	g	t	t	a	g	a	a	g	g	a	c	t	t	a	a	c	c	c	t	t	t	c	c	a	g	g	a	c	c	a	t	a	g	c	c
MH709375	3	a	t	c	t	t	c	a	a	a	c	g	c	g	c	g	t	t	a	g	c	t	a	a	a	t	t	g	t	a	g	g	t	a	a	g	t	g	t	a	g	t	t	a	g	a	a	g	g	a	c	t	t	a	a	c	c	c	t	t	t	c	c	a	g	g	a	c	c	a	t	a	g	c	c
DQ452050	4	a	t	c	t	c	c	g	g	a	c	g	c	g	c	g	t	t	a	g	c	c	a	a	a	t	t	g	t	a	g	g	t	a	a	g	t	g	g	a	g	t	g	a	g	a	a	g	g	a	c	t	t	a	a	t	c	c	t	t	t	t	c	a	g	g	a	c	c	g	t	a	g	c	c
DQ479960	4	a	t	c	t	c	c	g	g	a	c	g	c	g	c	g	c	t	a	g	c	c	a	a	a	t	t	g	t	a	g	g	t	a	a	g	t	g	g	a	g	t	t	a	g	a	a	g	g	a	c	t	t	a	a	t	c	c	t	t	t	t	c	a	g	g	a	c	c	g	t	a	g	c	c
KP771898	4	a	t	c	t	c	c	g	g	a	c	g	c	g	c	g	t	t	a	g	c	c	a	a	a	t	t	g	t	a	g	g	t	a	a	g	t	g	g	a	g	t	t	a	g	a	a	g	g	a	c	t	t	a	a	t	c	c	t	t	t	t	c	a	g	g	a	c	c	g	t	a	g	c	c
MH709316	4	a	t	c	t	c	c	g	g	a	c	g	c	g	c	g	t	t	a	g	c	c	a	g	a	t	t	g	t	a	g	g	t	a	a	g	t	g	g	a	g	t	t	a	g	a	a	g	g	a	c	t	t	a	a	t	c	c	t	t	t	t	c	a	g	g	a	c	c	g	t	a	g	c	c
MH709318	4	a	t	c	t	c	c	g	g	a	c	g	c	g	c	g	t	t	a	g	c	c	a	a	a	t	t	g	t	a	g	g	t	a	a	g	t	g	g	a	g	t	t	a	g	a	a	g	g	a	c	t	t	a	a	t	c	c	t	t	t	t	c	a	g	g	a	c	c	g	t	a	g	c	c
MH709322	4	a	t	c	t	c	c	g	g	a	c	g	c	g	c	g	t	t	a	g	c	c	a	a	a	t	t	g	t	a	g	g	t	a	a	g	t	g	g	a	g	t	t	a	g	a	a	g	g	a	c	t	t	a	a	t	c	c	t	t	t	t	c	a	g	g	a	c	c	g	t	a	g	c	c
MH709323	4	a	t	c	t	c	c	g	g	a	c	g	c	g	c	g	t	t	a	g	c	c	a	a	a	t	t	g	t	a	g	g	t	a	a	g	t	g	g	a	g	t	t	a	g	a	a	g	g	a	c	t	t	a	a	t	c	c	t	t	t	t	c	a	g	g	a	c	c	g	t	a	g	c	c
MH709326	4	a	t	c	t	c	c	g	g	a	c	g	c	g	c	g	t	t	a	g	c	c	a	a	a	t	t	g	t	a	g	g	t	a	a	g	t	g	g	a	g	c	t	a	g	a	a	g	g	a	c	t	t	a	a	t	c	c	t	t	t	t	c	a	g	g	a	c	c	g	t	a	g	c	c
MH709341	4	a	t	c	t	c	c	g	g	a	c	g	c	g	c	g	t	t	a	g	c	c	a	a	a	t	t	g	t	a	g	g	t	a	a	g	t	g	g	a	g	t	t	a	g	a	a	g	g	a	c	t	t	a	a	t	c	c	t	t	t	t	c	a	g	g	a	c	c	g	t	a	g	c	c
MH709357	4	a	t	c	t	c	c	g	g	a	c	g	c	g	c	g	c	t	a	g	c	c	a	a	a	t	t	g	t	a	g	g	t	a	a	g	t	g	g	a	g	t	t	a	g	a	a	g	g	a	c	t	t	a	a	t	c	c	t	t	t	t	c	a	g	g	a	c	c	g	t	a	g	c	c
MH709364	4	a	t	c	t	c	c	g	g	a	c	g	c	g	c	g	c	t	a	g	c	c	a	a	a	t	t	g	t	a	g	g	t	a	a	g	t	g	g	a	g	t	t	a	g	a	a	g	g	a	c	t	t	a	a	t	c	c	t	t	t	t	c	a	g	g	a	c	c	g	t	a	g	c	c
MH709370	4	a	t	c	t	c	c	g	g	a	c	g	c	g	c	g	c	t	a	g	c	c	a	a	a	t	t	g	t	a	g	g	t	a	a	g	t	g	g	a	g	t	t	a	g	a	a	g	g	a	c	t	t	a	a	t	c	c	t	t	t	t	c	a	g	g	a	c	c	g	t	a	g	c	c
MH709371	4	a	t	c	t	c	c	g	g	a	c	g	c	g	c	g	c	t	a	g	c	c	a	a	a	t	t	g	t	a	g	g	t	a	a	g	t	g	g	a	g	t	t	a	g	a	a	g	g	a	c	t	t	a	a	t	c	c	t	t	t	t	c	a	g	g	a	c	c	g	t	a	g	c	c
MH709372	4	a	t	c	t	c	c	g	g	a	c	g	c	g	c	g	c	t	a	g	c	c	a	a	a	t	t	g	t	a	g	g	t	a	a	g	t	g	g	a	g	t	t	a	g	a	a	g	g	a	c	t	t	a	a	t	c	c	t	t	t	t	c	a	g	g	a	c	c	g	t	a	g	c	c
DQ457052	5	a	t	c	t	c	a	g	g	a	c	g	c	g	c	g	t	t	a	g	c	t	a	a	a	t	t	g	t	a	g	g	t	a	a	g	t	g	g	a	g	t	t	a	g	a	a	g	g	a	c	t	t	a	a	c	c	c	t	t	t	t	c	a	g	g	a	c	c	g	t	a	g	c	c
JN704704	5	a	t	c	t	c	c	g	g	a	c	g	c	g	c	g	t	t	a	g	c	t	a	a	a	t	t	g	t	a	g	g	t	a	a	g	t	g	g	a	g	t	t	a	g	a	a	g	g	a	c	t	t	a	a	c	c	c	t	t	t	t	c	a	g	g	a	c	c	g	t	g	g	c	c
JN704705	5	a	t	c	t	c	c	g	g	a	c	g	c	g	c	g	t	t	a	g	c	t	a	a	a	t	t	g	t	a	g	g	t	a	a	g	t	g	g	a	g	t	t	a	g	a	a	g	g	a	c	t	t	a	a	c	c	c	t	t	t	t	c	a	g	g	a	c	c	g	t	a	g	c	c
JN704706	5	a	t	c	t	c	c	g	g	a	c	g	c	g	c	g	t	t	a	g	c	t	a	a	a	t	t	g	t	a	g	g	t	a	a	g	t	g	g	a	g	t	t	a	g	a	a	g	g	a	c	t	t	a	a	c	c	c	t	t	t	t	c	a	g	g	a	c	c	g	t	a	g	c	c
JN704707	5	a	t	c	t	c	c	g	g	a	c	g	c	g	c	g	t	t	a	g	c	t	a	a	a	t	t	g	t	a	g	g	t	a	a	g	t	g	g	a	g	t	t	a	g	a	a	g	g	a	c	t	t	a	a	c	c	c	t	t	t	t	c	a	g	g	a	c	c	g	t	a	g	c	c
JN704708	5	a	t	c	t	c	c	g	g	a	c	g	c	g	c	g	t	t	a	g	c	t	a	a	a	t	t	g	t	a	g	g	t	a	a	g	t	g	g	a	g	t	t	a	g	a	a	g	g	a	c	t	t	a	a	c	c	c	t	t	t	t	c	a	g	g	a	c	c	g	t	a	g	c	c
KP771904	5	a	t	c	t	c	c	g	g	a	c	g	c	g	c	a	t	t	a	g	c	t	a	a	a	t	t	g	t	a	a	g	t	a	a	g	t	g	g	a	g	t	t	a	g	a	a	g	g	a	c	t	t	a	a	c	c	c	t	t	t	t	c	a	g	g	a	c	c	g	t	a	g	c	c
KP771905	5	a	t	c	t	c	c	g	g	a	c	g	c	g	c	g	t	t	a	g	c	t	a	a	a	t	t	g	t	a	g	g	t	a	a	g	t	g	g	a	g	t	t	a	g	a	a	g	g	a	c	t	t	a	a	c	c	c	t	t	t	t	c	a	g	g	c	c	c	g	t	a	g	c	c
KP771906	5	a	t	c	t	c	c	g	g	a	c	g	c	g	c	g	t	t	a	g	c	t	a	a	a	t	t	g	t	a	g	g	t	a	a	g	t	g	g	a	g	t	t	a	g	a	a	g	g	a	c	t	t	a	a	c	c	c	t	t	t	t	c	a	g	g	a	c	c	g	t	a	g	c	c
KP771919	5	a	t	c	t	c	c	g	g	a	c	g	c	g	c	g	t	t	a	g	c	t	a	a	a	t	t	g	t	a	g	g	t	a	a	g	t	g	g	a	g	t	t	a	g	a	a	g	g	a	c	t	t	a	a	c	c	c	t	t	t	t	c	a	g	g	a	c	c	g	t	a	g	c	c
KP771922	5	a	t	c	t	c	c	g	g	a	c	g	c	g	c	g	t	t	a	g	c	t	a	a	a	t	t	g	t	a	g	g	t	a	a	g	t	g	g	a	g	t	t	a	g	a	a	g	g	a	c	t	t	a	a	c	c	c	t	t	t	t	c	a	g	g	a	c	c	g	t	a	g	c	c
KP771924	5	a	t	c	t	c	c	g	g	a	c	g	c	g	c	g	t	t	a	g	c	t	a	a	a	t	t	g	t	a	g	g	t	a	a	g	t	g	g	a	g	t	t	a	g	a	a	g	g	a	c	t	t	a	a	c	c	c	t	t	t	t	c	a	g	g	a	c	c	g	t	a	g	c	c
KM355696	5	a	t	c	t	c	c	g	g	a	c	g	c	g	c	g	t	t	a	g	c	t	a	a	a	t	t	g	t	a	g	g	t	a	a	g	t	g	g	a	g	t	t	a	g	a	a	g	g	a	c	t	t	a	a	c	c	c	t	t	t	t	c	a	g	g	a	c	c	g	t	a	g	c	c
KM355697	5	a	t	c	t	c	c	g	g	a	c	g	c	g	c	g	t	t	a	g	c	t	a	a	a	t	t	g	t	a	g	g	t	a	a	g	t	g	g	a	g	t	t	a	g	a	a	g	g	a	c	t	t	a	a	c	c	c	t	t	t	t	c	a	g	g	a	c	c	g	t	a	g	c	c
KM355698	5	a	t	c	t	c	c	g	g	a	c	g	c	g	c	g	t	t	a	g	c	t	a	a	a	t	t	g	t	a	g	g	t	a	a	g	t	g	g	a	g	t	t	a	g	a	a	g	g	a	c	t	t	a	a	c	c	c	t	t	t	t	c	a	g	g	a	c	c	g	t	a	g	c	c
KM355699	5	a	t	c	t	c	c	g	g	a	c	g	c	g	c	g	t	t	a	g	c	t	a	a	a	t	t	g	t	a	g	g	t	a	a	g	t	g	g	a	g	t	t	a	g	a	a	g	g	a	c	t	t	a	a	c	c	c	t	t	t	t	c	a	g	g	a	c	c	g	t	a	g	c	c
KM355701	5	a	t	c	t	c	c	g	g	a	t	g	c	g	c	g	t	t	a	g	c	t	a	a	a	t	t	g	t	a	g	g	t	a	a	g	t	g	g	a	g	t	t	a	g	a	a	g	g	a	c	t	t	a	a	c	c	c	t	t	t	t	c	a	g	g	a	c	c	g	t	a	g	c	c
KM355702	5	a	t	c	t	c	c	g	g	a	c	g	c	g	c	g	t	t	a	g	c	t	a	a	a	t	t	g	t	a	g	g	t	a	a	g	t	g	g	a	g	t	t	a	g	a	a	g	g	a	c	t	t	a	a	c	c	c	t	t	t	t	c	a	g	g	a	c	c	g	t	a	g	c	c
KM355703	5	a	t	c	t	c	c	g	g	a	c	g	c	g	c	g	t	t	a	g	c	t	a	a	a	t	t	g	t	a	g	g	t	a	a	g	t	g	g	a	g	t	t	a	g	a	a	g	g	a	c	t	t	a	a	c	c	c	t	t	t	t	c	a	g	g	a	c	c	g	t	a	g	c	c
KM355704	5	a	t	c	t	c	c	g	g	a	c	g	c	g	c	g	t	t	a	g	c	t	a	a	a	t	t	g	t	a	g	g	t	a	a	g	t	g	g	a	g	t	t	a	g	a	a	g	g	a	c	t	t	a	a	c	c	c	t	t	t	t	c	a	g	g	a	c	c	g	t	a	g	c	c
KM355705	5	a	t	c	t	c	c	g	g	a	c	g	c	g	c	g	t	t	a	g	c	t	a	a	a	t	t	g	t	a	g	g	t	a	a	g	t	g	g	a	g	t	t	a	g	a	a	g	g	a	c	t	t	a	a	c	c	c	t	t	t	t	c	a	g	g	a	c	c	g	t	a	g	c	c
KM355706	5	a	t	c	t	c	c	g	g	a	c	g	c	g	c	g	t	t	a	g	c	t	a	a	a	t	t	g	t	a	g	g	t	a	a	g	t	g	g	a	g	t	t	a	g	a	a	g	g	a	c	t	t	a	a	c	c	c	t	t	t	t	c	a	g	g	a	c	c	g	t	a	g	c	c
KM355707	5	a	t	c	t	c	c	g	g	a	c	g	c	g	c	g	t	t	a	g	c	t	a	a	a	t	t	g	t	a	g	g	t	a	a	g	t	g	g	a	g	t	t	a	g	a	a	g	g	a	c	t	t	a	a	c	c	c	t	t	t	t	c	a	g	g	a	c	c	g	t	a	g	c	c
KM355708	5	a	t	c	t	c	c	g	g	a	c	g	c	g	c	g	t	t	a	g	c	t	a	a	a	t	t	g	t	a	g	g	t	a	a	g	t	g	g	a	g	t	t	a	g	a	a	g	g	a	c	t	t	a	a	c	c	c	t	t	t	t	c	a	g	g	a	c	c	g	t	a	g	c	c
KM355709	5	a	t	c	t	c	c	g	g	a	c	g	c	g	c	g	t	t	a	g	c	t	a	a	a	t	t	g	t	a	g	g	t	a	a	g	t	g	g	a	g	t	t	a	g	a	a	g	g	a	c	t	t	a	a	c	c	c	t	t	t	t	c	a	g	g	a	c	c	g	t	a	g	c	c
KM355711	5	a	t	c	t	c	c	g	g	a	c	g	c	g	c	g	t	t	a	g	c	t	a	a	a	t	t	g	t	a	g	g	t	a	a	g	t	g	g	a	g	t	t	a	g	a	a	g	g	a	c	t	t	a	a	c	c	c	t	t	t	t	c	a	g	g	a	c	c	g	t	a	g	c	c
KM355712	5	a	t	c	t	c	c	g	g	a	c	g	c	g	c	g	t	t	a	g	c	t	a	a	a	t	t	g	t	a	g	g	t	a	a	g	t	g	g	a	g	t	t	a	g	a	a	g	g	a	c	t	t	a	a	c	c	c	t	t	t	t	c	a	g	g	a	c	c	g	t	a	g	c	c
[illegible]	5	[illegible]	[illegible]	[illegible]	[illegible]	[illegible]	[illegible]	[illegible]	[illegible]	[illegible]	[illegible]	[illegible]	[illegible]	[illegible]	[illegible]	[illegible]	[illegible]	[illegible]	[illegible]	[illegible]	[illegible]	[illegible]	[illegible]	[illegible]	[illegible]	[illegible]	[illegible]	[illegible]	[illegible]	[illegible]	[illegible]	[illegible]	[illegible]	[illegible]	[illegible]	[illegible]	[illegible]	[illegible]	[illegible]	[illegible]	[illegible]	[illegible]	[illegible]	[illegible]	[illegible]	[illegible]	[illegible]	[illegible]	[illegible]	[illegible]	[illegible]	[illegible]	[illegible]	[illegible]	[illegible]	[illegible]	[illegible]	[illegible]	[illegible]	[illegible]	[illegible]	[illegible]	[illegible]	[illegible]	[illegible]	[illegible]	[illegible]	[illegible]	[illegible]	[illegible]	[illegible]	[illegible]	[illegible]	[illegible]	[illegible]

Supplementary Table 3: 2880 SN

GB-ID	Clade markers																					4																											1																											
	Position in Dumas	78107	78144	78145	78220	78385	78526	78545	78663	78666	78698	78699	78705	78730	78756	78813	78834	78850	79009	79094	79137	79190	79210	79263	79300	79363	79393	79463	79471	79555	79609	79658	79770	79973	80080	80095	80244	80298	80306	80435	80436	80521	80578	80625	80692	80737	80832	80840	80899	80959	80966	80986	80998	81090	81095	81187	81268	81289	81322	81353	81419	81454	81464	81466	81472	81478	81712	81738	81762	81854	81886	81895	81940	81967	81973
MH499467	5	a	t	c	t	c	c	g	g	a	c	g	c	g	c	g	t	t	a	g	c	t	a	a	a	t	t	g	t	a	g	g	t	a	a	g	t	g	g	a	g	t	t	a	g	a	a	g	g	a	c	t	t	a	a	c	c	c	t	t	t	t	c	a	g	g	a	c	c	g	t	a	g	c	c
MH499469	5	a	t	c	t	c	c	g	g	a	c	g	c	g	c	g	t	t	a	g	c	t	a	a	a	t	t	g	t	a	g	g	t	a	a	g	t	g	g	a	g	t	t	a	g	a	a	g	g	a	c	t	t	a	a	c	c	c	t	t	t	t	c	a	g	g	a	c	c	g	t	a	g	c	c
MH709321	5	a	t	c	t	c	c	g	g	a	c	g	c	g	c	g	t	t	a	g	c	t	a	a	a	t	t	g	t	a	g	g	t	a	a	g	t	g	g	a	g	t	t	a	g	a	a	g	g	a	c	t	t	a	a	c	c	c	t	t	t	t	c	a	g	g	a	c	c	g	t	a	g	c	c
MH709324	5	a	t	c	t	c	c	g	g	a	c	g	c	g	c	g	t	t	a	g	c	t	a	a	a	t	t	g	t	a	g	g	t	a	a	g	t	g	g	a	g	t	t	a	g	a	a	g	g	a	c	t	t	a	a	c	c	c	t	t	t	t	c	a	g	g	a	c	c	g	t	a	g	c	c
MH709325	5	a	t	c	t	c	c	g	g	a	c	g	c	g	c	g	t	t	a	g	c	t	a	a	a	t	t	g	t	a	g	g	t	a	a	g	t	g	g	a	g	t	t	a	g	a	a	g	g	a	c	t	t	a	a	c	c	c	t	t	t	t	c	a	g	g	a	c	c	g	t	a	g	c	c
MH709327	5	a	t	c	t	c	c	g	g	a	c	g	c	g	c	g	t	t	a	g	c	t	a	a	a	t	t	g	t	a	g	g	t	a	a	g	t	g	g	a	g	t	t	a	g	a	a	g	g	a	c	t	t	a	a	c	c	c	t	t	t	t	c	a	g	g	a	c	c	g	t	a	g	c	c
MH709328	5	a	t	c	t	c	c	g	g	a	c	g	c	g	c	g	t	t	a	g	c	t	a	a	a	t	t	g	t	a	g	g	t	a	a	g	t	g	g	a	g	t	t	a	g	a	a	g	g	a	c	t	t	a	a	c	c	c	t	t	t	t	c	a	g	g	a	c	c	g	t	a	g	c	c
MH709330	5	a	t	c	t	c	c	g	g	a	c	g	c	g	c	g	t	t	a	g	c	t	a	a	a	t	t	g	t	a	g	g	t	a	a	g	t	g	g	a	g	t	t	a	g	a	a	g	g	a	c	t	t	a	a	c	c	c	t	t	t	t	c	a	g	g	a	c	c	g	t	a	g	c	c
MH709332	5	a	t	c	t	c	c	g	g	a	c	g	c	g	c	g	t	t	a	g	c	t	a	a	a	t	t	g	t	a	g	g	t	a	a	g	t	g	g	a	g	t	t	a	g	a	a	g	g	a	c	t	t	a	a	c	c	c	t	t	t	t	c	a	g	g	a	c	c	g	t	a	g	c	c
MH709334	5	a	t	c	t	c	c	g	g	a	c	g	c	g	c	g	t	t	a	g	c	t	a	a	a	t	t	g	t	a	g	g	t	a	a	g	t	g	g	a	g	t	t	a	g	a	a	g	g	a	c	t	t	a	a	c	c	c	t	t	t	t	c	a	g	g	a	c	c	g	t	a	g	c	c
MH709335	5	a	t	c	t	c	a	g	g	a	c	g	c	g	c	g	t	t	a	g	c	t	a	a	a	t	t	g	t	a	g	g	t	a	a	g	t	g	g	a	g	t	t	a	g	a	a	g	g	a	c	t	t	a	a	c	c	c	t	t	t	t	c	a	g	g	a	c	c	g	t	a	g	c	c
MH709336	5	a	t	c	t	c	c	g	g	a	c	g	c	g	c	g	t	t	a	g	c	t	a	a	a	t	t	g	t	a	g	g	t	a	a	g	t	g	g	a	g	t	t	a	g	a	a	g	g	a	c	t	t	a	a	c	c	c	t	t	t	t	c	a	g	g	a	c	c	g	t	a	g	c	c
MH709338	5	a	t	c	t	c	c	g	g	a	c	g	c	g	c	g	t	t	a	g	c	t	a	a	a	t	t	g	t	a	g	g	t	a	a	g	t	g	g	a	g	t	t	a	g	a	a	g	g	a	c	t	t	a	a	c	c	c	t	t	t	t	c	a	g	g	a	c	c	g	t	a	g	c	c
MH709340	5	a	t	c	t	c	c	g	g	a	c	g	c	g	c	g	t	t	a	g	c	t	a	a	a	t	t	g	t	a	g	g	t	a	a	g	t	g	g	a	g	t	t	a	g	a	a	g	g	a	c	t	t	a	a	c	c	c	t	t	t	t	c	a	g	g	a	c	c	g	t	a	g	c	c
MH709342	5	a	t	c	t	c	c	g	g	a	c	g	c	g	c	g	t	t	a	g	c	t	a	a	a	t	t	g	t	a	g	g	t	a	a	t	t	g	g	a	g	t	t	a	g	a	a	g	g	a	c	t	t	a	a	c	c	c	t	t	t	t	c	a	g	g	a	c	c	g	t	a	g	c	c
MH709343	5	a	t	c	t	c	c	g	g	a	c	g	c	g	c	g	t	t	a	g	c	t	a	a	a	t	t	g	t	a	g	g	t	a	a	g	t	g	g	a	g	t	t	a	g	a	a	g	g	a	c	t	t	a	a	c	c	c	t	t	t	t	c	a	g	g	a	c	c	g	t	a	g	c	c
MH709345	5	a	t	c	t	c	a	g	g	a	c	g	c	g	c	g	t	t	a	g	c	t	a	a	a	g	t	g	t	a	g	g	t	a	a	g	t	g	g	a	g	t	t	a	g	a	a	g	g	a	c	t	t	a	a	c	c	c	t	t	t	t	c	a	g	g	a	c	c	g	t	a	g	c	c
MH709347	5	a	t	c	t	c	a	g	g	a	c	g	c	g	c	g	t	t	a	g	c	t	a	a	a	t	t	g	t	a	g	g	t	a	a	g	t	g	g	a	g	t	t	a	g	a	a	g	g	a	c	t	t	a	a	c	c	c	t	t	t	t	c	a	g	g	a	c	c	g	t	a	g	c	c
MH709349	5	a	t	c	t	c	c	g	g	a	c	g	a	g	c	g	t	t	a	g	c	t	a	a	a	t	t	g	t	a	g	g	t	a	a	g	t	g	g	a	g	t	t	a	g	a	a	g	g	a	c	t	t	a	a	c	c	c	t	t	t	t	c	a	g	g	a	c	c	g	t	a	g	t	t
MH709350	5	a	t	c	t	c	c	g	g	a	c	g	c	g	c	g	t	t	a	g	c	t	a	a	a	t	t	g	t	a	g	g	t	a	a	g	t	g	g	a	g	t	t	a	g	a	a	g	g	a	c	t	t	a	a	c	c	c	t	t	t	t	c	a	g	g	a	c	c	g	t	a	g	c	c
MH709351	5	a	t	c	t	c	c	g	g	a	c	g	c	g	c	g	t	t	a	g	c	t	a	a	a	t	t	g	t	a	g	g	t	a	a	g	t	g	g	a	g	t	t	a	g	a	a	g	g	a	c	t	t	a	a	c	c	c	t	t	t	t	c	a	g	g	a	c	c	g	t	a	g	c	c
MH709353	5	a	t	c	t	c	a	g	g	a	c	g	c	g	c	g	t	t	a	g	c	t	a	a	a	t	t	g	t	a	g	g	t	a	a	g	t	g	g	a	g	t	t	a	g	a	a	g	g	a	c	t	t	a	a	c	c	c	t	t	t	t	c	a	g	g	a	c	c	g	t	a	g	c	c
MH709358	5	a	t	c	t	c	c	g	g	a	c	g	c	g	c	g	t	t	a	g	c	t	a	a	a	t	t	g	t	a	g	t	t	a	a	g	t	g	g	a	g	t	t	a	g	a	a	g	g	a	c	t	t	a	a	c	c	c	t	t	t	t	c	a	g	g	a	c	c	g	t	a	g	c	c
MH709360	5	a	t	c	t	c	c	g	g	a	c	g	c	g	c	g	t	t	a	g	c	t	a	a	a	t	t	g	t	a	g	g	t	a	a	g	t	g	g	a	g	t	t	a	g	a	a	g	g	a	c	t	t	a	a	c	c	c	t	t	t	t	c	a	g	g	a	c	c	g	t	a	g	c	c
MH709362	5	a	t	c	t	c	a	g	g	a	c	g	c	g	c	g	t	t	a	g	c	t	a	a	a	t	t	g	t	a	g	g	t	a	a	g	t	g	g	a	g	t	t	a	g	a	a	g	g	a	c	t	t	a	a	c	c	c	t	t	t	t	c	a	g	g	a	c	c	g	t	a	g	c	c
MH709365	5	a	t	c	t	c	c	g	g	a	c	g	c	g	c	g	t	t	a	g	c	t	a	a	a	t	t	g	t	a	g	g	t	a	a	g	t	g	g	a	g	t	t	a	g	a	a	g	g	a	c	t	t	a	a	c	c	c	t	t	t	t	c	a	g	g	a	c	c	g	t	a	g	c	c
MH709373	5	a	t	c	t	c	c	g	g	a	c	g	c	g	c	g	t	t	a	g	c	t	a	a	a	t	t	g	t	a	g	g	t	a	a	g	t	g	g	a	g	t	t	a	g	a	a	g	g	a	c	t	t	a	a	c	c	c	t	t	t	t	c	a	g	g	a	c	c	g	t	a	g	c	c
MH709374	5	a	t	c	t	c	a	g	g	a	c	g	c	g	c	g	t	t	a	g	c	t	a	a	a	t	t	g	t	a	g	g	t	a	a	g	t	g	g	a	g	t	t	a	g	a	a	g	g	a	c	t	t	a	a	c	c	c	t	t	t	t	c	a	g	g	a	c	c	g	t	a	g	c	c
KC112914	6	a	t	c	t	t	c	a	g	a	c	g	c	g	c	g	t	t	a	t	c	t	a	a	a	t	t	g	t	a	g	g	t	a	a	g	t	g	t	a	g	t	t	a	g	a	a	g	g	a	c	t	t	a	a	c	c	c	t	t	t	t	c	a	g	g	a	c	c	a	t	a	g	c	c
KP771914	6	a	t	c	t	t	c	a	g	a	c	g	c	g	c	g	t	t	a	t	c	t	a	a	a	t	t	g	t	a	g	g	t	a	a	g	t	g	t	a	g	t	t	a	g	a	a	g	g	a	c	t	t	a	a	c	c	c	t	t	t	t	c	a	g	g	a	c	c	a	t	a	g	c	c
KP702725	6	a	t	c	t	t	c	a	g	a	c	g	c	g	c	g	t	t	a	t	c	t	a	a	a	t	t	g	t	a	g	g	t	a	a	g	t	g	t	a	g	t	t	a	g	a	a	g	g	a	c	t	t	a	a	c	t	c	t	t	t	t	c	a	g	g	a	c	c	a	t	a	g	c	c
KP771890	9	a	t	c	t	t	c	a	g	a	c	g	c	g	c	g	t	t	a	g	c	t	a	a	a	t	t	g	t	a	g	g	t	a	a	g	t	g	t	a	g	t	t	g	g	a	a	g	g	a	c	t	t	a	a	c	c	c	t	t	t	t	c	a	g	g	a	c	c	a	t	a	g	c	c
JN704710	9	a	t	a	t	c	c	a	g	a	c	g	c	g	c	g	t	t	a	g	c	t	a	a	a	t	t	g	t	a	g	g	t	a	a	g	t	g	t	a	g	t	t	a	g	a	a	g	g	a	c	t	t	a	a	c	c	c	t	t	t	t	c	a	g	g	a	c	c	g	t	a	g	c	c
KY037798	9	a	c	c	t	t	c	a	g	a	c	g	c	g	c	g	t	t	a	g	c	t	a	a	a	t	t	g	t	a	g	g	t	a	a	g	t	g	t	a	g	t	t	a	g	a	a	g	g	a	c	t	t	a	a	c	c	c	t	t	t	t	c	a	g	g	a	c	c	a	t	a	g	c	c
MH709312	9	a	c	c	t	t	c	a	g	a	c	g	c	g	c	g	t	t	a	g	c	t	a	a	a	t	t	g	t	a	g	g	t	a	a	g	t	g	t	a	g	t	t	a	g	a	a	g	g	a	c	t	t	a	a	c	c	c	t	t	t	t	c	a	g	g	a	c	c	a	t	a	g	c	c
MH709352	9	a	c	c	t	t	c	a	g	a	c	g	c	g	c	g	t	t	a	g	c	t	a	a	a	t	t	g	c	a	g	g	t	a	a	g	t	g	t	a	g	t	t	a	g	a	a	g	g	a	c	t	t	a	a	c	c	c	t	t	t	t	c	a	g	g	a	c	c	a	t	a	g	c	c
AB097933	Vac	a	t	c	t	c	c	g	g	a	c	g	c	g	c	g	t	t	a	g	c	t	a	a	a	t	t	g	t	a	g	g	t	a	a	g	t	g	g	a	g	t	t	a	g	a	a	g	g	a	c	t	t	a	a	t	c	c	t	t	t	t	c	a	g	g	a	c	c	g	t	a	g	c	c
JN704698	Vac	a	t	c	t	c	c	g	g	a	c	g	c	g	c	g	t	t	a	g	c	t	a	a	a	t	t	g	t	a	g	g	t	a	a	g	t	g	g	a	g	t	t	a	g	a	a	g	g	a	c	t	t	a	a	t	c	c	t	t	t	t	c	a	g	g	a	c	c	g	t	a	g	c	c
JN704697	Vac	a	t	c	t	c	c	g	g	a	c	g	c	g	c	g	t	t	a	g	c	t	a	a	a	t	t	g	t	a	g	g	t	a	a	g	t	g	g	a	g	t	t	a	g	a	a	g	g	a	c	t	t	a	a	t	c	c	t	t	t	t	c	a	g	g	a	c	c	g	t	a	g	c	c
DQ008355	Vac	a	t	c	t	c	c	g	g	a	c	g	c	g	c	g	t	t	a	g	c	t	a	a	a	t	t	g	t	a	g	g	t	a	a	g	t	g	g	a	g	t	t	a	g	a	a	g	g	a	c	t	t	a	a	t	c	c	t	t	t	t	c	a	g	g	a	c	c	g	t	a	g	c	c
JF306641	Vac	a	t	c	t	c	c	g	g	a	c	g	c	g	c	g	t	t	a	g	c	t	a	a	a	t	t	g	t	a	g	g	t	a	a	g	t	g	g	a	g	t	t	a	g	a	a	g	g	a	c	t	t	a	a	t	c	c	t	t	t	t	c	a	g	g	a	c	c	g	t	a	g	c	c
AB097932	Vac	a	t	c	t	c	c	g	g	a	c	g	c	g	c	g	t	t	a	g	c	t	a	a	a	t	t	g	t	a	g	g	t	a	a	g	t	g	g	a	g	t	t	a	g	a	a	g	g	a	c	t	t	a	a	t	c	c	t	t	t	t	c	a	g	g	a	c	c	g	t	a	g	c	c
DQ008354	Vac	a	t	c	t	c	c	g	g	a	c	g	c	g	c	g	t	t	a	g	c	t	a	a	a	t	t	g	t	a	g	g	t	a	a	g	t	g	g	a	g	t	t	a	g	a	a	g	g	a	c	t	t	a	a	t	c	c	t	t	t	t	c	a	g	g	a	c	c	g	t	a	g	c	c
KU926312	Vac	a	t	c	t	c	c	g	g	a	c	g	c	g	c	g	t	t	a	g	c	t	a	a	a	t	t	g	t	a	g	g	t	a	a	g	t	g	g	a	g	t	t	a	g	a	a	g	g	a	c	t	t	a	a	t	c	c	t	t	t	t	c	a	g	g	a	c	c	g	t	a	g	c	c
KU926313	Vac	a	t	c	t	c	c	g	g	a	c	g	c	g	c	g	t	t	a	g	c	t	a	a	a	t	t	g	t	a	g	g	t	a	a	g	t	g	g	a	g	t	t	a	g	a	a	g	g	a	c	t	t	a	a	t	c	c	t	t	t	t	c	a	g	g	a	c	c	g	t	a	g	c	c
KU926314	Vac	a	t	c	t	c	c	g	g	a	c	g	c	g	c	g	t	t	a	g	c	t	a	a	a	t	t	g	t	a	g	g	t	a	a	g	t	g	g	a	g	t	t	a	g	a	a	g	g	a	c	t	t	a	a	t	c	c	t	t	t	t	c	a	g	g	a	c	c	g	t	a	g	c	c
KF558371	Vac	a	t	c	t	c	c	g	g	a	c	g	c	g	c	g	t	t	a	g	c	t	a	a	a	t	t	g	t	a	g	g	t	a	a	g	t	g	g	a	g	t	t	a	g	a	a	g	g	a	c	t	t	a	a	t	c	c	t	t	t	t	c	a	g	g	a	c	c	g	t	a	g	c	c
KF558372	Vac	a	t	c	t	c	c	g	g	a	c	g	c	g	c	g	t	t	a	g	c	t	a	a	a	t	t	a	t	a	g	g	t	a	a	g	t	g	g	a	g	t	t	a	g	a	a	g	g	a	c	t	t	a	a	t	c	c	t	t	t	t	c	a	g	g	a	c	c	g	t	a	g	c	c
KF558373	Vac	a	t	c	t	c	c	g	g	a	c	g	c	g	c	g	t	t	a	g	c	t	a	a	a	t	t	g	t	a	g	g	t	g	a	g	t	g	g	a	g	t	t	a	g	a	a	g	g	a	c	t	t	a	a	t	c	c	t	t	t	t	c	a	g	g	a	c	c	g	t	a	g	c	c
KF558374	Vac	a	t	c	t	c	c	g	g	a	c	g	c	g	c	g	t	t	a	g	c	t	a	a	a	t	t	g	t	a	g	g	t	a	a	g	t	g	g	a	g	t	t	a	g	a	a	g	g	a	c	t	t	a	a	t	c	c	t	t	t	t	c	a	g	g	a	c	c	g	t	a	g	c	c
KF558375	Vac	a	t	c	t	c	c	g	g	a	c	g	c	g	c	g	t	t	a	g	c	t	a	a	a	t	t	g	t	a	g	g	t	a	a	g	t	g	g	a	g	t	t	a	g	a	a	g	g	a	c	t	t	a	a	t	c	c	t	t	t	t	c	a	g	g	a	c	c	g	t	a	g	c	c
KF558376	Vac	a	t	c	t	c	c	g	g	a	c	g	c	g	c	g	t	t	a	g	c	t	a	a	a	t	t	g	t	a	g	g	t	a	a	g	t	g	g	a	g	t	t	a	g	a	a	g	g	a	c	t	t	a	a	t	c	c	t	t	t	t	c	a	g	g	a	c	c	g	c	a	g	c	c
KF558377	Vac	a	t	c	t	c	c	g	g	a	c	g	c	g	c	g	t	t	a	g	c	t	a	a	a	t	t	g	t	a	g	g	t	a	a	g	t	g	g	a	g	t	t	a	g	a	a	g	g	a	c	t	t	a	a	t	c	c	c	t	t	t	c	a	g	g	a	c	c	g	t	a	a	c	c
KF558378	Vac	a	t	c	t	c	c	g	g	a	c	g	c	g	c	g	t	t	a	g	c	t	a	a	a	t	t	g	t	a	g	g	t	a	a	g	t	t	g	a	g	t	t	a	g	a	a	g	g	a	c	t	t	a	a	t	c	c	t	t	t	t	c	a	g	g	a	c	c	g	t	a	g	c	c
KF558379	Vac	a	t	c	t	c	c	g	g	a	c	g	c	g	c	g	t	a	a	g	c	t	a	a	a	t	t	g	t	a	g	g	t	a	a	g	t	g	g	a	g	t	t	a	g	a	a	g	g	a	c	t	t	a	a	t	c	c	t	t	t	t	c	a	g	g	a	c	c	g	t	a	g	c	c
KF558380	Vac	a	t	c	t	c	c	g	g	a	c	g	c	g	c	g	t	t	a	g	c	t	a	a	a	t	t	g	t	a	g	g	t	a	a	g	t	g	g	a	g	t	t	a	g	a	a	g	g	a	c	t	t	a	a	t	c	c	t	t	t	t	c	a	g	g	a	c	c	g	t	a	g	c	c
KF558381	Vac	a	t	c	t	c	c	g	g	a	c	g	c	g	c	g	t	t	a	g	c	t	a	a	a	t	t	g	t	a	g	g	t	a	a	g	t	g	g	a	g	t	t	a	g	a	a	g	g	a	c	t	t	a	a	t	c	c	t	t	t	t	c	a	g	g	a	c	c	g	t	a	g	c	c
KF558382	Vac	a	t	c	t	c	c	g	g	a	c	g	c	g	c	g	t	t	a	g	c	t	a	a	a	t	t	g	t	a	g	g	t	a	a	g	t	g	g	a	g	t	t	a	g	a	a	g	g	a	c	t	t	a	a	t	c	c	t	t	t	t	c	a	g	g	a	c	c	g	t	a	g	c	c
KF558383	Vac	a	t	c	t	c	c	g	g	a	c	g	c	g	c	g	t	t	a	g	c	t	a	a	a	t	t	g	t	a	g	g	t	a	a	g	t	g	g	a	g	t	t	a	g	a	a	g	g	a	c	t	t	a	a	t	c	c	t	t	t	t	c	a	g	g	a	c	c	g	t	a	g	c	c
KF558384	Vac	a	t	c	t	c	c	g	g	a	c	g	c	g	c	g	t	t	a	g	c	t	a	a	a	t	t	g	t	a	g	g	t	a	a	g	t	g	g	a	g	t	t	a	g	a	a	g	g	a	c	t	t	a	a	t	c	c	t	t	t	t	c	a	g	g	a	c	c	g	t	a	g	c	c
KF558385	Vac	a	t	c	t	c	c	g	g	a	c	g	c	g	c	g	t	t	a	g	c	t	a	a	a	t	t	g	t	a	g	g	t	a	a	g	t	g	g	a	g	t	t	a	g	a	a	g	g	a	c	t	t	a	a	t	c	c	t	t	t	t	c	a	g	g	a	c	c	g	t	a	g	c	c
KF558386	Vac	a	t	c	t	c	c	g	g	g	c	g	c	g	c	g	t	t	a	g	c	t	a	a	a	t	t	g	t	a	g	g	t	a	a	g	t	g	g	a	g	t	t	a	g	a	a	g	g	a	c	t	t	a	a	t	c	c	c	t	t	t	c	a	g	g	a	c	c	g	t	a	g	c	c
KF558387	Vac	a	t	c	t	c	c	g	g	a	c	g	c	g	c	g	t	t	a	g	c	t	a	a	a	t	t	g	t	a	g	g	t	a	a	g	t	g	g	a	g	t	t	a	g	a	a	g	g	a	c	t	t	a	a	t	c	c	t	t	t	t	c	a	g	g	a	c	c	g	t	a	g	c	c
KF558388	Vac	a	t	c	t	c	c	g	g	a	c	g	c	g	c	g	t	t	a	g	c	t	a	a	a	t	t	g	t	a	g	g	t	a	a	g	t	g	g	a	g	t	t	a	g	a	a	g	g	a	c	t	t	a	a	t	c	c	t	t	t	t	c	a	g	g	a	c	c	g	t	a	g	c	c
KF558389	Vac	a	t	c	t	c	c	g	g	a	c	g	c	g	c	g	t	t	a	g	c	t	a	a	a	t	t	g	t	a	g	g	t	a	a	g	t	g	g	a	g	t	t	a	g	a	a	g	g	a	c	t	t	a	a	t	c	c	t	t	t	t	c	a	g	g	a	c	c	g	t	a	g	c	c
KF558390	Vac	a	t	c	t	c	c	g	g	a	c	g	c	g	c	g	t	t	a	g	c	t	a	a	a	t	t	g	t	a	g	g	t	a	a	g	t	g	g	a	g	t	t	a	g	a	a	g	g	a	c	t	t	a	a	t	c	c	t	t	t	t	c	a	g	g	a	c	c	g	t	a	g	c	c
KF558391	Vac	a	t	c	t	c	c	g	g	a	c	g	c	g	c	g	t	t	a	g	c	t	a	a	a	t	t	g	t	a	g	g	t	a	a	g	t	g	g	a	g	t	t	a	g	a	a	g	g	a	c	t	t	a	a	t	c	c	t	t	t	t	c	a	g	g	a	c	c	g	t	a	g	c	c
KF811485	Vac	a	t	c	t	c	c	g	g	a	c	g	c	g	c	g	t	t	a	g	c	t	a	a	a	t	t	g	t	a	g	g	t	a	a	g	t	g	g	a	g	t	t	a	g	a	a	g	g	a	t	t	t	a	a	t	c	c	t	t	t	t	c	a	g	g	a	c	c	g	t	a	g	c	c
KF853225	Vac	a	t	c	t	c	c	g	g	a	c	g	c	g	c	g	t	t	a	g	c	t	a	a	a	t	t	g	t	a	g	g	t	a	a	g	t	t	g	a	g	t	t	a	g	a	a	g	g	a	c	t	t	a	a	t	c	c	t	t	t	t	c	a	g	g	a	c	c	g	t	a	g	c	c
KF853226	Vac	a	t	c	t	c	c	g	g	a	c	g	c	g	c	g	t	t	a	g	c	t	a	a	a	t	t	g	t	a	g	g	t	a	a	g	t	g	g	a	g	t	t	a	g	a	a	g	g	a	c	t	t	a	a	t	c	c	t	t	t	t	c	a	g	g	a	c	c	g	t	a	g	c	c
KF853227	Vac	a	t	c	t	c	c	g	g	a	c	g	c	g	c	g	t	t	a	g	c	t	a	a	a	t	t	g	t	a	g	g	t	a	a	g	t	g	g	a	g	t	t	a	g	a	a	g	g	a	c	t	t	a	a	t	c	c	t	t	t	t	c	a	g	g	a	c	c	g	c	a	g	c	c
KF853228	Vac	a	t	c	t	c	c	g	g	a	c	g	c	g	c	g	t	t	a	g	c	t	a	a	a	t	t	g	t	a	g	g	t	a	a	g	t	g	g	a	g	t	t	a	g	a	a	g	g	a	c	t	t	a	a	t	c	c	t	t	t	t	c	a	g	g	a	c	c	g	t	a	g	c	c
KF853229	Vac	a	t	c	t	c	c	g	g	a	c	g	c	g	c	g	t	t	a	g	c	t	a	a	a	t	t	g	t	a	g	g	t	a	a	g	t	g	g	a	g	t	t	a	g	a	a	g	g	a	c	t	t	a	a	t	c	c	t	t	t	t	c	a	g	g	a	c	c	g	t	a	g	c	c
KF853230	Vac	a	t	c	t	c	c	g	g	a	c	g	c	g	c	g	t	t	g	g	c	t	a	a	a	t	t	g	t	a	g	g	t	a	a	g	t	g	g	a	g	t	t	a	g	a	a	g	g	a	c	t	t	a	a	t	c	c	t	t	t	t	c	a	g	g	a	c	c	g	t	a	g	c	c
KF853231	Vac	a	t	c	t	c	c	g	g	g	c	g	c	g	c	g	t	t	a	g	c	t	a	a	a	t	t	g	t	a	g	g	t	a	a	g	t	g	g	a	g	t	t	a	g	a	a	g	g	a	c	t	t	a	a	t	c	c	c	t	t	t	c	a	g	g	a	c	c	g	t	a	g	c	c
KF853232	Vac	a	t	c	t	c	c	g	g	a	c	g	c	g	c	g	t	t	a	g	c	t	a	a	a	t	t	g	t	a	g	g	t	a	a	g	t	g	g	a	g	t	t	a	g	a	a	g	g	a	c	t	t	a	a	t	c	c	t	t	t	t	c	a	g	g	a	c	c	g	t	a	g	c	c
KF853233	Vac	a	t	c	t	c	c	g	g	a	c	g	c	g	c	g	t	t	g	g	c	t	a	a	a	t	t	g	t	a	g	g	t	a	a	g	t	g	g	a	g	t	t	a	g	a	a	g	g	a	c	t	t	a	a	t	c	c	t	t	t	t	c	a	g	g	a	c	c	g	t	a	g	c	c
KF853234	Vac	a	t	c	t	c	c	g	g	a	c	g	c	g	c	g	t	t	a	g	c	t	a	a	a	t	t	g	t	a	g	g	t	a	a	g	t	g	g	a	g	t	t	a	g	a	a	g	g	a	c	t	t	a	a	t	c	c	t	t	t	t	c	a	g	g	a	c	c	g	t	a	g	c	c
KF853235	Vac	a	t	c	t	c	c	g	g	a	c	g	c	g	c	g	t	t	a	g	c	t	a	a	a	t	t	g	t	a	g	g	t	a	a	g	t	g	g	a	g	t	t	a	g	a	a	g	g	a	c	t	t	a	a	t	c	c	t	t	t	t	c	a	g	g	a	c	c	g	t	a	g	c	c
MF898328	Vac	a	t	c	t	c	c	g	g	a	c	g	c	g	c	g	t	t	a	g	c	t	a	a	a	t	t	g	t	a	g	g	t	a	a	g	t	g	g	a	g	t	t	a	g	a	a	g	g	a	c	t	t	a	a	t	c	c	t	t	t	t	c	a	g	g	a	c	c	g	t	a	g	c	c
JN704709	VIII	g	t	c	t	t	c	g	g	a	c	g	c	g	c	g	t	t	a	g	c	t	a	a	a	t	t	g	t	a	g	g	t	a	a	g	t	g	t	a	g	t	t	a	g	a	a	g	g	a	c	t	t	a	a	c	c	c	t	g	t	t	c	a	g	a	a	c	c	a	t	a	g	c	c

Supplementary Table 3: 2880 SN

GB-ID	Clade markers				9																																4	1																																							
	Position in Dumas	82141	82225	82294	82300	82495	82533	82601	82645	82663	82700	82765	82880	82882	82896	82906	83085	83129	83258	83303	83396	83455	83662	83722	83785	83845	83888	84001	84004	84010	84080	84084	84091	84433	84515	84547	84553	84611	84616	84655	84728	84810	84822	84834	84864	84970	84979	84983	85013	85041	85059	85122	85180	85181	85188	85272	85314	85377	85398	85431	85440	85560	85563	85594	85621	85686	85805	85957	85974	86018	86079	86115	86170	86199	86225
AY548170	1	a	a	t	c	c	g	c	a	t	g	t	a	a	g	a	a	t	a	a	t	a	t	a	g	t	g	a	a	a	t	a	g	t	c	c	g	g	a	t	g	g	g	t	t	c	c	g	a	a	t	t	a	c	a	g	a	a	t	a	g	t	c	a	a	c	c	t	a	t	c	g	c	g	g
AY548171	1	a	a	t	c	c	g	c	a	t	g	t	a	a	g	a	a	t	a	c	t	a	t	a	g	t	g	a	a	a	t	a	g	t	c	c	g	g	a	t	g	g	g	t	t	c	c	g	a	a	t	t	a	c	a	g	a	a	t	a	g	t	c	a	a	c	c	t	a	t	c	g	c	g	g
DQ479953	1	a	a	t	c	c	g	c	a	t	g	t	a	a	g	a	a	t	a	a	t	a	t	a	g	t	g	a	a	a	t	a	g	t	c	c	g	g	a	t	g	g	g	t	t	c	c	g	a	a	t	t	a	c	a	g	a	a	t	a	g	t	c	a	a	c	c	t	a	t	c	g	c	g	g
DQ479954	1	a	a	t	c	c	g	c	a	t	g	t	a	a	g	a	a	t	a	a	t	a	t	a	g	t	g	a	a	a	t	a	g	t	c	c	g	g	a	t	g	t	g	t	t	c	c	g	a	a	t	t	a	c	a	g	a	a	t	a	g	g	c	a	a	c	c	t	a	t	c	g	c	g	g
DQ479958	1	a	a	t	c	c	g	c	a	t	g	t	a	a	g	a	a	t	a	a	t	a	t	a	g	t	g	a	a	a	t	a	g	t	c	c	g	g	a	t	g	g	g	t	t	c	c	g	a	a	t	t	a	c	a	g	a	a	t	a	g	t	c	a	a	c	c	t	a	t	c	g	c	g	g
DQ479959	1	a	a	t	c	c	g	c	a	t	g	t	a	a	g	a	a	t	a	a	t	a	t	a	g	t	g	a	a	a	t	a	g	t	c	c	g	g	a	t	g	g	g	t	t	c	c	g	a	a	t	t	a	c	a	g	a	a	t	a	g	t	c	a	a	c	c	t	a	t	c	g	c	g	g
DQ479961	1	a	a	t	c	c	g	c	a	t	g	t	a	a	g	a	a	t	a	a	t	a	t	a	g	t	g	a	a	a	t	a	g	t	c	c	g	g	a	t	g	g	g	t	t	c	c	g	a	a	t	t	a	c	a	g	a	a	t	a	g	t	c	a	a	c	c	t	a	t	c	g	c	g	g
DQ479962	1	a	a	t	c	c	g	c	a	t	g	t	a	a	g	a	a	t	a	a	t	a	t	a	g	t	g	a	a	a	t	a	g	t	c	c	g	g	a	t	g	g	g	t	t	c	c	g	a	a	t	t	a	c	a	g	a	a	t	a	g	t	c	a	a	c	c	t	a	t	c	g	c	g	g
DQ479963	1	a	a	t	c	c	g	c	a	t	g	t	a	a	g	a	a	t	a	a	t	a	t	a	g	t	g	a	a	a	t	a	g	t	c	c	g	g	a	t	g	g	g	t	t	c	c	g	a	a	t	t	a	c	a	g	a	a	t	a	g	t	c	a	a	c	c	t	a	t	c	g	c	g	g
DQ674250	1	a	a	t	c	c	g	c	a	t	g	t	a	a	g	a	a	t	a	a	t	a	t	a	g	t	g	a	a	a	t	a	g	t	c	c	g	g	a	t	g	g	g	t	t	c	c	g	a	a	t	t	a	c	a	g	a	a	t	a	g	t	c	a	a	c	c	t	a	t	c	g	c	g	g
KX262864	1	a	a	t	c	c	g	c	a	t	g	t	a	a	g	a	a	t	a	a	t	a	t	a	g	t	g	a	a	a	t	a	g	t	c	c	g	g	a	t	g	g	g	t	t	c	c	g	a	a	t	t	a	c	a	g	a	a	t	a	g	t	c	a	a	c	c	t	a	t	c	g	c	g	g
KX262865	1	a	a	t	c	c	g	c	a	t	g	t	a	a	g	a	a	t	a	a	t	a	t	a	g	t	g	a	a	a	t	a	g	t	c	c	g	g	a	t	g	g	g	t	t	c	c	g	a	a	t	t	a	c	a	g	a	a	t	a	g	t	c	a	a	c	c	t	a	t	c	g	c	g	g
KX262866	1	a	a	t	c	c	g	c	a	t	g	t	a	a	g	a	a	t	a	a	t	a	t	a	g	t	g	a	a	a	t	a	g	t	c	c	g	g	a	t	g	g	g	t	t	c	c	g	a	a	t	t	a	c	a	g	a	a	t	a	g	t	c	a	a	c	c	g	a	t	c	g	c	g	g
EU154348	1	a	a	t	c	c	g	c	a	t	g	t	a	a	g	a	a	t	a	a	t	a	t	a	g	t	g	a	a	a	t	a	g	t	c	c	g	g	a	t	g	g	g	t	t	c	c	g	a	a	t	t	a	c	a	g	a	a	t	a	g	t	c	a	a	c	c	t	a	t	c	g	c	g	g
JN704690	1	a	a	t	c	c	g	c	a	t	g	t	a	a	g	a	a	t	a	a	t	a	t	a	g	t	g	a	a	a	t	a	g	t	c	c	g	g	a	t	g	g	g	t	t	c	c	g	a	a	t	t	a	c	a	g	a	a	t	a	g	t	c	a	a	c	c	t	a	t	c	g	c	g	g
JN704691	1	a	a	t	c	c	g	c	a	t	g	t	a	a	g	a	a	t	a	a	t	a	t	a	g	t	g	a	a	a	t	a	g	t	c	c	g	g	a	t	g	g	g	t	t	c	c	g	a	a	t	t	a	c	a	g	a	a	t	a	g	t	c	a	a	c	c	t	a	t	c	g	c	g	g
JN704692	1	a	a	t	c	c	g	c	a	t	g	t	a	a	g	a	a	t	a	a	t	a	t	a	g	t	g	a	a	a	t	a	g	t	c	c	g	g	a	t	g	g	g	t	t	c	c	g	a	a	t	t	a	c	a	g	a	a	t	a	g	t	c	a	a	c	c	t	a	t	c	g	c	g	g
JN704693	1	a	a	t	c	c	g	c	a	t	g	t	a	a	g	a	a	t	a	a	t	a	t	a	g	t	g	a	a	a	t	a	g	t	c	c	g	g	a	t	g	g	g	t	t	c	c	g	a	a	t	t	a	c	a	g	a	a	t	a	g	t	c	a	a	c	t	t	a	t	c	g	c	g	g
JN704694	1	a	a	t	c	c	g	c	a	t	g	t	a	a	g	a	a	t	a	a	t	a	t	a	g	t	g	a	a	a	t	a	g	t	c	c	g	g	a	t	g	g	g	t	t	c	c	g	a	a	t	t	a	c	a	g	a	a	t	a	g	t	c	a	a	c	c	t	a	t	c	g	c	g	g
JN704695	1	a	a	t	c	c	g	c	a	t	g	t	a	a	g	a	a	t	a	a	t	a	t	a	g	t	g	a	a	a	t	a	g	t	c	c	g	g	a	t	g	g	g	t	t	c	c	g	a	a	t	t	a	c	a	g	a	a	t	a	g	t	c	a	a	c	c	t	a	t	c	g	c	g	g
JN704696	1	a	a	t	c	c	g	c	a	t	g	t	a	a	g	a	a	t	a	a	t	a	t	a	g	t	g	a	a	a	t	a	g	t	c	c	g	g	a	t	g	g	g	t	t	c	c	g	a	a	t	t	a	c	a	g	a	a	t	a	g	t	c	a	a	c	c	t	a	t	c	g	c	g	g
NC001348	1	a	a	t	c	c	g	c	a	t	g	t	a	a	g	a	a	t	a	a	t	a	t	a	g	t	g	a	a	a	t	a	g	t	c	c	g	g	a	t	g	g	g	t	t	c	c	g	a	a	t	t	a	c	a	g	a	a	t	a	g	t	c	a	a	c	c	t	a	t	c	g	c	g	g
KP771892	1	a	a	t	c	c	g	c	a	t	g	t	a	a	g	a	a	t	a	a	t	a	t	a	g	t	g	a	a	a	t	a	g	t	c	c	g	g	a	t	g	g	g	t	t	c	c	g	a	a	t	t	a	c	a	g	a	a	t	a	g	t	c	a	a	t	c	t	a	t	c	g	c	g	a
KP771894	1	a	a	t	c	c	g	c	a	t	g	t	a	a	g	a	a	t	a	a	t	a	t	a	g	t	g	a	a	a	t	a	g	t	c	c	g	g	a	c	g	g	g	t	t	c	c	g	a	a	t	t	a	c	a	g	a	a	t	a	g	t	c	a	a	c	c	t	a	t	c	g	c	g	g
KP771900	1	a	a	t	c	c	g	c	a	t	g	t	a	a	g	c	a	t	a	a	t	a	t	a	g	t	g	a	a	a	t	a	g	t	c	c	g	g	a	t	g	g	g	t	t	c	c	g	a	a	t	t	a	c	a	g	a	a	t	a	g	t	c	a	a	c	c	t	a	t	c	g	c	g	g
KP771901	1	a	a	t	c	c	g	c	a	t	g	t	a	a	g	c	a	t	a	a	t	a	t	a	g	t	g	a	a	a	t	a	g	t	c	c	g	g	a	t	g	g	g	t	t	c	c	g	a	a	t	t	a	c	a	g	a	a	t	a	g	t	c	a	a	c	c	t	a	t	c	g	c	g	g
KP771902	1	a	a	t	c	c	g	c	a	t	g	t	a	a	g	c	a	t	a	a	t	a	t	a	g	t	g	a	a	a	t	a	g	t	c	c	g	g	a	t	g	g	g	t	t	c	c	g	a	a	t	t	a	c	a	g	a	a	t	a	g	t	c	a	a	c	c	t	a	t	c	g	c	g	g
KP771908	1	a	a	t	c	c	g	c	a	t	g	t	a	a	g	a	a	t	a	a	t	a	t	a	g	t	g	a	a	a	t	a	g	t	c	c	g	g	a	t	g	g	g	t	t	c	c	g	a	a	t	t	a	c	a	g	a	a	t	a	g	t	c	a	a	c	c	t	a	t	c	g	c	g	g
KP771912	1	a	a	t	c	c	g	c	a	t	g	t	a	a	g	a	a	t	a	a	t	a	t	a	g	t	g	a	a	a	t	a	g	t	c	c	g	g	a	t	g	g	g	t	t	c	c	g	a	a	t	t	a	c	a	g	a	a	t	a	g	t	c	a	a	c	c	t	a	t	c	g	c	g	g
KP771913	1	a	a	t	c	c	g	c	a	t	g	t	a	a	g	a	a	t	a	a	t	a	t	a	g	t	g	a	a	a	t	a	g	t	c	c	g	g	a	t	g	g	g	t	t	c	c	g	a	a	t	t	a	c	a	g	a	a	t	a	g	t	c	a	a	c	c	t	a	t	c	g	c	g	g
KP771917	1	a	a	t	c	c	g	c	a	t	g	t	a	a	g	a	a	t	a	a	t	a	t	a	g	t	g	a	a	a	t	a	g	t	c	c	g	g	a	t	g	g	g	t	t	c	c	g	a	a	t	t	a	c	a	g	a	a	t	a	g	t	c	a	a	c	c	t	a	t	c	g	c	g	g
KP771918	1	a	a	t	c	c	g	c	a	t	g	t	a	a	g	a	a	t	a	a	t	a	t	a	g	t	g	a	a	a	t	a	g	t	c	c	g	g	a	t	g	g	g	t	t	c	c	g	a	a	t	t	a	c	a	g	a	a	t	a	g	t	c	a	a	c	c	t	a	t	c	g	c	g	g
KP771920	1	a	a	t	c	c	g	c	a	t	g	t	a	a	g	a	a	t	a	a	t	a	t	a	g	t	g	a	a	a	t	a	g	t	c	c	g	g	a	t	g	g	g	t	t	c	c	g	a	a	t	t	a	c	a	g	a	a	t	a	g	t	c	a	a	c	c	t	a	t	c	g	c	g	g
MH709361	1	a	a	t	c	c	g	c	a	t	g	t	a	a	g	a	a	t	a	a	t	a	t	a	g	t	g	a	a	a	t	a	g	t	c	c	g	g	a	t	g	g	g	t	t	c	c	g	a	a	t	t	a	c	a	g	a	a	t	a	g	t	c	a	a	c	c	t	a	t	c	g	c	g	g
KU926318	2	a	a	t	c	c	g	c	a	t	g	t	a	a	g	a	a	t	a	a	t	a	t	a	g	t	g	g	a	a	t	a	g	t	c	c	g	g	g	t	g	g	g	t	t	c	c	a	a	a	c	t	a	c	a	g	a	a	t	a	g	t	t	a	a	c	c	t	a	t	c	g	a	g	g
KU926319	2	a	a	t	c	c	g	c	a	t	g	t	a	g	g	a	a	t	a	a	t	a	t	a	g	t	g	a	a	a	t	a	g	t	c	c	g	g	g	t	g	g	g	t	t	c	c	a	a	a	t	t	a	c	a	g	a	a	t	a	g	t	t	a	a	c	c	t	a	t	c	g	a	g	g
KU926320	2	a	a	t	c	c	g	c	a	t	g	t	a	a	g	a	a	t	a	a	t	a	t	a	g	c	g	a	g	a	t	a	g	t	c	c	g	g	g	t	g	g	g	t	t	c	c	a	a	a	t	t	a	c	a	g	a	g	t	a	g	t	t	a	a	c	c	t	a	t	c	g	a	g	g
KU926321	2	a	a	t	c	c	g	c	a	t	g	t	a	a	g	a	a	t	a	a	t	a	t	a	g	t	g	a	a	a	t	a	g	t	c	c	g	g	g	t	g	g	g	t	t	c	c	a	a	a	t	t	a	c	a	g	a	a	t	a	g	t	t	a	a	c	c	t	a	t	c	g	a	g	g
KU926322	2	a	a	t	c	c	g	c	a	t	g	t	a	a	g	a	a	t	a	a	t	a	t	a	g	t	g	a	a	a	t	a	g	t	c	c	g	g	g	t	g	g	g	t	t	c	c	a	a	a	t	t	a	c	a	g	a	a	t	a	g	t	t	a	a	c	c	t	a	t	c	g	a	g	g
KC847290	2	a	a	t	c	c	g	c	a	t	g	t	a	a	g	a	a	t	a	a	t	a	t	a	g	t	g	a	a	a	t	a	g	t	c	c	g	g	g	t	g	g	g	t	t	c	c	a	a	a	t	t	a	c	a	g	a	a	t	a	g	t	t	a	a	c	c	t	a	t	c	g	a	g	g
KJ767491	2	a	a	t	c	c	g	c	a	t	g	t	a	a	g	a	a	t	a	a	t	a	t	a	g	t	g	a	a	a	t	a	g	t	c	c	g	g	g	t	g	g	g	t	t	c	c	a	a	a	t	t	a	c	a	g	a	a	t	a	g	t	t	a	a	c	c	t	a	t	c	g	a	g	g
KJ767492	2	a	a	t	c	c	g	c	a	t	g	t	a	a	g	a	a	t	a	a	t	a	t	a	g	t	g	a	a	a	t	a	g	t	c	c	g	g	g	t	g	g	g	t	t	c	c	a	a	a	t	t	a	c	a	g	a	a	t	a	g	t	t	a	a	c	c	t	a	t	c	g	a	g	g
KJ808816	2	a	a	t	c	c	g	c	a	t	g	t	a	a	g	a	a	t	a	a	t	a	t	a	g	t	g	a	a	a	t	a	g	t	c	c	g	g	g	t	g	g	g	t	t	c	c	a	a	a	t	t	a	c	a	g	a	a	t	a	g	t	t	a	a	c	c	t	a	t	c	g	a	g	g
KP771925	2	a	a	t	c	c	g	c	a	t	g	t	a	a	g	a	a	t	a	a	t	a	t	a	g	t	g	a	a	a	t	a	g	t	c	c	g	g	g	t	g	g	g	t	t	c	c	a	a	a	t	t	a	c	a	g	a	a	t	a	g	t	t	a	a	c	c	t	a	t	c	g	a	g	g
KU926315	2	a	a	t	c	c	g	c	a	t	g	t	a	a	g	a	a	t	a	a	t	a	t	a	g	t	g	a	a	a	t	a	g	t	c	c	g	g	g	t	g	g	g	t	t	c	c	a	a	a	t	t	a	c	a	g	a	a	t	a	g	t	t	a	a	c	c	t	a	t	c	g	a	g	g
KU926316	2	a	a	t	c	c	g	c	a	t	g	t	a	a	g	a	a	t	a	a	t	a	t	a	g	t	g	a	a	a	t	a	g	t	c	c	g	g	g	t	g	g	g	t	t	c	c	a	a	a	t	t	a	c	a	g	a	a	t	a	g	t	t	a	a	c	c	t	a	t	c	g	a	g	g
KU926317	2	a	a	t	c	c	g	c	a	t	g	t	a	a	g	a	a	t	a	a	t	a	t	a	g	t	g	a	a	a	t	a	g	t	c	c	g	g	g	t	g	g	g	t	t	c	c	a	a	a	t	t	a	c	a	g	a	a	t	a	g	t	t	a	a	c	c	t	a	t	c	g	a	g	g
KY037796	2	a	a	t	c	c	g	c	a	t	g	t	a	a	g	a	a	t	a	a	t	a	t	a	g	t	g	a	a	a	t	a	g	t	c	c	g	g	g	t	g	g	g	t	t	c	c	a	a	a	t	t	a	c	a	g	a	a	t	a	g	t	t	a	a	c	c	t	a	t	c	g	a	g	g
MF004348	2	a	a	t	c	c	g	c	a	t	g	t	a	a	g	a	a	t	a	a	t	a	t	a	g	t	g	a	a	a	t	a	g	t	c	c	g	g	g	t	g	g	g	t	t	c	c	a	a	a	t	t	a	c	a	g	a	a	t	a	g	t	t	a	a	c	c	t	a	t	c	g	a	g	g
MH709309	2	a	a	t	c	c	g	c	a	t	g	t	a	a	g	a	a	t	a	a	t	a	t	a	g	t	g	a	a	a	t	a	g	t	c	c	g	g	g	t	g	g	g	t	t	c	c	a	a	a	t	t	a	c	a	g	a	a	t	a	g	t	t	a	a	c	c	t	a	t	c	g	a	g	g
MH709310	2	a	a	t	c	c	g	c	a	t	g	t	a	a	g	a	a	t	a	a	t	a	t	a	g	t	g	a	a	a	t	a	g	t	c	c	g	g	g	t	g	g	g	t	t	c	c	a	a	a	t	t	a	c	a	g	a	a	t	a	g	t	t	a	a	c	c	t	a	t	c	g	a	g	g
MH709311	2	a	a	t	c	c	g	c	a	t	g	t	a	a	g	a	a	t	a	a	t	a	t	a	g	t	g	a	a	a	t	a	g	t	a	c	g	g	g	t	g	g	t	t	t	c	a	a	a	a	t	t	a	c	a	g	a	a	t	a	g	t	t	a	a	c	c	t	a	t	c	g	a	g	g
MH709313	2	a	a	t	c	c	g	c	a	t	g	t	a	a	g	a	a	t	a	a	t	a	t	a	g	t	g	a	a	a	t	a	g	t	c	c	g	g	g	t	g	g	g	t	t	c	c	a	a	a	t	t	a	c	a	g	a	a	t	a	g	t	t	a	a	c	c	t	g	t	c	g	a	a	g
MH709314	2	a	a	t	c	c	g	c	a	t	g	t	a	a	g	a	a	t	a	a	t	a	t	a	g	t	g	a	a	a	t	a	g	t	c	c	g	g	g	t	g	g	g	t	t	c	c	a	a	a	t	t	a	c	a	g	a	a	t	a	g	t	t	a	a	c	c	t	a	t	c	g	a	g	g
MH709315	2	a	a	t	c	c	g	c	a	t	g	t	a	a	g	a	a	t	a	a	t	a	t	a	g	t	g	a	a	a	t	a	g	t	c	c	g	g	g	t	g	g	g	t	t	c	c	a	a	a	t	t	a	c	a	g	a	a	t	a	g	t	t	a	g	c	c	t	a	t	c	g	a	g	g
MH709319	2	a	a	t	c	c	g	c	a	t	g	t	a	a	g	a	a	t	a	a	t	a	t	a	g	t	g	a	a	a	t	a	g	t	c	c	g	g	g	t	g	g	g	t	t	c	c	a	a	a	t	t	a	c	a	g	a	a	t	a	g	t	t	a	a	c	c	t	a	t	c	g	a	g	g
MH709320	2	a	a	t	c	c	g	c	a	t	g	t	a	a	g	a	a	t	a	a	t	a	t	a	g	t	g	a	a	a	t	a	g	t	c	c	g	g	g	t	g	g	g	t	t	c	c	a	a	a	t	t	a	c	a	g	a	a	t	a	g	t	t	a	a	c	c	t	a	t	c	g	a	g	g
MH709329	2	a	a	t	c	c	g	c	a	t	g	t	a	a	g	a	a	t	a	a	t	a	t	a	g	t	g	a	a	a	t	a	g	t	c	c	g	g	g	t	g	g	g	t	t	c	c	a	a	a	t	t	a	c	a	g	a	a	t	a	g	t	t	a	a	c	c	t	a	t	c	g	a	g	g
MH709331	2	a	a	t	c	c	g	c	a	t	g	t	a	a	g	a	a	t	a	a	t	a	t	a	g	t	g	a	a	a	t	a	g	t	c	c	g	g	g	t	g	g	g	t	t	c	c	a	a	a	t	t	a	c	a	g	a	a	t	a	g	t	t	a	a	c	c	t	a	t	c	g	a	g	g
MH709333	2	a	a	t	c	c	g	c	a	t	g	t	a	a	g	a	a	t	a	a	t	a	t	a	g	t	g	a	a	a	t	a	g	t	c	c	g	g	g	t	g	g	g	t	t	c	c	a	a	a	t	t	a	c	a	g	a	a	t	a	g	t	t	a	a	c	c	t	a	t	c	g	a	g	g
MH709337	2	a	a	t	c	c	g	c	a	t	g	t	a	a	g	a	a	t	a	a	t	a	t	a	g	t	g	a	a	a	t	a	g	t	c	c	g	g	g	t	g	g	g	t	t	c	c	a	a	a	t	t	a	c	a	g	a	a	t	a	g	t	t	a	a	c	c	t	a	t	c	g	a	g	g
MH709339	2	a	a	t	c	c	g	c	a	t	g	t	a	a	g	a	a	t	a	a	t	a	t	a	g	t	g	a	a	a	t	a	g	t	c	c	g	g	g	t	g	g	g	t	t	c	c	a	a	a	t	t	a	c	a	g	a	a	t	a	g	t	t	a	a	c	c	t	a	t	c	g	a	g	g
MH709344	2	a	a	t	c	c	g	c	a	t	g	t	a	a	g	a	a	t	a	a	t	a	t	a	g	t	g	a	a	a	t	a	g	t	c	c	g	g	g	t	g	g	g	t	t	c	c	a	a	a	t	t	a	c	a	g	a	a	t	a	g	t	t	a	a	c	c	t	a	t	c	g	a	g	g
MH709348	2	a	a	t	c	c	g	c	a	t	g	t	a	a	g	a	a	t	a	a	t	a	t	a	g	t	g	a	a	a	t	a	g	t	c	c	g	g	g	t	g	g	g	t	t	c	c	a	a	a	t	t	a	c	a	g	a	a	t	a	g	t	t	a	a	c	c	t	a	t	c	g	a	g	g
MH709355	2	a	a	t	c	c	g	c	a	t	g	t	a	a	g	a	a	t	a	a	t	a	t	a	g	t	g	a	a	a	t	a	g	t	c	c	g	g	g	t	g	g	g	t	t	c	c	a	c	a	t	t	a	c	a	g	a	a	t	a	g	t	t	a	a	c	c	t	a	t	c	g	a	g	g
MH709359	2	a	a	t	c	c	g	c	a	t	g	t	a	a	g	a	a	t	a	a	t	a	t	a	g	t	g	a	a	a	t	a	g	t	c	c	g	g	g	t	g	g	g	t	t	c	c	a	a	a	t	t	a	c	a	g	a	a	t	a	g	t	t	a	a	c	c	t	a	t	c	g	a	g	g
MH709363	2	a	a	t	c	c	g	c	a	t	g	t	a	a	g	a	a	t	a	a	t	a	t	a	g	t	g	a	a	a	t	a	g	t	c	c	g	g	g	t	g	g	g	t	t	c	c	a	a	a	t	t	a	c	a	g	a	a	t	a	g	t	t	a	a	c	c	t	a	t	c	g	a	g	g
MH709366	2	a	a	t	c	c	g	c	a	t	g	t	a	a	g	a	a	t	a	a	t	a	t	a	g	t	g	a	a	a	t	a	g	t	c	c	g	g	g	t	g	g	g	t	t	c	c	a	a	a	t	t	a	c	a	g	a	a	t	a	g	t	t	a	a	c	c	t	a	t	c	g	a	g	g
MH709376	2	a	a	t	c	c	g	c	a	t	g	t	a	a	g	a	a	t	a	a	t	a	t	a	g	t	g	a	a	a	t	a	g	t	c	c	g	g	g	t	g	g	g	t	t	c	c	a	a	a	t	t	a	c	a	g	a	a	t	a	g	t	t	a	a	c	c	t	a	t	c	g	a	g	g
MH709377	2	a	a	t	c	c	g	c	a	t	g	t	a	a	g	a	a	t	a	a	t	a	t	a	g	t	g	a	a	a	t	a	g	t	c	c	g	g	g	t	g	g	g	t	t	c	c	a	a	a	t	t	a	c	a	g	a	a	t	a	g	t	t	a	a	c	c	t	a	t	c	g	a	g	g
**MW316406**	2	a	a	t	c	c	g	c	a	t	g	t	a	a	g	a	a	t	a	a	t	a	t	a	g	t	g	a	a	a	t	a	g	t	c	c	g	g	g	t	g	g	g	t	t	c	c	a	a	a	t	t	a	c	a	g	a	a	t	a	g	t	t	a	a	c	c	t	a	t	c	g	a	g	g
AJ871403	3	c	a	t	c	c	g	c	a	t	g	t	a	a	g	a	a	t	a	a	t	a	t	a	g	t	g	a	a	a	t	a	g	t	c	t	g	g	g	t	g	g	g	t	t	c	c	g	a	a	t	t	a	c	a	g	a	a	t	a	g	t	c	a	a	c	c	t	a	t	c	g	c	g	g
DQ479955	3	c	a	t	c	c	g	c	a	t	g	t	a	a	g	a	a	t	a	a	t	a	t	a	g	t	g	a	a	a	t	a	g	t	c	c	g	g	g	t	g	g	g	t	t	c	c	g	a	a	t	t	a	c	a	g	a	a	t	a	g	t	c	a	a	c	c	t	a	t	c	g	c	g	g
DQ479956	3	c	a	t	c	c	g	c	a	t	g	t	a	a	g	a	a	t	a	a	t	a	t	a	g	t	g	a	a	a	c	a	g	t	c	c	g	g	g	t	g	g	g	t	t	c	c	g	a	a	t	t	a	c	a	g	a	a	t	a	g	t	c	a	a	c	c	t	a	t	c	g	c	g	g
DQ479957	3	c	a	t	c	c	g	c	a	t	g	t	a	a	g	a	a	t	a	a	t	a	t	a	g	t	g	a	a	a	t	a	g	t	c	c	g	g	g	t	g	g	g	t	t	c	c	g	a	a	t	t	a	c	a	g	a	a	t	a	g	t	c	a	a	c	c	t	a	t	c	g	c	g	g
JN704699	3	c	a	t	c	c	g	c	a	t	g	t	a	a	g	a	a	t	a	a	t	a	t	a	g	t	g	a	a	a	t	a	g	t	c	c	g	g	g	t	g	g	g	t	t	c	c	g	a	a	t	t	c	c	a	g	a	a	t	a	g	t	c	a	a	c	c	t	a	t	c	g	c	g	g
JN704700	3	c	a	t	c	c	g	t	a	t	g	t	a	a	g	a	a	t	a	a	g	a	t	a	g	t	g	a	a	a	t	a	g	t	c	c	g	g	g	t	g	g	g	t	t	c	c	g	a	a	t	t	a	c	a	t	a	a	t	a	g	t	c	a	a	c	c	t	a	t	c	g	c	g	g
JN704701	3	c	a	t	c	c	g	c	a	t	g	t	a	a	g	a	a	t	a	a	t	a	t	a	g	t	g	a	a	a	t	a	g	t	c	c	g	g	g	t	g	g	g	t	t	c	c	g	a	a	t	t	c	c	a	g	a	a	t	a	g	t	c	a	a	c	c	t	a	t	c	g	c	g	g
JN704702	3	c	a	t	c	c	g	c	a	t	g	t	a	a	g	a	a	t	a	a	g	a	t	a	g	t	g	a	a	a	t	a	g	t	c	c	g	g	g	t	g	g	g	t	t	c	c	g	a	a	t	t	a	c	a	g	a	a	t	a	g	t	c	a	a	c	c	t	a	t	c	g	c	g	g
JN704703	3	c	a	t	c	c	g	c	a	t	g	t	a	a	g	a	a	t	g	a	g	a	t	a	g	t	g	a	a	a	t	a	g	t	c	c	g	g	g	t	g	g	g	t	t	c	c	g	a	a	t	t	a	c	a	g	a	a	t	a	g	t	c	a	a	c	c	t	a	t	c	g	c	g	g
KP771891	3	c	a	t	c	c	g	c	a	t	g	t	a	a	g	a	a	t	a	a	t	a	t	a	g	t	g	a	a	a	t	a	g	t	c	c	g	g	g	t	g	g	g	t	t	c	c	g	a	a	t	t	c	c	a	g	a	a	t	a	g	t	c	a	a	c	c	t	a	t	c	g	c	g	g
KP771893	3	c	a	t	c	c	g	c	a	t	g	t	a	a	g	a	a	t	a	a	t	a	t	a	g	t	g	a	a	a	t	a	g	t	c	c	g	g	g	t	g	g	g	t	t	c	c	g	a	a	t	t	a	c	a	g	a	a	t	a	g	t	c	a	a	c	c	t	a	t	c	g	c	g	g
KP771895	3	c	a	t	c	c	g	c	a	t	g	t	a	a	g	a	a	t	a	a	t	a	t	a	g	t	g	a	a	a	t	a	g	t	c	t	g	g	g	t	g	g	g	t	t	c	c	g	a	a	t	t	a	c	a	g	a	a	t	a	g	t	c	a	a	c	c	t	a	t	c	g	c	g	g
KP771896	3	c	a	t	c	c	g	c	a	t	g	t	a	a	g	a	a	t	a	a	t	a	t	a	g	t	g	a	a	a	t	a	g	t	c	c	g	g	g	t	g	g	g	t	t	c	c	g	a	a	t	t	a	c	a	g	a	a	t	a	g	t	c	a	a	c	c	t	a	g	c	g	c	g	g
KP771897	3	c	a	t	c	c	g	c	a	t	g	t	a	a	g	a	a	t	a	a	t	a	t	a	g	t	g	a	a	a	t	a	g	t	c	c	g	g	g	t	g	g	g	t	t	c	c	g	a	a	t	t	a	c	a	g	a	a	t	a	g	t	c	a	a	c	c	t	a	t	c	g	c	g	g
KP771899	3	c	a	t	c	c	g	c	a	t	g	t	a	a	g	a	a	t	a	a	t	a	t	a	g	t	g	a	a	a	t	a	g	t	c	t	g	g	g	t	g	g	g	t	t	c	c	g	a	a	t	t	a	c	a	g	a	a	t	a	g	t	c	a	a	c	c	t	a	t	c	g	c	g	g
KP771903	3	c	a	t	c	c	g	c	a	t	g	t	a	a	g	a	a	t	a	a	t	a	t	a	g	t	g	a	a	a	t	a	g	t	c	c	g	g	g	t	g	g	g	t	t	c	c	g	a	a	t	t	c	c	a	g	a	a	t	a	g	t	c	a	a	c	c	t	a	t	c	g	c	g	g
KP771907	3	c	a	t	c	c	g	c	a	t	g	t	a	a	g	a	a	t	a	a	t	a	t	a	g	t	g	a	a	a	t	a	g	t	c	t	g	g	g	t	g	g	g	t	t	c	c	g	a	a	t	t	a	c	a	g	g	a	t	a	g	t	c	a	a	c	c	t	a	t	c	g	c	g	g
KP771910	3	c	a	t	c	c	g	c	a	t	g	t	a	a	g	a	a	t	a	a	t	a	t	a	g	t	g	a	a	a	t	a	g	t	c	c	g	g	g	t	g	g	g	t	t	c	c	g	a	a	t	t	c	c	a	g	a	a	t	a	g	t	c	a	a	c	c	t	a	t	c	g	c	g	g
KP771915	3	c	a	t	c	c	g	c	a	t	g	t	a	a	g	a	a	t	a	a	t	a	t	a	t	t	g	a	a	a	t	a	g	t	c	c	g	g	g	t	g	g	g	t	t	c	c	g	a	a	t	t	a	c	a	g	a	a	t	a	g	t	c	a	a	c	c	t	a	t	c	g	c	g	g
KP771916	3	c	a	t	c	c	g	c	a	t	g	t	a	a	g	a	a	t	a	a	t	a	t	a	g	t	g	a	a	a	c	a	g	t	c	c	g	g	g	t	g	g	g	t	t	c	c	g	a	a	t	t	a	c	a	g	a	a	t	a	g	t	c	a	a	c	c	t	a	t	c	g	c	g	g
KP771921	3	c	a	t	c	c	g	c	a	t	g	t	a	a	g	a	a	t	a	a	t	a	t	a	g	t	g	a	a	a	t	a	g	t	c	c	g	g	g	t	g	g	g	t	t	c	c	g	a	a	t	t	c	c	a	g	a	a	t	a	g	t	c	a	a	c	c	t	a	t	c	g	c	g	g
KP771923	3	c	a	t	c	c	g	c	a	t	g	t	a	a	g	a	a	t	a	a	t	a	t	a	g	t	g	a	a	a	t	a	g	t	c	c	g	g	g	t	g	g	g	t	t	c	c	g	a	a	t	t	a	c	a	g	a	a	c	a	g	t	c	a	a	c	c	t	a	t	c	g	c	g	g
KP771889	3	a	a	t	c	c	g	c	a	t	g	t	a	a	g	a	a	t	a	a	t	a	t	a	t	t	g	a	a	a	t	a	g	t	c	c	g	g	g	t	g	g	g	t	t	c	c	g	a	a	t	t	a	c	a	g	a	a	t	a	g	t	c	a	a	c	c	t	a	t	c	g	c	g	g
KU926311	3	c	a	t	c	c	g	c	g	t	g	t	a	a	g	a	a	t	a	a	t	a	t	a	g	t	g	a	a	a	c	a	g	t	c	c	g	g	g	t	g	g	g	t	t	c	c	g	a	a	t	t	a	t	a	g	a	a	t	a	g	t	c	a	a	c	c	t	a	t	c	g	c	g	g
JQ911599	3	c	a	t	c	c	g	c	a	t	g	t	a	a	g	a	a	t	a	a	g	g	t	a	g	t	g	a	a	a	t	a	g	t	c	c	g	g	g	t	t	g	g	t	t	c	c	g	a	a	t	c	a	c	a	g	a	a	t	a	g	t	c	a	a	c	c	t	a	t	c	g	c	g	g
MH709317	3	c	a	t	c	c	g	c	a	t	a	t	a	a	g	a	a	t	a	a	t	a	t	a	g	t	g	a	a	a	t	a	g	t	c	c	g	g	g	t	g	g	g	t	t	a	c	g	a	a	t	t	a	c	a	g	a	a	t	a	g	t	c	a	a	c	c	t	a	t	c	g	c	g	g
MH709346	3	c	a	t	c	c	g	c	a	t	a	t	a	a	g	a	a	t	a	a	t	a	t	a	g	t	g	a	a	a	t	a	g	t	c	c	g	g	g	t	g	g	g	t	t	a	c	g	a	a	t	t	a	c	a	g	a	a	t	a	g	t	c	a	a	c	c	t	a	t	c	g	c	g	g
MH709375	3	c	a	t	c	c	g	c	a	t	a	t	a	a	g	a	a	t	a	a	t	a	t	a	g	t	g	a	a	a	t	a	g	t	c	c	g	g	g	t	g	g	g	t	t	a	c	g	a	a	t	t	a	c	a	g	a	a	t	a	g	t	c	a	a	c	c	t	a	t	c	g	c	g	g
DQ452050	4	a	a	t	c	c	a	c	a	t	g	t	a	a	g	a	a	t	a	a	t	a	t	a	g	t	g	a	a	a	t	a	g	t	c	c	a	a	g	t	g	g	g	t	t	c	c	a	a	g	t	t	a	c	g	g	a	a	t	a	g	t	t	a	a	c	c	t	a	t	c	g	a	g	g
DQ479960	4	a	a	t	c	c	g	c	a	t	g	t	a	a	g	a	a	t	a	a	t	a	t	a	g	t	g	a	a	a	t	a	g	t	c	c	g	a	g	t	g	g	g	t	t	c	c	a	a	g	t	t	a	c	a	g	a	a	t	a	g	t	t	a	a	c	c	t	a	t	c	g	a	g	g
KP771898	4	a	a	t	c	c	a	c	a	t	g	t	a	a	g	a	a	t	a	a	t	a	t	a	g	t	g	a	a	a	t	a	g	t	c	c	g	a	g	t	g	g	g	t	t	c	c	a	a	g	t	t	a	c	a	g	a	a	t	a	g	t	t	a	a	c	c	t	a	t	c	g	a	g	g
MH709316	4	c	a	t	c	c	a	c	a	t	g	t	a	a	a	a	a	t	a	a	t	a	t	a	g	t	g	a	a	a	t	a	g	t	c	c	g	a	g	t	g	g	g	t	g	c	c	a	a	g	t	t	a	c	a	g	a	a	t	c	g	t	c	a	a	c	c	t	a	t	c	g	a	g	g
MH709318	4	a	a	t	c	c	g	c	a	t	g	t	a	a	g	a	a	t	a	a	t	a	t	a	g	t	g	a	a	a	t	a	g	t	c	c	g	a	g	t	g	g	g	t	t	c	c	a	a	g	t	t	a	c	a	g	a	a	t	a	g	t	t	a	a	c	c	t	a	t	c	g	a	g	g
MH709322	4	a	a	t	c	c	g	c	a	t	g	t	a	a	g	a	a	t	a	a	t	a	t	a	g	t	g	a	a	a	t	a	g	t	c	c	g	a	g	t	g	g	g	t	t	c	c	a	a	g	t	t	a	c	a	g	a	a	t	a	g	t	t	a	a	c	c	t	a	t	c	g	a	g	g
MH709323	4	a	a	t	c	c	g	c	a	t	g	t	a	a	g	a	a	t	a	a	t	a	t	a	g	t	g	a	a	a	t	a	g	t	c	c	g	a	g	t	g	g	g	t	t	c	c	a	a	g	t	t	a	c	a	g	a	a	t	a	g	t	t	a	a	c	c	t	a	t	c	g	a	g	g
MH709326	4	a	a	t	c	c	g	c	a	t	g	t	a	a	g	a	a	t	a	a	t	a	t	a	g	t	g	a	a	a	t	a	g	t	c	c	g	a	g	t	g	g	g	t	t	c	c	a	a	g	t	t	a	c	a	g	a	a	t	a	g	t	t	a	a	c	c	t	a	t	c	g	a	g	g
MH709341	4	a	a	t	c	c	a	c	a	t	g	t	a	a	g	a	a	t	a	a	t	a	t	a	g	t	g	a	a	a	t	a	g	t	c	c	g	a	g	t	g	g	g	t	t	c	c	a	a	g	t	t	a	c	a	g	a	a	t	a	g	t	t	a	a	c	c	t	a	t	c	g	a	g	g
MH709357	4	a	a	t	c	c	g	c	a	t	g	t	a	a	g	a	a	t	a	a	t	a	t	a	g	t	g	a	a	a	t	a	g	t	c	c	g	a	g	t	g	g	g	t	t	c	c	a	a	g	t	t	a	c	a	g	a	a	t	a	g	t	t	a	a	c	c	t	a	t	c	g	a	g	g
MH709364	4	a	a	t	c	c	g	c	a	t	g	t	a	a	g	a	a	t	a	a	t	a	t	a	g	t	g	a	a	a	t	a	g	t	c	c	g	a	g	t	g	g	g	t	t	c	c	a	a	g	t	t	a	c	a	g	a	a	t	a	g	t	t	a	a	c	c	t	a	t	c	g	a	g	g
MH709370	4	a	a	t	c	c	g	c	a	t	g	t	a	a	g	a	a	t	a	a	t	a	t	a	g	t	g	a	a	a	t	a	g	t	c	c	g	a	g	t	g	g	g	t	t	c	c	a	a	g	t	t	a	c	a	g	a	a	t	a	g	t	t	a	a	c	c	t	a	t	c	g	a	g	g
MH709371	4	a	a	t	c	c	g	c	a	t	g	t	a	a	g	a	a	t	a	a	t	a	t	a	g	t	g	a	a	a	t	a	g	t	c	c	g	a	g	t	g	g	g	t	t	c	c	a	a	g	t	t	a	c	a	g	a	a	t	a	g	t	t	a	a	c	c	t	a	t	c	g	a	g	g
MH709372	4	a	a	t	c	c	g	c	a	t	g	t	a	a	g	a	a	t	a	a	t	a	t	a	g	t	g	a	a	a	t	a	g	t	c	c	g	a	g	t	g	g	g	t	t	c	c	a	a	g	t	t	a	c	a	g	a	a	t	a	g	t	t	a	a	c	c	t	a	t	c	a	a	g	g
DQ457052	5	c	a	t	c	c	g	c	a	t	g	t	c	a	g	a	a	t	a	a	t	a	t	a	g	t	g	a	a	a	t	a	g	g	c	c	g	g	g	t	g	g	g	t	t	c	c	a	a	g	t	t	a	c	a	g	a	a	t	a	t	t	c	a	a	c	c	t	a	t	c	g	c	g	g
JN704704	5	c	a	t	c	c	g	c	a	t	g	t	c	a	g	a	a	t	a	a	t	a	t	a	g	t	g	a	a	a	t	a	g	t	c	c	g	g	g	t	g	g	g	t	t	c	c	a	a	g	t	t	a	c	a	g	a	a	t	a	g	t	c	a	a	c	c	t	a	t	c	g	c	g	g
JN704705	5	c	a	t	c	c	g	c	a	t	g	t	a	a	g	a	a	t	a	a	t	a	t	a	g	t	g	a	a	a	t	a	g	t	c	c	g	g	g	t	g	g	g	t	t	c	c	a	a	g	t	t	a	c	a	g	a	a	t	a	g	t	c	a	a	c	c	t	a	t	c	g	c	g	g
JN704706	5	c	a	t	c	c	g	c	a	t	g	t	c	a	g	a	a	t	a	a	t	a	t	a	g	t	g	a	a	a	t	a	g	t	c	c	g	g	g	t	g	g	g	g	t	c	c	a	a	g	t	t	a	c	a	g	a	a	t	a	g	t	c	a	a	c	c	t	a	t	c	g	c	g	g
JN704707	5	c	a	t	c	c	g	c	a	t	g	t	c	a	g	a	a	t	a	a	t	a	c	a	g	t	g	a	a	a	t	a	g	t	c	c	g	g	g	t	g	g	g	t	t	c	c	a	a	g	t	t	a	c	a	g	a	a	t	a	g	t	c	a	a	c	c	t	a	t	c	g	c	g	g
JN704708	5	c	a	t	c	c	g	c	a	t	g	t	a	a	g	a	a	t	a	a	t	a	t	a	g	t	g	a	a	a	t	a	g	t	c	c	g	g	g	t	g	g	g	t	t	c	c	a	a	g	t	t	a	c	a	g	a	a	t	a	g	t	c	a	a	c	c	t	a	t	c	g	c	g	g
KP771904	5	c	a	t	c	c	g	c	a	t	g	t	c	a	g	a	a	t	a	a	t	a	t	a	g	t	g	a	a	a	t	a	g	t	c	c	g	g	g	t	g	g	g	t	t	c	c	a	a	g	t	t	a	c	a	g	a	a	t	a	g	t	c	a	a	c	c	t	a	t	c	g	c	g	g
KP771905	5	c	a	t	c	c	g	c	a	t	g	t	c	a	g	a	a	t	a	a	t	a	t	a	g	t	g	a	a	a	t	a	g	t	c	c	g	g	g	t	g	g	g	t	t	c	c	a	a	g	t	g	a	c	a	g	a	a	t	a	g	t	c	a	a	c	c	t	a	t	c	g	c	g	g
KP771906	5	c	a	t	c	c	g	c	a	t	g	t	c	a	g	a	a	t	a	a	t	a	t	a	g	t	g	a	a	a	t	a	g	t	c	c	g	g	g	t	g	g	g	t	t	c	c	a	a	g	t	g	a	c	a	g	a	a	t	a	g	t	c	a	a	c	c	t	a	t	c	g	c	g	g
KP771919	5	c	a	t	c	c	g	c	a	t	g	t	c	a	g	a	a	t	a	a	t	a	t	a	g	t	g	a	a	a	t	a	g	t	c	c	g	g	g	t	g	g	g	t	t	c	c	a	a	g	t	t	a	c	a	g	a	a	t	a	g	t	c	a	a	c	c	t	a	t	c	g	c	g	g
KP771922	5	c	a	t	c	c	g	c	a	t	g	t	c	a	g	a	a	t	a	a	t	a	t	a	g	t	g	a	a	a	t	a	g	t	c	c	g	g	g	t	g	g	g	t	t	c	c	a	a	g	t	t	a	c	a	g	a	a	t	a	g	t	c	a	a	c	c	t	a	t	c	g	c	g	g
KP771924	5	c	a	t	c	c	g	c	a	t	g	t	c	a	g	a	a	t	a	a	t	a	t	a	g	t	g	a	a	a	t	a	g	t	c	c	g	g	g	t	g	g	g	t	t	c	c	a	a	g	t	t	a	c	a	g	a	a	t	a	g	t	c	a	a	c	c	t	a	t	c	g	c	g	g
KM355696	5	c	a	t	c	c	g	c	a	t	g	t	c	a	g	a	a	t	a	a	t	a	t	a	g	t	g	a	a	a	t	a	g	t	c	c	g	g	g	t	g	g	g	t	t	c	c	a	a	g	t	t	a	c	a	g	a	a	t	a	g	t	c	a	a	c	c	t	a	t	c	g	c	g	g
KM355697	5	c	a	t	c	c	g	c	a	t	g	t	c	a	g	a	a	t	a	a	t	a	t	a	g	t	g	a	a	a	t	a	g	t	c	c	g	g	g	t	g	g	g	t	t	c	c	a	a	g	t	t	a	c	a	g	a	a	t	a	g	t	c	a	a	c	c	t	a	t	c	g	c	g	g
KM355698	5	c	a	t	c	c	g	c	a	t	g	t	c	a	g	a	a	t	a	a	t	a	t	a	g	t	g	a	a	a	t	a	g	t	c	c	g	g	g	t	g	g	g	t	t	c	c	a	a	g	t	t	a	c	a	g	a	a	t	a	g	t	c	a	a	c	c	t	a	t	c	g	c	g	g
KM355699	5	c	a	t	c	c	g	c	a	t	g	t	c	a	g	a	a	t	a	a	t	a	t	a	g	t	g	a	a	a	t	a	g	t	c	c	g	g	g	t	g	g	g	t	t	c	c	a	a	g	t	t	a	c	a	g	a	a	t	a	g	t	c	a	a	c	c	t	a	t	c	g	c	g	g
KM355701	5	c	a	t	c	c	g	c	a	t	g	t	c	a	g	a	a	t	a	a	t	a	t	a	g	t	g	a	a	a	t	a	g	t	c	c	g	g	g	t	g	g	g	t	t	c	c	a	a	g	t	t	a	c	a	g	a	a	t	a	g	t	c	a	a	c	c	t	a	t	c	g	c	g	g
KM355702	5	c	a	t	c	c	g	c	a	t	g	t	c	a	g	a	a	t	a	a	t	a	t	a	g	t	g	a	a	a	t	a	g	t	c	c	g	g	g	t	g	g	g	t	t	c	c	a	a	g	t	t	a	c	a	g	a	a	t	a	g	t	c	a	a	c	c	t	a	t	c	g	c	g	g
KM355703	5	c	a	t	c	c	g	c	a	t	g	t	c	a	g	a	a	t	a	a	t	a	t	a	g	t	g	a	a	a	t	a	g	t	c	c	g	g	g	t	g	g	g	t	t	c	c	a	a	g	t	t	a	c	a	g	a	a	t	a	g	t	c	a	a	c	c	t	a	t	c	g	c	g	g
KM355704	5	c	a	t	c	c	g	c	a	t	g	t	c	a	g	a	a	t	a	a	t	a	t	a	g	t	g	a	a	a	t	a	g	t	c	c	g	g	g	t	g	g	g	t	t	c	c	a	a	g	t	t	a	c	a	g	a	a	t	a	g	t	c	a	a	c	c	t	a	t	c	g	c	g	g
KM355705	5	c	a	t	c	c	g	c	a	t	g	t	c	a	g	a	a	t	a	a	t	a	t	a	g	t	g	a	a	a	t	a	g	t	c	c	g	g	g	t	g	g	g	t	t	c	c	a	a	g	t	t	a	c	a	g	a	a	t	a	g	t	c	a	a	c	c	t	a	t	c	g	c	g	g
KM355706	5	c	a	t	c	c	g	c	a	t	g	t	c	a	g	a	a	t	a	a	t	a	t	a	g	t	g	a	a	a	t	a	g	t	c	c	g	g	g	t	g	g	g	t	t	c	c	a	a	g	t	t	a	c	a	g	a	a	t	a	g	t	c	a	a	c	c	t	a	t	c	g	c	g	g
KM355707	5	c	a	t	c	c	g	c	a	t	g	t	c	a	g	a	a	t	a	a	t	a	t	a	g	t	g	a	a	a	t	a	g	t	c	c	g	g	g	t	g	g	g	t	t	c	c	a	a	g	t	t	a	c	a	g	a	a	t	a	g	t	c	a	a	c	c	t	a	t	c	g	c	g	g
KM355708	5	c	a	t	c	c	g	c	a	t	g	t	c	a	g	a	a	t	a	a	t	a	t	a	g	t	g	a	a	a	t	a	g	t	c	c	g	g	g	t	g	g	g	t	t	c	c	a	a	g	t	t	a	c	a	g	a	a	t	a	g	t	c	a	a	c	c	t	a	t	c	g	c	g	g
KM355709	5	c	a	t	c	c	g	c	a	t	g	t	c	a	g	a	a	t	a	a	t	a	t	a	g	t	g	a	a	a	t	a	g	t	c	c	g	g	g	t	g	g	g	t	t	c	c	a	a	g	t	t	a	c	a	g	a	a	t	a	g	t	c	a	a	c	c	t	a	t	c	g	c	g	g
KM355711	5	c	a	t	c	c	g	c	a	t	g	t	c	a	g	a	a	t	a	a	t	a	t	a	g	t	g	a	a	a	t	a	g	t	c	c	g	g	g	t	g	g	g	t	t	c	c	a	a	g	t	t	a	c	a	g	a	a	t	a	g	t	c	a	a	c	c	t	a	t	c	g	c	g	g
KM355712	5	c	a	t	c	c	g	c	a	t	g	t	c	a	g	a	a	t	a	a	t	a	t	a	g	t	g	a	a	a	t	a	g	t	c	c	g	g	g	t	g	g	g	t	t	c	c	a	a	g	t	t	a	c	a	g	a	a	t	a	g	t	c	a	a	c	c	t	a	t	c	g	c	g	g
KY037797	5	[illegible]	[illegible]	[illegible]	[illegible]	[illegible]	[illegible]	[illegible]	[illegible]	[illegible]	[illegible]	[illegible]	[illegible]	[illegible]	[illegible]	[illegible]	[illegible]	[illegible]	[illegible]	[illegible]	[illegible]	[illegible]	[illegible]	[illegible]	[illegible]	[illegible]	[illegible]	[illegible]	[illegible]	[illegible]	[illegible]	[illegible]	[illegible]	[illegible]	[illegible]	[illegible]	[illegible]	[illegible]	[illegible]	[illegible]	[illegible]	[illegible]	[illegible]	[illegible]	[illegible]	[illegible]	[illegible]	[illegible]	[illegible]	[illegible]	[illegible]	[illegible]	[illegible]	[illegible]	[illegible]	[illegible]	[illegible]	[illegible]	[illegible]	[illegible]	[illegible]	[illegible]	[illegible]	[illegible]	[illegible]	[illegible]	[illegible]	[illegible]	[illegible]	[illegible]	[illegible]	[illegible]	[illegible]	[illegible]	[illegible]

Supplementary Table 3: 2880 SN

GB-ID	Clade markers				9																																	4	1																																					
	Position in Dumas	82141	82225	82294	82300	82495	82533	82601	82645	82663	82700	82765	82880	82882	82896	82906	83085	83129	83258	83303	83396	83455	83662	83722	83785	83845	83888	84001	84004	84010	84080	84084	84091	84433	84515	84547	84553	84611	84616	84655	84728	84810	84822	84834	84864	84970	84979	84983	85013	85041	85059	85122	85180	85181	85188	85272	85314	85377	85398	85431	85440	85560	85563	85594	85621	85686	85805	85957	85974	86018	86079	86115	86170	86199	86225
MH499467	5	c	a	t	c	c	g	c	a	t	g	t	c	a	g	a	a	t	a	a	t	a	t	a	g	t	g	a	a	a	t	a	g	t	c	c	g	g	g	t	g	g	g	t	t	c	c	a	a	g	t	t	a	c	a	g	a	a	t	a	g	t	c	a	a	c	c	t	a	t	c	g	c	g	g
MH499469	5	c	a	t	c	c	g	c	a	t	g	t	c	a	g	a	a	t	a	a	t	a	t	a	g	t	g	a	a	a	t	a	g	t	c	c	g	g	g	t	g	g	g	t	t	c	c	a	a	g	t	t	a	c	a	g	a	a	t	a	g	t	c	a	a	c	c	t	a	t	c	g	c	g	g
MH709321	5	c	a	t	c	c	g	c	a	t	g	t	c	a	g	a	a	t	a	a	t	a	t	a	g	t	g	a	a	a	t	a	g	t	c	c	g	g	g	t	g	g	g	t	t	c	c	a	a	g	t	t	a	c	a	g	a	a	t	a	g	t	c	a	a	c	c	t	a	t	c	g	c	g	g
MH709324	5	c	a	t	c	c	g	c	a	t	g	t	c	a	g	a	a	t	a	a	t	a	t	a	g	t	g	a	a	a	t	a	g	t	c	c	g	g	g	t	g	g	g	t	t	c	c	a	a	g	t	t	a	c	a	g	a	a	t	a	g	t	c	a	a	c	c	t	a	t	c	g	c	g	g
MH709325	5	c	a	t	c	c	g	c	a	t	g	t	c	a	g	a	a	t	a	a	t	a	t	a	g	t	g	a	a	a	t	a	g	t	c	c	g	g	g	t	g	g	g	t	t	c	c	a	a	g	t	t	a	c	a	g	a	a	t	a	g	t	c	a	a	c	c	t	a	t	c	g	c	g	g
MH709327	5	c	a	t	c	c	g	c	a	t	g	t	c	a	g	a	a	t	a	a	t	a	t	a	g	t	g	a	a	a	t	a	g	t	c	c	g	g	g	t	g	g	g	t	t	c	c	a	a	g	t	t	a	c	a	g	a	a	t	a	g	t	c	a	a	c	c	t	a	t	c	g	c	g	g
MH709328	5	c	a	t	c	c	g	c	a	t	g	t	c	a	g	a	a	t	a	a	t	a	t	a	g	t	g	a	a	a	t	a	g	t	c	c	g	g	g	t	g	g	g	t	t	c	c	a	a	g	t	t	a	c	a	g	a	a	t	a	g	t	c	a	a	c	c	t	a	t	c	g	c	g	g
MH709330	5	c	a	t	c	c	g	c	a	t	g	t	c	a	g	a	a	t	a	a	t	a	t	a	g	t	g	a	a	a	t	a	g	t	c	c	g	g	g	t	g	g	g	t	t	c	c	a	a	g	t	t	a	c	a	g	a	a	t	a	g	t	c	a	a	c	c	t	a	t	c	g	c	g	g
MH709332	5	c	a	t	c	c	g	c	a	t	g	t	c	a	g	a	a	t	a	a	t	a	t	a	g	t	g	a	a	a	t	a	g	t	c	c	g	g	g	t	g	g	g	t	t	c	c	a	a	g	t	t	a	c	a	g	a	a	t	a	g	t	c	a	a	c	c	t	a	t	c	g	c	g	g
MH709334	5	c	a	t	c	c	g	c	a	t	g	t	c	a	g	a	a	t	a	a	t	a	t	a	g	t	g	a	a	a	t	a	g	t	c	c	g	g	g	t	g	g	g	t	t	c	c	a	a	g	t	t	a	c	a	g	a	a	t	a	g	t	c	a	a	c	c	t	a	t	t	g	c	g	g
MH709335	5	c	a	t	c	c	g	c	a	t	g	t	c	a	g	a	a	t	a	a	t	a	t	a	g	t	g	a	a	a	t	a	g	g	c	c	g	g	g	t	g	g	g	t	t	c	c	a	a	g	t	t	a	c	a	g	a	a	t	a	t	t	c	a	a	c	c	t	a	t	c	g	c	g	g
MH709336	5	c	a	t	c	c	g	c	a	t	g	t	c	a	g	a	a	t	a	a	t	a	t	a	g	t	g	a	a	a	t	a	g	t	c	c	g	g	g	t	g	g	g	t	t	c	c	a	a	g	t	t	a	c	a	g	a	a	t	a	g	t	c	a	a	c	c	t	a	t	c	g	c	g	g
MH709338	5	c	a	t	c	c	g	c	a	t	g	t	c	a	g	a	a	t	a	a	t	a	t	a	g	t	g	a	a	a	t	a	g	t	c	c	g	g	g	t	g	g	g	t	t	c	c	a	a	g	t	t	a	c	a	g	a	a	t	a	g	t	c	a	a	c	c	t	a	t	c	g	c	g	g
MH709340	5	c	a	t	c	c	g	c	a	t	g	t	c	a	g	a	a	t	a	a	t	a	t	a	g	t	g	a	a	g	t	a	g	t	c	c	g	g	g	t	g	g	g	t	t	c	c	a	a	g	t	t	a	c	a	g	a	a	t	a	g	t	c	a	a	c	c	t	a	t	c	g	c	g	g
MH709342	5	c	a	t	c	c	g	c	a	t	g	t	c	a	g	a	a	t	a	a	t	a	t	a	g	t	g	a	a	a	t	a	g	t	c	c	g	g	g	t	g	g	g	t	t	c	c	a	a	g	t	t	a	c	a	g	a	a	t	a	g	t	c	a	a	c	c	t	a	t	c	g	c	g	g
MH709343	5	c	a	t	c	c	g	c	a	t	g	t	c	a	g	a	a	t	a	a	t	a	t	a	g	t	g	a	a	a	t	a	g	t	c	c	g	g	g	t	g	g	g	t	t	c	c	a	a	g	t	t	a	c	a	g	a	a	t	a	g	t	c	a	a	c	c	t	a	t	c	g	c	g	g
MH709345	5	c	a	t	c	c	g	c	a	t	g	t	c	a	g	a	a	t	a	a	t	a	t	a	g	t	g	a	a	a	t	a	g	g	c	c	g	g	g	t	g	g	g	t	t	c	c	a	a	g	t	t	a	c	a	g	a	a	t	a	t	t	c	a	a	c	c	t	a	t	c	g	c	g	g
MH709347	5	c	a	t	c	c	g	c	a	t	g	t	c	a	g	a	a	t	a	a	t	a	t	a	g	t	g	a	a	a	t	a	g	t	c	c	g	g	g	t	g	g	g	t	t	c	c	a	a	g	t	t	a	c	a	g	a	a	t	a	t	t	c	a	a	c	c	t	a	t	c	g	c	g	g
MH709349	5	c	a	t	c	c	g	c	a	t	g	t	c	a	g	a	c	t	a	a	t	a	t	a	g	t	g	a	a	a	t	a	g	t	c	c	g	g	g	t	g	g	g	t	t	c	c	a	a	g	t	t	a	c	a	g	a	a	t	a	g	t	c	a	a	c	c	t	a	t	c	g	c	g	g
MH709350	5	c	a	t	c	c	g	c	a	t	g	t	c	a	g	a	a	t	a	a	t	a	t	a	g	t	g	a	a	a	t	a	g	t	c	c	g	g	g	t	g	g	g	t	t	c	c	a	a	g	t	t	a	c	a	g	a	a	t	a	g	t	c	a	a	c	c	t	a	t	c	g	c	g	g
MH709351	5	c	a	t	c	c	g	c	a	t	g	t	c	a	g	a	a	t	a	a	t	a	t	g	g	t	g	a	a	g	t	a	g	t	c	c	g	g	g	t	g	g	g	t	t	c	c	a	a	g	t	t	a	c	a	g	a	a	t	a	g	t	c	a	a	c	c	t	a	t	c	g	c	g	g
MH709353	5	c	a	t	c	c	g	c	a	t	g	t	c	a	g	a	a	t	a	a	t	a	t	a	g	t	g	a	a	a	t	a	g	g	c	c	g	g	g	t	g	g	g	t	t	c	c	a	a	g	t	t	a	c	a	g	a	a	t	a	t	t	c	a	a	c	c	t	a	t	c	g	c	g	g
MH709358	5	c	a	t	c	c	g	c	a	t	g	t	c	a	g	a	a	t	a	a	t	a	t	a	g	t	g	a	a	a	t	a	g	t	c	c	g	g	g	t	g	g	g	t	t	c	c	a	a	g	t	t	a	c	a	g	a	a	t	a	g	t	c	a	a	c	c	t	a	t	c	g	c	g	g
MH709360	5	c	a	t	c	c	g	c	a	t	g	t	c	a	g	a	a	t	a	a	t	a	t	a	g	t	g	a	a	a	t	a	g	t	c	c	g	g	g	t	g	g	g	t	t	c	c	a	a	g	t	t	a	c	a	g	a	a	t	a	g	t	c	a	a	c	c	t	a	t	c	g	c	g	g
MH709362	5	c	a	t	c	c	g	c	a	t	g	t	c	a	g	a	a	t	a	a	t	a	t	a	g	t	g	a	a	a	t	a	g	g	c	c	g	g	g	t	g	g	g	t	t	c	c	a	a	g	t	t	a	c	a	g	a	a	t	a	t	t	c	a	a	c	c	t	a	t	c	g	c	g	g
MH709365	5	c	a	t	c	c	g	c	a	t	g	t	c	a	g	a	a	t	a	a	t	a	t	a	g	t	g	a	a	a	t	a	g	t	c	c	g	g	g	t	g	g	g	t	t	c	c	a	a	g	t	t	a	c	a	g	a	a	t	a	g	t	c	a	a	c	c	t	a	t	c	g	c	g	g
MH709373	5	c	a	t	c	c	g	c	a	t	g	t	c	a	g	a	a	t	a	a	t	a	t	a	g	t	g	a	a	a	t	a	g	t	c	c	g	g	g	t	g	g	g	t	t	c	c	a	a	g	t	t	a	c	a	g	a	a	t	a	g	t	c	a	a	c	c	t	a	t	c	g	c	g	g
MH709374	5	c	a	t	c	c	g	c	a	t	g	t	c	a	g	a	a	t	a	a	t	a	t	a	g	t	g	a	a	a	t	a	g	t	c	c	g	g	g	t	g	g	g	t	t	c	c	a	a	g	t	t	a	c	a	g	a	a	t	a	t	t	c	a	a	c	c	t	a	t	c	g	c	g	g
KC112914	6	c	a	t	c	c	g	c	a	t	g	t	a	a	g	a	a	t	g	a	t	a	t	a	g	t	g	a	a	a	t	a	g	t	c	c	g	g	g	t	g	g	g	t	t	c	c	g	a	a	t	t	a	c	a	g	a	a	t	a	g	t	c	a	a	c	c	t	a	t	c	g	c	g	g
KP771914	6	c	a	t	c	c	g	c	a	t	g	t	a	a	g	a	a	t	g	a	t	a	t	a	g	t	g	a	a	a	t	a	g	t	c	c	g	g	g	t	g	g	g	t	t	c	c	g	a	a	t	t	a	c	a	g	a	a	t	a	g	t	c	a	a	c	c	t	a	t	c	g	c	g	g
KP702725	6	c	a	t	c	c	g	c	a	t	g	t	a	a	g	a	a	t	g	a	t	a	t	a	g	t	g	a	a	a	t	a	g	t	c	c	g	g	g	t	g	g	g	t	t	c	c	g	a	a	t	t	a	c	a	g	a	a	t	a	g	t	c	a	a	c	c	t	a	t	c	g	c	g	g
KP771890	9	c	a	c	t	c	g	c	a	t	g	t	a	a	g	a	a	g	a	a	t	a	t	a	g	t	g	a	a	a	t	g	g	t	c	c	g	g	g	t	g	g	g	t	t	c	c	g	a	a	t	t	a	c	a	g	a	a	t	a	g	t	c	a	a	c	c	t	a	t	c	g	c	g	g
JN704710	9	c	a	t	t	c	g	c	a	t	g	t	a	a	g	a	a	t	a	a	t	a	t	a	g	t	g	a	a	a	t	a	g	t	c	c	g	g	g	t	g	g	g	t	t	c	c	g	a	a	t	t	a	c	a	g	a	a	t	a	g	t	c	a	a	c	c	t	a	t	c	g	c	g	g
KY037798	9	c	a	t	t	c	g	c	a	t	g	t	a	a	g	a	a	g	a	a	t	a	t	a	g	t	g	a	a	a	t	g	g	t	c	c	g	g	g	t	g	g	g	t	t	c	c	g	a	a	t	t	a	c	a	g	a	a	t	a	g	t	c	a	a	c	c	t	a	t	c	g	c	g	g
MH709312	9	c	a	t	t	c	g	c	a	t	g	t	a	a	g	a	a	g	a	a	t	a	t	a	g	t	g	a	a	a	t	g	g	t	c	c	g	g	g	t	g	g	g	t	t	c	c	g	a	a	t	t	a	c	a	g	a	a	t	a	g	t	c	a	a	c	c	t	a	t	c	g	c	g	g
MH709352	9	c	a	t	t	c	g	c	a	t	g	t	a	a	g	a	a	g	a	a	t	a	t	a	g	t	g	a	a	a	t	g	g	t	c	c	g	g	g	t	g	g	g	t	t	c	c	g	a	a	t	t	a	c	a	g	a	a	t	a	g	t	c	a	a	c	c	t	a	t	c	g	c	g	g
AB097933	Vac	a	a	t	c	c	g	c	a	t	g	t	a	a	g	a	a	t	a	a	t	a	t	a	g	t	g	a	a	a	t	a	g	t	c	c	g	g	g	t	g	g	g	t	t	c	c	a	a	a	t	t	a	c	a	g	a	a	t	a	g	t	t	a	a	c	c	t	a	t	c	g	a	g	g
JN704698	Vac	a	a	t	c	c	g	c	a	t	g	t	a	a	g	a	a	t	a	a	t	a	t	a	g	t	g	a	a	a	t	a	g	t	c	c	g	g	g	t	g	g	g	t	t	c	c	a	a	a	t	t	a	c	a	g	a	a	t	a	g	t	t	a	a	c	c	t	a	t	c	g	c	g	g
JN704697	Vac	a	g	t	c	c	g	c	a	t	g	t	a	a	g	a	a	t	a	a	t	a	t	a	g	t	g	a	a	a	t	a	a	t	c	c	g	g	g	t	g	g	g	t	t	c	c	a	a	a	t	t	a	c	a	g	a	a	t	a	g	t	t	g	a	c	c	t	a	t	c	g	c	g	g
DQ008355	Vac	a	a	t	c	c	g	c	a	t	g	t	a	a	g	a	a	t	a	a	t	a	t	a	g	t	g	a	a	a	t	a	g	t	c	c	g	g	g	t	g	g	g	t	t	c	c	a	a	a	t	t	a	c	a	g	a	a	t	a	g	t	t	r	a	c	c	t	a	t	c	g	a	g	g
JF306641	Vac	a	g	t	c	c	g	c	a	t	g	t	a	a	g	a	a	t	a	a	t	a	t	a	g	t	g	a	a	a	t	a	g	t	c	c	g	g	g	t	g	g	g	t	t	c	c	a	a	a	t	t	a	c	a	g	a	a	t	a	g	t	t	a	a	c	c	t	a	t	c	g	a	g	g
AB097932	Vac	a	r	t	c	c	g	c	a	t	g	t	a	a	g	a	a	t	a	a	t	a	t	a	g	t	g	a	a	a	t	a	r	t	c	c	g	g	g	t	g	g	g	t	t	c	c	a	a	a	t	t	a	c	a	g	a	a	t	a	g	t	t	a	a	c	c	t	a	t	c	g	a	g	g
DQ008354	Vac	a	a	t	c	c	g	c	a	t	g	t	a	a	g	a	a	t	a	a	t	a	t	a	g	t	g	a	a	a	t	a	g	t	c	c	g	g	g	t	g	g	g	t	t	c	c	a	a	a	t	t	a	c	a	g	a	a	t	a	g	t	t	r	a	c	c	t	a	t	c	g	a	g	g
KU926312	Vac	a	g	t	c	c	g	c	a	t	g	t	a	a	g	a	a	t	a	a	t	a	t	a	g	t	g	a	a	a	t	a	g	t	c	c	g	g	g	t	g	g	g	t	t	c	c	a	a	a	t	t	a	c	a	g	a	a	t	a	g	t	t	a	a	c	c	t	a	t	c	g	a	g	g
KU926313	Vac	a	a	t	c	c	g	c	a	t	g	t	a	a	g	a	a	t	a	a	t	a	t	a	g	t	g	a	a	a	t	a	g	t	c	c	g	g	g	t	g	g	g	t	t	c	c	a	a	a	t	t	a	c	a	g	a	a	t	a	g	t	t	a	a	c	c	t	a	t	c	g	a	g	g
KU926314	Vac	a	a	t	c	c	g	c	a	t	g	t	a	a	g	a	a	t	a	a	t	a	t	a	g	t	g	a	a	a	t	a	g	t	c	c	g	g	g	t	g	g	g	t	t	c	c	a	a	a	t	t	a	c	a	g	a	a	t	a	g	t	t	a	a	c	c	t	a	t	c	g	a	g	g
KF558371	Vac	a	a	t	c	c	g	c	a	t	g	t	a	a	g	a	a	t	a	a	t	a	t	a	g	t	g	a	a	a	t	a	g	t	c	c	g	g	g	t	g	g	g	t	t	c	c	a	a	a	t	t	a	c	a	g	a	a	t	a	g	t	t	a	a	c	c	t	a	t	c	g	a	g	g
KF558372	Vac	a	a	t	c	c	g	c	a	t	g	t	a	a	g	a	a	t	a	a	t	a	t	a	g	t	g	a	a	a	t	a	g	t	c	c	g	g	g	t	g	g	g	t	t	c	c	a	a	a	t	t	a	c	a	g	a	a	t	a	g	t	t	a	a	c	c	t	a	t	c	g	a	g	g
KF558373	Vac	a	a	t	c	c	g	c	a	t	g	t	a	a	g	a	a	t	a	a	t	a	t	a	g	t	g	a	a	a	t	a	g	t	c	c	g	g	g	t	g	g	g	t	t	c	c	a	a	a	t	t	a	c	a	g	a	a	t	a	g	t	t	a	a	c	c	t	a	t	c	g	a	g	g
KF558374	Vac	a	a	t	c	c	g	c	a	t	g	t	a	a	g	a	a	t	a	a	t	a	t	a	g	t	a	a	a	a	t	a	g	t	c	c	g	g	g	t	g	g	g	t	t	c	c	a	a	a	t	t	a	c	a	g	a	a	t	a	g	t	t	a	a	c	c	t	a	t	c	g	a	g	g
KF558375	Vac	a	a	t	c	c	g	c	a	t	g	t	a	a	g	a	a	t	a	a	t	a	t	a	g	t	g	a	a	a	t	a	g	t	c	c	g	g	g	t	g	g	g	t	t	c	c	a	a	a	t	t	a	c	a	g	a	a	t	a	g	t	t	a	a	c	c	t	a	t	c	g	a	g	g
KF558376	Vac	a	a	t	c	c	g	c	a	t	g	c	a	a	g	a	a	t	a	a	t	a	t	a	g	t	g	a	a	a	t	a	g	t	c	c	g	g	g	t	g	g	g	t	t	c	c	a	a	a	t	t	a	c	a	g	a	a	t	a	g	t	t	a	a	c	c	t	a	t	c	g	a	g	g
KF558377	Vac	a	a	t	c	c	g	c	a	t	g	t	a	a	g	a	a	t	a	a	t	a	t	a	g	t	g	a	a	a	t	a	g	t	c	c	g	g	g	t	g	g	g	t	t	c	c	a	a	a	t	t	a	c	a	g	a	a	t	a	g	t	t	g	a	c	c	t	a	t	c	g	a	g	g
KF558378	Vac	a	a	t	c	c	g	c	a	t	g	t	a	a	g	a	a	t	a	a	t	a	t	a	g	t	g	a	a	a	t	a	g	t	c	c	g	g	g	t	g	g	g	t	t	c	c	a	a	a	t	t	a	c	a	g	a	a	t	a	g	t	t	a	a	c	c	t	a	t	c	g	a	g	g
KF558379	Vac	a	a	t	c	c	g	c	a	t	g	t	a	a	g	a	a	t	a	a	t	a	t	a	g	t	g	a	a	a	t	a	g	t	c	c	g	g	g	t	g	g	g	t	t	c	c	a	a	a	t	t	a	c	a	g	a	a	t	a	g	t	t	a	a	c	c	t	a	t	c	g	a	g	g
KF558380	Vac	a	g	t	c	c	g	c	a	t	g	t	a	a	g	a	a	t	a	a	t	a	t	a	g	t	g	a	a	a	t	a	g	t	c	c	g	g	g	t	g	g	g	t	t	c	c	a	a	a	t	t	a	c	a	g	a	a	t	a	g	t	t	a	a	c	c	t	a	t	c	g	a	g	g
KF558381	Vac	a	a	t	c	c	g	c	a	t	g	t	a	a	g	a	a	t	a	a	t	a	t	a	g	t	g	a	a	a	t	a	g	t	c	c	g	g	g	t	g	g	g	t	t	c	c	a	a	a	t	t	a	c	a	g	a	a	t	a	g	t	t	g	a	c	c	t	a	t	c	g	a	g	g
KF558382	Vac	a	a	t	c	c	g	c	a	t	g	t	a	a	g	a	a	t	a	a	t	a	t	a	g	t	g	a	a	a	t	a	g	t	c	c	g	g	g	t	g	g	g	t	t	c	c	a	a	a	t	t	a	c	a	g	a	a	t	a	g	t	t	a	a	c	c	t	a	t	c	g	a	g	g
KF558383	Vac	a	a	t	c	c	g	c	a	t	g	t	a	a	g	a	a	t	a	a	t	a	t	a	g	t	g	a	a	a	t	a	g	t	c	c	g	g	g	t	g	g	g	t	t	c	c	a	a	a	t	t	a	c	a	g	a	a	t	a	g	t	t	a	a	c	c	t	a	t	c	g	a	g	g
KF558384	Vac	a	a	t	c	c	g	c	a	t	g	t	a	a	g	a	a	t	a	a	t	a	t	a	g	t	g	a	a	a	t	a	g	t	c	c	g	g	g	t	g	g	g	t	t	c	c	a	a	a	t	t	a	c	a	g	a	a	t	a	g	t	t	a	a	c	c	t	a	t	c	g	a	g	g
KF558385	Vac	a	a	t	c	c	g	c	a	t	g	t	a	a	g	a	a	t	a	a	t	a	t	a	g	t	g	a	a	a	t	a	g	t	c	c	g	g	g	t	g	g	g	t	t	c	c	a	a	a	t	t	a	c	a	g	a	a	t	a	g	t	t	a	a	c	c	t	a	t	c	g	a	g	g
KF558386	Vac	a	a	t	c	c	g	c	a	t	g	t	a	a	g	a	a	t	a	a	t	a	t	a	g	t	g	a	a	a	t	a	g	t	c	c	g	g	g	t	g	g	g	t	t	c	c	a	a	a	t	t	a	c	a	g	a	a	t	a	g	t	t	a	a	c	c	t	a	t	c	g	a	g	g
KF558387	Vac	a	a	t	c	c	g	c	a	t	g	t	a	a	g	a	a	t	a	a	t	a	t	a	g	t	g	a	a	a	t	a	g	t	c	c	g	g	g	t	g	g	g	t	t	c	c	a	a	a	t	t	a	c	a	g	a	a	t	a	g	t	t	a	a	c	c	t	a	t	c	g	a	g	g
KF558388	Vac	a	a	t	c	c	g	c	a	t	g	t	a	a	g	a	a	t	a	a	t	a	t	a	g	t	g	a	a	a	t	a	g	t	c	c	g	g	g	t	g	g	g	t	t	c	c	a	a	a	t	t	a	c	a	g	a	a	t	a	g	t	t	a	a	c	c	t	a	t	c	g	a	g	g
KF558389	Vac	a	a	t	c	c	g	c	a	t	g	t	a	a	g	a	a	t	a	a	t	a	t	a	g	t	a	a	a	a	t	a	g	t	c	c	g	g	g	t	g	g	g	t	t	c	c	a	a	a	t	t	a	c	a	g	a	a	t	a	g	t	t	a	a	c	c	t	a	t	c	g	a	g	g
KF558390	Vac	a	a	t	c	c	g	c	a	t	g	t	a	a	g	a	a	t	a	a	t	a	t	a	g	t	g	a	a	a	t	a	g	t	c	c	g	g	g	t	g	g	g	t	t	c	c	a	a	a	t	t	a	c	a	g	a	a	t	a	g	t	t	a	a	c	c	t	a	t	c	g	a	g	g
KF558391	Vac	a	a	t	c	c	g	c	a	t	g	t	a	a	g	a	a	t	a	a	t	a	t	a	g	t	g	a	a	a	t	a	g	t	c	c	g	g	g	t	g	g	g	t	t	c	c	a	a	a	t	t	a	c	a	g	a	a	t	a	g	t	t	a	a	c	c	t	a	t	c	g	a	g	g
KF811485	Vac	a	a	t	c	c	g	c	a	t	g	t	a	a	g	a	a	t	a	a	t	a	t	a	g	t	g	a	a	a	t	a	g	t	c	c	g	g	g	t	g	g	g	t	t	c	c	a	a	a	t	t	a	c	a	g	a	a	t	a	g	t	t	a	a	c	c	t	a	t	c	g	a	g	g
KF853225	Vac	a	a	t	c	c	g	c	a	t	g	t	a	a	g	a	a	t	a	a	t	a	t	a	g	t	g	a	a	a	t	a	g	t	c	c	g	g	g	t	g	g	g	t	t	c	c	a	a	a	t	t	a	c	a	g	a	a	t	a	g	t	t	a	a	c	c	t	a	t	c	g	a	g	g
KF853226	Vac	a	a	t	c	c	g	c	a	t	g	t	a	a	g	a	a	t	a	a	t	a	t	a	g	t	g	a	a	a	t	a	g	t	c	c	g	g	g	t	g	g	g	t	t	c	c	a	a	a	t	t	a	c	a	g	a	a	t	a	g	t	t	a	a	c	c	t	a	t	c	g	a	g	g
KF853227	Vac	a	a	t	c	c	g	c	a	c	g	t	a	a	g	a	a	t	a	a	t	a	t	a	g	t	g	a	a	a	t	a	g	t	c	c	g	g	g	t	g	g	g	t	t	c	c	a	a	a	t	t	a	c	a	g	a	a	t	a	g	t	t	a	a	c	c	t	a	t	c	g	a	g	g
KF853228	Vac	a	a	t	c	c	g	c	a	t	g	t	a	a	g	a	a	t	a	a	t	a	t	a	g	t	g	a	a	a	t	a	g	t	c	c	g	g	g	t	g	g	g	t	t	c	c	a	a	a	t	t	a	c	a	g	a	a	t	a	g	t	t	a	a	c	c	t	a	t	c	g	a	g	g
KF853229	Vac	a	a	t	c	c	g	c	a	t	g	t	a	a	g	a	a	t	a	a	t	a	t	a	g	t	g	a	a	a	t	a	g	t	c	c	g	g	g	t	g	g	g	t	t	c	c	a	a	a	t	t	a	c	a	g	a	a	t	a	g	t	t	a	a	c	c	t	a	t	c	g	a	g	g
KF853230	Vac	a	a	t	c	c	g	c	a	t	g	t	a	a	g	a	a	t	a	a	t	a	t	a	g	t	g	a	a	a	t	a	g	t	c	c	g	g	g	t	g	g	g	t	t	c	c	a	a	a	t	t	a	c	a	g	a	a	t	a	g	t	t	a	a	c	c	t	a	t	c	g	a	g	g
KF853231	Vac	a	a	t	c	c	g	c	a	t	g	t	a	a	g	a	a	t	a	a	t	a	t	a	g	t	g	a	a	a	t	a	g	t	c	c	g	g	g	t	g	g	g	t	t	c	c	a	a	a	t	t	a	c	a	g	a	a	t	a	g	t	t	a	a	c	c	t	a	t	c	g	a	g	g
KF853232	Vac	a	a	t	c	c	g	c	a	t	g	t	a	a	g	a	a	t	a	a	t	a	t	a	g	t	g	a	a	a	t	a	g	t	c	c	g	g	g	t	g	g	g	t	t	c	c	a	a	a	t	t	a	c	a	g	a	a	t	a	g	t	t	a	a	c	c	t	a	t	c	g	a	g	g
KF853233	Vac	a	a	t	c	c	g	c	a	t	g	t	a	a	g	a	a	t	a	a	t	a	t	a	g	t	g	a	a	a	t	a	g	t	c	c	g	g	g	t	g	g	g	t	t	c	c	a	a	a	t	t	a	c	a	g	a	a	t	a	g	t	t	a	a	c	c	t	a	t	c	g	a	g	g
KF853234	Vac	a	a	t	c	c	g	c	a	t	g	t	a	a	g	a	a	t	a	a	t	a	t	a	g	t	g	a	a	a	t	a	g	t	c	c	g	g	g	t	g	g	g	t	t	c	c	a	a	a	t	t	a	c	a	g	a	a	t	a	g	t	t	a	a	c	c	t	a	t	c	g	a	g	g
KF853235	Vac	a	a	t	c	c	g	c	a	t	g	t	a	a	g	a	a	t	a	a	t	a	t	a	g	t	g	a	a	a	t	a	g	t	c	c	g	g	g	t	g	g	g	t	t	c	c	a	a	a	t	t	a	c	a	g	a	a	t	a	g	t	t	g	a	c	c	t	a	t	c	g	a	g	g
MF898328	Vac	a	r	t	c	c	g	c	a	t	g	t	a	a	g	a	a	t	a	a	t	a	t	a	g	t	g	a	a	a	t	a	g	t	c	c	g	g	g	t	g	g	g	t	t	c	c	a	a	a	t	t	a	c	a	g	a	a	t	a	g	t	t	r	a	c	c	t	a	t	c	g	a	g	g
JN704709	VIII	c	a	t	c	c	g	c	a	t	g	t	a	a	g	a	a	t	a	a	t	a	t	a	g	t	g	a	a	a	t	a	g	t	c	c	g	g	g	t	g	g	g	t	t	c	c	a	a	a	t	t	a	c	a	g	a	a	t	a	g	t	t	a	a	c	c	t	a	t	c	g	c	g	g

Supplementary Table 3: 2880 SN

GB-ID	Clade markers																					9																		1														5																				
	Position in Dumas	86258	86441	86480	86484	86485	86486	86497	86516	86523_1	86534	86556	86589	86620	86656	86671	86777	86782	86826	86866	86869	86893	86932	86966	86986	87016	87063	87095	87130	87251	87280	87306	87352	87415	87463	87489	87543	87747	87815	87841	88000	88124	88240	88405	88477	88681	88744	88780	88792	88846	88855	88867	88966	88984	88997	89137	89182	89203	89362	89406	89522	89605	89618	89647	89662	89676	89692	89698	89734	89827	89905	89965	89986	90088
AY548170	1	a	g	a	c	c	t	c	a	-	t	t	t	g	g	t	c	c	t	a	t	a	c	g	t	t	t	t	t	a	a	t	c	t	a	t	t	t	a	c	c	c	g	g	g	c	t	a	t	c	g	c	g	t	a	a	t	c	c	g	a	t	g	g	t	t	a	c	a	g	t	c	a	t
AY548171	1	a	g	a	c	c	t	c	a	-	t	t	t	g	g	t	c	c	t	a	t	a	c	g	t	t	t	t	t	a	a	t	c	t	a	t	t	t	a	c	c	c	g	g	g	c	t	a	t	c	g	c	g	t	a	a	t	c	c	g	a	t	g	g	t	t	a	c	a	g	t	c	a	t
DQ479953	1	a	g	a	c	c	t	c	a	-	t	t	t	g	g	t	c	c	t	a	t	a	c	g	t	t	t	t	t	a	a	t	c	t	a	t	t	t	a	c	c	c	g	g	g	c	t	a	t	c	g	c	g	t	a	a	t	c	c	g	a	t	g	g	t	t	a	c	a	g	t	c	a	t
DQ479954	1	a	g	a	c	c	t	c	a	-	t	t	t	g	g	c	c	c	t	a	t	a	c	g	t	t	t	t	t	a	a	t	c	t	a	t	t	t	a	c	c	c	g	g	g	c	t	a	t	c	g	c	g	t	a	a	t	c	c	g	a	t	g	g	t	t	a	c	a	g	t	c	a	t
DQ479958	1	a	g	a	c	c	t	c	a	-	t	t	t	g	g	t	c	c	t	a	t	a	c	g	t	t	t	t	t	a	a	t	c	t	a	t	t	t	a	c	c	c	g	g	g	c	t	a	t	c	g	c	g	t	a	a	t	c	c	g	a	t	g	g	t	t	a	c	a	g	t	c	a	c
DQ479959	1	a	g	a	c	c	t	c	a	-	t	t	t	g	g	t	c	c	t	a	t	a	c	g	t	t	t	t	t	a	a	t	c	t	a	t	t	t	a	c	c	c	g	g	g	c	t	a	t	c	g	c	g	t	a	a	t	c	c	g	a	t	g	g	t	t	a	c	a	g	t	c	a	c
DQ479961	1	a	g	a	c	c	t	c	a	-	t	t	t	g	g	t	c	c	t	a	t	a	c	g	t	t	t	t	t	a	a	t	c	t	a	t	t	t	a	c	c	c	g	g	g	c	t	a	t	c	g	c	g	t	a	a	t	c	c	g	a	t	g	g	t	t	a	c	a	g	t	c	a	t
DQ479962	1	a	g	a	c	c	t	c	a	-	t	t	t	g	g	t	c	c	t	a	t	a	c	g	t	t	t	t	t	a	a	t	c	t	a	t	t	t	a	c	c	c	g	g	g	c	t	a	t	c	g	c	g	t	a	a	t	c	c	g	a	t	g	g	t	t	a	c	a	g	t	c	a	t
DQ479963	1	a	g	a	c	c	t	c	a	-	t	t	t	g	g	t	c	c	t	a	t	a	c	g	t	t	t	t	t	a	a	t	c	t	a	t	t	t	a	c	c	c	g	g	g	c	t	a	t	c	g	c	g	t	a	a	t	c	c	g	a	t	g	g	t	t	a	c	a	g	t	c	a	t
DQ674250	1	a	g	a	c	c	t	c	a	-	t	t	t	g	g	t	c	c	t	a	t	a	c	g	t	t	t	t	t	a	a	t	c	t	a	t	t	t	a	c	c	a	g	g	g	c	t	a	t	c	g	c	g	t	a	a	t	c	c	g	a	t	g	g	t	t	a	c	a	g	t	c	a	t
KX262864	1	a	g	a	c	c	t	c	a	-	t	t	t	g	g	t	c	c	t	a	t	a	c	g	t	t	t	t	t	a	a	t	c	t	a	t	t	t	a	c	c	c	g	g	g	c	t	a	t	c	g	c	g	t	a	a	t	c	c	g	a	t	g	g	t	t	a	c	a	g	t	c	a	t
KX262865	1	a	g	a	c	c	t	c	a	-	t	t	t	g	g	t	c	c	t	a	t	a	c	g	t	t	t	t	t	a	a	t	c	t	a	t	t	t	a	c	c	c	g	g	g	c	t	a	t	c	g	c	g	t	a	a	t	c	c	g	a	t	g	g	t	t	a	c	a	g	t	c	a	t
KX262866	1	a	g	a	c	c	t	c	a	-	t	t	t	g	g	t	c	c	t	a	t	a	c	g	t	t	t	t	t	a	a	t	c	t	a	t	t	t	a	c	c	c	g	g	g	c	t	a	t	c	g	c	g	t	a	a	t	c	c	g	a	t	g	g	t	t	a	c	a	g	t	c	a	t
EU154348	1	a	g	a	c	c	t	c	a	-	t	t	t	g	g	t	c	c	t	a	t	a	c	g	t	t	t	t	t	a	a	t	c	t	a	t	t	t	a	c	c	c	g	g	g	c	t	a	t	c	g	c	g	t	a	a	t	c	c	g	a	t	g	g	t	t	a	c	a	g	t	c	a	t
JN704690	1	a	g	a	c	c	t	c	a	-	t	t	t	g	g	t	c	c	t	a	t	a	c	g	t	t	t	t	t	a	a	t	c	t	a	t	t	t	a	c	c	c	g	g	g	c	t	a	t	c	g	c	g	t	a	a	t	c	c	g	a	t	g	g	t	t	a	c	a	g	t	c	a	t
JN704691	1	a	g	a	c	c	t	c	a	-	t	t	t	g	g	t	c	c	t	a	t	a	c	g	t	t	t	t	t	a	a	t	c	t	a	t	t	t	a	c	c	c	g	g	g	c	t	a	t	c	g	c	g	t	a	a	t	c	c	g	a	t	g	g	t	t	a	c	a	g	t	c	a	t
JN704692	1	a	g	a	c	c	t	c	a	-	t	t	t	g	g	t	c	c	t	a	t	a	c	g	t	t	t	t	t	a	a	t	c	t	a	t	t	t	a	c	c	c	g	g	g	c	t	a	t	c	g	c	g	t	a	a	t	c	c	g	a	t	g	g	t	t	a	c	a	g	t	c	a	t
JN704693	1	a	g	a	c	c	t	c	a	-	t	t	t	g	g	t	c	c	t	g	t	a	c	g	t	t	t	t	t	a	a	t	c	t	a	t	t	t	a	c	c	c	g	g	g	c	t	a	t	c	g	c	g	t	a	a	t	c	c	g	a	t	g	g	t	t	a	c	a	g	t	c	a	t
JN704694	1	a	g	a	c	c	t	c	a	-	t	t	t	g	g	t	c	c	t	a	t	a	c	g	t	t	t	t	t	a	a	t	c	t	a	t	t	t	a	c	c	c	g	g	g	c	t	a	t	c	g	c	g	t	a	a	t	c	c	g	a	t	g	g	t	t	a	c	a	g	t	c	a	t
JN704695	1	a	g	a	c	c	t	c	a	-	t	t	t	g	g	t	c	c	t	a	t	a	c	g	t	t	t	t	t	a	a	t	c	t	a	t	t	t	a	c	c	c	g	g	g	c	t	a	t	c	g	c	g	t	a	a	t	c	c	g	a	t	g	g	t	t	a	c	a	g	t	c	a	t
JN704696	1	a	g	a	c	c	t	c	a	-	t	t	t	g	g	t	c	c	t	a	t	a	c	g	t	t	t	t	t	a	a	t	c	t	a	t	t	t	a	c	c	c	g	g	g	c	t	a	t	c	g	c	g	t	a	a	t	c	c	g	a	t	g	g	t	t	a	c	a	g	t	c	a	t
NC001348	1	a	g	a	c	c	t	c	a	-	t	t	t	g	g	t	c	c	t	a	t	a	c	g	t	t	t	t	t	a	a	t	c	t	a	t	t	t	a	c	c	c	g	g	g	c	t	a	t	c	g	c	g	t	a	a	t	c	c	g	a	t	g	g	t	t	a	c	a	g	t	c	a	t
KP771892	1	a	g	a	c	c	t	c	a	-	t	t	t	g	g	t	c	c	t	a	t	a	c	g	t	t	t	t	t	a	a	t	c	t	a	t	t	t	a	c	c	c	g	g	g	c	t	a	t	c	g	c	g	t	a	a	t	c	c	g	a	t	g	g	t	t	a	c	a	g	t	c	a	t
KP771894	1	a	g	a	c	c	t	c	a	-	t	t	t	g	g	t	c	c	t	a	t	a	c	g	t	t	t	t	t	a	a	t	c	t	a	t	t	t	a	c	c	c	g	g	g	c	t	a	t	c	g	c	g	t	a	a	t	c	c	g	a	t	g	g	t	t	a	c	a	g	t	c	a	t
KP771900	1	a	g	a	c	c	t	c	a	-	t	t	t	g	g	t	c	c	t	a	t	a	c	g	t	t	t	t	t	a	a	t	c	t	a	t	t	t	a	c	c	c	g	g	g	c	t	a	t	c	g	c	g	t	a	a	t	c	c	g	a	t	g	g	t	t	a	c	a	g	t	c	a	t
KP771901	1	a	g	a	c	c	t	c	a	-	t	t	t	g	g	t	c	c	t	a	t	a	c	g	t	t	t	t	t	a	a	t	c	t	a	t	t	t	a	c	c	c	g	g	g	c	t	a	t	c	g	c	g	t	a	a	t	c	c	g	a	t	g	g	t	t	a	c	a	g	t	c	a	t
KP771902	1	a	g	a	c	c	t	c	a	-	t	t	t	g	g	t	c	c	t	a	t	a	c	g	t	t	t	t	t	a	a	t	c	t	a	t	t	t	a	c	c	c	g	g	g	c	t	a	t	c	g	c	g	t	a	a	t	c	c	g	a	t	g	g	t	t	a	c	a	g	t	c	a	t
KP771908	1	a	g	a	c	c	t	c	a	-	t	t	t	g	g	t	c	c	t	a	t	a	c	g	t	t	t	t	t	a	a	t	c	t	a	t	t	t	a	c	c	c	g	g	g	c	t	a	t	c	g	c	g	t	a	a	t	c	c	g	a	t	g	g	t	t	a	c	a	g	t	c	a	t
KP771912	1	a	g	a	c	c	t	c	a	-	t	t	t	g	g	t	c	c	t	a	t	a	c	g	t	t	t	t	t	a	a	t	c	t	a	t	t	t	a	c	c	c	g	g	g	c	t	a	t	c	g	c	g	t	a	a	t	c	c	g	a	t	g	g	t	t	a	c	a	g	t	c	a	t
KP771913	1	a	g	a	c	c	t	c	a	-	t	t	t	g	g	t	c	c	t	a	t	a	c	g	t	t	t	t	t	a	a	t	c	t	a	t	t	t	a	c	c	c	g	g	g	c	t	a	t	c	g	c	g	t	a	a	t	c	c	g	a	t	g	g	t	t	a	c	a	g	t	c	a	t
KP771917	1	a	g	a	c	c	t	c	a	-	t	t	t	g	g	t	c	c	t	a	t	a	c	g	t	t	t	t	t	a	a	t	c	t	a	t	t	t	a	c	c	c	g	a	g	c	t	a	t	c	g	c	g	t	a	a	t	t	c	g	a	t	g	g	t	t	a	c	a	g	t	c	a	t
KP771918	1	g	g	a	c	c	t	c	a	-	t	t	t	g	g	t	c	c	t	a	t	a	c	g	t	t	t	t	t	a	a	t	c	t	a	t	t	t	a	c	c	c	g	g	g	c	t	a	t	c	g	c	g	t	a	a	t	c	c	g	a	t	g	g	t	t	a	c	a	g	t	c	a	t
KP771920	1	a	g	a	c	c	t	c	a	-	t	t	t	g	g	t	c	c	t	a	t	a	c	g	t	t	t	t	t	a	a	t	c	t	a	t	t	t	a	c	c	c	g	g	g	c	t	a	t	c	g	c	g	t	a	a	t	c	c	g	a	t	g	g	t	t	a	c	a	g	t	c	a	t
MH709361	1	a	g	a	c	c	t	c	a	-	t	t	t	g	g	t	c	c	t	a	t	a	c	g	t	t	t	t	t	a	a	t	c	t	a	t	t	t	a	c	c	c	g	g	g	c	t	a	t	c	g	c	g	t	a	a	t	c	c	g	a	t	g	g	t	t	a	c	a	g	t	c	a	t
KU926318	2	a	g	-	-	-	t	c	a	-	t	t	t	g	g	t	c	c	t	a	t	a	c	g	c	t	t	t	t	a	a	t	c	t	g	t	t	c	a	t	c	c	g	g	t	c	t	a	t	c	g	c	g	t	a	g	t	c	c	g	a	t	g	g	t	t	a	c	a	g	c	c	a	t
KU926319	2	a	g	-	-	-	t	c	a	-	t	t	t	g	g	t	c	c	t	a	t	a	c	g	t	t	t	t	t	a	a	t	c	t	a	t	t	t	a	t	c	c	g	g	t	c	t	a	t	c	g	t	g	t	a	a	t	c	c	g	a	t	g	g	t	t	a	c	a	g	c	c	a	t
KU926320	2	a	g	-	-	-	t	c	a	-	t	t	t	g	g	t	c	c	c	a	t	a	c	g	t	t	t	t	t	a	a	t	c	t	a	t	t	t	a	t	c	c	g	g	t	c	c	a	t	c	g	t	g	t	a	a	t	c	c	g	a	t	g	g	t	t	a	c	a	g	c	c	a	t
KU926321	2	a	g	-	-	-	t	c	a	-	t	t	t	g	g	t	c	c	t	a	t	a	c	g	t	t	t	t	t	a	a	t	c	t	a	t	t	t	a	t	c	c	g	g	t	c	t	a	t	c	g	t	g	t	a	a	t	c	c	g	a	t	g	g	t	t	a	c	a	g	c	c	a	t
KU926322	2	a	g	-	-	-	t	c	a	-	t	t	t	g	g	t	c	c	t	a	t	a	c	g	t	t	t	t	t	a	a	t	c	t	a	t	t	t	a	t	c	c	g	g	t	c	t	a	t	c	g	t	g	t	a	a	t	c	c	g	a	t	g	g	t	t	a	c	a	g	c	c	a	t
KC847290	2	a	g	-	-	-	t	c	a	-	t	t	t	g	g	t	c	c	t	a	t	a	c	g	t	t	t	t	t	a	a	t	c	t	a	t	t	t	a	t	c	c	g	g	t	c	t	a	t	c	g	c	g	t	a	a	t	c	c	g	a	t	g	g	t	t	a	c	a	g	c	c	a	t
KJ767491	2	a	g	-	-	-	t	c	a	-	t	t	t	g	g	t	c	c	t	a	t	a	c	g	t	t	t	t	t	a	a	t	c	t	a	t	t	t	a	t	c	c	g	g	t	c	t	a	t	c	g	t	g	t	a	a	t	c	c	g	a	t	g	g	t	t	a	c	a	g	c	c	a	t
KJ767492	2	a	g	-	-	-	t	c	a	-	t	t	t	g	g	t	c	c	t	a	t	a	c	g	t	t	t	t	t	a	a	t	c	t	a	t	t	t	a	t	c	c	g	g	t	c	t	a	t	c	g	t	g	t	a	a	t	c	c	g	a	t	g	g	t	t	a	c	a	g	c	c	a	t
KJ808816	2	a	g	-	-	-	t	c	a	-	t	t	t	g	g	t	c	c	t	a	t	a	c	g	t	t	t	t	t	a	a	t	c	t	a	t	t	t	a	t	c	c	g	g	t	c	t	a	t	c	g	t	g	t	a	a	t	c	c	g	a	t	g	g	t	t	a	c	a	g	c	c	a	t
KP771925	2	a	g	a	c	c	t	c	a	-	t	t	t	g	g	t	c	c	t	a	t	a	c	g	t	t	t	t	t	a	a	t	c	t	a	t	t	t	a	t	c	c	g	g	t	c	t	a	t	c	g	t	g	t	a	a	t	c	c	g	a	t	g	g	t	t	a	c	a	g	c	c	a	t
KU926315	2	a	g	-	-	-	t	c	a	-	t	t	t	g	g	t	c	c	t	a	t	a	c	g	t	t	t	t	t	a	a	t	c	t	a	t	t	t	a	t	c	c	g	g	t	c	t	a	t	c	g	c	g	t	a	a	t	c	c	g	a	t	g	g	t	t	a	c	a	g	c	c	a	t
KU926316	2	a	g	-	-	-	t	c	a	-	t	t	t	g	g	t	c	c	t	a	t	a	c	g	t	t	t	t	t	a	a	t	c	t	a	t	t	t	a	t	c	c	g	g	t	c	t	a	t	c	g	c	g	t	a	a	t	c	c	g	a	t	g	g	t	t	a	c	a	g	c	c	a	t
KU926317	2	a	g	-	-	-	t	c	a	-	t	t	t	g	g	t	c	c	t	a	t	a	c	g	t	t	t	t	t	a	a	t	c	t	a	t	t	t	a	t	c	c	g	g	t	c	t	a	t	c	g	c	g	t	a	a	t	c	c	g	a	t	g	g	t	t	a	c	a	g	c	c	a	t
KY037796	2	a	g	-	-	-	t	c	a	-	t	t	t	g	g	t	c	c	t	a	t	a	c	g	t	t	t	t	t	a	a	t	c	t	a	t	t	t	a	t	c	c	a	g	t	c	t	a	t	c	g	c	g	t	a	a	t	c	c	g	a	t	g	g	t	t	a	c	a	g	c	c	a	t
MF004348	2	a	g	-	-	-	t	c	a	-	t	t	t	g	g	t	c	c	t	a	t	a	c	g	t	t	t	t	t	a	a	t	c	t	a	t	t	t	a	t	c	c	g	g	t	c	t	a	t	c	g	t	g	t	a	a	t	c	c	g	a	t	g	g	t	t	a	c	a	g	c	c	a	t
MH709309	2	a	g	a	c	c	t	c	a	-	t	t	t	g	g	t	c	c	t	a	t	a	c	g	t	t	t	t	t	a	a	t	c	t	a	t	t	t	a	t	c	c	g	g	t	c	t	a	t	c	g	c	g	t	a	a	g	c	c	g	a	t	g	g	t	t	a	c	a	g	c	c	a	t
MH709310	2	a	g	a	g	t	t	c	a	-	t	t	t	g	g	t	c	c	t	a	t	a	c	g	t	t	t	t	t	a	a	t	c	t	a	t	t	t	a	t	c	c	g	g	t	c	t	a	t	c	g	c	g	t	a	a	t	c	c	g	a	t	g	g	t	t	a	c	a	g	c	c	a	t
MH709311	2	a	g	-	-	-	t	c	a	-	t	t	t	g	g	t	c	c	t	a	t	a	c	g	t	t	t	t	t	a	a	t	c	t	a	t	t	t	a	t	c	c	g	g	t	c	t	a	t	c	g	c	g	t	a	a	t	c	c	g	a	t	g	g	t	t	a	a	a	g	c	c	a	t
MH709313	2	a	g	a	c	c	t	c	a	-	t	t	t	g	g	t	t	a	t	a	t	a	c	g	t	t	t	t	t	a	a	t	c	t	a	t	t	t	a	t	c	c	g	g	t	c	t	a	t	c	g	c	g	t	a	a	t	c	c	g	a	t	g	g	t	t	a	c	a	g	c	c	a	t
MH709314	2	a	g	a	c	c	t	c	a	-	t	t	t	g	g	t	c	c	t	a	t	a	c	g	t	t	t	t	t	a	a	t	c	t	a	t	t	t	a	t	c	c	g	g	t	c	t	a	t	c	g	c	g	t	a	a	t	c	c	g	a	t	g	g	t	t	a	c	a	g	c	c	a	t
MH709315	2	a	g	a	c	c	t	c	a	-	t	t	t	g	g	t	c	c	t	a	t	a	c	g	t	t	t	t	t	a	a	t	c	t	a	t	t	t	a	t	c	c	g	g	t	c	t	a	t	c	g	c	g	t	a	a	t	c	c	g	a	t	g	g	t	t	a	c	a	g	c	c	a	t
MH709319	2	a	g	a	c	c	t	c	a	-	t	t	t	g	g	t	c	c	t	a	t	a	c	g	t	t	t	t	t	a	a	t	c	t	a	t	t	t	a	t	c	c	g	g	t	c	t	a	t	c	g	c	g	t	a	a	t	c	c	g	a	t	g	g	t	t	a	c	a	g	c	c	a	t
MH709320	2	a	g	a	g	c	t	c	a	-	t	t	t	g	g	t	c	c	t	a	t	a	c	g	t	t	t	t	t	a	a	t	c	t	a	t	t	t	a	t	c	c	g	g	t	c	t	a	t	c	g	c	g	t	a	a	t	c	c	g	a	t	g	g	t	t	a	t	a	g	c	c	a	t
MH709329	2	a	g	a	g	c	t	c	a	-	t	t	t	g	g	t	c	c	t	a	t	a	c	g	t	t	t	t	t	a	a	t	c	t	a	t	t	t	a	t	c	c	g	g	t	c	t	a	t	c	g	c	g	t	a	a	t	c	c	g	a	t	g	g	t	t	a	c	a	g	c	c	a	t
MH709331	2	a	g	a	c	c	t	c	a	-	t	t	t	g	g	t	c	c	t	a	t	a	c	g	t	t	t	t	t	a	a	t	c	t	a	t	t	t	a	t	c	c	g	g	t	c	t	a	t	c	g	c	g	t	a	a	t	c	c	g	a	t	g	g	t	t	a	c	a	g	c	c	a	t
MH709333	2	a	g	a	c	c	t	c	a	-	t	t	t	g	g	t	c	c	t	a	t	a	c	g	t	t	t	t	t	a	a	t	c	t	a	t	t	t	a	t	c	c	g	g	t	c	t	a	t	c	g	c	g	t	a	a	t	c	c	g	a	t	g	g	t	t	a	c	a	g	c	c	a	t
MH709337	2	a	g	a	g	t	t	c	a	-	t	t	t	g	g	t	c	c	t	a	t	a	c	g	t	t	t	t	t	a	a	t	c	t	a	t	t	t	a	t	c	c	g	g	t	c	t	a	t	c	g	c	g	t	a	a	t	c	c	g	a	t	g	g	t	t	a	c	a	g	c	c	a	t
MH709339	2	a	g	-	-	-	t	c	a	-	t	t	t	g	g	t	c	c	t	a	t	a	c	g	t	t	t	t	t	a	a	t	c	t	a	t	t	t	a	t	t	c	g	g	t	c	t	a	t	c	g	c	g	t	a	a	t	c	c	g	a	t	g	g	t	t	a	c	a	g	c	c	a	t
MH709344	2	a	g	a	c	c	t	c	a	-	c	t	t	g	g	t	c	c	t	a	t	a	c	g	t	t	t	t	t	a	a	t	c	t	a	t	t	t	a	t	c	c	g	g	t	c	t	a	t	c	g	c	g	t	a	a	t	c	c	g	a	t	g	g	t	t	a	c	a	g	c	c	a	t
MH709348	2	a	g	a	c	c	t	c	a	-	t	t	t	g	g	t	c	c	t	a	t	a	c	g	t	t	t	t	t	a	a	t	c	t	a	t	t	t	a	t	c	c	g	g	t	c	t	a	t	c	g	c	g	t	a	a	t	c	c	g	a	t	g	g	t	t	a	c	a	g	c	c	a	t
MH709355	2	a	g	a	c	c	t	c	a	-	t	t	t	g	g	t	c	c	t	a	t	a	c	g	t	t	t	t	t	a	a	t	c	t	a	t	t	t	a	t	c	c	g	g	t	c	t	a	t	c	g	c	g	t	a	a	t	c	c	g	a	t	t	g	t	t	a	c	a	g	c	c	a	t
MH709359	2	a	g	a	c	c	t	c	a	-	t	t	t	g	g	t	c	c	t	a	t	a	c	g	t	t	t	t	t	a	a	t	c	t	a	t	t	t	a	t	c	c	g	g	t	c	t	a	t	c	g	c	g	t	a	a	t	c	c	g	a	t	g	g	t	t	a	c	a	g	c	c	a	t
MH709363	2	a	g	a	c	c	t	c	a	-	t	t	t	g	g	t	c	c	t	a	t	a	c	g	t	t	t	t	t	a	a	t	c	t	a	t	t	t	a	t	c	c	g	g	t	c	t	a	t	c	g	c	g	t	a	a	t	c	c	g	a	t	g	g	t	t	a	t	a	g	c	c	a	t
MH709366	2	a	g	a	c	c	t	c	a	-	t	t	t	g	g	t	c	c	t	a	t	a	c	g	t	t	t	t	t	a	a	t	c	t	a	t	t	t	a	t	c	c	g	g	t	c	t	a	t	c	g	c	g	t	a	a	t	c	c	g	a	t	g	g	t	t	g	c	a	g	c	c	a	t
MH709376	2	a	g	a	c	c	t	c	a	-	t	t	t	g	g	t	c	c	t	a	t	a	c	g	t	t	t	t	t	a	a	t	c	t	a	t	t	t	a	t	c	c	g	g	t	c	t	a	t	c	g	c	g	t	a	a	t	c	c	a	a	t	g	g	t	t	a	c	a	g	c	c	a	t
MH709377	2	a	g	a	c	c	t	c	a	-	t	t	g	g	g	t	c	c	t	a	t	a	c	g	t	t	t	t	t	a	a	t	c	t	a	t	t	t	a	t	c	c	g	g	t	c	t	a	t	c	g	t	g	t	a	a	t	c	c	g	a	t	g	g	t	t	a	c	a	g	c	c	a	t
**MW316406**	2	a	g	-	-	-	t	c	a	-	t	t	t	g	g	t	c	c	t	a	t	a	c	g	t	t	t	t	t	a	a	t	c	t	a	t	t	t	a	t	c	c	g	g	t	c	t	a	t	c	g	c	g	t	a	a	t	c	c	g	a	t	g	g	t	t	a	c	a	g	c	c	a	t
AJ871403	3	a	g	a	c	c	t	c	a	-	t	t	t	a	g	t	c	c	t	a	t	a	c	g	t	t	t	t	t	a	a	t	c	t	a	t	t	t	a	t	c	c	g	g	t	c	t	a	t	c	g	c	g	t	a	a	t	c	c	g	a	t	g	g	t	t	a	c	a	g	t	c	a	t
DQ479955	3	a	g	a	c	c	t	c	a	-	t	t	t	a	g	t	c	c	t	a	t	a	c	g	t	t	t	t	t	a	a	t	c	t	a	t	t	t	a	t	c	c	g	g	t	c	t	a	t	c	g	c	g	t	a	a	t	c	c	g	a	t	g	g	t	t	a	c	a	g	t	c	a	t
DQ479956	3	a	g	a	c	c	t	c	a	-	t	t	t	a	g	t	c	c	t	a	t	a	c	g	t	t	t	t	t	a	a	t	c	t	a	t	t	t	a	t	c	c	g	g	t	c	t	a	t	c	g	c	g	t	a	a	t	c	c	g	a	t	g	g	t	t	a	c	a	g	t	c	a	t
DQ479957	3	a	g	a	c	c	t	c	a	-	t	t	t	a	g	t	c	c	t	a	t	a	c	g	t	t	t	t	t	a	a	t	c	t	a	t	t	t	a	t	c	c	g	g	t	c	t	a	t	c	g	c	g	t	a	a	t	c	c	g	a	t	g	g	t	t	a	c	a	g	t	c	a	t
JN704699	3	a	g	a	c	c	t	c	a	-	t	t	t	a	-	t	c	c	t	a	t	a	c	g	t	t	t	t	t	a	a	t	c	t	a	t	t	t	a	t	c	c	g	g	t	c	t	a	t	c	g	c	g	t	a	a	t	c	c	g	a	t	g	g	t	t	a	c	a	g	t	c	a	t
JN704700	3	a	g	a	c	c	t	c	a	-	t	t	t	a	g	t	c	c	t	a	t	a	c	g	t	t	t	t	t	a	a	t	c	t	a	t	t	t	a	t	c	c	g	g	t	c	t	a	t	c	g	c	g	t	a	a	t	c	c	g	a	t	g	g	g	t	a	c	a	g	t	c	a	t
JN704701	3	a	g	a	c	c	t	c	a	-	t	t	t	a	-	t	c	c	t	a	t	a	c	g	t	t	t	t	t	a	a	t	c	t	a	t	t	t	a	t	c	c	g	g	t	c	t	a	t	c	g	c	g	t	a	a	t	c	c	g	a	t	g	g	t	t	a	c	a	g	t	c	a	t
JN704702	3	a	g	a	c	c	t	t	a	-	t	t	t	a	g	t	c	c	t	a	t	a	c	g	t	t	t	t	t	a	a	t	c	t	a	t	t	t	a	t	c	c	g	g	t	c	t	a	t	c	g	c	g	t	a	a	t	c	c	g	a	t	g	g	t	t	a	c	a	g	t	c	a	t
JN704703	3	a	g	a	c	c	t	c	a	-	t	t	t	a	g	t	c	c	t	a	t	a	c	g	t	t	t	t	t	a	a	t	c	t	a	t	t	t	a	t	c	c	g	g	t	c	t	a	t	c	g	c	g	t	a	a	t	c	c	g	a	t	g	g	t	t	a	c	a	g	t	c	a	t
KP771891	3	a	g	a	c	c	t	c	a	-	t	t	t	a	g	t	c	c	t	a	t	a	c	g	t	t	t	t	t	a	a	t	c	t	a	t	t	t	a	t	c	c	g	g	t	c	t	a	t	c	g	c	g	t	a	a	t	c	c	g	a	t	g	g	t	t	a	c	a	g	t	c	a	t
KP771893	3	a	g	a	c	c	t	c	a	-	t	t	t	a	g	t	c	c	t	a	t	a	c	g	t	t	t	t	t	a	a	t	c	t	a	t	t	t	a	t	c	c	g	g	t	c	t	a	t	c	g	c	g	t	a	a	t	c	c	g	a	t	g	g	t	t	a	c	a	g	t	c	a	t
KP771895	3	a	g	a	c	c	t	c	a	-	t	t	t	a	g	t	c	c	t	a	t	a	c	g	t	t	t	t	t	a	a	t	c	t	a	t	t	t	a	t	c	c	g	g	t	c	t	a	t	c	g	c	g	t	a	a	t	c	c	g	a	t	g	g	t	t	a	c	a	g	t	c	a	t
KP771896	3	a	g	a	c	c	t	c	a	-	t	t	t	a	g	t	c	c	t	a	t	a	c	g	t	t	t	t	t	a	a	t	c	t	a	t	t	t	a	t	c	c	g	g	t	c	t	a	t	c	g	c	g	t	a	a	t	c	c	g	a	t	g	g	t	t	a	c	a	g	t	c	a	t
KP771897	3	a	g	a	c	c	t	c	a	-	t	t	t	a	g	t	c	c	t	a	t	a	c	g	t	t	t	t	t	a	a	t	c	t	a	t	c	t	a	t	c	c	g	g	t	c	t	a	t	c	g	c	g	t	a	a	t	c	c	g	a	t	g	g	t	t	a	c	a	g	t	c	a	t
KP771899	3	a	g	a	c	c	t	c	a	-	t	t	t	a	g	t	c	c	t	a	t	a	c	g	t	t	t	t	t	a	a	t	c	t	a	t	t	t	a	t	c	c	g	g	t	t	t	a	t	c	g	c	g	t	a	a	t	c	c	g	a	t	g	g	t	t	a	c	a	g	t	c	a	t
KP771903	3	a	g	a	c	c	t	c	a	-	t	t	t	a	g	t	c	c	t	a	t	a	c	g	t	t	t	t	t	a	a	t	c	t	a	t	t	t	a	t	c	c	g	g	t	t	t	a	t	c	g	c	g	t	a	a	t	c	c	g	a	t	g	g	t	t	a	c	a	g	t	c	a	t
KP771907	3	a	g	a	c	c	t	c	a	-	t	t	t	a	g	t	c	c	t	a	t	a	c	g	t	t	t	t	t	a	a	t	c	t	a	t	t	t	a	t	c	c	g	g	t	c	t	a	t	c	g	c	g	t	a	a	t	c	c	g	a	t	g	g	t	t	a	c	a	g	t	c	a	t
KP771910	3	a	g	a	c	c	t	c	a	-	t	t	t	a	g	t	c	c	t	a	t	a	c	g	t	t	t	t	t	a	a	t	c	t	a	t	t	t	a	t	c	c	g	g	t	c	t	a	t	c	g	c	g	t	a	a	t	c	c	g	a	t	g	g	t	t	a	c	a	g	t	c	a	t
KP771915	3	a	g	a	c	c	t	c	a	-	t	t	t	a	g	t	c	c	t	a	t	a	c	g	t	t	t	t	t	a	a	t	c	t	a	t	c	t	a	t	c	c	g	g	t	c	t	a	t	c	g	c	g	t	a	a	t	c	c	g	a	t	g	g	t	t	a	c	a	g	t	c	a	t
KP771916	3	a	g	a	c	c	t	c	a	-	t	t	t	a	g	t	c	c	t	a	t	a	c	g	t	t	t	t	t	a	a	t	c	t	a	t	t	t	a	t	c	c	g	g	t	c	t	a	t	c	g	c	g	t	a	a	t	c	c	g	a	t	g	g	t	t	a	c	a	g	t	c	a	t
KP771921	3	a	g	a	c	c	t	c	a	-	t	t	t	a	g	t	c	c	t	a	t	a	c	g	t	t	t	t	t	a	a	t	c	t	a	t	t	t	a	t	c	c	g	g	t	c	t	a	t	c	g	c	g	t	a	a	t	c	c	g	a	t	g	g	t	t	a	c	a	g	t	c	a	t
KP771923	3	a	g	a	c	c	t	c	a	-	t	t	t	a	g	t	c	c	t	a	t	a	c	g	t	c	t	t	t	a	a	t	c	t	a	t	t	t	a	t	c	c	g	g	t	c	t	a	t	t	g	c	g	t	a	a	t	c	c	g	a	t	g	g	t	t	a	c	a	g	t	c	a	t
KP771889	3	a	g	a	c	c	t	c	a	-	t	t	t	a	g	t	c	c	t	a	t	a	c	g	t	t	t	t	t	a	a	t	c	t	a	t	c	t	a	t	c	c	g	g	t	c	t	a	t	c	g	c	g	t	a	a	t	c	c	g	a	t	g	g	t	t	a	c	a	g	t	c	a	t
KU926311	3	a	g	a	c	c	t	c	a	-	t	t	t	a	g	t	c	c	t	a	t	a	c	g	t	t	t	t	t	a	a	t	c	t	a	t	t	t	a	t	c	c	g	g	t	c	t	a	t	c	g	c	g	t	a	a	t	c	c	g	a	t	g	g	t	c	a	c	a	g	t	c	a	t
JQ911599	3	a	g	a	c	c	t	c	a	-	t	t	t	a	a	t	c	c	t	a	t	a	c	g	t	t	t	t	t	a	a	t	c	t	a	t	t	t	a	t	c	c	g	g	t	c	t	a	t	c	g	c	g	t	a	a	t	c	c	g	a	t	g	g	t	t	a	c	a	g	c	c	a	t
MH709317	3	a	g	a	c	c	t	c	a	-	t	t	t	a	g	t	c	c	t	a	t	a	c	g	t	t	t	t	t	a	a	t	c	t	a	t	t	t	a	t	c	c	g	g	t	c	t	a	t	c	g	c	g	t	a	a	t	c	c	g	a	t	g	g	t	t	a	c	a	g	c	c	a	t
MH709346	3	a	g	a	c	c	t	c	a	-	t	t	t	a	g	t	c	c	t	a	t	a	c	g	t	t	t	t	t	a	a	t	c	t	a	t	t	t	a	t	c	c	g	g	t	c	t	a	t	c	g	c	g	t	a	a	t	c	c	g	a	t	g	g	t	t	a	c	a	g	c	c	a	t
MH709375	3	a	g	a	c	c	t	c	a	-	t	t	t	a	g	t	c	c	t	a	t	a	c	g	t	t	t	t	g	a	a	t	c	t	a	t	t	t	a	t	c	c	g	g	t	c	t	a	t	c	g	c	g	t	a	a	t	c	c	g	a	t	g	g	t	t	a	c	a	g	c	c	a	t
DQ452050	4	a	g	-	-	-	t	c	a	-	t	t	t	g	g	t	c	c	t	a	t	a	c	g	t	t	g	t	t	a	a	t	c	t	a	t	t	t	a	t	c	c	g	g	g	c	t	a	t	c	g	c	g	t	a	a	t	c	c	g	a	t	g	g	t	t	a	c	a	g	c	c	a	t
DQ479960	4	a	g	-	-	-	t	c	a	-	t	t	t	g	g	t	c	c	t	a	t	a	c	g	t	t	t	t	t	a	a	t	c	t	a	t	t	t	a	t	c	c	g	g	g	c	t	a	t	c	g	c	g	t	a	a	t	c	c	g	a	t	g	g	t	t	a	c	a	g	c	c	a	t
KP771898	4	a	g	a	c	c	t	c	a	-	t	t	t	g	g	t	c	c	t	a	t	a	c	g	t	t	t	t	t	a	a	t	c	t	a	t	t	t	a	t	c	c	g	g	g	c	t	a	c	c	g	c	g	t	a	a	t	c	c	g	a	t	g	g	t	t	a	c	a	g	c	c	a	t
MH709316	4	a	g	a	c	c	t	c	a	-	t	t	t	g	g	t	c	c	t	a	t	a	c	g	t	t	t	c	t	a	a	t	c	t	a	t	t	t	a	t	c	c	g	g	g	c	t	a	t	c	g	c	g	t	a	a	t	c	c	g	a	t	g	g	t	t	a	c	a	g	c	c	a	t
MH709318	4	a	g	a	c	c	t	c	a	-	t	t	t	g	g	t	c	c	t	a	t	a	c	g	t	t	t	t	t	c	a	t	c	t	a	t	t	t	a	t	c	c	g	g	g	c	t	a	t	c	g	c	g	t	a	a	t	c	c	g	a	t	g	g	t	t	a	c	a	g	c	c	a	t
MH709322	4	a	g	a	g	c	t	c	a	-	t	t	t	g	g	t	c	c	t	a	t	a	c	g	t	t	t	t	t	c	a	t	c	t	a	t	t	t	a	t	c	c	g	g	g	c	t	a	t	c	g	c	g	t	a	a	t	c	c	g	a	t	g	g	t	t	a	c	a	g	c	c	a	t
MH709323	4	a	g	a	c	c	t	c	a	-	t	t	t	g	g	t	c	c	t	a	t	a	c	g	t	t	t	t	t	a	a	t	c	t	a	t	t	t	a	t	c	c	g	g	g	c	t	a	t	c	g	c	g	t	a	a	t	c	c	g	a	t	g	g	t	t	a	c	a	g	c	c	a	t
MH709326	4	a	g	a	c	c	t	c	a	-	t	t	t	g	g	t	c	c	t	a	t	a	c	g	t	t	t	t	t	c	a	t	c	g	a	t	t	t	a	t	c	c	g	g	g	c	t	a	t	c	g	c	g	t	a	a	t	c	c	g	a	t	g	g	t	t	a	c	a	g	c	c	a	t
MH709341	4	a	g	a	c	c	t	c	a	-	t	t	t	g	g	t	c	c	t	a	t	a	c	g	t	t	t	t	t	a	a	t	c	t	a	t	t	t	a	t	c	c	g	g	g	c	t	a	t	c	g	c	g	t	a	a	t	c	c	g	a	t	g	g	t	t	a	c	a	g	c	c	a	t
MH709357	4	a	g	a	g	t	t	c	a	-	t	t	t	g	g	t	c	c	t	a	t	a	c	g	t	t	t	t	t	a	a	t	c	t	a	t	t	t	a	t	c	c	g	g	g	c	t	a	t	c	g	c	g	t	a	a	t	c	c	g	a	t	g	g	t	t	a	c	a	g	c	c	a	t
MH709364	4	a	g	-	-	-	t	c	a	-	t	t	t	g	g	t	c	c	t	a	t	a	c	g	t	t	t	t	t	a	a	t	c	t	a	t	t	t	a	t	c	c	g	g	g	c	t	a	t	c	g	c	g	t	a	a	t	c	c	g	a	t	g	g	t	t	a	c	a	g	c	c	a	t
MH709370	4	a	g	a	c	c	t	c	a	-	t	t	t	g	g	t	c	c	t	a	t	a	c	g	t	t	t	t	t	a	a	t	c	t	a	t	t	t	a	t	c	c	g	g	g	c	t	a	t	c	g	c	g	t	a	a	t	c	c	g	a	t	g	g	t	t	a	c	a	g	c	c	a	t
MH709371	4	a	g	-	-	-	t	c	a	-	t	t	t	g	g	t	c	c	t	a	t	a	c	g	t	t	t	t	t	a	a	t	c	t	a	t	t	t	a	t	c	c	g	g	g	c	t	a	t	c	g	c	g	t	a	a	t	c	c	g	a	t	g	g	t	t	a	c	a	g	c	c	a	t
MH709372	4	a	g	a	c	c	t	c	a	-	t	t	t	g	g	t	c	c	t	a	t	a	c	g	t	t	t	t	t	a	a	t	c	t	a	t	t	t	a	t	c	c	g	g	g	c	t	a	t	c	g	c	g	t	a	a	t	c	c	g	a	t	g	g	t	t	a	c	a	g	c	c	a	t
DQ457052	5	a	g	a	c	c	t	c	a	-	t	t	t	a	g	t	c	c	t	a	t	a	c	g	t	t	t	t	t	a	a	t	c	t	a	t	t	t	a	t	c	c	g	g	t	c	t	a	t	c	g	c	g	g	a	a	t	c	c	g	a	t	g	g	t	t	a	c	a	g	c	c	a	t
JN704704	5	a	g	a	c	c	t	c	a	-	t	t	t	a	g	t	c	c	t	a	t	a	c	g	t	t	t	t	t	a	a	t	c	t	a	t	t	t	a	t	c	c	g	g	t	c	t	a	t	c	g	c	g	g	a	a	t	c	c	g	a	t	g	g	t	t	a	c	a	a	c	c	a	t
JN704705	5	a	g	a	c	c	t	c	a	-	t	t	t	a	g	t	c	c	t	a	t	a	c	g	t	t	t	t	t	a	a	t	c	t	a	t	t	t	a	t	c	c	g	g	t	c	t	a	t	c	g	c	a	g	a	a	t	c	c	g	a	t	g	g	t	t	a	c	a	g	c	c	a	t
JN704706	5	a	g	a	c	c	t	c	a	-	t	t	t	a	g	t	c	c	t	a	t	a	c	g	t	t	t	t	t	a	a	t	c	t	a	t	t	t	a	t	c	c	g	g	t	c	t	a	t	c	g	c	a	g	a	a	t	c	t	g	a	t	g	g	t	t	a	c	a	g	c	c	a	t
JN704707	5	a	g	a	c	c	t	c	a	-	t	t	t	a	g	t	c	c	t	a	t	a	c	g	t	t	t	t	t	a	a	t	c	t	a	t	t	t	a	t	c	c	g	g	t	c	t	a	t	c	g	c	g	g	a	a	t	c	c	g	a	t	g	g	t	t	a	c	a	a	c	c	a	t
JN704708	5	a	g	a	c	c	t	c	a	-	t	t	t	a	g	t	c	c	t	a	t	a	c	g	t	t	t	t	t	a	a	t	c	t	a	t	t	t	a	t	c	c	g	g	t	c	t	a	t	c	g	c	a	g	a	a	t	c	c	g	a	t	g	g	t	t	a	c	a	g	c	c	a	t
KP771904	5	a	g	a	c	c	t	c	a	-	t	t	t	a	g	t	c	c	t	a	t	a	c	g	t	t	t	t	t	a	a	t	c	t	a	t	t	t	a	t	c	c	g	g	t	c	t	a	t	c	g	c	g	g	a	a	t	c	c	g	a	t	g	g	t	t	a	c	a	g	c	c	a	t
KP771905	5	a	g	a	c	c	t	c	a	-	t	t	t	a	g	t	c	c	t	a	t	a	c	g	t	t	t	t	t	a	a	t	c	t	a	t	t	t	a	t	c	c	g	g	t	c	t	a	t	c	g	c	g	g	a	a	t	c	c	g	a	t	g	g	t	t	a	c	a	a	c	c	a	t
KP771906	5	a	g	a	c	c	t	c	a	-	t	t	t	a	g	t	c	c	t	a	t	a	c	g	t	t	t	t	t	a	a	t	c	t	a	t	t	t	a	t	c	c	g	g	t	c	t	a	t	c	g	c	g	g	a	a	t	c	c	g	a	t	g	g	t	t	a	c	a	a	c	c	a	t
KP771919	5	a	g	a	c	c	t	c	a	-	t	t	t	a	g	t	c	c	t	a	t	a	c	g	t	t	t	t	t	a	a	t	c	t	a	t	t	t	a	t	c	c	g	g	t	c	t	a	t	c	g	c	g	g	a	a	t	c	c	g	a	t	g	g	t	t	a	c	a	a	c	c	a	t
KP771922	5	a	g	a	c	c	t	c	a	-	t	t	t	a	g	t	c	c	t	a	t	a	c	g	t	t	t	t	t	a	a	t	c	t	a	t	t	t	a	t	c	c	g	g	t	c	t	a	t	c	g	c	a	g	a	a	t	c	c	g	a	t	g	g	t	t	a	c	a	g	c	c	a	t
KP771924	5	a	g	a	c	c	t	c	a	-	t	t	t	a	g	t	c	c	t	a	t	a	c	g	t	t	t	t	t	a	a	t	c	t	a	t	t	t	a	t	c	c	g	g	t	c	t	a	t	c	g	c	a	g	a	a	t	c	c	g	a	t	g	g	t	t	a	c	a	g	c	c	a	t
KM355696	5	a	g	a	c	c	t	c	a	-	t	t	t	a	g	t	c	c	t	a	t	a	c	g	t	t	t	t	t	a	a	t	c	t	a	t	t	t	a	t	c	c	g	g	t	c	t	a	t	c	g	c	a	g	a	a	t	c	c	g	a	t	g	g	t	t	a	c	a	g	c	c	a	t
KM355697	5	a	g	a	c	c	t	c	a	-	t	t	t	a	g	t	c	c	t	a	t	a	c	g	t	t	t	t	t	a	a	t	c	t	a	t	t	t	a	t	c	c	g	g	t	c	t	a	t	c	g	c	a	g	a	a	t	c	c	g	a	t	g	g	t	t	a	c	a	g	c	c	a	t
KM355698	5	a	g	a	c	c	t	c	a	-	t	t	t	a	g	t	c	c	t	a	t	a	c	g	t	t	t	t	t	a	a	t	c	t	a	t	t	t	a	t	c	c	g	g	t	c	t	a	t	c	g	c	a	g	a	a	t	c	c	g	a	t	g	g	t	t	a	c	a	g	c	c	a	t
KM355699	5	a	g	a	c	c	t	c	a	-	t	t	t	a	g	t	c	c	t	a	t	a	c	g	t	t	t	t	t	a	a	t	c	t	a	t	t	t	a	t	c	c	g	g	t	c	t	a	t	c	g	c	a	g	a	a	t	c	c	g	a	t	g	g	t	t	a	c	a	g	c	c	a	t
KM355701	5	a	g	a	c	c	t	c	a	-	t	t	t	a	g	t	c	c	t	a	t	a	c	g	t	t	t	t	t	a	a	t	c	t	a	c	t	t	a	t	c	c	g	g	t	c	t	a	t	c	g	c	a	g	a	a	t	c	c	g	a	t	g	g	t	t	a	c	a	g	c	c	a	t
KM355702	5	a	g	a	c	c	t	c	a	-	t	t	t	a	g	t	c	c	t	a	t	a	c	g	t	t	t	t	t	a	a	t	c	t	a	t	t	t	a	t	c	c	g	g	t	c	t	a	t	c	g	c	a	g	a	a	t	c	c	g	a	t	g	g	t	t	a	c	a	g	c	c	a	t
KM355703	5	a	g	a	c	c	t	c	a	-	t	t	t	a	g	t	c	c	t	a	t	a	c	g	t	t	t	t	t	a	a	t	c	t	a	t	t	t	a	t	c	c	g	g	t	c	t	a	t	c	g	c	a	g	a	a	t	c	c	g	a	t	g	g	t	t	a	c	a	g	c	c	a	t
KM355704	5	a	g	a	c	c	t	c	a	-	t	t	t	a	g	t	c	c	t	a	t	a	c	g	t	t	t	t	t	a	a	t	c	t	a	t	t	t	a	t	c	c	g	g	t	c	t	a	t	c	g	c	a	g	a	a	t	c	c	g	a	t	g	g	t	t	a	c	a	g	c	c	a	t
KM355705	5	a	g	a	c	c	t	c	a	-	t	t	t	a	g	t	c	c	t	a	t	a	c	g	t	t	t	t	t	a	a	t	c	t	a	t	t	t	a	t	c	c	g	g	t	c	t	a	t	c	g	c	a	g	a	a	t	c	c	g	a	t	g	g	t	t	a	c	a	g	c	c	a	t
KM355706	5	a	g	a	c	c	t	c	a	-	t	t	t	a	g	t	c	c	t	a	t	a	c	g	t	t	t	t	t	a	a	t	c	t	a	t	t	t	a	t	c	c	g	g	t	c	t	a	t	c	g	c	a	g	a	a	t	c	c	g	a	t	g	g	t	t	a	c	a	g	c	c	a	t
KM355707	5	a	g	a	c	c	t	c	a	-	t	t	t	a	g	t	c	c	t	a	t	a	c	g	t	t	t	t	t	a	a	t	c	t	a	t	t	t	a	t	c	c	g	g	t	c	t	a	t	c	g	c	a	g	a	a	t	c	c	g	a	t	g	g	t	t	a	c	a	g	c	c	a	t
KM355708	5	a	g	a	c	c	t	c	a	-	t	t	t	a	g	t	c	c	t	a	t	a	c	g	t	t	t	t	t	a	a	t	c	t	a	t	t	t	a	t	c	c	g	g	t	c	t	a	t	c	g	c	a	g	a	a	t	c	c	g	a	t	g	g	t	t	a	c	a	g	c	c	a	t
KM355709	5	a	g	a	c	c	t	c	a	-	t	t	t	a	g	t	c	c	t	a	t	a	c	g	t	t	t	t	t	a	a	t	c	t	a	t	t	t	a	t	c	c	g	g	t	c	t	a	t	c	g	c	a	g	a	a	t	c	c	g	a	t	g	g	t	t	a	c	a	g	c	c	a	t
KM355711	5	a	g	a	c	c	t	c	a	-	t	t	t	a	g	t	c	c	t	a	t	a	c	g	t	t	t	t	t	a	a	t	c	t	a	t	t	t	a	t	c	c	g	g	t	c	t	a	t	c	g	c	a	g	a	a	t	c	c	g	a	t	g	g	t	t	a	c	a	g	c	c	a	t
KM355712	5	a	g	a	c	c	t	c	a	-	t	t	t	a	g	t	c	c	t	a	t	a	c	g	t	t	t	t	t	a	a	t	c	t	a	t	t	t	a	t	c	c	g	g	t	c	t	a	t	c	g	c	a	g	a	a	t	c	c	g	a	t	g	g	t	t	a	c	a	g	c	c	a	t
KY037797	5	[illegible]	[illegible]	[illegible]	[illegible]	[illegible]	[illegible]	[illegible]	[illegible]	[illegible]	[illegible]	[illegible]	[illegible]	[illegible]	[illegible]	[illegible]	[illegible]	[illegible]	[illegible]	[illegible]	[illegible]	[illegible]	[illegible]	[illegible]	[illegible]	[illegible]	[illegible]	[illegible]	[illegible]	[illegible]	[illegible]	[illegible]	[illegible]	[illegible]	[illegible]	[illegible]	[illegible]	[illegible]	[illegible]	[illegible]	[illegible]	[illegible]	[illegible]	[illegible]	[illegible]	[illegible]	[illegible]	[illegible]	[illegible]	[illegible]	[illegible]	[illegible]	[illegible]	[illegible]	[illegible]	[illegible]	[illegible]	[illegible]	[illegible]	[illegible]	[illegible]	[illegible]	[illegible]	[illegible]	[illegible]	[illegible]	[illegible]	[illegible]	[illegible]	[illegible]	[illegible]	[illegible]	[illegible]	[illegible]

Supplementary Table 3: 2880 SN

GB-ID	Clade markers																					9																		1														5																				
	Position in Dumas	86258	86441	86480	86484	86485	86486	86497	86516	86523_1	86534	86556	86589	86620	86656	86671	86777	86782	86826	86866	86869	86893	86932	86966	86986	87016	87063	87095	87130	87251	87280	87306	87352	87415	87463	87489	87543	87747	87815	87841	88000	88124	88240	88405	88477	88681	88744	88780	88792	88846	88855	88867	88966	88984	88997	89137	89182	89203	89362	89406	89522	89605	89618	89647	89662	89676	89692	89698	89734	89827	89905	89965	89986	90088
MH499467	5	a	g	a	c	c	t	c	a	-	t	t	t	a	g	t	c	c	t	a	t	a	c	g	t	t	t	t	t	a	a	t	c	t	a	t	t	t	a	t	c	c	g	g	t	c	t	a	t	c	g	c	g	g	a	a	t	c	c	g	a	t	g	g	t	t	a	c	a	a	c	c	a	t
MH499469	5	a	g	a	c	c	t	c	a	-	t	t	t	a	g	t	c	c	t	a	t	a	c	g	t	t	t	t	t	a	a	t	c	t	a	t	t	t	a	t	c	c	g	g	t	c	t	a	t	c	g	c	g	g	a	a	t	c	c	g	a	t	g	g	t	t	a	c	a	a	c	c	a	t
MH709321	5	a	g	g	c	c	t	c	a	-	t	t	t	a	g	t	c	c	t	a	t	a	c	g	t	t	t	t	t	a	a	t	c	t	a	t	t	t	a	t	c	c	g	g	t	c	t	a	t	c	g	c	g	g	a	a	t	c	c	g	a	t	g	g	t	t	a	c	a	g	c	c	a	t
MH709324	5	a	g	a	c	c	t	c	a	-	t	t	t	a	g	t	c	c	t	a	t	a	c	g	t	t	t	t	t	a	a	t	c	t	a	t	t	t	a	t	c	c	g	g	t	c	t	a	t	c	g	c	g	g	a	a	t	c	c	g	a	t	g	g	t	t	a	c	a	a	c	c	a	t
MH709325	5	a	g	a	c	c	t	c	a	-	t	t	t	a	g	t	c	c	t	a	t	a	c	g	t	t	t	t	t	a	a	t	c	t	a	t	t	t	a	t	c	c	g	g	t	c	t	a	t	c	g	c	a	g	a	a	t	c	c	g	a	t	g	g	t	t	a	c	a	g	c	c	a	t
MH709327	5	a	g	a	c	c	t	c	a	-	t	t	t	a	g	t	c	c	t	a	t	a	c	g	t	t	t	t	t	a	a	t	c	t	a	t	t	t	a	t	c	c	g	g	t	c	t	a	t	c	g	c	g	g	a	a	t	c	c	g	a	t	g	g	t	t	a	c	a	a	c	c	a	t
MH709328	5	a	g	a	c	c	t	c	a	-	t	t	t	a	g	t	c	c	t	a	t	a	c	g	t	t	t	t	t	a	a	t	c	t	a	t	t	t	a	t	c	c	g	g	t	c	t	a	t	c	g	c	g	g	a	a	t	c	c	g	a	t	g	g	t	t	a	c	a	a	c	c	a	t
MH709330	5	a	g	a	c	c	t	c	a	-	t	t	t	a	g	t	c	c	t	a	t	a	c	g	t	t	t	t	t	a	a	t	t	t	a	t	t	t	a	t	c	c	g	g	t	c	t	a	t	c	g	c	g	g	a	a	t	c	c	g	a	t	g	g	t	t	a	c	a	a	c	c	a	t
MH709332	5	a	g	a	c	c	t	c	a	-	t	t	t	a	g	t	c	c	t	a	t	a	c	g	t	t	t	t	t	a	a	t	c	t	a	t	t	t	a	t	c	c	g	g	t	c	t	a	t	c	g	c	g	g	a	a	t	c	c	g	a	t	g	g	t	t	a	c	a	a	c	c	a	t
MH709334	5	a	g	a	c	c	t	c	a	-	t	t	t	a	g	t	c	c	t	a	t	a	c	g	t	t	t	t	t	a	a	t	c	t	a	t	t	t	a	t	c	c	g	g	t	c	t	a	t	c	g	c	g	g	a	a	t	c	c	g	a	t	g	g	t	t	a	c	a	a	c	c	a	t
MH709335	5	a	g	a	c	c	t	c	a	-	t	t	t	a	g	t	c	c	t	a	t	a	c	g	t	t	t	t	t	a	a	t	c	t	a	t	t	t	a	t	c	c	g	g	t	c	t	a	t	c	g	c	g	g	a	a	t	c	c	g	a	t	g	g	t	t	a	c	a	a	c	c	a	t
MH709336	5	a	g	a	c	c	t	c	a	-	t	t	t	a	g	t	c	c	t	a	t	a	c	g	t	t	t	t	t	a	a	t	c	t	a	t	t	t	a	t	c	c	g	g	t	c	t	a	t	c	g	c	g	g	a	a	t	c	c	g	a	t	g	g	t	t	a	c	a	a	c	c	a	t
MH709338	5	a	g	a	c	c	t	c	a	-	t	t	t	a	g	t	c	c	t	a	t	a	c	g	t	t	t	t	t	a	a	t	c	t	a	t	t	t	a	t	c	c	g	g	t	c	t	a	t	c	g	c	g	g	a	a	t	c	c	g	a	t	g	g	t	t	a	c	a	a	c	c	a	t
MH709340	5	a	g	a	c	c	t	c	a	-	t	t	t	a	g	t	c	c	t	a	t	a	c	g	t	t	t	t	t	a	a	t	c	t	a	t	t	t	a	t	c	c	g	g	t	c	t	a	t	c	g	c	g	g	a	a	t	c	c	g	a	t	g	g	t	t	a	c	a	g	c	c	a	t
MH709342	5	a	g	a	c	c	t	c	a	-	t	t	t	a	g	t	c	c	t	a	t	a	c	g	t	t	t	t	t	a	a	t	c	t	a	t	t	t	a	t	c	c	g	g	t	c	t	a	t	c	g	c	g	g	a	a	t	c	c	g	a	t	g	g	t	t	a	c	a	g	c	c	a	t
MH709343	5	a	g	a	c	c	t	c	a	-	t	t	t	a	g	t	c	c	t	a	t	a	c	g	t	t	t	t	t	a	a	t	c	t	a	t	t	t	a	t	c	c	g	g	t	c	t	a	t	c	g	c	g	g	a	a	t	c	c	g	a	t	g	g	t	t	a	c	a	a	c	c	a	t
MH709345	5	a	g	a	c	c	t	c	a	-	t	t	t	a	g	t	c	c	t	a	t	a	c	g	t	t	t	t	t	a	a	t	c	t	a	t	t	t	a	t	c	c	g	g	t	c	t	a	t	c	g	c	g	g	a	a	t	c	c	g	a	t	g	g	t	t	a	c	a	a	c	c	a	t
MH709347	5	a	g	a	c	c	t	c	a	-	t	t	t	a	g	t	c	c	t	a	t	a	c	g	t	t	t	t	t	a	a	t	c	t	a	t	t	t	a	t	c	c	g	g	t	c	t	a	t	c	g	c	g	g	a	a	t	c	c	g	a	t	g	g	t	t	a	c	a	a	c	c	a	t
MH709349	5	a	g	a	c	c	t	c	a	-	t	t	t	a	g	t	c	c	t	a	t	a	c	g	t	t	t	t	t	a	a	t	c	t	a	t	t	t	a	t	c	c	g	g	t	c	t	a	t	c	g	c	g	g	a	a	t	c	c	g	a	t	g	g	t	t	a	c	a	a	c	c	a	t
MH709350	5	a	g	a	c	c	t	c	a	-	t	t	t	a	g	t	c	c	t	a	t	a	c	g	t	t	t	t	t	a	a	t	c	t	a	t	t	t	a	t	c	c	g	g	t	c	t	a	t	c	g	c	g	g	a	a	t	c	c	g	a	t	g	g	t	t	a	c	a	a	c	c	a	t
MH709351	5	a	g	a	c	c	t	c	a	-	t	t	t	a	g	t	c	c	t	a	t	a	c	g	t	t	t	t	t	a	a	t	c	t	a	t	t	t	a	t	c	c	g	g	t	c	t	a	t	c	g	c	g	g	a	a	t	c	c	g	a	t	g	t	t	t	a	c	a	g	c	c	a	t
MH709353	5	a	g	a	c	c	t	c	a	-	t	t	t	a	g	t	c	c	t	a	t	a	c	g	t	t	t	t	t	a	a	t	c	t	a	t	t	t	a	t	c	c	g	g	t	c	t	a	t	c	g	c	g	g	a	a	t	c	c	g	a	t	g	g	t	t	a	c	a	a	c	c	a	t
MH709358	5	a	g	a	c	c	t	c	a	-	t	t	t	a	g	t	c	c	t	a	t	a	c	g	t	t	t	t	t	a	a	t	c	t	a	t	t	t	a	t	c	c	g	g	t	c	t	a	t	c	g	c	a	g	a	a	t	c	c	g	a	t	g	g	t	t	a	c	a	g	c	c	a	t
MH709360	5	a	g	a	c	c	t	c	a	-	t	t	t	a	g	t	c	c	t	a	t	a	c	g	t	t	t	t	t	a	a	t	c	t	a	t	t	t	a	t	c	c	g	g	t	c	t	a	t	c	g	c	g	g	a	a	t	c	c	g	a	t	g	g	t	t	a	c	a	a	c	c	a	t
MH709362	5	a	g	a	c	c	t	c	a	-	t	t	t	a	g	t	c	c	t	a	t	a	c	g	t	t	t	t	t	a	a	t	c	t	a	t	t	t	a	t	c	c	g	g	t	c	t	a	t	c	g	c	g	g	a	a	t	c	c	g	a	t	g	g	t	t	a	c	a	a	c	c	a	t
MH709365	5	a	g	a	c	c	t	c	a	-	t	t	t	a	g	t	c	c	t	a	t	a	c	g	t	t	t	t	t	a	a	t	c	t	a	t	t	t	a	t	c	c	g	g	t	c	t	a	t	c	g	c	g	g	a	a	t	c	c	g	a	t	g	g	t	t	a	c	a	a	c	c	a	t
MH709373	5	a	a	a	c	c	t	c	a	-	t	t	t	a	g	t	c	c	t	a	t	a	c	a	t	t	t	t	t	a	a	t	c	t	a	t	t	t	a	t	c	c	g	g	t	c	t	a	t	c	g	c	g	g	a	a	t	c	c	g	a	t	g	g	t	t	a	c	a	a	c	c	a	t
MH709374	5	a	g	a	c	c	t	c	a	-	t	t	t	a	g	t	c	c	t	a	t	a	c	g	t	t	t	t	t	a	a	t	c	t	a	t	t	t	a	t	c	c	g	g	t	c	t	a	t	c	g	c	g	g	a	a	t	c	c	g	a	t	g	g	t	t	a	c	a	a	c	c	a	t
KC112914	6	a	g	a	c	c	t	c	a	-	t	t	t	a	g	t	c	c	t	a	t	a	c	g	t	t	t	t	t	a	a	t	c	t	a	t	t	t	a	t	c	c	g	g	t	c	t	a	t	c	g	c	g	t	a	a	t	c	c	g	a	t	g	g	t	t	a	c	a	g	t	c	a	t
KP771914	6	a	g	a	c	c	t	c	a	-	t	t	t	a	g	t	c	c	t	a	t	a	c	g	t	t	t	t	t	a	a	t	c	t	a	t	t	t	a	t	c	c	g	g	t	c	t	a	t	c	g	c	g	t	a	a	t	c	c	g	a	t	g	g	t	t	a	c	a	g	t	c	a	t
KP702725	6	a	g	a	c	c	t	c	a	-	t	t	t	a	g	t	c	c	t	a	t	a	c	g	t	t	t	t	t	a	a	t	c	t	a	t	t	t	a	t	c	c	g	g	t	c	t	a	t	c	g	c	g	t	a	a	t	c	c	g	a	c	g	g	t	t	a	c	a	g	t	c	a	t
KP771890	9	a	g	a	c	c	t	c	a	-	t	t	t	a	g	t	c	c	t	a	t	g	c	g	t	t	t	t	t	a	a	t	c	t	a	t	t	t	a	t	c	c	g	g	t	c	t	c	t	c	g	c	g	t	a	a	t	c	c	g	c	t	g	g	t	t	a	c	a	g	c	c	a	t
JN704710	9	a	g	a	c	c	t	c	a	-	t	t	t	a	g	t	c	c	t	a	t	g	c	g	t	t	t	t	t	a	a	t	c	t	a	t	t	t	a	t	c	c	g	g	t	c	t	a	t	c	a	c	g	t	a	a	t	c	c	g	a	t	g	g	t	t	a	c	a	g	c	c	a	t
KY037798	9	a	g	a	c	c	t	c	a	-	t	t	t	a	g	t	c	c	t	a	t	g	c	g	t	t	t	t	t	a	a	t	c	t	a	t	t	t	a	t	c	c	g	g	t	c	t	a	t	c	g	c	g	t	a	a	t	c	c	g	a	t	g	g	t	t	a	c	a	g	c	c	a	t
MH709312	9	a	g	a	c	c	t	c	a	-	t	t	t	a	g	t	c	c	t	a	t	g	c	g	t	t	t	t	t	a	a	t	c	t	a	t	t	t	a	t	c	c	g	g	t	c	t	a	t	c	g	c	g	t	a	a	t	c	c	g	a	t	g	g	t	t	a	c	a	g	c	c	a	t
MH709352	9	a	g	a	c	c	t	c	a	t	t	t	t	a	g	t	c	c	t	a	t	g	c	g	t	t	t	t	t	a	a	t	c	t	a	t	t	t	a	t	c	c	g	g	t	c	t	a	t	c	g	c	g	t	a	a	t	c	c	g	a	t	g	g	t	t	a	c	a	g	c	c	a	t
AB097933	Vac	a	g	-	-	-	t	c	a	-	t	g	t	g	g	t	c	c	t	a	t	a	c	g	t	t	t	t	t	a	a	t	c	t	a	t	t	t	a	t	c	c	g	g	t	c	t	a	t	c	g	c	g	t	a	a	t	c	c	g	a	t	g	g	t	t	a	c	a	g	c	c	a	t
JN704698	Vac	a	g	-	-	-	t	c	a	-	t	g	t	g	g	t	c	c	t	a	t	a	c	g	t	t	t	t	t	a	a	t	c	t	a	t	t	t	a	t	c	c	g	g	t	c	t	a	t	c	g	c	g	t	a	a	t	c	c	g	a	t	g	g	t	t	a	c	a	g	c	c	a	t
JN704697	Vac	a	g	-	-	-	t	c	a	-	t	g	t	g	g	t	c	c	t	a	t	a	c	g	t	t	t	t	t	a	g	c	c	t	a	t	t	t	a	t	c	c	g	g	t	c	t	a	t	c	g	c	g	t	a	a	t	c	c	g	a	t	g	g	t	t	a	c	g	g	c	c	a	t
DQ008355	Vac	a	g	-	-	-	t	c	a	-	t	g	t	g	g	t	c	c	t	a	t	a	c	g	t	t	t	t	t	a	a	t	c	t	a	t	t	t	a	t	c	c	g	g	t	c	t	a	t	c	g	c	g	t	a	a	t	c	c	g	a	t	g	g	t	t	a	c	a	g	c	c	a	t
JF306641	Vac	a	g	-	-	-	t	c	a	-	t	g	t	g	g	t	c	c	t	a	t	a	c	g	t	t	t	t	t	a	a	t	c	t	a	t	t	t	a	t	c	c	g	g	t	c	t	a	t	c	g	c	g	t	a	a	t	c	c	g	a	t	g	g	t	t	a	c	g	g	c	c	a	t
AB097932	Vac	a	g	-	-	-	t	c	a	-	t	g	t	g	g	t	c	c	t	a	t	a	c	g	t	t	t	t	t	a	r	y	c	t	a	t	t	t	a	t	c	c	g	g	t	c	t	a	t	c	g	c	g	t	a	a	t	c	c	g	a	t	g	g	t	t	a	c	g	g	c	c	a	t
DQ008354	Vac	a	g	-	-	-	t	c	a	-	t	g	t	g	g	t	c	c	t	a	t	a	c	g	t	t	t	t	t	a	a	t	c	t	a	t	t	t	a	t	c	c	g	g	t	c	t	a	t	c	g	c	g	t	a	a	t	c	c	g	a	t	g	g	t	t	a	c	g	g	c	c	a	t
KU926312	Vac	a	g	-	-	-	t	c	a	-	t	g	t	g	g	t	c	c	t	a	t	a	c	g	t	t	t	t	t	a	a	t	c	t	a	t	t	t	a	t	c	c	g	g	t	c	t	a	t	c	g	c	g	t	a	a	t	c	c	g	a	t	g	g	t	t	a	c	g	g	c	c	a	t
KU926313	Vac	a	g	-	-	-	t	c	a	-	t	g	t	g	g	t	c	c	t	a	t	a	c	g	t	t	t	t	t	a	a	t	c	t	a	t	t	t	a	t	c	c	g	g	t	c	t	a	t	c	g	c	g	t	a	a	t	c	c	g	a	t	g	g	t	t	a	c	g	g	c	c	a	t
KU926314	Vac	a	g	-	-	-	t	c	a	-	t	g	t	g	g	t	c	c	t	a	t	a	c	g	t	t	t	t	t	a	a	t	c	t	a	t	t	t	a	t	c	c	g	g	t	c	t	a	t	c	g	c	g	t	a	a	t	c	c	g	a	t	g	g	t	t	a	c	a	g	c	c	a	t
KF558371	Vac	a	g	-	-	-	t	c	a	-	t	g	t	g	g	t	c	c	t	a	t	a	c	g	t	t	t	t	t	a	a	t	c	t	a	t	t	t	a	t	c	c	g	g	t	c	t	a	t	c	g	c	g	t	a	a	t	c	c	g	a	t	g	g	t	t	a	c	a	g	c	c	a	t
KF558372	Vac	a	g	-	-	-	t	c	a	-	t	g	t	g	g	t	c	c	t	a	t	a	c	g	t	t	t	t	t	a	a	t	c	t	a	t	t	t	a	t	c	c	g	g	t	c	t	a	t	c	g	c	g	t	a	a	t	c	c	g	a	t	g	g	t	t	a	c	a	g	c	c	a	t
KF558373	Vac	a	g	-	-	-	t	c	a	-	t	g	t	g	g	t	c	c	t	a	t	a	c	g	t	t	t	t	t	a	a	t	c	t	a	t	t	t	g	t	c	c	g	g	t	c	t	a	t	c	g	c	g	t	a	a	t	c	c	g	a	t	g	g	t	t	a	c	a	g	c	t	g	t
KF558374	Vac	a	g	-	-	-	t	c	a	-	t	g	t	g	g	t	c	c	t	a	t	a	c	g	t	t	t	t	t	a	a	t	c	t	a	t	t	t	a	t	c	c	g	g	t	c	t	a	t	c	g	c	g	t	a	a	t	c	c	g	a	t	g	g	t	t	a	c	a	g	c	t	a	t
KF558375	Vac	a	g	-	-	-	t	c	a	-	t	g	t	g	g	t	c	c	t	a	t	a	c	g	t	t	t	t	t	a	a	t	c	t	a	t	t	t	a	t	c	c	g	g	t	c	t	a	t	c	g	c	g	t	a	a	t	c	c	g	a	t	g	g	t	t	a	c	a	g	c	c	a	t
KF558376	Vac	a	g	-	-	-	t	c	a	-	t	g	t	g	g	t	c	c	t	a	t	a	c	g	t	t	t	t	t	a	a	t	c	t	a	t	t	t	g	t	c	c	g	g	t	c	t	a	t	c	g	c	g	t	a	a	t	c	c	g	a	t	g	g	t	t	a	c	a	g	c	c	a	t
KF558377	Vac	a	g	-	-	-	t	c	a	-	t	g	t	g	g	t	c	c	t	a	t	a	c	g	t	t	t	t	t	a	a	t	c	t	a	t	t	t	a	t	c	c	g	g	t	c	t	a	t	c	g	c	g	t	a	a	t	c	c	g	a	t	g	g	t	t	a	c	a	g	c	c	a	t
KF558378	Vac	a	g	-	-	-	t	c	a	-	t	g	t	g	g	t	c	c	t	a	t	a	c	g	t	t	t	t	t	a	a	t	c	t	a	t	t	t	a	t	c	c	g	g	t	c	t	a	t	c	g	c	g	t	a	a	t	c	c	g	a	t	g	g	t	t	a	c	a	g	c	c	a	t
KF558379	Vac	a	g	-	-	-	t	c	a	-	t	g	t	g	g	t	c	c	t	a	t	a	c	g	t	t	t	t	t	a	a	t	c	t	a	t	t	t	a	t	c	c	g	g	t	c	t	a	t	c	g	c	g	t	a	a	t	c	c	g	a	t	g	g	t	t	a	c	a	g	c	c	a	t
KF558380	Vac	a	g	-	-	-	t	c	a	-	t	g	t	g	g	t	c	c	t	a	t	a	c	g	t	t	t	t	t	a	a	t	c	t	a	t	t	t	a	t	c	c	g	g	t	c	t	a	t	c	g	c	g	t	a	a	t	c	c	g	a	t	g	g	t	t	a	c	a	g	c	c	a	t
KF558381	Vac	a	g	-	-	-	t	c	a	-	t	g	t	g	g	t	c	c	t	a	t	a	c	g	t	t	t	t	t	a	a	t	c	t	a	t	t	t	a	t	c	c	g	g	t	c	t	a	t	c	g	c	g	t	a	a	t	c	c	g	a	t	g	g	t	t	a	c	a	g	c	c	a	t
KF558382	Vac	a	g	-	-	-	t	c	a	-	t	g	t	g	g	t	c	c	t	a	t	a	c	g	t	t	t	t	t	a	a	t	c	t	a	t	t	t	g	t	c	c	g	g	t	c	t	a	t	c	g	c	g	t	a	a	t	c	c	g	a	t	g	g	t	t	a	c	a	g	c	c	a	t
KF558383	Vac	a	g	-	-	-	t	c	a	-	t	g	t	g	g	t	c	c	t	a	t	a	c	g	t	t	t	t	t	a	a	t	c	t	a	t	t	t	a	t	c	c	g	g	t	c	t	a	t	c	g	c	g	t	a	a	t	c	c	g	a	t	g	g	t	t	a	c	a	g	c	c	a	t
KF558384	Vac	a	g	-	-	-	t	c	a	-	t	g	t	g	g	t	c	c	t	a	t	a	c	g	t	t	t	t	t	a	a	t	c	t	a	t	t	t	a	t	c	c	g	g	t	c	t	a	t	c	g	c	g	t	a	a	t	c	c	g	a	t	g	g	t	t	a	c	a	g	c	c	a	t
KF558385	Vac	a	g	-	-	-	t	c	a	-	t	g	t	g	g	t	c	c	t	a	t	a	c	g	t	t	t	t	t	a	a	t	c	t	a	t	t	t	a	t	c	c	g	g	t	c	t	a	t	c	g	c	g	t	a	a	t	c	c	g	a	t	g	g	t	t	a	c	a	g	c	c	a	t
KF558386	Vac	a	g	-	-	-	t	c	a	-	t	g	t	g	g	t	c	c	t	a	t	a	c	g	t	t	t	t	t	a	a	t	c	t	a	t	t	t	a	t	c	c	g	g	t	c	t	a	t	c	g	c	g	t	a	a	t	c	c	g	a	t	g	g	t	t	a	c	a	g	c	c	a	t
KF558387	Vac	a	g	-	-	-	t	c	a	-	t	g	t	g	g	t	c	c	t	a	t	a	c	g	t	t	t	t	t	a	a	t	c	t	a	t	t	t	a	t	c	c	g	g	t	c	t	a	t	c	g	c	g	t	a	a	t	c	c	g	a	t	g	g	t	t	a	c	a	g	c	c	a	t
KF558388	Vac	a	g	-	-	-	t	c	a	-	t	g	t	g	g	t	c	c	t	a	t	a	c	g	t	t	t	t	t	a	a	t	c	t	a	t	t	t	a	t	c	c	g	g	t	c	t	a	t	c	g	c	g	t	g	a	t	c	c	g	a	t	g	g	t	t	a	c	a	g	c	c	a	t
KF558389	Vac	a	g	-	-	-	t	c	a	-	t	g	t	g	g	t	c	c	t	a	t	a	c	g	t	t	t	t	t	a	a	t	c	t	a	t	t	t	a	t	c	c	g	g	t	c	t	a	t	c	g	c	g	t	a	a	t	c	c	g	a	t	g	g	t	t	a	c	a	g	c	t	a	t
KF558390	Vac	a	g	-	-	-	t	c	a	-	t	g	t	g	g	t	c	c	t	a	t	a	c	g	t	t	t	t	t	a	a	t	c	t	a	t	t	t	a	t	c	c	g	g	t	c	t	a	t	c	g	c	g	t	a	a	t	c	c	g	a	t	g	g	t	t	a	c	a	g	c	c	a	t
KF558391	Vac	a	g	-	-	-	t	c	a	-	t	g	t	g	g	t	c	c	t	a	t	a	c	g	t	t	t	t	t	a	g	t	c	t	a	t	t	t	a	t	c	c	g	g	t	c	t	a	t	c	g	c	g	t	a	a	t	c	c	g	a	t	g	g	t	t	a	c	a	g	c	c	a	t
KF811485	Vac	a	g	-	-	-	t	c	a	-	t	g	t	g	g	t	c	c	t	a	t	a	c	g	t	t	t	t	t	a	a	t	c	t	a	t	t	t	a	t	c	c	g	g	t	c	t	a	t	c	g	c	g	t	a	a	t	c	c	g	a	t	g	g	t	t	a	c	a	g	c	c	a	t
KF853225	Vac	a	g	-	-	-	t	c	a	-	t	g	t	g	g	t	c	c	t	a	t	a	c	g	t	t	t	t	t	a	g	t	c	t	a	t	t	t	a	t	c	c	g	g	t	c	t	a	t	c	g	c	g	t	a	a	t	c	c	g	a	t	g	g	t	t	a	c	a	g	c	c	a	t
KF853226	Vac	a	g	-	-	-	t	c	a	-	t	g	t	g	g	t	c	c	t	a	t	a	c	g	t	t	t	t	t	a	a	t	c	t	a	t	t	t	a	t	c	c	g	g	t	c	t	a	t	c	g	c	g	t	a	a	t	c	c	g	a	t	g	g	t	t	a	c	a	g	c	c	a	t
KF853227	Vac	a	g	-	-	-	t	c	g	-	t	g	t	g	g	t	c	c	t	a	t	a	c	g	t	t	t	t	t	a	g	t	c	t	a	t	t	t	a	t	c	c	g	g	t	c	t	a	t	c	g	c	g	t	a	a	t	c	c	g	a	t	g	g	t	t	a	c	a	g	c	c	a	t
KF853228	Vac	a	g	-	-	-	t	c	a	-	t	g	t	g	g	t	c	c	t	a	t	a	c	g	t	t	t	t	t	a	g	t	c	t	a	t	t	t	a	t	c	c	g	g	t	c	t	a	t	c	g	c	g	t	a	a	t	c	c	g	a	t	g	g	t	t	a	c	a	g	c	c	a	t
KF853229	Vac	a	g	-	-	-	t	c	a	-	t	g	t	g	g	t	c	c	t	a	t	a	c	g	t	t	t	t	t	a	a	t	c	t	a	t	t	t	a	t	c	c	g	g	t	c	t	a	t	c	g	c	g	t	a	a	t	c	c	g	a	t	g	g	t	t	a	c	a	g	c	t	a	t
KF853230	Vac	a	g	-	-	-	t	c	a	-	t	g	t	g	g	t	c	c	t	a	t	a	c	g	t	t	t	t	t	a	g	t	c	t	a	t	t	t	g	t	c	c	g	g	t	c	t	a	t	c	g	c	g	t	a	a	t	c	c	g	a	t	g	g	t	t	a	c	a	g	c	c	a	t
KF853231	Vac	a	g	-	-	-	t	c	a	-	t	g	t	g	g	t	c	c	t	a	t	a	c	g	t	t	t	t	t	a	a	t	c	t	a	t	t	t	a	t	c	c	g	g	t	c	t	a	t	c	g	c	g	t	a	a	t	c	c	g	a	t	g	g	t	t	a	c	a	g	c	c	a	t
KF853232	Vac	a	g	-	-	-	t	c	a	-	t	g	t	g	g	t	c	c	t	a	t	a	c	g	t	t	t	t	t	a	a	t	c	t	a	t	t	t	a	t	c	c	g	g	t	c	t	a	t	c	g	c	g	t	a	a	t	c	c	g	a	t	g	g	t	t	a	c	a	g	c	c	a	t
KF853233	Vac	a	g	-	-	-	t	c	a	-	t	g	t	g	g	t	c	c	t	a	t	a	c	g	t	t	t	t	t	a	a	c	c	t	a	t	t	t	a	t	c	c	g	g	t	c	t	a	t	c	g	c	g	t	a	a	t	c	c	g	a	t	g	g	t	t	a	c	a	g	c	c	a	t
KF853234	Vac	a	g	-	-	-	t	c	g	-	t	g	t	g	g	t	c	c	t	a	t	a	c	g	t	t	t	t	t	a	g	t	c	t	a	t	t	t	a	t	c	c	g	g	t	c	t	a	t	c	g	c	g	t	a	a	t	c	c	g	a	t	g	g	t	t	a	c	a	g	c	c	a	t
KF853235	Vac	a	g	-	-	-	c	c	a	-	t	g	t	g	g	t	c	c	t	a	t	a	c	g	t	t	t	t	t	a	a	t	c	t	a	t	t	t	a	t	c	c	g	g	t	c	t	a	t	c	g	c	g	t	a	a	t	c	c	g	a	t	g	g	t	t	a	c	a	g	c	c	a	t
MF898328	Vac	a	g	-	-	-	t	c	a	-	t	g	t	g	g	t	c	c	t	a	t	a	c	g	t	t	t	t	t	a	r	y	c	t	a	t	t	t	a	t	c	c	g	g	t	c	t	a	t	c	g	c	g	t	a	a	t	c	c	g	a	t	g	g	t	t	a	c	g	g	c	c	a	t
JN704709	VIII	a	g	a	c	c	t	c	a	-	t	t	t	a	g	t	c	c	t	a	c	a	t	g	t	t	t	t	t	a	a	t	c	t	a	t	t	t	a	t	c	c	g	g	t	c	t	a	t	c	g	c	g	t	a	a	t	c	c	g	a	t	g	g	t	t	a	c	a	g	c	c	a	t

Supplementary Table 3: 2880 SN

GB-ID	Clade markers																						5										v														5																										
	Position in Dumas	90139	90202	90215	90217	90318	90340	90392	90417	90417_1	90418	90423	90424	90425	90437	90466	90467	90535	90621	90637	90648	90796	90810	90846	90891	90895	90903	90925	90948	91002	91063	91066	91191	91343	91524	91661	91770	91773	91831	91924	91953	91961	92026	92092	92118	92129	92348	92361	92375	92409	92432	92514	92523	92535	92544	92635	92640	92646	92670	92694	92725	92836	92861	92878	92879	92920	92999	93107	93108	93234	93319	93367	93368	93386
AY548170	1	a	t	t	t	t	g	g	a	-	g	g	g	t	t	a	a	a	c	a	t	g	t	c	t	a	a	c	t	t	c	a	c	g	g	a	t	c	a	a	c	a	a	a	a	a	g	t	a	g	t	a	a	a	c	c	a	t	c	a	c	c	c	t	t	t	t	c	t	a	g	g	g	t
AY548171	1	a	t	t	t	t	g	g	a	-	g	g	g	t	t	a	a	a	c	a	t	g	t	c	t	a	a	c	t	t	c	a	c	g	g	a	t	c	a	a	c	a	a	a	a	a	g	t	a	g	t	a	a	a	c	c	a	t	c	a	c	c	t	t	t	t	t	c	t	a	g	g	g	t
DQ479953	1	a	t	t	t	t	g	g	a	-	g	g	g	t	t	a	a	a	c	a	t	g	t	c	t	a	a	c	t	t	c	a	c	g	g	a	t	c	a	a	c	a	a	a	a	a	g	t	a	g	t	a	a	a	c	c	a	t	c	a	c	c	t	t	t	t	t	c	t	a	g	g	g	t
DQ479954	1	a	t	t	t	t	g	g	a	-	g	g	g	t	t	a	a	a	c	a	t	g	t	c	t	a	a	c	t	t	c	a	c	g	g	a	t	c	a	a	c	a	a	a	a	a	g	t	a	g	t	a	a	g	c	c	a	t	c	a	c	c	t	c	t	t	t	c	t	a	g	g	g	t
DQ479958	1	a	t	t	t	t	g	g	a	-	g	g	g	t	t	a	a	a	c	a	t	g	t	c	t	a	a	c	t	t	c	a	c	g	g	a	t	c	a	a	c	a	a	a	a	a	g	t	a	g	t	a	a	a	c	c	a	t	c	a	c	c	t	t	t	t	t	c	t	a	g	g	g	t
DQ479959	1	a	t	t	t	t	g	g	a	-	g	g	g	t	t	a	a	a	c	a	t	g	t	c	t	a	a	c	t	t	c	a	c	g	g	a	t	c	a	a	c	a	a	a	a	a	g	t	a	g	t	a	a	a	c	c	a	t	c	a	c	c	t	t	t	t	t	c	t	a	g	g	g	t
DQ479961	1	a	t	t	t	t	g	g	a	-	g	g	g	t	t	a	a	a	c	a	t	g	t	c	t	a	a	c	t	t	c	a	c	g	g	a	t	c	a	a	c	a	a	a	a	a	g	t	a	g	t	a	a	g	c	c	a	t	c	a	c	c	t	t	t	t	t	c	t	a	g	g	g	t
DQ479962	1	a	t	t	t	t	g	g	a	-	g	g	g	t	t	a	a	a	c	a	t	g	t	c	t	a	a	c	t	t	c	a	c	g	g	a	t	c	a	a	c	a	a	a	a	a	g	t	a	g	t	a	a	g	c	c	a	t	c	a	c	c	t	t	t	t	t	c	t	a	g	g	g	t
DQ479963	1	a	t	t	t	t	g	g	a	-	g	g	g	t	t	a	a	a	c	a	t	g	t	c	t	a	a	c	t	t	c	a	c	g	g	a	t	c	a	a	c	a	a	a	a	a	g	t	a	g	t	a	a	g	c	c	a	t	c	a	c	c	t	t	t	t	t	c	t	a	g	g	g	t
DQ674250	1	a	t	t	t	t	g	g	a	-	g	g	g	t	t	a	a	a	c	a	t	g	t	c	t	a	a	c	t	t	c	a	c	g	g	a	t	c	a	a	c	a	a	a	a	a	g	t	a	g	t	a	a	a	c	c	a	t	c	a	c	c	t	t	t	t	t	c	t	a	g	g	g	t
KX262864	1	a	t	t	t	t	g	g	a	-	g	g	g	t	t	a	a	a	c	a	t	g	t	c	t	a	a	c	t	t	c	a	c	g	g	a	t	c	a	a	c	a	a	a	a	a	g	t	a	g	t	a	a	a	c	c	a	t	c	a	c	c	t	t	t	t	t	c	t	a	g	g	g	t
KX262865	1	a	t	t	t	t	g	g	a	-	g	g	g	t	t	a	a	a	c	a	t	g	t	c	t	a	a	c	t	t	c	a	c	g	g	a	t	c	a	a	c	a	a	a	a	a	g	t	a	g	t	a	a	a	c	c	a	t	c	a	c	c	t	t	t	t	t	c	t	a	g	g	g	t
KX262866	1	a	t	t	t	t	g	g	a	-	g	g	g	t	t	a	a	a	c	a	t	g	t	c	t	a	a	c	t	t	c	a	c	g	g	a	t	c	a	a	c	a	a	a	a	a	g	t	a	g	t	a	a	a	c	c	a	t	c	a	c	c	t	t	t	t	t	c	t	a	g	g	g	t
EU154348	1	a	t	t	t	t	g	g	a	-	g	g	g	t	t	a	a	a	c	a	t	g	t	c	t	a	a	c	t	t	c	a	c	g	g	a	t	c	a	a	c	a	a	a	a	a	g	t	a	g	t	a	a	g	c	c	a	t	c	a	c	c	t	t	t	t	t	c	t	a	g	g	g	t
JN704690	1	a	t	t	t	t	g	g	a	-	g	g	g	t	t	a	a	a	c	a	t	g	t	c	t	a	a	c	t	t	c	a	c	g	g	a	t	c	a	a	c	a	a	a	a	a	g	t	a	g	t	a	a	a	c	c	a	t	c	a	c	c	t	t	t	t	t	c	t	a	g	g	g	t
JN704691	1	a	t	t	t	t	g	g	a	-	g	g	g	t	t	a	a	a	c	a	t	g	t	c	t	a	a	c	t	t	c	a	c	g	g	a	t	c	a	a	c	a	a	a	a	a	g	t	a	g	t	a	a	a	c	c	a	t	c	a	c	c	t	t	t	t	t	c	t	a	g	g	g	t
JN704692	1	a	t	t	t	t	g	g	a	-	g	g	g	t	t	a	a	a	c	a	t	g	t	c	t	a	a	t	t	t	c	a	c	g	g	a	t	c	a	a	c	a	a	a	a	a	g	t	a	g	t	a	a	a	c	c	a	t	c	a	c	c	t	t	t	t	t	c	t	a	g	g	g	t
JN704693	1	a	t	t	t	t	g	g	a	-	g	g	g	t	t	a	a	a	c	a	t	g	t	c	t	a	g	c	t	c	c	a	c	g	g	a	t	c	a	a	c	a	a	a	a	a	g	t	a	g	t	a	a	a	c	c	a	t	c	a	c	c	t	t	t	t	t	c	t	a	g	g	g	t
JN704694	1	a	g	t	t	t	g	g	a	-	g	g	g	t	t	a	a	a	c	a	t	g	t	c	t	a	a	c	t	t	c	a	c	g	g	a	t	c	a	a	c	a	a	a	a	a	g	t	a	g	t	a	a	a	c	c	a	t	c	a	c	c	c	t	t	t	t	c	t	a	g	g	g	t
JN704695	1	a	t	t	t	t	g	g	a	-	g	g	g	t	t	a	a	a	c	a	t	g	t	c	t	a	a	c	t	t	c	a	c	g	g	a	t	c	a	a	c	a	a	a	a	a	g	t	a	g	t	a	a	a	c	c	a	t	c	a	c	c	t	t	t	t	t	c	t	a	g	g	g	t
JN704696	1	a	t	t	t	t	g	g	a	-	g	g	g	t	t	a	a	a	c	a	t	g	t	c	t	a	a	c	t	t	c	a	c	g	g	a	t	c	a	a	c	a	a	a	a	a	g	t	a	g	t	a	a	a	c	c	a	t	c	a	c	c	t	t	t	t	t	c	t	a	g	g	g	t
NC001348	1	a	g	t	t	t	g	g	a	-	g	g	g	t	t	a	a	a	c	a	t	g	t	c	t	a	a	c	t	t	c	a	c	g	g	a	t	c	a	a	c	a	a	a	a	a	g	t	a	g	t	a	a	a	c	c	a	t	c	a	c	c	t	t	t	t	t	c	t	a	g	g	g	t
KP771892	1	a	t	t	t	t	g	g	a	-	g	g	g	t	t	a	a	a	c	a	t	g	t	c	t	a	a	c	t	t	c	a	c	g	g	a	t	c	a	a	c	a	a	a	a	a	g	t	a	g	t	a	a	a	c	c	a	t	c	a	c	c	t	t	t	t	t	c	t	a	g	g	g	t
KP771894	1	a	t	t	t	t	g	g	a	-	g	g	g	t	t	a	a	a	c	a	t	g	t	c	t	a	a	c	t	t	a	a	c	g	g	a	t	c	a	a	c	a	a	a	a	a	g	t	a	g	t	a	a	a	c	c	a	t	c	a	c	c	t	t	t	t	t	c	t	a	g	g	g	t
KP771900	1	a	t	t	t	t	g	g	a	-	g	g	g	t	t	a	a	a	c	a	t	g	t	c	t	a	a	c	t	t	c	a	c	g	g	a	t	c	a	a	c	a	a	a	a	a	g	t	a	g	t	a	a	a	c	c	a	t	c	a	c	c	t	t	t	t	t	c	t	a	g	g	g	t
KP771901	1	a	t	t	t	t	g	g	a	-	g	g	g	t	t	a	a	a	c	a	t	g	t	c	t	a	a	c	t	t	c	a	c	g	g	a	t	c	a	a	c	a	a	a	a	a	g	t	a	g	t	a	a	a	c	c	a	t	c	a	c	c	t	t	t	t	t	c	t	a	g	g	g	t
KP771902	1	a	t	t	t	t	g	g	a	-	g	g	g	t	t	a	a	a	c	a	t	g	t	c	t	a	a	c	t	t	c	a	c	g	g	a	t	c	a	a	c	a	a	a	a	a	g	t	a	g	t	a	a	a	c	c	a	t	c	a	c	c	t	t	t	t	t	c	t	a	g	g	g	t
KP771908	1	a	t	t	t	t	g	g	a	-	g	g	g	t	t	a	a	a	c	a	t	g	t	c	t	a	a	c	t	t	c	a	c	g	g	a	t	c	a	a	c	a	a	a	a	a	g	t	a	g	t	a	a	a	c	c	a	t	c	a	c	c	t	t	t	t	t	c	t	a	g	g	g	t
KP771912	1	a	t	t	t	t	g	g	a	-	g	g	g	t	t	a	a	a	c	a	t	g	t	c	t	a	a	c	t	t	c	a	c	g	g	a	t	c	a	a	c	a	a	a	a	a	g	t	a	g	t	a	a	a	c	c	a	t	c	a	c	c	t	t	t	t	t	c	t	a	g	g	g	t
KP771913	1	a	t	t	t	t	g	g	a	-	g	g	g	t	t	a	a	a	c	a	t	g	t	c	t	a	a	c	t	t	c	a	c	g	g	a	t	c	a	a	c	a	a	a	a	a	g	t	a	g	t	a	a	a	c	c	a	t	c	a	c	c	t	t	t	t	t	c	t	a	g	g	g	t
KP771917	1	a	t	t	t	t	g	g	a	-	g	g	g	t	t	a	a	a	c	a	t	g	t	c	t	a	a	c	t	t	c	a	c	g	g	a	t	c	a	a	c	a	a	a	a	a	g	t	a	g	t	a	a	a	c	c	a	t	c	a	c	c	t	t	t	t	t	c	t	a	g	g	g	t
KP771918	1	a	t	t	t	t	g	g	a	-	g	g	g	t	t	a	a	a	c	a	t	g	t	c	g	a	a	c	t	t	c	a	c	g	g	a	t	c	a	a	c	a	a	a	a	a	g	t	a	g	t	a	a	a	c	c	a	t	c	a	c	c	t	t	t	t	t	c	t	a	g	g	g	t
KP771920	1	a	t	t	t	t	g	g	a	-	g	g	g	t	t	a	a	a	c	a	t	g	t	c	t	a	a	c	t	t	c	a	c	g	g	a	t	c	a	a	c	a	a	a	a	a	g	t	a	g	t	a	a	a	c	c	a	t	c	a	c	c	c	t	t	t	t	c	t	a	g	g	g	t
MH709361	1	a	t	t	t	t	g	g	a	-	g	g	g	t	t	a	a	a	c	a	t	g	t	c	t	a	a	c	t	t	c	a	c	g	g	a	t	c	a	a	c	g	g	g	g	a	g	t	g	g	t	a	a	a	c	c	a	t	c	a	c	c	t	c	g	t	c	c	t	g	g	g	g	t
KU926318	2	a	t	t	c	t	g	a	a	-	g	g	g	t	t	g	a	a	c	a	t	g	t	c	t	a	a	c	t	t	c	a	c	g	g	a	t	c	a	a	c	a	g	g	a	a	g	t	g	g	t	a	a	a	c	c	a	t	c	a	c	c	t	t	t	t	c	c	t	a	g	g	g	t
KU926319	2	a	t	t	c	t	g	a	a	-	g	g	g	t	t	a	a	a	c	a	t	g	t	c	t	a	a	c	t	t	c	a	c	g	g	a	t	c	a	a	c	a	g	g	a	a	g	t	g	g	t	a	a	a	c	c	a	t	c	a	c	c	t	t	t	t	c	c	t	a	g	g	g	t
KU926320	2	g	t	t	c	t	g	a	a	-	g	g	g	t	t	a	a	a	c	a	t	g	t	c	t	a	a	c	t	t	c	a	c	g	g	a	t	c	a	a	c	a	g	g	a	a	g	t	g	g	t	a	a	a	c	c	a	t	c	a	c	t	t	t	t	t	c	c	t	a	g	g	g	t
KU926321	2	a	t	t	c	t	g	a	a	-	g	g	g	t	t	a	a	a	c	a	t	g	t	c	t	a	a	c	t	t	c	a	c	g	g	a	t	c	a	a	c	a	g	g	a	a	g	t	g	g	t	a	a	a	c	c	a	t	c	a	c	c	t	t	t	t	c	c	t	a	g	g	g	t
KU926322	2	a	t	t	c	t	g	a	a	-	g	g	g	t	t	a	a	a	c	a	t	g	t	c	t	a	a	c	t	t	c	a	c	g	g	a	t	c	a	a	c	a	g	g	a	a	g	t	g	g	t	a	a	a	c	c	a	t	c	a	c	t	t	t	t	t	c	c	t	a	g	g	g	t
KC847290	2	a	t	t	c	t	g	a	a	-	g	g	g	t	t	a	a	a	c	a	t	a	t	c	t	a	a	c	t	t	c	a	c	g	g	a	t	c	a	a	c	a	g	g	a	a	g	t	g	g	t	a	a	a	c	c	a	t	c	a	c	c	t	t	t	t	c	c	t	a	g	g	g	t
KJ767491	2	a	t	t	c	t	g	a	a	-	g	g	g	t	t	a	a	a	c	a	t	g	t	c	t	a	a	c	t	t	c	a	c	g	g	a	t	c	a	a	c	a	g	g	a	a	g	t	g	g	t	a	a	a	c	c	a	t	c	a	c	c	t	t	g	t	c	c	t	a	g	g	g	t
KJ767492	2	a	t	t	c	t	g	a	a	-	g	g	g	t	t	a	a	a	c	a	t	g	t	c	t	a	a	c	t	t	c	a	c	g	g	a	t	c	a	a	c	a	g	g	a	a	g	t	g	g	t	a	a	a	c	c	a	t	c	a	c	c	t	t	t	t	c	c	t	a	g	g	g	t
KJ808816	2	a	t	t	c	t	g	a	a	-	g	g	g	t	t	a	a	a	c	a	t	g	t	c	t	a	a	c	t	t	c	a	c	g	g	a	t	c	a	a	c	a	g	g	a	a	g	t	g	g	t	a	a	a	c	c	a	t	c	a	c	t	t	t	t	t	c	c	t	a	g	g	g	t
KP771925	2	a	t	t	c	t	g	a	a	-	g	g	g	t	t	a	a	a	c	a	t	g	t	c	t	a	a	c	t	t	c	a	c	g	g	a	t	c	a	a	c	a	g	g	a	a	g	t	g	g	t	a	a	a	c	c	a	t	c	a	c	c	t	t	t	t	c	c	t	a	g	g	g	t
KU926315	2	a	t	t	c	t	g	a	a	-	g	g	g	t	t	a	a	a	c	a	t	g	t	c	t	a	a	c	t	t	c	a	c	g	g	a	t	c	a	a	c	a	g	g	a	a	g	t	g	g	t	a	a	a	c	c	a	t	c	a	c	c	t	t	g	t	c	c	t	a	g	g	g	t
KU926316	2	a	t	t	c	t	g	a	a	-	g	g	g	t	t	a	a	a	c	a	t	g	t	c	t	a	a	c	t	t	c	a	c	g	g	a	t	c	a	a	c	a	g	g	a	a	g	t	g	g	t	a	a	a	c	c	a	t	c	a	c	c	t	t	g	t	c	c	t	a	g	g	g	t
KU926317	2	a	t	t	c	t	g	a	a	-	g	g	g	t	t	a	a	a	c	a	t	g	t	c	t	a	a	c	t	t	c	a	c	g	g	a	t	c	a	a	c	a	g	g	a	a	g	t	g	g	t	a	a	a	c	c	a	t	c	a	c	c	t	t	g	t	c	c	t	a	g	g	g	t
KY037796	2	a	t	t	c	t	g	a	a	-	g	g	g	t	t	a	a	a	c	a	t	g	t	c	t	a	a	c	t	t	c	a	c	g	g	a	t	c	a	a	c	a	g	g	a	a	g	t	g	g	t	a	a	a	c	c	a	t	c	a	c	c	t	t	t	t	c	c	t	a	g	g	g	t
MF004348	2	a	t	t	c	t	g	a	a	-	g	g	g	t	t	a	a	a	c	a	t	g	t	c	t	a	a	c	t	t	c	a	c	g	g	a	t	c	a	a	c	a	g	g	a	a	g	t	g	g	t	a	a	a	c	c	a	t	c	a	c	c	t	t	t	t	c	c	t	a	g	g	g	t
MH709309	2	a	t	t	c	t	g	a	a	-	g	g	g	t	t	a	a	a	c	a	t	g	t	c	t	a	a	c	t	t	c	a	c	g	g	a	t	c	a	a	c	a	g	g	a	a	g	t	g	g	t	a	a	a	c	c	a	t	c	a	c	c	t	t	t	t	c	c	t	a	g	g	g	t
MH709310	2	a	t	t	c	t	t	a	a	-	g	g	g	t	t	a	a	a	c	a	t	g	t	c	t	a	a	c	t	t	c	a	c	g	g	a	t	c	a	a	c	a	g	g	a	a	g	t	g	a	t	a	a	a	c	c	a	t	c	a	c	c	t	t	t	t	c	c	t	a	g	g	g	t
MH709311	2	a	t	t	c	t	g	a	a	-	g	g	g	t	t	a	a	a	c	a	t	g	t	c	t	a	a	c	t	t	c	a	c	g	g	a	t	c	a	a	c	a	g	g	a	a	g	t	g	g	t	a	a	a	c	c	a	t	c	a	c	c	t	t	t	t	c	c	t	a	g	g	g	t
MH709313	2	a	t	t	c	t	g	a	a	-	g	g	g	t	t	a	a	a	c	a	t	g	t	c	t	a	a	c	t	t	c	a	c	g	g	a	t	c	a	a	c	a	g	g	a	a	g	t	g	g	t	a	a	a	c	c	a	t	c	a	c	c	t	t	t	t	c	c	t	a	g	g	g	t
MH709314	2	a	t	t	c	t	g	a	a	-	g	g	g	t	t	a	a	a	c	a	t	g	t	c	t	a	a	c	t	t	c	a	c	g	g	a	t	c	a	a	c	a	g	g	a	a	g	t	g	g	t	a	a	a	c	c	a	t	c	a	c	c	t	t	t	t	c	c	t	a	g	g	g	t
MH709315	2	a	t	t	c	t	g	a	a	-	g	g	g	t	t	a	a	a	c	a	t	g	t	c	t	a	a	c	t	t	c	a	c	g	g	a	t	c	a	a	c	a	g	g	a	a	g	t	g	g	t	a	a	a	c	c	a	t	c	a	c	c	t	t	t	t	c	c	t	a	g	g	g	t
MH709319	2	a	t	t	c	t	g	a	a	-	g	g	g	t	t	a	a	a	c	a	t	g	t	c	t	a	a	c	t	t	c	a	c	g	g	a	t	c	a	a	c	a	g	g	a	a	g	t	g	g	t	a	a	a	c	c	a	t	c	a	c	c	t	t	t	t	c	c	t	a	g	g	g	t
MH709320	2	a	t	t	c	t	g	a	a	-	g	g	g	t	t	a	a	a	c	a	t	g	t	c	t	a	a	c	t	t	c	a	c	g	g	a	t	c	a	a	c	a	g	g	a	a	g	t	g	g	t	a	a	a	c	c	a	t	c	a	c	c	t	t	t	t	c	c	t	a	g	g	g	t
MH709329	2	a	t	t	c	t	g	a	a	-	g	g	g	t	t	a	a	a	c	a	t	g	t	c	t	a	a	c	t	t	c	a	c	g	g	a	t	c	a	a	c	a	g	g	a	a	g	t	g	g	t	a	a	a	c	c	a	t	c	a	c	c	t	t	t	t	c	c	t	a	g	g	g	t
MH709331	2	a	t	t	c	t	g	a	a	-	g	g	g	t	t	a	a	a	c	a	t	g	t	c	t	a	a	c	t	t	c	a	c	g	g	a	t	c	a	a	c	a	g	g	a	a	g	t	g	g	t	a	a	a	c	c	a	t	c	a	c	c	c	t	t	t	c	c	t	a	g	g	g	t
MH709333	2	a	t	t	c	t	g	a	a	-	g	g	g	t	t	a	a	a	c	a	t	g	t	c	t	a	a	c	t	t	c	a	c	g	g	a	t	c	a	a	c	a	g	g	a	a	g	t	g	g	t	a	a	a	c	t	a	t	c	a	c	c	t	t	t	t	c	c	t	a	g	g	g	t
MH709337	2	a	t	t	c	t	g	a	a	-	g	g	g	t	t	a	a	a	c	a	t	g	t	c	t	a	a	c	t	t	c	a	c	g	g	a	t	c	a	a	c	a	g	g	a	a	g	t	g	g	t	a	a	a	c	c	a	t	c	a	c	c	t	t	t	t	c	c	t	a	g	g	g	t
MH709339	2	a	t	t	c	t	g	a	a	-	g	g	g	t	t	a	a	a	c	a	t	g	t	c	t	a	a	c	t	t	c	a	c	g	g	a	t	c	a	a	c	a	g	g	a	a	g	t	g	g	t	a	a	a	c	c	a	t	c	a	c	c	t	t	t	t	c	c	t	a	g	g	g	t
MH709344	2	a	t	t	c	t	g	a	a	-	g	g	g	t	t	a	a	a	c	a	t	g	t	c	t	a	a	c	t	t	c	a	c	g	g	a	t	c	a	a	c	a	g	g	a	a	g	t	g	g	t	a	a	a	c	c	a	t	c	a	c	c	t	t	t	t	c	c	t	a	g	g	g	t
MH709348	2	a	t	t	c	t	g	a	a	-	g	g	g	t	t	a	a	a	c	a	t	g	t	c	t	a	a	c	t	t	c	a	c	g	g	a	t	c	a	a	c	a	g	g	a	a	g	t	g	g	t	a	a	a	c	c	a	t	c	a	c	c	t	t	t	g	c	c	t	a	g	g	g	t
MH709355	2	a	t	t	c	t	g	a	a	-	g	g	g	t	t	a	a	a	c	a	t	g	t	c	t	a	a	c	t	t	c	a	c	g	g	a	t	c	a	a	c	a	g	g	a	a	g	t	g	g	t	a	a	a	c	c	a	t	c	a	c	c	t	t	t	t	c	c	t	a	g	g	g	t
MH709359	2	a	t	t	c	t	g	a	a	-	g	g	g	t	t	a	a	a	c	a	t	g	t	c	t	a	a	c	t	t	c	a	c	g	g	a	t	c	a	a	c	a	g	g	a	a	g	t	g	g	t	a	a	a	c	c	a	t	c	a	c	c	t	t	t	t	c	c	t	a	g	g	g	t
MH709363	2	a	t	t	c	t	g	a	a	-	g	g	g	t	t	a	a	a	c	a	t	g	t	c	t	a	a	c	t	t	c	a	c	g	g	a	t	c	a	a	c	a	g	g	a	a	g	t	g	g	t	a	a	a	c	c	a	t	c	a	c	c	t	t	t	t	c	c	t	a	g	g	g	t
MH709366	2	a	t	t	c	t	g	a	a	-	g	g	g	t	t	a	a	a	c	a	t	g	t	c	t	a	a	c	t	t	c	a	c	g	g	a	t	c	a	a	c	a	g	g	a	a	g	t	g	g	t	a	a	a	c	c	a	t	c	a	c	c	t	t	t	t	c	c	t	a	g	g	g	t
MH709376	2	a	t	t	c	t	g	a	a	-	g	g	g	t	t	a	a	a	c	a	t	g	t	c	t	a	a	c	t	t	c	a	c	g	g	a	t	c	a	a	c	a	g	g	a	a	g	t	g	g	t	a	a	a	c	c	a	t	c	a	c	c	t	t	t	t	c	c	t	a	g	g	g	t
MH709377	2	a	t	t	c	t	g	a	a	-	g	g	g	t	t	a	a	a	c	a	t	g	t	c	t	a	a	c	t	t	c	a	c	g	g	a	t	c	a	a	c	a	g	g	a	a	g	t	g	g	t	a	a	a	c	c	a	t	c	a	c	c	t	t	t	t	c	c	t	a	g	g	g	t
**MW316406**	2	a	t	t	c	t	g	a	a	-	g	g	g	t	t	a	a	a	c	a	t	g	t	c	t	a	a	c	t	t	c	a	c	g	g	a	t	c	a	a	c	a	g	g	a	a	g	t	g	g	t	a	a	a	c	c	a	t	c	a	c	c	t	t	t	t	c	c	t	a	g	g	g	t
AJ871403	3	a	t	t	t	t	g	g	a	-	g	g	g	t	t	a	a	a	c	a	t	g	t	c	t	a	a	c	g	t	t	a	c	g	g	a	t	c	a	a	c	a	a	g	a	a	g	t	a	g	t	a	a	a	c	c	a	t	t	a	c	c	t	t	t	t	c	c	t	a	g	g	g	t
DQ479955	3	a	t	t	t	t	g	g	a	-	g	g	g	t	t	a	a	a	c	a	t	g	t	c	t	a	a	c	t	t	c	a	c	g	g	a	t	c	a	a	c	a	a	g	a	a	g	t	a	g	t	a	a	a	c	c	a	t	c	a	c	c	t	t	t	t	c	c	t	a	g	g	g	t
DQ479956	3	a	t	t	t	t	g	g	-	-	a	g	g	t	t	a	a	a	c	a	t	g	t	c	t	a	a	c	t	t	c	a	c	g	g	a	t	c	a	a	c	a	a	g	a	a	g	t	a	g	t	a	a	a	c	c	a	t	c	a	c	c	t	t	t	t	c	c	t	a	g	g	g	t
DQ479957	3	a	t	t	t	t	g	g	a	-	g	g	g	t	t	a	a	a	c	a	t	g	t	c	t	a	a	c	t	t	c	a	c	g	g	a	t	c	a	a	c	a	a	g	a	a	g	t	a	g	t	a	a	a	c	c	a	t	c	a	c	c	t	t	t	t	c	c	t	a	g	g	g	t
JN704699	3	a	t	t	t	t	g	g	-	-	g	g	g	t	t	a	a	a	c	a	t	g	t	c	t	a	a	c	t	t	c	a	c	g	g	a	t	c	a	a	c	a	a	g	a	a	g	t	a	g	t	a	a	a	c	c	a	t	c	a	c	c	t	t	t	t	c	c	t	a	g	g	g	t
JN704700	3	a	g	t	t	t	g	g	a	-	g	g	g	t	t	a	a	a	c	a	t	g	t	c	t	a	a	c	t	t	c	a	c	g	g	a	t	c	a	a	c	a	a	g	a	a	g	t	a	g	t	a	a	a	c	c	a	t	c	a	c	c	t	t	t	t	c	c	t	a	g	g	g	t
JN704701	3	a	t	t	t	t	g	g	-	-	g	g	g	t	t	a	a	a	c	a	t	g	t	c	t	a	a	c	t	t	c	a	c	g	g	a	t	c	a	a	c	a	a	g	a	a	g	t	a	g	t	a	a	a	c	c	a	t	c	a	c	c	t	t	t	t	c	c	t	a	g	g	g	t
JN704702	3	a	t	t	t	t	g	g	a	-	g	g	g	t	t	a	a	a	c	a	t	g	t	c	t	a	a	c	t	t	c	a	c	g	g	a	t	c	a	a	c	a	a	g	a	a	g	t	a	g	t	a	a	a	c	c	a	t	c	a	c	c	t	t	t	t	c	c	t	a	g	g	g	t
JN704703	3	a	t	t	t	t	g	g	a	-	g	g	g	t	t	a	a	a	c	a	t	g	t	c	t	a	a	c	t	t	c	a	c	g	g	a	t	c	a	a	c	a	a	g	a	a	g	t	a	g	c	a	a	a	c	c	a	t	c	a	c	c	t	t	t	t	c	c	t	a	g	g	g	t
KP771891	3	a	t	t	t	t	g	g	-	-	g	g	g	t	t	a	a	a	c	a	t	g	t	c	t	a	a	c	t	t	c	a	c	g	g	a	t	c	a	a	c	a	a	g	a	a	g	t	a	g	t	a	a	a	c	c	a	t	c	a	c	c	t	t	t	t	c	c	t	a	g	g	g	t
KP771893	3	a	t	t	t	t	g	g	-	-	g	g	g	t	t	a	a	a	c	a	t	g	t	c	t	a	a	c	t	t	c	a	c	g	g	a	t	c	a	a	c	a	a	g	a	a	g	t	a	g	t	a	a	a	c	c	a	t	c	a	c	c	t	t	t	t	c	c	t	a	g	g	g	t
KP771895	3	a	t	t	t	t	g	g	a	-	g	g	g	t	t	a	a	a	c	a	t	g	t	c	t	a	a	c	t	t	c	a	c	g	g	a	t	c	a	a	c	a	a	g	a	a	g	t	a	g	t	a	a	a	c	c	a	t	c	a	c	c	t	t	t	t	c	c	t	a	g	g	g	t
KP771896	3	a	t	t	t	t	g	g	a	-	g	g	g	t	t	a	a	a	c	a	t	g	t	c	t	a	a	c	t	t	c	a	c	a	g	a	t	c	a	a	c	a	a	g	a	a	g	c	a	g	t	a	a	a	c	c	a	t	c	a	c	c	t	t	t	t	c	c	t	a	g	g	g	t
KP771897	3	a	t	t	t	t	g	g	a	-	g	g	g	t	t	a	a	a	c	a	t	g	t	c	t	a	a	c	t	t	c	a	c	g	g	a	t	c	a	a	c	a	a	g	a	a	g	t	a	g	t	a	a	a	c	c	a	t	c	a	c	c	t	t	t	t	c	c	t	a	g	g	g	t
KP771899	3	a	t	t	t	t	g	g	a	-	g	g	g	t	t	a	a	a	c	a	t	g	t	c	t	a	a	c	t	t	c	a	c	g	g	a	t	c	a	a	c	a	a	g	a	a	g	t	a	g	t	a	a	a	c	c	a	t	c	a	c	c	t	t	t	t	c	c	t	a	g	g	g	t
KP771903	3	a	t	t	t	t	g	g	-	-	g	g	g	t	t	a	a	a	c	a	t	g	t	c	t	a	a	c	t	t	c	a	c	g	g	a	t	c	a	a	c	a	a	g	a	a	g	t	a	g	t	a	a	a	c	c	a	t	c	a	c	c	t	t	t	t	c	c	t	a	g	g	g	t
KP771907	3	a	t	t	t	t	g	g	a	-	g	g	g	t	t	a	a	a	c	a	t	g	t	c	t	a	a	c	t	t	c	a	c	g	g	a	t	c	a	a	c	a	a	g	a	a	g	t	a	g	t	a	a	a	c	c	a	t	c	a	c	c	t	t	t	t	c	c	t	a	g	g	g	t
KP771910	3	a	t	t	t	t	g	g	-	-	g	g	g	t	t	a	a	a	c	a	t	g	t	c	t	a	a	c	t	t	c	a	c	g	g	a	t	c	a	a	c	a	a	g	a	a	g	t	a	g	t	a	a	a	c	c	a	t	c	a	c	c	t	t	t	t	c	c	t	a	g	g	g	t
KP771915	3	a	t	t	t	t	g	g	a	-	g	g	g	t	t	a	a	a	c	a	t	g	t	c	t	a	a	c	t	t	c	a	c	g	g	a	t	c	a	a	c	a	a	g	a	a	g	t	a	g	t	a	a	a	c	c	a	t	c	a	c	c	t	t	t	t	c	c	t	a	g	g	g	t
KP771916	3	a	t	t	t	t	g	g	a	-	g	g	g	t	t	a	a	a	c	a	t	g	t	c	t	a	a	c	t	t	c	a	c	g	g	a	t	c	a	a	c	a	a	g	a	a	g	t	a	g	t	a	a	a	c	c	a	t	c	a	c	c	t	t	t	t	c	c	t	a	g	g	g	t
KP771921	3	a	t	t	t	t	g	g	-	-	g	g	g	t	t	a	a	a	c	a	t	g	t	c	t	a	a	c	t	t	c	a	c	g	a	a	t	c	a	a	c	a	a	g	a	a	g	t	a	g	t	a	a	a	c	c	a	t	c	a	c	c	t	t	t	t	c	c	t	a	g	g	g	t
KP771923	3	a	t	t	t	t	g	g	-	-	g	g	g	t	t	a	a	a	c	a	t	g	t	c	t	a	a	c	t	t	c	a	c	g	g	a	t	c	a	a	c	a	a	g	a	a	g	t	a	g	t	a	a	a	c	c	a	t	c	a	c	c	t	t	t	t	c	c	t	a	g	g	g	t
KP771889	3	a	t	t	t	t	g	g	a	-	g	g	g	t	t	a	a	a	c	a	t	g	t	c	t	a	a	c	t	t	c	a	c	g	g	a	t	c	a	a	c	a	a	g	a	a	g	t	a	g	t	a	a	a	c	c	a	t	c	a	c	c	t	t	t	t	c	c	t	a	g	g	g	t
KU926311	3	a	t	t	t	t	g	g	a	-	g	g	g	t	t	a	a	a	c	a	t	g	t	c	t	a	a	c	t	t	c	a	c	g	g	a	t	c	a	a	c	a	a	g	a	a	g	t	a	g	t	a	a	a	c	c	a	t	c	a	c	c	t	t	t	t	c	c	t	a	g	g	g	t
JQ911599	3	a	t	t	t	t	g	g	a	-	g	g	g	t	t	a	a	a	c	a	t	g	t	c	t	a	a	c	t	t	c	g	c	g	g	a	t	c	a	a	c	a	a	g	a	a	g	t	a	g	t	a	a	a	t	c	a	t	c	a	c	c	t	t	t	t	c	c	t	a	g	g	g	t
MH709317	3	a	t	t	t	t	g	a	a	-	g	g	g	t	t	a	a	a	c	a	t	g	t	c	t	a	a	c	t	t	c	a	c	g	g	a	t	c	a	a	c	a	g	g	a	a	g	t	a	g	t	a	a	a	c	c	a	t	c	a	c	c	t	t	t	t	c	c	t	a	g	g	g	t
MH709346	3	a	t	t	t	t	g	a	a	-	g	g	g	t	t	a	a	a	c	a	t	g	t	c	t	a	a	c	t	t	c	a	c	g	g	a	t	c	a	a	c	a	g	g	a	a	g	t	a	g	t	a	a	a	c	c	a	t	c	a	c	c	t	t	t	t	c	c	t	a	g	g	g	t
MH709375	3	a	t	t	t	t	g	a	a	-	g	g	g	t	t	a	a	a	c	a	t	g	t	c	t	a	a	c	t	t	c	a	c	g	g	a	c	c	a	a	t	a	g	g	a	a	g	t	a	g	t	a	a	a	c	c	a	t	c	a	c	c	t	t	t	t	c	c	t	a	g	g	g	t
DQ452050	4	a	t	t	c	t	g	a	a	-	-	g	g	t	t	a	a	a	c	a	t	g	t	c	t	a	a	c	t	t	c	a	c	g	g	a	t	c	a	a	c	a	g	g	a	a	g	t	a	g	t	a	g	a	c	c	a	t	c	a	c	c	t	t	t	t	c	c	t	a	g	a	a	t
DQ479960	4	a	t	t	c	t	g	a	a	-	-	g	g	t	t	a	a	a	t	a	t	g	t	c	t	a	a	c	t	t	c	a	c	g	g	a	t	c	a	a	c	a	g	g	a	a	g	t	a	g	t	a	g	a	c	c	a	t	c	a	c	c	t	t	t	t	c	c	t	a	g	a	a	t
KP771898	4	a	t	t	c	t	g	a	a	-	g	g	g	t	t	a	a	a	c	a	t	g	t	c	t	a	a	c	t	t	c	a	c	g	g	a	t	c	a	a	c	a	g	g	a	a	g	t	a	g	t	a	g	a	c	c	a	t	c	a	c	c	t	t	t	t	c	c	t	a	g	a	a	t
MH709316	4	a	t	t	c	t	g	a	a	-	g	g	g	t	t	a	a	a	c	a	t	g	t	c	t	a	a	c	t	t	c	a	c	g	g	a	t	c	a	a	c	a	g	g	a	a	g	t	a	g	t	a	g	a	c	c	a	t	c	a	c	c	t	t	t	t	c	c	t	a	g	a	a	t
MH709318	4	a	t	t	c	t	g	a	a	-	g	g	g	t	t	a	a	a	c	a	t	g	t	c	t	a	a	c	t	t	c	a	c	g	g	a	t	c	a	a	c	a	g	g	a	a	g	t	a	g	t	a	g	a	c	c	a	t	c	a	c	c	t	t	t	t	c	c	t	a	g	a	a	t
MH709322	4	a	t	t	c	t	g	a	a	-	g	g	g	t	t	a	a	a	c	a	t	g	t	c	t	a	a	c	t	t	c	a	c	g	g	a	t	c	a	a	c	a	g	g	a	a	g	t	a	g	t	a	g	a	c	c	a	t	c	a	c	c	t	t	t	t	c	c	t	a	g	a	a	t
MH709323	4	a	t	t	c	t	g	a	a	-	g	g	g	t	t	a	a	a	c	a	t	g	t	c	t	a	a	c	t	t	c	a	c	g	g	a	t	c	a	a	c	a	g	g	a	a	g	t	a	g	t	a	g	a	c	c	a	t	c	a	c	c	t	t	t	t	c	c	t	a	g	a	a	t
MH709326	4	a	t	t	c	t	g	a	a	-	g	g	g	t	t	a	a	a	c	a	t	g	t	c	t	a	a	c	t	t	c	a	c	g	g	a	t	t	a	a	c	a	g	g	a	a	g	t	a	g	t	a	g	a	c	c	a	t	c	a	c	c	t	t	t	t	c	c	t	a	g	a	a	t
MH709341	4	a	t	t	c	t	g	a	a	-	g	g	g	t	t	a	a	a	c	a	t	g	t	c	t	a	a	c	t	t	c	a	c	g	g	a	t	c	a	a	c	a	g	g	a	a	g	t	a	g	t	a	g	a	c	c	a	t	c	a	c	c	t	t	t	t	c	c	t	a	g	a	a	t
MH709357	4	a	t	t	c	t	g	a	a	-	g	g	g	t	t	a	a	a	c	a	t	g	t	c	t	a	a	c	t	t	c	a	c	g	g	a	t	c	a	a	c	a	g	g	a	a	g	t	a	g	t	a	g	a	c	c	a	t	c	a	c	c	t	t	t	t	c	c	t	a	g	a	a	t
MH709364	4	a	t	t	c	t	g	a	a	-	g	g	g	t	t	a	a	a	c	a	t	g	t	c	t	a	a	c	t	t	c	a	c	g	g	a	t	c	a	a	c	a	g	g	a	a	g	t	a	g	t	a	g	a	c	c	a	t	c	a	c	c	t	t	t	t	c	c	t	a	g	a	a	c
MH709370	4	a	t	t	c	t	g	a	a	-	g	t	t	t	t	a	a	a	c	a	t	g	t	c	t	a	a	c	t	t	c	a	c	g	g	a	t	c	a	a	c	a	g	g	a	a	g	t	a	g	t	a	g	a	c	c	a	t	c	a	c	c	t	t	t	t	c	c	t	a	g	a	a	t
MH709371	4	a	t	t	c	t	g	a	a	-	-	g	g	t	t	a	a	a	c	a	t	g	t	c	t	a	a	c	t	t	c	a	c	g	g	a	t	c	a	a	c	a	g	g	a	a	g	t	a	g	t	a	g	a	c	c	a	t	c	a	c	c	t	t	t	t	c	c	t	a	g	a	a	t
MH709372	4	a	t	t	c	t	g	a	a	-	g	g	g	t	t	a	a	a	c	a	t	g	t	c	t	a	a	c	t	t	c	a	c	g	g	a	t	c	a	a	c	a	g	g	a	a	g	t	a	g	t	a	g	a	c	c	a	t	c	a	c	c	t	t	t	t	c	c	t	a	g	a	a	t
DQ457052	5	a	t	t	c	t	g	a	a	-	g	g	g	t	t	a	a	a	c	a	t	g	g	c	t	a	a	c	t	t	c	a	c	g	g	a	t	c	a	a	c	a	g	g	a	a	a	t	a	g	t	a	a	a	c	c	a	t	c	a	c	c	t	t	t	t	c	c	t	a	g	a	a	t
JN704704	5	a	t	t	c	t	g	a	a	-	g	g	g	t	t	a	a	a	c	a	t	g	g	c	t	a	a	c	t	t	c	a	c	g	g	a	t	c	a	a	c	a	g	g	a	a	a	t	a	g	t	a	a	a	c	c	a	t	c	a	c	c	t	t	t	t	c	c	t	a	g	a	a	t
JN704705	5	a	t	t	c	t	g	a	a	-	g	g	g	t	t	a	a	a	c	a	t	g	g	c	t	a	a	c	t	t	c	a	c	g	g	a	t	c	a	a	c	a	g	g	a	a	a	t	a	g	t	a	a	a	c	c	a	t	c	a	c	c	t	t	t	t	c	c	t	a	g	a	a	t
JN704706	5	a	t	g	c	t	g	a	a	-	g	g	g	t	t	a	a	a	c	a	t	g	g	c	t	a	a	c	t	t	c	a	c	g	g	a	t	c	a	a	c	a	g	g	a	a	a	t	a	g	t	a	a	a	c	c	a	t	c	a	c	c	t	t	t	t	c	c	t	a	g	a	a	t
JN704707	5	a	t	t	c	t	g	a	a	-	g	g	g	t	t	a	a	a	c	a	t	g	g	c	t	a	a	c	t	t	c	a	c	g	g	a	t	c	a	a	c	a	g	g	a	a	a	t	a	g	t	a	a	a	c	c	a	t	c	a	c	c	t	t	t	t	c	c	t	a	g	a	a	t
JN704708	5	a	t	t	c	t	g	a	a	-	g	g	g	t	t	a	a	a	c	a	t	g	g	c	t	a	a	c	t	t	c	a	c	g	g	a	t	c	a	a	c	a	g	g	a	a	a	t	a	g	t	a	a	a	c	c	a	t	c	a	c	c	t	t	t	t	c	c	t	a	g	a	a	t
KP771904	5	a	t	t	c	t	g	a	a	-	g	g	g	t	t	a	a	a	c	a	t	g	g	c	t	a	a	c	t	t	c	a	c	g	g	a	t	c	a	a	c	a	g	g	a	a	a	t	a	g	t	a	a	a	c	c	a	t	c	a	c	c	t	t	t	t	c	c	t	a	g	a	a	t
KP771905	5	a	t	t	c	t	g	a	a	-	g	g	g	t	t	a	a	a	c	a	t	g	g	c	t	a	a	c	t	t	c	a	c	g	g	a	t	c	a	a	c	a	g	g	a	a	a	t	a	g	t	a	a	a	c	c	a	t	c	a	c	c	t	t	t	t	c	c	t	a	g	a	a	t
KP771906	5	a	t	t	c	t	g	a	a	-	g	g	g	t	t	a	a	a	c	a	t	g	g	c	t	a	a	c	t	t	c	a	c	g	g	a	t	c	a	a	c	a	g	g	a	a	a	t	a	g	t	a	a	a	c	c	a	t	c	a	c	c	t	t	t	t	c	c	t	a	g	a	a	t
KP771919	5	a	t	t	c	t	g	a	a	-	g	g	g	t	t	a	a	a	c	a	t	g	g	c	t	a	a	c	t	t	c	a	c	g	g	a	t	c	a	a	c	a	g	g	a	a	a	t	a	g	t	a	a	a	c	c	a	t	c	a	c	c	t	t	t	t	c	c	t	a	g	a	a	t
KP771922	5	a	t	t	c	t	g	a	g	-	g	g	g	t	t	a	a	a	c	a	t	g	g	c	t	a	a	c	t	t	c	a	c	g	g	a	t	c	a	a	c	a	g	g	a	a	a	t	a	g	t	a	a	a	c	c	a	t	c	a	c	c	t	t	t	t	c	c	t	a	g	a	a	t
KP771924	5	a	t	t	c	t	g	a	a	-	g	g	g	t	t	a	a	a	c	a	t	g	g	c	t	a	a	c	t	t	c	a	c	g	g	a	t	c	a	a	c	a	g	g	a	a	a	t	a	g	t	a	a	a	c	c	a	t	c	a	c	c	t	t	t	t	c	c	t	a	g	a	a	t
KM355696	5	a	t	t	c	t	g	a	a	-	g	g	g	t	t	a	a	a	c	a	t	g	g	a	t	a	a	c	t	t	c	a	c	g	g	a	t	c	a	a	c	a	g	g	a	a	a	t	a	g	t	a	a	a	c	c	a	t	c	a	c	c	t	t	t	t	c	c	t	a	g	a	a	t
KM355697	5	a	t	t	c	t	g	a	a	-	g	g	g	t	t	a	a	a	c	a	t	g	g	a	t	a	a	c	t	t	c	a	c	g	g	a	t	c	a	a	c	a	g	g	a	a	a	t	a	g	t	a	a	a	c	c	a	t	c	a	c	c	t	t	t	t	c	c	t	a	g	a	a	t
KM355698	5	a	t	t	c	t	g	a	a	-	g	g	g	t	t	a	a	a	c	a	t	g	g	c	t	a	a	c	t	t	c	a	c	g	g	a	t	c	a	a	c	a	g	g	a	a	a	t	a	g	t	a	a	a	c	c	a	t	c	a	c	c	t	t	t	t	c	c	t	a	g	a	a	t
KM355699	5	a	t	t	c	t	g	a	a	-	g	g	g	t	t	a	a	a	c	a	t	g	g	c	t	a	a	c	t	t	c	a	c	g	g	a	t	c	a	a	c	a	g	g	a	a	a	t	a	g	t	a	a	a	c	c	a	t	c	a	c	c	t	t	t	t	c	c	t	a	g	a	a	t
KM355701	5	a	t	t	c	t	g	a	a	-	g	g	g	t	t	a	a	a	c	a	t	g	g	a	t	a	a	c	t	t	c	a	c	g	g	a	t	c	a	a	c	a	g	g	a	a	a	t	a	g	t	a	a	a	c	c	g	t	c	a	c	c	t	t	t	t	c	c	t	a	g	a	a	t
KM355702	5	a	t	t	c	t	g	a	a	-	g	g	g	t	t	a	a	a	c	a	t	g	g	c	t	a	a	c	t	t	c	a	c	g	g	a	t	c	a	a	c	a	g	g	a	a	a	t	a	g	t	a	a	a	c	c	a	t	c	a	c	c	t	t	t	t	c	c	t	a	g	a	a	t
KM355703	5	a	t	t	c	t	g	a	a	-	g	g	g	t	t	a	a	a	c	a	t	g	g	a	t	a	a	c	t	t	c	a	c	g	g	a	t	c	a	a	c	a	g	g	a	a	a	t	a	g	t	a	a	a	c	c	a	t	c	a	c	c	t	t	t	t	c	c	t	a	g	a	a	t
KM355704	5	a	t	t	c	t	g	a	a	-	g	g	g	t	t	a	a	a	c	a	t	g	g	a	t	a	a	c	t	t	c	a	c	g	g	a	t	c	a	a	c	a	g	g	a	a	a	t	a	g	t	a	a	a	c	c	a	t	c	a	c	c	t	t	t	t	c	c	t	a	g	a	a	t
KM355705	5	a	t	t	c	t	g	a	a	-	g	g	g	t	t	a	a	a	c	a	t	g	g	c	t	a	a	c	t	t	c	a	c	g	g	a	t	c	a	a	c	a	g	g	a	a	a	t	a	g	t	a	a	a	c	c	a	t	c	a	c	c	t	t	t	t	c	c	t	a	g	a	a	t
KM355706	5	a	t	t	c	t	g	a	a	-	g	g	g	t	t	a	a	a	c	a	t	g	g	c	t	a	a	c	t	t	c	a	c	g	g	a	t	c	a	a	c	a	g	g	a	a	a	t	a	g	t	a	a	a	c	c	a	t	c	a	c	c	t	t	t	t	c	c	t	a	g	a	a	t
KM355707	5	a	t	t	c	t	g	a	a	-	g	g	g	t	t	a	a	a	c	a	t	g	g	a	t	a	a	c	t	t	c	a	c	g	g	a	t	c	a	a	c	a	g	g	a	a	a	t	a	g	t	a	a	a	c	c	a	t	c	a	c	c	t	t	t	t	c	c	t	a	g	a	a	t
KM355708	5	a	t	t	c	t	g	a	a	-	g	g	g	t	t	a	a	a	c	a	t	g	g	a	t	a	a	c	t	t	c	a	c	g	g	a	t	c	a	a	c	a	g	g	a	a	a	t	a	g	t	a	a	a	c	c	a	t	c	a	c	c	t	t	t	t	c	c	t	a	g	a	a	t
KM355709	5	a	t	t	c	t	g	a	a	-	g	g	g	t	t	a	a	a	c	a	t	g	g	c	t	a	a	c	t	t	c	a	c	g	g	a	t	c	a	a	c	a	g	g	a	a	a	t	a	g	t	a	a	a	c	c	a	t	c	a	c	c	t	t	t	t	c	c	t	a	g	a	a	t
KM355711	5	a	t	t	c	t	g	a	a	-	g	g	g	t	t	a	a	a	c	a	t	g	g	c	t	a	a	c	t	t	c	a	c	g	g	a	t	c	a	a	c	a	g	g	a	a	a	t	a	g	t	a	a	a	c	c	a	t	c	a	c	c	t	t	t	t	c	c	t	a	g	a	a	t
KM355712	5	a	t	t	c	t	g	a	a	-	g	g	g	t	t	a	a	a	c	a	t	g	g	c	t	a	a	c	t	t	c	a	c	g	g	a	t	c	a	a	c	a	g	g	a	a	a	t	a	g	t	a	a	a	c	c	a	t	c	a	c	c	t	t	t	t	c	c	t	a	g	a	a	t
KY037797	5	[illegible]	[illegible]	[illegible]	[illegible]	[illegible]	[illegible]	[illegible]	[illegible]	[illegible]	[illegible]	[illegible]	[illegible]	[illegible]	[illegible]	[illegible]	[illegible]	[illegible]	[illegible]	[illegible]	[illegible]	[illegible]	[illegible]	[illegible]	[illegible]	[illegible]	[illegible]	[illegible]	[illegible]	[illegible]	[illegible]	[illegible]	[illegible]	[illegible]	[illegible]	[illegible]	[illegible]	[illegible]	[illegible]	[illegible]	[illegible]	[illegible]	[illegible]	[illegible]	[illegible]	[illegible]	[illegible]	[illegible]	[illegible]	[illegible]	[illegible]	[illegible]	[illegible]	[illegible]	[illegible]	[illegible]	[illegible]	[illegible]	[illegible]	[illegible]	[illegible]	[illegible]	[illegible]	[illegible]	[illegible]	[illegible]	[illegible]	[illegible]	[illegible]	[illegible]	[illegible]	[illegible]	[illegible]	[illegible]

Supplementary Table 3: 2880 SN

GB-ID	Clade markers																						5										v														5																											
	Position in Dumas	90139	90202	90215	90217	90318	90340	90392	90417	90417_1	90418	90423	90424	90425	90437	90466	90467	90535	90621	90637	90648	90796	90810	90846	90891	90895	90903	90925	90948	91002	91063	91066	91191	91343	91524	91661	91770	91773	91831	91924	91953	91961	92026	92092	92118	92129	92348	92361	92375	92409	92432	92514	92523	92535	92544	92635	92640	92646	92670	92694	92725	92836	92861	92878	92879	92920	92999	93107	93108	93234	93319	93367	93368	93386
MH499467	5	a	t	t	c	t	g	a	a	-	g	g	g	t	t	a	a	a	c	a	t	g	g	c	t	a	a	c	t	t	c	a	c	g	g	a	t	c	a	a	c	a	g	g	a	a	a	t	a	g	t	a	a	a	c	c	a	t	c	a	a	c	t	t	t	t	c	c	t	a	g	a	a	t
MH499469	5	a	t	t	c	t	g	a	a	-	g	g	g	t	t	a	a	a	c	a	t	g	g	c	t	a	a	c	t	t	c	a	c	g	g	a	t	c	a	a	c	a	g	g	a	a	a	t	a	g	t	a	a	a	c	c	a	t	c	a	c	c	t	t	t	t	c	c	t	a	g	a	a	t
MH709321	5	a	t	g	c	t	g	a	-	-	g	g	g	t	t	a	a	a	c	a	c	g	g	c	t	a	a	c	t	t	c	a	c	g	g	a	t	c	a	a	c	a	g	g	a	a	a	t	a	g	t	a	a	a	c	c	a	t	c	a	c	c	t	t	t	t	c	c	t	a	g	a	a	t
MH709324	5	a	t	t	c	t	g	a	a	-	g	g	g	t	t	a	a	a	c	a	t	g	g	c	t	a	a	c	t	t	c	a	c	g	g	a	t	c	a	a	c	a	g	g	a	a	a	t	a	g	t	a	a	a	c	c	a	t	c	a	c	c	t	t	t	t	c	c	t	a	g	a	a	t
MH709325	5	a	t	t	c	t	g	a	a	-	g	g	g	t	t	a	a	a	c	a	t	g	g	c	t	a	a	c	t	t	c	a	c	g	g	a	t	c	a	a	c	a	g	g	a	a	a	t	a	g	t	a	a	a	c	c	a	t	c	a	c	c	t	t	t	t	c	c	t	a	g	a	a	t
MH709327	5	a	t	t	c	t	g	a	a	-	g	g	g	t	t	a	a	a	c	a	t	g	g	c	t	a	a	c	t	t	c	a	c	g	g	a	t	c	a	a	c	a	g	g	a	a	a	t	a	g	t	a	a	a	c	c	a	t	c	a	c	c	t	t	t	t	c	c	t	a	g	a	a	t
MH709328	5	a	t	t	c	t	g	a	a	-	g	g	g	t	t	a	a	a	c	a	t	g	g	c	t	a	a	c	t	t	c	a	c	g	g	a	t	c	a	a	c	a	g	g	a	a	a	t	a	g	t	a	a	a	c	c	a	t	c	a	c	c	t	t	t	t	c	c	t	a	g	a	a	t
MH709330	5	a	t	t	c	t	g	a	a	-	g	g	g	t	t	a	a	a	c	a	t	g	g	c	t	a	a	c	t	t	c	a	c	g	g	a	t	c	a	a	c	a	g	g	a	a	a	t	a	g	t	a	a	a	c	c	a	t	c	a	c	c	t	t	t	t	c	c	t	a	g	a	a	t
MH709332	5	a	t	t	c	t	g	a	a	-	g	g	g	t	t	a	a	a	c	a	t	g	g	c	t	a	a	c	t	t	c	a	c	g	g	a	t	c	a	g	c	a	g	g	a	a	a	t	a	g	t	a	a	a	c	c	a	t	c	a	c	c	t	t	t	t	c	c	t	a	g	a	a	t
MH709334	5	a	t	t	c	t	g	a	a	-	g	g	g	t	t	a	a	a	c	a	t	g	g	c	t	a	a	c	t	t	c	a	c	g	g	a	t	c	a	a	c	a	g	g	a	a	a	t	a	g	t	a	a	a	c	c	a	t	c	a	c	c	t	t	t	t	c	c	t	a	g	a	a	t
MH709335	5	a	t	t	c	t	g	a	a	-	g	g	g	t	t	a	a	a	c	a	t	g	g	c	t	a	a	c	t	t	c	a	c	g	g	a	t	c	a	a	c	a	g	g	a	a	a	t	a	g	t	a	a	a	c	c	a	t	c	a	c	c	t	t	t	t	c	c	t	a	g	a	a	t
MH709336	5	a	t	t	c	t	g	a	a	-	g	g	g	t	t	a	a	a	c	a	t	g	g	c	t	a	a	c	t	t	c	a	c	g	g	a	t	c	a	a	c	a	g	g	a	a	a	t	a	g	t	a	a	a	c	c	a	t	c	a	c	c	t	t	t	t	c	c	t	a	g	a	a	t
MH709338	5	a	t	t	c	t	g	a	a	-	g	g	g	t	t	a	a	a	c	a	t	g	g	c	t	a	a	c	t	t	c	a	c	g	g	a	t	c	a	a	c	a	g	g	a	a	a	t	a	g	t	a	a	a	c	c	a	t	c	a	c	c	t	t	t	t	c	c	t	a	g	a	a	t
MH709340	5	a	t	t	c	t	g	a	a	-	g	g	g	t	t	a	a	a	c	g	t	g	g	c	t	a	a	c	t	t	c	a	c	g	g	a	t	c	a	a	c	a	g	g	a	a	a	t	a	g	t	a	a	a	c	c	a	t	c	a	c	c	t	t	t	t	c	c	t	a	g	a	a	t
MH709342	5	a	t	g	c	t	g	a	-	-	g	g	g	t	t	a	a	a	c	a	t	g	g	c	t	a	a	c	t	t	c	a	c	g	g	a	t	c	a	a	c	a	g	g	a	a	a	t	a	g	t	a	a	a	c	c	a	t	c	a	c	c	t	t	t	t	c	t	t	a	g	a	a	t
MH709343	5	a	t	t	c	t	g	a	a	-	g	g	g	t	t	a	a	a	c	a	t	g	g	c	t	a	a	c	t	t	c	a	c	g	g	a	t	c	a	a	c	a	g	g	a	a	a	t	a	g	t	a	a	a	c	c	a	t	c	c	c	c	t	t	t	t	c	c	t	a	g	a	a	t
MH709345	5	a	t	t	c	t	g	a	a	-	g	g	g	t	t	a	a	a	c	a	t	g	g	c	t	a	a	c	t	t	c	a	c	g	g	a	t	c	a	a	c	a	g	g	a	a	a	t	a	g	t	a	a	a	c	c	a	t	c	a	c	c	t	t	t	t	c	c	g	a	g	a	a	t
MH709347	5	a	t	t	c	t	g	a	a	-	g	g	g	t	t	a	a	a	c	a	t	g	g	c	t	a	a	c	t	t	c	a	c	g	g	a	t	c	a	a	c	a	g	g	a	a	a	t	a	g	t	a	a	a	c	c	a	t	c	a	c	c	t	t	t	t	c	c	t	a	g	a	a	t
MH709349	5	a	t	t	c	t	g	a	a	-	g	g	g	t	t	a	a	a	c	a	t	g	g	c	t	g	a	c	t	t	c	a	c	g	g	a	t	c	a	a	c	a	g	g	a	a	a	t	a	g	t	a	a	a	c	c	a	t	c	a	c	c	t	t	t	t	c	c	t	a	g	a	a	t
MH709350	5	a	t	t	c	t	g	a	a	-	g	g	g	t	t	a	a	a	c	a	t	g	g	c	t	a	a	c	t	t	c	a	c	g	g	a	t	c	a	a	c	a	g	g	a	a	a	t	a	g	t	a	a	a	c	c	a	t	c	a	c	c	t	t	t	t	c	c	t	a	g	a	a	t
MH709351	5	a	t	t	c	t	g	a	a	-	g	g	g	g	t	a	a	a	c	g	t	g	g	c	t	a	a	c	t	t	c	a	c	g	g	a	t	c	a	a	c	a	g	g	a	a	a	t	a	g	t	a	a	a	c	c	a	t	c	a	c	c	t	t	t	t	c	c	t	a	g	a	a	t
MH709353	5	a	t	t	c	t	g	a	a	-	g	g	g	t	t	a	a	a	c	a	t	g	g	c	t	a	a	c	t	t	c	a	c	g	g	a	t	c	a	a	c	a	g	g	a	a	a	t	a	g	t	a	a	a	c	c	a	t	c	a	c	c	t	t	t	t	c	c	t	a	g	a	a	t
MH709358	5	a	t	t	c	t	g	a	a	-	g	g	g	t	t	a	a	a	c	a	t	g	g	c	t	a	a	c	t	t	c	a	c	g	g	a	t	c	a	a	c	a	g	g	a	a	a	t	a	g	t	a	a	a	c	c	a	t	c	a	c	c	t	t	t	t	c	c	t	a	g	a	a	t
MH709360	5	a	t	t	c	t	g	a	a	-	g	g	g	t	t	a	a	a	c	a	t	g	g	c	t	a	a	c	t	t	c	a	c	g	g	a	t	c	a	a	c	a	g	g	a	a	a	t	a	g	t	a	a	a	c	c	a	t	c	a	c	c	t	t	t	t	c	c	t	a	g	a	a	t
MH709362	5	a	t	t	c	t	g	a	a	-	g	g	g	t	t	a	a	a	c	a	t	g	g	c	t	a	a	c	t	t	c	a	c	g	g	a	t	c	a	a	c	a	g	g	a	a	a	t	a	g	t	a	a	a	c	c	a	t	c	a	c	c	t	t	t	t	c	c	t	a	g	a	a	t
MH709365	5	a	t	t	c	t	g	a	a	-	g	g	g	t	t	a	a	a	c	a	t	g	g	c	t	a	a	c	t	t	c	a	c	g	g	a	t	c	a	a	c	a	g	g	a	a	a	t	a	g	t	a	a	a	c	c	a	t	c	a	c	c	t	t	t	t	c	c	t	a	g	a	a	t
MH709373	5	a	t	t	c	t	g	a	g	-	g	g	g	t	t	a	a	a	c	a	t	g	g	c	t	a	a	c	t	t	c	a	c	g	g	a	t	c	a	a	c	a	g	g	a	a	a	t	a	g	t	a	a	a	c	c	a	t	c	a	c	c	t	t	t	t	c	c	t	a	g	a	a	t
MH709374	5	a	t	t	c	t	g	a	a	-	g	g	g	t	t	a	a	a	c	a	t	g	g	c	t	a	a	c	t	t	c	a	c	g	g	a	t	c	a	a	c	a	g	g	a	a	a	t	a	g	t	a	a	a	c	c	a	t	c	a	c	c	t	t	t	t	c	c	t	a	g	a	a	t
KC112914	6	a	t	t	t	t	g	g	a	-	g	g	g	t	t	a	a	a	c	a	t	g	t	c	t	a	a	c	t	t	c	a	c	g	g	a	t	c	a	a	c	a	a	g	a	a	g	t	a	g	t	a	a	a	c	c	a	t	c	a	c	c	t	t	t	t	c	c	t	a	g	g	g	t
KP771914	6	a	t	t	t	t	g	g	a	g	g	g	g	t	t	a	a	a	c	a	t	g	t	c	t	a	a	c	t	t	c	a	c	g	g	a	t	c	a	a	c	a	a	g	a	a	g	t	a	g	t	a	a	a	c	c	a	t	c	a	c	c	t	t	t	t	c	c	t	a	g	g	g	t
KP702725	6	a	t	t	t	t	g	g	a	-	g	g	g	t	t	a	a	a	c	a	t	g	t	c	t	a	a	c	t	t	c	a	c	g	g	a	t	c	a	a	c	a	a	g	a	a	g	t	a	g	t	a	a	a	c	c	a	t	c	a	c	c	t	t	t	t	c	c	t	a	g	g	g	t
KP771890	9	a	t	t	t	t	g	a	a	-	g	g	g	t	t	a	a	a	c	a	t	g	t	c	t	a	a	c	t	t	c	a	c	g	g	a	t	c	c	a	c	a	g	g	a	a	g	t	a	g	t	g	g	a	c	c	a	t	c	a	c	c	t	t	t	t	c	c	t	a	g	g	g	t
JN704710	9	a	g	t	t	t	g	g	a	-	g	g	g	t	t	a	a	a	c	a	t	g	t	c	t	a	a	c	t	t	c	a	c	g	g	a	t	c	a	a	c	a	a	a	a	a	g	t	a	g	t	a	a	a	c	c	a	t	c	a	c	c	t	t	t	t	t	c	t	a	g	a	a	t
KY037798	9	a	t	t	t	t	g	a	a	-	g	g	g	t	t	a	a	a	c	a	t	g	t	c	t	a	a	c	t	t	c	a	c	g	g	a	t	c	c	a	c	a	g	g	a	a	g	t	a	g	t	g	g	a	c	c	a	t	c	a	c	c	t	t	t	t	c	c	t	a	g	a	a	t
MH709312	9	a	t	t	t	t	g	a	a	-	g	g	g	t	t	a	a	a	c	a	t	g	t	c	t	a	a	c	t	t	c	a	c	g	g	a	t	c	c	a	c	a	g	g	a	a	g	t	a	g	t	g	g	a	c	c	a	t	c	a	c	c	t	t	t	t	c	c	t	a	g	a	a	t
MH709352	9	a	t	t	t	t	g	a	a	-	g	g	g	t	t	a	a	a	c	a	t	g	t	c	t	a	a	c	t	t	c	a	c	g	g	a	t	c	c	a	c	a	g	g	a	a	g	t	a	g	t	g	g	a	c	c	a	t	c	a	c	c	t	t	t	t	c	c	t	a	g	a	a	t
AB097933	Vac	a	t	t	c	t	g	a	a	-	g	g	g	t	t	a	a	a	c	a	t	g	t	c	t	a	a	c	t	t	c	a	t	g	g	a	t	c	a	a	c	a	g	g	a	a	g	t	g	g	t	a	a	a	c	c	a	t	c	a	c	c	t	t	t	t	c	c	t	a	g	g	g	t
JN704698	Vac	a	t	t	c	t	g	a	a	-	g	g	g	t	t	a	a	a	c	a	t	g	t	c	t	a	a	c	t	t	c	a	t	g	g	a	t	c	a	a	c	a	g	g	a	a	g	t	g	g	t	a	a	a	c	c	a	t	c	a	c	c	t	t	t	t	c	c	t	a	g	g	g	t
JN704697	Vac	a	t	t	c	t	g	a	a	-	g	g	g	t	t	a	a	g	c	a	t	g	t	c	t	a	a	c	t	t	c	a	t	g	g	a	t	c	a	a	c	a	g	g	a	a	g	t	g	g	t	a	a	a	c	c	a	t	c	a	c	c	t	t	t	t	c	c	t	a	g	g	g	t
DQ008355	Vac	a	t	t	c	t	g	a	a	-	g	g	g	t	t	a	a	a	c	a	t	g	t	c	t	a	a	c	t	t	c	a	t	g	g	a	t	c	a	a	c	a	g	g	a	a	g	t	g	g	t	a	a	a	c	c	a	t	c	a	c	c	t	t	t	t	c	c	t	a	g	g	g	t
JF306641	Vac	a	t	t	c	t	g	a	a	-	g	g	g	t	t	a	a	g	c	a	t	g	t	c	t	a	a	c	t	t	c	a	t	g	g	a	t	c	a	a	c	a	g	g	a	a	g	t	g	g	t	a	a	a	c	c	a	t	c	a	c	c	t	t	t	t	c	c	t	a	g	g	g	t
AB097932	Vac	a	t	t	c	t	g	a	a	-	g	g	g	t	t	a	a	r	c	a	t	g	t	c	t	a	a	c	t	t	c	a	t	g	g	a	t	c	a	a	c	a	g	g	a	a	g	t	g	g	t	a	a	a	c	c	a	t	c	a	c	c	t	t	t	t	c	c	t	a	g	g	g	t
DQ008354	Vac	a	t	t	c	t	g	a	a	-	g	g	g	t	t	a	a	r	c	a	t	g	t	c	t	a	a	c	t	t	c	a	t	g	g	a	t	c	a	a	c	a	g	g	a	a	g	t	g	g	t	a	a	a	c	c	a	t	c	a	c	c	t	t	t	t	c	c	t	a	g	g	g	t
KU926312	Vac	a	t	t	c	t	g	a	a	-	g	g	g	t	t	a	a	g	c	a	t	g	t	c	t	a	a	c	t	t	c	a	t	g	g	a	t	c	a	a	c	a	g	g	a	a	g	t	g	g	t	a	a	a	c	c	a	t	c	a	c	c	t	t	t	t	c	c	t	a	g	g	g	t
KU926313	Vac	a	t	t	c	t	g	a	a	-	g	g	g	t	t	a	a	a	c	a	t	g	t	c	t	a	a	c	t	t	c	a	t	g	g	a	t	c	a	a	c	a	g	g	a	a	g	t	g	g	t	a	a	a	c	c	a	t	c	a	c	c	t	t	t	t	c	c	t	a	g	g	g	t
KU926314	Vac	a	t	t	c	t	g	a	a	-	g	g	g	t	t	a	a	a	c	a	t	g	t	c	t	a	a	c	t	t	c	a	t	g	g	a	t	c	a	a	c	a	g	g	a	a	g	t	g	g	t	a	a	a	c	c	a	t	c	a	c	c	t	t	t	t	c	c	t	a	g	g	g	t
KF558371	Vac	a	t	t	c	t	g	a	a	-	g	g	g	t	t	a	a	a	c	a	t	g	t	c	t	a	a	c	t	t	c	a	t	g	g	a	t	c	a	a	c	a	g	g	a	a	g	t	g	g	t	a	a	a	c	c	a	t	c	a	c	c	t	t	t	t	c	c	t	a	g	g	g	t
KF558372	Vac	a	t	t	c	t	g	a	a	-	g	g	g	t	t	a	a	a	c	a	t	g	t	c	t	a	a	c	t	t	c	a	t	g	g	a	t	c	a	a	c	a	g	g	a	a	g	t	g	g	t	a	a	a	c	c	a	t	c	a	c	c	t	t	t	t	c	c	t	a	g	g	g	t
KF558373	Vac	a	t	t	c	t	g	a	a	-	g	g	g	t	t	a	g	a	c	a	t	g	t	c	t	a	a	c	t	t	c	a	t	g	g	a	t	c	a	a	c	a	g	g	a	a	g	t	g	g	t	a	a	a	c	c	a	t	c	a	c	c	t	t	t	t	c	c	t	a	g	g	g	t
KF558374	Vac	a	t	t	c	t	g	a	a	-	g	g	g	t	t	a	a	a	c	a	t	g	t	c	t	a	a	c	t	t	c	a	t	g	g	a	t	c	a	a	c	a	g	g	a	a	g	t	g	g	t	a	a	a	c	c	a	t	c	a	c	c	t	t	t	t	c	c	t	a	g	g	g	t
KF558375	Vac	a	t	t	c	t	g	a	a	-	g	g	g	t	t	a	a	a	c	a	t	g	t	c	t	a	a	c	t	t	c	a	t	g	g	a	t	c	a	a	c	a	g	g	a	a	g	t	g	g	t	a	a	a	c	c	a	t	c	a	c	c	t	t	t	t	c	c	t	a	g	g	g	t
KF558376	Vac	a	t	t	c	t	g	a	a	-	g	g	g	t	t	a	a	a	c	a	t	g	t	c	t	a	a	c	t	t	c	a	t	g	g	a	t	c	a	a	c	a	g	g	a	a	g	t	g	g	t	a	a	a	c	c	a	t	c	a	c	c	t	t	t	t	c	c	t	a	g	g	g	t
KF558377	Vac	a	t	t	c	t	g	a	a	-	g	g	g	t	c	a	a	a	c	a	t	g	t	c	t	a	a	c	t	t	c	a	t	g	g	g	t	c	a	a	c	a	g	g	a	a	g	t	g	g	t	a	a	a	c	c	a	t	c	a	c	c	t	t	t	t	c	c	t	a	g	g	g	t
KF558378	Vac	a	t	t	c	t	g	a	a	-	g	g	g	t	t	a	a	a	c	a	t	g	t	c	t	a	a	c	t	t	c	a	t	g	g	a	t	c	a	a	c	a	g	g	a	a	g	t	g	g	t	a	a	a	c	c	a	t	c	a	c	c	t	t	t	t	c	c	t	a	g	g	g	t
KF558379	Vac	a	t	t	c	t	g	a	a	-	g	g	g	t	t	a	a	a	c	a	t	g	t	c	t	a	a	c	t	t	c	a	t	g	g	a	t	c	a	a	c	a	g	g	a	a	g	t	g	g	t	a	a	a	c	c	a	t	c	a	c	c	t	t	t	t	c	c	t	a	g	g	g	t
KF558380	Vac	a	t	t	c	t	g	a	a	-	g	g	g	t	t	a	a	a	c	a	t	g	t	c	t	a	a	c	t	t	c	a	t	g	g	a	t	c	a	a	c	a	g	g	a	a	g	t	g	g	t	a	a	a	c	c	a	t	c	a	c	c	t	t	t	t	c	c	t	a	g	g	g	t
KF558381	Vac	a	t	t	c	t	g	a	a	-	g	g	g	t	t	a	a	a	c	a	t	g	t	c	t	a	a	c	t	t	c	a	t	g	g	a	t	c	a	a	c	a	g	g	a	a	g	t	g	g	t	a	a	a	c	c	a	t	c	a	c	c	t	t	t	t	c	c	t	a	g	g	g	t
KF558382	Vac	a	t	t	c	t	g	a	a	-	g	g	g	t	t	a	a	a	c	a	t	g	t	c	t	a	a	c	t	t	c	a	t	g	g	a	t	c	a	a	c	a	g	g	a	a	g	t	g	g	t	a	a	a	c	c	a	t	c	a	c	c	t	t	t	t	c	c	t	a	g	g	g	t
KF558383	Vac	a	t	t	c	t	g	a	a	-	g	g	g	t	t	a	a	a	c	a	t	g	t	c	t	a	a	c	t	t	c	a	t	g	g	a	t	c	a	a	c	a	g	g	a	a	g	t	g	g	t	a	a	a	c	c	a	t	c	a	c	c	t	t	t	t	c	c	t	a	g	g	g	t
KF558384	Vac	a	t	t	c	t	g	a	a	-	g	g	g	t	t	a	a	a	c	a	t	g	t	c	t	a	a	c	t	t	c	a	t	g	g	a	t	c	a	a	c	a	g	g	a	a	g	t	g	g	t	a	a	a	c	c	a	t	c	a	c	c	t	t	t	t	c	c	t	a	g	g	g	t
KF558385	Vac	a	t	t	c	t	g	a	a	-	g	g	g	t	t	a	a	a	c	a	t	g	t	c	t	a	a	c	t	t	c	a	t	g	g	a	t	c	a	a	c	a	g	g	a	a	g	t	g	g	t	a	a	a	c	c	a	t	c	a	c	c	t	t	t	t	c	c	t	a	g	g	g	t
KF558386	Vac	a	t	t	c	t	g	a	a	-	g	g	g	t	t	a	a	a	c	a	t	g	t	c	t	a	a	c	t	t	c	a	t	g	g	a	t	c	a	a	c	a	g	g	a	a	g	t	g	g	t	a	a	a	c	c	a	t	c	a	c	c	t	t	t	t	c	c	t	a	g	g	g	t
KF558387	Vac	a	t	t	c	t	g	a	a	-	g	g	g	t	t	a	a	a	c	a	t	g	t	c	t	a	a	c	t	t	c	a	t	g	g	a	t	c	a	a	c	a	g	g	a	a	g	t	g	g	t	a	a	a	c	c	a	t	c	a	c	c	t	t	t	t	c	c	t	a	g	g	g	t
KF558388	Vac	a	t	t	c	t	g	a	a	-	g	g	g	t	t	a	a	a	c	a	t	g	t	c	t	a	a	c	t	t	c	a	t	g	g	a	t	c	a	a	c	a	g	g	a	a	g	t	g	g	t	a	a	a	c	c	a	t	c	a	c	c	t	t	t	t	c	c	t	a	g	g	g	t
KF558389	Vac	a	t	t	c	t	g	a	a	-	g	g	g	t	t	a	a	a	c	a	t	g	t	c	t	a	a	c	t	t	c	a	t	g	g	a	t	c	a	a	c	a	g	g	a	a	g	t	g	g	t	a	a	a	c	c	a	t	c	a	c	c	t	t	t	t	c	c	t	a	g	g	g	t
KF558390	Vac	a	t	t	c	t	g	a	a	-	g	g	g	t	t	a	a	a	c	a	t	g	t	c	t	a	a	c	t	t	c	a	t	g	g	a	t	c	a	a	c	a	g	g	a	a	g	t	g	g	t	a	a	a	c	c	a	t	c	a	c	c	t	t	t	t	c	c	t	a	g	g	g	t
KF558391	Vac	a	t	t	c	c	g	a	a	-	g	g	g	t	t	a	a	a	c	a	t	g	t	c	t	a	a	c	t	t	c	a	t	g	g	a	t	c	a	a	c	a	g	g	a	a	g	t	g	g	t	a	a	a	c	c	a	t	c	a	c	c	t	t	t	t	c	c	t	a	g	g	g	t
KF811485	Vac	a	t	t	c	t	g	a	a	-	g	g	g	t	t	a	a	a	c	a	t	g	t	c	t	a	a	c	t	t	c	a	t	g	g	a	t	c	a	a	c	a	g	g	a	a	g	t	g	g	t	a	a	a	c	c	a	t	c	a	c	c	t	t	t	t	c	c	t	a	g	g	g	t
KF853225	Vac	a	t	t	c	t	g	a	a	-	g	g	g	t	t	a	a	a	c	a	t	g	t	c	t	a	a	c	t	t	c	a	t	g	g	a	t	c	a	a	c	a	g	g	a	a	g	t	g	g	t	a	a	a	c	c	a	t	c	a	c	c	t	t	t	t	c	c	t	a	g	g	g	t
KF853226	Vac	a	t	t	c	t	g	a	a	-	g	g	g	t	t	a	a	a	c	a	t	g	t	c	t	a	a	c	t	t	c	a	t	g	g	a	t	c	a	a	c	a	g	g	a	a	g	t	g	g	t	a	a	a	c	c	a	t	c	a	c	c	t	t	t	t	c	c	t	a	g	g	g	t
KF853227	Vac	a	t	t	c	t	g	a	a	-	g	g	g	t	t	a	a	a	c	a	t	g	t	c	t	a	a	c	t	t	c	a	t	g	g	a	t	c	a	a	c	a	g	g	a	a	g	t	g	g	t	a	a	a	c	c	a	t	c	a	c	c	t	t	t	t	c	c	t	a	g	g	g	t
KF853228	Vac	a	t	t	c	c	g	a	a	-	g	g	g	t	t	a	a	a	c	a	t	g	t	c	t	a	a	c	t	t	c	a	t	g	g	a	t	c	a	a	c	a	g	g	a	a	g	t	g	g	t	a	a	a	c	c	a	t	c	a	c	c	t	t	t	t	c	c	t	a	g	g	g	t
KF853229	Vac	a	t	t	c	t	g	a	a	-	g	g	g	t	t	a	a	a	c	a	t	g	t	c	t	a	a	c	t	t	c	a	t	g	g	a	t	c	a	a	c	a	g	g	a	a	g	t	g	g	t	a	a	a	c	c	a	t	c	a	c	c	t	t	t	t	c	c	t	a	g	g	g	t
KF853230	Vac	a	t	t	c	t	g	a	a	-	g	g	g	t	t	a	a	g	c	a	t	g	t	c	t	a	a	c	t	t	c	a	t	g	g	a	t	c	a	a	c	a	g	g	a	g	g	t	g	g	t	a	a	a	c	c	a	t	c	a	c	c	t	t	t	t	c	c	t	a	g	g	g	t
KF853231	Vac	a	t	t	c	t	g	a	a	-	g	g	g	t	t	a	a	a	c	a	t	g	t	c	t	a	a	c	t	t	c	a	t	g	g	a	t	c	a	a	c	a	g	g	a	a	g	t	g	g	t	a	a	a	c	c	a	t	c	a	c	c	t	t	t	t	c	c	t	a	g	g	g	t
KF853232	Vac	a	t	t	c	t	g	a	a	-	g	g	g	t	t	a	a	a	c	a	t	g	t	c	t	a	a	c	t	t	c	a	t	g	g	a	t	c	a	a	c	a	g	g	a	a	g	t	g	g	t	a	a	a	c	c	a	t	c	a	c	c	t	t	t	t	c	c	t	a	g	g	g	t
KF853233	Vac	a	t	t	c	t	g	a	a	-	g	g	g	t	t	a	a	a	c	a	t	g	t	c	t	a	a	c	t	t	c	a	t	g	g	a	t	c	a	a	c	a	g	g	a	a	g	t	g	g	t	a	a	a	c	c	a	t	c	a	c	c	t	t	t	t	c	c	t	a	g	g	g	t
KF853234	Vac	a	t	t	c	t	g	a	a	-	g	g	g	t	t	a	a	a	c	a	t	g	t	c	t	a	a	c	t	t	c	a	t	g	g	a	t	c	a	a	c	a	g	g	a	a	g	t	g	g	t	a	a	a	c	c	a	c	c	a	c	c	t	t	t	t	c	c	t	a	g	g	g	t
KF853235	Vac	a	t	t	c	t	g	a	a	-	g	g	g	t	t	a	a	a	c	a	t	g	t	c	t	a	a	c	t	t	c	a	t	g	g	a	t	c	a	a	c	a	g	g	a	a	g	t	g	g	t	a	a	a	c	c	a	t	c	a	c	c	t	t	t	t	c	c	t	a	g	g	g	t
MF898328	Vac	a	t	t	c	t	g	a	a	-	g	g	g	t	t	a	a	r	c	a	t	g	t	c	t	a	a	c	t	t	c	a	t	g	g	a	t	c	a	a	c	a	g	g	a	a	g	t	g	g	t	a	a	a	c	c	a	t	c	a	c	c	t	t	t	t	c	c	t	a	g	g	g	t
JN704709	VIII	a	t	t	t	t	g	a	a	-	g	g	g	t	t	a	a	a	c	a	t	g	t	c	t	a	a	c	t	t	c	a	c	g	g	a	t	c	a	a	c	a	g	a	a	a	g	t	a	g	t	a	g	a	c	c	a	t	c	a	c	c	t	t	t	t	c	c	t	a	a	g	g	t

Supplementary Table 3: 2880 SN

GB-ID	Clade markers														9													2												5										2							SD14																	
	Position in Dumas	93395	93419	93590	93609	93664	93701	93803	93812	93818	93854	93998	94008	94020	94032	94095	94167	94173	94227	94260	94296	94320	94347	94443	94521	94545	94560	94599	94632	94641	94740	94778	94911	94941	95012	95014	95178	95226	95241	95262	95300	95308	95319	95326	95331	95333	95339	95355	95514	95546	95601	95610	95687	95691	95692	95693	95736	95845	95863	95896	95900	95905	95927	95934	95935	95941	95954	95958	95985	96029	96109	96148	96166	96371	96397
AY548170	1	c	c	t	a	t	a	g	t	g	c	a	t	g	t	c	t	g	a	a	a	c	g	t	t	g	c	c	a	a	t	g	a	c	c	t	c	t	t	g	c	g	t	g	a	t	c	t	t	g	t	t	c	a	g	a	g	t	a	t	a	t	c	t	t	t	t	a	t	c	t	t	t	t	g
AY548171	1	c	c	t	a	t	a	g	t	g	c	a	t	g	t	c	t	g	a	a	a	c	g	t	t	g	c	c	a	a	t	g	a	c	c	t	c	t	t	g	c	g	t	g	a	t	c	t	t	g	t	t	c	a	g	a	g	t	a	t	a	t	c	t	t	t	t	a	t	c	t	t	t	t	g
DQ479953	1	c	c	t	a	t	a	g	t	g	c	a	t	g	t	c	t	g	a	a	a	c	g	t	t	g	c	c	a	a	t	g	a	c	c	t	c	t	t	g	c	g	t	g	a	t	c	t	t	g	t	t	c	a	g	a	g	t	a	t	a	t	c	t	t	t	t	a	t	c	t	t	t	t	g
DQ479954	1	c	c	t	a	t	a	g	t	g	c	a	t	g	t	c	t	g	a	a	a	c	g	t	t	g	c	c	a	a	t	g	a	c	c	t	c	t	t	g	c	g	t	g	a	t	c	t	t	g	t	t	c	a	g	a	g	t	a	t	a	t	c	t	t	t	t	a	t	c	t	t	t	t	g
DQ479958	1	c	c	t	a	t	a	g	t	g	c	a	t	g	t	c	t	g	a	a	a	c	g	t	t	g	c	c	a	a	t	g	a	c	c	t	c	t	t	g	c	g	t	g	a	t	c	t	t	g	t	t	c	a	g	a	g	t	a	t	a	t	c	t	t	t	t	a	t	c	t	t	t	t	g
DQ479959	1	c	c	t	a	t	a	g	t	g	c	a	t	g	t	c	t	g	a	a	a	c	g	t	t	g	c	c	a	a	t	g	a	c	c	t	c	t	t	g	c	g	t	g	a	t	c	t	t	g	t	t	c	a	g	a	g	t	a	t	a	t	c	t	t	t	t	a	t	c	t	t	t	t	g
DQ479961	1	c	c	t	a	t	a	g	t	g	c	a	t	g	t	c	t	g	a	a	a	c	g	t	t	g	c	c	a	a	t	g	a	c	c	t	c	t	t	g	c	g	t	g	a	t	c	t	t	g	t	t	c	a	g	a	g	t	a	t	a	t	c	t	t	t	t	a	t	c	t	t	t	t	g
DQ479962	1	c	c	t	a	t	a	g	t	g	c	a	t	g	t	c	t	g	a	a	a	c	g	t	t	g	c	c	a	a	t	g	a	c	c	t	c	t	t	g	c	g	t	g	a	t	c	t	t	g	t	t	c	a	g	a	g	t	a	t	a	t	c	t	t	t	t	a	t	c	t	t	t	t	g
DQ479963	1	c	c	t	a	t	a	g	t	g	c	a	t	g	t	c	t	g	a	a	a	c	g	t	t	g	c	c	a	a	t	g	a	c	c	t	c	t	t	g	c	g	t	g	a	t	c	t	t	g	t	t	c	a	g	a	g	t	a	t	a	t	c	t	t	t	t	a	t	c	t	t	t	t	g
DQ674250	1	c	c	t	a	t	a	g	t	g	c	a	t	g	t	c	t	g	a	a	a	c	g	t	a	g	c	c	a	a	t	g	a	c	c	t	c	t	t	g	c	g	t	g	a	t	c	t	t	g	t	t	c	a	g	a	g	t	a	t	a	t	c	t	t	t	t	a	t	c	t	t	t	t	g
KX262864	1	c	c	t	a	t	a	g	t	g	c	a	t	g	t	c	t	g	a	a	a	c	g	t	t	g	c	c	a	a	t	g	a	c	c	t	c	t	t	g	c	g	t	g	a	t	c	t	t	g	t	t	c	a	g	a	g	t	a	t	a	t	c	t	t	t	t	a	t	c	t	t	t	t	g
KX262865	1	c	c	t	a	t	a	g	t	g	c	a	t	g	t	c	t	g	a	a	a	c	g	t	t	g	c	c	a	a	t	g	a	c	c	t	c	t	t	g	c	g	t	g	a	t	c	t	t	g	t	t	c	a	g	a	g	t	a	t	a	t	c	t	t	t	t	a	t	c	t	t	t	t	g
KX262866	1	c	c	t	a	t	a	g	t	g	c	a	t	g	t	c	t	g	a	a	a	c	g	t	t	g	c	c	a	a	t	g	a	c	c	t	c	t	t	g	c	g	t	g	a	t	c	t	t	g	t	t	c	a	g	a	g	t	a	t	a	t	c	t	t	t	t	a	t	c	t	t	t	t	g
EU154348	1	c	c	t	a	t	a	g	t	g	c	a	t	g	t	c	t	g	a	a	a	c	g	t	t	g	c	c	a	a	t	g	a	c	c	t	c	t	t	g	c	g	t	g	a	t	c	t	t	g	t	t	c	a	g	a	g	t	a	t	a	t	c	t	t	t	t	a	t	c	t	t	t	t	g
JN704690	1	c	c	t	a	t	a	g	t	g	c	a	t	g	t	c	t	g	a	a	a	c	g	t	t	g	c	c	a	a	t	g	a	c	c	t	c	t	t	t	c	g	t	g	a	t	c	t	t	g	t	t	c	a	g	a	g	t	a	t	a	t	c	t	t	t	t	a	t	c	t	t	t	t	g
JN704691	1	c	c	t	a	t	a	g	t	g	c	a	t	g	t	c	t	g	a	a	a	c	g	t	t	g	c	c	a	a	t	g	a	c	c	t	c	t	t	g	c	g	t	g	a	t	c	t	t	g	t	t	c	a	g	a	g	t	a	t	a	t	c	t	t	t	t	a	t	c	t	t	t	t	g
JN704692	1	c	c	t	a	t	a	g	t	g	c	a	t	g	t	c	t	g	a	a	a	c	g	t	t	g	c	c	a	a	t	g	a	c	c	t	c	t	t	g	c	g	t	g	a	t	t	t	t	g	t	t	c	a	g	a	g	t	a	t	a	t	a	t	t	t	t	a	t	c	t	t	t	t	g
JN704693	1	c	c	t	a	t	a	g	t	g	c	a	t	g	t	c	t	g	a	a	a	c	g	t	t	g	c	c	a	a	t	g	a	c	c	t	c	t	t	g	c	g	t	g	a	t	c	t	t	g	t	t	c	a	g	a	g	t	a	t	a	t	c	t	t	t	t	a	t	c	t	t	t	t	g
JN704694	1	c	c	t	a	t	a	g	t	g	c	a	t	g	t	c	t	g	a	a	a	c	g	t	t	g	c	c	a	a	t	g	a	c	c	t	c	t	t	g	c	g	t	g	a	t	c	t	t	g	t	t	c	a	g	a	g	t	a	t	a	t	c	t	c	t	t	a	t	c	t	t	t	t	g
JN704695	1	t	c	t	a	t	a	g	t	g	c	a	t	g	t	c	t	g	a	a	a	c	g	t	t	g	c	c	a	a	t	g	a	c	c	t	c	t	t	t	c	g	t	g	a	t	c	t	t	g	t	t	c	a	g	a	g	t	a	t	a	t	c	t	t	t	t	a	t	c	t	t	t	t	g
JN704696	1	c	c	t	a	t	a	g	t	g	c	a	t	g	t	c	t	g	a	a	a	c	g	t	t	g	c	c	a	a	t	g	a	c	c	t	c	t	t	g	c	g	t	g	a	t	c	t	t	g	t	t	c	a	g	a	g	t	a	t	a	t	c	t	t	t	t	a	t	c	t	t	t	t	g
NC001348	1	c	c	t	a	t	a	g	t	g	c	a	t	g	t	c	t	g	a	a	a	c	g	t	t	g	c	c	a	a	t	g	a	c	c	t	c	t	t	g	c	g	t	g	a	t	c	t	t	g	t	t	c	a	g	a	g	t	a	t	a	t	c	t	t	t	t	a	t	c	t	t	t	t	g
KP771892	1	c	c	t	a	t	a	g	t	g	c	a	t	g	t	c	t	g	a	a	a	c	g	t	t	g	c	c	a	a	t	g	a	c	c	t	c	t	t	g	c	g	t	g	a	t	c	t	t	g	t	t	c	a	g	a	g	t	a	t	a	t	c	t	t	t	t	a	t	c	t	t	t	t	g
KP771894	1	c	c	t	a	t	a	a	t	g	c	a	t	g	t	c	t	g	a	a	a	c	g	t	t	g	c	c	a	a	t	g	a	c	c	t	c	t	t	g	c	g	t	g	a	t	c	t	t	g	t	t	c	a	g	a	g	t	a	t	a	t	c	t	t	t	t	a	t	c	t	t	t	t	g
KP771900	1	c	c	t	a	t	a	g	t	g	c	a	t	g	t	c	t	g	a	a	a	c	g	t	t	g	c	c	a	a	t	g	a	c	c	t	c	t	t	g	c	g	t	g	a	t	c	t	t	g	t	t	c	a	g	a	g	t	a	t	a	t	c	t	t	t	t	a	t	c	t	t	t	t	g
KP771901	1	c	c	t	a	t	a	g	t	g	c	a	t	g	t	c	t	g	a	a	a	c	g	t	t	g	c	c	a	a	t	g	a	c	c	t	c	t	t	g	c	g	t	g	a	t	c	t	t	g	t	t	c	a	g	a	g	t	a	t	a	t	c	t	t	t	t	a	t	c	t	t	t	t	g
KP771902	1	c	c	t	a	t	a	g	t	g	c	a	t	g	t	c	t	g	a	a	a	c	g	t	t	g	c	c	a	a	t	g	a	c	c	t	c	t	t	g	c	g	t	g	a	t	c	t	t	g	t	t	c	a	g	a	g	t	a	t	a	t	c	t	t	t	t	a	t	c	t	t	t	t	g
KP771908	1	c	c	t	a	t	a	g	t	g	c	a	t	g	t	c	t	g	a	a	a	c	g	t	t	g	c	c	a	a	t	g	a	c	c	t	c	t	t	g	c	g	t	g	a	t	c	t	t	g	t	t	c	a	g	a	g	t	a	t	a	t	c	t	t	t	t	a	t	c	t	g	t	t	g
KP771912	1	c	c	t	a	t	a	g	t	g	c	a	t	g	t	c	t	g	a	a	a	c	g	t	t	g	c	c	a	a	t	g	a	c	c	t	c	t	t	g	c	g	t	g	a	t	c	t	t	g	t	t	c	a	g	a	g	t	a	t	a	t	c	t	t	t	t	a	t	c	t	t	t	t	g
KP771913	1	c	c	t	a	t	a	g	t	g	c	a	t	g	t	c	t	g	a	a	a	c	g	t	t	g	c	c	a	a	t	g	a	c	c	t	c	t	t	g	c	g	t	g	a	t	c	t	t	g	t	t	c	a	g	a	g	t	a	t	a	t	c	t	t	t	t	a	t	c	t	t	t	t	g
KP771917	1	c	c	t	a	t	a	g	t	g	c	a	t	g	t	c	t	g	a	a	a	c	g	t	t	g	c	c	a	a	t	g	a	c	c	t	c	t	t	g	c	a	t	g	a	t	c	t	t	g	t	t	c	a	g	a	g	t	a	t	a	t	c	t	t	t	t	a	t	c	t	t	t	t	g
KP771918	1	c	c	t	a	t	a	g	t	g	c	a	t	g	t	c	t	g	a	a	a	c	g	t	t	g	c	c	a	a	t	g	a	c	c	t	c	t	t	g	c	g	t	g	a	t	c	t	t	g	t	t	c	a	g	a	g	t	a	t	a	t	c	t	t	t	t	a	t	c	t	t	t	t	g
KP771920	1	c	c	t	a	t	a	g	t	g	c	a	t	g	t	c	t	g	a	a	a	c	g	t	t	g	c	c	a	a	t	g	a	c	c	t	c	t	t	g	c	g	t	g	a	t	c	t	t	g	t	t	c	a	g	a	g	t	a	t	a	t	c	t	t	t	t	a	t	c	t	t	t	t	g
MH709361	1	c	c	t	a	t	a	g	t	g	c	a	t	g	t	c	t	g	a	a	a	c	g	t	t	g	c	c	a	a	t	g	a	c	c	t	c	t	t	g	c	g	t	g	a	t	c	t	t	g	t	t	c	a	g	a	g	t	a	t	a	t	c	t	t	t	t	a	t	c	t	t	t	t	g
KU926318	2	c	c	t	a	t	a	g	t	g	c	a	t	g	t	c	t	g	g	a	g	c	g	t	t	g	c	c	g	t	t	g	a	c	c	t	c	t	c	g	c	g	t	g	a	t	c	t	t	a	g	t	c	a	g	a	g	t	a	t	a	t	c	t	t	t	t	a	c	c	t	t	t	t	g
KU926319	2	c	c	t	a	t	a	g	t	g	c	a	t	g	t	c	t	g	a	a	a	c	g	t	t	g	c	c	g	t	t	g	a	c	c	t	c	t	c	g	c	g	t	g	a	t	c	t	t	a	g	t	c	a	g	a	g	t	a	t	a	t	c	t	t	t	t	a	t	c	t	t	t	t	g
KU926320	2	c	c	t	a	t	a	g	t	g	c	a	t	g	t	c	t	g	a	a	g	c	g	t	t	g	c	c	g	t	t	g	a	c	c	t	c	t	c	g	c	g	t	g	g	t	c	t	t	a	g	t	c	a	g	a	g	t	a	t	a	t	c	t	t	t	c	a	t	c	t	t	t	t	g
KU926321	2	c	c	t	a	t	a	g	t	g	c	a	t	g	t	c	t	g	a	a	a	c	g	t	t	g	c	t	g	t	t	g	a	c	c	t	c	t	c	g	c	g	t	g	a	t	c	t	t	a	g	t	c	a	g	a	g	t	a	t	a	t	c	t	t	t	t	a	t	c	t	t	t	t	g
KU926322	2	c	c	t	a	t	a	g	t	g	c	a	t	g	t	c	t	g	a	a	a	c	g	t	t	g	c	t	g	t	t	g	a	c	c	t	c	t	c	g	c	g	t	g	a	t	c	t	t	a	g	t	c	a	g	a	g	t	a	t	a	t	c	t	t	t	t	a	t	c	t	t	t	t	g
KC847290	2	c	c	t	a	t	a	g	t	g	c	a	t	g	t	c	t	g	a	a	a	c	g	t	t	g	c	c	g	t	t	g	a	c	c	t	c	t	c	g	c	g	t	g	a	t	c	t	t	a	g	t	c	a	g	a	g	t	a	t	a	t	c	t	t	t	t	a	t	c	t	t	t	t	g
KJ767491	2	c	c	t	a	t	a	g	t	g	c	a	t	g	t	c	t	g	a	a	a	c	g	t	t	g	c	c	g	t	t	g	a	c	c	t	c	t	c	g	c	g	t	g	a	t	c	t	t	a	g	t	c	a	g	a	g	t	a	t	a	t	c	t	t	t	t	a	t	c	t	t	t	t	g
KJ767492	2	c	c	t	a	t	a	g	t	g	c	a	t	g	t	c	t	g	a	a	a	c	g	t	t	g	c	c	g	t	t	g	a	c	c	t	c	t	c	g	c	g	t	g	a	t	c	t	t	a	g	t	c	a	g	a	g	t	a	t	a	t	c	t	t	t	t	a	t	c	t	t	t	t	g
KJ808816	2	c	c	t	a	t	a	g	t	g	c	a	t	g	t	c	t	g	a	a	a	c	g	t	t	g	c	t	g	t	t	g	a	c	c	t	c	t	c	g	c	g	t	g	a	t	c	t	t	a	g	t	c	a	g	a	g	t	a	t	a	t	c	t	t	t	t	a	t	c	t	t	t	t	g
KP771925	2	c	c	t	a	t	a	g	t	g	c	a	t	g	t	c	t	g	a	a	a	c	g	t	t	g	t	c	g	t	t	g	a	c	c	t	c	t	c	g	c	g	t	g	a	t	c	t	t	a	g	t	c	a	g	a	g	t	a	t	a	t	c	t	t	t	t	a	t	c	t	t	t	t	g
KU926315	2	c	c	t	a	t	a	g	t	g	c	a	t	g	t	c	t	g	a	a	a	c	g	t	t	g	c	c	g	t	t	g	a	c	c	t	c	t	c	g	c	g	t	g	a	t	c	t	t	a	g	t	c	a	g	a	g	t	a	t	a	t	c	t	t	t	t	a	t	c	t	t	t	t	g
KU926316	2	c	c	t	a	t	a	g	t	g	c	a	t	g	t	c	t	g	a	a	a	c	g	t	t	g	c	c	g	t	t	g	a	c	c	t	c	t	c	g	c	g	t	g	a	t	c	t	t	a	g	t	c	a	g	a	g	t	a	t	a	t	c	t	t	t	t	a	t	c	t	t	t	t	g
KU926317	2	c	c	t	a	t	a	g	t	g	c	a	t	g	t	c	t	g	a	a	a	c	g	t	t	g	c	c	g	t	t	g	a	c	c	t	c	t	c	g	c	g	t	g	a	t	c	t	t	a	g	t	c	a	g	a	g	t	a	t	a	t	c	t	t	t	t	a	t	c	t	t	t	t	g
KY037796	2	c	c	t	a	t	a	g	t	g	c	a	t	g	t	c	t	g	a	a	a	c	g	t	t	g	c	c	g	t	t	g	a	c	c	t	c	t	c	g	c	g	t	g	a	t	c	t	t	a	g	t	c	a	g	a	g	t	a	t	c	t	c	t	t	t	t	a	t	c	t	t	t	t	g
MF004348	2	c	c	t	a	t	a	g	t	g	c	a	t	g	t	c	t	g	a	a	a	c	g	t	t	g	c	c	g	t	t	g	a	c	c	t	c	t	c	g	c	g	t	g	a	t	c	t	t	a	g	t	c	a	g	a	g	t	a	t	a	t	c	t	t	t	t	a	t	c	t	t	t	t	g
MH709309	2	c	c	t	a	t	a	g	t	g	c	a	t	g	t	c	t	g	a	a	a	c	g	t	t	g	c	c	g	t	t	g	a	c	c	t	c	t	c	g	c	g	t	g	a	t	c	t	t	a	g	t	c	a	g	a	g	t	a	t	a	t	c	t	t	t	t	a	t	c	t	t	t	t	g
MH709310	2	c	c	t	a	t	a	g	t	g	c	a	t	g	t	c	t	g	a	a	a	c	g	t	t	g	c	c	g	t	t	g	a	t	c	t	c	t	c	g	c	g	t	g	a	t	c	t	t	a	g	t	c	a	g	a	g	t	a	t	a	t	c	t	t	t	t	a	t	c	t	t	t	t	g
MH709311	2	c	c	t	a	t	a	g	c	g	c	a	t	g	t	c	t	g	a	a	a	c	g	t	t	g	c	c	g	t	t	g	a	c	c	t	c	t	c	g	c	g	t	g	a	t	c	t	t	a	g	t	c	a	g	a	g	t	a	t	a	t	c	t	t	t	t	a	t	c	t	t	t	t	g
MH709313	2	c	c	t	a	t	a	g	t	g	c	a	t	g	t	c	t	g	a	a	a	c	g	t	t	g	c	c	g	t	t	g	a	c	c	t	c	t	c	g	c	g	t	g	a	t	c	t	t	a	g	t	c	a	g	a	g	t	a	t	a	t	c	t	t	t	t	a	t	c	t	t	t	t	g
MH709314	2	c	c	t	a	t	a	g	t	g	c	a	t	g	t	c	t	g	a	a	a	c	g	t	t	g	c	c	g	t	t	g	a	c	c	t	c	t	c	g	c	g	t	g	a	t	c	t	t	a	g	t	c	a	g	a	g	t	a	t	a	t	c	t	t	t	t	a	t	c	t	t	t	t	g
MH709315	2	c	c	t	a	t	a	g	t	g	c	a	t	g	t	c	t	g	a	a	a	c	g	t	t	g	c	c	g	t	t	g	a	c	c	t	c	t	c	g	c	g	t	g	a	t	c	t	t	a	g	t	c	a	g	a	g	t	a	t	a	t	c	t	t	t	t	a	t	c	t	t	t	c	g
MH709319	2	c	c	t	a	t	a	g	t	g	c	a	t	g	t	c	t	g	a	a	a	c	g	t	t	g	c	c	g	t	t	g	a	c	c	t	c	t	c	g	c	g	t	g	a	t	c	t	t	a	g	t	c	a	g	a	g	t	a	t	a	t	c	t	t	t	t	a	t	c	t	t	t	t	g
MH709320	2	c	c	t	a	t	a	g	t	g	c	a	t	g	t	c	t	g	a	a	a	c	g	t	t	g	c	c	g	t	t	g	a	c	c	t	c	t	c	g	c	g	t	g	a	t	c	t	t	a	g	t	c	a	g	a	g	t	a	t	a	g	c	t	t	t	t	a	t	c	t	t	t	t	g
MH709329	2	c	c	t	a	t	a	g	t	g	c	a	t	g	t	c	t	g	a	a	a	c	g	t	t	g	c	c	g	t	t	g	a	c	c	t	c	t	c	g	c	g	t	g	a	t	c	t	t	a	g	t	c	a	g	a	g	t	a	t	a	t	c	t	t	t	t	a	t	c	t	t	t	t	g
MH709331	2	c	c	t	a	t	a	g	t	g	c	a	t	g	t	c	t	g	a	a	a	c	g	t	t	g	c	c	g	t	t	g	a	c	c	t	c	t	c	g	c	g	t	g	a	t	c	t	t	a	g	t	c	a	g	a	g	t	a	t	a	t	c	t	t	t	t	a	t	c	t	t	t	t	g
MH709333	2	c	c	t	a	t	a	g	t	g	c	a	t	g	t	c	t	g	a	a	a	c	g	t	t	g	c	c	g	t	t	g	a	c	c	t	c	t	c	g	c	g	t	g	a	t	c	t	t	a	g	t	c	a	g	a	g	t	a	t	a	t	c	t	t	t	t	a	t	c	t	t	t	t	g
MH709337	2	c	t	t	a	t	a	g	t	g	c	a	t	g	t	c	t	g	a	a	a	c	g	t	t	g	c	c	g	t	t	g	a	c	c	t	t	t	c	g	c	g	t	a	a	t	c	t	t	a	g	t	c	a	g	a	g	t	a	t	a	t	c	t	t	t	t	a	t	c	t	t	t	t	g
MH709339	2	c	c	t	a	t	a	g	t	g	c	a	t	g	t	c	t	g	a	a	a	c	g	t	t	g	c	c	g	t	t	g	a	c	c	t	c	t	c	g	c	g	t	g	a	t	c	t	t	a	g	t	c	a	g	a	g	t	a	t	a	t	c	t	t	t	t	a	t	c	t	t	t	t	g
MH709344	2	c	c	t	a	t	a	g	t	g	c	a	t	g	t	c	t	g	a	a	a	c	g	t	t	g	c	c	g	t	t	g	a	c	c	t	c	t	c	g	c	g	t	g	a	t	c	t	t	a	g	t	c	a	g	a	g	t	a	t	a	t	c	t	t	t	t	a	t	c	t	t	t	t	g
MH709348	2	c	c	t	a	t	a	g	t	g	c	a	t	g	t	c	t	g	a	a	a	c	g	t	t	g	c	c	g	t	t	g	a	c	c	t	c	t	c	g	c	g	t	g	a	t	c	t	t	a	g	t	c	a	g	a	g	t	a	t	a	t	c	t	t	t	t	a	t	c	t	t	t	t	t
MH709355	2	c	c	t	a	t	a	g	t	g	c	a	t	g	t	c	t	g	a	a	a	c	g	t	t	g	c	c	g	t	t	g	a	c	c	t	c	t	c	g	c	g	t	g	a	t	c	t	t	a	g	t	c	a	g	a	g	t	a	t	a	t	c	t	t	t	t	a	t	c	t	t	t	t	g
MH709359	2	c	c	t	a	t	a	g	t	g	c	a	t	g	t	c	t	g	a	a	a	c	g	t	t	g	c	c	g	t	t	g	a	c	c	t	c	t	c	g	c	g	t	g	a	t	c	t	t	a	g	t	c	a	g	a	g	t	a	t	a	t	c	t	t	t	t	a	t	c	t	t	t	t	g
MH709363	2	c	c	t	a	t	a	g	t	g	c	a	t	g	t	c	t	g	a	a	a	c	g	t	t	g	c	c	g	t	t	g	a	c	c	t	c	t	c	g	c	g	t	g	a	t	c	t	t	a	g	t	c	a	g	a	g	t	a	t	a	g	c	t	t	t	t	a	t	c	t	t	t	t	g
MH709366	2	c	c	t	a	t	g	g	t	g	c	a	t	g	t	c	t	g	a	a	a	c	g	t	t	g	c	c	g	t	t	g	a	c	c	t	c	t	c	g	c	g	t	g	a	t	c	t	t	a	g	t	c	a	g	a	g	t	a	t	a	t	c	t	t	t	t	a	t	c	t	t	t	t	g
MH709376	2	c	c	t	a	t	a	g	t	g	c	a	t	g	t	c	t	g	a	a	a	c	g	t	t	g	c	c	g	t	t	g	a	c	c	t	c	t	c	g	c	g	t	g	a	t	c	t	t	a	g	t	c	a	g	a	g	t	a	t	a	t	c	t	t	t	t	a	t	c	t	t	t	t	g
MH709377	2	c	c	t	a	t	a	g	t	g	c	a	t	g	t	c	t	g	a	a	a	c	g	t	t	g	c	c	g	t	t	g	a	c	c	t	c	t	c	g	c	g	t	g	a	t	c	t	t	a	g	t	c	a	g	a	g	t	a	t	a	t	c	t	t	t	t	a	t	c	t	t	t	t	g
**MW316406**	2	c	c	t	a	t	a	g	t	g	c	a	t	g	t	c	t	g	a	a	a	c	g	t	t	g	c	c	g	t	t	g	a	c	c	t	c	t	c	g	c	g	t	g	a	t	c	t	t	a	g	t	c	a	g	a	g	c	a	t	a	t	c	t	t	t	t	a	t	c	t	t	t	t	g
AJ871403	3	c	c	t	a	t	a	g	t	g	t	a	t	a	t	c	t	g	a	a	a	c	g	t	t	g	c	c	a	a	t	g	a	c	c	t	c	t	t	g	c	g	t	g	a	t	c	t	t	g	t	t	c	a	g	a	g	t	a	t	a	t	c	t	t	t	t	a	t	c	t	t	t	t	g
DQ479955	3	c	c	t	a	t	a	g	t	g	c	a	t	g	t	c	t	g	a	a	a	c	g	t	t	g	c	c	a	a	t	g	a	c	c	t	c	t	t	g	c	g	t	g	a	t	c	t	t	g	t	t	c	a	g	a	g	t	a	t	a	t	c	t	t	t	t	a	t	c	t	t	t	t	g
DQ479956	3	c	c	t	a	t	a	g	t	g	c	a	t	g	t	c	t	g	a	a	a	c	g	t	t	g	c	c	a	a	t	g	a	c	c	t	c	t	t	g	c	g	t	g	a	t	c	t	t	g	t	t	c	a	g	a	g	t	a	t	a	t	c	t	t	t	t	a	t	c	t	t	t	t	g
DQ479957	3	c	c	t	a	t	a	g	t	g	c	a	t	g	t	c	t	a	a	a	a	c	g	t	t	t	c	c	a	a	t	g	a	c	c	t	c	t	t	g	c	g	t	g	a	t	c	t	t	g	t	t	c	a	g	a	g	t	a	t	a	t	c	t	t	t	t	a	t	c	t	t	t	t	g
JN704699	3	c	c	t	a	t	a	g	t	g	c	a	t	g	t	c	t	g	a	a	a	c	g	t	t	g	c	c	a	a	t	g	a	c	c	t	c	t	t	g	c	g	t	g	a	t	c	t	t	g	t	t	c	a	g	a	g	t	a	t	a	t	c	t	t	t	t	a	t	c	t	t	t	t	g
JN704700	3	c	c	t	a	t	a	g	t	g	c	a	t	g	t	c	t	g	a	a	a	c	g	t	t	g	c	c	a	a	t	g	a	c	c	t	c	t	t	g	c	g	g	g	a	t	c	t	t	g	t	t	c	a	g	a	g	t	a	t	a	t	c	t	t	t	t	a	t	c	t	t	t	t	g
JN704701	3	c	c	t	a	t	a	g	t	g	c	a	t	g	t	c	t	g	a	a	a	c	g	t	t	g	c	c	a	a	t	g	a	c	c	t	c	t	t	g	c	g	t	g	a	t	c	t	t	g	t	t	c	a	g	a	g	t	a	t	a	t	c	t	t	t	t	a	t	c	t	t	t	t	g
JN704702	3	c	c	t	a	t	a	g	t	g	c	a	t	g	t	c	t	g	a	a	a	c	g	t	t	g	c	c	a	a	t	g	a	c	c	t	c	g	t	g	c	g	t	g	a	t	c	t	t	g	t	t	c	a	g	a	g	t	a	t	a	t	c	t	t	c	t	a	t	c	t	t	t	t	g
JN704703	3	c	c	t	a	t	a	g	t	g	c	a	t	g	t	c	t	g	a	a	a	c	g	t	t	g	c	c	a	a	t	g	a	c	c	t	c	t	t	g	c	g	t	g	a	t	c	t	t	g	t	t	c	a	g	a	g	t	a	t	a	t	c	t	t	t	t	a	t	c	t	t	t	t	g
KP771891	3	c	c	t	a	t	a	g	t	g	c	a	t	g	t	c	t	g	a	a	a	c	g	t	t	g	c	c	a	a	t	g	a	c	c	t	c	t	t	g	c	g	t	g	a	t	c	t	t	g	t	t	c	a	g	a	g	t	a	t	a	t	c	t	t	t	t	a	t	c	t	t	t	t	g
KP771893	3	c	c	t	a	t	a	g	t	g	c	a	t	g	t	c	t	g	a	a	a	c	g	t	t	g	c	c	a	a	t	g	a	c	c	t	c	t	t	g	c	g	t	g	a	t	c	t	t	g	t	t	c	a	g	a	g	t	a	t	a	t	c	t	t	t	t	a	t	c	t	t	t	t	g
KP771895	3	c	c	t	a	t	a	g	t	g	t	a	t	a	t	c	t	g	a	a	a	c	g	t	t	g	c	c	a	a	t	g	a	c	c	t	c	t	t	g	c	g	t	g	a	t	c	t	t	g	t	t	c	a	g	a	g	t	a	t	a	t	c	t	t	t	t	a	t	c	t	t	t	t	g
KP771896	3	c	c	t	a	t	a	g	t	g	c	a	t	g	t	c	t	g	a	a	a	c	g	t	t	g	c	c	a	a	t	g	a	c	c	t	c	t	t	g	c	g	t	g	a	t	c	t	t	g	t	t	c	a	g	a	g	t	a	t	a	t	c	t	t	t	t	a	t	c	t	t	t	t	g
KP771897	3	c	c	t	a	t	a	g	t	g	c	a	t	g	t	c	t	g	a	a	a	c	g	t	t	g	c	c	a	a	t	g	a	c	c	t	c	t	t	g	c	g	t	g	a	t	c	t	t	g	t	t	c	a	g	a	g	t	a	t	a	t	c	c	t	t	t	a	t	c	t	t	t	t	g
KP771899	3	c	c	t	a	t	a	g	t	g	t	a	t	a	t	c	t	g	a	a	a	c	g	t	t	g	c	c	a	a	t	g	a	c	c	t	c	t	t	g	c	g	t	g	a	t	c	t	t	g	t	t	c	a	g	a	g	t	a	t	a	t	c	t	t	t	t	a	t	c	t	t	t	t	g
KP771903	3	c	c	t	a	t	a	g	t	g	c	a	t	g	t	c	t	g	a	a	a	c	g	t	t	g	c	c	a	a	t	g	a	c	c	t	c	t	t	g	c	g	t	g	a	t	c	t	t	g	t	t	c	a	g	a	g	t	a	t	a	t	c	t	t	t	t	a	t	c	t	t	t	t	g
KP771907	3	c	c	t	a	t	a	g	t	g	t	a	t	a	t	c	t	g	a	a	a	c	g	t	t	g	c	c	a	a	t	g	a	c	c	t	c	t	t	g	c	g	t	g	a	t	c	t	t	g	t	t	c	a	g	a	g	t	a	t	a	t	c	t	t	t	t	a	t	c	t	t	t	t	g
KP771910	3	c	c	t	a	t	a	g	t	g	c	a	t	g	t	c	t	g	a	a	a	c	g	t	t	g	c	c	a	a	t	g	a	c	c	t	c	t	t	g	c	g	t	g	a	t	c	t	t	g	t	t	c	a	g	a	g	t	a	t	a	t	c	t	t	t	t	a	t	c	t	t	t	t	g
KP771915	3	c	c	t	a	t	a	g	t	g	c	a	t	g	t	c	t	g	a	a	a	c	g	t	t	g	c	c	a	a	t	g	a	c	c	t	c	t	t	g	c	g	t	g	a	t	c	t	t	g	t	t	c	a	g	a	g	t	a	t	a	t	c	c	t	t	t	a	t	c	t	t	t	t	g
KP771916	3	c	c	t	a	t	a	g	t	g	c	a	t	g	t	c	t	g	a	a	a	c	g	t	t	g	c	c	a	a	t	g	a	c	c	t	c	t	t	g	c	g	t	g	a	t	c	t	t	g	t	t	c	a	g	a	g	t	a	t	a	t	c	t	t	t	t	a	t	c	t	t	t	t	g
KP771921	3	c	c	t	a	t	a	g	t	c	c	a	t	g	t	c	t	g	a	a	a	c	g	t	t	g	c	c	a	a	t	g	a	c	c	t	c	t	t	g	c	g	t	g	a	t	c	t	t	g	t	t	c	a	g	a	g	t	a	t	a	t	c	t	t	t	t	a	t	c	t	t	t	t	g
KP771923	3	c	c	t	a	t	a	g	t	g	c	a	t	g	t	c	t	g	a	g	a	c	g	t	t	g	c	c	a	a	t	g	a	c	c	t	c	t	t	g	c	g	t	g	a	t	c	t	t	g	t	t	c	a	g	a	g	t	a	t	a	t	c	t	t	t	t	a	t	c	t	t	t	t	g
KP771889	3	c	c	t	a	t	a	g	t	g	c	a	t	g	t	c	t	g	a	a	a	c	g	t	t	g	c	c	a	a	t	g	a	c	c	t	c	t	t	g	c	g	t	g	a	t	c	t	t	g	t	t	c	a	g	a	g	t	a	t	a	t	c	t	t	t	t	a	t	c	t	t	t	t	g
KU926311	3	c	c	t	a	t	a	g	t	g	c	a	t	g	t	c	t	g	a	a	a	c	g	t	t	g	c	c	a	a	t	g	a	c	c	t	c	t	t	g	c	g	t	g	a	t	c	c	t	g	t	t	c	a	g	a	g	t	a	t	a	t	c	t	t	t	t	a	t	c	t	t	c	t	g
JQ911599	3	c	c	t	a	t	a	g	t	g	c	a	t	g	t	c	t	g	a	a	a	c	g	t	t	g	c	c	a	a	t	g	a	c	c	t	c	t	t	g	c	g	t	g	a	t	c	t	t	g	t	t	c	a	g	a	g	t	a	t	a	t	c	t	t	t	t	a	t	c	t	t	t	t	g
MH709317	3	c	c	t	a	t	a	g	t	g	c	a	t	g	t	c	t	g	a	a	a	c	g	t	t	g	c	c	a	a	t	g	a	c	c	t	c	t	t	g	c	g	t	g	a	t	c	t	t	g	t	t	c	a	g	a	g	t	a	t	a	t	c	t	t	t	t	a	t	c	t	t	t	t	g
MH709346	3	c	c	t	a	t	a	g	t	g	c	a	t	g	t	c	t	g	a	a	a	c	g	t	t	g	c	c	a	a	t	g	a	c	c	t	c	t	t	g	c	g	t	g	a	t	c	t	t	g	t	t	c	a	g	a	g	t	a	t	a	t	c	t	t	t	t	a	t	c	t	t	t	t	g
MH709375	3	c	c	t	a	t	a	g	t	g	c	a	t	g	t	c	t	g	a	a	a	c	g	t	t	g	c	c	a	a	t	g	a	c	c	t	c	t	t	g	c	g	t	g	a	t	c	t	t	g	t	t	c	a	g	a	g	t	a	t	a	t	c	t	t	t	t	a	t	c	t	t	t	t	g
DQ452050	4	c	c	t	a	t	a	g	t	g	c	a	t	g	t	c	t	g	a	a	a	c	g	t	t	g	c	c	a	t	t	g	a	c	c	t	c	t	c	g	c	g	t	g	a	t	c	t	t	a	t	t	c	a	g	a	g	t	a	t	a	t	c	t	t	t	t	a	t	c	t	t	t	t	g
DQ479960	4	c	c	t	a	t	a	g	t	g	c	a	t	g	t	c	t	g	a	a	a	c	g	t	t	g	c	c	a	t	t	g	a	c	c	t	c	t	c	g	c	g	t	g	a	t	c	t	t	a	t	t	c	a	g	a	g	t	a	t	a	t	c	t	t	t	t	a	t	c	t	t	t	t	g
KP771898	4	c	c	t	a	t	a	g	t	g	c	a	t	g	t	c	t	g	a	a	a	c	g	t	t	g	c	c	a	t	t	g	a	c	c	t	c	t	c	g	c	g	t	g	a	t	c	t	t	a	t	t	c	a	g	a	g	t	a	t	a	t	c	t	t	t	t	a	t	t	t	t	t	t	g
MH709316	4	c	c	t	a	t	a	g	t	g	c	a	t	g	t	c	t	g	a	a	a	c	g	t	t	g	c	c	a	t	t	g	a	c	c	t	c	t	c	g	c	g	t	g	a	t	c	t	t	a	t	t	t	a	g	a	a	t	a	t	a	t	c	t	t	t	t	a	t	c	t	t	t	t	g
MH709318	4	c	c	t	a	t	a	g	t	g	c	a	t	g	t	c	t	g	a	a	a	c	g	t	t	g	c	c	a	t	t	g	a	c	c	t	c	t	c	g	c	g	t	g	a	t	c	t	t	a	t	t	c	a	g	a	g	t	a	t	a	t	c	t	t	t	t	a	t	c	t	t	t	t	g
MH709322	4	c	c	t	a	t	a	g	t	g	c	a	t	g	t	c	t	g	a	a	a	t	g	t	t	g	c	c	a	t	t	g	a	c	c	t	c	t	c	g	c	g	t	g	a	t	c	t	t	a	t	t	c	a	g	a	g	t	a	t	a	t	c	t	t	t	t	a	t	c	t	t	t	t	g
MH709323	4	c	c	t	a	t	a	g	t	g	c	a	t	g	t	c	t	g	a	a	a	c	g	t	t	g	c	c	a	t	t	g	a	c	c	t	c	t	c	g	c	g	t	g	a	t	c	t	t	a	t	t	c	a	g	a	g	t	a	t	a	t	c	t	t	t	t	a	t	c	t	t	t	t	g
MH709326	4	c	c	t	a	t	a	g	t	g	c	a	t	g	t	c	t	g	a	a	a	c	g	t	t	g	c	c	a	t	t	g	a	c	c	t	c	t	c	g	c	g	t	g	a	t	c	t	t	a	t	t	c	a	g	a	g	t	a	t	a	t	c	t	t	t	t	a	t	c	t	t	t	t	g
MH709341	4	c	c	t	a	t	a	g	t	g	c	a	t	g	t	c	t	g	a	a	a	c	g	t	t	g	c	c	a	t	t	g	a	c	c	t	c	t	c	g	c	g	t	g	a	t	c	t	t	a	t	t	c	t	g	a	g	t	a	t	a	t	c	t	t	t	t	a	t	c	t	t	t	t	g
MH709357	4	c	c	t	a	t	a	g	t	g	c	a	t	g	t	c	t	g	a	a	a	c	g	t	t	g	c	c	a	t	t	g	a	c	c	t	c	t	c	g	c	g	t	g	a	t	c	t	t	a	t	t	c	a	g	a	g	t	a	t	a	t	c	t	t	t	t	a	t	c	t	t	t	t	g
MH709364	4	c	c	t	a	t	a	g	t	g	c	a	t	g	t	c	t	g	a	a	a	c	g	t	t	g	c	c	a	t	t	g	a	c	c	t	c	t	c	g	c	g	t	g	a	t	c	t	t	a	t	t	c	a	g	a	g	t	a	t	a	t	c	t	t	t	t	a	t	c	t	t	t	t	g
MH709370	4	c	c	t	a	t	a	g	t	g	c	a	t	g	t	c	t	g	a	a	a	c	g	t	t	g	c	c	a	t	t	g	a	c	c	t	c	t	c	g	c	g	t	g	a	t	c	t	t	a	t	t	c	a	g	a	g	t	a	t	a	t	c	t	t	t	t	a	t	c	t	t	t	t	g
MH709371	4	c	c	t	a	t	a	g	t	g	c	a	t	g	t	c	t	g	a	a	a	c	g	t	t	g	c	c	a	t	t	g	a	c	c	t	c	t	c	g	c	g	t	g	a	t	c	t	t	a	t	t	c	a	g	a	g	t	a	t	a	t	c	t	t	t	t	a	t	c	t	t	t	t	g
MH709372	4	c	c	t	a	t	a	g	t	g	c	a	t	g	t	c	t	g	a	a	a	c	g	t	t	g	c	c	a	t	t	g	a	c	c	t	c	t	c	g	c	g	t	g	a	t	c	t	t	a	t	t	c	a	g	a	g	t	a	t	a	t	c	t	t	t	t	a	t	c	t	t	t	t	g
DQ457052	5	c	c	t	a	t	a	g	t	g	c	g	t	g	t	c	c	g	a	a	a	c	g	t	t	g	c	c	a	t	t	g	a	c	c	t	c	t	c	g	t	g	t	g	a	t	c	t	t	a	t	g	c	a	g	a	g	t	a	t	a	t	c	t	t	t	t	a	t	c	t	t	t	t	g
JN704704	5	c	c	t	a	t	a	g	t	g	c	a	t	g	t	c	t	g	a	a	a	c	g	t	t	g	c	c	a	t	t	g	a	c	c	t	c	t	c	g	t	g	t	g	a	t	c	t	t	a	t	t	c	a	g	a	g	t	a	t	a	t	c	t	t	t	t	a	t	c	t	t	t	t	g
JN704705	5	c	c	t	a	t	a	g	t	g	c	a	c	g	t	c	t	g	a	a	a	c	g	t	t	g	c	c	a	t	t	g	a	c	c	t	c	t	c	g	t	g	t	g	a	c	c	t	t	a	t	t	c	a	g	a	g	t	a	t	a	t	c	t	t	t	t	a	t	c	t	t	t	t	g
JN704706	5	c	c	t	a	t	a	g	t	g	c	a	t	g	t	c	t	g	a	a	a	c	g	t	t	g	c	c	a	t	t	g	a	c	c	t	c	t	c	g	t	g	t	g	a	t	c	t	t	a	t	t	c	a	g	a	g	t	a	t	a	t	c	t	t	t	t	a	t	c	t	t	t	t	g
JN704707	5	c	c	t	a	t	a	g	t	g	c	a	t	g	t	c	t	g	a	a	a	c	g	t	t	g	c	c	a	t	t	g	a	c	c	t	c	t	c	g	t	g	t	g	a	t	c	t	t	a	t	t	c	a	g	a	g	t	a	t	a	t	c	t	t	t	t	a	t	c	t	t	t	t	g
JN704708	5	c	c	t	a	t	a	g	t	g	c	a	c	g	t	c	t	g	a	a	a	c	g	t	t	g	c	c	a	t	t	g	a	c	c	t	c	t	c	g	t	g	t	g	a	c	c	t	t	a	t	t	c	a	g	a	g	t	a	t	a	t	c	t	t	t	t	a	t	c	t	t	t	t	g
KP771904	5	c	c	t	a	t	a	g	t	g	c	a	t	g	t	c	t	g	a	a	a	c	g	t	t	g	c	c	a	t	t	a	a	c	t	t	c	t	c	g	t	g	t	g	a	t	c	t	t	a	t	t	c	a	g	a	g	t	a	t	a	t	c	t	t	t	t	a	t	c	t	t	t	t	g
KP771905	5	c	c	t	a	t	a	g	t	g	c	a	t	g	t	c	t	g	a	a	a	c	g	t	t	g	c	c	a	t	t	g	a	c	c	t	c	t	c	g	t	g	t	g	a	t	c	t	t	a	t	t	c	a	g	a	g	t	a	t	a	t	c	t	t	t	t	a	t	c	t	t	t	t	g
KP771906	5	c	c	t	a	t	a	g	t	g	c	a	t	g	t	c	t	g	a	a	a	c	g	t	t	g	c	c	a	t	t	g	a	c	c	t	c	t	c	g	t	g	t	g	a	t	c	t	t	a	t	t	c	a	g	a	g	t	a	t	a	t	c	t	t	t	t	a	t	c	t	t	t	t	g
KP771919	5	c	c	t	a	t	a	g	t	g	c	a	t	g	t	c	t	g	a	a	a	c	g	t	t	g	c	c	a	t	t	a	a	c	c	t	c	t	c	g	t	g	t	g	a	t	c	t	t	a	t	t	c	a	g	a	g	t	a	t	a	t	c	t	t	t	t	g	t	c	t	t	t	t	g
KP771922	5	c	c	t	a	t	a	g	t	g	c	a	t	g	c	c	t	g	a	a	a	c	g	t	t	g	c	c	a	t	c	g	a	c	c	t	c	t	c	g	t	g	t	g	a	t	c	t	t	a	t	t	c	a	g	a	g	t	a	t	a	t	c	t	t	t	t	a	t	c	t	t	t	t	g
KP771924	5	c	c	t	a	t	a	g	t	g	c	a	c	g	t	c	t	g	a	a	a	c	g	t	t	g	c	c	a	t	t	g	a	c	c	t	c	t	c	g	t	g	t	g	a	c	c	t	t	a	t	t	c	a	g	a	g	t	a	t	a	t	c	t	t	t	t	a	t	c	t	t	t	t	g
KM355696	5	c	c	t	a	t	a	g	t	g	c	a	c	g	t	c	t	g	a	a	a	c	g	t	t	g	c	c	a	t	t	g	a	c	c	t	c	t	c	g	t	g	t	g	a	t	c	t	t	a	t	t	c	a	g	a	g	t	a	t	a	t	c	t	t	t	t	a	t	c	t	t	t	t	g
KM355697	5	c	c	t	a	t	a	g	t	g	c	a	c	g	t	c	t	g	a	a	a	c	g	t	t	g	c	c	a	t	t	g	a	c	c	t	c	t	c	g	t	g	t	g	a	t	c	t	t	a	t	t	c	a	g	a	g	t	a	t	a	t	c	t	t	t	t	a	t	c	t	t	t	t	g
KM355698	5	c	c	t	a	t	a	g	t	g	c	a	c	g	t	c	t	g	a	a	a	c	g	t	t	g	c	c	a	t	t	g	a	c	c	t	c	t	c	g	t	g	t	g	a	t	c	t	t	a	t	t	c	a	g	a	g	t	a	t	a	t	c	t	t	t	t	a	t	c	c	t	t	t	g
KM355699	5	c	c	t	a	t	a	g	t	g	c	a	c	g	t	c	t	g	a	a	a	c	g	t	t	g	c	c	a	t	t	g	a	c	c	t	c	t	c	g	t	g	t	g	a	t	c	t	t	a	t	t	c	a	g	a	g	t	a	t	a	t	c	t	t	t	t	a	t	c	c	t	t	t	g
KM355701	5	c	c	t	a	t	a	g	t	g	c	a	c	g	t	c	t	g	a	a	a	c	g	t	t	g	c	c	a	t	t	g	a	c	c	t	c	t	c	g	t	g	t	g	a	t	c	t	t	a	t	t	c	a	g	a	g	t	a	t	a	t	c	t	t	t	t	a	t	c	t	t	t	t	g
KM355702	5	c	c	t	a	t	a	g	t	g	c	a	c	g	t	c	t	g	a	a	a	c	g	t	t	g	c	c	a	t	t	g	a	c	c	t	c	t	c	g	t	g	t	g	a	t	c	t	t	a	t	t	c	a	g	a	g	t	a	t	a	t	c	t	t	t	t	a	t	c	c	t	t	t	g
KM355703	5	c	c	t	a	t	a	g	t	g	c	a	c	g	t	c	t	g	a	a	a	c	g	t	t	g	c	c	a	t	t	g	a	c	c	t	c	t	c	g	t	g	t	g	a	t	c	t	t	a	t	t	c	a	g	a	g	t	a	t	a	t	c	t	t	t	t	a	t	c	t	t	t	t	g
KM355704	5	c	c	t	a	t	a	g	t	g	c	a	c	g	t	c	t	g	a	a	a	c	g	t	t	g	c	c	a	t	t	g	a	c	c	t	c	t	c	g	t	g	t	g	a	t	c	t	t	a	t	t	c	a	g	a	g	t	a	t	a	t	c	t	t	t	t	a	t	c	t	t	t	t	g
KM355705	5	c	c	t	a	t	a	g	t	g	c	a	c	g	t	c	t	g	a	a	a	c	g	t	t	g	c	c	a	t	t	g	a	c	c	t	c	t	c	g	t	g	t	g	a	t	c	t	t	a	t	t	c	a	g	a	g	t	a	t	a	t	c	t	t	t	t	a	t	c	c	t	t	t	g
KM355706	5	c	c	t	a	t	a	g	t	g	c	a	c	g	t	c	t	g	a	a	a	c	g	t	t	g	c	c	a	t	t	g	a	c	c	t	c	t	c	g	t	g	t	g	a	t	c	t	t	a	t	t	c	a	g	a	g	t	a	t	a	t	c	t	t	t	t	a	t	c	c	t	t	t	g
KM355707	5	c	c	t	a	t	a	g	t	g	c	a	c	g	t	c	t	g	a	a	a	c	g	t	t	g	c	c	a	t	t	g	a	c	c	t	c	t	c	g	t	g	t	g	a	t	c	t	t	a	t	t	c	a	g	a	g	t	a	t	a	t	c	t	t	t	t	a	t	c	t	t	t	t	g
KM355708	5	c	c	t	a	t	a	g	t	g	c	a	c	g	t	c	t	g	a	a	a	c	g	t	t	g	c	c	a	t	t	g	a	c	c	t	c	t	c	g	t	g	t	g	a	t	c	t	t	a	t	t	c	a	g	a	g	t	a	t	a	t	c	t	t	t	t	a	t	c	t	t	t	t	g
KM355709	5	c	c	t	a	t	a	g	t	g	c	a	c	g	t	c	t	g	a	a	a	c	g	t	t	g	c	c	a	t	t	g	a	c	c	t	c	t	c	g	t	g	t	g	a	t	c	t	t	a	t	t	c	a	g	a	g	t	a	t	a	t	c	t	t	t	t	a	t	c	c	t	t	t	g
KM355711	5	c	c	t	a	t	a	g	t	g	c	a	c	g	t	c	t	g	a	a	a	c	g	t	t	g	c	c	a	t	t	g	a	c	c	t	c	t	c	g	t	g	t	g	a	t	c	t	t	a	t	t	c	a	g	a	g	t	a	t	a	t	c	t	t	t	t	a	t	c	c	t	t	t	g
KM355712	5	c	c	t	a	t	a	g	t	g	c	a	c	g	t	c	t	g	a	a	a	c	g	t	t	g	c	c	a	t	t	g	a	c	c	t	c	t	c	g	t	g	t	g	a	t	c	t	t	a	t	t	c	a	g	a	g	t	a	t	a	t	c	t	t	t	t	a	t	c	c	t	t	t	g
KY037797	5	[illegible]	[illegible]	[illegible]	[illegible]	[illegible]	[illegible]	[illegible]	[illegible]	[illegible]	[illegible]	[illegible]	[illegible]	[illegible]	[illegible]	[illegible]	[illegible]	[illegible]	[illegible]	[illegible]	[illegible]	[illegible]	[illegible]	[illegible]	[illegible]	[illegible]	[illegible]	[illegible]	[illegible]	[illegible]	[illegible]	[illegible]	[illegible]	[illegible]	[illegible]	[illegible]	[illegible]	[illegible]	[illegible]	[illegible]	[illegible]	[illegible]	[illegible]	[illegible]	[illegible]	[illegible]	[illegible]	[illegible]	[illegible]	[illegible]	[illegible]	[illegible]	[illegible]	[illegible]	[illegible]	[illegible]	[illegible]	[illegible]	[illegible]	[illegible]	[illegible]	[illegible]	[illegible]	[illegible]	[illegible]	[illegible]	[illegible]	[illegible]	[illegible]	[illegible]	[illegible]	[illegible]	[illegible]	[illegible]	[illegible]

Supplementary Table 3: 2880 SN

GB-ID	Clade markers															9													2												5										2							SD14																	
	Position in Dumas	93395	93419	93590	93609	93664	93701	93803	93812	93818	93854	93998	94008	94020	94032	94095	94167	94173	94227	94260	94296	94320	94347	94443	94521	94545	94560	94599	94632	94641	94740	94778	94911	94941	95012	95014	95178	95226	95241	95262	95300	95308	95319	95326	95331	95333	95339	95355	95514	95546	95601	95610	95687	95691	95692	95693	95736	95845	95863	95896	95900	95905	95927	95934	95935	95941	95954	95958	95985	96029	96109	96148	96166	96371	96397
MH499467	5	c	c	t	a	t	a	g	t	g	c	a	t	g	t	c	t	g	a	a	a	c	g	t	t	g	c	c	a	t	t	g	a	c	c	t	c	t	c	g	t	g	t	g	a	t	c	t	t	a	t	t	c	a	g	a	g	t	a	t	a	t	c	t	t	t	t	a	t	c	t	t	t	t	g
MH499469	5	c	c	t	a	t	a	g	t	g	c	a	t	g	t	c	t	g	a	a	a	c	g	t	t	g	c	c	a	t	t	a	a	c	c	t	c	t	c	g	t	g	t	g	a	t	c	t	t	a	t	t	c	a	g	a	g	t	a	t	a	t	c	t	t	t	t	a	t	c	t	t	t	t	g
MH709321	5	c	c	t	c	t	a	g	t	g	c	a	t	g	t	c	t	g	a	a	a	c	g	t	t	g	c	c	a	t	t	g	a	c	c	t	c	t	c	g	t	g	t	g	a	t	c	t	t	a	t	t	c	a	g	a	g	t	a	t	a	t	c	t	t	t	t	a	t	c	t	t	t	t	g
MH709324	5	c	c	t	a	t	a	g	t	g	c	a	t	g	t	c	t	g	a	a	a	c	g	t	t	g	c	c	a	t	t	a	a	c	c	t	c	t	c	g	t	g	t	g	a	t	c	t	t	a	t	t	c	a	g	a	g	t	a	t	a	t	c	t	t	t	t	a	t	c	t	t	t	t	g
MH709325	5	c	c	t	a	t	a	g	t	g	c	a	c	g	t	c	t	g	a	a	a	c	g	t	t	g	c	c	a	t	t	g	a	c	c	t	c	t	c	g	t	g	t	g	a	t	c	t	t	a	t	t	c	a	g	a	g	t	a	t	a	t	c	t	t	t	t	a	t	c	c	t	t	t	g
MH709327	5	c	c	t	a	t	a	g	t	g	c	a	t	g	t	c	c	g	a	a	a	c	g	t	t	g	c	c	a	t	t	g	a	c	c	t	c	t	c	g	t	g	t	g	a	t	c	t	t	a	t	g	c	a	g	a	g	t	a	t	a	t	c	t	t	t	t	a	t	c	t	t	t	t	g
MH709328	5	c	c	t	a	t	a	g	t	g	c	a	t	g	t	c	t	g	a	a	a	c	g	t	t	g	c	c	a	t	t	a	a	c	c	t	c	t	c	g	t	g	t	g	a	t	c	t	t	a	t	t	c	a	g	a	g	t	g	t	a	t	c	t	t	t	t	g	t	c	t	t	t	t	g
MH709330	5	c	c	t	a	t	a	g	t	g	c	a	t	g	t	c	t	g	a	a	a	c	g	t	t	g	c	c	a	t	t	a	a	c	c	t	c	t	c	g	t	g	t	g	a	t	c	t	t	a	t	t	c	a	g	a	g	t	a	t	a	t	c	t	t	t	t	a	t	c	t	t	t	t	g
MH709332	5	c	c	t	a	t	a	g	t	g	c	a	t	g	t	c	t	g	a	a	a	c	g	t	t	g	c	c	a	t	t	a	a	c	c	t	c	t	c	g	t	g	t	g	a	t	c	t	t	a	t	t	c	a	g	a	g	t	a	t	a	t	c	t	t	t	t	a	t	c	t	t	t	t	g
MH709334	5	c	c	t	a	t	a	g	t	g	c	a	t	g	t	c	c	g	a	a	a	c	g	t	t	g	c	c	a	t	t	g	a	c	c	t	c	t	c	g	t	g	t	g	a	t	c	t	t	a	t	g	c	a	g	a	g	t	a	t	a	t	c	t	t	t	t	a	t	c	t	t	t	t	g
MH709335	5	c	c	t	a	t	a	g	t	g	c	a	t	g	t	c	c	g	a	a	a	c	g	t	t	g	c	c	a	t	t	g	a	c	c	t	c	t	c	g	t	g	t	g	a	t	c	t	t	a	t	g	c	a	g	a	g	t	a	t	a	t	c	t	t	t	t	a	t	c	t	t	t	t	g
MH709336	5	c	c	t	a	t	a	g	t	g	c	a	t	g	t	c	c	g	a	a	a	c	g	t	t	g	c	c	a	t	t	g	a	c	c	t	c	t	c	g	t	g	t	g	a	t	c	t	t	a	t	g	c	a	g	a	g	t	a	t	a	t	c	t	t	t	t	a	t	c	t	t	t	t	g
MH709338	5	c	c	t	a	t	a	g	t	g	c	a	t	g	t	c	c	g	a	a	a	c	g	t	t	g	c	c	a	t	t	g	a	c	c	t	c	t	c	g	t	g	t	g	a	t	c	t	t	a	t	g	c	a	g	a	g	t	a	t	a	t	c	t	t	t	t	a	t	c	t	t	t	t	g
MH709340	5	c	c	t	a	t	a	g	t	g	c	a	t	g	t	c	t	g	a	a	a	c	a	t	t	g	c	c	a	t	t	g	a	c	c	t	c	t	c	g	t	g	t	g	a	t	c	t	t	a	t	t	c	a	g	a	g	t	a	t	a	t	c	t	t	t	t	a	t	c	t	t	t	t	g
MH709342	5	c	c	t	a	t	a	g	t	g	c	a	t	g	t	c	t	g	a	a	a	c	g	t	t	g	c	c	a	t	t	g	a	c	c	t	c	t	c	g	t	g	t	g	a	t	c	t	t	a	t	t	c	a	g	a	g	t	a	t	a	t	c	t	t	t	t	a	t	c	t	t	t	t	g
MH709343	5	c	c	t	a	t	a	g	t	g	c	a	t	g	t	c	t	g	a	a	a	c	g	t	t	g	c	c	a	t	t	g	a	c	c	t	c	t	c	g	t	g	t	g	a	t	c	t	t	a	t	t	c	a	g	a	g	t	a	t	a	t	c	t	t	t	t	a	t	c	t	t	t	t	g
MH709345	5	c	c	t	a	t	a	g	t	g	c	a	t	g	t	c	c	g	a	a	a	c	g	t	t	g	c	c	a	t	t	g	a	c	c	t	c	t	c	g	t	g	t	g	a	t	c	t	t	a	t	g	c	a	g	a	g	t	a	t	a	t	c	t	t	t	t	a	t	c	t	t	t	t	g
MH709347	5	c	c	t	a	t	a	g	t	g	c	a	t	g	t	c	c	g	a	a	a	c	g	t	t	g	c	c	a	t	t	g	a	c	c	t	c	t	c	g	t	g	t	g	a	t	c	t	t	a	t	g	c	a	g	a	g	t	a	t	a	t	c	t	t	t	t	a	t	c	t	t	t	t	g
MH709349	5	c	c	t	a	t	a	g	t	g	c	a	t	g	t	c	t	g	a	a	a	c	g	t	t	g	c	c	a	t	t	g	a	c	c	t	c	t	c	g	t	g	t	g	a	t	c	t	t	a	t	t	c	a	g	a	g	t	a	t	a	t	c	t	t	t	t	a	t	c	t	t	t	t	g
MH709350	5	c	c	t	a	t	a	g	t	g	c	a	t	g	t	c	c	g	a	a	a	c	g	t	t	g	c	c	a	t	t	g	a	c	c	t	c	t	c	g	t	g	t	g	a	t	c	t	t	a	t	g	c	a	g	a	g	t	a	t	a	t	c	t	t	t	t	a	t	c	t	t	t	t	g
MH709351	5	c	c	t	a	t	a	g	t	g	c	a	t	g	t	c	t	g	a	a	a	c	g	t	t	g	c	c	a	t	t	g	a	c	c	t	c	t	c	g	t	g	t	g	a	t	c	t	t	a	t	t	c	a	g	a	g	t	a	t	a	t	c	t	t	t	t	a	t	c	t	t	t	t	g
MH709353	5	c	c	t	a	t	a	g	t	g	c	a	t	g	t	c	c	g	a	a	a	c	g	t	t	g	c	c	a	t	t	g	a	c	c	t	c	t	c	g	t	g	t	g	a	t	c	t	t	a	t	g	c	a	g	a	g	t	a	t	a	t	c	t	t	t	t	a	t	c	t	t	t	t	g
MH709358	5	c	c	t	a	t	a	g	t	g	c	a	c	g	t	c	t	g	a	a	a	c	g	t	t	g	c	c	a	t	t	g	a	c	c	c	c	t	c	g	t	g	t	g	a	t	c	t	t	a	t	t	c	a	g	a	g	t	a	t	a	t	c	t	t	t	t	a	t	c	t	t	t	t	g
MH709360	5	c	c	t	a	t	a	g	t	g	c	a	t	g	t	c	t	g	a	a	a	c	g	t	t	g	c	c	a	t	t	a	a	c	c	t	c	t	c	g	t	g	t	g	a	t	c	t	t	a	t	t	c	a	g	a	g	t	a	t	a	t	c	t	t	t	t	a	t	c	t	t	t	t	g
MH709362	5	c	c	t	a	t	a	g	t	g	c	a	t	g	t	c	c	g	a	a	a	c	g	t	t	g	c	c	a	t	t	g	a	c	c	t	c	t	c	g	t	g	t	g	a	t	c	t	t	a	t	g	c	a	g	a	g	t	a	t	a	t	c	t	t	t	t	a	t	c	t	t	t	t	g
MH709365	5	c	c	t	a	t	a	g	t	g	c	a	t	g	t	c	c	g	a	a	a	c	g	t	t	g	c	c	a	t	t	g	a	c	c	t	c	t	c	g	t	g	t	g	a	t	c	t	t	a	t	g	c	a	g	a	g	t	a	t	a	t	c	t	t	t	t	a	t	c	t	t	t	t	g
MH709373	5	c	c	t	a	t	a	g	t	g	c	a	t	g	t	c	t	g	a	a	a	c	g	t	t	g	c	c	a	t	c	g	a	c	c	t	c	t	c	g	t	g	t	g	a	t	c	t	t	a	t	t	c	a	g	a	g	t	a	t	a	t	c	t	t	t	t	a	t	c	t	t	t	t	g
MH709374	5	c	c	t	a	t	a	g	t	g	c	a	t	g	t	c	c	g	a	a	a	c	g	t	t	g	c	c	a	t	t	g	a	c	c	t	c	t	c	g	t	g	t	g	a	t	c	t	t	a	t	g	c	a	g	a	g	t	a	t	a	t	c	t	t	t	t	a	t	c	t	t	t	t	g
KC112914	6	c	c	t	a	t	a	g	t	g	c	a	t	g	t	c	t	g	a	a	a	c	g	t	t	g	c	c	a	a	t	g	a	c	c	t	c	t	t	g	c	g	t	g	a	t	c	t	t	g	t	t	c	a	g	a	g	t	a	g	a	t	c	t	t	t	t	a	t	c	t	t	t	t	g
KP771914	6	c	c	t	a	t	a	g	t	g	c	a	t	g	t	c	t	g	a	a	a	c	g	c	t	g	c	c	a	a	t	g	a	c	c	t	c	t	t	g	c	g	t	g	a	t	c	t	t	g	t	t	c	a	g	a	g	t	a	g	a	t	c	t	t	t	t	a	t	c	t	t	t	t	g
KP702725	6	c	c	t	a	t	a	g	t	g	c	a	t	g	t	c	t	g	a	a	a	c	g	t	t	g	c	c	a	a	t	g	a	c	c	t	c	t	t	g	c	g	t	g	a	t	c	t	t	g	t	t	c	a	g	a	g	t	a	g	a	t	c	t	t	t	t	a	t	c	t	t	t	t	g
KP771890	9	c	c	t	a	t	a	g	t	g	c	a	t	g	t	t	t	g	a	a	a	c	g	t	t	g	c	c	a	t	t	g	a	c	c	t	c	t	c	g	c	g	t	g	a	t	c	t	t	a	t	t	c	a	g	a	g	t	a	t	a	t	c	t	t	t	t	a	t	c	t	t	t	t	g
JN704710	9	c	c	t	a	t	a	g	t	g	c	a	t	g	t	t	t	g	a	a	a	c	g	t	t	g	c	c	a	t	t	g	a	c	c	t	c	t	c	g	c	g	t	g	a	t	c	t	t	g	t	t	c	a	t	c	g	t	a	t	a	t	c	t	t	t	t	a	t	c	t	t	t	t	g
KY037798	9	c	c	t	a	t	a	g	t	g	c	a	t	g	t	t	t	g	a	a	a	c	g	t	t	g	c	c	a	t	t	g	a	c	c	t	c	t	c	g	c	g	t	g	a	t	c	t	t	a	t	t	c	a	g	a	g	t	a	t	a	t	c	t	t	t	t	a	t	c	t	t	t	t	g
MH709312	9	c	c	t	a	t	a	g	t	g	c	a	t	g	t	t	t	g	a	a	a	c	g	t	t	g	c	c	a	t	t	g	a	c	c	t	c	t	c	g	c	g	t	g	a	t	c	t	t	a	t	t	c	a	g	a	g	t	a	t	a	t	c	t	t	t	t	a	t	c	t	t	t	t	g
MH709352	9	c	c	t	a	t	a	g	t	g	c	a	t	g	t	t	t	g	a	a	a	c	g	t	t	g	c	c	a	t	t	g	a	c	c	t	c	t	c	g	c	g	t	g	a	t	c	t	t	a	t	t	c	a	g	a	g	t	a	t	a	t	c	t	t	t	t	a	t	c	t	t	t	t	g
AB097933	Vac	c	c	t	a	t	a	g	t	g	c	a	t	g	t	c	t	g	a	a	a	c	g	t	t	g	c	c	g	t	t	g	a	c	c	t	c	t	c	g	c	g	t	g	a	t	c	t	t	a	g	t	c	a	g	a	g	t	a	t	a	t	c	t	t	t	t	a	t	c	t	t	t	t	g
JN704698	Vac	c	c	t	a	t	a	g	t	g	c	a	t	g	t	c	t	g	a	a	a	c	g	t	t	g	c	c	g	t	t	g	a	c	c	t	c	t	c	g	c	g	t	g	a	t	c	t	t	a	g	t	c	a	g	a	g	t	a	t	a	t	c	t	t	t	t	a	t	c	t	t	t	t	g
JN704697	Vac	c	c	t	a	t	a	g	t	g	c	a	t	g	t	c	c	g	a	a	a	c	g	t	t	g	c	c	g	t	t	g	a	c	c	t	c	t	c	g	c	g	t	g	a	t	c	t	t	a	g	t	c	a	g	a	g	t	a	t	a	t	c	t	t	t	t	a	t	c	t	t	t	t	g
DQ008355	Vac	c	c	t	a	t	a	g	t	g	c	a	t	g	t	c	y	g	a	a	a	c	g	t	t	g	c	c	g	t	t	g	a	c	c	t	c	t	c	g	c	g	t	g	a	t	c	t	t	a	g	t	c	a	g	a	g	t	a	t	a	t	c	t	t	t	t	a	t	c	t	t	t	t	g
JF306641	Vac	c	c	t	a	t	a	g	t	g	c	a	t	g	t	c	c	g	a	a	a	c	g	t	t	g	c	c	g	t	t	g	a	c	c	t	c	t	c	g	c	g	t	g	a	t	c	t	t	a	g	t	c	a	g	a	g	t	a	t	a	t	c	t	t	t	t	a	t	c	t	t	t	t	g
AB097932	Vac	c	c	t	a	t	a	g	t	g	c	a	t	g	t	c	c	g	a	a	a	c	g	t	t	g	c	c	g	t	t	g	a	c	c	t	c	t	c	g	c	g	t	g	a	t	c	t	t	a	g	t	c	a	g	a	g	t	a	t	a	t	c	t	t	t	t	a	t	c	t	t	t	t	g
DQ008354	Vac	c	c	t	a	t	a	g	t	g	c	a	t	g	t	c	c	g	a	a	a	c	g	t	t	g	c	c	g	t	t	g	a	c	c	t	c	t	c	g	c	g	t	g	a	t	c	t	t	a	g	t	c	a	g	a	g	t	a	t	a	t	c	t	t	t	t	a	t	c	t	t	t	t	g
KU926312	Vac	c	c	t	a	t	a	g	t	g	c	a	t	g	t	c	c	g	a	a	a	c	g	t	t	g	c	c	g	t	t	g	a	c	c	t	c	t	c	g	c	g	t	g	a	t	c	t	t	a	g	t	c	a	g	a	g	t	a	t	a	t	c	t	t	t	t	a	t	c	t	t	t	t	g
KU926313	Vac	c	c	t	a	t	a	g	t	g	c	a	t	g	t	c	c	g	a	a	a	c	g	t	t	g	c	c	g	t	t	g	a	c	c	t	c	t	c	g	c	g	t	g	a	t	c	t	t	a	g	t	c	a	g	a	g	t	a	t	a	t	c	t	t	t	t	a	t	c	t	t	t	t	g
KU926314	Vac	c	c	t	a	t	a	g	t	g	c	a	t	g	t	c	c	g	a	a	a	c	g	t	t	g	c	c	g	t	t	g	a	c	c	t	c	t	c	g	c	g	t	g	a	t	c	t	t	a	g	t	c	a	g	a	g	t	a	t	a	t	c	t	t	t	t	a	t	c	t	t	t	t	g
KF558371	Vac	c	c	c	a	t	a	g	t	g	c	a	t	g	t	c	c	g	a	a	a	c	g	t	t	g	c	c	g	t	t	g	a	c	c	t	c	t	c	g	c	g	t	g	a	t	c	t	t	a	g	t	c	a	g	a	g	t	a	t	a	t	c	t	t	t	t	a	t	c	t	t	t	t	g
KF558372	Vac	c	c	t	a	t	a	g	t	g	c	a	t	g	t	c	c	g	a	a	a	c	g	t	t	g	c	c	g	t	t	g	a	c	c	t	c	t	c	g	c	g	t	g	a	t	c	t	t	a	g	t	c	a	g	a	g	t	a	t	a	t	c	t	t	t	t	a	t	c	t	t	t	t	g
KF558373	Vac	c	c	t	a	t	a	g	t	g	c	a	t	g	t	c	c	g	a	a	a	c	g	t	t	g	c	c	g	t	t	g	a	c	c	t	c	t	c	g	c	g	t	g	a	t	c	t	t	a	g	t	c	a	g	a	g	t	a	t	a	t	c	t	t	t	t	a	t	c	t	t	t	t	g
KF558374	Vac	c	c	t	a	t	a	g	t	g	c	a	t	g	t	c	c	g	a	a	a	c	g	t	t	g	c	c	g	t	t	g	g	c	c	t	c	t	c	g	c	g	t	g	a	t	c	t	t	a	g	t	c	a	g	a	g	t	a	t	a	t	c	t	t	t	t	a	t	c	t	t	t	t	g
KF558375	Vac	c	c	c	a	t	a	g	t	g	c	a	t	g	t	c	c	g	a	a	a	c	g	t	t	g	c	c	g	t	t	g	a	c	c	t	c	t	c	g	c	g	t	g	a	t	c	t	t	a	g	t	c	a	g	a	g	t	a	t	a	t	c	t	t	t	t	a	t	c	t	t	t	t	g
KF558376	Vac	c	c	t	a	t	a	g	t	g	c	a	t	g	t	c	c	g	a	a	a	c	g	t	t	g	c	c	g	t	t	g	a	c	c	t	c	t	c	g	c	g	t	g	a	t	c	t	t	a	g	t	c	a	g	a	g	t	a	t	a	t	c	t	t	t	t	a	t	c	t	t	t	t	g
KF558377	Vac	c	c	t	a	t	a	g	t	g	c	a	t	g	t	c	c	g	a	a	a	c	g	t	t	g	c	c	g	t	t	g	a	c	c	t	c	t	c	g	c	g	t	g	a	t	c	t	t	a	g	t	c	a	g	a	g	t	a	t	a	t	c	t	t	t	t	a	t	c	t	t	t	t	g
KF558378	Vac	c	c	t	a	t	a	g	t	g	c	a	t	g	t	c	c	g	a	a	a	c	g	t	t	g	c	c	g	t	t	g	a	c	c	t	c	t	c	g	c	g	t	g	a	t	c	t	t	a	g	t	c	a	g	a	g	t	a	t	a	t	c	t	t	t	t	a	t	c	t	t	t	t	g
KF558379	Vac	c	c	t	a	t	a	g	t	g	c	a	t	g	t	c	t	g	a	a	a	c	g	t	t	g	c	c	g	t	t	g	a	c	c	t	c	t	c	g	c	g	t	g	a	t	c	t	t	a	g	t	c	a	g	a	g	t	a	t	a	t	c	t	t	t	t	a	t	c	t	t	t	t	g
KF558380	Vac	c	c	t	a	t	a	g	t	g	c	a	t	g	t	c	c	g	a	a	a	c	g	t	t	g	c	c	g	t	t	g	a	c	c	t	c	t	c	g	c	g	t	g	a	t	c	t	t	a	g	t	c	a	g	a	g	t	a	t	a	t	c	t	t	t	t	a	t	c	t	t	t	t	g
KF558381	Vac	c	c	t	a	t	a	g	t	g	c	a	t	g	t	c	t	g	a	g	a	c	g	t	t	g	c	c	g	t	t	g	a	c	c	t	c	t	c	g	c	g	t	g	a	t	c	t	t	a	g	t	c	a	g	a	g	t	a	t	a	t	c	t	t	t	t	a	t	c	t	t	t	t	g
KF558382	Vac	c	c	t	a	t	a	g	t	g	c	a	t	g	t	c	c	g	a	a	a	c	g	t	t	g	c	c	g	t	t	g	a	c	c	t	c	t	c	g	c	g	t	g	a	t	c	t	t	a	g	t	c	a	g	a	g	t	a	t	a	t	c	t	t	t	t	a	t	c	t	t	t	t	g
KF558383	Vac	c	c	t	a	t	a	g	t	g	c	a	t	g	t	c	c	g	a	a	a	c	g	t	t	g	c	c	g	t	t	g	a	c	c	t	c	t	c	g	c	g	t	g	a	t	c	t	t	a	g	t	c	a	g	a	g	t	a	t	a	t	c	t	t	t	t	a	t	c	t	t	t	t	g
KF558384	Vac	c	c	t	a	t	a	g	t	g	c	a	t	g	t	c	c	g	a	a	a	c	g	t	t	g	c	c	g	t	t	g	a	c	c	t	c	t	c	g	c	g	t	g	a	t	c	t	t	a	g	t	c	a	g	a	g	t	a	t	a	t	c	t	t	t	t	a	t	c	t	t	t	t	g
KF558385	Vac	c	c	t	a	t	a	g	t	g	c	a	t	g	t	c	c	g	a	a	a	c	g	t	t	g	c	c	g	t	t	g	a	c	c	t	c	t	c	g	c	g	t	g	a	t	c	t	t	a	g	t	c	a	g	a	g	t	a	t	a	t	c	t	t	t	t	a	t	c	t	t	t	t	g
KF558386	Vac	c	c	t	a	t	a	g	t	g	c	a	t	g	t	c	c	g	a	a	a	c	g	t	t	g	c	c	g	t	t	g	a	c	c	t	c	t	c	g	c	g	t	g	a	t	c	t	t	a	g	t	c	a	g	a	g	t	a	t	a	t	c	t	t	t	t	a	t	c	t	t	t	t	g
KF558387	Vac	c	c	t	a	t	a	g	t	g	c	a	t	g	t	c	c	g	a	a	a	c	g	t	t	g	c	c	g	t	t	g	a	c	c	t	c	t	c	g	c	g	t	g	a	t	c	t	t	a	g	t	c	a	g	a	g	t	a	t	a	t	c	t	t	t	t	a	t	c	t	t	t	t	g
KF558388	Vac	c	c	t	a	t	a	g	t	g	c	a	t	g	t	c	c	g	a	a	a	c	g	t	t	g	c	c	g	t	t	g	a	c	c	t	c	t	c	g	c	g	t	g	a	t	c	t	t	a	g	t	c	a	g	a	g	t	a	t	a	t	c	t	t	t	t	a	t	c	t	t	t	t	g
KF558389	Vac	c	c	t	a	t	a	g	t	g	c	a	t	g	t	c	c	g	a	a	a	c	g	t	t	g	c	c	g	t	t	g	g	c	c	t	c	t	c	g	c	g	t	g	a	t	c	t	t	a	g	t	c	a	g	a	g	t	a	t	a	t	c	t	t	t	t	a	t	c	t	t	t	t	g
KF558390	Vac	c	c	c	a	t	a	g	t	g	c	a	t	g	t	c	c	g	a	a	a	c	g	t	t	g	c	c	g	t	t	g	a	c	c	t	c	t	c	g	c	g	t	g	a	t	c	t	t	a	g	t	c	a	g	a	g	t	a	t	a	t	c	t	t	t	t	a	t	c	t	t	t	t	g
KF558391	Vac	c	c	t	a	t	a	g	t	g	c	a	t	g	t	c	c	g	a	a	a	c	g	t	t	g	c	c	g	t	t	g	a	c	c	t	c	t	c	g	c	g	t	g	a	t	c	t	t	a	g	t	c	a	g	a	g	t	a	t	a	t	c	t	t	t	t	a	t	c	t	t	t	t	g
KF811485	Vac	c	c	t	a	t	a	g	t	g	c	a	t	g	t	c	c	g	a	a	a	c	g	t	t	g	c	c	g	t	t	g	a	c	c	t	c	t	c	g	c	g	t	g	a	t	c	t	t	a	g	t	c	a	g	a	g	t	a	t	a	t	c	t	t	t	t	a	t	c	t	t	t	t	g
KF853225	Vac	c	c	t	a	t	a	g	t	g	c	a	t	g	t	c	c	g	a	a	a	c	g	t	t	g	c	c	g	t	t	g	a	c	c	t	c	t	c	g	c	g	t	g	a	t	c	t	t	a	g	t	c	a	g	a	g	t	a	t	a	t	c	t	t	t	t	a	t	c	t	t	t	t	g
KF853226	Vac	c	c	t	a	t	a	g	t	g	c	a	t	g	t	c	c	g	a	a	a	c	g	t	t	g	c	c	g	t	t	g	a	c	c	t	c	t	c	g	c	g	t	g	a	t	c	t	t	a	g	t	c	a	g	a	g	t	a	t	a	t	c	t	t	t	t	a	t	c	t	t	t	t	g
KF853227	Vac	c	c	t	a	t	a	g	t	g	c	a	t	g	t	c	c	g	a	a	a	c	g	t	t	g	c	c	g	t	t	g	a	c	c	t	c	t	c	g	c	g	t	g	a	t	c	t	t	a	g	t	c	a	g	a	g	t	a	t	a	t	c	t	t	t	t	a	t	c	t	t	t	t	g
KF853228	Vac	c	c	t	a	t	a	g	t	g	c	a	t	g	t	c	c	g	a	a	a	c	g	t	t	g	c	c	g	t	t	g	a	c	c	t	c	t	c	g	c	g	t	g	a	t	c	t	t	a	g	t	c	a	g	a	g	t	a	t	a	t	c	t	t	t	t	a	t	c	t	t	t	t	g
KF853229	Vac	c	c	t	a	t	a	g	t	g	c	a	t	g	t	c	c	g	a	a	a	c	g	t	t	g	c	c	g	t	t	g	a	c	c	t	c	t	c	g	c	g	t	g	a	t	c	t	t	a	g	t	c	a	g	a	g	t	a	t	a	t	c	t	t	t	t	a	t	c	t	t	t	t	g
KF853230	Vac	c	c	t	a	t	a	g	t	g	c	a	t	g	t	c	c	g	a	a	a	c	g	t	t	g	c	c	g	t	t	g	a	c	c	t	c	t	c	g	c	g	t	g	a	t	c	t	t	a	g	t	c	a	g	a	g	t	a	t	a	t	c	t	t	t	t	a	t	c	t	t	t	t	g
KF853231	Vac	c	c	t	a	t	a	g	t	g	c	a	t	g	t	c	c	g	a	a	a	c	g	t	t	g	c	c	g	t	t	g	a	c	c	t	c	t	c	g	c	g	t	g	a	t	c	t	t	a	g	t	c	a	g	a	g	t	a	t	a	t	c	t	t	t	t	a	t	c	t	t	t	t	g
KF853232	Vac	c	c	t	a	t	a	g	t	g	c	a	t	g	t	c	t	g	a	a	a	c	g	t	t	g	c	c	g	t	t	g	g	c	c	t	c	t	c	g	c	g	t	g	a	t	c	t	t	a	g	t	c	a	g	a	g	t	a	t	a	t	c	t	t	t	t	a	t	c	t	t	t	t	g
KF853233	Vac	c	c	t	a	t	a	g	t	g	c	a	t	g	t	c	t	g	a	a	a	c	g	t	t	g	c	c	g	t	t	g	a	c	c	t	c	t	c	g	c	g	t	g	a	t	c	t	c	a	g	t	c	a	g	a	g	t	a	t	a	t	c	t	t	t	t	a	t	c	t	t	t	t	g
KF853234	Vac	c	c	t	a	t	a	g	t	g	c	a	t	g	t	c	t	g	a	a	a	c	g	t	t	g	c	c	g	t	t	g	a	c	c	t	c	t	c	g	c	g	t	g	a	t	c	t	t	a	g	t	c	a	g	a	g	t	a	t	a	t	c	t	t	t	t	a	t	c	t	t	t	t	g
KF853235	Vac	c	c	t	a	t	a	g	t	g	c	a	t	g	t	c	c	g	a	a	a	c	g	t	t	g	c	c	g	t	t	g	a	c	c	t	c	t	c	g	c	g	t	g	a	t	c	t	t	a	g	t	c	a	g	a	g	t	a	t	a	t	c	t	t	t	t	a	t	c	t	t	t	t	g
MF898328	Vac	c	c	t	a	t	a	g	t	g	c	a	t	g	t	c	c	g	a	a	a	c	g	t	t	g	c	c	g	t	t	g	a	c	c	t	c	t	c	g	c	g	t	g	a	t	c	t	t	a	g	t	c	a	g	a	g	t	a	t	a	t	c	t	t	t	t	a	t	c	t	t	t	t	g
JN704709	VIII	c	c	t	a	g	a	g	t	g	c	a	t	g	t	c	t	g	a	a	a	c	g	t	t	g	c	c	a	a	t	g	a	c	c	t	c	t	t	g	c	g	t	g	a	t	c	t	t	g	t	t	c	a	g	a	g	t	a	g	a	t	c	t	t	t	t	a	t	c	t	t	t	t	g

Supplementary Table 3: 2880 SN

GB-ID	Clade markers																										2						5		9							9									2	2																									
	Position in Dumas	96444	96491	96499	96553	96607	96615	96629	96724	96739	96791	96904	96920	96937	97034	97066	97081	97093	97141	97162	97222	97276	97399	97444	97479	97495	97525	97591	97697	97748	97780	97796	97804	97842	97891	97954	98261	98262	98264	98365	98410	98437	98468	98494	98647	98659	98662	98676	98692	98712	98742	98765	98807	98809	98816	98825	98832	98893	98897	98911	98960	99004	99008	99041	99064_1	99071	99137	99186	99223	99225	99227	99228	99235	99256
AY548170	1	a	t	g	a	a	g	t	t	a	a	a	g	t	a	t	t	c	t	t	g	a	a	t	t	a	a	c	t	g	g	t	t	a	t	c	t	a	g	a	a	t	a	t	t	c	a	t	t	a	a	t	a	t	a	t	a	t	a	t	t	t	g	a	-	g	g	t	c	t	t	t	a	a
AY548171	1	a	t	g	a	a	g	t	t	a	a	a	g	t	a	t	t	c	t	t	g	a	a	t	t	a	a	c	t	g	g	t	t	a	t	c	t	a	g	a	a	t	a	t	t	c	a	t	t	a	a	t	a	t	a	t	a	t	a	t	t	t	g	a	-	g	g	c	c	t	t	t	a	a
DQ479953	1	a	t	g	a	a	g	t	t	a	a	a	g	t	a	t	t	t	t	t	g	a	a	t	t	a	a	c	t	g	g	t	t	a	t	c	t	a	g	a	a	t	a	t	t	c	a	t	t	a	a	t	a	t	a	t	a	t	a	t	t	t	g	a	-	g	g	t	c	t	t	t	a	a
DQ479954	1	a	t	g	a	a	g	t	t	a	a	a	g	t	a	t	t	c	t	t	a	a	a	t	t	a	a	c	t	g	g	t	t	a	t	c	t	a	g	a	a	t	a	t	t	c	a	t	t	a	a	t	a	t	a	t	a	t	a	t	t	t	g	a	-	g	g	t	c	t	t	t	a	a
DQ479958	1	a	t	g	a	a	g	t	t	a	a	a	g	t	a	t	t	c	t	t	g	a	a	t	t	a	a	c	t	g	g	t	t	a	t	c	t	a	g	a	a	t	a	t	t	c	a	t	t	a	a	t	a	t	a	t	a	t	a	t	t	t	g	a	-	g	g	c	c	t	t	t	a	a
DQ479959	1	a	t	g	a	a	g	t	t	a	a	a	g	t	a	t	t	c	t	t	g	a	a	t	t	a	a	c	t	g	g	t	t	a	t	c	t	a	g	a	a	t	a	t	t	c	a	t	t	a	a	t	a	t	a	t	a	t	a	t	t	t	g	a	-	g	g	c	c	t	t	t	a	a
DQ479961	1	a	t	g	a	a	g	t	t	a	a	a	g	t	a	t	t	c	t	t	g	a	a	t	t	a	a	c	t	g	g	t	t	a	t	c	t	a	g	a	a	t	a	t	t	c	a	t	t	a	a	t	a	t	a	t	a	t	a	t	t	t	g	a	-	g	g	t	c	t	t	t	a	a
DQ479962	1	a	t	g	a	a	g	t	t	a	a	a	g	t	a	t	t	c	t	t	g	a	a	t	t	a	a	c	t	g	g	t	t	a	t	c	t	a	g	a	a	t	a	t	t	c	a	t	t	a	a	t	a	t	a	t	a	t	a	t	t	t	g	a	-	g	g	t	c	t	t	t	a	a
DQ479963	1	a	t	g	a	a	g	t	t	a	a	a	g	t	a	t	t	c	t	t	g	a	a	t	t	a	a	c	t	g	g	t	t	a	t	c	t	a	g	a	a	t	a	t	t	c	a	t	t	a	a	t	a	t	a	t	a	t	a	t	t	t	g	a	-	g	g	t	c	t	t	t	a	a
DQ674250	1	a	t	g	a	a	g	t	t	a	a	a	g	t	a	t	t	c	t	t	g	a	a	t	t	a	a	c	t	g	g	t	t	a	t	c	t	a	g	a	a	t	a	t	t	c	a	t	t	a	a	t	a	t	a	t	a	t	a	t	t	t	g	a	-	g	g	t	c	t	t	t	a	a
KX262864	1	a	t	g	a	a	g	t	t	a	a	a	g	t	a	t	t	c	t	t	g	a	a	t	t	a	a	c	t	g	g	t	t	a	t	c	t	a	g	a	a	t	a	t	t	c	a	t	t	a	a	t	a	t	a	t	a	t	a	t	t	t	g	a	-	g	g	c	c	t	t	t	a	a
KX262865	1	a	t	g	a	a	g	t	c	a	a	a	g	t	a	t	t	c	t	t	g	a	a	t	t	a	a	c	t	g	g	t	t	a	t	c	t	a	g	a	a	t	a	t	t	c	a	t	t	a	a	t	a	t	a	t	a	t	a	t	t	t	g	a	-	g	g	c	c	t	t	t	a	a
KX262866	1	a	t	g	a	a	g	t	t	a	a	a	g	t	a	t	t	c	t	t	g	a	a	t	t	a	a	c	t	g	g	t	t	a	t	c	t	a	g	a	a	t	a	t	t	c	a	t	t	a	a	t	a	t	a	t	a	t	a	t	t	t	g	a	-	g	g	c	c	t	t	t	a	a
EU154348	1	a	t	g	a	a	g	t	t	a	a	a	g	t	a	t	t	c	t	t	g	a	a	t	t	a	a	c	t	g	g	t	t	a	t	c	t	a	g	a	a	t	a	t	t	c	a	t	t	a	a	t	a	t	a	t	a	t	a	t	t	t	g	a	-	g	g	t	c	t	t	t	a	a
JN704690	1	a	t	g	a	a	g	t	t	a	a	a	g	t	a	t	t	c	t	t	g	a	a	t	t	a	a	c	t	g	g	t	t	a	t	c	t	a	g	a	a	t	a	t	t	c	a	t	t	a	a	t	a	t	a	t	a	t	a	t	t	t	g	a	-	g	g	t	c	t	t	t	a	a
JN704691	1	a	t	g	a	a	g	t	t	a	a	a	g	t	a	t	t	c	t	t	g	a	a	t	t	a	a	c	t	g	g	t	t	a	t	c	t	a	g	a	a	t	a	t	t	c	a	t	t	a	a	t	a	t	a	t	a	t	a	t	t	t	g	a	-	g	g	t	c	t	t	t	a	a
JN704692	1	a	t	g	a	a	g	t	t	a	a	a	g	t	a	t	t	c	t	t	g	a	a	t	t	a	a	c	t	g	g	t	t	a	t	c	t	a	g	a	a	t	a	t	t	c	a	t	t	a	a	t	a	t	a	t	a	t	a	t	t	t	g	a	-	g	g	t	c	t	t	t	a	a
JN704693	1	a	t	g	a	a	g	t	t	a	a	a	g	t	a	t	t	c	t	t	g	a	a	t	t	a	a	c	t	g	g	t	c	a	t	c	t	a	g	a	a	t	a	t	t	c	a	t	t	a	a	t	a	t	a	t	a	t	a	t	t	t	g	a	-	g	g	t	c	t	t	t	a	a
JN704694	1	a	t	g	a	a	g	t	t	a	a	a	g	t	a	t	t	c	t	t	g	a	a	t	t	a	a	c	t	g	g	t	t	a	t	c	t	a	g	a	a	t	a	t	t	c	a	t	t	a	a	t	a	t	a	t	a	t	a	t	t	t	g	a	-	g	g	t	c	t	t	t	a	a
JN704695	1	a	t	g	a	a	g	t	t	a	a	a	g	t	a	t	t	c	t	t	g	a	a	t	t	a	a	c	t	g	g	t	t	a	t	c	t	a	g	a	a	t	a	t	t	c	a	t	t	a	a	t	a	t	a	t	a	t	a	t	t	t	g	a	-	g	g	t	c	t	t	t	a	a
JN704696	1	a	t	g	a	a	g	t	t	a	a	a	g	t	a	t	t	c	t	t	g	a	a	t	t	a	a	c	t	g	g	t	t	a	t	c	t	a	g	a	a	t	a	t	t	c	a	t	t	a	a	t	a	t	a	t	a	t	a	t	t	t	g	a	-	g	g	t	c	t	t	t	a	a
NC001348	1	a	t	g	a	a	g	t	t	a	a	a	g	t	a	t	t	c	t	t	g	a	a	t	t	a	a	c	t	g	g	t	t	a	t	c	t	a	g	a	a	t	a	t	t	c	a	t	t	a	a	t	a	t	a	t	a	t	a	t	t	t	g	a	-	g	g	t	c	t	t	t	a	a
KP771892	1	a	t	g	a	a	g	t	t	a	a	a	g	t	a	t	t	c	t	t	g	a	a	t	t	a	a	c	t	g	g	t	t	a	t	c	t	a	g	a	a	t	a	t	t	c	a	t	t	a	a	t	a	t	a	t	a	t	a	t	t	t	g	a	-	g	g	t	c	t	t	t	a	a
KP771894	1	a	t	g	a	a	g	t	t	a	a	a	g	t	a	t	t	c	t	t	g	a	a	t	t	a	a	c	t	g	g	t	t	a	t	c	t	a	g	a	a	t	a	t	t	c	a	t	t	a	a	t	a	t	a	t	a	t	a	t	t	t	g	a	-	a	g	t	c	t	t	t	a	a
KP771900	1	a	t	g	a	a	g	t	t	a	a	a	g	t	a	t	t	c	t	t	g	a	a	t	t	a	a	c	t	g	g	t	t	a	t	c	t	a	g	a	a	t	a	t	t	c	a	t	t	a	a	t	a	t	a	t	a	t	a	t	t	t	g	a	-	g	g	t	c	t	t	t	a	a
KP771901	1	a	t	g	a	a	g	t	t	a	a	a	g	t	a	t	t	c	t	t	g	a	a	t	t	a	a	c	t	g	g	t	t	a	t	c	t	a	g	a	a	t	a	t	t	c	a	t	t	a	a	t	a	t	a	t	a	t	a	t	t	t	g	a	-	g	g	t	c	t	t	t	a	a
KP771902	1	a	t	g	a	a	g	t	t	a	a	a	g	t	a	t	t	c	t	t	g	a	a	t	t	a	a	c	t	g	g	t	t	a	t	c	t	a	g	a	a	t	a	t	t	c	a	t	t	a	a	t	a	t	a	t	a	t	a	t	t	t	g	a	-	g	g	t	c	t	t	t	a	a
KP771908	1	a	t	g	a	a	g	t	t	a	a	a	g	t	a	t	t	c	t	t	g	a	a	t	t	a	a	c	t	g	g	t	t	a	t	c	t	a	g	a	a	t	a	t	t	c	a	t	t	a	a	t	a	t	a	t	a	t	a	t	t	t	g	a	-	g	g	c	c	t	t	t	a	a
KP771912	1	a	t	g	a	a	g	t	t	a	a	a	g	t	a	t	t	c	t	t	g	a	a	t	t	a	a	c	t	g	g	t	t	a	t	c	t	a	g	a	a	t	a	t	t	c	a	t	t	a	a	t	a	t	a	t	a	t	a	t	t	t	g	a	-	g	g	c	c	t	t	t	a	a
KP771913	1	a	t	g	a	a	g	t	t	a	a	a	g	t	a	t	t	c	t	t	g	a	a	t	t	a	a	c	t	g	g	t	t	a	t	c	t	a	g	a	a	t	a	t	t	c	a	t	t	a	a	t	a	t	a	t	a	t	a	t	t	t	g	a	-	g	g	c	c	t	t	t	a	a
KP771917	1	a	t	g	a	a	g	t	t	a	a	g	g	t	a	t	t	c	t	t	g	a	a	t	t	a	a	c	t	g	g	t	t	a	t	c	t	a	g	a	a	t	a	t	t	c	a	t	t	a	a	t	a	t	a	t	a	t	a	t	t	t	g	a	-	g	g	t	c	t	t	t	a	a
KP771918	1	a	t	g	a	a	g	t	t	a	a	a	g	t	a	t	t	c	t	t	g	a	a	t	t	a	a	c	t	g	g	t	t	a	t	c	t	a	g	a	a	t	a	t	t	c	a	t	t	a	a	t	a	t	a	t	a	t	a	t	t	t	g	a	-	g	g	t	c	t	t	t	a	a
KP771920	1	a	t	g	a	a	g	t	t	a	a	a	g	t	a	t	t	c	t	t	g	a	a	t	t	a	a	c	t	g	g	t	t	a	t	c	t	a	g	a	a	t	a	t	t	c	a	t	t	a	a	t	a	t	a	t	a	t	a	t	t	t	g	a	-	g	g	t	c	t	t	t	a	a
MH709361	1	a	t	g	a	a	g	t	t	a	a	a	g	t	a	t	t	c	t	t	g	a	a	t	t	a	a	c	t	g	g	t	t	a	t	c	t	a	g	a	a	t	a	t	t	c	a	t	t	a	a	t	a	t	a	t	a	t	a	t	t	t	g	a	-	g	g	c	c	t	t	t	a	a
KU926318	2	g	t	g	a	a	g	t	t	g	a	a	g	t	g	t	c	c	c	t	g	a	g	t	t	a	g	t	t	g	g	t	t	a	t	c	t	a	g	a	a	c	a	t	t	c	a	t	t	g	a	c	c	t	a	t	a	t	a	t	t	t	g	a	-	g	g	t	c	t	-	-	g	a
KU926319	2	a	t	g	a	a	g	t	t	a	a	a	g	t	a	t	t	c	c	t	g	a	a	t	t	a	a	t	t	g	g	t	t	a	t	c	t	a	g	a	a	c	a	t	t	c	a	t	t	a	a	c	c	t	a	t	a	t	a	t	t	t	g	a	-	g	g	t	c	t	-	-	a	a
KU926320	2	a	t	g	a	a	g	t	t	a	a	a	g	t	a	t	t	c	c	t	g	a	a	t	t	a	a	t	t	g	g	t	t	a	t	c	t	g	g	a	a	c	a	t	t	c	a	t	t	a	a	c	c	c	a	t	a	t	a	c	t	t	g	a	-	g	g	t	c	t	-	-	a	c
KU926321	2	a	t	g	a	a	g	t	t	a	a	a	g	t	a	t	t	c	c	t	g	a	a	t	t	a	a	t	t	g	g	t	t	a	t	c	t	a	g	a	a	c	a	t	t	c	a	t	t	a	a	c	c	t	a	t	a	t	a	t	t	t	g	a	-	g	g	t	c	t	-	-	a	a
KU926322	2	a	t	g	a	a	g	t	t	a	a	a	g	t	a	t	t	c	c	t	g	a	a	t	t	a	a	t	t	g	g	t	t	a	t	c	t	a	g	a	a	c	a	t	t	c	a	t	t	a	a	c	c	t	a	t	a	t	a	t	t	c	g	a	-	g	g	t	c	t	-	-	a	a
KC847290	2	a	t	g	a	a	g	t	t	a	a	a	g	t	a	t	t	c	c	t	g	a	a	t	t	a	a	t	t	g	g	t	t	a	t	c	t	a	g	a	a	c	a	t	t	c	a	t	t	a	a	c	c	t	a	t	a	t	a	t	t	t	g	a	-	g	g	t	c	t	-	-	a	a
KJ767491	2	a	t	g	a	a	g	t	t	a	a	a	g	t	a	t	t	c	c	t	g	a	a	t	t	a	g	t	t	g	g	t	t	a	t	c	t	a	g	a	a	c	a	t	t	c	a	t	t	a	a	c	c	t	a	t	a	t	a	t	t	t	g	a	-	g	g	t	c	t	-	-	a	a
KJ767492	2	a	t	g	a	a	g	t	t	a	a	a	g	t	a	t	t	c	c	t	g	a	a	t	t	a	a	t	t	g	g	t	t	a	t	c	t	a	g	a	a	c	a	t	t	c	a	t	t	a	a	c	c	t	a	t	a	t	a	t	t	t	g	a	-	g	g	t	c	t	-	-	a	a
KJ808816	2	a	t	g	a	a	g	t	t	a	a	a	g	t	a	t	t	c	c	t	g	a	a	t	t	a	a	t	t	g	g	t	t	a	t	c	t	a	g	a	a	c	a	t	t	c	a	t	t	a	a	c	c	t	a	t	a	t	a	t	t	t	g	a	-	g	g	t	c	t	-	-	a	a
KP771925	2	a	t	g	a	a	g	t	t	a	a	a	g	t	a	t	t	c	c	t	g	a	a	t	t	a	a	t	t	g	g	t	t	a	t	c	t	a	g	a	a	c	a	t	t	c	a	t	t	a	a	c	c	t	a	t	a	t	a	t	t	t	g	a	-	g	g	t	c	t	t	t	a	a
KU926315	2	a	t	g	a	a	g	t	t	a	a	a	g	t	a	t	t	c	c	t	g	a	a	t	t	a	g	t	t	g	g	t	t	a	t	c	t	a	g	a	a	c	a	t	t	c	a	t	t	a	a	c	c	t	a	t	a	t	a	t	t	t	g	a	-	g	g	t	c	t	-	-	a	a
KU926316	2	a	t	g	a	a	g	t	t	a	a	a	g	t	a	t	t	c	c	t	g	a	a	t	t	a	g	t	t	g	g	t	t	a	t	c	t	a	g	a	a	c	a	t	t	c	a	t	t	a	a	c	c	t	a	t	a	t	a	t	t	t	g	a	-	g	g	t	c	t	-	-	a	a
KU926317	2	a	t	g	a	a	g	t	t	g	a	a	g	t	g	t	t	c	c	t	g	a	a	t	t	a	g	t	t	g	g	t	t	a	t	c	t	a	g	a	a	c	a	t	t	c	a	t	t	a	a	c	c	t	a	t	a	t	a	t	t	t	g	a	-	g	g	t	c	t	-	-	a	a
KY037796	2	a	t	g	a	a	g	t	t	a	a	a	g	t	a	t	t	c	c	t	g	a	a	t	t	a	a	t	t	g	g	t	t	a	t	c	t	a	g	a	a	c	a	t	t	c	a	t	t	a	a	c	c	t	a	t	a	t	a	t	t	t	g	a	-	g	g	t	c	t	-	-	a	a
MF004348	2	a	t	g	a	a	g	t	t	a	a	a	g	t	a	t	t	c	c	t	g	a	a	t	t	a	a	t	t	g	g	t	t	a	t	c	t	a	g	a	a	c	a	t	t	c	a	t	t	a	a	c	c	t	a	t	a	t	a	t	t	t	g	a	-	g	g	t	c	t	-	-	a	a
MH709309	2	a	t	g	a	a	g	t	t	a	a	a	g	t	a	t	t	c	c	t	g	a	a	t	t	a	a	t	t	g	g	t	t	a	t	c	t	a	g	a	a	c	a	t	t	c	a	t	t	a	a	c	c	t	a	t	a	t	a	t	t	t	g	a	-	g	g	t	c	c	t	t	a	a
MH709310	2	a	t	g	a	a	g	t	t	a	a	a	g	t	a	t	t	c	c	t	g	a	a	t	t	a	a	t	t	g	g	t	t	a	t	c	t	a	g	a	a	c	a	t	t	c	a	t	t	a	a	c	c	t	a	t	a	t	a	t	t	t	g	a	-	g	g	t	c	t	a	c	a	a
MH709311	2	a	t	a	a	a	g	t	t	a	a	a	g	t	a	t	t	c	c	t	g	a	a	t	t	a	a	t	t	g	g	t	t	a	t	c	t	a	g	a	a	c	a	t	t	c	a	t	t	a	a	c	c	t	a	t	a	t	a	t	t	t	g	a	-	g	g	t	c	t	-	-	a	a
MH709313	2	a	t	g	a	a	g	t	t	a	a	a	g	t	a	t	t	c	c	t	g	a	a	t	t	a	a	t	t	g	g	t	t	a	t	c	t	a	g	a	a	c	a	t	t	c	a	t	t	a	a	c	c	t	a	t	a	t	a	t	t	t	g	a	-	g	g	t	c	c	t	t	a	a
MH709314	2	a	t	g	a	a	g	t	t	a	a	a	g	t	a	t	t	c	c	t	g	a	a	t	t	a	a	t	t	g	g	t	t	a	t	c	t	a	g	a	a	c	a	t	t	c	a	t	t	a	a	c	c	t	a	t	a	t	a	t	t	t	g	a	-	g	g	t	c	t	t	t	a	a
MH709315	2	a	t	g	a	a	g	t	t	a	a	a	g	t	a	t	t	c	c	t	g	a	a	t	t	a	a	t	t	g	g	t	t	a	t	c	t	a	g	a	a	c	a	t	t	c	a	t	t	a	a	c	c	t	a	t	a	t	a	t	t	t	g	a	-	g	g	t	c	t	a	c	a	a
MH709319	2	a	t	g	a	a	g	t	t	a	a	a	g	t	a	t	t	c	c	t	g	a	a	t	t	a	a	t	t	g	g	t	t	a	t	c	t	a	g	a	a	c	a	t	t	c	a	t	t	a	a	c	c	t	a	t	a	t	a	t	t	t	g	a	-	g	g	t	c	t	a	c	a	a
MH709320	2	a	t	g	a	a	g	t	t	a	a	a	g	t	a	t	t	c	c	t	g	a	a	t	t	a	a	t	t	g	g	t	t	a	t	c	t	a	g	a	a	c	a	t	t	c	a	t	t	a	a	c	c	t	a	t	a	t	a	t	t	t	g	a	-	g	g	t	c	t	a	t	a	a
MH709329	2	a	t	g	a	a	g	t	t	a	a	a	g	t	a	t	t	c	c	t	g	a	a	t	t	a	a	t	t	g	g	t	t	a	t	c	t	a	g	a	a	c	a	t	t	c	a	t	t	a	a	c	c	t	a	t	a	t	a	t	t	t	g	a	-	g	g	t	c	t	a	t	a	a
MH709331	2	a	t	g	a	a	g	t	t	a	a	a	g	t	a	t	t	c	c	t	g	a	a	t	t	a	a	t	t	g	g	t	t	a	t	c	t	a	g	a	a	c	a	t	t	c	a	t	t	a	a	c	c	t	a	t	a	t	a	t	t	t	g	a	-	g	g	t	c	t	a	t	a	a
MH709333	2	a	t	g	a	a	g	t	t	a	a	a	g	t	a	t	t	c	c	t	g	a	a	t	t	a	a	t	t	g	g	t	t	a	t	c	t	a	g	a	a	c	a	t	t	c	a	t	t	a	a	c	c	t	a	t	a	t	a	t	t	t	g	a	-	g	g	t	c	t	a	c	a	a
MH709337	2	a	t	g	a	a	g	t	t	a	a	a	g	t	a	t	t	c	c	t	g	a	a	t	t	a	a	t	t	g	g	t	t	a	t	c	t	a	g	a	a	c	a	t	t	c	a	t	t	a	a	c	c	t	a	t	a	t	a	t	t	t	g	a	-	g	g	t	c	t	a	c	a	a
MH709339	2	a	t	g	a	a	g	t	t	a	a	a	g	t	a	t	t	c	c	t	g	a	a	t	t	a	a	t	t	g	g	t	t	a	t	c	t	a	g	a	a	c	a	t	t	c	a	t	t	a	a	c	c	t	a	t	a	t	a	t	t	t	g	a	-	g	g	t	c	t	t	t	a	a
MH709344	2	a	t	g	a	a	g	t	t	a	a	a	g	t	a	t	t	c	c	t	g	a	a	t	t	a	a	t	t	g	g	t	t	a	t	c	t	a	g	a	a	c	a	t	t	c	a	t	t	a	a	c	c	t	a	t	c	t	a	t	t	t	g	a	-	g	g	t	c	c	t	t	a	a
MH709348	2	a	t	g	a	a	g	t	t	a	a	a	g	t	a	t	t	c	c	t	g	a	a	t	t	a	a	t	t	g	g	t	t	a	t	c	t	a	g	a	a	c	a	t	t	c	a	t	t	a	a	c	c	t	a	t	a	t	a	t	t	t	g	a	-	g	g	t	c	t	a	c	a	a
MH709355	2	a	t	g	a	a	g	t	t	a	a	a	g	t	a	t	t	c	c	t	g	a	a	t	t	a	a	t	t	g	g	t	t	a	t	c	c	a	g	a	a	c	a	t	t	c	a	t	t	a	a	c	c	t	a	t	a	t	a	t	t	t	g	g	-	g	g	t	c	t	a	c	a	a
MH709359	2	a	t	g	a	a	g	t	t	a	a	a	g	t	a	t	t	c	c	t	g	a	a	t	t	a	a	t	t	g	g	t	t	a	t	c	t	a	g	a	a	c	a	t	t	c	a	t	t	a	a	c	c	t	a	t	a	t	a	t	t	t	g	a	-	g	g	t	c	t	t	t	a	a
MH709363	2	a	t	g	a	a	g	t	t	a	a	a	g	t	a	t	t	c	c	t	g	a	a	t	t	a	a	t	t	g	g	t	t	a	t	c	t	a	g	a	a	c	a	t	t	c	a	t	t	a	a	c	c	t	a	t	a	t	a	t	t	t	g	a	-	g	g	t	c	t	a	c	a	a
MH709366	2	a	t	g	a	a	g	t	t	a	a	a	g	t	a	t	t	c	c	t	g	a	a	t	t	a	a	t	t	g	g	t	t	a	t	c	t	a	g	a	a	c	a	t	t	c	a	t	t	a	a	c	c	t	a	t	a	t	a	t	t	t	g	a	-	g	g	t	c	c	t	t	a	a
MH709376	2	a	t	g	a	a	g	t	t	a	a	a	g	t	a	t	t	c	c	t	g	a	a	t	t	a	a	t	t	g	g	t	t	a	t	c	t	a	g	a	a	c	a	t	t	c	t	t	t	a	a	c	c	t	a	t	a	t	a	t	t	t	g	a	-	g	g	t	c	t	a	c	a	a
MH709377	2	a	t	g	a	a	g	t	t	a	a	a	g	t	a	t	t	c	c	t	g	a	a	t	t	a	a	t	t	g	g	t	t	a	t	c	t	a	g	a	a	c	a	t	t	c	a	t	t	a	a	c	c	t	a	t	a	t	a	t	t	t	g	a	-	g	g	t	c	t	a	c	a	a
**MW316406**	2	a	t	g	a	a	g	t	t	a	a	a	g	t	a	t	t	c	c	t	g	a	a	t	t	a	a	t	t	g	g	t	t	a	t	c	t	a	g	a	a	c	a	t	t	c	a	t	t	a	a	c	c	t	a	t	a	t	a	t	t	t	g	a	-	g	g	t	c	t	-	-	a	a
AJ871403	3	a	t	g	a	a	g	t	t	a	a	a	g	t	a	t	t	c	t	t	g	a	a	t	t	a	a	c	t	g	g	t	t	a	t	c	t	a	g	a	a	t	a	t	t	c	a	t	t	a	a	t	a	t	a	t	a	t	a	t	t	t	g	a	-	g	g	t	c	t	t	t	a	a
DQ479955	3	a	t	g	a	a	g	t	t	a	a	a	g	t	a	t	t	c	t	t	g	a	a	t	t	a	a	c	t	g	g	t	t	a	t	c	t	a	g	a	a	t	a	t	t	c	a	t	t	a	a	t	a	t	a	t	a	t	a	t	t	t	g	a	-	g	g	t	c	t	t	t	a	a
DQ479956	3	a	t	g	a	a	g	t	t	a	a	a	g	t	a	t	t	c	t	t	g	a	a	t	t	a	a	c	t	g	g	t	t	a	t	c	t	a	g	a	a	t	a	t	t	c	a	t	t	a	a	t	a	t	a	t	a	t	a	t	t	t	g	a	-	g	g	t	c	t	t	t	a	a
DQ479957	3	a	t	g	a	a	g	t	t	a	a	a	g	t	a	t	t	c	t	t	g	a	a	t	t	a	a	c	t	g	g	t	t	a	t	c	t	a	g	a	a	t	a	t	t	c	a	t	t	a	a	t	a	t	a	t	a	t	a	t	t	t	g	a	-	g	g	t	c	t	t	t	a	a
JN704699	3	a	t	g	a	a	g	t	t	a	a	a	g	t	a	t	t	c	t	t	g	a	a	t	t	a	a	c	t	g	g	t	t	a	t	c	t	a	g	a	a	t	a	t	t	c	a	t	t	a	a	t	a	t	a	t	a	t	a	t	t	t	g	a	-	g	g	t	c	t	t	t	a	a
JN704700	3	a	t	g	a	a	g	t	t	a	a	a	g	t	a	t	t	c	t	t	g	a	a	t	t	a	a	c	t	g	g	t	t	a	t	c	t	a	g	a	a	t	a	t	t	c	a	t	t	a	a	t	a	t	a	t	a	t	a	t	t	t	g	a	-	g	g	t	c	t	t	t	a	a
JN704701	3	a	t	g	a	a	g	t	t	a	a	a	g	t	a	t	t	c	t	t	g	a	a	t	t	a	a	c	t	g	g	t	t	a	t	c	t	a	g	a	a	t	a	t	t	c	a	t	t	a	a	t	a	t	a	t	a	t	a	t	t	t	g	a	-	g	g	t	c	t	t	t	a	a
JN704702	3	a	t	g	a	a	g	t	t	a	a	a	g	t	a	t	t	c	t	t	g	a	a	t	t	a	a	c	t	g	g	t	t	a	t	c	t	a	g	a	a	t	a	t	t	c	a	t	t	a	a	t	a	t	a	t	a	t	a	t	t	t	g	a	-	g	g	t	c	t	t	t	a	a
JN704703	3	a	t	g	a	a	g	t	t	a	a	a	g	t	a	t	t	c	t	t	g	a	a	t	t	a	a	c	t	g	g	t	t	a	t	c	t	a	g	a	a	t	a	t	t	c	a	t	t	a	a	t	a	t	a	t	a	t	a	t	t	t	g	a	-	g	g	t	c	t	t	t	a	a
KP771891	3	a	t	g	a	a	g	t	t	a	a	a	g	t	a	t	t	c	t	t	g	a	a	t	t	a	a	c	t	g	g	t	t	a	t	c	t	a	g	a	a	t	a	t	t	c	a	t	t	a	a	t	a	t	a	t	a	t	a	t	t	t	g	a	-	g	g	t	c	t	t	t	a	a
KP771893	3	a	t	g	a	a	g	t	t	a	a	a	g	t	a	t	t	c	t	c	g	a	a	t	t	a	a	c	t	g	g	t	t	a	t	c	t	a	g	a	a	t	a	t	t	c	a	t	t	a	a	t	a	t	a	t	a	t	a	t	t	t	g	a	-	g	g	t	c	t	t	t	a	a
KP771895	3	a	t	g	a	a	g	t	t	a	a	a	g	t	a	t	t	c	t	t	g	a	a	t	t	a	a	c	t	g	g	t	t	a	t	c	t	a	g	a	a	t	a	t	t	c	a	t	t	a	a	t	a	t	a	t	a	t	a	t	t	t	g	a	-	g	g	t	c	t	t	t	a	a
KP771896	3	a	t	g	a	a	g	t	t	a	a	a	g	t	a	t	t	c	t	t	g	a	a	t	t	a	a	c	t	g	g	t	t	a	t	c	t	a	g	a	a	t	a	t	t	c	a	t	t	a	a	t	a	t	a	t	a	t	a	t	t	t	g	a	-	g	c	t	c	t	t	t	a	a
KP771897	3	a	t	g	a	a	g	t	t	a	a	a	g	t	a	t	t	c	t	t	g	a	a	t	t	a	a	c	t	g	g	t	t	a	t	c	t	a	g	a	a	t	a	t	t	c	a	t	t	a	a	t	a	t	a	t	a	t	a	t	t	t	g	a	-	g	g	t	c	t	t	t	a	a
KP771899	3	a	t	g	a	a	g	t	t	a	a	a	g	t	a	t	t	c	t	t	g	a	a	t	t	a	a	c	t	g	g	t	t	a	t	c	t	a	g	a	a	t	a	c	t	c	a	t	t	a	a	t	a	t	a	t	a	t	a	t	t	t	g	a	-	g	g	t	c	t	t	t	a	a
KP771903	3	a	t	g	a	a	g	t	t	a	a	a	g	t	a	t	t	c	t	t	g	a	a	t	t	a	a	c	t	g	g	t	t	a	t	c	t	a	g	a	a	t	a	t	t	c	a	c	t	a	a	t	a	t	a	t	a	t	a	t	t	t	g	a	-	g	g	t	c	t	t	t	a	a
KP771907	3	a	t	g	a	a	g	t	t	a	a	a	g	t	a	t	t	c	t	t	g	a	a	t	t	a	a	c	t	g	g	t	t	a	t	c	t	a	g	a	a	t	a	t	t	c	a	t	g	a	a	t	a	t	a	t	a	t	a	t	t	t	g	a	-	g	g	t	c	t	t	t	a	a
KP771910	3	a	t	g	a	a	g	t	t	a	a	a	g	t	a	t	t	c	t	t	g	a	a	t	t	a	a	c	t	g	g	t	t	a	t	c	t	a	g	a	a	t	a	t	t	c	a	t	t	a	a	t	a	t	a	t	a	t	a	t	t	t	g	a	-	g	g	t	c	t	t	t	a	a
KP771915	3	a	t	g	a	a	g	t	t	a	a	a	g	t	a	t	t	c	t	t	g	a	a	t	t	a	a	c	t	g	g	t	t	a	t	c	t	a	g	a	a	t	a	t	t	c	a	t	t	a	a	t	a	t	a	t	a	t	a	t	t	t	g	a	-	g	g	t	c	t	t	t	a	a
KP771916	3	a	t	g	a	a	g	t	t	a	a	a	g	t	a	t	t	c	t	t	g	a	a	t	t	a	a	c	t	g	g	t	t	a	t	c	t	a	g	a	a	t	a	t	t	c	a	t	t	a	a	t	a	t	a	t	a	t	a	t	t	t	g	a	-	g	g	t	c	t	t	t	a	a
KP771921	3	a	t	g	a	a	g	t	t	a	a	a	g	t	a	t	t	c	t	t	g	a	a	t	t	a	a	c	t	g	g	t	t	a	t	c	t	a	g	a	a	t	a	t	t	c	a	t	t	a	a	t	a	t	a	t	a	t	a	t	t	t	g	a	-	g	g	t	c	t	t	t	a	a
KP771923	3	a	t	g	a	a	g	t	t	a	a	a	g	t	a	t	t	c	t	t	g	a	a	t	t	a	a	c	t	g	g	t	t	a	t	c	t	a	g	a	a	t	a	t	t	c	a	t	t	a	a	t	a	t	a	t	a	t	a	t	t	t	g	a	-	g	g	t	c	t	t	t	a	a
KP771889	3	a	t	g	a	a	g	t	t	a	a	a	g	t	a	t	t	c	t	t	g	a	a	t	t	a	a	c	t	g	g	t	t	a	t	c	t	a	g	a	a	t	a	t	t	c	a	t	t	a	a	t	a	t	g	t	a	t	a	t	t	t	g	a	-	g	g	t	c	t	t	t	a	a
KU926311	3	a	t	g	a	a	g	t	t	a	a	a	g	t	a	t	t	c	t	t	g	a	a	t	t	a	a	c	t	g	g	t	t	a	t	c	t	a	g	a	a	t	a	t	t	c	a	t	t	a	a	t	a	t	a	t	a	t	a	t	t	t	g	a	-	g	g	t	c	t	t	t	a	a
JQ911599	3	a	t	g	a	a	g	t	t	a	a	a	g	t	a	t	t	c	t	t	g	a	a	t	t	a	a	c	c	g	g	t	t	a	t	c	t	a	g	a	a	t	a	t	t	c	a	t	t	a	a	t	a	t	a	t	a	t	a	t	t	t	g	a	-	g	g	t	c	t	t	t	a	a
MH709317	3	a	t	g	a	a	g	t	t	a	a	a	g	t	a	t	t	c	t	t	g	a	a	t	t	a	a	c	t	g	g	t	t	a	t	c	t	a	g	a	a	t	a	t	t	c	a	t	t	a	a	t	a	t	a	t	a	t	a	t	t	t	g	a	-	g	g	t	c	t	t	t	a	a
MH709346	3	a	t	g	a	g	g	t	t	a	a	a	g	t	a	t	t	c	t	t	g	a	a	t	t	a	a	c	t	g	g	t	t	a	t	c	t	a	g	a	a	t	a	t	t	c	a	t	t	a	a	t	a	t	a	t	a	t	a	t	t	t	g	a	-	g	g	t	c	t	t	t	a	a
MH709375	3	a	t	g	a	a	g	t	t	a	a	a	g	t	a	t	t	c	t	t	g	a	a	t	t	a	a	c	t	g	g	t	t	a	t	c	t	a	g	a	a	t	a	t	t	c	a	t	t	a	a	t	a	t	a	t	a	t	a	t	t	t	g	a	-	g	g	t	c	t	t	t	a	a
DQ452050	4	a	t	g	a	a	g	t	t	a	a	a	g	t	a	t	t	c	c	t	g	a	a	t	t	a	a	c	t	g	a	t	t	a	t	c	t	a	g	a	a	c	a	t	t	t	a	t	t	a	a	t	a	t	a	c	a	t	a	t	t	t	a	a	-	g	g	t	c	t	t	t	a	a
DQ479960	4	a	t	g	a	a	g	t	t	a	a	a	g	t	a	t	t	c	c	t	g	a	a	t	t	a	a	c	t	g	a	t	t	a	t	c	t	a	g	a	a	c	a	t	t	t	a	t	t	a	a	t	a	t	a	c	a	t	a	t	t	t	a	a	-	g	g	t	c	t	t	t	a	a
KP771898	4	a	t	g	a	a	g	t	t	a	a	a	g	t	a	t	t	c	c	t	g	a	a	t	t	a	a	c	t	g	a	t	t	a	t	c	t	a	g	a	a	c	a	t	t	t	a	t	t	a	a	t	a	t	a	c	a	t	a	t	t	t	a	a	-	g	g	t	c	t	t	t	a	a
MH709316	4	a	t	g	a	a	g	t	t	a	a	a	g	t	a	t	t	c	c	t	g	a	a	t	t	a	a	c	t	g	a	t	t	a	t	c	t	a	g	a	a	c	a	t	c	t	a	t	t	a	a	t	a	t	a	c	a	t	a	t	t	t	a	a	-	g	g	t	c	t	t	t	a	a
MH709318	4	a	t	g	a	a	g	t	t	a	a	a	g	t	a	t	t	c	c	t	g	a	a	t	t	a	a	c	t	g	a	t	t	a	t	c	t	a	g	a	a	c	a	t	t	t	a	t	t	a	a	t	a	t	a	c	a	t	a	t	t	t	a	a	-	g	g	t	c	t	t	t	a	a
MH709322	4	a	t	g	a	a	g	t	t	a	a	a	g	t	a	t	t	c	c	t	g	g	a	t	t	a	a	c	t	g	a	t	t	a	t	c	t	a	g	a	a	c	a	t	t	t	a	t	t	a	a	t	a	t	a	c	a	t	a	t	t	t	a	a	-	g	g	t	c	t	t	t	a	a
MH709323	4	a	t	g	a	a	g	t	t	a	a	a	g	t	a	t	t	c	c	t	g	g	a	t	t	a	a	c	t	g	a	t	t	a	t	c	t	a	g	a	a	c	a	t	t	t	a	t	t	a	a	t	a	t	a	c	a	t	a	t	t	t	a	a	-	g	g	t	c	t	t	t	a	a
MH709326	4	a	t	g	a	a	g	t	t	a	a	a	g	t	a	t	t	c	c	t	g	a	a	t	t	a	a	c	t	g	a	t	t	a	t	c	t	a	g	a	a	c	a	t	t	t	a	t	t	a	a	t	a	t	a	c	a	t	a	t	t	t	a	a	-	g	g	t	c	t	t	t	a	a
MH709341	4	a	t	g	a	a	g	t	t	a	a	a	g	t	a	t	t	c	c	t	g	g	a	t	t	a	a	c	t	g	a	t	t	a	t	c	t	a	g	a	a	c	a	t	t	t	a	t	t	a	a	t	a	t	a	c	a	t	a	t	t	t	a	a	-	g	g	t	c	t	t	t	a	a
MH709357	4	a	t	g	a	a	g	t	t	a	a	a	g	t	a	t	t	c	c	t	g	a	a	t	t	a	a	c	t	g	a	t	t	a	t	c	t	a	g	a	a	c	a	t	t	t	a	t	t	a	a	t	a	t	a	c	a	t	a	t	t	t	a	a	-	g	g	t	c	t	t	t	a	a
MH709364	4	a	t	g	a	a	g	t	t	a	a	a	g	t	a	t	t	c	c	t	g	a	a	t	t	a	a	c	t	g	a	t	t	a	t	c	t	a	g	a	a	c	a	t	t	t	a	t	t	a	a	t	a	t	a	c	a	t	a	t	t	t	a	a	-	g	g	t	c	t	t	t	a	a
MH709370	4	a	t	g	a	a	g	t	t	a	a	a	g	t	a	t	t	c	c	t	g	a	a	t	t	a	a	c	t	g	a	t	t	a	t	c	t	a	g	a	a	c	a	t	t	t	a	t	t	a	a	t	a	t	a	c	a	t	a	t	t	t	a	a	-	g	g	t	c	t	t	t	a	a
MH709371	4	a	t	g	a	a	g	t	t	a	a	a	g	t	a	t	t	c	c	t	g	a	a	t	t	a	a	c	t	g	a	t	t	a	t	c	t	a	g	a	a	c	a	t	t	t	a	t	t	a	a	t	a	t	a	c	a	t	a	t	t	t	a	a	-	g	g	t	c	t	t	t	a	a
MH709372	4	a	t	g	a	a	g	t	t	a	a	a	g	t	a	t	t	c	c	t	g	a	a	t	t	a	a	c	t	g	a	t	t	a	t	c	t	a	g	a	a	c	a	t	t	t	a	t	t	a	a	t	a	t	a	c	a	t	a	t	t	t	a	a	-	g	g	t	c	t	t	t	a	a
DQ457052	5	a	t	g	g	a	g	t	t	a	a	a	g	t	a	t	t	c	t	t	g	a	a	t	t	a	a	c	t	g	g	t	t	g	t	c	t	a	g	a	a	t	a	t	t	c	a	t	t	a	a	t	a	t	a	c	a	t	a	t	t	t	g	a	-	g	g	t	c	t	t	t	a	a
JN704704	5	a	t	g	g	a	g	t	t	a	a	a	g	t	a	t	t	c	t	t	g	a	a	t	t	a	a	c	t	g	g	t	t	g	t	c	t	a	g	a	a	t	a	t	t	c	a	t	t	a	c	t	a	t	a	c	a	t	a	t	t	t	g	a	-	g	g	t	c	t	t	t	a	a
JN704705	5	a	t	g	a	a	g	t	t	a	a	a	g	t	a	t	t	c	t	t	g	a	a	t	t	a	a	c	t	g	g	t	t	g	t	c	t	a	g	a	a	t	a	t	t	c	a	t	t	a	a	t	a	t	a	c	a	t	a	t	t	t	g	a	-	g	g	t	c	t	t	t	a	a
JN704706	5	a	t	g	a	a	g	t	t	a	a	a	g	t	a	t	t	c	t	t	g	a	a	t	t	a	a	c	t	g	g	t	t	g	t	c	t	a	g	a	a	t	a	t	t	c	a	t	t	a	a	t	a	t	a	c	a	t	a	t	t	t	g	a	-	g	g	t	c	t	t	t	a	a
JN704707	5	a	t	g	g	a	g	t	t	a	a	a	g	t	a	t	t	c	t	t	g	a	a	t	t	a	a	c	t	g	g	t	t	g	t	c	t	a	a	a	a	t	a	t	t	c	a	t	t	a	a	t	a	t	a	c	a	t	a	t	t	t	g	a	-	g	g	t	c	t	-	-	a	a
JN704708	5	a	t	g	a	a	g	t	t	a	a	a	g	t	a	t	t	c	t	t	g	a	a	t	t	a	a	c	t	g	g	t	t	g	t	c	t	a	g	a	a	t	a	t	t	c	a	t	t	a	a	t	a	t	a	c	a	t	a	t	t	t	g	a	-	g	g	t	c	t	t	t	a	a
KP771904	5	a	t	g	g	a	g	t	t	a	a	a	g	t	a	t	t	c	t	t	g	a	a	t	t	a	a	c	t	g	g	t	t	g	t	c	t	a	g	a	a	t	a	t	t	c	a	t	t	a	a	t	a	t	a	c	a	t	a	t	t	t	g	a	-	g	g	t	c	t	t	t	a	a
KP771905	5	a	t	g	g	a	g	t	t	a	a	a	g	c	a	t	t	c	t	t	g	a	a	t	t	a	a	c	t	g	g	t	t	g	t	c	t	a	g	a	a	t	a	t	t	c	a	t	t	a	a	t	a	t	a	c	a	t	a	t	t	t	g	a	-	g	g	t	c	t	t	t	a	a
KP771906	5	a	t	g	g	a	g	t	t	a	a	a	g	c	a	t	t	c	t	t	g	a	a	t	t	a	a	c	t	g	g	t	t	g	t	c	t	a	g	a	a	t	a	t	t	c	a	t	t	a	a	t	a	t	a	c	a	t	a	t	t	t	g	a	-	g	g	t	c	t	t	t	a	a
KP771919	5	a	t	g	g	a	g	t	t	a	a	a	g	t	a	t	t	c	t	t	g	a	a	t	t	a	a	c	t	g	g	t	t	g	t	c	t	a	g	a	a	t	a	t	t	c	a	t	t	a	a	t	a	t	a	c	a	t	a	t	t	t	g	a	-	g	g	t	c	t	t	t	a	a
KP771922	5	a	t	g	g	a	g	t	t	a	a	a	g	t	a	t	c	c	t	t	g	a	a	t	t	a	a	c	t	g	g	t	t	g	t	c	t	a	g	a	a	t	a	t	t	c	a	t	t	a	a	t	a	t	a	c	a	t	a	t	t	t	g	a	-	g	g	t	c	t	t	t	a	a
KP771924	5	a	t	g	a	a	g	t	t	a	a	a	g	t	a	t	t	c	t	t	g	a	a	t	t	a	a	c	t	g	g	t	t	g	t	c	t	a	g	a	a	t	a	t	t	c	a	t	t	a	a	t	a	t	a	c	a	t	a	t	t	t	g	a	-	g	g	t	c	t	t	t	a	a
KM355696	5	a	t	g	a	a	g	t	t	a	a	a	g	t	a	t	t	c	t	t	g	a	a	t	t	a	a	c	t	g	g	t	t	g	t	c	t	a	g	a	a	t	a	t	t	c	a	t	t	a	a	t	a	t	a	c	a	t	a	t	t	t	g	a	-	g	g	t	c	t	t	t	a	a
KM355697	5	a	t	g	a	a	g	t	t	a	a	a	g	t	a	t	t	c	t	t	g	a	a	t	t	a	a	c	t	g	g	t	t	g	t	c	t	a	g	a	a	t	a	t	t	c	a	t	t	a	a	t	a	t	a	c	a	t	a	t	t	t	g	a	-	g	g	t	c	t	t	t	a	a
KM355698	5	a	t	g	a	a	g	t	t	a	a	a	g	t	a	t	t	c	t	t	g	a	a	t	t	a	a	c	t	g	g	t	t	g	t	c	t	a	g	a	a	t	a	t	t	c	a	t	t	a	a	t	a	t	a	c	a	t	a	t	t	t	g	a	-	g	g	t	c	t	t	t	a	a
KM355699	5	a	t	g	a	a	g	t	t	a	a	a	g	t	a	t	t	c	t	t	g	a	a	t	t	a	a	c	t	g	g	t	t	g	t	c	t	a	g	a	a	t	a	t	t	c	a	t	t	a	a	t	a	t	a	c	a	t	a	t	t	t	g	a	-	g	g	t	c	t	t	t	a	a
KM355701	5	a	t	g	a	a	g	t	t	a	a	a	t	t	a	t	t	c	t	t	g	a	a	t	t	a	a	c	t	g	g	t	t	g	t	c	t	a	g	a	a	t	a	t	t	c	a	t	t	a	a	t	a	t	a	c	a	t	a	t	t	t	g	a	-	g	g	t	c	t	t	t	a	a
KM355702	5	a	t	g	a	a	g	t	t	a	a	a	g	t	a	t	t	c	t	t	g	a	a	t	t	a	a	c	t	g	g	t	t	g	t	c	t	a	g	a	a	t	a	t	t	c	a	t	t	a	a	t	a	t	a	c	a	t	a	t	t	t	g	a	-	g	g	t	c	t	t	t	a	a
KM355703	5	a	t	g	a	a	g	t	t	a	a	a	g	t	a	t	t	c	t	t	g	a	a	t	t	a	a	c	t	g	g	t	t	g	t	c	t	a	g	a	a	t	a	t	t	c	a	t	t	a	a	t	a	t	a	c	a	t	a	t	t	t	g	a	-	g	g	t	c	t	t	t	a	a
KM355704	5	a	t	g	a	a	g	t	t	a	a	a	g	t	a	t	t	c	t	t	g	a	a	t	t	a	a	c	t	g	g	t	t	g	t	c	t	a	g	a	a	t	a	t	t	c	a	t	t	a	a	t	a	t	a	c	a	t	a	t	t	t	g	a	-	g	g	t	c	t	t	t	a	a
KM355705	5	a	t	g	a	a	g	t	t	a	a	a	g	t	a	t	t	c	t	t	g	a	a	t	t	a	a	c	t	g	g	t	t	g	t	c	t	a	g	a	a	t	a	t	t	c	a	t	t	a	a	t	a	t	a	c	a	t	a	t	t	t	g	a	-	g	g	t	c	t	t	t	a	a
KM355706	5	a	t	g	a	a	g	t	t	a	a	a	g	t	a	t	t	c	t	t	g	a	a	t	t	a	a	c	t	g	g	t	t	g	t	c	t	a	g	a	a	t	a	t	t	c	a	t	t	a	a	t	a	t	a	c	a	t	a	t	t	t	g	a	-	g	g	t	c	t	t	t	a	a
KM355707	5	a	t	g	a	a	g	t	t	a	a	a	g	t	a	t	t	c	t	t	g	a	a	t	t	a	a	c	t	g	g	t	t	g	t	c	t	a	g	a	a	t	a	t	t	c	a	t	t	a	a	t	a	t	a	c	a	t	a	t	t	t	g	a	-	g	g	t	c	t	t	t	a	a
KM355708	5	a	t	g	a	a	g	t	t	a	a	a	g	t	a	t	t	c	t	t	g	a	a	t	t	a	a	c	t	g	g	t	t	g	t	c	t	a	g	a	a	t	a	t	t	c	a	t	t	a	a	t	a	t	a	c	a	t	a	t	t	t	g	a	-	g	g	t	c	t	t	t	a	a
KM355709	5	a	t	g	a	a	g	t	t	a	a	a	g	t	a	t	t	c	t	t	g	a	a	t	t	a	a	c	t	g	g	t	t	g	t	c	t	a	g	a	a	t	a	t	t	c	a	t	t	a	a	t	a	t	a	c	a	t	a	t	t	t	g	a	-	g	g	t	c	t	t	t	a	a
KM355711	5	a	t	g	a	a	g	t	t	a	a	a	g	t	a	t	t	c	t	t	g	a	a	t	t	a	a	c	t	g	g	t	t	g	t	c	t	a	g	a	a	t	a	t	t	c	a	t	t	a	a	t	a	t	a	c	a	t	a	t	t	t	g	a	-	g	g	t	c	t	t	t	a	a
KM355712	5	a	t	g	a	a	g	t	t	a	a	a	g	t	a	t	t	c	t	t	g	a	a	t	t	a	a	c	t	g	g	t	t	g	t	c	t	a	g	a	a	t	a	t	t	c	a	t	t	a	a	t	a	t	a	c	a	t	a	t	t	t	g	a	-	g	g	t	c	t	t	t	a	a
KY037797	5	a	t	g	a	a	g	t	t	a	a	a	g	t	a	t	t	c	t	t	g	a	a	t	t	a	a	c	t	g	g	t	t	g	t	c	t	a	g	a	a	t	a	t	t	c	a	t	t	a	a	t	a	t	a	c	a	t	a	t	t	t	g	a	-	g	g	t	c	t	t	t	a	a

Supplementary Table 3: 2880 SN

GB-ID	Clade markers																								
	Position in Dumas	96444	96491	96499	96553	96607	96615	96629	96724	96739	96791	96904	96920	96937	97034	97066	97081	97093	97141	97162	97222	97276	97399	97444	97479
MH499467	5	a	t	g	g	a	g	t	t	a	a	a	g	t	a	t	t	c	t	t	g	a	a	t	t
MH499469	5	a	t	g	g	a	g	t	t	a	a	a	g	t	a	t	t	c	t	t	g	a	a	t	t
MH709321	5	a	t	g	a	a	g	t	t	a	g	a	g	t	a	t	t	c	t	t	g	a	a	g	t
MH709324	5	a	g	g	g	a	g	t	t	a	a	a	g	t	a	t	t	c	t	t	g	a	a	t	t
MH709325	5	a	t	g	a	a	g	t	t	a	a	a	g	t	a	t	t	c	t	t	g	a	a	t	t
MH709327	5	a	t	g	g	a	g	t	t	a	a	a	g	t	a	t	t	c	t	t	g	a	a	t	t
MH709328	5	a	t	g	g	a	g	t	t	a	a	a	g	t	a	t	t	c	t	t	g	a	a	t	t
MH709330	5	a	t	g	g	a	g	t	t	a	a	a	g	t	a	t	t	c	t	t	g	a	a	t	t
MH709332	5	a	t	g	g	a	g	t	t	a	a	a	g	t	a	t	t	c	t	t	g	a	a	t	t
MH709334	5	a	t	g	g	a	g	t	t	a	a	a	g	t	a	t	t	c	t	t	g	a	a	t	t
MH709335	5	a	t	g	g	a	g	t	t	a	a	a	g	t	a	t	t	c	t	t	g	a	a	t	t
MH709336	5	a	t	g	g	a	g	t	t	a	a	a	g	t	a	t	t	c	t	t	g	a	a	t	t
MH709338	5	a	t	g	g	a	g	t	t	a	a	a	g	t	a	t	t	c	t	t	g	a	a	t	t
MH709340	5	a	t	g	a	a	g	c	t	a	a	a	g	t	a	t	t	c	t	t	g	a	a	t	t
MH709342	5	a	t	g	a	a	g	t	t	a	a	a	g	t	a	t	t	c	t	t	g	a	a	t	t
MH709343	5	a	t	g	g	a	g	t	t	a	a	a	g	t	a	t	t	c	t	t	g	a	a	t	t
MH709345	5	a	t	g	g	a	g	t	t	a	a	a	g	t	a	t	t	c	t	t	g	a	a	t	t
MH709347	5	a	t	g	g	a	g	t	t	a	a	a	g	t	a	t	t	c	t	t	g	a	a	t	t
MH709349	5	a	t	g	g	a	g	t	t	a	a	a	g	t	a	t	t	c	t	t	g	a	a	t	t
MH709350	5	a	t	g	g	a	g	t	t	a	a	a	g	t	a	t	t	c	t	t	g	a	a	t	t
MH709351	5	a	t	g	a	a	g	c	t	a	a	a	g	t	a	t	t	c	t	t	g	a	a	t	t
MH709353	5	a	t	g	g	a	g	t	t	a	a	a	g	t	a	t	t	c	t	t	g	a	a	t	t
MH709358	5	a	t	g	a	a	g	t	t	a	a	a	g	t	a	t	t	c	t	t	g	a	a	t	t
MH709360	5	a	t	g	g	a	t	t	t	a	a	a	g	t	a	t	t	c	t	t	g	a	a	t	t
MH709362	5	a	t	g	g	a	g	t	t	a	a	a	g	t	a	t	t	c	t	t	g	a	a	t	t
MH709365	5	a	t	g	g	a	g	t	t	a	a	a	g	t	a	t	t	c	t	t	g	a	a	t	t
MH709373	5	a	t	g	g	a	g	t	t	a	a	a	g	t	a	t	t	c	t	t	g	a	a	t	t
MH709374	5	a	t	g	g	a	g	t	t	a	a	a	g	t	a	t	t	c	t	t	g	a	a	t	t
KC112914	6	a	t	g	a	a	g	t	t	a	a	a	g	t	a	g	t	c	t	t	g	a	a	t	t
KP771914	6	a	t	g	a	a	g	t	t	a	a	a	g	t	a	g	t	c	t	t	g	a	a	t	t
KP702725	6	a	t	g	a	a	g	t	t	a	a	a	g	t	a	g	t	c	t	t	g	a	a	t	t
KP771890	9	a	t	g	a	a	g	t	t	a	a	a	g	t	a	t	t	c	c	t	g	a	a	t	t
JN704710	9	a	t	g	a	a	g	t	t	a	a	a	g	t	a	t	t	c	c	t	g	a	a	t	t
KY037798	9	a	t	g	a	a	g	t	t	a	a	a	g	t	a	t	t	c	c	t	g	a	a	t	t
MH709312	9	a	t	g	a	a	g	t	t	a	a	a	g	t	a	t	t	c	c	t	g	a	a	t	t
MH709352	9	a	t	g	a	a	g	t	t	a	a	a	g	t	a	t	t	c	c	t	g	a	a	t	t
AB097933	Vac	a	t	g	a	a	g	t	t	a	a	a	g	t	a	t	t	c	c	t	g	a	a	t	t
JN704698	Vac	a	t	g	a	a	g	t	t	a	a	a	g	t	a	t	t	c	c	t	g	a	a	t	t
JN704697	Vac	a	t	g	a	a	g	t	t	a	a	a	g	t	a	t	t	c	c	t	g	a	a	t	t
DQ008355	Vac	a	t	g	a	a	g	t	t	a	a	a	g	t	a	t	t	c	c	t	g	a	a	t	y
JF306641	Vac	a	t	g	a	a	g	t	t	a	a	a	g	t	a	t	t	c	c	t	g	a	a	t	t
AB097932	Vac	a	t	g	a	a	g	t	t	a	a	a	g	t	a	t	t	c	c	t	g	a	a	t	t
DQ008354	Vac	a	t	g	a	a	g	t	t	a	a	a	g	t	a	t	t	c	c	t	g	a	a	t	t
KU926312	Vac	a	t	g	a	a	g	t	t	a	a	a	g	t	a	t	t	c	c	t	g	a	a	t	t
KU926313	Vac	a	t	g	a	a	g	t	t	a	a	a	g	t	a	t	t	c	c	t	g	a	a	t	t
KU926314	Vac	a	t	g	a	a	g	t	t	a	a	a	g	t	a	t	t	c	c	t	g	a	a	t	t
KF558371	Vac	a	t	g	a	a	g	t	t	a	a	a	g	t	a	t	t	c	c	t	g	a	a	t	t
KF558372	Vac	a	t	g	a	a	g	t	t	a	a	a	g	t	a	t	t	c	c	t	g	a	a	t	t
KF558373	Vac	a	t	g	a	a	g	t	t	a	a	a	g	t	a	t	t	c	c	t	g	a	a	t	c
KF558374	Vac	a	t	g	a	a	g	t	t	a	a	a	g	t	a	t	t	c	c	t	g	a	a	t	c
KF558375	Vac	a	t	g	a	a	g	t	t	a	a	a	g	t	a	t	t	c	c	t	g	a	a	t	c
KF558376	Vac	a	t	g	a	a	g	t	t	a	a	a	g	t	a	t	t	c	c	t	g	a	a	t	t
KF558377	Vac	a	t	g	a	a	g	t	t	a	a	a	g	t	a	t	t	c	c	t	g	a	a	t	c
KF558378	Vac	a	t	g	a	a	g	t	t	a	a	a	g	t	a	t	t	c	c	t	g	a	a	t	c
KF558379	Vac	a	t	g	a	a	g	t	t	a	a	a	g	t	a	t	t	c	c	t	g	a	a	t	c
KF558380	Vac	a	t	g	a	a	g	t	t	a	a	a	g	t	a	t	t	c	c	t	g	a	a	t	t
KF558381	Vac	a	t	g	a	a	g	t	t	a	a	a	g	t	a	t	t	c	c	t	g	a	a	t	c
KF558382	Vac	a	t	g	a	a	g	t	t	a	a	a	g	t	a	t	t	c	c	t	g	a	a	t	c
KF558383	Vac	a	t	g	a	a	g	t	t	a	a	a	g	t	a	t	t	c	c	t	g	a	a	t	t
KF558384	Vac	a	t	g	a	a	g	t	t	a	a	a	g	t	a	t	t	c	c	t	g	a	a	t	t
KF558385	Vac	a	t	g	a	a	g	t	t	a	a	a	g	t	a	t	t	c	c	t	g	a	a	t	t
KF558386	Vac	a	t	g	a	a	g	t	t	a	a	a	g	t	a	t	t	c	c	t	g	a	a	t	c
KF558387	Vac	a	t	g	a	a	g	t	t	a	a	a	g	t	a	t	t	c	c	t	g	a	a	t	t
KF558388	Vac	a	t	g	a	a	g	t	t	a	a	a	g	t	a	t	t	c	c	t	g	a	a	t	t
KF558389	Vac	a	t	g	a	a	g	t	t	a	a	a	g	t	a	t	t	c	c	t	g	a	a	t	c
KF558390	Vac	a	t	g	a	a	g	t	t	a	a	a	g	t	a	t	t	c	c	t	g	a	a	t	c
KF558391	Vac	a	t	g	a	a	g	t	t	a	a	a	g	t	a	t	t	c	c	t	g	a	a	t	c
KF811485	Vac	a	t	g	a	a	g	t	t	a	a	a	g	t	a	t	t	c	c	t	g	a	a	t	t
KF853225	Vac	a	t	g	a	a	g	t	t	a	a	a	g	t	a	t	t	c	c	t	g	a	a	t	t
KF853226	Vac	a	t	g	a	a	g	t	t	a	a	a	g	t	a	t	t	c	c	t	g	a	a	t	t
KF853227	Vac	a	t	g	a	a	g	t	t	a	a	a	g	t	a	t	t	c	c	t	g	a	a	t	c
KF853228	Vac	a	t	g	a	a	g	t	t	a	a	a	g	t	a	t	t	c	c	t	g	a	a	t	c
KF853229	Vac	a	t	g	a	a	g	t	t	a	a	a	g	t	a	t	t	c	c	t	g	a	a	t	c
KF853230	Vac	a	t	g	a	a	g	t	t	a	a	a	g	t	a	t	t	c	c	t	g	a	a	t	t
KF853231	Vac	a	t	g	a	a	g	t	t	a	a	a	g	t	a	t	t	c	c	t	g	a	a	t	c
KF853232	Vac	a	t	g	a	a	g	t	t	a	a	a	g	t	a	t	t	c	c	t	g	a	a	t	t
KF853233	Vac	a	t	g	a	a	g	t	t	a	a	a	g	t	a	t	t	c	c	t	g	a	a	t	c
KF853234	Vac	a	t	g	a	a	g	t	t	a	a	a	g	t	a	t	t	c	c	t	g	a	a	t	c
KF853235	Vac	a	t	g	a	a	g	t	t	a	a	a	g	t	a	t	t	c	c	t	g	a	a	t	t
MF898328	Vac	a	t	g	a	a	g	t	t	a	a	a	g	t	a	t	t	c	c	t	g	a	a	t	y
JN704709	VIII	a	t	g	a	a	g	t	t	a	a	a	g	t	a	g	t	c	t	t	g	a	a	t	t

GB-ID			2						5		9							9								
	97495	97525	97591	97697	97748	97780	97796	97804	97842	97891	97954	98261	98262	98264	98365	98410	98437	98468	98494	98647	98659	98662	98676	98692	98712	98742
MH499467	a	a	c	t	g	g	t	t	g	t	c	t	a	g	a	a	t	a	t	t	c	a	t	t	a	a
MH499469	a	a	c	t	g	g	t	t	g	t	c	t	a	g	a	a	t	a	t	t	c	a	t	t	a	a
MH709321	a	a	c	t	g	g	t	t	g	t	c	t	a	g	a	a	t	a	t	t	c	a	t	t	a	a
MH709324	a	a	c	t	g	g	t	t	g	t	c	t	a	g	a	a	t	a	t	t	c	a	t	t	a	a
MH709325	a	a	c	t	g	g	t	t	g	t	c	t	a	g	a	a	t	a	t	t	c	a	t	t	a	a
MH709327	a	a	c	t	g	g	t	t	g	t	c	t	a	g	a	a	t	a	t	t	c	a	t	t	a	a
MH709328	a	a	c	t	g	g	t	t	g	t	c	t	a	g	a	a	t	a	t	t	c	a	t	t	a	a
MH709330	a	a	c	t	g	g	t	t	g	t	c	t	a	g	a	a	t	a	t	t	c	a	t	t	a	a
MH709332	a	a	c	t	g	g	t	t	g	t	c	t	a	g	a	a	t	a	t	t	c	a	t	t	a	a
MH709334	a	a	c	t	g	g	t	t	g	t	c	t	a	g	a	a	t	a	t	t	c	a	t	t	a	a
MH709335	a	a	c	t	g	g	t	t	g	t	c	t	a	g	a	a	t	a	t	t	c	a	t	t	a	a
MH709336	a	a	c	t	g	g	t	t	g	t	c	t	a	g	a	a	t	a	t	t	c	a	t	t	a	a
MH709338	a	a	c	t	g	g	t	t	g	t	c	t	a	g	a	a	t	a	t	t	c	a	t	t	a	a
MH709340	a	a	c	t	g	g	t	t	g	t	c	t	a	g	a	a	t	a	t	t	c	a	t	t	a	a
MH709342	a	a	c	t	g	g	t	t	g	t	c	t	a	g	a	a	t	a	t	t	c	a	t	t	a	a
MH709343	a	a	c	t	g	g	t	t	g	t	c	t	a	g	a	a	t	a	t	t	c	a	t	t	a	a
MH709345	a	a	c	t	g	g	t	t	g	t	c	t	a	g	a	a	t	a	t	t	c	a	t	t	a	a
MH709347	a	a	c	t	g	g	t	t	g	t	c	t	a	g	a	a	t	a	t	t	c	a	t	t	a	a
MH709349	a	a	c	t	g	g	t	t	g	t	c	t	a	g	a	a	t	a	t	t	c	a	t	t	a	a
MH709350	a	a	c	t	g	g	t	t	g	t	c	t	a	g	a	a	t	a	t	t	c	a	t	t	a	a
MH709351	a	a	c	t	g	g	t	t	g	t	c	t	a	g	g	a	t	a	t	t	c	a	t	t	a	a
MH709353	a	a	c	t	g	g	t	t	g	t	c	t	a	g	a	a	t	a	t	t	c	a	t	t	a	a
MH709358	a	a	c	t	g	g	t	t	g	t	c	t	a	g	a	a	t	a	t	t	c	a	t	t	a	a
MH709360	a	a	c	t	g	g	t	t	g	t	c	t	a	g	a	a	t	a	t	t	c	a	t	t	a	a
MH709362	a	a	c	t	g	g	t	t	g	t	c	t	a	g	a	a	t	a	t	t	c	a	t	t	a	a
MH709365	g	a	c	t	g	g	t	t	g	t	c	t	a	g	a	a	t	a	t	t	c	a	t	t	a	a
MH709373	a	a	c	t	g	g	t	t	g	t	c	t	a	g	a	a	t	a	t	t	c	a	t	t	a	a
MH709374	a	a	c	t	g	g	t	t	g	t	c	t	a	g	a	a	t	a	t	t	c	a	t	t	a	a
KC112914	a	a	c	t	g	g	t	t	a	t	c	t	a	g	a	a	c	a	t	t	t	a	t	t	a	a
KP771914	a	a	c	t	g	g	t	t	a	t	c	t	a	g	a	a	c	a	t	t	t	a	t	t	a	a
KP702725	a	a	c	t	g	g	t	t	a	t	c	t	a	g	a	a	c	a	t	t	t	a	t	t	a	a
KP771890	a	a	c	t	g	a	t	t	a	t	a	t	a	g	a	a	c	g	t	t	t	a	t	t	a	a
JN704710	a	a	c	t	g	a	t	t	a	t	a	t	a	g	a	a	c	g	t	t	t	a	t	t	a	a
KY037798	a	a	c	t	g	a	t	t	a	t	a	t	a	g	a	a	c	g	t	t	t	a	t	t	a	a
MH709312	a	a	c	t	g	a	t	t	a	t	a	t	a	g	a	a	c	g	t	t	t	a	t	t	a	a
MH709352	a	a	c	t	g	a	t	t	a	t	a	t	a	g	a	a	c	g	t	t	t	a	t	t	a	a
AB097933	a	a	t	t	g	g	t	t	a	t	c	t	a	g	a	a	c	a	t	t	c	a	t	t	a	a
JN704698	a	a	t	t	g	g	t	t	a	t	c	t	a	g	a	a	c	a	t	t	c	a	t	t	a	a
JN704697	a	a	t	t	a	g	c	t	a	t	c	t	a	g	a	a	c	a	t	t	c	a	t	t	a	a
DQ008355	a	a	t	t	r	g	t	t	a	t	c	t	a	g	a	a	c	a	t	t	c	a	t	t	a	a
JF306641	a	a	t	t	g	g	c	t	a	t	c	t	a	g	a	a	c	a	t	t	c	a	t	t	a	a
AB097932	a	a	t	t	r	g	y	t	a	t	c	t	a	g	a	a	c	a	t	t	c	a	t	t	a	a
DQ008354	a	a	t	t	r	g	y	t	a	t	c	t	a	g	a	a	c	a	t	t	c	a	t	t	a	a
KU926312	a	a	t	t	g	g	c	t	a	t	c	t	a	g	a	a	c	a	t	t	c	a	t	t	a	a
KU926313	a	a	t	t	a	g	t	t	a	t	c	t	a	g	a	a	c	a	t	t	c	a	t	t	a	a
KU926314	a	a	t	t	g	g	t	t	a	t	c	t	a	g	a	a	c	a	t	t	c	a	t	t	a	a
KF558371	a	a	t	t	a	g	t	t	a	t	c	t	a	g	a	a	c	a	t	t	c	a	t	t	a	a
KF558372	a	a	t	t	g	g	c	t	a	t	c	t	a	g	a	a	c	a	t	t	c	a	t	t	a	a
KF558373	a	a	t	t	g	g	t	t	a	t	c	t	a	g	a	a	c	a	t	t	c	a	t	t	a	a
KF558374	a	a	t	t	g	g	t	t	a	t	c	t	a	g	a	a	c	a	t	t	c	a	t	t	a	a
KF558375	a	a	t	t	g	g	t	t	a	t	c	t	a	g	a	a	c	a	t	t	c	a	t	t	a	a
KF558376	a	a	t	t	g	g	t	t	a	g	c	t	a	g	a	a	c	a	t	t	c	a	t	t	a	a
KF558377	a	a	t	t	g	g	t	t	a	t	c	t	a	g	a	a	c	a	t	t	c	a	t	t	a	a
KF558378	a	a	t	t	g	g	t	t	a	t	c	t	a	g	a	a	c	a	t	t	c	a	t	t	a	a
KF558379	a	a	t	t	g	g	t	t	a	t	c	t	a	g	a	a	c	a	t	t	c	a	t	t	a	a
KF558380	a	a	t	t	g	g	c	t	a	t	c	t	a	g	a	a	c	a	t	t	c	a	t	t	a	a
KF558381	a	a	t	t	g	g	t	t	a	t	c	t	a	g	a	g	c	a	t	t	c	a	t	t	a	a
KF558382	a	a	t	t	g	g	t	t	a	t	c	t	a	g	a	a	c	a	t	t	c	a	t	t	a	a
KF558383	a	a	t	t	g	g	t	t	a	t	c	t	a	g	a	a	c	a	t	t	c	a	t	t	a	a
KF558384	a	a	t	t	g	g	t	t	a	t	c	t	a	g	a	a	c	a	t	t	c	a	t	t	a	a
KF558385	a	a	t	t	g	g	t	t	a	t	c	t	a	g	a	a	c	a	t	t	c	a	t	t	a	a
KF558386	a	a	t	t	g	g	t	t	a	t	c	t	a	g	a	a	c	a	t	t	c	a	t	t	a	a
KF558387	a	a	t	t	a	g	t	t	a	t	c	t	a	g	a	a	c	a	t	t	c	a	t	t	a	a
KF558388	a	a	t	t	g	g	t	t	a	t	c	t	a	g	a	a	c	a	t	t	c	a	t	t	a	a
KF558389	a	a	t	t	g	g	t	t	a	t	c	t	a	g	a	a	c	a	t	t	c	a	t	t	a	a
KF558390	a	a	t	t	g	g	t	t	a	t	c	t	a	g	a	g	c	a	t	t	c	a	t	t	a	a
KF558391	a	a	t	t	g	g	t	t	a	t	c	t	a	g	a	a	c	a	t	t	c	a	t	t	a	a
KF811485	a	a	t	t	g	g	t	t	a	t	c	t	a	g	a	a	c	a	t	t	c	a	t	t	a	a
KF853225	a	a	t	t	a	g	t	t	a	t	c	t	a	g	a	g	c	a	t	t	c	a	t	t	a	a
KF853226	a	a	t	t	a	g	t	t	a	t	c	t	a	g	a	a	c	a	t	t	c	a	t	t	a	a
KF853227	a	a	t	t	g	g	t	t	a	t	c	t	a	g	a	a	c	a	t	t	c	a	t	t	a	a
KF853228	a	a	t	t	g	g	t	t	a	t	c	t	a	g	a	a	c	a	t	t	c	a	t	t	a	a
KF853229	a	a	t	t	g	g	t	t	a	t	c	t	a	g	a	a	c	a	t	t	c	a	t	t	a	a
KF853230	a	a	t	t	a	g	t	t	a	t	c	t	a	g	a	a	c	a	t	t	c	a	t	t	a	a
KF853231	a	a	t	t	g	g	t	t	a	t	c	t	a	g	a	a	c	a	t	t	c	a	t	t	a	a
KF853232	a	a	t	t	g	g	c	t	a	t	c	t	a	g	a	a	c	a	t	t	c	a	t	t	a	a
KF853233	a	a	t	t	g	g	t	t	a	t	c	t	a	g	a	a	c	a	t	t	c	a	t	t	a	a
KF853234	a	a	t	t	g	g	t	t	a	t	c	t	a	g	a	a	c	a	t	t	c	a	t	t	a	a
KF853235	a	a	t	t	g	g	t	t	a	g	c	t	a	g	a	a	c	a	t	t	c	a	t	t	a	a
MF898328	a	a	t	t	r	g	y	t	a	t	c	t	a	g	a	a	c	a	t	t	c	a	t	t	a	a
JN704709	a	a	c	t	g	g	t	t	a	t	c	t	a	g	a	a	c	a	t	t	t	a	t	t	a	a

GB-ID	2	2																					
	98765	98807	98809	98816	98825	98832	98893	98897	98911	98960	99004	99008	99041	99064_1	99071	99137	99186	99223	99225	99227	99228	99235	99256
MH499467	t	a	t	a	c	a	t	a	t	t	t	g	a	-	g	g	t	c	t	t	t	a	a
MH499469	t	a	t	a	c	a	t	a	t	t	t	g	a	-	g	g	t	c	t	t	t	a	a
MH709321	t	a	t	a	c	a	t	a	t	t	t	g	a	-	g	g	t	c	t	t	t	a	a
MH709324	t	a	t	a	c	a	t	a	t	t	t	g	a	-	g	g	t	c	t	t	t	a	a
MH709325	t	a	t	a	c	a	t	a	t	t	t	g	a	-	g	g	t	c	t	t	t	a	a
MH709327	t	a	t	a	c	a	t	a	t	t	t	g	a	-	g	g	t	c	t	t	t	a	a
MH709328	t	a	t	a	c	a	t	a	t	t	t	g	a	-	g	g	t	c	t	t	t	a	a
MH709330	t	a	t	a	c	a	t	g	t	t	t	g	a	-	g	g	t	c	t	t	t	a	a
MH709332	t	a	t	a	c	a	t	a	t	t	t	g	a	-	g	g	t	c	t	t	t	a	a
MH709334	t	a	t	a	c	a	t	a	t	t	t	g	a	-	g	g	t	c	t	t	t	a	a
MH709335	t	a	t	a	c	a	t	a	t	t	t	g	a	-	g	g	t	c	t	t	t	a	a
MH709336	t	a	t	a	c	a	t	a	t	t	t	g	a	-	g	g	t	c	t	t	t	a	a
MH709338	t	a	t	a	c	a	t	a	t	t	t	g	a	-	g	g	t	c	t	t	t	a	a
MH709340	t	a	t	a	c	a	t	a	t	c	t	g	a	-	g	g	t	c	t	t	t	a	a
MH709342	t	a	t	a	c	a	t	a	t	t	t	g	a	-	g	g	t	c	t	t	t	a	a
MH709343	t	a	t	a	c	a	t	a	t	t	t	g	a	-	g	g	t	c	t	t	t	a	a
MH709345	t	a	t	a	c	a	t	a	t	t	t	g	a	-	g	g	t	c	t	t	t	a	a
MH709347	t	a	t	a	c	a	t	a	t	t	t	g	a	-	g	g	t	c	t	t	t	a	a
MH709349	t	a	t	a	c	a	t	a	t	t	t	g	a	-	g	g	t	c	t	t	t	a	a
MH709350	t	a	t	a	c	a	t	a	t	t	t	g	a	-	g	g	t	c	t	t	t	a	a
MH709351	t	a	t	a	c	a	t	a	t	t	t	g	a	-	g	g	t	c	t	t	t	a	a
MH709353	t	a	t	a	c	a	t	a	t	t	t	g	a	-	g	g	t	c	t	t	t	a	a
MH709358	t	a	t	a	c	a	t	a	t	t	t	g	a	-	g	g	t	c	t	t	t	a	a
MH709360	t	a	t	a	c	a	t	a	t	t	t	g	a	-	g	g	t	c	t	t	t	a	a
MH709362	t	a	t	a	c	a	t	a	t	t	t	g	a	-	g	g	t	c	t	t	t	a	a
MH709365	t	a	t	a	c	a	t	a	t	t	t	g	a	-	g	g	t	c	t	t	t	a	a
MH709373	t	a	t	a	c	a	t	a	t	t	t	g	a	-	g	g	t	c	t	t	t	a	a
MH709374	t	a	t	a	c	a	t	a	t	t	t	g	a	-	g	g	t	c	t	t	t	a	a
KC112914	t	a	t	a	c	a	t	a	t	t	t	a	a	-	g	g	t	c	t	t	t	a	a
KP771914	t	a	t	a	c	a	t	a	t	t	t	a	a	-	g	g	t	c	t	t	t	a	a
KP702725	t	a	t	a	c	a	t	a	t	t	t	a	a	-	g	g	t	t	t	t	t	a	a
KP771890	t	a	t	a	c	a	t	a	t	t	t	a	a	-	g	g	t	c	t	t	t	a	a
JN704710	t	a	t	a	c	a	t	a	t	t	t	g	a	-	g	g	t	c	t	t	t	a	a
KY037798	t	a	t	a	c	a	t	a	t	t	t	a	a	-	g	g	t	c	t	t	t	a	a
MH709312	t	a	t	a	c	a	t	a	t	t	t	a	a	-	g	g	t	c	t	t	t	a	a
MH709352	t	a	t	a	c	a	t	a	t	t	t	a	a	-	g	g	t	c	t	t	t	a	a
AB097933	c	c	t	a	t	a	t	a	t	t	t	g	a	-	g	g	t	c	t	-	-	a	a
JN704698	c	c	t	a	t	a	t	a	t	t	t	g	a	-	g	g	t	c	t	-	-	a	a
JN704697	c	c	t	a	t	a	t	a	t	t	t	g	a	-	g	g	t	c	t	-	-	a	a
DQ008355	c	c	t	a	t	a	t	a	t	t	t	g	a	-	g	g	t	c	t	-	-	a	a
JF306641	c	c	t	a	t	a	t	a	t	t	t	g	a	-	g	g	t	c	t	-	-	a	a
AB097932	c	c	t	a	t	a	t	a	t	t	t	g	a	-	g	g	t	c	t	-	-	a	a
DQ008354	c	c	t	a	t	a	t	a	t	t	t	g	a	-	g	g	t	c	t	-	-	a	a
KU926312	c	c	t	a	t	a	t	a	t	t	t	g	a	-	g	g	t	c	t	-	-	a	a
KU926313	c	c	t	a	t	a	t	a	t	t	t	g	a	-	g	g	t	c	t	-	-	a	a
KU926314	c	c	t	a	t	a	t	a	t	t	t	g	a	-	g	g	t	c	t	-	-	a	a
KF558371	c	c	t	a	t	a	t	a	t	t	t	g	a	-	g	g	t	c	t	-	-	a	a
KF558372	c	c	t	a	t	a	t	a	t	t	t	g	a	-	g	g	t	c	t	-	-	a	a
KF558373	c	c	t	a	t	a	t	a	t	t	t	g	a	-	g	g	t	c	t	-	-	a	a
KF558374	c	c	t	a	t	a	t	a	t	t	t	g	a	-	g	g	t	c	t	-	-	a	a
KF558375	c	c	t	a	t	a	t	a	t	t	t	g	a	-	g	g	t	c	t	-	-	a	a
KF558376	c	c	t	a	t	a	t	a	t	t	t	g	a	-	g	g	t	c	t	-	-	a	a
KF558377	c	c	t	a	t	a	c	a	t	t	t	g	a	-	g	g	t	c	t	-	-	a	a
KF558378	c	c	t	a	t	a	t	a	t	t	t	g	a	-	g	g	t	c	t	-	-	a	a
KF558379	c	c	t	a	t	a	t	a	t	t	t	g	a	-	g	g	t	c	t	-	-	a	a
KF558380	c	c	t	a	t	a	t	a	t	t	t	g	a	-	g	g	t	c	t	-	-	a	a
KF558381	c	c	t	a	t	a	t	a	t	t	t	g	a	-	g	g	t	c	t	-	-	a	a
KF558382	c	c	t	a	t	a	t	a	t	t	t	g	a	-	g	g	t	c	t	-	-	a	a
KF558383	c	c	t	a	t	a	t	a	t	t	t	g	a	-	g	g	t	c	t	-	-	a	a
KF558384	c	c	t	a	t	a	t	a	t	t	t	g	a	-	g	g	t	c	t	-	-	a	a
KF558385	c	c	t	a	t	a	t	a	t	t	t	g	a	-	g	g	t	c	t	-	-	a	a
KF558386	c	c	t	a	t	a	t	a	t	t	t	g	a	-	g	g	t	c	t	-	-	a	a
KF558387	c	c	t	a	t	a	t	a	t	t	t	g	a	-	g	g	t	c	t	-	-	a	a
KF558388	c	c	t	a	t	a	t	a	t	t	t	g	a	-	g	g	t	c	t	-	-	a	a
KF558389	c	c	t	a	t	a	t	a	t	t	t	g	a	-	g	g	t	c	t	-	-	a	a
KF558390	c	c	t	a	t	a	t	a	t	t	t	g	a	-	g	g	t	c	t	-	-	a	a
KF558391	c	c	t	a	t	a	t	a	t	t	t	g	a	-	g	g	t	c	t	-	-	a	a
KF811485	c	c	t	a	t	a	t	a	t	t	t	g	a	-	g	g	t	c	t	-	-	a	a
KF853225	c	c	t	a	t	a	t	a	t	t	t	g	a	-	g	g	t	c	t	-	-	a	a
KF853226	c	c	t	a	t	a	t	a	t	t	t	g	a	-	g	g	t	c	t	-	-	a	a
KF853227	c	c	t	a	t	a	t	a	t	t	t	g	a	-	g	g	t	c	t	-	-	a	a
KF853228	c	c	t	a	t	a	t	a	t	t	t	g	a	-	g	g	t	c	t	-	-	a	a
KF853229	c	c	t	a	t	a	t	a	t	t	t	g	a	g	g	g	t	c	t	-	-	a	a
KF853230	c	c	t	a	t	a	t	a	t	t	t	g	a	-	g	g	t	c	t	-	-	a	a
KF853231	c	c	t	a	t	a	t	a	t	t	t	g	a	-	g	g	t	c	t	-	-	a	a
KF853232	c	c	t	a	t	a	t	a	t	t	t	g	a	-	g	g	t	c	t	-	-	a	a
KF853233	c	c	t	a	t	a	t	a	t	t	t	g	a	-	g	g	t	c	t	-	-	a	a
KF853234	c	c	t	a	t	a	t	a	t	t	t	g	a	-	g	g	t	c	t	-	-	a	a
KF853235	c	c	t	a	t	a	t	a	t	t	t	g	a	-	g	g	t	c	t	-	-	a	a
MF898328	c	c	t	a	t	a	t	a	t	t	t	g	a	-	g	g	t	c	t	-	-	a	a
JN704709	t	a	t	a	t	a	t	a	t	t	t	a	a	-	g	g	t	c	t	t	t	a	a

Supplementary Table 3: 2880 SN

GB-ID	Clade markers						2																				2																								2																			
	Position in Dumas	99288	99312	99334	99341	99343	99421	99431	99464	99504	99510	99523	99619	99685	99709	99728	99762	99766	99784	99789	99803	99823	99828	99871	99894	99906	99981	99997	100043	100110	100114	100116	100117	100123	100126	100128	100148	100151	100159	100172	100177	100178	100181	100182	100184	100190	100195	100281	100283	100287	100289	100292	100296	100298	100329	100364	100383	100384	100387	100434	100436	100450	100461	100533	100653	100749	100770	100774
AY548170	1	g	c	t	a	t	t	a	t	a	t	a	a	g	a	a	a	t	a	t	a	a	t	t	g	t	c	g	a	g	t	t	t	t	a	c	t	t	a	g	c	t	t	t	a	g	c	g	a	g	g	g	g	a	t	c	a	a	g	a	a	t	c	c	c	a	g	g
AY548171	1	g	c	t	a	t	t	a	t	a	t	a	a	g	g	a	a	t	a	c	a	a	t	t	g	t	c	g	a	g	t	t	t	g	a	c	t	t	a	g	c	t	t	t	a	g	c	g	a	g	g	g	g	a	t	c	a	a	g	a	a	t	c	c	c	a	g	g
DQ479953	1	g	c	t	a	t	t	a	t	a	t	a	a	g	a	a	a	t	a	t	a	a	t	t	g	t	c	g	a	g	t	t	t	t	a	c	t	t	a	g	c	t	t	t	a	g	c	g	a	g	g	g	g	a	t	c	a	a	g	a	a	t	c	c	c	a	g	g
DQ479954	1	g	c	t	a	t	t	a	t	a	t	a	a	g	a	a	a	t	a	t	a	a	t	t	g	t	c	g	a	g	t	t	t	t	a	c	t	t	a	g	c	t	t	t	a	g	c	g	a	g	g	g	g	a	t	c	a	a	g	a	a	t	c	c	c	a	g	g
DQ479958	1	g	c	t	a	t	t	a	t	a	t	a	a	g	g	a	a	t	a	t	a	a	t	t	g	t	c	g	a	g	t	t	t	t	a	c	t	t	a	g	c	t	t	t	a	g	c	g	a	g	g	g	g	a	t	c	a	a	g	a	a	t	c	c	c	a	g	g
DQ479959	1	g	c	t	a	t	t	a	t	a	t	a	a	g	g	a	a	t	a	t	a	a	t	t	g	t	c	g	a	g	t	t	t	t	a	c	t	t	a	g	c	t	t	t	a	g	c	g	a	g	g	g	g	a	t	c	a	a	g	a	a	t	c	c	c	a	g	g
DQ479961	1	g	c	t	a	t	t	a	t	a	t	a	a	g	a	a	a	t	a	t	a	a	t	t	g	t	c	g	a	g	t	t	t	t	a	c	t	t	a	g	c	t	t	t	a	g	c	g	a	g	g	g	g	a	t	c	a	a	g	a	a	t	c	c	c	a	g	g
DQ479962	1	g	c	t	a	t	t	a	t	a	t	a	a	g	a	a	a	t	a	t	a	a	t	t	g	t	c	g	a	g	t	t	t	t	a	c	t	t	a	g	c	t	t	t	a	g	c	g	a	g	g	g	g	a	t	c	a	a	g	a	a	t	c	c	c	a	g	g
DQ479963	1	g	c	t	a	t	t	a	t	a	t	a	a	g	a	a	a	t	a	t	a	a	t	t	g	t	c	g	a	g	t	t	t	t	a	c	t	t	a	g	c	t	t	t	a	g	c	g	a	g	g	g	g	a	t	c	a	a	g	a	a	t	c	c	c	a	g	g
DQ674250	1	g	c	t	a	t	t	a	t	a	t	a	a	g	g	a	a	t	a	t	a	a	t	t	g	t	c	g	a	g	t	t	t	t	a	c	t	t	a	g	c	t	t	t	a	g	c	g	a	g	g	g	g	a	t	c	a	a	g	a	a	t	c	c	c	a	g	g
KX262864	1	g	c	t	a	t	t	a	t	a	t	a	a	g	g	a	a	t	a	t	a	a	t	t	g	t	c	g	a	g	t	t	t	t	a	c	t	t	a	g	c	t	t	t	a	g	c	g	a	g	g	g	g	a	t	c	a	a	g	a	a	t	c	c	c	a	g	g
KX262865	1	g	c	t	a	t	t	a	t	a	t	a	a	g	g	a	a	t	a	t	a	a	t	t	g	t	c	g	a	g	t	t	t	t	a	c	t	t	a	g	c	t	t	t	a	g	c	g	a	g	g	g	g	a	t	c	a	a	g	a	a	t	c	c	c	a	g	g
KX262866	1	g	c	t	a	t	t	a	t	a	t	a	a	g	g	a	a	t	a	t	a	a	t	t	g	t	c	g	a	g	t	t	t	t	a	c	t	t	a	g	c	t	t	t	a	g	c	g	a	g	g	g	g	a	t	c	a	a	g	a	a	t	c	c	c	a	g	g
EU154348	1	g	c	t	a	t	t	a	t	a	t	a	a	g	a	a	a	t	a	t	a	a	t	t	g	t	c	g	a	g	t	t	t	t	a	c	t	t	a	g	c	t	t	t	a	g	c	g	a	g	g	g	g	a	t	c	a	a	g	a	a	t	c	c	c	a	g	g
JN704690	1	g	c	t	a	t	t	a	t	a	t	a	a	g	a	a	a	t	a	t	a	a	t	t	g	t	c	g	a	g	t	t	t	t	a	c	t	t	a	g	c	t	t	t	a	g	c	g	a	g	g	g	g	a	t	c	a	a	g	a	a	t	c	c	c	a	g	g
JN704691	1	g	c	t	a	t	t	a	t	a	t	a	a	g	a	a	a	t	a	t	a	a	t	t	g	t	c	g	a	g	t	t	t	t	a	c	t	t	a	g	c	t	t	t	a	g	c	g	a	g	g	g	g	a	t	c	a	a	g	a	a	t	c	c	c	a	g	g
JN704692	1	g	c	t	a	t	t	a	t	a	t	a	a	g	a	a	a	t	a	t	a	a	t	t	g	t	c	g	a	g	t	t	t	t	a	c	t	t	a	g	c	t	t	t	a	g	c	g	a	g	g	g	g	a	t	c	a	a	g	a	a	t	c	c	c	a	g	g
JN704693	1	g	c	t	a	t	t	a	t	a	t	a	a	g	a	a	a	t	a	t	a	a	t	t	g	t	c	g	a	g	t	t	t	t	a	c	t	t	a	g	c	t	t	t	a	g	c	g	a	g	g	g	g	a	t	c	a	a	g	a	a	t	c	c	c	a	g	g
JN704694	1	g	c	t	a	t	t	a	t	a	t	a	a	g	a	a	a	t	a	t	a	a	t	t	g	t	c	g	a	g	t	t	t	t	a	c	t	t	a	g	c	t	t	t	a	g	c	g	a	g	g	g	g	a	t	c	a	a	g	a	a	t	c	c	c	a	g	g
JN704695	1	g	c	t	a	t	t	a	t	a	t	a	a	g	a	a	a	t	a	t	a	a	t	t	g	t	c	g	a	g	t	t	t	t	a	c	t	t	a	g	c	t	t	t	a	g	c	g	a	g	g	g	g	a	t	c	a	a	g	a	a	t	c	c	c	a	g	g
JN704696	1	g	c	t	a	t	t	a	g	a	t	a	a	g	a	a	a	t	a	t	a	a	t	t	g	t	c	g	a	g	t	t	t	t	a	c	t	t	a	g	c	t	t	t	a	g	c	g	a	g	g	g	g	a	t	c	a	a	g	a	a	t	c	c	c	a	g	g
NC001348	1	g	c	t	a	t	t	a	t	a	t	a	a	g	a	a	a	t	a	t	a	a	t	t	g	t	c	g	a	g	t	t	t	t	a	c	t	t	a	g	c	t	t	t	a	g	c	g	a	g	g	g	g	a	t	c	a	a	g	a	a	t	c	c	c	a	g	g
KP771892	1	g	c	t	a	t	t	a	t	a	t	a	a	g	a	a	a	t	a	t	a	a	t	t	g	t	c	g	a	g	t	t	t	t	a	c	t	g	a	g	c	t	t	t	a	g	c	g	a	g	g	g	g	a	t	c	a	a	g	a	a	t	c	c	c	a	g	g
KP771894	1	g	c	t	a	t	t	a	t	a	t	a	a	g	a	a	a	t	a	t	a	a	t	t	g	t	c	g	a	g	t	t	t	t	a	c	t	t	a	g	c	t	t	t	a	g	c	g	a	g	g	g	g	a	t	c	a	a	g	a	a	t	c	c	c	a	g	g
KP771900	1	g	c	t	a	c	t	a	t	a	t	a	a	g	a	a	a	t	a	t	a	a	t	t	g	t	c	g	a	g	t	t	t	t	a	c	t	t	a	g	c	t	t	t	a	g	c	g	a	g	g	g	g	a	t	c	a	a	g	a	a	t	c	c	c	a	g	g
KP771901	1	g	c	t	a	t	t	a	t	a	t	a	a	g	a	a	a	t	a	t	a	a	t	t	g	t	c	g	a	g	t	t	t	t	a	c	t	t	a	g	c	t	t	t	a	g	c	g	a	g	g	g	g	a	t	c	a	a	g	a	a	t	c	c	c	a	g	g
KP771902	1	g	c	t	a	t	t	a	t	a	t	a	a	g	a	a	a	t	a	t	a	a	t	t	g	t	c	g	a	g	t	t	t	t	a	c	t	t	a	g	c	t	t	t	a	g	c	g	a	g	g	g	g	a	t	c	a	a	g	a	a	t	c	c	c	a	g	a
KP771908	1	g	c	t	a	t	t	a	t	a	t	a	a	g	g	a	a	t	a	t	a	a	t	t	g	t	c	g	a	g	t	t	t	t	a	c	t	t	a	g	c	t	t	t	a	g	c	g	a	g	g	g	g	a	t	c	a	a	g	a	a	t	c	c	c	a	g	g
KP771912	1	g	c	t	a	t	t	a	t	a	t	a	a	g	g	a	a	t	a	t	a	a	t	t	g	t	c	g	a	g	t	t	t	t	a	c	t	t	a	g	c	t	t	t	a	g	c	g	a	g	g	g	g	a	t	c	a	a	g	a	a	t	c	c	c	a	g	g
KP771913	1	g	c	t	a	t	t	a	t	a	t	a	a	g	g	a	a	t	a	t	a	a	t	t	g	t	c	g	a	g	t	t	t	t	a	c	t	t	a	g	c	t	t	t	a	g	c	g	a	g	g	g	g	a	t	c	a	a	g	a	a	t	c	c	c	a	g	g
KP771917	1	g	c	t	a	t	t	a	t	a	t	a	a	g	a	a	a	t	a	t	a	a	t	t	g	t	c	g	a	g	t	t	t	t	a	c	t	t	a	g	c	t	t	t	a	g	c	g	a	g	g	g	g	a	t	c	a	a	g	g	a	t	c	c	c	a	g	g
KP771918	1	g	c	t	a	t	t	a	t	a	t	a	a	g	a	a	a	t	a	t	a	a	t	t	g	t	c	g	a	g	t	t	t	t	a	c	t	t	a	g	c	t	t	t	a	g	c	g	a	g	g	g	g	a	t	c	a	a	g	a	a	t	c	c	c	a	g	g
KP771920	1	g	c	t	a	t	t	a	t	a	t	a	a	g	a	a	a	t	a	t	a	a	t	t	g	t	c	g	a	g	t	t	t	t	a	c	t	t	a	g	c	t	t	t	a	g	c	g	a	g	g	g	g	a	t	c	a	a	g	a	a	t	c	c	c	a	g	g
MH709361	1	g	t	t	a	t		a	t	a	t	a	a	g	g	a	a	t	a	t	a	a	t	t	g	t	c	g	a	g	t	t	t	t	a	c	t	t	a	g	c	t	t	t	a	g	c	g	a	g	g	g	g	a	t	c	a	a	g	a	a	t	c	c	c	a	g	g
KU926318	2	g	c	t	a	t	g	a	t	a	t	a	a	g	g	g	a	t	a	t	a	g	t	t	g	t	t	g	a	g	a	t	t	t	a	c	t	g	a	g	c	c	t	t	a	g	c	g	g	g	g	g	g	a	t	c	a	a	g	a	g	t	c	c	c	a	g	g
KU926319	2	g	c	t	a	t	g	a	t	a	t	a	a	g	g	a	a	t	a	t	a	a	t	t	g	t	t	g	a	g	a	t	t	t	a	c	t	g	a	g	c	t	t	t	a	g	c	g	g	g	g	g	g	a	t	c	a	a	g	a	a	t	c	c	t	a	g	g
KU926320	2	g	c	t	g	t	g	a	t	g	t	a	a	g	g	a	g	t	a	t	g	a	t	t	g	t	t	g	a	g	a	t	t	t	a	c	t	g	a	g	c	t	t	t	a	g	c	g	g	g	g	g	g	a	t	c	a	a	g	a	a	t	c	c	t	a	g	g
KU926321	2	g	c	t	a	t	g	a	t	a	t	g	a	g	g	a	a	t	a	t	a	a	t	t	g	t	t	g	a	g	a	t	t	t	a	c	t	g	a	g	c	t	t	t	a	g	c	g	g	g	g	g	g	a	t	c	a	a	g	a	a	t	c	c	t	a	g	g
KU926322	2	g	c	t	a	t	g	a	t	a	t	g	a	g	g	a	a	t	a	t	a	a	t	t	g	t	t	g	a	g	a	t	t	t	a	c	t	g	a	g	c	t	t	t	a	g	c	g	g	g	g	g	g	a	t	c	a	a	g	a	a	t	c	c	t	a	g	g
KC847290	2	g	c	t	a	t	g	a	t	a	t	a	a	g	g	a	a	t	a	t	a	a	t	t	g	t	t	g	a	g	a	t	t	t	a	c	t	g	a	g	c	t	t	t	a	g	c	g	g	g	g	g	g	a	t	c	a	a	g	a	a	t	c	c	t	a	g	g
KJ767491	2	g	c	t	a	t	g	a	t	a	t	a	a	g	g	a	a	t	a	t	a	a	t	t	g	t	t	g	a	g	a	t	t	t	a	c	t	g	a	g	c	t	t	t	a	g	c	g	g	g	g	g	g	a	t	c	a	a	g	a	a	t	c	c	c	a	g	g
KJ767492	2	g	c	t	a	t	g	a	t	a	t	a	a	g	g	a	a	t	a	t	a	a	t	t	g	t	t	g	a	g	a	t	t	t	a	c	t	g	a	g	c	t	t	t	a	g	c	g	g	g	g	g	g	a	t	c	a	a	g	a	a	t	c	c	t	a	g	g
KJ808816	2	g	c	t	a	t	g	a	t	a	t	g	a	g	g	a	a	t	a	t	a	a	t	t	g	t	t	g	a	g	a	t	t	t	a	c	t	g	a	g	c	t	t	t	a	g	c	g	g	g	g	g	g	a	t	c	a	a	g	a	a	t	c	c	t	a	g	g
KP771925	2	g	c	t	a	t	g	a	t	a	t	a	a	g	g	a	a	t	a	t	a	a	t	t	g	t	t	g	a	g	a	t	t	t	a	c	t	g	a	g	c	t	t	t	a	g	c	g	g	g	g	g	g	a	t	c	a	a	g	a	a	t	c	c	t	a	g	g
KU926315	2	g	c	t	a	t	g	a	t	a	t	a	a	g	g	a	a	t	a	t	a	a	t	t	g	t	t	g	a	g	a	t	t	t	a	c	t	g	a	g	c	t	t	t	a	g	c	g	g	g	g	g	g	a	t	c	a	a	g	a	a	t	c	c	c	a	g	g
KU926316	2	g	c	t	a	t	g	a	t	a	t	a	a	g	g	a	a	t	a	t	a	a	t	t	g	t	t	g	a	g	a	t	t	t	a	c	t	g	a	g	c	t	t	t	a	g	c	g	g	g	g	g	g	a	t	c	a	a	g	a	a	t	c	c	c	a	g	g
KU926317	2	g	c	t	a	t	g	a	t	a	t	a	a	g	g	a	a	t	a	t	a	a	t	t	g	t	t	g	a	g	a	t	t	t	a	c	t	g	a	g	c	t	t	t	a	g	c	g	g	g	g	g	g	a	t	c	a	a	g	a	a	t	c	c	c	a	g	g
KY037796	2	g	c	t	a	t	g	a	t	a	t	a	a	g	g	a	a	t	a	t	a	a	t	t	g	t	t	g	a	g	a	t	t	t	a	c	t	g	a	g	c	t	t	t	a	g	c	g	g	g	g	g	g	a	t	c	a	a	g	a	a	t	c	c	c	a	g	g
MF004348	2	g	c	t	a	t	g	a	t	a	t	a	a	g	g	a	a	t	a	t	a	a	t	t	g	t	t	g	a	g	a	t	t	t	a	c	t	g	a	g	c	t	t	t	a	g	c	g	g	g	g	g	g	a	t	c	a	a	g	a	a	t	c	c	t	a	g	g
MH709309	2	g	c	t	a	t	g	a	t	a	t	a	a	g	g	a	a	t	a	t	a	a	t	t	g	t	t	g	a	g	a	t	t	t	a	c	t	g	a	g	c	t	t	t	a	g	c	g	g	g	g	g	g	a	t	c	a	a	g	a	a	t	c	c	c	a	g	g
MH709310	2	g	c	t	a	t	g	a	t	a	t	a	a	g	g	a	a	t	a	t	a	a	t	t	g	t	t	g	a	g	a	t	t	t	a	c	t	g	a	g	c	t	t	t	a	g	c	g	g	g	g	g	g	a	t	c	a	a	g	a	a	t	c	c	c	a	g	g
MH709311	2	g	c	t	a	t	g	a	t	c	t	a	a	g	g	a	a	t	a	t	a	a	t	t	g	t	t	g	a	g	a	t	t	t	a	c	t	g	a	g	c	t	t	t	a	g	c	g	g	g	g	g	g	a	t	c	a	a	g	a	a	t	c	c	c	a	g	g
MH709313	2	g	c	t	a	t	g	a	t	a	t	a	a	g	g	a	a	t	a	t	a	a	t	t	g	t	t	g	a	g	a	t	t	t	a	c	t	g	a	g	c	t	t	t	a	g	c	g	g	g	g	g	g	a	t	c	a	a	g	a	a	t	c	c	c	a	g	g
MH709314	2	g	c	t	a	t	g	a	t	a	t	a	a	g	g	a	a	t	a	t	a	a	t	t	g	t	t	g	a	g	a	t	t	t	a	c	t	g	a	g	c	t	t	t	a	g	c	g	g	g	g	g	g	a	t	c	a	a	g	a	a	t	c	c	c	a	g	g
MH709315	2	g	c	t	a	t	g	a	t	a	t	a	a	g	g	a	a	t	a	t	a	a	t	t	g	t	t	g	a	g	a	t	t	t	a	c	t	g	a	g	c	t	t	t	a	g	c	g	g	g	g	g	g	a	t	c	a	a	g	a	a	t	c	c	c	a	g	g
MH709319	2	g	c	t	a	t	g	a	t	a	t	a	a	g	g	a	a	t	a	t	a	a	t	t	g	t	t	g	a	g	a	t	t	t	a	c	t	g	a	g	c	t	t	t	a	g	c	g	g	g	g	g	g	a	t	c	a	a	g	a	a	t	c	c	c	a	g	g
MH709320	2	g	c	t	a	t	g	a	t	a	t	a	a	g	g	a	a	t	g	t	a	a	t	t	t	t	t	g	a	g	a	t	t	t	a	c	t	g	a	g	c	t	t	t	a	g	c	g	g	g	g	g	g	a	t	c	a	a	g	a	a	t	c	c	c	a	g	g
MH709329	2	g	c	t	a	t	g	a	t	a	t	a	a	g	g	a	a	t	a	t	a	a	t	t	g	t	t	g	a	g	a	t	t	t	a	c	t	g	a	g	c	t	t	t	a	g	c	g	g	g	g	g	g	a	t	c	a	a	g	a	a	t	c	c	c	a	g	g
MH709331	2	g	c	t	a	t	g	a	t	a	t	a	a	g	g	a	a	t	a	t	a	a	t	t	g	t	t	g	a	g	a	t	t	t	a	c	t	g	a	g	c	t	t	t	a	g	c	g	g	g	g	g	g	a	t	c	a	a	g	a	a	t	c	c	c	a	g	g
MH709333	2	g	c	t	a	t	g	a	t	a	t	a	a	g	g	a	a	t	a	t	a	a	t	t	g	t	t	g	a	g	a	t	t	t	a	c	t	g	a	g	c	t	t	t	a	g	c	g	g	g	g	g	g	a	t	c	a	a	g	a	a	t	c	c	c	a	g	g
MH709337	2	g	c	t	a	t	g	a	t	a	t	a	a	g	g	a	a	t	a	t	a	a	t	t	g	t	t	g	a	g	a	t	t	t	a	c	t	g	a	g	c	t	t	t	a	g	c	g	g	g	g	g	g	a	t	c	a	a	g	a	a	t	c	c	c	a	g	g
MH709339	2	g	c	g	a	t	g	a	t	a	t	a	a	g	g	a	a	t	a	t	a	a	t	t	g	t	t	g	a	g	a	t	t	t	a	c	t	g	a	g	c	t	t	t	a	g	c	g	g	g	g	g	g	a	t	c	a	a	g	a	a	t	c	c	c	a	g	g
MH709344	2	g	c	t	a	t	g	a	t	a	t	a	a	g	g	a	a	t	a	t	a	a	t	t	g	t	t	g	a	g	a	t	t	t	a	c	t	g	a	g	c	t	t	t	a	g	c	g	g	g	g	g	g	a	t	c	a	a	g	a	a	t	c	c	c	a	g	g
MH709348	2	g	c	t	a	t	g	a	t	a	t	a	a	g	g	a	a	t	a	t	a	a	t	t	g	t	t	g	a	g	a	t	t	t	a	c	t	g	a	g	c	t	t	t	a	g	c	g	g	g	g	g	g	a	t	c	a	a	g	a	a	t	c	c	c	a	g	g
MH709355	2	g	c	t	a	t	g	a	t	a	t	a	a	g	g	a	a	t	a	t	a	a	t	t	g	t	t	g	a	g	a	t	t	t	a	c	t	g	a	a	c	t	t	t	a	g	c	g	g	g	g	g	g	a	t	c	a	a	g	a	a	t	c	c	c	a	g	g
MH709359	2	g	c	t	a	t	g	a	t	a	t	a	a	g	g	a	a	t	a	t	a	a	t	t	g	t	t	g	a	g	a	t	t	t	a	c	t	g	a	g	c	t	t	t	a	g	c	g	g	g	g	g	g	a	t	c	a	a	g	a	a	t	c	c	c	a	g	g
MH709363	2	g	c	t	a	t	g	a	t	a	t	a	a	g	g	a	a	t	g	t	a	a	t	t	t	t	t	g	a	g	a	t	t	t	a	c	t	g	a	g	c	t	t	t	a	g	c	g	g	g	g	g	g	a	t	c	a	a	g	a	a	t	c	c	c	a	g	g
MH709366	2	g	c	t	a	t	g	a	t	a	t	a	a	g	g	a	a	t	a	t	a	a	t	t	g	t	t	g	a	g	a	t	t	t	a	c	t	g	a	g	c	t	t	t	a	g	c	g	g	g	g	g	g	a	t	c	a	a	g	a	a	t	c	c	c	a	g	g
MH709376	2	g	c	t	a	t	g	a	t	a	t	a	a	g	g	a	a	t	a	t	a	a	t	t	g	t	t	g	a	g	a	t	t	t	a	c	t	g	a	g	c	t	t	t	a	g	c	g	g	g	g	g	g	a	t	c	a	a	g	a	a	t	c	c	c	a	g	g
MH709377	2	g	c	t	a	t	g	a	t	a	t	g	a	g	g	a	a	t	a	t	a	a	t	t	g	t	t	g	a	g	a	t	t	t	a	c	t	g	a	g	c	t	t	t	a	g	c	g	g	g	g	g	g	a	t	c	a	a	g	a	a	t	c	c	t	a	g	g
**MW316406**	2	g	c	t	a	t	g	a	t	a	t	a	a	g	g	a	a	t	a	t	a	a	t	t	g	t	t	g	a	g	a	t	t	t	a	c	t	g	a	g	c	t	t	t	a	g	c	g	g	g	g	g	g	a	t	c	a	a	g	a	a	t	c	c	c	a	g	g
AJ871403	3	g	c	t	a	t	t	a	t	a	t	a	a	g	g	a	a	t	a	t	a	a	t	t	g	t	c	g	a	g	t	t	t	t	a	c	t	t	a	g	c	t	t	t	a	g	c	g	a	g	g	g	g	a	t	c	a	a	g	a	a	t	c	c	c	a	g	g
DQ479955	3	g	c	t	a	t	t	a	t	a	t	a	a	g	g	a	a	t	a	t	a	a	t	t	g	t	c	g	a	g	t	t	t	t	a	c	t	t	a	g	c	t	t	t	a	g	c	g	a	g	g	g	g	a	t	c	a	a	g	a	a	t	c	c	c	a	g	g
DQ479956	3	g	c	t	a	t	t	a	t	a	t	a	a	g	g	a	a	t	a	t	a	a	t	t	g	t	c	g	a	g	t	t	t	t	a	c	t	t	a	g	c	t	t	t	a	g	c	g	a	g	g	g	g	a	t	c	a	a	g	a	a	t	c	c	c	a	g	g
DQ479957	3	g	c	t	a	t	t	a	t	a	t	a	a	g	g	a	a	c	a	t	a	a	t	t	g	t	c	g	a	g	t	t	t	t	a	c	t	t	a	g	c	t	t	t	a	g	c	g	a	g	g	g	g	a	t	c	a	a	g	a	a	t	c	c	c	a	g	g
JN704699	3	g	c	t	a	t	t	a	t	a	t	a	a	g	g	a	a	t	a	t	a	a	t	t	g	t	c	g	a	g	t	t	t	t	a	c	t	t	a	g	c	t	t	t	a	g	c	g	a	g	g	g	g	a	t	c	a	a	g	a	a	t	c	c	c	a	g	g
JN704700	3	g	c	t	a	t	t	a	t	a	t	a	a	g	g	a	a	t	a	t	a	a	t	t	g	t	c	a	a	g	t	t	t	t	a	c	t	t	a	g	c	t	t	t	a	g	c	g	a	g	g	g	g	a	t	c	a	a	g	a	a	t	c	c	c	a	g	g
JN704701	3	g	c	t	a	t	t	a	t	a	t	a	a	g	g	a	a	t	a	t	a	a	t	t	g	t	c	g	a	g	t	t	t	t	a	c	t	t	a	g	c	t	t	t	a	g	c	g	a	g	g	g	g	a	t	c	a	a	g	a	a	t	c	c	c	a	g	g
JN704702	3	g	c	t	a	t	t	a	t	a	t	a	a	g	g	a	a	t	a	t	a	a	t	t	g	t	c	g	a	g	t	t	t	t	a	c	t	t	a	g	c	t	t	t	a	g	c	g	a	g	g	g	g	a	t	c	a	a	g	a	a	t	c	c	c	a	g	g
JN704703	3	g	c	t	a	t	t	a	t	a	t	a	a	g	g	a	a	t	a	t	a	a	t	t	g	t	c	g	a	g	t	t	t	t	a	c	t	t	a	g	c	t	t	t	a	g	c	g	a	g	g	g	g	a	t	c	a	a	g	a	a	t	c	c	c	a	g	g
KP771891	3	g	c	t	a	t	t	a	t	a	t	a	a	g	g	a	a	t	a	t	a	a	t	t	g	t	c	g	a	g	t	t	t	t	a	c	t	t	a	g	c	t	t	t	a	a	c	g	a	g	g	g	g	a	t	c	a	a	g	a	a	t	c	c	c	a	g	g
KP771893	3	g	c	t	a	t	t	a	t	a	t	a	a	g	g	a	a	t	a	t	a	a	t	t	g	t	c	g	a	g	t	t	t	t	a	c	t	t	a	g	c	t	t	t	a	g	c	g	a	g	g	g	g	a	t	c	a	a	g	a	a	t	c	c	c	a	g	g
KP771895	3	g	c	t	a	t	t	a	t	a	t	a	a	g	g	a	a	t	a	t	a	a	t	g	g	t	c	g	a	g	t	t	t	t	a	c	t	t	a	g	c	t	t	t	a	g	c	g	a	g	g	g	g	a	t	c	a	a	g	a	a	t	c	c	c	a	g	g
KP771896	3	g	c	t	a	t	t	a	t	a	t	a	a	g	g	a	a	t	a	t	a	a	t	t	g	t	c	g	a	g	t	t	t	t	a	c	t	t	a	g	c	t	t	t	a	g	c	g	a	g	g	g	g	a	t	c	a	a	g	a	a	t	c	c	c	a	g	g
KP771897	3	g	c	t	a	t	t	a	t	a	t	a	a	g	g	a	a	t	a	t	a	a	t	t	g	t	c	g	a	g	t	t	t	t	a	c	t	t	a	g	c	t	t	t	a	g	c	g	a	g	g	g	g	a	t	c	a	a	g	a	a	t	c	c	c	a	g	g
KP771899	3	g	c	t	a	t	t	a	t	a	t	a	a	g	g	a	a	t	a	t	a	a	t	t	g	t	c	g	a	g	t	t	t	t	a	c	t	t	a	g	c	t	t	t	a	g	a	g	a	g	g	g	g	a	t	c	a	a	g	a	a	t	c	c	c	a	g	g
KP771903	3	g	c	t	a	t	t	a	t	a	t	a	a	g	g	a	a	t	a	t	a	a	t	t	g	t	c	g	a	g	t	t	t	t	a	c	t	t	a	g	c	t	t	t	a	g	c	g	a	g	g	g	g	a	t	c	a	a	g	a	a	t	c	c	c	a	g	g
KP771907	3	a	c	t	a	t	t	a	t	a	t	a	a	g	g	a	a	t	a	t	a	a	t	t	g	t	c	g	a	g	t	t	t	t	a	c	t	t	a	g	c	t	t	t	a	g	a	g	a	g	g	g	g	a	t	c	a	a	g	a	a	t	c	c	c	a	g	g
KP771910	3	g	c	t	a	t	t	a	t	a	t	a	a	g	g	a	a	t	a	t	a	a	t	t	g	t	c	g	a	g	t	t	t	t	a	c	t	t	a	g	c	t	t	t	a	g	c	g	a	g	g	g	g	a	t	c	a	a	g	a	a	t	c	c	c	a	g	g
KP771915	3	g	c	t	a	t	t	a	t	a	t	a	a	g	g	a	a	t	a	t	a	a	t	t	g	t	c	g	a	g	t	t	t	t	a	c	t	t	a	g	c	t	t	t	a	g	c	g	a	g	g	g	g	a	t	c	a	a	g	a	a	t	c	c	c	a	g	g
KP771916	3	g	c	t	a	t	t	a	t	a	t	a	a	g	g	a	a	t	a	t	a	a	t	t	g	t	c	g	a	g	t	t	t	t	a	c	t	t	a	g	c	t	t	t	a	g	c	g	a	g	g	g	g	a	t	c	a	a	g	a	a	t	c	c	c	a	g	g
KP771921	3	g	c	t	a	t	t	a	t	a	t	a	a	g	g	a	a	t	a	t	a	a	t	t	g	t	c	g	a	g	t	t	t	t	a	c	t	t	a	g	c	t	t	t	a	g	c	g	a	g	g	g	g	a	t	c	a	a	g	a	a	t	c	c	c	a	g	g
KP771923	3	g	c	t	a	t	t	a	t	a	t	a	a	g	g	a	a	t	a	t	a	a	t	t	g	t	c	g	a	g	t	t	t	t	a	c	t	t	a	g	c	t	t	t	a	g	c	g	a	g	g	g	g	a	t	c	a	a	g	a	a	t	c	c	c	a	g	g
KP771889	3	g	c	t	a	t	t	a	t	a	t	a	a	g	g	a	a	t	a	t	a	a	t	t	g	t	c	g	a	g	t	t	t	t	a	c	t	t	a	g	c	t	t	t	a	g	c	g	a	g	g	g	g	a	t	c	a	a	g	a	a	t	c	c	c	a	g	g
KU926311	3	g	c	t	a	t	t	g	t	a	t	a	a	g	g	a	a	t	a	t	a	a	t	t	g	t	c	g	a	g	t	t	t	t	a	c	t	t	a	g	c	t	t	t	a	g	c	g	a	g	g	g	g	a	t	c	a	a	g	a	a	c	c	c	c	a	g	g
JQ911599	3	g	c	t	a	t	t	a	t	a	t	a	a	g	g	a	a	t	a	t	a	a	t	t	g	t	c	g	a	g	t	t	t	t	a	c	t	t	a	g	c	t	t	t	a	g	c	g	a	g	g	g	g	a	t	c	a	a	g	a	a	t	c	c	c	a	g	g
MH709317	3	g	c	t	a	t	t	a	t	a	t	a	a	g	g	a	a	t	a	t	a	a	t	t	g	t	c	g	a	g	a	t	t	t	a	c	t	t	a	g	c	t	t	t	a	g	c	a	a	g	g	g	g	a	t	c	a	a	g	a	a	t	c	c	c	a	g	g
MH709346	3	g	c	t	a	t	t	a	t	a	t	a	a	g	g	a	a	t	a	t	a	a	t	t	g	t	c	g	a	g	a	t	t	t	a	c	t	t	a	g	c	t	t	t	a	g	c	a	a	g	g	g	g	a	t	c	a	a	g	a	a	t	c	c	c	a	g	g
MH709375	3	g	c	t	a	t	t	a	t	a	t	a	a	g	g	a	a	t	a	t	a	a	t	t	g	t	c	g	a	g	a	t	t	t	a	c	t	t	a	g	c	t	t	t	a	g	c	a	a	g	g	g	g	a	t	c	a	a	g	a	a	t	c	c	c	a	g	g
DQ452050	4	g	c	t	a	t	t	a	t	a	t	a	a	g	g	a	a	t	a	t	a	a	t	t	g	t	c	g	a	g	a	t	t	t	a	c	t	t	a	g	c	t	t	t	a	g	c	g	a	g	g	g	g	a	t	c	a	a	g	a	a	t	c	c	c	a	g	g
DQ479960	4	g	c	t	a	t	t	a	t	a	t	a	a	g	g	a	a	t	a	t	a	a	t	t	g	t	c	g	a	g	a	t	t	t	a	c	t	t	a	g	c	t	t	t	a	g	c	g	a	g	g	g	g	a	t	c	a	a	g	a	a	t	c	c	c	a	g	g
KP771898	4	g	c	t	a	t	t	a	t	a	t	a	a	g	g	a	a	t	a	t	a	a	t	t	g	t	c	g	a	g	a	t	t	t	a	c	t	t	a	g	c	t	t	t	a	g	c	g	a	g	g	g	g	a	t	c	a	a	g	a	a	t	c	c	c	a	g	g
MH709316	4	g	c	t	a	t	t	a	t	a	t	a	a	g	g	a	a	t	a	t	a	a	t	t	g	t	c	g	a	g	a	t	t	t	a	c	t	t	a	g	c	t	t	t	a	g	c	g	a	g	g	g	g	a	t	c	a	a	g	a	a	t	c	c	c	a	g	g
MH709318	4	g	c	t	a	t	t	a	t	a	t	a	a	g	g	a	a	t	a	t	a	a	t	t	g	t	c	g	a	g	a	t	t	t	a	c	t	t	a	g	c	t	t	t	a	g	c	g	a	g	g	g	g	a	t	c	a	a	g	a	a	t	c	c	c	a	g	g
MH709322	4	g	c	t	a	t	t	a	t	a	t	a	a	g	g	a	a	t	a	t	a	a	t	t	g	t	c	g	a	g	a	t	t	t	a	c	t	t	a	g	c	t	t	t	a	g	c	g	a	g	g	g	g	a	t	c	a	a	g	a	a	t	c	c	c	a	g	g
MH709323	4	g	c	t	a	t	t	a	t	a	t	a	a	g	g	a	a	t	a	t	a	a	t	t	g	t	c	g	a	g	a	t	t	t	a	c	t	t	a	g	c	t	t	t	a	g	c	g	a	g	g	g	g	a	t	c	a	a	g	a	a	t	c	c	c	a	g	g
MH709326	4	g	c	t	a	t	t	a	t	a	t	a	a	g	g	a	a	t	a	t	a	a	t	t	g	t	c	g	a	g	a	t	t	t	a	c	t	t	a	g	c	t	t	t	a	g	c	g	a	g	g	g	g	a	t	c	a	a	g	a	a	t	c	c	c	a	g	g
MH709341	4	g	c	t	a	t	t	a	t	a	t	a	g	g	g	a	a	t	a	t	a	a	t	t	g	t	c	g	a	g	a	t	t	t	a	c	t	t	a	g	c	t	t	t	a	g	c	g	a	g	g	g	g	a	t	c	a	a	g	a	a	t	c	c	c	a	g	g
MH709357	4	g	c	t	a	t	t	a	t	a	t	a	a	g	g	a	a	t	a	t	a	a	t	t	g	t	c	g	a	g	a	t	t	t	a	c	t	t	a	g	c	t	t	t	a	g	c	g	a	g	g	g	g	a	t	c	a	a	g	a	a	t	c	c	c	a	g	g
MH709364	4	g	c	t	a	t	t	a	t	a	t	a	a	g	g	a	a	t	a	t	a	a	t	t	g	t	c	g	a	g	a	t	t	t	a	c	t	t	a	g	c	t	t	t	a	g	c	g	a	g	g	g	g	a	t	c	a	a	g	a	a	t	c	c	c	a	g	g
MH709370	4	g	c	t	a	t	t	a	t	a	t	a	a	g	g	a	a	t	a	t	a	a	t	t	g	t	c	g	a	g	a	t	t	t	a	c	t	t	a	g	c	t	t	t	a	g	c	g	a	g	g	g	g	a	t	c	a	a	g	a	a	t	c	c	c	a	g	g
MH709371	4	g	c	t	a	t	t	a	t	a	t	a	a	g	g	a	a	t	a	t	a	a	t	t	g	t	c	g	a	g	a	t	t	t	a	c	t	t	a	g	c	t	t	t	a	g	c	g	a	g	g	a	g	a	t	c	a	a	g	a	a	t	c	c	c	a	g	g
MH709372	4	g	c	t	a	t	t	a	t	a	t	a	a	g	g	a	a	t	a	t	a	a	t	t	g	t	c	g	a	g	a	t	t	t	a	c	t	t	a	g	c	t	t	t	a	g	c	g	a	g	g	g	g	a	t	c	a	a	g	a	a	t	c	c	c	a	g	g
DQ457052	5	g	c	t	a	t	t	a	t	a	t	a	a	t	g	a	a	t	a	t	a	a	t	t	g	t	c	g	a	g	a	t	t	t	a	c	t	t	a	g	c	t	t	t	a	g	c	g	a	g	g	g	g	a	t	c	a	a	g	a	a	t	c	c	c	a	g	g
JN704704	5	g	c	t	a	t	t	a	t	a	t	a	a	g	g	a	a	t	a	t	a	a	t	t	g	t	c	g	a	g	a	t	t	t	a	c	t	t	a	g	c	t	t	t	a	g	c	g	a	g	g	g	g	a	t	c	a	a	g	a	a	t	c	c	c	a	g	g
JN704705	5	g	c	t	a	t	t	a	t	a	t	a	a	g	g	a	a	t	a	t	a	a	t	t	g	t	c	g	a	g	a	t	t	t	a	c	t	t	a	g	c	t	t	t	a	g	c	g	a	g	g	g	g	a	t	c	a	a	g	a	a	t	c	c	c	a	g	g
JN704706	5	g	c	t	a	t	t	a	t	a	t	a	a	g	g	a	a	t	a	t	a	a	t	t	g	t	c	g	a	g	a	t	t	t	a	c	t	t	a	g	c	t	t	t	a	g	c	g	a	g	g	g	g	a	t	c	a	a	g	a	a	t	c	c	c	a	g	g
JN704707	5	g	c	t	a	t	t	a	t	a	t	a	a	g	g	a	a	t	a	t	a	a	t	t	g	t	c	g	a	g	a	t	t	t	a	c	t	t	a	g	c	t	t	t	a	g	c	g	a	g	a	g	g	a	t	c	a	a	g	a	a	t	c	c	c	a	g	g
JN704708	5	g	c	t	a	t	t	a	t	a	t	a	a	g	g	a	a	t	a	t	a	a	t	t	g	t	c	g	a	g	a	t	t	t	a	c	t	t	a	g	c	t	t	t	a	g	c	g	a	g	g	g	g	a	t	c	a	a	g	a	a	t	c	c	c	a	g	g
KP771904	5	g	c	t	a	t	t	a	t	a	t	a	a	g	g	a	a	t	a	t	a	a	t	t	g	t	c	g	a	g	a	t	t	t	a	c	t	t	a	g	c	t	t	t	a	g	c	g	a	g	g	g	g	a	t	c	a	a	g	a	a	t	c	c	c	a	g	g
KP771905	5	g	c	t	a	t	t	a	t	a	t	a	a	g	g	a	a	t	a	t	a	a	t	t	g	t	c	g	a	g	a	t	t	t	a	c	t	t	a	g	c	t	t	t	a	g	c	g	a	g	g	g	g	a	t	c	a	a	g	a	a	t	c	c	c	a	g	g
KP771906	5	g	c	t	a	t	t	a	t	a	t	a	a	g	g	a	a	t	a	t	a	a	t	t	g	t	c	g	a	g	a	t	t	t	a	c	t	t	a	g	c	t	t	t	a	g	c	g	a	g	g	g	g	a	t	c	a	a	g	a	a	t	c	c	c	a	g	g
KP771919	5	g	c	t	a	t	t	a	t	a	t	a	a	g	g	a	a	t	a	t	a	a	t	t	g	t	c	g	a	g	a	t	t	t	a	c	g	t	a	g	c	t	t	t	a	g	c	g	a	g	g	g	g	a	t	c	a	a	g	a	a	t	c	c	c	a	g	g
KP771922	5	g	c	t	a	t	t	a	t	a	t	a	a	g	g	a	a	t	a	t	a	a	g	t	g	t	c	g	a	g	a	t	t	t	a	c	t	t	a	g	c	t	t	t	a	g	c	g	a	g	g	g	g	a	t	c	a	a	g	a	a	t	c	c	c	a	g	g
KP771924	5	g	c	t	a	t	t	a	t	a	t	a	a	g	g	a	a	t	a	t	a	a	t	t	g	t	c	g	a	g	w	t	t	t	a	c	t	t	a	g	c	t	t	t	a	g	c	g	a	g	g	g	g	a	t	c	a	a	g	a	a	t	c	c	c	a	g	g
KM355696	5	g	c	t	a	t	t	a	t	a	t	a	a	g	g	a	a	t	a	t	a	a	t	t	g	t	c	g	a	g	a	t	t	t	a	c	t	t	a	g	c	t	t	t	a	g	c	g	a	g	g	g	g	a	t	c	a	a	g	a	a	t	c	c	c	a	g	g
KM355697	5	g	c	t	a	t	t	a	t	a	t	a	a	g	g	a	a	t	a	t	a	a	t	t	g	t	c	g	a	g	a	t	t	t	a	c	t	t	a	g	c	t	t	t	a	g	c	g	a	g	g	g	g	a	t	c	a	a	g	a	a	t	c	c	c	a	g	g
KM355698	5	g	c	t	a	t	t	a	t	a	t	a	a	g	g	a	a	t	a	t	a	a	t	t	g	t	c	g	a	g	a	t	t	t	a	c	t	t	a	g	c	t	t	t	a	g	c	g	a	g	g	g	g	a	t	c	a	a	g	a	a	t	c	c	c	a	g	g
KM355699	5	g	c	t	a	t	t	a	t	a	t	a	a	g	g	a	a	t	a	t	a	a	t	t	g	t	c	g	a	g	a	t	t	t	a	c	t	t	a	g	c	t	t	t	a	g	c	g	a	g	g	g	g	a	t	c	a	a	g	a	a	t	c	c	c	a	g	g
KM355701	5	g	c	t	a	t	t	a	t	a	t	a	a	g	g	a	a	t	a	t	a	a	t	t	g	t	c	g	a	g	a	t	t	t	a	c	t	t	a	g	c	t	t	t	a	g	c	g	a	g	g	g	g	a	t	c	a	a	g	a	a	t	c	c	c	a	g	g
KM355702	5	g	c	t	a	t	t	a	t	a	t	a	a	g	g	a	a	t	a	t	a	a	t	t	g	t	c	g	a	g	a	t	t	t	a	c	t	t	a	g	c	t	t	t	a	g	c	g	a	g	g	g	g	a	t	c	a	a	g	a	a	t	c	c	c	a	g	g
KM355703	5	g	c	t	a	t	t	a	t	a	t	a	a	g	g	a	a	t	a	t	a	a	t	t	g	t	c	g	a	g	a	t	t	t	a	c	t	t	a	g	c	t	t	t	a	g	c	g	a	g	g	g	g	a	t	c	a	a	g	a	a	t	c	c	c	a	g	g
KM355704	5	g	c	t	a	t	t	a	t	a	t	a	a	g	g	a	a	t	a	t	a	a	t	t	g	t	c	g	a	g	a	t	t	t	a	c	t	t	a	g	c	t	t	t	a	g	c	g	a	g	g	g	g	a	t	c	a	a	g	a	a	t	c	c	c	a	g	g
KM355705	5	g	c	t	a	t	t	a	t	a	t	a	a	g	g	a	a	t	a	t	a	a	t	t	g	t	c	g	a	g	a	t	t	t	a	c	t	t	a	g	c	t	t	t	a	g	c	g	a	g	g	g	g	a	t	c	a	a	g	a	a	t	c	c	c	a	g	g
KM355706	5	g	c	t	a	t	t	a	t	a	t	a	a	g	g	a	a	t	a	t	a	a	t	t	g	t	c	g	a	g	a	t	t	t	a	c	t	t	a	g	c	t	t	t	a	g	c	g	a	g	g	g	g	a	t	c	a	a	g	a	a	t	c	c	c	a	g	g
KM355707	5	g	c	t	a	t	t	a	t	a	t	a	a	g	g	a	a	t	a	t	a	a	t	t	g	t	c	g	a	g	a	t	t	t	a	c	t	t	a	g	c	t	t	t	a	g	c	g	a	g	g	g	g	a	t	c	a	a	g	a	a	t	c	c	c	a	g	g
KM355708	5	g	c	t	a	t	t	a	t	a	t	a	a	g	g	a	a	t	a	t	a	a	t	t	g	t	c	g	a	g	a	t	t	t	a	c	t	t	a	g	c	t	t	t	a	g	c	g	a	g	g	g	g	a	t	c	a	a	g	a	a	t	c	c	c	a	g	g
KM355709	5	g	c	t	a	t	t	a	t	a	t	a	a	g	g	a	a	t	a	t	a	a	t	t	g	t	c	g	a	g	a	t	t	t	a	c	t	t	a	g	c	t	t	t	a	g	c	g	a	g	g	g	g	a	t	c	a	a	g	a	a	t	c	c	c	a	g	g
KM355711	5	g	c	t	a	t	t	a	t	a	t	a	a	g	g	a	a	t	a	t	a	a	t	t	g	t	c	g	a	g	a	t	t	t	a	c	t	t	a	g	c	t	t	t	a	g	c	g	a	g	g	g	g	a	t	c	a	a	g	a	a	t	c	c	c	a	g	g
KM355712	5	g	c	t	a	t	t	a	t	a	t	a	a	g	g	a	a	t	a	t	a	a	t	t	g	t	c	g	a	g	a	t	t	t	a	c	t	t	a	g	c	t	t	t	a	g	c	g	a	g	g	g	g	a	t	c	a	a	g	a	a	t	c	c	c	a	g	g
KY037797	5	[illegible]	[illegible]	[illegible]	[illegible]	[illegible]	[illegible]	[illegible]	[illegible]	[illegible]	[illegible]	[illegible]	[illegible]	[illegible]	[illegible]	[illegible]	[illegible]	[illegible]	[illegible]	[illegible]	[illegible]	[illegible]	[illegible]	[illegible]	[illegible]	[illegible]	[illegible]	[illegible]	[illegible]	[illegible]	[illegible]	[illegible]	[illegible]	[illegible]	[illegible]	[illegible]	[illegible]	[illegible]	[illegible]	[illegible]	[illegible]	[illegible]	[illegible]	[illegible]	[illegible]	[illegible]	[illegible]	[illegible]	[illegible]	[illegible]	[illegible]	[illegible]	[illegible]	[illegible]	[illegible]	[illegible]	[illegible]	[illegible]	[illegible]	[illegible]	[illegible]	[illegible]	[illegible]	[illegible]	[illegible]	[illegible]	[illegible]	[illegible]

Supplementary Table 3: 2880 SN

GB-ID	Clade markers						2																				2																								2																			
	Position in Dumas	99288	99312	99334	99341	99343	99421	99431	99464	99504	99510	99523	99619	99685	99709	99728	99762	99766	99784	99789	99803	99823	99828	99871	99894	99906	99981	99997	100043	100110	100114	100116	100117	100123	100126	100128	100148	100151	100159	100172	100177	100178	100181	100182	100184	100190	100195	100281	100283	100287	100289	100292	100296	100298	100329	100364	100383	100384	100387	100434	100436	100450	100461	100533	100653	100749	100770	100774
MH499467	5	g	c	t	a	t	t	a	t	a	t	a	a	g	g	a	a	t	a	t	a	a	t	t	g	t	c	g	a	g	a	t	t	t	a	c	t	t	a	g	c	t	t	t	a	g	c	g	a	g	g	g	g	c	a	c	a	a	g	a	a	t	c	c	c	a	g	g
MH499469	5	g	c	t	a	t	t	a	t	a	t	a	a	g	g	a	a	t	a	t	a	a	t	t	g	t	c	g	a	g	a	t	t	t	a	c	t	t	a	g	c	t	t	t	a	g	c	g	a	g	g	g	g	a	t	c	a	a	g	a	a	t	c	c	c	a	g	g
MH709321	5	g	c	t	a	t	t	a	t	a	t	a	a	g	g	a	a	t	a	t	a	a	t	t	g	t	c	g	a	g	a	t	t	t	a	c	t	t	a	g	c	t	t	t	a	g	c	g	a	g	g	g	g	a	t	c	a	a	g	a	a	t	c	c	c	a	g	g
MH709324	5	g	c	t	a	t	t	a	t	a	t	a	a	g	g	a	a	t	a	t	a	a	t	t	g	t	c	g	a	g	a	t	t	t	a	c	t	t	a	g	c	t	t	t	a	g	c	g	a	g	g	g	g	a	t	c	a	a	g	a	a	t	c	c	c	a	g	g
MH709325	5	g	c	t	a	t	t	a	t	a	t	a	a	g	g	a	a	t	a	t	a	a	t	t	g	t	c	g	a	g	a	t	t	t	a	c	t	t	a	g	c	t	t	t	a	g	c	g	a	g	g	g	g	a	t	c	a	a	g	a	a	t	c	c	c	a	g	g
MH709327	5	g	c	t	a	t	t	a	t	a	t	a	a	g	g	a	a	t	a	t	a	a	t	t	g	t	c	g	a	g	a	t	t	t	a	c	t	t	a	g	c	t	t	t	a	g	c	g	a	g	g	g	g	a	t	c	a	a	g	a	a	t	c	c	c	a	g	g
MH709328	5	g	c	t	a	t	t	a	t	a	t	a	a	g	g	a	a	t	a	t	a	a	t	t	g	t	c	g	a	g	a	t	t	t	a	c	t	t	a	g	c	t	t	t	a	g	c	g	a	g	g	g	g	a	t	c	a	a	g	a	a	t	c	c	c	a	g	g
MH709330	5	g	c	t	a	t	t	a	t	a	t	a	a	g	g	a	a	t	a	t	a	a	t	t	g	t	c	g	a	g	a	t	t	t	a	c	t	t	a	g	c	t	t	t	a	g	c	g	a	g	g	g	g	a	t	c	a	a	g	a	a	t	c	c	c	a	g	g
MH709332	5	g	c	t	a	t	t	a	t	a	t	a	a	g	g	a	a	t	a	t	a	a	t	t	g	t	c	g	a	g	a	t	t	t	a	c	t	t	a	g	c	t	t	t	a	g	c	g	a	g	g	g	g	a	t	c	a	a	g	a	a	t	c	c	c	a	g	g
MH709334	5	g	c	t	a	t	t	a	t	a	t	a	a	g	g	a	a	t	a	t	a	a	t	t	g	t	c	g	a	g	a	t	t	t	a	c	t	t	a	g	c	t	t	t	a	g	c	g	a	g	g	g	g	a	t	c	a	a	g	a	a	t	c	c	c	a	g	g
MH709335	5	g	c	t	a	t	t	a	t	a	t	a	a	g	g	a	a	t	a	t	a	a	t	t	g	t	c	g	a	g	a	t	t	t	a	c	t	t	a	g	c	t	t	t	a	g	c	g	a	g	g	g	g	a	t	c	a	a	g	a	a	t	c	c	c	a	g	g
MH709336	5	g	c	t	a	t	t	a	t	a	t	a	a	g	g	a	a	t	a	t	a	a	t	t	g	t	c	g	a	g	a	t	t	t	a	c	t	t	a	g	c	t	t	t	a	g	c	g	a	g	g	g	g	a	t	c	a	a	g	a	a	t	c	c	c	a	g	g
MH709338	5	g	c	t	a	t	t	a	t	a	t	a	a	g	g	a	a	t	a	t	a	a	t	t	g	t	c	g	a	g	a	t	t	t	a	c	t	t	a	g	c	t	t	t	a	g	c	g	a	g	g	g	g	a	t	c	a	a	g	a	a	t	c	c	c	a	g	g
MH709340	5	g	c	t	a	t	t	a	t	a	t	a	a	g	g	a	a	t	a	t	a	a	t	t	g	t	c	g	a	g	a	t	t	t	a	c	t	t	a	g	c	t	t	t	a	g	c	g	a	g	g	g	g	a	t	c	a	a	g	a	a	t	c	c	c	a	g	g
MH709342	5	g	c	t	a	t	t	a	t	a	t	a	a	g	g	a	a	t	a	t	a	a	t	t	g	t	c	g	a	g	a	t	t	t	a	c	t	t	a	g	c	t	t	t	a	g	c	g	a	g	g	g	g	a	t	c	a	a	g	a	a	t	c	c	c	a	g	g
MH709343	5	g	c	t	a	t	t	a	t	a	t	a	a	g	g	a	a	t	a	t	a	a	t	t	g	t	c	g	a	g	a	t	t	t	a	c	t	t	a	g	c	t	t	t	a	g	c	g	a	g	g	g	g	a	t	c	a	a	g	a	a	t	c	t	c	a	g	g
MH709345	5	g	c	t	a	t	t	a	t	a	t	a	a	t	g	a	a	t	a	t	a	a	t	t	g	t	c	g	a	g	a	t	t	t	a	c	t	t	a	g	c	t	t	t	a	g	c	g	a	g	g	g	g	a	t	c	a	a	g	a	a	t	c	c	c	a	g	g
MH709347	5	g	c	t	a	t	t	a	t	a	t	a	a	g	g	a	a	t	a	t	a	a	t	t	g	t	c	g	a	g	a	t	t	t	a	c	t	t	a	g	c	t	t	t	a	g	c	g	a	g	g	g	g	a	t	c	a	a	g	a	a	t	c	c	c	a	g	g
MH709349	5	g	c	t	a	t	t	a	t	a	t	a	a	g	g	a	a	t	a	t	a	a	t	t	g	t	c	g	a	g	a	t	t	t	a	c	t	t	a	g	c	t	t	t	a	g	c	g	a	g	g	g	g	a	t	c	a	a	g	a	a	t	c	c	c	a	g	g
MH709350	5	g	c	t	a	t	t	a	t	a	t	a	a	g	g	a	a	t	a	t	a	a	t	t	g	t	c	g	a	g	a	t	t	t	a	c	t	t	a	g	c	t	t	t	a	g	c	g	a	g	g	g	g	a	t	c	a	a	g	a	a	t	c	c	c	a	g	g
MH709351	5	g	c	t	a	t	t	a	t	a	t	a	a	g	g	a	a	t	a	t	a	a	t	t	g	t	c	g	a	g	a	t	t	t	a	c	t	t	a	g	c	t	t	t	a	g	c	g	a	g	g	g	g	a	t	c	a	a	g	a	a	t	t	c	c	a	g	g
MH709353	5	g	c	t	a	t	t	a	t	a	t	a	a	g	g	a	a	t	a	t	a	a	t	t	g	t	c	g	a	g	a	t	t	t	a	c	t	t	a	g	c	t	t	t	a	g	c	g	a	g	g	g	g	a	t	c	a	a	g	a	a	t	c	c	c	a	g	g
MH709358	5	g	c	t	a	t	t	a	t	a	t	a	a	g	g	a	a	t	a	t	a	a	t	t	g	t	c	g	a	g	a	t	t	t	a	c	t	t	a	g	c	t	t	t	a	g	c	g	a	g	g	g	g	a	t	c	a	a	g	a	a	t	c	c	c	a	g	g
MH709360	5	g	c	t	a	t	t	a	t	a	t	a	a	g	g	a	a	t	a	t	a	a	t	t	g	t	c	g	a	g	a	t	t	t	a	c	t	t	a	g	c	t	t	t	a	g	c	g	a	g	g	g	g	a	t	t	a	a	g	a	a	t	c	c	c	a	g	g
MH709362	5	g	c	t	a	t	t	a	t	a	t	a	a	t	g	a	a	t	a	t	a	a	t	t	g	t	c	g	a	g	a	t	t	t	a	c	t	t	a	g	c	t	t	t	a	g	c	g	a	g	g	g	g	a	t	c	a	a	g	a	a	t	c	c	c	a	g	g
MH709365	5	g	c	t	a	t	t	a	t	a	t	a	a	g	g	a	a	t	a	t	a	a	t	t	g	t	c	g	a	g	a	t	t	t	a	c	t	t	a	g	c	t	t	t	a	g	c	g	a	g	g	g	g	a	t	c	a	a	g	a	a	t	c	c	c	a	g	g
MH709373	5	g	c	t	a	t	t	a	t	a	t	a	a	g	g	a	a	t	a	t	a	a	t	t	g	t	c	g	a	g	a	t	t	t	a	c	t	t	a	g	c	t	t	t	a	g	c	g	a	g	g	g	g	a	t	c	a	a	g	a	a	t	c	c	c	a	g	g
MH709374	5	g	c	t	a	t	t	a	t	a	t	a	a	g	g	a	a	t	a	t	a	a	t	t	g	t	c	g	a	g	a	t	t	t	a	c	t	t	a	g	c	t	t	t	a	g	c	g	a	g	g	g	g	a	t	c	a	a	g	a	a	t	c	c	c	a	g	g
KC112914	6	g	c	t	a	t	t	a	t	a	t	a	a	g	g	a	a	t	a	t	a	a	t	t	g	t	c	g	a	g	a	t	t	t	a	c	t	t	a	g	c	t	t	t	a	g	c	g	a	g	g	g	g	a	t	c	a	a	g	a	a	t	c	c	c	a	g	g
KP771914	6	g	c	t	a	t	t	a	t	a	t	a	a	g	g	a	a	t	a	t	a	a	t	t	g	t	c	g	a	g	a	t	t	t	a	c	t	t	a	g	c	t	t	t	a	g	c	g	a	g	g	g	g	a	t	c	a	a	g	a	a	t	c	c	c	a	g	g
KP702725	6	g	c	t	a	t	t	a	t	a	t	a	a	g	g	a	a	t	a	t	a	a	t	t	g	t	c	g	a	g	a	t	t	t	a	c	t	t	a	g	c	t	t	t	a	g	c	g	a	g	g	g	g	a	t	c	a	a	g	a	a	t	c	c	c	a	g	g
KP771890	9	g	c	t	a	t	t	a	t	a	c	a	a	g	g	a	a	t	a	t	a	a	t	t	g	t	c	g	a	g	t	t	t	t	a	c	t	t	a	g	c	t	t	t	a	g	c	g	a	g	g	g	g	a	t	c	a	a	g	a	a	t	c	c	c	a	g	g
JN704710	9	g	c	t	a	t	t	a	t	a	t	a	a	g	a	a	a	t	a	t	a	a	t	t	g	t	c	g	a	g	a	t	t	t	a	c	t	t	a	g	c	t	t	t	a	g	c	g	a	g	g	g	g	a	t	c	a	a	g	a	a	t	c	c	t	a	g	g
KY037798	9	g	c	t	a	t	t	a	t	a	c	a	a	g	g	a	a	t	a	t	a	a	t	t	g	t	c	g	a	g	a	t	t	t	a	c	t	t	a	g	c	t	t	t	a	g	c	g	a	g	g	g	g	a	t	c	a	a	g	a	a	t	c	c	c	a	g	g
MH709312	9	g	c	t	a	t	t	a	t	a	c	a	a	g	g	a	a	t	a	t	a	a	t	t	g	t	c	g	a	g	a	t	t	t	a	c	t	t	a	g	c	t	t	t	a	g	c	g	a	g	g	g	g	a	t	c	a	a	g	a	a	t	c	c	c	a	g	g
MH709352	9	g	c	t	a	t	t	a	t	a	c	a	a	g	g	a	a	t	a	t	a	a	t	t	g	t	c	g	a	g	a	t	t	t	a	c	t	t	a	g	c	t	t	t	a	g	c	g	a	g	g	g	g	a	t	c	a	a	g	a	a	t	c	c	c	a	g	g
AB097933	Vac	g	c	t	a	t	g	a	t	a	t	a	a	g	g	a	a	t	a	t	a	a	t	t	g	t	t	g	a	g	a	t	t	t	a	c	t	g	a	g	c	t	t	t	a	g	c	g	g	g	g	g	g	a	t	c	a	a	g	a	a	t	c	c	c	a	g	g
JN704698	Vac	g	c	t	a	t	g	a	t	a	t	a	a	g	g	a	a	t	a	t	a	a	t	t	g	t	t	g	g	g	a	t	t	t	a	c	t	g	a	g	c	t	t	t	a	g	c	g	g	g	g	g	g	a	t	c	a	a	g	a	a	t	c	c	c	a	g	g
JN704697	Vac	g	c	t	a	t	g	a	t	a	t	a	a	g	g	a	a	t	a	t	a	a	t	t	g	t	t	g	a	g	a	t	t	t	a	c	t	g	g	g	c	t	t	t	a	g	c	g	g	g	g	g	g	a	t	c	a	a	g	a	a	t	c	c	c	a	g	g
DQ008355	Vac	g	c	t	a	t	g	a	t	a	t	a	a	g	g	a	a	t	a	t	a	a	t	t	g	t	t	g	a	g	a	t	t	t	a	c	t	g	a	g	c	t	t	t	a	g	c	g	g	g	g	g	g	a	t	c	a	a	g	a	a	t	c	c	c	a	g	g
JF306641	Vac	g	c	t	a	t	g	a	t	a	t	a	a	g	g	a	a	t	a	t	a	a	t	t	g	t	t	g	a	g	a	t	t	t	a	c	t	g	a	g	c	t	t	t	a	g	c	g	g	g	g	g	g	a	t	c	a	a	g	a	a	t	c	c	c	a	g	g
AB097932	Vac	g	c	t	a	t	g	a	t	a	t	a	a	g	g	a	a	t	a	t	a	a	t	t	g	t	t	g	a	g	a	t	t	t	a	c	t	g	a	g	c	t	t	t	a	g	c	g	g	g	g	g	g	a	t	c	a	a	g	a	a	t	c	c	c	a	g	g
DQ008354	Vac	g	c	t	a	t	g	a	t	a	t	a	a	g	g	a	a	t	a	t	a	a	t	t	g	t	t	g	a	g	a	t	t	t	a	c	t	g	a	g	c	t	t	t	a	g	c	g	g	g	g	g	g	a	t	c	a	a	g	a	a	t	c	c	c	a	g	g
KU926312	Vac	g	c	t	a	t	g	a	t	a	t	a	a	g	g	a	a	t	a	t	a	a	t	t	g	t	t	g	a	g	a	t	t	t	a	c	t	g	a	g	c	t	t	t	a	g	c	g	g	g	g	g	g	a	t	c	a	a	g	a	a	t	c	c	c	a	g	g
KU926313	Vac	g	c	t	a	t	g	a	t	a	t	a	a	g	g	a	a	t	a	t	a	a	t	t	g	t	t	g	a	g	a	t	t	t	a	c	t	g	a	g	c	t	t	t	a	g	c	g	g	g	g	g	g	a	t	c	a	a	g	a	a	t	c	c	c	a	g	g
KU926314	Vac	g	c	t	a	t	g	a	t	a	t	a	a	g	g	a	a	t	a	t	a	a	t	t	g	t	t	g	a	g	a	t	t	t	a	c	t	g	a	g	c	t	t	t	a	g	c	g	g	g	g	g	g	a	t	c	a	a	g	a	a	t	c	c	c	a	g	g
KF558371	Vac	g	c	t	a	t	g	a	t	a	t	a	a	g	g	a	a	t	a	t	a	a	t	t	g	t	t	g	a	g	a	t	t	t	a	c	t	g	a	g	c	t	t	t	a	g	c	g	g	g	g	g	g	a	t	c	a	a	g	a	a	t	c	c	c	a	g	g
KF558372	Vac	g	c	t	a	t	g	a	t	a	t	a	a	g	g	a	a	t	a	t	a	a	t	t	g	t	t	g	a	g	a	t	t	t	a	c	t	g	a	g	c	t	t	t	a	g	c	g	g	g	g	g	g	a	t	c	a	a	g	a	a	t	c	c	c	a	g	g
KF558373	Vac	g	c	t	a	t	g	a	t	a	t	a	a	g	g	a	a	t	a	t	a	a	t	t	g	t	t	g	a	g	a	t	t	t	a	c	t	g	a	g	c	t	t	t	a	g	c	g	g	g	g	g	g	a	t	c	a	a	g	a	a	t	c	c	c	a	g	g
KF558374	Vac	g	c	t	a	t	g	a	t	a	t	a	a	g	g	a	a	t	a	t	a	a	t	t	g	c	t	g	a	g	a	t	t	t	a	c	t	g	a	g	c	t	t	t	a	g	c	g	g	g	g	g	g	a	t	c	a	a	g	a	a	t	c	c	c	a	g	g
KF558375	Vac	g	c	t	a	t	g	a	t	a	t	a	a	g	g	a	a	t	a	t	a	a	t	t	g	t	t	g	a	t	a	a	c	g	t	g	t	g	a	g	a	t	c	g	c	g	c	g	g	g	g	g	g	a	t	c	g	g	c	a	a	t	c	c	c	a	g	g
KF558376	Vac	g	c	t	a	t	g	a	t	a	t	a	a	g	g	a	a	t	a	t	a	a	t	t	g	t	t	g	a	g	a	t	t	t	a	c	t	g	a	g	c	t	t	t	a	g	c	g	g	g	g	g	g	a	t	c	a	a	g	a	a	t	c	c	c	a	g	g
KF558377	Vac	g	c	t	a	t	g	a	t	a	t	a	a	g	g	a	a	t	a	t	a	a	t	t	g	t	t	g	a	g	a	t	t	t	a	c	t	g	a	g	c	t	t	t	a	g	c	g	g	g	g	g	g	a	t	c	a	a	g	a	a	t	c	c	c	a	g	g
KF558378	Vac	g	c	t	a	t	g	a	t	a	t	a	a	g	g	a	a	t	a	t	a	a	t	t	g	t	t	g	a	g	t	t	t	t	a	c	t	g	a	g	c	t	t	t	a	g	c	g	g	g	g	g	g	a	t	c	a	a	g	a	a	t	c	c	c	a	g	g
KF558379	Vac	g	c	t	a	t	g	a	t	a	t	a	a	g	g	a	a	t	a	t	a	a	t	t	g	t	t	g	a	g	a	t	t	t	a	c	t	g	a	g	c	t	t	t	a	g	c	g	g	g	g	g	g	a	t	c	a	a	g	a	a	t	c	c	c	a	g	g
KF558380	Vac	g	c	t	a	t	g	a	t	a	t	a	a	g	g	a	a	t	a	t	a	a	t	t	g	t	t	g	a	g	a	t	t	t	a	c	t	g	a	g	c	t	t	t	a	g	c	g	g	g	g	g	g	a	t	c	a	a	g	a	a	t	c	c	c	a	g	g
KF558381	Vac	g	c	t	a	t	g	a	t	a	t	a	a	g	g	a	a	t	a	t	a	a	t	t	g	t	t	g	a	g	a	t	t	t	a	c	t	g	a	g	c	t	t	t	a	g	c	g	g	g	g	g	g	a	t	c	a	a	g	a	a	t	c	c	c	a	g	g
KF558382	Vac	g	c	t	a	t	g	a	t	a	t	a	a	g	g	a	a	t	a	t	a	a	t	t	g	t	t	g	a	g	a	t	t	t	a	c	t	g	a	g	c	t	t	t	a	g	c	g	g	g	g	g	g	a	t	c	a	a	g	a	a	t	c	c	c	a	g	g
KF558383	Vac	g	c	t	a	t	g	a	t	a	t	a	a	g	g	a	a	t	a	t	a	a	t	t	g	t	t	g	a	g	a	t	t	t	a	c	t	g	a	g	c	t	t	t	a	g	c	g	g	g	g	g	g	a	t	c	a	a	g	a	a	t	c	c	c	a	g	g
KF558384	Vac	g	c	t	a	t	g	a	t	a	t	a	a	g	g	a	a	t	a	t	a	a	t	t	g	t	t	g	a	g	a	t	t	t	a	c	t	g	a	g	c	t	t	t	a	g	c	g	g	g	g	g	g	a	t	c	a	a	g	a	a	t	c	c	c	a	g	g
KF558385	Vac	g	c	t	a	t	g	a	t	a	t	a	a	g	g	a	a	t	a	t	a	a	t	t	g	t	t	g	a	g	a	t	t	t	a	c	t	g	a	g	c	t	t	t	a	g	c	g	g	g	g	g	g	a	t	c	a	a	g	a	a	t	c	c	c	a	g	g
KF558386	Vac	g	c	t	a	t	g	a	t	a	t	a	a	g	g	a	a	t	a	t	a	a	t	t	g	t	t	g	a	g	a	t	t	t	a	c	t	g	a	g	c	t	t	t	a	g	c	g	g	g	g	g	t	a	t	c	a	a	g	a	a	t	c	c	c	a	g	g
KF558387	Vac	g	c	t	a	t	g	a	t	a	t	a	a	g	g	a	a	t	a	t	a	a	t	t	g	t	t	g	a	g	a	t	t	t	a	c	t	g	a	g	c	t	t	t	a	g	c	g	g	g	g	g	g	a	t	c	a	a	g	a	a	t	c	c	c	a	g	g
KF558388	Vac	g	c	t	a	t	g	a	t	a	t	a	a	g	g	a	a	t	a	t	a	a	t	t	g	t	t	g	a	g	a	t	t	t	a	c	t	g	a	g	c	t	t	t	a	g	c	g	g	g	g	g	g	a	t	c	a	a	g	a	a	t	c	c	c	a	g	g
KF558389	Vac	g	c	t	a	t	g	a	t	a	t	a	a	g	g	a	a	t	a	t	a	a	t	t	g	c	t	g	a	g	a	t	t	t	a	c	t	g	a	g	c	t	t	t	a	g	c	g	g	g	g	g	g	a	t	c	a	a	g	a	a	t	c	c	c	a	g	g
KF558390	Vac	g	c	t	a	t	g	a	t	a	t	a	a	g	g	a	a	t	a	t	a	a	t	t	g	t	t	g	a	g	a	t	t	t	a	c	t	g	a	g	c	t	t	t	a	g	c	g	g	g	g	g	g	a	t	c	a	a	g	a	a	t	c	c	c	a	g	g
KF558391	Vac	g	c	t	a	t	g	a	t	a	t	a	a	g	g	a	a	t	a	t	a	a	t	t	g	t	t	g	a	g	a	t	t	t	a	c	t	g	a	g	c	t	t	t	a	g	c	g	g	g	g	g	g	a	t	c	a	a	g	a	a	t	c	c	c	a	g	g
KF811485	Vac	g	c	t	a	t	g	a	t	a	t	a	a	g	g	a	a	t	a	t	a	a	t	t	g	t	t	g	a	g	a	t	t	t	a	c	t	g	a	g	c	t	t	t	a	g	c	g	g	g	g	g	g	a	t	c	a	a	g	a	a	t	c	c	c	a	g	g
KF853225	Vac	g	c	t	a	t	g	a	t	a	t	a	a	g	g	a	a	t	a	t	a	a	t	t	g	t	t	g	a	g	a	t	t	t	a	c	t	g	a	g	c	t	t	t	a	g	c	g	g	g	g	g	g	a	t	c	a	a	g	a	a	t	c	c	c	a	g	g
KF853226	Vac	g	c	t	a	t	g	a	t	a	t	a	a	g	g	a	a	t	a	t	a	a	t	t	g	c	t	g	a	g	a	t	t	t	a	c	t	g	a	g	c	t	t	t	a	g	c	g	g	g	g	g	g	a	t	c	a	a	g	a	a	t	c	c	c	a	g	g
KF853227	Vac	g	c	t	a	t	g	a	t	a	t	a	a	g	g	a	a	t	a	t	a	a	t	t	g	t	t	g	a	g	a	t	t	t	a	c	t	g	a	g	c	t	t	t	a	g	c	g	g	g	g	g	g	a	t	c	a	a	g	a	a	t	c	c	c	a	a	g
KF853228	Vac	g	c	t	a	t	g	a	t	a	t	a	a	g	g	a	a	t	a	t	a	a	t	t	g	t	t	g	a	g	a	t	t	t	a	c	t	g	a	g	c	t	t	t	a	g	c	g	g	g	g	g	g	a	t	c	a	a	g	a	a	t	c	c	c	a	g	g
KF853229	Vac	g	c	t	a	t	g	a	t	a	t	a	a	g	g	a	a	t	a	t	a	a	t	t	g	t	t	g	a	g	a	t	t	t	a	c	t	g	a	g	c	t	t	t	a	g	c	g	g	g	g	g	g	a	t	c	a	a	g	a	a	t	c	c	c	a	g	g
KF853230	Vac	g	c	t	a	t	g	a	t	a	t	a	a	g	g	a	a	t	a	t	a	a	t	t	g	t	t	g	a	g	a	t	t	t	a	c	t	g	a	g	c	t	t	t	a	g	c	g	g	g	g	g	g	a	t	c	a	a	g	a	a	t	c	c	c	a	g	g
KF853231	Vac	g	c	t	a	t	g	a	t	a	t	a	a	g	g	a	a	t	a	t	a	a	t	t	g	t	t	g	a	g	a	t	t	t	a	c	t	g	a	g	c	t	t	t	a	g	c	g	g	g	g	g	t	a	t	c	a	a	g	a	a	t	c	c	c	a	g	g
KF853232	Vac	g	c	t	a	t	g	a	t	a	t	a	a	g	g	a	a	t	a	t	a	a	t	t	g	t	t	g	a	g	a	t	t	t	a	c	t	g	a	g	c	t	t	t	a	g	c	g	g	g	g	g	g	a	t	c	a	a	g	a	a	t	c	c	c	a	g	g
KF853233	Vac	g	c	t	a	t	g	a	t	a	t	a	a	g	g	a	a	t	a	t	a	a	t	t	g	t	t	g	a	g	a	t	t	t	a	c	t	g	a	g	c	t	t	t	a	g	c	g	g	g	g	g	g	a	t	c	a	a	g	a	a	t	c	c	c	a	g	g
KF853234	Vac	g	c	t	a	t	g	a	t	a	t	a	a	g	g	a	a	t	a	t	a	a	t	t	g	t	t	g	a	g	a	t	t	t	a	c	t	g	a	g	c	t	t	t	a	g	c	g	g	g	g	g	g	a	t	c	a	a	g	a	a	t	c	c	c	a	g	g
KF853235	Vac	g	c	t	a	t	g	a	t	a	t	a	a	g	g	a	a	t	a	t	a	a	t	t	g	t	t	g	a	g	t	t	t	t	a	c	t	g	a	g	c	t	t	t	a	g	c	g	g	g	g	g	g	a	t	c	a	a	g	a	a	t	c	c	c	a	g	g
MF898328	Vac	g	c	t	a	t	g	a	t	a	t	a	a	g	g	a	a	t	a	t	a	a	t	t	g	y	t	g	a	g	a	t	t	t	a	c	t	g	a	g	c	t	t	t	a	g	c	g	g	g	g	g	g	a	t	c	a	a	g	a	a	t	c	c	c	a	g	g
JN704709	VIII	g	c	t	a	t	t	a	t	a	t	a	a	g	g	a	a	t	a	t	a	a	t	t	g	t	c	g	a	g	a	t	t	t	a	c	t	t	a	g	c	t	t	t	a	g	c	g	a	t	g	g	g	a	t	c	a	a	g	a	a	t	c	c	c	a	g	g

Supplementary Table 3: 2880 SN

GB-ID	Clade markers														2		9																																															
	Position in Dumas	100812	100887	100970	101071	101089	101166	101198	101218	101236	101243	101245	101260	101327	101331	101334	101353	101366	101425	101459	101464	101515	101619	101620	101626	101627	101652	101697	101706	101720	101768	101785	101806	101807	101808	101830	101847	101875	101883	101886	101900	101902	101937	101944	101949	101991	102002	102020	102021	102021_1	102037	102051	102054	102060	102062	102067	102080	102144	102179	102192	102203	102232	102268	102309
AY548170	1	t	g	t	a	a	c	t	a	g	g	a	t	t	c	g	t	t	a	g	c	c	c	t	c	a	c	a	a	a	t	t	a	a	t	c	a	a	a	t	c	t	t	g	c	c	t	a	c	-	t	c	c	g	c	a	a	c	a	g	g	c	t	c
AY548171	1	t	g	t	a	a	c	t	a	g	g	a	t	t	c	g	t	t	a	g	c	c	c	t	c	a	c	a	a	a	t	t	a	a	t	c	a	a	a	t	c	t	t	g	c	t	t	a	c	-	t	c	c	g	c	a	a	c	a	g	g	c	t	c
DQ479953	1	t	g	t	a	a	c	t	a	g	g	a	t	t	c	g	t	t	a	g	c	c	c	t	c	a	c	a	a	a	t	t	a	a	t	c	a	a	a	t	c	t	t	g	c	c	t	a	c	-	t	c	c	g	c	a	a	c	a	g	g	c	t	c
DQ479954	1	t	g	t	a	a	c	t	a	g	g	a	t	t	c	g	t	t	a	g	c	c	c	t	c	a	c	a	a	a	t	t	a	a	t	c	a	a	a	t	c	t	t	g	c	c	t	a	c	-	t	c	c	g	c	a	a	c	a	g	g	c	t	c
DQ479958	1	t	g	t	a	a	c	t	a	a	g	a	t	t	c	g	t	t	a	g	c	c	c	t	c	a	c	a	a	a	t	t	a	a	t	c	a	a	a	t	c	t	t	g	c	t	t	a	c	-	t	a	c	g	c	a	a	c	a	g	g	c	t	c
DQ479959	1	t	g	t	a	a	c	t	a	a	g	a	t	t	c	g	t	t	a	g	c	c	c	t	c	a	c	a	a	a	t	t	a	a	t	c	a	a	a	t	c	t	t	g	c	t	t	a	c	-	t	a	c	g	c	a	a	c	a	g	g	c	t	c
DQ479961	1	t	g	t	a	a	c	t	a	g	g	a	t	t	c	g	t	t	a	g	c	c	c	t	c	a	c	a	a	a	t	t	a	a	t	c	a	a	a	t	c	t	t	g	c	c	t	a	c	-	t	c	c	g	c	a	a	c	a	g	g	c	t	c
DQ479962	1	t	g	t	a	a	c	t	a	g	g	a	t	t	c	g	t	t	a	g	c	c	c	t	c	a	c	a	a	a	t	t	a	a	t	c	a	a	a	t	c	t	t	g	c	c	t	a	c	-	t	c	c	g	c	a	a	c	a	g	g	c	t	c
DQ479963	1	t	g	t	a	a	c	t	a	g	g	a	t	t	c	g	t	t	a	g	c	c	c	t	c	a	c	a	a	a	t	t	a	a	t	c	a	a	a	t	c	t	t	g	c	c	t	a	c	-	t	c	c	g	c	a	a	c	a	g	g	c	t	c
DQ674250	1	t	g	t	a	a	c	t	a	g	g	a	t	t	c	g	t	t	a	g	c	c	c	t	c	a	c	a	a	a	t	t	a	a	t	c	a	a	a	t	c	t	t	g	c	t	t	a	c	-	t	c	c	g	c	a	a	c	a	g	g	c	t	c
KX262864	1	t	g	t	a	a	c	t	a	g	g	a	t	t	c	g	t	t	a	g	c	c	c	t	c	a	c	a	a	a	t	t	a	a	t	c	a	a	a	t	c	t	t	g	c	t	t	n	n	-	n	n	n	n	n	n	n	n	n	n	n	c	t	c
KX262865	1	t	g	t	a	a	c	t	a	g	g	a	t	t	c	a	t	t	a	g	c	c	c	t	c	a	c	a	a	a	t	t	a	a	t	c	a	a	a	t	c	t	t	g	c	t	t	n	n	-	n	n	n	n	n	n	n	n	n	n	n	c	t	c
KX262866	1	t	g	t	a	a	c	t	a	g	g	a	t	t	c	g	t	t	a	g	c	c	c	t	c	a	c	a	a	a	t	t	a	a	t	c	a	a	a	t	c	t	t	g	c	t	t	n	n	-	n	n	n	n	n	n	n	n	n	n	n	c	t	c
EU154348	1	t	g	t	a	a	c	t	a	g	g	a	t	t	c	g	t	t	g	g	c	c	c	t	c	a	c	a	a	a	t	t	a	a	t	c	a	a	a	t	c	t	t	g	c	c	t	a	c	-	t	c	c	g	c	a	a	c	a	g	g	c	t	c
JN704690	1	c	g	t	a	a	c	t	a	g	g	a	t	t	c	g	t	t	a	g	c	c	c	t	c	a	c	a	a	a	t	t	a	a	t	c	a	a	a	t	c	t	t	g	c	c	t	a	c	-	t	c	c	g	c	a	a	c	a	g	a	c	t	c
JN704691	1	t	g	t	a	a	c	t	a	g	g	a	t	t	c	g	t	t	a	g	c	c	c	t	c	a	c	a	a	a	t	t	a	a	t	c	a	a	a	t	c	t	t	g	c	c	t	a	c	-	t	c	c	g	c	a	a	c	a	g	a	c	t	c
JN704692	1	t	g	t	a	a	c	t	a	g	g	a	t	t	c	g	t	t	a	g	c	c	c	t	c	a	c	a	a	a	g	t	a	a	t	c	a	a	a	t	c	t	t	g	c	c	t	a	c	-	t	c	c	g	c	a	a	t	a	g	a	c	t	c
JN704693	1	t	g	t	a	a	c	t	a	g	g	a	t	t	c	g	t	t	a	g	c	c	c	t	c	a	c	a	a	a	t	t	a	a	t	c	a	a	g	t	c	t	t	g	c	c	t	a	c	-	t	c	c	g	c	a	a	c	a	g	g	c	t	c
JN704694	1	t	g	t	a	a	c	t	a	g	g	a	t	t	c	g	t	t	a	g	c	c	c	t	c	a	c	a	a	a	t	t	a	a	t	c	a	a	a	t	c	t	t	g	c	c	t	a	c	-	t	c	c	g	c	a	a	c	a	g	g	c	t	c
JN704695	1	t	g	t	a	a	c	t	a	g	g	a	t	t	c	g	t	t	a	g	c	c	c	t	c	a	c	a	a	a	t	t	a	a	t	c	a	a	a	t	c	t	t	g	c	c	t	a	c	-	t	c	c	g	c	a	a	c	a	g	a	c	t	c
JN704696	1	t	g	t	a	a	c	t	a	g	g	a	t	t	c	g	t	t	a	g	c	c	c	t	c	a	c	a	a	a	t	t	a	a	t	c	a	a	a	t	c	t	t	g	c	c	t	a	c	-	t	c	c	g	c	a	a	c	a	g	g	c	t	c
NC001348	1	t	g	t	a	a	c	t	a	g	g	a	t	t	c	g	t	t	a	g	c	c	c	t	c	a	c	a	a	a	t	t	a	a	t	c	a	a	a	t	c	t	t	g	c	c	t	a	c	-	t	c	c	g	c	a	a	c	a	g	a	c	t	c
KP771892	1	t	g	t	a	a	c	t	a	g	g	a	t	t	c	g	t	t	a	g	c	c	c	t	c	a	c	a	a	a	t	t	a	a	t	c	a	a	a	t	c	t	t	g	c	c	t	n	n	-	n	n	n	n	n	n	n	n	n	n	n	c	t	c
KP771894	1	t	g	t	a	a	c	t	a	g	g	a	t	t	c	g	t	t	a	g	c	c	c	t	c	a	c	a	a	a	t	t	a	a	t	c	a	a	a	t	c	t	t	g	c	c	t	n	n	-	n	n	n	n	n	n	n	n	n	n	n	c	t	c
KP771900	1	t	g	t	a	a	c	t	a	g	g	a	t	t	c	g	t	t	a	g	c	c	c	t	c	a	c	a	a	a	t	t	a	a	t	c	a	a	a	t	c	t	t	g	c	c	t	n	n	-	n	n	n	n	n	n	n	n	n	n	n	c	t	c
KP771901	1	t	g	t	a	a	c	t	a	g	g	a	t	t	c	g	t	t	a	g	c	c	c	t	c	a	c	a	a	a	t	t	a	a	t	c	a	a	a	t	c	t	t	g	c	c	t	n	n	-	n	n	n	n	n	n	n	n	n	n	n	c	t	c
KP771902	1	t	g	t	a	a	c	t	a	g	g	a	t	t	c	g	t	t	a	g	c	c	c	t	c	a	c	a	a	a	t	t	a	a	t	c	a	a	a	t	c	t	t	g	c	c	t	n	n	-	n	n	n	n	n	n	n	n	n	n	n	c	t	c
KP771908	1	t	g	t	a	a	c	t	a	g	g	a	t	t	c	g	t	t	a	g	c	c	c	t	c	a	c	a	a	a	t	t	a	a	t	c	a	a	a	t	c	t	t	g	c	t	t	n	n	-	n	n	n	n	n	n	n	n	n	n	n	c	t	c
KP771912	1	t	g	t	a	a	c	t	a	g	g	a	t	t	c	g	t	t	a	g	c	c	c	t	c	a	c	a	a	a	t	t	a	a	t	c	a	a	a	t	c	t	t	g	c	t	t	n	n	-	n	n	n	n	n	n	n	n	n	n	n	c	t	c
KP771913	1	t	g	t	a	a	c	t	a	g	g	a	t	t	c	g	t	t	a	g	c	c	c	t	c	a	c	a	a	a	t	t	a	a	t	c	a	a	a	t	c	t	t	g	c	t	t	n	n	-	n	n	n	n	n	n	n	n	n	n	n	c	t	c
KP771917	1	t	g	t	a	a	c	t	a	g	g	a	t	t	c	g	t	t	a	g	c	c	c	t	c	a	c	a	a	a	t	t	a	a	t	c	a	c	a	t	c	t	t	g	c	c	t	n	n	-	n	n	n	n	n	n	n	n	n	n	n	c	t	c
KP771918	1	t	g	t	a	a	c	t	a	g	g	a	t	t	c	g	t	t	a	g	c	c	c	t	c	a	c	a	a	a	t	t	a	a	t	c	a	a	a	t	t	t	t	g	c	c	t	n	n	-	n	n	n	n	n	n	n	n	n	n	n	c	t	c
KP771920	1	t	g	t	a	a	c	t	a	g	g	a	t	t	c	g	t	t	a	g	c	c	c	t	c	a	c	a	a	a	t	t	a	a	t	c	a	a	a	t	c	t	t	g	c	c	t	n	n	-	n	n	n	n	n	n	n	n	n	n	n	c	t	c
MH709361	1	t	g	t	a	a	c	t	a	g	g	a	t	t	c	g	t	t	a	g	c	c	c	t	c	a	c	a	a	a	t	t	a	a	t	c	a	a	a	t	c	t	t	g	c	t	t	a	c	a	t	c	c	g	c	a	a	c	a	a	g	c	t	c
KU926318	2	t	g	c	a	a	c	t	g	g	g	a	t	c	t	g	t	t	a	g	c	c	c	t	c	a	c	a	g	g	t	t	a	a	t	c	a	a	a	c	c	t	t	g	c	t	t	a	c	a	t	c	c	g	c	a	a	c	a	a	g	c	t	a
KU926319	2	t	g	t	a	a	c	t	a	g	g	a	t	t	t	g	t	t	a	g	c	c	c	t	c	a	c	a	a	a	t	t	a	a	t	c	a	a	a	c	c	t	t	g	c	t	t	a	c	a	t	c	c	g	c	a	a	c	a	a	g	c	t	a
KU926320	2	t	g	t	a	a	c	t	a	g	g	a	t	t	t	g	t	a	a	g	c	c	c	t	c	a	c	a	a	a	t	t	a	a	t	c	a	a	a	c	c	t	t	g	c	t	t	a	c	a	t	c	c	g	c	a	a	c	a	a	g	c	t	a
KU926321	2	t	g	t	a	a	c	t	a	g	g	a	t	t	t	g	t	t	a	g	c	c	c	t	c	a	c	a	a	a	t	t	a	a	t	c	a	a	a	c	c	t	t	g	c	t	t	a	c	a	t	c	c	g	c	a	a	c	a	a	g	c	t	a
KU926322	2	t	g	t	a	a	c	t	a	g	g	a	t	t	t	g	t	t	a	g	c	c	c	t	c	a	c	a	a	a	t	t	a	a	t	c	a	a	a	c	c	t	t	g	c	t	t	a	c	a	t	c	c	g	c	a	a	c	a	a	g	c	t	a
KC847290	2	t	g	t	a	a	c	t	a	g	g	a	t	t	t	g	t	t	a	g	c	c	c	t	c	a	c	a	a	a	t	t	a	a	t	c	a	a	a	c	c	t	t	g	c	t	t	a	c	-	t	c	c	g	c	a	a	c	a	a	g	c	t	a
KJ767491	2	t	g	t	a	a	c	t	a	g	g	a	t	t	t	g	t	t	a	g	c	c	c	t	c	a	c	a	a	a	t	t	a	a	t	c	a	a	a	c	c	t	t	g	c	t	t	a	c	a	t	c	c	g	c	a	a	c	a	a	g	c	t	a
KJ767492	2	t	g	t	a	a	c	t	a	g	g	a	t	t	t	g	t	t	a	g	c	c	c	t	c	a	c	a	a	a	t	t	a	a	t	c	a	a	a	c	c	t	t	g	c	t	t	a	c	a	t	c	c	g	c	a	a	c	a	a	g	c	t	a
KJ808816	2	t	g	t	a	a	c	t	a	g	g	a	t	t	t	g	t	t	a	g	c	c	c	t	c	a	c	a	a	a	t	t	a	a	t	c	a	a	a	c	c	t	t	g	c	t	t	a	c	a	t	c	c	g	c	a	a	c	a	a	g	c	t	a
KP771925	2	t	g	t	a	a	c	t	a	g	g	a	t	t	t	g	t	t	a	g	c	c	c	t	c	a	c	a	a	a	t	t	a	a	t	c	a	a	a	c	c	t	t	g	c	t	t	n	n	-	n	n	n	n	n	n	n	n	n	n	n	c	t	a
KU926315	2	t	g	t	a	a	c	t	a	g	g	a	t	t	t	g	t	t	a	g	c	c	c	t	c	a	c	a	a	a	t	t	a	a	t	c	a	a	a	c	c	t	t	g	c	t	t	a	c	a	t	c	c	g	c	a	a	c	a	a	g	c	t	a
KU926316	2	t	g	t	a	a	c	t	a	g	g	a	t	t	t	g	t	t	a	g	c	c	c	t	c	a	c	a	a	a	t	t	a	a	t	c	a	a	a	c	c	t	t	g	c	t	t	a	c	a	t	c	c	g	c	a	a	c	a	a	g	c	t	a
KU926317	2	t	g	t	a	a	c	t	a	g	g	a	t	t	t	g	t	t	a	g	c	c	c	t	c	a	c	a	a	a	t	t	a	a	t	c	a	a	a	c	c	t	t	g	c	t	t	a	c	a	t	c	c	g	c	a	a	c	a	a	g	c	t	a
KY037796	2	t	g	t	a	a	c	t	a	g	g	a	t	t	t	g	t	t	a	g	c	c	c	t	c	a	c	a	a	a	t	t	a	a	t	c	a	a	a	c	c	t	t	g	c	t	t	a	c	a	t	c	c	g	c	a	a	c	a	a	g	c	t	a
MF004348	2	t	g	t	a	a	c	t	a	g	g	a	t	t	t	g	t	t	a	g	c	c	c	t	c	a	c	a	a	a	t	t	a	a	t	c	a	a	a	c	c	t	t	g	c	t	t	a	c	a	t	c	c	g	c	a	a	c	a	a	g	c	t	a
MH709309	2	t	g	t	a	a	c	t	a	g	g	a	t	t	t	g	t	t	a	g	c	c	c	t	c	a	c	g	a	a	t	t	a	a	t	c	a	a	a	c	c	t	t	g	c	t	t	a	c	a	t	c	c	g	t	a	a	c	a	a	g	c	t	a
MH709310	2	t	g	t	a	a	c	t	a	g	g	a	t	t	t	g	t	t	a	g	c	c	c	t	c	a	c	a	a	a	t	t	a	a	t	c	a	a	a	c	c	t	t	g	c	t	t	a	c	-	t	c	c	g	c	a	a	c	a	a	g	c	t	a
MH709311	2	t	g	t	a	a	c	t	a	g	g	a	t	t	t	g	t	t	a	g	c	c	c	t	c	a	c	a	a	a	t	t	a	a	t	c	a	a	a	c	c	t	t	g	c	t	t	a	c	a	t	c	c	g	c	a	a	c	a	a	g	c	t	a
MH709313	2	t	g	t	a	a	c	t	a	g	g	a	t	t	t	g	t	t	a	g	c	c	c	t	c	a	c	a	a	a	t	t	a	a	t	c	a	a	a	c	c	t	t	g	c	t	t	a	c	-	t	c	c	g	c	a	a	c	a	a	g	c	t	a
MH709314	2	t	g	t	a	a	c	t	a	g	g	a	t	t	t	g	t	t	a	g	c	c	c	t	c	a	c	a	a	a	t	t	a	a	t	c	a	a	a	c	c	t	t	g	c	t	t	a	c	a	t	c	c	g	c	a	a	c	a	a	g	c	t	a
MH709315	2	t	g	t	a	a	c	t	a	g	g	a	t	t	t	g	t	t	a	g	c	c	c	t	c	a	c	a	a	a	t	g	a	a	t	c	a	a	a	c	c	t	t	g	c	t	t	a	c	a	t	c	c	g	c	a	a	c	a	a	g	c	t	a
MH709319	2	t	g	t	a	a	c	t	a	g	g	a	t	t	t	g	t	t	a	g	c	c	c	t	c	a	c	a	a	a	t	t	a	a	t	c	a	a	a	c	c	t	t	g	c	t	t	a	c	a	t	c	c	g	c	a	a	c	a	a	g	c	t	a
MH709320	2	t	g	t	a	a	c	t	a	g	g	a	t	t	t	g	t	t	a	g	c	c	c	t	c	a	c	a	a	a	t	t	a	a	t	c	a	a	a	c	c	t	t	g	c	t	t	a	c	a	t	c	c	g	c	a	a	c	a	a	g	c	t	a
MH709329	2	t	g	t	a	a	c	t	a	g	g	a	t	t	t	g	t	t	a	g	c	c	c	t	c	a	c	a	a	a	t	t	a	a	t	c	a	a	a	c	c	t	t	g	c	t	t	a	c	-	t	c	c	g	c	a	a	c	a	a	g	c	t	a
MH709331	2	t	g	t	a	a	c	t	a	g	g	a	t	t	t	g	t	t	a	g	c	c	c	t	c	a	c	a	a	a	t	t	a	a	t	c	a	a	a	c	c	t	t	g	c	t	t	a	c	a	t	c	c	g	c	a	a	c	a	a	g	c	t	a
MH709333	2	t	g	t	a	a	c	t	a	g	g	a	t	t	t	g	t	t	a	g	c	c	c	t	c	a	c	a	a	a	t	t	a	a	t	c	a	a	a	c	c	t	t	g	c	t	t	a	c	a	t	c	c	g	c	a	a	c	a	a	g	c	t	a
MH709337	2	t	g	t	a	a	c	t	a	g	g	a	t	t	t	g	t	t	a	g	c	c	c	t	c	a	c	a	a	a	t	t	a	a	t	c	a	a	a	c	c	t	t	g	c	t	t	a	c	a	t	c	c	g	c	a	a	c	a	a	g	c	t	a
MH709339	2	t	g	t	a	a	c	t	a	g	g	a	t	t	t	g	t	t	a	g	c	c	c	t	c	a	c	a	a	a	t	t	a	a	t	c	a	a	a	c	c	t	t	g	c	t	t	a	c	a	t	c	c	g	c	a	a	c	a	a	g	c	t	a
MH709344	2	t	g	t	a	a	c	t	a	g	g	a	t	t	t	g	t	t	a	g	c	c	c	t	c	a	c	a	a	a	t	t	a	a	t	c	a	a	a	c	c	t	t	g	c	t	t	a	c	a	t	c	c	g	c	a	a	c	a	a	g	c	t	a
MH709348	2	t	g	t	a	a	c	t	a	g	g	a	t	t	t	g	t	t	a	g	c	c	c	t	c	a	c	a	a	a	t	t	a	a	t	c	a	a	a	c	c	t	t	g	c	t	t	a	c	c	t	c	c	g	c	c	a	c	c	a	g	c	t	a
MH709355	2	t	g	t	a	a	c	t	a	g	g	g	t	t	t	g	t	t	a	g	c	c	c	t	c	a	c	a	a	a	t	t	a	a	t	c	a	a	a	c	c	t	t	g	c	t	t	a	c	a	t	c	c	g	c	a	a	c	a	a	g	c	t	a
MH709359	2	t	g	t	a	a	c	t	a	g	g	a	t	t	t	g	t	t	a	g	c	c	c	t	c	a	c	a	a	a	t	t	a	a	t	c	a	a	a	c	c	t	t	g	c	t	t	a	c	a	t	c	c	g	c	a	a	c	a	a	g	c	t	a
MH709363	2	t	g	t	a	a	c	t	a	g	g	a	t	t	t	g	t	t	a	g	c	c	c	c	c	a	c	a	a	a	t	t	a	a	t	c	a	a	a	c	c	t	t	g	c	t	t	a	c	-	t	c	c	g	c	a	a	c	a	a	g	c	t	a
MH709366	2	t	g	t	a	a	c	t	a	g	g	a	t	t	t	g	t	t	a	g	c	c	c	t	c	a	c	a	a	a	t	t	a	a	t	c	a	a	a	c	c	t	t	g	c	t	t	a	c	a	t	c	c	g	c	a	a	c	a	a	g	c	t	a
MH709376	2	t	g	t	a	a	c	t	a	g	g	a	t	t	t	g	t	t	a	g	c	c	c	t	c	a	c	a	a	a	t	t	a	a	t	c	a	a	a	c	c	t	t	g	c	t	t	a	c	a	t	c	c	g	c	a	a	c	a	a	g	c	t	a
MH709377	2	t	g	t	a	a	c	t	a	g	g	a	t	t	t	g	t	t	a	g	c	c	c	t	c	a	c	a	a	a	t	t	a	a	t	c	a	a	a	c	c	t	t	g	c	t	t	a	c	-	t	c	c	g	c	a	a	c	a	a	g	c	t	a
**MW316406**	2	t	g	t	a	a	c	t	a	g	g	a	t	t	t	g	t	t	a	g	c	c	c	t	c	a	c	a	a	a	t	t	a	a	t	c	a	a	a	c	c	t	t	g	c	t	t	a	c	-	t	c	c	g	c	a	a	c	a	a	g	c	t	a
AJ871403	3	t	g	t	a	a	c	t	a	g	g	a	t	t	c	g	t	t	a	g	c	c	c	t	c	a	c	a	a	a	t	t	a	a	t	c	a	a	a	t	c	t	t	g	c	t	t	a	c	-	t	c	c	g	c	a	a	c	a	g	g	c	t	c
DQ479955	3	t	g	t	a	a	c	t	a	g	g	a	t	t	c	g	t	t	a	a	c	c	c	t	c	a	c	a	a	a	t	t	a	a	t	c	a	a	a	t	c	t	t	g	c	t	t	a	c	-	t	c	c	g	c	a	a	c	a	g	g	c	t	c
DQ479956	3	t	g	t	a	a	c	t	a	g	g	a	t	t	c	g	t	t	a	g	c	c	c	t	c	a	c	a	a	a	t	t	a	a	t	c	a	a	a	t	c	t	t	g	c	t	t	a	c	-	t	c	c	g	c	a	a	c	a	g	g	c	t	c
DQ479957	3	t	g	t	a	a	c	t	a	g	g	a	t	t	c	g	t	t	a	g	c	c	c	t	t	a	c	a	a	a	t	t	a	a	t	c	a	a	a	t	c	t	t	g	c	t	t	a	c	-	t	c	c	g	c	a	a	c	a	g	g	c	t	c
JN704699	3	t	g	t	a	a	c	t	a	g	g	a	t	t	c	g	t	t	a	g	c	c	c	t	c	a	c	a	a	a	t	t	a	a	t	c	a	a	a	t	c	t	t	g	c	t	t	a	c	-	t	c	c	g	c	a	a	c	a	g	g	c	t	c
JN704700	3	t	g	t	a	a	c	t	a	g	g	a	t	t	c	g	t	t	a	g	c	t	c	t	c	a	c	a	a	a	t	t	a	a	t	c	a	a	a	t	c	t	t	g	c	t	t	a	c	-	t	c	c	g	c	a	g	c	a	g	g	a	t	c
JN704701	3	t	g	t	a	a	c	t	a	g	g	a	t	t	c	g	t	t	a	g	c	c	c	t	c	a	c	a	a	a	t	t	a	a	t	c	a	a	a	t	c	t	t	g	c	t	t	a	c	-	t	c	c	g	c	a	g	c	a	g	g	c	t	c
JN704702	3	t	g	t	a	a	c	t	a	g	g	a	t	t	c	g	t	t	a	g	c	c	c	t	c	a	c	a	a	a	t	t	a	a	t	c	a	a	a	t	c	c	t	g	c	t	t	a	c	-	t	c	c	g	c	a	g	c	a	g	g	c	t	c
JN704703	3	t	g	t	a	a	c	t	a	g	g	a	t	t	c	g	t	t	a	g	c	c	c	t	c	a	c	a	a	a	t	t	a	a	t	c	a	a	a	t	c	t	t	g	c	t	t	a	c	-	t	c	c	g	c	a	g	c	a	g	g	c	t	c
KP771891	3	t	g	t	a	a	c	t	a	g	g	a	g	t	c	g	t	t	a	g	c	c	c	t	c	a	c	a	a	a	t	t	a	a	t	c	a	a	a	t	c	t	t	g	c	t	t	n	n	-	n	n	n	n	n	n	n	n	n	n	n	c	t	c
KP771893	3	t	g	t	a	a	c	t	a	g	g	a	t	t	c	g	t	t	a	g	c	c	c	t	c	a	c	a	a	a	t	t	a	a	t	c	a	a	a	t	c	t	t	g	c	t	t	n	n	-	n	n	n	n	n	n	n	n	n	n	n	c	t	c
KP771895	3	t	g	t	a	a	c	t	a	g	g	a	t	t	c	g	t	t	a	g	c	c	c	t	c	a	c	a	a	a	t	t	a	a	t	c	a	a	a	t	c	t	t	g	c	t	t	n	n	-	n	n	n	n	n	n	n	n	n	n	n	c	t	c
KP771896	3	t	g	t	a	a	c	t	a	g	g	a	t	t	c	g	t	t	a	g	c	c	c	t	c	a	c	a	a	a	t	t	a	a	t	c	a	a	a	t	c	t	t	g	c	t	t	n	n	-	n	n	n	n	n	n	n	n	n	n	n	c	t	c
KP771897	3	t	g	t	a	a	c	t	a	g	g	a	t	t	c	g	t	t	a	g	c	c	c	t	c	a	c	a	a	a	t	t	a	a	t	c	a	a	a	t	c	t	t	g	c	t	t	n	n	-	n	n	n	n	n	n	n	n	n	n	n	c	t	c
KP771899	3	t	g	t	a	a	c	t	a	g	g	a	t	t	c	g	t	t	a	g	c	c	c	t	c	a	c	a	a	a	t	t	a	a	t	c	a	a	a	t	c	t	t	g	c	t	t	n	n	-	n	n	n	n	n	n	n	n	n	n	n	c	t	c
KP771903	3	t	g	t	a	a	c	t	a	g	g	a	t	t	c	g	t	t	a	g	c	c	c	t	c	a	c	a	a	a	t	t	a	a	t	c	a	a	a	t	c	t	t	g	c	t	t	n	n	-	n	n	n	n	n	n	n	n	n	n	n	c	t	c
KP771907	3	t	g	t	a	a	c	t	a	g	g	a	t	t	c	g	t	t	a	g	c	c	c	t	c	a	c	a	a	a	t	t	a	a	t	c	a	a	a	t	c	t	t	g	c	t	t	n	n	-	n	n	n	n	n	n	n	n	n	n	n	c	t	c
KP771910	3	t	g	t	a	a	c	t	a	g	g	a	t	t	c	g	t	t	a	g	c	c	c	t	c	a	c	a	a	a	t	t	a	a	t	c	a	a	a	t	c	t	t	g	c	t	t	n	n	-	n	n	n	n	n	n	n	n	n	n	n	c	t	c
KP771915	3	t	g	t	a	a	c	t	a	g	g	a	t	t	c	g	t	t	a	g	c	c	c	t	c	a	c	a	a	a	t	t	a	a	t	c	a	a	a	t	c	t	t	g	c	t	t	n	n	-	n	n	n	n	n	n	n	n	n	n	n	c	t	c
KP771916	3	t	g	t	a	a	c	t	a	g	g	a	t	t	c	g	t	t	a	g	c	c	c	t	c	a	c	a	a	a	t	t	a	a	t	c	a	a	a	t	c	t	t	g	c	t	t	n	n	-	n	n	n	n	n	n	n	n	n	n	n	c	t	c
KP771921	3	t	g	t	a	a	c	t	a	g	g	a	t	t	c	g	t	t	a	g	c	c	c	t	c	a	c	a	a	a	t	t	a	a	t	c	a	a	a	t	c	t	t	g	c	t	t	n	n	-	n	n	n	n	n	n	n	n	n	n	n	c	t	c
KP771923	3	t	a	t	a	a	c	t	a	g	g	a	t	t	c	g	t	t	a	g	c	c	c	t	c	a	c	a	a	a	t	t	a	a	t	c	a	a	a	t	c	t	t	g	c	t	t	n	n	-	n	n	n	n	n	n	n	n	n	n	n	c	t	c
KP771889	3	t	g	t	a	a	c	t	a	g	g	a	t	t	c	g	t	t	a	g	c	c	c	t	c	a	c	a	a	a	t	t	a	a	t	c	a	a	a	t	c	t	t	g	c	t	t	n	n	-	n	n	n	n	n	n	n	n	n	n	n	c	t	c
KU926311	3	t	g	t	a	a	c	t	a	g	g	a	t	t	c	g	t	t	a	g	c	c	c	t	c	a	c	a	a	a	t	t	a	a	t	c	a	a	a	t	c	t	t	g	c	t	t	a	c	-	t	c	c	g	c	a	a	c	a	g	g	c	t	c
JQ911599	3	t	g	t	a	a	c	t	a	g	g	a	t	t	c	g	t	t	a	g	c	c	c	t	c	a	c	a	a	a	t	t	a	a	t	c	a	a	a	t	c	t	t	g	c	t	t	a	c	-	t	c	a	g	c	a	g	c	a	g	g	c	t	c
MH709317	3	t	g	t	a	a	c	t	a	g	g	a	t	t	c	g	t	t	a	g	c	c	c	t	c	a	c	a	a	a	t	t	a	a	t	c	a	a	a	t	c	t	t	g	c	t	t	a	c	-	t	c	c	g	c	a	g	c	a	g	g	c	t	c
MH709346	3	t	g	t	a	a	c	t	a	g	g	a	t	t	c	g	t	t	a	g	a	c	c	t	c	a	c	a	a	a	t	t	a	a	t	c	a	a	a	t	c	t	t	g	c	t	t	a	c	-	t	c	c	g	c	a	g	c	a	g	g	c	t	c
MH709375	3	t	g	t	a	a	c	t	a	g	g	a	t	t	c	g	t	t	a	g	c	c	c	t	c	a	c	a	a	a	t	t	a	a	t	c	a	a	a	t	c	t	t	g	c	t	t	a	c	-	t	c	c	g	c	a	g	c	a	g	g	c	t	c
DQ452050	4	t	g	t	a	a	c	t	a	g	g	a	t	t	c	g	t	t	a	g	c	c	c	t	c	a	c	a	a	a	t	t	a	a	t	c	a	a	a	c	c	t	t	g	c	t	t	a	c	-	t	c	c	g	c	a	a	c	a	a	g	c	t	a
DQ479960	4	t	g	t	a	a	c	t	a	g	g	a	t	t	c	g	t	t	a	g	c	c	c	t	c	a	c	a	a	a	t	t	a	a	t	c	a	a	a	c	c	t	t	g	c	t	t	a	c	-	t	c	c	g	c	a	a	c	a	a	g	c	t	a
KP771898	4	t	g	t	a	a	c	t	a	g	g	a	t	t	c	g	t	t	a	g	c	c	c	t	c	t	c	a	a	a	t	t	a	a	t	c	a	a	a	c	c	t	t	g	c	t	t	n	n	-	n	n	n	n	n	n	n	n	n	n	n	c	t	a
MH709316	4	t	g	t	a	a	c	t	a	g	g	a	t	t	c	g	t	t	a	g	c	c	c	t	c	a	c	a	a	a	t	t	a	a	t	c	a	a	a	c	c	t	t	g	c	t	t	a	c	-	t	c	c	g	c	a	a	c	a	a	g	c	t	a
MH709318	4	t	g	t	a	a	c	t	a	g	g	a	t	t	c	g	t	t	a	g	c	c	c	t	c	a	c	a	a	a	t	t	a	a	t	c	a	a	a	c	c	t	t	g	c	t	t	a	c	-	t	c	c	g	c	a	a	c	a	a	g	c	t	a
MH709322	4	t	g	t	a	a	c	t	a	g	g	a	t	t	c	g	t	t	a	g	c	c	c	t	c	a	c	a	a	a	t	t	a	a	t	c	a	a	a	c	c	t	t	g	c	t	t	a	c	-	t	c	c	g	c	a	a	c	a	a	g	c	t	a
MH709323	4	t	g	t	a	a	c	t	a	g	g	a	t	t	c	g	t	t	a	g	c	c	c	t	c	a	c	a	a	a	t	t	a	a	t	c	a	a	a	c	c	t	t	g	c	t	t	a	a	-	t	c	c	g	c	a	a	c	a	a	g	c	t	a
MH709326	4	t	g	t	a	a	c	t	a	g	g	a	t	t	c	g	t	t	a	g	c	c	c	t	c	a	c	a	a	a	t	t	t	t	a	c	a	a	a	c	c	t	t	g	c	t	t	a	c	-	t	c	c	g	c	a	a	c	a	a	g	c	t	a
MH709341	4	t	g	t	a	a	c	t	a	g	g	a	t	t	c	g	t	t	a	g	c	c	c	t	c	a	c	a	a	a	t	t	a	a	t	c	a	a	a	c	c	t	t	g	c	t	t	a	c	-	t	c	c	g	c	a	a	c	a	a	g	c	t	a
MH709357	4	t	g	t	a	a	c	t	a	g	g	a	t	t	c	g	t	t	a	g	c	c	c	t	c	a	c	a	a	a	t	t	a	a	t	c	a	a	a	c	c	t	t	g	c	t	t	a	c	-	t	c	c	g	c	a	a	c	a	a	g	c	t	a
MH709364	4	t	g	t	a	a	c	t	a	g	g	a	t	t	c	g	t	t	a	g	c	c	c	t	c	a	c	a	a	a	t	t	a	a	t	c	a	a	a	c	c	t	t	g	c	t	t	a	c	-	t	c	c	g	c	a	a	c	a	a	g	c	t	a
MH709370	4	t	g	t	a	a	c	c	a	g	g	a	t	t	c	g	t	t	a	g	c	c	c	t	c	a	c	a	a	a	t	t	a	a	t	c	a	a	a	c	c	t	t	g	c	t	t	a	c	a	t	c	c	g	c	a	a	c	a	a	g	c	t	a
MH709371	4	t	g	t	a	a	c	t	a	g	g	a	t	t	c	g	t	t	a	g	c	c	c	t	c	a	t	a	a	a	t	t	a	a	t	c	a	a	a	c	c	t	t	g	c	t	t	a	c	-	t	c	c	g	c	a	a	c	a	a	g	c	t	a
MH709372	4	t	g	t	a	a	c	t	a	g	g	a	t	t	c	g	t	t	a	g	c	c	c	t	c	a	c	a	a	a	t	t	a	a	t	c	a	a	a	c	c	t	t	g	c	t	t	a	c	-	t	c	c	g	c	a	a	c	a	a	g	c	t	a
DQ457052	5	t	g	t	a	a	t	t	a	g	g	a	t	t	c	g	t	t	a	g	a	c	c	t	c	a	c	a	a	a	t	t	a	a	t	c	a	a	a	t	c	t	t	g	c	t	t	g	c	-	t	c	c	g	c	a	a	c	a	g	g	c	t	c
JN704704	5	t	g	t	a	a	c	t	a	g	g	a	t	t	c	g	t	t	a	g	a	c	c	t	c	a	c	a	a	a	t	t	a	a	t	c	a	a	a	t	c	t	t	g	c	t	t	g	c	-	t	c	c	g	c	a	a	c	a	g	g	c	t	c
JN704705	5	t	g	t	a	a	c	t	a	g	g	a	t	t	c	g	t	t	a	g	a	c	c	t	c	a	c	a	a	a	t	t	a	a	t	c	a	a	a	t	c	t	t	g	c	t	t	a	c	-	t	c	c	g	c	a	a	c	a	g	g	c	t	c
JN704706	5	t	g	t	g	a	c	t	a	g	g	a	t	t	c	g	t	t	a	g	a	c	c	t	c	a	c	a	a	a	t	t	a	a	t	c	a	a	a	t	c	t	t	g	c	t	t	a	c	-	t	c	c	g	c	a	a	c	a	g	g	c	t	c
JN704707	5	t	g	t	a	a	c	t	a	g	g	a	t	t	c	g	t	t	a	g	a	c	c	t	c	a	c	a	a	a	t	t	a	a	t	c	a	a	a	t	c	t	t	g	c	t	t	g	c	-	t	c	c	g	c	a	a	c	a	g	g	c	t	c
JN704708	5	t	g	t	a	a	c	t	a	g	g	a	t	t	c	g	t	t	a	g	a	c	c	t	c	a	c	a	a	a	t	t	a	a	t	c	a	a	a	t	c	t	t	g	c	t	t	a	c	-	t	c	c	g	c	a	a	c	a	g	g	c	t	c
KP771904	5	t	g	t	a	a	c	t	a	g	g	a	t	t	c	g	t	t	a	g	a	c	c	t	c	a	c	a	a	a	t	t	a	a	t	c	a	a	a	t	c	t	t	g	c	t	t	n	n	-	n	n	n	n	n	n	n	n	n	n	n	c	t	c
KP771905	5	t	g	t	a	a	c	t	a	g	g	a	t	t	c	g	t	t	a	g	a	c	c	t	c	a	c	a	a	a	t	t	a	a	t	c	a	a	a	t	c	t	t	g	c	t	t	n	n	-	n	n	n	n	n	n	n	n	n	n	n	c	t	c
KP771906	5	t	g	t	a	a	c	t	a	g	g	a	t	t	c	g	t	t	a	g	a	c	c	t	c	a	c	a	a	a	t	t	a	a	t	c	a	a	a	t	c	t	t	g	c	t	t	n	n	-	n	n	n	n	n	n	n	n	n	n	n	c	t	c
KP771919	5	t	g	t	a	a	c	t	a	g	g	a	t	t	c	g	t	t	a	g	a	c	c	t	c	a	c	a	a	a	t	t	a	a	t	c	a	a	a	t	c	t	t	g	c	t	t	n	n	-	n	n	n	n	n	n	n	n	n	n	n	c	t	c
KP771922	5	t	g	t	a	a	c	t	a	g	g	a	t	t	c	g	t	t	a	g	a	c	c	t	c	a	c	a	a	a	t	t	a	a	t	c	a	a	a	t	c	t	t	g	c	t	t	n	n	-	n	n	n	n	n	n	n	n	n	n	n	c	t	c
KP771924	5	t	g	t	a	a	c	t	a	g	g	a	t	t	c	g	t	t	a	g	a	c	c	t	c	a	c	a	a	a	t	t	a	a	t	c	a	a	a	t	c	t	t	g	c	t	t	n	n	-	n	n	n	n	n	n	n	n	n	n	n	c	t	c
KM355696	5	t	g	t	a	a	c	t	a	g	g	a	t	t	c	g	t	t	a	g	a	c	c	t	c	a	c	a	a	a	t	t	a	a	t	c	a	a	a	t	c	t	t	g	c	t	t	a	c	-	t	c	c	g	c	a	a	c	a	g	g	c	t	c
KM355697	5	t	g	t	a	a	c	t	a	g	g	a	t	t	c	g	t	t	a	g	a	c	c	t	c	a	c	a	a	a	t	t	a	a	t	c	a	a	a	t	c	t	t	g	c	t	t	a	c	-	t	c	c	g	c	a	a	c	a	g	g	c	t	c
KM355698	5	t	g	t	a	a	c	t	a	g	g	a	t	t	c	g	t	t	a	g	a	c	c	t	c	a	c	a	a	a	t	t	a	a	t	c	a	a	a	t	c	t	t	g	c	t	t	a	c	-	c	c	c	g	c	a	g	c	a	g	g	c	t	c
KM355699	5	t	g	t	a	a	c	t	a	g	g	a	t	t	c	g	t	t	a	g	a	c	c	t	c	a	c	a	a	a	t	t	a	a	t	c	a	a	a	t	c	t	t	g	c	t	t	a	c	-	c	c	c	g	c	a	g	c	a	g	g	c	t	c
KM355701	5	t	g	t	a	a	c	t	a	g	g	a	t	t	c	g	t	t	a	g	a	c	c	t	c	a	c	a	a	a	t	t	a	a	t	c	a	a	a	t	c	t	t	g	c	t	t	a	c	-	t	c	c	g	c	a	a	c	a	g	g	c	t	c
KM355702	5	t	g	t	a	a	c	t	a	g	g	a	t	t	c	g	t	t	a	g	a	c	c	t	c	a	c	a	a	a	t	t	a	a	t	c	a	a	a	t	c	t	t	g	c	t	t	a	c	-	c	c	c	g	c	a	g	c	a	g	g	c	t	c
KM355703	5	t	g	t	a	a	c	t	a	g	g	a	t	t	c	g	t	t	a	g	a	c	c	t	c	a	c	a	a	a	t	t	a	a	t	c	a	a	a	t	c	t	t	g	c	t	t	a	c	-	t	c	c	g	c	a	a	c	a	g	g	c	t	c
KM355704	5	t	g	t	a	a	c	t	a	g	g	a	t	t	c	g	t	t	a	g	a	c	c	t	c	a	c	a	a	a	t	t	a	a	t	c	a	a	a	t	c	t	t	g	c	t	t	a	c	-	t	c	c	g	c	a	a	c	a	g	g	c	t	c
KM355705	5	t	g	t	a	a	c	t	a	g	g	a	t	t	c	g	t	t	a	g	a	c	c	t	c	a	c	a	a	a	t	t	a	a	t	c	a	a	a	t	c	t	t	g	c	t	t	a	c	-	c	c	c	g	c	a	g	c	a	g	g	c	t	c
KM355706	5	t	g	t	a	a	c	t	a	g	g	a	t	t	c	g	t	t	a	g	a	c	c	t	c	a	c	a	a	a	t	t	a	a	t	c	a	a	a	t	c	t	t	g	c	t	t	a	c	-	c	c	c	g	c	a	g	c	a	g	g	c	t	c
KM355707	5	t	g	t	a	a	c	t	a	g	g	a	t	t	c	g	t	t	a	g	a	c	c	t	c	a	c	a	a	a	t	t	a	a	t	c	a	a	a	t	c	t	t	g	c	t	t	a	c	-	t	c	c	g	c	a	a	c	a	g	g	c	t	c
KM355708	5	t	g	t	a	a	c	t	a	g	g	a	t	t	c	g	t	t	a	g	a	c	c	t	c	a	c	a	a	a	t	t	a	a	t	c	a	a	a	t	c	t	t	g	c	t	t	a	c	-	t	c	c	g	c	a	a	c	a	g	g	c	t	c
KM355709	5	t	g	t	a	a	c	t	a	g	g	a	t	t	c	g	t	t	a	g	a	c	c	t	c	a	c	a	a	a	t	t	a	a	t	c	a	a	a	t	c	t	t	g	c	t	t	a	c	-	c	c	c	g	c	a	g	c	a	g	g	c	t	c
KM355711	5	t	g	t	a	a	c	t	a	g	g	a	t	t	c	g	t	t	a	g	a	c	c	t	c	a	c	a	a	a	t	t	a	a	t	c	a	a	a	t	c	t	t	g	c	t	t	a	c	-	c	c	c	g	c	a	g	c	a	g	g	c	t	c
KM355712	5	t	g	t	a	a	c	t	a	g	g	a	t	t	c	g	t	t	a	g	a	c	c	t	c	a	c	a	a	a	t	t	a	a	t	c	a	a	a	t	c	t	t	g	c	t	t	a	c	-	c	c	c	g	c	a	g	c	a	g	g	c	t	c
KY037797	[illegible]	[illegible]	[illegible]	[illegible]	[illegible]	[illegible]	[illegible]	[illegible]	[illegible]	[illegible]	[illegible]	[illegible]	[illegible]	[illegible]	[illegible]	[illegible]	[illegible]	[illegible]	[illegible]	[illegible]	[illegible]	[illegible]	[illegible]	[illegible]	[illegible]	[illegible]	[illegible]	[illegible]	[illegible]	[illegible]	[illegible]	[illegible]	[illegible]	[illegible]	[illegible]	[illegible]	[illegible]	[illegible]	[illegible]	[illegible]	[illegible]	[illegible]	[illegible]	[illegible]	[illegible]	[illegible]	[illegible]	[illegible]	[illegible]	[illegible]	[illegible]	[illegible]	[illegible]	[illegible]	[illegible]	[illegible]	[illegible]	[illegible]	[illegible]	[illegible]	[illegible]	[illegible]	[illegible]	[illegible]

Supplementary Table 3: 2880 SN

GB-ID	Clade markers														2		9																																															
	Position in Dumas	100812	100887	100970	101071	101089	101166	101198	101218	101236	101243	101245	101260	101327	101331	101334	101353	101366	101425	101459	101464	101515	101619	101620	101626	101627	101652	101697	101706	101720	101768	101785	101806	101807	101808	101830	101847	101875	101883	101886	101900	101902	101937	101944	101949	101991	102002	102020	102021	102021_1	102037	102051	102054	102060	102062	102067	102080	102144	102179	102192	102203	102232	102268	102309
MH499467	5	t	g	t	a	a	c	t	a	g	g	a	t	t	c	g	t	t	a	g	a	c	c	t	c	a	c	a	a	a	t	t	a	a	t	c	a	a	a	t	c	t	t	g	c	t	t	g	c	-	t	c	c	g	c	a	a	c	a	g	g	c	t	c
MH499469	5	t	g	t	a	a	c	t	a	g	g	a	t	t	c	g	t	t	a	g	a	c	c	t	c	a	c	a	a	a	t	t	a	a	t	c	a	a	a	t	c	t	t	g	c	t	t	g	c	-	t	c	c	g	c	a	a	c	a	g	g	c	t	c
MH709321	5	t	g	t	a	a	c	t	a	g	t	a	t	t	c	g	t	t	a	g	a	c	c	t	c	a	c	a	a	a	t	t	a	a	t	c	c	a	a	t	c	t	t	g	c	t	t	a	c	-	t	c	c	g	c	a	a	c	a	g	g	c	t	c
MH709324	5	t	g	t	a	a	c	t	a	g	g	a	t	t	c	g	t	t	a	g	a	c	c	t	c	a	c	a	a	a	t	t	a	a	t	c	a	a	a	t	c	t	t	g	c	t	t	g	c	-	t	c	c	g	c	a	a	c	a	g	g	c	t	c
MH709325	5	t	g	t	a	a	c	t	a	g	g	a	t	t	c	g	t	t	a	g	a	c	c	t	c	a	c	a	a	a	t	t	a	a	t	c	a	a	a	t	c	t	t	g	c	t	t	a	c	-	t	c	c	g	c	a	g	c	a	g	g	c	t	c
MH709327	5	t	g	t	a	a	c	t	a	g	g	a	t	t	c	g	t	t	a	g	a	c	c	t	c	a	c	a	a	a	t	t	a	a	t	c	a	a	a	t	c	t	t	g	c	t	t	g	c	-	t	c	c	g	c	a	a	c	a	g	g	c	t	c
MH709328	5	t	g	t	a	a	c	t	a	g	g	a	t	t	c	g	t	t	a	g	a	c	c	t	c	a	c	a	a	a	t	t	a	a	t	c	a	a	a	t	c	t	t	g	c	t	t	g	c	-	t	c	c	g	c	a	a	c	a	g	g	c	t	c
MH709330	5	t	g	t	a	a	c	t	a	g	g	a	t	t	c	g	t	t	a	g	a	c	c	t	c	a	c	a	a	a	t	t	a	a	t	c	a	a	a	t	c	t	t	g	c	t	t	g	c	-	t	c	c	g	c	a	a	c	a	g	g	c	t	c
MH709332	5	t	g	t	a	a	c	t	a	g	g	a	t	t	c	g	t	t	a	g	a	c	c	t	c	a	c	a	a	a	t	t	a	a	t	c	a	a	a	t	c	t	t	g	c	t	t	g	c	-	t	c	c	g	c	a	a	c	a	g	g	c	t	c
MH709334	5	t	g	t	a	a	c	t	a	g	g	a	t	t	c	g	t	t	a	g	a	c	c	t	c	a	c	a	a	a	t	t	a	a	t	c	a	a	a	t	c	t	t	g	c	t	t	g	c	-	t	c	c	g	c	a	g	c	a	g	g	c	t	c
MH709335	5	t	g	t	a	a	c	t	a	g	g	a	t	t	c	g	t	t	a	g	a	c	c	t	c	a	c	a	a	a	t	t	a	a	t	c	a	a	a	t	c	t	t	g	c	t	t	g	c	-	t	c	c	g	c	a	a	c	a	g	g	c	t	c
MH709336	5	t	g	t	a	a	c	t	a	g	g	a	t	t	c	g	t	t	a	g	a	c	c	t	c	a	c	a	a	a	t	t	a	a	t	c	a	a	a	t	c	t	t	g	t	t	t	g	c	-	t	c	c	g	c	a	a	c	a	g	g	c	t	c
MH709338	5	t	g	t	a	a	c	t	a	g	g	a	t	t	c	g	t	t	a	g	a	c	c	t	c	a	c	a	a	a	t	t	a	a	t	c	a	a	a	t	c	t	t	g	c	t	t	g	c	-	t	c	c	g	c	a	a	c	a	g	g	c	t	c
MH709340	5	t	g	t	a	a	c	t	a	g	g	a	t	t	c	g	t	t	a	g	a	c	c	t	c	a	c	a	a	a	t	t	a	a	t	t	a	a	a	t	c	t	t	g	c	t	t	a	c	-	t	c	c	g	c	a	a	c	a	g	g	c	t	c
MH709342	5	t	g	t	a	a	c	t	a	g	t	a	t	t	c	g	t	t	a	g	a	c	c	t	c	a	c	a	a	a	t	t	a	a	t	c	c	a	a	t	c	t	t	g	c	t	t	a	c	-	t	c	c	g	c	a	a	c	a	g	g	c	t	c
MH709343	5	t	g	t	a	a	c	t	a	g	g	a	t	t	c	g	t	t	a	g	a	c	c	t	c	a	c	a	a	a	t	t	a	a	t	c	a	a	a	t	c	t	t	g	c	t	t	g	c	-	t	c	c	g	c	a	a	c	a	g	g	c	t	c
MH709345	5	t	g	t	a	a	c	t	a	g	g	a	t	t	c	g	t	t	a	g	a	c	c	t	c	a	c	a	a	a	t	t	a	a	t	c	a	a	a	t	c	t	t	g	c	t	t	g	c	-	t	c	c	g	c	a	a	c	a	g	g	c	t	c
MH709347	5	t	g	t	a	a	c	t	a	g	g	a	t	t	c	g	t	t	a	g	a	c	c	t	c	a	c	a	a	a	t	t	a	a	t	c	a	a	a	t	c	t	t	g	c	t	t	g	c	-	t	c	c	g	c	a	a	c	a	g	g	c	t	c
MH709349	5	t	g	t	a	a	c	t	a	g	g	a	t	t	c	g	t	t	a	g	a	c	c	t	c	a	c	a	a	a	t	t	a	a	t	c	a	a	a	t	c	t	t	g	c	t	t	g	c	-	t	c	c	a	c	a	a	c	a	g	g	c	t	c
MH709350	5	t	g	t	a	a	c	t	a	g	g	a	t	t	c	g	t	t	a	g	a	c	c	t	c	a	c	a	a	a	t	t	a	a	t	c	a	a	a	t	c	t	t	g	c	t	t	g	c	-	t	c	c	g	c	a	a	c	a	g	g	c	t	c
MH709351	5	t	g	t	a	a	c	t	a	g	g	a	t	t	c	g	t	t	a	g	a	c	c	t	c	a	c	a	a	a	t	t	a	a	t	c	a	a	a	t	c	t	t	g	c	t	t	a	c	-	t	c	c	g	c	a	a	c	a	a	g	c	t	c
MH709353	5	t	g	t	a	a	c	t	a	g	g	a	t	t	c	g	t	t	a	g	a	c	c	t	c	a	c	a	a	a	t	t	a	a	t	c	a	a	a	t	c	t	t	g	c	t	t	g	c	-	t	c	c	g	c	a	a	c	a	g	g	c	t	c
MH709358	5	t	g	t	a	a	c	t	a	g	g	a	t	t	c	g	t	t	a	g	a	c	c	t	c	a	c	a	a	a	t	t	a	a	t	c	a	a	a	t	c	t	t	g	c	t	t	a	c	-	t	c	c	g	c	a	a	c	a	g	g	c	t	c
MH709360	5	t	g	t	a	a	c	t	a	g	g	a	t	t	c	g	t	t	a	g	a	c	c	t	c	a	c	a	a	a	t	t	a	a	t	c	a	a	a	t	c	t	t	g	c	t	t	g	c	-	t	c	c	g	c	a	a	c	a	g	g	c	t	c
MH709362	5	t	g	t	a	a	c	t	a	g	g	a	t	t	c	g	t	t	a	g	a	c	c	t	c	a	c	a	a	a	t	t	a	a	t	c	a	a	a	t	c	t	g	a	c	t	t	g	c	-	t	c	c	g	c	a	a	c	a	g	g	c	t	c
MH709365	5	t	g	t	a	a	c	t	a	g	g	a	t	t	c	g	t	t	a	g	a	c	c	t	c	a	c	a	a	a	t	t	a	a	t	c	a	a	a	t	c	t	t	g	c	t	t	g	c	-	t	c	c	g	c	a	a	c	a	g	g	c	t	c
MH709373	5	t	g	t	a	a	c	t	a	g	g	a	t	t	c	g	t	t	a	g	a	c	c	t	c	a	c	a	a	a	t	t	a	a	t	c	a	a	a	t	c	t	t	g	c	t	t	g	c	-	t	c	c	g	c	a	a	c	a	g	g	c	t	c
MH709374	5	t	g	t	a	a	c	t	a	g	g	a	t	t	c	g	t	t	a	g	a	c	c	t	c	a	c	a	a	a	t	t	a	a	t	c	a	a	a	t	c	t	t	g	c	t	t	g	c	-	t	c	c	g	c	a	a	c	a	g	g	c	t	c
KC112914	6	t	g	t	a	a	c	t	a	g	g	a	t	t	c	g	t	t	a	g	c	c	c	t	c	a	c	a	a	a	t	t	a	a	t	c	a	a	a	c	c	t	t	g	c	t	t	a	c	-	t	c	c	g	c	a	a	c	a	g	g	c	t	a
KP771914	6	t	g	t	a	a	c	t	a	g	g	a	t	t	c	g	t	t	a	g	c	c	c	t	c	a	c	a	a	a	t	t	a	a	t	c	a	a	a	c	c	t	t	g	c	t	t	n	n	-	n	n	n	n	n	n	n	n	n	n	n	c	t	a
KP702725	6	t	g	t	a	a	c	t	a	g	g	a	t	t	c	g	t	t	a	g	c	c	c	t	c	a	c	a	a	a	t	t	a	a	t	c	a	a	a	c	c	t	t	g	c	t	t	a	c	-	t	c	c	g	c	a	a	c	a	g	g	c	t	a
KP771890	9	t	g	t	a	a	c	t	a	g	g	a	t	t	c	g	g	t	a	g	c	c	c	t	c	a	c	a	a	a	t	t	a	a	t	c	a	a	a	c	c	t	t	g	c	t	t	n	n	-	n	n	n	n	n	n	n	n	n	n	n	c	t	a
JN704710	9	t	g	t	a	a	c	t	a	g	g	a	t	t	c	g	g	t	a	g	c	c	c	t	c	a	c	a	a	a	t	t	a	a	t	c	a	a	a	t	c	t	t	g	c	c	t	a	c	-	t	c	c	g	c	a	a	c	a	g	a	c	t	a
KY037798	9	t	g	t	a	a	c	t	a	g	g	a	t	t	c	g	g	t	a	g	c	c	c	t	c	a	c	a	a	a	t	t	a	a	t	c	a	a	a	c	c	t	t	g	c	t	t	a	c	-	t	c	c	g	c	a	a	c	a	a	g	c	t	a
MH709312	9	t	g	t	a	a	c	t	a	g	g	a	t	t	c	g	g	t	a	g	c	c	t	c	c	a	c	a	a	a	t	t	a	a	t	c	a	a	a	c	c	t	t	g	c	t	t	a	c	-	t	c	c	g	c	a	a	c	a	a	g	c	t	a
MH709352	9	t	g	t	a	a	c	t	a	g	g	a	t	t	c	g	g	t	a	g	c	c	c	t	c	a	c	a	a	a	t	t	a	a	t	c	a	a	a	c	c	t	t	g	c	t	t	a	c	a	t	c	c	g	c	a	a	c	a	a	g	c	t	a
AB097933	Vac	t	g	t	a	a	c	t	a	g	g	a	t	t	t	g	t	t	a	g	c	c	c	t	c	a	c	a	a	a	t	t	a	a	t	c	a	a	a	c	c	t	t	g	c	t	t	a	c	a	t	c	c	g	c	a	a	c	a	a	g	c	t	a
JN704698	Vac	t	g	t	a	a	c	t	a	g	g	a	t	t	t	g	t	t	a	g	c	c	c	t	c	a	c	a	a	a	t	t	a	a	t	c	a	a	a	c	c	t	t	g	c	t	t	a	c	-	t	c	c	g	c	a	a	c	a	a	g	c	t	a
JN704697	Vac	t	g	t	a	g	c	t	a	g	g	a	t	t	t	g	t	t	a	g	c	c	c	t	c	a	c	a	a	a	t	t	a	a	t	c	a	a	a	c	c	t	t	g	c	t	t	a	c	-	t	c	c	g	c	a	a	c	a	a	g	c	t	a
DQ008355	Vac	t	g	t	a	r	c	t	a	g	g	a	t	t	t	g	t	t	a	g	c	c	c	t	c	a	c	a	a	a	t	t	a	a	t	c	a	a	a	c	c	t	t	g	c	t	t	a	c	-	t	c	c	g	c	a	a	c	a	a	g	c	t	a
JF306641	Vac	t	g	t	a	g	c	t	a	g	g	a	t	t	t	g	t	t	a	g	c	c	c	t	c	a	c	a	a	a	t	t	a	a	t	c	a	a	a	c	c	t	t	g	c	t	t	a	c	-	t	c	c	g	c	a	a	c	a	a	g	c	t	a
AB097932	Vac	t	g	t	a	r	c	t	a	g	g	a	t	t	t	g	t	t	a	g	c	c	c	t	c	a	c	a	a	a	t	t	a	a	t	c	a	a	a	c	c	t	t	g	c	t	t	a	c	a	t	c	c	g	c	a	a	c	a	a	g	c	t	a
DQ008354	Vac	t	g	t	a	r	c	t	a	g	g	a	t	t	t	g	t	t	a	g	c	c	c	t	c	a	c	a	a	a	t	t	a	a	t	c	a	a	a	c	c	t	t	g	c	t	t	a	c	-	t	c	c	g	c	a	a	c	a	a	g	c	t	a
KU926312	Vac	t	g	t	a	g	c	t	a	g	g	a	t	t	t	g	t	t	a	g	c	c	c	t	c	a	c	a	a	a	t	t	a	a	t	c	a	a	a	c	c	t	t	g	c	t	t	a	c	-	t	c	c	g	c	a	a	c	a	a	g	c	t	a
KU926313	Vac	t	g	t	a	g	c	t	a	g	g	a	t	t	t	g	t	t	a	g	c	c	c	t	c	a	c	a	a	a	t	t	a	a	t	c	a	a	a	c	c	t	t	g	c	t	t	a	c	-	t	c	c	g	c	a	a	c	a	a	g	c	t	a
KU926314	Vac	t	g	t	a	a	c	t	a	g	g	a	t	t	t	g	t	t	a	g	c	c	c	t	c	a	c	a	a	a	t	t	a	a	t	c	a	a	a	c	c	t	t	g	c	t	t	a	c	-	t	c	c	g	c	a	a	c	a	a	g	c	t	a
KF558371	Vac	t	g	t	a	g	c	t	a	g	g	a	t	t	t	g	t	t	a	g	c	c	c	t	c	a	c	a	a	a	t	t	a	a	t	c	a	a	a	c	c	t	t	g	c	t	t	n	n	n	n	n	n	n	n	n	n	n	n	n	n	c	t	a
KF558372	Vac	t	g	t	a	a	c	t	a	g	g	a	t	t	t	g	t	t	a	g	c	c	c	t	c	a	c	a	a	a	t	t	a	a	t	c	a	a	a	c	c	t	t	g	c	t	t	n	n	n	n	n	n	n	n	n	n	n	n	n	n	c	t	a
KF558373	Vac	t	g	t	a	a	c	t	a	g	g	a	t	t	t	g	t	t	a	g	c	c	c	t	c	a	c	a	a	a	t	t	a	a	t	c	a	a	a	c	c	t	t	g	c	t	t	n	n	-	n	n	n	n	n	n	n	n	n	n	n	c	t	a
KF558374	Vac	t	g	t	a	a	c	t	a	g	g	a	t	t	t	g	t	t	a	g	c	c	c	t	c	a	c	a	a	a	t	t	a	a	t	c	a	a	a	c	c	t	t	g	c	t	c	n	n	-	n	n	n	n	n	n	n	n	n	n	n	c	t	a
KF558375	Vac	t	g	t	a	a	c	t	a	g	g	a	t	t	t	g	t	t	a	g	c	c	c	t	c	a	c	a	a	a	t	t	a	a	t	c	a	a	a	c	c	t	t	g	c	t	c	n	n	n	n	n	n	n	n	n	n	n	n	n	n	c	t	a
KF558376	Vac	t	g	t	a	g	c	t	a	g	g	a	t	t	t	g	t	t	a	g	c	c	c	t	c	a	c	a	a	a	t	t	a	a	t	c	a	a	a	c	c	t	t	g	c	t	t	n	n	-	n	n	n	n	n	n	n	n	n	n	n	c	t	a
KF558377	Vac	t	g	t	a	g	c	t	a	g	g	a	t	t	t	g	t	t	a	g	c	c	c	t	c	a	c	a	a	a	t	t	a	a	t	c	a	a	a	c	c	t	t	g	c	t	t	n	n	-	n	n	n	n	n	n	n	n	n	n	n	c	t	a
KF558378	Vac	t	g	t	a	a	c	t	a	g	g	a	t	t	t	g	t	t	a	g	c	c	c	t	c	a	c	a	a	a	t	t	a	a	t	c	a	a	a	c	c	t	t	g	c	t	t	n	n	n	n	n	n	n	n	n	n	n	n	n	n	c	t	a
KF558379	Vac	t	g	t	a	a	c	t	a	g	g	a	t	t	t	g	t	t	a	g	c	c	c	t	c	a	c	a	a	a	t	t	a	a	t	c	a	a	a	c	c	t	t	g	c	t	t	n	n	-	n	n	n	n	n	n	n	n	n	n	n	c	t	a
KF558380	Vac	t	g	t	a	a	c	t	a	g	g	a	t	t	t	g	t	t	a	g	c	c	c	t	c	a	c	a	a	a	t	t	a	a	t	c	a	a	a	c	c	t	t	g	c	t	t	n	n	-	n	n	n	n	n	n	n	n	n	n	n	c	t	a
KF558381	Vac	t	g	t	a	a	c	t	a	g	g	a	t	t	t	g	t	t	a	g	c	c	c	t	c	a	c	a	a	a	t	t	a	a	t	c	a	a	a	c	c	t	t	g	c	t	t	n	n	-	n	n	n	n	n	n	n	n	n	n	n	c	t	a
KF558382	Vac	t	g	t	a	a	c	t	a	g	g	a	t	t	t	g	t	t	a	g	c	c	c	t	c	a	c	a	a	a	t	t	a	a	t	c	a	a	a	c	c	t	t	g	c	t	t	n	n	-	n	n	n	n	n	n	n	n	n	n	n	c	t	a
KF558383	Vac	t	g	t	a	a	c	t	a	g	g	a	t	t	t	g	t	t	a	g	c	c	c	t	c	a	c	a	a	a	t	t	a	a	t	c	a	a	a	c	c	t	t	g	c	t	t	n	n	n	n	n	n	n	n	n	n	n	n	n	n	c	t	a
KF558384	Vac	t	g	t	a	a	c	t	a	g	g	a	t	t	t	g	t	t	a	g	c	c	c	t	c	a	c	a	a	a	t	t	a	a	t	c	a	a	a	c	c	t	t	g	c	t	t	n	n	n	n	n	n	n	n	n	n	n	n	n	n	c	t	a
KF558385	Vac	t	g	t	a	a	c	t	a	g	g	a	t	t	t	g	t	t	a	g	c	c	c	t	c	a	c	a	a	a	t	t	a	a	t	c	a	a	a	c	c	t	t	g	c	t	t	n	n	n	n	n	n	n	n	n	n	n	n	n	n	c	t	a
KF558386	Vac	t	g	t	a	a	c	t	a	g	g	a	t	t	t	g	t	t	a	g	c	c	c	t	c	a	c	a	a	a	t	t	a	a	t	c	a	a	a	c	c	t	t	g	c	t	t	n	n	-	n	n	n	n	n	n	n	n	n	n	n	c	t	a
KF558387	Vac	t	g	t	a	a	c	t	a	g	g	a	t	t	t	g	t	t	a	g	c	c	c	t	c	a	c	a	a	a	t	t	a	a	t	c	a	a	a	c	c	t	t	g	c	t	t	n	n	-	n	n	n	n	n	n	n	n	n	n	n	c	t	a
KF558388	Vac	t	g	t	a	g	c	t	a	g	g	a	t	t	t	g	t	t	a	g	c	c	c	t	c	a	c	a	a	a	t	t	a	a	t	c	a	a	a	c	c	t	t	g	c	t	t	n	n	n	n	n	n	n	n	n	n	n	n	n	n	c	t	a
KF558389	Vac	t	g	t	a	a	c	t	a	g	g	a	t	t	t	g	t	t	a	g	c	c	c	t	c	a	c	a	a	a	t	t	a	a	t	c	a	a	a	c	c	t	t	g	c	t	c	n	n	-	n	n	n	n	n	n	n	n	n	n	n	c	t	a
KF558390	Vac	t	g	t	a	a	c	t	a	g	g	a	t	t	t	g	t	t	a	g	c	c	c	t	c	a	c	a	a	a	t	t	a	a	t	c	a	a	a	c	c	t	t	g	c	t	t	n	n	-	n	n	n	n	n	n	n	n	n	n	n	c	c	a
KF558391	Vac	t	g	t	a	a	c	t	a	g	g	a	t	t	t	g	t	t	a	g	c	c	c	t	c	a	c	a	a	a	t	t	a	a	t	c	a	a	a	c	c	t	t	g	c	t	c	n	n	-	n	n	n	n	n	n	n	n	n	n	n	c	t	a
KF811485	Vac	t	g	t	a	a	c	t	a	g	g	a	t	t	t	g	t	t	a	g	c	c	c	t	c	a	c	a	a	a	t	t	a	a	t	c	a	a	a	c	c	t	t	g	c	t	t	a	c	-	t	c	c	g	c	a	a	c	a	a	g	c	t	a
KF853225	Vac	t	g	t	a	g	c	t	a	g	g	a	t	t	t	g	t	t	a	g	c	c	c	t	c	a	c	a	a	a	t	t	a	a	t	c	a	a	a	c	c	t	t	g	c	t	t	a	c	-	t	c	c	g	c	a	a	n	n	n	n	c	t	a
KF853226	Vac	t	g	t	a	a	c	t	a	g	g	a	t	t	t	g	t	t	a	g	c	c	c	t	c	a	c	a	a	a	t	t	a	a	t	c	a	a	a	c	c	t	t	g	c	t	t	a	c	-	t	c	c	g	c	a	a	n	n	n	n	c	t	a
KF853227	Vac	t	g	t	a	a	c	t	a	g	g	a	t	t	t	g	t	t	a	g	c	c	c	t	c	a	c	a	a	a	t	t	a	a	t	c	a	a	a	c	c	t	t	g	c	t	t	a	c	-	t	c	c	g	c	a	a	n	n	n	n	c	t	a
KF853228	Vac	t	g	t	a	a	c	t	a	g	g	a	t	t	t	g	t	t	a	g	c	c	c	t	c	a	c	a	a	a	t	t	a	a	t	c	a	a	a	c	c	t	t	g	c	t	c	a	c	-	t	c	c	g	c	a	a	n	n	n	n	c	t	a
KF853229	Vac	t	g	t	a	g	c	t	a	g	g	a	t	t	t	g	t	t	a	g	c	c	c	t	c	a	c	a	a	a	t	t	a	a	t	c	a	a	a	c	c	t	t	g	c	t	t	a	c	-	t	c	c	g	c	a	a	n	n	n	n	c	t	a
KF853230	Vac	t	g	t	a	g	c	t	a	g	g	a	t	t	t	g	t	t	a	g	c	c	c	t	c	a	c	a	a	a	t	t	a	a	t	c	a	a	a	c	c	t	t	g	c	t	t	a	c	-	t	c	c	g	c	a	a	n	n	n	n	c	t	a
KF853231	Vac	t	g	t	a	a	c	t	a	g	g	a	t	t	t	g	t	t	a	g	c	c	c	t	c	a	c	a	a	a	t	t	a	a	t	c	a	a	a	c	c	t	t	g	c	t	t	a	c	-	t	c	c	g	c	a	a	n	n	n	n	c	t	a
KF853232	Vac	t	g	t	a	a	c	t	a	g	g	a	t	t	t	g	t	t	a	g	c	c	c	t	c	a	c	a	a	a	t	t	a	a	t	c	a	a	a	c	c	t	t	g	c	t	t	a	c	-	t	c	c	g	c	a	a	n	n	n	n	c	t	a
KF853233	Vac	t	g	t	a	a	c	t	a	g	g	a	t	t	t	g	t	t	a	g	c	c	c	t	c	a	c	a	a	a	t	t	a	a	t	c	a	a	a	c	c	t	t	g	c	t	t	a	c	-	t	c	c	g	c	a	a	n	n	n	n	c	t	a
KF853234	Vac	t	g	t	a	g	c	t	a	g	g	a	t	t	t	g	t	t	a	g	c	c	c	t	c	a	c	a	a	a	t	t	a	a	t	c	a	a	a	c	c	t	t	g	c	t	t	a	c	-	t	c	c	g	c	a	a	n	n	n	n	c	t	a
KF853235	Vac	t	g	t	a	g	c	t	a	g	g	a	t	t	t	g	t	t	a	g	c	c	c	t	c	a	c	a	a	a	t	t	a	a	t	c	a	a	a	c	c	t	t	g	c	t	t	a	c	-	t	c	c	g	c	a	a	n	n	n	n	c	t	a
MF898328	Vac	t	g	t	a	g	c	t	a	g	g	a	t	t	t	g	t	t	a	g	c	c	c	t	c	a	c	a	a	a	t	t	a	a	t	c	a	a	a	c	c	t	t	g	c	t	t	a	c	a	t	c	c	g	c	a	a	c	a	a	g	c	t	a
JN704709	VIII	t	g	t	a	a	c	t	a	g	g	a	t	t	c	g	t	t	a	g	c	c	c	t	c	a	c	a	a	a	t	t	a	a	t	c	a	a	a	c	c	t	t	g	c	t	t	a	c	-	t	c	c	g	c	a	a	c	a	g	g	c	t	a

Supplementary Table 3: 2880 SN

GB-ID	Clade markers								9																																																						
	Position in Dumas	102324	102351	102401	102403	102436	102438	102458	102510	102530	102563	102567	102575	102583	102596	102601	102617	102684	102738	102799	102850	102856	102908	102918_1	102948	102968	102969	102976	103037	103043	103059	103169	103205	103261	103295	103322	103602	103613	103754	103778	103797	103799	103805	103831	103880	103896	103955	104047	104061	104120	104135	104156	104204	104275	104402	104405	104465	104470	104472	104477	104542	104574	104593	104635
AY548170	1	c	a	g	a	t	t	a	t	t	a	t	g	c	c	t	t	a	t	g	g	c	c	-	a	a	g	c	g	t	a	t	t	t	g	a	g	c	t	t	a	g	t	c	t	a	t	c	g	t	g	g	a	c	a	g	g	c	a	g	a	a	a	t
AY548171	1	c	a	g	a	t	t	a	t	t	a	t	a	c	c	t	c	a	t	g	g	c	c	-	a	a	a	c	g	t	a	t	t	t	g	a	g	c	t	t	a	g	t	c	t	a	t	c	g	t	g	g	a	c	a	a	g	c	a	g	a	a	a	t
DQ479953	1	c	a	g	c	t	t	a	t	t	a	t	a	c	c	t	c	a	t	g	g	c	c	-	a	a	a	c	g	t	a	t	t	t	g	a	g	c	t	t	a	g	t	c	t	a	t	c	g	t	g	g	a	c	a	g	g	c	a	g	a	a	a	t
DQ479954	1	c	a	g	c	t	t	a	t	t	a	t	a	c	c	t	c	a	t	g	g	c	c	-	a	a	a	c	g	t	a	t	t	t	g	a	g	c	t	t	a	g	t	c	t	a	t	c	g	t	g	g	a	c	a	g	g	c	a	g	a	a	a	t
DQ479958	1	c	a	g	a	t	t	a	t	t	a	t	a	c	c	t	c	a	t	g	g	c	c	-	a	a	a	c	g	t	a	t	t	t	g	a	g	c	t	t	a	g	t	c	t	a	t	c	g	t	g	g	a	c	a	a	g	c	a	g	a	a	a	t
DQ479959	1	c	a	g	a	t	t	a	t	t	a	t	a	c	c	t	c	a	t	g	g	c	c	-	a	a	a	c	g	t	a	t	t	t	g	a	g	c	t	t	a	g	t	c	t	a	t	c	g	t	g	g	a	c	a	a	g	c	a	g	a	a	a	t
DQ479961	1	c	a	g	c	t	t	a	t	t	a	t	a	c	c	t	c	a	t	g	g	c	c	-	a	a	a	c	g	t	a	t	t	t	g	a	g	c	t	t	a	g	t	c	t	a	t	c	g	t	g	g	a	c	a	g	g	c	a	g	a	a	a	t
DQ479962	1	c	a	g	c	t	t	a	t	t	a	t	a	c	c	t	c	a	t	g	g	c	c	-	a	a	a	c	g	t	a	t	t	t	g	a	g	c	t	t	a	g	t	c	t	a	t	c	g	t	g	g	a	c	a	g	a	c	a	g	a	a	a	t
DQ479963	1	c	a	g	c	t	t	a	t	t	a	t	a	c	c	t	c	a	t	g	g	c	c	-	a	a	a	c	g	t	a	t	t	t	g	a	g	c	t	t	a	g	t	c	t	a	t	c	g	t	g	g	a	c	a	g	g	c	a	g	a	a	a	t
DQ674250	1	c	a	g	a	t	t	a	t	t	a	t	a	c	c	t	c	a	t	g	g	c	c	-	a	a	a	c	g	t	a	t	t	t	g	a	g	c	t	t	a	g	t	c	t	a	t	c	g	t	g	g	a	c	a	g	g	c	a	g	a	a	a	t
KX262864	1	c	a	g	a	t	t	a	t	t	a	t	a	c	c	t	c	a	t	g	g	c	c	-	a	a	a	c	g	t	a	t	t	t	g	a	g	c	t	t	a	g	t	c	t	a	t	c	g	t	g	g	a	c	a	a	g	c	a	g	a	a	a	t
KX262865	1	c	a	g	a	t	t	a	t	t	a	t	a	c	c	t	c	a	t	g	g	c	c	-	a	a	a	c	g	t	a	t	t	t	g	a	g	c	t	t	a	g	t	c	t	a	t	c	g	t	g	g	c	c	a	g	g	c	a	g	a	a	a	t
KX262866	1	c	a	g	a	t	t	a	t	t	a	t	a	c	c	t	c	a	t	g	g	c	c	-	a	a	a	c	g	t	a	t	t	t	g	a	g	c	t	t	a	g	t	c	t	a	t	c	g	t	g	g	a	c	a	a	g	c	a	g	a	a	a	t
EU154348	1	c	a	g	c	t	t	a	t	t	a	t	a	c	c	t	c	a	t	g	g	c	c	-	a	a	a	c	g	t	a	t	t	t	g	a	g	c	t	t	a	g	t	c	t	a	t	c	g	t	g	g	a	c	a	g	g	c	a	g	a	a	a	t
JN704690	1	c	a	g	a	t	t	a	t	t	a	t	a	c	c	t	c	a	t	g	g	c	c	-	a	a	a	c	g	t	a	t	t	t	g	a	g	c	t	t	a	g	t	c	t	a	t	c	g	t	g	g	a	c	a	g	g	c	a	g	a	a	a	t
JN704691	1	c	a	g	a	t	t	a	t	t	a	t	a	c	c	t	c	a	t	g	g	c	c	-	a	a	a	c	g	t	a	t	t	t	g	a	g	c	t	t	a	g	t	c	t	a	t	c	g	t	g	a	a	c	a	g	g	c	a	g	a	a	a	t
JN704692	1	c	a	g	a	t	t	a	t	t	a	t	a	c	c	t	c	a	t	g	g	c	c	-	a	a	a	c	g	t	a	t	t	t	g	a	g	c	t	t	a	g	t	c	t	a	t	c	g	t	g	g	a	c	a	g	g	c	a	g	a	a	a	t
JN704693	1	c	a	g	g	t	t	a	t	t	a	t	a	c	c	t	c	a	t	g	g	c	c	-	a	a	a	c	g	t	a	t	t	t	g	a	g	c	t	t	a	g	c	c	t	a	t	c	g	t	g	g	a	c	a	g	g	c	a	g	a	a	a	t
JN704694	1	c	a	a	a	t	t	a	t	t	a	t	g	c	c	t	t	a	t	g	g	c	c	-	a	a	g	c	g	t	a	t	t	t	g	a	g	c	t	t	a	g	t	c	t	a	t	c	g	t	g	g	a	c	a	g	g	c	a	g	a	a	a	t
JN704695	1	c	a	g	a	t	t	a	t	t	a	t	a	c	c	t	c	a	t	g	g	c	c	-	a	a	a	c	g	t	a	t	t	t	g	a	g	c	t	t	a	g	t	c	t	a	t	c	g	t	g	g	a	c	a	g	g	c	a	g	a	a	a	t
JN704696	1	c	a	g	a	t	t	a	t	t	a	t	a	c	c	t	c	a	t	g	g	c	c	-	a	a	a	c	g	t	a	t	t	t	g	a	g	c	t	t	a	g	t	c	t	a	t	c	g	t	g	g	a	c	a	g	g	c	a	g	a	a	a	t
NC001348	1	c	a	g	a	t	t	a	t	t	a	t	a	c	c	t	c	a	t	g	g	c	c	-	a	a	a	c	g	t	a	t	t	t	g	a	g	c	t	t	a	g	t	c	t	a	t	c	g	t	g	g	a	c	a	g	g	c	a	g	a	a	a	t
KP771892	1	c	a	g	a	t	t	a	t	t	a	t	a	c	c	t	c	a	t	g	g	c	c	-	a	a	a	c	g	t	a	t	t	t	g	a	g	c	t	t	a	g	t	c	t	a	t	c	g	t	g	g	a	c	a	g	g	c	a	g	a	a	a	t
KP771894	1	c	a	g	a	t	t	a	t	t	a	t	a	c	c	t	c	a	t	g	g	c	c	-	a	a	a	c	g	t	a	t	t	t	g	a	g	c	t	t	a	g	t	c	t	a	t	c	g	t	g	g	a	c	a	g	g	c	a	g	a	a	a	t
KP771900	1	c	a	g	c	t	t	a	t	t	a	t	a	c	c	t	c	a	t	g	g	c	c	-	a	a	a	c	g	t	a	t	t	t	g	a	g	c	t	t	a	g	t	c	t	a	t	c	g	t	g	g	a	c	a	g	g	c	a	g	a	a	a	t
KP771901	1	c	a	g	c	t	t	a	t	t	a	t	a	c	c	t	c	a	t	g	g	c	c	-	a	a	a	c	g	t	a	t	t	t	g	a	g	c	t	t	a	g	t	c	t	a	t	c	g	t	g	g	a	c	a	g	g	c	a	a	a	a	a	t
KP771902	1	c	a	g	c	t	t	a	t	t	a	t	a	c	c	t	c	a	t	g	g	c	c	-	a	a	a	c	g	t	a	t	t	t	g	a	g	c	t	t	a	g	t	c	t	a	t	c	g	t	g	g	a	c	a	g	g	c	a	g	a	a	a	t
KP771908	1	c	a	g	a	t	t	a	t	t	a	t	a	c	c	t	c	a	t	g	g	c	c	-	a	a	a	c	g	t	a	t	t	t	g	a	g	c	t	t	a	g	t	c	t	a	t	c	g	t	g	g	a	c	a	a	g	c	a	g	a	a	a	t
KP771912	1	c	a	g	a	t	t	a	t	t	a	t	a	c	c	t	c	a	t	g	g	c	c	-	a	a	a	c	g	t	a	t	t	t	g	a	g	c	t	t	a	g	t	c	t	a	t	c	g	t	g	g	a	c	a	a	g	c	a	g	a	a	a	t
KP771913	1	c	a	g	a	t	t	a	t	t	a	t	a	c	c	t	c	a	t	g	g	c	c	-	a	a	a	c	g	t	a	t	t	t	g	a	g	c	t	t	a	g	t	c	t	a	t	c	g	t	g	g	a	c	a	a	g	c	a	g	a	a	a	t
KP771917	1	c	a	g	a	t	t	a	t	t	a	t	a	c	c	t	c	a	t	g	g	c	c	-	a	a	a	c	g	t	a	t	t	t	g	a	g	c	t	t	a	g	t	c	t	a	t	c	g	t	g	g	a	c	a	g	g	c	a	g	a	a	a	t
KP771918	1	c	a	g	a	t	t	a	t	t	a	t	a	c	c	t	c	a	t	g	g	c	c	-	a	a	a	c	g	t	a	t	t	t	g	a	g	c	t	t	a	g	t	c	t	a	t	c	g	t	g	g	a	c	a	g	g	c	a	g	a	a	a	t
KP771920	1	c	a	g	c	t	t	a	t	t	a	t	a	c	c	t	c	a	t	g	g	c	c	-	a	a	a	c	g	t	a	t	t	t	g	a	g	c	t	t	a	g	t	c	t	a	t	c	g	c	g	g	a	c	a	g	g	c	a	g	a	a	a	t
MH709361	1	c	a	g	a	t	t	a	t	t	a	t	a	c	c	t	c	a	t	g	g	c	c	-	a	a	a	c	g	t	a	t	t	t	g	a	g	c	t	t	a	g	t	c	t	a	t	c	g	t	g	g	a	c	a	a	g	c	a	g	a	a	a	t
KU926318	2	c	c	g	a	t	t	g	t	t	a	t	a	c	c	g	c	a	t	g	g	c	c	-	a	g	a	c	g	c	g	t	t	t	g	a	g	c	t	t	g	g	t	c	t	g	t	c	g	t	g	g	a	c	a	g	g	c	a	g	a	g	a	t
KU926319	2	c	c	g	a	t	t	g	t	t	a	t	a	c	c	g	c	a	t	g	g	c	c	-	a	a	a	c	g	c	a	t	t	t	g	a	g	c	t	t	a	g	t	c	t	a	t	c	g	t	g	g	a	c	a	g	g	c	a	g	a	a	a	t
KU926320	2	c	c	g	a	t	t	g	t	t	a	t	a	c	c	g	c	a	t	g	g	c	c	-	a	g	a	c	g	c	a	t	t	t	g	a	g	c	t	t	g	g	t	c	t	a	t	c	g	t	g	g	a	c	a	g	g	c	a	g	g	a	a	t
KU926321	2	c	c	g	a	t	t	g	t	t	a	t	a	c	c	g	c	a	t	g	g	c	c	-	a	a	a	c	g	c	a	t	t	t	g	a	g	c	t	t	a	g	t	c	t	a	t	c	g	t	g	g	a	c	a	g	g	c	a	g	a	a	a	t
KU926322	2	c	c	g	a	t	t	g	t	t	a	t	a	c	c	g	c	a	t	g	g	c	c	-	a	a	a	c	g	c	a	t	t	t	g	a	g	c	t	t	a	g	t	c	t	a	t	c	g	t	g	g	a	c	a	g	g	c	a	g	a	a	a	t
KC847290	2	c	c	g	a	t	t	g	t	t	a	t	a	c	c	g	c	a	t	g	g	c	c	-	a	a	a	c	g	c	a	t	t	t	g	a	g	c	t	t	a	g	t	c	t	a	t	c	g	t	g	g	a	c	a	g	g	c	a	g	a	a	a	t
KJ767491	2	c	c	g	a	t	t	g	t	t	a	t	a	c	c	g	c	a	t	g	g	c	c	-	a	a	a	c	g	c	a	t	t	t	g	a	g	c	t	t	a	g	t	c	t	a	t	c	g	t	g	g	a	c	a	g	g	c	a	g	a	a	a	t
KJ767492	2	c	c	g	a	t	t	g	t	t	a	t	a	c	c	g	c	a	t	g	g	c	c	-	a	a	a	c	g	c	a	t	t	t	g	a	g	c	t	t	a	g	t	c	t	a	t	c	g	t	g	g	a	c	a	g	g	c	a	g	a	a	a	t
KJ808816	2	c	c	g	a	t	t	g	t	t	a	t	a	c	c	g	c	a	t	g	g	c	c	-	a	a	a	c	g	c	a	t	t	t	g	a	g	c	t	t	a	g	t	c	t	a	t	c	g	t	g	g	a	c	a	g	g	c	a	g	a	a	a	t
KP771925	2	c	c	g	a	t	t	g	t	t	a	t	a	c	c	g	c	a	t	g	g	c	c	-	a	a	a	c	g	c	a	t	t	t	g	a	g	c	t	t	a	g	t	c	t	a	t	c	g	t	g	g	a	c	a	g	g	c	a	g	a	a	a	t
KU926315	2	c	c	g	a	t	t	g	t	t	a	t	a	c	c	g	c	a	t	g	g	c	c	-	a	a	a	c	g	c	a	t	t	t	g	a	g	c	t	t	a	g	t	c	t	a	t	c	g	t	g	g	a	c	a	g	g	c	a	g	a	a	a	t
KU926316	2	c	c	g	a	t	t	g	t	t	a	t	a	c	c	g	c	a	t	g	g	c	c	-	a	a	a	c	g	c	a	t	t	t	g	a	g	c	t	t	a	g	t	c	t	a	t	c	g	t	g	g	a	c	a	g	g	c	a	g	a	a	a	t
KU926317	2	c	c	g	a	t	t	g	t	t	a	t	a	c	c	g	c	a	t	g	g	c	c	-	a	a	a	c	g	c	a	t	t	t	g	a	g	c	t	t	a	g	t	c	t	a	t	c	g	t	g	g	a	c	a	g	g	c	a	g	a	a	a	t
KY037796	2	c	c	g	a	t	t	g	t	t	a	t	a	c	c	g	c	a	t	g	g	c	c	-	a	a	a	c	g	c	a	t	t	t	g	a	g	c	t	t	a	g	t	c	t	a	t	c	g	t	g	g	a	c	a	g	g	c	a	g	a	a	a	t
MF004348	2	c	c	g	a	t	t	g	t	t	a	t	a	c	c	g	c	a	t	g	g	c	c	-	a	a	a	c	g	c	a	t	t	t	g	a	g	c	t	t	a	g	t	c	t	a	t	c	g	t	g	g	a	c	a	g	g	c	a	g	a	a	a	t
MH709309	2	c	c	g	a	t	t	g	t	t	a	t	a	c	c	g	c	a	t	g	g	c	c	-	a	a	a	c	g	c	a	t	t	t	g	a	g	c	t	t	a	g	t	c	t	a	t	c	g	t	g	g	a	c	a	g	g	c	a	g	a	a	a	t
MH709310	2	c	c	g	a	t	t	g	t	t	a	t	a	c	c	g	c	a	t	g	g	c	c	-	a	a	a	c	g	c	a	t	t	t	g	a	g	c	t	t	a	g	t	c	t	a	t	c	g	t	g	g	a	c	a	g	g	c	a	g	a	a	a	t
MH709311	2	c	c	g	a	c	g	g	t	t	a	t	a	c	c	g	c	a	t	g	g	c	c	-	a	a	a	c	g	c	a	t	t	t	g	a	g	c	t	t	a	g	t	a	t	a	t	c	g	t	g	g	a	c	a	g	g	c	a	g	a	a	a	t
MH709313	2	c	c	g	a	t	t	g	t	t	a	t	a	c	c	g	c	a	t	g	g	c	c	-	a	a	a	c	g	c	a	t	t	t	g	a	g	c	t	t	a	g	t	c	t	a	t	c	g	t	g	g	a	c	a	g	g	c	a	g	a	a	a	t
MH709314	2	c	c	g	a	t	t	g	t	t	a	t	a	c	c	g	c	a	t	g	g	c	c	-	a	a	a	c	g	c	a	t	t	t	g	a	g	c	t	t	a	g	t	c	t	a	t	c	g	t	g	g	a	c	a	g	g	c	a	g	a	a	a	t
MH709315	2	c	c	g	a	t	t	g	t	t	a	t	a	c	c	g	c	a	t	g	g	c	c	-	a	a	a	c	g	c	a	t	t	t	g	a	g	c	t	t	a	g	t	c	t	a	t	c	g	t	g	g	a	c	a	g	g	c	a	g	a	a	a	t
MH709319	2	c	c	g	a	t	t	g	t	t	a	t	a	c	c	g	c	a	t	g	g	c	c	-	a	a	a	c	g	c	a	t	t	t	g	a	g	c	t	t	a	g	t	c	t	a	t	c	g	t	g	g	a	c	a	g	g	c	a	g	a	a	a	t
MH709320	2	c	c	g	a	t	t	g	t	c	a	t	a	c	c	g	c	a	t	g	g	c	c	-	a	a	a	c	g	c	a	t	t	t	g	a	g	c	t	t	a	g	t	c	t	a	t	c	g	t	g	g	a	c	a	g	g	c	a	g	a	a	a	t
MH709329	2	c	c	g	a	t	t	g	t	t	a	t	a	c	c	g	c	a	t	g	g	c	c	-	a	a	a	c	g	c	a	t	t	t	g	a	g	c	t	t	a	g	t	c	t	a	t	c	g	t	g	g	a	c	a	g	g	c	a	g	a	a	a	t
MH709331	2	c	c	g	a	t	t	g	t	t	a	t	a	c	c	g	c	a	t	g	g	c	c	-	a	a	a	c	a	c	a	t	t	t	g	a	g	c	t	t	a	g	t	c	t	a	t	c	g	t	g	g	a	c	a	g	g	c	a	g	a	a	a	t
MH709333	2	c	c	g	a	t	t	g	t	t	a	t	a	c	c	g	c	a	t	g	g	c	c	-	a	a	a	c	g	c	a	t	t	t	g	a	g	c	t	t	a	g	t	c	t	a	t	c	g	t	g	g	a	c	a	g	g	c	a	g	a	a	a	t
MH709337	2	c	c	g	a	t	t	g	t	t	a	t	a	c	c	g	c	a	t	g	g	c	c	-	a	a	a	c	g	c	a	t	t	t	g	a	g	c	t	t	a	g	t	c	t	a	t	c	g	t	g	g	a	c	a	g	g	c	a	g	a	a	a	t
MH709339	2	c	c	g	a	t	t	g	t	t	a	t	a	c	c	g	c	a	t	g	g	c	c	-	a	a	a	c	g	c	a	t	t	t	g	a	g	c	t	t	a	g	t	c	t	a	t	c	g	t	g	g	a	c	a	g	g	c	a	g	a	a	a	t
MH709344	2	c	c	g	a	t	t	g	t	t	a	t	a	c	c	g	c	a	t	g	g	c	c	-	a	a	a	c	g	c	a	t	t	t	g	a	g	c	t	t	a	g	t	c	t	a	t	c	g	t	g	g	a	c	a	g	g	c	a	g	a	a	a	t
MH709348	2	c	c	g	a	t	t	g	t	t	a	t	a	c	c	g	c	a	t	g	g	c	c	-	a	a	a	c	g	c	a	t	t	t	g	a	g	c	t	t	a	g	t	c	t	a	t	c	g	t	g	g	a	c	a	g	g	c	a	g	a	a	a	t
MH709355	2	c	c	g	a	t	t	g	t	t	a	t	a	c	c	g	c	a	t	g	g	c	c	-	a	a	a	c	g	c	a	t	t	t	g	a	g	c	t	t	a	g	t	c	t	a	t	c	g	t	g	g	a	c	a	g	g	c	a	g	a	a	a	t
MH709359	2	c	c	g	a	t	t	g	t	t	a	t	a	c	c	g	c	a	t	g	g	c	c	-	a	a	a	c	g	c	a	t	t	t	g	a	g	c	t	t	a	g	t	c	t	a	t	c	g	t	g	g	a	c	a	g	g	c	a	g	a	a	a	t
MH709363	2	c	c	g	a	t	t	g	t	c	a	t	a	c	c	g	c	a	t	g	g	c	c	-	a	a	a	c	g	c	a	t	t	t	g	a	g	c	t	t	a	g	t	c	t	a	t	c	g	t	g	g	a	c	a	g	g	c	a	g	a	a	a	t
MH709366	2	c	c	g	a	t	t	g	t	t	a	t	a	c	c	g	c	a	t	g	g	c	c	-	a	a	a	c	g	c	a	t	t	t	g	a	g	c	t	t	a	g	t	c	t	a	t	c	g	t	g	g	a	c	a	g	g	c	a	g	a	a	a	t
MH709376	2	c	c	g	a	t	t	g	t	t	a	t	a	c	c	g	c	a	t	g	g	c	c	-	a	a	a	c	g	c	a	t	t	t	g	a	g	c	t	t	a	g	t	c	t	a	t	c	g	t	g	g	a	c	a	g	g	c	a	g	a	a	a	t
MH709377	2	c	c	g	a	t	t	g	t	t	a	t	a	c	c	g	c	a	t	a	g	c	c	-	a	a	a	c	g	c	a	t	t	t	g	a	g	c	t	t	a	g	t	c	t	a	t	c	g	t	g	g	a	c	a	g	g	c	a	g	a	a	a	t
**MW316406**	2	c	c	g	a	t	t	g	t	t	a	t	a	c	c	g	c	a	t	g	g	c	c	-	a	a	a	c	g	c	a	t	t	t	g	a	g	c	t	t	a	g	t	c	t	a	t	c	g	t	g	g	a	c	a	g	g	c	a	g	a	a	a	t
AJ871403	3	c	a	g	a	t	t	a	t	t	a	t	a	c	c	t	c	a	t	g	g	c	c	-	a	a	a	c	g	t	a	t	t	t	g	a	g	c	t	t	a	g	t	c	t	a	g	c	g	t	g	g	a	c	a	g	g	c	a	g	a	a	a	t
DQ479955	3	c	a	g	a	t	t	a	t	t	a	t	a	c	c	t	c	a	t	g	g	c	c	-	a	a	a	c	g	t	a	t	t	t	g	a	g	c	t	t	a	g	t	c	t	a	t	c	g	t	g	g	a	c	a	g	g	c	g	g	a	a	a	t
DQ479956	3	c	a	g	a	t	t	a	t	t	a	t	a	c	c	t	c	a	t	g	g	c	c	-	a	a	a	c	g	t	a	t	t	c	g	a	g	c	t	t	a	g	t	c	t	a	t	c	g	t	g	g	a	c	a	g	g	c	a	g	a	a	a	t
DQ479957	3	c	a	g	a	t	t	a	t	t	a	t	a	c	c	t	c	a	t	g	g	c	c	-	a	a	a	c	g	t	a	t	t	t	a	a	g	c	t	t	a	g	t	c	t	a	t	c	g	t	g	g	a	c	a	g	g	c	a	g	a	a	a	t
JN704699	3	c	a	g	a	t	t	a	t	t	a	t	a	c	c	t	c	a	t	g	g	c	c	-	a	a	a	c	g	t	a	t	t	t	g	a	g	c	t	t	a	g	t	c	t	a	t	c	g	t	g	g	a	c	a	g	g	c	a	g	a	a	a	t
JN704700	3	c	a	g	a	t	t	a	t	t	a	t	a	c	c	t	c	a	t	g	g	c	c	-	a	a	a	c	g	t	a	t	t	t	a	a	g	c	t	t	a	g	t	c	t	a	t	c	g	t	g	g	a	c	a	g	g	c	a	g	a	a	a	t
JN704701	3	c	a	g	a	t	t	a	t	t	a	t	a	c	c	t	c	a	t	g	g	c	c	-	a	a	a	c	g	t	a	t	t	t	g	a	g	c	t	t	a	g	t	c	t	a	t	c	g	t	g	g	a	c	a	g	g	c	a	g	a	a	a	t
JN704702	3	c	a	g	a	t	t	a	t	t	a	t	a	c	c	t	c	a	t	g	g	c	c	-	a	a	a	c	g	t	a	t	t	t	g	a	g	c	t	t	a	g	t	c	t	a	t	t	g	t	g	g	a	c	a	g	g	c	a	g	a	a	a	t
JN704703	3	c	a	g	a	t	t	a	t	t	a	t	a	c	c	t	c	a	t	g	g	c	c	-	a	a	a	c	g	t	a	t	t	t	g	a	g	c	t	t	a	g	t	c	t	a	t	c	g	t	g	g	a	c	a	g	g	c	a	g	a	a	a	t
KP771891	3	c	a	g	a	t	t	a	t	t	a	t	a	c	c	t	c	a	t	g	g	c	c	-	a	a	a	c	g	t	a	t	t	t	a	a	g	c	t	t	a	g	t	c	t	a	t	c	g	t	g	g	a	c	a	g	g	c	a	g	a	a	a	t
KP771893	3	c	a	g	a	t	t	a	t	t	a	t	a	c	c	t	c	a	t	g	g	c	c	-	a	a	a	c	g	t	a	t	t	t	a	a	g	c	t	t	a	g	t	c	t	a	t	c	g	t	g	g	a	c	a	g	g	c	a	g	a	a	a	t
KP771895	3	c	a	g	a	t	t	a	t	t	a	t	a	c	c	t	c	t	t	g	g	c	c	-	a	a	a	c	g	t	a	t	t	t	g	a	g	c	t	t	a	g	t	c	t	a	t	c	g	t	g	g	a	c	a	g	g	c	a	g	a	a	a	t
KP771896	3	c	a	g	a	t	t	a	t	t	a	t	a	c	c	t	c	a	t	g	g	c	c	-	a	a	a	c	g	t	a	t	t	t	g	a	g	c	t	t	a	g	t	c	t	a	t	c	g	t	g	g	a	c	a	g	g	c	a	g	a	a	a	t
KP771897	3	c	a	g	a	t	t	a	t	t	a	t	a	c	c	t	c	a	t	g	g	c	c	-	a	a	a	c	g	t	a	t	t	t	g	a	g	c	t	t	a	g	t	c	t	a	t	c	g	t	g	g	a	c	a	g	g	c	a	g	a	a	a	t
KP771899	3	c	a	g	a	t	t	a	t	t	a	t	a	c	c	t	c	a	t	g	g	c	c	-	a	a	a	c	g	t	a	t	t	t	g	a	g	c	t	t	a	g	t	c	t	a	t	c	g	t	g	g	a	c	a	g	g	c	a	g	a	a	a	t
KP771903	3	c	a	g	a	t	t	a	t	t	a	t	a	c	c	t	c	a	t	g	g	c	c	-	a	a	a	c	g	t	a	t	t	t	a	a	g	c	t	t	a	g	t	c	t	a	t	c	g	t	g	g	a	c	a	g	g	c	a	g	a	a	a	t
KP771907	3	c	a	g	a	t	t	a	t	t	a	t	a	c	c	t	c	a	t	g	g	c	c	-	a	a	a	c	g	t	a	t	t	t	g	a	g	c	t	t	a	g	t	c	t	a	t	c	g	t	g	g	a	c	a	g	g	c	a	g	a	a	a	t
KP771910	3	c	a	g	a	t	t	a	t	t	a	t	a	c	c	t	c	a	t	g	g	c	c	-	a	a	a	c	g	t	a	t	t	t	a	a	g	c	t	t	a	g	t	c	t	a	t	c	g	t	g	g	a	c	a	g	g	c	a	g	a	a	a	t
KP771915	3	c	a	g	a	t	t	a	t	t	a	t	a	c	c	t	c	a	t	g	g	c	c	-	a	a	a	c	g	t	a	t	t	t	g	a	g	c	t	t	a	g	t	c	t	a	t	c	g	t	g	g	a	c	a	g	g	c	a	g	a	a	a	t
KP771916	3	c	a	g	a	t	t	a	t	t	a	t	a	c	c	t	c	a	t	g	g	c	c	g	a	a	a	c	g	t	a	t	t	t	g	a	g	c	t	t	a	g	t	c	t	a	t	c	g	t	g	g	a	c	a	g	g	c	a	g	a	a	a	t
KP771921	3	c	a	g	a	t	t	a	t	t	a	t	a	c	c	t	c	a	t	g	g	c	c	-	a	a	a	c	g	t	a	t	t	t	a	a	g	c	t	t	a	g	t	c	t	a	t	c	g	t	g	g	a	t	a	g	g	c	a	g	a	a	a	t
KP771923	3	c	a	g	a	t	t	a	t	t	a	t	a	c	c	t	c	a	t	g	g	c	c	-	a	a	a	c	g	t	a	t	t	t	a	a	g	c	t	t	a	g	t	c	t	a	t	c	g	t	g	g	a	c	a	g	g	c	a	g	a	a	a	t
KP771889	3	c	a	g	a	t	t	a	t	t	a	t	a	c	c	t	c	a	t	g	g	c	c	-	a	a	a	c	g	t	a	t	t	t	g	a	g	c	t	t	a	g	t	c	t	a	t	c	g	t	g	g	a	c	a	g	g	c	a	g	a	a	a	t
KU926311	3	c	a	g	a	t	t	a	t	t	a	t	a	c	c	t	c	a	t	g	g	c	c	-	a	a	a	c	g	t	a	t	t	t	g	a	g	c	t	t	a	g	t	c	t	a	t	c	g	t	g	g	a	c	a	g	g	t	a	g	a	a	a	t
JQ911599	3	c	a	g	a	t	t	a	t	t	a	t	a	-	c	t	c	a	t	g	g	c	c	-	a	a	a	c	g	t	a	t	t	t	g	a	g	c	t	t	a	g	t	c	t	a	t	c	g	t	a	g	a	c	a	g	g	c	a	g	a	a	a	t
MH709317	3	c	a	g	a	t	t	a	t	t	a	t	a	c	c	t	c	a	t	g	g	c	c	-	c	a	a	c	g	t	a	t	t	t	g	a	g	c	t	t	a	g	t	c	t	a	t	c	g	t	g	g	a	c	a	g	g	c	a	g	a	a	a	t
MH709346	3	c	a	g	a	t	t	a	t	t	a	t	a	c	c	t	c	a	t	g	g	c	c	-	a	a	a	c	g	t	a	t	t	t	g	a	g	c	t	t	a	g	t	c	t	a	t	c	g	t	g	g	a	c	a	g	g	c	a	g	a	a	a	t
MH709375	3	c	a	g	a	t	t	a	t	t	a	t	a	c	c	t	c	a	t	g	g	c	c	-	a	a	a	c	g	t	a	t	t	t	g	a	g	c	t	t	a	g	t	c	t	a	t	c	g	t	g	g	a	c	a	g	g	c	a	g	a	a	a	t
DQ452050	4	c	c	g	a	t	t	g	t	t	a	t	a	c	c	g	c	a	t	g	g	c	c	-	a	a	a	c	g	c	a	t	t	t	g	a	g	c	t	t	a	g	t	c	t	a	t	c	g	t	g	g	a	c	a	g	g	c	a	g	a	a	a	t
DQ479960	4	c	c	g	a	t	t	g	t	t	a	t	a	c	c	g	c	a	t	g	g	c	c	-	a	a	a	c	g	c	a	t	t	t	g	g	g	c	t	t	a	g	t	c	t	a	t	c	g	t	g	g	a	c	g	g	g	c	a	g	a	a	a	t
KP771898	4	c	c	g	a	t	t	g	t	t	a	t	a	c	c	g	c	a	t	g	g	c	c	-	a	a	a	c	g	c	a	t	t	t	g	a	g	c	t	t	a	g	t	c	t	a	t	c	g	t	g	g	a	c	a	g	g	c	a	g	a	a	a	t
MH709316	4	c	c	g	a	t	t	g	t	t	a	t	a	c	c	g	c	a	t	g	g	c	c	-	a	a	a	c	g	c	a	t	t	t	g	a	t	c	t	t	a	g	t	c	t	a	t	c	g	t	g	g	a	c	a	g	g	c	a	g	a	a	a	t
MH709318	4	c	c	g	a	t	t	g	t	t	a	t	a	c	c	g	c	a	t	g	g	c	c	-	a	a	a	c	g	c	a	t	t	t	g	a	g	c	t	t	a	g	t	c	t	a	t	c	g	t	g	g	a	c	a	g	g	c	a	g	a	a	a	t
MH709322	4	c	c	g	a	t	t	g	t	t	a	t	a	c	c	g	c	a	t	g	g	c	c	-	a	a	a	c	g	c	a	t	t	t	g	a	g	c	t	t	a	g	t	c	t	a	t	c	g	t	g	g	a	c	a	g	g	c	a	g	a	a	a	t
MH709323	4	c	c	g	a	t	t	g	t	t	a	t	a	c	c	g	c	a	t	g	g	c	c	-	a	a	a	c	g	c	a	t	t	t	g	a	g	c	t	t	a	g	t	c	t	a	t	c	a	t	g	g	a	c	a	g	g	c	a	g	a	a	a	t
MH709326	4	c	c	g	a	t	t	g	t	t	a	t	a	c	c	g	c	a	t	g	g	c	c	-	a	a	a	c	g	c	a	t	t	t	g	a	g	c	t	t	a	g	t	c	t	a	t	c	g	t	g	g	a	c	a	g	g	c	a	g	a	a	a	t
MH709341	4	c	c	g	a	t	t	g	t	t	a	t	a	c	c	g	c	a	t	g	g	c	c	-	a	a	a	c	g	c	a	t	t	t	g	a	g	c	t	t	a	g	t	c	t	a	t	c	g	t	g	g	a	c	a	g	g	c	a	g	a	a	a	t
MH709357	4	c	c	g	a	t	t	g	t	t	a	t	a	c	c	g	c	a	t	g	g	c	c	-	a	a	a	c	g	c	a	t	t	t	g	a	g	c	t	t	a	g	t	c	t	a	t	c	g	t	g	g	a	c	g	g	g	c	a	g	a	a	a	t
MH709364	4	c	c	g	a	t	t	g	t	t	a	t	a	c	c	g	c	a	t	g	g	c	c	-	a	a	a	c	g	c	a	t	t	t	g	a	g	c	t	t	a	g	t	c	t	a	t	c	g	t	g	g	a	c	g	g	g	c	a	g	a	a	a	t
MH709370	4	c	c	g	a	t	t	g	t	t	a	t	a	c	c	g	c	a	t	g	g	c	c	-	a	a	a	c	g	c	a	t	t	t	g	a	g	c	t	t	a	g	t	c	t	a	t	c	g	t	g	g	a	c	g	g	g	c	a	g	a	a	a	t
MH709371	4	c	c	g	a	t	t	g	t	t	a	t	a	c	c	g	c	a	t	g	g	c	c	-	a	a	a	c	g	c	a	t	t	t	g	a	g	c	t	t	a	g	t	c	t	a	t	c	g	t	g	g	a	c	g	g	g	c	a	g	a	a	a	t
MH709372	4	c	c	g	a	t	t	g	t	t	a	t	a	c	c	g	c	a	t	g	g	c	c	-	a	a	a	c	g	c	a	t	t	t	g	a	g	c	t	t	a	g	t	c	t	a	t	c	g	t	g	g	a	c	g	g	g	c	a	g	a	a	a	t
DQ457052	5	c	a	g	a	t	t	a	t	t	a	t	a	c	c	t	c	a	t	g	g	c	c	-	a	a	a	c	g	t	a	t	t	t	g	a	g	c	t	t	a	g	t	c	t	a	t	c	g	t	g	g	a	c	a	g	g	c	a	g	a	a	a	t
JN704704	5	c	a	g	a	t	t	a	t	t	a	t	a	c	c	t	c	a	t	g	g	c	c	-	a	a	a	c	g	t	a	t	t	t	g	a	g	c	t	t	a	g	t	c	t	a	t	c	g	t	g	g	a	c	a	g	g	c	a	g	a	a	a	t
JN704705	5	c	a	g	a	t	t	a	t	t	a	t	a	c	c	t	c	a	t	g	g	c	c	-	a	a	a	c	g	t	a	t	t	t	g	a	g	c	t	t	a	g	t	c	t	a	t	c	g	t	g	g	a	c	a	g	g	c	a	g	a	a	a	t
JN704706	5	c	a	g	a	t	t	a	t	t	a	t	a	c	a	t	c	a	t	g	g	c	c	-	a	a	a	c	g	t	a	t	t	t	g	a	g	c	t	t	a	g	t	c	t	a	t	c	g	t	g	g	a	c	a	g	g	c	a	g	a	a	a	t
JN704707	5	c	a	g	a	t	t	a	t	t	a	t	a	c	c	t	c	a	t	g	g	c	c	-	a	a	a	c	g	t	a	t	t	t	g	a	g	c	t	t	a	g	t	c	t	a	t	c	g	t	g	g	a	c	a	g	g	c	a	g	a	a	a	t
JN704708	5	c	a	g	a	t	t	a	t	t	a	t	a	c	c	t	c	a	t	g	g	c	c	-	a	a	a	c	g	t	a	t	t	t	g	a	g	c	t	t	a	g	t	c	t	a	t	c	g	t	g	g	a	c	a	g	g	c	a	g	a	a	a	t
KP771904	5	c	a	g	a	t	t	a	t	t	a	t	a	c	c	t	c	a	c	g	g	c	c	-	a	a	a	c	g	t	a	t	t	t	g	a	g	c	t	t	a	g	t	c	t	a	t	c	g	t	g	g	a	c	a	g	g	c	a	g	a	a	a	t
KP771905	5	c	a	g	a	t	t	a	t	t	a	t	a	c	c	t	c	a	t	g	g	c	c	-	a	a	a	c	g	t	a	t	t	t	g	a	g	a	t	c	a	g	t	c	t	a	t	c	g	t	g	g	a	c	a	g	g	c	a	g	a	a	a	t
KP771906	5	c	a	g	a	t	t	a	t	t	a	t	a	c	c	t	c	a	t	g	g	c	c	-	a	a	a	c	g	t	a	t	t	t	g	a	g	a	t	c	a	g	t	c	t	a	t	c	g	t	g	g	a	c	a	g	g	c	a	g	a	a	a	t
KP771919	5	c	a	g	a	t	t	a	t	t	a	t	a	c	c	t	c	a	c	g	g	c	c	-	a	a	a	c	g	t	a	t	c	t	g	a	g	c	t	t	a	g	t	c	t	a	t	c	g	t	g	g	a	c	a	g	g	c	a	g	a	a	a	t
KP771922	5	c	a	g	a	t	t	a	t	t	a	t	a	c	c	t	c	a	t	g	g	c	c	-	a	a	a	c	g	t	a	t	t	t	g	a	g	c	t	t	a	g	t	c	t	a	t	c	g	t	g	g	a	c	a	g	g	c	a	g	a	a	a	t
KP771924	5	c	a	g	a	t	t	a	t	t	a	t	a	c	c	t	c	a	t	g	g	c	c	-	a	a	a	c	g	t	a	t	t	t	g	a	g	c	t	t	a	g	t	c	t	a	t	c	g	t	g	g	a	c	a	g	g	c	a	g	a	a	a	t
KM355696	5	c	a	g	a	t	t	a	t	t	a	t	a	c	c	t	c	a	t	g	g	c	c	-	a	a	a	c	g	t	a	t	t	t	g	a	g	c	t	t	a	g	t	c	t	a	t	c	g	t	g	g	a	c	a	g	g	c	a	g	a	a	a	t
KM355697	5	c	a	g	a	t	t	a	t	t	a	t	a	c	c	t	c	a	t	g	g	c	c	-	a	a	a	c	g	t	a	t	t	t	g	a	g	c	t	t	a	g	t	c	t	a	t	c	g	t	g	g	a	c	a	g	g	c	a	g	a	a	a	t
KM355698	5	c	a	g	a	t	t	a	t	t	a	t	a	c	c	t	c	a	t	g	g	c	c	-	a	a	a	c	g	t	a	t	t	t	g	a	g	c	t	t	a	g	t	c	t	a	t	c	g	t	g	g	a	c	a	g	g	c	a	g	a	a	a	t
KM355699	5	c	a	g	a	t	t	a	t	t	a	t	a	c	c	t	c	a	t	g	g	c	c	-	a	a	a	c	g	t	a	t	t	t	g	a	g	c	t	t	a	g	t	c	t	a	t	c	g	t	g	g	a	c	a	g	g	c	a	g	a	a	a	t
KM355701	5	c	a	g	a	t	t	a	t	t	a	t	a	c	c	t	c	a	t	g	g	c	c	-	a	a	a	c	g	t	a	t	t	t	g	a	g	c	t	t	a	a	t	c	t	a	t	c	g	t	g	g	a	c	a	g	g	c	a	g	a	a	a	t
KM355702	5	c	a	g	a	t	t	a	t	t	a	t	a	c	c	t	c	a	t	g	g	c	c	-	a	a	a	c	g	t	a	t	t	t	g	a	g	c	t	t	a	g	t	c	t	a	t	c	g	t	g	g	a	c	a	g	g	c	a	g	a	a	a	t
KM355703	5	c	a	g	a	t	t	a	t	t	a	t	a	c	c	t	c	a	t	g	g	c	c	-	a	a	a	c	g	t	a	t	t	t	g	a	g	c	t	t	a	g	t	c	t	a	t	c	g	t	g	g	a	c	a	g	g	c	a	g	a	a	a	t
KM355704	5	c	a	g	a	t	t	a	t	t	a	t	a	c	c	t	c	a	t	g	g	c	c	-	a	a	a	c	g	t	a	t	t	t	g	a	g	c	t	t	a	g	t	c	t	a	t	c	g	t	g	g	a	c	a	g	g	c	a	g	a	a	a	t
KM355705	5	c	a	g	a	t	t	a	t	t	a	t	a	c	c	t	c	a	t	g	g	c	c	-	a	a	a	c	g	t	a	t	t	t	g	a	g	c	t	t	a	g	t	c	t	a	t	c	g	t	g	g	a	c	a	g	g	c	a	g	a	a	a	t
KM355706	5	c	a	g	a	t	t	a	t	t	a	t	a	c	c	t	c	a	t	g	g	c	c	-	a	a	a	c	g	t	a	t	t	t	g	a	g	c	t	t	a	g	t	c	t	a	t	c	g	t	g	g	a	c	a	g	g	c	a	g	a	a	a	t
KM355707	5	c	a	g	a	t	t	a	t	t	a	t	a	c	c	t	c	a	t	g	g	c	c	-	a	a	a	c	g	t	a	t	t	t	g	a	g	c	t	t	a	g	t	c	t	a	t	c	g	t	g	g	a	c	a	g	g	c	a	g	a	a	a	t
KM355708	5	c	a	g	a	t	t	a	t	t	a	t	a	c	c	t	c	a	t	g	g	c	c	-	a	a	a	c	g	t	a	t	t	t	g	a	g	c	t	t	a	g	t	c	t	a	t	c	g	t	g	g	a	c	a	g	g	c	a	g	a	a	a	t
KM355709	5	c	a	g	a	t	t	a	t	t	a	t	a	c	c	t	c	a	t	g	g	c	c	-	a	a	a	c	g	t	a	t	t	t	g	a	g	c	t	t	a	g	t	c	t	a	t	c	g	t	g	g	a	c	a	g	g	c	a	g	a	a	a	t
KM355711	5	c	a	g	a	t	t	a	t	t	a	t	a	c	c	t	c	a	t	g	g	c	c	-	a	a	a	c	g	t	a	t	t	t	g	a	g	c	t	t	a	g	t	c	t	a	t	c	g	t	g	g	a	c	a	g	g	c	a	g	a	a	a	t
KM355712	5	c	a	g	a	t	t	a	t	t	a	t	a	c	c	t	c	a	t	g	g	c	c	-	a	a	a	c	g	t	a	t	t	t	g	a	g	c	t	t	a	g	t	c	t	a	t	c	g	t	g	g	a	c	a	g	g	c	a	g	a	a	a	t
KY037797	5	c	a	g	a	t	t	a	t	t	a	t	a	c	c	t	c	a	t	g	g	c	c	-	a	a	a	c	g	t	a	t	t	t	g	a	g	c	t	t	a	g	t	c	t	a	t	c	g	t	g	g	a	c	a	g	g	c	a	g	a	a	a	t

Supplementary Table 3: 2880 SN

GB-ID	Clade markers								9												
	Position in Dumas	102324	102351	102401	102403	102436	102438	102458	102510	102530	102563	102567	102575	102583	102596	102601	102617	102684	102738	102799	102850	102856
MH499467	5	c	a	g	a	t	t	a	t	t	a	t	a	c	c	t	c	a	t	g	g	c
MH499469	5	c	a	g	a	t	t	a	t	t	g	t	a	c	c	t	c	a	c	g	g	c
MH709321	5	c	a	g	a	t	t	a	t	t	a	t	a	c	a	t	c	a	t	g	g	c
MH709324	5	c	a	g	a	t	t	a	t	t	a	t	a	c	c	t	c	a	c	g	g	c
MH709325	5	c	a	g	a	t	t	a	t	t	a	g	a	c	c	t	c	a	t	g	g	c
MH709327	5	c	a	g	a	t	t	a	t	t	a	t	a	c	c	t	c	a	t	g	g	c
MH709328	5	c	a	g	a	t	t	a	t	t	a	t	a	c	c	t	c	a	c	g	g	c
MH709330	5	c	a	g	a	t	t	a	t	t	a	t	a	c	c	t	c	a	c	g	g	c
MH709332	5	c	a	g	a	t	t	a	t	t	a	t	a	c	c	t	c	a	c	g	g	c
MH709334	5	c	a	g	a	t	t	a	t	t	a	t	a	c	c	t	c	a	t	g	g	c
MH709335	5	c	a	g	a	t	t	a	t	t	a	t	a	a	c	t	c	a	t	g	g	c
MH709336	5	c	a	g	a	t	t	a	t	t	a	t	a	c	c	t	c	a	t	g	g	c
MH709338	5	c	a	g	a	t	t	a	t	t	a	t	a	c	c	t	c	a	t	g	g	c
MH709340	5	c	a	g	a	t	t	a	t	t	a	t	g	c	c	t	c	a	t	g	g	c
MH709342	5	c	a	g	a	t	t	a	t	t	a	t	a	c	a	t	c	a	t	g	g	c
MH709343	5	c	a	g	a	t	t	a	t	t	a	t	a	c	c	t	c	a	t	g	g	c
MH709345	5	c	a	g	a	t	t	a	t	t	a	t	a	c	c	t	c	a	t	g	g	c
MH709347	5	c	a	g	a	t	t	a	t	t	a	t	a	c	c	t	c	a	t	g	g	c
MH709349	5	c	a	g	a	t	t	a	t	t	a	t	a	c	c	t	c	a	t	g	g	c
MH709350	5	c	a	g	a	t	t	a	t	t	a	t	a	c	c	t	c	a	t	g	g	c
MH709351	5	c	a	g	a	t	t	a	t	t	a	t	a	c	c	t	c	a	t	g	g	c
MH709353	5	c	a	g	a	t	t	a	t	t	a	t	a	c	c	t	c	a	t	g	g	c
MH709358	5	c	a	g	a	t	t	a	t	t	a	t	a	c	c	t	c	a	t	g	g	c
MH709360	5	c	a	g	a	t	t	a	t	t	a	t	a	c	c	t	c	a	c	g	g	c
MH709362	5	c	a	g	a	t	t	a	t	t	a	t	a	c	c	t	c	a	t	g	g	c
MH709365	5	c	a	g	a	t	t	a	t	t	a	t	a	c	c	t	c	a	t	g	g	c
MH709373	5	c	a	g	a	t	t	a	t	t	a	t	a	c	c	t	c	a	t	g	g	c
MH709374	5	c	a	g	a	t	t	a	t	t	a	t	a	c	c	t	c	a	t	g	g	c
KC112914	6	c	c	g	a	t	t	g	t	t	a	t	a	c	c	g	c	a	t	g	g	c
KP771914	6	c	c	g	a	t	t	g	t	t	a	t	a	c	c	g	t	a	t	g	g	c
KP702725	6	c	c	g	a	t	t	g	t	t	a	t	a	c	c	g	c	a	t	g	g	c
KP771890	9	c	a	g	a	t	t	g	c	t	a	t	a	c	c	t	c	a	t	g	g	c
JN704710	9	c	a	g	a	t	t	g	c	t	a	t	a	c	c	t	c	a	t	g	g	c
KY037798	9	c	a	g	a	t	t	g	c	t	a	t	a	c	c	t	c	a	t	g	g	t
MH709312	9	c	a	g	a	t	t	g	c	t	a	t	a	c	c	t	c	a	t	g	g	t
MH709352	9	a	a	g	a	t	t	g	c	t	a	t	a	c	c	t	c	a	t	g	g	t
AB097933	Vac	c	c	g	a	t	t	g	t	t	a	t	a	c	c	g	c	a	t	g	g	c
JN704698	Vac	c	c	g	a	t	t	g	t	t	a	t	a	c	c	g	c	a	t	g	g	c
JN704697	Vac	c	c	g	a	t	t	g	t	t	a	t	a	c	c	g	c	a	t	g	g	c
DQ008355	Vac	c	c	g	a	t	t	g	t	t	a	t	a	c	c	g	c	a	t	g	g	c
JF306641	Vac	c	c	g	a	t	t	g	t	t	a	t	a	c	c	g	c	a	t	g	g	c
AB097932	Vac	c	c	g	a	t	t	g	t	t	a	t	a	c	c	g	c	a	t	g	g	c
DQ008354	Vac	c	c	g	a	t	t	g	t	t	a	t	a	c	c	g	c	a	t	g	g	c
KU926312	Vac	c	c	g	a	t	t	g	t	t	a	t	a	c	c	g	c	a	t	g	g	c
KU926313	Vac	c	c	g	a	t	t	g	t	t	a	t	a	c	c	g	c	a	t	g	g	c
KU926314	Vac	c	c	g	a	t	t	g	t	t	a	t	a	c	c	g	c	a	t	g	g	c
KF558371	Vac	c	c	g	a	t	t	g	t	t	a	t	a	c	c	g	c	a	t	g	g	c
KF558372	Vac	c	c	g	a	t	t	g	t	t	a	t	a	c	c	g	c	a	t	g	a	c
KF558373	Vac	c	c	g	a	t	t	g	t	t	a	t	a	c	c	g	c	a	t	g	g	c
KF558374	Vac	c	c	g	a	t	t	g	t	t	a	t	a	c	c	g	c	a	t	g	g	c
KF558375	Vac	c	c	g	a	t	t	g	t	t	a	t	a	c	c	g	c	a	t	g	g	c
KF558376	Vac	c	c	g	a	t	t	g	t	t	a	t	a	c	c	g	c	a	t	g	g	c
KF558377	Vac	c	c	g	a	t	t	g	t	t	a	t	a	c	c	g	c	a	t	g	g	c
KF558378	Vac	c	c	g	a	t	t	g	t	t	a	t	a	c	c	g	c	a	t	g	g	c
KF558379	Vac	c	c	g	a	t	t	g	t	t	a	t	a	c	c	g	c	a	t	g	g	c
KF558380	Vac	c	c	g	a	t	t	g	t	t	a	t	a	c	c	g	c	a	t	g	g	c
[truncated: 723,362 more chars]
